# Global, regional, and national trends in routine childhood vaccination coverage from 1980 to 2023 with forecasts to 2030: a systematic analysis for the Global Burden of Disease Study 2023

**DOI:** 10.1016/S0140-6736(25)01037-2

**Published:** 2025-06-24

**Authors:** GBD 2023 Vaccine Coverage Collaborators

## Abstract

**Background:**

Since its inception in 1974, the Essential Programme on Immunization (EPI) has achieved remarkable success, averting the deaths of an estimated 154 million children worldwide through routine childhood vaccination. However, more recent decades have seen persistent coverage inequities and stagnating progress, which have been further amplified by the COVID-19 pandemic. In 2019, The World Health Organization (WHO) set ambitious goals for improving vaccine coverage globally through the Immunization Agenda 2030 (IA2030). Now halfway through the decade, understanding past and recent coverage trends can help inform and reorient strategies for approaching these aims in the next five years.

**Methods:**

Based on the Global Burden of Diseases, Injuries, and Risk Factors Study (GBD) 2023, this study provides updated global, regional, and national estimates of routine childhood vaccine coverage from 1980 to 2023 for 204 countries and territories for 11 vaccine-dose combinations recommended by the WHO for all children globally. Employing advanced modelling techniques, this analysis accounts for data biases and heterogeneity and integrates new methodologies to model vaccine scale-up and COVID-19 pandemic-related disruptions. To contextualize historic coverage trends and gains still needed to achieve IA2030 coverage targets, we supplement these results with several secondary analyses: 1) we assess the impact of the COVID-19 pandemic on vaccine coverage, 2) we forecast coverage of select life course vaccines through the year 2030, and 3) we analyse progress needed to reduce the number of zero-dose children by half between 2023 and 2030.

**Findings:**

Overall, global coverage for original EPI vaccines against diphtheria-tetanus-pertussis (DTP1 and DTP3, ie, first and third doses), measles (MCV1), polio (Pol3), and tuberculosis (BCG) nearly doubled from 1980 to 2023. However, this long-term trend masks recent challenges. Coverage gains slowed between 2010 and 2019 in many countries and territories, including declines in 21 of 36 high-income countries and territories for at least one of these vaccine-doses (excluding BCG, which has been removed from routine immunisation schedules in some countries and territories). The COVID-19 pandemic exacerbated these challenges, with global rates for these vaccines declining sharply since 2020, and still not returning to pre-COVID-19 pandemic levels as of 2023. Coverage for newer vaccines developed and introduced in more recent years, such as immunisations against pneumococcal disease (PCV3) and rotavirus (complete series, RotaC) and a second dose of the measles vaccine (MCV2) saw continued increases globally during the COVID-19 pandemic due to ongoing introductions and scale-ups, but at slower rates than expected in the absence of the pandemic. Forecasts to 2030 for DTP3, PCV3 and MCV2 suggest that only DTP3 would achieve the IA2030 target of 90% global coverage, and only under an optimistic scenario.

The number of “zero-dose children”, proxied as children under one year of age who do not receive DTP1, fell by 74·9% (95% uncertainty interval [UI] 72·1–77·3) globally between 1980 and 2019, with most of those declines achieved during the 1980s and the 2000s. After 2019, counts of zero-dose children rose to a COVID 19-era peak of 18·6 million (17·6–20·0) in 2021. Most zero-dose children remain concentrated in conflict-affected regions and those with various constraints on resources available to put towards vaccination services, particularly sub-Saharan Africa. As of 2023, more than 50% of the 15·7 million [14·6–17·0] global zero-dose children resided in just eight countries (Nigeria, India, Democratic Republic of the Congo, Ethiopia, Somalia, Sudan, Indonesia, and Brazil), emphasising persistent inequities.

**Interpretation:**

Our estimates of current vaccine coverage and forecasts to 2030 suggest that achieving IA2030 targets, such as halving zero-dose children compared to 2019 levels and reaching 90% global coverage for life-course vaccines DTP3, PCV3, and MCV2, will require accelerated progress. Substantial increases in coverage are necessary in many countries and territories, with those in sub-Saharan Africa and south Asia facing the greatest challenges. Recent declines will need to be reversed to restore previous coverage levels in Latin America and the Caribbean, especially for DTP1, DTP3 and Pol3.

These findings underscore the critical need for targeted, equitable immunisation strategies. Strengthening primary health-care systems, addressing vaccine misinformation and hesitancy, and adapting to local contexts are essential to advancing coverage. COVID-19 pandemic recovery efforts, such as WHO’s “Big Catch-Up” initiative, as well as efforts to bolster routine services must prioritise reaching marginalised populations and target subnational geographies to regain lost ground and achieve global immunisation goals.

## Introduction

Building on the success of the global campaign to eradicate smallpox, the Expanded Programme on Immunization (EPI) was launched in 1974 by the World Health Organization (WHO) to extend the benefits of universal immunisation against common childhood diseases to all the world’s children.^1^ EPI initially supported the deployment of vaccines to combat diphtheria, tetanus, pertussis, polio, measles, and tuberculosis. Over the ensuing 50 years, EPI—now renamed the Essential Programme on Immunization—has added more childhood vaccinations, recommending vaccinations for all children globally against hepatitis B, *Haemophilus influenzae* type b, pneumococcus, rotavirus, and rubella, along with a second dose of the measles vaccine, while broadening to include recommendations for adolescent vaccination against human papillomavirus.^2^ Through partnerships between local healthcare workers, national immunization programs, regional authorities, and international organizations, EPI has achieved remarkable health gains. The impact of making routine childhood immunisation (ie, regular and ongoing immunisation services, often delivered during routine health visits) widely available has been dramatic, resulting in an estimated 154 million deaths averted globally between 1974 and 2024, with nearly 95% of those in children younger than five years of age.^3^

Although delivering routine childhood vaccinations worldwide requires a tremendous investment of global resources, including approximately US$3.9 billion in development assistance for health in 2023,^4^ childhood immunisation has proven to be one of the most successful and cost-effective public health strategies known, both in terms of lives saved and return on investment.^5,6^ Estimates have shown the financial rate of return to be in some instances up to 44 times the cost of vaccination.^7^ Yet the remarkable successes of EPI have slowed in the past decade and in some cases reversed, suggesting weaknesses in health services that were further exposed during the global upheaval caused by the COVID-19 pandemic, including social distancing measures, health system diversions, and supply chain disruptions. Previous estimates suggest that coverage with the third dose of diphtheria-tetanus-pertussis vaccine (DTP3) decreased in 94 countries and territories between 2010 and 2019, and only 11 countries worldwide were estimated to have reached the 2019 target set by the Global Vaccine Action Plan (GVAP) of at least 90% coverage for all assessed vaccines.^8^ As coverage has stalled, new and increased outbreaks of vaccine-preventable illness such as measles, polio, and diphtheria have emerged in many countries and territories.^9^

To successfully further the reach, equity, and sustainability of global immunisation systems, it is necessary to overcome enduring and emerging challenges such as growing economic uncertainty and geopolitical instability that constrain funding for vaccination and global health, migration and population displacement,^10,11^ geographical and sociodemographic disparities in access to vaccines,^12–17^ disruptions to immunisation delivery related to events such as natural disasters or widespread infectious disease outbreaks such as those caused by the SARS-CoV-2 and Ebola viruses,^18–22^ and an upsurge in vaccine misinformation and hesitancy.^23–25^ To meet these challenges, WHO’s World Health Assembly endorsed Immunization Agenda 2030 (IA2030),^10^ an updated framework to envision and achieve universal immunisation, building on the previous GVAP approach with a broader scope and increased tailoring for local contexts.^26^ Focused on centring the expertise of local and country-level partners and authorities,^27^ aligned with the United Nations Sustainable Development Goal 3 to ensure healthy lives and promote well-being for all at all ages,^28^ IA2030 sets an ambitious global agenda to achieve “a world where everyone, everywhere, at every age, fully benefits from vaccines to improve health and well-being”.^29^ One of IA2030’s primary goals is to promote equity by halving (relative to 2019) the number of “zero-dose children”, that is, children missed by routine childhood vaccination, typically proxied by estimating those who have not received any DTP doses. Zero-dose children are more likely to miss out on subsequent vaccinations^30^ and experience other types of deprivation^17^, and strategies to reach these missed children with vaccination services can bolster routine health services more broadly. IA2030 further emphasises the necessity of extending the benefits of vaccination throughout the life course, reaching beyond early childhood to deliver essential catch-up vaccinations and booster doses, along with a growing number of new vaccines scheduled for administration after childhood. To this end, IA2030 sets a goal of achieving 90% global coverage for vaccines across the life-course, including DTP3, the third dose of pneumococcal conjugate vaccine (PCV3), the second dose of measles-containing vaccine (MCV2), and the complete human papillomavirus vaccine series (HPVc).^10,29^ These ambitious targets are vital to prevent the resurgence of vaccine-preventable diseases and to foster strong and resilient immunisation and health-care systems that will serve as a platform for the introduction of new vaccines. The need for strong routine health systems to enable vaccine delivery was underscored during the pandemic and holds true today as new vaccines for malaria, dengue, Ebola virus, and other diseases are being developed and deployed.

To advance universal childhood immunisation, a core principle of IA2030 is reliance on high-quality, targeted data to guide immunisation policies and programmes and to better measure progress extending vaccination coverage. Since 2000, a key source of vaccination data has been the WHO–UNICEF Estimates of National Immunization Coverage (WUENIC),^31^ which uses a rules-based approach to provide annual routine vaccination coverage estimates for all WHO member states using expert judgment and qualitative knowledge to compile primary data from available sources, WUENIC estimates use country-reported and administrative data gathered through the WHO-UNICEF Joint Reporting Form (JRF)^31–33^ and are informed by data from established household surveys.^34,35^ These estimates from WUENIC incorporate expert judgement and qualitative knowledge in their comprehensive compilation of primary data sources; however, the rules-based approach may lead to flat or noisy time trends, particularly in data-sparse locations, and is not able to account for uncertainty in the estimation process.^33^ Prior work from the Global Burden of Diseases, Injuries, and Risk Factors Study (GBD) has applied a comprehensive statistical model to these data to systematically generate national estimates of vaccine coverage from 1980 to 2019.^8^ Because primary vaccination data can be inconsistent (ie, discrepant sampling methods and/or results),^36^ sparse (ie, few data in certain locations), and subject to bias (ie, systematic error imposed through biased sampling methodology),^37,38^ the use of a statistical model to derive coverage estimates confers multiple advantages. These include the capacity to synthesise data from heterogeneous sources while accounting for the effects of discrepant data quality and types (eg, administrative versus survey) as well as the presence of systematic bias; to overcome data sparsity by leveraging time trends and other predictors; and to formally quantify estimation uncertainty.

Here, following the 50^th^ anniversary year of EPI’s founding and half-way through the decade of IA2030, we build on the framework of the previous GBD vaccine coverage study^8^ to generate updated estimates of vaccine coverage for 204 countries and territories from 1980 to 2023. We analyse progress over time in the coverage of key childhood vaccine-dose combinations and estimate trends in numbers of zero-dose individuals. We extend the prior GBD analysis to include the COVID-19 era, including many data sources delayed in reporting by the COVID-19 pandemic. We deploy new methods to account for the immediate and enduring effects of the COVID-19 pandemic in a unified framework, enhance estimation of the scale-up of newly-introduced vaccines, and improve our estimation of counts of zero-dose children by modelling DTP1 directly. Looking forward, we assess progress towards the IA2030 goals of 50% reduction in zero-dose children and 90% global coverage of select life course vaccines in secondary analyses. First, building off an early framework developed by Causey et al. (2021),^18^ we use a counterfactual approach to quantify the impact of the COVID-19 pandemic on the number of children who missed routine vaccinations between 2020 and 2023. Second, we evaluate progress needed to achieve a 50% reduction in zero-dose children by 2030. Last, we forecast future DTP3, PCV3, and MCV2 coverage through 2030 under three scenarios to illustrate the range of plausible future trajectories. These comprehensive, updated estimates illustrate progress and challenges in the effort to immunise against routine childhood diseases, providing crucial evidence to inform policies, programmes, and investments aimed at ensuring that all children, everywhere, receive life-saving vaccinations.

This manuscript was produced as part of the GBD Collaborator Network and in accordance with the GBD Protocol.^39^ Because newly available data and modified methods were used to update the full time series of estimates from 1980 through 2023, these results supersede all previous estimates.

## Methods

### Overview

To generate coverage estimates of routine childhood vaccination in 204 countries and territories from 1980 to 2023, our core analysis followed the previous GBD methods,^8^ applying a multi-step modelling approach using spatiotemporal Gaussian process regression (ST-GPR)^40^ and meta-regression—Bayesian, regularised, trimmed (MR-BRT)^41^ tools to synthesise data collected primarily through the WHO/UNICEF Joint Reporting Form (JRF)^32^ and through household surveys (figure S1).^34,35^ Annual country-specific coverage estimates were calculated for 11 childhood vaccine-dose combinations supported by EPI and administered via routine national immunisation programmes, including five vaccine-dose combinations from the four vaccines introduced in 1974 against diphtheria-tetanus-pertussis (first dose; DTP1, third dose; DTP3), measles (measles-containing vaccine, first dose; MCV1), polio (third dose of any form of polio vaccination; Pol3), and tuberculosis (Bacille Calmette-Guérin; BCG)—plus six vaccine-dose combinations rolled out in subsequent years (“newer vaccines”) targeting hepatitis B (third-dose; HepB3), *Haemophilus influenzae* type b (third-dose; Hib3), rotavirus (complete series; RotaC), pneumococcus (third dose, pneumococcal conjugate vaccine; PCV3), and rubella (first-dose, rubella-containing vaccine; RCV1), along with a second dose against measles (MCV2). Our core model adjusted for location- and time-varying bias in country-reported data from the JRF; leveraged data-dense evidence for specific locations, years, and vaccines to estimate coverage in instances of data sparsity; accounted for vaccine disruptions and country-specific years of vaccine introduction; and propagated uncertainty. Details regarding our innovations to the previous GBD methods and novel secondary analyses are provided in the sections below.

This study complies with the Guidelines for Accurate and Transparent Health Estimates Reporting (GATHER) statement (table S1).^42^ Analyses were conducted using R version 4.4.0.^43^ Statistical code used for estimation is publicly available online at https://ghdx.healthdata.org/record/ihme-data/gbd-2023-vaccination-coverage-1980-2030. Additional methods details are available in appendix 1 section 1.

### Data

We reviewed 8042 data sources between years 1980 to 2023, of which 1085 unique sources were included in the analysis (representing 128 new sources compared to the previous GBD Vaccine Coverage study, including 37 new sources from 2020–2023 and 91 from 2019 and earlier). These sources comprise 64 546 country-year-vaccine-dose-specific datapoints, including 14 700 data points from vaccination-related household surveys (eg, Demographic and Health Surveys, Multiple Indicator Cluster Surveys, and other multi-country and country-specific surveys), 49 800 data points from administrative and official country-reported vaccine coverage data from the JRF and other sources, and supplemental data regarding stockout events, vaccine introductions to national immunisation programmes and vaccine schedules, also reported through the JRF.^35,44–47^ Data were catalogued on the publicly-available Global Health Data Exchange (GHDx) at https://ghdx.healthdata.org/record/ihme-data/gbd-2023-vaccination-coverage-1980-2030.^48^ As in the previous study, we grouped coverage data by birth cohort (12–23 months, 24–35 months, 36–47 months, 48–59 months—excluding cohorts under one year of age at the time of the survey) and—to align survey-based data with country-reported data—used county-specific vaccine schedules and vaccine introduction years to assign each cohort to the year of expected vaccine delivery. See appendix 1 section 1.1, tables S2 and S3 and figure S2 for complete inclusion and exclusion criteria.

### Modelling of administrative data bias

To account for bias in country-reported coverage data,^49^ as in the GBD 2020 study, we used the MR-BRT modelling framework to assess differences in coverage within paired observations of survey data and original country-reported coverage from the same country-years. This bias was modelled as the ratio of survey data coverage to country-reported coverage, adjusting for the Healthcare Access and Quality (HAQ) Index (a composite index designed to assess and compare healthcare access and quality)^50^, with the expectation that bias in reporting may vary based on the quality of healthcare services. These MR-BRT bias predictions then served as a first-stage input for ST-GPR models. These bias adjustments were only applied directly for the original EPI vaccines; estimates for newer vaccines leveraged bias adjustments for the original EPI comparator vaccines through the ratio modelling process described below. New for this study, bias was directly modelled for both DTP1 and DTP3, rather than DTP3 only. See appendix 1 section 1.3 for further details.

### Modelling of stockouts and other disruptions to vaccine coverage, including COVID-19

To account for acute temporal disruptions (ie, drops) in coverage due to stockouts or other isolated events, we first modelled the magnitude of disruptions for vaccine-country-years with reported stockout events reported via the JRF^45^ or other identified disruption events (table S4). Disruption magnitudes were then included as a covariate in vaccine coverage modelling. New in this study, this covariate was devised by calculating the difference in coverage between country-reported data in vaccine-country-years identified as experiencing disruptions and counterfactual coverage estimates from models that excluded these vaccine-country-years. To account for disruptions due to COVID-19, also new to this study, we considered all vaccine-country-years for 2020–2023 as candidates for disruption events (appendix 1 section 1.4). New in this study, for vaccine-country-years in this period without available country-reported data, we imputed disruption magnitudes based on vaccine-year-specific distributions from locations with data (appendix 1 section 1.5).

### Vaccine coverage model

Our core analysis relied on modelling in ST-GPR to implement a multi-step approach that produced location-specific annual estimates of vaccine coverage for 11 routine childhood vaccine-dose combinations in 204 countries and territories over the period 1980 to 2023. ST-GPR is a stochastic modelling tool designed to synthesise heterogenous inputs and flexibly smooth data over space and time, leveraging available high-density data to guide predictions in cases of absent or sparse data and to minimise prediction error.^40^ The model uses a three stage approach, starting first with a regression incorporating covariates which may affect vaccine coverage. The second stage implements spatiotemporal smoothing, and the final stage uses a Gaussian Process regression to reduce error around high-precision data.

Importantly, improvements to this study include modifications to the modelling strategy for DTP. In previous GBD cycles, DTP3 coverage was modelled directly, and DTP1 was estimated using a continuation ratio ordinal regression approach.^8^ For GBD 2023, with increasing global focus on zero-dose children (proxied as those who have not received DTP1), we now model DTP1 directly and, to ensure internal consistency where DTP1 > DTP3, estimate DTP3 by modelling the DTP3/DTP1 ratio.

For the directly modelled vaccines—DTP1, MCV1, Pol3, and BCG—country- and year-specific estimates of coverage were produced using ST-GPR models fit to bias-adjusted official country-reported data and survey data. ST-GPR models included covariates for the HAQ Index;^50^ mortality rates due directly to war and terror events;^51–53^ and vaccine disruptions. We also used this approach to model the ratio of DTP3/DTP1 coverage (using bias-adjusted official country-reported data and survey for both numerator and denominator), which was multiplied post-hoc by our modelled estimates of DTP1 coverage by draw to calculate DTP3 coverage.

For other vaccines, we modelled the ratio of coverage to that of one of the original EPI vaccines, using ST-GPR to allow for similarities and differences in these relationships across space and time. DTP3 served as the denominator, or reference vaccine, for modelling HepB3, Hib3, PCV3, and RotaC vaccine coverage ratios, given that it is typically given either as part of a combination vaccine and/or on the same schedule as these vaccines, while MCV1 was used as the reference vaccine for MCV2 and RCV1, to ensure that MCV2 coverage does not exceed MCV1 coverage, and because RCV1 is often delivered in a combination vaccine with MCV1). All ratios were constrained to be less than one, assuming newer vaccine coverage will be less than the original corresponding reference vaccine. New for GBD 2023, we estimated the scale-up of each newer vaccine ratio as a function of years since introduction. By explicitly modelling scale-up patterns and allowing these to vary by country and vaccine, we improved estimation in early years after introduction and in settings with sparse data. We fit predictive spline models of coverage ratios using MR-BRT in a geographical cascade: vaccine-specific models were first fit across all countries and territories, and the global model fits then served as priors for country-specific models. In this process, coverage ratios were modelled as a function of vaccine disruptions, years-since-introduction (YSI, fit using a spline), and a country-level random effect (appendix 1 section 1.6 and figure S3). The results of these spline models were used as first-stage estimates in subsequent ST-GPR coverage ratio models. Last, we multiplied the predicted coverage ratios from ST-GPR by the corresponding reference vaccine coverage to generate final estimates of coverage.

Uncertainty was propagated by sampling 1000 random draws from the posterior distribution of each modelling step and conducting all subsequent calculations by draw. Results were summarised using the mean of all draws and the ordinal 2·5^th^ and 97·5^th^ percentile of draws to compute 95% uncertainty intervals (UIs). Super-regional and global aggregate estimates were calculated at the draw level as target population-weighted means (table S5).^54,55^ In- and out-of-sample goodness of fit statistics were calculated (appendix 1 section 1.7). Coverage estimates were also compared to estimates from the previous GBD2020 vaccine coverage study and estimates published by WUENIC in 2024 (appendix 1 section 2).^8,56^

### Secondary and post-hoc analyses

#### COVID-free counterfactual

To understand the impact of the COVID-19 pandemic on childhood vaccination rates, we calculated vaccine coverage in a counterfactual (ie, alternative) scenario where coverage was not affected by disruptions due to COVID-19 (“COVID-free”). This was achieved by removing post-hoc the disruption covariate effects from coverage estimates in years 2020–2023 (appendix 1 section 1.8). To account for potential disruptions that would have occurred even without the COVID-19 pandemic, we applied an adjustment scalar to the counterfactual estimates based on country-draw-level averages of disruption sizes for the preceding five (2015–2019; figure S4). This year range was chosen to reflect the most recent patterns in the occurrence and magnitude of disruptions.

#### Progress needed to achieve IA2030 reduction targets in zero-dose children by 2030

To assess progress towards the IA2030 goal of reducing the number of zero-dose children globally by half by the year 2030, compared to 2019, we considered a hypothetical scenario where all countries and territories reduce zero-dose children by 50% between these years (ie, equal contributions towards this goal). Using GBD population forecasts,^57^ we calculated the number of zero-dose children needed to be reached by 2030 and corresponding DTP1 coverage.

To contextualise required DTP1 coverage increases, we calculated the 2023–2030 annualised rate of change (AROC) in DTP1 coverage needed for each country to meet this target (appendix 1 section 1.9). We then compared these to the distribution of AROCs across historical seven-year periods from 2000 to 2019 for all countries and territories.

#### Progress towards life-course vaccines (DTP3, PCV3, MCV2) 90% coverage targets by 2030

To predict progress toward IA2030’s target of 90% coverage in 2030 for routine childhood vaccination across the life course, we adapted methods of Foreman and colleagues^58^ and GBD 2021 Forecasting Collaborators^59^ to forecast future coverage for DTP3, PCV3, and MCV2.

We produced reference vaccine coverage forecasts for 2030 that capture the most likely “reference” future scenario. Forecasted DTP3 coverage for the reference scenario was estimated in a predictive modelling framework that leverages historic relationships between vaccine coverage estimates and the Socio-demographic Index (SDI), a composite indicator measuring a country’s development status, using a logistic regression framework with SDI as the sole covariate.^55^ To illustrate a plausible range of future coverage trajectories, we also produced alternative “better” and “worse” scenario forecasts based on historic rates of change. The better and worse scenario estimates were forecasts based on the 85th and 15th percentiles, respectively, of the distribution of past rates of change in coverage (in natural log space) between subsequent years. The historic distributions were calculated by draw, pulling from across countries and territories and all year pairs 1980–2019, excluding years impacted by the COVID-19 pandemic. Rates of change from more recent years were weighted more heavily in the distribution compared to those from earlier in the time series. Forecasts of PCV3/DTP3 and MCV2/MCV1 coverage ratios were produced using equivalent ratio modelling techniques as used in historical coverage estimation. We then multiplied by forecasted DTP3 and MCV1 estimates, respectively, for each scenario to calculate PCV3 and MCV2 coverage. For further details, see appendix 1 section 1.10.

### Role of the funding source

The funders of this study had no role in study design, data collection, data analysis, data interpretation, or the writing of the report. The lead and senior authors had full access to the data in the study and final responsibility for the decision to submit for publication.

## Results

### Trends in vaccine coverage, 1980–2019

**Figure 1:**
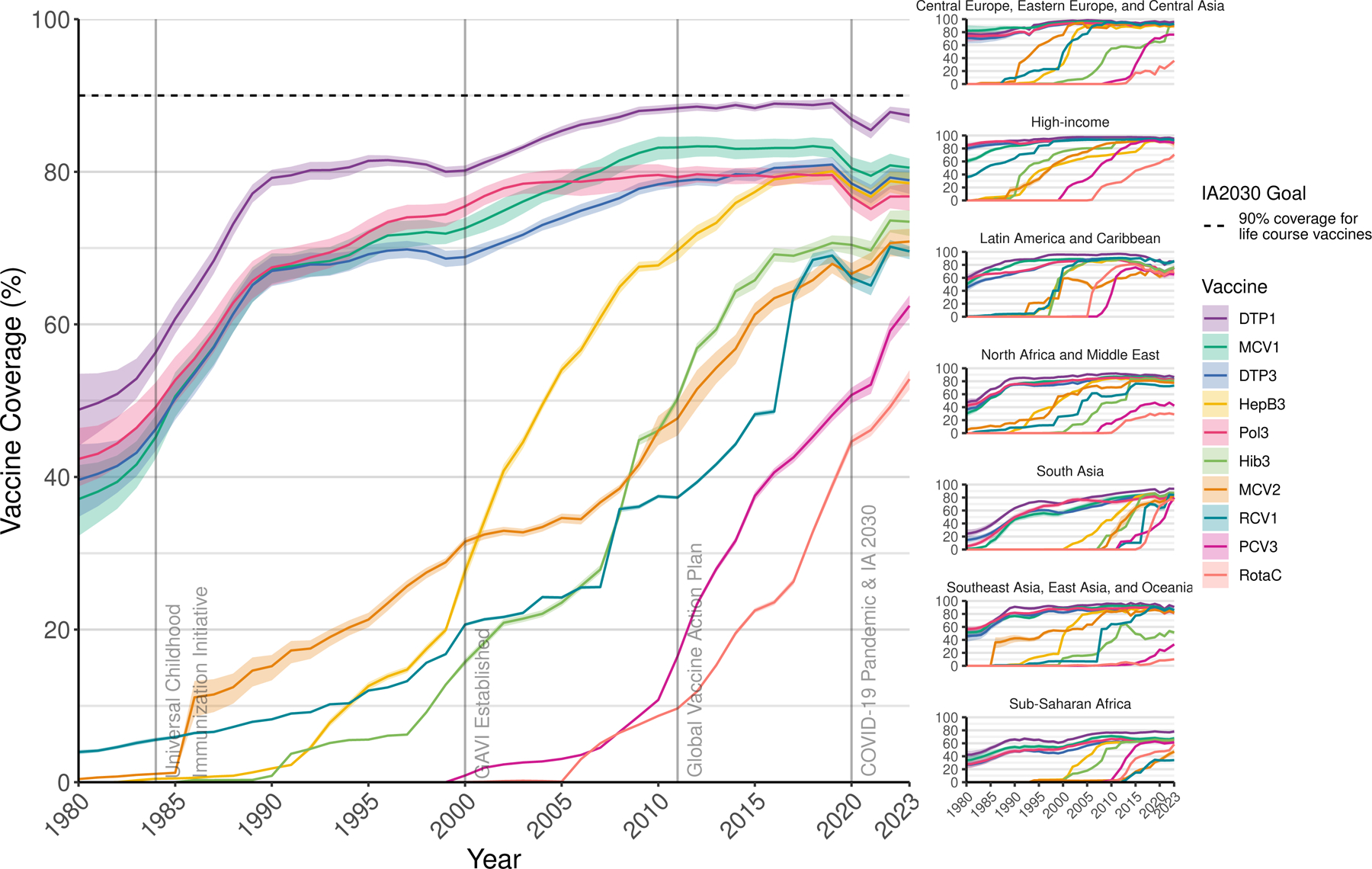
Global and super-regional estimates of vaccine coverage over time. Mean global (left) and super-regional (right) coverage estimates for the target age population by year for each vaccine, with 95% uncertainty intervals. The dashed horizontal line indicates the coverage required to meet the IA2030 goal of 90% coverage for life-course vaccines. DTP1=diphtheria-tetanus-pertussis, first dose. DTP3=diphtheria-tetanus-pertussis, third dose. HepB3=hepatitis B vaccine, third dose. Hib3=Haemophilus influenzae type b vaccine, third dose. MCV1=measles-containing vaccine, first dose. MCV2=measles-containing vaccine, second dose. PCV3=pneumococcal conjugate vaccine, third dose. Pol3=polio vaccine, third dose. RCV1=rubella-containing vaccine, first dose. RotaC=completed rotavirus series.

Between 1980 and 2019, global vaccine coverage for the original EPI vaccines—BCG, MCV1, DTP1 & 3, and Pol3—approximately doubled, from 38.1% (95% UI 33.9–42.3) in 1980 to 83·3% (82·7–84·0) in 2019 for BCG, 37.1% (32.3–41.6) to 83·1% (81·8–84·3) for MCV1, 48.8% (44.1–53.5) to 89·0% (88·3–89·6) for DTP1, 39.6% (34.8–44.3) to 80·9% (79·9–81·9) for DTP3, and 42.4% (38.8–46.4) to 79·6% (78·3–81·0) for Pol3 (figures 1, S5 & S6). Across this timeframe, this equates to an estimated 4·1 billion (4·07–4·12) children vaccinated with BCG, 4·01 billion (3·98–4·04) with MCV1, 4·48 billion (4·45–4·51) with DTP1, 3·89 billion (3·85–3·93) with DTP3, and 4·00 billion (3·96–4·04) with Pol3 through routine immunisation programmes. However, these gains slowed or reversed between 2010 and 2019, even prior to COVID-19 pandemic-related disruptions in subsequent years. For MCV1, coverage declined between 2010 and 2019 for 100 of 204 countries and territories (figures S5 & S6), with the biggest decrease in the Latin America and the Caribbean super-region (90.4% [88.6–91.9] in 2010; 86.8% [85.0–88.4] in 2019). For DTP1, DTP3, and Pol3, coverage declined between 2010 and 2019 for 100, 98, and 107 countries and territories, respectively, with the largest decreases similarly in Latin America and the Caribbean (DTP1 96·5% [96·0–96·9] in 2010 and 85·4% [83·3–87·3] in 2019; DTP3 89·8% [89·0–90·6] in 2010 and 73·9% [70·9–76·3] in 2019; Pol3 87·8% [86·9–88·7] in 2010 and 76·9% [74·9–78·6] in 2019). Of 158 countries and territories with BCG in the national immunisation schedule for all years between 2010 and 2019,^44^ coverage declined for 88 countries and territories over this period.

For newer vaccines, coverage gains were more consistent. Coverage for HepB3 (80·1% [95% UI 79·0–81·0]), Hib3 (70·7% [69·6–71·7]), MCV2 (67·9% [66·7–69·2]), and RCV1 (69·0% [68·0–69·9]) had begun to approach that of the original EPI vaccines by 2019 (figure 1). Global expansion of coverage for PCV3 and RotaC did not begin until the mid-2000s, but by 2019 global coverage reached 48·1% (47·3–49·0) for PCV3 and 38·8% (38·2–39·5) for RotaC.

### Trends in zero-dose children, 1980–2019

**Figure 2:**
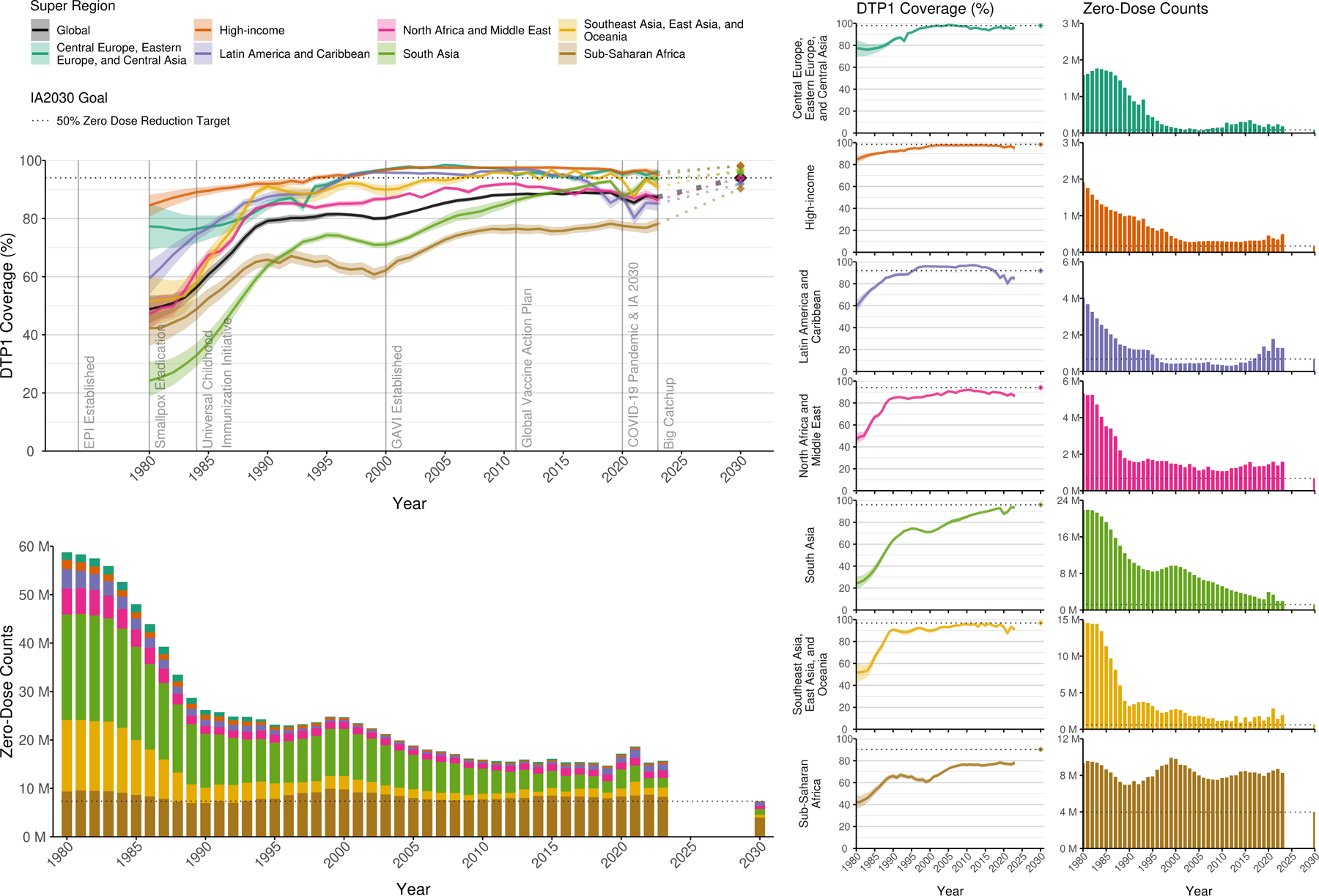
Global and super-regional trends in zero-dose children over time. Top left plot shows lines of global (in black) and super-regional (in colour) mean DTP1 coverage estimates for the population under one year of age with 95% uncertainty intervals by year for years 1980–2023. Bottom left plot shows the super-regional (colour subsets) and global (full bars) estimates of zero-dose children by year for years 1980–2023. Right plots show the same results separated by super-region. For all plots, points or bars in year 2030 and the dashed horizontal line indicate coverage or zero-dose levels required to meet IA2030 50% zero-dose reduction goal, which varies by geography. DTP1=diphtheria-tetanus-pertussis, first dose. IA2030=Immunisation Agenda 2030. EPI=Expanded Programme on Immunisation. GAVI=Global Alliance for Vaccination and Immunisation.

Between 1980 and 2019, the global number of zero-dose children—as represented by children under one year of age who have not received a DTP1 dose—fell by an estimated 74·9% (95% UI 72·1–77·3), from 58·8 million (53·4–64·2) to 14·7 million (13·8–15·6; figure 2). Most of these decreases occurred at the beginning of EPI from 1980 to 1990, when zero-dose counts fell by 55·3% (49·1–60·5), and then following the launch of Gavi when zero-dose counts fell by another 35·4% (31·5–39·5) from 2000–2010.

At the super-regional level, the greatest reductions in the number of zero-dose children between 1980 and 2019 came in south Asia, with 19·5 million (95% UI 17·6–21·0) fewer zero-dose children in 2019: an 89·3% (87·9–90·4) decrease. In sub-Saharan Africa, coverage DTP1 coverage nearly doubled between 1980 and 2019, from (from 42·3% [36·3–48·7] to 78·2% [76·7–79·8]). However, the super-regional target population also grew by 125% during that time period, resulting in a more modest reduction of 1·41 million [0·168–2·46] fewer zero-dose children in 2019 than 1980. In 1980, 53·5% (52·3–54·7) of zero-dose children lived in just five countries: India, China, Indonesia, Pakistan and Bangladesh. By 2019, most (52·8% [51·0–54·3] zero-dose children still lived in only seven countries: Nigeria, India, Ethiopia, Democratic Republic of the Congo, Brazil, Somalia, and Pakistan.

### Vaccine coverage trends, 2020–2023: impact of the COVID-19 pandemic

**Figure 3:**
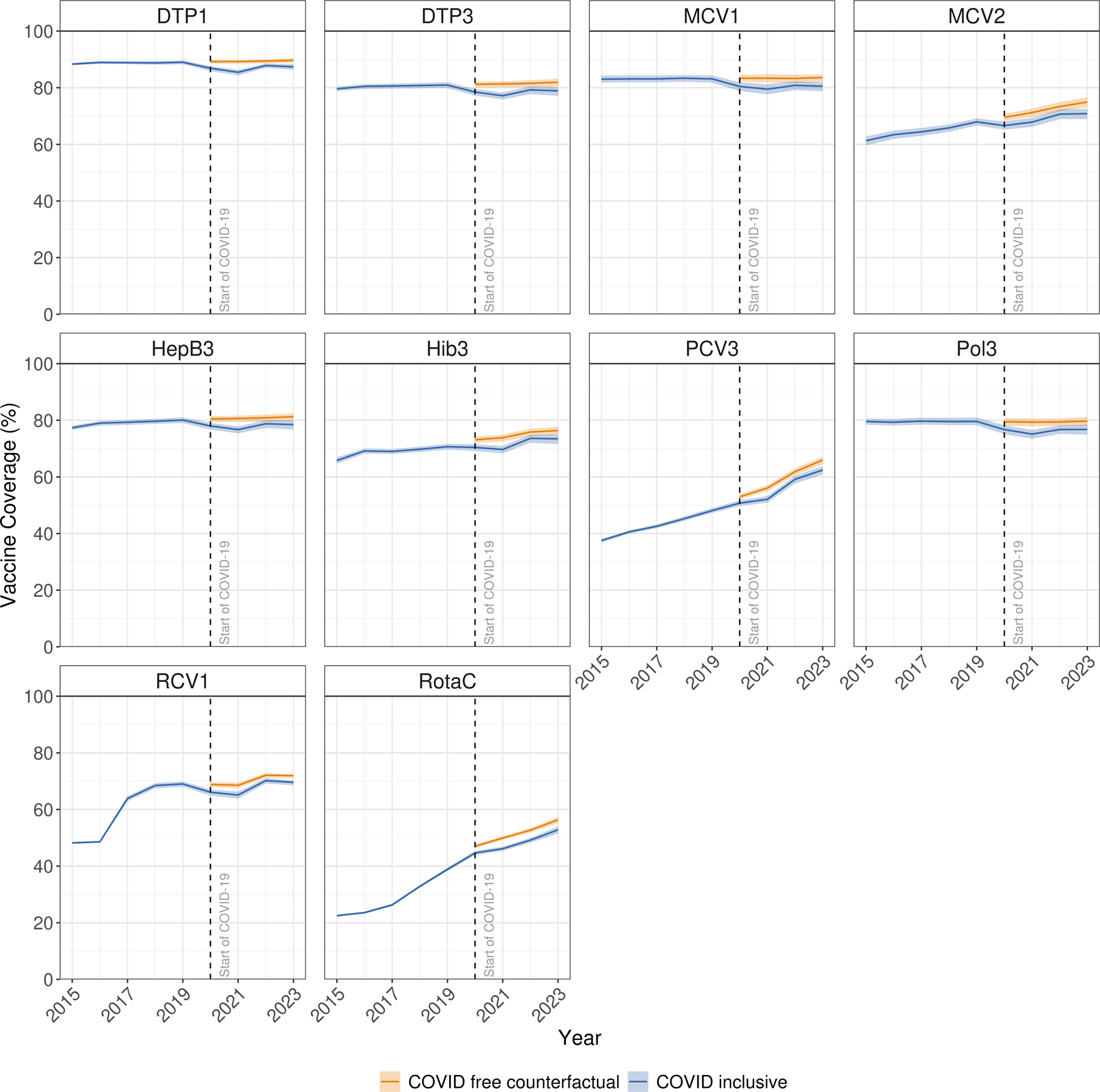
Impacts of the COVID-19 pandemic to global vaccine coverage. Comparisons of global vaccine coverage estimates for the target population during the COVID-19 pandemic (blue line) versus those expected in the absence of COVID-19 pandemic-associated disruptions (orange line), with 95% uncertainty intervals. COVID-19 pandemic-related disruptions were estimated for years 2020–2023; coverage estimates for years 2015–2019 are included as a reference. DTP1=diphtheria-tetanus-pertussis, first dose. DTP3=diphtheria-tetanus-pertussis, third dose. HepB3=hepatitis B vaccine, third dose. Hib3=Haemophilus influenzae type b vaccine, third dose. MCV1=measles-containing vaccine, first dose. MCV2=measles-containing vaccine, second dose. PCV3=pneumococcal conjugate vaccine, third dose. Pol3=polio vaccine, third dose. RCV1=rubella-containing vaccine, first dose. RotaC=completed rotavirus series.

Global coverage for all original EPI vaccines declined following the onset of the COVID-19 pandemic. Substantial COVID-19 pandemic-related disruptions to global coverage for the original EPI vaccines began in 2020, generally increased in 2021 and 2022, then improved but did not fully resolve by 2023 (figure 3). The greatest decreases between 2019 (the final pre-pandemic comparator year) and 2023 were estimated for Pol3 coverage (2·8 percentage points; [pp] [95% UI 0·7–5·0]) and the smallest decreases for DTP1 (1·6 pp [0·5–2·9]).

Global coverage for most of the newer vaccines continued to expand over the course of the COVID-19 pandemic, driven by both continued introductions and scale-up (figure 1). The largest gains between 2019 (the year prior to the COVID-19 pandemic) and 2023 were estimated for PCV3 (14·3 pp [95% UI 12·9–15·6]). All newer vaccines reached higher coverage levels in 2023 compared to 2019, except for HepB3. HepB3 is typically given as a part of a pentavalent vaccine with DTP, and global HepB3 coverage more closely mirrored DTP3 coverage and experienced similar disruptions during this time period. Global HepB3 coverage in 2023 remained 1·6pp (0·2–3·2) lower than that seen in 2019.

Compared to a counterfactual scenario absent COVID-related disruptions, global DTP3 coverage was 2·7 pp (95% UI 2·4–3·2) lower in 2020, 4·2 pp (3·8–4·6) lower in 2021, 2·3 pp (2·0–2·9) lower in 2022, and 3·1 pp (2·7–3·5) lower in 2023, with similar trends for MCV1, Pol3, and BCG (figure 3). The COVID-19 pandemic resulted in an estimated 15·6 million (14·4–16·9) fewer children vaccinated with DTP3 globally between 2020 and 2023, 15·6 million (14·4–17·0) fewer with MCV1, 15·9 million (15·0–17·2) with Pol3, and 9·18 million (8·20–10·2) with BCG. While coverage continued to increase over 2020–2023 for newer vaccines, these gains did not keep pace with expectations absent the pandemic (figure 3). Among the newer vaccines, the largest pandemic impacts were estimated for RotaC, with 16·6 million (15·7–17·7) fewer children vaccinated between 2020 and 2023 than if the pandemic had not occurred, followed by MCV2 (16·5 million [15·3–18·0] fewer children), PCV3 (15·8 million [15·0–16·8]), Hib3 (15·3 million [14·2–16·5]), HepB3 (14·4 million [13·1–15·7]) and RCV1 (13·4 million [12·2–14·7]).

Both the magnitude of COVID-19 pandemic-related disruptions to vaccine coverage and the degree of post-pandemic recovery varied by vaccine and super-region. Compared to the COVID-free counterfactual scenario, the largest single-year coverage disruptions were all estimated to have occurred in Latin America and the Caribbean, for PCV3 in 2023 and 2021, and DTP1 in 2021 (decreases of 11·6 pp [95% UI 9·6–13·9], 11·2 pp [9·7–14·1], and 11·2 pp [9·6–12·7], respectively). Among other super-regions, greatest single-year disruptions were for RotaC in sub-Saharan Africa in 2022 (a decrease of 7·7 pp [7·3–8·7]), RotaC in Central Europe, Eastern Europe, and Central Asia in 2021 (7·2 pp [6·7–7·8]), and PCV3 in North Africa and Middle East in 2021 (7·0 pp [6·2–8·4]). As of 2023, coverage of BCG, DTP1, DTP3, MCV1, and Pol3 had recovered to levels near expected without the COVID-19 pandemic (within one percentage point) only for BCG in sub-Saharan Africa and central Europe, eastern Europe, and central Asia, DTP1 in south Asia and central Europe, eastern Europe, DTP3 in and central Europe, eastern Europe, MCV1 in and central Europe, eastern Europe, and for Pol3 in south Asia (table S6, figure S7).

Sub-Saharan Africa as a super-region saw the greatest cumulative disruptions to vaccine coverage across years 2020 to 2023 in absolute numbers for RotaC, PCV3 and Pol3 (6·96 million [95% UI 6·73–7·42], 5·31 million [5·09–5·57], and 4·94 million [4·74–5·19] fewer children vaccinated, respectively). The COVID-19 pandemic also resulted in an estimated 4·12 million (3·90–4·38) additional children missing routine MCV1 vaccination in south Asia and 4·64 million (3·59–5·64) in sub-Saharan Africa. Relative to target population size, cumulative disruptions were greatest in Latin America and Caribbean, for PCV3, RotaC and DTP3, with cumulative percent losses in children vaccinated of 9·4% (8·2–10·6), 7·8% (6·7–8·9), and 7·6% (6·5–8·8), respectively. Other notable relative disruptions occurred in north Africa and Middle East for PCV3 (a cumulative loss of 5·5% [5·1–6·0]), sub-Saharan Africa for RotaC (a cumulative loss of 4·7% [4·5–5·0]), and southeast Asia, East Asia, and Oceania for Pol3 (a cumulative loss of 4·3% [3·9–4·6]). Greatest cumulative absolute and proportional COVID-19 pandemic disruptions tended to occur in Latin America and the Caribbean, sub-Saharan Africa, and south Asia (figure S7).

### Trends in zero-dose children, 2020–2023: impact of the COVID-19 pandemic and progress needed to achieve the IA2030 target of 50% reduction by 2030

The COVID-19 pandemic has reversed previous gains in reducing zero-dose children globally. As of 2023, there were 15·7 million (95% UI 14·6–17·0) zero-dose children worldwide, compared to 14·7 million (13·8–15·6) in 2019: a 2·9% (2·6–3·2) increase. This reversal follows a long period of progress, during which global zero-dose counts fell from 58·8 million (53·4–64·2) in 1980 to 14·7 million (13·8–15·6) in 2019. The global number of zero-dose children rose to 18·6 million (17·6–20·0) in 2021 before declining and still remains above pre-pandemic levels. To achieve the IA2030 zero-dose target, the global number of zero-dose children would need to be halved from 2019 levels to 7·35 million (6·92–7·82) by 2030 (figure 2, table S7). This would equate to increasing global DTP1 coverage from 87·4% (86·4–88·3) in 2019 to 94·0% (93·6–94·3) in 2023. Between 2020 and 2023, COVID-19 pandemic-related disruptions to DTP1 coverage resulted in a total of 12·8 million (11·7–14·0) additional zero-dose children over these four years. South Asia was the only super-region whose DTP1 coverage in 2023 neared levels expected in absence of the pandemic (within 0·8 pp [–0·9 to 1·9]). As of 2023, 51·1% (47·7–53·6) of all zero-dose children lived in eight countries, primarily in sub-Saharan Africa and South Asia (Nigeria, India, Democratic Republic of the Congo, Ethiopia, Somalia, Sudan, Indonesia, and Brazil). Compared to 2019, this distribution reflects recent rising numbers of zero-dose children in Sudan and Indonesia and decreases in Pakistan due to rising coverage (table S7).

Under a scenario where all countries and territories contribute equally to the IA2030 zero-dose reduction goal, accounting for anticipated population changes, 51·2% (95% UI 35·9–63·9) of the additional zero-dose children (8·34 million [7·12–9·64]) needed to be reached by vaccination in 2030 compared to 2023 would live in eight countries: Nigeria, India, Democratic Republic of the Congo, Sudan, Somalia, Indonesia, Ethiopia, and Viet Nam (table S7). The largest absolute reductions in zero-dose children over 2023–2030 would be required in sub-Saharan Africa and south Asia (4·28 million [3·46–5·10] and 1·33 million [1·07–1·61], respectively). The super-regions of Latin America and the Caribbean and central Europe, eastern Europe, and central Asia have historically achieved the DTP1 coverage levels that would be needed to reach their IA2030 targets (91·9% [90·7–92·9] and 98·0% [97·5–98·4], respectively). For south Asia, 95·9% (95·5–96·3) DTP1 coverage would be required by 2030, 2·2 pp (1·8–2·5) higher than the highest historical coverage. Sub-Saharan Africa would require 90·3% (89·6–91·0) DTP1 coverage, 12·1 pp (11·4–12·8) higher than highest historical coverage in the super-region. Together, these two super-regions account for 65·1% (62·8–68·6) of the total global reduction in zero-dose children required between 2023 and 2030.

**Figure 4:**
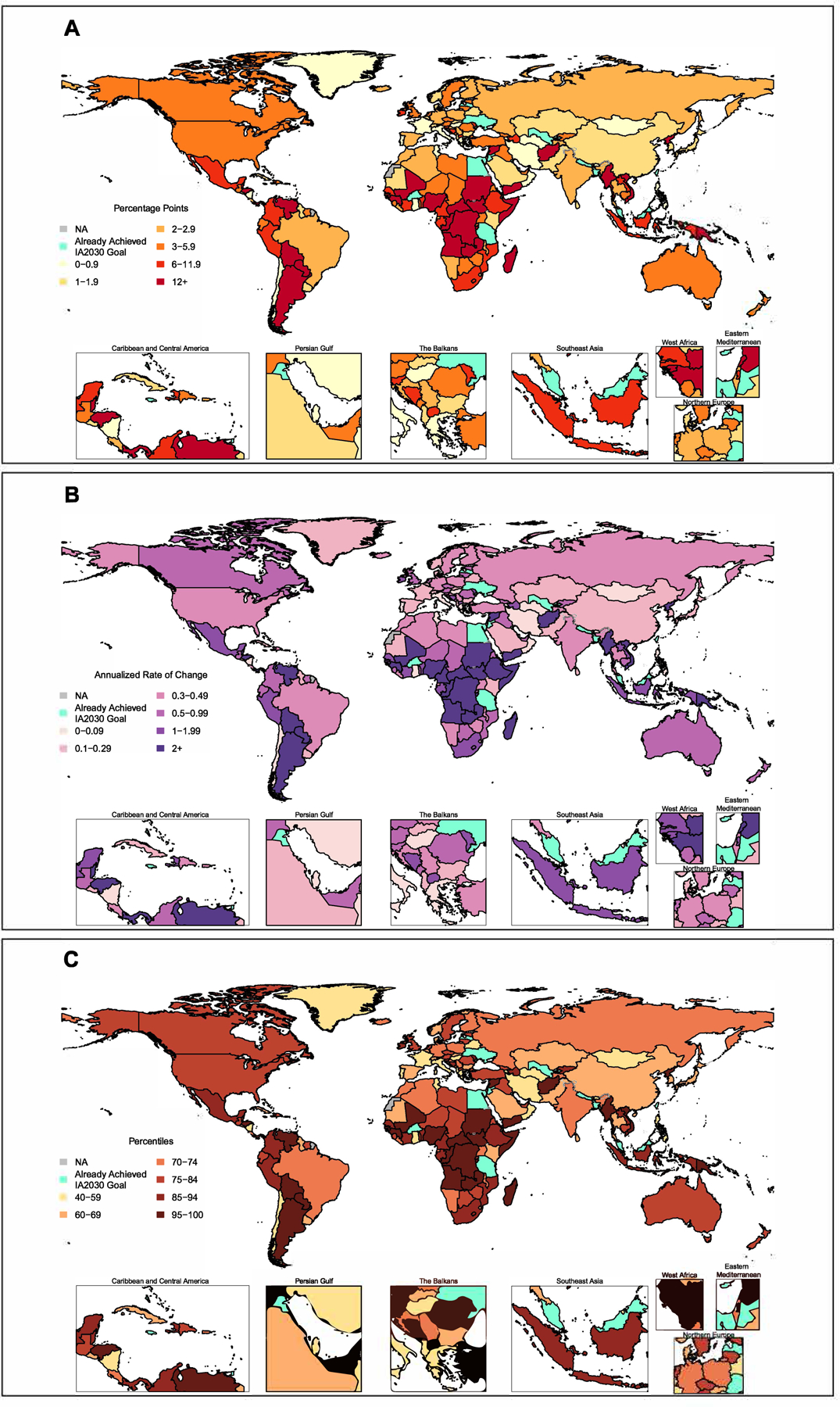
Change required to achieve IA2030 goal of 50% reduction in zero-dose children. A: Map of percentage point change from DTP1 coverage for children under one year of age in 2023 required by each country to achieve the IA2030 goal of a 50% reduction in zero-dose children by 2030. B: The annualised rate of change (AROC) in DTP1 coverage from 2023 required to achieve 50% reduction in zero-dose children by 2030. C: The AROC in DTP1 coverage required between 2023 and 2030 to achieve the same goal, expressed as a percentile of the distribution of all country-level DTP1 coverage AROCs from all seven-year periods between years 2000 and 2019. DTP1=diphtheria-tetanus-pertussis, first dose.

At the country level, 18 of 204 countries and territories had achieved a 50% reduction in zero-dose children by 2023 (figure 4). Among those countries and territories with birth cohorts of at least 10 000, Tanzania, Jordan, and Malaysia achieved the greatest percent reductions in zero-dose children, while Trinidad and Tobago, Ukraine, and Jordan achieved the greatest percentage point gains in DTP1 coverage (figure S5, table S7). Conversely, compared to 2023 levels, 40 (20%) countries and territories require a >10 pp DTP1 coverage increase by 2030, and these account for 62·4% (95% UI 52·4–71·4) of the total reduction in global zero-dose children required over this time. Many countries and territories would need to substantially outpace historical trends to reach 2030 zero-dose targets. Of 186 countries and territories not meeting this target by 2023, only 18 (10%) require a future AROC below the median of historical AROCs, and nearly half (n = 80, 43%) would need to exceed the 80^th^ percentile of historical AROCs (figure 4C). Among the eight countries needed to contribute the largest reductions in zero-dose children (4·22 million out of 8·34 million, 51·2%), seven would need to exceed the 80^th^ percentile of past DTP1 AROCs (Somalia, Sudan, Democratic Republic of the Congo, Viet Nam, Nigeria, Ethiopia, and Indonesia), six would need to exceed the 90^th^ percentile (Somalia, Sudan, Democratic Republic of the Congo, Viet Nam, Nigeria, and Ethiopia), five would need to exceed the 95^th^ percentile (Somalia, Sudan, Democratic Republic of the Congo, Viet Nam, and Nigeria), and two would need to exceed the 99^th^ percentile (Somalia and Sudan). For these countries, improvements in DTP1 coverage would need to outpace almost any gain that any country in the world has achieved since the year 2000. As of 2019, 38 countries and territories had achieved 99% DTP1 coverage or greater, but this number fell to 24 countries and territories by 2023.

### Forecasting progress towards IA2030 90% coverage targets for life-course vaccines

**Figure 5:**
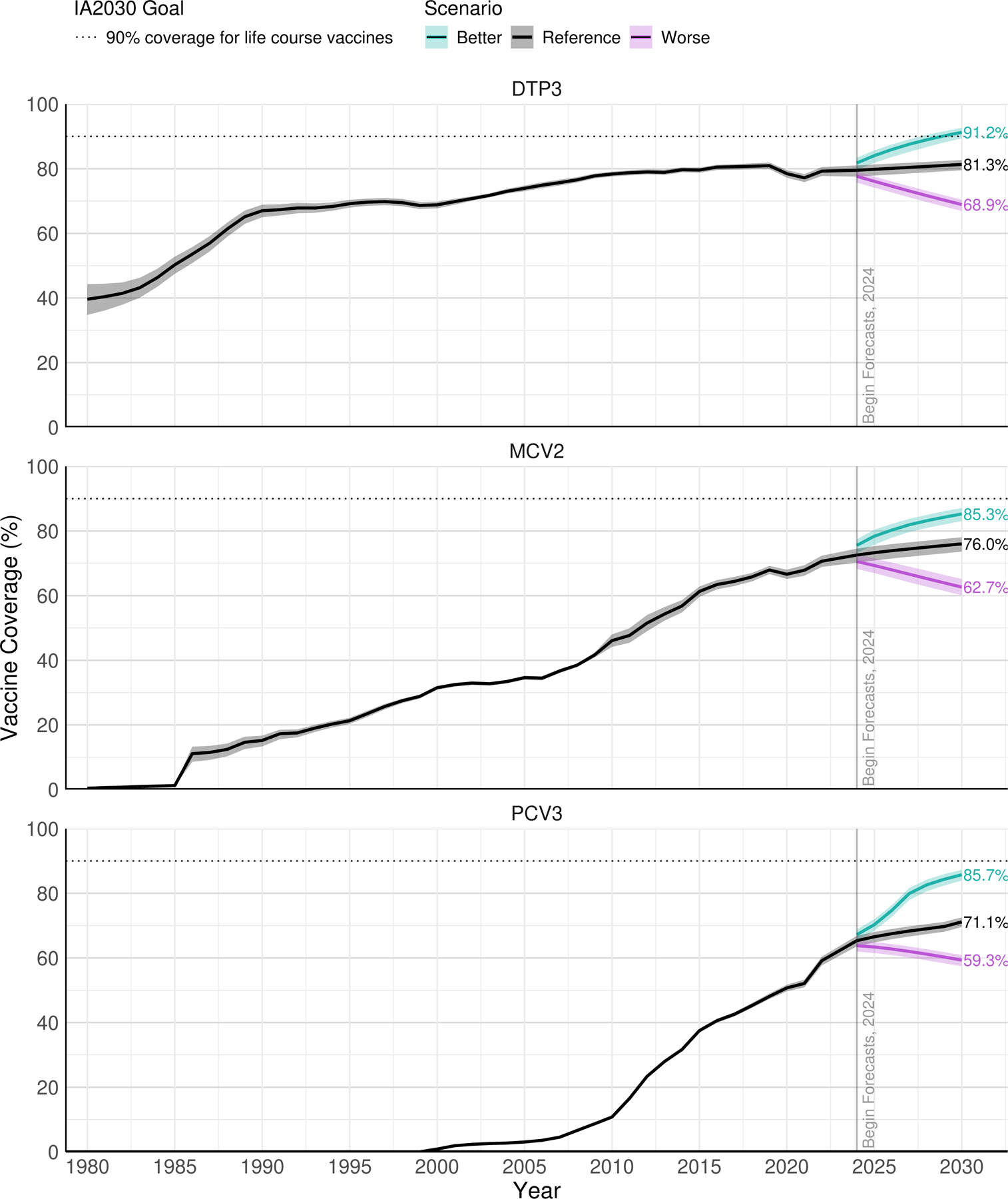
Forecasted global vaccine coverage. Historical mean global vaccine coverage for years 1980–2023 and forecast coverage for the target population for years 2024–2030 for DTP3 (top), MCV2 (middle), and PCV3 (bottom). Forecasts are displayed for the reference scenario (black) along with “better” (green) and “worse” (pink) scenarios, as well as 95% uncertainty intervals for all scenarios. Better and worse scenarios are calculated using the 85^th^ and 15^th^ percentiles of past rates of change in coverage, respectively. For all plots, the dashed horizontal line indicates the coverage required to meet the IA2030 goal of 90% coverage for life-course vaccines. DTP3=diphtheria-tetanus-pertussis, third dose. MCV2=measles-containing vaccine, second dose. PCV3=pneumococcal conjugate vaccine, third dose.

To assess progress towards the IA2030 goals of 90% global coverage for life course vaccines, we forecasted vaccine coverage for three scenarios (reference, better and worse). By 2030, globally, vaccine coverage under the reference scenario is forecasted to reach 81·3% (95% UI 79·5–82·7) for DTP3 (2·4 pp [0·6–3·8] higher than in 2023), 71·1% (69·6–72·5) for PCV3 (8·7 pp [7·1–10·1] higher than in 2023), and 76·0% (73·7–78·1) for MCV2 (5·2 pp [2·8–7·2] higher than in 2023 (figure 5). In contrast, under the “worse” scenario, global vaccine coverage could decline between 2023 and 2030 to 68·9% (66·9–70·4) for DTP3, 59·3% (57·4–60·9) for PCV3, and 62·7% (60·1–65·1) for MCV2. Alternatively, under the “better” scenario, global vaccine coverage could increase to 91·2% (89·4–92·7) for DTP3, 85·7% (84·0–87·3) for PCV3, and 85·3% (83·1–87·1) for MCV2. Even under the better scenario, only DTP3 coverage (historically higher than the more recently-introduced MCV2 and PCV3) is forecasted to achieve 90% global coverage. However, reference scenario forecasts varied substantially by GBD super-region (appendix 1 figure S8).

85 of 204 countries and territories are estimated to have achieved 90% coverage by 2023 for DTP3, 56 for PCV3, and 57 for MCV2. Under the reference scenario, an additional 23 countries and territories are forecasted to reach 90% coverage for DTP3 by 2030 (108 of 204 total), 27 for PCV3 (83 of 204 total), and 34 for MCV2 (91 of 204 total; appendix 1 figure S9). Notably, only the High-Income super region is expected to reach or retain at least 90% coverage for the three life-course vaccines by 2030 under the reference scenario. Under the better scenario, these achievements would improve to 186 of 204 countries and territories for DTP3, 171 for PCV3, and 161 for MCV2. Under the worse alternative scenario, all countries and territories that had achieved 90% coverage for DTP3, PCV3, and MCV2 by 2023 would drop below this target by 2030.

## Discussion

### Overview of main findings

The first five decades of EPI have fundamentally transformed the landscape of global health through the vaccination of more than four billion children, a doubling of coverage for the original EPI vaccines, the successful introduction and scale-up of new lifesaving vaccines, and a three-quarters reduction in the number of zero-dose children since 1980. In recent years, however, this progress has stalled and in some areas of the world reversed—a period of stagnation that began in the decade preceding the COVID-19 pandemic for the original EPI vaccines. We estimate that the COVID-19 pandemic resulted in tens of millions of additional children missing doses from across these 11 routine childhood vaccines since 2020, including an additional 12·8 million zero-dose children, compared to expectations had the pandemic not occurred. While vaccine coverage in 2023 remained lower than expected in the absence of the pandemic, there are signs of recovery across many vaccines and super-regions, thanks to the concerted efforts of local, national, regional, and global vaccine advocates.

Five decades on, therefore, the promise of EPI—to extend the lifesaving benefits of vaccines to all children around the world—has been only partially fulfilled. As these results underscore, global vaccine coverage targets cannot be met without transformational improvements in equity.

### Challenges to sustaining and improving on EPI’s successes

Despite the remarkable public health successes achieved around the globe by routine childhood vaccination over the past 50 years, efforts to preserve and extend these gains face considerable challenges. Inequalities in coverage, including large numbers of children who remain unvaccinated, persist across and within regions, countries and territories, and communities.^8,9,14,15,60,61^ As the present findings highlight—although steep drops in counts of unvaccinated zero-dose children took place over the past five decades in nearly all regions of the world—these successes were not matched in sub-Saharan Africa, where declines were considerably less pronounced. Zero-dose numbers even increased in some areas during certain periods: in sub-Saharan Africa and south Asia during the 1990s, and in Latin America and the Caribbean and in central Europe, eastern Europe, and central Asia after 2010. As of 2023, more than 50% of the world’s zero-dose children lived in just eight countries, characterised variously by weak health systems, large birth cohorts, geographic isolation, erosion of vaccine confidence and exposure to conflict^9^. Indeed, our results show that Sudan was close to achieving 90% DTP1 coverage in 2019, but with civil war arising in 2023,^51^ coverage nearly halved. Our estimates reflect complex interactions between these interrelated factors and underscore the need for targeted interventions tailored to each circumstance.

#### Impact of the COVID-19 pandemic

Starting in 2020, much of the long-term progress achieved in the global campaign to reduce mortality and morbidity through routine immunisation was halted or reversed during the massive global upheaval caused by the COVID-19 pandemic and has not fully recovered since. The crisis and its cascading effects placed extraordinary pressure on health systems and providers, immunisation supply chains, and health spending, which—combined with social distancing and stay-at-home measures—severely limited the ability of health workers and those in need of care to provide and access services.^62^ Other studies found during the height of the pandemic that coverage inequalities within regions grew during the pandemic.^63^ Even after these measures were lifted, the effects of the COVD-19 pandemic have been ongoing. Even as late as 2023, 84% of countries and territories were still reporting some disruption to health services, and immunisation ranked third highest in terms of services disrupted.^11,64^ Our estimates show that between 2019 and 2023, global numbers of zero-dose children rose to their highest levels in 2021 at 18·6 million, with counts in 2023 remaining at 15·7 million, 989 000 more than in 2019. In addition, global coverage decreased for all the original EPI vaccines between 2019 and 2023, with the greatest declines occurring in 2021.

Our analysis suggests that the COVID-19 pandemic, along with disruptions in immunization services due to recent conflicts, have resulted in tens of millions of additional children globally missing routine vaccines since 2020, increasing their risk for preventable disease and death. In 2022, 33 countries and territories reported sizeable measles outbreaks, compared to 22 in 2021. In addition, increasing numbers of wild-type polio cases have been reported in Pakistan and Afghanistan, and new outbreaks of wild-type polio occurred in Malawi and Mozambique in 2024. A resurgence of diphtheria has also been reported, with outbreaks in Bangladesh, Nepal, Nigeria, Pakistan, Venezuela, and Yemen.^65,66^ These disease trends were already on the rise before the COVID-19 pandemic^67^ and reflect longstanding inequalities in vaccine coverage but pose a global risk, including to high-income countries and territories where coverage has stagnated or declined in recent years.

Despite these challenges, the COVID-19 pandemic’s impact on vaccination coverage could have been even greater. In a previous analysis using partial-year data from the first months of the pandemic, Causey and colleagues estimated 2020 global DTP3 coverage at approximately 76.7%, which is 7.7 pp lower than was expected in the absence of the pandemic.^18^ In contrast, with the benefit of time to account for delayed reporting from many countries and data sources, our present estimates using more complete data indicate that global DTP3 coverage in 2020 was 78·5%, or just 2.7 pp lower than expected without the pandemic. The mitigation of the pandemic’s influence on vaccine coverage reflects the tremendous efforts of vaccinators worldwide and the concerted and coordinated efforts on the part of immunisation organisations to continue the delivery of essential health services in extremely challenging circumstances.^68,69^

#### Challenges and opportunities in different zero-dose populations

Due to the known challenges to vaccine delivery posed by poverty, lack of accessibility, and the presence of civil or regional conflicts,^60,61,70,71^ research and policy work focused on zero-dose children has concentrated primarily on the urban poor or those living in remote or conflict-affected areas.^72–74^ Given the consistently strong relationship between mothers’ education and whether their children are immunised,^61,75^ there is also growing awareness of the impact of the social construct of gender on vaccination coverage.^15^ The low societal status of women —manifesting in a lack of resources, agency, and power—is increasingly shown to be one of the most universal factors adversely impacting equitable childhood immunisation.^75–78^ The intersection of gender inequalities and socioeconomic factors—including migration status, poverty, ethnicity/caste, access to family planning and geographical setting—interact to depress childhood immunisation outcomes for under-resourced populations.^75,79^

Countries and territories with the highest zero-dose burdens face a demographic double challenge: while the global birth cohort is projected to shrink by 1·6% globally between 2023 and 2030,^80^ many high-burden countries and territories will experience substantial growth in their vaccination target populations. Nigeria, Ethiopia, and Democratic Republic of the Congo —which collectively contain 26·8% (24·2–28·8) of global zero-dose children as of 2023—will see birth cohorts expand by 16·1%, 10·6%, and 5·9%, respectively, during this period. These demographic trends translate directly into increased resource requirements for achieving vaccination targets or even maintaining present coverage levels. These population pressures, combined with existing health system constraints, underscore the need for vaccination strategies that scale more rapidly than population growth in these settings, including enhanced outreach services, simplified delivery models, and innovative workforce approaches. Beyond population growth challenges, even successful 50% reductions in zero-dose counts across all countries and territories would still leave 26 with less than 90% DTP1 coverage in 2030, reflecting the magnitude of current inequalities in coverage.

The diversity of challenges and barriers leading to the failure to immunise vary broadly from country to country and community to community, highlighting the need for new and tailored solutions.^72–74,81^ Strategies such as the Identify-Reach-Monitor-Measure-Advocate framework^82^ have been proposed as a way to develop and deliver community-specific plans to reach zero-dose children. Some key strategies that have yielded improved vaccine uptake include awareness and education, communication, mobility of vaccination units, community engagement, motivational incentives, positive reinforcement and assurance of vaccine safety.^83,84^ These efforts must be supported by strong evidence and data, including robust, timely, and local estimates of coverage and a better understanding of the location and characteristics of zero-dose children within each country.^85^

#### Vaccine hesitancy

Although some of the COVID-19 pandemic-specific challenges to vaccination have receded, several key challenges persist—including increasing disparities in resource-constrained, conflict-affected, or politically-volatile countries and territories,^86,87^ intensification of migration and displacement; and climate-related crises.^10,11^ An additional challenge to progress has been the threat of growing vaccine hesitancy. Deriving from many complex origins, vaccine misinformation^88,89^ and scepticism were already challenges before the pandemic, identified by WHO in 2019 as one of the ten leading threats to global health.^23,90,91^ The COVID-19 pandemic—which in many areas bred declining trust in public health institutions^92^ and polarised opinions about the necessity and safety of vaccination against COVID-19^89^ has had varying effects on public perceptions regarding the importance of routine childhood vaccination and willingness to vaccinate. A 2023 global analysis reported that vaccine hesitancy prevalence ranged from a low of 13.3% in the WHO Region of the Americas to a high of 27.9% in the Eastern Mediterranean region,^93^ and even higher in select African countries.^94^ In the United States of America (USA), most parents remained convinced of the benefits and effectiveness of childhood vaccines between 2020 and 2022, with confidence levels ranging from 89.5% to 92.5%, though concerns about vaccine safety and side effects increased over that time,^95^ and kindergarten vaccine exemption rates in 2023–24 are the highest ever reported.^96^

While overall confidence in routine childhood immunisation remains relatively high, the COVID-19 pandemic clearly exposed a vein of public distrust regarding health policy that is likely to influence public perception of childhood vaccines into the future.^91^ Strategies to improve vaccine confidence include bolstering scientific literacy to protect against an erosion of trust in science, implementing targeted public health campaigns to promote routine childhood immunisation, including community input in scientific research and policy making, engaging with community and religious leaders as advocates for immunization, and elevating and equipping health-care providers—who remain the most trusted voices on vaccination—to have impactful conversations about decisions to immunise.^25,90,91^

### Looking forward to Immunization Agenda 2030 targets

To track progress in vaccination across the life course, IA2030 targets include halving the global number of zero-dose children and achieving 90% global coverage of DTP3, PCV3, MCV2, and HPVc by 2030. (HPVc was not included in the present analysis due to the lack of currently available data.) These targets were ambitious at the time of their creation and present even greater challenges now, following the impact of the COVID-19 pandemic on global vaccination rates. For many countries and territories, the IA2030 targets may be achievable, though may require acceleration of progress. In the countries, territories, and super-regions with the largest numbers of un- and under-immunised children, however, achieving these targets would require extraordinary improvements in vaccination coverage. By 2023, numbers of zero-dose children remained higher than in 2019 in all super-regions except south Asia. Our forecasts indicate that achieving the ambitious IA2030 goal of 90% global coverage by 2030 for each of the life-course vaccines DTP3, PCV3, and MCV2^29^ is also unlikely. Moreover, coverage disparities seen in 2023 for DTP3 and MCV2 will persist in 2030, with coverage rates in sub-Saharan Africa remaining substantially below other super-regions.

Our analysis suggests similar challenges in meeting zero-dose reduction goals. Even if optimistic forecast scenarios were to be achieved, or if all countries and territories were to meet zero-dose reduction targets, these results suggest that substantial geographical disparities will persist in 2030—particularly for DTP3 and MCV2. For PCV3, coverage disparities will lessen due to ongoing introductions and scale-up, though would nevertheless persist even in the “better” scenario.

The success of the first 50 years of EPI has only been possible due to broad and sustained cooperation at all levels, from local health workers to national immunization programs to regional and global partnerships. At the global level, the WHO coordinates and provides vaccination guidance for all countries and territories and plays a central role in data collection. For example, this study relies on 49,710 vaccine-country-years of data reported by country offices that was collected, collated and reported annually by the WHO through the Joint Reporting Form,^97^ and these estimates additionally benefit from contextual insights regarding these coverage data generated by WUENIC.^56^ Gavi, the Vaccine Alliance, supports qualifying countries and territories in strengthening their immunisation programmes, and currently provides vaccines for routine immunisation in 54 countries and territories while they work towards more sustained domestic financing strategies.^98^ This paper demonstrates the substantial scaling up of newer vaccines in low- and middle-income countries and territories, and Gavi has played a central role in supporting these country-led introductions. The United States Agency for International Development (USAID) has also played a key role in monitoring vaccine coverage in low- and middle-income countries through the Demographic and Health Surveys, which provide a major source of population-based data about vaccination rates at national and local scales. This study includes data from 313 DHS surveys, which represent over half (50.2%) of all survey data sources included from low- and middle-income countries.

With the large-scale termination of USAID-supported programs, announced cuts to U.S. funding for Gavi and WHO, and a broader environment of decreased commitments to developmental assistance for health globally,^99–102^ the historical and future progress of vaccination programs is at risk. With reduced fiscal space, any further new vaccine introductions are in jeopardy, vaccination coverage rates may fall, and the risk of vaccine-preventable disease is heightened. In this time of risk, accurate estimates of vaccine coverage become even more important. With the closure of the DHS program, strategic and coordinated efforts to assess coverage through targeted surveys and support for country immunization data systems will be needed.

Childhood immunization is an outstanding investment with excellent returns in health and economic benefits, across countries and territories of all income levels.^103–106^ Proposed reductions in immunization spending are likely to disproportionally affect low- and middle-income countries, but high-income countries and territories are also likely to incur healthcare costs associated with new and more frequent disease outbreaks.^107,108^ Europe saw its highest number of measles cases in 2024 since 1997, and the first measles-related death in the last decade in the United States occurred in an unvaccinated child as part of a measles outbreak in Texas in early 2025.^109^ Without concerted efforts to bolster immunization rates in all countries and territories, these risks will continue to increase.^110^

Due to ongoing uncertainty about the final scope and magnitude of proposed funding cuts, the impacts of these decisions are not considered in our forecasts of the IA2030 life course vaccines. If these proposed funding cuts are fully implemented, however, the forecasts present here— which already illustrate that global coverage is not on track to reach the IA2030 targets—are likely too optimistic. Similarly, the scenario of “equal contribution” presented here with all countries and territories contributing proportionally to 50% zero-dose reduction targets becomes even more unlikely given the disproportionate impacts of additional funding constraints. Nevertheless, it is important to note that any gains in coverage and reduction of existing disparities—even if they fall short of the ambitious goals set by IA2030—would still result in massive public health gains. Each percentage point increase in global vaccination coverage represents protection for millions of additional children against deadly diseases. This perspective does not diminish the importance of ambitious targets, but rather emphasizes the substantial value of continued, incremental progress in all settings.

### Limitations

Our present estimates of routine childhood vaccination coverage are limited by various methodological considerations. First, although we applied models designed to adjust for bias in vaccine coverage data, we were not able to account for all potential sources of bias. Displaced or otherwise disenfranchised individuals may be under-represented in the data. Survey data may not accurately capture effects such as migration, catch-up vaccination, and differential survival by immunisation status across the age cohorts on which we based our analyses. Both surveys and country-reported data may be limited in their ability to assess immunization rates in conflict-affected areas, leading to potential overestimation absent data from these locations and time periods. Reporting of surveys and country-reported data was in many instances delayed by the COVID-19 pandemic; while the timing of this study has allowed for catch-up in reporting, decreases in survey participation rates and other forms of reporting biases during that time may have also occurred and are not reflected in this study. Data that relied on parental or observer recall are subject to recall bias, which we did not to adjust for, based on evidence indicating highly variable effects of recall bias on coverage estimates.^111–113^ Nor were we able to systematically account for methodological variability across surveys. We provide statistical uncertainty from our modelling framework to reflect confidence in the reported coverage values. Although ST-GPR and MR-BRT partially mitigate the limitations of data sparsity in select locations, estimates in such areas may demonstrate greater uncertainty. In this analysis, we produce estimates for all national locations included in the GBD 2023 study, including those for which data are sparse and estimates are uncertain. This approach follows the principles of the GBD study,^39^ which prioritize comprehensive comparisons and recognize that the absence of an estimate often results in the exclusion of that location from strategic planning and policy decisions. While we aim to select covariates plausibly linked to vaccination coverage, our models do not allow for potential interactions between covariates such as SDI and disruption magnitude or conflict. Due to the multi-step nature of ST-GPR, these limitations are likely to most affect data-sparse locations, where estimates are more heavily informed by covariates and regional trends. Similarly, while our modelled estimates of administrative bias allow variation in bias over time where multiple overlapping coverage observations from survey and country-reported data are available, estimates of trends in bias may be less reliable in data-sparse locations, or where abrupt discontinuities in administrative reporting methodology occur (eg, when countries switch to using electronic systems like DHIS2 for ascertaining vaccine delivery counts).^114^

Second, our reliance on DTP1 coverage as a measure of zero-dose children—although standard practice^9,115^—could overestimate the number of those who do not receive any childhood vaccinations, as children may in some cases receive other vaccinations even after missing DTP1. Similarly, our understanding of trends in zero-dose children rely on estimates and forecasts of populations. Following standard GBD practices, we did not account for uncertainty in these population estimates, and the uncertainty intervals for zero-dose and other counts included here thus represent uncertainty only in the associated coverage estimates. The uncertainty intervals for these count-based estimates, therefore, may underestimate the true degree of uncertainty in these measures.

Third, our analysis does not include data on vaccinations that take place outside EPI-designated routine childhood immunisation schedules, such as vaccinations administered in limited immunisation campaigns targeting specific populations or administered through private markets (except for selected private vaccinations in China, which are included in our analysis). Due to a lack of comprehensive data, our analysis does not account for catch-up vaccination activities, including global efforts through the “Big Catch-up” to reach children who missed routine immunisations during the COVID-19 pandemic.^65^ While age-specific survey data can provide some insight into catch-up vaccination, comprehensive administrative data are not available to inform such models. Additional efforts are needed to collect data and develop analytic methods to estimate coverage across all ages, including the impact of catch-up activities. Even so, catch-up activities represent a stop-gap measure that cannot replace improvements to routine healthcare services. Our results show that disruptions due to COVID-19 have had long-lasting and persistent impacts on routine services, which serve as the foundation for strong immunization programs.

Fourth, our analysis estimates the size of the impact of the COVID-19 pandemic on vaccine coverage by first estimating coverage in the absence of stockouts or any other disruptions to coverage. We then assume that, absent the COVID-19 pandemic, each country would have experienced the average degree of coverage disruption due to stockouts or other factors, as was observed from 2015 to 2019. This year range was selected to try to reflect the most recent patterns in disruptions, but nevertheless it is challenging to disentangle the ongoing historical trends from the impacts of the COVID-19 pandemic, and it is not necessarily the case that recent disruption trends would have continued apace. In particular, for countries and territories where large disruptions were common prior to the COVID-19 pandemic or coverage levels were changing rapidly, we may over- or under-estimate COVID-19 pandemic-related impacts. We have therefore presented the results of this post-hoc analysis in aggregate at super-region and global levels only. In this counterfactual estimation, we do not account for the impact of delays in the introduction of new vaccines that may have occurred due to the COVID-19 pandemic. For these newer vaccines, it is therefore likely that we may underestimate the global impact of the pandemic.

Fifth, we imputed disruption estimates for countries and territories without country-reported data during the COVID-19 pandemic, using vaccine- and year-specific distributions of disruption magnitudes from locations with available data. As our results show, however, actual disruption magnitudes varied widely. Our results in these country-vaccine-years may thus reflect either over- or underestimation of actual disruptions. In particular, many high-income countries and territories have incomplete data reporting during the COVID-19 pandemic and estimates of COVID-19 pandemic-related disruptions in these locations should be interpreted with caution.

Sixth, our process for estimating coverage of newer vaccines first as ratios relative to a reference vaccine (DTP3 for HepB3, Hib3, PCV3 and RotaC, and MCV1 for MCV2 and RCV1) constrains our estimates of these newer vaccines to be lower than that of their respective reference vaccines. For vaccines given in combination with the reference vaccine (eg, RCV1 and MCV1, or Hib3 and DTP3), or where these constraints are implied by definition (eg, MCV2 < MCV1, or DTP3 < DTP1), this assumption is appropriate. PCV and rotavirus vaccines, are typically given on the same schedule as DTP; however, coverage could exceed that of DTP3 in rare cases, particularly in the setting of intensive scale-up or vaccine-specific disruptions to DTP3 coverage. Future improvements to this work could consider independent modelling of PCV3 and/or RotaC coverage.

Although not a focus of the present analysis, characterising within-country differences in coverage is also important. Assessing coverage by critical sociodemographic factors—eg, by geography at subnational scales, wealth, education, women’s status, refugee status, and race and ethnicity—can help identify persistent disparities in routine childhood vaccination masked by the national overview estimates presented here. Similarly, although HPVc is one of IA2030’s 90% life-course vaccine coverage targets and has been recommended globally by WHO since 2009,^116^ we were not able to generate estimates or forecasts of HPVc coverage due to the lack of currently available survey data in most settings. Additional work will be needed to generate coverage estimates for HPVc and other vaccines not included here, using rigorous statistical frameworks that can leverage both survey and administrative data sources. Nor in this paper did we measure rates of under-vaccinated children, those who have received only some but not all vaccine-doses in their vaccination schedule, or assess patterns in the timeliness of vaccination. Comprehensive estimates of the full spectrum of vaccination, beyond the coverage metrics presented here, would improve understanding of population susceptibility and risks of disease outbreaks.

### Conclusions

Over the past 50 years, EPI has achieved extraordinary success in the urgent public health campaign to immunise the world’s children against life-threatening diseases. The next 50 years will require sustained efforts at global, regional, national, and community levels to successfully preserve and extend existing gains. Enduring coverage inequities and the persistent effects of the COVID-19 pandemic only serve to underscore the importance of advancing routine childhood vaccination, one of the most powerful public health interventions known.

Current trends and forecasts, along with proposed reductions to global immunization financing, suggest that reaching the ambitious goals of IA2030—aimed at reducing mortality and morbidity from vaccine-preventable diseases for everyone, everywhere—are unlikely to be realised unless the global community redoubles its commitment to equitable and universal vaccination strategies. Effective programmes and policies must integrate vaccination services into revitalised primary health-care systems, focus on context-specific and community-driven immunisation strategies, increase and optimise investment in vaccination, and prioritise community-led approaches to build vaccine confidence. Yet these present and future challenges should be met with firm confidence in the power and promise of vaccination, rooted in the successes of the past 50 years of EPI. It is vital that the global health community embrace our shared responsibility and whole-heartedly reaffirm our collective commitment to routine childhood vaccination to deliver on the promise of EPI to provide all people, everywhere the opportunity to live full and healthy lives.

## Supplementary Material

Appendix 1

Appendix 2

## Data Availability

This study follows the Guidelines for Accurate and Transparent Health Estimates Reporting (GATHER). To download the estimates produced in these analyses, please visit the Global Health Data Exchange website at https://ghdx.healthdata.org/record/ihme-data/gbd-2023-vaccination-coverage-1980-2030. Data sources are also listed by location and institution in appendix 1 (table S2).

## Research in context

### Evidence before this study

Accurate and comprehensive estimates of childhood immunisation coverage are essential to guide efforts to combat vaccine-preventable disease and to measure progress in the global campaign launched 50 years ago by EPI—the Expanded Programme on Immunization—to provide all children, everywhere, access to life-saving vaccines. Annual WHO–UNICEF Estimates of National Immunization Coverage (WUENIC), reliant on expert local knowledge and qualitative reasoning to compile and evaluate country-reported administrative and household survey data, have served as an important source of information on routine childhood vaccine coverage since 2000. Previous work from the Global Burden of Diseases, Injuries, and Risk Factors Study (GBD) further applied comprehensive statistical models to systematically analyse these disparate survey and country-reported coverage data sources in an overarching framework designed to address recurrent issues of data sparsity (ie, incomplete data from some countries and regions), heterogeneity (ie, variability in sampling frameworks), and bias (ie, systematic errors in data collection) and to formally quantify estimation uncertainty in vaccine coverage from 1980 to 2019. Both WUENIC and GBD estimates demonstrated marked global increases in coverage across vaccines since 1980, but slower progress and in some settings declines in coverage from 2010 to 2019. Subsequently, following the onset of the COVID-19 pandemic in 2020, numerous sources – including WHO global pulse surveys on continuity of essential health services, country-reported administrative data, WUENIC estimates, and GBD models – suggested large disruptions to vaccination services. Early GBD statistical models utilising monthly administrative data estimated large and heterogeneous disruptions to vaccination coverage during 2020, with variable recovery as the year progressed, leading to millions of children missing vaccine doses in that year. The Scorecard for the Immunization Agenda 2030 (IA2030), the comprehensive global vision and strategy to reduce death and illness from vaccine-preventable disease, demonstrates that as of 2023, global vaccine coverage levels had still not returned to pre-COVID-19 pandemic levels, and progress towards coverage targets for 2030 is not on track. Still missing, however, is a comprehensive, statistical, quantitative assessment of the long-term impacts of the COVID-19 pandemic on childhood routine immunization and its impact on progress towards the IA2030 targets.

### Added value of this study

Building on the established GBD evidentiary and analytic framework, the present study refines these models and extends this time series by leveraging additional years of data and improved analytic techniques to generate updated and extended estimates of annual routine childhood vaccination coverage in 204 countries and territories from 1980 to 2023 for 11 childhood vaccine-dose combinations—targeting diphtheria, tetanus, and pertussis (DTP1 and DTP3, ie, first and third doses), measles (MCV1 and MCV2), polio (Pol3, ie, any three doses of the polio vaccine), tuberculosis (BCG), hepatitis B (HepB3), *Haemophilus influenzae* type b (Hib3), *Streptococcus pneumoniae* (PCV3), rubella (RCV1), and rotavirus (RotaC, complete series).

We complement these estimates of historic coverage trends with three secondary analyses designed to contextualize recent disruptions in coverage due to COVID-19 and progress needed between now and 2030 to achieve important targets set by IA2030. In these analyses, we 1) assessed the impact of the COVID-19 pandemic on routine childhood immunisation during the years 2020 to 2023 by comparing declines in coverage attributable to the COVID-19 pandemic with coverage levels expected in the absence of the pandemic, 2) rigorously evaluated progress needed to achieve the IA2030 goal of a 50% reduction in numbers of “zero-dose children” (proxied as children under one year of age who have never received a dose of DTP1), and 3) forecasted the plausibility of achieving IA2030’s 90% global coverage targets for life-course vaccines by generating forecasts of DTP3, PCV3, and MCV2 coverage for the year 2030—the last representing the first forecasts published for PCV3 and MCV2 coverage.

### Implications of all the available evidence

The overarching public health benefits of the first 50 years of EPI have been immense, saving the lives of an estimated 154 million children and providing a total of 10·2 billion years of full health. This was achieved through a near doubling of global rates of immunisation against diphtheria, tetanus, pertussis, measles, polio, and tuberculosis between 1980 and 2023, a reduction in numbers of unvaccinated “zero-dose” children by more than 70%, and the introductions of a multitude of critical new vaccines and vaccine-dose combinations (including HepB3, Hib3, MCV2, PCV3, RotaC and RCV1). However, substantial disparities persist, including markedly lower coverage and higher rates of under- and un-vaccinated children in the low- and middle-income countries—especially sub-Saharan Africa—with over 52·6% (95% uncertainty interval [UI] 51·4–53·8) of zero-dose children living in sub-Saharan Africa and 12·5% (11·4–14·8) living in south Asia. Moreover, stagnating immunisation progress worldwide after 2010, COVID-19 pandemic-related decreases in coverage for all five original EPI vaccine-dose combinations (BCG, DTP1, DTP3, MCV1 and Pol3), and increases in the number of zero-dose children that have persisted into 2023 make it unlikely that ambitious IA2030 goals will be reached unless considerable course correction occurs. Enduring and emerging socioeconomic inequities related to rising numbers of displaced people and growing disparities due to armed conflict, political volatility, economic uncertainty, climate-related crises, and vaccine misinformation and hesitancy stand as fundamental obstacles to extending equitable vaccine coverage.

Advancing equitable childhood vaccination will require both collective global engagement and the input of local stakeholders to shape vaccination strategies responsive to context-specific realities and to build confidence in immunisation policies. It will require the political and financial will to ensure robust primary health-care systems that can support the strong, resilient, equitable immunisation programmes needed to continue delivering existing vaccines, to serve as a platform to provide new vaccines as they become available, and to expand their reach and promise to all.

## Collaborators

Emily Haeuser*, Sam Byrne, Jason Nguyen, Catalina Raggi, Susan A McLaughlin, Catherine Bisignano, Ashley A Harris, Amanda E Smith, Paulina A Lindstedt, Georgia Smith, Samuel James Herold, Olivia D Nesbit, Taylor Noyes, Noga Shalev, Latera Tesfaye Olana, Mohammad Amin Aalipour, Hasan Aalruz, Mitra Abbasifard, Faezeh Abbaspour, Hedayat Abbastabar, Samar Abd ElHafeez, Emad M. Abdallah, Reda Abdel-Hameed, Atef Abdelkader, Sherief Abd-Elsalam, Wakgari Mosisa Abdisa, Meriem Abdoun, Arman Abdous, Deldar Morad Abdulah, Adam Abdullahi, Auwal Abdullahi, Toufik Abdul-Rahman, Kulmira Abdykerimova, Armita Abedi, Asrat Agalu Abejew, Roberto Ariel Abeldaño Zuñiga, Syed Hani Abidi, Olumide Abiodun, Rahim Abo Kasem, Richard Gyan Aboagye, Hassan Abolhassani, Ulric Sena Abonie, Abdullahi Tunde Aborode, Nagah Mohamed Abourashed, Mohamed Abouzid, Dmitry Abramov, Lucas Guimarães Abreu, Dariush Abtahi, Rana Kamal Abu Farha, Bilyaminu Abubakar, Eman Abu-Gharbieh, Hana J Abukhadijah, Salahdein Aburuz, Anirudh Balakrishna Acharya, Meshack Achore, Juan Manuel Acuna, Ousman Adal, Lisa C. Adams, Abdu A Adamu, Tajudeen Adesanmi Adebisi, David Adedia, Kamoru Ademola Adedokun, Oluwatobi Emmanuel/E Adegbile, Oyelola A Adegboye, Nurudeen A Adegoke, Olumide Thomas Adeleke, Juliana Bunmi Adetunji, Mache Tsadik Adhana, Ripon Kumar Adhikary, Usha Adiga, Mohd Adnan, Qorinah Estiningtyas Sakilah Adnani, Prince Owusu Adoma, Leticia Akua Adzigbli, Giuseppina Affinito, Aanuoluwapo Adeyimika Afolabi, Habeeb Abiodun Afolabi, Rotimi Felix Afolabi, Saira Afzal, Suneth Buddhika Agampodi, Dhiraj Motilal Agarwal, Sepehr Aghajanian, Constanza Elizabeth Aguilera Arriagada, Williams Agyemang-Duah, Mahsa Ahadi, Aqeel Ahmad, Danish Ahmad, Khurshid Ahmad, Rabbiya Ahmad, Shoaib Ahmad, Tauseef Ahmad, Ayman Ahmed, Haroon Ahmed, Meqdad Saleh Ahmed, Muktar Beshir Ahmed, Mushood Ahmed, Naveed Ahmed, Syed Anees Ahmed, Simeon Okechukwu Ajakwe, Dolapo Emmanuel Ajala, Gizachew Taddesse Akalu, Oluwasefunmi Akeju, Roland Eghoghosoa Akhigbe, Karolina Akinosoglou, Mohammed Ahmed Akkaif, Hammad Akram, Ashley E Akrami, Alaa Al Amiry, Salah Al Awaidy, Hanadi Al Hamad, Mohammad Khaled Al nawayseh, Omar Al Omari, Yazan Al Thaher, Omar Ali Mohammed Al Zaabi, Mohammad Ahmmad Mahmoud Al Zoubi, Yazan Al-Ajlouni, Ziyad Al-Aly, Khurshid Alam, Mohammad Khursheed Alam, Nazmul Alam, Rasmieh Mustafa Al-amer, Turki M Alanzi, Jude Oluwapelumi Alao, Fahmi Y. Al-Ashwal, Mohammed Albashtawy, Mohammad T AlBataineh, Abdulelah Mastour Aldhahir, Mohammed S Aldossary, Shereen M. Aleidi, Tekletsadik Tekleslassie Alemayehu, Ayman Al-Eyadhy, Ali M Alfalki, Fahad D Algahtani, Abdelazeem M Algammal, Ashraf Alhumaidi, Abid Ali, Irfan Ali, Liaqat Ali, Mohammad Daud Ali, Rafat Ali, Shahid Ali, Syed Shujait Ali, Montaha Al-Iede, Sheikh Mohammad Alif, Hamid Alinejad Rokny, Morteza Alipour, Samah W Al-Jabi, Adel Al-Jumaily, Ahmad Alkhatib, Mustafa Alkhawam, Mohammed Z. Allouh, Wesam Taher Almagharbeh, Sabah Al-Marwani, Joseph Uy Almazan, Hesham M Al-Mekhlafi, Amr Almobayed, Hasan Yaser Alniss, Mohammad R. Alosta, Jaber S Alqahtani, Mohammad R. Alqudimat, Ahmed Yaseen Alqutaibi, Ahmad Alrawashdeh, Rami H Al-Rifai, Intima Alrimawi, Sahel Majed Alrousan, Mohammed A Alsabri, Zaid Altaany, Alaa B. Al-Tammemi, Jaffar A Al-Tawfiq, Malik A Althobiani, Khalid A Altirkawi, Nelson Alvis-Guzman, Nelson J Alvis-Zakzuk, Hassan Alwafi, Mohammad Al-Wardat, Yaser Mohammed Al-Worafi, Hany Aly, Mohammad Sharif Ibrahim Alyahya, Abdallah Alzoubi, Karem H Alzoubi, Md. Akib Al-Zubayer, Ekiyor Joseph Amafah, Amr Amin, Saeed Amini, Nafiu Aminu, Ayodeji Amobonye, Dickson A Amugsi, Filippos Anagnostakis, Michael Anderson, Song Peng Ang, Nguyen Hoang Anh, Abhishek Anil, Abdul-Azeez Adeyemi Anjorin, Hossein Ansariniya, Catherine M Antony, Boluwatife Stephen Anuoluwa, Saeid Anvari, Saleha Anwar, Jalal Arabloo, Jesil Mathew Aranjani, Aleksandr Y Aravkin, Demelash Areda, Abdulfatai Aremu, Olatunde Aremu, Ghazal Arjmand, Jesu Arockiaraj, Mahwish Arooj, Anton A Artamonov, Ashokan Arumugam, Deepavalli Arumuganainar, Nurila Aryntayeva, Mahsa Asadi Anar, Muhammad Asaduzzaman, Syed Mohammed Basheeruddin Asdaq, Shewatatek Melaku Melaku Asefa, Akram Ashames, Tahira Ashraf, Mitra Ashrafi, Bernard Kwadwo Yeboah Asiamah-Asare, Muhammad Shahzad Aslam, Saeed Aslani, Yuni Asri, Dereje Zewdu Assefa, Batyrbek Assembekov, Sachin R Atre, Alok Atreya, Julie Alaere Atta, Zeenah A Atwan, Matteo Augello, Khursheed Aurangzeb, Andargie Abate Awoke, Babafela B Awosile, Seyyed HamidReza Ayatizadeh, Yusuf Oloruntoyin Ayipo, Sina Azadnajafabad, Mohd Yusmaidie Aziz, Sadat Abdulla Aziz, Amin Azizan, Ahmed Y. Azzam, Abisola Esther Babatope, Rasha Babiker, Ashish D Badiye, Sara Bagheri, Fereshteh Baghizadeh, Razieh Bahreini, Yogesh Bahurupi, Atif Amin Baig, Senthilkumar Balakrishnan, Maher Balkis, Rajon Banik, Hansi Bansal, Shirin Barati, Hiba Jawdat Barqawi, Zarrin Basharat, Shahid Bashir, Azadeh Bashiri, Rehana Basri, Quique Bassat, Mohammad-Mahdi Bastan, Saurav Basu, Kavita Batra, Ravi Batra, Mahdis Bayat, Narasimha M Beeraka, Bezawit K Bekele, Tariku Tesfaye Bekuma, Sewunet admasu Belachew, Asnake Gashaw Belayneh, Melesse Belayneh, Michael Belingheri, Umar Muhammad Bello, Samiun Nazrin Bente Kamal Tune, Abiye Assefa Berihun, Amiel Nazer C Bermudez, Robert S Bernstein, Ajeet Singh Bhadoria, Akshaya Srikanth Bhagavathula, Neeraj Bhala, Dinesh Bhandari, Pankaj Bhardwaj, Ashish Bhargava, Sonu Bhaskar, Priyadarshini Bhattacharjee, Krittika Bhattacharyya, Ashmin Hari Bhattarai, Jasvinder Singh Bhatti, Can Bilgin, Saeed Biroudian, Bijit Biswas, Mohammad Shahangir Biswas, Monirujjaman Biswas, Molalegne Bitew, Bruno Bizzozero-Peroni, Firew Tekle Bobo, Trupti Bodhare, Lucimere Bohn, Obasanjo Afolabi Bolarinwa, Paria Bolourinejad, Alejandro Botero Carvajal, Souad Bouaoud, Dejana Braithwaite, Hermann Brenner, Nikolay Ivanovich Briko, Danilo Buonsenso, Felix Busch, Yasser Bustanji, Nadeem Shafique Butt, Zahid A Butt, Mehtap Çakmak Barsbay, Luis Alberto Cámera, Angelo Capodici, Giulia Carreras, Andrea Carugno, Felix Carvalho, Joao Mauricio Castaldelli-Maia, Carlos A Castañeda-Orjuela, Luca Cegolon, Francieli Cembranel, Muthia Cenderadewi, Muge Cevik, Chiranjib Chakraborty, Sandip Chakraborty, Rama Mohan Chandika, Vijay Kumar Chattu, Galmesa Bekana Chemeda, An-Tian Chen, Hana Chen, Haowei Chen, Nicholas WS Chew, Patrick R Ching, William C S Cho, Bryan Chong, Hitesh Chopra, Shivani Chopra, Dinh-Toi Chu, Sheng-Chia Chung, Sunghyun Chung, Muhammad Chutiyami, Alyssa Columbus, Joao Conde, Alexandru Corlateanu, Claudia Cosma, Natalia Cruz-Martins, Alanna Gomes da Silva, Bashir Dabo, Omid Dadras, Xiaochen Dai, Emanuele D’Amico, Lalit Dandona, Rakhi Dandona, Lucio D’Anna, Samuel Demissie Darcho, Latefa Ali Dardas, Gary L Darmstadt, Aso Mohammad Darwesh, Dimash Davletov, Fernando Pio De la Hoz, Sindhura Deekonda, Aniket Dehadrai, Tadesse Asmamaw Dejenie, Marco Del Riccio, Mohammad Delsoz, Huiyin Deng, Edgar Denova-Gutiérrez, Anteneh Assefa Desalegn, Pradeep Kumar Devarakonda, Syed Masudur Rahman Dewan, Arkadeep Dhali, Kuldeep Dhama, Meghnath Dhimal, Sameer Dhingra, Bibha Dhungel, Stefano Di Bella, Marcello Di Pumpo, Diana Dias da Silva, Daniel Diaz, Xueting Ding, Thanh Chi Do, Sushil Dohare, Fariba Dorostkar, Wendel Mombaque dos Santos, Ojas Prakashbhai Doshi, Robert Kokou Dowou, Menayit Tamrat Dresse, John Dube, Senbagam Duraisamy, Oyewole Christopher Durojaiye, Sulagna Dutta, Osamudiamen Ebohon, Lamiaa Labieb Mahmoud Ebraheim, Mohammad Hossein --- Ebrahimi, Rasoul Ebrahimi, Abdelaziz Ed-Dra, Ferry Efendi, Behrad Eftekhari, Ashkan Eighaei Sedeh, Ebrahim Eini, Michael Ekholuenetale, Rabie Adel El Arab, Ibrahim Farahat El Bayoumy, Maysaa El Sayed Zaki, Mohamed Ahmed Eladl, Aya Elalfy, Said El-Ashker, Iffat Elbarazi, Noha Mousaad Elemam, Muhammed Elhadi, Mohamed Hassan Elnaem, Mohammed Elshaer, Abdelgawad Salah Abdelgawad Eltahawy, Theophilus I Emeto, Talha Bin Emran, Misganu Endriyas, Setegn Eshetie, Gilbert Eshun, Sharareh Eskandarieh, Majid Eslami, Maysa Eslami, Fahima Nasrin Eva, Adewale Oluwaseun Fadaka, Heidar Fadavian, Adeniyi Francis Fagbamigbe, Ayesha Fahim, Razana Faiz, Ildar Ravisovich Fakhradiyev, Niloofar Faraji, Seyed Nooreddin Faraji, Mahsa Faramarzpour, Mohammad Fareed, MoezAlIslam Ezzat Mahmoud Faris, Andre Faro, Syed Muhammad Yousaf Farooq, Emmanuel Toluwani Fasusi, Zareen Fatima, Pooria Fazeli, Alireza Feizkhah, Ginenus Fekadu, Ulrich Membe Femoe Membe, Rodrigo Fernandez-Jimenez, Natan Feter, Claudio Fiorilla, Florian Fischer, Marco Fonzo, Takeshi Fukumoto, Nancy Fullman, Muktar A Gadanya, Dominic Dormenyo Gadeka, Márió Gajdács, Balasankar Ganesan, Xiang Gao, Bashiru Garba, Fernando Barroga Garcia, Jacopo Garlasco, Anteneh Gashaw, Zisis Gatzioufas, Rupesh K Gautam, Feven Sahle Gebre, Miglas Welay Gebregergis, Nsikakabasi Samuel George, Ubong Uwem George, Gebremariam Wulie Geremew, Genanew K Getahun, Habtamu Abebe Getahun, Fekadeselassie Belege Getaneh, Gebremariam Getaneh, Kazem Ghaffari, Roya Ghafoury, Arin Ghamkhar, Shakiba Ghasemi Assl, Haniyeh Ghasrsaz, Ramy Mohamed Ghazy, Gloria Gheno, Nermin Ghith, Arun Ghuge, Artyom Urievich Gil, Alessandro Girombelli, Laszlo Göbölös, Amit Goel, Mahaveer Golechha, Davide Golinelli, MReza Goodarzian, Aman Goyal, Shi-Yang Guan, Giovanni Guarducci, Stefano Guicciardi, Amit Gulati, Sasidhar Gunturu, Anish Kumar Gupta, Ishita Gupta, Sapna Gupta, Veer Bala Gupta, Vivek Kumar Gupta, Reyna Alma Gutiérrez, Roberth Steven Gutiérrez-Murillo, Jose Guzman-Esquivel, Annie Haakenstad, Awoke Derbie Habteyohannes, Dariush Haghmorad, Haimanot Ewnetu Hailu, Pritam Halder, Islam M Hamad, Nadia M Hamdy, Sajid Hameed, Samer Hamidi, Asif Hanif, Nasrin Hanifi, Graeme J Hankey, Harapan Harapan, Arief Hargono, Josep Maria Haro, Ahmed I Hasaballah, Mohammad Jahid Hasan, Hamidreza Hasani, Mohammad Hashem Hashempur, Ammarah Hasnain, Ibrahim Nagmeldin Hassan, Md. Imtaiyaz Hassan, Muhammad Hassan, Nageeb Hassan, Khezar Hayat, Jiawei He, Wen-Qiang He, Mohamed I Hegazy, Golnaz Heidari, Mohammad Heidari, Minoo Heidari Almasi, Sumudu Avanthi Hewage, Majid Heydari, Kamal Hezam, Yuta Hiraike, Alamgir Hossain, Lubna Hossain, Md Mahbub Hossain, Md Sabbir Hossain, Md. Jubayer Hossain, Mehdi Hosseinzadeh, Md Munnaf Hossen, Mihaela Hostiuc, Priya Hotwani, Hanno Hoven, Chengxi Hu, Junjie Huang, Kiavash Hushmandi, Javid Hussain, M. Azhar Hussain, Nawfal R Hussein, Mohamed Ibrahim Husseiny, Hong-Han Huynh, Bing-Fang Hwang, Segun Emmanuel Ibitoye, Khalid S Ibrahim, Nuheila Ibrahim, Anel Ibrayeva, Olayinka Stephen Ilesanmi, Irena M Ilic, Milena D Ilic, Mohammad Tarique Imam, Arit Inok, Mustafa Alhaji Isa, Benni Iskandar, Teresa R. Iskander, Md Sahidul Islam, Md. Fakrul Islam, Sheikh Mohammed Shariful Islam, Faisal Ismail, Leila Ismail, Mosimah Charles Ituka, Masao Iwagami, Chinwe Juliana Iwu-Jaja, Louis Jacob, Ali Jadidi, Abdollah Jafarzadeh, Haitham Jahrami, Ayushi Jain, Ammar Abdulrahman Jairoun, Mihajlo Jakovljevic, Mohamed Jalloh, Armaan Jamal, Qazi Mohammad Sajid Jamal, Melika Jameie, Jerin James, Hasan Jamil, Roland Dominic G Jamora, Syed Sarmad Javaid, Talha Jawaid, Qassim Jawell Odah Abed, Shubha Jayaram, Seogsong Jeong, Ravi Prakash Jha, Wenyi Jin, Mohammad Jokar, Jobin Jose, Jobinse Jose, Nitin Joseph, Charity Ehimwenma Joshua, Kripa Josten, Farahnaz Joukar, Jacek Jerzy Jozwiak, Zubair Kabir, Vidya Kadashetti, Dler H. Hussein Kadir, Ashish Kumar Kakkar, Md Moustafa Kamal, Mehnaz Kamal, Rajesh Kamath, Ramat T. Kamorudeen, Naser Kamyari, Oleksandr Kamyshnyi, Mona Kanaan, Saddam Fuad Kanaan, Jiseung Kang, Samuel Berchi Kankam, Kehinde Kazeem Kanmodi, Suthanthira Kannan S, Rami S Kantar, Neeti Kapoor, Jafar Karami, Reema A Karasneh, Ibraheem M Karaye, André Karch, Mohmed Isaqali Karobari, Tomasz M. Karpiński, Manoj Kumar Kashyap, Himanshu Khajuria, Mohammad Ali Khaksar, Nauman Khalid, Anees Ahmed Khalil, Faham Khamesipour, Abdul Arif Khan, Ajmal Khan, Faiz Ullah Khan, Gulfaraz Khan, Maseer Khan, Md Abdullah Saeed Khan, Moien AB Khan, Muhammad Umer Khan, Ramsha Mushtaq Khan, Sumaiya Khan Khan, Ubaid Khan, Yusuf Saleem Khan, Zahid Khan, Vishnu Khanal, Sameer Uttamaro Khasbage, Khaled Khatab, Haitham Khatatbeh, Moawiah Mohammad Khatatbeh, Afshin Khazaei, Khalid A Kheirallah, Farbod Khosravi, Grace Kim, Jinho Kim, Kwanghyun Kim, Min Seo Kim, Ruth W Kimokoti, Yohannes Kinfu, Adnan Kisa, Sezer Kisa, Shivakumar KM, Sonali Kochhar, Michail Kokkorakis, Ali-Asghar Kolahi, Farzad Kompani, Vladimir Andreevich Korshunov, Oleksii Korzh, Karel Kostev, Parvaiz A Koul, Irene Akwo Kretchy, James-Paul Kretchy, Kewal Krishan, Barthelemy Kuate Defo, Raja Amir Hassan Kuchay, Mohammed Kuddus, Ilari Kuitunen, Mukhtar Kulimbet, Emmanuel Kumah, Dewesh Kumar, G Anil Kumar, Jogender Kumar, Kamal Kumar, Narendar Kumar, Rakesh Kumar, Satyajit Kundu, Setor K Kunutsor, Maria Dyah Kurniasari, Pramod Kumar Kushawaha, Asep Kusnali, Dian Kusuma, Assylkhan Kuttybayev, Wai Hang Patrick Kwong, Frank Kyei-Arthur, Ville Kytö, Dr Pallavi L C, Carlo La Vecchia, Muhammad Awwal Ladan, Chandrakant Lahariya, Balzhan Lakanova, Iván Landires, Savita Lasrado, Colleen L Lau, Huu-Hoai Le, Minh Huu Nhat Le, Nhi Huu Hanh Le, Trang Diep Thanh Le, Caterina Ledda, Sergey Vadimovich Lee, Seung Won Lee, Wei-Chen Lee, Awol Yemane Legesse, Elvynna Leong, Ming-Chieh Li, Peng Li, Wei Li, Virendra S Ligade, Jialing Lin, John C Lin, Queran Lin, Gang Liu, Haipeng Liu, Jue Liu, Patrick Y Liu, Xuefeng Liu, Zhe Liu, Erand Llanaj, José Francisco López-Gil, Platon D Lopukhov, Giancarlo Lucchetti, Abhilash Ludhiadch, Peng Luo, Angelina M Lutambi, Lei Lv, Kaung Suu Lwin, Miltiadis D. Lytras, Ellina Lytvyak, Ahmed M. Afifi, Kevin Sheng-Kai Ma, Zheng Feei Ma, Shamsuddeen Yusuf Yusuf Ma’aruf, Mahmoud Mabrok, Monika Machoy, Firoozeh Madadi, Farzan Madadizadeh, Seyed Ataollah Madinezad, Aurea Marilia Madureira-Carvalho, Sasikumar Mahalingam, Samatar Abshir Mahamed, Nozad H. Mahmood, Mansour Adam Mahmoud, Farhad Mahmoudi, Hardeep Singh Malhotra, Ahmad Azam Malik, Shahid Malik, Tabarak Malik, Deborah Carvalho Malta, Biniyam Tedla Tedla Mamo, Lokesh Manjani, Kamaruddeen Mannethodi, Farheen Mansoor, Mohammad Ali Mansournia, Shaista Manzoor, Tahir Maqbool, Bishnu P Marasini, Hamid Reza Marateb, Konstantinos Margetis, Michael Marks-Hultström, Adolfo Martinez-Valle, Francisco Rogerlândio Martins-Melo, Miquel Martorell, Roy Rillera Marzo, Sammer Marzouk, Stefano Masi, Clara N Matei, Yasith Mathangasinghe, Medha Mathur, Neeta Mathur, Fernanda Penido Matozinhos, Richard James Maude, Chioma Ngozichukwu Pauline Mbachu, Ikechukwu Innocent Mbachu, Steven M McPhail, María Paz Medel Salas, Rishi P Mediratta, Vini Mehta, Subhash Mehto, James Meiring, Tesfahun Mekene Meto, Tesfaye Hambisa Mekonnen, Hadush Negash Meles, Endalkachew Belayneh Melese, Ziad Ahmed Memish, Walter Mendoza, Godfred Antony Menezes, Ritesh G Menezes, Emiru Ayalew Mengistie, Leweyehu Alemaw Mengstie, Alexios-Fotios A Mentis, Sultan Ayoub Ayoub Meo, Atte Meretoja, Tomislav Mestrovic, Sachith Mettananda, Mohamed M.M. Metwally, Irmina Maria Michalek, Giuseppe Minervini, Wai-kit Ming, Andreea Mirica, Alireza Mirkheshti, Vinaytosh Mishra, Heba M. Mohamed, Hebatalla Mohamed, Jama Mohamed, Mona Gamal Mohamed, Nouh Saad Mohamed, Khabab Abbasher Hussien Abbasher Hussien Mohamed Ahmed, Taj Mohammad, Abdolreza Mohammadi, Shafiu Mohammed, Yahaya Mohammed, Syam Mohan, Mohammad Mohseni, Amin Mohsenzadeh, Ali H Mokdad, Peyman Mokhtarzadehazar, Lorenzo Monasta, Mohammad Ali Moni, Maryam Moradi, Yousef Moradi, Paula Moraga, Anthony Kwame Morgan, Shane Douglas Morrison, Mahmoud M Morsy, Seyed Ahmad Mousavi, Seyed Mohamad Sadegh Mousavi Kiasary, Hagar Mowafy, Kimia Mozahheb Yousefi, Rabia Mubarak, Sumaira Mubarik, Ulrich Otto Mueller, Sumoni Mukherjee, Francesk Mulita, Mulyadi Mulyadi, Kavita Munjal, Anjana Munshi, Christopher J L Murray, Fungai Musaigwa, Sherzad Ibrahim Mustafa, Mubarak Taiwo Mustapha, Saravanan Muthupandian, Claude Mambo Mambo Muvunyi, Muhammad Muzaffar, Woojae Myung, Ayoub Nafei, Pirouz Naghavi, Amirhossein Naghibzadeh, Mobin Naghshbandi, Ganesh R Naik, Gurudatta Naik, Firzan Nainu, Tapas Sadasivan Nair, Soroush Najdaghi, Hastyar Hama Rashid Najmuldeen, Arindam Nandi, Sreenivas Narasimha Swamy, Shumaila Nargus, Abdulqadir J Nashwan, Mahmoud Nassar, Zuhair S Natto, Zakira Naureen, Samidi Nirasha Kumari Navaratna, Biswa Prakash Nayak, Shalini Ganesh Ganesh Nayak, Md Fahad Shahariar Nayon, Athare Nazri-Panjaki, Pacifique Ndishimye, Ionut Negoi, Samata Nepal, Henok Biresaw Netsere, Kieu Viet Nhi Nguyen, Nhan Nguyen, Nhien Ngoc Y Nguyen, Quan Nguyen Khoi, Nguyen Ngoc Yen Nhi, Robina Khan Niazi, Luciano Nieddu, Afewerki Tesfahunegn Tesfahunegn Nigusse, Ali Nikoobar, Behnaz Niroomand, Chukwudi A Nnaji, Shuhei Nomura, Syed Toukir Ahmed Noor, Masoud Noroozi, Valentine C. Nriagu, Chisom Adaobi Nri-Ezedi, Jean Claude Nshimiyimana, Fred Nugen, Mengistu H Nunemo, Felix Kwasi Nyande, Bogdan Oancea, Mary Aigbiremo Oboh, Ramez M. Odat, Ismail A Odetokun, Michael Safo Oduro, Tunde Emmanuel Ogundare, Oluwafunmilayo Tosin Ogundeko-Olugbami, Olusegun Olatunji Ojedoyin, Akinkunmi Paul Okekunle, Onyedika A Okoli, Osaretin Christabel Okonji, John Olayemi Okunlola, Oluyemi Adewole Okunlola, Oluwaseyi Isaiah Olabisi, Antonio Olivas-Martinez, Gláucia Maria Moraes Oliveira, Abdulhakeem Abayomi Olorukooba, Samson Bamidele Olorunju, Comfort Z. Z Olorunsaiye, Bolajoko Olubukunola Olusanya, Oluwafemi G. Gabriel Oluwole, Folorunsho Bright Omage, Obinna E Onwujekwe, Chizaram A Onyeaghala, Marcel Opitz, Michal Ordak, Verner N Orish, Atakan Orscelik, Alberto Ortiz, Edgar Ortiz-Brizuela, Esteban Ortiz-Prado, Augustus Osborne, Eric Osei, Elham H. Othman, Oche Joseph Otorkpa, Amel Ouyahia, Mayowa O Owolabi, Kolapo Oyebola, Tope Oyelade, Oyetunde T Oyeyemi, Ilker Ozsahin, Jagadish Rao Padubidri, Yeganeh Pakbaz, Tamás Palicz, Sujogya Kumar Panda, Georgios D Panos, Leonidas D. D Panos, Mario Virgilio Papa, Ilias Papadimopoulos, Shahina Pardhan, Utsav Parekh, Romil R Parikh, Chulwoo Park, Roberto Passera, Mitesh Patel, Neel Navinkumar Patel, Shankargouda Patil, Dimitrios Patoulias, Shrikant Pawar, Shubhadarshini Pawar, Hamidreza Pazoki Toroudi, Jarmila Pekarcikova, Veincent Christian Filipino Pepito, Prince Peprah, Gavin Pereira, Gladymar Perez Chacon, Simone Perna, Pavlo Petakh, Olumuyiwa James Peter, Nhat Truong Pham, Tung Thanh Pham, Zahra Zahid Piracha, Edoardo Pirera, Dimitri Poddighe, Roman V Polibin, Ramesh Poluru, Sajjad Pourasghary, Reza Pourbabaki, Farzad Pourghazi, Naeimeh Pourtaheri, Ashwathi Prakash, Elton Junio Sady Prates, Jyotirekha Purohit, Jagadeesh Puvvula, Husam Qanash, Nameer Hashim Qasim, Asma Saleem Qazi, Xiang Qi, Zhipeng Qi, Gangzhen Qian, Navid Rabiee, Basuki Rachmat, Venkatraman Radhakrishnan, Fakher Rahim, Sajjad Rahimi, Vafa Rahimi-Movaghar, Fryad Majeed Rahman, Md. Mosfequr Rahman, Md. Obaidur Rahman, Mosiur Rahman, Muhammad Aziz Rahman, Saeed Rahmani, Hakim Rahmoune, Sunil Kumar Raina, Jeffrey Pradeep Raj, Adarsh Raja, Gunaseelan Rajendran, Judah Rajendran, Mohammad Amin Rajizadeh, Siddheesh Rajpurohit, Mahmoud Mohammed Ramadan, Chitra Ramasamy, Shakthi Kumaran Ramasamy, Kamleshun Ramphul, Kirtan Rana, Rishabh Kumar Rana, Chhabi Lal Ranabhat, Nemanja Rancic, Smitha Rani, Chythra R Rao, Sowmya J Rao, Md. Abdur Rashid, Mohammad-Mahdi Rashidi, Azad Rasul, Devarajan Rathish, Abdur Rauf, Santosh Kumar Rauniyar, David Laith Rawaf, Salman Rawaf, Elrashdy Redwan, Wajiha Rehman, Luis Felipe Reyes, Mina Rezaei, Nazila Rezaei, Mohsen Rezaeian, Abanoub Riad, Moattar Raza Rizvi, Hannah Elizabeth Robinson-Oden, Hermano Alexandre Lima Rocha, Thales Philipe Rodrigues da Silva, Leonardo Roever, Amirhossein Roshanshad, Himanshu Sekhar Rout, Shiva Rouzbahani, Adrija Roy, Nitai Roy, Sharmistha Roy, Shubhanjali Roy, Guilherme de Andrade Ruela, Godfrey M Rwegerera, Aly M A Saad, Maha Mohamed Saber-Ayad, Seyed Kiarash Sadat Rafiei, Basema Ahmad Saddik, Tarannom Sadegh, Fatemeh Sadeghi-Ghyassi, Mohd Saeed, Umar Saeed, Mehdi Safari, Mastooreh Sagharichi, Dominic Sagoe, Narjes Saheb Sharif-Askari, S. Mohammad Sajadi, Md Refat Uz Zaman Sajib, Mirza Rizwan Sajid, Morteza Saki, Dorsa Salabat, Nasir Salam, Afeez Abolarinwa Salami, Mohamed A Saleh, Mahdi Salehi, Aanuoluwa James Salemcity, Dauda Salihu, Sohrab Salimi, Pegah Salimi Pormehr, Malik Sallam, Hossein Samadi Kafil, Saad Samargandy, Yoseph Leonardo Samodra, Nahom Samuel Samuel, Abdallah M Samy, Sathish Sankar, Adekunle Sanyaolu, Jacob Owusu Sarfo, Hemen Sarma, Mohammad Sarmadi, Sachin C Sarode, Brijesh Sathian, Maheswar Satpathy, Mehrdad Savabi Far, Monika Sawhney, Ganesh Kumar Saya, Christophe Schinckus, Ione Jayce Ceola Schneider, Art Schuermans, Amin Sedigh, Mohammad H Semreen, Sabyasachi Senapati, Ashenafi Kibret Sendekie, Pallav Sengupta, Yigit Can Senol, Subramanian Senthilkumaran, Dragos Serban, Yashendra Sethi, Seyed Mohammad Seyed Alshohadaei, Abubakar Sha’aban, Muhammad Shahab, Samiah Shahid, Syed Ahsan Ahsan Shahid, Wajeehah Shahid, Farshad Shahkarami, Fatemeh Shahrahmani, Moyad Jamal Shahwan, Ahmed Shaikh, Masood Ali Shaikh, Nafhat Shaikh, Alireza Shakeri, Mehran Shams-Beyranvand, Mohammad Ali Shamshirgaran, Anas Shamsi, Alfiya Shamsutdinova, Dan Shan, Mohammed Shannawaz, Amin Sharifan, Bunty Sharma, Manoj Sharma, Vishal Sharma, Ramzi Shawahna, Maryam Shayan, Suchitra M Shenoy, Samendra P Sherchan, Shiran Shetty, Md. Monir Hossain Shimul, Aminu Shittu, Zahra Shokati Eshkiki, Azad Shokri, Sina Shool, Seyed Afshin Shorofi, Kerem Shuval, Zahra Siavashpour, Emmanuel Edwar Siddig, Ayesha Siddiqua, Gustavo Correia Basto da Silva, Luís Manuel Lopes Rodrigues Silva, Amit Singh, Baljinder Singh, Bhim Pratap Singh, Harmanjit Singh, Jasvinder A Singh, Kalpana Singh, Poornima Suryanath Singh, Samer Singh, Satwinder Singh, Mukesh Kumar Sinha, Ebrahim Abdela Siraj, Natia Skhvitaridze, Valentin Yurievich Skryabin, Md.Salman Sohel, Anton Sokhan, Ahmed M. Soliman, May Mohamed Sherif Soliman, Noha Salah Soliman, Sameh S M Soliman, Weiyi Song, Aayushi Sood, Prashant Sood, Soroush Soraneh, Reed J D Sorensen, Michele Sorrentino, Michael Spartalis, Manraj Singh Sra, Chandrashekhar T Sreeramareddy, Bahadar S Srichawla, Vignes Anand Srinivasalu, Manikandan Srinivasan, Devin Bailey Srivastava, Andy Stergachis, Aleksandar Stevanović, Omer Subasi, Surajo Kamilu Kamilu Sulaiman, Muritala Odidi Suleiman Odidi, Muhammad Suleman, Mark J M Sullman, Anusha Sultan Meo, Zhong Sun, Thanigaivel Sundaram, David Sunkersing, Tarun Kumar Suvvari, Chandan Kumar Swain, Lukasz Szarpak, Rafael Tabarés-Seisdedos, Seyed-Amir Tabatabaeizadeh, Celine Tabche, Ramin Tabibi, Takahiro Tabuchi, Lidia S. Seifu Tadesse, Farzad Taghizadeh-Hesary, Moslem Taheri Soodejani, Shima - Tajabadi, Iman M Talaat, Byomkesh Talukder, Mircea Tampa, Jacques Lukenze Tamuzi, Ker-Kan Tan, Saba Tariq, Anika Tasnim, Nathan Y Tat, Vivian Y Tat, Birhan Tsegaw Taye, Yibekal Manaye Tefera, Gizaw Hailiye Teferi, Wegayehu Zeneb Teklehaimanot, Mohamad-Hani Temsah, Reem Mohamad Hani Temsah, Wegen Beyene Tesfamariam, Jay Tewari, Kavumpurathu Raman Thankappan, Samar Tharwat, Muthu Thiruvengadam, Jansje Henny Vera Ticoalu, Marius Belmondo Tincho, Sojit Tomo, Marcos Roberto Tovani-Palone, Khaled Trabelsi, Quynh Thuy Huong Tran, Tam Quoc Minh Tran, Thang Huu Tran, Nguyen Tran Minh Duc, Indang Trihandini, Tulika Tripathi, Samuel Joseph Tromans, Claudia Truppa, Daniel Hsiang-Te Tsai, Aristidis Tsatsakis, Abraham Tsedalu Tsedalu Amare, Munkhtuya Tumurkhuu, Biruk Shalmeno Tusa, Lilian Tzivian, Atta Ullah, Riaz Ullah, Saeed Ullah, Lawan Umar, Muhammad Umar, Brigid Unim, Era Upadhyay, Jeba Mahiad Urmey, Jibrin Sammani Usman, Hande Uzunçıbuk, Nazim Uzzaman, Pratyusha Vadagam, Asokan Govindaraj Vaithinathan, Jef Van den Eynde, Joe Varghese, Tommi Juhani Vasankari, Srivatsa Surya Vasudevan, Baskar Venkidasamy, Simone Villa, Jorge Hugo Villafañe, Leonardo Villani, Manish Vinayak, Francesco S Violante, Senthil Visaga Ambi, Yasir Waheed, Megha Walia, Cong Wang, Qingzhi Wang, Ruixuan Wang, Wei Wang, Xing Wang, Ahmed Bilal Waqar, Muhammad Waqas, Joseph L Ward, Yilkal Abebaw Wassie, Ishanka Weerasekara, Nuwan Darshana Darshana Wickramasinghe, Angga Wilandika, Peter Willeit, Marcin W Wojewodzic, Yohannes Chemere Wondmeneh, Haileyesus Gedamu Wondyifraw, Florence Gyembuzie Wongnaah, Minichil Chanie Chanie Worku, Felicia Wu, James Fan Wu, Qing Xia, Guangqin Xiao, Lishun Xiao, Wanqing Xie, Site Xu, Mingyang Xue, Mukesh Kumar Yadav, Sajad Yaghoubi, Saba Yahoo (Syed), Galal Yahya, Hanwen YANG, Xinxin Yang, Laiang Yao, Mohamed A Yassin, Yuichi Yasufuku, Sanni Yaya, Meghdad Yeganeh, Subah Abderehim Yesuf, Saber Yezli, Yazachew Engida Engida Yismaw, Dong Keon Yon, Naohiro Yonemoto, Chuanhua Yu, Chun-Wei Yuan, Ghazala Yunus, Umar Yunusa, Manijeh Zaghampour, Fathiah Zakham, Giulia Zamagni, Michael Zastrozhin, Mohammed Zawiah, Mohammed G M Zeariya, Alemu Birara Zemariam, Tiansong Zhan, Casper J P Zhang, Jinpeng Zhang, Xiyu Zhang, Anthony Zhong, Jiayan Zhou, Bin Zhu, Abzal Zhumagaliuly, Hafsa Zia, Magdalena Zielińska, Ghazal Zoghi, Rafat Mohammad Zrieq, Ahed H. Zyoud, Sa’ed H Zyoud, Shaher H. Zyoud, Stein Emil Vollset, Simon I Hay, Stephen S Lim, Jonathan F Mosser**.

*Lead author

**Senior author

## Affiliations

Please see appendix 2 (pp 8–40) for the affiliations for individual authors.

## Contributors

Please see appendix 2 (pp 40–53) for more detailed information about individual author contributions to the research, divided into the following categories: managing the overall research enterprise; writing the first draft of the manuscript; primary responsibility for applying analytical methods to produce estimates; primary responsibility for seeking, cataloguing, extracting, or cleaning data; designing or coding figures and tables; providing data or critical feedback on data sources; developing methods or computational machinery; providing critical feedback on methods or results; drafting the manuscript or revising it critically for important intellectual content; and managing the estimation or publications process. The lead, corresponding, and senior authors had full access to the data in the study and had final responsibility for the decision to submit for publication.
