# Supplementary material for: Global, regional, and national trends in routine childhood vaccination coverage from 1980 to 2023 with forecasts to 2030: a systematic analysis for the Global Burden of Disease Study 2023
Source: Lancet. Author manuscript; Available in PMC 2025 Aug 11. (PMC12338332; doi:10.1016/S0140-6736(25)01037-2)
Supplement: Appendix 2 [file NIHMS2095684-supplement-Appendix_2.pdf]

Appendix 2: Authorship appendix to “Global, regional, and national trends in routine childhood vaccination coverage from 1980 to 2023 with forecasts to 2030: a systematic analysis for the Global Burden of Disease Study 2023”

This appendix provides further authorship detail for “Global, regional, and national trends in routine childhood vaccination coverage from 1980 to 2023 with forecasts to 2030: a systematic analysis for the Global Burden of Disease Study 2023”

Table of Contents

Appendix 2: Authorship appendix to “Global, regional, and national trends in routine childhood vaccination coverage from 1980 to 2023 with forecasts to 2030: a systematic analysis for the Global Burden of Disease Study 2023” 1

GBD 2023 Vaccine Coverage Collaborators........ 2

Affiliations........ 8

Authors’ Contributions........ 40

Managing the overall research enterprise........ 40

Writing the first draft of the manuscript........ 40

Primary responsibility for applying analytical methods to produce estimates........ 40

Primary responsibility for seeking, cataloguing, extracting, or cleaning data; designing or coding figures and tables........ 40

Providing data or critical feedback on data sources........ 40

Developing methods or computational machinery........ 43

Providing critical feedback on methods or results........ 43

Managing the estimation or publications process........ 53

GBD 2023 Vaccine Coverage Collaborators

Emily Haeuser*, Sam Byrne, Jason Nguyen, Catalina Raggi, Susan A McLaughlin, Catherine Bisignano, Ashley A Harris, Amanda E Smith, Paulina A Lindstedt, Georgia Smith, Samuel James Herold, Olivia D Nesbit, Taylor Noyes, Noga Shalev, Latera Tesfaye Olana, Mohammad Amin Aalipour, Hasan Aalruz, Mitra Abbasifard, Faezeh Abbaspour, Hedayat Abbastabar, Samar Abd ElHafeez, Emad M. Abdallah, Reda Abdel-Hameed, Atef Abdelkader, Sherief Abd-Elsalam, Wakgari Mosisa Abdisa, Meriem Abdoun, Arman Abdous, Deldar Morad Abdulah, Adam Abdullahi, Auwal Abdullahi, Toufik Abdul-Rahman, Kulmira Abdykerimova, Armita Abedi, Asrat Agalu Abejew, Roberto Ariel Abeldaño Zuñiga, Syed Hani Abidi, Olumide Abiodun, Rahim Abo Kasem, Richard Gyan Aboagye, Hassan Abolhassani, Ulric Sena Abonie, Abdullahi Tunde Aborode, Nagah Mohamed Abourashed, Mohamed Abouzid, Dmitry Abramov, Lucas Guimarães Abreu, Dariush Abtahi, Rana Kamal Abu Farha, Bilyaminu Abubakar, Eman Abu-Gharbieh, Hana J Abukhadijah, Salahdein Aburuz, Anirudh Balakrishna Acharya, Meshack Achore, Juan Manuel Acuna, Ousman Adal, Lisa C. Adams, Abdu A Adamu, Tajudeen Adesanmi Adebisi, David Adedia, Kamoru Ademola Adedokun, Oluwatobi Emmanuel/E Adegbile, Oyelola A Adegboye, Nurudeen A Adegoke, Olumide Thomas Adeleke, Juliana Bunmi Adetunji, Mache Tsadik Adhana, Ripon Kumar Adhikary, Usha Adiga, Mohd Adnan, Qorinah Estiningtyas Sakilah Adnani, Prince Owusu Adoma, Leticia Akua Adzigbli, Giuseppina Affinito, Aanuoluwapo Adeyimika Afolabi, Habeeb Abiodun Afolabi, Rotimi Felix Afolabi, Saira Afzal, Suneth Buddhika Agampodi, Dhiraj Motilal Agarwal, Sepehr Aghajanian, Constanza Elizabeth Aguilera Arriagada, Williams Agyemang-Duah, Mahsa Ahadi, Aqeel Ahmad, Danish Ahmad, Khurshid Ahmad, Rabbiya Ahmad, Shoaib Ahmad, Tauseef Ahmad, Ayman Ahmed, Haroon Ahmed, Meqdad Saleh Ahmed, Muktar Beshir Ahmed, Mushood Ahmed, Naveed Ahmed, Syed Anees Ahmed, Simeon Okechukwu Ajakwe, Dolapo Emmanuel Ajala, Gizachew Taddesse Akalu, Oluwasefunmi Akeju, Roland Eghoghosoa Akhigbe, Karolina Akinosoglou, Mohammed Ahmed Akkaif, Hammad Akram, Ashley E Akrami, Alaa Al Amiry, Salah Al Awaidy, Hanadi Al Hamad, Mohammad Khaled Al nawayseh, Omar Al Omari, Yazan Al Thaher, Omar Ali Mohammed Al Zaabi, Mohammad Ahmmad Mahmoud Al Zoubi, Yazan Al-Ajlouni, Ziyad Al-Aly, Khurshid Alam, Mohammad Khursheed Alam, Nazmul Alam, Rasmieh Mustafa Al-amer, Turki M Alanzi, Jude Oluwapelumi Alao, Fahmi Y. Al-Ashwal, Mohammed Albashtawy, Mohammad T AlBataineh, Abdulelah Mastour Aldhahir, Mohammed S Aldossary, Shereen M. Aleidi, Tekletsadik Tekleslassie Alemayehu, Ayman Al-Eyadhy, Ali M Alfalki, Fahad D Algahtani, Abdelazeem M Algammal, Ashraf Alhumaidi, Abid Ali, Irfan Ali, Liaqat Ali, Mohammad Daud Ali, Rafat Ali, Shahid Ali, Syed Shujait Ali, Montaha Al-Iede, Sheikh Mohammad Alif, Hamid Alinejad Rokny, Morteza Alipour, Samah W Al-Jabi, Adel Al-Jumaily, Ahmad Alkhatib, Mustafa Alkhawam, Mohammed Z. Allouh, Wesam Taher Almagharbeh, Sabah Al-Marwani, Joseph Uy Almazan, Hesham M Al-Mekhlafi, Amr Almobayed, Hasan Yaser Alniss, Mohammad R. Alosta, Jaber S Alqahtani, Mohammad R. Alqudimat, Ahmed Yaseen Alqutaibi, Ahmad Alrawashdeh, Rami H Al-Rifai, Intima Alrimawi, Sahel Majed Alrousan, Mohammed A Alsabri, Zaid Altaany, Alaa B. Al-Tammemi, Jaffar A Al-Tawfiq, Malik A Althobiani, Khalid A Altirkawi, Nelson Alvis-Guzman, Nelson J Alvis-Zakzuk, Hassan Alwafi, Mohammad Al-Wardat, Yaser Mohammed Al-Worafi, Hany Aly, Mohammad Sharif Ibrahim Alyahya, Abdallah Alzoubi, Karem H Alzoubi, Md. Akib Al-Zubayer, Ekiyor Joseph Amafah, Amr Amin, Saeed Amini, Nafiu Aminu, Ayodeji Amobonye, Dickson A Amugsi, Filippos Anagnostakis, Michael Anderson, Song Peng Ang, Nguyen Hoang Anh, Abhishek Anil, Abdul-Azeez Adeyemi Anjorin, Hossein Ansariniya, Catherine M Antony, Boluwatife Stephen Anuoluwa, Saeid Anvari, Saleha Anwar, Jalal Arabloo, Jesil Mathew Aranjani, Aleksandr Y Aravkin, Demelash Areda, Abdulfatai Aremu, Olatunde Aremu, Ghazal Arjmand, Jesu Arockiaraj,

Mahwish Arooj, Anton A Artamonov, Ashokan Arumugam, Deepavalli Arumuganainar, Nurila Aryntayeva, Mahsa Asadi Anar, Muhammad Asaduzzaman, Syed Mohammed Basheeruddin Asdaq, Shewatatek Melaku Melaku Asefa, Akram Ashames, Tahira Ashraf, Mitra Ashrafi, Bernard Kwadwo Yeboah Asiamah-Asare, Muhammad Shahzad Aslam, Saeed Aslani, Yuni Asri, Dereje Zewdu Assefa, Batyrbek Assembekov, Sachin R Atre, Alok Atreya, Julie Alaere Atta, Zeenah A Atwan, Matteo Augello, Khursheed Aurangzeb, Andargie Abate Awoke, Babafela B Awosile, Seyyed HamidReza Ayatizadeh, Yusuf Oloruntoyin Ayipo, Sina Azadnajafabad, Mohd Yusmaidie Aziz, Sadat Abdulla Aziz, Amin Azizan, Ahmed Y. Azzam, Abisola Esther Babatope, Rasha Babiker, Ashish D Badiye, Sara Bagheri, Fereshteh Baghizadeh, Razieh Bahreini, Yogesh Bahurupi, Atif Amin Baig, Senthilkumar Balakrishnan, Maher Balkis, Rajon Banik, Hansi Bansal, Shirin Barati, Hiba Jawdat Barqawi, Zarrin Basharat, Shahid Bashir, Azadeh Bashiri, Rehana Basri, Quique Bassat, Mohammad-Mahdi Bastan, Saurav Basu, Kavita Batra, Ravi Batra, Mahdis Bayat, Narasimha M Beeraka, Bezawit K Bekele, Tariku Tesfaye Bekuma, Sewunet admasu Belachew, Asnake Gashaw Belayneh, Melesse Belayneh, Michael Belingheri, Umar Muhammad Bello, Samiun Nazrin Bente Kamal Tune, Abiye Assefa Berihun, Amiel Nazer C Bermudez, Robert S Bernstein, Ajeet Singh Bhadoria, Akshaya Srikanth Bhagavathula, Neeraj Bhala, Dinesh Bhandari, Pankaj Bhardwaj, Ashish Bhargava, Sonu Bhaskar, Priyadarshini Bhattacharjee, Krittika Bhattacharyya, Ashmin Hari Bhattarai, Jasvinder Singh Bhatti, Can Bilgin, Saeed Biroudian, Bijit Biswas, Mohammad Shahangir Biswas, Monirujjaman Biswas, Molalegne Bitew, Bruno Bizzozero-Peroni, Firew Tekle Bobo, Trupti Bodhare, Lucimere Bohn, Obasanjo Afolabi Bolarinwa, Paria Bolourinejad, Alejandro Botero Carvajal, Souad Bouaoud, Dejana Braithwaite, Hermann Brenner, Nikolay Ivanovich Briko, Danilo Buonsenso, Felix Busch, Yasser Bustanji, Nadeem Shafique Butt, Zahid A Butt, Mehtap Çakmak Barsbay, Luis Alberto Cámera, Angelo Capodici, Giulia Carreras, Andrea Carugno, Felix Carvalho, Joao Mauricio Castaldelli-Maia, Carlos A Castañeda-Orjuela, Luca Cegolon, Francieli Cembranel, Muthia Cenderadewi, Muge Cevik, Chiranjib Chakraborty, Sandip Chakraborty, Rama Mohan Chandika, Vijay Kumar Chattu, Galmesa Bekana Chemeda, An-Tian Chen, Hana Chen, Haowei Chen, Nicholas WS Chew, Patrick R Ching, William C S Cho, Bryan Chong, Hitesh Chopra, Shivani Chopra, Dinh-Toi Chu, Sheng-Chia Chung, Sunghyun Chung, Muhammad Chutiyami, Alyssa Columbus, Joao Conde, Alexandru Corlateanu, Claudia Cosma, Natalia Cruz-Martins, Alanna Gomes da Silva, Bashir Dabo, Omid Dadras, Xiaochen Dai, Emanuele D'Amico, Lalit Dandona, Rakhi Dandona, Lucio D'Anna, Samuel Demissie Darcho, Latefa Ali Dardas, Gary L Darmstadt, Aso Mohammad Darwesh, Dimash Davletov, Fernando Pio De la Hoz, Sindhura Deekonda, Aniket Dehadrai, Tadesse Asmamaw Dejenie, Marco Del Riccio, Mohammad Delsoz, Huiyin Deng, Edgar Denova-Gutiérrez, Anteneh Assefa Desalegn, Pradeep Kumar Devarakonda, Syed Masudur Rahman Dewan, Arkadeep Dhali, Kuldeep Dhama, Meghnath Dhimal, Sameer Dhingra, Bibha Dhungel, Stefano Di Bella, Marcello Di Pumpo, Diana Dias da Silva, Daniel Diaz, Xueting Ding, Thanh Chi Do, Sushil Dohare, Fariba Dorostkar, Wendel Mombaque dos Santos, Ojas Prakashbhai Doshi, Robert Kokou Dowou, Menayit Tamrat Dresse, John Dube, Senbagam Duraisamy, Oyewole Christopher Durojaiye, Sulagna Dutta, Osamudiamen Ebohon, Lamiaa Labieb Mahmoud Ebraheim, Mohammad Hossein --- Ebrahimi, Rasoul Ebrahimi, Abdelaziz Ed-Dra, Ferry Efendi, Behrad Eftekhari, Ashkan Eighaei Sedeh, Ebrahim Eini, Michael Ekholuenetale, Rabie Adel El Arab, Ibrahim Farahat El Bayoumy, Maysaa El Sayed Zaki, Mohamed Ahmed Eladl, Aya Elalfy, Said El-Ashker, Iffat Elbarazi, Noha Mousaad Elemam, Muhammed Elhadi, Mohamed Hassan Elnaem, Mohammed Elshaer, Abdelgawad Salah Abdelgawad Eltahawy, Theophilus I Emeto, Talha Bin Emran, Misganu Endriyas, Setegn Eshetie, Gilbert Eshun, Sharareh Eskandarieh, Majid Eslami, Maysa Eslami, Fahima Nasrin Eva, Adewale Oluwaseun Fadaka, Heidar Fadavian, Adeniyi Francis Fagbamigbe, Ayesha Fahim, Razana Faiz, Ildar Ravisovich Fakhradiyev, Niloofar Faraji, Seyed Nooreddin

Faraji, Mahsa Faramarzpour, Mohammad Fareed, MoezAlIslam Ezzat Mahmoud Faris, Andre Faro, Syed Muhammad Yousaf Farooq, Emmanuel Toluwani Fasusi, Zareen Fatima, Pooria Fazeli, Alireza Feizkhah, Ginenus Fekadu, Ulrich Membe Femoe Membe, Rodrigo Fernandez-Jimenez, Natan Feter, Claudio Fiorilla, Florian Fischer, Marco Fonzo, Takeshi Fukumoto, Nancy Fullman, Muktar A Gadanya, Dominic Dormenyo Gadeka, Márió Gajdács, Balasankar Ganesan, Xiang Gao, Bashiru Garba, Fernando Barroga Garcia, Jacopo Garlasco, Anteneh Gashaw, Zisis Gatzioufas, Rupesh K Gautam, Feven Sahle Gebre, Miglas Welay Gebregergis, Nsikakabasi Samuel George, Ubong Uwem George, Gebremariam Wulie Geremew, Genanew K Getahun, Habtamu Abebe Getahun, Fekadeselassie Belege Getaneh, Gebremariam Getaneh, Kazem Ghaffari, Roya Ghafoury, Arin Ghamkhar, Shakiba Ghasemi Assl, Haniyeh Ghasrsaz, Ramy Mohamed Ghazy, Gloria Gheno, Nermin Ghith, Arun Ghuge, Artyom Urievich Gil, Alessandro Girombelli, Laszlo Göbölös, Amit Goel, Mahaveer Golechha, Davide Golinelli, MReza Goodarzian, Aman Goyal, Shi-Yang Guan, Giovanni Guarducci, Stefano Guicciardi, Amit Gulati, Sasidhar Gunturu, Anish Kumar Gupta, Ishita Gupta, Sapna Gupta, Veer Bala Gupta, Vivek Kumar Gupta, Reyna Alma Gutiérrez, Roberth Steven Gutiérrez-Murillo, Jose Guzman-Esquivel, Annie Haakenstad, Awoke Derbie Habteyohannes, Dariush Haghmorad, Haimanot Ewnetu Hailu, Pritam Halder, Islam M Hamad, Nadia M Hamdy, Sajid Hameed, Samer Hamidi, Asif Hanif, Nasrin Hanifi, Graeme J Hankey, Harapan Harapan, Arief Hargono, Josep Maria Haro, Ahmed I Hasaballah, Mohammad Jahid Hasan, Hamidreza Hasani, Mohammad Hashem Hashempur, Ammarah Hasnain, Ibrahim Nagmeldin Hassan, Md. Imtaiyaz Hassan, Muhammad Hassan, Nageeb Hassan, Khezar Hayat, Jiawei He, Wen-Qiang He, Mohamed I Hegazy, Golnaz Heidari, Mohammad Heidari, Minoo Heidari Almasi, Sumudu Avanthi Hewage, Majid Heydari, Kamal Hezam, Yuta Hiraike, Alamgir Hossain, Lubna Hossain, Md Mahbub Hossain, Md Sabbir Hossain, Md. Jubayer Hossain, Mehdi Hosseinzadeh, Md Munnaf Hossen, Mihaela Hostiuc, Priya Hotwani, Hanno Hoven, Chengxi Hu, Junjie Huang, Kiavash Hushmandi, Javid Hussain, M. Azhar Hussain, Nawfal R Hussein, Mohamed Ibrahim Husseiny, Hong-Han Huynh, Bing-Fang Hwang, Segun Emmanuel Ibitoye, Khalid S Ibrahim, Nuheila Ibrahim, Anel Ibrayeva, Olayinka Stephen Ilesanmi, Irena M Ilic, Milena D Ilic, Mohammad Tarique Imam, Arit Inok, Mustafa Alhaji Isa, Benni Iskandar, Teresa R. Iskander, Md Sahidul Islam, Md. Fakrul Islam, Sheikh Mohammed Shariful Islam, Faisal Ismail, Leila Ismail, Mosimah Charles Ituka, Masao Iwagami, Chinwe Juliana Iwu-Jaja, Louis Jacob, Ali Jadidi, Abdollah Jafarzadeh, Haitham Jahrami, Ayushi Jain, Ammar Abdulrahman Jairoun, Mihajlo Jakovljevic, Mohamed Jalloh, Armaan Jamal, Qazi Mohammad Sajid Jamal, Melika Jameie, Jerin James, Hasan Jamil, Roland Dominic G Jamora, Syed Sarmad Javaid, Talha Jawaid, Qassim Jawell Odah Abed, Shubha Jayaram, Seogsong Jeong, Ravi Prakash Jha, Wenyi Jin, Mohammad Jokar, Jobin Jose, Jobinse Jose, Nitin Joseph, Charity Ehimwenma Joshua, Kripa Josten, Farahnaz Joukar, Jacek Jerzy Jozwiak, Zubair Kabir, Vidya Kadashetti, Dler H. Hussein Kadir, Ashish Kumar Kakkar, Md Moustafa Kamal, Mehnaz Kamal, Rajesh Kamath, Ramat T. Kamorudeen, Naser Kamyari, Oleksandr Kamyshnyi, Mona Kanaan, Saddam Fuad Kanaan, Jiseung Kang, Samuel Berchi Kankam, Kehinde Kazeem Kanmodi, Suthanthira Kannan S, Rami S Kantar, Neeti Kapoor, Jafar Karami, Reema A Karasneh, Ibraheem M Karaye, André Karch, Mohmed Isaqali Karobari, Tomasz M. Karpiński, Manoj Kumar Kashyap, Himanshu Khajuria, Mohammad Ali Khaksar, Nauman Khalid, Anees Ahmed Khalil, Faham Khamesipour, Abdul Arif Khan, Ajmal Khan, Faiz Ullah Khan, Gulfaraz Khan, Maseer Khan, Md Abdullah Saeed Khan, Moien AB Khan, Muhammad Umer Khan, Ramsha Mushtaq Khan, Sumaiya Khan Khan, Ubaid Khan, Yusuf Saleem Khan, Zahid Khan, Vishnu Khanal, Sameer Uttamaro Khasbage, Khaled Khatab, Haitham Khatatbeh, Moawiah Mohammad Khatatbeh, Afshin Khazaei, Khalid A Kheirallah, Farbod Khosravi, Grace Kim, Jinho Kim, Kwanghyun Kim, Min Seo Kim, Ruth W Kimokoti, Yohannes Kinfu, Adnan Kisa, Sezer Kisa, Shivakumar KM, Sonali Kochhar, Michail

Kokkorakis, Ali-Asghar Kolahi, Farzad Kompani, Vladimir Andreevich Korshunov, Oleksii Korzh, Karel Kostev, Parvaiz A Koul, Irene Akwo Kretchy, James-Paul Kretchy, Kewal Krishan, Barthelemy Kuate Defo, Raja Amir Hassan Kuchay, Mohammed Kuddus, Ilari Kuitunen, Mukhtar Kulimbet, Emmanuel Kumah, Dewesh Kumar, G Anil Kumar, Jogender Kumar, Kamal Kumar, Narendar Kumar, Rakesh Kumar, Satyajit Kundu, Setor K Kunutsor, Maria Dyah Kurniasari, Pramod Kumar Kushawaha, Asep Kusnali, Dian Kusuma, Assylkhan Kuttybayev, Wai Hang Patrick Kwong, Frank Kyei-Arthur, Ville Kytö, Dr Pallavi L C, Carlo La Vecchia, Muhammad Awwal Ladan, Chandrakant Lahariya, Balzhan Lakanova, Iván Landires, Savita Lasrado, Colleen L Lau, Huu-Hoai Le, Minh Huu Nhat Le, Nhi Huu Hanh Le, Trang Diep Thanh Le, Caterina Ledda, Sergey Vadimovich Lee, Seung Won Lee, Wei-Chen Lee, Awol Yemane Legesse, Elvynna Leong, Ming-Chieh Li, Peng Li, Wei Li, Virendra S Ligade, Jialing Lin, John C Lin, Queran Lin, Gang Liu, Haipeng Liu, Jue Liu, Patrick Y Liu, Xuefeng Liu, Zhe Liu, Erand Llanaj, José Francisco López-Gil, Platon D Lopukhov, Giancarlo Lucchetti, Abhilash Ludhiadch, Peng Luo, Angelina M Lutambi, Lei Lv, Kaung Suu Lwin, Miltiadis D. Lytras, Ellina Lytvyak, Ahmed M. Afifi, Kevin Sheng-Kai Ma, Zheng Feei Ma, Shamsuddeen Yusuf Yusuf Ma'aruf, Mahmoud Mabrok, Monika Machoy, Firoozeh Madadi, Farzan Madadizadeh, Seyed Ataollah Madinezad, Aurea Marilia Madureira-Carvalho, Sasikumar Mahalingam, Samatar Abshir Mahamed, Nozad H. Mahmood, Mansour Adam Mahmoud, Farhad Mahmoudi, Hardeep Singh Malhotra, Ahmad Azam Malik, Shahid Malik, Tabarak Malik, Deborah Carvalho Malta, Biniyam Tedla Tedla Mamo, Lokesh Manjani, Kamaruddeen Mannethodi, Farheen Mansoor, Mohammad Ali Mansournia, Shaista Manzoor, Tahir Maqbool, Bishnu P Marasini, Hamid Reza Marateb, Konstantinos Margetis, Michael Marks-Hultström, Adolfo Martinez-Valle, Francisco Rogerlândio Martins-Melo, Miquel Martorell, Roy Rillera Marzo, Sammer Marzouk, Stefano Masi, Clara N Matei, Yasith Mathangasinghe, Medha Mathur, Neeta Mathur, Fernanda Penido Matozinhos, Richard James Maude, Chioma Ngozichukwu Pauline Mbachu, Ikechukwu Innocent Mbachu, Steven M McPhail, María Paz Medel Salas, Rishi P Mediratta, Vini Mehta, Subhash Mehto, James Meiring, Tesfahun Mekene Meto, Tesfaye Hambisa Mekonnen, Hadush Negash Meles, Endalkachew Belayneh Melese, Ziad Ahmed Memish, Walter Mendoza, Godfred Antony Menezes, Ritesh G Menezes, Emiru Ayalew Mengistie, Leweyehu Alemaw Mengstie, Alexios-Fotios A Mentis, Sultan Ayoub Ayoub Meo, Atte Meretoja, Tomislav Mestrovic, Sachith Mettananda, Mohamed M.M. Metwally, Irmina Maria Michalek, Giuseppe Minervini, Wai-kit Ming, Andreea Mirica, Alireza Mirkheshti, Vinaytosh Mishra, Heba M. Mohamed, Hebatalla Mohamed, Jama Mohamed, Mona Gamal Mohamed, Nouh Saad Mohamed, Khabab Abbasher Hussien Abbasher Hussien Mohamed Ahmed, Taj Mohammad, Abdolreza Mohammadi, Shafiu Mohammed, Yahaya Mohammed, Syam Mohan, Mohammad Mohseni, Amin Mohsenzadeh, Ali H Mokdad, Peyman Mokhtarzadehazar, Lorenzo Monasta, Mohammad Ali Moni, Maryam Moradi, Yousef Moradi, Paula Moraga, Anthony Kwame Morgan, Shane Douglas Morrison, Mahmoud M Morsy, Seyed Ahmad Mousavi, Seyed Mohamad Sadegh Mousavi Kiasary, Hagar Mowafy, Kimia Mozahheb Yousefi, Rabia Mubarak, Sumaira Mubarik, Ulrich Otto Mueller, Sumoni Mukherjee, Francesk Mulita, Mulyadi Mulyadi, Kavita Munjal, Anjana Munshi, Christopher J L Murray, Fungai Musaigwa, Sherzad Ibrahim Mustafa, Mubarak Taiwo Mustapha, Saravanan Muthupandian, Claude Mambo Mambo Muvunyi, Muhammad Muzaffar, Woojae Myung, Ayoub Nafei, Pirouz Naghavi, Amirhossein Naghibzadeh, Mobin Naghshbandi, Ganesh R Naik, Gurudatta Naik, Firzan Nainu, Tapas Sadasivan Nair, Soroush Najdaghi, Hastyar Hama Rashid Najmuldeen, Arindam Nandi, Sreenivas Narasimha Swamy, Shumaila Nargus, Abdulqadir J Nashwan, Mahmoud Nassar, Zuhair S Natto, Zakira Naureen, Samidi Nirasha Kumari Navaratna, Biswa Prakash Nayak, Shalini Ganesh Ganesh Nayak, Md Fahad Shahariar Nayon, Athare Nazri-Panjaki, Pacifique Ndishimye, Ionut Negoi, Samata Nepal, Henok Biresaw Netsere, Kieu Viet Nhi Nguyen, Nhan Nguyen, Nhien Ngoc Y Nguyen, Quan

Nguyen Khoi, Nguyen Ngoc Yen Nhi, Robina Khan Niazi, Luciano Nieddu, Afewerki Tesfahunegn Tesfahunegn Nigusse, Ali Nikoobar, Behnaz Niroomand, Chukwudi A Nnaji, Shuhei Nomura, Syed Toukir Ahmed Noor, Masoud Noroozi, Valentine C. Nriagu, Chisom Adaobi Nri-Ezedi, Jean Claude Nshimiyimana, Fred Nugen, Mengistu H Nunemo, Felix Kwasi Nyande, Bogdan Oancea, Mary Aigbiremo Oboh, Ramez M. Odat, Ismail A Odetokun, Michael Safo Oduro, Tunde Emmanuel Ogundare, Oluwafunmilayo Tosin Ogundeko-Olugbami, Olusegun Olatunji Ojedoyin, Akinkunmi Paul Okekunle, Onyedika A Okoli, Osaretin Christabel Okonji, John Olayemi Okunlola, Oluyemi Adewole Okunlola, Oluwaseyi Isaiah Olabisi, Antonio Olivas-Martinez, Gláucia Maria Moraes Oliveira, Abdulhakeem Abayomi Olorukooba, Samson Bamidele Olorunju, Comfort Z. Z Olorunsaiye, Bolajoko Olubukunola Olusanya, Oluwafemi G. Gabriel Oluwole, Folorunsho Bright Omage, Obinna E Onwujekwe, Chizaram A Onyeaghala, Marcel Opitz, Michal Ordak, Verner N Orish, Atakan Orscelik, Alberto Ortiz, Edgar Ortiz-Brizuela, Esteban Ortiz-Prado, Augustus Osborne, Eric Osei, Elham H. Othman, Oche Joseph Otorkpa, Amel Ouyahia, Mayowa O Owolabi, Kolapo Oyebola, Tope Oyelade, Oyetunde T Oyeyemi, Ilker Ozsahin, Jagadish Rao Padubidri, Yeganeh Pakbaz, Tamás Palicz, Sujogya Kumar Panda, Georgios D Panos, Leonidas D. D Panos, Mario Virgilio Papa, Ilias Papadimopoulos, Shahina Pardhan, Utsav Parekh, Romil R Parikh, Chulwoo Park, Roberto Passera, Mitesh Patel, Neel Navinkumar Patel, Shankargouda Patil, Dimitrios Patoulias, Shrikant Pawar, Shubhadarshini Pawar, Hamidreza Pazoki Toroudi, Jarmila Pekarcikova, Veincent Christian Filipino Pepito, Prince Peprah, Gavin Pereira, Gladymar Perez Chacon, Simone Perna, Pavlo Petakh, Olumuyiwa James Peter, Nhat Truong Pham, Tung Thanh Pham, Zahra Zahid Piracha, Edoardo Pirera, Dimitri Poddighe, Roman V Polibin, Ramesh Poluru, Sajjad Pourasghary, Reza Pourbabaki, Farzad Pourghazi, Naeimeh Pourtaheri, Ashwathi Prakash, Elton Junio Sady Prates, Jyotirekha Purohit, Jagadeesh Puvvula, Husam Qanash, Nameer Hashim Qasim, Asma Saleem Qazi, Xiang Qi, Zhipeng Qi, Gangzhen Qian, Navid Rabiee, Basuki Rachmat, Venkatraman Radhakrishnan, Fakher Rahim, Sajjad Rahimi, Vafa Rahimi-Movaghar, Fryad Majeed Rahman, Md. Mosfequr Rahman, Md. Obaidur Rahman, Mosiur Rahman, Muhammad Aziz Rahman, Saeed Rahmani, Hakim Rahmoune, Sunil Kumar Raina, Jeffrey Pradeep Raj, Adarsh Raja, Gunaseelan Rajendran, Judah Rajendran, Mohammad Amin Rajizadeh, Siddheesh Rajpurohit, Mahmoud Mohammed Ramadan, Chitra Ramasamy, Shakthi Kumaran Ramasamy, Kamleshun Ramphul, Kirtan Rana, Rishabh Kumar Rana, Chhabi Lal Ranabhat, Nemanja Rancic, Smitha Rani, Chythra R Rao, Sowmya J Rao, Md. Abdur Rashid, Mohammad-Mahdi Rashidi, Azad Rasul, Devarajan Rathish, Abdur Rauf, Santosh Kumar Rauniyar, David Laith Rawaf, Salman Rawaf, Elrashdy Redwan, Wajiha Rehman, Luis Felipe Reyes, Mina Rezaei, Nazila Rezaei, Mohsen Rezaeian, Abanoub Riad, Moattar Raza Rizvi, Hannah Elizabeth Robinson-Oden, Hermano Alexandre Lima Rocha, Thales Philipe Rodrigues da Silva, Leonardo Roever, Amirhossein Roshanshad, Himanshu Sekhar Rout, Shiva Rouzbahani, Adrija Roy, Nitai Roy, Sharmistha Roy, Shubhanjali Roy, Guilherme de Andrade Ruela, Godfrey M Rwegerera, Aly M A Saad, Maha Mohamed Saber-Ayad, Seyed Kiarash Sadat Rafiei, Basema Ahmad Saddik, Tarannom Sadegh, Fatemeh Sadeghi-Ghyassi, Mohd Saeed, Umar Saeed, Mehdi Safari, Mastooreh Sagharichi, Dominic Sagoe, Narjes Saheb Sharif-Askari, S. Mohammad Sajadi, Md Refat Uz Zaman Sajib, Mirza Rizwan Sajid, Morteza Saki, Dorsa Salabat, Nasir Salam, Afeez Abolarinwa Salami, Mohamed A Saleh, Mahdi Salehi, Aanuoluwa James Salemcity, Dauda Salihu, Sohrab Salimi, Pegah Salimi Pormehr, Malik Sallam, Hossein Samadi Kafil, Saad Samargandy, Yoseph Leonardo Samodra, Nahom Samuel Samuel, Abdallah M Samy, Sathish Sankar, Adekunle Sanyaolu, Jacob Owusu Sarfo, Hemen Sarma, Mohammad Sarmadi, Sachin C Sarode, Brijesh Sathian, Maheswar Satpathy, Mehrdad Savabi Far, Monika Sawhney, Ganesh Kumar Saya, Christophe Schinckus, Ione Jayce Ceola Schneider, Art Schuermans, Amin Sedigh, Mohammad H Semreen, Sabyasachi Senapati, Ashenafi Kibret

Sendekie, Pallav Sengupta, Yigit Can Senol, Subramanian Senthilkumaran, Dragos Serban, Yashendra Sethi, Seyed Mohammad Seyed Alshohadaei, Abubakar Sha'aban, Muhammad Shahab, Samiah Shahid, Syed Ahsan Ahsan Shahid, Wajeehah Shahid, Farshad Shahkarami, Fatemeh Shahrahmani, Moyad Jamal Shahwan, Ahmed Shaikh, Masood Ali Shaikh, Nafhat Shaikh, Alireza Shakeri, Mehran Shams-Beyranvand, Mohammad Ali Shamshirgaran, Anas Shamsi, Alfiya Shamsutdinova, Dan Shan, Mohammed Shannawaz, Amin Sharifan, Bunty Sharma, Manoj Sharma, Vishal Sharma, Ramzi Shawahna, Maryam Shayan, Suchitra M Shenoy, Samendra P Sherchan, Shiran Shetty, Md. Monir Hossain Shimul, Aminu Shittu, Zahra Shokati Eshkiki, Azad Shokri, Sina Shool, Seyed Afshin Shorofi, Kerem Shuval, Zahra Siavashpour, Emmanuel Edwar Siddig, Ayesha Siddiqua, Gustavo Correia Basto da Silva, Luís Manuel Lopes Rodrigues Silva, Amit Singh, Baljinder Singh, Bhim Pratap Singh, Harmanjit Singh, Jasvinder A Singh, Kalpana Singh, Poornima Suryanath Singh, Samer Singh, Satwinder Singh, Mukesh Kumar Sinha, Ebrahim Abdela Siraj, Natia Skhvitaridze, Valentin Yurievich Skryabin, Md.Salman Sohel, Anton Sokhan, Ahmed M. Soliman, May Mohamed Sherif Soliman, Noha Salah Soliman, Sameh S M Soliman, Weiyi Song, Aayushi Sood, Prashant Sood, Soroush Soraneh, Reed J D Sorensen, Michele Sorrentino, Michael Spartalis, Manraj Singh Sra, Chandrashekhar T Sreeramareddy, Bahadar S Srichawla, Vignes Anand Srinivasalu, Manikandan Srinivasan, Devin Bailey Srivastava, Andy Stergachis, Aleksandar Stevanović, Omer Subasi, Surajo Kamilu Kamilu Sulaiman, Muritala Odidi Suleiman Odidi, Muhammad Suleman, Mark J M Sullman, Anusha Sultan Meo, Zhong Sun, Thanigaivel Sundaram, David Sunkersing, Tarun Kumar Suvvari, Chandan Kumar Swain, Lukasz Szarpak, Rafael Tabarés-Seisdedos, Seyed-Amir Tabatabaeizadeh, Celine Tabche, Ramin Tabibi, Takahiro Tabuchi, Lidia S. Seifu Tadesse, Farzad Taghizadeh-Hesary, Moslem Taheri Soodejani, Shima - Tajabadi, Iman M Talaat, Byomkesh Talukder, Mircea Tampa, Jacques Lukenze Tamuzi, Ker-Kan Tan, Saba Tariq, Anika Tasnim, Nathan Y Tat, Vivian Y Tat, Birhan Tsegaw Taye, Yibekal Manaye Tefera, Gizaw Hailiye Teferi, Wegayehu Zeneb Teklehaimanot, Mohamad-Hani Temsah, Reem Mohamad Hani Temsah, Wegen Beyene Tesfamariam, Jay Tewari, Kavumpurathu Raman Thankappan, Samar Tharwat, Muthu Thiruvengadam, Jansje Henny Vera Ticoalu, Marius Belmondo Tincho, Sojit Tomo, Marcos Roberto Tovani-Palone, Khaled Trabelsi, Quynh Thuy Huong Tran, Tam Quoc Minh Tran, Thang Huu Tran, Nguyen Tran Minh Duc, Indang Trihandini, Tulika Tripathi, Samuel Joseph Tromans, Claudia Truppa, Daniel Hsiang-Te Tsai, Aristidis Tsatsakis, Abraham Tsedalu Tsedalu Amare, Munkhtuya Tumurkhuu, Biruk Shalmeno Tusa, Lilian Tzivian, Atta Ullah, Riaz Ullah, Saeed Ullah, Lawan Umar, Muhammad Umar, Brigid Unim, Era Upadhyay, Jeba Mahiad Urmey, Jibrin Sammani Usman, Hande Uzunçıbuk, Nazim Uzzaman, Pratyusha Vadagam, Asokan Govindaraj Vaithinathan, Jef Van den Eynde, Joe Varghese, Tommi Juhani Vasankari, Srivatsa Surya Vasudevan, Baskar Venkidasamy, Simone Villa, Jorge Hugo Villafañe, Leonardo Villani, Manish Vinayak, Francesco S Violante, Senthil Visaga Ambi, Yasir Waheed, Megha Walia, Cong Wang, Qingzhi Wang, Ruixuan Wang, Wei Wang, Xing Wang, Ahmed Bilal Waqar, Muhammad Waqas, Joseph L Ward, Yilkal Abebaw Wassie, Ishanka Weerasekara, Nuwan Darshana Darshana Wickramasinghe, Angga Wilandika, Peter Willeit, Marcin W Wojewodzic, Yohannes Chemere Wondmeneh, Haileyesus Gedamu Wondyifraw, Florence Gyembuzie Wongnaah, Minichil Chanie Chanie Worku, Felicia Wu, James Fan Wu, Qing Xia, Guangqin Xiao, Lishun Xiao, Wanqing Xie, Site Xu, Mingyang Xue, Mukesh Kumar Yadav, Sajad Yaghoubi, Saba Yahoo (Syed), Galal Yahya, Hanwen YANG, Xinxin Yang, Laiang Yao, Mohamed A Yassin, Yuichi Yasufuku, Sanni Yaya, Meghdad Yeganeh, Subah Abderehim Yesuf, Saber Yezli, Yazachew Engida Engida Yismaw, Dong Keon Yon, Naohiro Yonemoto, Chuanhua Yu, Chun-Wei Yuan, Ghazala Yunus, Umar Yunusa, Manijeh Zaghampour, Fathiah Zakham, Giulia Zamagni, Michael Zastrozhin, Mohammed Zawiah, Mohammed G M Zeariya, Alemu Birara Birara Zemariam, Tiansong Zhan, Casper J P Zhang, Jinpeng Zhang, Xiyu Zhang, Anthony Zhong,

Jiayan Zhou, Bin Zhu, Abzal Zhumagaliuly, Hafsa Zia, Magdalena Zielińska, Ghazal Zoghi, Rafat Mohammad Zrieq, Ahed H. Zyoud, Sa'ed H Zyoud, Shaher H. Zyoud, Stein Emil Vollset, Simon I Hay, Stephen S Lim, Jonathan F Mosser**.

*Lead author
*Senior author

Affiliations

Institute for Health Metrics and Evaluation (E Haeuser PhD, S Byrne MPH, J Nguyen MS, C Raggi MS, S A McLaughlin PhD, C Bisignano MPH, A A Harris MPH, A E Smith MPA, P A Lindstedt MPH, G Smith MS, S J Herold BS, O D Nesbit MA, T Noyes MPH, N Shalev MD, L T Olana BSc, C M Antony MA, A Y Aravkin PhD, X Dai PhD, Prof L Dandona MD, Prof R Dandona PhD, N Fullman MPH, A Haakenstad ScD, J He MSc, T Mestrovic PhD, Prof A H Mokdad PhD, Prof C J L Murray DPhil, H E Robinson-Oden MLIS, R J D Sorensen PhD, C Yuan PhD, Prof S E Vollset DrPH, Prof S I Hay FMedSci, Prof S S Lim PhD, J F Mosser MD), Department of Applied Mathematics (A Y Aravkin PhD), Department of Health Metrics Sciences, School of Medicine (A Y Aravkin PhD, X Dai PhD, Prof R Dandona PhD, Prof A H Mokdad PhD, Prof C J L Murray DPhil, Prof A Stergachis PhD, Prof S E Vollset DrPH, Prof S I Hay FMedSci, Prof S S Lim PhD), Department of Radiology (F Khosravi MD), Cardiothoracic Imaging Section (F Khosravi MD), Department of Global Health (S Kochhar MD, R J D Sorensen PhD), Department of Biostatistics (A Olivas-Martinez MD), Department of Pharmacy (Prof A Stergachis PhD), Department of Epidemiology (H Zia BDS), University of Washington, Seattle, WA, USA; National Data Management Center for Health (NDMC) (L T Olana BSc), Department of Microbial, Cellular, and Molecular Biology (G T Akalu MSc), Health Biotechnology Directorate at Bio and Emerging Technology Institute (M Bitew PhD), College of Health Sciences (F S Gebre MD), Addis Ababa University, Addis Ababa, Ethiopia; Shahid Beheshti University of Medical Sciences (M Aalipour MD), Department of Anesthesiology (Prof D Abtahi MD, Prof A Mirkheshti MD, S Salimi MD, S Seyed Alshohadaei MD, A Shakeri MD), School of Medicine (G Arjmand MD, M Asadi Anar MD, R Ebrahimi MD, S Madinezad MD, B Niroomand MD, S Sadat Rafiei MD), School of Medical Education and Learning Technologies (S Bagheri PhD), Taleghani Anesthesiologist (F Baghizadeh MD), Cancer Research Center (M Bayat MD), Obesity Research Center (M Heidari Almasi MD), Social Determinants of Health Research Center (A Kolahi MD, A Nikoobar BSc, M Rashidi MD), Anesthesiology Research Center (F Madadi MD), School of Public Health (S Sadat Rafiei MD), Department of Health (M Safari PhD), Faculty of Medicine (M Sagharichi HSDipl), Ophthalmic Research Center (ORC) (M Shayan MD), Radio-Oncology Department of Shohadaye Tajrish Hospital (Z Siavashpour PhD), Shahid Beheshti University of Medical Sciences, Tehran, Iran; Department of Nursing (H Aalruz PhD), Al Zaytoonah University of Jordan, Amman, Jordan; Department of Internal Medicine (M Abbasifard MD), Clinical Research Development Unit (M Abbasifard MD), Department of Immunology (Prof A Jafarzadeh PhD), Department of Epidemiology and Biostatistics (Prof M Rezaeian PhD), Rafsanjan University of Medical Sciences, Rafsanjan, Iran; Department of Medicine (F Abbaspour MD), Department of Global Health Sciences (S Ghasemi Assl MD), Department of Neurosurgery (A Orscelik MD, Y Senol MD), Department of Bioengineering and Therapeutical Sciences (Prof M Zastrozhin PhD), University of California San Francisco, San Francisco, CA, USA; Advanced Diagnostic and Interventional Radiology Research Center (H Abbastabar PhD), Research Center for Immunodeficiencies (H Abolhassani PhD), Rheumatology Research Center (A Azizan PhD), Non-communicable Diseases Research Center (M Bastan MD, M Rashidi

MD, N Rezaei MD, D Salabat MD), Pastor Institute (M Bayat MD), Iranian Research Center for HIV/AIDS (IRCHA) (O Dadras PhD), Multiple Sclerosis Research Center (S Eskandarieh PhD), Sina Trauma and Surgery Research Center (M Jalloh MD, Prof V Rahimi-Movaghar MD, S Shool MD), Iranian Center of Neurological Research (M Jameie MD), Department of Immunology (J Karami PhD), Children's Medical Center (Prof F Kompani MD), Department of Epidemiology and Biostatistics (M Mansournia PhD), Urology Research Center (A Mohammadi MD), Urology Department (A Mohammadi MD), Cardiovascular Diseases Research Institute (A Mohsenzadeh MSc), School of Medicine (D Salabat MD), Department of Research and Development (P Salimi Pormehr MSc), Department of Medical Education (A Sedigh PhD), Department of Internal Medicine (F Shahkarami MD), Sina Hospital (A Sharifan PharmD), Tehran University of Medical Sciences, Tehran, Iran (A Azizan PhD, K Ghaffari PhD); Department of Epidemiology (S Abd ElHafeez DrPH), Tropical Health Department (R M Ghazy PhD), Department of Pathology (Prof I M Talaat PhD), Alexandria University, Alexandria, Egypt; Department of Biology (Prof E M Abdallah PhD), Department of Health Informatics, College of Applied Medical Sciences (Q Jamal PhD), Qassim University, Buraydah, Saudi Arabia; Basic Science Department (Prof R Abdel-Hameed PhD), University of Ha'il, Hail, Saudi Arabia; Chemistry Department, Faculty of Science (Prof R Abdel-Hameed PhD), Department of Zoology and Entomology (A I Hasaballah PhD, M G M Zeariya PhD), Al-Azhar University, Cairo, Egypt; Department of Mathematics and Sciences (A Abdelkader PhD), College of Pharmacy and Health Sciences (A Al Amiry MS), Center of Medical and Bio-allied Health Sciences Research (A Al Amiry MS), Department of Pathological Sciences (A Alzoubi PhD), Ajman University (A Ashames PhD), College of Medicine (S Dutta PhD), Center for Medical and Bio-Allied Health Sciences Research (Prof M J Shahwan PhD, A Shamsi PhD), Ajman University, Ajman, United Arab Emirates (Prof N Hassan PhD); Department of Tropical Medicine and Infectious Diseases (S Abd-Elsalam PhD), Tanta University, Tanta, Egypt; Institute of Health (W M Abdisa MPH), Jimma University, Dambi Dollo, Ethiopia; Department of Medicine (Prof M Abdoun PhD), Faculty of Medicine (H Rahmoune MD), LIRSSEI Research Lab (H Rahmoune MD), University of Setif Algeria, Sétif, Algeria; Department of Health, Sétif, Algeria (Prof M Abdoun PhD); Faculty of Veterinary Medicine (A Abdous MD), Young Researchers and Elite Club (M Jokar DVM), Islamic Azad University, Karaj, Iran; Community and Maternity Nursing Unit (D M Abdulah MPH), Department of Pathology and Microbiology (M S Ahmed PhD), University of Duhok, Duhok, Iraq; Department of Population and Global Health (A Abdullahi PhD), T. H. Chan School of Public Health (S B Kankam MD), Department of Medicine (M Kokkorakis BSc), Department of Global Health & Population (H Mohamed MS), Department of Health Policy and Oral Epidemiology (Z S Natto DrPH), Department of Ophthalmology (M Shayan MD), Harvard Medical School (A Zhong MA), Harvard University, Boston, MA, USA; Department of Physiotherapy (A Abdullahi PhD, J S Usman PhD), Department of Community Medicine (Prof M A Gadanya MD), Department of Nursing Science (M Ladan PhD), Bayero University Kano, Kano, Nigeria; Department of Physiotherapy (A Abdullahi PhD), Federal University Wukari, Wukari, Nigeria; Department of Research (T Abdul-Rahman MD), Toufik's World Medical Association, Antonova, Ukraine; Department of General Surgery and Clinical Anatomy (K Abdykerimova MD), Department of Public Health (N Aryntayeva MSPH, A Kuttybayev MSc), Atchabar Scientific Research Institute (B Assembekov PhD), B. Atchabarov Scientific-Research Institute of Fundamental and Applied Medicine (D Davletov MD), Director of the Scientific and Technological Park (I R Fakhradiyev PhD), Science and Technology Park (A Ibrayeva PhD), Research and Publication Activity Division (M Kulimbet MSc), Department of Research (B Lakanova MD), Science Department (A Shamsutdinova MD), Atchabarov Scientific-Research Institute of Fundamental and Applied Medicine (A Zhumagaliuly MD), Kazakh National Medical University, Almaty, Kazakhstan (S V Lee PhD); Department of Emergency Medicine (A

Abedi MD), School of Medicine (M Ashrafi MD), Department of Critical Care and Emergency Nursing (N Hanifi PhD), Zanjan University of Medical Sciences, Zanjan, Iran; School of Pharmacy (A Abejew MSc, T T Alemayehu MSc, E Siraj MSc), Department of Emergency and Critical Care (O Adal MSc), School of Veterinary Medicine (S M Asefa MSc), College of Medicine and Health Sciences (A A Awoke PhD, H B Netsere MSc), Department of Emergency and Critical Care Nursing (A G Belayneh MSc), Department of Public Health (M Belayneh PhD), Health Systems Management and Health Economics (G Getaneh MPH), Department of Medical Microbiology (A D Habteyohannes PhD), Department of Adult Health Nursing (E A Mengistie MSc), Bahir Dar University (H G Wondyifraw MSc), Department of Pharmacology (Y E Yismaw MSc), Bahir Dar University, Bahir Dar, Ethiopia; Postgraduate Department (Prof R Abeldaño Zuñiga PhD), University of Sierra Sur, Miahuatlan de Porfirio Diaz, Mexico; Yhteiskuntadatatieteen Keskus (Centre for Social Data Science) (Prof R Abeldaño Zuñiga PhD), University of Helsinki, Helsinki, Finland; Department of Biomedical Sciences (S Abidi PhD), Nazarbayev University School of Medicine, Astana, Kazakhstan; Department of Community Medicine (Prof O Abiodun MPH), Babcock University, Ilishan-Remo, Nigeria; Department of Neurosurgery (R Abo Kasem MD), University of Louisville, Louisville, KY, USA; Department of Surgery (R Abo Kasem MD), Damascus University, Damascus, Syria; Department of Family and Community Health (R G Aboagye MPH), School of Basic and Biomedical Sciences (D Adedia PhD), Department of Epidemiology and Biostatistics (L A Adzigbli BSc, R K Dowou MPhil), Department of Nursing (F K Nyande PhD), Department of Microbiology and Immunology (Prof V N Orish PhD), University of Health and Allied Sciences (E Osei PhD), University of Health and Allied Sciences, Ho, Ghana; School of Population Health (R G Aboagye MPH, Prof B A Saddik PhD), The Graduate School of Biomedical Engineering (Prof H Alinejad Rokny PhD), International Centre for Future Health Systems (J Lin PhD), Kirby Institute (G Perez Chacon PhD), University of New South Wales, Sydney, NSW, Australia; Department of Medical Biochemistry and Biophysics (H Abolhassani PhD), Department of Neurobiology, Care Sciences and Society, Aging Research Center (B Bizzozero-Peroni PhD), Karolinska Institute, Stockholm, Sweden; Department of Sport, Exercise and Rehabilitation (U S Abonie PhD), Northumbria University, Newcastle, UK; Department of Research and Development (A T Aborode MSc), Healthy Africans Platform, Ibadan, Nigeria; Department of Clinical Laboratory Sciences (N M Abourashed PhD), University of Ha'il, Hail, Saudi Arabia; Zoology Department (N M Abourashed PhD), Benha University, Benha, Egypt; Department of Physical Pharmacy and Pharmacokinetics (M Abouzid PharmD), Chair and Department of Medical Microbiology (Prof T Karpiński), Poznan University of Medical Sciences, Poznan, Poland; Cardiovascular Disease (D Abramov MD), Loma Linda University Medical Center, Loma Linda, CA, USA; Department of Pediatric Dentistry (Prof L Abreu PhD), School of Nursing (A da Silva PhD), Department of Maternal-Child Nursing and Public Health (Prof D C Malta PhD, Prof F P Matozinhos PhD, E J S Prates BS), Vaccination Research Observatory (T Rodrigues da Silva PhD), Faculty of Dentistry (Prof G C B D Silva PhD), Federal University of Minas Gerais, Belo Horizonte, Brazil; Clinical Pharmacy and Therapeutics Department (Prof R K Abu Farha PhD), Applied Science Research Center (A Al-Tammemi MPH), Department of Clinical Nutrition and Dietetics (Prof M E M Faris PhD), Faculty of Nursing (E H Othman PhD), Applied Science Research Centre (R M Zrieq PhD), Applied Science Private University, Amman, Jordan; Department of Pharmacology and Toxicology (B Abubakar PhD), Department of Pharmaceutical Sciences (N Aminu PhD), Department of Veterinary Public Health and Preventive Medicine (B Garba PhD, A Shittu MSc), Usmanu Danfodiyo University, Sokoto, Sokoto, Nigeria; Department of Biochemistry and Nutrition (K Oyebola PhD), Nigerian Institute of Medical Research, Lagos, Nigeria (B Abubakar PhD); Department of Clinical Sciences (Prof E Abu-Gharbieh PhD, Prof M M Ramadan PhD), Department of Restorative Dentistry (A B Acharya PhD), College of Pharmacy

(S M Aleidi PhD, H Y Alniss PhD, Prof M H Semreen PhD), Department of Pharmacy Practice and Pharmacotherapeutics (Prof K H Alzoubi PhD), College of Medicine (Prof A Amin PhD, Prof B A Saddik PhD, Prof M A Saleh PhD), Department of Physiotherapy (A Arumugam PhD), Clinical Sciences Department (H J Barqawi Mphil, Prof M M Saber-Ayad PhD, N Saheb Sharif-Askari PhD, Prof I M Talaat PhD), Department of Basic Biomedical Sciences (Prof Y Bustanji PhD), Department of Basic Medical Sciences (M A Eladl PhD), Sharjah Institute for Medical Research (N M Elemam PhD), Department of Finance and Economics (Prof M Hussain PhD), Research Institute for Medical and Health Science (S Manzoor PhD), Research Institute of Medical & Health Sciences (Prof M H Semreen PhD), Department of Medicinal Chemistry (S S M Soliman PhD), University of Sharjah, Sharjah, United Arab Emirates (K A Altirkawi MD); Department of Biopharmaceutics and Clinical Pharmacy (Prof E Abu-Gharbieh PhD), College of Pharmacy (Prof S Aburuz PhD), University of Jordan, Amman, Jordan; Medical Research Center (H J Abukhadijah MPH), Department of Geriatric and Long Term Care (H Al Hamad MD, B Sathian PhD), Rumailah Hospital (H Al Hamad MD), Corporate Nursing and Midwifery Research Department (K Mannethodi MPH), Nursing & Midwifery Research Department (NMRD) (A J Nashwan PhD), Research Department (K Singh PhD), Hematology Section (Prof M A Yassin MD), Hamad Medical Corporation, Doha, Qatar; Department of Pharmacology and Therapeutics (Prof S Aburuz PhD), College of Medicine and Health Sciences (Prof M Z Allouh PhD, Prof G Khan PhD), Institute of Public Health (R H Al-Rifai PhD, I Elbarazi DrPH), Department of Computer Science and Software Engineering (Prof L Ismail PhD), Family Medicine Department (M A Khan MSc), United Arab Emirates University, Al Ain, United Arab Emirates; Department of Population Health (M Achore PhD), School of Health Professions and Human Services (I M Karaye MD), Hofstra University, Hempstead, NY, USA; Department of Clinical Medicine (Prof J M Acuna MD), American University of Antigua, Coolidge, Antigua and Barbuda; FIU Robert Stempel College of Public Health & Social Work (Prof J M Acuna MD), Department of Global Health (B Talukder PhD), Florida International University, Miami, FL, USA; Department of Diagnostic and Interventional Radiology (L C Adams PhD), School of Medicine and Health (F Busch MD), Technical University of Munich, Munich, Germany; Division of Pediatric Hospital Medicine (R P Mediratta MD), School of Medicine (J Zhou PhD), Stanford University, Palo Alto, CA, USA (L C Adams PhD); Department of Global Health (A A Adamu PhD, C J Iwu-Jaja PhD), Department of Epidemiology (J L Tamuzi MSc), Stellenbosch University, Cape Town, South Africa; Cochrane South Africa (A A Adamu PhD), Department of Global Health (C J Iwu-Jaja PhD), South African Medical Research Council, Cape Town, South Africa; Department of Microbiology (T A Adebisi BSc), Ladoke Akintola University, Osogbo, Nigeria; Department of Immunology (K A Adedokun MSc), Roswell Park Comprehensive Cancer Center Buffalo, United States, Buffalo, NY, USA; Department of Pediatrics (O E Adegbile MD), East Tennessee State University, Johnson City, TN, USA; Center for Cardiovascular Risk Research (O E Adegbile MD), Center for Cardiovascular Risk Research, Johnson City, TN, USA; Menzies School of Health Research (Prof O A Adegboye PhD), Charles Darwin University, Darwin, NT, Australia; Translational Research Team (N A Adegoke PhD), Melanoma Institute Australia (N A Adegoke PhD), The University of Sydney, Sydney, NSW, Australia; Family Medicine Department (O T Adeleke MD), Bowen University Hospital (D E Ajala BSN), Bowen University, Iwo, Nigeria; Department of Family Medicine (O T Adeleke MD), Bowen University Teaching Hospital, Ogbomoso, Nigeria; Department of Biochemistry (J B Adetunji PhD), Osun State University, Osogbo, Nigeria; School of Public Health (M T Adhana PhD), Obstetrics and Gynecology (A Y Legesse MD), Department of Epidemiology (A T Nigusse MPH), Department of Medical Biochemistry and Molecular Biology (W B Tesfamariam MSc), Mekelle University, Mekelle, Ethiopia; Department of Fisheries and Marine Bioscience (R K Adhikary PhD), Jashore University of Science and Technology, Jashore, Bangladesh; Research School of Population

Health (R K Adhikary PhD), School of Medicine and Psychology (D Ahmad PhD), Australian National University, Canberra, ACT, Australia; Apollo Institute Of Medical Sciences & Research Chittoor (Prof U Adiga PhD), Apollo Hospital, Chittoor, India; Department of Biology, College of Science (M Adnan PhD), Department of Public Health (Prof F D Algahtani PhD, M G M Zeariya PhD), College of Medicine (Y S Khan MD), Department of Biochemistry (Prof M Kuddus PhD), College of Public Health & Health Informatics (R Kumar PhD), Department of Medical Laboratory Science (H Qanash PhD), Department of Biology (Prof M Saeed PhD), Medical and Diagnostic Research Centre (Prof C T Sreeramareddy MD), Department of Basic Science (G Yunus PhD), Department of Medical-Surgical Nursing (R M Zrieq PhD), University of Hail, Hail, Saudi Arabia; Department of Public Health (Q Adnani PhD), Universitas Padjadjaran (Padjadjaran University), Bandung, Indonesia; Department of Health Administration and Education (P O Adoma PhD), University of Education Winneba, Winneba, Ghana; Department of Public Health and Preventive Medicine (G Affinito PhD), Department of Public Health (C Fiorilla MD), University of Naples, "Federico II", Naples, Italy; Technical Services Directorate (A A Afolabi MPH), MSI Nigeria Reproductive Choices, Abuja, Nigeria; Department of Statistics (H A Afolabi PhD), Osun State University, Osogbo, Osogbo, Nigeria; Department of Epidemiology and Medical Statistics (R F Afolabi PhD, A F Fagbamigbe PhD, S B Olorunju MSc), Department of Health Promotion and Education (S Ibitoye PhD), College of Medicine (A P Okekunle PhD, O I Olabisi PhD), Department of Medicine (Prof M O Owolabi DrM), University of Ibadan, Ibadan, Nigeria; Department of Community Medicine (Prof S Afzal PhD), King Edward Memorial Hospital, Lahore, Pakistan; Department of Public Health (Prof S Afzal PhD), Public Health Institute, Lahore, Pakistan; Department of New Initiatives (Prof S B Agampodi MD), International Vaccine Institute, Seoul, South Korea; Vadu Rural Health Program (D M Agarwal PhD), KEM Hospital Research Centre, Pune, India; Department of Neurosurgery (S Aghajanian MD), School of Medicine (M Shams-Beyranvand MSc), Alborz University of Medical Sciences, Karaj, Iran; Neuroscience Research Center (S Aghajanian MD, M Jameie MD), Health Management and Economics Research Center (J Arabloo PhD), School of Medicine (M Bastan MD), Medical Ethics Department (S Biroudian PhD), Department of Medical Laboratory Sciences (F Dorostkar PhD), Institute of Endocrinology and Metabolism (R Ghafoury MD), Department of Ophthalmology (H Hasani MD), Department of Health Services Management (M Mohseni PhD), Antimicrobial Resistance Research Center (K Mozahheb Yousefi MD), Hazrat-e-Rasool General Hospital (K Mozahheb Yousefi MD), Rajaei Cardiovascular Research Center (M Naghshbandi MD), Breast Health and Cancer Research Center (Y Pakbaz MD), Physiology Research Center (H Pazoki Toroudi PhD), Department of Physiology (H Pazoki Toroudi PhD), Center for Technology and Innovation in Cardiovascular Informatics (S Shool MD), The Five Senses Health Institute (F Taghizadeh-Hesary MD), Iran University of Medical Sciences, Tehran, Iran; Pfizer Andean Cluster (C E Aguilera Arriagada MD), Pfizer Inc., Santiago, Chile; Department of Public Health Sciences (W Agyemang-Duah PhD), Queen's University, Kingston, ON, Canada; Rajaie Trauma Research center (M Ahadi MD), Trauma Research Center (S Ayatizadeh MD, P Fazeli MSc), Health Information Management (A Bashiri PhD), Department of Pathology (S Faraji PhD), Department of Medical Immunology (P Fazeli MSc), Family Medicine Department (M Goodarzian MD), Research Center for Traditional Medicine and History of Medicine (Prof M Hashempur PhD), Department of Occupational Health and Safety Engineering (R Pourbabaki PhD), Shiraz University of Medical Sciences, Shiraz, Iran (S Mousavi Kiasary DVM-MPH); College of Medicine (A Ahmad PhD), Shaqra University, Shaqra, Saudi Arabia; Public Health Foundation of India, Gandhinagar, India (D Ahmad PhD); Department of Health Informatics (K Ahmad PhD), Qassim University, Buraidha, Saudi Arabia; Department of Clinical Pharmacy (R Ahmad PhD), Advanced Medical & Dental Institute (M Aziz PhD), Universiti Sains Malaysia, Penang, Malaysia; Department of Pharmacy

Practice (R Ahmad PhD), The Islamia University of Bahawalpur, Pakistan, Bahawalpur, Pakistan; St. Joseph Hospital and Medical Center, Phoenix, AZ, USA (S Ahmad MD); Punjab Medical College, Faisalabad, Pakistan (S Ahmad MD); School of Public Health (T Ahmad PhD), Zhejiang University, Hangzhou, China; Institute of Endemic Diseases (A Ahmed MSc), Faculty of Medicine (K A H Mohamed Ahmed MD), Unit of Basic Medical Sciences (E E Siddig MD), University of Khartoum, Khartoum, Sudan; Swiss Tropical and Public Health Institute (A Ahmed MSc), Department of Ophthalmology (Prof Z Gatzioufas PhD), University of Basel, Basel, Switzerland; Department of Biosciences (H Ahmed PhD), COMSATS Institute of Information Technology, Islamabad, Pakistan; Department of Epidemiology (M B Ahmed PhD), Institute of Health (Prof T Malik PhD), Institute of Health, School of Medicine (L S Tadesse MD), Jimma University, Jimma, Ethiopia; College of Medicine and Public Health (M B Ahmed PhD, G R Naik PhD), Department of Nursing and Health Sciences (S Shorofi PhD), Flinders University, Adelaide, SA, Australia; Department of Medicine (M Ahmed MD), Rawalpindi Medical University, Rawalpindi, Pakistan; Department of Assistance Medical Sciences (N Ahmed PhD), Faculty of Nursing (W T Almagharbeh PhD), Prince Fahad bin Sultan Chair for Biomedical Research (S Muthupandian PhD), University of Tabuk, Tabuk, Saudi Arabia (S Muthupandian PhD); Department of Medical Microbiology and Parasitology (N Ahmed PhD), Universiti Sains Malaysia, Kota Bharu, Malaysia; Brody School of Medicine (S Ahmed PhD), East Carolina University, Greenville, NC, USA; ICT Convergence Research Centre (S O Ajakwe PhD), Kumoh National Institute of Technology, Gumi, South Korea; Department of Microbiology, Immunology and Parasitology (G T Akalu MSc), Department of Family Medicine (S A Yesuf MSc), St. Paul's Hospital Millennium Medical College, Addis Ababa, Ethiopia; Oxford Vaccine Group (O Akeju MPH), Nuffield Department of Medicine (Prof R J Maude PhD), University of Oxford, Oxford, UK; Department of Physiology (R E Akhigbe PhD), Ladoke Akintola University, Ogbomoso, Nigeria; Department of Internal Medicine (K Akinosoglou PhD), University of Patras, Patras, Greece; Department of Internal Medicine and Infectious Diseases (K Akinosoglou PhD), University General Hospital of Patras, Patras, Greece; Department of Cardiology (M Akkaif PhD), Fudan University, Shanghai, China; Department of Infection Prevention & Control (H Akram MD), Baylor Scott & White Health, Frisco, TX, USA; VentrureBlick, (H Akram MD); Chicago College of Osteopathic Medicine (A E Akrami BS), Midwestern University, Downers Grove, IL, USA; Feinberg School of Medicine (A E Akrami BS, A Dehadrai BS, D B Srivastava BA), Department of Microbiology and Immunology (O Ebohon MPH), Medical Scientist Training Program (S Marzouk MA), Northwestern University, Chicago, IL, USA; Department of Communicable Diseases (S Al Awaidy MSc), Ministry of Health, Muscat, Oman; Middle East, Eurasia, and Africa Influenza Stakeholders Network, Muscat, Oman (S Al Awaidy MSc); The university of Jordan (M K Al nawayseh PhD), Jordanian Public Health Society, Amman, Jordan; Fundamentals and Administration Department (Prof O Al Omari PhD), Department of Adult Health and Critical Care (O A M Al Zaabi PhD), Sultan Qaboos University, Muscat, Oman; Faculty of Pharmacy (Y Al Thaher PhD), Philadelphia University, Amman, Jordan; School of Pharmacy (Y Al Thaher PhD), Division of Population Medicine (A Sha'aban PhD), Cardiff University, Cardiff, UK; School of Public Health (M A M Al Zoubi PhD), Management Policy and Community Health Department (J A Atta MPH), University of Texas, Houston, TX, USA; School of Medicine (Y Al-Ajlouni MD), New York Medical College, Valhalla, NY, USA; Department of Epidemiology (Y Al-Ajlouni MD), Columbia University, New York, NY, USA (D Shan MD); Department of Research and Development (Z Al-Aly MD), Department of Surgery (S Azadnajafabad MD, C Wang PhD), Brown School of Public Health (Y C Wondmeneh MD), Washington University in St. Louis, St. Louis, MO, USA; Clinical Epidemiology Center (Z Al-Aly MD), US Department of Veterans Affairs (VA), St. Louis, MO, USA; Murdoch Business School (K Alam PhD), Murdoch University, Perth, WA, Australia; Preventive Dentistry Department (Prof M K Alam

PhD), College of Medicine (R Basri PhD), Jouf University, Sakaka, Saudi Arabia; Department of Public Health (Prof N Alam DrPH), Sam Houston State University, Hunsville, TX, USA; Asian University for Women, Chittagong, Bangladesh (Prof N Alam DrPH); School of Nursing (R M Al-amer PhD), Faculty of Medicine (Prof M T AlBataineh PhD), Department of Basic Sciences (Z Altaany PhD), Department of Basic Medical Sciences (R A Karasneh PhD, Prof M M Khatatbeh PhD), Faculty of Nursing (H Khatatbeh PhD), Yarmouk University, Irbid, Jordan; School of Nursing and Midwifery (R M Al-amer PhD), Western Sydney University, Sydney, NSW, Australia; Department of Health Information Management and Technology (T M Alanzi PhD), Deanship of Preparatory Year and Supporting Studies (Prof S El-Ashker PhD), Division of Forensic Medicine (Prof R G Menezes MD), Imam Abdulrahman Bin Faisal University, Dammam, Saudi Arabia; School of Public Health and Interdisciplinary studies (J O Alao MSc), Auckland University of Technology, Auckland, New Zealand; Department of Clinical Pharmacy (F Y Al-Ashwal PhD), Al-Ayen Iraqi University, Thi-Qar, Iraq; Department of Clinical Pharmacy and Pharmacy Practice (F Y Al-Ashwal PhD), University of Science and Technology, Sana'a, Yemen; Department of Community and Mental Health (Prof M Albashtawy PhD), Al al-Bayt University, Mafraq, Jordan; Respiratory Therapy Program (A M Aldhahir PhD), Clinical Nutrition Department (R M Chandika PhD), Department of Public Health (S Dohare MD, W Rehman MS), Epidemiology Program (M Khan MD), College of Public Health and Tropical Medicine (J Varghese PhD), Jazan University, Jazan, Saudi Arabia; General Directorate of Research and Studies (M S Aldossary MClinDent), Research & Innovation Center (Prof Z A Memish MD), Ministry of Health, Riyadh, Saudi Arabia; School of Pharmacy (S M Aleidi PhD, Prof Y Bustanji PhD), The School of Medicine (M Al-Iede MD), The University of Jordan (Prof L A Dardas PhD), Department of Pathology, Microbiology and Forensic Medicine (M Sallam PhD), Department of Clinical Laboratories and Forensic Medicine (M Sallam PhD), The University of Jordan, Amman, Jordan; Pediatric Intensive Care Unit (A Al-Eyadhy MD, Prof M Temsah MD), Department of Computer Engineering (K Aurangzeb PhD), Department of Physiology (Prof S A Meo PhD), University Diabetes Center (A Sultan Meo MPH), King Saud University, Riyadh, Saudi Arabia; Department of Epidemiology and Biostatistics (A M Alfalki MPH), University of South Carolina, Columbia, SC, USA; Department of Bacteriology, Immunology, and Mycology (Prof A M Algammal PhD), Faculty of Veterinary Medicine (M Mabrok PhD), Suez Canal University, Ismailia, Egypt; Faculty of Dentistry (A Alhumaidi DDS), Ibn Al-Nafis University for Medical Sciences, Sana'a, Yemen; Department of Zoology (A Ali PhD), Abdul Wali Khan University Mardan, Mardan, Pakistan; Department of Statistics and Operations Research (I Ali PhD), Aligarh Muslim University, Aligarh, India; Department of Biological Sciences (L Ali PhD, A S Qazi PhD, R Ullah PhD), National University of Medical Sciences (NUMS), Rawalpindi, Pakistan; Department of Pharmacy (M Ali PhD), Mohammed Al-Mana College for Medical Sciences, Dammam, Saudi Arabia; Department of Biosciences (R Ali PhD, N Salam PhD), Centre for Interdisciplinary Research in Basic Sciences (CIRBSc) (S Anwar PhD), Centre for Interdisciplinary Research in Basic Sciences (M Hassan PhD, S K Khan MSc, T Mohammad PhD), Centre For Interdisciplinary Research In Basic Sciences (CIRBSc) (A Shamsi PhD), Jamia Millia Islamia, New Delhi, India; Centre for Biotechnology and Microbiology (S Ali PhD), University of Swat, Charbagh, Pakistan; Center for Biotechnology and Microbiology (S S Ali PhD, M Suleman PhD), University of Swat, Swat, Pakistan; Institute of Health and Wellbeing (S M Alif PhD), Federation University Australia, Melbourne, VIC, Australia; School of Public Health and Preventive Medicine (S M Alif PhD), Faculty of Medicine, Nursing, and Health Sciences (S Aslani PhD), Monash Addiction Research Center (D Z Assefa MSc), Department of Public Health and Preventive Medicine (H Hailu MPH), General Practice (T H Mekonnen MPH), Monash University, Melbourne, VIC, Australia; Department of Physics (M Alipour BSc), University of Hamburg, Hamburg, Germany; Department of Clinical and Community Pharmacy (Prof S W Al-Jabi

PhD, Prof S H Zyoud PhD), Department of Physiology, Pharmacology, and Toxicology (Prof R Shawahna PhD), Department of Chemistry (Prof A H Zyoud PhD), An-Najah National University, Nablus, Palestine; School of Physics, Mathematics and Computing (Prof A Al-Jumaily PhD), Centre for Neuromuscular and Neurological Disorders (Perron Institute) (Prof G J Hankey MD), The University of Western Australia, Perth, WA, Australia; Information and Communication Technology Research Pole (Lab-STICC) (Prof A Al-Jumaily PhD), ENSTA Bretagne, Brest, France; College of Life Sciences (Prof A Alkhatib PhD), Department of Public Health (O Aremu PhD), Birmingham City University, Birmingham, UK; Cardiovascular Divivsion (M Alkhawam MD), University of Alabama, Birmingham, AL, USA; Faculty of Medicine (Prof M Z Allouh PhD, Prof M S I Alyahya PhD, A Alzoubi PhD, R M Odat MD), Department of Allied Medical Sciences (A Alrawashdeh PhD), Department of Rehabilitation Sciences (M Al-Wardat PhD), Department of Clinical Pharmacy (Prof K H Alzoubi PhD), Department of Public Health (Prof K A Kheirallah PhD), Jordan University of Science and Technology, Irbid, Jordan; Independent Consultant, Amman, Jordan (S Al-Marwani MSc); Department of Medicine (J U Almazan PhD), Nazarbayev University, Astana, Kazakhstan; Department of Parasitology (Prof H M Al-Mekhlafi PhD), University of Malaya, Kuala Lumpur, Malaysia; Department of Parasitology (Prof H M Al-Mekhlafi PhD), Department of Emergency Medicine (M A Alsabri MD), Sana'a University, Sana'a, Yemen; Ophthalmology Department (A Almobayed MD), Neurology Department (F Mahmoudi MD), Department of Ophthalmology (S Rouzbahani MD), University of Miami, Miami, FL, USA; Faculty of Nursing (M R Alosta PhD), Zarqa University, Zarqa, Jordan; Department of Respiratory Care (J S Alqahtani PhD), Prince Sultan Military College of Health Sciences, Dammam, Saudi Arabia; American University of the Middle East, Egaila, Kuwait (M R Alqudimat PhD); Department of Prosthodontics and Implant Dentistry (A Alqutaibi PhD), Taibah University, Medinah, Saudi Arabia; Department of Prosthodontics and Implant Dentistry (A Alqutaibi PhD), Ibb University, Ibb, Yemen; Department of Nursing (I Alrimawi PhD), Georgetown University, Washington, DC, USA; Macro-Fiscal Policy Department (S M Alrousan PhD), Ministry of Finance, Dubai, United Arab Emirates; Pediatric Emergency Medicine Department (M A Alsabri MD), School of Biomedical Engineering, Science and Health Systems (M Noroozi BSc), Drexel University, Philadelphia, PA, USA; Research, Policy, and Training Directorate (A Al-Tammemi MPH), Jordan Center for Disease Control, Amman, Jordan; Department of Specialty Internal Medicine (Prof J A Al-Tawfiq MD), Johns Hopkins Aramco Healthcare, Dhahran, Saudi Arabia; Department of Medicine (Prof J A Al-Tawfiq MD), Indiana University School of Medicine, Indianapolis, IN, USA; Department of Respiratory Therapy (M A Althobiani PhD), Respiratory Therapy Unit (M A Althobiani PhD), Department of Family and Community Medicine (Prof N S Butt PhD), Rabigh Faculty of Medicine (Prof A Malik PhD), Department of Dental Public Health (Z S Natto DrPH), Department of Community Medicine (S Samargandy PhD), King Abdulaziz University, Jeddah, Saudi Arabia; Research Group in Health Economics (Prof N Alvis-Guzman PhD), Universidad de Cartagena (University of Cartagena), Cartagena, Colombia; Research Group in Hospital Management and Health Policies (Prof N Alvis-Guzman PhD), Department of Economic Sciences (N J Alvis-Zakzuk MSc), Universidad de la Costa (University of the Coast), Barranquilla, Colombia; National Health Observatory (N J Alvis-Zakzuk MSc), National Institute of Health, Bogota, Colombia; Department of Clinical Pharmacology and Toxicology (H Alwafi PhD), Umm Al-Qura University, Makkah, Saudi Arabia; Department of Medical Sciences (Prof Y M Al-Worafi PhD), Azal University for Human Development, Sana'a, Yemen; Department of Clinical Sciences (Prof Y M Al-Worafi PhD), University of Science and Technology of Fujairah, Fujairah, United Arab Emirates; Department of Pediatrics (Prof H Aly MD), Lerner College of Medicine (M Balkis MD), Internal Medicine Department (A Goyal MD), Lerner Research Institute (Prof X Liu PhD), Department of Cardiovascular Medicine (J Rajendran MD), Cleveland Clinic,

Cleveland, OH, USA; Maternal and Child Health Division (MCHD) (M Al-Zubayer MSc), Maternal and Child Health Division (R Banik MS, S Noor MS), Department of Maternal and Child Health (L Hossain MPH), International Centre for Diarrhoeal Disease Research, Bangladesh, Dhaka, Bangladesh; London School of Hygiene and Tropical Medicine (E J Amafah MSc), University of London, London, UK; Molecular and Medicine Research Center (S Amini PhD), Department of Laboratory Sciences (K Ghaffari PhD), Laboratory Science Department (J Karami PhD), Operating Room Department (A Sedigh PhD), Khomein University of Medical Sciences, Khomein, Iran; Department of Polymer Chemistry and Technology (A Amobonye PhD), Kaunas University of Technology, Kaunas, Lithuania; Department of Maternal and Child Wellbeing (D A Amugsi PhD), African Population and Health Research Center, Nairobi, Kenya; Center for Biomedical Image Computing & Analytics (F Anagnostakis MD), Department of Pathobiology (U M Femoe Membe PhD), Department of Medicine (J C Lin BS), Center for Global Health (K Ma DDS), Department of Biostatistics, Epidemiology, and Informatics (J Puvvula PhD), University of Pennsylvania, Philadelphia, PA, USA; Department of Biomedical and Neuromotor Sciences (S Guicciardi MD), Department of Medicine and Surgery (I Papadimopoulos MD), Department of Medical and Surgical Sciences (Prof F S Violante MD), University of Bologna, Bologna, Italy (F Anagnostakis MD); Centre for Primary Care & Health Services Research (M Anderson PhD), University of Manchester, Manchester, UK; Department of Internal Medicine (S Ang MD), Rutgers University, Toms River, NJ, USA; Department of General Medicine (N Anh MD), Thai Binh University of Medicine and Pharmacy in Vietnam, Thai Binh City, Viet Nam; Department of Pharmacology (A Anil MD), Department of Community Medicine and Family Medicine (Prof P Bhardwaj MD, A Prakash MBBS), School of Public Health (Prof P Bhardwaj MD), Department of Biochemistry (S Tomo MD), All India Institute of Medical Sciences, Jodhpur, India; All India Institute of Medical Sciences, Bhubaneswar, India (A Anil MD); Department of Microbiology (A A Anjorin PhD), Lagos State University, Ojo, Nigeria; Reproductive Immunology Research Center (H Ansariniya PhD), Immunology Department (H Ansariniya PhD), Department of Biostatistics and Epidemiology (M Taheri Soodejani PhD), Shahid Sadoughi University of Medical Sciences, Yazd, Iran; Department of Environmental and Occupational Health (B S Anuoluwa MPH), Health Information Management (A E Babatope MPH), Mathematical and Computer Sciences (O A Okunlola MSc), Department of Biosciences and Biotechnology (O T Oyeyemi PhD), Mathematics and Computer Sciences, Epidemiology and Biostastistics (O Peter PhD), Department of Biochemistry (A J Salemcity PhD), University of Medical Sciences, Ondo, Ondo, Nigeria; Regenerative Medicine, Organ Procurement and Transplantation Multi-disciplinary Center (S Anvari MD), Gastrointestinal and Liver Disease Research Center (B Eftekhari MD), Gastrointestinal and Liver Diseases Research Center (N Faraji MSc, F Joukar PhD), Department of Social Medicine and Epidemiology (A Feizkhah MD), Caspian Digestive Disease Research Center (F Joukar PhD), Guilan University of Medical Sciences, Rasht, Iran; School of Chemical and Life Sciences (SCLS) (S Anwar PhD), Jamia Hamdard, New Delhi, India; Department of Pharmaceutical Biotechnology (J M Aranjani PhD), Department of Physiotherapy (A Arumugam PhD, M K Sinha PhD), Prasanna School of Public Health (R Kamath MHA), Kasturba Medical College Manipal (D L C MD, J P Raj DM), Department of Pharmaceutical Regulatory Affairs and Management (V S Ligade PhD), Manipal College of Nursing (S G Nayak PhD), Department of Hepatology (S Rajpurohit PhD), Department of Community Medicine (C R Rao MD), Manipal Academy of Higher Education, Manipal, India; College of Art and Science (D Areda PhD), Ottawa University, Surprise, AZ, USA; School of Life Sciences (D Areda PhD), Arizona State University, Tempe, AZ, USA; Department of Veterinary Pharmacology and Toxicology (A Aremu PhD), Department of Veterinary Public Health and Preventive Medicine (I A Odetokun PhD), University of Ilorin, Ilorin, Nigeria; Department of Biotechnology-Faculty of Science and Humanities (Prof

J Arockiaraj PhD), Department of Biotechnology (T Sundaram PhD), Sri Ramaswamy Memorial Institute of Science and Technology, Kattankulathur, India; University College of Medicine & Dentistry (Prof M Arooj PhD), University Institute of Food Science and Technology (S Bashir PhD), University Institute of Radiological Sciences and Medical Imaging Technology (S Farooq PhD, Prof Z Fatima PhD), University College of Dentistry (M Hassan PhD), University Institute of Diet and Nutritional Sciences (A Khalil PhD), Institute of Molecular Biology and Biotechnology (M Khan PhD, T Maqbool PhD, S Shahid PhD), Department of Technology (M Muzaffar MBA), Research Centre for Health Sciences (RCHS) (M Muzaffar MBA, S Shahid PhD, M Umar MBA), University Institute of Public Health (S Nargus PhD), Department of Physics (W Shahid PhD), Lahore Business School (M Umar MBA), Faculty of Sciences (Prof A B Waqar PhD), The University of Lahore, Lahore, Pakistan; Institute for Biomedical Problems (A A Artamonov PhD), Russian Academy of Sciences, Moscow, Russia; Department of Periodontics (D Arumuganainar PhD), Department of Biosciences (S Chopra MPH), Saveetha Medical College and Hospital (Prof M Karobari PhD), Saveetha Dental College and Hospitals (G Minervini PhD, M Tovani-Palone PhD), Department of Biomaterials (N Rabiee PhD), Department of Microbiology (S Sankar PhD), Department of Oral and Maxillofacial Surgery (B Venkidasamy PhD), Saveetha University, Chennai, India; Department of Clinical Disciplines (N Aryntayeva MSPH), Al Farabi Kazakh National University, Almaty, Kazakhstan; College of Medicine (M Asadi Anar MD), University of Arizona, Tucson, AZ, USA; Department of Community Medicine and Global Health (M Asaduzzaman MPH), Department of Pharmacy (A Desalegn PhD), University of Oslo, Oslo, Norway; Department of Pharmacy Practice (Prof S Asdaq PhD), College of Medicine (M Fareed PhD), AlMaarefa University, Riyadh, Saudi Arabia; Pioneer Journal of Biostatistics and Medical Research (PJBMR), Pakistan, Pakistan (T Ashraf PhD); Deakin Health Economics/School of Health and Social Development (B K Y Asiamah-Asare PhD), Deakin University, Melbourne, VIC, Australia; School of Traditional Chinese Medicine (M Aslam PhD), Xiamen University Malaysia, Sepang, Malaysia; Nursing Department (Y Asri PhD), Faculty of Health Science (Y Asri PhD), Institute of Technology and Health Science RS dr Soepraoen, Malang, Indonesia; Hospital and Research Centre (S R Atre PhD), Department of Oral Pathology and Microbiology (Prof S C Sarode PhD), Dr. D. Y. Patil Vidyapeeth Pune (Deemed to be University), Pune, India; Center for Clinical Global Health Education (S R Atre PhD), School of Public Health (A A Berihun MA), Department of Biostatistics (A Columbus MS), Johns Hopkins University, Baltimore, MD, USA (E Melese MD); Department of Forensic Medicine (A Atreya MD), Department of Community Medicine (S Nepal MD), Lumbini Medical College, Palpa, Nepal; College of Medicine (Prof Z A Atwan PhD), University of Basrah, Basrah, Iraq; Department of Health Sciences (M Augello MD), Department of Clinical Sciences and Community Health (Prof C La Vecchia MD), University of Milan, Milan, Italy; Clinic of Infectious Diseases and Tropical Medicine (M Augello MD), ASST Santi Paolo e Carlo, Milan, Italy; School of Veterinary Medicine (Prof B B Awosile PhD), Texas Tech University, Amarillo, TX, USA; Medicinal Chemistry Unit (Y O Ayipo PhD), Kwara State University, Malete, Ilorin, Nigeria; Centre for Drug Research (Y O Ayipo PhD), Universiti Sains Malaysia, Pinang, Malaysia; Department of Anesthesia (S A Aziz PhD), Cihan University -Sulaimaniya, Sulaymaniyah, Iraq; Department of Basic Sciences (S A Aziz PhD), College of Science (F M Rahman PhD), University of Sulaimani, Sulaymaniyah, Iraq (H H R Najmuldeen PhD); ASIDE Healthcare, Lewes, DE, USA (A Azzam MD); Faculty of Medicine (A Azzam MD), October 6 University, 6th of October City, Egypt; Physiology Department (R Babiker PhD), Department of Medical Microbiology and Immunology (Prof G A Menezes PhD), RAK College of Nursing (M Mohamed PhD), RAK Medical and Health Sciences University, Ras Al Khaimah, United Arab Emirates; Department of Forensic Science (A D Badiye PhD, H Bansal MSc, N Kapoor PhD), Government Institute of Forensic Science Nagpur, Nagpur, India; Rashtrasant Tukadoji

Maharaj Nagpur University, Nagpur, India (A D Badiye PhD); College of Optometry (R Bahreini MS), Pacific University, Forest Grove, OR, USA; Community Medicine Department (Y Bahurupi MD), All India Institute of Medical Sciences, Nagpur, India; International Medical School (A A Baig PhD), Management and Science University, Alam, Malaysia; Division of Biological Sciences (S Balakrishnan PhD), Tamil Nadu State Council for Science and Technology, Chennai, India; Chen Senior Medical Center, Tamarac, FL, USA (M Balkis MD); Department of Anatomy (S Barati PhD), Saveh University of Medical Sciences, Saveh, Iran; Alpha Genomics Private Limited, Islamabad, Pakistan (Z Basharat PhD); Barcelona Institute for Global Health (Prof Q Bassat MD), Universitat de Barcelona, Barcelona, Spain; Catalan Institution for Research and Advanced Studies (ICREA), Barcelona, Spain (Prof Q Bassat MD); Department of Community Medicine (S Basu MD), ESI-PGIMSR, ESIC Medical College & Hospital Joka, Kolkata, Kolkata, India; Department of Medical Education (K Batra PhD), School of Public Health (R Batra MS), Department of Social and Behavioral Health (Prof M Sharma PhD), University of Nevada Las Vegas, Las Vegas, NV, USA; IT Department (R Batra MS), Coforge, Georgia, GA, USA; Department of Human Anatomy and Histology (Prof N Beeraka PhD), Department of Epidemiology and Evidence-Based Medicine (Prof N I Briko DSc, V A Korshunov PhD, P D Lopukhov PhD, R V Polibin PhD), I.M. Sechenov First Moscow State Medical University, Moscow, Russia; Milken Institute of Public Health (B K Bekele MPH), Department of Global Health (R S Bernstein MD), George Washington University, Washington, DC, USA; Department of Public Health (T T Bekuma MPH), Department of Pharmacy (G Fekadu PhD), Wollega University, Nekemte, Ethiopia; School of Public Health (S Belachew PhD), Centre for Clinical Research (Prof C L Lau PhD), The University of Queensland, Brisbane, QLD, Australia (M Moni PhD); Department of Public Health (M Belayneh PhD), University of South Africa, Pretoria, South Africa; Department of Medicine and Surgery (M Belingheri MD), University of Milano - Bicocca, Milano, Italy; Direzione Sanitaria (M Belingheri MD), Fondazione IRCCS San Gerardo dei Tintori, Monza, Italy; Department of Physiotherapy and Paramedicine (U M Bello PhD), Glasgow Caledonian University, Glasgow, UK; BRAC James P Grant School of Public Health (S Bente Kamal Tune MPH), BRAC University, Dhaka, Bangladesh; Department of Epidemiology and Biostatistics (A C Bermudez MD), Department of Health Policy and Administration (Prof F B Garcia PhD), Department of Neurosciences (Prof R G Jamora PhD), University of the Philippines Manila, Manila, Philippines; Department of Epidemiology (A C Bermudez MD), Department of Pathology and Laboratory Medicine (T Emran DSc), Brown University, Providence, RI, USA; Hubert Department of Global Health (R S Bernstein MD), Emory University, Atlanta, GA, USA; Department of Community and Family Medicine (A S Bhadoria MD), All India Institute of Medical Sciences, Rishikesh, India; Community Health Department (A S Bhadoria MD), University of South Wales, South Wales, UK; Department of Public Health (A S Bhagavathula PhD), North Dakota State University, Fargo, ND, USA; Institute of Applied Health Research (N Bhala PhD), Division of Ophthalmology & Visual Sciences (Prof G D Panos MD), University of Nottingham, Nottingham, UK; Institute of Applied Health Research (N Bhala PhD), Department of Metabolism and Systems Science (S Tariq PhD), University of Birmingham, Birmingham, UK; School of Nursing and Midwifery (D Bhandari PhD), Department of Anatomy and Developmental Biology (Y Mathangasinghe PhD), Monash University, Clayton, VIC, Australia; School of Public Health (D Bhandari PhD), University of Adelaide, Adelaide, SA, Australia; Department of Internal Medicine (A Bhargava MD), Wayne State University, Gross Pointe Woods, MI, USA; Global Health Neurology Lab (S Bhaskar MD), NSW Brain Clot Bank, Sydney, NSW, Australia; Division of Cerebrovascular Medicine and Neurology (S Bhaskar MD), National Cerebral and Cardiovascular Center, Suita, Japan; The Translational and Clinical Research Institute (P Bhattacharjee MD), Newcastle University, Newcastle upon Tyne, UK; Department of Clinical Medicine (P Bhattacharjee MD), Cambridge University Hospitals NHS Foundation

Trust, Cambridge, UK; Department of Statistics (K Bhattacharyya MSc), University of Calcutta, Kolkata, India; NTAGI Secretariat (K Bhattacharyya MSc), National Institute of Health and Family Welfare, New Delhi, India; Program Management Department (A H Bhattarai MPH), Emotional Well-Being Institute Canada, Burnaby, BC, Canada; Department of Human Genetics and Molecular Medicine (Prof J Bhatti PhD, Prof A Munshi PhD, S Senapati PhD), Department of Microbiology (P K Kushawaha PhD, A Singh PhD, M Yadav PhD), Department of Biochemistry (B Singh PhD), Department of Computer Science & Engineering (Prof S Singh PhD), Central University of Punjab, Bathinda, India; TAF Uludag Winter Training Center (C Bilgin MD), Turkish Ministry of Defence, Bursa, Turkiye; Department of Community Medicine and Family Medicine (B Biswas MD), All India Institute of Medical Sciences, Deoghar, India; Department of Biochemistry and Biotechnology (M Biswas PhD), University of Science and Technology Chittagong, Chittagong, Bangladesh; Centre for the Study of Regional Development (M Biswas PhD), Jawahar Lal Nehru University, New Delhi, India; Department of Physical Education and Health (B Bizzozero-Peroni PhD), Universidad de la República, Rivera, Uruguay; Centre for Disability Research and Policy (F T Bobo PhD), Faculty of Medicine and Health (W He PhD, M M Kamal MPH), University of Sydney, Sydney, NSW, Australia; Department of Community and Family Medicine (Prof T Bodhare MD), All India Institute of Medical Sciences, Ramanathapuram, India; Faculty of Psychology, Education and Sport (L Bohn PhD), University Lusofona, Porto, Portugal; Research Centre for Physical Activity, Health, and Leisure (L Bohn PhD), Research Unit on Applied Molecular Biosciences (UCIBIO) (Prof F Carvalho PhD), Institute for Research and Innovation in Health (i3S) (Prof N Cruz-Martins PhD), UCIBIO Applied Molecular Biosciences Uni (Prof D Dias da Silva PhD), University of Porto, Porto, Portugal; Department of Demography and Population Studies (O A Bolarinwa MSc), University of Witwatersrand, Johannesburg, South Africa; Ophthalmology Department (P Bolourinejad MD), Department of Health Services Management (M Mohseni PhD), Heart Failure Research Center (S Najdaghi MD), Neuroscience Research Center (S Najdaghi MD), Isfahan University of Medical Sciences, Isfahan, Iran; Facultad de Salud (Faculty of Health) (Prof A Botero Carvajal PhD), Universidad Santiago de Cali (Santiago de Cali University), Cali, Colombia; Department of Medicine (Prof S Bouaoud DrPH), Faculty of Medicine (Prof A Ouyahia PhD), University Ferhat Abbas of Setif, Sétif, Algeria; Department of Epidemiology and Preventive Medicine (Prof S Bouaoud DrPH), University Hospital Saadna Abdenour, Sétif, Algeria; Department of Epidemiology (D Braithwaite PhD), Department of Health Services Research, Management and Policy (R Wang MPP), University of Florida, Gainesville, FL, USA; Cancer Population Sciences Program (D Braithwaite PhD), University of Florida Health Cancer Center, Gainesville, FL, USA; Division of Clinical Epidemiology and Aging Research (Prof H Brenner MD), German Cancer Research Center, Heidelberg, Germany; Department of Woman and Child Health and Public Health (D Buonsenso MD), Fondazione Policlinico Universitario A. Gemelli IRCCS (Agostino Gemelli University Polyclinic IRCCS), Rome, Italy; Global Health Research Institute (D Buonsenso MD), Department of Health Science and Public Health (Prof L Villani MD), Università Cattolica del Sacro Cuore (Catholic University of Sacred Heart), Rome, Italy; Department of Radiology (F Busch MD), Department of Public Health and Primary Care (Prof P Willeit PhD), University of Cambridge, Cambridge, UK; School of Public Health Sciences (Z A Butt PhD), University of Waterloo, Waterloo, ON, Canada; Al Shifa School of Public Health (Z A Butt PhD), Al Shifa Trust Eye Hospital, Rawalpindi, Pakistan; Faculty of Health Sciences Healthcare Management Department (M Çakmak Barsbay PhD), Ankara University, Ankara, Turkiye; Department of Internal and Geriatric Medicine (Prof L A Cámera MD), Hospital Italiano de Buenos Aires (Italian Hospital of Buenos Aires), Buenos Aires, Argentina; Board of Directors (Prof L A Cámera MD), Argentine Society of Medicine, Buenos Aires, Argentina; Unit of Hygiene and Public Health (A Capodici MD), Romagna Local Health

Authority, Forlì-Cesena, Italy; Interdisciplinary Research Center for Health Science (A Capodici MD), Sant'Anna School of Advanced Studies, Pisa, Italy; Institute for Cancer Research, Prevention and Clinical Network, Florence, Italy (G Carreras PhD); Department of Medicine and Surgery (A Carugno PhD), University of Insubria, Varese, Italy; Department of Psychiatry (Prof J Castaldelli-Maia PhD), University of Sao Paulo, São Paulo, Brazil; Public Health Department (C A Castañeda-Orjuela PhD), Epidemiology and Public Health Evaluation Group (C A Castañeda-Orjuela PhD), Department of Public Health (Prof F P De la Hoz PhD), National University of Colombia, Bogota, Colombia; Department of Medical, Surgical, and Health Sciences (Prof L Cegolon PhD), Clinical Department of Medical, Surgical, and Health Sciences (Prof S Di Bella MD), University of Trieste, Trieste, Italy; Public Health Unit (Prof L Cegolon PhD), University Health Agency Giuliano-Isontina (ASUGI), Trieste, Italy; Department of Nutrition (Prof F Cembranel DSc), Federal University of Santa Catarina, Florianópolis, Brazil; College of Public Health, Medical, and Veterinary Sciences (M Cenderadewi MPHTM), Department of Public Health and Tropical Medicine (T I Emeto PhD), James Cook University, Townsville, QLD, Australia; Department of Public Health (M Cenderadewi MPHTM), University of Mataram, Mataram, Indonesia; Infection and Global Health Research (M Cevik MD), University of St Andrews, St Andrews, UK; Regional Infectious Diseases Unit (M Cevik MD), NHS National Services Scotland, Edinburgh, UK; Department of Biotechnology (Prof C Chakraborty PhD), Adamas University, Kolkata, India; Institute for Skeletal Aging & Orthopedic Surgery (Prof C Chakraborty PhD), Hallym University, Chuncheon, South Korea; State Disease Investigation Laboratory (S Chakraborty M.V.Sc), Animal Resources Development Department, Agartala, India; Temerty Faculty of Medicine (V Chattu MD), University of Toronto, Toronto, ON, Canada; Department of Community Medicine (V Chattu MD), Datta Meghe Institute of Medical Sciences, Sawangi, India; EPI (G B Chemeda MPH), Oromia Health Bureau, Addis Ababa, Ethiopia; Fuwai Hospital (A Chen PhD), Chinese Academy of Medical Sciences, Beijing, China; Faculty of Humanities and Health Sciences (H Chen MSc), Curtin University, Miri, Malaysia; Clinical Research Center (H Chen PhD), Zhujiang Hospital of Southern Medical University, Guangzhou, China; Yong Loo Lin School of Medicine (N W Chew MBChB), Department of Medicine (B Chong MBBS), SingHealth Duke-NUS Global Health Institute (K Lwin PhD), Department of Surgery (K Tan PhD), National University of Singapore, Singapore, Singapore; Division of Infectious Diseases (P R Ching MD), Virginia Commonwealth University, Richmond, VA, USA; Department of Clinical Oncology (W C S Cho PhD), Queen Elizabeth Hospital, Hong Kong, China; Centre for Research Impact & Outcome (H Chopra PhD), Chitkara University, Rajpura, India; The Interdisciplinary Research Group on Biomedicine and Health (D Chu PhD), Faculty of Applied Sciences (D Chu PhD), VNU International School (VNUIS), Hanoi, Viet Nam; Department of Health Informatics (S Chung PhD), Division of Medicine (T Oyelade PhD), Department of Population Health Sciences (D Sunkersing PhD), University College London, London, UK; Health Data Research UK, London, UK (S Chung PhD); Department of Health Behavior (S Chung MPH), Texas A&M University, College Station, TX, USA; School of Nursing and Midwifery (M Chutiyami PhD), School of Life Sciences (G Liu PhD), University of Technology Sydney, Sydney, NSW, Australia; Nova Medical School (Prof J Conde PhD), Nova University of Lisbon, Lisbon, Portugal; Department of Respiratory Medicine and Allergology (Prof A Corlateanu PhD), Nicolae Testemitanu State University of Medicine and Pharmacy, Chisinau, Moldova; Department of Health Sciences (C Cosma MD, M Del Riccio MD), University of Florence, Florence, Italy; Life and Health Sciences Research Institute (ICVS) (Prof N Cruz-Martins PhD), University of Minho, Braga, Portugal; Department of Epidemiology and Biostatistics (B Dabo MSPH), University of South Florida, Tampa, FL, USA; Department of Medical Microbiology and Parasitology (B Dabo MSPH), Bayero University, Kano, Nigeria; Research Center for Child Psychiatry (O Dadras PhD), Heart Center (V Kytö MD), University of

Turku, Turku, Finland; Department of Medical and Surgical Sciences and Advanced Technologies "GF Ingrassia" (Prof E D'Amico MD), Department of Clinical and Experimental Medicine (Prof C Ledda PhD), University of Catania, Catania, Italy; Public Health Foundation of India, Gurugram, India (Prof L Dandona MD, Prof R Dandona PhD, G Kumar PhD); Department of Brain Sciences (L D'Anna PhD), WHO Collaborating Centre for Public Health Education and Training (Q Lin MPH, D L Rawaf MD), Department of Primary Care and Public Health (Prof S Rawaf MD, C Tabche MSc), The George Institute for Global Health (Prof S Yaya PhD), Imperial College London, London, UK; Department of Public Health (S D Darcho MPH), College of Heath and Medical Sciences (N S Samuel MSc), Department of Epidemiology and Biostatistics (B S Tusa MPH), Haramaya University, Harar, Ethiopia; Department of Pediatrics (Prof G L Darmstadt MD), Stanford University School of Medicine, Stanford, CA, USA; Institute of Research and Development (A M Darwesh PhD), School of Computer Science (Prof M Hosseinzadeh PhD), Duy Tan University, Da Nang, Viet Nam; Department of Information Technology (A M Darwesh PhD), University of Human Development, Sulaymaniyah, Iraq; Department of Pediatrics (S Deekonda MD), Brookdale University Hospital Medical Center, Brooklyn, NY, USA; Department of Medical Biochemistry (T A Dejenie MSc), College of Medicine and Health science (G W Geremew MSc), Department of Epidemiology and Biostatistics (H A Getahun MSc), Department of Internal Medicine (E Melese MD), School of Nursing (H B Netsere MSc), Department of Clinical Pharmacy (A K Sendekie MSc), Department of Pharmacy (M C Worku MSc), University of Gondar, Gondar, Ethiopia; Infectieziekten in de Eerste Lijn Programme (M Del Riccio MD), Netherlands Institute for Health Services Research, Utrecht, Netherlands; Ophthalmology Department (M Delsoz MD), University of Tennessee, Memphis, TN, USA; Department of Anesthesiology (H Deng PhD), Central South University, Changsha, China; Center for Nutrition and Health Research (E Denova-Gutiérrez DSc), National Institute of Public Health, Cuernavaca, Mexico; Department of Cardiology (P Devarakonda MD, M Vinayak MD), Department of Psychiatry (S Gunturu MD), Department of Neurosurgery (K Margetis MD), Icahn School of Medicine at Mount Sinai, New York, NY, USA (A Shaikh MD); Department of Pharmacy (S Dewan PhD), United International University, Dhaka, Bangladesh; Pharmacology Division (S Dewan PhD), Center for Life Sciences Research Bangladesh, Dhaka, Bangladesh; Sheffield Teaching Hospitals NHS Foundation Trust, Sheffield, UK (A Dhali MBBS); Division of Pathology (K Dhama PhD), ICAR-Indian Veterinary Research Institute, Bareilly, India; Research Department (M Dhimal PhD, B P Marasini PhD), Nepal Health Research Council, Kathmandu, Nepal; Institute of Occupational, Social and Environmental Medicine (M Dhimal PhD), Goethe University, Frankfurt am Main, Germany; Department of Pharmacy Practice (S Dhingra PhD), National Institute of Pharmaceutical Education and Research Hajipur, Hajipur, India; Population Interventions Unit (B Dhungel DrPH), School of Health Sciences (A Meretoja MD), University of Melbourne, Melbourne, VIC, Australia; Department of Life Science and Public Health (M Di Pumpo DrPH), Università Cattolica del Sacro Cuore, Rome, Italy; Escola Superior de Saúde (Prof D Dias da Silva PhD), Instituto Politécnico do Porto, Porto, Portugal; Faculty of Science (Prof D Diaz PhD), Health Policy and Population Research Center (CIPPS) (A Martinez-Valle PhD), National Autonomous University of Mexico, Mexico City, Mexico; Joe C. Wen School of Population & Public Health (X Ding MA), University of California Irvine, Irvine, CA, USA; Department of Medicine (T C Do MD), Department of General Medicine (N N Y Nguyen MD), Faculty of Medicine (N N Y Nguyen MD), Pham Ngoc Thach University of Medicine, Ho Chi Minh City, Viet Nam; Departamento de Responsabilidade Social (Department of Social Responsibility) (W M dos Santos PhD), Oswaldo Cruz German Hospital, São Paulo, Brazil; Brazilian Centre for Evidence-based Healthcare (W M dos Santos PhD), Joanna Briggs Institute, São Paulo, Brazil; Independent Consultant, South Plainfield, NJ, USA (O P Doshi MS); Department of Epidemiology (M

Dresse MD), University of Pittsburgh, Pittsburgh, PA, USA; Department of Psychiatry (M Dresse MD), University of Pittsburgh Medical Center, Pittsburgh, PA, USA; Office of Institutional Analysis (J Dube MA), University of Windsor, Windsor, ON, Canada; Faculty of Science and Humanities (S Duraisamy PhD), SRM Institute of Science and Technology, Kattankulathur, India; Department of Infection and Tropical Medicine (O C Durojaiye MPH), School of Medicine and Population Health (N S George MPH), University of Sheffield, Sheffield, UK; Department of Biological and Chemical Sciences (O Ebohon MPH), Michael and Cecilia Ibru University, Delta State, Nigeria; Histology Department (L L M Ebraheim PhD), Zagazig university, zagazig, Egypt; Environmental and Occupational Health Research Center (M - Ebrahimi MD), Shahroud University of Medical Sciences, Shahroud, Iran; Higher School of Technology (Prof A Ed-Dra PhD), Sultan Moulay Slimane University, Beni Mellal, Morocco; Advanced Nursing Department (F Efendi PhD), Department of Epidemiology Population Biostatistics and Health Promotion (A Hargono PhD), Universitas Airlangga (Airlangga University), Surabaya, Indonesia; Isenberg School of Management (A Eighaei Sedeh MD), University of Massachusetts Amherst, Amherst, MA, USA; Department of Anesthesia, Critical Care and Pain Medicine (Prof J Kang PhD), Department of Orthopaedics (O Subasi PhD), Massachusetts General Hospital, Boston, MA, USA (A Eighaei Sedeh MD, M Kim MD); Private Orthodontist, Ahvaz, Iran (E Eini MSD); Faculty of Science and Health (M Ekholuenetale PhD), University of Portsmouth, Hampshire, UK; Almoosa College of Health Sciences, Al Ahsa, Saudi Arabia (R A El Arab PhD); Department of Public Health and Community Medicine (Prof I F El Bayoumy DrPH), Tanta University, Tanta city, Egypt; School of Public Health (Prof I F El Bayoumy DrPH), Texila American University, Guyana, Guyana; Clinical Pathology Department-Faculty of Medicine (Prof M El Sayed Zaki PhD), Department of Anatomy and Embryology (M A Eladl PhD), Department of Clinical Pathology (Prof M Elshaer PhD), Department of Cardiology (Prof M M Ramadan PhD), Faculty of Pharmacy (Prof M A Saleh PhD), Rheumatology and Immunology Unit (Prof S Tharwat MD), Mansoura University, Mansoura, Egypt; Internal Medicine Residency (A Elalfy DO), Mayo Clinic, Jacksonville, FL, USA; College of Medicine (M Elhadi MD, Prof S Jeong PhD), Department of Medicine (I R Fakhradiyev PhD), School of Health and Environmental Science (Prof J Kang PhD), Department of Health Policy and Management (Prof J Kim PhD), Korea University, Seoul, South Korea; Houston Methodist Hospital, Houston, TX, USA (M Elhadi MD); School of Pharmacy and Pharmaceutical Sciences (M Elnaem PhD), Ulster University, Coleraine, UK; Faculty of Veterinary Medicine (Prof A S A Eltahawy PhD), Damanhour University, Damanhur, Egypt; Health Research and Technology Transfer Directorate (M Endriyas MSc), South Ethiopia Region Public Health Institute, Jinka, Ethiopia; Department of Public Health (M Endriyas MSc), Hawassa University, Hawassa, Ethiopia; Australian Center for Precision Health (S Eshetie MSc), University of South Australia, Adelaide, SA, Australia; Wassa Amenfi East Municipal Health Directorate (G Eshun BSc), Ghana Health Service, Wassa Akropong, Ghana; Department of Bacteriology and Virology (M Eslami PhD), Department of Immunology (D Haghmorad PhD), Cancer Research Center (D Haghmorad PhD), Semnan University of Medical Sciences, Semnan, Iran; Cancer Research Center (M Eslami PhD), Semnan University of Medical Scinces, Semnan, Iran; Gastroenterology and Hepatology Department (M Eslami MD), Department of Radiology (F Nugen PhD), Department of Physiology and Biomedical Engineering (F Pourghazi MD), Department of Endocrinology (M Salehi MD), Department of Hematology (M S Sra MD), Mayo Clinic, Rochester, MN, USA; Department of Public Health (F Eva MPH), North South University, Dhaka, Bangladesh; Department of Anesthesia (A O Fadaka PhD), Cincinnati Children's Hospital Medical Center, Cincinnati, OH, USA; Department of Biotechnology (A O Fadaka PhD), School of Pharmacy (O C Okonji MSc), University of the Western Cape, Cape Town, South Africa; Department of Electrical and Computer Engineering (H Fadavian MSc), Tarbiat Modares University, Tehran, Iran; Research Centre for Healthcare

and Community (A F Fagbamigbe PhD), Centre for Intelligent Healthcare (H Liu PhD), Coventry University, Coventry, UK; Department of Oral Biology (A Fahim PhD), Riphah International University, Islamabad, Pakistan (Z Z Piracha PhD); Independent Consultant, Male, Maldives (R Faiz MPH); Leishmaniasis Research Center (M Faramarzpour MD), Pathology and Stem Cell Research Center (M Faramarzpour MD), Department of Immunology (Prof A Jafarzadeh PhD), Physiology Research Center (M Rajizadeh PhD), Kerman University of Medical Sciences, Kerman, Iran; Saveetha Medical College and Hospital (M Fareed PhD), Saveetha Institute of Medical and Technical Sciences (SIMATS), Chennai, India; Department of Psychology (A Faro PhD), Federal University of Sergipe, São Cristóvão, Brazil; Department of Chemistry and Biochemistry (E T Fasusi MSc), University System of Georgia, Statesboro, GA, USA; Pharmacy Department (E T Fasusi MSc), Department of Community Medicine (O S Ilesanmi PhD), Department of Medicine (Prof M O Owolabi DrM), Department of Oral and Maxillofacial Surgery (A A Salami BDS), University College Hospital, Ibadan, Ibadan, Nigeria; Department of Infectious Diseases and Public Health (G Fekadu PhD, Prof W Ming MD), Department of Biomedical Sciences (W Jin MD), City University of Hong Kong, Hong Kong, China; Department of Microbiology and Physiology (U M Femoe Membe PhD), Department of International Health and Sustainable Development (Prof A Kisa PhD), School of Medicine (F Musaigwa PhD), Department of Environmental Health Sciences (S P Sherchan PhD), Tulane University, New Orleans, LA, USA (E Lytvyak MD); Cardiovascular Health and Imaging Laboratory (R Fernandez-Jimenez PhD), Centro Nacional de Investigaciones Cardiovasculares (CNIC), Madrid, Spain; Department of Cardiology (R Fernandez-Jimenez PhD), Hospital Clinico San Carlos, IdISSC, Madrid, Spain; Deparment of Biological Sciences (N Feter PhD), University of Southern California, Los Angeles, CA, USA; School of Medicine (N Feter PhD), Federal University of Rio Grande do Sul, Porto Alegre, Brazil; Institute of Public Health (F Fischer PhD), Charité Universitätsmedizin Berlin (Charité Medical University Berlin), Berlin, Germany; Department of Cardiac, Thoracic, Vascular Sciences and Public Health (M Fonzo MD), University of Padova, Padova, Italy; Department of Dermatology (T Fukumoto PhD), Kobe University, Kobe, Japan; Department of Community Medicine (Prof M A Gadanya MD), Aminu Kano Teaching Hospital, Kano, Nigeria; School of Public Health (D Gadeka PhD), School of Pharmacy (Prof I A Kretchy PhD), University of Ghana, Legon, Ghana; Department of Oral Biology and Experimental Dental Research (M Gajdács PhD), University of Szeged, Szeged, Hungary; School of Public Health (B Ganesan PhD), Institute of Health & Management, Melbourne, VIC, Australia; Department of Biostatistics (Prof X Gao PhD), Key Lab of Environment and Health (Prof X Gao PhD), School of Public Health (Prof W Song PhD, Q Wang PhD, Prof W Wang PhD, L Xiao PhD, X Yang PhD, T Zhan PhD), Xuzhou Medical University, Xuzhou, China; Department of Public Health (B Garba PhD), SIMAD University Mogadishu. Somalia, Mogadishu, Somalia; Infectious Diseases Unit (J Garlasco MD), University of Verona, Verona, Italy; Midwifery Department (A Gashaw MSc), Dilla University, Dilla, Ethiopia; Department of Pharmacology (Prof R K Gautam PhD), Indore Institute of Pharmacy, Indore, India; Department of Midwifery (M W Gebregergis MSc), Department of Medical Laboratory Sciences (H N Meles MSc), Adigrat University, Adigrat, Ethiopia; Institute of Public Health (N S George MPH), Jagiellonian University Medical College, Krakow, Poland; Akwa Ibom State University (U U George PhD), University of Uyo, Akwa Ibom State, Nigeria; Department of Public Health (G K Getahun MPH), Menelik II Medical and Health Science College, Addis Ababa, Ethiopia; Department of Nursing (F B Getaneh MSc), Wollo University, Dessie, Ethiopia; Research Committee (A Ghamkhar BSc), Qom University of Medical Sciences, Qom, Iran; School of Medicine (H Ghasrsaz MD), Mazandaran University of Medical Sciences, Mazandaran, Iran; Family and Community Medicine Department (R M Ghazy PhD), King Khalid University, Abha, Saudi Arabia; Department of Statistics (G Gheno PhD), Ronin Institute, Montclair, NJ,

USA; Research Group for Childhood Cancer (N Ghith PhD), Cancer Research Institute, Danish Cancer Society, Copenhagen, Denmark; Department of Biology (A Ghuge MPhil), Government Institute of Science, Nagpur, India; Department of Clinical Research (A Ghuge MPhil), National Institute For Research In Reproductive and Child Health, Mumbai, India; Country Office (A U Gil PhD), World Health Organization (WHO), Astana, Kazakhstan; Anesthesiology and Critical Care Medicine (A Girombelli MD), Ente Ospedaliero Cantonale, lugano, Switzerland; Department of Cardiac Surgery (Prof L Göbölös PhD), Cleveland Clinic Abu Dhabi, Abu Dhabi, United Arab Emirates; Lerner College of Medicine (Prof L Göbölös PhD), Department of Quantitative Health Science (Prof X Liu PhD), Department of Endocrinology (A Sood MD), Case Western Reserve University, Cleveland, OH, USA; Department of Hepatology (Prof A Goel DM), Sanjay Gandhi Postgraduate Institute of Medical Sciences, Lucknow, India; Department of Health Systems and Policy Research (Prof M Golechha PhD), Indian Institute of Public Health, Gandhinagar, India; Department of Life Sciences, Health and Healthcare Professions (Prof D Golinelli MD), Link Campus University, Rome, Italy; Health Services Research, Evaluation and Policy Unit (Prof D Golinelli MD), AUSL della Romagna, Ravenna, Italy; Department of Epidemiology and Biostatistics (S Guan MD), Anhui Medical University, Hefei, China; Health Direction (G Guarducci MD), Local Health Authority of Ferrara, Ferrara, Italy; Department of the Health Directorate (S Guicciardi MD), Local Health Authority of Bologna, Bologna, Italy; Department of Cardiology (A Gulati MD), Icahn School of Medicine at Mount Sinai, New York City, NY, USA; Department of Psychiatry (S Gunturu MD), Bronxcare Health System, Bronx, NY, USA; Department of Nephrology (A K Gupta PharmD), Max Super Speciality Hospital, New Delhi, India; Non-communicable Diseases Division (NCD) (A K Gupta PharmD), Indian Council of Medical Research, New Delhi, India; Department of Internal Medicine (I Gupta MD), Independent Consultant, Bharatpur, India; Independent Consultant, Delhi, India (I Gupta MD); Department of Toxicology (S Gupta PhD), Shriram Institute for Industrial Research, Delhi, India; School of Medicine (V Gupta PhD), Deakin University, Geelong, VIC, Australia; Faculty of Medicine Health and Human Sciences (Prof V K Gupta PhD), Australian Institute of Health Innovation (P Peprah MSc), Macquarie University, Sydney, NSW, Australia; Department of Epidemiology and Psychosocial Research (R A Gutiérrez PhD), Ramón de la Fuente Muñiz National Institute of Psychiatry, Mexico City, Mexico; Department of Biomedical Gerontology (R S Gutiérrez-Murillo PhD), Pontifical Catholic University of Rio Grande do Sul, Porto Alegre, Brazil; Research Unit (J Guzman-Esquivel PhD), Mexican Institute of Social Security, Colima, Mexico; Department of Global Health and Population (A Haakenstad ScD), T.H. Chan School of Public Health, Boston, MA, USA; DCMSPH (P Halder MD), Department of Pharmacology (A K Kakkar MD), Department of Pediatrics (J Kumar MD), Post Graduate Institute of Medical Education and Research, Chandigarh, India; CCM (P Halder MD), Department of Biophysics (T Mohammad PhD), Department of Laboratory Medicine (A Singh PhD), All India Institute of Medical Sciences, New Delhi, India; Department of Pharmacy (Prof I M Hamad PhD), American University of Madaba, Amman, Jordan; Biochemistry Department (Prof N M Hamdy PhD), Department of Entomology (A M Samy PhD), Medical Ain Shams Research Institute (MASRI) (A M Samy PhD), Ain Shams University, Cairo, Egypt; Department of Public Health (S Hameed PhD), Green International University, Lahore, Lahore, Pakistan; School of Health and Environmental Studies (Prof S Hamidi DrPH), Hamdan Bin Mohammed Smart University, Dubai, United Arab Emirates; Sakarya University, Turkey, Sakarya, Turkiye (A Hanif PhD); Stroke Research Centre (Prof G J Hankey MD), Perron Institute for Neurological and Translational Science, Perth, WA, Australia; Medical Research Unit (H Harapan PhD), Universitas Syiah Kuala (Syiah Kuala University), Banda Aceh, Indonesia; Research Unit (J M Haro MD), Parc Sanitari Sant Joan de Deu, Barcelona, Spain; Department of Mental Health (J M Haro MD), Carlos III Health Institute (Prof R Tabarés-Seisdedos PhD), Biomedical

Research Networking Center for Mental Health Network (CiberSAM), Madrid, Spain; Department of Public Health (M Hasan MPH), Tropical Disease and Health Research Center, Dhaka, Bangladesh; Department of Biotechnology (A Hasnain PhD), Lahore University of Biological and Applied Sciences, Lahore, Pakistan; Department of Medicine (I Hassan MD), University of Khartoum Faculty of Medicine, Khartoum, Sudan; Institute of Pharmaceutical Sciences (K Hayat MS), University of Veterinary and Animal Sciences, Lahore, Pakistan; Department of Pharmacy Administration and Clinical Pharmacy (K Hayat MS), Xian Jiaotong University, Xian, China; Department of Neurology (M I Hegazy PhD), Faculty of Pharmacy (H Mohamed PhD), Faculty of Medicine (H Mowafy MD), Faculty of Medicine, Clinical and Chemical Pathology Department (Prof M M S Soliman MD), Kasr-AlAiny Faculty of Medicine (Prof N S Soliman MD), Cairo University, Cairo, Egypt; Independent Consultant, Santa Clara, CA, USA (G Heidari MD); Community-Oriented Nursing Midwifery Research Center (M Heidari PhD), Shahrekord University of Medical Sciences, Shahrekord, Iran; Australian Centre for Health Service Innovations (S A Hewage MD), Australian Centre for Health Services Innovation (Q Xia PhD), Queensland University of Technology, Brisbane, QLD, Australia; National Agency for Strategic Research in Medical Sciences Education (M Heydari PhD), Ministry of Health and Medical Education, Tehran, Iran; Department of Microbiology (K Hezam PhD), Taiz University, Taiz, Yemen; School of Medicine (K Hezam PhD), Nankai University, Tianjin, China; Graduate School of Medicine (Y Hiraike PhD), Department of Global Health Policy (Prof S Nomura PhD, S K Rauniyar PhD), University of Tokyo, Tokyo, Japan; Department of Physics (A Hossain PhD), Department of Population Science and Human Resource Development (Prof M Rahman PhD, Prof M Rahman DrPH), University of Rajshahi, Rajshahi, Bangladesh; Department of Decision and Information Sciences (M Hossain DrPH), Department of Biology and Biochemistry (S Ullah MSc), University of Houston, Houston, TX, USA; Public Health Research Group (M Hossain DrPH), Nature Study Society of Bangladesh, Khulna, Bangladesh; Department of Statistics (M Hossain BSc, M Islam BSc, S Noor MS), Shahjalal University of Science and Technology, Sylhet, Bangladesh; Center for Health Innovation, Research, Action and Learning - Bangladesh (CHIRAL Bangladesh) (M Hossain MSc), Jagannath University, Dhaka, Bangladesh; Jadara Research Center (Prof M Hosseinzadeh PhD), Jadara University, Irbid, Jordan; School of Health and Biomedical Sciences (M Hossen PhD), Royal Melbourne Institute of Technology (RMIT) University, Melbourne, VIC, Australia; Department of Internal Medicine (M Hostiuc PhD), Department of Dermatology (C N Matei PhD, M Tampa PhD), Department of General Surgery (I Negoi PhD, D Serban PhD), Carol Davila University of Medicine and Pharmacy, Bucharest, Romania; Internal Medicine Department (P Hotwani MD), Parkview Health, Fort wayne, IN, USA; Department of Medicine (P Hotwani MD, N Shaikh MBBS), Liaquat University Of Medical and Health Sciences, Jamshoro, Pakistan; Centre for Global Health Inequalities Research (CHAIN) (H Hoven DrPH), Norwegian University of Science and Technology (NTNU), Trondheim, Norway; Institute for Occupational and Maritime Medicine (ZfAM) (H Hoven DrPH), University Medical Center Hamburg-Eppendorf (UKE), Hamburg, Germany; Department of Psychology (C Hu PhD), Tsinghua University, Beijing, China; Faculty of Medicine (J Huang MD), School of Public Health and Primary Care (L Yao MSc), The Chinese University of Hong Kong, Hong Kong, China; Nephrology and Urology Research Center (K Hushmandi PhD), Baqiyatallah University of Medical Sciences, Tehran, Iran; Department of Biological Sciences and Chemistry (Prof J Hussain PhD), Natural and Medical Sciences Research Center (A Khan PhD, S A Shahid MPhil, A Ullah MS), Dept of Biological Sciences and Chemistry (Z Naureen PhD), University of Nizwa, Nizwa, Oman; Department of Social Sciences and Business (Prof M Hussain PhD), Roskilde University, Roskilde, Denmark; Department of Biomolecular Sciences (Prof N R Hussein PhD), Department of Biology (K S Ibrahim PhD), University of Zakho, Zakho, Iraq; Artur Riggs Diabetes & Metabolism Research

Institute (Prof M I Husseiny PhD), Cancer Prevention and Research Institute, Duarte, CA, USA; Department of Pathology (Prof M M Metwally PhD), Cardiovascular Department (Prof A M A Saad MD), Department of Microbiology and Immunology (G Yahya PhD), Zagazig University, Zagazig, Egypt (Prof M I Husseiny PhD); International Master Program for Translational Science (H Huynh BS), School of Pharmacy (B Iskandar PhD), School of Nursing (M Kurniasari PhD), International Ph.D. Program in Medicine (M H N Le MD), Research Center for Artificial Intelligence in Medicine (M H N Le MD), College of Medicine (K Nguyen PhD), Taipei Medical University, Taipei, Taiwan; Department of Occupational Safety and Health (Prof B Hwang PhD), China Medical University, Taiwan, Taichung, Taiwan; Department of Occupational Therapy (Prof B Hwang PhD), Asia University, Taiwan, Taichung, Taiwan; Nursing Department (N Ibrahim MSc), University of Massachusetts Boston, Boston, MA, USA; West Africa RCC (O S Ilesanmi PhD), Africa Centre for Disease Control and Prevention, Abuja, Nigeria; Faculty of Medicine (I M Ilic PhD, A Stevanović MD), University of Belgrade, Belgrade, Serbia; Faculty of Medical Sciences (Prof M D Ilic PhD), University of Kragujevac, Kragujevac, Serbia; Department of Clinical Pharmacy (M Imam PhD), Prince Sattam bin Abdulaziz University, Al Kharj, Saudi Arabia; Faculty of Health and Life Sciences (A Inok PhD), University of Exeter, Exeter, UK; Department of Microbiology (M A Isa PhD), University of Maiduguri, Maiduguri, Nigeria; Department of Biotechnology (M A Isa PhD), Sharda University, Greater Noida, India; Department of Pharmaceutical Technology (B Iskandar PhD), Sekolah Tinggi Ilmu Farmasi Riau, Pekanbaru, Indonesia; Independent Researcher, Cairo, Egypt (T R Iskander BSc); Research and Publication Department (M Islam MSc), World Health Organization (WHO), Dhaka, Bangladesh; Institute for Physical Activity and Nutrition (Prof S Islam PhD), Deakin University, Burwood, VIC, Australia; Clinical Laboratory Department (F Ismail PhD), Tobruk University, Tobruk, Libya; Department of Blood Transmitted Diseases (F Ismail PhD), National Centre for Disease Control (NCDC), Tobruk, Libya; Department of Medicine (M C Ituka MD), University of Yaoundé, Yaounde, Cameroon; Department of Health Services Research (M Iwagami PhD), University of Tsukuba, Tsukuba, Japan; Department of Non-Communicable Disease Epidemiology (M Iwagami PhD), London School of Hygiene & Tropical Medicine, London, UK; Department of Physical and Medicine (L Jacob MD), Université Paris Cité, Paris, France; Research and Development Unit (L Jacob MD), Biomedical Research Networking Center for Mental Health Network (CiberSAM), Barcelona, Spain; Department of Nursing, Arak School of Nursing (A Jadidi PhD), Arak University of Medical Sciences, Arak, Iran; College of Medicine and Health Sciences (H Jahrami PhD), Arabian Gulf University, Manama, Bahrain; Government Hospitals, Manama, Bahrain (H Jahrami PhD); Department of Oral Pathology and Microbiology (A Jain MDS), Department of Neurology (Prof H S Malhotra DM), Internal Medicine Department (J Tewari MBBS), King George's Medical University, Lucknow, India; Department of Health and Safety (A A Jairoun PhD), Dubai Municipality, Dubai, United Arab Emirates; UNESCO-TWAS Section of Economic & Social Sciences, Humanities & Arts (Prof M Jakovljevic PhD), The World Academy of Sciences UNESCO-TWAS, Trieste, Italy; Shaanxi University of Technology, Hanzhong, China (Prof M Jakovljevic PhD); Department of Neurosurgery (M Jalloh MD), Division of Hematology and Oncology (J F Wu MD), Medical College of Wisconsin, Milwaukee, WI, USA; Public Health Sciences (A Jamal BS), University of Chicago, Chicago, IL, USA; SRM Medical College Hopsital and Research Centre (J James MD), Sri Ramaswamy Memorial Institute of Science and Technology, Chengelpet, India; Graduate School of Public Health (H Jamil MD), St. Luke's International University, Tokyo, Japan; Division of Population Data Science, Institute for Cancer Control (H Jamil MD), National Cancer Center, Tokyo, Japan; Institute for Neurosciences (Prof R G Jamora PhD), St. Luke's Medical Center, Bonifacio Global City, Philippines; Department of Medicine (S Javaid MD), University of Mississippi Medical Center, Jackson, MS, USA; Department of Medicine (S Javaid MD),

Jinnah Sindh Medical University, Karachi, Pakistan; Department of Pharmacology (T Jawaid PhD), Imam Mohammad Ibn Saud Islamic University, Riyadh, Saudi Arabia; Department of Nursing (Q Jawell Odah Abed PhD), Middle Technical University of Kut Technical Institute, Baghdad, Iraq; Department of Biochemistry (Prof S Jayaram MD), Government Medical College, Mysuru, India; Department of Community Medicine (R P Jha MSc), Dr. Baba Saheb Ambedkar Medical College & Hospital, Delhi, India; Department of Community Medicine (R P Jha MSc), Institute of Medical Sciences (S Singh PhD), Banaras Hindu University, Varanasi, India; Department of Orthopedics (W Jin MD), Department of Epidemiology and Biostatistics (Prof S Mubarik PhD, Prof C Yu PhD), Wuhan University, Wuhan, China; Faculty of Veterinary Medicine (M Jokar DVM), University of Calgary, Calgary, AB, Canada; NGSM Institute of Pharmaceutical Sciences (J Jose PhD), Nitte University, Mangalore, India; Department of Community Medicine (J Jose MD), Jubilee Mission Medical College & Research Institute, Thrissur, Thrissur, India; Department of Community Medicine (N Joseph MD), Department of Forensic Medicine and Toxicology (Prof J Padubidri MD), Manipal Academy of Higher Education, Mangalore, India; Department of Economics (C E Joshua BSc), National Open University, Benin City, Nigeria; Manipal College of Health Professions (K Josten MSc), Department of Gastroenterology and Hepatology (Prof S Shetty MD), Manipal Academy of Higher Education, Udupi, India; Department of Family Medicine and Public Health (J J Jozwiak PhD), University of Opole, Opole, Poland; School of Public Health (Z Kabir PhD), University College Cork, Cork, Ireland; Research Department (Z Kabir PhD), Tobacco free Research Institute Ireland, Dublin, Ireland; Department of Oral and Maxillofacial Pathology (V Kadashetti MDS), KIMS Deemed to be University, Karad, India; Department of Statistics (Prof D H Kadir PhD), Salahaddin University, Erbil, Iraq; Department of Business Administrations (Prof D H Kadir PhD), Cihan University-Erbil, Erbil, Iraq; Canberra Business School (M M Kamal MPH), University of Canberra, Hawker, ACT, Australia; College of Pharmacy (M Kamal PhD), Jamia Hamdard, Al Kharj, Saudi Arabia; Care and Public Health Research Institute (CAPHRI) (R Kamath MHA), Maastricht University, Maastricht, Netherlands; Department of Public Health (R T Kamorudeen MPH), South Wales University, Treforest, UK; Osun State Hospital Management Board, (R T Kamorudeen MPH); Department of Biostatistics and Epidemiology (N Kamyari PhD), Abadan University of Medical Sciences, Abadan, Iran; Microbiology, Virology and Immunology Department (Prof O Kamyshnyi DSc), I. Horbachevsky Ternopil National Medical University, Ternopil, Ukraine; Department of Health Sciences (Prof M Kanaan PhD), University of York, York, UK; Department of Rehabilitation Sciences (S F Kanaan PhD), College of Medicine (Prof Y Kinfu PhD, Prof M A Yassin MD), Qatar University, Doha, Qatar; Cephas Health Research Initiative Inc, Ibadan, Nigeria (Prof K K Kanmodi DDS); Department of Community Medicine (S Kannan S MD), ESIC Medical College and Hospital Chennai, Chennai, India; The Hansjörg Wyss Department of Plastic and Reconstructive Surgery (R S Kantar MD), NYU Langone Health, New York, NY, USA; Cleft Lip and Palate Surgery Division (R S Kantar MD), Global Smile Foundation, Norwood, MA, USA; Department of Anesthesiology (I M Karaye MD), Montefiore Medical Center, Bronx, NY, USA; Institute for Epidemiology and Social Medicine (A Karch MD), University of Münster, Münster, Germany; Amity Stem Cell Institute (ASCI) (Prof M K Kashyap PhD), Department of Mathematics (Prof K Kumar PhD), Amity University Haryana, Gurugram, India; Amity Institute of Forensic Sciences (H Khajuria PhD, B P Nayak PhD), Amity Institute of Pharmacy (K Munjal PhD), Amity Institute of Public Health (M Shannawaz PhD), Amity institute of Public Health and Hospital Administration (P S Singh PhD), Amity University, Noida, India; Cancer and Cardiovascular Research Building (M Khaksar MD), Division of Health Policy and Management (R R Parikh MD), University of Minnesota, Minneapolis, MN, USA; Cellular and Molecular Research Center (M Khaksar MD), Department of Biostatistics (M Moradi PhD), Department of Microbiology (M Saki PhD), Alimentary Tract Research Center (Z Shokati

Eshkiki PhD), Ahvaz Jundishapur University of Medical Sciences, Ahvaz, Iran; College of Health Sciences (N Khalid PhD), Abu Dhabi University, Abu Dhabi, United Arab Emirates; School of Food and Agricultural Sciences (N Khalid PhD), University of Management and Technology, Lahore, Pakistan; Halal Research Center of the Islamic Republic of Iran (IRI) (F Khamesipour PhD), Iran Food and Drug Administration, Tehran, Iran; National Institute of Translational Virology and AIDS Research (A Khan PhD), Indian Council of Medical Research, Pune, India; Department of Pharmacy Administration and Clinical Pharmacy (F U Khan PhD), Department of Epidemiology and Biostatistics (Prof J Liu PhD), Peking University, Beijing, China (X Zhang MPH); Department of Pharmacy Administration and Clinical Pharmacy (F U Khan PhD), Department of Thoracic Surgery (P Li PhD), The First Affiliated Hospital of Xi'an Jiaotong University, Xi'an, China; Department of Community Medicine (M Khan MPH), National Institute of Preventive and Social Medicine, Dhaka, Bangladesh; Primary Care Department (M A Khan MSc), NHS North West London, London, UK; Department of Community and Preventive Medicine (R Khan MD), King Edward Medical University, Lahore, Pakistan; Department of Medicine (U Khan MD), Department of Sociology, Anthropology, and Public Health (C Park DrPH), University of Maryland, Baltimore, MD, USA; Department of Cardiology (Z Khan MD), University of South Wales, Treforest, UK; Department of Cardiology (Z Khan MD), University of Buckingham, Buckingham, UK; Department of Health (V Khanal PhD), Nepal Development Society, Chitwan, Nepal; Department of Preventable Non Communicable Disease (V Khanal PhD), Menzies School of Health Research, Alice Springs, NT, Australia; Department of Pharmacology (S U Khasbage MD), All India Institute of Medical Sciences, Raipur, India; College of Health, Wellbeing and Life Sciences (Prof K Khatab PhD), Sheffield Hallam University, Sheffield, UK; College of Arts and Sciences (Prof K Khatab PhD), Ohio University, Zanesville, OH, USA; Asadabad School of Medical Sciences (A Khazaei PhD), Asadabad School of Medical Sciences, Asadabad, Iran; Department of Pediatrics (G Kim MD), Case Western Reserve University School of Medicine, Cleveland, OH, USA; Division of Pediatric Hospital Medicine (G Kim MD), UH Rainbow Babies and Children's Hospital, Cleveland, OH, USA; School of Medicine (Prof K Kim PhD), Creighton University, Omaha, NE, USA; Cardiovascular Disease Initiative (M Kim MD), Broad Institute of MIT and Harvard, Cambridge, MA, USA; Millennium Prevention, Inc., Westwood, MA, USA (R W Kimokoti MD); Pacific Community, Noumea, New Caledonia (Prof Y Kinfu PhD); School of Health Sciences (Prof A Kisa PhD), Kristiania University College, Oslo, Norway; Department of Nursing and Health Promotion (S Kisa PhD), Oslo Metropolitan University, Oslo, Norway; Department of Public Health Dentistry (Prof S KM MD), Krishna Vishwa Vidyapeeth (Deemed to be University), Karad, India; Global Healthcare Consulting, New Delhi, India (S Kochhar MD); Department of General Practice and Family Medicine (Prof O Korzh DSc), Department of Infectious Diseases (Prof A Sokhan PhD), Kharkiv National Medical University, Kharkiv, Ukraine; Department of Epidemiology (Prof K Kostev PhD), IQVIA, Frankfurt am Main, Germany; University Hospital Marburg, Marburg, Germany (Prof K Kostev PhD); Department of Internal and Pulmonary Medicine (Prof P A Koul MD), Sheri Kashmir Institute of Medical Sciences, Srinagar, India; Department of Public Health (J Kretchy PhD), Central University, Accra, Ghana (J Kretchy PhD); Department of Anthropology (Prof K Krishan PhD), Institute of Forensic Science & Criminology (V Sharma PhD), Panjab University, Chandigarh, India; Department of Demography (Prof B Kuate Defo PhD), Department of Social and Preventive Medicine (Prof B Kuate Defo PhD), University of Montreal, Montreal, QC, Canada; Department of Biotechnology (R H Kuchay PhD), Baba Ghulam Shah Badshah University, Jammu and Kashmir, India; Department of Pediatrics (I Kuitunen PhD), Kuopio University Hospital, Kuopio, Finland; Institute of Clinical Medicine (I Kuitunen PhD), University of Eastern Finland, Kuopio, Finland; Center of Medicine and Public Health (M Kulimbet MSc), Director of Central Asia Research Collaboration Group

(Prof F Rahim PhD), Asfendiyarov Kazakh National Medical University, Almaty, Kazakhstan; Department of Health Administration and Education (E Kumah PhD), University of Education, Winneba, Winneba, Ghana; Department of Community Medicine (D Kumar MD), Rajendra Institute of Medical Sciences, Ranchi, India; Department of Pharmacy Practice (N Kumar PhD), University of Sindh, Jamshoro, Pakistan; Department of Public Health (S Kundu MPH), Griffith University, Gold Coast, QLD, Australia; Section of Cardiology (Prof S K Kunutsor PhD), University of Manitoba, Winnipeg, MB, Canada; Translational Health Sciences (Prof S K Kunutsor PhD), University of Bristol, Bristol, UK; Faculty of Medicine and Health Science (M Kurniasari PhD), Universitas Kristen Satya Wacana, Salatiga, Indonesia; National Research and Innovation Agency (BRIN), Jakarta, Indonesia (A Kusnali MA); Department of Public Health and Epidemiology (D Kusuma DSc), Khalifa University of Science and Technology, Abu Dhabi, United Arab Emirates; Faculty of Public Health (D Kusuma DSc, Prof I Trihandini PhD), University of Indonesia, Depok, Indonesia; Department of Rehabilitation Sciences (W P Kwong PhD, J S Usman PhD), Hong Kong Polytechnic University, Hong Kong, China; Department of Environment and Public Health (F Kyei-Arthur PhD), University of Environment and Sustainable Development, Somanya, Ghana; Clinical Research Center (V Kytö MD), Turku University Hospital, Turku, Finland; Division of Evidence Synthesis (C Lahariya MD), Foundation for People-centric Health Systems, New Delhi, India; Division of Lifestyle Medicine (C Lahariya MD), Centre for Health: The Specialty Practice, New Delhi, India; Unidad de Genética y Salud Pública (Prof I Landires MD), Instituto de Ciencias Médicas, Las Tablas, Panama; Ministry of Health (Prof I Landires MD), Hospital Joaquín Pablo Franco Sayas, Las Tablas, Panama; Department of Otorhinolaryngology (S Lasrado MS), Father Muller Medical College, Mangalore, India; Faculty of Medicine (H Le MD, N Le MD), Department of Internal Medicine (T H Tran MD), University of Medicine and Pharmacy at Ho Chi Minh City, Ho Chi Minh City, Viet Nam (T D T Le MD); Department of Cardiovascular Research (H Le MD, N Le MD), Methodist Hospital, Merrillville, IN, USA; Independent Consultant, Ho Chi Minh City, Viet Nam (T D T Le MD); Department of Precision Medicine (Prof S Lee MD), Department of Integrative Biotechnology (N Pham MS), Sungkyunkwan University, Suwon, South Korea; Department of Family Medicine (W Lee PhD), University of Texas Medical Branch, Galveston, TX, USA; Faculty of Science (E Leong PhD), Universiti Brunei Darussalam (University of Brunei Darussalam), Bandar Seri Begawan, Brunei; Department of Health Promotion and Health Education (M Li PhD), National Taiwan Normal University, Taipei, Taiwan; Department of Psychiatry (W Li PhD), Department of Genetics (S Pawar PhD), Department of Radiology and Biomedical Imaging (S Rahmani MD), Yale University, New Haven, CT, USA; Department of Food Science and Human Nutrition (Q Lin MPH), Iowa State University, Ames, IA, USA; David Geffen School of Medicine (P Y Liu MPH), Radiology Department (N Nguyen BSc), University of California Los Angeles, Los Angeles, CA, USA; College of Mathematics and Computer (Prof Z Liu PhD), Xinyu University, Xinyu, China; Department of Molecular Epidemiology (E Llanaj PhD), German Institute of Human Nutrition Potsdam-Rehbrücke, Potsdam, Germany; German Center for Diabetes Research (DZD), München-Neuherberg, Germany (E Llanaj PhD); School of Medicine (J López-Gil PhD), Universidad Espíritu Santo(University of the Americas), Samborondón, Ecuador; Vicerrectoría de Investigación y Postgrado, Osorno, Chile (J López-Gil PhD); School of Medicine (Prof G Lucchetti PhD), Federal University of Juiz de Fora, Juiz de Fora, Brazil; Department of Community and Family Medicine (A Ludhiadch PhD), All India Institute of Medical Sciences, Bathinda, India; Zhujiang Hospital (P Luo MD), Southern Medical University, Guangzhou, China; Dodoma Medical Research Centre (A M Lutambi PhD), National Institute for Medical Research, Dodoma, Tanzania; Department of Clinical Data Science and Evidence (L Lv PhD), Novo Nordisk, Plainsboro, NJ, USA; College of Engineering (Prof M D Lytras PhD), Effat University, Jeddah, Saudi Arabia; Management of Information Systems Department

(Prof M D Lytras PhD), The American College of Greece, Aghia Paraskevi, Greece; Department of Medicine (E Lytvyak MD), University of Alberta, Edmonton, AB, Canada; Department of Surgery (A M. Afifi MD), University of Toledo, Toledo, OH, USA; Department of Dermatology (K Ma DDS), Massachusetts General Hospital, Boston, USA; Centre for Public Health and Wellbeing (Z Ma PhD), University of the West of England, Bristol, UK; School of Public Health (S Y Ma'aruf MSc), University of Saskatchewan, Saskatoon, SK, Canada; Department of Medical Laboratory Science (S Y Ma'aruf MSc), Kaduna State University, Kaduna, Nigeria; Department of Microbiology and Parasitology (M Mabrok PhD), Faculty of Veterinary Medicine, King Salman International University, South of Sinai, Egypt; Department of Periodontology (Prof M Machoy PhD), Pomeranian Medical University, Szczecin, Poland; Department of Biostatistics and Epidemiology (F Madadizadeh PhD), Yazd University of Medical Sciences, Yazd, Iran; Institute for Health and Bioeconomy (A M Madureira-Carvalho PhD), One Health Toxicology Research Unit (1H-TOXRUN) (A M Madureira-Carvalho PhD), University Institute of Health Sciences, Gandra, Portugal; Department of Emergency Medicine (S Mahalingam MD), Sri Lakshmi Narayana Institute of Medical Science, Puducherry, Pondicherry, India; Department of One Health in Tropical Infectiousness Diseases (S A Mahamed MSc), Jigjiga University, Jigjiga, Ethiopia; College of Health Science (S A Mahamed MSc), Amoud University, Borama, Somalia; Research Center (N H Mahmood PhD), Cihan University-Sulaimaniya, Sulaymaniyah, Iraq; Department of Clinical and Hospital Pharmacy (M A Mahmoud PhD), Ashok and Rita Patel Institute of Physiotherapy, Al-Madinah Al-Munawarrah, Saudi Arabia; Department of Biochemistry (S Malik MSc), All India Institute of Medical Sciences, Gorakhpur, India; Global One Health (B T Mamo MD), Ohio State University, Addis Ababa, Ethiopia; Internal Medicine Department (L Manjani MD), MedStar Health, Washington, DC, USA; International Center for Chemical and Biological Sciences (F Mansoor MS, S Ullah MSc), University of Karachi, Karachi, Pakistan; Department of Biotechnology (B P Marasini PhD), Tribhuvan University, Kathmandu, Nepal; Department of Biomedical Engineering (H Marateb PhD, M Noroozi BSc), University of Isfahan, Isfahan, Iran; Automatic Control Department (ESAII) and Institute for Research and Innovation in Health (IRIS) (H Marateb PhD), Universitat Politècnica de Catalunya (Barcelona Tech - UPC), Barcelona, Spain; Department of Surgical Sciences (M Marks-Hultström PhD), Department of Medical Cell Biology (M Marks-Hultström PhD), Uppsala University, Uppsala, Sweden; Joint Learning Network for Universal Coverage, Arlington, VA, USA (A Martinez-Valle PhD); Campus Fortaleza (F R Martins-Melo PhD), Federal Institute of Education, Science and Technology of Ceará, Fortaleza, Brazil; Department of Nutrition and Dietetics (M Martorell PhD), Centre for Healthy Living (M Martorell PhD), University of Concepción, Concepción, Chile; Faculty of Humanities and Health Sciences (Prof R R Marzo MD), Curtin University, Sarawak, Malaysia; Jeffrey Cheah School of Medicine and Health Sciences (Prof R R Marzo MD), Monash University, Subang Jaya, Malaysia; Department of Clinical and Experimental Medicine (Prof S Masi PhD), University of Pisa, Pisa, Italy; Board of Directors (C N Matei PhD), Association of Resident Physicians, Bucharest, Romania; Department of Anatomy, Genetics and Biomedical Informatics (Y Mathangasinghe PhD), Postgraduate Institute of Medicine (S N K Navaratna MD), University of Colombo, Colombo, Sri Lanka; Department of Community Medicine (M Mathur MD), Geetanjali Medical College and Hospital, Udaipur, India; Community Medicine Department (N Mathur MD, A Roy MD), Apollo Institute of Medical Sciences and Research, Hyderabad, India; Department of Epidemiology (Prof R J Maude PhD), Mahidol-Oxford Tropical Medicine Research Unit, Bangkok, Thailand; Department of Paediatrics (C N P Mbachu PhD), Nnamdi Azikiwe University, Nnewi, Nigeria; Department of Obstetrics and Gynaecology (Prof I I Mbachu MBBS), Department of Paediatrics (C A Nri-Ezedi PhD), Nnamdi Azikiwe University, Awka, Nigeria; Australian Centre for Health Services Innovation (Prof S M McPhail

PhD), Queensland University of Technology, Kelvin Grove, QLD, Australia; Digital Health and Informatics Directorate (Prof S M McPhail PhD), Queensland Health, Brisbane, QLD, Australia; National Child Health Program (M P Medel Salas MD), Ministry of Health, Santiago, Chile; Department of Dental Research Cell (Prof V Mehta PhD), Dr. D. Y. Patil University, Pune, India; Department of Biosciences and Bioengineering (S Mehto PhD), Indian Institute of Technology Dharwad, Dharwad, India; Department of Clinical Infection, Microbiology and Immunity (J Meiring PhD), University of Liverpool, Liverpool, UK; Department of Public Health (T Mekene Meto MPH), Arba Minch University, Arba Minch, Ethiopia; College of Medicine (Prof Z A Memish MD), College of Pharmacy (R M H Temsah PharmD), Alfaisal University, Riyadh, Saudi Arabia; Universidad Científica del Sur, Lima, Peru (W Mendoza MD); Debre Berhan University (L A Mengstie MSc), School of Nursing and Midwifery (B Taye MSc), Department of Pediatrics and Child Health Nursing (W Z Teklehaimanot MSc), Debre Berhan University, Debre Berhan, Ethiopia; International Dx Department (A A Mentis MD), BGI Genomics, Copenhagen, Denmark; General Administration Department (A Meretoja MD), Helsinki University Hospital, Helsinki, Finland; University Centre Varazdin (T Mestrovic PhD), University North, Varazdin, Croatia; Department of Paediatrics (Prof S Mettananda DPhil), University of Kelaniya, Ragama, Sri Lanka; University Paediatrics Unit (Prof S Mettananda DPhil), Colombo North Teaching Hospital, Ragama, Sri Lanka; Department of Pathology (I Michalek PhD), Maria Sklodowska-Curie National Research Institute of Oncology, Warsaw, Poland; Multidisciplinary Department of Medical-Surgical and Dental Specialties (G Minervini PhD), University of Campania Luigi Vanvitelli, Naples, Italy; Department of Statistics and Econometrics (A Mirica PhD), Bucharest University of Economic Studies, Bucharest, Romania; College of Healthcare Management and Economics (V Mishra PhD), Department of Biomedical Sciences (P Sengupta PhD), Gulf Medical University, Ajman, United Arab Emirates; Research and Development Department (V Mishra PhD), Panacea Institute of Interdisciplinary Research and Education, Varanasi, India; Health Sciences Division Pharmacy Program (H Mohamed PhD), Higher Colleges of Technology, Dubai, United Arab Emirates; College of Applied and Natural Science (J Mohamed MSc), University of Hargeisa, Hargeisa, Somalia; Nursing College (M Mohamed PhD), Sohag University, Sohag, Egypt; Molecular Biology Unit (N S Mohamed MSc), Bio-Statistical and Molecular Biology Department (N S Mohamed MSc), Sirius Training and Research Centre, Khartoum, Sudan; Health Systems and Policy Research Unit (Prof S Mohammed PhD), Department of Community Medicine (A A Olorukooba MD), Ahmadu Bello University, Zaria, Nigeria; Heidelberg Institute of Global Health (HIGH) (Prof S Mohammed PhD), Heidelberg University, Heidelberg, Germany; Medical Microbiology Department (Prof Y Mohammed FWACP), Usmanu Danfodiyo University, Sokoto, Nigeria; Medical Microbiology Department (Prof Y Mohammed FWACP), Usmanu Danfodiyo University Teaching Hospital, Sokoto, Nigeria; Department of Pharmacology (S Mohan PhD), Dale View College of Pharmacy and Research Centre, Thruvananthapuram, India; School of Health Sciences (S Mohan PhD), University of Petroleum and Energy Studies, Dehradun, India; School of Medicine (P Mokhtarzadehazar MD, S Pourasghary MD, S Soraneh MD), Urmia University of Medical Sciences, Urmia, Iran; Clinical Epidemiology and Public Health Research Unit (L Monasta DSc, G Zamagni MSc), Burlo Garofolo Institute for Maternal and Child Health, Trieste, Italy; AI & Cyber Futures Institute (M Moni PhD), Charles Sturt University, Bathurst, NSW, Australia; Department of Epidemiology and Biostatistics (Y Moradi PhD), Neurosciences Research Center (M Naghshbandi MD), Social Determinants of Health Research Center (A Shokri PhD), Kurdistan University of Medical Sciences, Sanandaj, Iran; Computer, Electrical, and Mathematical Sciences and Engineering Division (P Moraga PhD), King Abdullah University of Science and Technology, Thuwal, Saudi Arabia; Department of Applied Social Sciences (A K Morgan MSc), Hong Kong Polytechnic University, Kowloon, China; Division of Plastic and

Reconstructive Surgery (S D Morrison MD), University of Washington Medical Center, Seattle, WA, USA; Faculty of Medicine (M Morsy MD), October 6 University, Giza, Egypt; Department of Stem Cells and Developmental Biology (S Mousavi MSc), Royan Institution, Tehran, Iran; Department of Physical and Environmental Sciences (S Mousavi Kiasary DVM-MPH), Texas A&M University, Corpus Christi, TX, USA; Arid Agriculture University Rawalpindi (R Mubarak MSc), PMAS Arid Agriculture University Rawalpindi, Rawalpindi, Pakistan; Unit of Pharmacotherapy, Epidemiology and Economics (Prof S Mubarik PhD), University of Groningen (Rijksuniversiteit Groningen), Groningen, Netherlands; Federal Institute for Population Research, Wiesbaden, Germany (Prof U O Mueller MD); Center for Population and Health, Wiesbaden, Germany (Prof U O Mueller MD); Knowledge Management Department (S Mukherjee PhD), Prahlad Omkarwati Foundation (POF), Mumbai, India; Changescape Consulting (S Mukherjee PhD), Independent Consultant, New Delhi, India (P Sood PhD); Department of Surgery (F Mulita PhD), General University Hospital of Patras, Patras, Greece; Faculty of Medicine (F Mulita PhD), University of Thessaly, Larissa, Greece; Department of Nursing (M Mulyadi PhD), Sam Ratulangi University, Manado, Indonesia; Department of Pathology and Microbiology (S I Mustafa PhD), Duhok University, Duhok, Iraq; Operational Research Center in Healthcare (M T Mustapha PhD), Near East University, Nicosia, Cyprus; Director General (Prof C M Muvunyi PhD), Rwanda Biomedical Centre, Kigali, Rwanda; College of Medicine and Health Sciences (Prof C M Muvunyi PhD), University of Rwanda, Kigali, Rwanda; Department of Psychiatry (W Myung PhD), Department of Food and Nutrition (A P Okekunle PhD), Seoul National University, Seoul, South Korea (Prof M Thiruvengadam PhD); Department of Neuropsychiatry (W Myung PhD), Seoul National University Bundang Hospital, Seongnam, South Korea; Elderly Health Research Center (A Nafei PhD), Research and Academic Institution, Tehran, Iran; Department of Computer Science (P Naghavi MS), University of Illinois, Urbana, IL, USA; Research Committee (A Naghibzadeh BSc), Kermanshah University of Medical Sciences, Kermanshah, Iran; Department of Computer Science and IT (G R Naik PhD), Torrens University, Adelaide, SA, Australia; Department Health Services Research (G Naik MPH), University of Alabama at Birmingham, Birmingham, AL, USA; Faculty of Pharmacy (Prof F Nainu PhD), Hasanuddin University, Makassar, Indonesia; Department of Community Medicine (T S Nair MD), MOSC Medical College, Kolenchery, India; College of Health Sciences (H H R Najmuldeen PhD), Cihan University Sulaimaniya, Sulaymaniyah, Iraq; Social and Behavioral Science Research (A Nandi PhD), Population Council, New York, NY, USA; Mysore Medical College and Research Institute (Prof S Narasimha Swamy MD), Government Medical College, Mysore, India; Department of Medicine (M Nassar PhD), University at Buffalo, Buffalo, NY, USA; Department of Internal Medicine (M Nassar PhD), Mount Sinai Health System, Queens, NY, USA; Department of Community Medicine (S N K Navaratna MD), University of Peradeniya, Kandy, Sri Lanka; Zilber College of Public Health (M Nayon MPH), University of Wisconsin Milwaukee, Milwaukee, WI, USA; Department of Health Promotion (A Nazri-Panjaki MSc), Zahedan University of Medical Sciences, Zahedan, Iran; Research and Innovation Center (P Ndishimye PhD), Dalhousie University, Kigali, Rwanda; African Institute for Mathematical Sciences, Kigali, Rwanda (P Ndishimye PhD); Department of General Surgery (I Negoi PhD), Emergency University Hospital of Bucharest, Bucharest, Romania; College of Health Sciences (Q Nguyen Khoi MD, T T Pham PhD), College of Health Sciences (CHS) (Prof D Poddighe PhD), VinUniversity, Hanoi, Viet Nam; Pediatric Department (N N Y Nhi MD), Pham Ngoc Thach University Of Medicine, Ho Chi Minh City, Viet Nam; International Islamic University Islamabad, Islamabad, Pakistan (R K Niazi PhD); Department of Humanities and Social Science (L Nieddu PhD), University for International Studies in Rome, Rome, Italy; Technical Department (C A Nnaji PhD), School of Public Health and Family Medicine (C A Nnaji PhD), Institute of Infectious Disease and Molecular Medicine (O G Oluwole PhD), University of Cape Town,

Cape Town, South Africa; Global Research Institute (Prof S Nomura PhD), Keio University, Tokyo, Japan; Internal Medicine Department (V C Nriagu MD), Maimonides Medical Center, Brooklyn, NY, USA; Global Health Department (J Nshimiyimana MPH), Euclid University, Banqui, Central African Republic; School of Information (F Nugen PhD), University of California Berkeley, Berkeley, CA, USA; Department of Public Health (M H Nunemo MPH), Wachemo University, Hossana, Ethiopia; Department of Applied Economics and Quantitative Analysis (Prof B Oancea PhD), University of Bucharest, Bucharest, Romania; Bioinformatics Department (Prof B Oancea PhD), National Institute of Research and Development for Biological Sciences, Bucharest, Romania; Disease Control and Elimination (M A Oboh PhD), Medical Research Council Unit, The Gambia, Banjul, The Gambia; Remote Researcher (R M Odat MD), University of Pittsburgh, PA, USA; PSSM Data Sciences, Pfizer Research & Development (M Oduro PhD), Pfizer Inc., Groton, CT, USA; Department of Animal Science (T E Ogundare MSc), North Carolina Agricultural and Technical State University, Greensboro, NC, USA; Technical Unit (O T Ogundeko-Olugbami MSc), Malaria Consortium, London, UK; Department of Physiotherapy (O O Ojedoyin PhD), The Redeemed Christian Church of God, Ede, Nigeria; Management Science and Healthcare Analytics (O A Okoli MS), University of Michigan, Ann Arbor, MI, USA; Department of Education Leadership and Management (J O Okunlola PhD), University of Johannesburg, Johannesburg, South Africa; College of Health Sciences (O I Olabisi PhD), Bowen University, Iwo, Iwo, Nigeria; Department of Medicine (A Olivas-Martinez MD), Department of Infectious Diseases (E Ortiz-Brizuela MD), Instituto Nacional de Nutrición Salvador Zubirán (Salvador Zubiran National Institute of Medical Sciences and Nutrition), Mexico City, Mexico; Cardiology Department (Prof G M M Oliveira PhD), Federal University of Rio de Janeiro, Rio de Janeiro, Brazil; Department of Public Health (S B Olorunju MSc), Association for Reproductive and Family Health (ARFH), Abuja, Nigeria; Department of Public Health (C Z Olorunsaiye PhD), Arcadia University, Glenside, PA, USA; Executive Director (B O Olusanya PhD), Centre for Healthy Start Initiative, Lagos, Nigeria; Institute of Chemistry (F B Omage PhD), Universidade Estadual de Campinas, Campinas, Brazil; Department of Computational Biology (F B Omage PhD), Brazilian Agricultural Research Institute (EMBRAPA), Campinas, SP, Brazil; Department of Pharmacology and Therapeutics (Prof O E Onwujekwe PhD), University of Nigeria Nsukka, Enugu, Nigeria; Department of Internal Medicine (C A Onyeaghala MD), University of Port Harcourt Teaching Hospital (UPTH), Port Harcourt, Nigeria; Institute of Diagnostic and Interventional Radiology and Neuroradiology (M Opitz MD), University Hospital Essen, Essen, Germany; Department of Pharmacotherapy and Pharmaceutical Care (M Ordak PhD), Department of Biochemistry and Pharmacogenomics (M Zielińska MPharm), Medical University of Warsaw, Warsaw, Poland; Sickle Cell Unit (Prof V N Orish PhD), Ho Teaching Hospital, Ho, Ghana; Department of Nephrology and Hypertension (Prof A Ortiz MD), IIS-Fundacion Jimenez Diaz, Madrid, Spain; Department of Medicine (Prof A Ortiz MD), Autonomous University of Madrid, Madrid, Spain; Unidad de Investigación de Salud en el Trabajo (E Ortiz-Brizuela MD), Instituto Mexicano del Seguro Social, Mexico City, Mexico; One Health Global Research Group (Prof E Ortiz-Prado PhD), Universidad de las Americas (University of the Americas), Quito, Ecuador; Department of Biological Sciences (A Osborne MSc), Njala University, Sierra Leone, Freetown, Sierra Leone; School of Public Health (O J Otorkpa PhD), Texila American University, Georgetown, Guyana; Division of Infectious Diseases (Prof A Ouyahia PhD), University Hospital of Setif, Sétif, Algeria; School of Medicine (T Oyelade PhD), Keele University, Keele, UK; Operational Research Center in Healthcare (I Ozsahin PhD, Prof U Saeed PhD), Near East University, Nicosia, Turkiye; Health Services Management Training Centre (T Palicz MD), Semmelweis University, Budapest, Hungary; Hungarian Health Management Association, Budapest, Hungary (T Palicz MD); Centre for Biotechnology (S K Panda PhD), Siksha 'O' Anusandhan (Deemed to be University),

Bhubaneswar, India; First Department of Ophthalmology (Prof G D Panos MD), Second Department of Cardiology (Prof D Patoulias PhD), Aristotle University of Thessaloniki, Thessaloniki, Greece; Department of Neurology (L D Panos MD), University of Bern, Biel/Biene, Switzerland; Department of Neurology (L D Panos MD), University of Cyprus, Nicosia, Cyprus; University of Padua, Padua, Italy (M Papa MD); Medical University of Vienna, Vienna, Austria (I Papadimopoulos MD); Vision and Eye Research Institute (Prof S Pardhan PhD), Anglia Ruskin University, Cambridge, UK; Department of Forensic Medicine and Toxicology (U Parekh MD), All India Institute of Medical Sciences, Rajkot, India; Department of Medical Sciences (R Passera PhD), University of Torino, Torino, Italy; Department of Imaging (R Passera PhD), AOU Città della Salute e della Scienza di Torino, Torino, Italy; Research and Development Cell (M Patel PhD), Parul University, Vadodara, India; Department of Cardiovascular Medicine (N N Patel MD), University of Tennessee, Nashville, TN, USA; College of Dental Medicine (Prof S Patil PhD), Roseman University of Health Sciences, South Jordan, UT, USA; Department of Interventional Cardiology (S Pawar MD), Cedars Sinai Medical Center, Los Angeles, CA, USA; Department of Public Health (J Pekarcikova PhD), Trnava University, Trnava, Slovakia; Center for Research and Innovation (V F Pepito MSc), Ateneo De Manila University, Pasig City, Philippines; School of Population Health (Prof G Pereira PhD), Curtin University, Bentley, WA, Australia; Centre for Fertility and Health (Prof G Pereira PhD), Department of Chemical Toxicology (M W Wojewodzic PhD), Norwegian Institute of Public Health, Oslo, Norway; Department of Food, Environmental and Nutritional Sciences (Prof S Perna PhD), Department of Biomedical Sciences for Health (S Villa MD), University of Milan, Milano, Italy; Department of Biochemistry and Pharmacology (P Petakh PhD), Uzhhorod National University, Uzhhorod, Ukraine; Research Advancement Consortium in Health, Hanoi, Viet Nam (T T Pham PhD); International Center of Medical Sciences Research (Z Z Piracha PhD), International Center of Medical Sciences Research, Islamabad, Pakistan; Department of Health Promotion, Mother and Child Care, Internal Medicine and Medical Specialties (PROMISE) (E Pirera MD), University of Palermo, Palermo, Italy; Department of Data Management and Analysis (R Poluru PhD), The INCLEN Trust International, New Delhi, India; Non-communicable Diseases Research Center (N Pourtaheri PhD), Bam University of Medical Sciences, Bam, Iran; Department of Economics (J Purohit PhD), Women's College, Jharsuguda, Jhasrsuguda, India; Department of Medical instrumentation Techniques Engineering (N H Qasim DSc), Al-Rafidain University College, Baghdad, Iraq; Department of Cybersecurity (N H Qasim DSc), Kyiv National University of Construction and Architecture, Kyiv, Ukraine; Rory Meyers College of Nursing (X Qi PhD), New York University, New York, NY, USA; School of Public Health (Prof Z Qi PhD), Xuzhou Medical University (徐州医科大学公共卫生学院), Xuzhou, China; Department of Cardiology (G Qian MS), Guiqian International General Hospital, Guiyang, China; Research Center for Public Health and Nutrition (B Rachmat MPH), National Research and Innovation Agency of Indonesia, Jakarta, Indonesia; Department of Medical Oncology (Prof V Radhakrishnan MD), Cancer Institute (W.I.A), Chennai, India; Osh State University, Osh, Kyrgyzstan (Prof F Rahim PhD); Department of Environmental Health Engineering, School of Health (S Rahimi PhD, M Sarmadi MSc), Health Science Research Centre (S Rahimi PhD), Health Sciences Research Center (M Sarmadi MSc), Torbat Heydariyeh University of Medical Sciences, Torbat Heydariyeh, Iran; Faculty of Health Sciences (F M Rahman PhD), Qaiwan International University, Sulaymaniyah, Iraq; National Institute of Infectious Diseases (M Rahman PhD), Center for Surveillance, Immunization, and Epidemiologic Research, Tokyo, Japan; Center for Evidence-Based Medicine and Clinical Research, Dhaka, Bangladesh (M Rahman PhD); Institute of Health and Wellbeing (Prof M Rahman PhD), Federation University Australia, Berwick, VIC, Australia; School of Nursing and Midwifery (Prof M Rahman PhD), La Trobe University, Melbourne, VIC, Australia; Dr. Rajendra Prasad Government Medical

College, Tanda, Kangra, India (Prof S K Raina MD); Department of Cardiology (A Raja MD), Dow University of Health Sciences, Karachi, Pakistan; Emergency Medicine Department (G Rajendran MD), Sri Manakula Vinayagar Medical College and Hospital, Puducherry, India; Department of Anatomy (C Ramasamy MD), Govt. Siddhartha Medical College, Vijayawada, India; Department of Radiology (S Ramasamy MD), Stanford University, Stanford, CA, USA; Independent Consultant, Las Vegas, NV, USA (K Ramphul MD); Community Medicine (K Rana MD), NKP Salve Institute of Medical Sciences and Research Centre, Nagpur, India; Department of Community Medicine (R K Rana MD), Shaheed Nirmal Mahto Medical College and Hospital, Dhanbad, India; Department of Research (C L Ranabhat PhD), Eastern Scientific LLC, Richmond, KY, USA; Planetary Health Research Centre (PHRC), Kathmandu, Nepal (C L Ranabhat PhD); Centre for Clinical Pharmacology (N Rancic PhD), University of Defence in Belgrade, Belgrade, Serbia; Centre for Clinical Pharmacology (N Rancic PhD), Medical College of Georgia at Augusta University, Belgrade, Serbia; Department of Forensic Medicine and Toxicology (S Rani MD), Jagadguru Sri Shivarathreeswara University, Mysore, India; Department of Oral Pathology, Microbiology and Forensic Odontology (S Rao MDS), Sharavathi Dental College and Hospital, Shimogga, India; Department of Pharmaceutical Chemistry (M Rashid PhD), University of Dhaka, Dhaka, Bangladesh; School of Pharmaceutical Sciences (M Rashid PhD), State University of Bangladesh, Dhaka, Bangladesh; Department of Geography (A Rasul PhD), Soran University, Soran, Iraq; Department of Family Medicine (Prof D Rathish PhD), Department of Community Medicine (N D Wickramasinghe MD), Rajarata University of Sri Lanka, Anuradhapura, Sri Lanka; University of Swabi (A Rauf PhD), University of Swabi, Swabi, Pakistan; Inovus Medical, St Helens, UK (D L Rawaf MD); Academic Public Health England (Prof S Rawaf MD), Public Health England, London, UK; Department of Biological Sciences (Prof E Redwan PhD), King Abdulaziz University, Jeddah, Egypt; Department of Protein Research (Prof E Redwan PhD), Research and Academic Institution, Alexandria, Egypt; The School of Pharmaceutical Sciences (W Rehman MS), University of Science Malaysia, Penang, Malaysia; Unisabana Center for Translational Science (L Reyes PhD), Universidad de La Sabana (Savannah University), Chia, Colombia; Critical Care Department (L Reyes PhD), Clinica Universidad De La Sabana (Savannah University Clinic), Chia, Colombia; School of Environment (M Rezaei PhD), Tehran University, Tehran, Iran; Department of Public Health (A Riad PhD), Czech National Centre for Evidence-based Healthcare and Knowledge Translation (A Riad PhD), Masaryk University, Brno, Czech Republic; Department of Physiology and Physiotherapy (Prof M R Rizvi PhD), DIT University, Delhi, India; Community Health Department (Prof H A L Rocha PhD), Federal University of Ceará, Fortaleza, Brazil; Department of Nursing in Women's Health (T Rodrigues da Silva PhD), Federal University of São Paulo, São Paulo, Brazil; Department of Clinical Research (Prof L Roever PhD), University of Sao Paulo, Ribeirão Preto, Brazil; Gilbert and Rose-Marie Chagoury School of Medicine (Prof L Roever PhD), Lebanese American University, Beirut, Lebanon; Department of Ophthalmology and Visual Sciences (A Roshanshad MD), University of Wisconsin-Madison, Madison, WI, USA; Department of Analytical and Applied Economics (Prof H Rout PhD, C Swain MPhil), RUSA Centre of Excellence in Public Policy and Governance (Prof H Rout PhD), UGC Centre of Advanced Study in Psychology (Prof M Satpathy PhD), Utkal University, Bhubaneswar, India; Achutha Menon Centre for Health Science Studies (A Roy MD), Sree Chitra Tirunal Institute for Medical Sciences and Technology, Thiruvananthapuram, India; Department of Biochemistry and Food Analysis (N Roy PhD), Patuakhali Science and Technology University, Patuakhali, Bangladesh; Department of Public Health (S Roy MD), New Mexico State University, Las Cruces, NM, USA; Research Department (S Roy MSc), Indian Institute of Public Health, Delhi, India; Advanced Campus Governador Valadares (Prof G D A Ruela MSc), Juiz de For a Federal University, Governador Valadares, Brazil; Department of Internal Medicine (G M

Rwegerera MD), University of Botswana, Gaborone, Botswana; Department of Medical Pharmacology (Prof M M Saber-Ayad PhD), Cairo University, Giza, Egypt; Department of Computer (T Sadegh MSc), University of Science and Culture, Tehran, Iran; Research Center for Evidence-Based Medicine (F Sadeghi-Ghyassi PhD), Drug Applied Research Center (H Samadi Kafil PhD), Tabriz University of Medical Sciences, Tabriz, Iran; International Center of Medical Sciences Research (ICMSR), Islamabad, Pakistan (Prof U Saeed PhD); Department of Psychosocial Science (Prof D Sagoe PhD), University of Bergen, Bergen, Norway; College of Pharmacy (Prof S Sajadi PhD), Al-Hadba University, Mosul, Iraq, Mosul, Iraq; Department of Health and Kinesiology (M Sajib BDS), University of Illinois, Urbana-Champaign, IL, USA; Department of Statistics (M R Sajid PhD), University of Gujrat, Gujrat, Pakistan; Faculty of Dentistry (A A Salami BDS), University of Puthisastra, Phnom Penh, Cambodia; Student Research Committee (M Salehi MD), Kashan University of Medical Sciences, Kashan, Iran; College of Nursing (D Salihu PhD), Jouf University, Jouf, Saudi Arabia; Institute of Epidemiology and Preventive Medicine (Y L Samodra PhD), National Taiwan University, Taipei, Taiwan; Benang Merah Research Center (BMRC), Minahasa Utara, Indonesia (Y L Samodra PhD); Department of Osteopathic Medicine (Prof A Sanyaolu PhD), D'Youville University, Buffalo, NY, USA; Department of Health, Physical Education and Recreation (J O Sarfo PhD), University of Cape Coast, Cape Coast, Ghana; Bodoland University (H Sarma PhD), Botany Department, Kokrajhar, India; Faculty of Health & Social Sciences (B Sathian PhD), Bournemouth University, Bournemouth, UK; Udyam-Global Association for Sustainable Development, Bhubaneswar, India (Prof M Satpathy PhD); Precision Medicine Department (M Savabi Far MD, S - Tajabadi MSc), Università degli studi della Campania Luigi Vanvitelli, Naples, Italy; Department of Public Health Sciences (M Sawhney PhD), University of North Carolina at Charlotte, Charlotte, NC, USA; Department of Preventive and Social Medicine (G Saya MD), Jawaharlal Institute of Postgraduate Medical Education and Research, Puducherry, India; Faculty of Business and Computing (Prof C Schinckus PhD), University of the Fraser Valley, Abbotsford, BC, Canada; Department of Finance (Prof C Schinckus PhD), International School of Management, Paris, France; Department of Physiotherapy (I J C Schneider PhD), Federal University of Santa Catarina, Araranguá, Brazil; Faculty of Medicine (A Schuermans BSc), Department of Cardiovascular Sciences (A Schuermans BSc, J Van den Eynde BSc), Katholieke Universiteit Leuven, Leuven, Belgium; School of Pharmacy (A K Sendekie MSc), Curtin University, Perth, WA, Australia; Emergency Department (S Senthilkumaran PhD), Manian Medical Centre, Erode, India; Fourth Department of General Surgery (D Serban PhD), Emergency University Hospital Bucharest, Bucharest, Romania; Department of Medicine (Y Sethi MD), Swami Vivekanand Subharti University, Meerut, India; Dongguan Key Laboratory of Computer-Aided Drug Design (M Shahab PhD), Key Laboratory of Computer-Aided Drug Design (M Waqas PhD), Guangdong Medical University, Dongguan, China; State Key Laboratories of Chemical Resources Engineering (M Shahab PhD), Beijing University Of Chemical Technology, Beijing, China; Department of Biotechnology (S A Shahid MPhil), Quaid-i-Azam University Islamabad, Islamabad, Pakistan; Faculty of Medicine (F Shahrahmani MD), Mashhad University of Medical Sciences, Mashhad, Iran; Institute for Critical Care Medicine (A Shaikh MD), Mount Sinai Health System, New York, NY, USA; Independent Consultant, Karachi, Pakistan (M A Shaikh MD); Department of Pathobiology (M Shamshirgaran PhD), Shahid Bahonar University of Kerman, Kerman, Iran; National University of Ireland - Galway, Galway, Ireland (D Shan MD); Department for Evidence-based Medicine and Evaluation (A Sharifan PharmD), University for Continuing Education Krems, Krems, Austria; Department of Biotechnology (B Sharma PhD), Graphic Era (Deemed to be University), Dehradun, India; Department of Microbiology (S M Shenoy MD), Kasturba Medical College, Mangalore, India; Department of Biology (S P Sherchan PhD), Morgan State University, Baltimore, MD, USA; Department of Public

Health (M Shimul MPH), Department of Development Studies (M Sohel MPH), Daffodil International University, Dhaka, Bangladesh; Department of Medical-Surgical Nursing (S Shorofi PhD), Mazandaran University of Medical Sciences, Sari, Iran; Kenneth H. Cooper Institute (Prof K Shuval PhD), Texas Tech University Health Sciences Center, Dallas, TX, USA; Department of Medical Microbiology and Infectious Diseases (E E Siddig MD), Erasmus University, Rotterdam, Netherlands; Quaid-e-Azam Medical College (A Siddiqua MD), University of Health Sciences, Bahawalpur, Pakistan; Sport Physical Activity and Health Research & Innovation Center (SPRINT) (Prof L M L R Silva PhD), Polytechnic Institute of Guarda, Guarda, Portugal; CICS-UBI Health Sciences Research Center (Prof L M L R Silva PhD), University of Beira Interior, Covilhã, Portugal; Institute of National Importance on Food Technology (Prof B P Singh PhD), National Institute of Food Technology Entrepreneurship and Management, Sonipat, India; Department of Pharmacology (H Singh DM), Government Medical College and Hospital, Chandigarh, India; School of Medicine (Prof J A Singh MD), Baylor College of Medicine, Houston, TX, USA; Department of Medicine Service (Prof J A Singh MD), US Department of Veterans Affairs (VA), Houston, TX, USA; Global and European Health Education and Study Institute (Prof N Skhvitaridze PhD), University of Georgia, Tbilisi, Georgia; NCDC (Prof N Skhvitaridze PhD), National Center for Disease Control and Public Health, Tbilisi, Georgia; Books Committee (V Y Skryabin MD), Royal College of Psychiatrists, London, UK; Royal college of psychiatrists, London, UK (V Y Skryabin MD); Clinical Science Line (Prof A Sokhan PhD), Ludwig Boltzmann Institute of Osteologie, Vienna, Austria; Faculty of Pharmacy (A M Soliman PhD), Kafrelsheikh University, Kafr El-Sheikh, Egypt; Faculty of Science (A M Soliman PhD), University of Regina, Regina, SK, Canada; School of Medicine (S Soraneh MD), Babol University of Medical Sciences, Babol, Iran; Department of Public Health (M Sorrentino MD), University of Naples "Federico II", Naples, Italy; Department of Public Health, Experimental and Forensic Medicine (M Sorrentino MD), University of Pavia, Pavia, Italy; 3rd Department of Cardiology (M Spartalis PhD), University of Athens, Athens, Greece; Department of Public Health and Community Medicine (Prof C T Sreeramareddy MD), International Medical University, Kuala Lumpur, Malaysia; Department of Neurology (B S Srichawla MD), University of Massachusetts Medical School, Worcester, MA, USA; Department of Clinical Research (V Srinivasalu MBBS), National Institute for Research in Tuberculosis, Chennai, India; JIPMER International School of Public Health (M Srinivasan MD), Jawaharlal Institute of Postgraduate Medical Education and Research, Pondicherry, India; Department of Orthopaedics (O Subasi PhD), Harvard University, Cambridge, MA, USA; Department of Physiotherapy (S K Sulaiman PhD), Tishk International University, Erbil, Iraq; Department of Human Anatomy (M O Suleiman Odidi PhD), Federal University, Dutse, Dutse, Nigeria; School of Life Sciences (M Suleman PhD), Xiamen University, Xiamen, China; Department of Social Sciences (Prof M J M Sullman PhD), Department of Life and Health Sciences (Prof M J M Sullman PhD), University of Nicosia, Nicosia, Cyprus; School of Medicine, Medical Sciences and Nutrition (A Sultan Meo MPH), University of Aberdeen, Aberdeen, UK; Department of Biomedical Sciences (Z Sun PhD), Universiti Putra Malaysia, Selangor, Malaysia; Squad Medicine and Research (SMR) (T Suvvari MD), Indian Council of Medical Research, Amadalavalasa, India; Department of Clinical Research and Development (Prof L Szarpak PhD), LUXMED Group, Warsaw, Poland; Collegium Medicum (Prof L Szarpak PhD), John Paul II Catholic University of Lublin, Lublin, Poland; Department of Medicine (Prof R Tabarés-Seisdedos PhD), University of Valencia, Valencia, Spain; Department of Basic Medical Sciences (S Tabatabaeizadeh PhD), Department of Internal Medicine (S Tabatabaeizadeh PhD), Islamic Azad University, Mashhad, Iran; Department of Health, Safety, and Environmental Management (R Tabibi PhD), Abadan School of Medical Sciences, Abadan, Iran; Division of Epidemiology (T Tabuchi MD), Tohoku University, Sendai, Japan; Department of Dermato-Venereology (M Tampa PhD), Dr. Victor

Babes Clinical Hospital of Infectious Diseases and Tropical Diseases, Bucharest, Romania; Department of Medicine (J L Tamuzi MSc), Northlands Medical Group, Omuthiya, Namibia; Department of Pharmacology and Therapeutics (S Tariq PhD), The University of Faisalabad, Faisalabad, Pakistan; Department of Public Health and Informatics (A Tasnim MPH), Bangladesh Medical University, Dhaka, Bangladesh; Taking Our Best Shot, Houston, TX, USA (N Y Tat MS); Department of Research and Innovation (N Y Tat MS), Enventure Medical Innovation, Houston, TX, USA; Department of Pathology (V Y Tat BS), University of Texas, Galveston, TX, USA; Department of Public Health (Y M Tefera MPH), Dire Dawa University, Dire Dawa, Ethiopia; Department of Health Informatics (G H Teferi MPH), Debre Markos University, Debre Markos, Ethiopia; Public Health Department (Prof K R Thankappan MD), Amrita Institute of Medical Sciences, Kochi, India; Faculty of Public Health (J H V Ticoalu MPH), Universitas Sam Ratulangi (Sam Ratulangi University), Manado, Indonesia; Department of Biochemistry and Molecular Biology (M B Tincho PhD), University of Buea, Buea, Cameroon; High Institute of Sport and Physical Education of Sfax (K Trabelsi PhD), University of Sfax, Sfax, Tunisia; Second Department of Internal Medicine (Q T H Tran MD), Kansai Medical University, Osaka, Japan; John T. Milliken Department of Medicine (T Q M Tran MSc), Washington University in St. Louis, Saint Louis, MO, USA; Department of Business Analytics (T H Tran MD), University of Massachusetts Dartmouth, Dartmouth, MA, USA; Molecular Neuroscience Research Center (N Tran Minh Duc MD), Shiga University of Medical Science, Shiga, Japan; Department of Studies in Economics and Planning (T Tripathi PhD), Central University of Gujarat, Gandhinagar, India; Department of Health Sciences (S J Tromans PhD), University of Leicester, Leicester, UK; Adult Learning Disability Service (S J Tromans PhD), Leicestershire Partnership National Health Service Trust, Leicester, UK; CRIMEDIM Center for Research and Training in Global Health, Humanitarian Aid and Disaster Medicine (C Truppa MD), University of Eastern Piedmont, Novara, Italy; Department of Primary Care (C Truppa MD), Geneva University Hospital, Geneva, Switzerland; School of Pharmacy (D Tsai MSc), Population Health Data Center (D Tsai MSc), National Cheng Kung University, Tainan, Taiwan; Department of Medicine (Prof A Tsatsakis DSc), University of Crete, Heraklion, Greece; Department of Comprehensive Nursing (A T Tsedalu Amare MSc), Debre Tabor University, Debre Tabor, Ethiopia; Department of Internal Medicine (M Tumurkhuu PhD), Wake Forest University, Winston-Salem, NC, USA; Institute of Clinical and Preventive Medicine (Prof L Tzivian PhD), University of Latvia, Riga, Latvia; Federal University of Health Sciences Azare (L Umar PhD), Federal Teaching Hospital, Azare, Nigeria; Federal Teaching Hospital Azare (L Umar PhD), Federal Medical Centre, Azare, Nigeria; Department of Cardiovascular, Endocrine-metabolic Diseases and Aging (B Unim PhD), National Institute of Health, Rome, Italy; Amity Institute of Biotechnology (E Upadhyay PhD), Amity University Rajasthan, Jaipur, India; Begum Rokeya University (J Urmey BS), Begum Rokeya University, Rangpur, Bangladesh; Department of Orthodontics (H Uzunçıbuk PhD), University of Trakya, Edirne, Turkiye; Community Respiratory Centre (N Uzzaman PhD), Bangladesh Primary Care Respiratory Society (BPCRS), Khulna, Bangladesh; Centre for Population Health Sciences (N Uzzaman PhD), University of Edinburgh, Edinburgh, UK; Johnson & Johnson (P Vadagam MS), Duquesne University, Pittsburgh, PA, USA; College of Health and Sport Sciences (A G Vaithinathan MSc), University of Bahrain, Zallaq, Bahrain; UKK Institute, Tampere, Finland (Prof T J Vasankari PhD); Faculty of Medicine and Health Technology (Prof T J Vasankari PhD), Tampere University, Tampere, Finland; Department of Otolaryngology Head and Neck Surgery (S Vasudevan MS), Louisiana State University Health Sciences Center, Shreveport, LA, USA; Department of Physiotherapy (J H Villafañe PhD), Universidad Europea de Madrid (European University of Madrid), Villaviciosa de Odón, Spain; Saint Camillus International University of Health Sciences - UniCamillus, Rome, Italy (Prof L Villani MD); Occupational Medicine Unit (Prof F S Violante

MD), Sant'Orsola Malpighi Hospital, Bologna, Italy; Department of Bioengineering, School of Chemical and Biotechnology (S Visaga Ambi PhD), SASTRA Deemed to be University, Thanjavur, India; NUST School of Health Sciences (Prof Y Waheed PhD), National University of Sciences and Technology (NUST), Islamabad, Pakistan; Széchenyi István University, Gyor, Hungary (Prof Y Waheed PhD); Department of Forensic Science (M Walia MPhil), Shree Guru Gobind Singh Tricentenary University, Gurugram, India; Brigham and Women's Hospital, Boston, MA, USA (C Wang PhD); College of Agriculture (X Wang PhD), Northwest A&F University, Xianyang City, China; Department of Biotechnology and Genetic Engineering (M Waqas PhD), Hazara University Mansehra, Mansehra, Pakistan; Kings College London, Department of Women and Children's Health (J L Ward PhD), King's College London, London, UK; College of Medicine and Health Science (Y A Wassie MSc), Ethiopian Academy of Medical Science, Gondar, Ethiopia; Institute of Health and Wellbeing (I Weerasekara PhD), Federation University, Melbourne, VIC, Australia; University of Newcastle, Newcastle, NSW, Australia (I Weerasekara PhD); Department of Nursing (A Wilandika PhD), Universitas Aisyiyah Bandung, Bandung, Indonesia; Institute of Clinical Epidemiology (Prof P Willeit PhD), Medical University Innsbruck, Innsbruck, Austria; Department of Research (M W Wojewodzic PhD), Cancer Registry of Norway, Oslo, Norway; Abuakwa North Municipal Health Directorate (F G Wongnaah MSc), Ghana Health Service, Kukurantumi, Ghana; Department of Food Science and Human Nutrition (Prof F Wu PhD), Michigan State University, East Lansing, MI, USA; Tongji Medical College (G Xiao MD), Huazhong University of Science and Technology, Wuhan, China; Department of Intelligent Medical Engineering (Prof W Xie DrPH), Anhui Medical University, Anhui, China; Department of Surgery (Prof W Xie DrPH), The First Affiliated Hospital of Anhui Medical University, Hefei, Anhui, China; Ruijin Hospital (S Xu MPH), Shanghai Jiao Tong University, Shanghai, China; School of Medicine (M Xue MSc), Kunming University of Science and Technology, Kunming, China; Department of Basic Medical Sciences (S Yaghoubi PhD), Neyshabur University of Medical Sciences, Neyshabur, Iran; Department of Community Medicine (S Yahoo (Syed) MD), Apollo Institute of Medical Sciences and Research, hyderabad, India; Department of Cells and Tissues (G Yahya PhD), Molecular Biology Institute of Barcelona, Barcelona, Spain; Department of Hepatobiliary Surgery (H YANG PhD), Capital Medical University, Beijing, China; Department of Biostatistics and Data Science (Y Yasufuku MSc), The University of Osaka, Suita, Japan; School of Biotechnology (M Yeganeh PhD), University of Tehran, Tehran, Iran; Family Medicine Department (S A Yesuf MSc), St. Peter's Specialized Hospital, Addis Ababa, Ethiopia; Biostatics, Epidemiology, and Science Computing Department (S Yezli PhD), King Faisal Specialist Hospital & Research Center, Riyadh, Saudi Arabia; Pharmacy Department (Y E Yismaw MSc), Alkan Health Science, Business and Technology College, Bahir Dar, Ethiopia; Department of Pediatrics (Prof D Yon MD), Kyung Hee University, Seoul, South Korea; Department of Biostatistics (Prof N Yonemoto PhD), University of Toyama, Toyama, Japan; Department of Public Health (Prof N Yonemoto PhD), Juntendo University, Tokyo, Japan; Department of Nursing Science (U Yunusa PhD), Bayero University, Nigeria, Kano, Nigeria; Faculty of Nursing (U Yunusa PhD), University of Alberta, Edmonton, AB, Nigeria; Islamic Azad University, Tehran, Iran (M Zaghampour MD); Faculty of Medicine and Health Sciences (F Zakham PhD), Pharmacy Practice Department (M Zawiah PhD), Hodeidah University, Hodeidah, Yemen; Department of Administration (Prof M Zastrozhin PhD), PGxAI, San Francisco, CA, USA; College of Pharmacy, Department of Clinical Practice (M Zawiah PhD), Northern Border University, Rafha, Saudi Arabia; Department of Pediatrics and Child Health Nursing (A B Zemariam MSc), Woldia University, Woldia, Ethiopia; School of Public Health (C J P Zhang PhD), University of Hong Kong, Hong Kong, China; XuZhou Medical University (Prof J Zhang DrPH), University of Medicine, XuZhou, China; School of Public Health and Emergency Management (B Zhu PhD), Southern University of Science and

Technology, Shenzhen, China; Institute of Public Health and Social Sciences (H Zia BDS), Khyber Medical University, Peshawar, Pakistan; Endocrinology and Metabolism Research Center (G Zoghi MD), Hormozgan University of Medical Sciences, Bandar Abbas, Iran; Clinical Research Centre (Prof S H Zyoud PhD), An-Najah National University Hospital, Nablus, Palestine; Department of Building Engineering and Environment (S H Zyoud PhD), Civil Engineering and Sustainable Structures (S H Zyoud PhD), Palestine Technical University (Kadoorie), Tulkarem, Palestine; GBD Collaborating Unit (Prof S E Vollset DrPH), Norwegian Institute of Public Health, Bergen, Norway.

Authors' Contributions

Managing the overall research enterprise

Emily Haeuser, Ashley A Harris, Amanda E Smith, Paulina A Lindstedt, Olivia D Nesbit, Stein Emil Vollset, Simon I Hay, Stephen S Lim, Jonathan F Mosser

Writing the first draft of the manuscript

Emily Haeuser, Sam Byrne, Jason Nguyen, Susan A McLaughlin, Catherine Bisignano, Taylor Noyes, Noga Shalev, Latera Tesfaye Olana, Stephen S Lim, Jonathan F Mosser

Primary responsibility for applying analytical methods to produce estimates

Emily Haeuser, Sam Byrne, Catalina Raggi, Georgia Smith, Samuel James Herold

Primary responsibility for seeking, cataloguing, extracting, or cleaning data; designing or coding figures and tables

Emily Haeuser, Sam Byrne, Jason Nguyen

Providing data or critical feedback on data sources

Mohammad Amin Aalipour, Hedayat Abbastabar, Samar Abd ElHafeez, Wakgari Mosisa Abdisa, Auwal Abdullahi, Armita Abedi, Roberto Ariel Abeldaño Zuñiga, Rahim Abo Kasem, Richard Gyan Aboagye, Hassan Abolhassani, Abdullahi Tunde Aborode, Lucas Guimarães Abreu, Hana J Abukhadijah, Salahdein Aburuz, Meshack Achore, Juan Manuel Acuna, Ousman Adal, Tajudeen Adesanmi Adebisi, Kamoru Ademola Adedokun, Oluwatobi Emmanuel/E Adegbile, Olumide Thomas Adeleke, Mohd Adnan, Qorinah Estiningtyas Sakilah Adnani, Leticia Akua Adzigbli, Saira Afzal, Constanza Elizabeth Aguilera Arriagada, Danish Ahmad, Rabbiya Ahmad, Tauseef Ahmad, Ayman Ahmed, Haroon Ahmed, Muktar Beshir Ahmed, Naveed Ahmed, Syed Anees Ahmed, Simeon Okechukwu Ajakwe, Oluwasefunmi Akeju, Roland Eghoghosoa Akhigbe, Hammad Akram, Alaa Al Amiry, Salah Al Awaidy, Hanadi Al Hamad, Mohammad Khaled Al nawayseh, Omar Al Omari, Yazan Al Thaher, Omar Ali Mohammed Al Zaabi, Rasmieh Mustafa Al-amer, Turki M Alanzi, Mohammed Albashtawy, Tekletsadik Tekleslassie Alemayehu, Abdelazeem M Algammal, Ashraf Alhumaidi, Abid Ali, Liaqat Ali, Shahid Ali, Syed Shujait Ali, Montaha Al-Iede, Sheikh Mohammad Alif, Hamid Alinejad Rokny, Samah W Al-Jabi, Mustafa Alkhawam, Mohammed Z. Allouh, Sabah Al-Marwani, Joseph Uy Almazan, Amr Almobayed, Hasan Yaser Alniss, Jaber S Alqahtani, Mohammed A Alsabri, Alaa B. Al-Tammemi, Nelson Alvis-Guzman, Nelson J Alvis-Zakzuk, Hassan Alwafi, Mohammad Al-Wardat, Hany Aly, Abdallah Alzoubi, Amr Amin, Saeed Amini, Nafiu Aminu, Dickson A Amugsi, Michael Anderson, Song Peng Ang, Nguyen Hoang Anh, Abdul-Azeez Adeyemi Anjorin, Hossein

Ansariniya, Saeid Anvari, Saleha Anwar, Jalal Arabloo, Jesil Mathew Aranjani, Olatunde Aremu, Jesu Arockiaraj, Anton A Artamonov, Deepavalli Arumuganainar, Nurila Aryntayeva, Mahsa Asadi Anar, Shewatatek Melaku Melaku Asefa, Tahira Ashraf, Mitra Ashrafi, Muhammad Shahzad Aslam, Yuni Asri, Alok Atreya, Khursheed Aurangzeb, Babafela B Awosile, Mohd Yusmaidie Aziz, Ahmed Y. Azzam, Atif Amin Baig, Senthilkumar Balakrishnan, Shahid Bashir, Mohammad-Mahdi Bastan, Narasimha M Beeraka, Melesse Belayneh, Samiun Nazrin Bente Kamal Tune, Robert S Bernstein, Akshaya Srikanth Bhagavathula, Dinesh Bhandari, Sonu Bhaskar, Priyadarshini Bhattacharjee, Jasvinder Singh Bhatti, Saeed Biroudian, Bijit Biswas, Monirujjaman Biswas, Molalegne Bitew, Lucimere Bohn, Obasanjo Afolabi Bolarinwa, Souad Bouaoud, Dejana Braithwaite, Danilo Buonsenso, Felix Busch, Nadeem Shafique Butt, Sam Byrne, Mehtap Çakmak Barsbay, Joao Mauricio Castaldelli-Maia, Carlos A Castañeda-Orjuela, Francieli Cembranel, Vijay Kumar Chattu, Haowei Chen, Nicholas WS Chew, William C S Cho, Bryan Chong, Hitesh Chopra, Shivani Chopra, Dinh-Toi Chu, Sunghyun Chung, Alyssa Columbus, Natalia Cruz-Martins, Xiaochen Dai, Lalit Dandona, Rakhi Dandona, Lucio D'Anna, Samuel Demissie Darcho, Aso Mohammad Darwesh, Fernando Pio De la Hoz, Sindhura Deekonda, Aniket Dehadrai, Pradeep Kumar Devarakonda, Meghnath Dhimal, Diana Dias da Silva, Xueting Ding, Thanh Chi Do, Wendel Mombaque dos Santos, Ojas Prakashbhai Doshi, Robert Kokou Dowou, Senbagam Duraisamy, Mohammad Hossein ---- Ebrahimi, Ashkan Eighaei Sedeh, Ebrahim Eini, Michael Ekholuenetale, Rabie Adel El Arab, Maysaa El Sayed Zaki, Aya Elalfy, Abdelgawad Salah Abdelgawad Eltahawy, Talha Bin Emran, Setegn Eshetie, Gilbert Eshun, Majid Eslami, Fahima Nasrin Eva, Adewale Oluwaseun Fadaka, Heidar Fadavian, Adeniyi Francis Fagbamigbe, Ayesha Fahim, Ildar Ravisovich Fakhradiyev, Alireza Feizkhah, Ginenus Fekadu, Ulrich Membe Femoe Membe, Takeshi Fukumoto, Muktar A Gadanya, Balasankar Ganesan, Fernando Barroga Garcia, Anteneh Gashaw, Nsikakabasi Samuel George, Gebremariam Wulie Geremew, Gebremariam Getaneh, Kazem Ghaffari, Arin Ghamkhar, Shakiba Ghasemi Assl, Gloria Gheno, Arun Ghuge, Laszlo Göbölös, Mahaveer Golechha, Aman Goyal, Shi-Yang Guan, Amit Gulati, Ishita Gupta, Sapna Gupta, Veer Bala Gupta, Vivek Kumar Gupta, Emily Haeuser, Pritam Halder, Islam M Hamad, Nadia M Hamdy, Harapan Harapan, Josep Maria Haro, Muhammad Hassan, Simon I Hay, Wen-Qiang He, Golnaz Heidari, Minoo Heidari Almasi, Alamgir Hossain, Md Mahbub Hossain, Md. Jubayer Hossain, Mehdi Hosseinzadeh, Priya Hotwani, Chengxi Hu, Nawfal R Hussein, Mohamed Ibrahim Husseiny, Hong-Han Huynh, Segun Emmanuel Ibitoye, NUHEILA IBRAHIM, Olayinka Stephen Ilesanmi, Benni Iskandar, Teresa R. Iskander, Md Sahidul Islam, Sheikh Mohammed Shariful Islam, Haitham Jahrami, Ammar Abdulrahman Jairoun, Mihajlo Jakovljevic, Mohamed Jalloh, Armaan Jamal, Syed Sarmad Javaid, Qassim Jawell Odah Abed, Shubha Jayaram, Wenyi Jin, Jobin Jose, Charity Ehimwenma Joshua, Jacek Jerzy Jozwiak, Zubair Kabir, Vidya Kadashetti, Dler H. Hussein Kadir, Ashish Kumar Kakkar, Ramat T. Kamorudeen, Oleksandr Kamyshnyi, Rami S Kantar, André Karch, Mohmed Isaqali Karobari, Tomasz M. Karpiński, Manoj Kumar Kashyap, Himanshu Khajuria, Mohammad Ali Khaksar, Nauman Khalid, Anees Ahmed Khalil, Faham Khamesipour, Abdul Arif Khan, Ajmal Khan, Maseer Khan, Moien AB Khan, Muhammad Umer Khan, Ramsha Mushtaq Khan, Ubaid Khan, Zahid Khan, Sameer Uttamaro Khasbage, Khaled Khatab, Farbod Khosravi, Grace Kim, Jinho Kim, Min Seo Kim, Yohannes Kinfu, Adnan Kisa, Sezer Kisa, Shivakumar KM, Michail Kokkorakis, Oleksii Korzh, Irene Akwo Kretchy, James-Paul Kretchy, Kewal Krishan, Barthelemy Kuate Defo, Raja Amir Hassan Kuchay, Dewesh Kumar, G Anil Kumar, Jogender Kumar, Kamal Kumar, Satyajit Kundu, Maria Dyah Kurniasari, Asep Kusnali, Dian Kusuma, Ville Kytö, Dr Pallavi L C, Muhammad Awwal Ladan, Chandrakant Lahariya, Savita Lasrado, Huu-Hoai Le, Minh Huu Nhat Le, Nhi Huu Hanh Le, Trang Diep Thanh Le, Caterina Ledda, Sergey Vadimovich Lee, Seung Won Lee, Wei-Chen Lee, Virendra S Ligade, Stephen S Lim, Queran Lin, Gang Liu, Jue Liu, Xuefeng Liu, Zhe Liu,

Erand Llanaj, Abhilash Ludhiadch, Peng Luo, Kaung Suu Lwin, Miltiadis D. Lytras, Ellina Lytvyak, Kevin Sheng-Kai Ma, Zheng Feei Ma, Shamsuddeen Yusuf Yusuf Ma'aruf, Monika Machoy, Seyed Ataollah Madinezad, Aurea Marilia Madureira-Carvalho, Sasikumar Mahalingam, Samatar Abshir Mahamed, Nozad H. Mahmood, Ahmad Azam Malik, Shahid Malik, Deborah Carvalho Malta, Lokesh Manjani, Mohammad Ali Mansournia, Michael Marks-Hultström, Francisco Rogerlândio Martins-Melo, Roy Rillera Marzo, Sammer Marzouk, Stefano Masi, Clara N Matei, Medha Mathur, Chioma Ngozichukwu Pauline Mbachu, Ikechukwu Innocent Mbachu, Vini Mehta, Tesfahun Mekene Meto, Endalkachew Belayneh Melese, Ziad Ahmed Memish, Walter Mendoza, Godfred Antony Menezes, Ritesh G Menezes, Leweyehu Alemaw Mengstie, Atte Meretoja, Tomislav Mestrovic, Sachith Mettananda, Wai-kit Ming, Vinaytosh Mishra, Heba M. Mohamed, Mona Gamal Mohamed, Nouh Saad Mohamed, Khabab Abbasher Hussien Abbasher Hussien Mohamed Ahmed, Shafiu Mohammed, Yahaya Mohammed, Ali H Mokdad, Lorenzo Monasta, Mohammad Ali Moni, Maryam Moradi, Yousef Moradi, Mahmoud M Morsy, Jonathan F Mosser, Rabia Mubarak, Sumaira Mubarik, Ulrich Otto Mueller, Sumoni Mukherjee, Francesk Mulita, Christopher J L Murray, Saravanan Muthupandian, Muhammad Muzaffar, Ayoub Nafei, Ganesh R Naik, Arindam Nandi, Sreenivas Narasimha Swamy, Shumaila Nargus, Mahmoud Nassar, Zuhair S Natto, Zakira Naureen, Samidi Nirasha Kumari Navaratna, Biswa Prakash Nayak, Md Fahad Shahariar Nayon, Ionut Negoi, Henok Biresaw Netsere, Kieu Viet Nhi Nguyen, Nhan Nguyen, Nhien Ngoc Y Nguyen, Quan Nguyen Khoi, Nguyen Ngoc Yen Nhi, Robina Khan Niazi, Luciano Nieddu, Chukwudi A Nnaji, Shuhei Nomura, Syed Toukir Ahmed Noor, Masoud Noroozi, Fred Nugen, Bogdan Oancea, Ismail A Odetokun, Michael Safo Oduro, John Olayemi Okunlola, Bolajoko Olubukunola Olusanya, Folorunsho Bright Omage, Obinna E Onwujekwe, Marcel Opitz, Atakan Orscelik, Alberto Ortiz, Eric Osei, Amel Ouyahia, Mayowa O Owolabi, Oyetunde T Oyeyemi, Ilker Ozsahin, Jagadish Rao Padubidri, Sujogya Kumar Panda, Shahina Pardhan, Romil R Parikh, Chulwoo Park, Mitesh Patel, Neel Navinkumar Patel, Shankargouda Patil, Shrikant Pawar, Shubhadarshini Pawar, Prince Peprah, Gavin Pereira, Simone Perna, Nhat Truong Pham, Tung Thanh Pham, Zahra Zahid Piracha, Ramesh Poluru, Sajjad Pourasghary, Naeimeh Pourtaheri, Jagadeesh Puvvula, Husam Qanash, Asma Saleem Qazi, Xiang Qi, Navid Rabiee, Venkatraman Radhakrishnan, Sajjad Rahimi, Vafa Rahimi-Movaghar, Md. Obaidur Rahman, Jeffrey Pradeep Raj, Adarsh Raja, Mohammad Amin Rajizadeh, Siddheesh Rajpurohit, Mahmoud Mohammed Ramadan, Chitra Ramasamy, Shakthi Kumaran Ramasamy, Chhabi Lal Ranabhat, Nemanja Rancic, Smitha Rani, Chythra R Rao, Sowmya J Rao, Santosh Kumar Rauniyar, David Laith Rawaf, Salman Rawaf, Elrashdy Redwan, Wajiha Rehman, Luis Felipe Reyes, Mina Rezaei, Moattar Raza Rizvi, Hannah Elizabeth Robinson-Oden, Hermano Alexandre Lima Rocha, Leonardo Roever, Amirhossein Roshanshad, Shiva Rouzbahani, Sharmistha Roy, Shubhanjali Roy, Godfrey M Rwegerera, Aly M A Saad, Seyed Kiarash Sadat Rafiei, Basema Ahmad Saddik, Tarannom Sadegh, Mohd Saeed, Umar Saeed, Mehdi Safari, Mastooreh Sagharichi, Narjes Saheb Sharif-Askari, S. Mohammad Sajadi, Mirza Rizwan Sajid, Morteza Saki, Afeez Abolarinwa Salami, Mahdi Salehi, Malik Sallam, Hossein Samadi Kafil, Nahom Samuel Samuel, Abdallah M Samy, Sathish Sankar, Hemen Sarma, Mohammad Sarmadi, Brijesh Sathian, Maheswar Satpathy, Christophe Schinckus, Ione Jayce Ceola Schneider, Mohammad H Semreen, Yigit Can Senol, Subramanian Senthilkumaran, Yashendra Sethi, Samiah Shahid, Syed Ahsan Ahsan Shahid, Ahmed Shaikh, Masood Ali Shaikh, Mehran Shams-Beyranvand, Anas Shamsi, Dan Shan, Mohammed Shannawaz, Amin Sharifan, Vishal Sharma, Ramzi Shawahna, Maryam Shayan, Samendra P Sherchan, Shiran Shetty, Md. Monir Hossain Shimul, Aminu Shittu, Zahra Shokati Eshkiki, Luís Manuel Lopes Rodrigues Silva, Baljinder Singh, Harmanjit Singh, Jasvinder A Singh, Kalpana Singh, Samer Singh, Georgia Smith, Ahmed M. Soliman, May Mohamed Sherif Soliman, Noha Salah Soliman, Aayushi Sood, Soroush Soraneh, Michael Spartalis,

Chandrashekhar T Sreeramareddy, Devin Bailey Srivastava, Andy Stergachis, Muritala Odidi Suleiman Odidi, Muhammad Suleman, Anusha Sultan Meo, Thanigaivel Sundaram, David Sunkersing, Tarun Kumar Suvvari, Chandan Kumar Swain, Lukasz Szarpak, Rafael Tabarés-Seisdedos, Takahiro Tabuchi, Lidia S. Seifu Tadesse, Mircea Tampa, Ker-Kan Tan, Anika Tasnim, Birhan Tsegaw Taye, Yibekal Manaye Tefera, Mohamad-Hani Temsah, Jay Tewari, Marcos Roberto Tovani-Palone, Quynh Thuy Huong Tran, Tam Quoc Minh Tran, Daniel Hsiang-Te Tsai, Munkhtuya Tumurkhuu, Lawan Umar, Era Upadhyay, Jef Van den Eynde, Joe Varghese, Tommi Juhani Vasankari, Srivatsa Surya Vasudevan, Yasir Waheed, Megha Walia, Yilkal Abebaw Wassie, Angga Wilandika, Peter Willeit, Yohannes Chemere Wondmeneh, Haileyesus Gedamu Wondyifraw, Florence Gyembuzie Wongnaah, Minichil Chanie Chanie Worku, Felicia Wu, Mingyang Xue, Sajad Yaghoubi, Mohamed A Yassin, Sanni Yaya, Naohiro Yonemoto, Chuanhua Yu, Michael Zastrozhin, Alemu Birara Birara Zemariam, Anthony Zhong, Abzal Zhumagaliuly, Magdalena Zielińska, Rafat Mohammad Zrieq, Ahed H. Zyoud, Shaher H. Zyoud

Developing methods or computational machinery

Aleksandr Y Aravkin, Sam Byrne, Xiaochen Dai, Emily Haeuser, Simon I Hay, Jiawei He, Samuel James Herold, Stephen S Lim, Ali H Mokdad, Jonathan F Mosser, Christopher J L Murray, Catalina Raggi, Georgia Smith, Reed J D Sorensen, Stein Emil Vollset, Chun-Wei Yuan

Providing critical feedback on methods or results

Mohammad Amin Aalipour, Mitra Abbasifard, Faezeh Abbaspour, Hedayat Abbastabar, Samar Abd ElHafeez, Emad M. Abdallah, Reda Abdel-Hameed, Sherief Abd-Elsalam, Wakgari Mosisa Abdisa, Meriem Abdoun, Arman Abdous, Deldar Morad Abdulah, Adam Abdullahi, Auwal Abdullahi, Toufik Abdul-Rahman, Kulmira Abdykerimova, Armita Abedi, Asrat Agalu Abejew, Roberto Ariel Abeldaño Zuñiga, Syed Hani Abidi, Rahim Abo Kasem, Richard Gyan Aboagye, Hassan Abolhassani, Ulric Sena Abonie, Abdullahi Tunde Aborode, Nagah Mohamed Abourashed, Mohamed Abouzid, Dmitry Abramov, Lucas Guimarães Abreu, Dariush Abtahi, Rana Kamal Abu Farha, Bilyaminu Abubakar, Eman Abu-Gharbieh, Hana J Abukhadijah, Salahdein Aburuz, Anirudh Balakrishna Acharya, Meshack Achore, Juan Manuel Acuna, Ousman Adal, Lisa C. Adams, Abdu A Adamu, Tajudeen Adesanmi Adebisi, David Adedia, Kamoru Ademola Adedokun, Oluwatobi Emmanuel/E Adegbile, Oyelola A Adegboye, Nurudeen A Adegoke, Olumide Thomas Adeleke, Mache Tsadik Adhana, Ripon Kumar Adhikary, Mohd Adnan, Qorinah Estiningtyas Sakilah Adnani, Prince Owusu Adoma, Leticia Akua Adzigbli, Giuseppina Affinito, Aanuoluwapo Adeyimika Afolabi, Rotimi Felix Afolabi, Saira Afzal, Suneth Buddhika Agampodi, Dhiraj Motilal Agarwal, Sepehr Aghajanian, Williams Agyemang-Duah, Mahsa Ahadi, Aqeel Ahmad, Danish Ahmad, Khurshid Ahmad, Rabbiya Ahmad, Shoaib Ahmad, Tauseef Ahmad, Ayman Ahmed, Haroon Ahmed, Meqdad Saleh Ahmed, Muktar Beshir Ahmed, Naveed Ahmed, Simeon Okechukwu Ajakwe, Dolapo Emmanuel Ajala, Gizachew Taddesse Akalu, Oluwasefunmi Akeju, Roland Eghoghosoa Akhigbe, Karolina Akinosoglou, Hammad Akram, Ashley E Akrami, Salah Al Awaidy, Hanadi Al Hamad, Mohammad Khaled Al nawayseh, Omar Al Omari, Yazan Al Thaher, Omar Ali Mohammed Al Zaabi, Mohammad Ahmmad Mahmoud Al Zoubi, Yazan Al-Ajlouni, Ziyad Al-Aly, Khurshid Alam, Mohammad Khursheed Alam, Nazmul Alam, Rasmieh Mustafa Al-amer, Turki M Alanzi, Jude Oluwapelumi Alao, Fahmi Y. Al-Ashwal, Mohammed Albashtawy, Mohammad T AlBataineh, Abdulelah Mastour Aldhahir, Shereen M. Aleidi, Tekletsadik Tekleslassie Alemayehu, Ali M Alfalki, Fahad D Algahtani, Abdelazeem M Algammal,

Ashraf Alhumaidi, Abid Ali, Irfan Ali, Liaqat Ali, Mohammad Daud Ali, Rafat Ali, Shahid Ali, Syed Shujait Ali, Montaha Al-Iede, Sheikh Mohammad Alif, Hamid Alinejad Rokny, Morteza Alipour, Samah W Al-Jabi, Adel Al-Jumaily, Mustafa Alkhawam, Mohammed Z. Allouh, Wesam Taher Almagharbeh, Sabah Al-Marwani, Joseph Uy Almazan, Hesham M Al-Mekhlafi, Amr Almobayed, Hasan Yaser Alniss, Mohammad R. Alosta, Jaber S Alqahtani, Mohammad R. Alqudimat, Ahmed Yaseen Alqutaibi, Rami H Al-Rifai, Intima Alrimawi, Mohammed A Alsabri, Zaid Altaany, Alaa B. Al-Tammemi, Jaffar A Al-Tawfiq, Malik A Althobiani, Khalid A Altirkawi, Nelson Alvis-Guzman, Nelson J Alvis-Zakzuk, Hassan Alwafi, Mohammad Al-Wardat, Yaser Mohammed Al-Worafi, Hany Aly, Mohammad Sharif Ibrahim Alyahya, Abdallah Alzoubi, Karem H Alzoubi, Md. Akib Al-Zubayer, Ekiyor Joseph Amafah, Amr Amin, Saeed Amini, Nafiu Aminu, Ayodeji Amobonye, Dickson A Amugsi, Filippos Anagnostakis, Michael Anderson, Song Peng Ang, Nguyen Hoang Anh, Abdul-Azeez Adeyemi Anjorin, Hossein Ansariniya, Boluwatife Stephen Anuoluwa, Saeid Anvari, Saleha Anwar, Jalal Arabloo, Jesil Mathew Aranjani, Demelash Areda, Olatunde Aremu, Jesu Arockiaraj, Mahwish Arooj, Anton A Artamonov, Ashokan Arumugam, Deepavalli Arumuganainar, Mahsa Asadi Anar, Muhammad Asaduzzaman, Syed Mohammed Basheeruddin Asdaq, Shewatatek Melaku Melaku Asefa, Akram Ashames, Tahira Ashraf, Mitra Ashrafi, Bernard Kwadwo Yeboah Asiamah-Asare, Muhammad Shahzad Aslam, Saeed Aslani, Yuni Asri, Dereje Zewdu Assefa, Batyrbek Assembekov, Sachin R Atre, Alok Atreya, Julie Alaere Atta, Matteo Augello, Khursheed Aurangzeb, Andargie Abate Awoke, Babafela B Awosile, Seyyed HamidReza Ayatizadeh, Yusuf Oloruntoyin Ayipo, Sina Azadnajafabad, Mohd Yusmaidie Aziz, Sadat Abdulla Aziz, Amin Azizan, Ahmed Y. Azzam, Rasha Babiker, Sara Bagheri, Fereshteh Baghizadeh, Razieh Bahreini, Yogesh Bahurupi, Atif Amin Baig, Senthilkumar Balakrishnan, Rajon Banik, Hansi Bansal, Hiba Jawdat Barqawi, Zarrin Basharat, Shahid Bashir, Rehana Basri, Quique Bassat, Mohammad-Mahdi Bastan, Saurav Basu, Kavita Batra, Ravi Batra, Mahdis Bayat, Narasimha M Beeraka, Bezawit K Bekele, Tariku Tesfaye Bekuma, Sewunet admasu Belachew, Asnake Gashaw Belayneh, Melesse Belayneh, Umar Muhammad Bello, Samiun Nazrin Bente Kamal Tune, Abiye Assefa Berihun, Amiel Nazer C Bermudez, Robert S Bernstein, Ajeet Singh Bhadoria, Akshaya Srikanth Bhagavathula, Neeraj Bhala, Dinesh Bhandari, Ashish Bhargava, Sonu Bhaskar, Priyadarshini Bhattacharjee, Krittika Bhattacharyya, Ashmin Hari Bhattarai, Jasvinder Singh Bhatti, Can Bilgin, Saeed Biroudian, Bijit Biswas, Mohammad Shahangir Biswas, Monirujjaman Biswas, Molalegne Bitew, Bruno Bizzozero-Peroni, Firew Tekle Bobo, Trupti Bodhare, Lucimere Bohn, Obasanjo Afolabi Bolarinwa, Paria Bolourinejad, Alejandro Botero Carvajal, Souad Bouaoud, Dejana Braithwaite, Hermann Brenner, Felix Busch, Yasser Bustanji, Nadeem Shafique Butt, Zahid A Butt, Sam Byrne, Mehtap Çakmak Barsbay, Luis Alberto Cámera, Angelo Capodici, Joao Mauricio Castaldelli-Maia, Luca Cegolon, Francieli Cembranel, Muthia Cenderadewi, Muge Cevik, Chiranjib Chakraborty, Rama Mohan Chandika, Vijay Kumar Chattu, Galmesa Bekana Chemeda, An-Tian Chen, Hana Chen, Haowei Chen, Nicholas WS Chew, William C S Cho, Bryan Chong, Hitesh Chopra, Shivani Chopra, Dinh-Toi Chu, Sheng-Chia Chung, Sunghyun Chung, Alyssa Columbus, Joao Conde, Alexandru Corlateanu, Claudia Cosma, Natalia Cruz-Martins, Alanna Gomes da Silva, Bashir Dabo, Omid Dadras, Xiaochen Dai, Lalit Dandona, Rakhi Dandona, Lucio D'Anna, Samuel Demissie Darcho, Latefa Ali Dardas, Gary L Darmstadt, Aso Mohammad Darwesh, Dimash Davletov, Fernando Pio De la Hoz, Sindhura Deekonda, Aniket Dehadrai, Tadesse Asmamaw Dejenie, Mohammad Delsoz, Huiyin Deng, Anteneh Assefa Desalegn, Pradeep Kumar Devarakonda, Arkadeep Dhali, Kuldeep Dhama, Meghnath Dhimal, Bibha Dhungel, Stefano Di Bella, Marcello Di Pumpo, Diana Dias da Silva, Daniel Diaz, Xueting Ding, Thanh Chi Do, Fariba Dorostkar, Wendel Mombaque dos Santos, Ojas Prakashbhai Doshi, Robert Kokou Dowou, Senbagam Duraisamy, Oyewole Christopher Durojaiye, Sulagna Dutta, Osamudiamen Ebohon, Lamiaa Labieb Mahmoud Ebraheim, Mohammad Hossein ---

Ebrahimi, Abdelaziz Ed-Dra, Ferry Efendi, Ashkan Eighaei Sedeh, Ebrahim Eini, Michael Ekholuenetale, Rabie Adel El Arab, Maysaa El Sayed Zaki, Mohamed Ahmed Eladl, Aya Elalfy, Said El-Ashker, Noha Mousaad Elemam, Muhammed Elhadi, Mohamed Hassan Elnaem, Mohammed Elshaer, Abdelgawad Salah Abdelgawad Eltahawy, Theophilus I Emeto, Talha Bin Emran, Misganu Endriyas, Setegn Eshetie, Gilbert Eshun, Sharareh Eskandarieh, Majid Eslami, Maysa Eslami, Fahima Nasrin Eva, Adewale Oluwaseun Fadaka, Heidar Fadavian, Adeniyi Francis Fagbamigbe, Ayesha Fahim, Razana Faiz, Ildar Ravisovich Fakhradiyev, Niloofar Faraji, Seyed Nooreddin Faraji, Mahsa Faramarzpour, Mohammad Fareed, Andre Faro, Syed Muhammad Yousaf Farooq, Emmanuel Toluwani Fasusi, Zareen Fatima, Pooria Fazeli, Alireza Feizkhah, Ginenus Fekadu, Ulrich Membe Femoe Membe, Rodrigo Fernandez-Jimenez, Natan Feter, Claudio Fiorilla, Florian Fischer, Takeshi Fukumoto, Nancy Fullman, Muktar A Gadanya, Márió Gajdács, Balasankar Ganesan, Xiang Gao, Bashiru Garba, Fernando Barroga Garcia, Jacopo Garlasco, Anteneh Gashaw, Zisis Gatzioufas, Rupesh K Gautam, Feven Sahle Gebre, Miglas Welay Gebregergis, Nsikakabasi Samuel George, Gebremariam Wulie Geremew, Genanew K Getahun, Habtamu Abebe Getahun, Fekadeselassie Belege Getaneh, Gebremariam Getaneh, Kazem Ghaffari, Roya Ghafoury, Arin Ghamkhar, Shakiba Ghasemi Assl, Haniyeh Ghasrsaz, Ramy Mohamed Ghazy, Gloria Gheno, Nermin Ghith, Arun Ghuge, Alessandro Girombelli, Laszlo Göbölös, Amit Goel, Mahaveer Golechha, Davide Golinelli, MReza Goodarzian, Aman Goyal, Shi-Yang Guan, Stefano Guicciardi, Amit Gulati, Sasidhar Gunturu, Anish Kumar Gupta, Ishita Gupta, Sapna Gupta, Veer Bala Gupta, Vivek Kumar Gupta, Reyna Alma Gutiérrez, Roberth Steven Gutiérrez-Murillo, Jose Guzman-Esquivel, Annie Haakenstad, Awoke Derbie Habteyohannes, Emily Haeuser, Dariush Haghmorad, Haimanot Ewnetu Hailu, Pritam Halder, Islam M Hamad, Nadia M Hamdy, Samer Hamidi, Asif Hanif, Nasrin Hanifi, Harapan Harapan, Ahmed I Hasaballah, Mohammad Jahid Hasan, Hamidreza Hasani, Mohammad Hashem Hashempur, Ammarah Hasnain, Ibrahim Nagmeldin Hassan, Md. Imtaiyaz Hassan, Muhammad Hassan, Simon I Hay, Khezar Hayat, Wen-Qiang He, Golnaz Heidari, Mohammad Heidari, Minoo Heidari Almasi, Sumudu Avanthi Hewage, Majid Heydari, Kamal Hezam, Yuta Hiraike, Alamgir Hossain, Lubna Hossain, Md Mahbub Hossain, Md Sabbir Hossain, Md. Jubayer Hossain, Mehdi Hosseinzadeh, Md Munnaf Hossen, Mihaela Hostiuc, Priya Hotwani, Hanno Hoven, Chengxi Hu, Kiavash Hushmandi, Javid Hussain, M. Azhar Hussain, Nawfal R Hussein, Mohamed Ibrahim Husseiny, Hong-Han Huynh, Bing-Fang Hwang, Segun Emmanuel Ibitoye, NUHEILA IBRAHIM, Olayinka Stephen Ilesanmi, Irena M Ilic, Milena D Ilic, Mohammad Tarique Imam, Arit Inok, Mustafa Alhaji Isa, Benni Iskandar, Teresa R. Iskander, Md Sahidul Islam, Md. Fakrul Islam, Sheikh Mohammed Shariful Islam, Leila Ismail, Mosimah Charles Ituka, Masao Iwagami, Chinwe Juliana Iwu-Jaja, Louis Jacob, Haitham Jahrami, Ayushi Jain, Ammar Abdulrahman Jairoun, Mihajlo Jakovljevic, Mohamed Jalloh, Armaan Jamal, Melika Jameie, Jerin James, Hasan Jamil, Roland Dominic G Jamora, Syed Sarmad Javaid, Talha Jawaid, Qassim Jawell Odah Abed, Shubha Jayaram, Seogsong Jeong, Ravi Prakash Jha, Wenyi Jin, Mohammad Jokar, Jobin Jose, Jobinse Jose, Nitin Joseph, Charity Ehimwenma Joshua, Kripa Josten, Farahnaz Joukar, Jacek Jerzy Jozwiak, Zubair Kabir, Vidya Kadashetti, Dler H. Hussein Kadir, Ashish Kumar Kakkar, Md Moustafa Kamal, Mehnaz Kamal, Rajesh Kamath, Ramat T. Kamorudeen, Naser Kamyari, Oleksandr Kamyshnyi, Mona Kanaan, Saddam Fuad Kanaan, Jiseung Kang, Samuel Berchi Kankam, Kehinde Kazeem Kanmodi, Suthanthira Kannan S, Rami S Kantar, Jafar Karami, Ibraheem M Karaye, André Karch, Mohmed Isaqali Karobari, Tomasz M. Karpiński, Manoj Kumar Kashyap, Himanshu Khajuria, Mohammad Ali Khaksar, Nauman Khalid, Anees Ahmed Khalil, Faham Khamesipour, Abdul Arif Khan, Ajmal Khan, Faiz Ullah Khan, Maseer Khan, Md Abdullah Saeed Khan, Moien AB Khan, Muhammad Umer Khan, Ramsha Mushtaq Khan, Sumaiya Khan Khan, Ubaid Khan, Yusuf Saleem Khan, Zahid Khan, Vishnu Khanal, Sameer Uttamaro Khasbage, Khaled

Khatab, Haitham Khatatbeh, Moawiah Mohammad Khatatbeh, Afshin Khazaei, Khalid A Kheirallah, Farbod Khosravi, Grace Kim, Jinho Kim, Kwanghyun Kim, Min Seo Kim, Ruth W Kimokoti, Yohannes Kinfu, Adnan Kisa, Sezer Kisa, Shivakumar KM, Sonali Kochhar, Michail Kokkorakis, Ali-Asghar Kolahi, Farzad Kompani, Oleksii Korzh, Irene Akwo Kretchy, James-Paul Kretchy, Kewal Krishan, Barthelemy Kuate Defo, Mohammed Kuddus, Ilari Kuitunen, Emmanuel Kumah, Dewesh Kumar, G Anil Kumar, Jogender Kumar, Kamal Kumar, Narendar Kumar, Satyajit Kundu, Setor K Kunutsor, Maria Dyah Kurniasari, Pramod Kumar Kushawaha, Asep Kusnali, Dian Kusuma, Assylkhan Kuttybayev, Wai Hang Patrick Kwong, Frank Kyei-Arthur, Ville Kytö, Dr Pallavi L C, Carlo La Vecchia, Muhammad Awwal Ladan, Chandrakant Lahariya, Iván Landires, Savita Lasrado, Colleen L Lau, Huu-Hoai Le, Minh Huu Nhat Le, Nhi Huu Hanh Le, Trang Diep Thanh Le, Caterina Ledda, Sergey Vadimovich Lee, Seung Won Lee, Wei-Chen Lee, Awol Yemane Legesse, Elvynna Leong, Ming-Chieh Li, Peng Li, Wei Li, Virendra S Ligade, Stephen S Lim, Jialing Lin, John C Lin, Queran Lin, Gang Liu, Haipeng Liu, Jue Liu, Patrick Y Liu, Xuefeng Liu, Zhe Liu, Erand Llanaj, José Francisco López-Gil, Giancarlo Lucchetti, Abhilash Ludhiadch, Peng Luo, Angelina M Lutambi, Lei Lv, Kaung Suu Lwin, Miltiadis D. Lytras, Ellina Lytvyak, Ahmed M. Afifi, Kevin Sheng-Kai Ma, Zheng Feei Ma, Shamsuddeen Yusuf Yusuf Ma'aruf, Mahmoud Mabrok, Monika Machoy, Firoozeh Madadi, Farzan Madadizadeh, Seyed Ataollah Madinezad, Aurea Marilia Madureira-Carvalho, Sasikumar Mahalingam, Samatar Abshir Mahamed, Nozad H. Mahmood, Farhad Mahmoudi, Hardeep Singh Malhotra, Ahmad Azam Malik, Shahid Malik, Tabarak Malik, Deborah Carvalho Malta, Biniyam Tedla Tedla Mamo, Lokesh Manjani, Farheen Mansoor, Mohammad Ali Mansournia, Shaista Manzoor, Tahir Maqbool, Hamid Reza Marateb, Konstantinos Margetis, Michael Marks-Hultström, Adolfo Martinez-Valle, Francisco Rogerlândio Martins-Melo, Miquel Martorell, Roy Rillera Marzo, Sammer Marzouk, Stefano Masi, Clara N Matei, Yasith Mathangasinghe, Medha Mathur, Neeta Mathur, Fernanda Penido Matozinhos, Richard James Maude, Chioma Ngozichukwu Pauline Mbachu, Ikechukwu Innocent Mbachu, Steven M McPhail, Rishi P Mediratta, Vini Mehta, Subhash Mehto, James Meiring, Tesfahun Mekene Meto, Tesfaye Hambisa Mekonnen, Hadush Negash Meles, Endalkachew Belayneh Melese, Ziad Ahmed Memish, Walter Mendoza, Godfred Antony Menezes, Ritesh G Menezes, Emiru Ayalew Mengistie, Leweyehu Alemaw Mengstie, Alexios-Fotios A Mentis, Sultan Ayoub Ayoub Meo, Atte Meretoja, Tomislav Mestrovic, Sachith Mettananda, Mohamed M.M. Metwally, Irmina Maria Michalek, Giuseppe Minervini, Wai-kit Ming, Andreea Mirica, Alireza Mirkheshti, Vinaytosh Mishra, Heba M. Mohamed, Jama Mohamed, Mona Gamal Mohamed, Nouh Saad Mohamed, Khabab Abbasher Hussien Abbasher Hussien Mohamed Ahmed, Taj Mohammad, Abdolreza Mohammadi, Shafiu Mohammed, Yahaya Mohammed, Syam Mohan, Mohammad Mohseni, Amin Mohsenzadeh, Ali H Mokdad, Peyman Mokhtarzadehazar, Mohammad Ali Moni, Maryam Moradi, Yousef Moradi, Paula Moraga, Anthony Kwame Morgan, Mahmoud M Morsy, Jonathan F Mosser, Seyed Ahmad Mousavi, Seyed Mohamad Sadegh Mousavi Kiasary, Hagar Mowafy, Kimia Mozahheb Yousefi, Rabia Mubarak, Sumaira Mubarik, Ulrich Otto Mueller, Sumoni Mukherjee, Francesk Mulita, Mulyadi Mulyadi, Kavita Munjal, Anjana Munshi, Christopher J L Murray, Fungai Musaigwa, Sherzad Ibrahim Mustafa, Mubarak Taiwo Mustapha, Saravanan Muthupandian, Claude Mambo Mambo Muvunyi, Muhammad Muzaffar, Woojae Myung, Pirouz Naghavi, Amirhossein Naghibzadeh, Mobin Naghshbandi, Ganesh R Naik, Gurudatta Naik, Firzan Nainu, Tapas Sadasivan Nair, Soroush Najdaghi, Hastyar Hama Rashid Najmuldeen, Arindam Nandi, Sreenivas Narasimha Swamy, Shumaila Nargus, Abdulqadir J Nashwan, Mahmoud Nassar, Zuhair S Natto, Zakira Naureen, Samidi Nirasha Kumari Navaratna, Biswa Prakash Nayak, Shalini Ganesh Ganesh Nayak, Md Fahad Shahariar Nayon, Athare Nazri-Panjaki, Pacifique Ndishimye, Ionut Negoi, Henok Biresaw Netsere, Kieu Viet Nhi Nguyen, Nhan Nguyen, Nhien Ngoc Y Nguyen, Quan Nguyen Khoi, Nguyen Ngoc Yen Nhi,

Robina Khan Niazi, Luciano Nieddu, Afewerki Tesfahunegn Tesfahunegn Nigusse, Ali Nikoobar, Behnaz Niroomand, Chukwudi A Nnaji, Shuhei Nomura, Syed Toukir Ahmed Noor, Masoud Noroozi, Taylor Noyes, Valentine C. Nriagu, Chisom Adaobi Nri-Ezedi, Jean Claude Nshimiyimana, Fred Nugen, Mengistu H Nunemo, Felix Kwasi Nyande, Bogdan Oancea, Ramez M. Odat, Ismail A Odetokun, Michael Safo Oduro, Tunde Emmanuel Ogundare, Oluwafunmilayo Tosin Ogundeko-Olugbami, Olusegun Olatunji Ojedoyin, Akinkunmi Paul Okekunle, Onyedika A Okoli, Osaretin Christabel Okonji, John Olayemi Okunlola, Oluyemi Adewole Okunlola, Oluwaseyi Isaiah Olabisi, Latera Tesfaye Olana, Antonio Olivas-Martinez, Abdulhakeem Abayomi Olorukooba, Samson Bamidele Olorunju, Comfort Z. Z Olorunsaiye, Bolajoko Olubukunola Olusanya, Oluwafemi G. Gabriel Oluwole, Folorunsho Bright Omage, Obinna E Onwujekwe, Chizaram A Onyeaghala, Marcel Opitz, Michal Ordak, Atakan Orscelik, Edgar Ortiz-Brizuela, Eric Osei, Elham H. Othman, Oche Joseph Otorkpa, Amel Ouyahia, Mayowa O Owolabi, Kolapo Oyebola, Tope Oyelade, Oyetunde T Oyeyemi, Ilker Ozsahin, Jagadish Rao Padubidri, Tamás Palicz, Sujogya Kumar Panda, Georgios D Panos, Leonidas D. D Panos, Mario Virgilio Papa, Ilias Papadimopoulos, Shahina Pardhan, Utsav Parekh, Romil R Parikh, Chulwoo Park, Roberto Passera, Mitesh Patel, Neel Navinkumar Patel, Shankargouda Patil, Dimitrios Patoulias, Shrikant Pawar, Shubhadarshini Pawar, Jarmila Pekarcikova, Prince Peprah, Gavin Pereira, Gladymar Perez Chacon, Simone Perna, Pavlo Petakh, Olumuyiwa James Peter, Nhat Truong Pham, Tung Thanh Pham, Zahra Zahid Piracha, Roman V Polibin, Ramesh Poluru, Sajjad Pourasghary, Reza Pourbabaki, Farzad Pourghazi, Naeimeh Pourtaheri, Ashwathi Prakash, Elton Junio Sady Prates, Jagadeesh Puvvula, Husam Qanash, Nameer Hashim Qasim, Asma Saleem Qazi, Xiang Qi, Zhipeng Qi, Gangzhen Qian, Navid Rabiee, Basuki Rachmat, Venkatraman Radhakrishnan, Fakher Rahim, Sajjad Rahimi, Vafa Rahimi-Movaghar, Fryad Majeed Rahman, Md. Mosfequr Rahman, Md. Obaidur Rahman, Mosiur Rahman, Muhammad Aziz Rahman, Saeed Rahmani, Hakim Rahmoune, Sunil Kumar Raina, Jeffrey Pradeep Raj, Adarsh Raja, Judah Rajendran, Mohammad Amin Rajizadeh, Siddheesh Rajpurohit, Mahmoud Mohammed Ramadan, Chitra Ramasamy, Shakthi Kumaran Ramasamy, Kirtan Rana, Rishabh Kumar Rana, Nemanja Rancic, Smitha Rani, Chythra R Rao, Sowmya J Rao, Md. Abdur Rashid, Mohammad-Mahdi Rashidi, Azad Rasul, Devarajan Rathish, Abdur Rauf, Santosh Kumar Rauniyar, David Laith Rawaf, Salman Rawaf, Elrashdy Redwan, Wajiha Rehman, Luis Felipe Reyes, Mina Rezaei, Nazila Rezaei, Mohsen Rezaeian, Abanoub Riad, Moattar Raza Rizvi, Hermano Alexandre Lima Rocha, Thales Philipe Rodrigues da Silva, Leonardo Roever, Amirhossein Roshanshad, Himanshu Sekhar Rout, Shiva Rouzbahani, Adrija Roy, Sharmistha Roy, Shubhanjali Roy, Godfrey M Rwegerera, Aly M A Saad, Maha Mohamed Saber-Ayad, Seyed Kiarash Sadat Rafiei, Basema Ahmad Saddik, Tarannom Sadegh, Fatemeh Sadeghi-Ghyassi, Mohd Saeed, Umar Saeed, Mehdi Safari, Mastooreh Sagharichi, Dominic Sagoe, Narjes Saheb Sharif-Askari, S. Mohammad Sajadi, Md Refat Uz Zaman Sajib, Mirza Rizwan Sajid, Morteza Saki, Dorsa Salabat, Afeez Abolarinwa Salami, Mohamed A Saleh, Mahdi Salehi, Sohrab Salimi, Pegah Salimi Pormehr, Malik Sallam, Saad Samargandy, Yoseph Leonardo Samodra, Nahom Samuel Samuel, Abdallah M Samy, Sathish Sankar, Jacob Owusu Sarfo, Hemen Sarma, Mohammad Sarmadi, Sachin C Sarode, Brijesh Sathian, Maheswar Satpathy, Mehrdad Savabi Far, Ganesh Kumar Saya, Christophe Schinckus, Ione Jayce Ceola Schneider, Art Schuermans, Amin Sedigh, Mohammad H Semreen, Sabyasachi Senapati, Ashenafi Kibret Sendekie, Pallav Sengupta, Yigit Can Senol, Subramanian Senthilkumaran, Dragos Serban, Yashendra Sethi, Seyed mohammad Seyed alshohadaei, Abubakar Sha'aban, Samiah Shahid, Syed Ahsan Ahsan Shahid, Wajeehah Shahid, Farshad Shahkarami, Fatemeh Shahrahmani, Masood Ali Shaikh, Nafhat Shaikh, Mehran Shams-Beyranvand, Mohammad Ali Shamshirgaran, Anas Shamsi, Alfiya Shamsutdinova, Dan Shan, Mohammed Shannawaz, Amin Sharifan, Bunty Sharma, Vishal Sharma, Ramzi Shawahna, Maryam Shayan, Suchitra M

Shenoy, Samendra P Sherchan, Shiran Shetty, Md. Monir Hossain Shimul, Aminu Shittu, Sina Shool, Seyed Afshin Shorofi, Kerem Shuval, Zahra Siavashpour, Emmanuel Edwar Siddig, Ayesha Siddiqua, Gustavo Correia Basto da Silva, Luís Manuel Lopes Rodrigues Silva, Amit Singh, Baljinder Singh, Harmanjit Singh, Jasvinder A Singh, Kalpana Singh, Samer Singh, Satwinder Singh, Mukesh Kumar Sinha, Ebrahim Abdela Siraj, Natia Skhvitaridze, Valentin Yurievich Skryabin, Amanda E Smith, Md.Salman Sohel, Ahmed M. Soliman, May Mohamed Sherif Soliman, Noha Salah Soliman, Sameh S M Soliman, Weiyi Song, Aayushi Sood, Prashant Sood, Soroush Soraneh, Reed J D Sorensen, Michele Sorrentino, Michael Spartalis, Manraj Singh Sra, Chandrashekhar T Sreeramareddy, Vignes Anand Srinivasalu, Manikandan Srinivasan, Devin Bailey Srivastava, Andy Stergachis, Omer Subasi, Muritala Odidi Suleiman Odidi, Muhammad Suleman, Mark J M Sullman, Anusha Sultan Meo, Zhong Sun, Thanigaivel Sundaram, David Sunkersing, Tarun Kumar Suvvari, Chandan Kumar Swain, Lukasz Szarpak, Rafael Tabarés-Seisdedos, Celine Tabche, Ramin Tabibi, Takahiro Tabuchi, Lidia S. Seifu Tadesse, Farzad Taghizadeh-Hesary, Moslem Taheri Soodejani, Shima - Tajabadi, Iman M Talaat, Mircea Tampa, Jacques Lukenze Tamuzi, Ker-Kan Tan, Saba Tariq, Anika Tasnim, Birhan Tsegaw Taye, Yibekal Manaye Tefera, Gizaw Hailiye Teferi, Wegayehu Zeneb Teklehaimanot, Mohamad-Hani Temsah, Jay Tewari, Kavumpurathu Raman Thankappan, Jansje Henny Vera Ticoalu, Marius Belmondo Tincho, Marcos Roberto Tovani-Palone, Khaled Trabelsi, Quynh Thuy Huong Tran, Tam Quoc Minh Tran, Nguyen Tran Minh Duc, Indang Trihandini, Tulika Tripathi, Samuel Joseph Tromans, Daniel Hsiang-Te Tsai, Abraham Tsedalu Tsedalu Amare, Munkhtuya Tumurkhuu, Biruk Shalmeno Tusa, Lilian Tzivian, Atta Ullah, Riaz Ullah, Saeed Ullah, Lawan Umar, Muhammad Umar, Era Upadhyay, Jeba Mahiad Urmey, Jibrin Sammani Usman, Hande Uzunçıbuk, Nazim Uzzaman, Jef Van den Eynde, Joe Varghese, Srivatsa Surya Vasudevan, Simone Villa, Jorge Hugo Villafañe, Manish Vinayak, Francesco S Violante, Senthil Visaga Ambi, Stein Emil Vollset, Yasir Waheed, Megha Walia, Cong Wang, Qingzhi Wang, Ruixuan Wang, Wei Wang, Xing Wang, Ahmed Bilal Waqar, Muhammad Waqas, Joseph L Ward, Yilkal Abebaw Wassie, Ishanka Weerasekara, Nuwan Darshana Darshana Wickramasinghe, Angga Wilandika, Peter Willeit, Yohannes Chemere Wondmeneh, Haileyesus Gedamu Wondyifraw, Florence Gyembuzie Wongnaah, Minichil Chanie Chanie Worku, Felicia Wu, James Fan Wu, Qing Xia, Guangqin Xiao, Lishun Xiao, Wanqing Xie, Site Xu, Mingyang Xue, Mukesh Kumar Yadav, Saba Yahoo (Syed), Galal Yahya, Hanwen YANG, Xinxin Yang, Laiang Yao, Mohamed A Yassin, Sanni Yaya, Meghdad Yeganeh, Subah Abderehim Yesuf, Saber Yezli, Yazachew Engida Engida Yismaw, Dong Keon Yon, Naohiro Yonemoto, Chuanhua Yu, Ghazala Yunus, Umar Yunusa, Manijeh Zaghampour, Fathiah Zakham, Giulia Zamagni, Michael Zastrozhin, Mohammed Zawiah, Mohammed G M Zeariya, Alemu Birara Birara Zemariam, Tiansong Zhan, Casper J P Zhang, Jinpeng Zhang, Xiyu Zhang, Anthony Zhong, Jiayan Zhou, Bin Zhu, Magdalena Zielińska, Rafat Mohammad Zrieq, Ahed H. Zyoud, Sa'ed H Zyoud, Shaher H. Zyoud

Drafting the work or revising it critically for important intellectual content

Mohammad Amin Aalipour, Hasan Aalruz, Mitra Abbasifard, Hedayat Abbastabar, Samar Abd ElHafeez, Emad M. Abdallah, Reda Abdel-Hameed, Atef Abdelkader, Sherief Abd-Elsalam, Wakgari Mosisa Abdisa, Arman Abdous, Auwal Abdullahi, Toufik Abdul-Rahman, Armita Abedi, Roberto Ariel Abeldaño Zuñiga, Olumide Abiodun, Rahim Abo Kasem, Hassan Abolhassani, Ulric Sena Abonie, Abdullahi Tunde Aborode, Nagah Mohamed Abourashed, Mohamed Abouzid, Dmitry Abramov, Lucas Guimarães Abreu, Rana Kamal Abu Farha, Bilyaminu Abubakar, Eman Abu-Gharbieh, Hana J Abukhadijah, Salahdein Aburuz, Anirudh Balakrishna Acharya, Meshack Achore, Juan Manuel Acuna, Lisa C. Adams, Tajudeen Adesanmi

Adebisi, David Adedia, Kamoru Ademola Adedokun, Oluwatobi Emmanuel/E Adegbile, Oyelola A Adegboye, Nurudeen A Adegoke, Olumide Thomas Adeleke, Juliana Bunmi Adetunji, Mache Tsadik Adhana, Ripon Kumar Adhikary, Usha Adiga, Mohd Adnan, Qorinah Estiningtyas Sakilah Adnani, Prince Owusu Adoma, Aanuoluwapo Adeyimika Afolabi, Habeeb Abiodun Afolabi, Rotimi Felix Afolabi, Saira Afzal, Suneth Buddhika Agampodi, Sepehr Aghajanian, Mahsa Ahadi, Danish Ahmad, Rabbiya Ahmad, Shoaib Ahmad, Tauseef Ahmad, Ayman Ahmed, Haroon Ahmed, Muktar Beshir Ahmed, Mushood Ahmed, Naveed Ahmed, Syed Anees Ahmed, Simeon Okechukwu Ajakwe, Dolapo Emmanuel Ajala, Gizachew Taddesse Akalu, Roland Eghoghosoa Akhigbe, Mohammed Ahmed Akkaif, Ashley E Akrami, Alaa Al Amiry, Salah Al Awaidy, Mohammad Khaled Al nawayseh, Yazan Al Thaher, Omar Ali Mohammed Al Zaabi, Mohammad Ahmmad Mahmoud Al Zoubi, Yazan Al-Ajlouni, Khurshid Alam, Rasmieh Mustafa Al-amer, Fahmi Y. Al-Ashwal, Mohammed Albashtawy, Mohammad T AlBataineh, Abdulelah Mastour Aldhahir, Mohammed S Aldossary, Tekletsadik Tekleslassie Alemayehu, Ayman Al-Eyadhy, Fahad D Algahtani, Abdelazeem M Algammal, Ashraf Alhumaidi, Abid Ali, Liaqat Ali, Shahid Ali, Syed Shujait Ali, Montaha Al-Iede, Hamid Alinejad Rokny, Morteza Alipour, Samah W Al-Jabi, Ahmad Alkhatib, Mustafa Alkhawam, Mohammed Z. Allouh, Hesham M Al-Mekhlafi, Amr Almobayed, Hasan Yaser Alniss, Mohammad R. Alosta, Jaber S Alqahtani, Mohammad R. Alqudimat, Ahmed Yaseen Alqutaibi, Ahmad Alrawashdeh, Rami H Al-Rifai, Intima Alrimawi, Sahel Majed Alrousan, Zaid Altaany, Alaa B. Al-Tammemi, Jaffar A Al-Tawfiq, Malik A Althobiani, Nelson Alvis-Guzman, Nelson J Alvis-Zakzuk, Hassan Alwafi, Mohammad Al-Wardat, Yaser Mohammed Al-Worafi, Hany Aly, Mohammad Sharif Ibrahim Alyahya, Abdallah Alzoubi, Karem H Alzoubi, Amr Amin, Saeed Amini, Nafiu Aminu, Dickson A Amugsi, Filippos Anagnostakis, Michael Anderson, Song Peng Ang, Abhishek Anil, Abdul-Azeez Adeyemi Anjorin, Hossein Ansariniya, Catherine M Antony, Boluwatife Stephen Anuoluwa, Saeid Anvari, Saleha Anwar, Jalal Arabloo, Jesil Mathew Aranjani, Abdulfatai Aremu, Olatunde Aremu, Ghazal Arjmand, Jesu Arockiaraj, Mahwish Arooj, Ashokan Arumugam, Deepavalli Arumuganainar, Mahsa Asadi Anar, Muhammad Asaduzzaman, Syed Mohammed Basheeruddin Asdaq, Akram Ashames, Mitra Ashrafi, Bernard Kwadwo Yeboah Asiamah-Asare, Muhammad Shahzad Aslam, Yuni Asri, Dereje Zewdu Assefa, Batyrbek Assembekov, Alok Atreya, Zeenah A Atwan, Matteo Augello, Khursheed Aurangzeb, Andargie Abate Awoke, Babafela B Awosile, Seyyed HamidReza Ayatizadeh, Yusuf Oloruntoyin Ayipo, Sina Azadnajafabad, Sadat Abdulla Aziz, Ahmed Y. Azzam, Abisola Esther Babatope, Rasha Babiker, Ashish D Badiye, Yogesh Bahurupi, Atif Amin Baig, Senthilkumar Balakrishnan, Shirin Barati, Hiba Jawdat Barqawi, Shahid Bashir, Azadeh Bashiri, Quique Bassat, Mohammad-Mahdi Bastan, Saurav Basu, Mahdis Bayat, Bezawit K Bekele, Tariku Tesfaye Bekuma, Melesse Belayneh, Michael Belingheri, Umar Muhammad Bello, Samiun Nazrin Bente Kamal Tune, Ajeet Singh Bhadoria, Akshaya Srikanth Bhagavathula, Neeraj Bhala, Dinesh Bhandari, Pankaj Bhardwaj, Ashish Bhargava, Sonu Bhaskar, Priyadarshini Bhattacharjee, Jasvinder Singh Bhatti, Saeed Biroudian, Catherine Bisignano, Bijit Biswas, Mohammad Shahangir Biswas, Monirujjaman Biswas, Molalegne Bitew, Bruno Bizzozero-Peroni, Lucimere Bohn, Obasanjo Afolabi Bolarinwa, Paria Bolourinejad, Alejandro Botero Carvajal, Souad Bouaoud, Dejana Braithwaite, Hermann Brenner, Nikolay Ivanovich Briko, Danilo Buonsenso, Felix Busch, Yasser Bustanji, Sam Byrne, Mehtap Çakmak Barsbay, Angelo Capodici, Giulia Carreras, Andrea Carugno, Felix Carvalho, Joao Mauricio Castaldelli-Maia, Carlos A Castañeda-Orjuela, Luca Cegolon, Francieli Cembranel, Muthia Cenderadewi, Muge Cevik, Chiranjib Chakraborty, Sandip Chakraborty, Rama Mohan Chandika, Vijay Kumar Chattu, An-Tian Chen, Hana Chen, Haowei Chen, Nicholas WS Chew, Patrick R Ching, William C S Cho, Bryan Chong, Hitesh Chopra, Shivani Chopra, Sunghyun Chung, Muhammad Chutiyami, Alyssa Columbus, Joao Conde, Alexandru Corlateanu, Claudia Cosma, Natalia Cruz-Martins, Alanna Gomes da

Silva, Bashir Dabo, Emanuele D'Amico, Lucio D'Anna, Samuel Demissie Darcho, Latefa Ali Dardas, Gary L Darmstadt, Aso Mohammad Darwesh, Sindhura Deekonda, Aniket Dehadrai, Marco Del Riccio, Edgar Denova-Gutiérrez, Anteneh Assefa Desalegn, Pradeep Kumar Devarakonda, Syed Masudur Rahman Dewan, Arkadeep Dhali, Meghnath Dhimal, Sameer Dhingra, Stefano Di Bella, Marcello Di Pumpo, Diana Dias da Silva, Daniel Diaz, Xueting Ding, Thanh Chi Do, Sushil Dohare, Wendel Mombaque dos Santos, Ojas Prakashbhai Doshi, Menayit Tamrat Dresse, John Dube, Senbagam Duraisamy, Oyewole Christopher Durojaiye, Osamudiamen Ebohon, Lamiaa Labieb Mahmoud Ebraheim, Mohammad Hossein --- Ebrahimi, Rasoul Ebrahimi, Ferry Efendi, Behrad Eftekhari, Ashkan Eighaei Sedeh, Ebrahim Eini, Michael Ekholuenetale, Rabie Adel El Arab, Ibrahim Farahat El Bayoumy, Maysaa El Sayed Zaki, Aya Elalfy, Said El-Ashker, Iffat Elbarazi, Noha Mousaad Elemam, Muhammed Elhadi, Mohamed Hassan Elnaem, Mohammed Elshaer, Abdelgawad Salah Abdelgawad Eltahawy, Theophilus I Emeto, Talha Bin Emran, Misganu Endriyas, Sharareh Eskandarieh, Maysa Eslami, Heidar Fadavian, Adeniyi Francis Fagbamigbe, Ayesha Fahim, Niloofar Faraji, Seyed Nooreddin Faraji, Mohammad Fareed, MoezAlIslam Ezzat Mahmoud Faris, Andre Faro, Zareen Fatima, Ulrich Membe Femoe Membe, Rodrigo Fernandez-Jimenez, Natan Feter, Claudio Fiorilla, Florian Fischer, Marco Fonzo, Takeshi Fukumoto, Nancy Fullman, Muktar A Gadanya, Dominic Dormenyo Gadeka, Márió Gajdács, Balasankar Ganesan, Xiang Gao, Bashiru Garba, Jacopo Garlasco, Anteneh Gashaw, Zisis Gatzioufas, Rupesh K Gautam, Miglas Welay Gebregergis, Nsikakabasi Samuel George, Ubong Uwem George, Gebremariam Wulie Geremew, Gebremariam Getaneh, Kazem Ghaffari, Roya Ghafoury, Arin Ghamkhar, Shakiba Ghasemi Assl, Haniyeh Ghasrsaz, Ramy Mohamed Ghazy, Nermin Ghith, Arun Ghuge, Artyom Urievich Gil, Alessandro Girombelli, Laszlo Göbölös, Davide Golinelli, Aman Goyal, Shi-Yang Guan, Giovanni Guarducci, Amit Gulati, Sasidhar Gunturu, Ishita Gupta, Sapna Gupta, Veer Bala Gupta, Vivek Kumar Gupta, Reyna Alma Gutiérrez, Roberth Steven Gutiérrez-Murillo, Awoke Derbie Habteyohannes, Emily Haeuser, Dariush Haghmorad, Haimanot Ewnetu Hailu, Pritam Halder, Islam M Hamad, Nadia M Hamdy, Sajid Hameed, Nasrin Hanifi, Graeme J Hankey, Harapan Harapan, Arief Hargono, Josep Maria Haro, Ahmed I Hasaballah, Hamidreza Hasani, Mohammad Hashem Hashempur, Ibrahim Nagmeldin Hassan, Md. Imtaiyaz Hassan, Muhammad Hassan, Nageeb Hassan, Simon I Hay, Wen-Qiang He, Mohamed I Hegazy, Golnaz Heidari, Minoo Heidari Almasi, Kamal Hezam, Yuta Hiraike, Alamgir Hossain, Lubna Hossain, Md Mahbub Hossain, Md Sabbir Hossain, Md. Jubayer Hossain, Mehdi Hosseinzadeh, Priya Hotwani, Hanno Hoven, Junjie Huang, M. Azhar Hussain, Nawfal R Hussein, Mohamed Ibrahim Husseiny, Hong-Han Huynh, Segun Emmanuel Ibitoye, Khalid S Ibrahim, Anel Ibrayeva, Olayinka Stephen Ilesanmi, Irena M Ilic, Milena D Ilic, Arit Inok, Mustafa Alhaji Isa, Teresa R. Iskander, Md Sahidul Islam, Sheikh Mohammed Shariful Islam, Faisal Ismail, Leila Ismail, Mosimah Charles Ituka, Chinwe Juliana Iwu-Jaja, Louis Jacob, Ali Jadidi, Abdollah Jafarzadeh, Haitham Jahrami, Mihajlo Jakovljevic, Mohamed Jalloh, Armaan Jamal, Qazi Mohammad Sajid Jamal, Melika Jameie, Jerin James, Hasan Jamil, Syed Sarmad Javaid, Qassim Jawell Odah Abed, Shubha Jayaram, Seogsong Jeong, Wenyi Jin, Jobin Jose, Nitin Joseph, Charity Ehimwenma Joshua, Jacek Jerzy Jozwiak, Vidya Kadashetti, Dler H. Hussein Kadir, Ashish Kumar Kakkar, Md Moustafa Kamal, Rajesh Kamath, Ramat T. Kamorudeen, Oleksandr Kamyshnyi, Mona Kanaan, Saddam Fuad Kanaan, Jiseung Kang, Samuel Berchi Kankam, Kehinde Kazeem Kanmodi, Suthanthira Kannan S, Rami S Kantar, Neeti Kapoor, Reema A Karasneh, André Karch, Mohmed Isaqali Karobari, Tomasz M. Karpiński, Manoj Kumar Kashyap, Himanshu Khajuria, Mohammad Ali Khaksar, Anees Ahmed Khalil, Ajmal Khan, Gulfaraz Khan, Maseer Khan, Md Abdullah Saeed Khan, Moien AB Khan, Muhammad Umer Khan, Ramsha Mushtaq Khan, Sumaiya Khan Khan, Yusuf Saleem Khan, Zahid Khan, Vishnu Khanal, Sameer Uttamaro Khasbage, Khaled Khatab, Haitham Khatatbeh, Moawiah Mohammad Khatatbeh, Khalid A Kheirallah, Grace Kim,

Kwanghyun Kim, Min Seo Kim, Adnan Kisa, Sezer Kisa, Shivakumar KM, Sonali Kochhar, Michail Kokkorakis, Vladimir Andreevich Korshunov, Oleksii Korzh, Karel Kostev, Parvaiz A Koul, James-Paul Kretchy, Kewal Krishan, Barthelemy Kuate Defo, Raja Amir Hassan Kuchay, Mohammed Kuddus, Ilari Kuitunen, Mukhtar Kulimbet, Dewesh Kumar, Jogender Kumar, Kamal Kumar, Narendar Kumar, Rakesh Kumar, Satyajit Kundu, Setor K Kunutsor, Maria Dyah Kurniasari, Asep Kusnali, Dian Kusuma, Wai Hang Patrick Kwong, Frank Kyei-Arthur, Dr Pallavi L C, Carlo La Vecchia, Muhammad Awwal Ladan, Chandrakant Lahariya, Balzhan Lakanova, Iván Landires, Savita Lasrado, Colleen L Lau, Huu-Hoai Le, Minh Huu Nhat Le, Nhi Huu Hanh Le, Caterina Ledda, Sergey Vadimovich Lee, Awol Yemane Legesse, Elvynna Leong, Stephen S Lim, John C Lin, Queran Lin, Jue Liu, Zhe Liu, Erand Llanaj, José Francisco López-Gil, Platon D Lopukhov, Giancarlo Lucchetti, Peng Luo, Angelina M Lutambi, Miltiadis D. Lytras, Ellina Lytvyak, Ahmed M. Afifi, Kevin Sheng-Kai Ma, Zheng Feei Ma, Shamsuddeen Yusuf Yusuf Ma'aruf, Mahmoud Mabrok, Monika Machoy, Farzan Madadizadeh, Seyed Ataollah Madinezad, Aurea Marilia Madureira-Carvalho, Sasikumar Mahalingam, Samatar Abshir Mahamed, Mansour Adam Mahmoud, Hardeep Singh Malhotra, Ahmad Azam Malik, Shahid Malik, Tabarak Malik, Deborah Carvalho Malta, Biniyam Tedla Tedla Mamo, Lokesh Manjani, Kamaruddeen Mannethodi, Tahir Maqbool, Bishnu P Marasini, Hamid Reza Marateb, Konstantinos Margetis, Michael Marks-Hultström, Adolfo Martinez-Valle, Francisco Rogerlândio Martins-Melo, Miquel Martorell, Roy Rillera Marzo, Sammer Marzouk, Stefano Masi, Clara N Matei, Yasith Mathangasinghe, Medha Mathur, Fernanda Penido Matozinhos, Chioma Ngozichukwu Pauline Mbachu, Ikechukwu Innocent Mbachu, Susan A McLaughlin, Steven M McPhail, María Paz Medel Salas, Rishi P Mediratta, Vini Mehta, James Meiring, Tesfahun Mekene Meto, Tesfaye Hambisa Mekonnen, Hadush Negash Meles, Endalkachew Belayneh Melese, Walter Mendoza, Godfred Antony Menezes, Ritesh G Menezes, Leweyehu Alemaw Mengstie, Alexios-Fotios A Mentis, Sultan Ayoub Ayoub Meo, Atte Meretoja, Tomislav Mestrovic, Sachith Mettananda, Mohamed M.M. Metwally, Irmina Maria Michalek, Giuseppe Minervini, Wai-kit Ming, Heba M. Mohamed, Hebatalla Mohamed, Mona Gamal Mohamed, Nouh Saad Mohamed, Khabab Abbasher Hussien Abbasher Hussien Mohamed Ahmed, Taj Mohammad, Shafiu Mohammed, Yahaya Mohammed, Mohammad Mohseni, Ali H Mokdad, Lorenzo Monasta, Mohammad Ali Moni, Maryam Moradi, Paula Moraga, Shane Douglas Morrison, Mahmoud M Morsy, Jonathan F Mosser, Seyed Mohamad Sadegh Mousavi Kiasary, Hagar Mowafy, Ulrich Otto Mueller, Francesk Mulita, Anjana Munshi, Christopher J L Murray, Fungai Musaigwa, Sherzad Ibrahim Mustafa, Saravanan Muthupandian, Claude Mambo Mambo Muvunyi, Muhammad Muzaffar, Ayoub Nafei, Mobin Naghshbandi, Soroush Najdaghi, Sreenivas Narasimha Swamy, Shumaila Nargus, Mahmoud Nassar, Zuhair S Natto, Samidi Nirasha Kumari Navaratna, Biswa Prakash Nayak, Shalini Ganesh Ganesh Nayak, Md Fahad Shahariar Nayon, Ionut Negoi, Samata Nepal, Jason Nguyen, Kieu Viet Nhi Nguyen, Quan Nguyen Khoi, Robina Khan Niazi, Luciano Nieddu, Afewerki Tesfahunegn Tesfahunegn Nigusse, Masoud Noroozi, Valentine C. Nriagu, Chisom Adaobi Nri-Ezedi, Jean Claude Nshimiyimana, Fred Nugen, Bogdan Oancea, Mary Aigbiremo Oboh, Ramez M. Odat, Ismail A Odetokun, Tunde Emmanuel Ogundare, Olusegun Olatunji Ojedoyin, Akinkunmi Paul Okekunle, Osaretin Christabel Okonji, John Olayemi Okunlola, Antonio Olivas-Martinez, Gláucia Maria Moraes Oliveira, Abdulhakeem Abayomi Olorukooba, Samson Bamidele Olorunju, Comfort Z. Z Olorunsaiye, Bolajoko Olubukunola Olusanya, Obinna E Onwujekwe, Chizaram A Onyeaghala, Marcel Opitz, Michal Ordak, Verner N Orish, Atakan Orscelik, Alberto Ortiz, Edgar Ortiz-Brizuela, Esteban Ortiz-Prado, Augustus Osborne, Eric Osei, Elham H. Othman, Oche Joseph Otorkpa, Amel Ouyahia, Mayowa O Owolabi, Kolapo Oyebola, Tope Oyelade, Oyetunde T Oyeyemi, Ilker Ozsahin, Jagadish Rao Padubidri, Yeganeh Pakbaz, Tamás Palicz, Sujogya Kumar Panda, Leonidas D. D Panos, Mario Virgilio Papa, Ilias Papadimopoulos,

Shahina Pardhan, Romil R Parikh, Roberto Passera, Mitesh Patel, Neel Navinkumar Patel, Shankargouda Patil, Dimitrios Patoulias, Shrikant Pawar, Shubhadarshini Pawar, Hamidreza Pazoki Toroudi, Veincent Christian Filipino Pepito, Gavin Pereira, Olumuyiwa James Peter, Nhat Truong Pham, Zahra Zahid Piracha, Edoardo Pirera, Dimitri Poddighe, Sajjad Pourasghary, Reza Pourbabaki, Ashwathi Prakash, Elton Junio Sady Prates, Jyotirekha Purohit, Jagadeesh Puvvula, Nameer Hashim Qasim, Asma Saleem Qazi, Xiang Qi, Zhipeng Qi, Gangzhen Qian, Venkatraman Radhakrishnan, Fakher Rahim, Sajjad Rahimi, Vafa Rahimi-Movaghar, Md. Mosfequr Rahman, Saeed Rahmani, Hakim Rahmoune, Sunil Kumar Raina, Jeffrey Pradeep Raj, Gunaseelan Rajendran, Judah Rajendran, Mohammad Amin Rajizadeh, Siddheesh Rajpurohit, Mahmoud Mohammed Ramadan, Chitra Ramasamy, Shakthi Kumaran Ramasamy, Kamleshun Ramphul, Rishabh Kumar Rana, Nemanja Rancic, Smitha Rani, Chythra R Rao, Sowmya J Rao, Devarajan Rathish, David Laith Rawaf, Salman Rawaf, Elrashdy Redwan, Wajiha Rehman, Luis Felipe Reyes, Mina Rezaei, Abanoub Riad, Moattar Raza Rizvi, Hermano Alexandre Lima Rocha, Thales Philipe Rodrigues da Silva, Leonardo Roever, Amirhossein Roshanshad, Himanshu Sekhar Rout, Shiva Rouzbahani, Nitai Roy, Sharmistha Roy, Shubhanjali Roy, Guilherme de Andrade Ruela, Godfrey M Rwegerera, Aly M A Saad, Maha Mohamed Saber-Ayad, Seyed Kiarash Sadat Rafiei, Basema Ahmad Saddik, Tarannom Sadegh, Fatemeh Sadeghi-Ghyassi, Mohd Saeed, Umar Saeed, Mehdi Safari, Mastooreh Sagharichi, Dominic Sagoe, Narjes Saheb Sharif-Askari, S. Mohammad Sajadi, Md Refat Uz Zaman Sajib, Mirza Rizwan Sajid, Morteza Saki, Nasir Salam, Afeez Abolarinwa Salami, Mahdi Salehi, Aanuoluwa James Salemcity, Dauda Salihu, Malik Sallam, Saad Samargandy, Abdallah M Samy, Sathish Sankar, Adekunle Sanyaolu, Jacob Owusu Sarfo, Hemen Sarma, Mohammad Sarmadi, Sachin C Sarode, Maheswar Satpathy, Monika Sawhney, Ganesh Kumar Saya, Ione Jayce Ceola Schneider, Art Schuermans, Ashenafi Kibret Sendekie, Yigit Can Senol, Dragos Serban, Yashendra Sethi, Seyed mohammad Seyed alshohadaei, Abubakar Sha'aban, Muhammad Shahab, Samiah Shahid, Farshad Shahkarami, Moyad Jamal Shahwan, Ahmed Shaikh, Alireza Shakeri, Mehran Shams-Beyranvand, Anas Shamsi, Alfiya Shamsutdinova, Dan Shan, Mohammed Shannawaz, Amin Sharifan, Bunty Sharma, Manoj Sharma, Vishal Sharma, Ramzi Shawahna, Samendra P Sherchan, Md. Monir Hossain Shimul, Aminu Shittu, Azad Shokri, Sina Shool, Seyed Afshin Shorofi, Kerem Shuval, Zahra Siavashpour, Emmanuel Edwar Siddig, Ayesha Siddiqua, Luís Manuel Lopes Rodrigues Silva, Bhim Pratap Singh, Harmanjit Singh, Jasvinder A Singh, Poornima Suryanath Singh, Samer Singh, Satwinder Singh, Natia Skhvitaridze, Valentin Yurievich Skryabin, Amanda E Smith, Anton Sokhan, Ahmed M. Soliman, May Mohamed Sherif Soliman, Noha Salah Soliman, Weiyi Song, Prashant Sood, Soroush Soraneh, Michele Sorrentino, Michael Spartalis, Manraj Singh Sra, Chandrashekhar T Sreeramareddy, Bahadar S Srichawla, Manikandan Srinivasan, Devin Bailey Srivastava, Aleksandar Stevanović, Omer Subasi, Surajo Kamilu Kamilu Sulaiman, Muhammad Suleman, Mark J M Sullman, Anusha Sultan Meo, Thanigaivel Sundaram, David Sunkersing, Tarun Kumar Suvvari, Lukasz Szarpak, Rafael Tabarés-Seisdedos, Seyed-Amir Tabatabaeizadeh, Celine Tabche, Ramin Tabibi, Takahiro Tabuchi, Lidia S. Seifu Tadesse, Iman M Talaat, Mircea Tampa, Jacques Lukenze Tamuzi, Ker-Kan Tan, Saba Tariq, Anika Tasnim, Nathan Y Tat, Vivian Y Tat, Birhan Tsegaw Taye, Yibekal Manaye Tefera, Mohamad-Hani Temsah, Reem Mohamad Hani Temsah, Wegen Beyene Tesfamariam, Samar Tharwat, Muthu Thiruvengadam, Marius Belmondo Tincho, Sojit Tomo, Marcos Roberto Tovani-Palone, Khaled Trabelsi, Tam Quoc Minh Tran, Thang Huu Tran, Nguyen Tran Minh Duc, Indang Trihandini, Samuel Joseph Tromans, Claudia Truppa, Aristidis Tsatsakis, Lilian Tzivian, Lawan Umar, Brigid Unim, Era Upadhyay, Jeba Mahiad Urmey, Jibrin Sammani Usman, Hande Uzunçıbuk, Pratyusha Vadagam, Asokan Govindaraj Vaithinathan, Jef Van den Eynde, Joe Varghese, Tommi Juhani Vasankari, Srivatsa Surya Vasudevan, Baskar Venkidasamy, Simone Villa, Jorge Hugo Villafañe, Leonardo

Villani, Manish Vinayak, Stein Emil Vollset, Yasir Waheed, Cong Wang, Qingzhi Wang, Ruixuan Wang, Wei Wang, Xing Wang, Ahmed Bilal Waqar, Joseph L Ward, Yilkal Abebaw Wassie, Ishanka Weerasekara, Nuwan Darshana Darshana Wickramasinghe, Angga Wilandika, Peter Willeit, Marcin W Wojewodzic, Yohannes Chemere Wondmeneh, Minichil Chanie Chanie Worku, James Fan Wu, Qing Xia, Site Xu, Mingyang Xue, Mukesh Kumar Yadav, Saba Yahoo (Syed), Galal Yahya, Hanwen YANG, Xinxin Yang, Laiang Yao, Mohamed A Yassin, Yuichi Yasufuku, Sanni Yaya, Meghdad Yeganeh, Saber Yezli, Dong Keon Yon, Naohiro Yonemoto, Ghazala Yunus, Umar Yunusa, Manijeh Zaghampour, Fathiah Zakham, Michael Zastrozhin, Mohammed Zawiah, Mohammed G M Zeariya, Alemu Birara Birara Zemariam, Jinpeng Zhang, Xiyu Zhang, Anthony Zhong, Jiayan Zhou, Bin Zhu, Hafsa Zia, Magdalena Zielińska, Ghazal Zoghi, Rafat Mohammad Zrieq, Ahed H. Zyoud, Sa'ed H Zyoud, Shaher H. Zyoud

Managing the estimation or publications process

Emily Haeuser, Ashley A Harris, Simon I Hay, Stephen S Lim, Paulina Lindstedt, Ali H Mokdad, Jonathan F Mosser, Christopher J L Murray, Amanda E Smith
