# Supplementary material for: Global, regional, and national trends in routine childhood vaccination coverage from 1980 to 2023 with forecasts to 2030: a systematic analysis for the Global Burden of Disease Study 2023
Source: Lancet. Author manuscript; Available in PMC 2025 Aug 11. (PMC12338332; doi:10.1016/S0140-6736(25)01037-2)

Appendix 1: supplementary methods and results for "Global, regional, and national trends in routine childhood vaccination coverage from 1980 to 2023 with forecasts to 2030: a systematic analysis for the Global Burden of Disease Study 2023"

Section 1 Supplemental Methods 2
- Section 1.1 Data inclusion and exclusion criteria 2
- Section 1.2 DTP1 imputation 2
- Section 1.3 Administrative data bias modelling 3
- Section 1.4 Modelling of stockouts and other disruptions to vaccine coverage, including COVID-19 3
- Section 1.5 Imputation of disruptions due to COVID-19 4
- Section 1.6 Scale-up modelling of newer vaccines 4
- Section 1.7 Model validation 5
- Section 1.8 Calculating COVID-free counterfactual coverage 5
- Section 1.9 Tracking Progress towards IA2030 50% zero-dose reduction target 6
- Section 1.10 Forecasting life-course vaccine coverage 6

Section 2 Model validation and comparisons 7
- Section 2.1 Goodness of fit 7
- Section 2.2 Comparisons to GBD 2020 8
- Section 2.3 Comparisons to WUENIC 8

Section 3 Supplemental references 9

Section 4 List of supplemental tables and figures 10

Section 5 Supplemental tables 14
- Table S1 GATHER Compliance 14
- Table S2 Citation table 15
- Table S3 Excluded sources 146
- Table S4 Supplemental stockout and disruption events 217
- Table S5 Super-regions and corresponding countries and territories 228
- Table S6 COVID-inclusive vs. COVID-free counterfactual coverage of EPI vaccines by super-region in 2023 229
- Table S7 Zero-dose counts and Immunization Coverage for 2019 and 2023, with 2030 Projections 232
- Table S8 Global model fit parameters used as priors in country-level post-introduction scale-up modelling ..238
- Table S9 Validation metrics for ST-GPR models 239
- Table S10 Validation metrics for MR-BRT first stage models 240

Section 6 Supplemental figures 241
- Figure S1 Flow chart of modelling processes 242

Figure S2 Flow chart of data screening processes 244
Figure S3 Predicted fits of global scale-up models by newer vaccine ratio 245
Figure S4 Global disruption magnitudes by year 246
Figure S5 Vaccine coverage time series plots 247
Figure S6 Vaccine coverage maps....452
Figure S7 Impact of COVID-19 pandemic on vaccine coverage by super-region. 486
Figure S8 Forecast vaccine coverage for years 2024-2030 by super-region 487
Figure S9 Vaccine coverage in 2023 vs. forecast vaccine coverage in 2030 by super-region....488
Figure S10 Distributions sampled to impute COVID-related disruption magnitudes by year....489
Figure S11 Global vaccine coverage time series plots, GBD 2023 vs GBD 2020 vs. WUENIC 490
Figure S12 Vaccine coverage in GBD2020 vs. vaccine coverage in GBD2023 491
Figure S13 WUENIC vaccine coverage vs. GBD vaccine coverage in 2023....492

Section 1 Supplemental Methods

Section 1.1 Data inclusion and exclusion criteria

Administrative JRF data report the number of doses delivered and number of children in the target population per location, year, and vaccine dose, whereas official country-reported data comprise a single coverage location-year-vaccine-dose-specific value. Because administrative JRF data are only available starting in 2000, we primarily used official country-reported JRF coverage data for the years of the study period, from 1980 to 2023.[1] For survey-based sources, we extracted individual-level microdata when available; otherwise, national-level coverage aggregates were used.

Similar to the GBD 2020 Vaccine Coverage study, we defined coverage of a given vaccine as the proportion of children ages 12 to 59 months who received at least the proscribed dosage of that vaccine as part of a national immunization programme providing routine childhood vaccinations; doses received during targeted vaccination campaigns were excluded when possible. We considered children reported in the microdata as vaccinated with a given dose based on either physical evidence of vaccine receipt (vaccination card or home-based record) or parental recall. Microdata or other sources that did not include data on children aged 12 to 59 months were excluded. Also excluded were sources that were not nationally representative, either in terms of geography or sociodemographic characteristics; sources that did not include dose-specific vaccine coverage from at least one vaccine in or after the country-reported national introduction year; and coverage data from before the introduction of the given vaccine or otherwise judged to be implausible.

Due to a lack of available coverage data for select newer vaccines in China, as in the 2020 GBD study, we used lot release data from the China National Institutes for Food and Drug Control (China National Institutes for Food and Drug Control[8] and Y Teng, Linksbridge SPC, personal communication) to inform our coverage estimates of Hib3, PCV3 and RotaC in China by constraining coverage for each of these vaccines based on theoretical maximums of coverage per year since introduction based on annual reported doses. For years missing stock information, dose counts from the last year with available data were used. The methods for this constraint follow those used in the earlier study.

Section 1.2 DTP1 imputation

For years where country-reported or administrative data for DTP3 was available but DTP1 data was not, we imputed DTP1 data from DTP3 country-reported data based on relationships between DTP1 and DTP3 country-reported data in years with data for both doses, modelled using spatiotemporal Gaussian process regression (ST-GPR).[2] The methods here mirrored those used in GBD 2020, however we extended this method to be applied to data in any year with missing DTP1 data, rather than just years 1980-2000.

In few instances, survey data would include information regarding DTP3 coverage, but not DTP1. In the new approach of modelling DTP1 directly and DTP3 as a ratio of DTP3/DTP1, these few surveys were no longer eligible for inclusion in our coverage models. To retain the information these surveys provided in administrative data bias modelling (see below), we imputed DTP1 coverage values for these surveys to include in DTP1 bias modelling only. To impute this data, we fit a linear regression of DTP1 survey-to-admin data log ratios predicted by DTP3 survey-to-admin data log ratios. These imputed values were included only in DTP1 administrative data bias modelling, and not in the coverage models themselves.

Section 1.3 Administrative data bias modelling

To quantify location- and time-varying bias in country-reported data, we modelled the relationships between matching location-year-vaccine-specific country-reported and survey-based data pairs, using country-specific Healthcare Access and Quality (HAQ) Index[3] values as a covariate. Survey-based coverage data are considered more accurate and less biased[4] but are collected irregularly and infrequently compared to data reported annually from countries. Select survey sources that produced implausible effects when used to inform bias adjustments to country-reported data this modelling process were omitted from bias modelling while still being retained in the final coverage models (table S3). After generating bias-adjustment factors, we multiplied each year of official country-reported coverage data by its corresponding bias factor to yield bias-adjusted country-reported coverage data. Bias estimates were calculated and directly applied for DTP1, DTP3, MCV1, Pol3, and BCG. Notably, bias models were generated for both DTP1 and DTP3 and their input data adjusted independently, and data calculations for the modelling of DTP3/DTP1 ratio inputs included bias-adjusted country-reported data for both the numerator and denominator. For the newer vaccines also modelled as ratios—HepB3, Hib3, PCV3, RotaC, MCV2, and RCV1—ratio data calculations used unadjusted data for both numerators and denominators. For these newer vaccines, final coverage was produced by multiplying these ratios by final estimates of DTP3 or MCV1 coverage, which had incorporated bias-adjusted administrative data. This approach leverages the bias adjustments for DTP3 and MCV1, assuming that the ratio of newer vaccine coverage to coverage of these reference vaccines in the administrative data is unbiased. These methods mirror those used in the previous GBD 2020 study.[5]

Section 1.4 Modelling of stockouts and other disruptions to vaccine coverage, including COVID-19

The spatiotemporal gaussian process regression (ST-GPR) approach we used to model vaccine coverage benefits from borrowing strength in vaccination trends across space and time. However, vaccine coverage in certain locations may experience acute disruptions due to isolated events like stockouts or conflicts that may temporarily limit access to vaccinations, with resumption of services following the event bringing about a sharp increase in coverage to earlier levels. To account for such events, we included a covariate in our coverage models to estimate the relative size of a coverage disruption in a given country-vaccine-year, with zero indicating no disruption. To identify potential disruption events, we looked at the WHO stockout reporting data[6] and supplemented this list with other known events (table S4). To account for disruptions due to the COVID-19 pandemic, starting from 2020 forward, every country-vaccine-year was considered to be a candidate for a disruption. This included years 2022 and 2023, as a review of the administrative and survey data from these years indicated continued global reductions in coverage relative to global coverage in 2019.[7]

To estimate the size of disruptions to coverage for these candidate vaccine-country-years, we first produced vaccine coverage models using ST-GPR for a counterfactual scenario assuming no disruptions. These models included only country-reported data (ie, excluded survey data) and excluded data from any vaccine-country-year flagged as experiencing potential disruptions. The results of this model therefore reflect expected country-reported coverage in each candidate disruption year, absent any disruptions. After predicting out this counterfactual coverage, we then calculated the difference in logit space between these estimates and those left-out data points observed in disruption

vaccine-country-years. This discrepancy indicated the size of the disruption observed in the given vaccine-country-year and was used to form the disruption covariate. Where observed data coverage was equal to or higher than the predicted counterfactual, or when no disruption had been reported, disruption values for that country-year were set to zero. These disruption values then served as a covariate in the first stage of ST-GPR vaccination coverage modelling. For vaccines modelled as ratios, these same steps were implemented, using coverage ratio data and modelling.

Section 1.5 Imputation of disruptions due to COVID-19

The method used to estimate disruption effects relies on the availability of country-reported data. For disruptions due to COVID-19 in years 2020-2023, where country-reported data was unavailable for a given vaccine-year, we imputed disruptions to coverage post-hoc. Disruptions were imputed for 1926 (21·5%) of the 8976 vaccine-country-years modeled during this time period. These values were imputed at the draw level by sampling from a two-stage distribution of disruption sizes. For each vaccine-year, we first simulated a binomial distribution based on the probability of a disruption occurring, based upon the proportion of countries with available data that reported disruptions for that vaccine and year. Second, we simulated an exponential distribution, based on a rate parameter that resulted in the best fit to the distribution of disruption magnitudes for countries with data available and that reported disruptions for that vaccine and year. This set of distributions was selected by comparing the goodness of fit of several distribution options using the *distchoose* function from the EnvStats package.[8] Taking 1000 draws from each of these two simulated distributions, we multiplied the two together to impute disruptions (figure S10). By draw, these imputed disruption samples were then applied to coverage estimates for each vaccine-country-year in 2020-2023 where country-reported data was missing to calculate coverage accounting for disruptions due to COVID-19 ($coverage_{CI,imputed}$):

$$coverage_{CI,imputed} = coverage - coverage * disruption\ effect$$

Section 1.6 Scale-up modelling of newer vaccines

For the newer vaccines (ie, HepB3, Hib3, MCV2, PCV3, RotaC, and RCV1), modelling coverage was complicated by later, country-specific introductions that occurred during the estimation period. Many such introductions included a “scale-up” period with low initial coverage followed by gradual increases over several years before reaching a plateau. We implemented new methods with this analysis to better account for these patterns. First, to account for varied introduction dates, when modelling coverage ratios across space and time for these vaccines using ST-GPR, we considered time relative to “years since introduction”, thereby allowing our models to borrow information and smooth across space and time for countries and territories that may have introduced these vaccines at different time points. Second, to account for scale-up of coverage following introduction, we replaced the traditional linear modelling first stage of ST-GPR with cascading spline models using meta-regression—Bayesian, regularised, trimmed (MR-BRT) tools.[9] This approach begins by fitting a global scale-up curve for each vaccine, which is then used as a prior for country-specific models where data are available. This approach allows for the estimation of country-specific scale-up patterns where supported by robust data, while allowing scale-up estimates in comparatively data-sparse locations to benefit from patterns observed in other countries and territories.

For each vaccine ratio, we first fit a model globally:

$$p = \beta_0 + \beta_1 X + spline(YSI)$$

Where p is the proportion of children vaccinated, β_0 is the intercept, $\beta_1 X$ is the disruption covariate, and $spline(YSI)$ is a spline fits to years since introduction with knots placed at the year of introduction, five years following introduction, and 50%, 95% and 100% of the year range (which varied by vaccine). The first year of introduction was considered to be the first year that the vaccine was introduced anywhere in the country, regardless of whether that represented a national or subnational initial roll-out. The fit of knot values for the $spline(YSI)$ parameter were constrained to be monotonically increasing, meaning that coverage ratios were constrained to increase over time, apart from modelled disruption events. However, because reference vaccines were not

constrained in this way, absolute coverage of a newer vaccine was still allowed to decrease over time if such a trend were present in its respective reference vaccine's coverage estimates. For each global model, 10% of the data was trimmed using MR-BRT's algorithm to detect and remove outliers. Covariates for the HAQ index and mortality due to conflict were not directly included in the ratio vaccine models—rather, for these vaccines, we assume that these relationships would mirror those of the reference vaccines.

To allow for variation in scale-up curves between locations, we then used the fits from these global models to inform country-specific models of the same formula (figure S3). The coefficient values (mean and variance) for intercept, stockout covariate and years-since-introduction spline knot placement from the global models served as priors on these respective parameters in the country-specific models (table S8). Mean coverage values from 1000 prediction draws for each vaccine-country-year were combined and used as custom stage 1 results in ST-GPR ratio coverage models. For countries and territories with no data available, predictions based on global model fits were used. This allowed for global trends to be used in locations with no data availability.

Section 1.7 Model validation

Five-fold out of sample cross validation was used to assess performance of ST-GPR coverage models and MR-BRT scale-up models (tables S9 & S10). Strategic decision-making about vaccine policy requires an accurate understand of current coverage and recent coverage trends. Survey data, however, are often subject to delays in data cleaning and release, or may not be available. In these cases, modelled coverage estimates in more recent years are informed by past surveys, trends in bias-adjusted country-reported data, covariate patterns, and residual trends across space and time. We therefore designed a cross-validation strategy to assess the model's performance in more recent years, in the absence of recent survey data. Survey data sources from years 2016-2023 were randomly assigned to one of five folds, with all data from a given survey assigned to that fold across all years and vaccines. We then re-ran all coverage models five times, once for each fold, with all data assigned to the fold withheld from the corresponding models. Last, we paired each withheld survey observation with the estimated value from model from which that data point had been withheld (ie, the out-of-sample prediction). We repeated these calculations using the estimated values from the coverage models from the main manuscript, ie, models fit to all available data, in order to derive corresponding in-sample validation metrics.

As statistical models were fit to vaccine ratios, fit metrics were calculated for the estimated of vaccine ratios, to more directly assess the models' ability to estimate scale-up of newer vaccines. Root mean square error (RMSE) and median absolute error (MedAE) were used to assess model accuracy in estimating the central tendency. Median error was used to assess bias in the model fit. RMSE was calculated according to the formula:

$$RMSE = \sqrt{\frac{1}{n}\sum_{i-1}^{n}(y_i - \hat{y}_i)^2}$$

where n is the number of expected/observed data pairs, y_i is the vaccine coverage value for the ith survey and $\hat{y}_i$ is the modelled vaccine coverage estimate for the ith comparison. Median absolute error and median error were calculated as:

$$MedAE = median(|y_1 - \hat{y}_1|, \dots, |y_n - \hat{y}_n|) Median\ Error = median(y_1 - \hat{y}_1, \dots, y_n - \hat{y}_n)$$

where $y - \hat{y}$ is the residual between each survey and the paired model estimate from 1 to n surveys.

Section 1.8 Calculating COVID-free counterfactual coverage

COVID-free counterfactual coverage was calculated by first calculating expected coverage in the absence of any disruptions. To accomplish this, we removed the disruption covariate effect in years affected by COVID-19 from our coverage estimates using the following formula:

$$coverage_{disruption-} = invlogit(logit(coverage_{with\ COVID}) + \beta_{disruption} * (-X))$$

where X is the disruption covariate input value and $\beta_{disruption}$ is the fitted parameter value taken from the linear model stage of the ST-GPR coverage model for each vaccine. Where the disruption covariate value equalled zero (ie, no disruptions were seen), coverage with disruptions and the disruption-free counterfactual estimates were identical. These calculations were performed at the draw level.

Even in the absence of the COVID-19 pandemic, however, it is unlikely that no other disruption events would have occurred anywhere globally during the years 2020-2023. To account for other potential disruptions in our estimation of COVID-free counterfactual coverage, we assessed trends in disruptions preceding the COVID-19 pandemic in each country. We first estimated "disruption-free" counterfactual coverage for the five years preceding the COVID-19 pandemic (2015-2019) using the same formula as described above. Then, by draw, we calculated the differences in coverage with and without disruptions for each country- and vaccine in each of these pre-COVID-19 pandemic years (figure S4). To calculate adjusted COVID-free counterfactual coverage for years 2020-2023 ($coverage_{CF,adjusted}$) by draw, we then used the following formula:

$$coverage_{CF,adjusted} = coverage_{CF} - coverage_{CF} * disruption\ effect$$

Where $coverage_{CF}$ are the disruption-free coverage estimates from 2020-2023 prior to adjustment, and the $disruption\ effect$ is the percent difference between the disruption-inclusive and disruption-free counterfactual estimates for years 2015-2019, ie, before the COVID-19 pandemic. Given the unpredictable nature of such disruptions at the country level, we do not present any country-level estimates of counterfactual disruptions and present only the aggregate estimated COVID-19 pandemic effects at the super-regional and global levels.

Section 1.9 Tracking Progress towards IA2030 50% zero-dose reduction target

For each country, we calculated the annualized rate of change (AROC) in DTP1 coverage needed between 2023 and 2030 to achieve the IA2030 target. We first calculated numbers of zero-dose children in each country in 2019 as $(1 - DTP1\ coverage) * population\ under\ 1$ (table S7).[10] Dividing this product in half yielded country-specific zero-dose target counts for 2030, with 2030 DTP1 coverage targets calculated as $(2030\ forecasted\ population\ under\ 1 - zero - dose\ target)/forecasted\ 2030\ population\ under\ 1$.[11] We then compared these 2030 target percentages with 2023 country-specific DTP1 coverage estimates to yield percentage point change in DTP1 coverage needed between 2023 and 2030 in each country to achieve IA2030's zero-dose reduction targets.

To estimate the AROC required for individual countries and territories to achieve the IA2030 goal of 50% zero-dose child reduction, we used the following formula:

$$AROC = ln(DTP1_{2030} / DTP1_{2023}) / (2030 - 2023)$$

If future coverage resulted in a negative AROC for any country (due to coverage increases from 2019 to 2023 and/or declining populations of children under one year), that country was excluded from further analysis.

To contextualize DTP1 coverage change needed to achieve the IA2030 target, we calculated AROC values for all countries and territories for all seven-year windows (ie, 2000-2007, 2001-2008, etc) between 2000 and 2019.We then compared each country-specific AROC needed between 2023 and 2030 to the global distribution of these historical AROC values, as a percentile.

Section 1.10 Forecasting life-course vaccine coverage

Forecasted coverage of DTP3 was modelled in a simple logistic regression using the following formula:

$$logit(DTP3\ coverage) \sim SDI$$

The model was fit using historic estimates of DTP3 coverage and SDI, and forecasts for future years were predicted using forecasted estimates of SDI.[10,11] These constituted the most likely "reference scenario" vaccine forecasts. We also produced vaccine coverage forecasts for alternative "better" and "worse" scenarios. These were forecasted based on the 85th and 15th percentiles, respectively, of the distribution of past rates of change in coverage (in natural log space) between subsequent years across all countries and territories. The historic distributions were calculated by draw, pulling from across countries and year pairs 1980-2019, excluding years impacted by the COVID-19 pandemic. Rates of change from more recent years were weighted more heavily in the distribution compared to those from earlier in the time series, with rates of change from 1980–1981 receiving a weight of 1, 1981–1982 receiving a weight of 2, 1982–1983 receiving a weight of 3, etc.

Forecasts of PCV3/DTP3 and MCV2/MCV1 coverage ratios were produced using equivalent ratio modelling techniques as used in historical coverage estimation. For countries and territories where PCV3 or MCV2 had not yet been introduced, introduction dates beyond 2023 were simulated using a Weibull distribution with Gavi eligibility status, forecasted SDI and DTP3 or MCV1 coverage were included as covariates. The better and worse scenario introductions were set at the 15th and 85th percentiles of simulated introduction dates.

To forecast coverage of PCV3 and MCV2 following these simulated introductions, we modelled the logit of the forecasted ratio of coverage of these vaccines to coverage of the reference vaccine (DTP3 for PCV3, MCV1 for MCV2) as a linear mixed effects model:

$$logit(coverage\ ratio) = \beta_0 + \beta_1 \ln(LDI) + \beta_2 Cov_{ref} + \beta_3 YSI + (1|maternal\ education) + (1|country)$$

Where β_0 is the intercept, β_1 is the coefficient on natural-log-transformed forecasted lag-distributed income (*LDI*), β_2 is the coefficient on forecasted reference vaccine coverage (Cov_{ref}, ie, DTP3 or MCV1), and β_3 is the coefficient on years since introduction (*YSI*), with random intercepts on maternal education and country. Predicted coverage ratios were then multiplied by the DTP3 and MCV1 vaccine coverage respectively for each scenario to produce forecasts for PCV3 and MCV2 through 2030.

Section 2 Model validation and comparisons

Section 2.1 Goodness of fit

In-sample goodness of fit metrics (RMSA, MedAE, and median error, table S9) demonstrated that the models were generally well-calibrated, with relatively little bias (in-sample median error ranging from 0.0008 for the Hib3-DTP3 ratio to 0.0105 for Pol3 coverage) and accuracy (MedAE of 0.0964 for the MCV2-MCV1 ratio, and 0.0354 or less for all other vaccines; RMSE ranging from 0.0558 for the RotaC-DTP3 ratio to 0.279 for the MCV2-MCV1 ratio). We note that the ST-GPR-based modeling framework that is used to produce these estimates accounts for heterogeneous data that can be reflected as noise in the time series, and to leverage both country-reported data and survey data, which can be contradictory. As a result, by design, the model will not exactly fit each survey data point but rather will produce estimates that account for all of the available data and their underlying variance.

Compared to in-sample models, the out-of-sample predictions remained relatively unbiased, as reflected in low out-of-sample median error. As expected, the out-of-sample predictions tended to display decreased accuracy, as illustrated by increases in out-of-sample MedAE and RMSE compared to in-sample values, though in most cases the differences were relatively small (ie, 2-3 percentage point difference in MedAE). For both in- and out-of-sample models, median error tended to skew towards positive values (ie, slight underestimation compared to observed values) for the ST-GPR models of both direct coverage and ratios.

Of the newer vaccine models, models for vaccines usually given as part of combination products with the reference vaccine (eg, HepB3-DTP3, Hib3-DTP3, and RCV1-MCV1) tended to have lower in- and out-of-sample MedAE and median error. These results likely reflect the fact that coverage of these vaccines tends to be equivalent to that of the reference vaccine within several years after introduction, after multivalent combination vaccines have been fully introduced. Of vaccines not given as part of combination products with the corresponding reference vaccines,

RotaC-DTP3 and PCV3-DTP3 tended to perform better than MCV2-MCV1 across in- and out-of-sample validation metrics. This may reflect more underlying homogeneity in RotaC and PCV3 scale-up patterns, particularly across Gavi-supported countries in recent years, compared to MCV2. MCV2, by contrast, was first introduced in some few countries and territories very early in the modeling period, but between-country roll-out has been very long, with many locations only introducing MCV2 in the last ten years and some countries and territories still yet to introduce this vaccine-dose. As a result, many different trends in scale-up have been observed between countries, and there is more variation around the global trends for this vaccine compared to the others.

We also assessed these metrics for the MR-BRT scale-up models (table S10). In general, these first-stage models demonstrated somewhat larger MedAE and RMSE values compared to their ST-GPR counterparts, with in-sample MedAE ranging from 0.0005 for RCV1 to 0.2964 to RotaC. Out-of-sample predictions followed similar trends. These results support the approach used in this paper of using the scale-up models as the first stage in a subsequent ST-GPR model, as the additional ST-GPR modeling stage improved both in- and out-of-sample in the final results.

Section 2.2 Comparisons to GBD 2020

At global and super-regional levels, the vaccine coverage estimates presented in this study are largely in agreement with those published in the GBD 2020 study (for overlapping years, 1980-2019, (figures S11 & S12). For 2019, out of 2244 vaccine-country pairs, coverage was within two percentage points (pp) between the two versions for 1896 vaccine-country pairs (84·5%), within five pp for 2055 vaccine-country pairs (91·6%), and within 10 pp for 2164 vaccine-country pairs (96·4%). Differences between versions were derived from several points: firstly, 140 new data sources were included in the present study, including 88 with data from years in or before 2019 (ie, overlapping with the year range of the previous study). Methods changes for the direct modelling of DTP1, along with direct adjustments of bias in DTP1 country-reported data led to comparably larger changes in coverage for this vaccine, particularly with decreases in coverages earlier in the time series—in 1993, global DTP1 coverage diverged by a peak of 3·2%pp, driven largely by adjustments in estimates for India and change brought on there through the direct modelling of administrative bias for DTP1 country-reported data. Except where new data sources were added (table S2), estimates of DTP3, MCV1 and Pol3 remained largely unchanged. For newer vaccines (HepB3, Hib3, MCV2, PCV3, RCV1, RotaC), improvements to the process of modelling country-specific scale-up following introduction and the inclusion of new introduction year information (eg, for RCV1 in China) led to more country-level changes, but the effects of these changes varied by year given the varied timing of introductions across countries and vaccines. These updates most affected estimated coverage in the initial years of introduction between 1980 and 2019. In these initial introduction years, out of 1062 vaccine-country pairs, 74 vaccine-country estimates (7·0%) were more than five pp apart from each other between this study and the GBD 2020 study, and 51 vaccine-country pairs (4·8%) were more than 10 pp apart. In order to maintain consistency throughout the entire estimated time series, and given the improvements in data and methods described above, these results therefore are meant to supersede the results of the GBD 2020 analysis for all estimated vaccines and years.

Section 2.3 Comparisons to WUENIC

While not as closely aligned as estimates from the GBD 2020 and this GBD 2023 study of vaccine coverage, those from this study and from WUENIC are nevertheless in agreement overall (figures S11 & S13). In 2023, of the 1968 comparable vaccine-country pairs, 1667 (84·7%) were within two pp when comparing this study's mean coverage estimates with those from WUENIC, while 1817 (92·3%) were within five pp and 1912 (97·2%) were within 10 pp.

Drivers of differences between the GBD 2020 coverage estimates and those from WUENIC were discussed in the previous GBD 2020 report and are largely unchanged for those in this update. Both studies are based upon country-reported data and surveys, and all sources used by WUENIC were reviewed for inclusion in this study.[12] Differences between GBD and WUENIC estimates are largely due to several differences in modeling approaches. First, that the WUENIC rules-based approach prioritizes alignment with country-reported estimates, unless survey data is in marked disagreement with that data (ie, if coverage values from country-reported estimates are within a certain threshold of the survey-based data, the country-reported estimates are used). In our modelling framework, we use bias adjustments to align country-reported data with survey data prior to coverage modeling, regardless of the degree of discrepancy between the two sources. Country-reported data tend to be higher than survey data, though this

relationship is not universal; as a result, this difference in approach likely contributes to the general tendency of these estimates to be lower than WUENIC estimates. Second, WUENIC employs adjustments to survey data for third doses of select vaccines (DTP3, HepB3, Hib3, Pol3), in an effort to account for bias in the recall of parental survey respondents, with the assumption that recall for first doses will be more reliable than that for third doses, and assuming that the drop-out between first and third doses will be the same for individuals without vaccine cards as in those with cards. Given that previous studies have demonstrated wide variation in the direction and magnitude of this bias,[13] and given concern that true drop-out rates may be correlated with card retention, we do not include such an adjustment in this study – though we acknowledge this as a limitation of these estimates. This recall bias adjustment largely accounts for the observation that the differences between global WUENIC estimates for third-dose vaccine coverage estimates tend to be larger than for first-dose vaccine coverage estimates, again with WUENIC estimates generally higher than GBD estimates. Last, while our estimates of coverage largely agree in years affected by COVD-19 for vaccine-country-years with available country-reported data, we impute disruptions in those instances where data is not available to indicate disruption magnitudes. In such cases our estimates tend to be somewhat lower than those of WUENIC (though with high uncertainty).

Section 3 Supplemental references

1 World Health Organization. WHO Immunization data: Immunization data. https://immunizationdata.who.int/global (accessed Jan 30, 2025).

2 GBD 2021 Diseases and Injuries Collaborators. Global incidence, prevalence, years lived with disability (YLDs), disability-adjusted life-years (DALYs), and healthy life expectancy (HALE) for 371 diseases and injuries in 204 countries and territories and 811 subnational locations, 1990-2021: a systematic analysis for the Global Burden of Disease Study 2021. *Lancet Lond Engl* 2024; **403**: 2133–61.

3 GBD 2019 Healthcare Access and Quality Collaborators. Assessing performance of the Healthcare Access and Quality Index, overall and by select age groups, for 204 countries and territories, 1990-2019: a systematic analysis from the Global Burden of Disease Study 2019. *Lancet Glob Health* 2022; **10**: e1715–43.

4 Lim SS, Stein DB, Charrow A, Murray CJL. Tracking progress towards universal childhood immunisation and the impact of global initiatives: a systematic analysis of three-dose diphtheria, tetanus, and pertussis immunisation coverage. *Lancet* 2008; **372**: 2031–46.

5 Galles, N., Liu, P, Updike, R., *et al.* Measuring routine childhood vaccination coverage in 204 countries and territories, 1980-2019: a systematic analysis for the Global Burden of Disease 2019 Study. *Lancet 60392* 2021; published online Jan 19.

6 World Health Organization. WHO Immunization data: Vaccine supply and logistics. https://immunizationdata.who.int/global/wiise-detail-page/vaccine-supply-and-logistics (accessed Jan 30, 2025).

7 World Health Organization. Global childhood immunization levels stalled in 2023, leaving many without life-saving protection. 2024; published online July 15. https://www.who.int/news/item/15-07-2024-global-childhood-immunization-levels-stalled-in-2023-leaving-many-without-life-saving-protection (accessed Jan 30, 2025).

8 Millard SP. EnvStats: An R Package for Environmental Statistics, 2nd edn. New York: Springer, 2013.

9 Zheng P, Barber R, Sorensen RJD, Murray CJL, Aravkin AY. Trimmed constrained mixed effects models: formulations and algorithms. *J Comput Graph Stat* 2021; **30**: 544–56.

10 GBD 2021 Demographics Collaborators. Global age-sex-specific mortality, life expectancy, and population estimates in 204 countries and territories and 811 subnational locations, 1950-2021, and the impact of the COVID-19 pandemic: a comprehensive demographic analysis for the Global Burden of Disease Study 2021. *Lancet Lond Engl* 2024; **403**: 1989–2056.

11 GBD 2021 Forecasting Collaborators. Burden of disease scenarios for 204 countries and territories, 2022-2050: a forecasting analysis for the Global Burden of Disease Study 2021. *Lancet Lond Engl* 2024; **403**: 2204–56.

12 World Health Organization, UNICEF. WUENIC Trends. https://worldhealthorg.shinyapps.io/wuenic-trends/ (accessed April 4, 2025).

13 Dansereau E, Brown D, Stashko L, Danovaro-Holliday MC. A systematic review of the agreement of recall, home-based records, facility records, BCG scar, and serology for ascertaining vaccination status in low and middle-income countries. *Gates Open Res* 2019; **3**: 923.

14 Murray CJ, Ezzati M, Flaxman AD, *et al.* GBD 2010: design, definitions, and metrics. *The Lancet* 2012; **380**: 2063–6.

Section 4 List of supplemental tables and figures

Table S1: GATHER Compliance Checklist.

Table S2: Citation table. List of sources with GHDx identification number included in the final analysis. Vaccines with an "I" were included, vaccines with an "E" had all cohorts excluded, vaccines with an "e" had selected cohorts excluded, and vaccines with "-" were not present. Sources marked with an asterisk (*) next to the GHDx ID are newly added since the GBD 2020 paper. These sources can also be explored following this link to the GHDx input data sources tool: https://ghdx.healthdata.org/record/ihme-data/gbd-2023-vaccination-coverage-1980-2030. For rationale behind exclusions, please refer to Table S3. BCG=Bacillus Calmette-Guérin. DTP1=diphtheria-tetanus-pertussis, first dose. DTP3=diphtheria-tetanus-pertussis, third dose. HepB3=hepatitis B vaccine, third dose. Hib3=Haemophilus influenzae type b vaccine, third dose. MCV1=measles-containing vaccine, first dose. MCV2=measles-containing vaccine, second dose. PCV3=pneumococcal conjugate vaccine, third dose. Pol3=polio vaccine, third dose. RCV1=rubella-containing vaccine, first dose. RotaC=completed rotavirus series.

Table S3: Excluded sources. Citations, series, country, years, and antigens are provided for each excluded dataset along with rationale for their exclusion. BCG=Bacillus Calmette-Guérin. DTP1=diphtheria-tetanus-pertussis, first dose. Sources marked with an asterisk (*) next to the GHDx ID are excluded from administrative data bias modeling as a methodological decision to improve model performance. DTP3=diphtheria-tetanus-pertussis, third dose. HepB3=hepatitis B vaccine, third dose. Hib3=Haemophilus influenzae type b vaccine, third dose. MCV1=measles-containing vaccine, first dose. MCV2=measles-containing vaccine, second dose. PCV3=pneumococcal conjugate vaccine, third dose. Pol3=polio vaccine, third dose. RCV1=rubella-containing vaccine, first dose. RotaC=completed rotavirus series.

Table S4: Supplemental stockout and disruption events. WHO-reported stockout/supply disruption events in national and subnational units, disruption meaning vaccine-specific acute disruption to vaccine delivery. WHO=World Health Organisation.

Table S5: Super-regions and corresponding countries and territories. Groups of countries and territories identified for analysis and comparison on the bases of epidemiological homogeneity, and geographical contiguity in line with earlier GBD studies.[14]

Table S6: COVID-inclusive vs. COVID-free counterfactual coverage of EPI vaccines for the target population by super-region in 2023, as well as differences in coverage between these scenarios calculated by draw. COVID-free and COVID-inclusive pairs where the difference is within one percentage point of each other are in bold. BCG=Bacillus Calmette-Guérin. DTP1=diphtheria-tetanus-pertussis, first dose. DTP3=diphtheria-tetanus-pertussis, third dose. MCV1=measles-containing vaccine, first dose. Pol3=Pol vaccine, third dose.

Table S7: Zero-dose counts and Immunization Coverage for 2019 and 2023, with 2030 Projections. Zero-dose counts for 2019 and 2023, along with immunization coverage estimates for both, and projections for the 2030 population and coverage goal for each country. Zero-dose children are defined as those to have never received a routine childhood immunization of any kind, proxied as children under one year of age who have never received a single dose of DTP. DTP=diphtheria-tetanus-pertussis.

Table S8: Scale-up model global fit parameters. Parameters fit to global models of coverage scale-up for newer vaccine ratios, used as priors in country-level model fits. DTP3=diphtheria-tetanus-pertussis, third dose. HepB3=hepatitis B vaccine, third dose. Hib3=Haemophilus influenzae type b vaccine, third dose. MCV1=measles-containing vaccine, first dose. MCV2=measles-containing vaccine, second dose. PCV3=pneumococcal conjugate vaccine, third dose. RCV1=rubella-containing vaccine, first dose. RotaC=completed rotavirus series. YSI=years since introduction.

Table S9: Validation metrics for ST-GPR models. In-sample and out-of-sample (using 5-fold cross validation modelling) metrics assessing goodness of fit for vaccine coverage and coverage ratio models fit using ST-GPR. coverage and scale-up modelling global in-sample and out-of-sample metrics of fit. MedAE=median absolute error. MedErr=median error. RMSE=root mean square error. BCG=Bacillus Calmette-Guérin. DTP1=diphtheria-tetanus-pertussis, first dose. DTP3=diphtheria-tetanus-pertussis, third dose. HepB3=hepatitis B vaccine, third dose. Hib3=Haemophilus influenzae type b vaccine, third dose. MCV1=measles-containing vaccine, first dose. MCV2=measles-containing vaccine, second dose. PCV3=pneumococcal conjugate vaccine, third dose. RCV1=rubella-containing vaccine, first dose. RotaC=completed rotavirus series. ST-GPR= Spatiotemporal Gaussian Process Regression.

Table S10: Validation metrics for MR-BRT first stage models. In-sample and out-of-sample (using 5-fold cross validation modelling) metrics assessing goodness of fit for vaccine coverage ratio models fit as first stage models for ST-GPR where scale-up for newer vaccine ratios was fit using MR-BRT.Vaccine coverage and scale-up modelling global in-sample and out-of-sample metrics of fit. MedAE=median absolute error. MedErr=median error. RMSE=root mean square error. DTP3=diphtheria-tetanus-pertussis, third dose. HepB3=hepatitis B vaccine, third dose. Hib3=Haemophilus influenzae type b vaccine, third dose. MCV1=measles-containing vaccine, first dose. MCV2=measles-containing vaccine, second dose. PCV3=pneumococcal conjugate vaccine, third dose. RCV1=rubella-containing vaccine, first dose. RotaC=completed rotavirus series. MR-BRT= Meta Regression; Bayesian; Regularized; Trimmed.

Figure S1: Flow chart of modelling processes. Conceptual flow of GBD vaccine coverage modelling pipeline. White ovals refer to screened and processed vaccination coverage data, covariates are shown as blue cylinders, modelling and processing steps are shown in light peach rectangles, intermediate outputs are shown in green parallelograms, and final coverage outputs are represented with the blue parallelogram. Estimation begins with data preparation steps (upper light blue box) and ends with coverage modelling and post-processing (lower light blue box). JRF=Joint Reporting Form. SDI=Socio-demographic Index. ST-GPR=spatiotemporal Gaussian process regression. HAQ Index=Healthcare Access and Quality Index. MR-BRT=meta-regression—Bayesian, regularised, trimmed. GAM=generalize additive model. UI=uncertainty interval. BCG=Bacillus Calmette-Guérin. DTP1=diphtheria-tetanus-pertussis, first dose. DTP3=diphtheria-tetanus-pertussis, third dose. HepB3=hepatitis B vaccine, third dose. Hib3=Haemophilus influenzae type b vaccine, third dose. MCV1=measles-containing vaccine, first dose. MCV2=measles-containing vaccine, second dose. PCV3=pneumococcal conjugate vaccine, third dose. Pol3=polio vaccine, third dose. RCV1=rubella-containing vaccine, first dose. RotaC=completed rotavirus series.

Figure S2: Flow chart of data screening processes. Flow chart describing the processes for intake of raw survey and country reported data (blue squares) and screening processes (green ovals) to generate intermediate data (turquoise squares), to exclude data given listed criteria (red squares), and to ultimately derive final model input data (purple square). GHDx=Global Health Data Exchange. WUENIC=WHO-UNICEF Estimates of National Immunization Coverage. N= sample size.

Figure S3: Predicted fits of global scale-up models by newer vaccine ratio. These predictions of scale up of coverage ratios with 95% uncertainty intervals across years-since-introduction (YSI) were fit using meta-

regression—Bayesian, regularised, trimmed (MR-BRT) across a global dataset. The parameters fit in these global models served as priors for country-specific models where data was unavailable. DTP3=diphtheria-tetanus-pertussis, third dose. HepB3=hepatitis B vaccine, third dose. Hib3=Haemophilus influenzae type b vaccine, third dose. MCV1=measles-containing vaccine, first dose. MCV2=measles-containing vaccine, second dose. PCV3=pneumococcal conjugate vaccine, third dose. RCV1=rubella-containing vaccine, first dose. RotaC=completed rotavirus series.

Figure S4: Global disruption magnitudes by year. The global population-weighted mean aggregate percent difference, by vaccine or vaccine ratio, between estimates including and excluding disruptions by year and vaccine (ie, disruption magnitude). Blue bars indicate disruption magnitudes in years preceding COVID, compared to red bars indicating disruption magnitudes during the COVID-19 pandemic. Vaccine-country-draw means across years preceding the COVID-19 pandemic (ie, 2015–2019) were used to subsequently adjust disruption levels during the pandemic to account for disruptions that may have occurred absent the COVID-19 pandemic. BCG=Bacillus Calmette-Guérin. DTP1=diphtheria-tetanus-pertussis, first dose. DTP3=diphtheria-tetanus-pertussis, third dose. HepB3=hepatitis B vaccine, third dose. Hib3=Haemophilus influenzae type b vaccine, third dose. MCV1=measles-containing vaccine, first dose. MCV2=measles-containing vaccine, second dose. PCV3=pneumococcal conjugate vaccine, third dose. Pol3=polio vaccine, third dose. RCV1=rubella-containing vaccine, first dose. RotaC=completed rotavirus series.

Figure S5: Vaccine coverage time series. Country- and vaccine-specific estimates of coverage proportions for the target population, 1980-2023. Data used in the model are coloured in black. Data excluded from the model are coloured in light blue. Point shapes correspond to different input data types. The solid green line illustrates estimated annual coverage. 95% uncertainty intervals are represented by the shaded, light green band around each mean estimate. WUENIC estimates released July 2024 are shown in light blue. WUENIC=WHO-UNICEF Estimates of National Immunization Coverage. Original Country reported= raw, unadjusted official country-reported data from the Joint Reporting Form (JRF). GBD = Global Burden of Disease. Country-reported=official country-reported data from the JRF, after bias adjustment. DHS=Demographics and Health Survey. MICS=Multiple Indicator Cluster Survey. Other microdata= Non-DHS, non-MICS survey microdata. Other tabulation=survey report tabulation. Imputed=statistically imputed as described in appendix 1 section 1.2. BCG=Bacillus Calmette-Guérin. DTP1=diphtheria-tetanus-pertussis, first dose. DTP3=diphtheria-tetanus-pertussis, third dose. HepB3=hepatitis B vaccine, third dose. Hib3=Haemophilus influenzae type b vaccine, third dose. MCV1=measles-containing vaccine, first dose. MCV2=measles-containing vaccine, second dose. PCV3=pneumococcal conjugate vaccine, third dose. Pol3=polio vaccine, third dose. RCV1=rubella-containing vaccine, first dose. RotaC=completed rotavirus series.

Figure S6: Vaccine coverage maps. For all vaccines (BCG, DTP1, DTP3, HepB3, Hib3, MCV1, MCV2, PCV3, Pol3, RCV1, and RotaC), maps showing vaccine coverage for the target population for select years (1980, 1990, 2000, 2010, 2019, 2020, 2021, 2022, and 2023). Dark blue indicates high coverage, dark red indicates low-to-no coverage, light grey indicates the vaccine pre-introduction year, and dark grey indicates no data Insets magnify coverage in smaller countries and territories. BCG=Bacillus Calmette-Guérin. DTP1=diphtheria-tetanus-pertussis, first dose. DTP3=diphtheria-tetanus-pertussis, third dose. HepB3=hepatitis B vaccine, third dose. Hib3=Haemophilus influenzae type b vaccine, third dose. MCV1=measles-containing vaccine, first dose. MCV2=measles-containing vaccine, second dose. PCV3=pneumococcal conjugate vaccine, third dose. Pol3=polio vaccine, third dose.
RCV1=rubella-containing vaccine, first dose. RotaC=completed rotavirus series.

Figure S7: Impact of COVID-19 pandemic on vaccine coverage by super-region. Vaccine coverage aggregated by super-region for years 2015–2023, depicting mean coverage for the target population inclusive of COVID-19 pandemic-related disruptions (“COVID inclusive”) during years 2020-2023 (blue lines), as well as coverage in a “COVID free counterfactual” scenario absent disruptions related to COVID-19 (orange lines), as well as 95% uncertainty intervals for both. DTP1=diphtheria-tetanus-pertussis, first dose. DTP3=diphtheria-tetanus-pertussis, third dose. HepB3=hepatitis B vaccine, third dose. Hib3=Haemophilus influenzae type b vaccine, third dose. MCV1=measles-containing vaccine, first dose. MCV2=measles-containing vaccine, second dose. PCV3=pneumococcal conjugate vaccine, third dose. Pol3=polio vaccine, third dose. RCV1=rubella-containing vaccine, first dose. RotaC=completed rotavirus series.

Figure S8: Forecast vaccine coverage for years 2024-2030 by super-region. Historic vaccine coverage means for DTP3, MCV2 and PCV3 ("life course vaccines") for the target population by super-region for years 1980–2023, as well as mean forecasted vaccine coverage for years 2024–2030 under "reference" (black), "better" (green) and "worse" (pink) scenarios, as well as 95% uncertainty intervals for all scenarios. DTP3=diphtheria-tetanus-pertussis, third dose. MCV2=measles-containing vaccine, second dose. PCV3=pneumococcal conjugate vaccine, third dose.

Figure S9: Vaccine coverage in 2023 vs. forecast vaccine coverage in 2030 by super-region. Comparison of historic vaccine coverage in 2023 for DTP3, MCV2 and PCV3 for the target population compared to forecasted vaccine coverage in 2030 under the "worse", "reference" and "better" scenarios. Points represent individual countries and territories, coloured by super-region and sized according to population under one year of age, forecasted for the year 2030. DTP3=diphtheria-tetanus-pertussis, third dose. MCV2=measles-containing vaccine, second dose. PCV3=pneumococcal conjugate vaccine, third dose.

Figure S10: Product of simulated binomial and exponential distributions of the global percent difference between COVID inclusive and COVID-free counterfactual scenario vaccine coverage estimates by draw, for each vaccine or vaccine ratio modelled, by year, for years 2020–2023. These binomial and exponential distributions were simulated based on differences between coverage scenarios for vaccine-country-years where country-reported data was available for estimating disruption magnitudes, and these distributions were sampled from to apply imputed disruption estimates in country-vaccine-years with such country-reported data missing. BCG=Bacillus Calmette-Guérin. DTP1=diphtheria-tetanus-pertussis, first dose. DTP3=diphtheria-tetanus-pertussis, third dose. HepB3=hepatitis B vaccine, third dose. Hib3=Haemophilus influenzae type b vaccine, third dose. MCV1=measles-containing vaccine, first dose. MCV2=measles-containing vaccine, second dose. PCV3=pneumococcal conjugate vaccine, third dose. Pol3=polio vaccine, third dose. RCV1=rubella-containing vaccine, first dose. RotaC=completed rotavirus series.

Figure S11: Global vaccine coverage time series plots, GBD 2023 vs GBD 2020 vs. WUENIC. Global vaccine-specific estimates of coverage proportions for the target population, 1980–2023. The solid red line illustrates GBD2023 estimated annual coverage, and previously published GBD2020 estimates are shown in solid purple. 95% uncertainty intervals surround each GBD mean estimate. WUENIC estimates released July 2024 are shown in light blue. WUENIC=WHO-UNICEF Estimates of National Immunization Coverage. GBD = Global Burden of Disease. DTP1=diphtheria-tetanus-pertussis, first dose. DTP3=diphtheria-tetanus-pertussis, third dose. HepB3=hepatitis B vaccine, third dose. Hib3=Haemophilus influenzae type b vaccine, third dose. MCV1=measles-containing vaccine, first dose. MCV2=measles-containing vaccine, second dose. PCV3=pneumococcal conjugate vaccine, third dose. Pol3=polio vaccine, third dose. RCV1=rubella-containing vaccine, first dose. RotaC=completed rotavirus series.

Figure S12: Vaccine coverage in GBD2020 vs. vaccine coverage in GBD2023. Comparison of vaccine coverage estimates for the target population in 2019, comparing the GBD 2023 vs. GBD 2020 manuscripts. Each point represents one country-year of estimated coverage, by GBD super-region, sized by the population under one year of age. BCG=Bacillus Calmette-Guérin. DTP1=diphtheria-tetanus-pertussis, first dose. DTP3=diphtheria-tetanus-pertussis, third dose. HepB3=hepatitis B vaccine, third dose. Hib3=Haemophilus influenzae type b vaccine, third dose. MCV1=measles-containing vaccine, first dose. MCV2=measles-containing vaccine, second dose. PCV3=pneumococcal conjugate vaccine, third dose. Pol3=polio vaccine, third dose. RCV1=rubella-containing vaccine, first dose. RotaC=completed rotavirus series.

Figure S13: WUENIC vaccine coverage vs. GBD vaccine coverage in 2023. Comparison of historic vaccine coverage for the target population in 2023, comparing WUENIC estimates to GBD2023. Each point represents one country-year of mean estimated coverage, by GBD super-region, sized by the population under one year of age (comparison limited to 195 locations common to both GBD and WUENIC estimates). BCG=Bacillus Calmette-Guérin. DTP1=diphtheria-tetanus-pertussis, first dose. DTP3=diphtheria-tetanus-pertussis, third dose. HepB3=hepatitis B vaccine, third dose. Hib3=Haemophilus influenzae type b vaccine, third dose. MCV1=measles-containing vaccine, first dose. MCV2=measles-containing vaccine, second dose. PCV3=pneumococcal conjugate vaccine, third dose. Pol3=polio vaccine, third dose. RCV1=rubella-containing vaccine, first dose. RotaC=completed rotavirus series.

Section 5 Supplemental tables

Table S1 GATHER Compliance

Item #	Checklist item	Reporting location
**Objectives and funding**		
**1**	Define the indicator(s), populations (including age, sex, and geographic entities), and time period(s) for which estimates were made.	Main text (Methods); Appendix 1 (Supplemental Methods)
**2**	List the funding sources for the work.	Main text (primary funders given in the "funding" section of the Summary)
**Data Inputs**		
For all data inputs from multiple sources that are synthesized as part of the study:		
**3**	Describe how the data were identified and how the data were accessed.	Main text (Methods); Appendix 1 (Supplemental Methods)
**4**	Specify the inclusion and exclusion criteria. Identify all ad-hoc exclusions.	Main text (Methods); Appendix 1 (Supplemental Methods)
**5**	Provide information on all included data sources and their main characteristics. For each data source used, report reference information or contact name/institution, population represented, data collection method, year(s) of data collection, sex and age range, diagnostic criteria or measurement method, and sample size, as relevant.	Main text (Methods); Appendix 1 (Supplemental Methods); additional information about these sources available at https://ghdx.healthdata.org/record/ihme-data/gbd-2023-vaccination-coverage-1980-2030
**6**	Identify and describe any categories of input data that have potentially important biases (e.g., based on characteristics listed in item 5).	Main text (Methods); Appendix 1 (Supplemental Methods)
For data inputs that contribute to the analysis but were not synthesized as part of the study:		
**7**	Describe and give sources for any other data inputs.	Global Health Data Exchange https://ghdx.healthdata.org/record/ihme-data/gbd-2023-vaccination-coverage-1980-2030
For all data inputs:		
**8**	Provide all data inputs in a file format from which data can be efficiently extracted (e.g., a spreadsheet rather than a PDF), including all relevant meta-data listed in item 5. For any data inputs that cannot be shared because of ethical or legal reasons, such as third-party ownership, provide a contact name or the name of the institution that retains the right to the data.	Global Health Data Exchange https://ghdx.healthdata.org/record/ihme-data/gbd-2023-vaccination-coverage-1980-2030 . Availability of data is dependent on data use agreements; contact information provided when not directly available.
**Data analysis**		
**9**	Provide a conceptual overview of the data analysis method. A diagram may be helpful.	Main text (Methods); Appendix 1 (Supplemental Methods)
**10**	Provide a detailed description of all steps of the analysis, including mathematical formulae. This description should cover, as relevant, data cleaning, data pre-processing, data adjustments and weighting of data sources, and mathematical or statistical model(s).	Main text (Methods); Appendix 1 (Supplemental Methods)
**11**	Describe how candidate models were evaluated and how the final model(s) were selected.	N/A
**12**	Provide the results of an evaluation of model performance, if done, as well as the results of any relevant sensitivity analysis.	Appendix 1 section 2; Tables S9 & S10
**13**	Describe methods for calculating uncertainty of the estimates. State which sources of uncertainty were, and were not, accounted for in the uncertainty analysis.	Main text (Methods)
**14**	State how analytic or statistical source code used to generate estimates can be accessed.	Remote code repository https://ghdx.healthdata.org/record/ihme-data/gbd-2023-vaccination-coverage-1980-2030
**Results and Discussion**		
**15**	Provide published estimates in a file format from which data can be efficiently extracted.	Global Health Data Exchange https://ghdx.healthdata.org/record/ihme-data/gbd-2023-vaccination-coverage-1980-2030
**16**	Report a quantitative measure of the uncertainty of the estimates (e.g. uncertainty intervals).	Main text (Results); Appendix 1 Results UIs given in all results, including in figures (as appropriate) and tables
**17**	Interpret results in light of existing evidence. If updating a previous set of estimates, describe the reasons for changes in estimates.	Main text (Research in Context, Methods, Results and Discussion)
**18**	Discuss limitations of the estimates. Include a discussion of any modelling assumptions or data limitations that affect interpretation of the estimates.	Main text (Limitations section of Discussion)

Table S2 Citation table

List of sources with GHDx identification number included in the final analysis. Vaccines with an “I” were included, vaccines with an “E” had all cohorts excluded, vaccines with an “e” had selected cohorts excluded, and vaccines with “-” were not present. Sources marked with an asterisk (*) next to the GHDx ID are newly added since the GBD 2020 paper. These sources can also be explored following this link to the GHDx input data sources tool: http://ghdx.healthdata.org/record/ihme-data/we_need_new_address. For rationale behind exclusions, please refer to Table S3. BCG=Bacillus Calmette-Guérin. DTP1=diphtheria-tetanus-pertussis, first dose. DTP3=diphtheria-tetanus-pertussis, third dose. HepB3=hepatitis B vaccine, third dose. Hib3=Haemophilus influenzae type b vaccine, third dose. MCV1=measles-containing vaccine, first dose. MCV2=measles-containing vaccine, second dose. PCV3=pneumococcal conjugate vaccine, third dose. Pol3=polio vaccine, third dose. RCV1=rubella-containing vaccine, first dose. RotaC=completed rotavirus series.

GHDx ID	Country	Series	Year	BCG	DTP1	DTP3	HepB3	Hib3	MCV1	MCV2	PCV3	Pol3	RCV1	RotaC	Citation	Link
454354	Global	WHO and UNICEF Reported Official Target Population, Number of Doses Administered and Official Coverage 1966-2018	1966-2018	e	e	e	I	I	e	E	E	e	I	E	United Nations Children's Fund (UNICEF), World Health Organization (WHO). WHO and UNICEF Reported Official Target Population, Number of Doses Administered and Official Coverage 1966-2019. Geneva, Switzerland: World Health Organization (WHO), 2020.	GHDx
141905	Afghanistan	Afghanistan Multiple Indicator Baseline Survey 1997	1997	I	-	I	-	-	I	-	-	I	-	-	CIET International, United Nations Children's Fund (UNICEF). Afghanistan Multiple Indicator Baseline Survey 1997.	GHDx
561	Afghanistan	Afghanistan Multiple Indicator Cluster Survey 2003	2003	I	I	I	-	-	e	-	-	I	-	-	Central Statistics Organization (Afghanistan), United Nations Children's Fund (UNICEF). Afghanistan Multiple Indicator Cluster Survey 2003.	GHDx
18468	Afghanistan	Afghanistan Health Survey 2006	2006	e	-	-	-	-	I	-	-	I	-	-	Indian Institute of Health Management Research (IIHMR), Johns Hopkins University, Ministry of Public Health (Afghanistan). Afghanistan Health Survey 2006.	GHDx
280108	Afghanistan	Afghanistan Health Survey 2012	2012	I	-	I	I	I	I	-	-	I	-	-	Indian Institute of Health Management Research (IIHMR), Johns Hopkins University, Ministry of Public Health (Afghanistan). Afghanistan Health Survey 2012.	GHDx
279695	Afghanistan	Afghanistan National Immunization Coverage Survey 2013	2013	I	I	I	I	I	I	-	-	I	-	-	Central Statistics Organization (Afghanistan), Health Protection and Research Organisation (Afghanistan), Ministry of Public Health (Afghanistan), United Nations Children's Fund (UNICEF). Afghanistan National Immunization Coverage Survey 2013.	GHDx
338771	Afghanistan	Afghanistan Health Survey 2015	2015	I	-	I	I	I	I	-	-	I	-	-	Ministry of Public Health (Afghanistan), Royal Tropical Institute. Afghanistan Health Survey 2015.	GHDx

GHDx ID	Country	Series	Year	BCG	DTP1	DTP3	HepB3	Hib3	MCV1	MCV2	PCV3	Pol3	RCV1	RotaC	Citation	Link
462714*	Afghanistan	Afghanistan Health Survey 2018	2018	I	I	I	I	I	I	-	-	I	-	-	Ministry of Public Health (Afghanistan), Royal Tropical Institute. Afghanistan Health Survey 2018.	GHDx
108905	Afghanistan	Afghanistan National Risk and Vulnerability Assessment 2007-2008	2007-2008	I	-	I	-	-	I	-	-	I	-	-	Central Statistics Organization (Afghanistan), Ministry of Rural Rehabilitation and Development (Afghanistan). Afghanistan National Risk and Vulnerability Assessment 2007-2008.	GHDx
56830	Afghanistan	Afghanistan Multiple Indicator Cluster Survey 2010-2011	2010-2011	I	I	I	-	-	I	-	-	I	-	-	Central Statistics Organization (Afghanistan), United Nations Children's Fund (UNICEF). Afghanistan Multiple Indicator Cluster Survey 2010-2011. New York, United States of America: United Nations Children's Fund (UNICEF), 2013.	GHDx
157018	Afghanistan	Afghanistan Demographic and Health Survey 2015-2016	2015-2016	I	I	I	I	I	I	I	E	I	-	-	Central Statistics Organization (Afghanistan), ICF International, Ministry of Public Health (Afghanistan). Afghanistan Demographic and Health Survey 2015-2016. Fairfax, United States of America: ICF International, 2017.	GHDx
329519	Afghanistan	Progress towards poliomyelitis eradication: Afghanistan, January 2015–August 2016	2015-2016	-	-	-	-	-	-	-	-	I	-	-	World Health Organization. Progress towards poliomyelitis eradication: Afghanistan, January 2015–August 2016. WHO Wkly Epidemiol Rec. 2016; 91(44): 517-24.	GHDx
527453*	Afghanistan	Afghanistan Multiple Indicator Cluster Survey 2022-2023	2022-2023	e	e	e	E	E	e	E	E	e	-	E	National Statistic and Information Authority (NSIA) (Afghanistan), United Nations Children's Fund (UNICEF). Afghanistan Multiple Indicator Cluster Survey 2022-2023. New York, United States of America: United Nations Children's Fund (UNICEF), 2023.	GHDx
18834	Albania	Albania Demographic and Health Survey 2008-2009	2008-2009	I	I	I	I	-	I	-	-	I	-	-	ICF Macro, Institute of Public Health (Albania), Institute of Statistics (Albania). Albania Demographic and Health Survey 2008-2009. Fairfax, United States of America: ICF International, 2009.	GHDx
224847	Algeria	Algeria National Immunization Coverage Survey 1989	1989	I	I	I	-	-	I	-	-	I	-	-	Algeria National Immunization Coverage Survey 1989.	GHDx
634	Algeria	Algeria Maternal and Child Health Survey 1992	1992	I	I	I	-	-	I	-	-	I	-	-	National Office of Statistics (Algeria), League of Arab States. Algeria Maternal and Child Health Survey 1992.	GHDx

GHDx ID	Country	Series	Year	BCG	DTP1	DTP3	HepB3	Hib3	MCV1	MCV2	PCV3	Pol3	RCV1	RotaC	Citation	Link
26449	Algeria	Algeria Multiple Indicator Cluster Survey 2000	2000	I	e	e	-	-	I	-	-	I	-	-	Ministry of Health and Population (Algeria), National Institute of Public Health (Algeria), National Office of Statistics (Algeria), United Nations Children's Fund (UNICEF). Algeria Multiple Indicator Cluster Survey 2000.	GHDx
641	Algeria	Algeria Multiple Indicator Cluster Survey 2006	2006	I	I	I	-	-	I	-	-	I	-	-	Joint United Nations Program on HIV/AIDS (UNAIDS), Ministry of Health, Population and Hospital Reform (Algeria), National Office of Statistics (Algeria), United Nations Children's Fund (UNICEF), United Nations Population Fund (UNFPA). Algeria Multiple Indicator Cluster Survey 2006.	GHDx
627	Algeria	Algeria Family Health Survey 2002-2003	2002-2003	I	I	I	-	-	I	-	-	-	-	-	National Office of Statistics (Algeria), Ministry of Health, Population and Hospital Reform (Algeria), League of Arab States. Algeria Family Health Survey 2002-2003.	GHDx
210614	Algeria	Algeria Multiple Indicator Cluster Survey 2012-2013	2012-2013	I	I	I	I	I	I	-	-	I	-	-	Ministry of Health and Population (Algeria), United Nations Children's Fund (UNICEF). Algeria Multiple Indicator Cluster Survey 2012-2013. New York, United States of America: United Nations Children's Fund (UNICEF), 2018.	GHDx
464103*	Algeria	Algeria Multiple Indicator Cluster Survey 2018-2019	2018-2019	I	e	e	E	E	I	E	E	e	I	-	Ministry of Health and Population (Algeria), United Nations Children's Fund (UNICEF). Algeria Multiple Indicator Cluster Survey 2018-2019. New York, United States of America: United Nations Children's Fund (UNICEF), 2021.	GHDx
674	Angola	Angola Multiple Indicator Cluster Survey 1996	1996	I	-	I	-	-	I	-	-	I	-	-	Living Conditions Monitoring Office (Angola), National Statistics Office (Angola), United Nations Children's Fund (UNICEF). Angola Multiple Indicator Cluster Survey 1996. New York, United States of America: United Nations Children's Fund (UNICEF).	GHDx
687	Angola	Angola Multiple Indicator Cluster Survey 2001	2001	I	I	I	-	-	I	-	-	I	-	-	National Institute of Statistics (Angola), United Nations Children's Fund (UNICEF). Angola Multiple Indicator Cluster Survey 2001. New York, United States of America: United Nations Children's Fund (UNICEF).	GHDx

GHDx ID	Country	Series	Year	BCG	DTP1	DTP3	HepB3	Hib3	MCV1	MCV2	PCV3	Pol3	RCV1	RotaC	Citation	Link
30394	Angola	Angola Integrated Inquiry into People's Well-Being 2008-2009	2008-2009	I	I	I	E	E	I	-	-	I	-	-	National Institute of Statistics (Angola), Oxford Policy Management, United Nations Children's Fund (UNICEF). Angola Integrated Inquiry into People's Well-Being 2008-2009.	GHDx
218555	Angola	Angola Demographic and Health Survey 2015-2016	2015-2016	I	I	I	I	I	I	E	I	I	-	E	ICF International, Ministry of Health (Angola), National Institute of Statistics (Angola), United Nations Children's Fund (UNICEF). Angola Demographic and Health Survey 2015-2016. Fairfax, United States of America: ICF International, 2017.	GHDx
215019	Armenia	Armenia Evaluation of the National Immunization Program 1999	1999	I	-	I	-	-	I	-	-	I	-	-	Ministry of Health (Armenia), United Nations Children's Fund (UNICEF). Armenia Evaluation of the National Immunization Program 1999.	GHDx
18843	Armenia	Armenia Demographic and Health Survey 2000	2000	I	I	I	E	-	I	-	-	I	-	-	Ministry of Health (Armenia), National Statistical Service (NSS), ORC Macro. Armenia Demographic and Health Survey 2000. Fairfax, United States of America: ICF International.	GHDx
31750	Armenia	Armenia Demographic and Health Survey 2010	2010	I	I	I	I	E	I	-	-	I	-	-	ICF Macro, Ministry of Health (Armenia), National Statistical Service of the Republic of Armenia. Armenia Demographic and Health Survey 2010. Fairfax, United States of America: ICF International, 2015.	GHDx
218563	Armenia	Armenia Demographic and Health Survey 2015-2016	2015-2016	I	I	I	I	I	I	-	E	I	I	I	ICF International, Ministry of Health (Armenia), National Statistical Service of the Republic of Armenia. Armenia Demographic and Health Survey 2015-2016. Fairfax, United States of America: ICF International, 2017.	GHDx
85056	Australia	Australia National Health Survey 2001	2001	-	-	-	-	-	I	-	-	e	-	-	Australian Bureau of Statistics. Australia National Health Survey 2001. Canberra, Australia: Australian Bureau of Statistics.	GHDx
881	Azerbaijan	Azerbaijan Multiple Indicator Cluster Survey 2000	2000	I	I	I	-	-	I	-	-	I	-	-	State Statistics Committee of Azerbaijan, United Nations Children's Fund (UNICEF). Azerbaijan Multiple Indicator Cluster Survey 2000. New York, United States: United Nations Children's Fund (UNICEF).	GHDx
281740	Azerbaijan	Azerbaijan Maternal and Child Health Survey 2000	2000	I	-	I	-	-	-	-	-	I	-	-	Save the Children USA. Azerbaijan Maternal and Child Health Survey 2000.	GHDx

GHDx ID	Country	Series	Year	BCG	DTP1	DTP3	HepB3	Hib3	MCV1	MCV2	PCV3	Pol3	RCV1	RotaC	Citation	Link
18865	Azerbaijan	Azerbaijan Demographic and Health Survey 2006	2006	I	I	I	I	-	I	-	-	I	E	-	Macro International, Inc, State Statistical Committee of Azerbaijan. Azerbaijan Demographic and Health Survey 2006. Fairfax, United States of America: ICF International.	GHDx
222676	Azerbaijan	Azerbaijan Demographic and Health Survey 2011	2011	I	I	I	I	-	I	-	-	I	I	-	Public Health and Reforms Center, Ministry of Health (PHRC) (Azerbaijan). Azerbaijan Demographic and Health Survey 2011.	GHDx
215053	Bahrain	Bahrain Review of the EPI 1980	1980	-	I	I	-	-	I	-	-	I	-	-	Bahrain Review of the EPI 1980.	GHDx
215056	Bahrain	Bahrain Ministry of Health Reported Immunization Coverage Data, In Response to Draft Estimates, June 2001	1988	-	-	I	-	-	I	-	-	I	-	-	Ministry of Health (Bahrain). Bahrain Ministry of Health Reported Immunization Coverage Data, In Response to Draft Estimates, June 2001.	GHDx
905	Bahrain	Bahrain Child Health Survey 1989	1989	-	e	e	-	-	e	-	-	e	E	-	Central Statistics Organization (Bahrain), Ministry of Health (Bahrain). Bahrain Child Health Survey 1989.	GHDx
907	Bahrain	Bahrain Family Health Survey 1995	1995	-	I	I	-	-	I	-	-	I	-	-	Ministry of Health (Bahrain), Council of Health Ministers of GCC States. Bahrain Family Health Survey 1995. Manama, Bahrain: Ministry of Health (Bahrain).	GHDx
215038	Bangladesh	Bangladesh EPI Coverage Evaluation Survey 1992	1992	I	I	I	-	-	I	-	-	I	-	-	Directorate General of Health Services, Ministry of Health and Family Welfare (Bangladesh), United Nations Children's Fund (UNICEF), World Health Organization (WHO). Bangladesh EPI Coverage Evaluation Survey 1992.	GHDx
189502	Bangladesh	Bangladesh Multiple Indicator Cluster Survey 1995	1995	-	I	I	-	-	I	-	-	-	-	-	Bangladesh Bureau of Statistics (BBS), United Nations Children's Fund (UNICEF). Bangladesh Multiple Indicator Cluster Survey 1995.	GHDx
22129	Bangladesh	Bangladesh Multiple Indicator Cluster Survey 1996	1996	-	I	I	-	-	-	-	-	I	-	-	Bangladesh Bureau of Statistics (BBS), United Nations Children's Fund (UNICEF). Bangladesh Multiple Indicator Cluster Survey 1996.	GHDx
215044	Bangladesh	Achieving the Mid-Decade Goals for Children in Bangladesh 1996	1996	-	-	I	-	-	I	-	-	I	-	-	Bangladesh Bureau of Statistics (BBS), United Nations Children's Fund (UNICEF). Achieving the Mid-Decade Goals for Children in Bangladesh 1996.	GHDx

GHDx ID	Country	Series	Year	BCG	DTP1	DTP3	HepB3	Hib3	MCV1	MCV2	PCV3	Pol3	RCV1	RotaC	Citation	Link
215045	Bangladesh	Bangladesh EPI Coverage Evaluation Survey 1997	1997	I	-	I	-	-	I	-	-	I	-	-	Directorate General of Health Services, Ministry of Health and Family Welfare (Bangladesh), United Nations Children's Fund (UNICEF), World Health Organization (WHO). Bangladesh EPI Coverage Evaluation Survey 1997.	GHDx
215046	Bangladesh	Bangladesh EPI Coverage Evaluation Survey 1998	1998	I	-	I	-	-	I	-	-	I	-	-	Directorate General of Health Services, Ministry of Health and Family Welfare (Bangladesh), United Nations Children's Fund (UNICEF), World Health Organization (WHO). Bangladesh EPI Coverage Evaluation Survey 1998.	GHDx
215047	Bangladesh	Bangladesh EPI Coverage Evaluation Survey 2000	2000	I	I	I	-	-	I	-	-	I	-	-	Directorate General of Health Services, Ministry of Health and Family Welfare (Bangladesh), United Nations Children's Fund (UNICEF), World Health Organization (WHO). Bangladesh EPI Coverage Evaluation Survey 2000.	GHDx
215049	Bangladesh	Bangladesh EPI Coverage Evaluation Survey 2001	2001	I	I	I	-	-	I	-	-	I	-	-	Directorate General of Health Services, Ministry of Health and Family Welfare (Bangladesh), United Nations Children's Fund (UNICEF), World Health Organization (WHO). Bangladesh EPI Coverage Evaluation Survey 2001.	GHDx
215048	Bangladesh	Bangladesh EPI Coverage Evaluation Survey 2002	2002	I	I	I	-	-	I	-	-	I	-	-	Directorate General of Health Services, Ministry of Health and Family Welfare (Bangladesh), United Nations Children's Fund (UNICEF), World Health Organization (WHO). Bangladesh EPI Coverage Evaluation Survey 2002.	GHDx
215050	Bangladesh	Bangladesh EPI Coverage Evaluation Survey 2003	2003	I	I	I	-	-	I	-	-	I	-	-	Directorate General of Health Services, Ministry of Health and Family Welfare (Bangladesh), United Nations Children's Fund (UNICEF), World Health Organization (WHO). Bangladesh EPI Coverage Evaluation Survey 2003.	GHDx
18902	Bangladesh	Bangladesh Demographic and Health Survey 2004	2004	I	I	I	-	-	I	-	-	I	-	-	Mitra and Associates, ORC Macro. Bangladesh Demographic and Health Survey 2004. Fairfax, United States of America: ICF International.	GHDx
108614	Bangladesh	Bangladesh EPI Coverage Evaluation Survey 2005	2005	I	I	I	-	-	I	-	-	I	-	-	ACNielsen, Directorate General of Health Services, Ministry of Health and Family Welfare (Bangladesh), United Nations Children's Fund (UNICEF), World Health Organization (WHO). Bangladesh EPI Coverage Evaluation Survey 2005.	GHDx

GHDx ID	Country	Series	Year	BCG	DTP1	DTP3	HepB3	Hib3	MCV1	MCV2	PCV3	Pol3	RCV1	RotaC	Citation	Link
951	Bangladesh	Bangladesh Multiple Indicator Cluster Survey 2006	2006	I	I	I	I	-	I	-	-	I	-	-	Bangladesh Bureau of Statistics (BBS), Mitra and Associates, United Nations Children's Fund (UNICEF). Bangladesh Multiple Indicator Cluster Survey 2006. New York, United States of America: United Nations Children's Fund (UNICEF).	GHDx
107342	Bangladesh	Bangladesh EPI Coverage Evaluation Survey 2006	2006	I	I	I	-	-	I	-	-	I	-	-	ACNielsen Bangladesh, Expanded Program on Immunization (Bangladesh), United Nations Children's Fund (UNICEF), World Health Organization (WHO). Bangladesh EPI Coverage Evaluation Survey 2006.	GHDx
18913	Bangladesh	Bangladesh Demographic and Health Survey 2007	2007	I	I	I	I	-	I	-	-	I	-	-	Macro International, Inc, Mitra and Associates, National Institute of Population Research and Training (NIPORT). Bangladesh Demographic and Health Survey 2007. Fairfax, United States of America: ICF International, 2009.	GHDx
268016	Bangladesh	Bangladesh EPI Coverage Evaluation Survey 2011	2011	I	I	I	I	I	I	-	-	I	-	-	Directorate General of Health Services, Ministry of Health and Family Welfare (Bangladesh), Nielsen Company Bangladesh, United Nations Children's Fund (UNICEF), World Health Organization (WHO). Bangladesh EPI Coverage Evaluation Survey 2011.	GHDx
282828	Bangladesh	Bangladesh Health and Morbidity Status Survey 2012	2012	I	I	I	I	I	I	-	-	I	-	-	Bangladesh Bureau of Statistics (BBS), Ministry of Planning (Bangladesh). Bangladesh Health and Morbidity Status Survey 2012. Dhaka, Bangladesh: Bangladesh Bureau of Statistics (BBS).	GHDx
215052	Bangladesh	Bangladesh EPI Coverage Evaluation Survey 2013	2013	I	I	I	I	I	I	-	-	I	-	-	ACNielsen Bangladesh, Directorate General of Health Services, Ministry of Health and Family Welfare (Bangladesh), United Nations Children's Fund (UNICEF), World Health Organization (WHO). Bangladesh EPI Coverage Evaluation Survey 2013.	GHDx
275001	Bangladesh	Bangladesh Utilization of Essential Service Delivery Survey 2013	2013	I	I	I	I	I	I	-	-	I	-	-	Associates for Community and Population Research (ACPR), National Institute of Population Research and Training (NIPORT). Bangladesh Utilization of Essential Service Delivery Survey 2013. Dhaka, Bangladesh: National Institute of Population Research and Training (NIPORT).	GHDx

GHDx ID	Country	Series	Year	BCG	DTP1	DTP3	HepB3	Hib3	MCV1	MCV2	PCV3	Pol3	RCV1	RotaC	Citation	Link
157021	Bangladesh	Bangladesh Demographic and Health Survey 2014	2014	I	I	I	I	I	I	-	-	I	-	-	ICF International, Mitra and Associates, National Institute of Population Research and Training (NIPORT). Bangladesh Demographic and Health Survey 2014. Fairfax, United States of America: ICF International, 2015.	GHDx
482836*	Bangladesh	Bangladesh EPI Coverage Evaluation Survey 2019	2019	I	I	I	I	I	I	I	-	I	I	-	Center for Social and Market Research (CSMR) (Bangladesh), Directorate General of Health Services, Ministry of Health and Family Welfare (Bangladesh), United Nations Children's Fund (UNICEF), World Health Organization (WHO). Bangladesh EPI Coverage Evaluation Survey 2019.	GHDx
189500	Bangladesh	Bangladesh Progotir Pathay 1994: Progress Towards the Achievement of the Goals for the 1990's	1990-1994	-	I	I	-	-	I	-	-	-	-	-	Bangladesh Bureau of Statistics (BBS), United Nations Children's Fund (UNICEF). Bangladesh Progotir Pathay 1994: Progress Towards the Achievement of the Goals for the 1990's. Dhaka, Bangladesh: Bangladesh Bureau of Statistics (BBS), 1994.	GHDx
215037	Bangladesh	Bangladesh EPI Coverage Evaluation Survey 1991	1991-1992	I	I	I	-	-	I	-	-	-	-	-	Directorate General of Health Services, Ministry of Health and Family Welfare (Bangladesh), United Nations Children's Fund (UNICEF), World Health Organization (WHO). Bangladesh EPI Coverage Evaluation Survey 1991.	GHDx
18889	Bangladesh	Bangladesh Demographic and Health Survey 1993-1994	1993-1994	I	I	I	-	-	I	-	-	I	-	-	Macro International, Inc, Mitra and Associates, National Institute of Population Research and Training (NIPORT). Bangladesh Demographic and Health Survey 1993-1994. Fairfax, United States of America: ICF International.	GHDx
18878	Bangladesh	Bangladesh Demographic and Health Survey 1996-1997	1996-1997	I	I	I	-	-	I	-	-	I	-	-	Macro International, Inc, Mitra and Associates, National Institute of Population Research and Training (NIPORT). Bangladesh Demographic and Health Survey 1996-1997. Fairfax, United States of America: ICF International.	GHDx
26826	Bangladesh	Bangladesh Demographic and Health Survey 1999-2000	1999-2000	I	I	I	-	-	I	-	-	I	-	-	Macro Systems, Inc, Mitra and Associates, National Institute of Population Research and Training (NIPORT). Bangladesh Demographic and Health Survey 1999-2000. Fairfax, United States of America: ICF International.	GHDx

GHDx ID	Country	Series	Year	BCG	DTP1	DTP3	HepB3	Hib3	MCV1	MCV2	PCV3	Pol3	RCV1	RotaC	Citation	Link
215051	Bangladesh	Bangladesh EPI Coverage Evaluation Survey 2007-2008	2007-2008	I	I	I	I	E	I	-	-	I	-	-	ACNielsen Bangladesh, Directorate General of Health Services, Ministry of Health and Family Welfare (Bangladesh), United Nations Children's Fund (UNICEF), World Health Organization (WHO). Bangladesh EPI Coverage Evaluation Survey 2007-2008.	GHDx
55956	Bangladesh	Bangladesh Demographic and Health Survey 2011-2012	2011-2012	I	I	I	I	E	I	-	-	I	-	-	ICF Macro, Mitra and Associates, National Institute of Population Research and Training (NIPORT). Bangladesh Demographic and Health Survey 2011-2012. Calverton, United States of America: ICF Macro.	GHDx
317284	Bangladesh	Bangladesh EPI Coverage Evaluation Survey 2015-2016	2015-2016	I	I	I	I	I	I	-	-	I	I	-	Center for Social and Market Research (CSMR) (Bangladesh), Directorate General of Health Services, Ministry of Health and Family Welfare (Bangladesh), United Nations Children's Fund (UNICEF), World Health Organization (WHO). Bangladesh EPI Coverage Evaluation Survey 2015-2016.	GHDx
446877	Bangladesh	Bangladesh Demographic and Health Survey 2017-2018	2017-2018	I	I	I	I	I	I	I	I	I	-	-	ICF International, Mitra and Associates, National Institute of Population Research and Training (NIPORT). Bangladesh Demographic and Health Survey 2017-2018. Fairfax, United States of America: ICF International, 2020.	GHDx
1039	Belarus	Belarus Multiple Indicator Cluster Survey 2005	2005	e	e	e	E	-	I	-	-	e	-	-	Ministry of Statistics and Analysis of the Republic of Belarus, United Nations Children's Fund (UNICEF). Belarus Multiple Indicator Cluster Survey 2005. New York, United States of America: United Nations Children's Fund (UNICEF).	GHDx
501884*	Belgium	Belgium - Brussels-Capital Region Vaccination Coverage Survey of Children Aged 18 to 24 Months 2019-2020	2019-2020	-	I	I	I	I	I	-	I	I	I	-	Free University of Brussels (ULB). Belgium - Brussels-Capital Region Vaccination Coverage Survey of Children Aged 18 to 24 Months 2019-2020.	GHDx
1089	Belize	Belize Multiple Indicator Cluster Survey 2006	2006	I	I	I	I	I	I	-	-	I	-	-	Statistical Institute of Belize, United Nations Children's Fund (UNICEF). Belize Multiple Indicator Cluster Survey 2006. New York, United States of America: United Nations Children's Fund (UNICEF).	GHDx

GHDx ID	Country	Series	Year	BCG	DTP1	DTP3	HepB3	Hib3	MCV1	MCV2	PCV3	Pol3	RCV1	RotaC	Citation	Link
76699	Belize	Belize Multiple Indicator Cluster Survey 2011	2011	I	I	I	I	I	I	-	-	I	-	-	Statistical Institute of Belize, United Nations Children's Fund (UNICEF). Belize Multiple Indicator Cluster Survey 2011. New York, United States of America: United Nations Children's Fund (UNICEF), 2013.	GHDx
215057	Belize	From Girls to Women: Growing up Healthy in Belize	1994-1995	I	-	I	-	-	I	-	-	I	-	-	Government of Belize. From Girls to Women: Growing up Healthy in Belize. Belmopan, Belize: Government of Belize, 1997.	GHDx
264910	Belize	Belize Multiple Indicator Cluster Survey 2015-2016	2015-2016	I	I	I	I	I	I	E	-	I	-	-	Government of Belize, Statistical Institute of Belize, UN Resident Coordinator Fund (UN ResCor), United Nations Children's Fund (UNICEF), United Nations Development Programme (UNDP). Belize Multiple Indicator Cluster Survey 2015-2016. New York, United States of America: United Nations Children's Fund (UNICEF), 2018.	GHDx
215030	Benin	Benin National Immunization Coverage Survey 1995	1995	I	I	I	-	-	I	-	-	I	-	-	Benin National Immunization Coverage Survey 1995.	GHDx
18938	Benin	Benin Demographic and Health Survey 1996	1996	I	I	I	-	-	I	-	-	I	-	-	Macro International, Inc, National Institute of Statistics and Economic Analysis (INSAE) (Benin). Benin Demographic and Health Survey 1996. Fairfax, United States of America: ICF International.	GHDx
215029	Benin	Benin EPI Evaluation of Immunisation Coverage and Social Mobilisation 1991	1998	I	-	I	-	-	I	-	-	-	-	-	Benin EPI Evaluation of Immunisation Coverage and Social Mobilisation 1991.	GHDx
18950	Benin	Benin Demographic and Health Survey 2001	2001	I	I	I	-	-	I	-	-	I	-	-	National Institute of Statistics and Economic Analysis (INSAE) (Benin), ORC Macro. Benin Demographic and Health Survey 2001. Fairfax, United States of America: ICF International.	GHDx
215031	Benin	Benin External EPI Review 2001	2001	I	I	I	-	-	I	-	-	I	-	-	Benin External EPI Review 2001.	GHDx
18959	Benin	Benin Demographic and Health Survey 2006	2006	I	I	I	I	E	I	-	-	I	-	-	Macro International, Inc, National Institute of Statistics and Economic Analysis (INSAE) (Benin), National Program Against AIDS (PNLS) (Benin). Benin Demographic and Health Survey 2006. Fairfax, United States of America: ICF International.	GHDx

GHDx ID	Country	Series	Year	BCG	DTP1	DTP3	HepB3	Hib3	MCV1	MCV2	PCV3	Pol3	RCV1	RotaC	Citation	Link
215032	Benin	Benin External Review of the Extended Programme on Immunisation 2008	2008	I	I	I	I	I	I	-	-	I	-	-	Benin External Review of the Extended Programme on Immunisation 2008.	GHDx
206075	Benin	Benin Multiple Indicator Cluster Survey 2014	2014	I	I	I	I	I	I	-	I	I	-	-	National Institute of Statistics and Economic Analysis (INSAE) (Benin), United Nations Children's Fund (UNICEF). Benin Multiple Indicator Cluster Survey 2014. New York, United States of America: United Nations Children's Fund (UNICEF), 2017.	GHDx
558939*	Benin	Benin National Vaccination Coverage Survey 2023	2023	I	I	I	I	I	I	-	I	I	-	I	Benin National Vaccination Coverage Survey 2023.	GHDx
218565	Benin	Benin Demographic and Health Survey 2017-2018	2017-2018	I	I	I	I	I	I	-	I	I	-	-	Hubert Koutoukou Maga National University Hospital Center (CNHU-HKM)(Benin), ICF International, National Institute of Statistics and Economic Analysis (INSAE) (Benin), National Malaria Control Program, Ministry of Health (Benin), Permanent Secretariat of the Food Council and Nutrition (SP-CAN)(Benin). Benin Demographic and Health Survey 2017-2018. Fairfax, United States of America: ICF International, 2018.	GHDx
539788*	Benin	Benin Multiple Indicator Cluster Survey 2021-2022	2021-2022	I	I	I	I	I	I	-	I	I	-	I	National Institute of Statistics and Demography (INStaD) (Benin), United Nations Children's Fund (UNICEF). Benin Multiple Indicator Cluster Survey 2021-2022. New York, United States of America: United Nations Children's Fund (UNICEF), 2023.	GHDx
165290	Bhutan	Bhutan Health Survey 2012-2013	2012-2013	I	I	I	-	I	I	-	-	I	I	-	Ministry of Health (Bhutan), National Statistics Bureau (Bhutan), United Nations Population Fund (UNFPA). Bhutan Health Survey 2012-2013.	GHDx
215060	Bolivia	Bolivia National Immunization Coverage Survey 1987	1987	I	-	I	-	-	I	-	-	I	-	-	Bolivia National Immunization Coverage Survey 1987.	GHDx
18979	Bolivia	Bolivia Demographic and Health Survey 1989	1989	I	I	I	-	-	I	-	-	I	-	-	Macro Systems, Inc.; Institute for Resource Development, National Institute of Statistics (Bolivia). Bolivia Demographic and Health Survey 1989. Fairfax, United States of America: ICF International.	GHDx
215061	Bolivia	Bolivia National Immunization Coverage Survey 1990	1990	I	-	I	-	-	I	-	-	I	-	-	Bolivia National Immunization Coverage Survey 1990.	GHDx

GHDx ID	Country	Series	Year	BCG	DTP1	DTP3	HepB3	Hib3	MCV1	MCV2	PCV3	Pol3	RCV1	RotaC	Citation	Link
18971	Bolivia	Bolivia Demographic and Health Survey 1998	1998	I	I	I	-	-	I	-	-	I	-	-	Macro International, Inc, National Institute of Statistics (Bolivia). Bolivia Demographic and Health Survey 1998. Fairfax, United States of America: ICF International.	GHDx
1245	Bolivia	Bolivia Household Survey 2000	2000	-	I	I	-	-	-	-	-	-	-	-	National Institute of Statistics (Bolivia), World Bank (WB), Inter-American Development Bank (IDB), United Nations Economic Commission for Latin America and the Caribbean (CEPAL). Bolivia Household Survey 2000. La Paz, Bolivia: National Institute of Statistics (Bolivia).	GHDx
1289	Bolivia	Bolivia Multiple Indicator Cluster Survey 2000	2000	I	I	I	-	-	I	-	-	I	-	-	Population Development and Environment (PODEMA), National Directorate of Epidemiology (Bolivia), United Nations Children's Fund (UNICEF). Bolivia Multiple Indicator Cluster Survey 2000. New York, United States: United Nations Children's Fund (UNICEF).	GHDx
1259	Bolivia	Bolivia Household Survey 2001	2001	-	I	I	-	-	-	-	-	-	-	-	National Institute of Statistics (Bolivia), World Bank (WB), Inter-American Development Bank (IDB), United Nations Economic Commission for Latin America and the Caribbean (CEPAL). Bolivia Household Survey 2001. La Paz, Bolivia: National Institute of Statistics (Bolivia).	GHDx
1278	Bolivia	Bolivia Household Survey 2002	2002	-	e	e	-	-	-	-	-	-	-	-	National Institute of Statistics (Bolivia), World Bank (WB). Bolivia Household Survey 2002. La Paz, Bolivia: National Institute of Statistics (Bolivia).	GHDx
215062	Bolivia	Bolivia Survey of Coverage by MR (15-39 Years) and MMR (Children 12-23 Months) 2006	2006	-	-	-	-	-	I	-	-	-	-	-	Bolivia Survey of Coverage by MR (15-39 Years) and MMR (Children 12-23 Months) 2006.	GHDx
148344	Bolivia	Bolivia Household Survey 2007	2007	-	I	I	I	I	-	-	-	I	-	-	National Institute of Statistics (Bolivia). Bolivia Household Survey 2007. La Paz, Bolivia: National Institute of Statistics (Bolivia).	GHDx
19016	Bolivia	Bolivia Demographic and Health Survey 2008	2008	I	I	I	I	I	I	-	-	I	-	-	Macro International, Inc, Ministry of Health and Sports (Bolivia), National Institute of Statistics (Bolivia). Bolivia Demographic and Health Survey 2008. Fairfax, United States of America: ICF International.	GHDx

GHDx ID	Country	Series	Year	BCG	DTP1	DTP3	HepB3	Hib3	MCV1	MCV2	PCV3	Pol3	RCV1	RotaC	Citation	Link
148345	Bolivia	Bolivia Household Survey 2008	2008	-	I	I	I	I	-	-	-	I	-	-	National Institute of Statistics (Bolivia). Bolivia Household Survey 2008. La Paz, Bolivia: National Institute of Statistics (Bolivia).	GHDx
148346	Bolivia	Bolivia Household Survey 2009	2009	-	I	I	I	I	-	-	-	I	-	-	National Institute of Statistics (Bolivia). Bolivia Household Survey 2009. La Paz, Bolivia: National Institute of Statistics (Bolivia).	GHDx
164634	Bolivia	Bolivia Household Survey 2011	2011	-	I	I	I	I	-	-	-	I	-	-	National Institute of Statistics (Bolivia). Bolivia Household Survey 2011. La Paz, Bolivia: National Institute of Statistics (Bolivia).	GHDx
323944	Bolivia	Bolivia Demographic and Health Survey 2016	2017	I	I	I	I	I	I	-	E	I	I	I	Ministry of Health (Bolivia), National Institute of Statistics (Bolivia). Bolivia Demographic and Health Survey 2016. La Paz, Bolivia: National Institute of Statistics (Bolivia), 2017.	GHDx
453324*	Bolivia	Bolivia National Immunization Coverage Survey 2019	2019	I	-	I	I	I	-	-	-	I	-	-	Ministry of Health (Bolivia). Bolivia National Immunization Coverage Survey 2019.	GHDx
18990	Bolivia	Bolivia Demographic and Health Survey 1993-1994	1993-1994	I	I	I	-	-	I	-	-	I	-	-	Macro International, Inc, National Institute of Statistics (Bolivia). Bolivia Demographic and Health Survey 1993-1994. Fairfax, United States of America: ICF International.	GHDx
19001	Bolivia	Bolivia Demographic and Health Survey 2003-2004	2003-2004	I	I	I	-	-	I	-	-	I	-	-	Macro International, Inc, Ministry of Health and Sports (Bolivia), National Institute of Statistics (Bolivia). Bolivia Demographic and Health Survey 2003-2004. Fairfax, United States of America: ICF International.	GHDx
32374	Bolivia	Bolivia Household Survey 2003-2004	2003-2004	-	I	I	-	-	-	-	-	I	-	-	National Institute of Statistics (Bolivia). Bolivia Household Survey 2003-2004. La Paz, Bolivia: National Institute of Statistics (Bolivia).	GHDx
26989	Bosnia and Herzegovina	Bosnia and Herzegovina Multiple Indicator Cluster Survey 2000	2000	I	I	I	-	-	I	-	-	I	-	-	Agency for Statistics (Bosnia and Herzegovina), Ministry of Health (Federation of Bosnia and Herzegovina), Ministry of Health and Social Welfare (Republic of Srpska), United Nations Children's Fund (UNICEF). Bosnia and Herzegovina Multiple Indicator Cluster Survey 2000. New York, United States of America: United Nations Children's Fund (UNICEF).	GHDx

GHDx ID	Country	Series	Year	BCG	DTP1	DTP3	HepB3	Hib3	MCV1	MCV2	PCV3	Pol3	RCV1	RotaC	Citation	Link
1385	Bosnia and Herzegovina	Bosnia and Herzegovina Multiple Indicator Cluster Survey 2006	2006	I	I	I	I	-	I	-	-	I	I	-	Directorate for Economic Planning (Bosnia and Herzegovina), Federal Office of Statistics (Federation of Bosnia and Herzegovina), Institute of Statistics (Republic of Srpska), Ministry of Health (Federation of Bosnia and Herzegovina), Ministry of Health and Social Welfare (Republic of Srpska), Public Health Institute of Federation of Bosnia and Herzegovina, United Nations Children's Fund (UNICEF). Bosnia and Herzegovina Multiple Indicator Cluster Survey 2006. New York, United States of America: United Nations Children's Fund (UNICEF).	GHDx
80731	Bosnia and Herzegovina	Bosnia and Herzegovina Multiple Indicator Cluster Survey 2011-2012	2011-2012	I	I	I	I	-	I	-	-	I	I	-	Agency for Statistics (Bosnia and Herzegovina), Federal Ministry of Health (Bosnia and Herzegovina), Ministry of Health and Social Welfare (Republic of Srpska), Public Health Institute of Federation of Bosnia and Herzegovina, United Nations Children's Fund (UNICEF), United Nations Entity for Gender Equality and the Empowerment of Women (UN Women). Bosnia and Herzegovina Multiple Indicator Cluster Survey 2011-2012.	GHDx
19019	Botswana	Botswana Demographic and Health Survey 1988	1988	I	I	I	-	-	I	-	-	I	-	-	Central Statistics Office (Botswana), Macro Systems, Inc.; Institute for Resource Development, Ministry of Health (Botswana). Botswana Demographic and Health Survey 1988. Fairfax, United States of America: ICF International.	GHDx
215181	Botswana	Botswana EPI Evaluation 1990	1990	I	I	I	-	-	I	-	-	I	-	-	World Health Organization (WHO). Botswana EPI Evaluation 1990.	GHDx
1404	Botswana	Botswana Multiple Indicator Cluster Survey 2000	2000	I	I	I	-	-	I	-	-	I	-	-	Central Statistics Office (Botswana), United Nations Children's Fund (UNICEF). Botswana Multiple Indicator Cluster Survey 2000. New York, United States of America: United Nations Children's Fund (UNICEF), 2015.	GHDx
215182	Botswana	Botswana EPI Coverage Survey 2007	2007	I	I	I	I	-	I	-	-	I	-	-	Ministry of Health (Botswana). Botswana EPI Coverage Survey 2007.	GHDx
215179	Botswana	Botswana EPI Evaluation 1987	1980-1987	I	I	I	-	-	I	-	-	I	-	-	Botswana EPI Evaluation 1987.	GHDx

GHDx ID	Country	Series	Year	BCG	DTP1	DTP3	HepB3	Hib3	MCV1	MCV2	PCV3	Pol3	RCV1	RotaC	Citation	Link
22125	Botswana	Botswana Family Health Survey 2007-2008	2007-2008	I	I	I	I	-	I	-	-	I	-	-	Central Statistics Office (Botswana). Botswana Family Health Survey 2007-2008. Gaborone, Botswana: Central Statistics Office (Botswana), 2009.	GHDx
151536	Botswana	Botswana Core Welfare Indicators Survey 2009-2010	2009-2010	-	I	I	I	-	I	-	-	I	-	-	Central Statistics Office (Botswana). Botswana Core Welfare Indicators Survey 2009-2010.	GHDx
19027	Brazil	Brazil Demographic and Health Survey 1986	1986	e	e	e	-	-	e	E	-	e	-	-	Brazilian Society for Family Welfare (BEMFAM), Westinghouse; Institute for Resource Development. Brazil Demographic and Health Survey 1986. Fairfax, United States of America: ICF International.	GHDx
19035	Brazil	Brazil Demographic and Health Survey 1991	1991	e	e	e	-	-	e	-	-	e	-	-	Brazilian Society for Family Welfare (BEMFAM), Macro International, Inc. Brazil Demographic and Health Survey 1991. Fairfax, United States of America: ICF International.	GHDx
19046	Brazil	Brazil Demographic and Health Survey 1996	1996	e	e	e	-	-	e	-	-	e	-	-	Brazilian Society for Family Welfare (BEMFAM), Macro International, Inc. Brazil Demographic and Health Survey 1996. Fairfax, United States of America: ICF International.	GHDx
1921	Burkina Faso	Burkina Faso Multiple Indicator Cluster Survey 1996	1996	I	-	I	-	-	I	-	-	I	-	-	Ministry of Communication and Culture (Burkina Faso), Ministry of Economy and Finance (Burkina Faso), Ministry of Health (Burkina Faso), National Institute of Statistics and Demography (Burkina Faso), United Nations Children's Fund (UNICEF). Burkina Faso Multiple Indicator Cluster Survey 1996.	GHDx
19088	Burkina Faso	Burkina Faso Demographic and Health Survey 2003	2003	I	I	I	-	-	I	-	-	I	-	-	Macro International, Inc, National Institute of Statistics and Demography (Burkina Faso). Burkina Faso Demographic and Health Survey 2003. Fairfax, United States of America: ICF International.	GHDx
215033	Burkina Faso	Burkina Faso EPI Review 2003	2003	I	I	I	-	-	I	-	-	I	-	-	Burkina Faso EPI Review 2003.	GHDx
1927	Burkina Faso	Burkina Faso Multiple Indicator Cluster Survey 2006	2006	I	I	I	-	-	I	-	-	I	-	-	National Institute of Statistics and Demography (Burkina Faso), United Nations Children's Fund (UNICEF). Burkina Faso Multiple Indicator Cluster Survey 2006. New York, United States of America: United Nations Children's Fund (UNICEF).	GHDx
26642	Burkina Faso	Burkina Faso Global Fund Household Health Coverage Survey 2008	2008	I	I	I	-	-	I	-	-	I	-	-	Global Fund to Fight Aids Tuberculosis and Malaria (GFATM). Burkina Faso Global Fund Household Health Coverage Survey 2008.	GHDx

GHDx ID	Country	Series	Year	BCG	DTP1	DTP3	HepB3	Hib3	MCV1	MCV2	PCV3	Pol3	RCV1	RotaC	Citation	Link
215034	Burkina Faso	Burkina Faso EPI Review 2009	2009	I	I	I	-	-	I	-	-	I	-	-	Burkina Faso EPI Review 2009.	GHDx
236156	Burkina Faso	Burkina Faso Continuous Multisectoral Survey 2014	2014	I	I	I	-	-	I	-	-	I	-	-	National Institute of Statistics and Demography (Burkina Faso), World Bank. Burkina Faso Continuous Multisectoral Survey 2014. Washington DC, United States of America: World Bank.	GHDx
482842*	Burkina Faso	Burkina Faso Routine Vaccine Coverage Survey Coupled with the November 2019 Measles and Rubella (RR) Post-Campaign Coverage Survey 2020	2020	I	I	I	I	I	-	I	I	I	I	-	Burkina Faso Routine Vaccine Coverage Survey Coupled with the November 2019 Measles and Rubella (RR) Post-Campaign Coverage Survey 2020.	GHDx
527622*	Burkina Faso	Burkina Faso Demographic and Health Survey 2021	2021	I	I	I	I	I	I	I	I	I	-	I	ICF International, National Institute of Public Health (INSP) (Burkina Faso), National Institute of Statistics and Demography (Burkina Faso). Burkina Faso Demographic and Health Survey 2021.	GHDx
19064	Burkina Faso	Burkina Faso Demographic and Health Survey 1992-1993	1992-1993	I	I	I	-	-	I	-	-	I	-	-	Macro International, Inc, National Institute of Statistics and Demography (Burkina Faso). Burkina Faso Demographic and Health Survey 1992-1993. Fairfax, United States of America: ICF International.	GHDx
19076	Burkina Faso	Burkina Faso Demographic and Health Survey 1998-1999	1998-1999	I	I	I	-	-	I	-	-	I	-	-	Macro International, Inc, National Institute of Statistics and Demography (Burkina Faso). Burkina Faso Demographic and Health Survey 1998-1999. Fairfax, United States of America: ICF International.	GHDx
19133	Burkina Faso	Burkina Faso Demographic and Health Survey 2010-2011	2010-2011	I	I	I	I	I	I	-	-	I	-	-	ICF Macro, Ministry of Health (Burkina Faso), National Institute of Statistics and Demography (Burkina Faso). Burkina Faso Demographic and Health Survey 2010-2011. Fairfax, United States of America: ICF International.	GHDx
19142	Burundi	Burundi Demographic and Health Survey 1987	1987	I	I	I	-	-	I	-	-	I	-	-	Population Department, Ministry of the Interior (Burundi), Westinghouse; Institute for Resource Development. Burundi Demographic and Health Survey 1987. Columbia, United States: Westinghouse; Institute for Resource Development.	GHDx
215026	Burundi	Burundi Immunization Coverage Survey for Children 12-23 Months 1989	1989	I	I	I	-	-	I	-	-	I	-	-	Burundi Immunization Coverage Survey for Children 12-23 Months 1989.	GHDx

GHDx ID	Country	Series	Year	BCG	DTP1	DTP3	HepB3	Hib3	MCV1	MCV2	PCV3	Pol3	RCV1	RotaC	Citation	Link
189141	Burundi	Burundi Multiple Indicator Cluster Survey 1996	1996	I	-	I	-	-	I	-	-	I	-	-	Burundi Institute of Statistics and Economic Studies, United Nations Children's Fund (UNICEF). Burundi Multiple Indicator Cluster Survey 1996.	GHDx
1994	Burundi	Burundi Multiple Indicator Cluster Survey 2000	2000	I	I	I	-	-	I	-	-	I	-	-	Burundi Institute of Statistics and Economic Studies, United Nations Children's Fund (UNICEF). Burundi Multiple Indicator Cluster Survey 2000. New York, United States: United Nations Children's Fund (UNICEF).	GHDx
1981	Burundi	Burundi Multiple Indicator Cluster Survey 2005	2005	I	I	I	-	-	I	-	-	I	-	-	United Nations Children's Fund (UNICEF), Burundi Institute of Statistics and Economic Studies, United Nations Population Fund (UNFPA). Burundi Multiple Indicator Cluster Survey 2005. New York, United States: United Nations Children's Fund (UNICEF).	GHDx
215027	Burundi	Burundi Routine Immunization Coverage Survey and Post Measles Campaign Survey 2012	2012	I	I	I	I	I	I	-	-	I	-	-	Burundi Routine Immunization Coverage Survey and Post Measles Campaign Survey 2012.	GHDx
559059*	Burundi	Burundi Post Vaccine Campaign Monitoring Survey Against Measles and Rubella Coupled to the National Routine Immunization Coverage Survey 2022	2022	I	I	I	I	I	-	-	I	I	-	I	Global Alliance for Vaccines and Immunization (GAVI), National Institute of Statistics (Burundi), United Nations Children's Fund (UNICEF), World Health Organization (WHO). Burundi Post Vaccine Campaign Monitoring Survey Against Measles and Rubella Coupled to the National Routine Immunization Coverage Survey 2022. Geneva, Switzerland: World Health Organization (WHO).	GHDx
30431	Burundi	Burundi Demographic and Health Survey 2010-2011	2010-2011	I	I	I	I	I	I	-	-	I	-	-	Burundi Institute of Statistics and Economic Studies, ICF International, Ministry of Public Health and the Fight Against AIDS (Burundi). Burundi Demographic and Health Survey 2010-2011. Fairfax, United States of America: ICF International, 2012.	GHDx
286766	Burundi	Burundi Demographic and Health Survey 2016-2017	2016-2017	I	I	I	I	I	I	I	I	I	-	I	Burundi Institute of Statistics and Economic Studies, ICF International, Ministry of Public Health and the Fight Against AIDS (Burundi). Burundi Demographic and Health Survey 2016-2017. Fairfax, United States of America: ICF International, 2018.	GHDx
217914	Cabo Verde	The Health of Children less than five years of age in Cape Verde, Volume I,	1994	I	-	I	-	-	I	-	-	I	-	-	The Health of Children less than five years of age in Cape Verde, Volume I, Epidemiologic Study, 1997.	GHDx

GHDx ID	Country	Series	Year	BCG	DTP1	DTP3	HepB3	Hib3	MCV1	MCV2	PCV3	Pol3	RCV1	RotaC	Citation	Link
		Epidemiologic Study, 1997														
217915	Cabo Verde	Cape Verde National Immunization Coverage Survey 2000	2000	I	I	I	-	-	I	-	-	I	-	-	Ministry of Health (Cape Verde), World Health Organization (WHO). Cape Verde National Immunization Coverage Survey 2000.	GHDx
217916	Cabo Verde	Cape Verde National Immunization Coverage Survey 2002	2002	I	-	I	-	-	I	-	-	I	-	-	Ministry of Health (Cape Verde), World Health Organization (WHO). Cape Verde National Immunization Coverage Survey 2002.	GHDx
21442	Cabo Verde	Cape Verde Demographic and Health Survey 2005	2005	I	I	I	I	-	I	-	-	I	-	-	Macro International, Inc, Ministry of Health (Cape Verde), National Institute of Statistics (Cape Verde). Cape Verde Demographic and Health Survey 2005.	GHDx
217918	Cabo Verde	Cape Verde National Immunization Coverage Survey 2009	2009	I	I	I	I	-	I	-	-	I	-	-	Ministry of Health (Cape Verde), World Health Organization (WHO). Cape Verde National Immunization Coverage Survey 2009.	GHDx
217920	Cabo Verde	Cape Verde Immunization Coverage Survey 2011	2011	I	I	I	I	-	I	-	-	I	-	-	Ministry of Health (Cape Verde), World Health Organization (WHO). Cape Verde Immunization Coverage Survey 2011.	GHDx
524155*	Cabo Verde	Cape Verde Demographic and Reproductive Health Survey 2018	2018	I	I	I	I	I	I	I	-	I	I	-	National Institute of Statistics (Cape Verde), Utica International. Cape Verde Demographic and Reproductive Health Survey 2018. Praia, Cabo Verde: National Institute of Statistics (Cape Verde), 2020.	GHDx
232764	Cambodia	Cambodia Vaccine Coverage Survey of Children 12-23 Months of Age 1995	1995	e	e	e	-	-	I	-	-	I	-	-	Cambodia Vaccine Coverage Survey of Children 12-23 Months of Age 1995.	GHDx
30758	Cambodia	Cambodia Socio-Economic Survey 1996	1996	e	e	e	-	-	e	-	-	-	-	-	National Institute of Statistics (Cambodia). Cambodia Socio-Economic Survey 1996. Phnom Penh, Cambodia: National Institute of Statistics (Cambodia).	GHDx
19170	Cambodia	Cambodia Special Demographic and Health Survey 1998	1998	I	I	I	-	-	I	-	-	I	-	-	Macro International, Inc, Ministry of Health (Cambodia), National Institute of Public Health (Cambodia). Cambodia Special Demographic and Health Survey 1998. Fairfax, United States of America: ICF International.	GHDx

GHDx ID	Country	Series	Year	BCG	DTP1	DTP3	HepB3	Hib3	MCV1	MCV2	PCV3	Pol3	RCV1	RotaC	Citation	Link
30842	Cambodia	Cambodia Socio-Economic Survey 1999	1999	e	e	e	-	-	I	-	-	I	-	-	National Institute of Statistics (Cambodia), United Nations Development Programme (UNDP), World Bank. Cambodia Socio-Economic Survey 1999. Phnom Penh, Cambodia: National Institute of Statistics (Cambodia).	GHDx
19156	Cambodia	Cambodia Demographic and Health Survey 2000	2000	I	I	I	-	-	I	-	-	I	-	-	Macro International, Inc, Ministry of Health (Cambodia), National Institute of Statistics (Cambodia). Cambodia Demographic and Health Survey 2000. Fairfax, United States of America: ICF International.	GHDx
157024	Cambodia	Cambodia Demographic and Health Survey 2014	2014	I	I	I	I	I	I	-	-	I	-	-	ICF International, Ministry of Health (Cambodia), National Institute of Statistics (Cambodia). Cambodia Demographic and Health Survey 2014. Fairfax, United States of America: ICF International, 2017.	GHDx
30963	Cambodia	Cambodia Socio-Economic Survey 2003-2005	2003-2005	I	I	I	-	-	I	-	-	I	-	-	National Institute of Statistics (Cambodia), Statistics Sweden. Cambodia Socio-Economic Survey 2003-2005. Phnom Penh, Cambodia: National Institute of Statistics (Cambodia).	GHDx
19167	Cambodia	Cambodia Demographic and Health Survey 2005-2006	2005-2006	I	I	I	-	-	I	-	-	I	-	-	Macro International, Inc, National Institute of Public Health (Cambodia), National Institute of Statistics (Cambodia). Cambodia Demographic and Health Survey 2005-2006. Fairfax, United States of America: ICF International.	GHDx
31050	Cambodia	Cambodia Socio-Economic Survey 2006-2007	2006-2007	I	I	I	-	-	I	-	-	I	-	-	National Institute of Statistics (Cambodia), Statistics Sweden. Cambodia Socio-Economic Survey 2006-2007. Phnom Penh, Cambodia: National Institute of Statistics (Cambodia).	GHDx
30379	Cambodia	Cambodia Demographic and Health Survey 2010-2011	2010-2011	I	I	I	I	E	I	-	-	I	-	-	ICF Macro, Ministry of Health (Cambodia), National Institute of Statistics (Cambodia). Cambodia Demographic and Health Survey 2010-2011. Fairfax, United States of America: ICF International.	GHDx
523643*	Cambodia	Cambodia Demographic and Health Survey 2021-2022	2021-2022	I	I	I	I	I	I	I	I	I	-	-	ICF International, Ministry of Health (Cambodia), National Institute of Statistics (Cambodia). Cambodia Demographic and Health Survey 2021-2022. Fairfax, United States of America: ICF International, 2023.	GHDx

GHDx ID	Country	Series	Year	BCG	DTP1	DTP3	HepB3	Hib3	MCV1	MCV2	PCV3	Pol3	RCV1	RotaC	Citation	Link
19188	Cameroon	Cameroon Demographic and Health Survey 1991	1991	I	I	I	-	-	I	-	-	I	-	-	Macro International, Inc, Ministry of Economy, Planning and Regional Development (Cameroon). Cameroon Demographic and Health Survey 1991. Fairfax, United States of America: ICF International.	GHDx
19198	Cameroon	Cameroon Demographic and Health Survey 1998	1998	I	I	I	-	-	I	-	-	I	-	-	Central Bureau of the Census and Population Studies (Cameroon), Macro International, Inc. Cameroon Demographic and Health Survey 1998. Fairfax, United States of America: ICF International.	GHDx
2053	Cameroon	Cameroon Multiple Indicator Cluster Survey 2000	2000	I	I	I	-	-	I	-	-	I	-	-	Directorate of Statistics and National Accounts, Ministry of Economics and Finance (Cameroon), United Nations Children's Fund (UNICEF). Cameroon Multiple Indicator Cluster Survey 2000. New York, United States of America: United Nations Children's Fund (UNICEF).	GHDx
2039	Cameroon	Cameroon Household Survey 2001	2001	I	I	-	-	-	I	-	-	-	-	-	National Institute of Statistics (Cameroon), Directorate of Statistics and National Accounts, Ministry of Economics and Finance (Cameroon), AFRISTAT. Cameroon Household Survey 2001. Yaounde, Cameroon: National Institute of Statistics (Cameroon).	GHDx
19211	Cameroon	Cameroon Demographic and Health Survey 2004	2004	I	I	I	-	-	I	-	-	I	-	-	Macro International, Inc, National Institute of Statistics (Cameroon). Cameroon Demographic and Health Survey 2004. Fairfax, United States of America: ICF International.	GHDx
217763	Cameroon	Cameroon National Evaluation of Immunization Coverage among Children Aged 12 to 23 Months 2005	2005	I	I	I	-	-	I	-	-	I	-	-	Cameroon National Evaluation of Immunization Coverage among Children Aged 12 to 23 Months 2005.	GHDx
2063	Cameroon	Cameroon Multiple Indicator Cluster Survey 2006	2006	I	I	I	-	-	I	-	-	I	-	-	United Nations Children's Fund (UNICEF), National Institute of Statistics (Cameroon). Cameroon Multiple Indicator Cluster Survey 2006. New York, United States: United Nations Children's Fund (UNICEF).	GHDx

GHDx ID	Country	Series	Year	BCG	DTP1	DTP3	HepB3	Hib3	MCV1	MCV2	PCV3	Pol3	RCV1	RotaC	Citation	Link
19274	Cameroon	Cameroon Demographic and Health Survey 2011	2011	I	I	I	I	E	I	-	-	I	-	-	ICF International, Ministry of Economy, Planning and Regional Development (Cameroon), Ministry of Public Health (Cameroon), National Institute of Statistics (Cameroon), Pasteur Center of Cameroon. Cameroon Demographic and Health Survey 2011. Fairfax, United States of America: ICF International.	GHDx
217764	Cameroon	Cameroon Post-Campaign Vaccination Coverage Survey 2011	2011	I	I	I	I	I	I	-	-	I	-	-	Ministry of Public Health (Cameroon), National Institute of Statistics (Cameroon). Cameroon Post-Campaign Vaccination Coverage Survey 2011.	GHDx
244455	Cameroon	Cameroon Multiple Indicator Cluster Survey 2014	2014	e	e	e	E	E	I	-	E	e	-	-	Ministry of Public Health (Cameroon), National Institute of Statistics (Cameroon), United Nations Children's Fund (UNICEF). Cameroon Multiple Indicator Cluster Survey 2014. New York, United States of America: United Nations Children's Fund (UNICEF), 2017.	GHDx
558938*	Cameroon	Cameroon Vaccination Coverage Survey 2023	2023	I	I	I	I	I	I	I	I	I	-	I	Cameroon Vaccination Coverage Survey 2023.	GHDx
217762	Cameroon	Cameroon Immunization Coverage Survey November-December 1990	1987-1990	I	I	I	-	-	I	-	-	I	-	-	Cameroon Immunization Coverage Survey November-December 1990.	GHDx
413167	Cameroon	Cameroon Demographic and Health Survey 2018-2019	2018-2019	I	I	I	I	I	I	-	I	I	-	I	ICF International, Ministry of Public Health (Cameroon), National Institute of Statistics (Cameroon). Cameroon Demographic and Health Survey 2018-2019. Fairfax, United States of America: ICF International.	GHDx
191172	Canada	Canada Childhood National Immunization Coverage Survey 2011	2011	-	-	I	-	-	I	-	-	I	-	-	Public Health Agency of Canada, Statistics Canada (StatCan). Canada Childhood National Immunization Coverage Survey 2011.	GHDx
546834*	Canada	Canada Childhood National Immunization Coverage Survey 2022	2022	-	-	e	E	E	I	-	E	I	I	E	Public Health Agency of Canada, Statistics Canada (StatCan). Canada Childhood National Immunization Coverage Survey 2022.	GHDx
453327	Canada	Canada Childhood National Immunization Coverage Survey 2017-2018	2017-2018	-	-	-	I	-	I	-	I	-	-	-	Public Health Agency of Canada, Statistics Canada (StatCan). Canada Childhood National Immunization Coverage Survey 2017-2018.	GHDx
482562*	Canada	Canada Childhood National Immunization Coverage Survey 2019-2020	2019-2020	-	-	e	-	E	I	-	E	I	I	-	Public Health Agency of Canada, Statistics Canada (StatCan). Canada Childhood National Immunization Coverage Survey 2019-2020.	GHDx

GHDx ID	Country	Series	Year	BCG	DTP1	DTP3	HepB3	Hib3	MCV1	MCV2	PCV3	Pol3	RCV1	RotaC	Citation	Link
215187	Central African Republic	Central African Republic EPI Review 1993	1993	I	I	I	-	-	I	-	-	I	-	-	Central African Republic EPI Review 1993.	GHDx
2209	Central African Republic	Central African Republic Multiple Indicator Cluster Survey 2000	2000	I	I	I	-	-	I	-	-	I	-	-	Division of Statistics and Economic and Social Studies (Central African Republic), Ministry of Economy, Planning and International Cooperation (Central African Republic), United Nations Children's Fund (UNICEF). Central African Republic Multiple Indicator Cluster Survey 2000. New York, United States of America: United Nations Children's Fund (UNICEF).	GHDx
215189	Central African Republic	Central African Republic External EPI Review 2002	2002	I	I	I	-	-	I	-	-	I	-	-	Government of Central African Republic, United Nations Children's Fund (UNICEF), World Health Organization (WHO). Central African Republic External EPI Review 2002.	GHDx
2223	Central African Republic	Central African Republic Multiple Indicator Cluster Survey 2006	2006	I	I	I	-	-	I	-	-	I	-	-	United Nations Children's Fund (UNICEF). Central African Republic Multiple Indicator Cluster Survey 2006. New York, United States: United Nations Children's Fund (UNICEF).	GHDx
215247	Central African Republic	Central African Republic EPI Coverage Survey 2012	2012	I	-	I	I	I	I	-	-	e	-	-	Ministry of Public Health, Population, and AIDS Control (Central African Republic). Central African Republic EPI Coverage Survey 2012.	GHDx
215186	Central African Republic	Central African Republic Immunization Coverage Survey February 1991	1989-1991	I	I	I	-	-	I	-	-	I	-	-	Central African Republic Immunization Coverage Survey February 1991.	GHDx
19292	Central African Republic	Central African Republic Demographic and Health Survey 1994-1995	1994-1995	I	I	I	-	-	I	-	-	I	-	-	Division of Statistics and Economic and Social Studies (Central African Republic), Macro International, Inc. Central African Republic Demographic and Health Survey 1994-1995. Fairfax, United States of America: ICF International.	GHDx
82832	Central African Republic	Central African Republic Multiple Indicator Cluster Survey 2010-2011	2010-2011	I	I	I	E	-	I	-	-	I	-	-	Central African Institute of Statistics, Economic and Social Studies (ICASEES) (Central African Republic), ICF International. Central African Republic Multiple Indicator Cluster Survey 2010-2011. Fairfax, United States of America: ICF International, 2013.	GHDx

GHDx ID	Country	Series	Year	BCG	DTP1	DTP3	HepB3	Hib3	MCV1	MCV2	PCV3	Pol3	RCV1	RotaC	Citation	Link
466384*	Central African Republic	Central African Republic Multiple Indicator Cluster Survey 2018-2019	2018-2019	I	I	I	I	I	I	-	I	I	E	-	Central African Institute of Statistics, Economic and Social Studies (ICASEES) (Central African Republic). Central African Republic Multiple Indicator Cluster Survey 2018-2019. New York, United States of America: United Nations Children's Fund (UNICEF), 2021.	GHDx
229585	Chad	Chad National Immunization Coverage Survey 1990	1990	I	-	I	-	-	I	-	-	I	-	-	Chad National Immunization Coverage Survey 1990.	GHDx
2244	Chad	Chad Multiple Indicator Cluster Survey 2000	2000	I	I	I	-	-	I	-	-	I	-	-	United Nations Children's Fund (UNICEF), Census Bureau (Chad), National Institute of Statistical, Economic and Demographic Studies (Chad). Chad Multiple Indicator Cluster Survey 2000. New York, United States: United Nations Children's Fund (UNICEF).	GHDx
229566	Chad	Chad EPI Review 2002	2002	I	I	I	-	-	I	-	-	I	-	-	Ministry of Public Health (Chad). Chad EPI Review 2002.	GHDx
19315	Chad	Chad Demographic and Health Survey 2004	2004	I	I	I	-	-	I	-	-	I	-	-	Macro International, Inc, National Institute for Statistics, Economic and Demographic Studies (INSEED) (Chad). Chad Demographic and Health Survey 2004. Fairfax, United States of America: ICF International.	GHDx
76701	Chad	Chad Multiple Indicator Cluster Survey 2010	2010	I	I	I	E	-	I	-	-	I	-	-	Ministry of Planning, Economy, and International Cooperation (Chad), National Institute for Statistics, Economic and Demographic Studies (INSEED) (Chad), United Nations Children's Fund (UNICEF). Chad Multiple Indicator Cluster Survey 2010. New York, United States of America: United Nations Children's Fund (UNICEF), 2014.	GHDx
229490	Chad	Chad Vaccination Coverage Survey 2012	2012	I	I	I	I	I	I	-	-	I	-	-	Chad Vaccination Coverage Survey 2012.	GHDx
438586	Chad	Chad Vaccine Coverage Survey 2017	2017	I	I	I	I	I	I	-	-	I	-	-	World Health Organization (WHO). Chad Vaccine Coverage Survey 2017.	GHDx
19305	Chad	Chad Demographic and Health Survey 1996-1997	1996-1997	I	I	I	-	-	I	-	-	I	-	-	Census Bureau (Chad), Macro International, Inc, National Institute for Statistics, Economic and Demographic Studies (INSEED) (Chad). Chad Demographic and Health Survey 1996-1997. Fairfax, United States of America: ICF International.	GHDx

GHDx ID	Country	Series	Year	BCG	DTP1	DTP3	HepB3	Hib3	MCV1	MCV2	PCV3	Pol3	RCV1	RotaC	Citation	Link
157025	Chad	Chad Demographic and Health Survey 2014-2015	2014-2015	I	I	I	I	I	I	-	-	I	-	-	ICF International, National Institute for Statistics, Economic and Demographic Studies (INSEED) (Chad). Chad Demographic and Health Survey 2014-2015. Fairfax, United States of America: ICF International, 2016.	GHDx
560287*	Chad	Chad National Routine Vaccination Coverage Survey 2023-2024	2023-2024	I	I	I	I	I	I	-	-	I	-	-	World Health Organization (WHO). Chad National Routine Vaccination Coverage Survey 2023-2024. Geneva, Switzerland: World Health Organization (WHO).	GHDx
217077	China	The EPI Programme in The People's Republic of China 1986	1985	I	-	I	-	-	I	-	-	I	-	-	The EPI Programme in The People's Republic of China 1986.	GHDx
194012	China	China Multiple Indicator Cluster Survey 1995	1995	I	-	I	-	-	I	-	-	I	-	-	State Statistical Bureau (China), United Nations Children's Fund (UNICEF). China Multiple Indicator Cluster Survey 1995.	GHDx
68809	China	China National Health Services Survey 2003	2003	I	-	I	I	-	I	-	-	I	-	-	Ministry of Health (China). China National Health Services Survey 2003.	GHDx
68803	China	China National Health Services Survey 2008	2008	I	-	I	I	-	I	-	-	I	-	-	Ministry of Health (China). China National Health Services Survey 2008.	GHDx
511668*	China	Coverage and Equity of Childhood Vaccines in China	2019	I	I	I	I	I	I	-	I	I	I	-	Zhang H, Lai X, Mak J, Sriudomporn S, Zhang H, Fang H, Patenaude B. Coverage and Equity of Childhood Vaccines in China. JAMA Netw Open. 2022; 5(12): e2246005.	GHDx
19333	Colombia	Colombia Demographic and Health Survey 1986	1986	I	I	I	-	-	I	-	-	I	-	-	Ministry of Health (Colombia), Regional Population Center (Colombia), Westinghouse; Institute for Resource Development. Colombia Demographic and Health Survey 1986. Fairfax, United States of America: ICF International, 1988.	GHDx
19341	Colombia	Colombia Demographic and Health Survey 1990	1990	I	I	I	-	-	I	-	-	I	-	-	Institute for Resource Development, Macro Systems, Profamilia (Colombia). Colombia Demographic and Health Survey 1990. Fairfax, United States of America: ICF International, 1991.	GHDx
19350	Colombia	Colombia Demographic and Health Survey 1995	1995	I	I	I	-	-	I	-	-	I	-	-	Macro International, Inc, Profamilia (Colombia). Colombia Demographic and Health Survey 1995. Fairfax, United States of America: ICF International, 1995.	GHDx

GHDx ID	Country	Series	Year	BCG	DTP1	DTP3	HepB3	Hib3	MCV1	MCV2	PCV3	Pol3	RCV1	RotaC	Citation	Link
19359	Colombia	Colombia Demographic and Health Survey 2000	2000	I	I	I	I	E	I	-	-	I	I	-	Macro International, Inc, Profamilia (Colombia). Colombia Demographic and Health Survey 2000. Fairfax, United States of America: ICF International, 2000.	GHDx
19324	Colombia	Colombia Demographic and Health Survey 2004-2005	2004-2005	I	I	I	I	I	I	-	-	I	I	-	Macro International, Inc, Profamilia (Colombia). Colombia Demographic and Health Survey 2004-2005. Fairfax, United States of America: ICF International, 2005.	GHDx
21281	Colombia	Colombia Demographic and Health Survey 2009-2010	2009-2010	I	I	I	I	I	I	-	-	I	I	-	ICF Macro, Profamilia (Colombia). Colombia Demographic and Health Survey 2009-2010. Fairfax, United States of America: ICF International, 2011.	GHDx
19370	Comoros	Comoros Demographic and Health Survey 1996	1996	I	I	I	-	-	I	-	-	I	-	-	Macro International, Inc, National Centre of Documentation and Scientific Research (Comoros). Comoros Demographic and Health Survey 1996. Fairfax, United States of America: ICF International.	GHDx
3114	Comoros	Comoros Multiple Indicator Cluster Survey 2000	2000	I	I	I	-	-	I	-	-	I	-	-	United Nations Development Programme (UNDP), United Nations Children's Fund (UNICEF). Comoros Multiple Indicator Cluster Survey 2000. New York, United States: United Nations Children's Fund (UNICEF).	GHDx
217775	Comoros	Comoros Immunization Coverage Survey November 2007	2007	I	I	I	I	-	I	-	-	-	-	-	Comoros Immunization Coverage Survey November 2007.	GHDx
217776	Comoros	Comoros Immunization Coverage Survey October 2010	2010	I	I	I	I	-	I	-	-	I	-	-	Comoros Immunization Coverage Survey October 2010.	GHDx
217777	Comoros	Comoros Post Measles Campaign and Routine Immunization Coverage Survey 2013	2013	I	I	I	-	-	I	-	-	I	-	-	Comoros Post Measles Campaign and Routine Immunization Coverage Survey 2013.	GHDx
447887	Comoros	Comoros Post Measles Campaign and Routine Immunization Coverage Survey 2016	2016	I	I	-	I	I	I	-	-	I	-	-	National Directorate of Health (Comoros), United Nations Children's Fund (UNICEF), World Health Organization (WHO). Comoros Post Measles Campaign and Routine Immunization Coverage Survey 2016.	GHDx

GHDx ID	Country	Series	Year	BCG	DTP1	DTP3	HepB3	Hib3	MCV1	MCV2	PCV3	Pol3	RCV1	RotaC	Citation	Link
541281*	Comoros	Comoros Multiple Indicator Cluster Survey 2022	2022	I	I	I	I	I	I	E	-	I	E	-	National Institute of Statistics and Economic and Demographic Studies (INSEED) (Comoros), United Nations Children's Fund (UNICEF). Comoros Multiple Indicator Cluster Survey 2022. New York, United States of America: United Nations Children's Fund (UNICEF), 2023.	GHDx
76850	Comoros	Comoros Demographic and Health Survey 2012-2013	2012-2013	I	I	I	I	E	I	-	-	I	-	-	General Directorate of Statistics and Forecasting (Comoros), ICF International. Comoros Demographic and Health Survey 2012-2013. Fairfax, United States of America: ICF International.	GHDx
217771	Congo (Brazzaville)	Congo EPI Review 1988	1985	I	I	I	-	-	I	-	-	I	-	-	Congo EPI Review 1988.	GHDx
217772	Congo (Brazzaville)	Congo EPI Review 1987	1987	I	I	I	-	-	I	-	-	I	-	-	Congo EPI Review 1987.	GHDx
217773	Congo (Brazzaville)	Congo International EPI Review 1990	1990	I	I	I	-	-	I	-	-	I	-	-	Congo International EPI Review 1990.	GHDx
19391	Congo (Brazzaville)	Congo Demographic and Health Survey 2005	2005	e	e	e	-	-	e	-	-	e	-	-	Macro International, Inc, National Center for Statistics and Economic Studies (Congo, Rep.). Congo Demographic and Health Survey 2005. Fairfax, United States of America: ICF International.	GHDx
217774	Congo (Brazzaville)	Congo External EPI Review 2010	2010	I	I	I	E	E	I	-	-	I	-	-	Congo External EPI Review 2010.	GHDx
56151	Congo (Brazzaville)	Congo Demographic and Health Survey 2011-2012	2011-2012	I	I	I	I	E	I	-	-	I	-	-	ICF International, Ministry of Health (Congo, Rep.), National Center for Statistics and Economic Studies (Congo, Rep.). Congo Demographic and Health Survey 2011-2012. Fairfax, United States of America: ICF International.	GHDx
234733	Congo (Brazzaville)	Congo Multiple Indicator Cluster Survey 2014-2015	2014-2015	I	I	I	I	I	I	-	I	I	-	-	National Institute of Statistics (INS) (Congo, Rep.), United Nations Children's Fund (UNICEF). Congo Multiple Indicator Cluster Survey 2014-2015. New York, United States of America: United Nations Children's Fund (UNICEF), 2018.	GHDx
125596	Costa Rica	Costa Rica Multiple Indicator Cluster Survey 2011	2011	I	I	I	I	I	I	-	E	I	-	-	Costa Rican Demographic Association, Ministry of Health (Costa Rica), United Nations Children's Fund (UNICEF). Costa Rica Multiple Indicator Cluster Survey 2011. New York, United States of America: United Nations Children's Fund (UNICEF), 2013.	GHDx

GHDx ID	Country	Series	Year	BCG	DTP1	DTP3	HepB3	Hib3	MCV1	MCV2	PCV3	Pol3	RCV1	RotaC	Citation	Link
452953*	Costa Rica	Costa Rica Multiple Indicator Cluster Survey 2018	2018	I	I	I	I	I	I	-	I	I	I	-	Ministry of Health (Costa Rica), National Institute of Statistics and Censuses (Costa Rica), United Nations Children's Fund (UNICEF). Costa Rica Multiple Indicator Cluster Survey 2018. New York, United States of America: United Nations Children's Fund (UNICEF), 2020.	GHDx
27638	Costa Rica	Costa Rica Reproductive Health Survey 1992-1993	1992-1993	I	I	I	-	-	I	-	-	I	-	-	Costa Rica Social Security Institute and Division of Reproductive Health-Centers for Disease Control and Prevention (CDC). Costa Rica 1993 Reproductive Health Survey. Atlanta, United States: Centers for Disease Control and Prevention (CDC).	GHDx
217174	Côte d'Ivoire	Côte d'Ivoire Immunization Coverage Survey February 1991	1991	-	I	I	-	-	I	-	-	I	-	-	Côte d'Ivoire Immunization Coverage Survey February 1991.	GHDx
18519	Côte d'Ivoire	Côte d'Ivoire Demographic and Health Survey 1994	1994	I	I	I	-	-	I	-	-	I	-	-	Macro International, Inc, National Institute of Statistics (Côte d'Ivoire). Côte d'Ivoire Demographic and Health Survey 1994. Fairfax, United States of America: ICF International.	GHDx
26445	Côte d'Ivoire	Côte d'Ivoire Multiple Indicator Cluster Survey 1996	1996	I	I	I	-	-	I	-	-	I	-	-	Ministry of Planning and Industrial Development (Côte d'Ivoire), National Statistics Institute (Côte d'Ivoire), United Nations Children's Fund (UNICEF). Côte d'Ivoire Multiple Indicator Cluster Survey 1996.	GHDx
26444	Côte d'Ivoire	Côte d'Ivoire Multiple Indicator Cluster Survey 2000	2000	I	I	I	-	-	I	-	-	I	-	-	National School for Statistics and Economics Applied (ENSEA), United Nations Children's Fund (UNICEF), United Nations Educational, Scientific and Cultural Organization (UNESCO). Côte d'Ivoire Multiple Indicator Cluster Survey 2000. New York, United States of America: United Nations Children's Fund (UNICEF).	GHDx
217175	Côte d'Ivoire	Côte d'Ivoire External EPI Review 2001	2001	I	I	I	-	-	I	-	-	I	-	-	Côte d'Ivoire External EPI Review 2001.	GHDx
26433	Côte d'Ivoire	Côte d'Ivoire Multiple Indicator Cluster Survey 2006	2006	I	I	I	I	-	I	-	-	I	-	-	United Nations Children's Fund (UNICEF), National Institute of Statistics (Côte d'Ivoire). Côte d'Ivoire Multiple Indicator Cluster Survey 2006. New York, United States: United Nations Children's Fund (UNICEF).	GHDx
217176	Côte d'Ivoire	Côte d'Ivoire External EPI Review 2010	2010	I	I	I	I	-	I	-	-	I	-	-	Côte d'Ivoire External EPI Review 2010.	GHDx

GHDx ID	Country	Series	Year	BCG	DTP1	DTP3	HepB3	Hib3	MCV1	MCV2	PCV3	Pol3	RCV1	RotaC	Citation	Link
217760	Côte d'Ivoire	Côte d'Ivoire Vaccination Coverage Survey 2013	2013	I	I	I	I	I	I	-	-	I	-	-	Côte d'Ivoire Vaccination Coverage Survey 2013.	GHDx
218611	Côte d'Ivoire	Cote d'Ivoire Multiple Indicator Cluster Survey 2016	2016	I	e	e	I	I	I	-	I	I	-	E	National Institute of Statistics (Côte d'Ivoire), United Nations Children's Fund (UNICEF). Cote d'Ivoire Multiple Indicator Cluster Survey 2016. New York, United States of America: United Nations Children's Fund (UNICEF), 2018.	GHDx
502491*	Côte d'Ivoire	Côte d'Ivoire Routine Vaccination Coverage Survey 2018	2018	I	I	I	I	I	I	-	I	I	E	-	Côte d'Ivoire Routine Vaccination Coverage Survey 2018.	GHDx
502492*	Côte d'Ivoire	Côte d'Ivoire National Routine Immunization Coverage Survey 2021	2021	I	I	I	I	I	I	-	I	I	I	I	Côte d'Ivoire National Routine Immunization Coverage Survey 2021.	GHDx
535004*	Côte d'Ivoire	Côte d'Ivoire Demographic and Health Survey 2021	2021	I	I	I	I	I	I	-	I	I	-	I	ICF International, Ministry of Health and Public Hygiene (MSHP) (Côte d'Ivoire), National Institute of Statistics (Côte d'Ivoire). Côte d'Ivoire Demographic and Health Survey 2021. Fairfax, United States of America: ICF International, 2023.	GHDx
18531	Côte d'Ivoire	Côte d'Ivoire Demographic and Health Survey 1998-1999	1998-1999	I	I	I	-	-	I	-	-	I	-	-	Macro International, Inc, National Institute of Statistics (Côte d'Ivoire). Côte d'Ivoire Demographic and Health Survey 1998-1999. Fairfax, United States of America: ICF International.	GHDx
18533	Côte d'Ivoire	Côte d'Ivoire Demographic and Health Survey 2011-2012	2011-2012	I	I	I	I	E	I	-	-	I	-	-	ICF International, Ministry of the Fight Against AIDS (Côte d'Ivoire), National Institute of Statistics (Côte d'Ivoire). Côte d'Ivoire Demographic and Health Survey 2011-2012. Fairfax, United States of America: ICF International.	GHDx
446845	Côte d'Ivoire	Côte d'Ivoire Measles Vaccination Campaign Evaluation 2014-2015	2014-2015	I	I	I	I	I	I	-	-	I	-	-	Ministry of Health and the Fight against AIDS (Côte d'Ivoire), United Nations Children's Fund (UNICEF), World Health Organization (WHO). Côte d'Ivoire Measles Vaccination Campaign Evaluation 2014-2015.	GHDx
3310	Cuba	Cuba Multiple Indicator Cluster Survey 2006	2006	I	I	I	I	I	I	-	-	I	I	-	United Nations Children's Fund (UNICEF), National Bureau of Statistics (Cuba). Cuba Multiple Indicator Cluster Survey 2006. New York, United States: United Nations Children's Fund (UNICEF).	GHDx

GHDx ID	Country	Series	Year	BCG	DTP1	DTP3	HepB3	Hib3	MCV1	MCV2	PCV3	Pol3	RCV1	RotaC	Citation	Link
169975	Cuba	Cuba Multiple Indicator Cluster Survey 2014	2014	I	I	I	I	I	I	-	-	I	I	-	Ministry of Public Health (Cuba), National Office of Statistics (Cuba), United Nations Children's Fund (UNICEF). Cuba Multiple Indicator Cluster Survey 2014. New York, United States of America: United Nations Children's Fund (UNICEF), 2005.	GHDx
464145*	Cuba	Cuba Multiple Indicator Cluster Survey 2019	2019	I	I	I	I	I	I	-	-	-	I	-	Ministry of Public Health (Cuba), National Office of Statistics (Cuba), United Nations Children's Fund (UNICEF). Cuba Multiple Indicator Cluster Survey 2019. New York, United States of America: United Nations Children's Fund (UNICEF), 2021.	GHDx
60935	Cuba	Cuba Multiple Indicator Cluster Survey 2010-2011	2010-2011	I	I	I	I	I	I	-	-	I	I	-	Ministry of Public Health (Cuba), United Nations Children's Fund (UNICEF). Cuba Multiple Indicator Cluster Survey 2010-2011. New York, United States of America: United Nations Children's Fund (UNICEF).	GHDx
217922	Cyprus	Cyprus Children's Immunization Survey 2009	2009	-	I	I	I	I	I	-	I	I	-	-	Ministry of Health (Cyprus). Cyprus ChildrenÃ¢Â€Â™s Immunization Survey 2009.	GHDx
453332	Cyprus	Cyprus Child Immunization Survey 2019	2019	-	I	I	I	I	I	-	-	-	-	-	Cyprus Child Immunization Survey 2019.	GHDx
3379	Djibouti	Djibouti Immunization, Diarrhoeal Disease, Maternal and Child Mortality Survey 1989	1989	I	I	I	-	-	I	-	-	I	-	-	United Nations Children's Fund (UNICEF). Djibouti Immunization, Diarrhoeal Disease, Maternal and Child Mortality Survey 1989.	GHDx
3392	Djibouti	Djibouti Family Health Survey 2002	2002	e	e	e	-	-	e	-	-	e	E	-	Department of Statistics and Demographic Studies (Djibouti), League of Arab States, Ministry of Health (Djibouti), Pan Arab Project for Family Health (PAPFAM). Djibouti Family Health Survey 2002.	GHDx
3404	Djibouti	Djibouti Multiple Indicator Cluster Survey 2006	2006	I	I	I	-	-	I	-	-	I	-	-	Ministry of Economy, Finance, and Planning in charge of Privatization (Djibouti), Ministry of Health (Djibouti), United Nations Children's Fund (UNICEF). Djibouti Multiple Indicator Cluster Survey 2006. New York, United States of America: United Nations Children's Fund (UNICEF).	GHDx
218033	Djibouti	Djibouti Immunization Coverage Survey 2008	2008	I	I	I	I	I	I	-	-	I	-	-	Ministry of Health (Djibouti). Djibouti Immunization Coverage Survey 2008.	GHDx

GHDx ID	Country	Series	Year	BCG	DTP1	DTP3	HepB3	Hib3	MCV1	MCV2	PCV3	Pol3	RCV1	RotaC	Citation	Link
218035	Djibouti	Djibouti Family Health Survey 2012	2012	I	I	I	E	E	I	E	-	e	-	-	Department of Statistics and Demographic Studies (Djibouti), League of Arab States, Ministry of Health (Djibouti), Pan Arab Project for Family Health (PAPFAM). Djibouti Family Health Survey 2012.	GHDx
502494*	Djibouti	Djibouti National Vaccination Coverage Survey 2020	2020	I	I	I	I	I	I	-	I	I	-	-	Djibouti National Vaccination Coverage Survey 2020.	GHDx
19400	Dominican Republic	Dominican Republic Demographic and Health Survey 1986	1986	I	I	I	-	-	I	-	-	I	-	-	National Council for Population and Family (Dominican Republic), Westinghouse; Institute for Resource Development. Dominican Republic Demographic and Health Survey 1986. Fairfax, United States of America: ICF International.	GHDx
19410	Dominican Republic	Dominican Republic Demographic and Health Survey 1991	1991	I	I	I	-	-	I	-	-	I	-	-	Institute for Resource Development, Macro Systems, National Planning Office (Dominican Republic), Profamilia (Colombia). Dominican Republic Demographic and Health Survey 1991. Fairfax, United States of America: ICF International.	GHDx
19421	Dominican Republic	Dominican Republic Demographic and Health Survey 1996	1996	I	I	I	-	-	I	-	-	I	-	-	Center for Social and Demographic Studies (Dominican Republic) (CESDEM), Macro International, Inc, National Planning Office (Dominican Republic), Profamilia (Colombia). Dominican Republic Demographic and Health Survey 1996. Fairfax, United States of America: ICF International.	GHDx
19431	Dominican Republic	Dominican Republic Experimental Demographic and Health Survey 1999	1999	I	I	I	-	-	I	-	-	I	-	-	Center for Social and Demographic Studies (Dominican Republic) (CESDEM), Macro International, Inc. Dominican Republic Experimental Demographic and Health Survey 1999. Fairfax, United States of America: ICF International.	GHDx
27069	Dominican Republic	Dominican Republic Multiple Indicator Cluster Survey 2000	2000	I	I	I	-	-	e	-	-	I	-	-	Center for Social and Demographic Studies (Dominican Republic) (CESDEM), United Nations Children's Fund (UNICEF). Dominican Republic Multiple Indicator Cluster Survey 2000. New York, United States of America: United Nations Children's Fund (UNICEF).	GHDx

GHDx ID	Country	Series	Year	BCG	DTP1	DTP3	HepB3	Hib3	MCV1	MCV2	PCV3	Pol3	RCV1	RotaC	Citation	Link
19444	Dominican Republic	Dominican Republic Demographic and Health Survey 2002	2002	I	I	I	I	E	I	-	-	I	-	-	Center for Social and Demographic Studies (Dominican Republic) (CESDEM), Macro International, Inc. Dominican Republic Demographic and Health Survey 2002. Fairfax, United States of America: ICF International.	GHDx
3455	Dominican Republic	Dominican Republic National Multipurpose Household Survey 2006	2006	I	I	I	I	I	I	E	-	e	E	-	National Statistics Office (Dominican Republic), United Nations Children's Fund (UNICEF). Dominican Republic National Multipurpose Household Survey 2006. Santo Domingo, Dominican Republic: National Statistics Office (Dominican Republic).	GHDx
3465	Dominican Republic	Dominican Republic Multiple Indicator Cluster Survey 2006	2006	I	I	I	-	-	I	-	-	I	-	-	National Statistics Office (Dominican Republic), United Nations Children's Fund (UNICEF). Dominican Republic Multiple Indicator Cluster Survey 2006.	GHDx
3441	Dominican Republic	Dominican Republic National Multipurpose Household Survey 2007	2007	-	-	-	-	-	I	-	-	-	-	-	National Statistics Office (Dominican Republic). Dominican Republic National Multipurpose Household Survey 2007. Santo Domingo, Dominican Republic: National Statistics Office (Dominican Republic).	GHDx
19456	Dominican Republic	Dominican Republic Demographic and Health Survey 2007	2007	I	I	I	I	I	I	-	-	I	E	-	Center for Social and Demographic Studies (Dominican Republic) (CESDEM), Macro International, Inc. Dominican Republic Demographic and Health Survey 2007. Fairfax, United States of America: ICF International.	GHDx
77819	Dominican Republic	Dominican Republic Demographic and Health Survey 2013	2013	I	I	I	I	I	I	-	-	I	-	E	Center for Social and Demographic Studies (Dominican Republic) (CESDEM), ICF International, Ministry of Public Health and Social Assistance (Dominican Republic). Dominican Republic Demographic and Health Survey 2013. Fairfax, United States of America: ICF International, 2014.	GHDx
200697	Dominican Republic	Dominican Republic Multiple Indicator Cluster Survey 2014	2014	I	I	I	I	I	I	-	E	I	I	I	National Statistics Office (Dominican Republic), United Nations Children's Fund (UNICEF). Dominican Republic Multiple Indicator Cluster Survey 2014. New York, United States of America: United Nations Children's Fund (UNICEF), 2016.	GHDx

GHDx ID	Country	Series	Year	BCG	DTP1	DTP3	HepB3	Hib3	MCV1	MCV2	PCV3	Pol3	RCV1	RotaC	Citation	Link
488697*	Dominican Republic	Dominican Republic Multiple Indicator Cluster Survey 2019	2019	I	I	I	I	I	I	E	I	e	I	I	National Statistics Office (Dominican Republic), United Nations Children's Fund (UNICEF). Dominican Republic Multiple Indicator Cluster Survey 2019. New York, United States of America: United Nations Children's Fund (UNICEF), 2021.	GHDx
65416	Dominican Republic	Dominican Republic National Multipurpose Household Survey 2009-2010	2009-2010	I	I	I	-	-	I	-	-	I	-	-	International Labour Organization (ILO), National Statistics Office (Dominican Republic), United Nations Children's Fund (UNICEF). Dominican Republic National Multipurpose Household Survey 2009-2010. 2011.	GHDx
22308	DR Congo	Zaire Immunization Survey 1991	1991	I	I	I	-	-	I	-	-	I	-	-	Expanded Program on Immunization and Childhood Disease Control (Zaire), Centers for Disease Control and Prevention (CDC), Rotary International, United Nations Children's Fund (UNICEF), World Health Organization (WHO). Zaire Immunization Survey 1991.	GHDx
3161	DR Congo	Democratic Republic of the Congo Multiple Indicator Cluster Survey 2001	2001	e	e	e	-	-	e	-	-	e	-	-	Ministry of Planning and Reconstruction (Congo, DR), United Nations Children's Fund (UNICEF). Congo, DR Multiple Indicator Cluster Survey 2001. New York, United States: United Nations Children's Fund (UNICEF).	GHDx
19381	DR Congo	Democratic Republic of the Congo Demographic and Health Survey 2007	2007	I	I	I	-	-	I	-	-	I	-	-	Macro International, Inc, Ministry of Planning (Congo, DR). Democratic Republic of the Congo Demographic and Health Survey 2007. Fairfax, United States of America: ICF International.	GHDx
26998	DR Congo	Democratic Republic of the Congo Multiple Indicator Cluster Survey 2010	2010	I	I	I	E	-	I	-	-	I	-	-	National Statistical Institute (Congo, DR), Ministry of Planning (Congo, DR), United Nations Children's Fund (UNICEF). Congo, DR Multiple Indicator Cluster Survey 2010. New York, United States: United Nations Children's Fund (UNICEF).	GHDx
217767	DR Congo	Democratic Republic of the Congo Immunization Coverage Survey 2012	2012	I	I	I	I	I	I	-	-	I	-	-	Congo, DR Immunization Coverage Survey 2012.	GHDx

GHDx ID	Country	Series	Year	BCG	DTP1	DTP3	HepB3	Hib3	MCV1	MCV2	PCV3	Pol3	RCV1	RotaC	Citation	Link
76878	DR Congo	Democratic Republic of the Congo Demographic and Health Survey 2013-2014	2013-2014	I	I	I	I	I	I	-	-	I	-	-	ICF International, Ministry of Planning and Monitoring Implementation of the Revolution of Modernity (Congo, DR), Ministry of Public Health (Congo, DR), National Institute of Statistics (Congo, DR). Democratic Republic of the Congo Demographic and Health Survey 2013-2014. Fairfax, United States of America: ICF International, 2014.	GHDx
437955	DR Congo	Democratic Republic of the Congo Multiple Indicator Cluster Survey 2017-2018	2017-2018	I	I	I	I	I	I	-	I	e	-	-	National Statistical Institute (Congo, DR), Ministry of Planning (Congo, DR), United Nations Children's Fund (UNICEF). Congo, DR Multiple Indicator Cluster Survey 2010. New York, United States: United Nations Children's Fund (UNICEF).	GHDx
501800*	DR Congo	Democratic Republic of the Congo Vaccination Coverage Survey among Children from 6 to 23 Months 2021-2022	2021-2022	I	I	I	I	I	I	-	I	I	-	I	School of Public Health, University of Kinshasa. Democratic Republic of the Congo Vaccination Coverage Survey among Children from 6 to 23 Months 2021-2022.	GHDx
563414*	DR Congo	Democratic Republic of the Congo Demographic and Health Survey 2023-2024	2023-2024	I	I	I	I	I	I	-	I	I	-	I	ICF International, Ministry of Planning and Monitoring Implementation of the Revolution of Modernity (Congo, DR), Ministry of Public Health (Congo, DR), National Institute of Statistics (Congo, DR). Democratic Republic of the Congo Demographic and Health Survey 2023-2024. Fairfax, United States of America: ICF International.	GHDx
27615	Ecuador	Ecuador Reproductive Health Survey 1994	1994	I	I	I	-	-	I	-	-	I	-	-	Center for Studies of Population and Social Development (CEPAR), Division of Reproductive Health-Centers for Disease Control and Prevention (CDC). (1995) Ecuador Reproductive Health Survey 1994. Atlanta, United States: Centers for Disease Control and Prevention (CDC).	GHDx
46804	Ecuador	Ecuador Living Standards Measurement Survey 1994	1994	I	I	I	-	-	-	-	-	I	-	-	Ecuadorian Professional Training Service (SECAP), World Bank. Ecuador Living Standards Measurement Survey 1994. Washington DC, United States of America: World Bank.	GHDx
46837	Ecuador	Ecuador Living Standards Measurement Survey 1995	1995	I	I	I	-	-	-	-	-	I	-	-	National Institute of Statistics and Censuses (Ecuador), World Bank. Ecuador Living Standards Measurement Survey 1995.	GHDx

GHDx ID	Country	Series	Year	BCG	DTP1	DTP3	HepB3	Hib3	MCV1	MCV2	PCV3	Pol3	RCV1	RotaC	Citation	Link
27621	Ecuador	Ecuador Reproductive Health Survey 1999	1999	I	I	I	-	-	I	-	-	I	-	-	Center for Studies of Population and Social Development (CEPAR) (Ecuador), Division of Reproductive Health-Centers for Disease Control and Prevention (CDC). Ecuador Reproductive Health Survey 1999. Atlanta, United States: Centers for Disease Control and Prevention (CDC), 2001.	GHDx
27630	Ecuador	Ecuador Reproductive Health Survey 2004	2004	I	e	e	-	-	e	-	-	I	-	-	Center for Studies of Population and Social Development (CEPAR) (Ecuador) and Division of Reproductive Health-Centers for Disease Control and Prevention (CDC). (2005) Ecuador Reproductive Health Survey 2004. Quito, Ecuador: CEPAR.	GHDx
153674	Ecuador	Ecuador National Health and Nutrition Survey 2012	2012	I	I	I	I	I	I	-	E	e	I	I	Ministry of Public Health (Ecuador), National Institute of Statistics and Censuses (Ecuador). Ecuador National Health and Nutrition Survey 2012.	GHDx
46924	Ecuador	Ecuador Living Conditions Survey 2005-2006	2005-2006	I	I	I	I	I	I	-	-	I	-	-	National Institute of Statistics and Censuses (Ecuador), Inter-American Development Bank (IDB). Ecuador Living Conditions Survey 2005-2006. Quito, Ecuador: National Institute of Statistics and Censuses (Ecuador).	GHDx
364181	Ecuador	Ecuador Living Conditions Survey 2013-2014	2013-2014	-	I	I	I	I	-	-	E	I	-	I	National Institute of Statistics and Censuses (Ecuador). Ecuador Living Conditions Survey 2013-2014. Quito, Ecuador: National Institute of Statistics and Censuses (Ecuador).	GHDx
224872	Egypt	Egypt National Cluster Survey for Vaccination Coverage 1984	1984	I	I	I	-	-	I	-	-	I	-	-	Egypt National Cluster Survey for Vaccination Coverage 1984.	GHDx
224874	Egypt	Egypt National Cluster Survey for Vaccination Coverage 1987	1987	I	I	I	-	-	I	-	-	I	-	-	United Nations Children's Fund (UNICEF). Egypt National Cluster Survey for Vaccination Coverage 1987.	GHDx
3583	Egypt	Egypt Maternal and Child Health Survey 1991	1991	I	I	I	-	-	I	-	-	I	-	-	Central Agency for Public Mobilization and Statistics (CAPMAS) (Egypt), League of Arab States. Egypt Maternal and Child Health Survey 1991.	GHDx
224903	Egypt	Egypt Expanded Programme on Immunization Survey 1991	1991	I	I	I	-	-	I	-	-	I	-	-	Ministry of Health and Population (Egypt), United Nations Children's Fund (UNICEF). Egypt Expanded Programme on Immunization Survey 1991.	GHDx

GHDx ID	Country	Series	Year	BCG	DTP1	DTP3	HepB3	Hib3	MCV1	MCV2	PCV3	Pol3	RCV1	RotaC	Citation	Link
3585	Egypt	Egypt Multiple Indicator Cluster Survey 1996	1996	I	-	I	I	-	I	-	-	I	-	-	Social Research Centre, American University in Cairo and United Nations Children's Fund (UNICEF). Egypt Multiple Indicator Cluster Survey 1996. New York, United States: United Nations Children's Fund (UNICEF).	GHDx
19511	Egypt	Egypt Demographic and Health Survey 2000	2000	I	I	I	I	-	I	-	-	I	-	-	Macro International, Inc, Population Council (Egypt). Egypt Demographic and Health Survey 2000. Fairfax, United States of America: ICF International.	GHDx
19529	Egypt	Egypt Interim Demographic and Health Survey 2003	2003	I	I	I	I	-	I	-	-	I	I	-	El-Zanaty and Associates, Macro International, Inc, Ministry of Health and Population (Egypt), Population Council (Egypt). Egypt Interim Demographic and Health Survey 2003. Fairfax, United States of America: ICF International.	GHDx
19521	Egypt	Egypt Demographic and Health Survey 2005	2005	I	I	I	I	-	I	-	-	I	I	-	El-Zanaty and Associates, Macro International, Inc, Ministry of Health and Population (Egypt), Population Council (Egypt). Egypt Demographic and Health Survey 2005. Fairfax, United States of America: ICF International.	GHDx
26842	Egypt	Egypt Demographic and Health Survey 2008	2008	I	I	I	I	-	I	-	-	I	I	-	El-Zanaty and Associates, Macro International, Inc, Ministry of Health and Population (Egypt). Egypt Demographic and Health Survey 2008. Fairfax, United States of America: ICF International.	GHDx
154897	Egypt	Egypt Demographic and Health Survey 2014	2014	I	I	I	I	E	I	I	-	I	I	-	El-Zanaty and Associates, ICF International, Ministry of Health and Population (Egypt). Egypt Demographic and Health Survey 2014. Fairfax, United States of America: ICF International.	GHDx
19472	Egypt	Egypt Demographic and Health Survey 1988-1989	1988-1989	I	I	I	-	-	I	-	-	I	-	-	Central Agency for Public Mobilization and Statistics (CAPMAS) (Egypt), Macro Systems, Inc.; Institute for Resource Development, Population Council (Egypt). Egypt Demographic and Health Survey 1988-1989. Fairfax, United States of America: ICF International.	GHDx
19482	Egypt	Egypt Demographic and Health Survey 1992-1993	1992-1993	I	I	I	E	-	I	-	-	I	-	-	Macro International, Inc, Population Council (Egypt). Egypt Demographic and Health Survey 1992-1993. Fairfax, United States of America: ICF International.	GHDx

GHDx ID	Country	Series	Year	BCG	DTP1	DTP3	HepB3	Hib3	MCV1	MCV2	PCV3	Pol3	RCV1	RotaC	Citation	Link
19493	Egypt	Egypt Demographic and Health Survey 1995-1996	1995-1996	I	I	I	E	-	I	-	-	I	-	-	Macro International, Inc, Population Council (Egypt). Egypt Demographic and Health Survey 1995-1996. Fairfax, United States of America: ICF International.	GHDx
19501	Egypt	Egypt Interim Demographic and Health Survey 1997-1998	1997-1998	I	I	I	I	-	I	-	-	I	-	-	El-Zanaty and Associates, Macro International, Inc. Egypt Interim Demographic and Health Survey 1997-1998.	GHDx
27572	El Salvador	El Salvador Family Planning/Maternal and Child Health Survey 1988	1988	I	-	I	-	-	I	-	-	I	-	-	Division of Reproductive Health-Centers for Disease Control and Prevention (CDC). El Salvador Family Planning/Maternal and Child Health Survey 1988. Final English Language Report. Atlanta, United States: Centers for Disease Control and Prevention (CDC).	GHDx
27590	El Salvador	El Salvador Reproductive Health Survey 1998	1998	I	I	I	-	-	I	-	-	I	-	-	El Salvador Demographic Association (ADS), Division of Reproductive Health-Centers for Disease Control and Prevention (CDC). El Salvador Reproductive Health Survey 1998. Atlanta, United States: Centers for Disease Control and Prevention (CDC).	GHDx
229697	El Salvador	El Salvador National Immunization Coverage Survey 2011	2011	I	I	I	I	I	I	-	-	I	-	-	Ministry of Health (El Salvador). El Salvador National Immunization Coverage Survey 2011.	GHDx
200636	El Salvador	El Salvador Multiple Indicator Cluster Survey 2014	2014	I	I	I	I	I	I	-	I	I	-	I	General Administration of Statistics and Censuses (El Salvador), Ministry of Health (El Salvador), United Nations Children's Fund (UNICEF). El Salvador Multiple Indicator Cluster Survey 2014. New York, United States of America: United Nations Children's Fund (UNICEF), 2017.	GHDx
526221*	El Salvador	El Salvador National Health Survey 2021	2021	I	I	I	I	I	I	E	I	I	I	I	General Directorate of Statistics and Census (DIGESTYC) (El Salvador), National Institute of Health (El Salvador). El Salvador National Health Survey 2021.	GHDx
27599	El Salvador	El Salvador Reproductive Health Survey 2002-2003	2002-2003	I	e	e	-	-	e	-	-	I	-	-	Asociación Demográfica Salvadoreña (ADS), Division of Reproductive Health-Centers for Disease Control and Prevention (CDC). (2004) El Salvador Reproductive Health Survey 2002-2003. San Salvador, El Salvador: ADS.	GHDx

GHDx ID	Country	Series	Year	BCG	DTP1	DTP3	HepB3	Hib3	MCV1	MCV2	PCV3	Pol3	RCV1	RotaC	Citation	Link
3655	Equatorial Guinea	Equatorial Guinea Multiple Indicator Cluster Survey 2000	2000	I	I	I	-	-	I	-	-	I	-	-	Ministry of Planning, Economic Development and Public Investment (Equatorial Guinea), United Nations Children's Fund (UNICEF). Equatorial Guinea Multiple Indicator Cluster Survey 2000. New York, United States of America: United Nations Children's Fund (UNICEF).	GHDx
76884	Equatorial Guinea	Equatorial Guinea Demographic and Health Survey 2011	2011	I	I	I	E	E	I	-	-	I	E	-	ICF International, Ministry of Health and Social Welfare (Equatorial Guinea), Ministry of Planning, Economic Development and Public Investment (Equatorial Guinea). Equatorial Guinea Demographic and Health Survey 2011. Fairfax, United States of America: ICF International, 2012.	GHDx
453344	Equatorial Guinea	Equatorial Guinea External Review of Expanded Programme on Immunization National Coverage Survey 2016	2016	I	I	I	I	I	I	-	-	I	-	-	Equatorial Guinea External Review of Expanded Programme on Immunization National Coverage Survey 2016.	GHDx
560285*	Equatorial Guinea	Equatorial Guinea Immunization Coverage Survey Among Children Aged 6-23 Months 2024	2024	I	I	I	I	I	I	I	-	I	-	-	World Health Organization (WHO). Equatorial Guinea Immunization Coverage Survey Among Children Aged 6-23 Months 2024. Geneva, Switzerland: World Health Organization (WHO).	GHDx
224896	Eritrea	Eritrea Health and Nutrition Survey 1993	1993	I	-	I	-	-	I	-	-	I	-	-	Ministry of Health (Eritrea). Eritrea Health and Nutrition Survey 1993.	GHDx
224915	Eritrea	Eritrea EPI Coverage Survey 2000	2000	I	I	I	-	-	I	-	-	I	-	-	Ministry of Health (Eritrea), United Nations Children's Fund (UNICEF). Eritrea EPI Coverage Survey 2000.	GHDx
19539	Eritrea	Eritrea Demographic and Health Survey 2002	2002	I	I	I	-	-	I	-	-	I	-	-	Macro International, Inc, National Statistics and Evaluation Office (Eritrea). Eritrea Demographic and Health Survey 2002. Fairfax, United States of America: ICF International.	GHDx
224928	Eritrea	Eritrea Routine Immunization Coverage Survey 2006	2006	I	I	I	I	-	I	-	-	I	-	-	Ministry of Health (Eritrea), United Nations Children's Fund (UNICEF). Eritrea Routine Immunization Coverage Survey 2006.	GHDx
224936	Eritrea	Eritrea EPI Coverage Survey 2009	2009	I	I	I	I	-	I	-	-	I	-	-	Ministry of Health (Eritrea), United Nations Children's Fund (UNICEF). Eritrea EPI Coverage Survey 2009.	GHDx

GHDx ID	Country	Series	Year	BCG	DTP1	DTP3	HepB3	Hib3	MCV1	MCV2	PCV3	Pol3	RCV1	RotaC	Citation	Link
249999	Eritrea	Eritrea Population and Health Survey 2010	2010	I	I	I	-	-	I	-	-	I	-	-	Kenya Medical Research Institute (KEMRI), National Statistics Office (Eritrea), The Fafo Research Foundation. Eritrea Population and Health Survey 2010.	GHDx
224938	Eritrea	Eritrea National EPI Coverage Survey 2013	2013	-	-	I	I	I	I	-	-	I	-	-	Ministry of Health (Eritrea), United Nations Children's Fund (UNICEF), World Health Organization (WHO). Eritrea National EPI Coverage Survey 2013.	GHDx
482843*	Eritrea	Eritrea National EPI Coverage Survey Report 2020	2020	I	I	I	I	I	I	I	-	I	-	-	Eritrea National EPI Coverage Survey Report 2020.	GHDx
156685	Eritrea	The Health Sector in Eritrea	1990-2000	I	-	I	-	-	I	-	-	I	-	-	Ministry of Health (Eritrea). The Health Sector in Eritrea. Washington DC, United States of America: World Bank, 2004.	GHDx
19546	Eritrea	Eritrea Demographic and Health Survey 1995-1996	1995-1996	I	I	I	-	-	I	-	-	I	-	-	Macro International, Inc, National Statistics Office (Eritrea). Eritrea Demographic and Health Survey 1995-1996. Fairfax, United States of America: ICF International.	GHDx
229639	Eswatini	Swaziland Unicef External Evaluation of Expanded Program on Immunization 1982	1982	I	I	I	-	-	I	-	-	I	-	-	Swaziland Unicef External Evaluation of Expanded Program on Immunization 1982.	GHDx
229638	Eswatini	Swaziland Unicef External Evaluation of Expanded Program on Immunization 1983	1983	I	I	I	-	-	I	-	-	I	-	-	Swaziland Unicef External Evaluation of Expanded Program on Immunization 1983.	GHDx
229636	Eswatini	Swaziland Unicef External Evaluation of Expanded Program on Immunization 1984	1984	I	I	I	-	-	I	-	-	I	-	-	Swaziland Unicef External Evaluation of Expanded Program on Immunization 1984.	GHDx
229635	Eswatini	Swaziland Unicef External Evaluation of Expanded Program on Immunization 1987	1987	I	I	I	-	-	I	-	-	I	-	-	Swaziland Unicef External Evaluation of Expanded Program on Immunization 1987.	GHDx
229634	Eswatini	Swaziland Unicef External Evaluation of Expanded Program on Immunization 1988	1988	I	I	I	-	-	I	-	-	I	-	-	Swaziland Unicef External Evaluation of Expanded Program on Immunization 1988.	GHDx

GHDx ID	Country	Series	Year	BCG	DTP1	DTP3	HepB3	Hib3	MCV1	MCV2	PCV3	Pol3	RCV1	RotaC	Citation	Link
229633	Eswatini	Swaziland Unicef External Evaluation of Expanded Program on Immunization 1989 - Preliminary Data	1989	I	I	I	-	-	I	-	-	I	-	-	Swaziland Unicef External Evaluation of Expanded Program on Immunization 1989 - Preliminary Data.	GHDx
12320	Eswatini	Swaziland Multiple Indicator Cluster Survey 2000	2000	I	I	I	I	-	I	-	-	I	-	-	Central Statistical Office (Swaziland), United Nations Children's Fund (UNICEF). Swaziland Multiple Indicator Cluster Survey 2000. New York, United States of America: United Nations Children's Fund (UNICEF).	GHDx
229631	Eswatini	Swaziland Report on National EPI Review 2003	2003	I	I	I	-	-	I	-	-	I	-	-	Swaziland Report on National EPI Review 2003.	GHDx
229599	Eswatini	Swaziland Measles Post Campaign Evaluation and EPI Coverage Survey 2006	2006	I	I	I	I	-	I	-	-	I	-	-	Swaziland Measles Post Campaign Evaluation and EPI Coverage Survey 2006.	GHDx
141312	Eswatini	Swaziland National Nutrition Survey 2008	2008	-	-	-	-	-	I	-	-	-	-	-	Ministry of Health (Swaziland). Swaziland National Nutrition Survey 2008.	GHDx
30325	Eswatini	Swaziland Multiple Indicator Cluster Survey 2010	2010	I	I	I	I	I	I	-	-	I	-	-	Central Statistical Office (Swaziland), United Nations Children's Fund (UNICEF). Swaziland Multiple Indicator Cluster Survey 2010. New York, United States of America: United Nations Children's Fund (UNICEF).	GHDx
229598	Eswatini	Swaziland Immunization Coverage Survey 2013	2013	I	I	I	I	I	I	I	-	I	-	-	Swaziland Immunization Coverage Survey 2013.	GHDx
200707	Eswatini	Swaziland Multiple Indicator Cluster Survey 2014	2014	I	I	I	I	I	I	I	E	I	-	-	Central Statistical Office (Swaziland), United Nations Children's Fund (UNICEF), United Nations Educational, Scientific and Cultural Organization (UNESCO), United Nations Population Fund (UNFPA). Swaziland Multiple Indicator Cluster Survey 2014. New York, United States of America: United Nations Children's Fund (UNICEF), 2016.	GHDx
12301	Eswatini	Swaziland Family Planning/Maternal and Child Health Survey 1988-1989	1988-1989	I	I	I	-	-	I	-	-	I	-	-	Division of Reproductive Health-Centers for Disease Control and Prevention (CDC) and Ministry of Health. (1989) Swaziland Family Planning/Maternal and Child Health Survey 1988. Atlanta, United States: Centers for Disease Control and Prevention (CDC).	GHDx

GHDx ID	Country	Series	Year	BCG	DTP1	DTP3	HepB3	Hib3	MCV1	MCV2	PCV3	Pol3	RCV1	RotaC	Citation	Link
20829	Eswatini	Swaziland Demographic and Health Survey 2006-2007	2006-2007	I	I	I	I	-	I	-	-	I	-	-	Central Statistical Office (Swaziland), Macro International, Inc. Swaziland Demographic and Health Survey 2006-2007. Fairfax, United States of America: ICF International.	GHDx
543406*	Eswatini	Eswatini Multiple Indicator Cluster Survey 2021-2022	2021-2022	I	I	I	I	I	e	E	I	I	E	I	Central Statistical Office (Eswatini), United Nations Children's Fund (UNICEF). Eswatini Multiple Indicator Cluster Survey 2021-2022. 2023.	GHDx
194013	Ethiopia	Ethiopia Multiple Indicator Cluster Survey 1995	1995	I	-	I	-	-	I	-	-	I	-	-	Ministry of Health (Ethiopia), United Nations Children's Fund (UNICEF). Ethiopia Multiple Indicator Cluster Survey 1995.	GHDx
34047	Ethiopia	Ethiopia Welfare Monitoring Survey 1996	1996	I	-	I	-	-	I	-	-	-	-	-	Central Statistics Agency (Ethiopia). Ethiopia Welfare Monitoring Survey 1996.	GHDx
19571	Ethiopia	Ethiopia Demographic and Health Survey 2000	2000	I	I	I	-	-	I	-	-	I	-	-	Central Statistics Agency (Ethiopia), ORC Macro. Ethiopia Demographic and Health Survey 2000. Calverton, United States of America: ORC Macro, 2001.	GHDx
19557	Ethiopia	Ethiopia Demographic and Health Survey 2005	2005	I	I	I	-	-	I	-	-	I	-	-	Macro International, Inc, Population and Housing Census Commissions Office (PHCCO). Ethiopia Demographic and Health Survey 2005. Fairfax, United States of America: ICF International.	GHDx
225023	Ethiopia	Ethiopia Immunization Coverage Survey 2012	2012	I	I	I	I	I	I	-	-	I	-	-	Ethiopia Immunization Coverage Survey 2012.	GHDx
218568	Ethiopia	Ethiopia Demographic and Health Survey 2016	2016	I	I	I	I	I	I	-	I	I	-	I	Central Statistics Agency (Ethiopia), ICF International. Ethiopia Demographic and Health Survey 2016. Fairfax, United States of America: ICF International, 2017.	GHDx
416582	Ethiopia	Ethiopia Interim Demographic and Health Survey 2019	2019	I	I	I	I	I	I	E	I	I	-	I	Central Statistics Agency (Ethiopia), Ethiopian Public Health Institute (EPHI), ICF International, Ministry of Health (Ethiopia). Ethiopia Interim Demographic and Health Survey 2019.	GHDx
560284*	Ethiopia	Ethiopia National Immunization Program Evaluation Preliminary Service Delivery Coverage Report 2023	2023	I	I	I	I	I	I	I	I	I	-	-	World Health Organization (WHO). Ethiopia National Immunization Program Evaluation Preliminary Service Delivery Coverage Report 2023. Geneva, Switzerland: World Health Organization (WHO).	GHDx
258106	Ethiopia	Ethiopia Health and Health Related Indicators 1999-2000	1999-2000	I	-	I	-	-	I	-	-	-	-	-	Ministry of Health (Ethiopia). Ethiopia Health and Health Related Indicators 1999-2000. Addis Ababa, Ethiopia: Ministry of Health (Ethiopia), 2000.	GHDx

GHDx ID	Country	Series	Year	BCG	DTP1	DTP3	HepB3	Hib3	MCV1	MCV2	PCV3	Pol3	RCV1	RotaC	Citation	Link
258108	Ethiopia	Ethiopia Health and Health Related Indicators 2000-2001	2000-2001	I	-	I	-	-	I	-	-	-	-	-	Ministry of Health (Ethiopia). Ethiopia Health and Health Related Indicators 2000-2001. Addis Ababa, Ethiopia: Ministry of Health (Ethiopia), 2001.	GHDx
21301	Ethiopia	Ethiopia Demographic and Health Survey 2010-2011	2010-2011	I	I	I	E	E	I	-	-	I	-	-	Central Statistics Agency (Ethiopia), ICF Macro, Ministry of Health (Ethiopia). Ethiopia Demographic and Health Survey 2010-2011. Fairfax, United States of America: ICF International.	GHDx
225373	Fiji	Fiji National EPI Survey 1999	1999	I	I	I	I	-	I	-	-	I	-	-	Fiji National EPI Survey 1999.	GHDx
191162	Fiji	Fiji National Immunisation Coverage Survey 2013	2013	I	I	I	I	I	I	-	-	I	I	-	Devi R, Volavola L, Jenkins K, Comrie-Thomson L, Stewart T, Chan G. Fiji National Immunisation Coverage Survey 2013. Fiji J Public Health. 2014; 3(2): 8-13.	GHDx
487664*	Fiji	Fiji Multiple Indicator Cluster Survey 2021	2021	I	I	I	I	I	I	I	I	I	I	I	Fiji Bureau of Statistics, Pacific Community Statistics for Development Division, United Nations Children's Fund (UNICEF), United Nations Population Fund (UNFPA). Fiji Multiple Indicator Cluster Survey 2021. New York, United States of America: United Nations Children's Fund (UNICEF), 2022.	GHDx
90712	Gabon	Gabon Multiple Indicator Cluster Survey 1996	1996	I	I	I	-	-	I	-	-	I	-	-	Ministry of Health and Social Affairs (Gabon), United Nations Children's Fund (UNICEF). Gabon Multiple Indicator Cluster Survey 1996.	GHDx
76706	Gabon	Gabon Demographic and Health Survey 2012	2012	I	I	I	I	E	I	-	-	I	-	-	General Directorate of Statistics (Gabon), ICF International, Ministry of Economy, Employment and Sustainable Development (Gabon), Ministry of Health and Social Affairs (Gabon). Gabon Demographic and Health Survey 2012. Fairfax, United States of America: ICF International, 2013.	GHDx
226776	Gabon	Gabon EPI External Review 2012	2012	I	I	I	I	I	I	-	-	I	-	-	Ministry of Health (Gabon). Gabon EPI External Review 2012.	GHDx
19579	Gabon	Gabon Demographic and Health Survey 2000-2001	2000-2001	I	I	I	E	E	I	-	-	I	-	-	General Directorate of Statistics and Economic Studies (Gabon), Macro International, Inc. Gabon Demographic and Health Survey 2000-2001. Fairfax, United States of America: ICF International.	GHDx

GHDx ID	Country	Series	Year	BCG	DTP1	DTP3	HepB3	Hib3	MCV1	MCV2	PCV3	Pol3	RCV1	RotaC	Citation	Link
529525*	Gabon	Gabon Demographic and Health Survey 2019-2021	2019-2021	I	I	I	I	I	I	-	-	I	-	-	General Directorate of Statistics (Gabon), ICF International, Ministry of Economy, Employment and Sustainable Development (Gabon), Ministry of Health and Social Affairs (Gabon), Ministry of Higher Education and Scientific Research (Gabon). Gabon Demographic and Health Survey 2019-2021. Fairfax, United States of America: ICF International, 2023.	GHDx
226576	Georgia	Georgia EPI Coverage Survey with add on Questions 1996	1996	I	I	I	-	-	I	-	-	I	-	-	Ministry of Health (Georgia), United Nations Children's Fund (UNICEF). Georgia EPI Coverage Survey with add on Questions 1996.	GHDx
3960	Georgia	Georgia Multiple Indicator Cluster Survey 1999	1999	I	I	I	-	-	I	-	-	I	-	-	National Center for Disease Control (Georgia), State Department of Statistics of Georgia, United Nations Children's Fund (UNICEF). Georgia Multiple Indicator Cluster Survey 1999.	GHDx
226577	Georgia	Georgia Immunization Programme Evaluation 1999	1999	I	I	I	-	-	I	-	-	I	-	-	Georgia Immunization Programme Evaluation 1999.	GHDx
19587	Ghana	Ghana Demographic and Health Survey 1988	1988	I	I	I	-	-	I	-	-	I	-	-	Ghana Statistical Service, Macro Systems, Inc.; Institute for Resource Development. Ghana Demographic and Health Survey 1988. Fairfax, United States of America: ICF International.	GHDx
4681	Ghana	Ghana Multiple Indicator Cluster Survey 1995	1995	I	I	I	-	-	I	-	-	I	-	-	Ministry of Health (Ghana), United Nations Children's Fund (UNICEF). Ghana Multiple Indicator Cluster Survey 1995.	GHDx
19627	Ghana	Ghana Demographic and Health Survey 2003	2003	I	I	I	-	-	I	-	-	I	-	-	Ghana Statistical Service, Macro International, Inc. Ghana Demographic and Health Survey 2003. Fairfax, United States of America: ICF International.	GHDx
4694	Ghana	Ghana Multiple Indicator Cluster Survey 2006	2006	I	I	I	-	-	I	-	-	I	E	-	Ministry of Health (MOH) (Ghana), Ghana Statistical Service and United Nations Children's Fund (UNICEF). Ghana Multiple Indicator Cluster Survey 2006. New York, United States: United Nations Children's Fund (UNICEF).	GHDx
21188	Ghana	Ghana Demographic and Health Survey 2008	2008	I	I	I	I	I	I	-	-	I	-	-	Ghana Statistical Service, Macro International, Inc, Ministry of Health (Ghana). Ghana Demographic and Health Survey 2008. Fairfax, United States of America: ICF International.	GHDx

GHDx ID	Country	Series	Year	BCG	DTP1	DTP3	HepB3	Hib3	MCV1	MCV2	PCV3	Pol3	RCV1	RotaC	Citation	Link
63993	Ghana	Ghana Multiple Indicator Cluster Survey 2011	2011	I	I	I	I	I	I	-	-	I	-	-	Centers for Disease Control and Prevention (CDC), Ghana Statistical Service, Government of Japan, ICF Macro, Ministry of Health (Ghana), Navrongo Health Research Centre, United Nations Children's Fund (UNICEF), United Nations Population Fund (UNFPA), United States Agency for International Development (USAID). Ghana Multiple Indicator Cluster Survey 2011. New York, United States of America: United Nations Children's Fund (UNICEF), 2013.	GHDx
227921	Ghana	Ghana EPI Cluster Survey 2012	2012	I	I	I	I	I	I	-	-	I	-	-	Ghana EPI Cluster Survey 2012.	GHDx
157027	Ghana	Ghana Demographic and Health Survey 2014	2014	I	I	I	I	I	I	E	E	I	-	E	Ghana Health Service, Ghana Statistical Service, ICF International. Ghana Demographic and Health Survey 2014. Fairfax, United States of America: ICF International, 2016.	GHDx
19604	Ghana	Ghana Demographic and Health Survey 1993-1994	1993-1994	I	I	I	-	-	I	-	-	I	-	-	Ghana Statistical Service, Macro International, Inc. Ghana Demographic and Health Survey 1993-1994. Fairfax, United States of America: ICF International.	GHDx
19614	Ghana	Ghana Demographic and Health Survey 1998-1999	1998-1999	I	I	I	-	-	I	-	-	I	-	-	Ghana Statistical Service, Macro International, Inc. Ghana Demographic and Health Survey 1998-1999. Fairfax, United States of America: ICF International.	GHDx
160576	Ghana	Ghana District Multiple Indicator Cluster Survey 2007-2008	2007-2008	I	I	I	I	-	I	-	-	I	E	-	Ghana Statistical Service, Ministry of Health (Ghana), United Nations Children's Fund (UNICEF). Ghana District Multiple Indicator Cluster Survey 2007-2008.	GHDx
236205	Ghana	Ghana Socioeconomic Panel Survey 2009-2010	2009-2010	-	e	e	-	-	I	-	-	I	-	-	Economic Growth Center, Yale University, Institute of Statistical, Social and Economic Research, University of Ghana. Ghana Socioeconomic Panel Survey 2009-2010. Washington DC, United States: World Bank.	GHDx

GHDx ID	Country	Series	Year	BCG	DTP1	DTP3	HepB3	Hib3	MCV1	MCV2	PCV3	Pol3	RCV1	RotaC	Citation	Link
437993	Ghana	Ghana Multiple Indicator Cluster Survey 2017-2018	2017-2018	I	I	I	I	I	I	-	I	I	I	I	Centers for Disease Control and Prevention (CDC), Ghana Statistical Service, Government of Japan, ICF Macro, Ministry of Health (Ghana), Navrongo Health Research Centre, United Nations Children's Fund (UNICEF), United Nations Population Fund (UNFPA), United States Agency for International Development (USAID). Ghana Multiple Indicator Cluster Survey 2017-2018. New York, United States of America: United Nations Children's Fund (UNICEF), 2013.	GHDx
527726*	Ghana	Ghana Demographic and Health Survey 2022-2023	2022-2023	I	I	I	I	I	I	I	I	I	I	-	Ghana Statistical Service, ICF International. Ghana Demographic and Health Survey 2022-2023. 2024.	GHDx
19647	Guatemala	Guatemala Demographic and Health Survey 1987	1987	I	I	I	-	-	e	-	-	I	-	-	Institute of Nutrition of Central America and Panama, Westinghouse; Institute for Resource Development. Guatemala Demographic and Health Survey 1987. Fairfax, United States of America: ICF International.	GHDx
19637	Guatemala	Guatemala Demographic and Health Survey 1995	1995	I	I	I	-	-	I	-	-	I	-	-	Macro International, Inc, National Statistics Institute (Guatemala). Guatemala Demographic and Health Survey 1995. Fairfax, United States of America: ICF International.	GHDx
27563	Guatemala	Guatemala Reproductive Health Survey 2002	2002	I	I	I	-	-	I	-	-	I	-	-	Guatemala Ministry of Health and Social Assistance, University of Valle, Division of Reproductive Health-Centers for Disease Control and Prevention (CDC). (2003) Guatemala Reproductive Health Survey 2002. Atlanta, United States: Centers for Disease Control and Prevention (CDC).	GHDx

GHDx ID	Country	Series	Year	BCG	DTP1	DTP3	HepB3	Hib3	MCV1	MCV2	PCV3	Pol3	RCV1	RotaC	Citation	Link
32127	Guatemala	Guatemala National Survey of Living Conditions 2006	2006	I	-	I	E	E	I	-	-	I	-	-	National Institute of Statistics (Guatemala), National Statistical System (Guatemala), World Bank (WB), United Nations Development Programme (UNDP), United Nations Economic Commission for Latin America and the Caribbean (CEPAL), Rafael Landívar University, United Nations Population Fund (UNFPA), Norwegian Agency for Development (NORAD), Swedish International Development Agency (SIDA), Secretary of Planning and Programming (SEGEPLAN) (Guatemala), Bank of Guatemala. Guatemala National Survey of Living Conditions 2006. Guatemala City, Guatemala: National Statistics Institute (Guatemala).	GHDx
19656	Guatemala	Guatemala Interim Demographic and Health Survey 1998-1999	1998-1999	I	I	I	-	-	I	-	-	I	-	-	Guatemala Ministry of Health and Social Assistance, Macro International, Inc, National Statistics Institute (Guatemala). Guatemala Interim Demographic and Health Survey 1998-1999. Fairfax, United States of America: ICF International.	GHDx
4779	Guatemala	Guatemala Reproductive Health Survey 2008-2009	2008-2009	I	I	I	-	-	I	-	-	I	-	-	Guatemala Ministry of Health and Social Assistance, University of Valle and Division of Reproductive Health-Centers for Disease Control and Prevention (CDC). Guatemala Reproductive Health Survey 2008-2009. Atlanta, United States: Centers for Disease Control and Prevention (CDC).	GHDx
157031	Guatemala	Guatemala Demographic and Health Survey 2014-2015	2014-2015	I	I	I	I	I	e	-	E	I	-	I	ICF International, Institute of Nutrition of Central America and Panama, Ministry of Public Health and Social Assistance (Guatemala), National Statistics Institute (Guatemala), Secretary of Planning and Programming of the Presidency (Segeplán) (Guatemala). Guatemala Demographic and Health Survey 2014-2015. Fairfax, United States of America: ICF International, 2017.	GHDx
233733	Guinea	Guinea Combating Childhood Communicable Diseases 1983	1983	I	I	I	-	-	I	-	-	I	-	-	Guinea Combating Childhood Communicable Diseases 1983.	GHDx
19659	Guinea	Guinea Demographic and Health Survey 1992	1992	I	I	I	-	-	I	-	-	I	-	-	Macro International, Inc, National Statistics Directorate (Guinea). Guinea Demographic and Health Survey 1992.	GHDx

GHDx ID	Country	Series	Year	BCG	DTP1	DTP3	HepB3	Hib3	MCV1	MCV2	PCV3	Pol3	RCV1	RotaC	Citation	Link
4787	Guinea	Guinea Multiple Indicator Cluster Survey 1996	1996	I	I	I	-	-	I	-	-	I	-	-	Ministry of Economy, Finance, and Planning (Guinea), National Statistics Directorate (Guinea), United Nations Children's Fund (UNICEF). Guinea Multiple Indicator Cluster Survey 1996.	GHDx
19670	Guinea	Guinea Demographic and Health Survey 1999	1999	I	I	I	-	-	I	-	-	I	-	-	Macro International, Inc, National Statistics Directorate (Guinea). Guinea Demographic and Health Survey 1999. Fairfax, United States of America: ICF International.	GHDx
233728	Guinea	Guinea EPI External Review 2000	2000	I	I	I	-	-	I	-	-	I	-	-	Government of Guinea. Guinea EPI External Review 2000.	GHDx
19683	Guinea	Guinea Demographic and Health Survey 2005	2005	I	I	I	-	-	I	-	-	I	-	-	Macro International, Inc, National Statistics Directorate (Guinea). Guinea Demographic and Health Survey 2005. Fairfax, United States of America: ICF International.	GHDx
233714	Guinea	Guinea EPI External Review 2011	2011	I	I	I	I	I	I	-	-	I	-	-	Ministry of Health and Public Hygiene (Guinea). Guinea EPI External Review 2011.	GHDx
69761	Guinea	Guinea Demographic and Health Survey 2012	2012	I	I	I	-	-	I	-	-	I	-	-	ICF Macro, Ministry of Health and Public Hygiene (Guinea), National Institute of Statistics (Guinea). Guinea Demographic and Health Survey 2012. Fairfax, United States of America: ICF International.	GHDx
303458	Guinea	Guinea Multiple Indicator Cluster Survey 2016	2016	I	I	I	I	I	I	-	-	I	-	-	National Institute of Public Health (NPHI) (Guinea), National Institute of Statistics (Guinea), National Malaria Control Program (Guinea), United Nations Children's Fund (UNICEF). Guinea Multiple Indicator Cluster Survey 2016. New York, United States of America: United Nations Children's Fund (UNICEF), 2018.	GHDx
396957	Guinea	Guinea Demographic and Health Survey 2018	2018	I	I	I	I	I	I	-	-	I	-	-	Ministry of Health (Guinea), Ministry of Planning and Economic Development (Guinea), National Institute of Statistics (Guinea). Guinea Demographic and Health Survey 2018. Fairfax, United States of America: ICF International, 2019.	GHDx
141983	Guinea	Guinea National Survey on Nutritional Status and Tracking Key Indicators of Child Survival 2007-2008	2007-2008	I	I	I	E	-	I	-	-	I	-	-	National Institute of Statistics (Guinea). Guinea National Survey on Nutritional Status and Tracking Key Indicators of Child Survival 2007-2008.	GHDx
233671	Guinea-Bissau	Guinea-Bissau EPI Review 1988	1988	I	I	I	-	-	I	-	-	I	-	-	Guinea-Bissau EPI Review 1988.	GHDx

GHDx ID	Country	Series	Year	BCG	DTP1	DTP3	HepB3	Hib3	MCV1	MCV2	PCV3	Pol3	RCV1	RotaC	Citation	Link
233666	Guinea-Bissau	Guinea-Bissau EPI Review 1994	1994	I	I	I	-	-	I	-	-	I	-	-	Ministry of Public Health (Guinea-Bissau). Guinea-Bissau EPI Review 1994.	GHDx
4808	Guinea-Bissau	Guinea-Bissau Multiple Indicator Cluster Survey 2000	2000	I	e	e	-	-	e	-	-	I	-	-	Secretary State of Planning, National Institute of Statistics and Census (INEC), United Nations Children's Fund (UNICEF). Guinea-Bissau Multiple Indicator Cluster Survey 2000. New York, United States: United Nations Children's Fund (UNICEF).	GHDx
4818	Guinea-Bissau	Guinea-Bissau Multiple Indicator Cluster Survey 2006	2006	I	I	I	-	-	I	-	-	I	-	-	United Nations Children's Fund (UNICEF), Government of Guinea-Bissau. Guinea-Bissau Multiple Indicator Cluster Survey 2006. New York, United States: United Nations Children's Fund (UNICEF).	GHDx
27215	Guinea-Bissau	Guinea-Bissau Multiple Indicator Cluster Survey 2010	2010	I	I	I	-	-	I	-	-	I	-	-	Centers for Disease Control and Prevention (CDC), National Statistics Institute (Guinea-Bissau), United Nations Children's Fund (UNICEF). Guinea-Bissau Multiple Indicator Cluster Survey 2010. New York, United States of America: United Nations Children's Fund (UNICEF), 2018.	GHDx
174049	Guinea-Bissau	Guinea-Bissau Multiple Indicator Cluster Survey 2014	2014	I	I	I	I	I	I	-	-	I	-	-	National Statistics Institute (Guinea-Bissau), United Nations Children's Fund (UNICEF). Guinea-Bissau Multiple Indicator Cluster Survey 2014. New York, United States of America: United Nations Children's Fund (UNICEF), 2016.	GHDx
502015*	Guinea-Bissau	Guinea-Bissau Expanded Program on Immunization Vaccination Coverage Survey 2017	2017	I	I	I	I	I	I	-	I	I	-	I	Ministry of Public Health (Guinea-Bissau), National Statistics Institute (Guinea-Bissau), World Health Organization (WHO). Guinea-Bissau Expanded Program on Immunization Vaccination Coverage Survey 2017.	GHDx
457894*	Guinea-Bissau	Guinea-Bissau Multiple Indicator Cluster Survey 2018-2019	2018-2019	I	I	I	I	I	I	-	I	I	E	I	National Statistics Institute (Guinea-Bissau), United Nations Children's Fund (UNICEF). Guinea-Bissau Multiple Indicator Cluster Survey 2018-2019. New York, United States of America: United Nations Children's Fund (UNICEF), 2020.	GHDx
4916	Guyana	Guyana Multiple Indicator Cluster Survey 2000	2000	I	I	I	-	-	I	-	-	I	I	-	Bureau of Statistics (Guyana), United Nations Children's Fund (UNICEF). Guyana Multiple Indicator Cluster Survey 2000. New York, United States: United Nations Children's Fund (UNICEF)	GHDx

GHDx ID	Country	Series	Year	BCG	DTP1	DTP3	HepB3	Hib3	MCV1	MCV2	PCV3	Pol3	RCV1	RotaC	Citation	Link
21348	Guyana	Guyana Demographic and Health Survey 2009	2009	I	I	I	I	I	I	E	-	I	I	-	Bureau of Statistics (Guyana), ICF Macro, Ministry of Health (Guyana). Guyana Demographic and Health Survey 2009. Fairfax, United States of America: ICF International, 2011.	GHDx
200598	Guyana	Guyana Multiple Indicator Cluster Survey 2014	2014	I	I	I	I	I	I	-	I	I	I	I	Bureau of Statistics (Guyana), Ministry of Health (Guyana), United Nations Children's Fund (UNICEF). Guyana Multiple Indicator Cluster Survey 2014. New York, United States of America: United Nations Children's Fund (UNICEF), 2016.	GHDx
4926	Guyana	Guyana Multiple Indicator Cluster Survey 2006-2007	2006-2007	I	-	-	-	-	I	-	-	I	I	-	United Nations Children's Fund (UNICEF), Bureau of Statistics (Guyana). Guyana Multiple Indicator Cluster Survey 2006. New York, United States: United Nations Children's Fund (UNICEF).	GHDx
474221*	Guyana	Guyana Multiple Indicator Cluster Survey 2019-2020	2019-2020	I	I	I	I	I	I	I	I	e	I	I	Bureau of Statistics (Guyana), Ministry of Health (Guyana), United Nations Children's Fund (UNICEF). Guyana Multiple Indicator Cluster Survey 2019-2020. New York, United States of America: United Nations Children's Fund (UNICEF), 2021.	GHDx
19708	Haiti	Haiti Demographic and Health Survey 2000	2000	I	I	I	-	-	I	-	-	I	-	-	Haitian Institute of Childhood (IHE), Macro International, Inc. Haiti Demographic and Health Survey 2000. Fairfax, United States of America: ICF International.	GHDx
26680	Haiti	Haiti Global Fund Household Survey 2008	2008	I	I	I	-	-	I	-	-	I	-	-	Global Fund to Fight Aids Tuberculosis and Malaria (GFATM). Haiti Global Fund Household Survey 2008.	GHDx
233645	Haiti	Haiti National Vaccination Coverage Survey 2009	2009	I	I	I	-	-	I	-	-	I	-	-	Centers for Disease Control and Prevention (CDC), Ministry of Public Health and Population (Haiti), World Health Organization (WHO). Haiti National Vaccination Coverage Survey 2009.	GHDx
65118	Haiti	Haiti Demographic and Health Survey 2012	2012	I	I	I	-	-	I	-	-	I	I	-	Centers for Disease Control and Prevention (CDC), Haitian Institute of Childhood (IHE), Haitian Institute of Statistics and Informatics, Macro International, Inc. Haiti Demographic and Health Survey 2012. Fairfax, United States of America: ICF International.	GHDx
19695	Haiti	Haiti Demographic and Health Survey 1994-1995	1994-1995	I	I	I	-	-	I	-	-	I	-	-	Haitian Institute of Childhood (IHE), Macro International, Inc. Haiti Demographic and Health Survey 1994-1995. Calverton, United States of America: Macro International, Inc.	GHDx

GHDx ID	Country	Series	Year	BCG	DTP1	DTP3	HepB3	Hib3	MCV1	MCV2	PCV3	Pol3	RCV1	RotaC	Citation	Link
19720	Haiti	Haiti Demographic and Health Survey 2005-2006	2005-2006	I	I	I	-	-	I	-	-	I	-	-	Haitian Institute of Childhood (IHE), Haitian Institute of Statistics and Informatics, Macro International, Inc. Haiti Demographic and Health Survey 2005-2006. Fairfax, United States of America: ICF International.	GHDx
218574	Haiti	Haiti Demographic and Health Survey 2016-2017	2016-2017	I	I	I	I	I	I	-	-	I	-	I	Haitian Institute of Childhood (IHE), Haitian Institute of Statistics and Informatics, ICF International, Ministry of Public Health and Population (Haiti). Haiti Demographic and Health Survey 2016-2017. Fairfax, United States of America: ICF International.	GHDx
5017	Honduras	Honduras Epidemiology and Family Health Survey 1987	1987	I	-	I	-	-	I	-	-	I	-	-	Honduras Family Planning Association (ASHOPLAFA), Minstry of Health (Honduras), Family Health International (FHI). (1989): Honduras Epidemiology and Family Health Survey 1987. Tegucigalpa, Honduras: ASHONPLAFA.	GHDx
27542	Honduras	Honduras Reproductive Health Survey 1996	1996	I	I	I	-	-	I	E	-	I	-	-	Ministry of Health (Honduras), Honduras Family Planning Association (ASHONPLAFA) and Division of Reproductive Health-Centers for Disease Control and Prevention (CDC). (1997) Honduras Reproductive Health Survey 1996. Tegucigalpa, Honduras: ASHONPLAFA.	GHDx
27551	Honduras	Honduras Reproductive Health Survey 2001	2001	I	-	I	-	-	I	-	-	I	-	-	Honduras Family Planning Association (ASHONPLAFA), Ministry of Health (Honduras), and Division of Reproductive Health-Centers for Disease Control and Prevention (CDC). Honduras Reproductive Health Survey 2001. Tegucigalpa, Honduras: Honduras Family Planning Association (ASHONPLAFA).	GHDx
490966*	Honduras	Honduras Multiple Indicator Cluster Survey 2019	2019	I	I	I	I	I	I	E	I	I	I	I	European Union (EU), Government of Canada, Government of Honduras, National Institute of Statistics (Honduras), Secretary of Health (Honduras), United Nations Children's Fund (UNICEF), United Nations Population Fund (UNFPA). Honduras Multiple Indicator Cluster Survey 2019. New York, United States of America: United Nations Children's Fund (UNICEF), 2021.	GHDx

GHDx ID	Country	Series	Year	BCG	DTP1	DTP3	HepB3	Hib3	MCV1	MCV2	PCV3	Pol3	RCV1	RotaC	Citation	Link
19728	Honduras	Honduras Demographic and Health Survey 2005-2006	2005-2006	I	I	I	I	I	I	-	-	I	-	-	Macro International, Inc, National Institute of Statistics (Honduras), Secretary of Health (Honduras). Honduras Demographic and Health Survey 2005-2006. Fairfax, United States of America: ICF International.	GHDx
95440	Honduras	Honduras Demographic and Health Survey 2011-2012	2011-2012	I	I	I	I	I	I	-	E	I	-	E	ICF Macro, National Institute of Statistics (Honduras). Honduras Demographic and Health Survey 2011-2012. Fairfax, United States of America: ICF International.	GHDx
5127	India	India Multiple Indicator Cluster Survey 2000	2000	I	I	I	-	-	I	-	-	I	-	-	United Nations Statistical Division, World Health Organization (WHO), United Nations Educational, Scientific, and Cultural Organization (UNESCO), United Nations Population Fund (UNFPA), World Bank (WB), London School of Hygiene and Tropical Medicine, United Nations Children's Fund (UNICEF). India Multiple Indicator Cluster Survey 2000. New York, United States: United Nations Children's Fund (UNICEF).	GHDx
65418	India	India Coverage Evaluation Survey 2005	2005	I	I	I	-	-	I	-	-	I	-	-	MODE Services (P) Ltd, Ministry of Health and Family Welfare (India), United Nations Children's Fund (UNICEF). India Coverage Evaluation Survey 2005.	GHDx
65420	India	India Coverage Evaluation Survey 2007	2007	I	I	I	-	-	I	-	-	I	-	-	Ministry of Health and Family Welfare (India), United Nations Children's Fund (UNICEF). India Coverage Evaluation Survey 2007.	GHDx
317159	India	India National Health Profile 2017	2017	I	-	I	-	-	I	-	-	I	-	-	Central Bureau of Health Intelligence (India), Ministry of Health and Family Welfare (India). India National Health Profile 2017. New Delhi, India: Central Bureau of Health Intelligence (India), 2017.	GHDx
19787	India	India Demographic and Health Survey 1992-1993	1992-1993	I	I	I	-	-	I	-	-	I	-	-	International Institute for Population Sciences (India). India Demographic and Health Survey 1992-1993. Mumbai, India: International Institute for Population Sciences (India).	GHDx
233610	India	India Evaluation of Routine Immunization Coverage Survey 1997-1998	1997-1998	I	-	I	-	-	I	-	-	I	-	-	Ministry of Health and Family Welfare (India), United Nations Children's Fund (UNICEF). India Evaluation of Routine Immunization Coverage Survey 1997-1998.	GHDx

GHDx ID	Country	Series	Year	BCG	DTP1	DTP3	HepB3	Hib3	MCV1	MCV2	PCV3	Pol3	RCV1	RotaC	Citation	Link
19950	India	India Demographic and Health Survey 1998-1999	1998-1999	I	I	I	-	-	I	-	-	I	-	-	International Institute for Population Sciences (India), Macro International, Inc. India Demographic and Health Survey 1998-1999. Calverton, United States of America: Macro International, Inc.	GHDx
233611	India	India Evaluation of Routine Immunization Coverage Survey 1998-1999	1998-1999	I	I	I	-	-	I	-	-	I	-	-	Ministry of Health and Family Welfare (India), United Nations Children's Fund (UNICEF). India Evaluation of Routine Immunization Coverage Survey 1998-1999.	GHDx
19963	India	India Demographic and Health Survey 2005-2006	2005-2006	I	I	I	-	-	I	-	-	I	-	-	International Institute for Population Sciences (India), Macro International, Inc. India Demographic and Health Survey 2005-2006. Fairfax, United States of America: ICF International.	GHDx
285887	India	India Rapid Survey on Children 2013-2014	2013-2014	I	I	I	I	-	I	-	-	I	-	-	Ministry of Women and Child Development (India). India Rapid Survey on Children 2013-2014 .	GHDx
157050	India	India Demographic and Health Survey 2015-2016	2015-2016	I	I	I	I	-	I	-	-	I	-	-	ICF International, International Institute for Population Sciences (India), Ministry of Health and Family Welfare (India). India Demographic and Health Survey 2015-2016. Fairfax, United States of America: ICF International, 2018.	GHDx
467681*	India	India Demographic and Health Survey 2019-2020	2019-2021	I	I	I	I	I	I	-	-	I	-	I	ICF International, International Institute for Population Sciences (India), Ministry of Health and Family Welfare (India), United States Agency for International Development (USAID). India Demographic and Health Survey 2019-2020. Fairfax, United States of America: ICF International, 2022.	GHDx
233632	Indonesia	Indonesia Expanded Program on Immunization 1978	1978	I	I	-	-	-	-	-	-	-	-	-	Indonesia Expanded Program on Immunization 1978.	GHDx
19979	Indonesia	Indonesia Demographic and Health Survey 1991	1991	I	I	I	-	-	I	-	-	I	-	-	Central Bureau of Statistics (Indonesia), Macro International, Inc, Ministry of Health (Indonesia), National Family Planning Coordinating Board (Indonesia). Indonesia Demographic and Health Survey 1991. Fairfax, United States of America: ICF International.	GHDx

GHDx ID	Country	Series	Year	BCG	DTP1	DTP3	HepB3	Hib3	MCV1	MCV2	PCV3	Pol3	RCV1	RotaC	Citation	Link
19990	Indonesia	Indonesia Demographic and Health Survey 1994	1994	I	I	I	-	-	I	-	-	I	-	-	Central Bureau of Statistics (Indonesia), Macro International, Inc, Ministry of Health (Indonesia), National Family Planning Coordinating Board (Indonesia). Indonesia Demographic and Health Survey 1994. Fairfax, United States of America: ICF International.	GHDx
141911	Indonesia	Indonesia Multiple Indicator Cluster Survey 1995	1995	I	-	I	-	-	I	-	-	I	-	-	Central Bureau of Statistics (Indonesia), United Nations Children's Fund (UNICEF). Indonesia Multiple Indicator Cluster Survey 1995.	GHDx
19999	Indonesia	Indonesia Demographic and Health Survey 1997	1997	I	I	I	-	-	I	-	-	I	-	-	Central Bureau of Statistics (Indonesia), Macro International, Inc, Ministry of Health (Indonesia), National Family Planning Coordinating Board (Indonesia). Indonesia Demographic and Health Survey 1997. Fairfax, United States of America: ICF International.	GHDx
233625	Indonesia	Indonesia NID and Routine Coverage Survey 2002	2002	I	I	I	I	-	I	-	-	I	-	-	Indonesia NID and Routine Coverage Survey 2002.	GHDx
20021	Indonesia	Indonesia Demographic and Health Survey 2007	2007	I	I	I	I	-	I	-	-	I	-	-	Macro International, Inc, Ministry of Health (Indonesia), National Family Planning Coordinating Board (Indonesia), Statistics Indonesia. Indonesia Demographic and Health Survey 2007. Fairfax, United States of America: ICF International.	GHDx
114622	Indonesia	Indonesia Basic Health Research 2010	2010	I	-	I	I	-	I	-	-	I	-	-	Agency of Health Research and Development (Indonesia). Indonesia Basic Health Research 2010.	GHDx
76705	Indonesia	Indonesia Demographic and Health Survey 2012	2012	I	I	I	I	-	I	-	-	I	-	-	ICF International, Ministry of Health (Indonesia), National Population and Family Planning Board (Indonesia), Statistics Indonesia. Indonesia Demographic and Health Survey 2012. Fairfax, United States of America: ICF International.	GHDx
286781	Indonesia	Indonesia Demographic and Health Survey 2017	2017	I	I	I	I	I	I	-	-	I	-	-	Ministry of Health (Indonesia), National Population and Family Planning Board (Indonesia), Statistics Indonesia. Indonesia Demographic and Health Survey 2017. Fairfax, United States of America: ICF International, 2019.	GHDx

GHDx ID	Country	Series	Year	BCG	DTP1	DTP3	HepB3	Hib3	MCV1	MCV2	PCV3	Pol3	RCV1	RotaC	Citation	Link
395694	Indonesia	Indonesia National Socioeconomic Survey 2017	2017	I	I	I	I	-	I	-	-	I	E	-	Central Bureau of Statistics (Indonesia). Indonesia National Socioeconomic Survey 2017. Jakarta, Indonesia: Central Bureau of Statistics (Indonesia), 2018.	GHDx
20011	Indonesia	Indonesia Demographic and Health Survey 2002-2003	2002-2003	I	I	I	I	-	I	-	-	I	-	-	Macro International, Inc, Ministry of Health (Indonesia), National Family Planning Coordinating Board (Indonesia), Statistics Indonesia. Indonesia Demographic and Health Survey 2002-2003. Fairfax, United States of America: ICF International.	GHDx
6464	Indonesia	Indonesia Family Life Survey 2007-2008	2007-2008	I	I	I	I	-	I	-	-	I	-	-	Center for Population and Policy Studies, Gadjah Mada University (Indonesia), RAND Corporation, SurveyMETER. Indonesia Family Life Survey 2007-2008. Santa Monica, United States of America: RAND Corporation.	GHDx
233624	Indonesia	Indonesia Immunization Coverage Survey 2007-2008	2007-2008	I	I	I	I	-	I	-	-	I	-	-	United Nations Children's Fund (UNICEF), World Health Organization (WHO). Indonesia Immunization Coverage Survey 2007-2008.	GHDx
264956	Indonesia	Indonesia Family Life Survey 2014-2015	2014-2015	I	I	I	I	-	I	-	-	I	-	-	RAND Corporation, SurveyMETER. Indonesia Family Life Survey 2014-2015. Santa Monica, United States of America: RAND Corporation, 2016.	GHDx
95514	Iran	Iran Multiple Indicator Cluster Survey 1997	1997	I	-	I	-	-	I	-	-	I	-	-	Ministry of Health and Medical Education (Iran), Statistical Centre of Iran, United Nations Children's Fund (UNICEF). Iran Multiple Indicator Cluster Survey 1997.	GHDx
81416	Iran	Iran Multiple Indicator Demographic and Health Survey 2010	2010	I	I	I	I	-	I	E	-	I	I	-	Ministry of Health and Medical Education (Iran), Statistical Centre of Iran. Iran Multiple Indicator Demographic and Health Survey 2010.	GHDx
453348*	Iran	Iran Final Report of the Study of the Immunization Coverage of 24 to 36 Months Old Children 2019	2019	I	I	I	I	I	I	I	-	I	I	-	Iran Final Report of the Study of the Immunization Coverage of 24 to 36 Months Old Children 2019.	GHDx
232992	Iraq	Iraq Immunization Coverage Survey 1986	1986	-	I	I	-	-	I	-	-	I	-	-	Iraq Immunization Coverage Survey 1986.	GHDx
232989	Iraq	Iraq Evaluation of Immunization Coverage and Diarrhoeal Diseases Program 1987	1987	I	I	I	-	-	I	-	-	I	-	-	Iraq Evaluation of Immunization Coverage and Diarrhoeal Diseases Program 1987.	GHDx

GHDx ID	Country	Series	Year	BCG	DTP1	DTP3	HepB3	Hib3	MCV1	MCV2	PCV3	Pol3	RCV1	RotaC	Citation	Link
232988	Iraq	Iraq Immunization Coverage Survey 1988	1988	I	I	I	-	-	I	-	-	I	-	-	Iraq Immunization Coverage Survey 1988.	GHDx
7018	Iraq	Iraq Immunization, Diarrheal Disease, Maternal and Childhood Mortality Survey 1990	1990	I	I	I	-	-	I	-	-	I	-	-	Ministry of Health (Iraq), Ministry of Planning (Iraq), United Nations Children's Fund (UNICEF). Iraq Immunization, Diarrheal Disease, Maternal and Childhood Mortality Survey 1990.	GHDx
7054	Iraq	Iraq Multiple Indicator Cluster Survey 2000	2000	I	I	I	-	-	I	-	-	I	-	-	Central Statistical Organization (Iraq), United Nations Children's Fund (UNICEF). Iraq Multiple Indicator Cluster Survey 2000. New York, United States: United Nations Children's Fund (UNICEF).	GHDx
7028	Iraq	Iraq Multiple Indicator Cluster Survey 2006	2006	I	I	I	I	-	I	-	-	I	I	-	United Nations Children's Fund (UNICEF), Central Organization for Statistics and Information Technology (Iraq), Kurdistan Regional Statistics Office. Iraq Multiple Indicator Cluster Survey 2006. New York, United States: United Nations Children's Fund (UNICEF).	GHDx
76707	Iraq	Iraq Multiple Indicator Cluster Survey 2011	2011	I	I	I	I	-	I	-	-	I	I	-	Central Organization for Statistics and Information Technology (Iraq), Kurdistan Regional Statistics Office, Ministry of Health (Iraq), United Nations Children's Fund (UNICEF). Iraq Multiple Indicator Cluster Survey 2011. New York, United States of America: United Nations Children's Fund (UNICEF), 2013.	GHDx
385708	Iraq	Iraq Multiple Indicator Cluster Survey 2018	2018	I	I	I	I	I	I	I	E	I	I	-	Central Statistical Organization (Iraq), United Nations Children's Fund (UNICEF). Iraq Multiple Indicator Cluster Survey 2018. New York, United States of America: United Nations Children's Fund (UNICEF), 2019.	GHDx
337359	Iraq	Situation Analysis of Children and Women in Iraq	1960-1999	I	I	I	-	-	I	-	-	I	-	-	United Nations Children's Fund (UNICEF). Situation Analysis of Children and Women in Iraq. 1998.	GHDx
34524	Iraq	Iraq Household Socioeconomic Survey 2006-2007	2006-2007	I	I	I	-	-	-	-	-	I	-	-	Central Organization for Statistics and Information Technology (Iraq), Kurdistan Regional Statistics Office, World Bank. Iraq Household Socioeconomic Survey 2006-2007. Washington DC, United States of America: World Bank.	GHDx

GHDx ID	Country	Series	Year	BCG	DTP1	DTP3	HepB3	Hib3	MCV1	MCV2	PCV3	Pol3	RCV1	RotaC	Citation	Link
232977	Italy	Italy National Children Vaccination Coverage Survey 1998	1998	-	-	I	I	-	I	-	-	I	-	-	National Institute of Health (Italy). Italy National Children Vaccination Coverage Survey 1998.	GHDx
232970	Italy	Italy National Infant Vaccination Coverage Survey 2003	2003	-	-	I	I	I	I	-	-	I	-	-	National Institute of Health (Italy). Italy National Infant Vaccination Coverage Survey 2003.	GHDx
45852	Jamaica	Jamaica Survey of Living Conditions 1998	1998	-	I	I	-	-	-	-	-	I	-	-	Planning Institute of Jamaica, Statistical Institute of Jamaica. Jamaica Survey of Living Conditions 1998.	GHDx
7222	Jamaica	Jamaica Survey of Living Conditions 2001	2001	-	I	I	-	-	-	-	-	I	-	-	Planning Institute of Jamaica, Statistical Institute of Jamaica. Jamaica Survey of Living Conditions 2001.	GHDx
7149	Jamaica	Jamaica Multiple Indicator Cluster Survey 2005	2005	I	I	I	-	-	I	-	-	I	-	-	Statistical Institute of Jamaica (STATIN) and United Nations Children's Fund (UNICEF). Jamaica Multiple Indicator Cluster Survey 2005. New York, United States: United Nations Children's Fund (UNICEF).	GHDx
141336	Jamaica	Jamaica Multiple Indicator Cluster Survey 2011	2011	I	I	I	E	E	e	-	-	I	-	-	Statistical Institute of Jamaica, United Nations Children's Fund (UNICEF). Jamaica Multiple Indicator Cluster Survey 2011. New York, United States of America: United Nations Children's Fund (UNICEF), 2015.	GHDx
550789*	Jamaica	Jamaica Multiple Indicator Cluster Survey 2022	2022	I	I	I	I	I	I	E	-	I	I	-	Inter-American Development Bank (IDB), Planning Institute of Jamaica, Statistical Institute of Jamaica, United Nations Children's Fund (UNICEF). Jamaica Multiple Indicator Cluster Survey 2022. New York, United States of America: United Nations Children's Fund (UNICEF), 2024.	GHDx
7276	Jordan	Jordan Family Planning/Maternal and Child Health Survey 1983	1983	-	I	I	-	-	I	-	-	I	-	-	Jordan Department of Statistics and Division of Reproductive Health-Centers for Disease Control and Prevention (CDC). (1984) Jordan Family Planning/Maternal and Child Health Survey 1983. Atlanta, United States: Centers for Disease Control and Prevention (CDC).	GHDx
7274	Jordan	Jordan EPI/CDD and Child Mortality Survey 1990	1990	-	I	I	-	-	I	-	-	-	-	-	Ministry of Health (Jordan), United Nations Children's Fund (UNICEF). Jordan EPI/CDD and Child Mortality Survey 1990.	GHDx

GHDx ID	Country	Series	Year	BCG	DTP1	DTP3	HepB3	Hib3	MCV1	MCV2	PCV3	Pol3	RCV1	RotaC	Citation	Link
20051	Jordan	Jordan Demographic and Health Survey 1990	1990	I	I	I	-	-	I	-	-	I	-	-	Department of Statistics (Jordan), Institute for Resource Development, Macro Systems, Ministry of Planning (Jordan). Jordan Demographic and Health Survey 1990. Fairfax, United States of America: ICF International.	GHDx
20060	Jordan	Jordan Demographic and Health Survey 1997	1997	I	I	I	I	-	I	E	-	I	-	-	Department of Statistics (Jordan), Macro International, Inc. Jordan Demographic and Health Survey 1997. Fairfax, United States of America: ICF International.	GHDx
20073	Jordan	Jordan Demographic and Health Survey 2002	2002	I	I	I	I	E	I	I	-	I	E	-	Department of Statistics (Jordan), Macro International, Inc. Jordan Demographic and Health Survey 2002. Fairfax, United States of America: ICF International.	GHDx
20083	Jordan	Jordan Demographic and Health Survey 2007	2007	I	I	I	I	I	I	-	-	I	I	-	Department of Statistics (Jordan), Macro International, Inc. Jordan Demographic and Health Survey 2007. Fairfax, United States of America: ICF International.	GHDx
77517	Jordan	Jordan Demographic and Health Survey 2012	2012	I	I	I	I	I	I	-	-	I	I	-	Department of Statistics (Jordan), ICF International. Jordan Demographic and Health Survey 2012. Fairfax, United States of America: ICF International.	GHDx
560560*	Jordan	Jordan Demographic and Health Survey 2023	2023	I	I	I	I	I	I	I	E	I	I	I	Department of Statistics (Jordan), ICF International. Jordan Demographic and Health Survey 2023. 2024.	GHDx
356955	Jordan	Jordan Demographic and Health Survey 2017-2018	2017-2018	I	I	I	I	I	I	I	-	I	-	I	Department of Statistics (Jordan), ICF International. Jordan Demographic and Health Survey 2017-2018. Fairfax, United States of America: ICF International, 2019.	GHDx
20092	Kazakhstan	Kazakhstan Demographic and Health Survey 1995	1995	I	I	I	-	-	I	-	-	I	-	-	Macro International, Inc, National Institute of Nutrition (Kazakhstan). Kazakhstan Demographic and Health Survey 1995. Fairfax, United States of America: ICF International.	GHDx
20103	Kazakhstan	Kazakhstan Demographic and Health Survey 1999	1999	I	I	I	E	-	I	-	-	I	-	-	Academy of Preventive Medicine (Kazakhstan), Macro International, Inc. Kazakhstan Demographic and Health Survey 1999. Fairfax, United States of America: ICF International.	GHDx

GHDx ID	Country	Series	Year	BCG	DTP1	DTP3	HepB3	Hib3	MCV1	MCV2	PCV3	Pol3	RCV1	RotaC	Citation	Link
7340	Kazakhstan	Kazakhstan Multiple Indicator Cluster Survey 2006	2006	e	I	I	I	-	I	-	-	I	-	-	Agency of the Republic of Kazakhstan on Statistics and United Nations Children's Fund (UNICEF). Kazakhstan Multiple Indicator Cluster Survey 2006. New York, United States: United Nations Children's Fund (UNICEF).	GHDx
260403	Kazakhstan	Kazakhstan Multiple Indicator Cluster Survey 2015	2015	I	I	I	I	I	I	-	-	I	I	-	Ministry of National Economy (Kazakhstan), United Nations Children's Fund (UNICEF). Kazakhstan Multiple Indicator Cluster Survey 2015. New York, United States of America: United Nations Children's Fund (UNICEF), 2017.	GHDx
76702	Kazakhstan	Kazakhstan Multiple Indicator Cluster Survey 2010-2011	2010-2011	I	I	I	E	-	I	-	-	I	I	-	Agency of the Republic of Kazakhstan on Statistics, United Nations Children's Fund (UNICEF). Kazakhstan Multiple Indicator Cluster Survey 2010-2011. New York, United States of America: United Nations Children's Fund (UNICEF), 2013.	GHDx
232815	Kenya	Kenya National Immunization Coverage Survey 1987	1987	-	I	I	-	-	I	-	-	I	-	-	Kenya National Immunization Coverage Survey 1987.	GHDx
232807	Kenya	Kenya EPI National Immunization Coverage Survey 1992	1992	I	I	I	-	-	I	-	-	I	-	-	Kenya EPI National Immunization Coverage Survey 1992.	GHDx
20120	Kenya	Kenya Demographic and Health Survey 1993	1993	I	I	I	-	-	I	-	-	I	-	-	Central Bureau of Statistics (Kenya), Macro International, Inc, National Council for Population Development (NCPD). Kenya Demographic and Health Survey 1993. Fairfax, United States of America: ICF International.	GHDx
20132	Kenya	Kenya Demographic and Health Survey 1998	1998	I	I	I	-	-	I	-	-	I	-	-	Central Bureau of Statistics (Kenya), Macro International, Inc, National Council for Population Development (NCPD). Kenya Demographic and Health Survey 1998. Fairfax, United States of America: ICF International.	GHDx
7387	Kenya	Kenya Multiple Indicator Cluster Survey 2000	2000	I	I	I	-	-	I	-	-	I	-	-	Central Bureau of Statistics (Kenya), United Nations Children's Fund (UNICEF). Kenya Multiple Indicator Cluster Survey 2000. New York, United States: United Nations Children's Fund (UNICEF).	GHDx

GHDx ID	Country	Series	Year	BCG	DTP1	DTP3	HepB3	Hib3	MCV1	MCV2	PCV3	Pol3	RCV1	RotaC	Citation	Link
20145	Kenya	Kenya Demographic and Health Survey 2003	2003	I	I	I	-	-	I	-	-	I	-	-	Centers for Disease Control and Prevention (CDC), Central Bureau of Statistics (Kenya), Macro International, Inc, Ministry of Health (Kenya), National Council for Population and Development (Kenya). Kenya Demographic and Health Survey 2003. Fairfax, United States of America: ICF International.	GHDx
157057	Kenya	Kenya Demographic and Health Survey 2014	2014	I	I	I	I	I	I	-	E	I	-	-	ICF International, Kenya Medical Research Institute (KEMRI), Kenya National Bureau of Statistics, Ministry of Health (Kenya), National AIDS Control Council (Kenya), National Council for Population and Development (Kenya). Kenya Demographic and Health Survey 2014. Fairfax, United States of America: ICF International.	GHDx
448112	Kenya	Kenya Post Measles-Rubella Supplementary Immunization Activities Coverage Survey 2016	2016	-	-	-	-	-	I	-	-	-	-	-	Centers for Disease Control and Prevention (CDC), Kenya National Bureau of Statistics, Ministry of Health (Kenya), World Health Organization (WHO). Kenya Post Measles-Rubella Supplementary Immunization Activities Coverage Survey 2016.	GHDx
529017*	Kenya	Kenya Demographic and Health Survey 2022	2022	I	I	I	I	I	I	I	I	I	-	I	ICF International, Kenya National Bureau of Statistics, Ministry of Health (Kenya). Kenya Demographic and Health Survey 2022. Fairfax, United States of America: ICF International, 2023.	GHDx
20109	Kenya	Kenya Demographic and Health Survey 1988-1989	1988-1989	I	I	I	-	-	I	-	-	I	-	-	Macro Systems, Inc.; Institute for Resource Development, National Council for Population Development (NCPD). Kenya Demographic and Health Survey 1988-1989. Fairfax, United States of America: ICF International.	GHDx
7375	Kenya	Kenya Integrated Household Budget Survey 2005-2006	2005-2006	I	I	I	-	-	I	-	-	I	-	-	Central Bureau of Statistics (Kenya), UK Department for International Development (DFID), United States Agency for International Development (USAID), European Union (EU), Danish International Development Agency (DANIDA), World Bank (WB), United Nations Development Programme (UNDP). Kenya Integrated Household Budget Survey 2005-2006. Nairobi, Kenya: Central Bureau of Statistics (Kenya).	GHDx

GHDx ID	Country	Series	Year	BCG	DTP1	DTP3	HepB3	Hib3	MCV1	MCV2	PCV3	Pol3	RCV1	RotaC	Citation	Link
49586	Kenya	Kenya Integrated Household Budget Survey Tabular Data 2005-2006	2005-2006	I	-	-	-	-	I	-	-	e	-	-	Kenya National Bureau of Statistics. Kenya Integrated Household Budget Survey Tabular Data 2005-2006. Nairobi, Kenya: Kenya National Bureau of Statistics.	GHDx
21365	Kenya	Kenya Demographic and Health Survey 2008-2009	2008-2009	I	I	I	I	I	I	-	-	I	-	-	ICF Macro, Kenya Medical Research Institute (KEMRI), Kenya National Bureau of Statistics, Ministry of Public Health and Sanitation (Kenya), National AIDS and STI Control Programme (NASCOP) (Kenya), National Association of County and City Health Officials (NACCHO) (United States), National Coordinating Agency for Population and Development (Kenya). Kenya Demographic and Health Survey 2008-2009. Fairfax, United States of America: ICF International.	GHDx
287198	Kenya	Kenya Integrated Household Budget Survey 2015-2016	2015-2016	-	-	-	-	-	I	E	-	-	-	-	Kenya National Bureau of Statistics. 2015-2016 Kenya Integrated Household Budget Survey. Nairobi, Kenya: Kenya National Bureau of Statistics, 2015.	GHDx
135533	Kiribati	Kiribati Demographic and Health Survey 2009	2009	I	I	I	-	-	I	-	-	I	-	-	Kiribati National Statistics Office, Secretariat of the Pacific Community (SPC). Kiribati Demographic and Health Survey 2009.	GHDx
438014	Kiribati	Kiribati Multiple Indicator Cluster Survey 2018-2019	2018-2019	I	I	I	I	I	I	-	I	I	I	-	Kiribati National Statistics Office, Ministry of Health and Medical Services (Kiribati), Secretariat of the Pacific Community (SPC), United Nations Children's Fund (UNICEF), United Nations Population Fund (UNFPA). Kiribati Multiple Indicator Cluster Survey 2018-2019. New York, United States of America: United Nations Children's Fund (UNICEF), 2020.	GHDx
7503	Kuwait	Kuwait Child Health Survey 1987	1987	e	-	I	-	-	I	-	-	I	-	-	Health Ministers' Council for GCC States, Ministry of Health (Kuwait). Kuwait Child Health Survey 1987.	GHDx
7504	Kuwait	Kuwait Family Health Survey 1996	1996	e	I	I	-	-	I	-	-	I	-	-	Health Ministers' Council for GCC States, Ministry of Health (Kuwait), United Nations Statistics Division (UNSD). Kuwait Family Health Survey 1996.	GHDx
232531	Kuwait	Kuwait Vaccine Assessment Survey 1998	1998	-	-	I	-	-	I	-	-	I	-	-	Kuwait Vaccine Assessment Survey 1998.	GHDx

GHDx ID	Country	Series	Year	BCG	DTP1	DTP3	HepB3	Hib3	MCV1	MCV2	PCV3	Pol3	RCV1	RotaC	Citation	Link
90716	Kyrgyzstan	Kyrgyzstan Multiple Indicator Cluster Survey 1995	1995	I	-	I	-	-	I	-	-	I	-	-	Government of Kyrgyzstan, Ministry of Health (Kyrgyzstan), United Nations Children's Fund (UNICEF). Kyrgyzstan Multiple Indicator Cluster Survey 1995.	GHDx
20154	Kyrgyzstan	Kyrgyzstan Demographic and Health Survey 1997	1997	I	I	I	-	-	I	-	-	I	-	-	Macro International, Inc, Ministry of Health (Kyrgyzstan), Research Institute of Obstetrics and Pediatrics (Kyrgyzstan). Kyrgyzstan Demographic and Health Survey 1997. Fairfax, United States of America: ICF International.	GHDx
77518	Kyrgyzstan	Kyrgyzstan Demographic and Health Survey 2012	2012	I	I	I	I	E	I	-	-	I	-	-	ICF International, Ministry of Health (Kyrgyzstan), National Statistical Committee of the Kyrgyz Republic. Kyrgyzstan Demographic and Health Survey 2012. Fairfax, United States of America: ICF International.	GHDx
162283	Kyrgyzstan	Kyrgyzstan Multiple Indicator Cluster Survey 2014	2014	e	I	I	I	I	e	-	-	I	-	-	National Statistical Committee of the Kyrgyz Republic, United Nations Children's Fund (UNICEF). Kyrgyzstan Multiple Indicator Cluster Survey 2014. New York, United States of America: United Nations Children's Fund (UNICEF), 2015.	GHDx
408226	Kyrgyzstan	Kyrgyzstan Multiple Indicator Cluster Survey 2018	2018	I	I	I	I	I	I	-	I	I	I	-	National Statistical Committee of the Kyrgyz Republic, United Nations Children's Fund (UNICEF). Kyrgyzstan Multiple Indicator Cluster Survey 2018. New York, United States of America: United Nations Children's Fund (UNICEF), 2019.	GHDx
7540	Kyrgyzstan	Kyrgyzstan Multiple Indicator Cluster Survey 2005-2006	2005-2006	I	e	e	-	-	I	-	-	e	-	-	United Nations Children's Fund (UNICEF), National Statistical Committee of the Kyrgyz Republic. Kyrgyzstan Multiple Indicator Cluster Survey 2005-2006. New York, United States: United Nations Children's Fund (UNICEF).	GHDx
7603	Laos	Laos Multiple Indicator Cluster Survey 1996	1996	I	-	I	-	-	I	-	-	I	-	-	Ministry of Education and Sports (Laos), Ministry of Health (Laos), National Statistical Center (Laos), United Nations Children's Fund (UNICEF). Laos Multiple Indicator Cluster Survey 1996. New York, United States of America: United Nations Children's Fund (UNICEF).	GHDx

GHDx ID	Country	Series	Year	BCG	DTP1	DTP3	HepB3	Hib3	MCV1	MCV2	PCV3	Pol3	RCV1	RotaC	Citation	Link
7618	Laos	Laos Multiple Indicator Cluster Survey 2000	2000	I	I	I	-	-	I	-	-	I	-	-	Ministry of Health (Laos), National Institute of Public Health (NIOPH), National Statistical Center (Laos), United Nations Children's Fund (UNICEF). Laos Multiple Indicator Cluster Survey 2000. New York, United States of America: United Nations Children's Fund (UNICEF).	GHDx
7629	Laos	Laos Multiple Indicator Cluster Survey 2006	2006	I	I	I	-	-	I	-	-	I	-	-	United Nations Children's Fund (UNICEF), Department of Statistics (Laos), Ministry of Health (Laos). Laos Multiple Indicator Cluster Survey 2006. New York, United States: United Nations Children's Fund (UNICEF).	GHDx
448337	Laos	Laos National Immunization Survey 2015	2015	I	I	I	I	I	I	-	-	I	I	-	Ministry of Health (Cambodia), World Health Organization (WHO). Laos National Immunization Survey 2015.	GHDx
375362	Laos	Laos Multiple Indicator Cluster Survey 2017	2017	I	I	I	I	I	I	-	I	I	I	-	Lao Statistics Bureau, Ministry of Education and Sports (Laos), Ministry of Health (Laos), United Nations Children's Fund (UNICEF). Laos Multiple Indicator Cluster Survey 2017. New York, United States of America: United Nations Children's Fund (UNICEF), 2018.	GHDx
543123*	Laos	Laos Multiple Indicator Cluster Survey 2023	2023	I	-	I	I	I	-	I	I	I	I	-	Lao Statistics Bureau, United Nations Children's Fund (UNICEF). Laos Multiple Indicator Cluster Survey 2023.	GHDx
103973	Laos	Laos Multiple Indicator Cluster Survey 2011-2012	2011-2012	I	I	I	I	I	I	-	-	I	-	-	Ministry of Education and Sports (Laos), Ministry of Health (Laos), Ministry of Planning and Investment (Laos). Laos Multiple Indicator Cluster Survey 2011-2012. New York, United States of America: United Nations Children's Fund (UNICEF), 2013.	GHDx
194100	Latvia	Latvia Maternal and Infant Health Care 2008	1950-2008	I	-	-	-	-	-	-	-	-	-	-	Ministry of Health of the Republic of Latvia. Latvia Maternal and Infant Health Care 2008. Riga, Latvia: Ministry of Health of the Republic of Latvia, 2009.	GHDx
232525	Lebanon	Lebanon National EPI CDD IMR Survey 1990	1990	-	-	I	-	-	I	-	-	I	-	-	Lebanon National EPI CDD IMR Survey 1990.	GHDx
232520	Lebanon	Lebanon National Immunization Coverage Survey 1992	1992	-	I	I	-	-	I	-	-	I	-	-	Lebanon National Immunization Coverage Survey 1992.	GHDx

GHDx ID	Country	Series	Year	BCG	DTP1	DTP3	HepB3	Hib3	MCV1	MCV2	PCV3	Pol3	RCV1	RotaC	Citation	Link
232519	Lebanon	Lebanon National Health Indicators of Children Survey 1994	1994	-	I	I	-	-	I	-	-	I	-	-	Lebanon National Health Indicators of Children Survey 1994.	GHDx
232518	Lebanon	Lebanon National EPI/CDD Survey 1996	1996	-	I	I	-	-	I	-	-	I	-	-	Ministry of Public Health (Lebanon), United Nations Children's Fund (UNICEF). Lebanon National EPI/CDD Survey 1996.	GHDx
7678	Lebanon	Lebanon Multiple Indicator Cluster Survey 2000	2000	-	-	I	-	-	I	-	-	I	I	-	Central Administration of Statistics (Lebanon), United Nations Children's Fund (UNICEF). Lebanon Multiple Indicator Cluster Survey 2000. New York, United States: United Nations Children's Fund (UNICEF).	GHDx
44861	Lebanon	Lebanon Family Health Survey 2004	2004	-	I	I	I	-	I	-	-	I	I	-	Central Administration of Statistics (Lebanon), League of Arab States, Ministry of Social Affairs (Lebanon), Pan Arab Project for Family Health (PAPFAM). Lebanon Family Health Survey 2004.	GHDx
138112	Lebanon	Lebanon Multiple Indicator Cluster Survey 2009	2009	-	I	I	I	I	I	-	-	I	-	-	Central Administration of Statistics (Lebanon). Lebanon Multiple Indicator Cluster Survey 2009.	GHDx
401878	Lebanon	Vaccination coverage in Lebanon following the Syrian crisis: results from the district-based immunization coverage evaluation survey 2016	2016	-	I	I	I	I	I	I	-	I	-	-	Mansour Z, Hamadeh R, Rady A, Danovaro-Holliday MC, Fahmy K, Said R, Brandt L, Warrak R, Ammar W. Vaccination coverage in Lebanon following the Syrian crisis: results from the district-based immunization coverage evaluation survey 2016. BMC Public Health. 2019; 19(1): 58.	GHDx
232403	Lesotho	Lesotho EPI Coverage Survey 1982	1982	I	I	I	-	-	I	-	-	I	-	-	Ministry of Health (Lesotho). Lesotho EPI Coverage Survey 1982.	GHDx
232404	Lesotho	Lesotho EPI Coverage Survey 1984	1984	I	I	I	-	-	I	-	-	I	-	-	Ministry of Health (Lesotho). Lesotho EPI Coverage Survey 1984.	GHDx
232443	Lesotho	Lesotho EPI Coverage Survey 1986	1986	I	I	I	-	-	I	-	-	I	-	-	Ministry of Health (Lesotho). Lesotho EPI Coverage Survey 1986.	GHDx
232445	Lesotho	Lesotho EPI Coverage Survey 1988	1988	I	I	I	-	-	I	-	-	I	-	-	Ministry of Health (Lesotho). Lesotho EPI Coverage Survey 1988.	GHDx
142213	Lesotho	Lesotho Multiple Indicator Cluster Survey 1996	1996	I	-	I	-	-	I	-	-	I	-	-	Bureau of Statistics (Lesotho), Ministry of Health (Lesotho), United Nations Children's Fund (UNICEF). Lesotho Multiple Indicator Cluster Survey 1996.	GHDx

GHDx ID	Country	Series	Year	BCG	DTP1	DTP3	HepB3	Hib3	MCV1	MCV2	PCV3	Pol3	RCV1	RotaC	Citation	Link
7721	Lesotho	Lesotho Multiple Indicator Cluster Survey 2000	2000	I	I	I	-	-	I	-	-	I	-	-	Bureau of Statistics (Lesotho), United Nations Children's Fund (UNICEF). Lesotho Multiple Indicator Cluster Survey 2000. New York, United States of America: United Nations Children's Fund (UNICEF).	GHDx
232279	Lesotho	Lesotho National Nutrition and EPI Cluster Survey 2002	2002	I	I	I	-	-	I	-	-	I	-	-	Government of Lesotho, Irish Aid, United Nations Children's Fund (UNICEF). Lesotho National Nutrition and EPI Cluster Survey 2002.	GHDx
232277	Lesotho	Lesotho Post SIAs and Routine Immunization Coverage Survey 2013	2013	I	I	I	I	I	I	I	-	I	-	-	Government of Lesotho. Lesotho Post SIAs and Routine Immunization Coverage Survey 2013.	GHDx
157058	Lesotho	Lesotho Demographic and Health Survey 2014	2014	I	I	I	-	-	I	-	-	I	-	-	ICF International, Ministry of Health and Social Welfare (Lesotho). Lesotho Demographic and Health Survey 2014. Fairfax, United States of America: ICF International.	GHDx
427778	Lesotho	Lesotho Multiple Indicator Cluster Survey 2018	2018	I	I	I	I	I	I	I	I	I	I	E	Bureau of Statistics (Lesotho), United Nations Children's Fund (UNICEF). Lesotho Multiple Indicator Cluster Survey 2018. New York, United States of America: United Nations Children's Fund (UNICEF), 2019.	GHDx
20167	Lesotho	Lesotho Demographic and Health Survey 2004-2005	2004-2005	I	I	I	E	-	I	-	-	I	-	-	Bureau of Statistics (Lesotho), Macro International, Inc, Ministry of Health and Social Welfare (Lesotho). Lesotho Demographic and Health Survey 2004-2005. Fairfax, United States of America: ICF International.	GHDx
21382	Lesotho	Lesotho Demographic and Health Survey 2009-2010	2009-2010	I	I	I	-	-	I	-	-	I	-	-	ICF Macro, Ministry of Health and Social Welfare (Lesotho). Lesotho Demographic and Health Survey 2009-2010. Fairfax, United States of America: ICF International.	GHDx
20180	Liberia	Liberia Demographic and Health Survey 1986	1986	I	I	I	-	-	I	-	-	I	-	-	Ministry of Planning and Economic Affairs (Liberia), Westinghouse; Institute for Resource Development. Liberia Demographic and Health Survey 1986. Fairfax, United States of America: ICF International.	GHDx
7751	Liberia	Liberia Multiple Indicator Cluster Survey 1995	1995	I	-	I	-	-	I	-	-	I	-	-	Bureau of Statistics (Liberia), United Nations Children's Fund (UNICEF). Liberia Multiple Indicator Cluster Survey 1995.	GHDx

GHDx ID	Country	Series	Year	BCG	DTP1	DTP3	HepB3	Hib3	MCV1	MCV2	PCV3	Pol3	RCV1	RotaC	Citation	Link
232515	Liberia	Liberia Integrated Management of Childhood Illness Household Baseline Survey 2000	2000	I	I	I	-	-	I	-	-	I	-	-	Liberia Integrated Management of Childhood Illness Household Baseline Survey 2000.	GHDx
232470	Liberia	Liberia EPI Cluster Survey 2005	2005	I	I	I	-	-	I	-	-	I	-	-	United Nations Children's Fund (UNICEF), World Health Organization (WHO). Liberia EPI Cluster Survey 2005.	GHDx
232467	Liberia	Liberia Routine Immunization Survey 2012	2012	I	I	I	I	I	I	-	-	I	-	-	Liberia Routine Immunization Survey 2012.	GHDx
77385	Liberia	Liberia Demographic and Health Survey 2013	2013	I	I	I	I	I	I	-	-	I	-	-	ICF International, Liberia Institute for Statistics and Geo-information Services (LISGIS), National AIDS and STI Control Program (NACP), Ministry of Health and Social Welfare (Liberia). Liberia Demographic and Health Survey 2013. Fairfax, United States of America: ICF International.	GHDx
286768	Liberia	Liberia Malaria Indicator Survey 2016	2016	I	I	I	I	I	I	-	E	I	-	-	ICF International, Liberia Institute for Statistics and Geo-information Services (LISGIS), National Malaria Control Program (Liberia). Liberia Malaria Indicator Survey 2016. Fairfax, United States of America: ICF International, 2017.	GHDx
90711	Liberia	Liberia Demographic and Health Survey 1999-2000	1999-2000	-	I	I	-	-	I	-	-	-	-	-	Ministry of Planning and Economic Affairs (Liberia), United Nations Population Fund (UNFPA), University of Liberia. Liberia Demographic and Health Survey 1999-2000.	GHDx
141586	Liberia	Liberia National Nutrition Survey 1999-2000	1999-2000	I	I	I	-	-	I	-	-	I	-	-	Christian Health Association of Liberia, Ministry of Health and Social Welfare (Liberia), United Nations Children's Fund (UNICEF). Liberia National Nutrition Survey 1999-2000.	GHDx
20191	Liberia	Liberia Demographic and Health Survey 2006-2007	2006-2007	I	I	I	-	-	I	-	-	I	-	-	Liberia Institute for Statistics and Geo-information Services (LISGIS), Macro International, Inc. Liberia Demographic and Health Survey 2006-2007. Fairfax, United States of America: ICF International.	GHDx
459845*	Liberia	Liberia Demographic and Health Survey 2019-2020	2019-2020	I	I	I	I	I	I	-	I	I	-	I	ICF International, Liberia Institute for Statistics and Geo-information Services (LISGIS), Ministry of Health and Social Welfare (Liberia). Liberia Demographic and Health Survey 2019-2020. 2021.	GHDx

GHDx ID	Country	Series	Year	BCG	DTP1	DTP3	HepB3	Hib3	MCV1	MCV2	PCV3	Pol3	RCV1	RotaC	Citation	Link
124079	Libya	Libya Multiple Indicator Cluster Survey 2003	2003	I	e	-	-	-	I	-	-	-	-	-	Public Commission for Health Care Planning (Libya), United Nations Children's Fund (UNICEF). Libya Multiple Indicator Cluster Survey 2003.	GHDx
107340*	Libya	Libya Family Health Survey 2007	2007	I	I	I	-	-	I	-	-	I	-	-	League of Arab States, National Center for Disease Control (Libya), Pan Arab Project for Family Health (PAPFAM). Libya Family Health Survey 2007.	GHDx
453457	Luxembourg	Luxembourg Immunization Coverage Survey 2018	2018	-	I	I	I	I	I	I	I	-	-	-	Ministry of Health (Luxembourg). Luxembourg Immunization Coverage Survey 2018.	GHDx
195061	Luxembourg	Luxembourg Immunization Coverage Survey 2007-2008	2007-2008	-	-	I	I	I	I	-	I	I	I	-	Free University of Brussels (ULB), Ministry of Health (Luxembourg). Luxembourg Immunization Coverage Survey 2007-2008. Luxembourg: Ministry of Health (Luxembourg).	GHDx
20202	Madagascar	Madagascar Demographic and Health Survey 1992	1992	I	I	I	-	-	I	-	-	I	-	-	Department of Applied Research for Development (Madagascar), Macro International, Inc. Madagascar Demographic and Health Survey 1992. Calverton, United States of America: Macro International, Inc.	GHDx
7855	Madagascar	Madagascar Multiple Indicator Cluster Survey 1995	1995	I	-	I	-	-	I	-	-	I	-	-	National Institute of Statistics (Madagascar), United Nations Children's Fund (UNICEF). Madagascar Multiple Indicator Cluster Survey 1995.	GHDx
20212	Madagascar	Madagascar Demographic and Health Survey 1997	1997	I	I	I	-	-	I	-	-	I	-	-	Macro International, Inc, National Institute of Statistics (Madagascar). Madagascar Demographic and Health Survey 1997. Fairfax, United States of America: ICF International.	GHDx
7876	Madagascar	Madagascar Household Priority Survey 1999	1999	I	-	I	-	-	I	-	-	I	-	-	National Institute of Statistics (Madagascar). Madagascar Household Priority Survey 1999.	GHDx
27020	Madagascar	Madagascar Multiple Indicator Cluster Survey 2000	2000	I	e	e	-	-	I	-	-	I	-	-	National Institute of Statistics (Madagascar), United Nations Children's Fund (UNICEF). Madagascar Multiple Indicator Cluster Survey 2000. New York, United States of America: United Nations Children's Fund (UNICEF).	GHDx
231848	Madagascar	Madagascar Immunization Coverage Survey 2008	2008	I	I	I	I	-	I	-	-	I	-	-	Madagascar Immunization Coverage Survey 2008.	GHDx
231845	Madagascar	Madagascar Immunization Coverage Evaluation 2011	2011	I	I	I	I	I	I	-	-	I	-	-	Madagascar Immunization Coverage Evaluation 2011.	GHDx

GHDx ID	Country	Series	Year	BCG	DTP1	DTP3	HepB3	Hib3	MCV1	MCV2	PCV3	Pol3	RCV1	RotaC	Citation	Link
399853	Madagascar	Madagascar Multiple Indicator Cluster Survey 2018	2018	I	I	I	I	I	I	-	I	I	-	I	National Institute of Statistics (Madagascar), United Nations Children's Fund (UNICEF). Madagascar Multiple Indicator Cluster Survey 2018. 2019.	GHDx
493562*	Madagascar	Madagascar Demographic and Health Survey 2021	2021	I	I	I	I	I	I	-	I	I	E	I	ICF Macro, National Institute of Statistics (Madagascar). Madagascar Demographic and Health Survey 2021. Fairfax, United States of America: ICF International, 2022.	GHDx
502059*	Madagascar	Madagascar Vaccine Coverage Survey 2021	2021	I	I	I	I	I	I	-	I	I	-	I	Ministry of Public Health (Madagascar), National Institute of Statistics (Madagascar). Madagascar Vaccine Coverage Survey 2021.	GHDx
20223	Madagascar	Madagascar Demographic and Health Survey 2003-2004	2003-2004	I	I	I	-	-	I	-	-	I	-	-	Macro International, Inc, National Institute of Statistics (Madagascar). Madagascar Demographic and Health Survey 2003-2004. Fairfax, United States of America: ICF International.	GHDx
21409	Madagascar	Madagascar Demographic and Health Survey 2008-2009	2008-2009	I	I	I	-	-	I	-	-	I	-	-	ICF Macro, National Institute of Statistics (Madagascar). Madagascar Demographic and Health Survey 2008-2009. Fairfax, United States of America: ICF International.	GHDx
20235	Malawi	Malawi Demographic and Health Survey 1992	1992	I	I	I	-	-	I	-	-	I	-	-	Macro International, Inc, National Statistical Office of Malawi. Malawi Demographic and Health Survey 1992. Fairfax, United States of America: ICF International.	GHDx
7907	Malawi	Malawi Multiple Indicator Cluster Survey 1995	1995	I	-	I	-	-	I	-	-	I	-	-	National Statistical Office of Malawi, United Nations Children's Fund (UNICEF). Malawi Multiple Indicator Cluster Survey 1995.	GHDx
80790	Malawi	Malawi Knowledge, Attitudes and Practices in Health Survey 1996	1996	I	I	I	-	-	I	-	-	I	-	-	Macro International, Inc, National Statistical Office of Malawi. Malawi Knowledge, Attitudes and Practices in Health Survey 1996. Fairfax, United States of America: ICF International.	GHDx
20252	Malawi	Malawi Demographic and Health Survey 2000	2000	I	I	I	-	-	I	-	-	I	-	-	Macro International, Inc, National Statistical Office of Malawi. Malawi Demographic and Health Survey 2000. Fairfax, United States of America: ICF International.	GHDx
7919	Malawi	Malawi Multiple Indicator Cluster Survey 2006	2006	I	I	I	I	I	I	-	-	I	-	-	United Nations Children's Fund (UNICEF), National Statistics Office (Malawi). Malawi Multiple Indicator Cluster Survey 2006. New York, United States: United Nations Children's Fund (UNICEF).	GHDx

GHDx ID	Country	Series	Year	BCG	DTP1	DTP3	HepB3	Hib3	MCV1	MCV2	PCV3	Pol3	RCV1	RotaC	Citation	Link
21393	Malawi	Malawi Demographic and Health Survey 2010	2010	I	I	I	I	I	I	-	-	I	-	-	ICF Macro, National Statistical Office of Malawi. Malawi Demographic and Health Survey 2010. Fairfax, United States of America: ICF International.	GHDx
20263	Malawi	Malawi Demographic and Health Survey 2004-2005	2004-2005	I	I	I	E	E	I	-	-	I	-	-	Macro International, Inc, National Statistical Office of Malawi. Malawi Demographic and Health Survey 2004-2005. Fairfax, United States of America: ICF International.	GHDx
26683	Malawi	Malawi Global Fund Household Health Coverage Survey 2007-2008	2007-2008	-	I	I	-	-	-	-	-	I	-	-	Ministry of Economic Planning and Development (Malawi), National Statistical Office of Malawi. Malawi Global Fund Household Health Coverage Survey 2007-2008.	GHDx
161662	Malawi	Malawi Multiple Indicator Cluster Survey 2013-2014	2013-2014	I	I	I	I	I	I	-	I	I	-	I	National Statistical Office of Malawi, United Nations Children's Fund (UNICEF). Malawi Multiple Indicator Cluster Survey 2013-2014. New York, United States of America: United Nations Children's Fund (UNICEF), 2015.	GHDx
218581	Malawi	Malawi Demographic and Health Survey 2015-2016	2015-2016	I	I	I	I	I	I	E	I	I	-	I	Emory University and Centers for Disease Control & Prevention Collaboration, ICF International, Ministry of Health (Malawi), National Statistical Office of Malawi. Malawi Demographic and Health Survey 2015-2016. Fairfax, United States of America: ICF International, 2017.	GHDx
327852	Malawi	Malawi Integrated Household Survey 2016-2017	2016-2017	-	-	-	-	-	I	-	-	-	-	-	National Statistical Office. Malawi - Fourth Integrated Household Survey 2016-2017, Ref. MWI_2016_IHS-IV_v02_M. Dataset downloaded from [http://microdata.worldbank.org/index.php/catalog/2936] on [December 18, 2017].	GHDx
494148*	Malawi	Malawi Multiple Indicator Cluster Survey 2019-2020	2019-2020	I	I	I	I	I	I	I	I	I	I	I	National Statistical Office of Malawi, United Nations Children's Fund (UNICEF). Malawi Multiple Indicator Cluster Survey 2019-2020. New York, United States of America: United Nations Children's Fund (UNICEF), 2022.	GHDx
284410	Malaysia	Malaysia National Health and Morbidity Survey 2016	2016	I	I	I	I	I	I	-	-	I	I	-	Institute for Public Health, Ministry of Health (Malaysia), National Institutes of Health (NIH). Malaysia National Health and Morbidity Survey 2016.	GHDx

GHDx ID	Country	Series	Year	BCG	DTP1	DTP3	HepB3	Hib3	MCV1	MCV2	PCV3	Pol3	RCV1	RotaC	Citation	Link
317418*	Malaysia	Malaysia Ministry of Health Annual Report 2014	1997-2014	-	-	I	I	I	I	-	-	I	I	-	Ministry of Health (Malaysia). Malaysia Ministry of Health Annual Report 2014. Putrajaya, Malaysia: Ministry of Health (Malaysia).	GHDx
90714	Maldives	Maldives Multiple Indicator Cluster Survey 1995	1995	I	-	I	-	-	I	-	-	I	-	-	United Nations Children's Fund (UNICEF). Maldives Multiple Indicator Cluster Survey 1995.	GHDx
8033	Maldives	Maldives Multiple Indicator Cluster Survey 2001	2001	I	I	I	-	-	I	-	-	I	-	-	Ministry of Health (Maldives), Republic of Maldives, United Nations Children's Fund (UNICEF). Maldives Multiple Indicator Cluster Survey 2001.	GHDx
21311	Maldives	Maldives Demographic and Health Survey 2009	2009	I	I	I	I	-	I	-	-	I	-	-	ICF Macro, Ministry of Health (Maldives). Maldives Demographic and Health Survey 2009. Fairfax, United States of America: ICF International.	GHDx
218582	Maldives	Maldives Demographic and Health Survey 2016-2017	2016-2017	I	I	I	I	I	I	I	-	I	-	-	ICF International, Ministry of Health (Maldives). Maldives Demographic and Health Survey 2016-2017. Fairfax, United States of America: ICF International, 2019.	GHDx
20283	Mali	Mali Demographic and Health Survey 1987	1987	I	I	I	-	-	I	-	-	I	-	-	Sahel Institute, Westinghouse; Institute for Resource Development. Mali Demographic and Health Survey 1987. Fairfax, United States of America: ICF International.	GHDx
231844	Mali	Mali Immunization Coverage Survey 1990	1990	I	I	I	-	-	I	-	-	I	-	-	Ministry of Health (Mali), UNICEF, WHO. Mali Immunization Coverage Survey 1990. Bamako, Mali: Ministry of Health.	GHDx
8043	Mali	Mali Multiple Indicator Cluster Survey 1996	1996	I	-	I	-	-	I	-	-	I	-	-	National Directorate of Statistics and Informatics (DNSI) (Mali), United Nations Children's Fund (UNICEF). Mali Multiple Indicator Cluster Survey 1996.	GHDx
20315	Mali	Mali Demographic and Health Survey 2001	2001	I	I	I	-	-	I	-	-	I	-	-	Macro International, Inc, National Directorate of Statistics and Informatics (DNSI) (Mali), Planning and Statistics Unit, Ministry of Health (Mali). Mali Demographic and Health Survey 2001. Fairfax, United States of America: ICF International.	GHDx
20274	Mali	Mali Demographic and Health Survey 2006	2006	I	I	I	E	-	I	-	-	I	-	-	Macro International, Inc, Ministry of Health (Mali), National Directorate of Statistics and Informatics (DNSI) (Mali). Mali Demographic and Health Survey 2006. Fairfax, United States of America: ICF International.	GHDx
231842	Mali	Mali External EPI Review 2006	2006	I	I	I	-	-	I	-	-	I	-	-	Mali External EPI Review 2006.	GHDx

GHDx ID	Country	Series	Year	BCG	DTP1	DTP3	HepB3	Hib3	MCV1	MCV2	PCV3	Pol3	RCV1	RotaC	Citation	Link
248224	Mali	Mali Multiple Indicator Cluster Survey 2015	2015	I	I	I	I	I	I	-	I	I	E	E	Ministry of Health (Mali), Ministry of Planning (Mali), National Institute of Statistics (INSTAT) (Mali), United Nations Children's Fund (UNICEF). Mali Multiple Indicator Cluster Survey 2015. New York, United States of America: United Nations Children's Fund (UNICEF), 2017.	GHDx
398033	Mali	Mali Demographic and Health Survey 2018	2018	I	I	I	I	I	I	-	I	I	-	I	ICF International, National Institute of Statistics (INSTAT) (Mali), Sector Planning and Statistics Unit Health-Social Development and Family Promotion (CPS / SS-DS-PF) (Mali). Mali Demographic and Health Survey 2018. Fairfax, United States of America: ICF International, 2019.	GHDx
20301	Mali	Mali Demographic and Health Survey 1995-1996	1995-1996	I	I	I	-	-	I	-	-	I	-	-	Macro International, Inc, National Directorate of Statistics and Informatics (DNSI) (Mali), Planning and Statistics Unit, Ministry of Health (Mali). Mali Demographic and Health Survey 1995-1996. Fairfax, United States of America: ICF International.	GHDx
231841	Mali	Mali Evaluation of Routine Immunization Coverage 2009-2010	2009-2010	I	I	I	I	I	I	-	-	I	-	-	Ministry of Health (Mali). Mali Evaluation of Routine Immunization Coverage 2009-2010.	GHDx
77388	Mali	Mali Demographic and Health Survey 2012-2013	2012-2013	I	I	I	E	E	I	-	-	e	-	-	ICF International, INFO-STAT (Mali), Ministry of Health (Mali), National Institute of Statistics (INSTAT) (Mali), Planning and Statistics Unit, Ministry of Health (Mali). Mali Demographic and Health Survey 2012-2013. Fairfax, United States of America: ICF International, 2014.	GHDx
260407	Mali	Mali Agricultural Integrated Economic Survey 2014-2015	2014-2015	I	I	I	-	-	I	-	-	I	-	-	Ministry of Rural Development (Mali), National Institute of Statistics (INSTAT) (Mali), World Bank. Mali Agricultural Integrated Economic Survey 2014-2015. Washington DC, United States of America: World Bank.	GHDx
24152	Marshall Islands	Marshall Islands Community Survey 2006	2006	I	-	I	I	I	I	-	-	I	I	-	Economic Policy, Planning and Statistics Office (Marshall Islands). Marshall Islands Community Survey 2006.	GHDx
8102	Mauritania	Mauritania Multiple Indicator Cluster Survey 1996	1996	I	-	I	-	-	I	-	-	I	-	-	United Nations Children's Fund (UNICEF). Mauritania Multiple Indicator Cluster Survey 1996.	GHDx

GHDx ID	Country	Series	Year	BCG	DTP1	DTP3	HepB3	Hib3	MCV1	MCV2	PCV3	Pol3	RCV1	RotaC	Citation	Link
440420	Mauritania	Mauritania National Vaccine Coverage Survey 1999	1999	-	I	I	-	-	I	-	-	I	-	-	World Health Organization (WHO). Mauritania National Vaccine Coverage Survey 1999.	GHDx
231813	Mauritania	Mauritania Immunization Coverage and Social Mobilization Survey 2004	2004	I	I	I	-	-	I	-	-	I	-	-	Mauritania Immunization Coverage and Social Mobilization Survey 2004.	GHDx
8115	Mauritania	Mauritania Multiple Indicator Cluster Survey 2007	2007	I	I	I	-	-	I	-	-	I	-	-	National Office of Statistics (Mauritania), United Nations Children's Fund (UNICEF). Mauritania Multiple Indicator Cluster Survey 2007. New York, United States of America: United Nations Children's Fund (UNICEF).	GHDx
152783	Mauritania	Mauritania Multiple Indicator Cluster Survey 2011	2011	I	I	I	I	E	I	-	-	I	-	-	National Office of Statistics (Mauritania), United Nations Children's Fund (UNICEF). Mauritania Multiple Indicator Cluster Survey 2011. New York, United States of America: United Nations Children's Fund (UNICEF), 2015.	GHDx
267343	Mauritania	Mauritania Multiple Indicator Cluster Survey 2015	2015	I	I	I	I	I	I	-	I	I	-	I	National Office of Statistics (Mauritania), United Nations Children's Fund (UNICEF). Mauritania Multiple Indicator Cluster Survey 2015. New York, United States of America: United Nations Children's Fund (UNICEF), 2018.	GHDx
558942*	Mauritania	Mauritania Expanded Vaccination Program Survey 2022	2022	I	I	I	I	I	I	-	I	I	-	-	Mauritania Expanded Vaccination Program Survey 2022.	GHDx
20322	Mauritania	Mauritania Demographic and Health Survey 2000-2001	2000-2001	I	I	I	-	-	I	-	-	I	-	-	Macro International, Inc, National Office of Statistics (Mauritania). Mauritania Demographic and Health Survey 2000-2001. Fairfax, United States of America: ICF International.	GHDx
470667*	Mauritania	Mauritania Demographic and Health Survey 2019-2020	2019-2021	I	I	I	I	I	I	-	I	I	-	I	National Office of Statistics (Mauritania). Mauritania Demographic and Health Survey 2019-2020. Fairfax, United States of America: ICF International, 2022.	GHDx
20326	Mexico	Mexico Demographic and Health Survey 1987	1987	I	I	I	-	-	I	-	-	I	-	-	Macro Systems, Inc.; Institute for Resource Development, Secretariat of Health (Mexico). Mexico Demographic and Health Survey 1987. Fairfax, United States of America: ICF International.	GHDx

GHDx ID	Country	Series	Year	BCG	DTP1	DTP3	HepB3	Hib3	MCV1	MCV2	PCV3	Pol3	RCV1	RotaC	Citation	Link
129896	Mexico	Mexico INEGI Information Bank - Proportion of Children Under 1 Year Vaccinated Against Measles	1990	-	-	-	-	-	I	-	-	-	-	-	National Institute of Statistics and Geography (INEGI) (Mexico). Mexico INEGI Information Bank - Proportion of Children Under 1 Year Vaccinated Against Measles. Mexico City, Mexico: National Institute of Statistics and Geography (INEGI) (Mexico).	GHDx
8442	Mexico	Mexico Family Life Survey 2002	2002	I	e	e	-	E	I	-	-	I	I	-	Center for Research and Teaching in Economics (CIDE) (Mexico), Ibero-American University, National Institute of Perinatology (Mexico), National Institute of Statistics and Geography (INEGI) (Mexico). Mexico Family Life Survey 2002.	GHDx
264590	Mexico	Mexico Multiple Indicator Cluster Survey 2015	2015	e	e	e	-	E	e	-	E	e	-	E	National Institute of Public Health (Mexico), United Nations Children's Fund (UNICEF). Mexico Multiple Indicator Cluster Survey 2015. New York, United States of America: United Nations Children's Fund (UNICEF), 2017.	GHDx
505231*	Mexico	Mexico Continuous National Health and Nutrition Survey 2021	2021	I	-	I	I	I	I	-	-	I	I	-	Government of Mexico, National Institute of Public Health (Mexico), Secretariat of Health (Mexico). Mexico Continuous National Health and Nutrition Survey 2021. Cuernavaca, Mexico: National Institute of Public Health (Mexico), 2022.	GHDx
130481	Mexico	Mexico Vaccinations for Under 1-year and Age 1-4 years 1998-2012	1998-2012	I	-	I	I	I	I	-	I	I	I	-	Secretariat of Health (Mexico). Mexico Vaccinations for Under 1-year and Age 1-4 years 1998-2012.	GHDx
8684	Mexico	Mexico National Performance Evaluation Survey 2002-2003	2002-2003	-	e	e	-	-	I	-	-	-	-	-	National Institute of Public Health (Mexico), Secretariat of Health (Mexico), World Health Organization (WHO). Mexico National Performance Evaluation Survey 2002-2003.	GHDx
8618	Mexico	Mexico National Survey of Health and Nutrition 2005-2006	2005-2006	I	I	I	-	I	I	-	-	I	I	-	National Institute of Public Health (Mexico). Mexico National Survey of Health and Nutrition 2005-2006. Cuernavaca, Mexico: National Institute of Public Health (Mexico).	GHDx
81748	Mexico	Mexico National Survey of Health and Nutrition 2011-2012	2011-2012	I	I	I	I	I	I	-	E	I	I	-	National Institute of Public Health (Mexico). Mexico National Survey of Health and Nutrition 2011-2012. Cuernavaca, Mexico: National Institute of Public Health (Mexico).	GHDx

GHDx ID	Country	Series	Year	BCG	DTP1	DTP3	HepB3	Hib3	MCV1	MCV2	PCV3	Pol3	RCV1	RotaC	Citation	Link
428683*	Mexico	Mexico National Survey of Health and Nutrition (ENSANUT) 2018-2019	2018-2019	I	I	I	I	I	I	-	I	I	I	I	National Institute of Public Health (Mexico), National Institute of Statistics and Geography (INEGI) (Mexico). Mexico National Survey of Health and Nutrition (ENSANUT) 2018-2019. Cuernavaca, Mexico: National Institute of Public Health (Mexico).	GHDx
8734	Moldova	Moldova Multiple Indicator Cluster Survey 2000	2000	I	I	I	I	-	I	-	-	I	-	-	National Scientific and Applied Center for Preventive Medicine (NCPM) (Moldova), United Nations Children's Fund (UNICEF). Moldova Multiple Indicator Cluster Survey 2000. New York, United States: United Nations Children's Fund (UNICEF).	GHDx
20339	Moldova	Moldova Demographic and Health Survey 2005	2005	I	I	I	I	-	I	-	-	I	-	-	Macro International, Inc, National Scientific and Applied Center for Preventive Medicine (Moldova). Moldova Demographic and Health Survey 2005. Fairfax, United States of America: ICF International.	GHDx
140200	Moldova	Moldova Multiple Indicator Cluster Survey 2012	2012	I	I	I	I	-	I	-	-	I	I	-	Ministry of Health (Moldova), National Office of Statistics (Moldova), United Nations Children's Fund (UNICEF). Moldova Multiple Indicator Cluster Survey 2012. New York, United States of America: United Nations Children's Fund (UNICEF), 2015.	GHDx
8788	Mongolia	Mongolia Multiple Indicator Cluster Survey 2000	2000	I	I	I	-	-	I	-	-	I	-	-	National Statistical Office of Mongolia. Mongolia Multiple Indicator Cluster Survey 2000. New York, United States of America: United Nations Children's Fund (UNICEF).	GHDx
8777	Mongolia	Mongolia Multiple Indicator Cluster Survey 2005	2005	I	I	I	-	-	I	-	-	I	-	-	National Statistical Office of Mongolia, United Nations Children's Fund (UNICEF). Mongolia Multiple Indicator Cluster Survey 2005. New York, United States of America: United Nations Children's Fund (UNICEF).	GHDx
76704	Mongolia	Mongolia Multiple Indicator Cluster Survey 2010	2010	I	I	I	I	I	I	-	-	I	I	-	National Statistical Office of Mongolia, United Nations Children's Fund (UNICEF). Mongolia Multiple Indicator Cluster Survey 2010. New York, United States of America: United Nations Children's Fund (UNICEF), 2013.	GHDx

GHDx ID	Country	Series	Year	BCG	DTP1	DTP3	HepB3	Hib3	MCV1	MCV2	PCV3	Pol3	RCV1	RotaC	Citation	Link
150866	Mongolia	Mongolia Multiple Indicator Cluster Survey 2013	2013	I	I	I	I	I	I	-	-	I	-	-	Government of Mongolia, National Statistical Office of Mongolia, United Nations Children's Fund (UNICEF), United Nations Population Fund (UNFPA). Mongolia Multiple Indicator Cluster Survey 2013. New York, United States of America: United Nations Children's Fund (UNICEF), 2016.	GHDx
427952	Mongolia	Mongolia Multiple Indicator Cluster Survey 2018	2018	I	I	I	I	I	I	I	-	I	I	-	Government of Mongolia, National Statistical Office of Mongolia, United Nations Children's Fund (UNICEF), United Nations Population Fund (UNFPA). Mongolia Multiple Indicator Cluster Survey 2018. New York, United States of America: United Nations Children's Fund (UNICEF), 2019.	GHDx
559752*	Mongolia	Mongolia Multiple Indicator Cluster Survey 2023	2023	I	-	I	I	I	I	I	I	I	I	-	National Statistical Office of Mongolia, United Nations Children's Fund (UNICEF), United Nations Population Fund (UNFPA). Mongolia Multiple Indicator Cluster Survey 2023.	GHDx
150526	Montenegro	Montenegro Multiple Indicator Cluster Survey 2013	2013	I	I	I	I	I	I	-	-	I	I	-	Statistical Office of Montenegro. Montenegro Multiple Indicator Cluster Survey 2013. New York, United States of America: United Nations Children's Fund (UNICEF), 2015.	GHDx
20347	Morocco	Morocco Demographic and Health Survey 1987	1987	I	I	I	-	-	I	-	-	I	-	-	Ministry of Public Health (Morocco), Westinghouse; Institute for Resource Development. Morocco Demographic and Health Survey 1987. Fairfax, United States of America: ICF International.	GHDx
231862	Morocco	Morocco National Immunization Coverage Survey 1988	1988	I	I	I	-	-	I	-	-	I	-	-	Morocco National Immunisation Coverage Survey 1988.	GHDx
231861	Morocco	Morocco National Immunization Coverage Survey 1989	1989	I	I	I	-	-	I	-	-	I	-	-	Morocco National Immunisation Coverage Survey 1989.	GHDx
20371	Morocco	Morocco Demographic and Health Survey 1992	1992	I	I	I	-	-	I	-	-	I	-	-	Macro International, Inc, Ministry of Public Health (Morocco). Morocco Demographic and Health Survey 1992. Fairfax, United States of America: ICF International.	GHDx
8829	Morocco	Morocco Maternal and Child Health Survey 1996-1997	1996-1997	I	I	I	-	-	I	-	-	I	-	-	Ministry of Health (Morocco), League of Arab States. Morocco Maternal and Child Health Survey 1996-1997.	GHDx

GHDx ID	Country	Series	Year	BCG	DTP1	DTP3	HepB3	Hib3	MCV1	MCV2	PCV3	Pol3	RCV1	RotaC	Citation	Link
20361	Morocco	Morocco Demographic and Health Survey 2003-2004	2003-2004	I	I	I	-	-	I	-	-	I	-	-	League of Arab States, Macro International, Inc, Ministry of Health (Morocco). Morocco Demographic and Health Survey 2003-2004. Fairfax, United States of America: ICF International.	GHDx
126909	Morocco	Morocco National Survey on Population and Family Health 2010-2011	2010-2011	I	e	e	I	I	I	-	-	I	-	-	Ministry of Health (Morocco), Pan Arab Project for Family Health (PAPFAM), United Nations Children's Fund (UNICEF), United Nations Population Fund (UNFPA), World Health Organization (WHO). Morocco National Survey on Population and Family Health 2010-2011.	GHDx
423654	Morocco	Morocco National Survey on Population and Family Health 2017-2018	2017-2018	I	I	I	I	I	I	-	-	I	-	-	Ministry of Health (Morocco), Pan Arab Project for Family Health (PAPFAM), United Nations Children's Fund (UNICEF), United Nations Population Fund (UNFPA), World Health Organization (WHO). Morocco National Survey on Population and Family Health 2017-2018.	GHDx
8881	Mozambique	Mozambique Multiple Indicator Cluster Survey 1995	1995	I	-	I	-	-	I	-	-	I	-	-	Ministry of Health (Mozambique), National Institute of Statistics (INE) (Mozambique), United Nations Children's Fund (UNICEF). Mozambique Multiple Indicator Cluster Survey 1995.	GHDx
20382	Mozambique	Mozambique Demographic and Health Survey 1997	1997	I	I	I	-	-	I	-	-	I	-	-	Macro International, Inc, National Institute of Statistics (INE) (Mozambique). Mozambique Demographic and Health Survey 1997. Fairfax, United States of America: ICF International.	GHDx
55975	Mozambique	Mozambique Demographic and Health Survey 2011	2011	I	I	I	I	E	I	-	-	I	-	-	ICF Macro, Manhica Health Research Center (CISM), Ministry of Health (Mozambique), National Institute of Statistics (INE) (Mozambique). Mozambique Demographic and Health Survey 2011. Fairfax, United States of America: ICF International.	GHDx
157060	Mozambique	Mozambique AIDS Indicator Survey 2015	2015	I	I	I	-	-	I	-	E	I	-	-	Centers for Disease Control and Prevention (CDC), ICF International, Ministry of Health (Mozambique), National Institute of Health (Mozambique), National Institute of Statistics (INE) (Mozambique). Mozambique AIDS Indicator Survey 2015. Fairfax, United States of America: ICF International, 2018.	GHDx

GHDx ID	Country	Series	Year	BCG	DTP1	DTP3	HepB3	Hib3	MCV1	MCV2	PCV3	Pol3	RCV1	RotaC	Citation	Link
20394	Mozambique	Mozambique Demographic and Health Survey 2003-2004	2003-2004	I	I	I	-	-	I	-	-	I	-	-	Macro International, Inc, National Institute of Statistics (INE) (Mozambique). Mozambique Demographic and Health Survey 2003-2004. Fairfax, United States of America: ICF International.	GHDx
27031	Mozambique	Mozambique Multiple Indicator Cluster Survey 2008-2009	2008-2009	I	I	I	-	-	I	-	-	I	-	-	United Nations Children's Fund (UNICEF), National Statistics Institute (Mozambique). Mozambique Multiple Indicator Cluster Survey 2008-2009. New York, United States: United Nations Children's Fund (UNICEF).	GHDx
553572*	Mozambique	Mozambique Demographic and Health Survey 2022-2023	2022-2023	I	I	I	I	I	I	I	I	I	-	I	ICF International, National Institute of Statistics (INE) (Mozambique). Mozambique Demographic and Health Survey 2022-2023. Fairfax, United States of America: ICF International, 2024.	GHDx
231821	Myanmar	Myanmar EPI Coverage Evaluation Survey 1991	1991	I	I	I	-	-	I	-	-	I	-	-	United Nations Children's Fund (UNICEF). Myanmar EPI Coverage Evaluation Survey 1991.	GHDx
8922	Myanmar	Myanmar Multiple Indicator Cluster Survey 1995	1995	I	-	I	-	-	I	-	-	I	-	-	Ministry of Health (Myanmar), United Nations Children's Fund (UNICEF). Myanmar Multiple Indicator Cluster Survey 1995.	GHDx
231817	Myanmar	Monitoring Progress Towards the Goals of National Program of Action for Myanmar's Children 1997	1997	I	-	I	-	-	I	-	-	I	-	-	Monitoring Progress Towards the Goals of National Program of Action for Myanmar's Children 1997.	GHDx
8932	Myanmar	Myanmar Multiple Indicator Cluster Survey 2000	2000	I	I	I	-	-	I	-	-	I	-	-	Ministry of Health (Myanmar), United Nations Children's Fund (UNICEF). Myanmar Multiple Indicator Cluster Survey 2000. New York, United States of America: United Nations Children's Fund (UNICEF).	GHDx
141910	Myanmar	Myanmar Multiple Indicator Cluster Survey 2003	2003	I	I	I	-	-	I	-	-	I	-	-	Ministry of Health (Myanmar), United Nations Children's Fund (UNICEF). Myanmar Multiple Indicator Cluster Survey 2003.	GHDx
231824	Myanmar	Myanmar EPI Review 1986-1987	1986-1987	I	I	I	-	-	I	-	-	I	-	-	Government of Myanmar, United Nations Children's Fund (UNICEF), World Health Organization (WHO). Myanmar EPI Review 1986-1987.	GHDx
40237	Myanmar	Myanmar Fertility and Reproductive Health Survey 2006-2007	2006-2007	I	-	I	I	-	I	-	-	I	-	-	Ministry of Immigration and Population (Myanmar), United Nations Population Fund (UNFPA). Myanmar Fertility and Reproductive Health Survey 2006-2007.	GHDx

GHDx ID	Country	Series	Year	BCG	DTP1	DTP3	HepB3	Hib3	MCV1	MCV2	PCV3	Pol3	RCV1	RotaC	Citation	Link
90696	Myanmar	Myanmar Multiple Indicator Cluster Survey 2009-2010	2009-2010	I	I	I	I	-	I	-	-	I	-	-	Ministry of Health (Myanmar), Ministry of National Planning and Economic Development (Myanmar), United Nations Children's Fund (UNICEF). Myanmar Multiple Indicator Cluster Survey 2009-2010.	GHDx
157061	Myanmar	Myanmar Demographic and Health Survey 2015-2016	2015-2016	I	I	I	I	-	I	-	-	I	-	-	ICF International, Ministry of Health and Sports (Myanmar). Myanmar Demographic and Health Survey 2015-2016. Fairfax, United States of America: ICF International, 2017.	GHDx
20404	Namibia	Namibia Demographic and Health Survey 1992	1992	e	e	e	-	-	e	-	-	e	-	-	Central Statistics Office (Namibia), Macro International, Inc, Ministry of Health and Social Services (Namibia). Namibia Demographic and Health Survey 1992. Fairfax, United States of America: ICF International.	GHDx
20417	Namibia	Namibia Demographic and Health Survey 2000	2000	I	I	I	-	-	I	-	-	I	-	-	Macro International, Inc, Ministry of Health and Social Services (Namibia), National Planning Commission (Namibia). Namibia Demographic and Health Survey 2000. Fairfax, United States of America: ICF International.	GHDx
231812	Namibia	Namibia Post Measles Supplemental Immunization and EPI Coverage Survey 2012	2012	I	I	I	I	I	I	-	-	I	-	-	Namibia Post Measles Supplemental Immunization and EPI Coverage Survey 2012.	GHDx
150382	Namibia	Namibia Demographic and Health Survey 2013	2013	I	I	I	I	I	I	-	-	I	-	-	ICF International, Ministry of Health and Social Services (Namibia), Namibia Institute of Pathology, Namibia Statistics Agency. Namibia Demographic and Health Survey 2013. Fairfax, United States of America: ICF International.	GHDx
20428	Namibia	Namibia Demographic and Health Survey 2006-2007	2006-2007	I	I	I	-	-	I	-	-	I	-	-	Macro International, Inc, Ministry of Health and Social Services (Namibia). Namibia Demographic and Health Survey 2006-2007. Fairfax, United States of America: ICF International.	GHDx
21436	Nauru	Nauru Demographic and Health Survey 2007	2007	I	I	I	-	-	I	-	-	I	-	-	Nauru Bureau of Statistics, Secretariat of the Pacific Community (SPC), Macro International, Inc., Nauru Demographic and Health Survey 2007. Noumea, New Caledonia, France: Secretariat of the Pacific Community (SPC).	GHDx

GHDx ID	Country	Series	Year	BCG	DTP1	DTP3	HepB3	Hib3	MCV1	MCV2	PCV3	Pol3	RCV1	RotaC	Citation	Link
20437	Nepal	Nepal Demographic and Health Survey 1996	1996	I	I	I	-	-	I	-	-	I	-	-	Macro International, Inc, Ministry of Health and Population (Nepal), New ERA. Nepal Demographic and Health Survey 1996. Fairfax, United States of America: ICF International.	GHDx
25030	Nepal	Nepal Intercensal Household Information Monitoring and Evaluation System 2000	2000	I	I	I	-	-	I	-	-	I	-	-	Central Bureau of Statistics (Nepal). Nepal Intercensal Household Information Monitoring and Evaluation System 2000.	GHDx
20450	Nepal	Nepal Demographic and Health Survey 2001	2001	I	I	I	-	-	I	-	-	I	-	-	Macro International, Inc, Ministry of Health and Population (Nepal), New ERA. Nepal Demographic and Health Survey 2001. Fairfax, United States of America: ICF International.	GHDx
20462	Nepal	Nepal Demographic and Health Survey 2006	2006	I	I	I	E	-	I	-	-	I	-	-	Macro International, Inc, Ministry of Health and Population (Nepal), New ERA. Nepal Demographic and Health Survey 2006. Fairfax, United States of America: ICF International.	GHDx
231789	Nepal	Nepal Immunization Coverage Survey 2009	2009	I	I	I	-	-	I	-	-	I	-	-	Ministry of Health and Population (Nepal). Nepal Immunization Coverage Survey 2009.	GHDx
21240	Nepal	Nepal Demographic and Health Survey 2011	2011	I	I	I	I	E	I	-	-	I	-	-	ICF Macro, Ministry of Health and Population (Nepal), New ERA. Nepal Demographic and Health Survey 2011. Fairfax, United States of America: ICF International.	GHDx
162317	Nepal	Nepal Multiple Indicator Cluster Survey 2014	2014	I	I	I	I	I	I	-	-	I	-	-	Central Bureau of Statistics (Nepal), United Nations Children's Fund (UNICEF). Nepal Multiple Indicator Cluster Survey 2014. New York, United States of America: United Nations Children's Fund (UNICEF), 2015.	GHDx
462617*	Nepal	Nepal Multiple Indicator Cluster Survey 2019	2019	I	I	I	I	I	I	I	I	I	I	-	Central Bureau of Statistics (Nepal), United Nations Children's Fund (UNICEF). Nepal Multiple Indicator Cluster Survey 2019. New York, United States of America: United Nations Children's Fund (UNICEF), 2020.	GHDx
528571*	Nepal	Nepal Demographic and Health Survey 2022	2022	I	I	I	I	I	I	I	I	I	-	I	ICF International, Ministry of Health and Population (Nepal), New ERA. Nepal Demographic and Health Survey 2022. Fairfax, United States of America: ICF International, 2023.	GHDx

GHDx ID	Country	Series	Year	BCG	DTP1	DTP3	HepB3	Hib3	MCV1	MCV2	PCV3	Pol3	RCV1	RotaC	Citation	Link
9002	Nepal	Nepal Fertility, Family Planning, and Health Survey 1991-1992	1991-1992	I	I	I	-	-	I	-	-	I	-	-	Ministry of Health (Nepal). Nepal Fertility, Family Planning, and Health Survey 1991-1992.	GHDx
141917	Nepal	Nepal Multiple Indicator Cluster Survey 1995-1998	1995-1998	I	I	I	-	-	I	-	-	I	-	-	Community Information and Epidemiological Technologies (CIET), National Planning Commission (Nepal), New ERA, United Nations Children's Fund (UNICEF). Nepal Multiple Indicator Cluster Survey 1995-1998. New York, United States of America: United Nations Children's Fund (UNICEF).	GHDx
153587	Nepal	Nepal - Chitwan Valley Family Study 1996-1997	1996-1997	I	I	I	-	-	I	-	-	I	-	-	Institute for Social Research, University of Michigan. Nepal - Chitwan Valley Family Study 1996-1997.	GHDx
286782	Nepal	Nepal Demographic and Health Survey 2016-2017	2016-2017	I	I	I	I	I	I	-	E	I	-	-	ICF International, Ministry of Health (Nepal), New ERA. Nepal Demographic and Health Survey 2016-2017. Fairfax, United States of America: ICF International, 2017.	GHDx
9310	Nicaragua	Nicaragua Living Standards Measurement Survey 1993	1993	-	I	I	-	-	I	E	-	I	-	-	National Institute of Statistics and Censuses (Nicaragua), World Bank. Nicaragua Living Standards Measurement Survey 1993.	GHDx
9422	Nicaragua	Nicaragua Living Standards Measurement Survey 2001	2001	I	e	e	-	-	I	-	-	I	E	-	National Institute of Statistics and Censuses (Nicaragua), World Bank. Nicaragua Living Standards Measurement Survey 2001.	GHDx
20487	Nicaragua	Nicaragua Demographic and Health Survey 2001	2001	I	I	I	-	-	I	-	-	I	I	-	Macro International, Inc, Ministry of Health (Nicaragua), National Institute of Statistics and Censuses (Nicaragua). Nicaragua Demographic and Health Survey 2001. Fairfax, United States of America: ICF International.	GHDx
9278	Nicaragua	Nicaragua Reproductive Health Survey 1992-1993	1992-1993	I	-	I	-	-	I	-	-	I	-	-	Profamilia and Division of Reproductive Health-Centers for Disease Control and Prevention (CDC). (1993) Nicaragua Reproductive Health Survey 1992-1993. Profamilia, Managua, Nicaragua.	GHDx
20478	Nicaragua	Nicaragua Demographic and Health Survey 1997-1998	1997-1998	I	I	I	-	-	I	-	-	I	-	-	Macro International, Inc, Ministry of Health (Nicaragua), National Institute of Statistics and Censuses (Nicaragua). Nicaragua Demographic and Health Survey 1997-1998. Fairfax, United States of America: ICF International.	GHDx

GHDx ID	Country	Series	Year	BCG	DTP1	DTP3	HepB3	Hib3	MCV1	MCV2	PCV3	Pol3	RCV1	RotaC	Citation	Link
9370	Nicaragua	Nicaragua Living Standards Measurement Survey 1998-1999	1998-1999	-	I	I	-	-	I	E	-	I	-	-	National Institute of Statistics and Censuses (Nicaragua), World Bank. Nicaragua Living Standards Measurement Survey 1998-1999.	GHDx
9270	Nicaragua	Nicaragua Reproductive Health Survey 2006-2007	2006-2007	I	I	I	I	I	I	-	-	I	I	-	Division of Reproductive Health, Centers for Disease Control and Prevention (CDC), National Institute for Development Information (Nicaragua). Nicaragua Reproductive Health Survey 2006-2007. Managua, Nicaragua: National Institute for Development Information (Nicaragua).	GHDx
20518	Niger	Niger Demographic and Health Survey 1992	1992	I	I	I	-	-	I	-	-	I	-	-	Department of Statistics and National Accounts (Niger), Macro International, Inc. Niger Demographic and Health Survey 1992. Fairfax, United States of America: ICF International.	GHDx
9469	Niger	Niger Continuous Survey on Economic and Social Conditions 1994	1994	I	I	I	-	-	I	-	-	I	-	-	Department of Statistics and National Accounts (Niger), United Nations Department of Economic and Social Development. Niger Continuous Survey on Economic and Social Conditions 1994. Niamey, Niger: Department of Statistics and National Accounts (Niger).	GHDx
9465	Niger	Niger Continuous Survey on Economic and Social Conditions 1995-1996	1995	I	I	I	-	-	I	-	-	I	-	-	Department of Statistics and National Accounts (Niger), United Nations Department of Economic and Social Development. Niger Continuous Survey on Economic and Social Conditions 1995-1996. Niamey, Niger: Department of Statistics and National Accounts (Niger).	GHDx
9477	Niger	Niger Multiple Indicator Cluster Survey 1996	1996	I	-	I	-	-	I	-	-	I	-	-	Centers for Disease Control and Prevention (CDC), Government of Niger, United Nations Children's Fund (UNICEF), United Nations Population Fund (UNFPA). Niger Multiple Indicator Cluster Survey 1996.	GHDx
20537	Niger	Niger Demographic and Health Survey 1998	1998	I	I	I	-	-	I	-	-	I	-	-	CARE International, Macro International, Inc. Niger Demographic and Health Survey 1998. Fairfax, United States of America: ICF International.	GHDx
9439	Niger	Niger Multiple Indicator Cluster Survey 2000	2000	I	I	I	-	-	I	-	-	I	-	-	Government of Niger, Macro International, Inc, United Nations Children's Fund (UNICEF). Niger Multiple Indicator Cluster Survey 2000. New York, United States of America: United Nations Children's Fund (UNICEF).	GHDx

GHDx ID	Country	Series	Year	BCG	DTP1	DTP3	HepB3	Hib3	MCV1	MCV2	PCV3	Pol3	RCV1	RotaC	Citation	Link
20499	Niger	Niger Demographic and Health Survey 2006	2006	I	I	I	-	-	I	-	-	I	-	-	Department of Statistics and National Accounts (Niger), Macro International, Inc. Niger Demographic and Health Survey 2006. Fairfax, United States of America: ICF International.	GHDx
160198	Niger	Niger Nutrition and Child Survival Survey 2008	2008	-	I	I	-	-	I	-	-	-	-	-	Ministry of Public Health (Niger), National Institute of Statistics (Niger). Niger Nutrition and Child Survival Survey 2008.	GHDx
160053	Niger	Niger Nutrition and Child Survival Survey 2009	2009	I	I	I	-	-	I	-	-	-	-	-	Ministry of Public Health (Niger), National Institute of Statistics (Niger). Niger Nutrition and Child Survival Survey 2009.	GHDx
160040	Niger	Niger Nutrition and Child Survival Survey 2010	2010	I	I	I	-	-	I	-	-	I	-	-	Ministry of Public Health (Niger), National Institute of Statistics (Niger). Niger Nutrition and Child Survival Survey 2010.	GHDx
74393	Niger	Niger Demographic and Health Survey 2012	2012	I	I	I	I	I	I	-	-	I	-	-	ICF International, Ministry of Public Health (Niger), National Institute of Statistics (Niger). Niger Demographic and Health Survey 2012. Fairfax, United States of America: ICF International, 2019.	GHDx
505627*	Niger	Niger National Vaccination Coverage Survey 2017	2017	I	I	I	I	I	-	-	I	I	-	I	Ministry of Public Health (Niger), United Nations Children's Fund (UNICEF), World Health Organization (WHO). Niger National Vaccination Coverage Survey 2017.	GHDx
20552	Nigeria	Nigeria Demographic and Health Survey 1990	1990	I	I	I	-	-	I	-	-	I	-	-	Federal Office of Statistics (Nigeria), Institute for Resource Development, Macro Systems. Nigeria Demographic and Health Survey 1990. Fairfax, United States of America: ICF International.	GHDx
9504	Nigeria	Nigeria Multiple Indicator Cluster Survey 1995	1995	I	-	I	-	-	I	-	-	I	-	-	Federal Office of Statistics (Nigeria), United Nations Children's Fund (UNICEF). Nigeria Multiple Indicator Cluster Survey 1995.	GHDx
58333	Nigeria	Nigeria Integrated Baseline Survey 1995	1995	I	-	I	-	-	I	-	-	-	-	-	Basic Support for Institutionalizing Child Survival (BASICS), Centers for Disease Control and Prevention (CDC), Johns Hopkins University, United States Agency for International Development (USAID). Nigeria Integrated Baseline Survey 1995.	GHDx
9506	Nigeria	Nigeria Multiple Indicator Cluster Survey 1999	1999	I	I	I	-	-	I	-	-	I	-	-	National Bureau of Statistics (Nigeria), United Nations Children's Fund (UNICEF). Nigeria Multiple Indicator Cluster Survey 1999. Abuja, Nigeria: National Bureau of Statistics (Nigeria).	GHDx

GHDx ID	Country	Series	Year	BCG	DTP1	DTP3	HepB3	Hib3	MCV1	MCV2	PCV3	Pol3	RCV1	RotaC	Citation	Link
20555	Nigeria	Nigeria Demographic and Health Survey 1999	1999	I	I	I	-	-	I	-	-	I	-	-	Macro International, Inc, National Population Commission of Nigeria. Nigeria Demographic and Health Survey 1999. Calverton, United States of America: Macro International, Inc.	GHDx
20567	Nigeria	Nigeria Demographic and Health Survey 2003	2003	I	I	I	-	-	I	-	-	I	-	-	Department for International Development (DFiD) (United Kingdom), National Population Commission of Nigeria, ORC Macro, United Nations Children's Fund (UNICEF), United Nations Population Fund (UNFPA). Nigeria Demographic and Health Survey 2003. Fairfax, United States of America: ICF International.	GHDx
231803	Nigeria	Nigeria National Immunization Coverage Survey 2003	2003	I	I	I	-	-	I	-	-	I	-	-	World Health Organization (WHO). Nigeria National Immunization Coverage Survey 2003.	GHDx
231801	Nigeria	Nigeria National Immunization Coverage Survey 2006	2006	I	I	I	I	-	I	-	-	I	-	-	World Health Organization (WHO). Nigeria National Immunization Coverage Survey 2006.	GHDx
9516	Nigeria	Nigeria Multiple Indicator Cluster Survey 2007	2007	I	I	I	E	-	I	-	-	I	-	-	United Nations Children's Fund (UNICEF), National Bureau of Statistics (Nigeria). Nigeria Multiple Indicator Cluster Survey 2007. New York, United States: United Nations Children's Fund (UNICEF).	GHDx
21433	Nigeria	Nigeria Demographic and Health Survey 2008	2008	I	I	I	-	-	I	-	-	I	-	-	Macro International, Inc, National Population Commission of Nigeria. Nigeria Demographic and Health Survey 2008. Fairfax, United States of America: ICF International, 2009.	GHDx
231794	Nigeria	Nigeria National Immunization Coverage Survey 2010	2010	I	I	I	-	-	I	-	-	I	-	-	National Primary Health Care Development Agency (NPHCDA) (Nigeria), World Health Organization (WHO). Nigeria National Immunization Coverage Survey 2010.	GHDx
76703	Nigeria	Nigeria Multiple Indicator Cluster Survey 2011	2011	I	I	I	I	-	I	-	-	I	-	-	National Bureau of Statistics (Nigeria), United Nations Children's Fund (UNICEF). Nigeria Multiple Indicator Cluster Survey 2011. New York, United States of America: United Nations Children's Fund (UNICEF), 2013.	GHDx
77390	Nigeria	Nigeria Demographic and Health Survey 2013	2013	I	I	I	I	E	I	-	-	I	-	-	ICF International, National Population Commission of Nigeria. Nigeria Demographic and Health Survey 2013. Fairfax, United States of America: ICF International.	GHDx

GHDx ID	Country	Series	Year	BCG	DTP1	DTP3	HepB3	Hib3	MCV1	MCV2	PCV3	Pol3	RCV1	RotaC	Citation	Link
151732	Nigeria	Nigeria Standardized Monitoring and Assessment of Relief and Transitions Survey, Round V 2013	2013	-	I	I	-	-	I	-	-	-	-	-	National Bureau of Statistics (Nigeria), United Nations Children's Fund (UNICEF). Nigeria Standardized Monitoring and Assessment of Relief and Transitions Survey, Round V 2013.	GHDx
151733	Nigeria	Nigeria Standardized Monitoring and Assessment of Relief and Transitions Survey, Round V 2013 - Women and Children Tabulations	2013	-	I	I	-	-	I	-	-	-	-	-	National Bureau of Statistics (Nigeria), United Nations Children's Fund (UNICEF). Nigeria Standardized Monitoring and Assessment of Relief and Transitions Survey, Round V 2013 - Women and Children Tabulations.	GHDx
274708	Nigeria	Nigeria National Nutrition and Health Survey 2014	2014	-	I	I	-	-	I	-	-	-	-	-	National Bureau of Statistics (Nigeria). National Nutrition and Health Survey 2014-v1.0.	GHDx
274707	Nigeria	Nigeria National Nutrition and Health Survey 2015	2015	-	I	I	-	-	I	-	-	-	-	-	National Bureau of Statistics (Nigeria). Nigeria National Nutrition and Health Survey 2015.Â v1.0.	GHDx
408484	Nigeria	Nigeria Demographic and Health Survey 2018	2018	I	I	I	I	I	I	E	I	I	-	-	Federal Ministry of Health (Nigeria), ICF International, National Population Commission (NPC). Nigeria Demographic and Health Survey 2018. Fairfax, United States of America: ICF International, 2020.	GHDx
494197*	Nigeria	Nigeria National Nutrition and Health Survey 2018	2018	-	I	I	I	I	I	-	-	-	-	-	Federal Ministry of Health (Nigeria), National Bureau of Statistics (Nigeria), National Population Commission of Nigeria. Nigeria National Nutrition and Health Survey 2018.	GHDx
505452*	Nigeria	Nigeria Multiple Indicator Cluster Survey and National Immunization Coverage Survey 2021	2021	I	I	I	I	I	I	-	I	I	-	-	National Bureau of Statistics (Nigeria), United Nations Children's Fund (UNICEF). Nigeria Multiple Indicator Cluster Survey and National Immunization Coverage Survey 2021. New York, United States of America: United Nations Children's Fund (UNICEF).	GHDx
20573	Nigeria	Nigeria - Ondo Special Demographic and Health Survey 1986-1987	1986-1987	I	I	I	-	-	I	-	-	I	-	-	Federal Ministry of Health (Nigeria), Macro Systems, Inc.; Institute for Resource Development, National Population Bureau (Nigeria). Nigeria - Ondo Special Demographic and Health Survey 1986-1987. Fairfax, United States of America: ICF International.	GHDx
253019	Nigeria	Nigeria Report on Children, Adolescents and Youth	1991-1999	I	I	I	-	-	I	-	-	I	-	-	National Population Commission of Nigeria. Nigeria Report on Children, Adolescents and Youth. Abuja, Nigeria: National Population Commission of Nigeria.	GHDx
25006*	Nigeria	Nigeria Living Standards Survey 2003-2004	2003-2004	I	I	I	-	-	I	-	-	-	-	-	Federal Office of Statistics (Nigeria). Nigeria Living Standards Survey 2003-2004.	GHDx

GHDx ID	Country	Series	Year	BCG	DTP1	DTP3	HepB3	Hib3	MCV1	MCV2	PCV3	Pol3	RCV1	RotaC	Citation	Link
33188	Nigeria	Nigeria Annual Abstract of Statistics 2009	2003-2008	I	I	I	-	-	I	-	-	I	-	-	National Bureau of Statistics (Nigeria). Nigeria Annual Abstract of Statistics 2009. Abuja, Nigeria: National Bureau of Statistics (Nigeria).	GHDx
151719	Nigeria	Nigeria Living Standards Survey 2008-2010	2008-2010	I	I	I	-	-	e	-	-	I	-	-	National Bureau of Statistics (Nigeria). Nigeria Living Standards Survey 2008-2010. Abuja, Nigeria: National Bureau of Statistics (Nigeria).	GHDx
151797	Nigeria	Nigeria General Household Survey, Panel 2012-2013, Wave 2	2012-2013	e	e	e	-	-	e	-	-	e	E	-	National Bureau of Statistics (Nigeria). Nigeria General Household Survey, Panel 2012-2013, Wave 2. Washington DC, United States of America: World Bank.	GHDx
437724*	Nigeria	Nigeria Multiple Indicator Cluster Survey with National Immunization Coverage Survey Supplement 2016-2017 - National Bureau of Statistics	2016-2017	I	I	I	I	I	I	-	I	I	-	-	National Agency for the Control of AIDS (Nigeria), National Bureau of Statistics (Nigeria), National Primary Health Care Development Agency (NPHCDA) (Nigeria), United Nations Children's Fund (UNICEF). Nigeria Multiple Indicator Cluster Survey with National Immunization Coverage Survey Supplement 2016-2017 - National Bureau of Statistics. New York, United States of America: United Nations Children's Fund (UNICEF), 2018.	GHDx
564903*	Nigeria	Nigeria Demographic and Health Survey 2023-2024	2023-2024	I	I	I	I	I	I	-	I	I	-	I	ICF International, National Population Commission of Nigeria. Nigeria Demographic and Health Survey 2023-2024. Fairfax, United States of America: ICF International.	GHDx
7465	North Korea	North Korea Multiple Indicator Cluster Survey 1998	1998	I	-	I	-	-	I	-	-	I	-	-	European Union (EU), Government of North Korea, United Nations Children's Fund (UNICEF), World Food Programme (WFP). North Korea Multiple Indicator Cluster Survey 1998.	GHDx
7467	North Korea	North Korea Multiple Indicator Cluster Survey 2000	2000	I	e	e	-	-	I	-	-	I	-	-	Central Bureau of Statistics (North Korea), United Nations Children's Fund (UNICEF). North Korea Multiple Indicator Cluster Survey 2000.	GHDx
58954	North Korea	North Korea Nutrition Assessment 2004	2004	I	-	I	-	-	I	-	-	I	-	-	Central Bureau of Statistics (North Korea), United Nations Children's Fund (UNICEF), World Food Programme (WFP). North Korea Nutrition Assessment 2004.	GHDx
58950	North Korea	North Korea EPI Coverage Evaluation Survey 2008	2008	I	-	-	-	-	I	-	-	I	-	-	Ministry of Public Health (North Korea), United Nations Children's Fund (UNICEF), World Health Organization (WHO). North Korea EPI Coverage Evaluation Survey 2008.	GHDx

GHDx ID	Country	Series	Year	BCG	DTP1	DTP3	HepB3	Hib3	MCV1	MCV2	PCV3	Pol3	RCV1	RotaC	Citation	Link
355051	North Korea	North Korea Multiple Indicator Cluster Survey 2017	2017	I	I	I	I	I	I	I	-	I	-	-	Central Bureau of Statistics (North Korea), United Nations Children's Fund (UNICEF). North Korea Multiple Indicator Cluster Survey 2017.	GHDx
502495*	North Korea	Democratic People's Republic of Korea National Immunization Coverage Survey 2017	2017	I	I	I	I	I	I	I	-	I	-	-	Democratic People's Republic of Korea National Immunization Coverage Survey 2017.	GHDx
124337	North Macedonia	Macedonia Multiple Indicator Cluster Survey 1999	1999	I	-	I	-	-	-	-	-	-	-	-	Clinic for Children's Diseases, Clinic Center Skopje (Macedonia), Institute for Mother and Child Health, Health Home Skopje (Macedonia), Ministry of Health (Macedonia), National Nutrition Institute (Italy), Republic Institute for Health Protection (Macedonia), United Nations Children's Fund (UNICEF). Macedonia Multiple Indicator Cluster Survey 1999.	GHDx
7843	North Macedonia	Macedonia Multiple Indicator Cluster Survey 2005	2005	I	I	I	-	-	I	-	-	I	-	-	State Statistical Office (Macedonia) and United Nations Children's Fund (UNICEF). Macedonia Multiple Indicator Cluster Survey 2005. New York, United States: United Nations Children's Fund (UNICEF).	GHDx
91324	North Macedonia	Macedonia Multiple Indicator Cluster Survey 2011	2011	I	I	I	-	E	I	-	-	I	-	-	Institute of Public Health (Republic of North Macedonia), Ipsos Strategic Puls, Ministry of Education and Science (Macedonia), Ministry of Labor and Social Policy (Macedonia), United Nations Children's Fund (UNICEF). Macedonia Multiple Indicator Cluster Survey 2011. New York, United States of America: United Nations Children's Fund (UNICEF), 2013.	GHDx
453328*	North Macedonia	North Macedonia Multiple Indicator Cluster Survey 2018-2019	2018-2019	I	I	I	I	I	I	-	-	I	I	-	State Statistical Office and UNICEF. 2020. 2018-2019 North Macedonia Multiple Indicator Cluster Survey and 2018-2019 North Macedonia Roma Settlements Multiple Indicator Cluster Survey, Survey Findings Report. Skopje, North Macedonia: State Statistical Office and UNICEF.	GHDx
454053*	Norway	Norway Immunization Registry 2018	2013-2018	-	-	I	-	I	I	-	I	I	I	-	Norwegian Institute of Public Health. Norway Immunization Registry 2018. Oslo, Norway: Norwegian Institute of Public Health.	GHDx

GHDx ID	Country	Series	Year	BCG	DTP1	DTP3	HepB3	Hib3	MCV1	MCV2	PCV3	Pol3	RCV1	RotaC	Citation	Link
231783	Pakistan	Pakistan EPI Coverage Evaluation Survey 2005	2005	I	I	I	I	-	I	-	-	I	-	-	Ministry of Health (Pakistan). Pakistan EPI Coverage Evaluation Survey 2005.	GHDx
141521	Pakistan	Pakistan National Nutrition Survey 2011	2011	I	-	I	I	I	I	-	-	I	-	-	Aga Khan University, Ministry of National Health Services, Regulations & Coordination (Pakistan), Ministry of Planning and Development (Pakistan), Pakistan Medical Research Council, United Nations Children's Fund (UNICEF). Pakistan National Nutrition Survey 2011.	GHDx
529979*	Pakistan	Pakistan Third-Party Verification of Immunization Coverage Survey 2022	2022	I	I	I	I	I	I	-	I	I	-	-	Aga Khan University, Ministry of Health (Pakistan), Pakistan Bureau of Statistics. Pakistan Third-Party Verification of Immunization Coverage Survey 2022. Karachi, Pakistan: Aga Khan University.	GHDx
20584	Pakistan	Pakistan Demographic and Health Survey 1990-1991	1990-1991	I	I	I	-	-	I	-	-	I	-	-	Institute for Resource Development, Macro Systems, National Institute of Population Studies (Pakistan). Pakistan Demographic and Health Survey 1990-1991. Fairfax, United States of America: ICF International.	GHDx
9602	Pakistan	Pakistan Integrated Household Survey 1995-1996	1995-1996	I	I	I	-	-	I	-	-	I	-	-	Pakistan Bureau of Statistics. Pakistan Integrated Household Survey 1995-1996.	GHDx
9585	Pakistan	Pakistan Fertility and Family Planning Survey 1996-1997	1996-1997	I	I	I	-	-	I	-	-	I	-	-	Centre for Population Studies, London School of Hygiene and Tropical Medicine, National Institute of Population Studies (Pakistan). Pakistan Fertility and Family Planning Survey 1996-1997.	GHDx
9658	Pakistan	Pakistan Integrated Household Survey/ Household Integrated Economic Survey 1998-1999	1998-1999	I	I	I	-	-	I	-	-	I	-	-	Federal Bureau of Statistics (Pakistan). Pakistan Integrated Household Survey 1998-1999. Islamabad, Pakistan: Federal Bureau of Statistics (Pakistan).	GHDx
9720	Pakistan	Pakistan Integrated Household Survey/ Household Integrated Economic Survey 2001-2002	2001-2002	I	I	I	-	-	I	-	-	I	-	-	Federal Bureau of Statistics (Pakistan). Pakistan Integrated Household Survey 2001-2002. Islamabad, Pakistan: Federal Bureau of Statistics (Pakistan).	GHDx
9951	Pakistan	Pakistan Social and Living Standards Measurement Survey/ Household Integrated Economic Survey 2004-2005	2004-2005	I	I	I	-	-	I	-	-	I	-	-	Federal Bureau of Statistics (Pakistan). Pakistan Social and Living Standards Measurement Survey 2004-2005. Islamabad, Pakistan: Federal Bureau of Statistics (Pakistan).	GHDx

GHDx ID	Country	Series	Year	BCG	DTP1	DTP3	HepB3	Hib3	MCV1	MCV2	PCV3	Pol3	RCV1	RotaC	Citation	Link
24818	Pakistan	Pakistan Social and Living Standards Measurement Survey/ Household Integrated Economic Survey 2005-2006	2005-2006	I	I	I	I	-	I	-	-	I	-	-	Federal Bureau of Statistics (Pakistan). Pakistan Social and Living Standards Measurement Survey 2005-2006. Islamabad, Pakistan: Federal Bureau of Statistics (Pakistan).	GHDx
20595	Pakistan	Pakistan Demographic and Health Survey 2006-2007	2006-2007	I	I	I	I	-	I	-	-	I	-	-	Macro International, Inc, National Institute of Population Studies (Pakistan). Pakistan Demographic and Health Survey 2006-2007. Fairfax, United States of America: ICF International.	GHDx
30634	Pakistan	Pakistan Social and Living Standards Measurement Survey/ Household Integrated Economic Survey 2007-2008	2007-2008	I	I	I	I	-	I	-	-	I	-	-	Federal Bureau of Statistics (Pakistan). Pakistan Social and Living Standards Measurement Survey 2007-2008. Islamabad, Pakistan: Federal Bureau of Statistics (Pakistan).	GHDx
165908	Pakistan	Pakistan Social and Living Standards Measurement Survey 2008-2009	2008-2009	I	I	I	-	-	I	-	-	I	-	-	Pakistan Bureau of Statistics. Pakistan Social and Living Standards Measurement Survey 2008-2009. Islamabad, Pakistan: Pakistan Bureau of Statistics.	GHDx
228002	Pakistan	Pakistan Social and Living Standards Measurement Survey 2010-2011	2010-2011	I	I	I	-	-	I	-	-	I	-	-	Pakistan Bureau of Statistics. Pakistan Social and Living Standards Measurement Survey 2010-2011. Islamabad, Pakistan: Pakistan Bureau of Statistics.	GHDx
77521	Pakistan	Pakistan Demographic and Health Survey 2012-2013	2012-2013	I	I	I	I	I	I	-	-	I	-	-	ICF International, National Institute of Population Studies (Pakistan), Pakistan Bureau of Statistics. Pakistan Demographic and Health Survey 2012-2013. Fairfax, United States of America: ICF International.	GHDx
238338	Pakistan	Pakistan Social and Living Standards Measurement Survey/ Household Integrated Economic Survey 2013-2014	2013-2014	I	I	I	I	I	I	-	-	I	-	-	Pakistan Bureau of Statistics. Pakistan Social and Living Standards Measurement Survey/ Household Integrated Economic Survey 2013-2014.	GHDx
286783	Pakistan	Pakistan Demographic and Health Survey 2017-2018	2017-2018	I	I	I	I	I	I	I	I	I	E	-	ICF International, Ministry of National Health Services, Regulations & Coordination (Pakistan), National Institute of Population Studies (Pakistan). Pakistan Demographic and Health Survey 2017-2018. Fairfax, United States of America: ICF International, 2018.	GHDx

GHDx ID	Country	Series	Year	BCG	DTP1	DTP3	HepB3	Hib3	MCV1	MCV2	PCV3	Pol3	RCV1	RotaC	Citation	Link
400526	Pakistan	Pakistan - Punjab Multiple Indicator Cluster Survey 2017-2018	2017-2018	I	I	I	I	I	I	I	I	I	-	-	Bureau of Statistics Punjab (Pakistan), United Nations Children's Fund (UNICEF). Pakistan - Punjab Multiple Indicator Cluster Survey 2017-2018. New York, United States of America: United Nations Children's Fund (UNICEF), 2019.	GHDx
446732*	Pakistan	Pakistan Social and Living Standards Measurement Survey/ Household Integrated Economic Survey 2018-2019	2018-2019	I	I	I	I	I	I	I	I	I	-	-	Government of Pakistan, Ministry of Planning and Development (Pakistan), Pakistan Bureau of Statistics. Pakistan Social and Living Standards Measurement Survey/ Household Integrated Economic Survey 2018-2019. Islamabad, Pakistan: Pakistan Bureau of Statistics, 2020.	GHDx
487290*	Pakistan	Pakistan Social and Living Standards Measurement Survey (District Level) 2019-2020	2019-2020	I	I	I	I	I	I	I	I	I	-	-	Pakistan Bureau of Statistics. Pakistan Social and Living Standards Measurement Survey (District Level) 2019-2020. Islamabad, Pakistan: Pakistan Bureau of Statistics, 2021.	GHDx
501977*	Pakistan	Pakistan Third-Party Verification of Immunization Coverage Survey 2020-2021	2020-2021	I	I	I	I	I	I	-	I	I	-	-	Aga Khan University, Ministry of Health (Pakistan), Pakistan Bureau of Statistics. Pakistan Third-Party Verification of Immunization Coverage Survey 2020-2021.	GHDx
142186	Palestine	Palestine - West Bank and Gaza Strip Multiple Indicator Cluster Survey 1996 - UNICEF	1996	e	-	-	-	-	I	-	-	I	E	-	Palestine - West Bank and Gaza Strip Health Survey 1996	GHDx
136572	Palestine	Palestine Health Status Annual Report 2000	2000	I	I	I	-	-	I	-	-	I	-	-	Ministry of Health (Palestine). Palestine Health Status Annual Report 2000. Nablus, Palestine: Ministry of Health (Palestine), 2001.	GHDx
125591	Palestine	Palestine Multiple Indicator Cluster Survey 2010	2010	I	I	I	I	-	I	-	-	I	-	-	Ministry of Health (Palestine), Palestinian Central Bureau of Statistics, United Nations Children's Fund (UNICEF), United Nations Population Fund (UNFPA). Palestine Multiple Indicator Cluster Survey 2010. New York, United States of America: United Nations Children's Fund (UNICEF), 2014.	GHDx
161590	Palestine	Palestine Multiple Indicator Cluster Survey 2014	2014	I	I	I	I	I	I	I	I	I	-	-	Ministry of Health (Palestine), Palestinian Central Bureau of Statistics, United Nations Children's Fund (UNICEF). Palestine Multiple Indicator Cluster Survey 2014. New York, United States of America: United Nations Children's Fund (UNICEF), 2015.	GHDx

GHDx ID	Country	Series	Year	BCG	DTP1	DTP3	HepB3	Hib3	MCV1	MCV2	PCV3	Pol3	RCV1	RotaC	Citation	Link
134754	Palestine	Palestine Health Status Annual Report 2001	1997-2001	I	I	-	-	-	I	-	-	I	-	-	Ministry of Health (Palestine). Palestine Health Status Annual Report 2001. Nablus, Palestine: Ministry of Health (Palestine), 2002.	GHDx
9999	Palestine	Palestine Family Health Survey 2006-2007	2006-2007	I	e	e	-	-	I	-	-	I	E	-	League of Arab States, Palestinian Central Bureau of Statistics, United Nations Children's Fund (UNICEF). Palestine Family Health Survey 2006-2007.	GHDx
464593*	Palestine	Palestine Multiple Indicator Cluster Survey 2019-2020	2019-2020	I	I	I	I	I	I	I	I	I	I	-	Ministry of Health (Palestine), Palestinian Central Bureau of Statistics, United Nations Children's Fund (UNICEF). Palestine Multiple Indicator Cluster Survey 2019-2020. New York, United States of America: United Nations Children's Fund (UNICEF), 2021.	GHDx
10063	Panama	Panama Family Planning/Maternal and Child Health Survey 1979	1979	I	-	I	-	-	I	-	-	I	-	-	Ministry of Health and Division of Reproductive Health-Centers for Disease Control and Prevention (CDC). Panama Family Planning/Maternal and Child Health Survey 1979. Ministry of Health, Ciudad de Panamá, Panamá, 1981.	GHDx
10277	Panama	Panama Living Standards Measurement Survey 1997	1997	I	I	I	-	-	-	-	-	I	-	-	Ministry of Planning and Economic Policy (Panama), World Bank. Panama Living Standards Measurement Survey 1997. Washington DC, United States of America: World Bank.	GHDx
10224	Panama	Panama Living Standard Measurement Survey 2003	2003	I	I	I	-	-	I	-	-	I	-	-	Census and Statistics Directorate (Panama), Ministry of Economy and Finance (Panama), World Bank. Panama Living Standard Measurement Survey 2003. Washington DC, United States of America: World Bank.	GHDx
46517	Panama	Panama Living Standard Measurement Survey 2008	2008	I	I	I	E	-	-	-	-	I	-	E	Census and Statistics Directorate (Panama), Ministry of Economy and Finance (Panama), World Bank. Panama Living Standard Measurement Survey 2008. Washington DC, United States of America: World Bank.	GHDx
161587	Panama	Panama Multiple Indicator Cluster Survey 2013	2013	I	I	I	I	I	I	E	E	I	I	I	National Institute of Statistics and Census (Panama), United Nations Children's Fund (UNICEF). Panama Multiple Indicator Cluster Survey 2013. New York, United States of America: United Nations Children's Fund (UNICEF).	GHDx

GHDx ID	Country	Series	Year	BCG	DTP1	DTP3	HepB3	Hib3	MCV1	MCV2	PCV3	Pol3	RCV1	RotaC	Citation	Link
20599	Papua New Guinea	Papua New Guinea Demographic and Health Survey 1996-1997	1996-1997	I	I	I	I	-	I	-	-	I	-	-	National Statistical Office (Papua New Guinea). Papua New Guinea Demographic and Health Survey 1996-1997.	GHDx
231774	Papua New Guinea	Papua New Guinea National Immunization Coverage Survey 2005-2006	2005-2006	I	I	I	I	-	I	-	-	I	-	-	National Department of Health (Papua New Guinea). Papua New Guinea National Immunization Coverage Survey 2005-2006.	GHDx
44870	Papua New Guinea	Papua New Guinea Demographic and Health Survey 2006-2007	2006-2007	I	I	I	I	-	I	-	-	I	-	-	National Statistical Office (Papua New Guinea), National Statistics Office (Philippines). Papua New Guinea Demographic and Health Survey 2006-2007.	GHDx
426238	Papua New Guinea	Papua New Guinea Demographic and Health Survey 2016-2018	2016-2018	I	I	I	I	I	I	E	I	I	I	-	ICF International, National Statistical Office (Papua New Guinea). Papua New Guinea Demographic and Health Survey 2016-2018. Fairfax, United States of America: ICF International, 2019.	GHDx
20608	Paraguay	Paraguay Demographic and Health Survey 1990	1990	I	I	I	-	-	I	-	-	I	-	-	Macro Systems, Inc.; Institute for Resource Development, Paraguayan Center for Population Studies (CEPEP). Paraguay Demographic and Health Survey 1990. Fairfax, United States of America: ICF International.	GHDx
10370	Paraguay	Paraguay Reproductive Health Survey 2004	2004	I	I	I	E	E	I	-	-	I	-	-	Division of Reproductive Health-Centers for Disease Control and Prevention (CDC). (2005): Paraguay Reproductive Health Survey 2004 Asunción, Paraguay, Paraguayan Center for Population Studies (CEPEP).	GHDx
41830	Paraguay	Paraguay Permanent Household Survey 2005	2005	I	-	-	-	-	I	-	-	-	-	-	Department of Statistics, Surveys and Censuses (Paraguay). Paraguay Permanent Household Survey 2005. AsunciÃƒÂ³n, Paraguay: Department of Statistics, Surveys and Censuses (Paraguay).	GHDx
41837	Paraguay	Paraguay Permanent Household Survey 2006	2006	I	-	-	-	-	I	-	-	-	-	-	Department of Statistics, Surveys and Censuses (Paraguay). Paraguay Permanent Household Survey 2006. AsunciÃƒÂ³n, Paraguay: Department of Statistics, Surveys and Censuses (Paraguay).	GHDx
27525	Paraguay	Paraguay Reproductive Health Survey 2008	2008	I	I	I	I	I	I	-	-	I	I	-	Paraguay Center for Population Studies (CEPEP). Paraguay Reproductive Health Survey 2008. AsunciÃƒÂ³n, Paraguay: Paraguayan Center for Population Studies (CEPEP).	GHDx

GHDx ID	Country	Series	Year	BCG	DTP1	DTP3	HepB3	Hib3	MCV1	MCV2	PCV3	Pol3	RCV1	RotaC	Citation	Link
231759	Paraguay	Paraguay National Vaccination Coverage Survey of Children Aged 12-35 Months 2011	2011	I	I	I	I	I	I	-	-	I	-	-	Paraguay National Vaccination Coverage Survey of Children Aged 12-35 Months 2011.	GHDx
324470	Paraguay	Paraguay Multiple Indicator Cluster Survey 2016	2016	I	I	I	I	I	I	-	-	I	-	I	General Directorate of Statistics, Surveys and Censuses (Paraguay), Ministry of Public Health and Social Welfare (Paraguay), United Nations Children's Fund (UNICEF). Paraguay Multiple Indicator Cluster Survey 2016. New York, United States of America: United Nations Children's Fund (UNICEF), 2017.	GHDx
10364	Paraguay	Paraguay Reproductive Health Survey 1995-1996	1995-1996	I	I	I	-	-	I	-	-	I	-	-	Division of Reproductive Health-Centers for Disease Control and Prevention (CDC). Paraguay Reproductive Health Survey 1995-1996. Asunción, Paraguay, Paraguayan Center for Population Studies (CEPEP).	GHDx
10357	Paraguay	Paraguay Integrated Household Survey 1997-1998	1997-1998	I	-	-	-	-	I	-	-	-	-	-	General Directorate of Statistics, Surveys and Censuses (Paraguay). Paraguay Integrated Household Survey 1997-1998. AsunciÃƒÂ³n, Paraguay: General Directorate of Statistics, Surveys and Censuses (Paraguay).	GHDx
10350	Paraguay	Paraguay Integrated Household Survey 2000-2001	2000-2001	I	-	-	-	-	I	-	-	-	-	-	General Directorate of Statistics, Surveys and Censuses (Paraguay). Paraguay Integrated Household Survey 2000-2001. AsunciÃƒÂ³n, Paraguay: General Directorate of Statistics, Surveys and Censuses (Paraguay).	GHDx
20638	Peru	Peru Demographic and Health Survey 1996	1996	I	I	I	-	-	I	-	-	I	-	-	Macro International, Inc, National Institute of Statistics (Peru). Peru Demographic and Health Survey 1996. Fairfax, United States of America: ICF International.	GHDx
43845	Peru	Peru National Household Survey, Second Quarter 1998	1998	I	I	I	-	-	I	-	-	I	-	-	National Institute of Statistics and Informatics (INEI) (Peru). Peru National Household Survey, Second Quarter 1998. Lima, Peru: National Institute of Statistics and Informatics (INEI) (Peru).	GHDx
20649	Peru	Peru Demographic and Health Survey 2000	2000	I	I	I	E	E	I	-	-	I	-	-	Macro International, Inc, National Institute of Statistics (Peru). Peru Demographic and Health Survey 2000. Fairfax, United States of America: ICF International.	GHDx

GHDx ID	Country	Series	Year	BCG	DTP1	DTP3	HepB3	Hib3	MCV1	MCV2	PCV3	Pol3	RCV1	RotaC	Citation	Link
44315	Peru	Peru National Household Survey, Second Quarter 2000	2000	-	I	I	-	-	-	-	-	I	-	-	National Institute of Statistics and Informatics (INEI) (Peru). Peru National Household Survey, Second Quarter 2000. Lima, Peru: National Institute of Statistics and Informatics (INEI) (Peru).	GHDx
270404	Peru	Peru Continuous Demographic and Health Survey 2009	2009	I	I	I	I	I	I	E	E	I	E	E	National Institute of Statistics and Informatics (Peru), ORC Macro. Peru Continuous Demographic and Health Survey 2009. Fairfax, United States of America: ICF International.	GHDx
270469	Peru	Peru Continuous Demographic and Health Survey 2010	2010	I	I	I	I	I	I	E	E	I	E	E	National Institute of Statistics and Informatics (Peru). Peru Continuous Demographic and Health Survey 2010. Fairfax, United States of America: ICF International.	GHDx
270470	Peru	Peru Continuous Demographic and Health Survey 2011	2011	I	I	I	I	I	I	E	E	I	E	I	Macro International, Inc, National Institute of Statistics and Informatics (Peru). Peru Continuous Demographic and Health Survey 2011. Fairfax, United States of America: ICF International.	GHDx
270471	Peru	Peru Continuous Demographic and Health Survey 2012	2012	I	I	I	I	I	I	E	E	I	E	I	Macro International, Inc, National Institute of Statistics and Informatics (Peru). Peru Continuous Demographic and Health Survey 2012. Fairfax, United States of America: ICF International.	GHDx
210231	Peru	Peru Continuous Demographic and Health Survey 2013 - INEI	2013	I	I	I	-	-	I	-	-	I	-	-	ICF International, National Institute of Statistics and Informatics (Peru). Peru Continuous Demographic and Health Survey 2013 - INEI. Lima, Peru: National Institute of Statistics and Informatics (Peru), 2014.	GHDx
210182	Peru	Peru Continuous Demographic and Health Survey 2014 - INEI	2014	I	I	I	-	-	I	-	-	I	-	-	ICF International, Ministry of Health (Peru), National Institute of Statistics and Informatics (Peru), National Police of Peru (PNP). Peru Continuous Demographic and Health Survey 2014 - INEI. Lima, Peru: National Institute of Statistics and Informatics (Peru), 2015.	GHDx
303663*	Peru	Peru Demographic and Family Health Survey 2015	2015	I	I	I	I	I	I	-	I	I	-	I	National Institute of Statistics and Informatics (Peru). Peru Demographic and Family Health Survey 2015. Lima, Peru: National Institute of Statistics and Informatics (Peru), 2017.	GHDx
303664*	Peru	Peru Demographic and Family Health Survey 2016	2016	I	I	I	-	-	I	-	-	I	-	-	National Institute of Statistics and Informatics (Peru). Peru Demographic and Family Health Survey 2016. Lima, Peru: National Institute of Statistics and Informatics (Peru), 2017.	GHDx

GHDx ID	Country	Series	Year	BCG	DTP1	DTP3	HepB3	Hib3	MCV1	MCV2	PCV3	Pol3	RCV1	RotaC	Citation	Link
358824	Peru	Peru Demographic and Family Health Survey 2017	2017	I	I	I	-	-	I	-	-	I	-	-	National Center for Food and Nutrition, National Institute of Health (Peru), National Institute of Statistics and Informatics (Peru), National Police of Peru (PNP). Peru Demographic and Family Health Survey 2017. Lima, Peru: National Institute of Statistics and Informatics (Peru).	GHDx
407869	Peru	Peru Demographic and Family Health Survey 2018	2018	I	I	I	-	-	I	-	-	I	-	-	National Institute of Statistics and Informatics (Peru). Peru Demographic and Family Health Survey 2018. Lima, Peru: National Institute of Statistics and Informatics (Peru), 2019.	GHDx
452894*	Peru	Peru Demographic and Family Health Survey 2019	2019	I	I	I	-	-	I	-	-	I	-	-	National Institute of Statistics and Informatics (Peru). Peru Demographic and Family Health Survey 2019. Lima, Peru: National Institute of Statistics and Informatics (Peru).	GHDx
20626	Peru	Peru Demographic and Health Survey 1991-1992	1991-1992	I	I	I	-	-	I	-	-	I	-	-	Macro International, Inc, National Institute of Statistics (Peru), PRISMA (Peru). Peru Demographic and Health Survey 1991-1992. Fairfax, United States of America: ICF International.	GHDx
275090	Peru	Peru Continuous Demographic and Health Survey 2003-2008	2003-2008	I	I	I	E	I	e	E	E	I	E	-	Ministry of Economy and Finance (Peru), National Institute of Statistics and Informatics (Peru), ORC Macro. Peru Continuous Demographic and Health Survey 2003-2008. Fairfax, United States of America: ICF International.	GHDx
129521	Peru	Peru Continuous Demographic and Health Survey 2004-2008	2004-2008	I	I	I	-	-	I	-	-	I	-	-	National Institute of Statistics and Informatics (Peru), Macro International, Inc. Peru Continuous Demographic and Health Survey 2003-2008.	GHDx
20674	Philippines	Philippines Demographic and Health Survey 1993	1993	I	I	I	-	-	I	-	-	I	-	-	Macro International, Inc, National Statistics Office (Philippines). Philippines Demographic and Health Survey 1993. Fairfax, United States of America: ICF International.	GHDx
10937	Philippines	Philippines Multiple Indicator Cluster Survey 1996	1996	I	-	I	-	-	I	-	-	I	-	-	National Statistics Office (Philippines), United Nations Children's Fund (UNICEF). Philippines Multiple Indicator Cluster Survey 1996.	GHDx
20683	Philippines	Philippines Demographic and Health Survey 1998	1998	I	I	I	I	-	I	-	-	I	-	-	Department of Health (Philippines), Macro International, Inc, National Statistics Office (Philippines). Philippines Demographic and Health Survey 1998. Fairfax, United States of America: ICF International.	GHDx

GHDx ID	Country	Series	Year	BCG	DTP1	DTP3	HepB3	Hib3	MCV1	MCV2	PCV3	Pol3	RCV1	RotaC	Citation	Link
338568	Philippines	Phillipines Maternal and Child Health Survey 2000	2000	I	I	I	I	-	I	-	-	I	-	-	National Statistics Office (Philippines), United States Census Bureau (USCB). Phillipines Maternal and Child Health Survey 2000.	GHDx
337557	Philippines	Philippines Maternal and Child Survey 2002	2002	I	I	I	-	-	I	-	-	I	-	-	Department of Health (Philippines), Philippines Statistics Authority. Philippines Maternal and Child Survey 2002. Manila, Philippines: Philippines Statistics Authority.	GHDx
20699	Philippines	Philippines Demographic and Health Survey 2003	2003	I	I	I	I	-	I	-	-	I	-	-	Macro International, Inc, National Statistics Office (Philippines). Philippines Demographic and Health Survey 2003. Fairfax, United States of America: ICF International.	GHDx
21421	Philippines	Philippines Demographic and Health Survey 2008	2008	I	I	I	I	E	I	-	-	I	-	-	Macro International, Inc, National Statistics Office (Philippines). Philippines Demographic and Health Survey 2008. Fairfax, United States of America: ICF International, 2010.	GHDx
248530	Philippines	Philippines Sub-Regional Multiple Indicator Survey 2012	2012	-	I	I	-	-	I	-	-	-	-	-	National Statistics Office (Philippines), United Nations Children's Fund (UNICEF). Philippines Sub-Regional Multiple Indicator Survey 2012.	GHDx
142943	Philippines	Philippines Demographic and Health Survey 2013	2013	I	I	I	E	E	I	-	-	I	E	E	ICF International, Philippines Statistics Authority. Philippines Demographic and Health Survey 2013. Fairfax, United States of America: ICF International, 2014.	GHDx
248532	Philippines	Philippines Multiple Indicator Survey 2014	2014	-	I	I	I	I	I	-	-	-	-	-	National Statistics Office (Philippines), United Nations Children's Fund (UNICEF), University of the Philippines, Manila. Philippines Multiple Indicator Survey 2014.	GHDx
264955	Philippines	Philippines Multiple Indicator Survey 2016	2016	-	I	I	I	I	I	E	-	I	-	I	Demographic Research and Development Foundation Inc. (Philippines), National Statistics Office (Philippines), United Nations Children's Fund (UNICEF). Philippines Multiple Indicator Survey 2016.	GHDx
337877	Philippines	Philippines Demographic and Health Survey 2017	2017	I	I	I	I	I	I	I	I	I	E	-	ICF International, Philippines Statistics Authority, United States Agency for International Development (USAID). Philippines Demographic and Health Survey 2017. Fairfax, United States of America: ICF International, 2018.	GHDx

GHDx ID	Country	Series	Year	BCG	DTP1	DTP3	HepB3	Hib3	MCV1	MCV2	PCV3	Pol3	RCV1	RotaC	Citation	Link
401704*	Philippines	Philippines Field Health Services Information System (FHSIS) Annual Report 2017	2017	I	I	I	I	I	I	I	I	I	I	-	Epidemiology Bureau, Department of Health (Philippines). Philippines Field Health Services Information System (FHSIS) Annual Report 2017. Philippines: Epidemiology Bureau, Department of Health (Philippines).	GHDx
415959*	Philippines	Philippines Field Health Services Information System (FHSIS) Annual Report 2018	2018	I	I	I	I	I	I	I	I	I	-	-	Epidemiology Bureau, Department of Health (Philippines). Philippines Field Health Services Information System (FHSIS) Annual Report 2018.	GHDx
527435*	Philippines	Philippines Demographic and Health Survey 2022	2022	I	I	I	I	I	I	I	I	I	-	-	ICF International, Philippine Statistics Authority, United States Agency for International Development (USAID). Philippines Demographic and Health Survey 2022. Fairfax, United States of America: ICF International, 2023.	GHDx
20711	Rwanda	Rwanda Demographic and Health Survey 1992	1992	I	I	I	-	-	I	-	-	I	-	-	Macro International, Inc, National Office of Population (Rwanda). Rwanda Demographic and Health Survey 1992. Fairfax, United States of America: ICF International.	GHDx
20722	Rwanda	Rwanda Demographic and Health Survey 2000	2000	I	I	I	-	-	I	-	-	I	-	-	Macro International, Inc, National Office of Population (Rwanda). Rwanda Demographic and Health Survey 2000. Fairfax, United States of America: ICF International.	GHDx
26930	Rwanda	Rwanda Multiple Indicator Cluster Survey 2000	2000	I	I	I	-	-	I	-	-	I	-	-	Department of Statistics (Rwanda), United Nations Children's Fund (UNICEF). Rwanda Multiple Indicator Cluster Survey 2000. New York, United States: United Nations Children's Fund (UNICEF).	GHDx
20740	Rwanda	Rwanda Demographic and Health Survey 2005	2005	I	I	I	-	-	I	-	-	I	-	-	Macro International, Inc, National Institute of Statistics of Rwanda. Rwanda Demographic and Health Survey 2005. Fairfax, United States of America: ICF International.	GHDx
231648	Rwanda	Rwanda National Immunization Coverage Survey 2007	2007	I	I	I	I	I	I	-	-	I	-	-	Ministry of Health (Rwanda). Rwanda National Immunization Coverage Survey 2007.	GHDx
231641	Rwanda	Rwanda Integrated Post Measles-Rubella Campaign and Routine Immunization Coverage Evaluation Survey 2013	2013	I	I	I	I	I	I	-	I	I	-	-	Government of Rwanda, World Health Organization (WHO). Rwanda Integrated Post Measles-Rubella Campaign and Routine Immunization Coverage Evaluation Survey 2013.	GHDx
558944*	Rwanda	Rwanda Routine Immunization Coverage Survey 2017	2017	I	I	I	I	I	I	-	I	I	-	I	Rwanda Routine Immunization Coverage Survey 2017.	GHDx

GHDx ID	Country	Series	Year	BCG	DTP1	DTP3	HepB3	Hib3	MCV1	MCV2	PCV3	Pol3	RCV1	RotaC	Citation	Link
11324	Rwanda	Rwanda Integrated Living Conditions Survey 2005-2006	2005-2006	I	I	-	-	-	-	-	-	-	-	-	National Institute of Statistics of Rwanda (NISR), Oxford Policy Management. Rwanda Integrated Living Conditions Survey 2005-2006. Kigali, Rwanda: National Institute of Statistics of Rwanda (NISR).	GHDx
21222	Rwanda	Rwanda Interim Demographic and Health Survey 2007-2008	2007-2008	I	I	I	-	-	I	-	-	I	-	-	Macro International, Inc, Ministry of Health (Rwanda), National Institute of Statistics of Rwanda. Rwanda Interim Demographic and Health Survey 2007-2008. Fairfax, United States of America: ICF International.	GHDx
56040	Rwanda	Rwanda Demographic and Health Survey 2010-2011	2010-2011	I	I	I	E	E	I	-	E	I	-	-	ICF Macro, Ministry of Health (Rwanda), National Institute of Statistics of Rwanda. Rwanda Demographic and Health Survey 2010-2011. Fairfax, United States of America: ICF International.	GHDx
151437	Rwanda	Rwanda Integrated Household Living Conditions Survey 2010-2011	2010-2011	I	I	I	-	-	I	-	-	I	-	-	National Institute of Statistics of Rwanda. Rwanda Integrated Household Living Conditions Survey 2010-2011. Kigali, Rwanda: National Institute of Statistics of Rwanda.	GHDx
157063	Rwanda	Rwanda Demographic and Health Survey 2014-2015	2014-2015	I	I	I	I	I	I	-	I	I	E	E	ICF International, Ministry of Health (Rwanda), National Institute of Statistics of Rwanda. Rwanda Demographic and Health Survey 2014-2015. Fairfax, United States of America: ICF International, 2016.	GHDx
462482*	Rwanda	Rwanda Demographic and Health Survey 2019-2020	2019-2020	I	I	I	I	I	I	I	I	I	-	I	Ministry of Health (Rwanda), National Institute of Statistics (Rwanda), Rwanda Biomedical Center (RBC). Rwanda Demographic and Health Survey 2019-2020. Fairfax, United States of America: ICF International, 2021.	GHDx
558943*	Rwanda	Rwanda Immunization Coverage Survey 2023-2024	2023-2024	I	I	I	I	I	I	I	I	I	-	-	Rwanda Immunization Coverage Survey 2023-2024.	GHDx
21313	Samoa	Samoa Demographic and Health Survey 2009	2009	I	I	I	-	-	I	-	-	I	-	-	ICF Macro, Ministry of Health (Samoa), Samoa Bureau of Statistics. Samoa Demographic and Health Survey 2009.	GHDx
267785	Samoa	Samoa Demographic and Health Survey 2014	2014	I	I	I	-	-	I	I	-	I	I	-	Ministry of Finance (Samoa), Ministry of Health (Samoa), Ministry of Women, Community and Social Development (Samoa). Samoa Demographic and Health Survey 2014.	GHDx

GHDx ID	Country	Series	Year	BCG	DTP1	DTP3	HepB3	Hib3	MCV1	MCV2	PCV3	Pol3	RCV1	RotaC	Citation	Link
456286*	Samoa	Samoa Multiple Indicator Cluster Survey 2019-2020	2019-2020	I	I	I	I	I	I	I	-	I	I	-	Pacific Community Statistics for Development Division, Samoa Bureau of Statistics, United Nations Children's Fund (UNICEF). Samoa Multiple Indicator Cluster Survey 2019-2020. New York, United States of America: United Nations Children's Fund (UNICEF), 2022.	GHDx
229674	São Tomé and Príncipe	Sao Tome and Principe Vaccination Coverage Survey 1990	1990	-	I	I	-	-	I	-	-	I	-	-	Sao Tome and Principe Vaccination Coverage Survey 1990.	GHDx
27055	São Tomé and Príncipe	Sao Tome and Principe Multiple Indicator Cluster Survey 2000	2000	e	e	e	-	-	e	-	-	e	-	-	National Institute of Statistics (Sao Tome and Principe), United Nations Children's Fund (UNICEF). Sao Tome and Principe Multiple Indicator Cluster Survey 2000. New York, United States of America: United Nations Children's Fund (UNICEF).	GHDx
11434	São Tomé and Príncipe	Sao Tome and Principe Multiple Indicator Cluster Survey 2006	2006	I	I	I	-	-	I	-	-	I	-	-	National Institute of Statistics (Sao Tome and Principe), United Nations Children's Fund (UNICEF). Sao Tome and Principe Multiple Indicator Cluster Survey 2006. New York, United States of America: United Nations Children's Fund (UNICEF).	GHDx
214640	São Tomé and Príncipe	Sao Tome and Principe Multiple Indicator Cluster Survey 2014	2014	I	I	I	I	I	I	-	I	I	-	-	Global Fund to Fight Aids Tuberculosis and Malaria (GFATM), ICF International, National Center for Endemic Diseases (CNE) (Sao Tome and Principe), National Institute of Statistics (Sao Tome and Principe), United Nations Children's Fund (UNICEF), United Nations Development Programme (UNDP). Sao Tome and Principe Multiple Indicator Cluster Survey 2014. New York, United States of America: United Nations Children's Fund (UNICEF), 2016.	GHDx
502496*	São Tomé and Príncipe	Sao Tome and Principe National Vaccination Coverage Survey 2017	2017	I	I	I	I	I	I	-	I	I	-	-	Sao Tome and Principe National Vaccination Coverage Survey 2017.	GHDx
464540*	São Tomé and Príncipe	Sao Tome and Principe Multiple Indicator Cluster Survey 2019	2019	I	I	I	I	I	I	I	I	I	I	-	National Institute of Statistics (Sao Tome and Principe), United Nations Children's Fund (UNICEF). Sao Tome and Principe Multiple Indicator Cluster Survey 2019. New York, United States of America: United Nations Children's Fund (UNICEF), 2021.	GHDx

GHDx ID	Country	Series	Year	BCG	DTP1	DTP3	HepB3	Hib3	MCV1	MCV2	PCV3	Pol3	RCV1	RotaC	Citation	Link
558941*	São Tomé and Príncipe	Sao Tome and Principe National Vaccination Coverage Survey 2023	2023	I	I	I	I	I	I	-	I	I	I	I	Sao Tome and Principe National Vaccination Coverage Survey 2023.	GHDx
26866	São Tomé and Príncipe	Sao Tome and Principe Demographic and Health Survey 2008-2009	2008-2009	I	I	I	-	-	I	-	-	I	-	-	ICF Macro, Ministry of Health (Sao Tome and Principe), National Institute of Statistics (Sao Tome and Principe). Sao Tome and Principe Demographic and Health Survey 2008-2009. Fairfax, United States of America: ICF International.	GHDx
11468	Saudi Arabia	Saudi Arabia Family Health Survey 1996-1997	1996-1997	I	I	I	-	-	I	-	-	I	-	-	Arab Fund for Economic and Social Development (AFESD), Arab Gulf Program for Development (AGFUND), Gulf-Co-operation Council (GCC), Ministry of Health (Saudi Arabia), United Nations Children's Fund (UNICEF), United Nations Population Fund (UNFPA), United Nations Statistics Division (UNSD), World Health Organization (WHO). Saudi Arabia Family Health Survey 1996-1997.	GHDx
20749	Senegal	Senegal Demographic and Health Survey 1986	1986	I	I	I	-	-	I	-	-	I	-	-	Department of Statistics (Senegal), Westinghouse; Institute for Resource Development. Senegal Demographic and Health Survey 1986. Fairfax, United States of America: ICF International.	GHDx
231637	Senegal	Senegal External EPI Review 2000	2000	I	I	I	-	-	I	-	-	I	-	-	Ministry of Health and Prevention (Senegal). Senegal External EPI Review 2000.	GHDx
26855	Senegal	Senegal Demographic and Health Survey 2005	2005	I	I	I	-	-	I	-	-	I	-	-	Ministry of Health and Prevention (Senegal), Research Center for Human Development (Senegal). Senegal Demographic and Health Survey 2005. Fairfax, United States of America: ICF International.	GHDx
231622	Senegal	Senegal EPI External Review 2010	2010	I	I	I	I	I	I	-	-	-	-	-	Maternal and Child Health Integrated Program (MCHIP), Ministry of Health and Prevention (Senegal), Program for Appropriate Technology in Health (PATH), United Nations Children's Fund (UNICEF), World Health Organization (WHO). Senegal EPI External Review 2010.	GHDx

GHDx ID	Country	Series	Year	BCG	DTP1	DTP3	HepB3	Hib3	MCV1	MCV2	PCV3	Pol3	RCV1	RotaC	Citation	Link
231585	Senegal	Senegal National EPI Coverage Survey 2013	2013	I	I	I	I	I	I	-	-	I	-	-	Global Alliance for Vaccines and Immunization (GAVI), Ministry of Health and Social Action (Senegal), Program for Appropriate Technology in Health (PATH), United Nations Children's Fund (UNICEF), United States Agency for International Development (USAID), World Health Organization (WHO). Senegal National EPI Coverage Survey 2013.	GHDx
191270	Senegal	Senegal Continuous Demographic and Health Survey 2014	2014	I	I	I	I	I	I	-	-	I	-	-	Cheikh Anta Diop University, ICF International, National Agency of Statistics and Demography (Senegal). Senegal Continuous Demographic and Health Survey 2014. Fairfax, United States of America: ICF International.	GHDx
218592	Senegal	Senegal Continuous Demographic and Health Survey 2015	2015	I	I	I	I	I	I	-	-	I	-	-	Cheikh Anta Diop University, ICF International, National Agency of Statistics and Demography (Senegal). Senegal Continuous Demographic and Health Survey 2015. Fairfax, United States of America: ICF International, 2016.	GHDx
286772	Senegal	Senegal Continuous Demographic and Health Survey 2016	2016	I	I	I	-	-	I	E	-	I	-	-	ICF International, Ministry of Health and Social Action (Senegal), National Agency of Statistics and Demography (Senegal). Senegal Continuous Demographic and Health Survey 2016. Fairfax, United States of America: ICF International, 2017.	GHDx
353526	Senegal	Senegal Continuous Demographic and Health Survey 2017	2017	I	I	I	I	I	I	I	I	I	-	I	ICF International, Ministry of Health and Social Action (Senegal), National Agency of Statistics and Demography (Senegal), Unit for the Fight Against Malnutrition (Senegal). Senegal Continuous Demographic and Health Survey 2017. Fairfax, United States of America: ICF International, 2018.	GHDx
450419	Senegal	Senegal Continuous Demographic and Health Survey 2018	2018	I	I	I	I	I	I	I	I	I	-	I	Directorate of Forecasting and Statistics, Ministry of the Economy, Finance and Planning (Senegal), ICF International, Ministry of Health and Social Action (Senegal), United States Agency for International Development (USAID). Senegal Continuous Demographic and Health Survey 2018. Fairfax, United States of America: ICF International, 2020.	GHDx

GHDx ID	Country	Series	Year	BCG	DTP1	DTP3	HepB3	Hib3	MCV1	MCV2	PCV3	Pol3	RCV1	RotaC	Citation	Link
460813*	Senegal	Senegal Continuous Demographic and Health Survey 2019	2019	e	e	e	E	E	e	I	E	e	-	E	ICF International, Ministry of Health and Social Action (Senegal), National Agency of Statistics and Demography (Senegal), United States Agency for International Development (USAID). Senegal Continuous Demographic and Health Survey 2019. Fairfax, United States of America: ICF International, 2020.	GHDx
553289*	Senegal	Senegal Continuous Demographic and Health Survey 2023	2023	I	I	I	I	I	I	I	I	I	-	-	ICF International, Ministry of Health and Social Action (Senegal), National Agency of Statistics and Demography (Senegal). Senegal Continuous Demographic and Health Survey 2023. Fairfax, United States of America: ICF International, 2024.	GHDx
20767	Senegal	Senegal Demographic and Health Survey 1992-1993	1992-1993	I	I	I	-	-	I	-	-	I	-	-	Directorate of Forecasting and Statistics, Ministry of the Economy, Finance and Planning (Senegal), Macro International, Inc. Senegal Demographic and Health Survey 1992-1993. Fairfax, United States of America: ICF International.	GHDx
20786	Senegal	Senegal Demographic and Health Survey 1999-2000	1999-2000	I	I	I	-	-	I	-	-	I	-	-	Groupe SERDHA, Macro International, Inc, Ministry of Health and Prevention (Senegal). Senegal Demographic and Health Survey 1999-2000. Fairfax, United States of America: ICF International.	GHDx
56063	Senegal	Senegal Demographic and Health Survey 2010-2011	2010-2011	I	I	I	I	I	I	-	-	I	-	-	Center for Research in Human Development (CRDH), Cheikh Anta Diop University, Hospital Aristide Le Dantec, ICF Macro, National Agency of Statistics and Demography (Senegal). Senegal Demographic and Health Survey 2010-2011. Fairfax, United States of America: ICF International.	GHDx
77392	Senegal	Senegal Continuous Service Provision Assessment 2012-2013	2012-2013	I	-	I	I	I	I	-	-	I	-	-	ICF International, Ministry of Health and Social Action (Senegal), National Agency of Statistics and Demography (Senegal). Senegal Continuous Service Provision Assessment 2012-2013. Fairfax, United States of America: ICF International, 2015.	GHDx
111432	Senegal	Senegal Continuous Demographic and Health Survey 2012-2013	2012-2013	I	I	I	I	I	I	-	-	I	-	-	ICF International, Ministry of Health and Social Action (Senegal), National Agency of Statistics and Demography (Senegal). Senegal Continuous Demographic and Health Survey 2012-2013. Fairfax, United States of America: ICF International.	GHDx

GHDx ID	Country	Series	Year	BCG	DTP1	DTP3	HepB3	Hib3	MCV1	MCV2	PCV3	Pol3	RCV1	RotaC	Citation	Link
287639	Senegal	Senegal - Dakar Urban Multiple Indicator Cluster Survey 2015-2016	2015-2016	I	I	I	I	I	I	I	I	I	I	-	National Agency of Statistics and Demography (Senegal), United Nations Children's Fund (UNICEF). Senegal - Dakar Urban Multiple Indicator Cluster Survey 2015-2016. New York, United States of America: United Nations Children's Fund (UNICEF), 2018.	GHDx
27022	Serbia	Yugoslavia, Federal Republic Multiple Indicator Cluster Survey 1996	1996	I	-	I	-	-	-	-	-	-	-	-	Institute for Public Health (Montenegro), Institute for Public Health (Pristina), Institute of Public Health of Serbia, Ministry of Health (FR Yugoslavia), Mother Theresa Charity, United Nations Children's Fund (UNICEF). Yugoslavia, Federal Republic Multiple Indicator Cluster Survey 1996.	GHDx
27024	Serbia	Yugoslavia, Federal Republic Multiple Indicator Cluster Survey 2000	2000	I	I	I	-	-	I	-	-	I	I	-	Institute for Public Health (Montenegro), Institute of Public Health of Serbia, United Nations Children's Fund (UNICEF), World Health Organization (WHO). Yugoslavia, Federal Republic Multiple Indicator Cluster Survey 2000.	GHDx
150870	Serbia	Serbia Multiple Indicator Cluster Survey 2014	2014	I	I	I	I	I	I	-	-	I	I	-	Statistical Office of the Republic of Serbia. Serbia Multiple Indicator Cluster Survey 2014. New York, United States of America: United Nations Children's Fund (UNICEF), 2015.	GHDx
459477*	Serbia	Serbia Multiple Indicator Cluster Survey 2019	2019	I	I	I	I	I	-	-	E	I	-	-	Statistical Office of the Republic of Serbia and UNICEF. 2019. Serbia Multiple Indicator Cluster Survey and Serbia Roma Settlements Multiple Indicator Cluster Survey, 2019, Survey Findings Report. Belgrade, Serbia: Statistical Office of the Republic of Serbia and UNICEF.	GHDx
8819	Serbia	Serbia and Montenegro - Montenegro Multiple Indicator Cluster Survey 2005-2006	2005-2006	I	I	I	-	-	I	-	-	I	-	-	United Nations Children's Fund (UNICEF), Statistical Office of Montenegro. Montenegro Multiple Indicator Cluster Survey 2005. New York, United States: United Nations Children's Fund (UNICEF).	GHDx
11551	Serbia	Serbia and Montenegro - Serbia Multiple Indicator Cluster Survey 2005-2006	2005-2006	I	I	I	-	-	I	-	-	I	I	-	United Nations Children's Fund (UNICEF), Statistical Office of the Republic of Serbia (SORS), Strategic Marketing Research Agency (SMMRI). Serbia Multiple Indicator Cluster Survey 2005-2006. New York, United States: United Nations Children's Fund (UNICEF).	GHDx

GHDx ID	Country	Series	Year	BCG	DTP1	DTP3	HepB3	Hib3	MCV1	MCV2	PCV3	Pol3	RCV1	RotaC	Citation	Link
173776	Sierra Leone	Sierra Leone Multiple Indicator Cluster Survey 1995	1995	I	-	I	-	-	I	-	-	I	-	-	Central Statistics Office (Sierra Leone), Government of Sierra Leone, United Nations Children's Fund (UNICEF). Sierra Leone Multiple Indicator Cluster Survey 1995.	GHDx
11639	Sierra Leone	Sierra Leone Multiple Indicator Cluster Survey 2000	2000	I	I	I	-	-	I	-	-	I	-	-	Central Statistics Office (Sierra Leone), United Nations Children's Fund (UNICEF). Sierra Leone Multiple Indicator Cluster Survey 2000. New York, United States of America: United Nations Children's Fund (UNICEF).	GHDx
231381	Sierra Leone	Sierra Leone EPI National Coverage Evaluation Survey 2001	2001	I	I	I	-	-	I	-	-	I	-	-	Sierra Leone EPI National Coverage Evaluation Survey 2001.	GHDx
11649	Sierra Leone	Sierra Leone Multiple Indicator Cluster Survey 2005	2005	I	I	I	-	-	I	-	-	I	E	-	United Nations Children's Fund (UNICEF), Statistics Sierra Leone. Sierra Leone Multiple Indicator Cluster Survey 2005. New York, United States: United Nations Children's Fund (UNICEF).	GHDx
21258	Sierra Leone	Sierra Leone Demographic and Health Survey 2008	2008	I	I	I	E	E	I	-	-	I	-	-	Macro International, Inc, Statistics Sierra Leone. Sierra Leone Demographic and Health Survey 2008. Fairfax, United States of America: ICF International.	GHDx
76700	Sierra Leone	Sierra Leone Multiple Indicator Cluster Survey 2010	2010	I	I	I	E	I	e	-	-	I	-	-	Statistics Sierra Leone, United Nations Children's Fund (UNICEF). Sierra Leone Multiple Indicator Cluster Survey 2010. New York, United States of America: United Nations Children's Fund (UNICEF).	GHDx
229711	Sierra Leone	Sierra Leone Immunization Cluster Coverage Survey 2010	2010	I	I	I	I	I	I	-	-	I	-	-	Sierra Leone Immunization Cluster Coverage Survey 2010.	GHDx
274896	Sierra Leone	Sierra Leone Nutrition Survey 2010	2010	-	-	-	-	-	I	-	-	-	-	-	Ministry of Health (Sierra Leone), Statistics Sierra Leone. Sierra Leone Nutrition Survey 2010.	GHDx
131467	Sierra Leone	Sierra Leone Demographic and Health Survey 2013	2013	I	I	I	I	I	I	-	E	I	-	-	ICF International, Ministry of Health and Sanitation (Sierra Leone), Statistics Sierra Leone. Sierra Leone Demographic and Health Survey 2013. Fairfax, United States of America: ICF International, 2014.	GHDx
229705	Sierra Leone	Sierra Leone Routine Immunization Coverage Survey 2013	2013	I	I	I	I	I	I	-	I	I	-	-	Government of Sierra Leone. Sierra Leone Routine Immunization Coverage Survey 2013.	GHDx

GHDx ID	Country	Series	Year	BCG	DTP1	DTP3	HepB3	Hib3	MCV1	MCV2	PCV3	Pol3	RCV1	RotaC	Citation	Link
218619	Sierra Leone	Sierra Leone Multiple Indicator Cluster Survey 2017	2017	I	I	I	I	I	I	-	I	I	-	I	Statistics Sierra Leone, United Nations Children's Fund (UNICEF). Sierra Leone Multiple Indicator Cluster Survey 2017. New York, United States of America: United Nations Children's Fund (UNICEF), 2018.	GHDx
375813	Sierra Leone	Sierra Leone National Nutrition Survey 2017	2017	-	-	-	-	-	I	-	-	-	-	-	Action Against Hunger (ACF), Ministry of Health and Sanitation (Sierra Leone), Statistics Sierra Leone. Sierra Leone National Nutrition Survey 2017.	GHDx
425283*	Sierra Leone	Sierra Leone Demographic and Health Survey 2019	2019	e	e	e	E	E	e	I	E	e	-	E	ICF International, Ministry of Health and Sanitation (Sierra Leone), Statistics Sierra Leone. Sierra Leone Demographic and Health Survey 2019. Fairfax, United States of America: ICF International, 2020.	GHDx
157111	Singapore	Singapore State of Health 2001	1968-2001	I	-	I	I	-	I	-	-	I	-	-	Ministry of Health (Singapore). Singapore State of Health 2001. Singapore, Singapore: Ministry of Health (Singapore).	GHDx
322237	Solomon Islands	Solomon Islands Demographic and Health Survey 2015	2015	I	I	I	I	I	I	-	-	I	-	-	Department of Foreign Affairs and Trade (Australia), Ministry of Health and Medical Services (Solomon Islands), Secretariat of the Pacific Community (SPC), Solomon Islands National Statistics Office (SINSO), Solomon Islands Resource Facility (SIRF), United Nations Children's Fund (UNICEF). Solomon Islands Demographic and Health Survey 2015.	GHDx
21446	Solomon Islands	Solomon Islands Demographic and Health Survey 2006-2007	2006-2007	I	I	I	-	-	I	-	-	I	-	-	Macro International, Inc, Ministry of Health (Solomon Islands), Secretariat of the Pacific Community (SPC), Solomon Islands National Statistics Office (SINSO). Solomon Islands Demographic and Health Survey 2006-2007.	GHDx
151893	Somalia	Somalia Family Health Survey 1983	1983	I	e	e	-	-	I	-	-	I	-	-	Ministry of Health (Somalia), Ministry of National Planning (Somalia), Westinghouse Health Systems, Inc.. Somalia Family Health Survey 1983.	GHDx
11759	Somalia	Somalia Multiple Indicator Cluster Survey 1999	1999	I	I	I	-	-	I	-	-	I	-	-	United Nations Children's Fund (UNICEF). Somalia Multiple Indicator Cluster Survey 1999.	GHDx
11774	Somalia	Somalia Multiple Indicator Cluster Survey 2006	2006	I	I	I	-	-	I	-	-	I	-	-	Pan Arab Project for Family Health (PAPFAM), United Nations Children's Fund (UNICEF). Somalia Multiple Indicator Cluster Survey 2006. New York, United States of America: United Nations Children's Fund (UNICEF).	GHDx

GHDx ID	Country	Series	Year	BCG	DTP1	DTP3	HepB3	Hib3	MCV1	MCV2	PCV3	Pol3	RCV1	RotaC	Citation	Link
454381	Somalia	Somalia Health and Demographic Survey 2018-2019	2018-2019	I	I	I	I	I	I	-	-	I	-	-	Directorate of National Statistics (Somalia), Federal Government of Somalia, United Nations Population Fund (UNFPA). Somalia Health and Demographic Survey 2018-2019.	GHDx
139810	South Africa	South Africa Anthropometric, Vitamin A, Iron and Immunisation Coverage Status in Children Aged 6 to 71 Months 1994	1994	I	I	I	-	-	I	-	-	I	-	-	South African Vitamin A Consultative Group (SAVACG). South Africa Anthropometric, Vitamin A, Iron and Immunisation Coverage Status in Children Aged 6 to 71 Months 1994.	GHDx
20796	South Africa	South Africa Demographic and Health Survey 1998	1998	I	I	I	E	-	I	-	-	I	-	-	Department of Health (South Africa), Macro International, Inc, South African Medical Research Council. South Africa Demographic and Health Survey 1998. Fairfax, United States of America: ICF International.	GHDx
157064	South Africa	South Africa Demographic and Health Survey 2016	2016	I	I	I	I	I	I	I	I	I	-	I	Department of Health (South Africa), ICF International, South African Medical Research Council, Statistics South Africa. South Africa Demographic and Health Survey 2016. Fairfax, United States of America: ICF International, 2019.	GHDx
482845*	South Africa	South Africa Expanded Programme on Immunization (EPI) National Coverage Survey Report 2020	2019	I	I	I	I	I	I	I	I	I	-	-	South Africa Expanded Programme on Immunization (EPI) National Coverage Survey Report 2020.	GHDx
20798	South Africa	South Africa Demographic and Health Survey 2003-2004	2003-2004	I	I	I	-	-	I	-	-	I	-	-	Department of Health (South Africa), Macro International, Inc, South African Medical Research Council. South Africa Demographic and Health Survey 2003-2004.	GHDx
232705	South Korea	South Korea National Vaccine Coverage Survey 2008-2009	2008-2009	-	-	-	-	-	-	-	-	I	-	-	South Korea National Vaccine Coverage Survey 2008-2009.	GHDx
32189	South Sudan	Sudan - South Multiple Indicator Cluster Survey 2010	2010	e	e	e	E	E	e	-	-	e	-	-	Central Bureau of Statistics (Sudan), Federal Ministry of Health (Sudan), Government of Sudan, Ministry of Health (South Sudan), Southern Sudan Centre for Census, Statistics and Evaluation. Sudan - South Multiple Indicator Cluster Survey 2010. New York, United States of America: United Nations Children's Fund (UNICEF), 2015.	GHDx

GHDx ID	Country	Series	Year	BCG	DTP1	DTP3	HepB3	Hib3	MCV1	MCV2	PCV3	Pol3	RCV1	RotaC	Citation	Link
229684	South Sudan	South Sudan EPI Coverage Survey 2011-2012	2011-2012	I	I	I	-	-	I	-	-	I	-	-	Ministry of Health (South Sudan). South Sudan EPI Coverage Survey 2011-2012.	GHDx
502070*	South Sudan	South Sudan Expanded Program on Immunization Coverage Survey 2017-2018	2017-2018	I	I	I	I	I	I	-	-	I	-	-	Ministry of Health (South Sudan), South Sudan National Bureau of Statistics. South Sudan Expanded Program on Immunization Coverage Survey 2017-2018.	GHDx
20806	Sri Lanka	Sri Lanka Demographic and Health Survey 1987	1987	I	I	I	-	-	e	-	-	I	-	-	Department of Census and Statistics (Sri Lanka), Westinghouse; Institute for Resource Development. Sri Lanka Demographic and Health Survey 1987. Fairfax, United States of America: ICF International.	GHDx
90705	Sri Lanka	Sri Lanka Demographic and Health Survey 1993	1993	I	e	e	-	-	I	-	-	I	-	-	Department of Census and Statistics (Sri Lanka). Sri Lanka Demographic and Health Survey 1993. 2009.	GHDx
326837	Sri Lanka	Sri Lanka Demographic and Health Survey 2016	2016	I	I	I	I	I	I	I	-	I	I	-	Department of Census and Statistics (Sri Lanka), ICF International. Sri Lanka Demographic and Health Survey 2016.	GHDx
18815	Sri Lanka	Sri Lanka Demographic and Health Survey 2006-2007	2006-2007	I	I	I	E	-	I	-	-	I	-	-	Department of Census and Statistics (Sri Lanka), Macro International, Inc. Sri Lanka Demographic and Health Survey 2006-2007. 2009.	GHDx
141914	Sudan	Sudan Multiple Indicator Cluster Survey 1995	1995	-	I	I	-	-	I	-	-	I	-	-	Central Bureau of Statistics (Sudan), Federal Ministry of Health (Sudan), United Nations Children's Fund (UNICEF), University of Khartoum. Sudan Multiple Indicator Cluster Survey 1995.	GHDx
12243	Sudan	Sudan Multiple Indicator Cluster Survey 2000	2000	I	I	I	-	-	I	-	-	I	-	-	Central Bureau of Statistics (Sudan), Federal Ministry of Health (Sudan), United Nations Children's Fund (UNICEF). Sudan Multiple Indicator Cluster Survey 2000. New York, United States of America: United Nations Children's Fund (UNICEF).	GHDx
153643	Sudan	Sudan - North Multiple Indicator Cluster Survey 2010	2010	e	I	I	I	I	e	-	-	I	-	-	Central Bureau of Statistics (Sudan), Ministry of Health (South Sudan). Sudan - North Multiple Indicator Cluster Survey 2010. New York, United States of America: United Nations Children's Fund (UNICEF), 2015.	GHDx
200617	Sudan	Sudan Multiple Indicator Cluster Survey 2014	2014	I	I	I	I	I	I	E	-	I	-	-	Central Bureau of Statistics (Sudan), Federal Ministry of Health (Sudan), United Nations Children's Fund (UNICEF). Sudan Multiple Indicator Cluster Survey 2014. New York, United States of America: United Nations Children's Fund (UNICEF), 2016.	GHDx

GHDx ID	Country	Series	Year	BCG	DTP1	DTP3	HepB3	Hib3	MCV1	MCV2	PCV3	Pol3	RCV1	RotaC	Citation	Link
12289	Suriname	Suriname Multiple Indicator Cluster Survey 2006	2006	-	I	I	-	-	I	-	-	I	-	-	General Statistical Office (Suriname), United Nations Children's Fund (UNICEF). Suriname Multiple Indicator Cluster Survey 2006. New York, United States of America: United Nations Children's Fund (UNICEF).	GHDx
81203	Suriname	Suriname Multiple Indicator Cluster Survey 2010	2010	-	e	e	E	-	I	-	-	I	I	-	General Bureau of Statistics (Suriname), Ministry of Planning and Development Cooperation (Suriname), Ministry of Social Affairs and Housing (Suriname), United Nations Children's Fund (UNICEF). Suriname Multiple Indicator Cluster Survey 2010. New York, United States of America: United Nations Children's Fund (UNICEF), 2013.	GHDx
427983	Suriname	Suriname Multiple Indicator Cluster Survey 2018	2018	-	I	I	I	I	I	I	-	I	I	-	General Bureau of Statistics (Suriname), Ministry of Planning and Development Cooperation (Suriname), Ministry of Social Affairs and Housing (Suriname), United Nations Children's Fund (UNICEF). Suriname Multiple Indicator Cluster Survey 2018. New York, United States of America: United Nations Children's Fund (UNICEF), 2019.	GHDx
12280	Suriname	Suriname Multiple Indicator Cluster Survey 1999-2000	1999-2000	-	I	I	-	-	I	-	-	I	-	-	General Bureau of Statistics (Suriname), Pan American Health Organization (PAHO), United Nations Children's Fund (UNICEF), United Nations Development Programme (UNDP). Suriname Multiple Indicator Cluster Survey 1999-2000. New York, United States of America: United Nations Children's Fund (UNICEF).	GHDx
229595	Syria	Syria Immunization Campaign 1986	1986	I	I	I	-	-	I	-	-	I	-	-	Syria Immunization Campaign 1986.	GHDx
229594	Syria	Syria EPI/CDD Review 1988	1988	I	I	I	-	-	I	-	-	I	-	-	Syria EPI/CDD Review 1988.	GHDx
12377	Syria	Syria EPI/CDD and Child Mortality Survey 1990	1990	I	I	I	-	-	I	-	-	I	-	-	United Nations Children's Fund (UNICEF), World Health Organization (WHO). Syria EPI/CDD and Child Mortality Survey 1990.	GHDx
12388	Syria	Syria Maternal and Child Health Survey 1993	1993	I	-	I	-	-	I	-	-	I	-	-	Central Bureau of Statistics (Syria), League of Arab States. Syria Maternal and Child Health Survey 1993.	GHDx
12389	Syria	Syria Multiple Indicator Cluster Survey 1995	1995	I	I	I	-	E	I	I	-	I	-	-	United Nations Children's Fund (UNICEF). Syria Multiple Indicator Cluster Survey 1995.	GHDx

GHDx ID	Country	Series	Year	BCG	DTP1	DTP3	HepB3	Hib3	MCV1	MCV2	PCV3	Pol3	RCV1	RotaC	Citation	Link
12379	Syria	Syria Family Health Survey 2001	2001	-	I	I	I	-	I	-	-	I	E	-	Central Bureau of Statistics (Syria), League of Arab States. Syria Family Health Survey 2001.	GHDx
12399	Syria	Syria Multiple Indicator Cluster Survey 2006	2006	I	I	I	-	-	I	-	-	I	-	-	United Nations Children's Fund (UNICEF), Central Bureau of Statistics (Syria), Ministry of Health (Syria), Pan Arab Project for Family Health (PAPFAM). Syria Multiple Indicator Cluster Survey 2006. New York, United States: United Nations Children's Fund (UNICEF).	GHDx
126911	Syria	Syria Family Health Survey 2009	2009	I	I	I	-	-	I	-	-	I	-	-	Central Bureau of Statistics (Syria), League of Arab States. Syria Family Health Survey 2009.	GHDx
300048	Taiwan (Province of China)	Taiwan Statistics of Communicable Diseases and Surveillance Report 2015	2015	I	-	I	-	I	-	-	-	I	-	-	Centers for Disease Control, R.O.C (Taiwan). Taiwan Statistics of Communicable Diseases and Surveillance Report 2015. Taipei, Taiwan (Province of China): Centers for Disease Control, R.O.C (Taiwan), 2016.	GHDx
12595	Tajikistan	Tajikistan Multiple Indicator Cluster Survey 2000	2000	I	I	I	-	-	I	-	-	I	-	-	National State Statistical Agency (Tajikistan), United Nations Children's Fund (UNICEF). Tajikistan Multiple Indicator Cluster Survey 2000 . New York, United States: United Nations Children's Fund (UNICEF).	GHDx
12608	Tajikistan	Tajikistan Multiple Indicator Cluster Survey 2005	2005	I	I	I	-	-	I	-	-	I	-	-	United Nations Children's Fund (UNICEF), State Committee on Statistics of the Republic of Tajikistan. Tajikistan Multiple Indicator Cluster Survey 2005. New York, United States: United Nations Children's Fund (UNICEF).	GHDx
74460	Tajikistan	Tajikistan Demographic and Health Survey 2012	2012	I	I	I	I	I	I	-	-	I	-	-	ICF International, Ministry of Health (Tajikistan), Statistical Agency under the President of the Republic of Tajikistan. Tajikistan Demographic and Health Survey 2012. Fairfax, United States of America: ICF International, 2013.	GHDx
341838	Tajikistan	Tajikistan Demographic and Health Survey 2017	2017	I	I	I	I	I	I	-	-	I	-	I	ICF International, Statistical Agency under the President of the Republic of Tajikistan. Tajikistan Demographic and Health Survey 2017. Fairfax, United States of America: ICF International, 2018.	GHDx

GHDx ID	Country	Series	Year	BCG	DTP1	DTP3	HepB3	Hib3	MCV1	MCV2	PCV3	Pol3	RCV1	RotaC	Citation	Link
156672	Tajikistan	Tajikistan Health Care Systems in Transition 2000	1990-1998	-	-	-	-	-	I	-	-	-	-	-	European Observatory on Health Systems and Policies, World Health Organization Regional Office for Europe (WHO/Europe). Tajikistan Health Care Systems in Transition 2000. Copenhagen, Denmark: World Health Organization Regional Office for Europe (WHO/Europe), 2000.	GHDx
20852	Tanzania	Tanzania Demographic and Health Survey 1996	1996	I	I	I	-	-	I	-	-	I	-	-	Bureau of Statistics (Tanzania), Macro International, Inc, Planning Commission (Tanzania). Tanzania Demographic and Health Survey 1996. Fairfax, United States of America: ICF International.	GHDx
194016	Tanzania	Tanzania Multiple Indicator Cluster Survey 1996	1996	I	-	I	-	-	I	-	-	I	-	-	Government of Tanzania, Revolutionary Government of Zanzibar, United Nations Children's Fund (UNICEF). Tanzania Multiple Indicator Cluster Survey 1996.	GHDx
20865	Tanzania	Tanzania Demographic and Health Survey 1999	1999	I	I	I	-	-	I	-	-	I	-	-	Macro International, Inc, National Bureau of Statistics (Tanzania). Tanzania Demographic and Health Survey 1999. Fairfax, United States of America: ICF International.	GHDx
228513	Tanzania	Tanzania Integrated Measles and Routine Immunization: Post Campaign Coverage Evaluation Survey 2011	2011	I	I	I	I	I	I	-	-	I	-	-	Tanzania Integrated Measles and Routine Immunization: Post Campaign Coverage Evaluation Survey 2011.	GHDx
448257	Tanzania	Tanzania Post Integrated Measles Rubella Campaign Evaluation and Routine Immunization Coverage Survey 2014	2014	I	I	I	I	I	I	-	I	I	-	-	Ministry of Health and Social Welfare (Tanzania), Muhimbili University of Health and Allied Sciences (Tanzania), National Bureau of Statistics (Tanzania), World Health Organization (WHO). Tanzania Post Integrated Measles Rubella Campaign Evaluation and Routine Immunization Coverage Survey 2014.	GHDx
453458	Tanzania	Tanzania Post Measles-Rubella Campaign Evaluation 2019	2019	I	I	I	I	I	I	-	I	I	-	-	Tanzania Post Measles-Rubella Campaign Evaluation 2019.	GHDx
538795*	Tanzania	Tanzania Demographic and Health Survey 2022	2022	I	I	I	I	I	I	I	I	I	-	I	Bureau of Statistics (Tanzania), ICF International, Ministry of Health (Tanzania), Ministry of Health (Zanzibar), Office of the Chief Government Statistician (OCGS) (Zanzibar), Tanzania Food and Nutrition Centre. Tanzania Demographic and Health Survey 2022. Fairfax, United States of America: ICF International, 2023.	GHDx

GHDx ID	Country	Series	Year	BCG	DTP1	DTP3	HepB3	Hib3	MCV1	MCV2	PCV3	Pol3	RCV1	RotaC	Citation	Link
20841	Tanzania	Tanzania Demographic and Health Survey 1991-1992	1991-1992	I	I	I	-	-	I	-	-	I	-	-	Bureau of Statistics (Tanzania), Macro International, Inc, Ministry of Health (Tanzania). Tanzania Demographic and Health Survey 1991-1992. Fairfax, United States of America: ICF International.	GHDx
50513	Tanzania	Tanzania Reproductive and Child Health Survey 1999-2000	1999-2000	I	I	I	-	-	I	-	-	I	-	-	MEASURE Evaluation Project, Carolina Population Center, University of North Carolina, Ministry of Health (Tanzania), National Bureau of Statistics (Tanzania). Tanzania Reproductive and Child Health Survey 1999-2000. Chapel Hill, United States of America: MEASURE Evaluation Project, Carolina Population Center, University of North Carolina.	GHDx
20875	Tanzania	Tanzania Demographic and Health Survey 2004-2005	2004-2005	I	I	I	E	E	I	-	-	I	-	-	Macro International, Inc, National Bureau of Statistics (Tanzania). Tanzania Demographic and Health Survey 2004-2005. Fairfax, United States of America: ICF International.	GHDx
27297	Tanzania	Tanzania National Panel Survey 2008-2009	2008-2009	-	I	I	-	-	-	-	-	I	-	-	National Bureau of Statistics (Tanzania). Tanzania Living Standards Measurement Study - Integrated Survey on Agriculture 2008-2009. Dar es Salaam, Tanzania: National Bureau of Statistics (Tanzania).	GHDx
21331	Tanzania	Tanzania Demographic and Health Survey 2009-2010	2009-2010	I	I	I	E	E	I	-	-	I	-	-	ICF Macro, National Bureau of Statistics (Tanzania). Tanzania Demographic and Health Survey 2009-2010. Fairfax, United States of America: ICF International.	GHDx
218593	Tanzania	Tanzania Demographic and Health Survey 2015-2016	2015-2016	I	I	I	I	I	I	E	I	I	-	I	ICF International, Ministry of Health (Zanzibar), Ministry of Health, Community Development, Gender, Elderly and Children (MoHCDEC) (Tanzania), National Bureau of Statistics (Tanzania), Office of the Chief Government Statistician (OCGS) (Zanzibar). Tanzania Demographic and Health Survey 2015-2016. Fairfax, United States of America: ICF International, 2016.	GHDx
20887	Thailand	Thailand Demographic and Health Survey 1987	1987	I	I	I	-	-	e	-	-	I	-	-	Chulalongkorn University, Institute of Population Studies (Thailand), Westinghouse; Institute for Resource Development. Thailand Demographic and Health Survey 1987. Fairfax, United States of America: ICF International.	GHDx
229377	Thailand	Thailand EPI Coverage Survey 1990	1990	I	-	I	-	-	I	-	-	I	-	-	ASEAN Institute for Health Development (AIHD) (Thailand). Thailand EPI Coverage Survey 1990.	GHDx

GHDx ID	Country	Series	Year	BCG	DTP1	DTP3	HepB3	Hib3	MCV1	MCV2	PCV3	Pol3	RCV1	RotaC	Citation	Link
229348	Thailand	Thailand UNICEF Reporting Form for Immunization and Case Reporting 1996	1996	I	-	I	I	-	I	-	-	I	-	-	Thailand UNICEF Reporting Form for Immunization and Case Reporting 1996.	GHDx
228965	Thailand	Thailand Immunization Coverage Survey 1999	1999	I	I	I	I	-	I	-	-	I	-	-	Thailand Immunization Coverage Survey 1999.	GHDx
148649	Thailand	Thailand Multiple Indicator Cluster Survey 2012	2012	I	I	I	E	-	I	-	-	I	-	-	College of Population Studies, Chulalongkorn University (Thailand), Institute for Population and Social Research, Mahidol University (Thailand), International Health Policy Program (Thailand), Ministry of Education (Thailand), Ministry of Public Health (Thailand), Ministry of Social Development and Human Security (MSDHS) (Thailand), National Health Security Office (Thailand), National Statistical Office (Thailand), Thai Health Promotion Foundation, United Nations Children's Fund (UNICEF). Thailand Multiple Indicator Cluster Survey 2012. New York, United States of America: United Nations Children's Fund (UNICEF), 2016.	GHDx
459434*	Thailand	Thailand Multiple Indicator Cluster Survey 2019	2019	I	I	I	I	-	I	I	-	I	I	-	National Health Security Office (Thailand), National Statistical Office (Thailand), United Nations Children's Fund (UNICEF). Thailand Multiple Indicator Cluster Survey 2019. New York, United States of America: United Nations Children's Fund (UNICEF), 2020.	GHDx
529982*	Thailand	Thailand Multiple Indicator Cluster Survey 2022	2022	I	I	I	I	-	I	I	-	I	I	-	National Statistical Office (Thailand), United Nations Children's Fund (UNICEF). Thailand Multiple Indicator Cluster Survey 2022.	GHDx
12732	Thailand	Thailand Multiple Indicator Cluster Survey 2005-2006	2005-2006	I	I	I	I	-	I	-	-	I	-	-	National Statistical Office (Thailand), United Nations Children's Fund (UNICEF). Thailand Multiple Indicator Cluster Survey 2005-2006. New York, United States of America: United Nations Children's Fund (UNICEF).	GHDx
296646	Thailand	Thailand Multiple Indicator Cluster Survey 2015-2016	2015-2016	I	I	I	I	-	I	-	-	I	-	-	National Health Security Office (Thailand), National Statistical Office (Thailand), United Nations Children's Fund (UNICEF). Thailand Multiple Indicator Cluster Survey 2015-2016. New York, United States of America: United Nations Children's Fund (UNICEF), 2018.	GHDx

GHDx ID	Country	Series	Year	BCG	DTP1	DTP3	HepB3	Hib3	MCV1	MCV2	PCV3	Pol3	RCV1	RotaC	Citation	Link
233704	The Gambia	Gambia EPI Coverage Evaluation Survey 1992	1992	I	I	I	-	-	I	-	-	I	-	-	Gambia EPI Coverage Evaluation Survey 1992.	GHDx
142212	The Gambia	Gambia Multiple Indicator Cluster Survey 1996	1996	I	-	I	-	-	I	-	-	I	-	-	Central Statistics Department (The Gambia), United Nations Children's Fund (UNICEF). Gambia Multiple Indicator Cluster Survey 1996.	GHDx
3922	The Gambia	Gambia Multiple Indicator Cluster Survey 2000	2000	I	I	I	-	-	I	-	-	I	-	-	Central Statistics Department (Gambia), United Nations Children's Fund (UNICEF). Gambia Multiple Indicator Cluster Survey 2000. New York, United States: United Nations Children's Fund (UNICEF).	GHDx
233696	The Gambia	Gambia Immunization Coverage Survey 2002	2002	I	-	I	I	I	I	-	-	I	-	-	Gambia Immunization Coverage Survey 2002.	GHDx
233682	The Gambia	Gambia Immunization Survey 2005	2005	I	-	I	I	I	I	-	-	I	-	-	Gambia Immunization Survey 2005.	GHDx
233680	The Gambia	Gambia Immunization Survey 2006	2006	I	-	I	I	I	I	-	-	I	-	-	Gambia Immunization Survey 2006.	GHDx
91506	The Gambia	Gambia Multiple Indicator Cluster Survey 2010	2010	I	I	I	E	-	I	-	E	I	-	-	Gambia Bureau of Statistics (GBOS), United Nations Children's Fund (UNICEF). Gambia Multiple Indicator Cluster Survey 2010. New York, United States of America: United Nations Children's Fund (UNICEF), 2018.	GHDx
233678	The Gambia	Gambia EPI Cluster Survey 2012	2012	I	I	I	I	I	I	-	I	I	-	-	United Nations Children's Fund (UNICEF). Gambia EPI Cluster Survey 2012.	GHDx
77384	The Gambia	Gambia Demographic and Health Survey 2013	2013	I	I	I	I	I	I	E	I	I	-	-	Gambia Bureau of Statistics (GBOS), ICF International, Ministry of Health and Social Welfare (The Gambia). Gambia Demographic and Health Survey 2013. Fairfax, United States of America: ICF International, 2015.	GHDx
424884	The Gambia	Gambia Multiple Indicator Cluster Survey 2018	2018	I	I	I	I	I	I	I	I	I	I	I	Gambia Bureau of Statistics (GBOS), United Nations Children's Fund (UNICEF). Gambia Multiple Indicator Cluster Survey 2018. New York, United States of America: United Nations Children's Fund (UNICEF), 2019.	GHDx
3935	The Gambia	Gambia Multiple Indicator Cluster Survey 2005-2006	2005-2006	I	I	I	-	-	I	-	-	I	-	-	Gambia Bureau of Statistics (GBOS), United Nations Children's Fund (UNICEF). Gambia Multiple Indicator Cluster Survey 2005-2006. New York, United States of America: United Nations Children's Fund (UNICEF).	GHDx

GHDx ID	Country	Series	Year	BCG	DTP1	DTP3	HepB3	Hib3	MCV1	MCV2	PCV3	Pol3	RCV1	RotaC	Citation	Link
459854*	The Gambia	Gambia Demographic and Health Survey 2019-2020	2019-2020	I	I	I	I	I	I	I	I	I	-	I	Gambia Bureau of Statistics (GBOS), ICF International, Ministry of Health and Social Welfare (The Gambia). Gambia Demographic and Health Survey 2019-2020. Fairfax, United States of America: ICF International, 2021.	GHDx
12865	Timor-Leste	Timor-Leste Multiple Indicator Cluster Survey 2002	2002	I	-	I	-	-	I	-	-	I	-	-	Insan Hitawasana Sejahtera, National Statistics Directorate (Timor-Leste), United Nations Children's Fund (UNICEF). Timor-Leste Multiple Indicator Cluster Survey 2002. Dili, Timor-Leste: National Statistics Directorate (Timor-Leste).	GHDx
20888	Timor-Leste	Timor-Leste Demographic and Health Survey 2003	2003	I	I	I	E	-	I	-	-	I	-	-	ACIL Australia Pty Ltd., Australian National University, Ministry of Health (Timor-Leste), National Statistics Directorate (Timor-Leste), University of Newcastle (Australia). Timor-Leste Demographic and Health Survey 2003. Newcastle, Australia: University of Newcastle (Australia).	GHDx
286211	Timor-Leste	Timor-Leste Food and Nutrition Survey 2013	2013	I	I	I	-	-	I	-	-	I	-	-	Ministry of Health (Timor-Leste). Timor-Leste Food and Nutrition Survey 2013.	GHDx
286785	Timor-Leste	Timor-Leste Demographic and Health Survey 2016	2016	I	I	I	I	I	I	-	-	I	-	-	ICF International, National Statistics Directorate (Timor-Leste). Timor-Leste Demographic and Health Survey 2016. Fairfax, United States of America: ICF International, 2018.	GHDx
502498*	Timor-Leste	Timor-Leste Vaccination Cluster Coverage Survey 2018	2018	I	I	I	I	I	I	I	-	I	I	-	Timor-Leste Vaccination Cluster Coverage Survey 2018.	GHDx
558940*	Timor-Leste	Timor-Leste National Expanded Program on Immunization Coverage Evaluation Survey 2023	2023	I	I	I	I	I	I	-	-	I	-	I	Timor-Leste National Expanded Program on Immunization Coverage Evaluation Survey 2023.	GHDx
21274	Timor-Leste	Timor-Leste Demographic and Health Survey 2009-2010	2009-2010	I	I	I	E	-	I	-	-	I	-	-	ICF Macro, Ministry of Finance (Timor-Leste), National Statistics Directorate (Timor-Leste). Timor-Leste Demographic and Health Survey 2009-2010. Fairfax, United States of America: ICF International.	GHDx
229484	Togo	Togo National EPI Assessment 1988	1988	I	I	I	-	-	I	-	-	I	-	-	Togo National EPI Assessment 1988.	GHDx

GHDx ID	Country	Series	Year	BCG	DTP1	DTP3	HepB3	Hib3	MCV1	MCV2	PCV3	Pol3	RCV1	RotaC	Citation	Link
229463	Togo	Togo National EPI Cost Assessment of Fully Vaccinating Children through the Anti-Malaria Program 1990	1990	I	I	I	-	-	I	-	-	I	-	-	Togo National EPI Cost Assessment of Fully Vaccinating Children through the Anti-Malaria Program 1990.	GHDx
189153	Togo	Togo Multiple Indicator Cluster Survey 1996	1996	I	-	I	-	-	I	-	-	I	-	-	Demographic Research Unit, University of Benin (Togo), Department of Statistics (Togo), Ministry of Interior and Security (Togo), Ministry of Planning and Territorial Development (Togo), United Nations Children's Fund (UNICEF). Togo Multiple Indicator Cluster Survey 1996. New York, United States of America: United Nations Children's Fund (UNICEF).	GHDx
20909	Togo	Togo Demographic and Health Survey 1998	1998	I	I	I	-	-	I	-	-	I	-	-	Department of Statistics (Togo), Macro International, Inc. Togo Demographic and Health Survey 1998. Fairfax, United States of America: ICF International.	GHDx
12886	Togo	Togo Multiple Indicator Cluster Survey 2000	2000	I	I	I	-	-	I	-	-	I	-	-	United Nations Children's Fund (UNICEF). Togo Multiple Indicator Cluster Survey 2000. New York, United States of America: United Nations Children's Fund (UNICEF).	GHDx
229459	Togo	Togo External EPI Review 2001	2001	I	I	I	-	-	I	-	-	I	-	-	Global Alliance for Vaccines and Immunization (GAVI). Togo External EPI Review 2001.	GHDx
12896	Togo	Togo Multiple Indicator Cluster Survey 2006	2006	I	I	I	-	-	I	-	-	I	-	-	Directorate General of Statistics and National Accounting (Togo), United Nations Children's Fund (UNICEF). Togo Multiple Indicator Cluster Survey 2006. New York, United States: United Nations Children's Fund (UNICEF).	GHDx
40021	Togo	Togo Multiple Indicator Cluster Survey 2010	2010	I	I	I	E	E	I	-	-	I	-	-	Directorate General of Statistics and National Accounting (Togo), United Nations Children's Fund (UNICEF). Togo Multiple Indicator Cluster Survey 2010. New York, United States: United Nations Children's Fund (UNICEF).	GHDx
393869*	Togo	Incomplete immunization among children aged 12-23Â months in Togo: a multilevel analysis of individual and contextual factors	2017	I	I	I	I	I	I	-	I	I	-	I	Ekouevi DK, Gbeasor-Komlanvi FA, Yaya I, Zida-Compaore WI, Boko A, Sewu E, Lacle A, Ndibu N, Toke Y, Landoh DE. Incomplete immunization among children aged 12-23Ã‚Â months in Togo: a multilevel analysis of individual and contextual factors. BMC Public Health. 2018; 18(1): 952.	GHDx

GHDx ID	Country	Series	Year	BCG	DTP1	DTP3	HepB3	Hib3	MCV1	MCV2	PCV3	Pol3	RCV1	RotaC	Citation	Link
429991	Togo	Togo Multiple Indicator Cluster Survey 2017	2017	I	I	I	I	I	I	-	I	I	-	I	Directorate General of Statistics and National Accounting (Togo), United Nations Children's Fund (UNICEF). Togo Multiple Indicator Cluster Survey 2010. New York, United States: United Nations Children's Fund (UNICEF).	GHDx
229452	Togo	Togo EPI Review 2012-2013	2012-2013	I	I	I	I	I	I	-	-	I	-	-	GAVI Alliance. Togo EPI Review 2012-2013.	GHDx
77515	Togo	Togo Demographic and Health Survey 2013-2014	2013-2014	I	I	I	-	-	I	-	-	I	-	-	Directorate General of Statistics and National Accounts (Togo), ICF International, Ministry of Health (Togo), Ministry of Planning, Development and Zoning (Togo). Togo Demographic and Health Survey 2013-2014. Fairfax, United States of America: ICF International, 2015.	GHDx
228787	Tonga	Tonga Evaluation of Immunization Program 2003	2003	I	-	I	I	-	I	-	-	I	-	-	Tonga Evaluation of Immunization Program 2003.	GHDx
12950	Trinidad and Tobago	Trinidad and Tobago Multiple Indicator Cluster Survey 2006	2006	-	I	I	E	E	I	-	-	I	I	-	Central Statistical Office (Trinidad and Tobago) and United Nations Children's Fund (UNICEF). Trinidad and Tobago Multiple Indicator Cluster Survey 2006. New York, United States: United Nations Children's Fund (UNICEF).	GHDx
332558	Trinidad and Tobago	Trinidad and Tobago Multiple Indicator Cluster Survey 2011	2011	-	I	I	I	I	I	-	E	e	-	-	Central Statistical Office (Trinidad and Tobago), Ministry of Social Development and Family Services (Trinidad and Tobago), United Nations Children's Fund (UNICEF). Trinidad and Tobago Multiple Indicator Cluster Survey 2011. New York, United States of America: United Nations Children's Fund (UNICEF), 2018.	GHDx
543576*	Trinidad and Tobago	Trinidad and Tobago Multiple Indicator Cluster Survey 2022	2022	-	I	I	I	I	I	I	I	I	I	-	Central Statistical Office (Trinidad and Tobago), United Nations Children's Fund (UNICEF). Trinidad and Tobago Multiple Indicator Cluster Survey 2022.	GHDx
228740	Tunisia	Tunisia National Immunization Coverage Survey 1990	1990	-	-	-	-	-	I	-	-	-	-	-	Tunisia National Immunization Coverage Survey 1990.	GHDx
12983	Tunisia	Tunisia Multiple Indicator Cluster Survey 2000	2000	I	-	I	I	E	e	-	-	I	-	-	Ministry of Public Health (Tunisia), United Nations Children's Fund (UNICEF). Tunisia Multiple Indicator Cluster Survey 2000.	GHDx

GHDx ID	Country	Series	Year	BCG	DTP1	DTP3	HepB3	Hib3	MCV1	MCV2	PCV3	Pol3	RCV1	RotaC	Citation	Link
12985	Tunisia	Tunisia Multiple Indicator Cluster Survey 2006	2006	I	-	I	-	-	I	-	-	-	-	-	Ministry of Public Health (Tunisia), National Office for Family and Population, Ministry of Public Health (Tunisia), United Nations Children's Fund (UNICEF). Tunisia Multiple Indicator Cluster Survey 2006.	GHDx
514117*	Tunisia	Tunisia Health Examination Survey 2016	2016	I	-	-	-	-	I	-	-	-	-	-	National Institute of Public Health, Ministry of Public Health (Tunisia). Tunisia Health Examination Survey 2016.	GHDx
546694*	Tunisia	Tunisia Multiple Indicator Cluster Survey 2023	2023	I	I	I	I	I	I	E	-	e	I	-	National Institute of Statistics (Tunisia), United Nations Children's Fund (UNICEF). Tunisia Multiple Indicator Cluster Survey 2023. New York, United States of America: United Nations Children's Fund (UNICEF), 2023.	GHDx
12970	Tunisia	Tunisia Maternal and Child Health Survey 1994-1995	1994-1995	I	I	I	-	-	I	-	-	I	-	-	Ministry of Public Health (Tunisia), National Office for Family and Population (Tunisia), League of Arab States. Tunisia Maternal and Child Health Survey 1994-1995.	GHDx
76709	Tunisia	Tunisia Multiple Indicator Cluster Survey 2011-2012	2011-2012	I	I	I	I	-	I	-	-	I	-	-	Ministry of Regional Development and Planning (Tunisia), National Institute of Statistics (Tunisia), United Nations Children's Fund (UNICEF). Tunisia Multiple Indicator Cluster Survey 2011-2012. New York, United States of America: United Nations Children's Fund (UNICEF), 2014.	GHDx
228736	Türkiye	Turkey National EPI Program Review 1988	1988	I	I	I	-	-	I	-	-	I	-	-	Turkey National EPI Program Review 1988.	GHDx
228654	Türkiye	Turkey Immunisation Coverage Survey 1989	1989	I	I	I	-	-	I	-	-	I	-	-	Turkey Immunisation Coverage Survey 1989.	GHDx
142116	Türkiye	Turkey Multiple Indicator Cluster Survey 1995	1995	I	I	I	-	-	I	-	-	I	-	-	State Institute of Statistics (Turkey), United Nations Children's Fund (UNICEF). Turkey Multiple Indicator Cluster Survey 1995. New York, United States of America: United Nations Children's Fund (UNICEF).	GHDx
20947	Türkiye	Turkey Demographic and Health Survey 1998	1998	I	I	I	E	-	I	-	-	I	-	-	Institute of Population Studies, Hacettepe University, Macro International, Inc. Turkey Demographic and Health Survey 1998. Fairfax, United States of America: ICF International.	GHDx

GHDx ID	Country	Series	Year	BCG	DTP1	DTP3	HepB3	Hib3	MCV1	MCV2	PCV3	Pol3	RCV1	RotaC	Citation	Link
32421	Türkiye	Turkey Demographic and Health Survey 2008	2008	I	I	I	I	-	I	-	-	I	-	-	Institute of Population Studies, Hacettepe University, Ministry of Health (Turkey), State Planning Organization (Turkey), Turkish Statistical Institute. Turkey Demographic and Health Survey 2008. Fairfax, United States of America: ICF International, 2019.	GHDx
20954	Türkiye	Turkey Demographic and Health Survey 2003-2004	2003-2004	I	I	I	I	-	I	-	-	I	-	-	Institute of Population Studies, Hacettepe University, Ministry of Health (Turkey). Turkey Demographic and Health Survey 2003-2004. Fairfax, United States of America: ICF International.	GHDx
223669	Türkiye	Turkey Demographic and Health Survey 2013-2014	2013-2014	I	I	I	E	E	I	-	E	I	I	-	Institute of Population Studies, Hacettepe University, Ministry of Development (Turkey), Ministry of Health (Turkey). Turkey Demographic and Health Survey 2013-2014. Fairfax, United States of America: ICF International, 2019.	GHDx
440151*	Türkiye	Turkey Demographic and Health Survey 2018-2019	2018-2019	I	e	e	E	E	I	-	E	I	I	-	Institute of Population Studies, Hacettepe University, Ministry of Development (Turkey), Ministry of Health (Turkey). Turkey Demographic and Health Survey 2018-2019.	GHDx
90713	Turkmenistan	Turkmenistan Multiple Indicator Cluster Survey 1995	1995	I	-	I	-	-	I	-	-	I	-	-	Ministry of Education (Turkmenistan), Ministry of Health and Medical Industry (Turkmenistan), State Committee on Statistics of Turkmenistan, United Nations Children's Fund (UNICEF). Turkmenistan Multiple Indicator Cluster Survey 1995.	GHDx
20956	Turkmenistan	Turkmenistan Demographic and Health Survey 2000	2000	I	I	I	-	-	I	-	-	I	-	-	Gurbansoltan Eje Clinical Research Center for Maternal and Child Health (GECRCMCH), Macro International, Inc, Ministry of Health and Medical Industry (Turkmenistan). Turkmenistan Demographic and Health Survey 2000.	GHDx
13064	Turkmenistan	Turkmenistan Multiple Indicator Cluster Survey 2006	2006	I	I	I	I	-	I	-	-	I	-	-	Ministry of Foreign Affairs (Turkmenistan), Ministry of Health and Medical Industry (Turkmenistan), National Institute of State Statistics and Information (Turkmenistan), United Nations Children's Fund (UNICEF). Turkmenistan Multiple Indicator Cluster Survey 2006. New York, United States of America: United Nations Children's Fund (UNICEF), 2016.	GHDx

GHDx ID	Country	Series	Year	BCG	DTP1	DTP3	HepB3	Hib3	MCV1	MCV2	PCV3	Pol3	RCV1	RotaC	Citation	Link
264583	Turkmenistan	Turkmenistan Multiple Indicator Cluster Survey 2015-2016	2015-2016	I	I	I	I	I	I	-	-	I	I	-	State Committee on Statistics of Turkmenistan, United Nations Children's Fund (UNICEF). Turkmenistan Multiple Indicator Cluster Survey 2015-2016. New York, United States of America: United Nations Children's Fund (UNICEF), 2017.	GHDx
489863*	Tuvalu	Tuvalu Multiple Indicator Cluster Survey 2019-2020	2019-2020	I	I	I	I	I	I	I	-	I	I	-	Tuvalu Central Statistics Division, United Nations Children's Fund (UNICEF). Tuvalu Multiple Indicator Cluster Survey 2019-2020. New York, United States of America: United Nations Children's Fund (UNICEF), 2021.	GHDx
20976	Uganda	Uganda Demographic and Health Survey 1995	1995	I	I	I	-	-	I	-	-	I	-	-	Macro International, Inc, Statistics Department (Uganda). Uganda Demographic and Health Survey 1995. Fairfax, United States of America: ICF International.	GHDx
50471	Uganda	Uganda Delivery of Improved Services for Health, Facility and Household Evaluation Surveys 1999	1999	I	-	I	-	-	I	-	-	I	-	-	MEASURE Evaluation Project, Carolina Population Center, University of North Carolina, Pathfinder International, Macro International, Inc., Ministry of Health (Uganda), Center for Communication Programs, Bloomberg School of Public Health, Johns Hopkins, Program for International Training in Health, University of North Carolina (INTRAH), Management Sciences for Health (MSH). Uganda Delivery of Improved Services for Health, Facility and Household Evaluation Surveys 1999. Chapel Hill, United States: MEASURE Evaluation Project, Carolina Population Center, University of North Carolina.	GHDx
281653	Uganda	Uganda Lot Quality Assurance Sampling Survey 2004	2004	-	I	I	-	-	-	-	-	-	-	-	John Snow, Inc., Uganda Program for Human and Holistic Development (UPHOLD). Uganda Lot Quality Assurance Sampling Survey 2004.	GHDx
228440	Uganda	Uganda EPI Plus Coverage Survey 2005	2005	I	I	I	I	I	I	-	-	I	-	-	Ministry of Health (Uganda). Uganda EPI Plus Coverage Survey 2005.	GHDx
21014	Uganda	Uganda Demographic and Health Survey 2006	2006	I	I	I	I	I	I	-	-	I	-	-	Macro International, Inc, Uganda Bureau of Statistics. Uganda Demographic and Health Survey 2006. Fairfax, United States of America: ICF International.	GHDx

GHDx ID	Country	Series	Year	BCG	DTP1	DTP3	HepB3	Hib3	MCV1	MCV2	PCV3	Pol3	RCV1	RotaC	Citation	Link
23289	Uganda	Uganda Child Verbal Autopsy Study 2007	2007	e	e	e	E	E	e	-	-	e	-	-	MEASURE Evaluation Project, Carolina Population Center, University of North Carolina, Macro International, Inc, Ministry of Health (Uganda), Uganda Bureau of Statistics. Uganda Child Verbal Autopsy Study 2007. Calverton, United States of America: Macro International, Inc.	GHDx
93320	Uganda	Uganda National Service Delivery Survey 2008	2008	I	I	I	I	I	I	-	-	I	-	-	Ministry of Public Service (Uganda), Uganda Bureau of Statistics. Uganda National Service Delivery Survey 2008.	GHDx
56021	Uganda	Uganda Demographic and Health Survey 2011	2011	I	I	I	I	I	I	-	-	I	-	-	ICF Macro, Uganda Bureau of Statistics. Uganda Demographic and Health Survey 2011. Fairfax, United States of America: ICF International.	GHDx
228366	Uganda	Uganda Routine Immunization Coverage Survey 2012	2012	I	I	I	-	-	I	-	-	-	-	-	Uganda Routine Immunization Coverage Survey 2012.	GHDx
286780	Uganda	Uganda Demographic and Health Survey 2016	2016	I	I	I	I	I	I	-	I	I	-	-	ICF International, Uganda Bureau of Statistics. Uganda Demographic and Health Survey 2016. Fairfax, United States of America: ICF International, 2018.	GHDx
502499*	Uganda	Uganda National Immunization Coverage Survey 2016	2016	I	I	I	I	I	I	-	I	I	-	-	Uganda National Immunization Coverage Survey 2016.	GHDx
557734*	Uganda	Uganda Demographic and Health Survey 2022	2022	I	I	I	I	I	I	-	-	-	-	I	ICF International, Uganda Bureau of Statistics. Uganda Demographic and Health Survey 2022. Fairfax, United States of America: ICF International.	GHDx
20964	Uganda	Uganda Demographic and Health Survey 1988-1989	1988-1989	I	I	I	-	-	I	-	-	I	-	-	Macro Systems, Inc.; Institute for Resource Development, Makerere University, Ministry of Health (Uganda). Uganda Demographic and Health Survey 1988-1989. Fairfax, United States of America: ICF International.	GHDx
228482	Uganda	Uganda Immunization Coverage Validation Survey 1998-1999	1998-1999	I	I	I	-	-	I	-	-	I	-	-	World Health Organization (WHO). Uganda Immunization Coverage Validation Survey 1998-1999.	GHDx
20993	Uganda	Uganda Demographic and Health Survey 2000-2001	2000-2001	I	I	I	-	-	I	-	-	I	-	-	Macro International, Inc, Uganda Bureau of Statistics. Uganda Demographic and Health Survey 2000-2001. Fairfax, United States of America: ICF International.	GHDx

GHDx ID	Country	Series	Year	BCG	DTP1	DTP3	HepB3	Hib3	MCV1	MCV2	PCV3	Pol3	RCV1	RotaC	Citation	Link
13084	Uganda	Uganda AIDS Indicator Survey 2004-2005	2004-2005	-	-	-	E	-	-	-	-	-	-	-	Division of Reproductive Health, Centers for Disease Control and Prevention (CDC), Ministry of Health (Uganda). Uganda AIDS Indicator Survey 2004-2005.	GHDx
81004	Uganda	Uganda Living Standards Measurement Survey - Integrated Survey on Agriculture 2009-2010	2009-2010	-	e	-	-	-	e	-	-	-	-	-	Uganda Bureau of Statistics. Uganda Living Standards Measurement Survey - Integrated Survey on Agriculture 2009-2010. Washington DC, United States of America: World Bank.	GHDx
142934	Uganda	Uganda National Panel Survey 2010-2011	2010-2011	-	I	-	-	-	I	-	-	-	-	-	Uganda Bureau of Statistics. Uganda National Panel Survey 2010-2011.Washington DC, United States of America: World Bank, 2022.	GHDx
142935	Uganda	Uganda National Panel Survey Wave III 2011-2012	2011-2012	-	I	-	-	-	I	-	-	-	-	-	The World Bank Living Standards Measurement Study - Integrated Surveys on Agriculture, Uganda Bureau of Statistics. Uganda National Panel Survey Wave III 2011-2012. Washington DC, United States of America: World Bank, 2014.	GHDx
264959	Uganda	Uganda National Panel Survey 2013-2014	2013-2014	-	e	-	-	-	e	-	-	-	-	-	Government of the Netherlands, Uganda Bureau of Statistics, World Bank. Uganda National Panel Survey 2013-2014. Washington DC, United States of America: World Bank, 2022.	GHDx
342461	Uganda	Uganda National Service Delivery Survey 2015	2015-2016	I	I	I	-	-	I	-	-	I	-	-	Ministry of Public Service (Uganda), Uganda Bureau of Statistics. Uganda National Service Delivery Survey 2015.	GHDx
132739	Ukraine	Ukraine Multiple Indicator Cluster Survey 2012	2012	I	I	I	-	I	I	-	-	I	I	-	StatInform Consulting, State Statistics Service (Ukraine), Ukrainian Center for Social Reforms (UCSR), United Nations Children's Fund (UNICEF). Ukraine Multiple Indicator Cluster Survey 2012. New York, United States of America: United Nations Children's Fund (UNICEF), 2014.	GHDx
215001	United Arab Emirates	United Arab Emirates Review of the EPI 1986	1986	I	I	I	-	-	I	-	-	I	-	-	United Arab Emirates Review of the EPI 1986.	GHDx
13224	United Arab Emirates	United Arab Emirates Child Health Survey 1987	1987	e	e	e	-	-	e	-	-	e	-	-	Ministry of Health and Prevention (United Arab Emirates). United Arab Emirates Child Health Survey 1987.	GHDx
13226	United Arab Emirates	United Arab Emirates Family Health Survey 1995	1995	I	I	I	-	-	I	-	-	I	-	-	Health Ministers' Council for GCC States, Ministry of Health and Prevention (United Arab Emirates). United Arab Emirates Family Health Survey 1995.	GHDx

GHDx ID	Country	Series	Year	BCG	DTP1	DTP3	HepB3	Hib3	MCV1	MCV2	PCV3	Pol3	RCV1	RotaC	Citation	Link
215017	United Arab Emirates	United Arab Emirates Survey of Immunization Coverage and the EPI Information System 1990-1991	1990-1991	-	-	-	-	-	I	-	-	-	-	-	United Arab Emirates Survey of Immunization Coverage and the EPI Information System 1990-1991.	GHDx
467505*	United Arab Emirates	United Arab Emirates World Health Survey 2017-2018	2017-2018	I	I	I	-	-	I	E	I	e	I	E	Abu Dhabi Department of Health (United Arab Emirates), Dubai Health Authority, Dubai Statistics Center (United Arab Emirates), Federal Competitiveness and Statistics Authority (United Arab Emirates), IQVIA, Ministry of Health and Prevention (United Arab Emirates), Statistics Centre Abu Dhabi. United Arab Emirates World Health Survey 2017-2018.	GHDx
429879*	USA	United States National Immunization Survey-Child 2019	2019	-	I	I	I	I	I	-	I	I	I	I	Centers for Disease Control and Prevention (CDC). United States National Immunization Survey-Child 2019. Atlanta, United States of America: Centers for Disease Control and Prevention (CDC), 2019.	GHDx
451043*	USA	United States National Immunization Survey-Child 2020	2020	-	I	I	I	I	I	-	I	I	I	I	Centers for Disease Control and Prevention (CDC). United States National Immunization Survey-Child 2020. Atlanta, United States of America: Centers for Disease Control and Prevention (CDC), 2020.	GHDx
476531*	USA	United States National Immunization Survey-Child 2021	2021	-	I	I	I	I	I	-	I	I	I	I	Centers for Disease Control and Prevention (CDC). United States National Immunization Survey-Child 2021.	GHDx
510724*	USA	United States National Immunization Survey-Child 2022	2022	-	I	I	I	I	I	-	I	I	-	I	Centers for Disease Control and Prevention (CDC). United States National Immunization Survey-Child 2022. Atlanta, United States of America: Centers for Disease Control and Prevention (CDC), 2023.	GHDx
84808	USA	United States National Immunization Survey-Child 1995-1996	1995-1996	-	I	I	I	I	I	-	-	I	I	-	Abt Associates Inc., National Center for Health Statistics, Centers for Disease Control and Prevention, National Center for Immunization and Respiratory Diseases, CDC (NCIRD). United States National Immunization Survey-Child 1995-1996. Hyattsville, United States of America: National Center for Health Statistics, Centers for Disease Control and Prevention.	GHDx

GHDx ID	Country	Series	Year	BCG	DTP1	DTP3	HepB3	Hib3	MCV1	MCV2	PCV3	Pol3	RCV1	RotaC	Citation	Link
84799	USA	United States National Immunization Survey-Child 1996-1997	1996-1997	-	I	I	I	I	I	-	-	I	I	-	Abt Associates Inc., National Center for Health Statistics, Centers for Disease Control and Prevention, National Center for Immunization and Respiratory Diseases, CDC (NCIRD). United States National Immunization Survey-Child 1996-1997. Hyattsville, United States of America: National Center for Health Statistics, Centers for Disease Control and Prevention.	GHDx
84789	USA	United States National Immunization Survey-Child 1996-1998	1996-1998	-	I	I	I	I	I	-	-	I	I	-	Abt Associates Inc., National Center for Health Statistics, Centers for Disease Control and Prevention, National Center for Immunization and Respiratory Diseases, CDC (NCIRD). United States National Immunization Survey-Child 1996-1998. Hyattsville, United States of America: National Center for Health Statistics, Centers for Disease Control and Prevention.	GHDx
84780	USA	United States National Immunization Survey-Child 1998-1999	1998-1999	-	I	I	I	I	I	-	-	I	I	-	Abt Associates Inc., National Center for Health Statistics, Centers for Disease Control and Prevention, National Center for Immunization and Respiratory Diseases, CDC (NCIRD). United States National Immunization Survey-Child 1998-1999. Hyattsville, United States of America: National Center for Health Statistics, Centers for Disease Control and Prevention.	GHDx
84771	USA	United States National Immunization Survey-Child 1999-2000	1999-2000	-	I	I	I	I	I	-	-	I	I	-	Abt Associates Inc., National Center for Health Statistics, Centers for Disease Control and Prevention, National Center for Immunization and Respiratory Diseases, CDC (NCIRD). United States National Immunization Survey-Child 1999-2000. Hyattsville, United States of America: National Center for Health Statistics, Centers for Disease Control and Prevention.	GHDx

GHDx ID	Country	Series	Year	BCG	DTP1	DTP3	HepB3	Hib3	MCV1	MCV2	PCV3	Pol3	RCV1	RotaC	Citation	Link
84761	USA	United States National Immunization Survey-Child 2000-2001	2000-2001	-	I	I	I	I	I	-	-	I	I	-	Abt Associates Inc., National Center for Health Statistics, Centers for Disease Control and Prevention, National Center for Immunization and Respiratory Diseases, CDC (NCIRD). United States National Immunization Survey-Child 2000-2001. Hyattsville, United States of America: National Center for Health Statistics, Centers for Disease Control and Prevention.	GHDx
84752	USA	United States National Immunization Survey-Child 2001-2002	2001-2002	-	I	I	I	I	I	-	E	I	I	-	Abt Associates Inc., National Center for Health Statistics, Centers for Disease Control and Prevention, National Center for Immunization and Respiratory Diseases, CDC (NCIRD). United States National Immunization Survey-Child 2001-2002. Hyattsville, United States of America: National Center for Health Statistics, Centers for Disease Control and Prevention.	GHDx
84743	USA	United States National Immunization Survey-Child 2002-2003	2002-2003	-	I	I	I	I	I	-	I	I	I	-	National Center for Health Statistics, Centers for Disease Control and Prevention, National Center for Immunization and Respiratory Diseases, CDC (NCIRD), National Opinion Research Center (NORC) (United States). United States National Immunization Survey-Child 2002-2003. Hyattsville, United States of America: National Center for Health Statistics, Centers for Disease Control and Prevention.	GHDx
84734	USA	United States National Immunization Survey-Child 2003-2004	2003-2004	-	I	I	I	I	I	-	I	I	I	-	Abt Associates Inc., National Center for Health Statistics, Centers for Disease Control and Prevention, National Center for Immunization and Respiratory Diseases, CDC (NCIRD). United States National Immunization Survey-Child 2003-2004. Hyattsville, United States of America: National Center for Health Statistics, Centers for Disease Control and Prevention.	GHDx

GHDx ID	Country	Series	Year	BCG	DTP1	DTP3	HepB3	Hib3	MCV1	MCV2	PCV3	Pol3	RCV1	RotaC	Citation	Link
84725	USA	United States National Immunization Survey-Child 2004-2005	2004-2005	-	I	I	I	I	I	-	I	I	I	-	Abt Associates Inc., National Center for Health Statistics, Centers for Disease Control and Prevention, National Center for Immunization and Respiratory Diseases, CDC (NCIRD). United States National Immunization Survey-Child 2004-2005. Hyattsville, United States of America: National Center for Health Statistics, Centers for Disease Control and Prevention.	GHDx
84716	USA	United States National Immunization Survey-Child 2005-2006	2005-2006	-	I	I	I	I	I	-	I	I	I	-	National Center for Health Statistics, Centers for Disease Control and Prevention, National Center for Immunization and Respiratory Diseases, CDC (NCIRD), National Opinion Research Center (NORC) (United States). United States National Immunization Survey-Child 2005-2006. Hyattsville, United States of America: National Center for Health Statistics, Centers for Disease Control and Prevention.	GHDx
84707	USA	United States National Immunization Survey-Child 2006-2007	2006-2007	-	I	I	I	I	I	-	I	I	I	E	National Center for Health Statistics, Centers for Disease Control and Prevention, National Center for Immunization and Respiratory Diseases, CDC (NCIRD), National Opinion Research Center (NORC) (United States). United States National Immunization Survey-Child 2006-2007. Hyattsville, United States of America: National Center for Health Statistics, Centers for Disease Control and Prevention.	GHDx
84694	USA	United States National Immunization Survey-Child 2007-2008	2007-2008	-	I	I	I	I	I	-	I	I	I	E	National Center for Health Statistics, Centers for Disease Control and Prevention, National Center for Immunization and Respiratory Diseases, CDC (NCIRD), National Opinion Research Center (NORC) (United States). United States National Immunization Survey-Child 2007-2008. Hyattsville, United States of America: National Center for Health Statistics, Centers for Disease Control and Prevention.	GHDx

GHDx ID	Country	Series	Year	BCG	DTP1	DTP3	HepB3	Hib3	MCV1	MCV2	PCV3	Pol3	RCV1	RotaC	Citation	Link
13389	USA	United States National Immunization Survey-Child 2008-2009	2008-2009	-	I	I	I	I	I	-	I	I	I	I	National Center for Health Statistics, Centers for Disease Control and Prevention, National Center for Immunization and Respiratory Diseases, CDC (NCIRD), National Opinion Research Center (NORC) (United States). United States National Immunization Survey-Child 2008-2009. Hyattsville, United States of America: National Center for Health Statistics, Centers for Disease Control and Prevention.	GHDx
135819	USA	United States National Immunization Survey-Child 2010-2011	2010-2011	-	I	I	I	I	I	-	I	I	I	I	NORC at the University of Chicago, National Center for Health Statistics, Centers for Disease Control and Prevention, National Center for Immunization and Respiratory Diseases, CDC (NCIRD). United States National Immunization Survey-Child 2010-2011. Hyattsville, United States of America: National Center for Health Statistics, Centers for Disease Control and Prevention.	GHDx
135820	USA	United States National Immunization Survey-Child 2011-2012	2011-2012	-	I	I	I	I	I	-	I	I	I	I	NORC at the University of Chicago, National Center for Health Statistics, Centers for Disease Control and Prevention, National Center for Immunization and Respiratory Diseases, CDC (NCIRD). United States National Immunization Survey-Child 2011-2012. Hyattsville, United States of America: National Center for Health Statistics, Centers for Disease Control and Prevention.	GHDx
137220	USA	United States National Immunization Survey-Child 2012-2013	2012-2013	-	I	I	I	I	I	-	I	I	I	I	NORC at the University of Chicago, National Center for Health Statistics, Centers for Disease Control and Prevention, National Center for Immunization and Respiratory Diseases, CDC (NCIRD). United States National Immunization Survey-Child 2012-2013. Hyattsville, United States of America: National Center for Health Statistics, Centers for Disease Control and Prevention.	GHDx

GHDx ID	Country	Series	Year	BCG	DTP1	DTP3	HepB3	Hib3	MCV1	MCV2	PCV3	Pol3	RCV1	RotaC	Citation	Link
224280	USA	United States National Immunization Survey-Child 2013-2014	2013-2014	-	I	I	I	I	I	-	I	I	I	I	NORC at the University of Chicago, National Center for Health Statistics, Centers for Disease Control and Prevention, National Center for Immunization and Respiratory Diseases, CDC (NCIRD). United States National Immunization Survey-Child 2013-2014. Atlanta, United States of America: Centers for Disease Control and Prevention (CDC).	GHDx
224282	USA	United States National Immunization Survey-Child 2014-2015	2014-2015	-	I	I	I	I	I	-	I	I	I	I	NORC at the University of Chicago, National Center for Health Statistics, Centers for Disease Control and Prevention, National Center for Immunization and Respiratory Diseases, CDC (NCIRD). United States National Immunization Survey-Child 2014-2015. Atlanta, United States of America: Centers for Disease Control and Prevention (CDC), 2015.	GHDx
354855	USA	United States National Immunization Survey-Child 2015-2016	2015-2016	-	I	I	I	I	I	-	I	I	I	I	NORC at the University of Chicago, National Center for Health Statistics, Centers for Disease Control and Prevention, National Center for Immunization and Respiratory Diseases, CDC (NCIRD). United States National Immunization Survey-Child 2015-2016. Atlanta, United States of America: Centers for Disease Control and Prevention (CDC), 2016.	GHDx
355985	USA	United States National Immunization Survey-Child 2016-2017	2016-2017	-	I	I	I	I	I	-	I	I	I	I	NORC at the University of Chicago, National Center for Health Statistics, Centers for Disease Control and Prevention, National Center for Immunization and Respiratory Diseases, CDC (NCIRD). United States National Immunization Survey-Child 2016-2017. Atlanta, United States of America: Centers for Disease Control and Prevention (CDC), 2017.	GHDx
356126	USA	United States National Immunization Survey-Child 2017-2018	2017-2018	-	I	I	I	I	I	-	I	I	I	I	NORC at the University of Chicago, National Center for Health Statistics, Centers for Disease Control and Prevention, National Center for Immunization and Respiratory Diseases, CDC (NCIRD). United States National Immunization Survey-Child 2017-2018. Atlanta, United States of America: Centers for Disease Control and Prevention (CDC), 2018.	GHDx

GHDx ID	Country	Series	Year	BCG	DTP1	DTP3	HepB3	Hib3	MCV1	MCV2	PCV3	Pol3	RCV1	RotaC	Citation	Link
429869	USA	United States National Immunization Survey-Child 2018-2019	2018-2019	-	I	I	I	I	I	-	I	I	I	I	NORC at the University of Chicago, National Center for Immunization and Respiratory Diseases, CDC (NCIRD). United States National Immunization Survey-Child 2018-2019. Atlanta, United States of America: Centers for Disease Control and Prevention (CDC).	GHDx
21033	Uzbekistan	Uzbekistan Demographic and Health Survey 1996	1996	I	I	I	-	-	I	-	-	I	-	-	Institute of Obstetrics and Gynecology, Ministry of Health (Uzbekistan), Macro International, Inc, Ministry of Health (Uzbekistan). Uzbekistan Demographic and Health Survey 1996. Fairfax, United States of America: ICF International.	GHDx
13436	Uzbekistan	Uzbekistan Multiple Indicator Cluster Survey 2000	2000	I	I	I	-	-	I	-	-	I	-	-	United Nations Children's Fund (UNICEF), Ministry of Macroeconomics and Statistics (Uzbekistan). Uzbekistan Multiple Indicator Cluster Survey 2000. New York, United States: United Nations Children's Fund (UNICEF).	GHDx
13445	Uzbekistan	Uzbekistan Multiple Indicator Cluster Survey 2006	2006	I	I	I	I	-	I	-	-	I	-	-	United Nations Children's Fund (UNICEF), State Committee of the Republic of Uzbekistan on Statistics. Uzbekistan Multiple Indicator Cluster Survey 2006. New York, United States: United Nations Children's Fund (UNICEF).	GHDx
509345*	Uzbekistan	Uzbekistan Multiple Indicator Cluster Survey 2021-2022	2021-2022	I	I	I	I	I	I	-	I	I	I	I	State Committee of the Republic of Uzbekistan on Statistics, United Nations Children's Fund (UNICEF). Uzbekistan Multiple Indicator Cluster Survey 2021-2022. New York, United States of America: United Nations Children's Fund (UNICEF), 2022.	GHDx
229477	Vanuatu	Vanuatu Demographic and Health Survey 2013	2013	I	I	I	I	I	I	-	-	I	-	-	Ministry of Health (Vanuatu), Secretariat of the Pacific Community (SPC), Vanuatu National Statistics Office. Vanuatu Demographic and Health Survey 2013.	GHDx
453459	Vanuatu	Vanuatu Vaccination Coverage Survey 2016	2016	I	I	I	I	I	I	-	-	I	-	-	Ministry of Health (Vanuatu). Vanuatu Vaccination Coverage Survey 2016.	GHDx

GHDx ID	Country	Series	Year	BCG	DTP1	DTP3	HepB3	Hib3	MCV1	MCV2	PCV3	Pol3	RCV1	RotaC	Citation	Link
561483*	Vanuatu	Vanuatu Multiple Indicator Cluster Survey 2023	2023	I	I	I	-	-	I	E	I	I	I	I	Bureau of Statistics (Vanuatu), New Zealand Aid Programme, Secretariat of the Pacific Community (SPC), United Nations Children's Fund (UNICEF), United Nations Population Fund (UNFPA). Vanuatu Multiple Indicator Cluster Survey 2023. New York, United States of America: United Nations Children's Fund (UNICEF), 2024.	GHDx
13465	Vanuatu	Vanuatu Multiple Indicator Cluster Survey 2007-2008	2007-2008	I	I	I	I	-	I	-	-	I	E	-	Ministry of Health (Vanuatu), United Nations Children's Fund (UNICEF). Vanuatu Multiple Indicator Cluster Survey 2007-2008. New York, United States of America: United Nations Children's Fund (UNICEF).	GHDx
13516	Venezuela	Venezuela Multiple Indicator Cluster Survey 2000	2000	I	I	I	-	-	I	-	-	I	-	-	National Institute of Statistics (Venezuela), United Nations Children's Fund (UNICEF). Venezuela Multiple Indicator Cluster Survey 2000. New York, United States: United Nations Children's Fund (UNICEF).	GHDx
228334	Viet Nam	Vietnam EPI Review 1989	1989	I	I	I	-	-	I	-	-	I	-	-	Vietnam EPI Review 1989.	GHDx
189110	Viet Nam	Vietnam Multiple Indicator Cluster Survey 1996	1996	I	I	I	-	-	I	-	-	I	-	-	General Statistics Office (Vietnam), United Nations Children's Fund (UNICEF). Vietnam Multiple Indicator Cluster Survey 1996.	GHDx
21049	Viet Nam	Vietnam Demographic and Health Survey 1997	1997	I	I	I	-	-	I	-	-	I	-	-	Macro International, Inc, National Committee for Population and Family Planning (Vietnam). Vietnam Demographic and Health Survey 1997. Calverton, United States of America: Macro International, Inc.	GHDx
13708	Viet Nam	Vietnam Multiple Indicator Cluster Survey 2000	2000	I	I	I	-	-	I	-	-	I	-	-	General Statistics Office (Vietnam), United Nations Children's Fund (UNICEF). Vietnam Multiple Indicator Cluster Survey 2000. New York, United States of America: United Nations Children's Fund (UNICEF).	GHDx
21058	Viet Nam	Vietnam Demographic and Health Survey 2002	2002	I	I	I	-	-	I	-	-	I	-	-	General Statistics Office (Vietnam), Macro International, Inc. Vietnam Demographic and Health Survey 2002. Fairfax, United States of America: ICF International.	GHDx

GHDx ID	Country	Series	Year	BCG	DTP1	DTP3	HepB3	Hib3	MCV1	MCV2	PCV3	Pol3	RCV1	RotaC	Citation	Link
13719	Viet Nam	Vietnam Multiple Indicator Cluster Survey 2006	2006	I	I	I	-	-	I	-	-	I	-	-	General Statistics Office (Vietnam), United Nations Children's Fund (UNICEF). Vietnam Multiple Indicator Cluster Survey 2006. New York, United States of America: United Nations Children's Fund (UNICEF).	GHDx
482844*	Viet Nam	Viet Nam Immunization Coverage Survey 2015	2015	I	I	I	I	I	I	I	-	I	-	-	Viet Nam Immunization Coverage Survey 2015.	GHDx
57999	Viet Nam	Vietnam Multiple Indicator Cluster Survey 2010-2011	2010-2011	I	I	I	I	-	I	-	-	I	-	-	General Statistics Office (Vietnam), United Nations Children's Fund (UNICEF). Vietnam Multiple Indicator Cluster Survey 2010-2011. New York, United States of America: United Nations Children's Fund (UNICEF).	GHDx
152735	Viet Nam	Vietnam Multiple Indicator Cluster Survey 2013-2014	2013-2014	I	I	I	I	I	I	-	-	I	-	-	General Statistics Office (Vietnam), United Nations Children's Fund (UNICEF). Vietnam Multiple Indicator Cluster Survey 2013-2014. New York, United States of America: United Nations Children's Fund (UNICEF), 2015.	GHDx
494214*	Viet Nam	Vietnam Multiple Indicator Cluster Survey 2020-2021	2020-2021	I	I	I	I	I	I	I	-	I	-	-	General Statistics Office (Vietnam), United Nations Children's Fund (UNICEF). Vietnam Multiple Indicator Cluster Survey 2020-2021. New York, United States of America: United Nations Children's Fund (UNICEF), 2022.	GHDx
228257	Yemen	Yemen Arab Republic Joint Government/WHO/Unicef EPI Coverage Review 1987	1987	I	I	I	-	-	I	-	-	I	-	-	Government of Yemen Arab Republic, World Health Organization (WHO). Yemen Arab Republic Joint Government/WHO/Unicef EPI Coverage Review 1987.	GHDx
142000	Yemen	Yemen Multiple Indicator Cluster Survey 1996	1996	I	I	-	-	-	I	-	-	-	-	-	Central Statistical Organization (Yemen), Ministry of Planning and Development (Yemen), United Nations Children's Fund (UNICEF). Yemen Multiple Indicator Cluster Survey 1996.	GHDx
21070	Yemen	Yemen Demographic and Health Survey 1997	1997	I	I	I	-	-	I	-	-	I	-	-	Central Statistical Organization (Yemen), Macro International, Inc. Yemen Demographic and Health Survey 1997.	GHDx
13795	Yemen	Yemen Family Health Survey 2003	2003	-	I	I	I	-	I	-	-	e	-	-	Central Statistical Organization (Yemen), League of Arab States, Ministry of Public Health and Population (Yemen), Pan Arab Project for Family Health (PAPFAM). Yemen Family Health Survey 2003.	GHDx

GHDx ID	Country	Series	Year	BCG	DTP1	DTP3	HepB3	Hib3	MCV1	MCV2	PCV3	Pol3	RCV1	RotaC	Citation	Link
13816	Yemen	Yemen Multiple Indicator Cluster Survey 2006	2006	I	I	I	I	E	I	-	-	I	-	-	Ministry of Health (Yemen) and United Nations Children's Fund (UNICEF). Yemen Multiple Indicator Cluster Survey 2006. New York, United States: United Nations Children's Fund (UNICEF).	GHDx
112500	Yemen	Yemen Demographic and Health Survey 2013	2013	I	I	I	I	I	I	-	E	I	-	-	Central Statistical Organization (Yemen), ICF International, Ministry of Public Health and Population (Yemen). Yemen Demographic and Health Survey 2013. Fairfax, United States of America: ICF International.	GHDx
22882	Yemen	Yemen Household Budget Survey 2005-2006	2005-2006	-	I	I	-	-	-	-	-	I	-	-	Central Statistical Organization (Yemen). Yemen Household Budget Survey 2005-2006. Sana'a, Yemen: Central Statistical Organization (Yemen).	GHDx
539851*	Yemen	Yemen Multiple Indicator Cluster Survey 2022-2023	2022-2023	I	I	I	I	I	I	I	I	I	I	I	Central Statistical Organization (Yemen), United Nations Children's Fund (UNICEF). Yemen Multiple Indicator Cluster Survey 2022-2023.	GHDx
228217	Zambia	Zambia Immunization Coverage Survey 1991	1991	I	I	I	-	-	I	-	-	I	-	-	Ministry of Health (Zambia), World Health Organization (WHO). Zambia Immunization Coverage Survey 1991.	GHDx
21079	Zambia	Zambia Demographic and Health Survey 1992	1992	I	I	I	-	-	I	-	-	I	-	-	Central Statistical Office (Zambia), Macro International, Inc, University of Zambia. Zambia Demographic and Health Survey 1992. Fairfax, United States of America: ICF International.	GHDx
14111	Zambia	Zambia Multiple Indicator Cluster Survey 1995	1995	I	-	I	-	-	I	-	-	I	-	-	Centers for Disease Control and Prevention (CDC), Central Statistical Office (Zambia), Food Security, Health and Nutrition Information System (Zambia), Food and Agriculture Organization of the United Nations (FAO), United Nations Children's Fund (UNICEF). Zambia Multiple Indicator Cluster Survey 1995.	GHDx
13973*	Zambia	Zambia Living Conditions Monitoring Survey 1996	1996	I	I	I	-	-	I	E	-	I	-	-	Central Statistical Office (Zambia). Zambia Living Conditions Monitoring Survey 1996. Lusaka, Zambia: Central Statistical Office (Zambia).	GHDx
14015	Zambia	Zambia Living Conditions Monitoring Survey 1998	1998	-	I	I	-	-	I	E	-	I	-	-	Central Statistical Office (Zambia), London School of Hygiene and Tropical Medicine. Zambia Living Conditions Monitoring Survey 1998. Lusaka, Zambia: Central Statistical Office (Zambia).	GHDx

GHDx ID	Country	Series	Year	BCG	DTP1	DTP3	HepB3	Hib3	MCV1	MCV2	PCV3	Pol3	RCV1	RotaC	Citation	Link
14122	Zambia	Zambia Multiple Indicator Cluster Survey 1999	1999	e	I	I	-	-	I	-	-	I	-	-	Central Statistical Office (Zambia), Food Security, Health and Nutrition Information System (Zambia), United Nations Children's Fund (UNICEF). Zambia Multiple Indicator Cluster Survey 1999. New York, United States of America: United Nations Children's Fund (UNICEF).	GHDx
228196	Zambia	Zambia EPI Cluster Survey Report 2001	2001	I	I	I	-	-	I	-	-	I	-	-	Ministry of Health (Zambia), World Health Organization (WHO). Zambia EPI Cluster Survey Report 2001.	GHDx
21117	Zambia	Zambia Demographic and Health Survey 2007	2007	I	I	I	E	E	I	-	-	I	-	-	Central Statistical Office (Zambia), Macro International, Inc. Zambia Demographic and Health Survey 2007. Fairfax, United States of America: ICF International.	GHDx
26702	Zambia	Zambia Global Fund Household Health Coverage Survey 2008	2008	I	e	e	E	-	I	-	-	I	-	-	Central Statistical Office (Zambia). Zambia Global Fund Household Health Coverage Survey 2008. Lusaka, Zambia: Central Statistical Office (Zambia).	GHDx
58660*	Zambia	Zambia Living Conditions Monitoring Survey 2010	2010	e	e	e	-	-	e	E	-	e	-	-	Central Statistical Office (Zambia). Zambia Living Conditions Monitoring Survey 2010.	GHDx
228195	Zambia	Zambia Expanded Program on Immunization Survey Using the Cluster Survey Methodology 2011	2011	I	I	I	I	I	I	-	-	I	-	-	Zambia Expanded Program on Immunization Survey using the cluster survey methodology 2011.	GHDx
284177	Zambia	Zambia Living Conditions Monitoring Survey 2015	2015	I	I	I	-	-	I	E	-	I	-	-	Central Statistical Office (Zambia), World Bank. Zambia Living Conditions Monitoring Survey 2015.	GHDx
559075*	Zambia	Zambia Post Measles Rubella Campaign Evaluation Survey 2021	2021	I	I	I	I	I	I	I	-	-	-	-	World Health Organization (WHO), Zambia Statistics Agency. Zambia Post Measles Rubella Campaign Evaluation Survey 2021. Geneva, Switzerland: World Health Organization (WHO).	GHDx
21090	Zambia	Zambia Demographic and Health Survey 1996-1997	1996-1997	I	I	I	-	-	I	-	-	I	-	-	Central Statistical Office (Zambia), Macro International, Inc, Ministry of Health (Zambia). Zambia Demographic and Health Survey 1996-1997. Fairfax, United States of America: ICF International.	GHDx
21102	Zambia	Zambia Demographic and Health Survey 2001-2002	2001-2002	I	I	I	-	-	I	-	-	I	-	-	Central Board of Health (Zambia), Central Statistical Office (Zambia), Macro International, Inc. Zambia Demographic and Health Survey 2001-2002. Fairfax, United States of America: ICF International.	GHDx

GHDx ID	Country	Series	Year	BCG	DTP1	DTP3	HepB3	Hib3	MCV1	MCV2	PCV3	Pol3	RCV1	RotaC	Citation	Link
14027	Zambia	Zambia Living Conditions Monitoring Survey 2002-2003	2002-2003	-	I	I	-	-	I	E	-	I	-	-	Central Statistical Office (Zambia). Zambia Living Conditions Monitoring Survey 2002-2003. Lusaka, Zambia: Central Statistical Office (Zambia).	GHDx
14063*	Zambia	Zambia Living Conditions Monitoring Survey 2004-2005	2004-2005	I	I	I	-	-	I	E	-	I	-	-	Central Statistical Office (Zambia). Zambia Living Conditions Monitoring Survey 2004-2005. Lusaka, Zambia: Central Statistical Office (Zambia).	GHDx
77516	Zambia	Zambia Demographic and Health Survey 2013-2014	2013-2014	I	I	I	I	I	I	-	-	I	-	-	Central Statistical Office (Zambia), ICF International, Ministry of Health (Zambia), Tropical Diseases Research Centre, University Teaching Hospital (Zambia), University of Zambia. Zambia Demographic and Health Survey 2013-2014. Fairfax, United States of America: ICF International.	GHDx
411301*	Zambia	Zambia Demographic and Health Survey 2018-2019	2018-2019	I	I	I	I	I	I	I	I	I	-	I	Central Statistical Office (Zambia), ICF International, Ministry of Health (Zambia), University Teaching Hospital (Zambia), University of Zambia. Zambia Demographic and Health Survey 2018-2019. Fairfax, United States of America: ICF International, 2020.	GHDx
21139	Zimbabwe	Zimbabwe Demographic and Health Survey 1994	1994	I	I	I	-	-	I	-	-	I	-	-	Central Statistical Office (Zimbabwe), Macro International, Inc. Zimbabwe Demographic and Health Survey 1994. Fairfax, United States of America: ICF International.	GHDx
141997	Zimbabwe	Zimbabwe National Maternal and Child Health Family Planning Survey 1997	1997	I	I	I	-	-	I	-	-	I	-	-	Zimbabwe National Maternal and Child Health Family Planning Survey 1997	GHDx
21151	Zimbabwe	Zimbabwe Demographic and Health Survey 1999	1999	I	I	I	-	-	I	-	-	I	-	-	Central Statistical Office (Zimbabwe), Macro International, Inc. Zimbabwe Demographic and Health Survey 1999. Fairfax, United States of America: ICF International.	GHDx
35493	Zimbabwe	Zimbabwe Multiple Indicator Monitoring Survey 2009	2009	I	I	I	I	-	I	-	-	I	-	-	Central Statistical Office (Zimbabwe). Zimbabwe Multiple Indicator Monitoring Survey 2009. New York, United States of America: United Nations Children's Fund (UNICEF).	GHDx
228136	Zimbabwe	Zimbabwe Routine Immunization Coverage Survey 2010	2010	I	I	I	-	-	I	-	-	I	-	-	Ministry of Health and Child Welfare (Zimbabwe). Zimbabwe Routine Immunization Coverage Survey 2010.	GHDx

GHDx ID	Country	Series	Year	BCG	DTP1	DTP3	HepB3	Hib3	MCV1	MCV2	PCV3	Pol3	RCV1	RotaC	Citation	Link
152720	Zimbabwe	Zimbabwe Multiple Indicator Cluster Survey 2014	2014	I	I	I	I	I	I	-	E	I	-	-	United Nations Children's Fund (UNICEF), Zimbabwe National Statistics Agency. Zimbabwe Multiple Indicator Cluster Survey 2014. New York, United States of America: United Nations Children's Fund (UNICEF), 2015.	GHDx
157066	Zimbabwe	Zimbabwe Demographic and Health Survey 2015	2015	I	I	I	I	I	I	-	I	I	-	E	ICF International, National Microbiology Reference Laboratory, Harare Central Hospital (NMRL) (Zimbabwe), Zimbabwe National Statistics Agency. Zimbabwe Demographic and Health Survey 2015. Fairfax, United States of America: ICF International, 2016.	GHDx
431951	Zimbabwe	Zimbabwe Multiple Indicator Cluster Survey 2019	2019	I	I	I	I	I	I	-	I	I	I	I	United Nations Children's Fund (UNICEF), Zimbabwe National Statistics Agency. Zimbabwe Multiple Indicator Cluster Survey 2019. New York, United States of America: United Nations Children's Fund (UNICEF), 2020.	GHDx
21126	Zimbabwe	Zimbabwe Demographic and Health Survey 1988-1989	1988-1989	I	I	I	-	-	I	-	-	I	-	-	Central Statistical Office (Zimbabwe), Macro Systems, Inc.; Institute for Resource Development. Zimbabwe Demographic and Health Survey 1988-1989. Fairfax, United States of America: ICF International.	GHDx
21163	Zimbabwe	Zimbabwe Demographic and Health Survey 2005-2006	2005-2006	I	I	I	I	-	I	E	-	I	-	-	Central Statistical Office (Zimbabwe), Macro International, Inc. Zimbabwe Demographic and Health Survey 2005-2006. Fairfax, United States of America: ICF International.	GHDx
55992	Zimbabwe	Zimbabwe Demographic and Health Survey 2010-2011	2010-2011	I	I	I	I	E	I	-	-	I	-	-	ICF Macro, Zimbabwe National Statistics Agency. Zimbabwe Demographic and Health Survey 2010-2011. Calverton, United States of America: ICF Macro, 2012.	GHDx

Table S3 Excluded sources

Excluded sources. Citations, series, country, years, and antigens are provided for each excluded dataset along with rationale for their exclusion. BCG=Bacillus Calmette-Guérin. DTP1=diphtheria-tetanus-pertussis, first dose. Sources marked with an asterisk (*) next to the GHDx ID are excluded from administrative data bias modeling as a methodological decision to improve model performance. DTP3=diphtheria-tetanus-pertussis, third dose. HepB3=hepatitis B vaccine, third dose. Hib3=Haemophilus influenzae type b vaccine, third dose. MCV1=measles-containing vaccine, first dose. MCV2=measles-containing vaccine, second dose. PCV3=pneumococcal conjugate vaccine, third dose. Pol3=polio vaccine, third dose. RCV1=rubella-containing vaccine, first dose. RotaC=completed rotavirus series.

GHDx ID	Country	Series	Year(s)	Cohort	Vaccine(s)	Citation	Rationale for exclusion
214992	Afghanistan	EPI Coverage Situation in Women and Children of Afghanistan, Report of Post NID's, Routine Coverage and Acceleration Campaign Survey in Afghanistan 1999	1999	all	BCG, DTP1, DTP3, MCV1, Pol3	EPI Coverage Situation in Women and Children of Afghanistan, Report of Post NIDs, Routine Coverage and Acceleration Campaign Survey in Afghanistan 1999.	Estimates considered implausible. Survey estimates are systematically high compared to administrative data and estimates from other established surveys series (Afghanistan Multiple Indicator Cluster Survey 2003). Survey removed from analysis.
561	Afghanistan	Afghanistan Multiple Indicator Cluster Survey 2003	2003	all	MCV1	Central Statistics Organization (Afghanistan), United Nations Children's Fund (UNICEF). Afghanistan Multiple Indicator Cluster Survey 2003.	MCV1 estimates are systematically high compared to administrative data and estimates from other established surveys series (Afghanistan Health Survey 2006). MCV1 removed from analysis.
18468	Afghanistan	Afghanistan Health Survey 2006	2006	all	BCG	Indian Institute of Health Management Research (IIHMR), Johns Hopkins University, Ministry of Public Health (Afghanistan). Afghanistan Health Survey 2006.	BCG estimates include implausibly high coverage rates of 100%. BCG removed from analysis.
157018	Afghanistan	Afghanistan Demographic and Health Survey 2015-2016	2015-2016	select	PCV3	Central Statistics Organization (Afghanistan), ICF International, Ministry of Public Health (Afghanistan). Afghanistan Demographic and Health Survey 2015-2016. Fairfax, United States of America: ICF International, 2017.	Older age-cohorts precede introduction of PCV3 to national immunization schedule. Select age-cohorts removed from analysis for PCV3.
527453	Afghanistan	Afghanistan Multiple Indicator Cluster Survey 2022-2023	2022-2023	select	BCG, DTP1, DTP3, HepB3, PCV3, MCV1, MCV2, PCV3, Pol3, RotaC	National Statistic and Information Authority (NSIA) (Afghanistan), United Nations Children's Fund (UNICEF). Afghanistan Multiple Indicator Cluster Survey 2022-2023. New York, United States of America: United Nations Children's Fund (UNICEF), 2023.	Estimates from survey microdata do not match coverage from survey report. Select age-cohorts removed from analysis.

GHDx ID	Country	Series	Year(s)	Cohort	Vaccine(s)	Citation	Rationale for exclusion
595	Albania	Albania Multiple Indicator Cluster Survey 2000	2000	all	BCG, DTP1, DTP3, MCV1, Pol3	National Institute of Statistics (Albania), United Nations Children's Fund (UNICEF). Albania Multiple Indicator Cluster Survey 2000. New York, United States: United Nations Children's Fund (UNICEF).	Estimates considered implausible. Survey estimates are systematically low compared to administrative data and estimates from other established surveys series. Survey removed from analysis.
608	Albania	Albania Multiple Indicator Cluster Survey 2005	2005	all	BCG, DTP1, DTP3, MCV1, Pol3	National Institute of Statistics (Albania), United Nations Children's Fund (UNICEF). Albania Multiple Indicator Cluster Survey 2005. New York, United States: United Nations Children's Fund (UNICEF).	Estimates considered implausible. Survey estimates are systematically low compared to administrative data and estimates from other established surveys series. Survey removed from analysis.
393876	Albania	Albania Demographic and Health Survey 2017-2018	2017-2018	all	BCG, DTP1, DTP3, HepB3, PCV3, MCV1, PCV3, Pol3	Albania Institute of Public Health (IPH), Albania Institute of Statistics (INSTAT), ICF International. Albania Demographic and Health Survey 2017-2018. Fairfax, United States of America: ICF International, 2019.	Survey data is based on health facility vaccination registries. Absence of maternal recall introduces potential bias; survey removed from analysis.
26449	Algeria	Algeria Multiple Indicator Cluster Survey 2000	2000	all	DTP1, DTP3	Ministry of Health and Population (Algeria), National Institute of Public Health (Algeria), National Office of Statistics (Algeria), United Nations Children's Fund (UNICEF). Algeria Multiple Indicator Cluster Survey 2000.	Survey reports higher DTP third dose coverage than first dose coverage. DTP3 removed from analysis due to reliability concerns.
464103	Algeria	Algeria Multiple Indicator Cluster Survey 2018-2019	2018-2019	all	DTP1, DTP3, HepB3, PCV3, MCV2, PCV3, Pol3	Ministry of Health and Population (Algeria), United Nations Children's Fund (UNICEF). Algeria Multiple Indicator Cluster Survey 2018-2019. New York, United States of America: United Nations Children's Fund (UNICEF), 2021.	Older age-cohorts precede introduction of these vaccines to national immunization schedule. Select age cohorts removed from analysis for these vaccines.
674*	Angola	Angola Multiple Indicator Cluster Survey 1996	1996	select	MCV1	Living Conditions Monitoring Office (Angola), National Statistics Office (Angola), United Nations Children's Fund (UNICEF). Angola Multiple Indicator Cluster Survey 1996. New York, United States of America: United Nations Children's Fund (UNICEF).	Selected cohorts excluded from administrative data bias modeling as a methodological decision to improve model performance

GHDx ID	Country	Series	Year(s)	Cohort	Vaccine(s)	Citation	Rationale for exclusion
687*	Angola	Angola Multiple Indicator Cluster Survey 2001	2001	select	Pol3	National Institute of Statistics (Angola), United Nations Children's Fund (UNICEF). Angola Multiple Indicator Cluster Survey 2001. New York, United States of America: United Nations Children's Fund (UNICEF).	Selected cohorts excluded from administrative data bias modeling as a methodological decision to improve model performance
151568	Angola	Angola Core Welfare Indicators Questionnaire Survey 2011	2011	all	BCG, DTP1, DTP3, MCV1, Pol3	Ministry of Planning and Territorial Development (Angola), National Institute of Statistics (Angola). Angola Core Welfare Indicators Questionnaire Survey 2011. Luanda, Angola: National Institute of Statistics (Angola).	Estimates considered implausible. Survey estimates are systematically low compared to administrative data and estimates from other established surveys series. Survey removed from analysis.
30394	Angola	Angola Integrated Inquiry into People's Well-Being 2008-2009	2008-2009	select	HepB3, PCV3	National Institute of Statistics (Angola), Oxford Policy Management, United Nations Children's Fund (UNICEF). Angola Integrated Inquiry into People's Well-Being 2008-2009.	Older age-cohorts precede introduction of PCV3 and HepB3 to national immunization schedule. Select age-cohorts removed from analysis for PCV3 and HepB3.
218555	Angola	Angola Demographic and Health Survey 2015-2016	2015-2016	select	MCV2, RotaC	ICF International, Ministry of Health (Angola), National Institute of Statistics (Angola), United Nations Children's Fund (UNICEF). Angola Demographic and Health Survey 2015-2016. Fairfax, United States of America: ICF International, 2017.	Older age-cohorts precede introduction of MCV2 and RotaC to national immunization schedule. Select age-cohorts removed from analysis for MCV2 and RotaC.
18843	Armenia	Armenia Demographic and Health Survey 2000	2000	select	HepB3	Ministry of Health (Armenia), National Statistical Service (NSS), ORC Macro. Armenia Demographic and Health Survey 2000. Fairfax, United States of America: ICF International.	Older age-cohorts precede introduction of HepB3 to national immunization schedule. Select age-cohorts removed from analysis for HepB3.
18854	Armenia	Armenia Demographic and Health Survey 2005	2005	all	BCG, DTP1, DTP3, HepB3, MCV1, Pol3	MOH Center for Health Information and Statistics, Macro International, Inc, National Statistical Service (NSS). Armenia Demographic and Health Survey 2005. Fairfax, United States of America: ICF International.	Survey removed from analysis due to small sample size and reliability concerns.

GHDx ID	Country	Series	Year(s)	Cohort	Vaccine(s)	Citation	Rationale for exclusion
31750	Armenia	Armenia Demographic and Health Survey 2010	2010	select	Hib3	ICF Macro, Ministry of Health (Armenia), National Statistical Service of the Republic of Armenia. Armenia Demographic and Health Survey 2010. Fairfax, United States of America: ICF International, 2015.	Older age-cohorts precede introduction of Hib3 to national immunization schedule. Select age-cohorts removed from analysis for Hib3.
218563	Armenia	Armenia Demographic and Health Survey 2015-2016	2015-2016	select	PCV3	ICF International, Ministry of Health (Armenia), National Statistical Service of the Republic of Armenia. Armenia Demographic and Health Survey 2015-2016. Fairfax, United States of America: ICF International, 2017.	Older age-cohorts precede introduction of PCV3 to national immunization schedule. Select age-cohorts removed from analysis for PCV3.
85056	Australia	Australia National Health Survey 2001	2001	select	Pol3	Australian Bureau of Statistics. Australia National Health Survey 2001. Canberra, Australia: Australian Bureau of Statistics.	Pol3 estimates do not follow a reasonable age structure. Older children have significantly higher coverage than reported in either administrative data or other survey series, suggesting survey is capturing results of catch-up immunization. Select age-cohorts removed from analysis for Pol3.
18865	Azerbaijan	Azerbaijan Demographic and Health Survey 2006	2006	select	RCV1	Macro International, Inc, State Statistical Committee of Azerbaijan. Azerbaijan Demographic and Health Survey 2006. Fairfax, United States of America: ICF International.	Older age-cohorts precede introduction of RCV1 to national immunization schedule. Select age-cohorts removed from analysis for RCV1.
215055	Bahrain	Bahrain Diarrhoeal Diseases Morbidity, Mortality and Treatment Rates and Immunization Coverage 1986	1986	all	DTP1, DTP3, MCV1, Pol3	Ministry of Health (Bahrain). Bahrain Diarrhoeal Diseases Morbidity, Mortality and Treatment Rates and Immunization Coverage 1986.	Estimates considered implausible. Survey estimates are systematically low compared to administrative data and estimates from other established surveys series. Survey removed from analysis.
905	Bahrain	Bahrain Child Health Survey 1989	1989	select	DTP1, DTP3, MCV1, Pol3, RCV1	Central Statistics Organization (Bahrain), Ministry of Health (Bahrain). Bahrain Child Health Survey 1989.	Estimates considered implausible. Survey estimates do not follow a reasonable age structure. Older children have significantly higher coverage than reported in either administrative data or other survey series, suggesting survey is capturing results of catch-up immunization. Select age-cohorts removed from analysis.

GHDx ID	Country	Series	Year(s)	Cohort	Vaccine(s)	Citation	Rationale for exclusion
482836*	Bangladesh	Bangladesh EPI Coverage Evaluation Survey 2019	2019	select	DTP3, MCV1	Center for Social and Market Research (CSMR) (Bangladesh), Directorate General of Health Services, Ministry of Health and Family Welfare (Bangladesh), United Nations Children's Fund (UNICEF), World Health Organization (WHO). Bangladesh EPI Coverage Evaluation Survey 2019.	Selected cohorts excluded from administrative data bias modeling as a methodological decision to improve model performance
215051	Bangladesh	Bangladesh EPI Coverage Evaluation Survey 2007-2008	2007-2008	all	PCV3	ACNielsen Bangladesh, Directorate General of Health Services, Ministry of Health and Family Welfare (Bangladesh), United Nations Children's Fund (UNICEF), World Health Organization (WHO). Bangladesh EPI Coverage Evaluation Survey 2007-2008.	Survey precedes introduction of PCV3 to national immunization schedule. PCV3 removed from analysis.
55956	Bangladesh	Bangladesh Demographic and Health Survey 2011-2012	2011-2012	select	Hib3	ICF Macro, Mitra and Associates, National Institute of Population Research and Training (NIPORT). Bangladesh Demographic and Health Survey 2011-2012. Calverton, United States of America: ICF Macro.	Older age-cohorts precede introduction of Hib3 to national immunization schedule. Select age-cohorts removed from analysis for Hib3.
464883	Bangladesh	Bangladesh Integrated Household Survey 2018-2019	2018-2019	all	BCG, DTP1, DTP3, HepB3, PCV3, MCV1, Pol3	International Food Policy Research Institute (IFPRI). 2020. Bangladesh Integrated Household Survey (BIHS) 2018-2019. Washington, DC: IFPRI [dataset]. https://doi.org/10.7910/DVN/NXKLZJ. Harvard Dataverse. Version 1.	Estimates considered implausible. Survey estimates are systematically low compared to administrative data and estimates from other established surveys series. Survey removed from analysis.
1039	Belarus	Belarus Multiple Indicator Cluster Survey 2005	2005	select	BCG, DTP1, DTP3, HepB3, Pol3	Ministry of Statistics and Analysis of the Republic of Belarus, United Nations Children's Fund (UNICEF). Belarus Multiple Indicator Cluster Survey 2005. New York, United States of America: United Nations Children's Fund (UNICEF).	Estimates considered implausible. Survey estimates do not follow a reasonable age structure. Older children have significantly higher coverage than reported in either administrative data or other survey series, suggesting survey is capturing results of catch-up immunization. Select age-cohorts removed from analysis.

GHDx ID	Country	Series	Year(s)	Cohort	Vaccine(s)	Citation	Rationale for exclusion
264910	Belize	Belize Multiple Indicator Cluster Survey 2015-2016	2015-2016	all	MCV2	Government of Belize, Statistical Institute of Belize, UN Resident Coordinator Fund (UN ResCor), United Nations Children's Fund (UNICEF), United Nations Development Programme (UNDP). Belize Multiple Indicator Cluster Survey 2015-2016. New York, United States of America: United Nations Children's Fund (UNICEF), 2018.	MCV2 estimates are implausibly low compared to administrative data and estimates from other established surveys series. MCV2 removed from analysis.
18959	Benin	Benin Demographic and Health Survey 2006	2006	select	Hib3	Macro International, Inc, National Institute of Statistics and Economic Analysis (INSAE) (Benin), National Program Against AIDS (PNLS) (Benin). Benin Demographic and Health Survey 2006. Fairfax, United States of America: ICF International.	Older age-cohorts precede introduction of Hib3 to national immunization schedule. Select age-cohorts removed from analysis for Hib3.
79839	Benin	Benin Demographic and Health Survey 2011-2012	2011-2012	all	BCG, DTP1, DTP3, HepB3, PCV3, MCV1, Pol3	ICF International, National Institute of Statistics and Economic Analysis (INSAE) (Benin), National Program Against AIDS (PNLS) (Benin). Benin Demographic and Health Survey 2011-2012. Fairfax, United States of America: ICF International, 2014.	Estimates considered implausible. Survey estimates are inconsistent with administrative data and estimates from other established survey series (Benin Demographic and Health Survey 2006). Survey removed from analysis.
215164	Bhutan	Bhutan National EPI Coverage Evaluation Survey 2002	2002	all	BCG, DTP1, DTP3, HepB3, MCV1, Pol3	Government of Bhutan, United Nations Children's Fund (UNICEF), World Health Organization (WHO). Bhutan National EPI Coverage Evaluation Survey 2002.	Survey estimates include implausibly high coverage rates of 100%. Survey removed from analysis.
215060*	Bolivia	Bolivia National Immunization Coverage Survey 1987	1987	select	BCG1, MCV1	Bolivia National Immunization Coverage Survey 1987.	Selected cohorts excluded from administrative data bias modeling as a methodological decision to improve model performance
18979*	Bolivia	Bolivia Demographic and Health Survey 1989	1989	select	BCG1, DTP1, MCV1	Macro Systems, Inc.; Institute for Resource Development, National Institute of Statistics (Bolivia). Bolivia Demographic and Health Survey 1989. Fairfax, United States of America: ICF International.	Selected cohorts excluded from administrative data bias modeling as a methodological decision to improve model performance

GHDx ID	Country	Series	Year(s)	Cohort	Vaccine(s)	Citation	Rationale for exclusion
32144	Bolivia	Bolivia Integrated Household Survey February 1989	1989	all	BCG, DTP1, DTP3, MCV1	National Institute of Statistics (Bolivia). Bolivia Integrated Household Survey February 1989. La Paz, Bolivia: National Institute of Statistics (Bolivia).	Estimates considered implausible. Survey estimates are systematically low compared to administrative data and estimates from other established surveys series. Survey removed from analysis.
32138	Bolivia	Bolivia Integrated Household Survey November 1989	1989	all	BCG, DTP1, DTP3, MCV1	National Institute of Statistics (Bolivia). Bolivia Integrated Household Survey November 1989. La Paz, Bolivia: National Institute of Statistics (Bolivia).	Estimates considered implausible. Survey estimates are systematically low compared to administrative data and estimates from other established surveys series. Survey removed from analysis.
215061*	Bolivia	Bolivia National Immunization Coverage Survey 1990	1990	select	BCG1	Bolivia National Immunization Coverage Survey 1990.	Selected cohorts excluded from administrative data bias modeling as a methodological decision to improve model performance
1278	Bolivia	Bolivia Household Survey 2002	2002	select	DTP1, DTP3	National Institute of Statistics (Bolivia), World Bank (WB). Bolivia Household Survey 2002. La Paz, Bolivia: National Institute of Statistics (Bolivia).	Survey removed from analysis due to small sample size and reliability concerns.
323944	Bolivia	Bolivia Demographic and Health Survey 2016	2016	select	PCV3	Ministry of Health (Bolivia), National Institute of Statistics (Bolivia). Bolivia Demographic and Health Survey 2016. La Paz, Bolivia: National Institute of Statistics (Bolivia), 2017.	Older age-cohorts precede introduction of PCV3 to national immunization schedule. Select age-cohorts removed from analysis for PCV3.
19027*	Brazil	Brazil Demographic and Health Survey 1986	1986	select	BCG, DTP1, DTP3, MCV1, MCV2, Pol3	Brazilian Society for Family Welfare (BEMFAM), Westinghouse; Institute for Resource Development. Brazil Demographic and Health Survey 1986. Fairfax, United States of America: ICF International.	Estimates considered implausible. Survey estimates do not follow a reasonable age structure. Older children have significantly higher coverage than reported in either administrative data or other survey series, suggesting survey is capturing results of catch-up immunization. Select age-cohorts removed from analysis. In addition, selected cohorts excluded from administrative data bias modeling as a methodological decision to improve model performance
19027*	Brazil	Brazil Demographic and Health Survey 1986	1986	select	DTP1	Brazilian Society for Family Welfare (BEMFAM), Westinghouse; Institute for Resource Development. Brazil Demographic and Health Survey 1986. Fairfax, United States of America: ICF International.	Selected cohorts excluded from administrative data bias modeling as a methodological decision to improve model performance

GHDx ID	Country	Series	Year(s)	Cohort	Vaccine(s)	Citation	Rationale for exclusion
19035*	Brazil	Brazil Demographic and Health Survey 1991	1991	select	BCG, DTP1, DTP3, MCV1, Pol3	Brazilian Society for Family Welfare (BEMFAM), Macro International, Inc. Brazil Demographic and Health Survey 1991. Fairfax, United States of America: ICF International.	Estimates considered implausible. Survey estimates do not follow a reasonable age structure. Older children have significantly higher coverage than reported in either administrative data or other survey series, suggesting survey is capturing results of catch-up immunization. Select age-cohorts removed from analysis. In addition, selected cohorts excluded from administrative data bias modeling as a methodological decision to improve model performance
19035*	Brazil	Brazil Demographic and Health Survey 1991	1991	select	DTP1, MCV1	Brazilian Society for Family Welfare (BEMFAM), Macro International, Inc. Brazil Demographic and Health Survey 1991. Fairfax, United States of America: ICF International.	Selected cohorts excluded from administrative data bias modeling as a methodological decision to improve model performance
19046*	Brazil	Brazil Demographic and Health Survey 1996	1996	select	BCG, DTP1, DTP3, MCV1, Pol3	Brazilian Society for Family Welfare (BEMFAM), Macro International, Inc. Brazil Demographic and Health Survey 1996. Fairfax, United States of America: ICF International.	Estimates considered implausible. Survey estimates do not follow a reasonable age structure. Older children have significantly higher coverage than reported in either administrative data or other survey series, suggesting survey is capturing results of catch-up immunization. Select age-cohorts removed from analysis. In addition, selected cohorts excluded from administrative data bias modeling as a methodological decision to improve model performance
19046*	Brazil	Brazil Demographic and Health Survey 1996	1996	select	DTP1, MCV1	Brazilian Society for Family Welfare (BEMFAM), Macro International, Inc. Brazil Demographic and Health Survey 1996. Fairfax, United States of America: ICF International.	Selected cohorts excluded from administrative data bias modeling as a methodological decision to improve model performance
141948	Brazil	Brazil National Demographic and Health Survey of Children and Women 2006-2007	2006-2007	all	DTP1	Brazilian Center for Analysis and Planning (CEBRAP), Brazilian Institute of Public Opinion and Statistics (IBOPE), Ministry of Health (Brazil). Brazil National Demographic and Health Survey of Children and Women 2006-2007. Rio de Janeiro, Brazil: Ministry of Health (Brazil).	DTP1 estimates are implausibly low compared to administrative data and estimates from other established surveys series. DTP1 removed from analysis.

GHDx ID	Country	Series	Year(s)	Cohort	Vaccine(s)	Citation	Rationale for exclusion
19088*	Burkina Faso	Burkina Faso Demographic and Health Survey 2003	2003	select	MCV1	Macro International, Inc, National Institute of Statistics and Demography (Burkina Faso). Burkina Faso Demographic and Health Survey 2003. Fairfax, United States of America: ICF International.	Selected cohorts excluded from administrative data bias modeling as a methodological decision to improve model performance
19064*	Burkina Faso	Burkina Faso Demographic and Health Survey 1992-1993	1992-1993	select	DTP3, Pol3	Macro International, Inc, National Institute of Statistics and Demography (Burkina Faso). Burkina Faso Demographic and Health Survey 1992-1993. Fairfax, United States of America: ICF International.	Selected cohorts excluded from administrative data bias modeling as a methodological decision to improve model performance
1970	Burundi	Burundi Priority Survey 1998-1999	1998-1999	all	BCG	Burundi Institute of Statistics and Economic Studies. Burundi Priority Survey 1998-1999.	BCG estimates include implausibly high coverage rates of 100%. BCG removed from analysis.
217919	Cabo Verde	Cape Verde Survey on DTP3 and Measles Coverage 2009	2009	all	DTP3, MCV1	Cape Verde Survey on DTP3 and Measles Coverage 2009.	Survey estimates include implausibly high coverage rates of 100%. Survey removed from analysis.
232764	Cambodia	Cambodia Vaccine Coverage Survey of Children 12-23 Months of Age 1995	1995	all	BCG, DTP1, DTP3	Cambodia Vaccine Coverage Survey of Children 12-23 Months of Age 1995.	BCG, DTP1 and DTP3 estimates are systematically high compared to administrative data and estimates from other established surveys series (Cambodia Special Demographic and Health Survey 1998). BCG, DTP3, DTP1 removed from analysis.
30758	Cambodia	Cambodia Socio-Economic Survey 1996	1996	select	BCG, DTP1, DTP3, MCV1	National Institute of Statistics (Cambodia). Cambodia Socio-Economic Survey 1996. Phnom Penh, Cambodia: National Institute of Statistics (Cambodia).	DTP1 and DTP3 estimates include implausibly low coverage rates of 0% for some age-cohorts. Select age cohorts remove d from analysis for DTP1 and DTP3.BCG and MCV1 removed from analysis due to small sample size and reliability concerns.
30907	Cambodia	Cambodia Socio-Economic Survey 1997	1997	all	BCG, DTP1, DTP3, MCV1, Pol3	National Institute of Statistics (Cambodia), World Bank. Cambodia Socio-Economic Survey 1997. Phnom Penh, Cambodia: National Institute of Statistics (Cambodia).	Survey estimates include implausibly high coverage rates of 100%. Survey removed from analysis.

GHDx ID	Country	Series	Year(s)	Cohort	Vaccine(s)	Citation	Rationale for exclusion
30842	Cambodia	Cambodia Socio-Economic Survey 1999	1999	all	BCG, DTP1, DTP3	National Institute of Statistics (Cambodia), United Nations Development Programme (UNDP), World Bank. Cambodia Socio-Economic Survey 1999. Phnom Penh, Cambodia: National Institute of Statistics (Cambodia).	Estimates considered implausible. Survey estimates are systematically high compared to administrative data and estimates from other established surveys series. Survey removed from analysis.
31143	Cambodia	Cambodia Socio-Economic Survey 2009	2009	all	BCG, DTP1, DTP3, MCV1	National Institute of Statistics (Cambodia), Statistics Sweden. Cambodia Socio-Economic Survey 2009. Phnom Penh, Cambodia: National Institute of Statistics (Cambodia).	Absence of maternal recall introduces potential bias; survey removed from analysis.
30379	Cambodia	Cambodia Demographic and Health Survey 2010-2011	2010-2011	all	Hib3	ICF Macro, Ministry of Health (Cambodia), National Institute of Statistics (Cambodia). Cambodia Demographic and Health Survey 2010-2011. Fairfax, United States of America: ICF International.	Survey precedes introduction of Hib3 to national immunization schedule. Hib3 removed from analysis.
19188*	Cameroon	Cameroon Demographic and Health Survey 1991	1991	select	BCG1, DTP1, DTP3, MCV1, Pol3	Macro International, Inc, Ministry of Economy, Planning and Regional Development (Cameroon). Cameroon Demographic and Health Survey 1991. Fairfax, United States of America: ICF International.	Selected cohorts excluded from administrative data bias modeling as a methodological decision to improve model performance
19274	Cameroon	Cameroon Demographic and Health Survey 2011	2011	select	Hib3	ICF International, Ministry of Economy, Planning and Regional Development (Cameroon), Ministry of Public Health (Cameroon), National Institute of Statistics (Cameroon), Pasteur Center of Cameroon. Cameroon Demographic and Health Survey 2011. Fairfax, United States of America: ICF International.	Older age-cohorts precede introduction of Hib3 to national immunization schedule. Select age-cohorts removed from analysis for Hib3.
244455	Cameroon	Cameroon Multiple Indicator Cluster Survey 2014	2014	select	BCG, DTP1, DTP3, HepB3, PCV3, PCV3, Pol3	Ministry of Public Health (Cameroon), National Institute of Statistics (Cameroon), United Nations Children's Fund (UNICEF). Cameroon Multiple Indicator Cluster Survey 2014. New York, United States of America: United Nations Children's Fund (UNICEF), 2017.	Survey estimates include implausibly high coverage rates of 100% for some age cohorts. Select age cohorts removed from analysis.

GHDx ID	Country	Series	Year(s)	Cohort	Vaccine(s)	Citation	Rationale for exclusion
217762*	Cameroon	Cameroon Immunization Coverage Survey November-December 1990	1987-1990	select	BCG1, DTP1, DTP3	Cameroon Immunization Coverage Survey November-December 1990.	Selected cohorts excluded from administrative data bias modeling as a methodological decision to improve model performance
546834	Canada	Canada Childhood National Immunization Coverage Survey 2022	2022	all	DTP3, HepB3, PCV3, PCV3, RotaC	Public Health Agency of Canada, Statistics Canada (StatCan). Canada Childhood National Immunization Coverage Survey 2022.	Estimates considered implausible. Survey estimates are systematically low compared to administrative data and estimates from other established surveys series. Survey removed from analysis.
482562	Canada	Canada Childhood National Immunization Coverage Survey 2019-2020	2019-2020	all	DTP3, PCV3, Hib3	Public Health Agency of Canada, Statistics Canada (StatCan). Canada Childhood National Immunization Coverage Survey 2019-2020.	DTP3, PCV3, Hib3 estimates do not follow a reasonable age structure. DTP3, PCV3, Hib3 removed from analysis
2223*	Central African Republic	Central African Republic Multiple Indicator Cluster Survey 2006	2006	select	Pol3	United Nations Children's Fund (UNICEF). Central African Republic Multiple Indicator Cluster Survey 2006. New York, United States: United Nations Children's Fund (UNICEF).	Selected cohorts excluded from administrative data bias modeling as a methodological decision to improve model performance
215247	Central African Republic	Central African Republic EPI Coverage Survey 2012	2012	all	Pol3	Ministry of Public Health, Population, and AIDS Control (Central African Republic). Central African Republic EPI Coverage Survey 2012.	Pol3 estimates are implausibly high compared to administrative data and estimates from other established surveys series (Central African Republic Multiple Indicator Cluster Survey 2010-2011). Pol3 removed from analysis.
156585	Central African Republic	Central African Republic Public Expenditure Review 2012	1986-2010	all	BCG, DTP3, HepB3, PCV3, MCV1, Pol3	World Bank. Central African Republic Public Expenditure Review 2012. Washington DC, United States of America: World Bank, 2012.	Estimates considered implausible. Survey estimates are systematically high compared to administrative data and estimates from other established surveys series. Survey removed from analysis.
215186*	Central African Republic	Central African Republic Immunization Coverage Survey February 1991	1989-1991	select	DTP1, DTP3, MCV1, Pol3	Central African Republic Immunization Coverage Survey February 1991.	Selected cohorts excluded from administrative data bias modeling as a methodological decision to improve model performance
82832	Central African Republic	Central African Republic Multiple Indicator Cluster Survey 2010-2011	2010-2011	select	HepB3	Central African Institute of Statistics, Economic and Social Studies (ICASEES) (Central African Republic), ICF International. Central African Republic Multiple Indicator Cluster Survey 2010-2011. Fairfax, United States of America: ICF International, 2013.	Older age-cohorts precede introduction of HepB3 to national immunization schedule. Select age cohorts removed from analysis for HepB3.

GHDx ID	Country	Series	Year(s)	Cohort	Vaccine(s)	Citation	Rationale for exclusion
466384	Central African Republic	Central African Republic Multiple Indicator Cluster Survey 2018-2019	2018-2019	all	RCV1	Central African Institute of Statistics, Economic and Social Studies (ICASEES) (Central African Republic). Central African Republic Multiple Indicator Cluster Survey 2018-2019. New York, United States of America: United Nations Children's Fund (UNICEF), 2021.	Survey precedes introduction of RCV1 to national immunization schedule. RCV1 removed from analysis.
2244*	Chad	Chad Multiple Indicator Cluster Survey 2000	2000	select	Pol3	United Nations Children's Fund (UNICEF), Census Bureau (Chad), National Institute of Statistical, Economic and Demographic Studies (Chad). Chad Multiple Indicator Cluster Survey 2000. New York, United States: United Nations Children's Fund (UNICEF).	Selected cohorts excluded from administrative data bias modeling as a methodological decision to improve model performance
19315*	Chad	Chad Demographic and Health Survey 2004	2004	select	Pol3	Macro International, Inc, National Institute for Statistics, Economic and Demographic Studies (INSEED) (Chad). Chad Demographic and Health Survey 2004. Fairfax, United States of America: ICF International.	Selected cohorts excluded from administrative data bias modeling as a methodological decision to improve model performance
76701	Chad	Chad Multiple Indicator Cluster Survey 2010	2010	select	HepB3	Ministry of Planning, Economy, and International Cooperation (Chad), National Institute for Statistics, Economic and Demographic Studies (INSEED) (Chad), United Nations Children's Fund (UNICEF). Chad Multiple Indicator Cluster Survey 2010. New York, United States of America: United Nations Children's Fund (UNICEF), 2014.	Older age-cohorts precede introduction of HepB3 to national immunization schedule. Select age-cohorts removed from analysis for HepB3.
466891	Chad	Chad Multiple Indicator Cluster Survey 2019	2019	all	BCG, DTP1, DTP3, HepB3, PCV3, MCV1, Pol3, RCV1	National Institute for Statistics, Economic and Demographic Studies (INSEED) (Chad), United Nations Children's Fund (UNICEF). Chad Multiple Indicator Cluster Survey 2019. New York, United States of America: United Nations Children's Fund (UNICEF), 2021.	Data collection error leading to reliability concerns. Survey removed from analysis.

GHDx ID	Country	Series	Year(s)	Cohort	Vaccine(s)	Citation	Rationale for exclusion
59878	China	China Health Statistics Yearbook 2005	2004	all	BCG, DTP3, MCV1, Pol3	Ministry of Health (China). China Health Statistics Yearbook 2005.	Estimates considered implausible. Survey estimates are systematically high compared to administrative data and estimates from other established survey series. Survey removed from analysis.
60341	China	China Health Statistics Yearbook 2007	2006	all	BCG, DTP3, MCV1, Pol3	Ministry of Health (China). China Health Statistics Yearbook 2007.	Estimates considered implausible. Survey estimates are systematically high compared to administrative data and estimates from other established survey series. Survey removed from analysis.
74241	China	China Health Statistics Yearbook 2009	2008	all	BCG, DTP3, MCV1, Pol3	Ministry of Health (China). China Health Statistics Yearbook 2009.	Estimates considered implausible. Survey estimates are systematically high compared to administrative data and estimates from other established survey series. Survey removed from analysis.
394214	China	Survey on the immunization status of category B vaccine among children aged 1 to 2 years in China	2009-2011	all	DTP3, PCV3, PCV3	Zheng Jing-shan, Cao Lei, Guo Shi-cheng. Survey on the immunization status of category B vaccine among children aged 1 to 2 years in China. Chin J Vaccines Immun. 2012; 18(3): 2337.	Survey removed from analysis due to small sample size and reliability concerns.
19333*	Colombia	Colombia Demographic and Health Survey 1986	1986	select	DTP1, DTP3, MCV1, Pol3	Ministry of Health (Colombia), Regional Population Center (Colombia), Westinghouse; Institute for Resource Development. Colombia Demographic and Health Survey 1986. Fairfax, United States of America: ICF International, 1988.	Selected cohorts excluded from administrative data bias modeling as a methodological decision to improve model performance
19341*	Colombia	Colombia Demographic and Health Survey 1990	1990	select	MCV1	Institute for Resource Development, Macro Systems, Profamilia (Colombia). Colombia Demographic and Health Survey 1990. Fairfax, United States of America: ICF International, 1991.	Selected cohorts excluded from administrative data bias modeling as a methodological decision to improve model performance
19359	Colombia	Colombia Demographic and Health Survey 2000	2000	select	PCV3	Macro International, Inc, Profamilia (Colombia). Colombia Demographic and Health Survey 2000. Fairfax, United States of America: ICF International, 2000.	Older age-cohorts precede introduction of PCV3 to national immunization schedule. Select age-cohorts removed from analysis for PCV3.

GHDx ID	Country	Series	Year(s)	Cohort	Vaccine(s)	Citation	Rationale for exclusion
3100	Colombia	Colombia National Health Survey 2007-2008	2007-2008	all	DTP1, DTP3	Administrative Department of Science, Technology, and Innovation (Colombia), Center for Development Projects, Pontifical Xavierian University, Ministry of Social Protection (Colombia), Specialized Information Systems. Colombia National Health Survey 2007-2008.	Survey estimates include implausibly low coverage rates of 0%. Survey removed from analysis.
541281	Comoros	Comoros Multiple Indicator Cluster Survey 2022	2022	select	MCV2, RCV1	National Institute of Statistics and Economic and Demographic Studies (INSEED) (Comoros), United Nations Children's Fund (UNICEF). Comoros Multiple Indicator Cluster Survey 2022. New York, United States of America: United Nations Children's Fund (UNICEF), 2023.	Older age-cohorts precede introduction of these vaccines to national immunization schedule. Select age cohorts removed from analysis for these vaccines.
76850	Comoros	Comoros Demographic and Health Survey 2012-2013	2012-2013	select	Hib3	General Directorate of Statistics and Forecasting (Comoros), ICF International. Comoros Demographic and Health Survey 2012-2013. Fairfax, United States of America: ICF International.	Older age-cohorts precede introduction of Hib3 to national immunization schedule. Select age-cohorts removed from analysis for Hib3.
21535	Congo (Brazzaville)	Congo World Health Survey 2003	2003	all	DTP1, DTP3, MCV1	World Health Organization (WHO). Congo World Health Survey 2003. Geneva, Switzerland: World Health Organization (WHO), 2005.	Estimates considered implausible. Survey estimates are inconsistent with administrative data and estimates from other established survey series. Survey removed from analysis.
19391	Congo (Brazzaville)	Congo Demographic and Health Survey 2005	2005	select	BCG, DTP1, DTP3, MCV1, Pol3	Macro International, Inc, National Center for Statistics and Economic Studies (Congo, Rep.). Congo Demographic and Health Survey 2005. Fairfax, United States of America: ICF International.	Estimates considered implausible. Survey estimates do not follow a reasonable age structure. Older children have significantly higher coverage than reported in either administrative data or other survey series, suggesting survey is capturing results of catch-up immunization. Select age-cohorts removed from analysis.
217774	Congo (Brazzaville)	Congo External EPI Review 2010	2010	all	HepB3, PCV3	Congo External EPI Review 2010.	Estimates considered implausible. Survey estimates are systematically high compared to administrative data and estimates from other established surveys series (Congo Demographic and Health Survey 2011-2012). Survey removed from analysis.

GHDx ID	Country	Series	Year(s)	Cohort	Vaccine(s)	Citation	Rationale for exclusion
56151	Congo (Brazzaville)	Congo Demographic and Health Survey 2011-2012	2011-2012	select	PCV3	ICF International, Ministry of Health (Congo, Rep.), National Center for Statistics and Economic Studies (Congo, Rep.). Congo Demographic and Health Survey 2011-2012. Fairfax, United States of America: ICF International.	Older age-cohorts precede introduction of PCV3 to national immunization schedule. Select age-cohorts removed from analysis for PCV3.
163120	Costa Rica	Costa Rica National Health Survey 2006	2006	all	DTP1, MCV1	Central American Population Center, University of Costa Rica. Costa Rica National Health Survey 2006. San José, Costa Rica: Central American Population Center, University of Costa Rica.	Estimates considered implausible. Survey estimates are systematically low compared to administrative data and estimates from other established surveys series (Costa Rica Survey of Family Health Services and Expenses 2008). Survey removed from analysis.
164089	Costa Rica	Costa Rica Survey of Family Health Services and Expenses 2008	2008	all	BCG, DTP1	Central American Population Center, University of Costa Rica. Costa Rica Survey of Family Health Services and Expenses 2008. San José, Costa Rica: Central American Population Center, University of Costa Rica.	Survey removed from analysis due to small sample size and reliability concerns.
125596	Costa Rica	Costa Rica Multiple Indicator Cluster Survey 2011	2011	select	PCV3	Costa Rican Demographic Association, Ministry of Health (Costa Rica), United Nations Children's Fund (UNICEF). Costa Rica Multiple Indicator Cluster Survey 2011. New York, United States of America: United Nations Children's Fund (UNICEF), 2013.	Older age-cohorts precede introduction of PCV3 to national immunization schedule. Select age-cohorts removed from analysis for PCV3.
217173	Cote d'Ivoire	Cote d'Ivoire Immunization Coverage Survey 1987	1987	all	DTP1, DTP3, MCV1, Pol3	Cote d'Ivoire Immunization Coverage Survey 1987.	Estimates considered implausible. Survey estimates are systematically high compared to administrative data and estimates from other established surveys series (Cote d'Ivoire Demographic and Health Survey 1994). Survey removed from analysis.
218611	Cote d'Ivoire	Cote d'Ivoire Multiple Indicator Cluster Survey 2016	2016	all	DTP1, DTP3, RotaC	National Institute of Statistics (Cote d'Ivoire), United Nations Children's Fund (UNICEF). Cote d'Ivoire Multiple Indicator Cluster Survey 2016. New York, United States of America: United Nations Children's Fund (UNICEF), 2018.	Survey estimates include implausibly high coverage rates of 100%. Survey removed from analysis.
502491	Cote d'Ivoire	Cote d'Ivoire Routine Vaccination Coverage Survey 2018	2018	all	RCV1	Cote d'Ivoire Routine Vaccination Coverage Survey 2018.	Survey precedes introduction of RCV1 to national immunization schedule. RCV1 removed from analysis.

GHDx ID	Country	Series	Year(s)	Cohort	Vaccine(s)	Citation	Rationale for exclusion
18533	Cote d'Ivoire	Cote d'Ivoire Demographic and Health Survey 2011-2012	2011-2012	select	PCV3	ICF International, Ministry of the Fight Against AIDS (Cote d'Ivoire), National Institute of Statistics (Cote d'Ivoire). Cote d'Ivoire Demographic and Health Survey 2011-2012. Fairfax, United States of America: ICF International.	Older age-cohorts precede introduction of PCV3 to national immunization schedule. Select age cohorts removed from analysis for PCV3.
194014	Croatia	Croatia Multiple Indicator Cluster Survey 1996	1996	all	BCG, DTP3	Children's Hospital Zagreb, National Institute of Health (Italy), United Nations Children's Fund (UNICEF). Croatia Multiple Indicator Cluster Survey 1996.	Estimates considered implausible. Survey estimates are systematically low compared to administrative data and estimates from other established surveys series. Survey removed from analysis.
7465*	Democratic People's Republic of Korea	North Korea Multiple Indicator Cluster Survey 1998	1998	select	DTP3	European Union (EU), Government of North Korea, United Nations Children's Fund (UNICEF), World Food Programme (WFP). North Korea Multiple Indicator Cluster Survey 1998.	Selected cohorts excluded from administrative data bias modeling as a methodological decision to improve model performance
3161*	Democratic Republic of the Congo	Democratic Republic of the Congo Multiple Indicator Cluster Survey 2001	2001	select	MCV1	Ministry of Planning and Reconstruction (Congo, DR), United Nations Children's Fund (UNICEF). Congo, DR Multiple Indicator Cluster Survey 2001. New York, United States: United Nations Children's Fund (UNICEF).	Selected cohorts excluded from administrative data bias modeling as a methodological decision to improve model performance
233644	Denmark	Denmark Act No. 707, Act to Amend the Law Prohibiting the Sale of Tobacco to Persons Under the Age of 18, and the Sale of Alcohol to Persons Under the Age of 16, 2010	2010	all	BCG, DTP1, DTP3, MCV1, Pol3	Ministry of Interior and Health (Denmark). Denmark Act No. 707, Act to Amend the Law Prohibiting the Sale of Tobacco to Persons Under the Age of 18, and the Sale of Alcohol to Persons Under the Age of 16, 2010. Copenhagen, Denmark: Government of Denmark.	Estimates considered implausible. Survey estimates are systematically low compared to administrative data and estimates from other established surveys series. Survey removed from analysis.
3386	Djibouti	Djibouti Household Survey - Social Indicators 1996	1996	all	BCG, DTP1, DTP3, MCV1, Pol3	National Directorate of Statistics, Ministry of Commerce and Tourism, World Bank (WB). Djibouti Household Survey - Social Indicators 1996. Djibouti, Djibouti: National Directorate of Statistics, Ministry of Commerce and Tourism.	Estimates considered implausible. Survey estimates are systematically high compared to administrative data and estimates from other established surveys series. Survey removed from analysis.

GHDx ID	Country	Series	Year(s)	Cohort	Vaccine(s)	Citation	Rationale for exclusion
3392	Djibouti	Djibouti Family Health Survey 2002	2002	select	BCG, DTP1, DTP3, MCV1, Pol3, RCV1	Department of Statistics and Demographic Studies (Djibouti), League of Arab States, Ministry of Health (Djibouti), Pan Arab Project for Family Health (PAPFAM). Djibouti Family Health Survey 2002.	Estimates considered implausible. Survey estimates do not follow a reasonable age structure. Older children have significantly higher coverage than reported in either administrative data or other survey series, suggesting survey is capturing results of catch-up immunization. Select age-cohorts removed from analysis.
218035	Djibouti	Djibouti Family Health Survey 2012	2012	select	HepB3, PCV3, MCV2, Pol3	Department of Statistics and Demographic Studies (Djibouti), League of Arab States, Ministry of Health (Djibouti), Pan Arab Project for Family Health (PAPFAM). Djibouti Family Health Survey 2012.	Survey removed from analysis due to small sample size and reliability concerns.
19400*	Dominican Republic	Dominican Republic Demographic and Health Survey 1986	1986	select	DTP1, DTP3, Pol3	National Council for Population and Family (Dominican Republic), Westinghouse; Institute for Resource Development. Dominican Republic Demographic and Health Survey 1986. Fairfax, United States of America: ICF International.	Selected cohorts excluded from administrative data bias modeling as a methodological decision to improve model performance
19410*	Dominican Republic	Dominican Republic Demographic and Health Survey 1991	1991	select	MCV1	Institute for Resource Development, Macro Systems, National Planning Office (Dominican Republic), Profamilia (Colombia). Dominican Republic Demographic and Health Survey 1991. Fairfax, United States of America: ICF International.	Selected cohorts excluded from administrative data bias modeling as a methodological decision to improve model performance
27069	Dominican Republic	Dominican Republic Multiple Indicator Cluster Survey 2000	2000	all	MCV1	Center for Social and Demographic Studies (Dominican Republic) (CESDEM), United Nations Children's Fund (UNICEF). Dominican Republic Multiple Indicator Cluster Survey 2000. New York, United States of America: United Nations Children's Fund (UNICEF).	MCV1 estimates are implausibly low compared to administrative data and estimates from other established survey series. MCV1 removed from analysis.
19444	Dominican Republic	Dominican Republic Demographic and Health Survey 2002	2002	select	PCV3	Center for Social and Demographic Studies (Dominican Republic) (CESDEM), Macro International, Inc. Dominican Republic Demographic and Health Survey 2002. Fairfax, United States of America: ICF International.	Older age-cohorts precede introduction of PCV3 to national immunization schedule. Select age-cohorts removed from analysis for PCV3.

GHDx ID	Country	Series	Year(s)	Cohort	Vaccine(s)	Citation	Rationale for exclusion
3455	Dominican Republic	Dominican Republic National Multipurpose Household Survey 2006	2006	select	MCV2, Pol3, RCV1	National Statistics Office (Dominican Republic), United Nations Children's Fund (UNICEF). Dominican Republic National Multipurpose Household Survey 2006. Santo Domingo, Dominican Republic: National Statistics Office (Dominican Republic).	MCV2, Pol3, and RCV1 estimates are systematically low compared to administrative data and estimates from other established surveys series. MCV2, Pol3, and RCV1 removed from analysis.
19456	Dominican Republic	Dominican Republic Demographic and Health Survey 2007	2007	select	RCV1	Center for Social and Demographic Studies (Dominican Republic) (CESDEM), Macro International, Inc. Dominican Republic Demographic and Health Survey 2007. Fairfax, United States of America: ICF International.	Older age-cohorts precede introduction of RCV1 to national immunization schedule. Select age-cohorts removed from analysis for RCV1.
21198	Dominican Republic	Dominican Republic Special Demographic and Health Survey 2007	2007	all	BCG, DTP1, DTP3, MCV1, Pol3	Center for Social and Demographic Studies (Dominican Republic) (CESDEM), Macro International, Inc. Dominican Republic Special Demographic and Health Survey 2007. Fairfax, United States of America: ICF International.	Survey is not nationally representative. Survey removed from analysis.
77819	Dominican Republic	Dominican Republic Demographic and Health Survey 2013	2013	select	RotaC	Center for Social and Demographic Studies (Dominican Republic) (CESDEM), ICF International, Ministry of Public Health and Social Assistance (Dominican Republic). Dominican Republic Demographic and Health Survey 2013. Fairfax, United States of America: ICF International, 2014.	Older age-cohorts precede introduction of rotavirus vaccine to national immunization schedule. Rotavirus vaccine removed from analysis.
165645	Dominican Republic	Dominican Republic Special Demographic and Health Survey 2013	2013	all	BCG, DTP1, DTP3, MCV1, Pol3	Center for Social and Demographic Studies (Dominican Republic) (CESDEM), ICF International, National Public Health Laboratory (Dominican Republic). Dominican Republic Special Demographic and Health Survey 2013. Fairfax, United States of America: ICF International, 2015.	Survey is not nationally representative. Survey removed from analysis.

GHDx ID	Country	Series	Year(s)	Cohort	Vaccine(s)	Citation	Rationale for exclusion
200697	Dominican Republic	Dominican Republic Multiple Indicator Cluster Survey 2014	2014	select	PCV3	National Statistics Office (Dominican Republic), United Nations Children's Fund (UNICEF). Dominican Republic Multiple Indicator Cluster Survey 2014. New York, United States of America: United Nations Children's Fund (UNICEF), 2016.	Survey precedes introduction of PCV3 to national immunization schedule. PCV3 removed from analysis.
488697	Dominican Republic	Dominican Republic Multiple Indicator Cluster Survey 2019	2019	all	MCV2, Pol3	National Statistics Office (Dominican Republic), United Nations Children's Fund (UNICEF). Dominican Republic Multiple Indicator Cluster Survey 2019. New York, United States of America: United Nations Children's Fund (UNICEF), 2021.	Pol3 estimates considered implausible. Survey estimates are inconsistent with administrative data and estimates from other established survey series. Pol3 removed from analysis. Survey precedes introduction of MCV2 to national immunization schedule. MCV2, Pol3 removed from analysis.
3161*	DR Congo	Democratic Republic of the Congo Multiple Indicator Cluster Survey 2001	2001	select	BCG, DTP1, DTP3, MCV1, Pol3	Ministry of Planning and Reconstruction (Congo, DR), United Nations Children's Fund (UNICEF). Congo, DR Multiple Indicator Cluster Survey 2001. New York, United States: United Nations Children's Fund (UNICEF).	Survey removed from analysis due to small sample size and reliability concerns. In addition, selected cohorts excluded from administrative data bias modeling as a methodological decision to improve model performance
26998	DR Congo	Democratic Republic of the Congo Multiple Indicator Cluster Survey 2010	2010	select	HepB3	National Statistical Institute (Congo, DR), Ministry of Planning (Congo, DR), United Nations Children's Fund (UNICEF). Congo, DR Multiple Indicator Cluster Survey 2010. New York, United States: United Nations Children's Fund (UNICEF).	Older age-cohorts precede introduction of HepB3 to national immunization schedule. Select age cohorts removed from analysis for HepB3.
437955	DR Congo	Democratic Republic of the Congo Multiple Indicator Cluster Survey 2017-2018	2017-2018	all	Pol3	National Statistical Institute (Congo, DR), Ministry of Planning (Congo, DR), United Nations Children's Fund (UNICEF). Congo, DR Multiple Indicator Cluster Survey 2010. New York, United States: United Nations Children's Fund (UNICEF).	Estimates considered implausible. Survey estimates are systematically low compared to administrative data and estimates from other established surveys series. Survey removed from analysis.
27630	Ecuador	Ecuador Reproductive Health Survey 2004	2004	select	DTP1, DTP3, MCV1	Center for Studies of Population and Social Development (CEPAR) (Ecuador) and Division of Reproductive Health-Centers for Disease Control and Prevention (CDC). (2005) Ecuador Reproductive Health Survey 2004. Quito, Ecuador: CEPAR.	DTP1, DTP3, and MCV1 estimates considered implausible for some age-cohorts due to sudden drop in coverage. Coverage trends are inconsistent with administrative data and estimates from other established survey series (Ecuador Living Conditions Survey 2005-2006). Select age-cohorts removed from analysis for DTP1, DTP3, and MCV1.

GHDx ID	Country	Series	Year(s)	Cohort	Vaccine(s)	Citation	Rationale for exclusion
153674	Ecuador	Ecuador National Health and Nutrition Survey 2012	2012	select	PCV3, Pol3	Ministry of Public Health (Ecuador), National Institute of Statistics and Censuses (Ecuador). Ecuador National Health and Nutrition Survey 2012.	Pol3 estimates considered implausible due to sudden drop in coverage inconsistent with administrative data and estimates from other established survey series (Ecuador Living Conditions Survey 2013-2014). Older age-cohorts precede introduction of PCV3 to national immunization schedule. Pol3 and select age-cohorts of PCV3 removed from analysis.
364181	Ecuador	Ecuador Living Conditions Survey 2013-2014	2013-2014	select	PCV3	National Institute of Statistics and Censuses (Ecuador). Ecuador Living Conditions Survey 2013-2014. Quito, Ecuador: National Institute of Statistics and Censuses (Ecuador).	Older age-cohorts precede introduction of PCV3 to national immunization schedule. Select age-cohorts removed from analysis for PCV3.
154897	Egypt	Egypt Demographic and Health Survey 2014	2014	all	PCV3	El-Zanaty and Associates, ICF International, Ministry of Health and Population (Egypt). Egypt Demographic and Health Survey 2014. Fairfax, United States of America: ICF International.	Survey precedes introduction of PCV3 to national immunization schedule. PCV3 removed from analysis.
19482	Egypt	Egypt Demographic and Health Survey 1992-1993	1992-1993	all	HepB3	Macro International, Inc, Population Council (Egypt). Egypt Demographic and Health Survey 1992-1993. Fairfax, United States of America: ICF International.	Survey precedes introduction of HepB3 to national immunization schedule. HepB3 removed from analysis.
19493	Egypt	Egypt Demographic and Health Survey 1995-1996	1995-1996	select	HepB3	Macro International, Inc, Population Council (Egypt). Egypt Demographic and Health Survey 1995-1996. Fairfax, United States of America: ICF International.	Older age-cohorts precede introduction of HepB3 to national immunization schedule. Select age cohorts removed from analysis for HepB3.
19533	El Salvador	El Salvador Demographic and Health Survey 1985	1985	all	BCG, DTP1, DTP3, MCV1, Pol3	Salvadoran Demographic Association (ADS), Westinghouse; Institute for Resource Development. El Salvador Demographic and Health Survey 1985. Fairfax, United States of America: ICF International.	Estimates considered implausible. Survey estimates are systematically high compared to administrative data and estimates from other established surveys series. Survey removed from analysis.
27606	El Salvador	El Salvador Reproductive Health Survey 2008	2008	all	BCG, DTP1, DTP3, MCV1, Pol3	Asociación Demográfica Salvadoreña (ADS), Division of Reproductive Health-Centers for Disease Control and Prevention (CDC). (2009) El Salvador Reproductive Health Survey 2008. San Salvador, El Salvador: ADS.	Estimates considered implausible. Survey estimates are systematically low compared to administrative data and estimates from other established surveys series. Survey removed from analysis.

GHDx ID	Country	Series	Year(s)	Cohort	Vaccine(s)	Citation	Rationale for exclusion
526221	El Salvador	El Salvador National Health Survey 2021	2021	all	MCV2	General Directorate of Statistics and Census (DIGESTYC) (El Salvador), National Institute of Health (El Salvador). El Salvador National Health Survey 2021.	Survey precedes introduction of MCV2 to national immunization schedule. MCV2 removed from analysis.
27599	El Salvador	El Salvador Reproductive Health Survey 2002-2003	2002-2003	select	DTP1, DTP3, MCV1	Asociación Demográfica Salvadoreña (ADS), Division of Reproductive Health-Centers for Disease Control and Prevention (CDC). (2004) El Salvador Reproductive Health Survey 2002-2003. San Salvador, El Salvador: ADS.	Estimates considered implausible. Survey estimates are systematically low compared to administrative data and estimates from other established surveys series. Survey removed from analysis.
233663	Equatorial Guinea	Equatorial Guinea National Immunization Coverage Survey 1994	1994	all	BCG, DTP1, DTP3, MCV1, Pol3	Equatorial Guinea National Immunization Coverage Survey 1994.	Estimates considered implausible. Survey estimates are systematically high compared to administrative data and estimates from other established surveys series (Equatorial Guinea Multiple Indicator Cluster Survey 2000). Survey removed from analysis.
76884	Equatorial Guinea	Equatorial Guinea Demographic and Health Survey 2011	2011	all	HepB3, PCV3, RCV1	ICF International, Ministry of Health and Social Welfare (Equatorial Guinea), Ministry of Planning, Economic Development and Public Investment (Equatorial Guinea). Equatorial Guinea Demographic and Health Survey 2011. Fairfax, United States of America: ICF International, 2012.	Survey precedes introduction of BCG, PCV, and RCV to national immunization schedule. BCG, PCV, and RCV removed from analysis.
453344*	Equatorial Guinea	Equatorial Guinea External Review of Expanded Programme on Immunization National Coverage Survey 2016	2016	select	BCG1, DTP1, DTP3, MCV1, Pol3	Equatorial Guinea External Review of Expanded Programme on Immunization National Coverage Survey 2016.	Selected cohorts excluded from administrative data bias modeling as a methodological decision to improve model performance
19546*	Eritrea	Eritrea Demographic and Health Survey 1995-1996	1995-1996	select	MCV1	Macro International, Inc, National Statistics Office (Eritrea). Eritrea Demographic and Health Survey 1995-1996. Fairfax, United States of America: ICF International.	Selected cohorts excluded from administrative data bias modeling as a methodological decision to improve model performance

GHDx ID	Country	Series	Year(s)	Cohort	Vaccine(s)	Citation	Rationale for exclusion
200707	Eswatini	Swaziland Multiple Indicator Cluster Survey 2014	2014	all	PCV3	Central Statistical Office (Swaziland), United Nations Children's Fund (UNICEF), United Nations Educational, Scientific and Cultural Organization (UNESCO), United Nations Population Fund (UNFPA). Swaziland Multiple Indicator Cluster Survey 2014. New York, United States of America: United Nations Children's Fund (UNICEF), 2016.	Survey precedes introduction of PCV3 to national immunization schedule. PCV3 removed from analysis.
543406	Eswatini	Eswatini Multiple Indicator Cluster Survey 2021-2022	2021-2022	all	MCV1, MCV2, RCV1	Central Statistical Office (Eswatini), United Nations Children's Fund (UNICEF). Eswatini Multiple Indicator Cluster Survey 2021-2022. 2023.	MCV and RCV1 estimate derived from a post-campaign coverage survey. MCV and RCV2 removed from analysis.
194013*	Ethiopia	Ethiopia Multiple Indicator Cluster Survey 1995	1995	select	DTP3, Pol3	Ministry of Health (Ethiopia), United Nations Children's Fund (UNICEF). Ethiopia Multiple Indicator Cluster Survey 1995.	Selected cohorts excluded from administrative data bias modeling as a methodological decision to improve model performance
34047*	Ethiopia	Ethiopia Welfare Monitoring Survey 1996	1996	select	DTP3, MCV1	Central Statistics Agency (Ethiopia). Ethiopia Welfare Monitoring Survey 1996.	Selected cohorts excluded from administrative data bias modeling as a methodological decision to improve model performance
3716	Ethiopia	Ethiopia Health and Nutrition Survey 1998	1998	all	BCG, DTP3, MCV1, Pol3	Central Statistics Agency (Ethiopia). Ethiopia Health and Nutrition Survey 1998.	Estimates considered implausible. Survey estimates are systematically high compared to administrative data and estimates from other established surveys series (Ethiopia Demographic and Health Survey 2000). Survey removed from analysis.
34059	Ethiopia	Ethiopia Welfare Monitoring Survey 1998	1998	all	BCG, DTP3, MCV1, Pol3	Central Statistics Agency (Ethiopia). Ethiopia Welfare Monitoring Survey 1998.	Estimates considered implausible. Survey estimates are systematically high compared to administrative data and estimates from other established surveys series (Ethiopia Demographic and Health Survey 2000). Survey removed from analysis.
225021	Ethiopia	Ethiopia National EPI Coverage Survey 2001	2001	all	BCG, DTP1, DTP3, MCV1, Pol3	Ethiopia National EPI Coverage Survey 2001.	Estimates considered implausible. Survey estimates are systematically high compared to administrative data and estimates from other established surveys series (Ethiopia Demographic and Health Survey 2000). Survey removed from analysis.

GHDx ID	Country	Series	Year(s)	Cohort	Vaccine(s)	Citation	Rationale for exclusion
21591	Ethiopia	Ethiopia World Health Survey 2003	2003	all	DTP1, DTP3, MCV1	World Health Organization (WHO). Ethiopia World Health Survey 2003. Geneva, Switzerland: World Health Organization (WHO), 2005.	Estimates considered implausible. Survey estimates are systematically high compared to administrative data and estimates from other established surveys series (Ethiopia Demographic and Health Survey 2005). Survey removed from analysis.
34085	Ethiopia	Ethiopia Welfare Monitoring Survey 2004	2004	all	BCG, DTP1, DTP3, MCV1, Pol3	Central Statistics Agency (Ethiopia). Ethiopia Welfare Monitoring Survey 2004.	Estimates considered implausible. Survey estimates are systematically high compared to administrative data and estimates from other established surveys series (Ethiopia Demographic and Health Survey 2005). Survey removed from analysis.
376302	Ethiopia	Ethiopia Young Lives: Household and Child Survey Round 5 2016 - UK Data Service	2016	all	BCG, DTP3, HepB3, PCV3, MCV1, PCV3, Pol3	Boyden, J., Duc, L. Thuc, Galab, S., Penny, M., Sanchez, A., Woldehanna, T. (2018). Young Lives: an International Study of Childhood Poverty: Round 5, 2016. [data collection]. UK Data Service. SN: 8357, http://doi.org/10.5255/UKDA-SN-8357-1.	Estimates considered implausible. Survey estimates are systematically high compared to administrative data and estimates from other established surveys series (Ethiopia Demographic and Health Survey 2016). Survey removed from analysis.
416582	Ethiopia	Ethiopia Interim Demographic and Health Survey 2019	2019	all	MCV2	Central Statistics Agency (Ethiopia), Ethiopian Public Health Institute (EPHI), ICF International, Ministry of Health (Ethiopia). Ethiopia Interim Demographic and Health Survey 2019.	Survey precedes introduction of MCV2 to national immunization schedule. MCV2 removed from analysis.
3726	Ethiopia	Ethiopia National Fertility and Family Survey 1990-1991	1990-1991	all	BCG, DTP1, DTP3, MCV1, Pol3	Central Statistics Agency (Ethiopia). Ethiopia National Fertility and Family Survey 1990-1991. Addis Ababa, Ethiopia: Central Statistics Agency (Ethiopia).	Survey estimates include implausibly high coverage rates of 100%. Survey removed from analysis.
258111	Ethiopia	Ethiopia Health and Health Related Indicators 2002-2003	2002-2003	all	BCG, DTP3, MCV1	Ministry of Health (Ethiopia). Ethiopia Health and Health Related Indicators 2002-2003. Addis Ababa, Ethiopia: Ministry of Health (Ethiopia), 2004.	Estimates considered implausible. Survey estimates are systematically high compared to administrative data and estimates from other established surveys series (Ethiopia Demographic and Health Survey 2000, Ethiopia Demographic and Health Survey 2005). Survey removed from analysis.
22259	Ethiopia	Ethiopia Health and Health Related Indicators 2003-2004	2003-2004	all	BCG, DTP3, MCV1	Ministry of Health (Ethiopia). Ethiopia Health and Health Related Indicators 2003-2004. Addis Ababa, Ethiopia: Ministry of Health (Ethiopia), 2004.	Estimates considered implausible. Survey estimates are systematically high compared to administrative data and estimates from other established surveys series (Ethiopia Demographic and Health Survey 2005). Survey removed from analysis.

GHDx ID	Country	Series	Year(s)	Cohort	Vaccine(s)	Citation	Rationale for exclusion
258113	Ethiopia	Ethiopia Health and Health Related Indicators 2004-2005	2004-2005	all	BCG, DTP3, MCV1	Ministry of Health (Ethiopia). Ethiopia Health and Health Related Indicators 2004-2005. Addis Ababa, Ethiopia: Ministry of Health (Ethiopia), 2005.	Estimates considered implausible. Survey estimates are systematically high compared to administrative data and estimates from other established surveys series (Ethiopia Demographic and Health Survey 2005). Estimates removed from analysis.
258115	Ethiopia	Ethiopia Health and Health Related Indicators 2005-2006	2005-2006	all	BCG, DTP3, MCV1, Pol3	Ministry of Health (Ethiopia). Ethiopia Health and Health Related Indicators 2005-2006. Addis Ababa, Ethiopia: Ministry of Health (Ethiopia), 2006.	Estimates considered implausible. Survey estimates are systematically high compared to administrative data and estimates from other established surveys series (Ethiopia Demographic and Health Survey 2005). Survey removed from analysis.
22261	Ethiopia	Ethiopia Health and Health Related Indicators 2006-2007	2006-2007	all	DTP3, MCV1	Ministry of Health (Ethiopia). Ethiopia Health and Health Related Indicators 2006-2007. Addis Ababa, Ethiopia: Ministry of Health (Ethiopia), 2007.	Estimates considered implausible. Survey estimates are systematically high compared to administrative data and estimates from other established surveys series (Ethiopia Demographic and Health Survey 2005). Survey removed from analysis.
258117	Ethiopia	Ethiopia Health and Health Related Indicators 2008-2009	2008-2009	all	DTP3, HepB3, PCV3, MCV1	Ministry of Health (Ethiopia). Ethiopia Health and Health Related Indicators 2008-2009. Addis Ababa, Ethiopia: Ministry of Health (Ethiopia).	Estimates considered implausible. Survey estimates are systematically high compared to administrative data and estimates from other established surveys series (Ethiopia Demographic and Health Survey 2010-2011). Survey removed from analysis.
152618	Ethiopia	Ethiopia Health and Health Related Indicators 2009-2010	2009-2010	all	DTP1, DTP3, HepB3, PCV3, MCV1	Ministry of Health (Ethiopia). Ethiopia Health and Health Related Indicators 2009-2010. Addis Ababa, Ethiopia: Ministry of Health (Ethiopia).	Estimates considered implausible. Survey estimates are systematically high compared to administrative data and estimates from other established surveys series (Ethiopia Demographic and Health Survey 2010-2011). Survey removed from analysis.
21301	Ethiopia	Ethiopia Demographic and Health Survey 2010-2011	2010-2011	select	HepB3, PCV3	Central Statistics Agency (Ethiopia), ICF Macro, Ministry of Health (Ethiopia). Ethiopia Demographic and Health Survey 2010-2011. Fairfax, United States of America: ICF International.	Survey precedes introduction of Hepb3 and PCV3 to national immunization schedule. Hepb3 and PCV3 removed from analysis.
258127	Ethiopia	Ethiopia Health and Health Related Indicators 2010-2011	2010-2011	all	DTP3, HepB3, PCV3, MCV1	Ministry of Health (Ethiopia). Ethiopia Health and Health Related Indicators 2010-2011. Addis Ababa, Ethiopia: Ministry of Health (Ethiopia), 2012.	Estimates considered implausible. Survey estimates are systematically high compared to administrative data and estimates from other established surveys series (Ethiopia Demographic and Health Survey 2010-2011). Survey removed from analysis.

GHDx ID	Country	Series	Year(s)	Cohort	Vaccine(s)	Citation	Rationale for exclusion
76706	Gabon	Gabon Demographic and Health Survey 2012	2012	select	Hib3	General Directorate of Statistics (Gabon), ICF International, Ministry of Economy, Employment and Sustainable Development (Gabon), Ministry of Health and Social Affairs (Gabon). Gabon Demographic and Health Survey 2012. Fairfax, United States of America: ICF International, 2013.	Survey precedes introduction of Hib3 to national immunization schedule. Hib3 removed from analysis.
19579*	Gabon	Gabon Demographic and Health Survey 2000-2001	2000-2001	all	HepB3, Hib3	General Directorate of Statistics and Economic Studies (Gabon), Macro International, Inc. Gabon Demographic and Health Survey 2000-2001. Fairfax, United States of America: ICF International.	Survey precedes introduction of HepB3 and Hib3 to national immunization schedule. HepB3 and Hib3 removed from analysis. In addition, selected cohorts excluded from administrative data bias modeling as a methodological decision to improve model performance
19579*	Gabon	Gabon Demographic and Health Survey 2000-2001	2000-2001	select	MCV1	General Directorate of Statistics and Economic Studies (Gabon), Macro International, Inc. Gabon Demographic and Health Survey 2000-2001. Fairfax, United States of America: ICF International.	Selected cohorts excluded from administrative data bias modeling as a methodological decision to improve model performance
226576*	Georgia	Georgia EPI Coverage Survey with add on Questions 1996	1996	select	BCG1	Ministry of Health (Georgia), United Nations Children's Fund (UNICEF). Georgia EPI Coverage Survey with add on Questions 1996.	Selected cohorts excluded from administrative data bias modeling as a methodological decision to improve model performance
3970	Georgia	Georgia Multiple Indicator Cluster Survey 2005	2005	all	BCG, DTP1, DTP3, MCV1, Pol3	National Center for Disease Control (Georgia), State Department of Statistics of Georgia, United Nations Children's Fund (UNICEF). Georgia Multiple Indicator Cluster Survey 2005. New York, United States of America: United Nations Children's Fund (UNICEF).	Estimates considered implausible. Survey estimates are systematically low compared to administrative data and estimates from other established surveys series. Survey removed from analysis.
19587*	Ghana	Ghana Demographic and Health Survey 1988	1988	select	DTP1, DTP3, MCV1, Pol3	Ghana Statistical Service, Macro Systems, Inc.; Institute for Resource Development. Ghana Demographic and Health Survey 1988. Fairfax, United States of America: ICF International.	Selected cohorts excluded from administrative data bias modeling as a methodological decision to improve model performance

GHDx ID	Country	Series	Year(s)	Cohort	Vaccine(s)	Citation	Rationale for exclusion
21622	Ghana	Ghana World Health Survey 2003	2003	all	DTP1, DTP3, MCV1	World Health Organization (WHO). Ghana World Health Survey 2003. Geneva, Switzerland: World Health Organization (WHO), 2005.	Estimates considered implausible. Survey estimates are inconsistent with administrative data and estimates from other established survey series (Ghana Demographic and Health Survey 2003). Survey removed from analysis.
4694	Ghana	Ghana Multiple Indicator Cluster Survey 2006	2006	all	RCV1	Ministry of Health (MOH) (Ghana), Ghana Statistical Service and United Nations Children's Fund (UNICEF). Ghana Multiple Indicator Cluster Survey 2006. New York, United States: United Nations Children's Fund (UNICEF).	Survey precedes introduction of RCV1 to national immunization schedule. RCV1 removed from analysis.
157027	Ghana	Ghana Demographic and Health Survey 2014	2014	select	MCV2, PCV3, RotaC	Ghana Health Service, Ghana Statistical Service, ICF International. Ghana Demographic and Health Survey 2014. Fairfax, United States of America: ICF International, 2016.	Older age-cohorts precede introduction of MCV2, PCV3, and RotaC to national immunization schedule. Select age-cohorts removed from analysis for MCV2, PCV3, and RotaC.
160576	Ghana	Ghana District Multiple Indicator Cluster Survey 2007-2008	2007-2008	all	RCV1	Ghana Statistical Service, Ministry of Health (Ghana), United Nations Children's Fund (UNICEF). Ghana District Multiple Indicator Cluster Survey 2007-2008.	Survey precedes introduction of RCV1 to national immunization schedule. RCV1 removed from analysis.
236205	Ghana	Ghana Socioeconomic Panel Survey 2009-2010	2009-2010	all	DTP1, DTP3	Economic Growth Center, Yale University, Institute of Statistical, Social and Economic Research, University of Ghana. Ghana Socioeconomic Panel Survey 2009-2010. Washington DC, United States: World Bank.	DTP3 estimates are systematically low compared to administrative data and estimates from other established surveys series (Ghana Multiple Indicator Cluster Survey 2011). DTP3 removed from analysis.
165101	Ghana	Ghana Living Standards Survey 2012-2013	2012-2013	all	BCG, DTP1, DTP3, HepB3, PCV3, MCV1, Pol3	Ghana Statistical Service, World Bank. Ghana Living Standards Survey 2012-2013. Accra, Ghana: Ghana Statistical Service.	Estimates considered implausible. Survey estimates are systematically low compared to administrative data and estimates from other established surveys series (Ghana Multiple Indicator Cluster Survey 2011). Survey removed from analysis.
317973	Ghana	Ghana Health Service Annual Report 2016	2014-2016	all	BCG, DTP3, HepB3, PCV3, MCV1, MCV2, PCV3, Pol3, RCV1	Ghana Health Service. Ghana Health Service Annual Report 2016. Accra, Ghana: Ghana Health Service, 2017.	Survey estimates include implausibly high coverage rates of 100% for multiple vaccines. These vaccines removed from analysis.

GHDx ID	Country	Series	Year(s)	Cohort	Vaccine(s)	Citation	Rationale for exclusion
19647*	Guatemala	Guatemala Demographic and Health Survey 1987	1987	all	MCV1	Institute of Nutrition of Central America and Panama, Westinghouse; Institute for Resource Development. Guatemala Demographic and Health Survey 1987. Fairfax, United States of America: ICF International.	MCV1 estimates are systematically low compared to administrative data and estimates from other established surveys series (Guatemala Demographic and Health Survey 1995). MCV1 removed from analysis. In addition, selected cohorts excluded from administrative data bias modeling as a methodological decision to improve model performance
19647*	Guatemala	Guatemala Demographic and Health Survey 1987	1987	select	BCG1, DTP1, DTP3, Pol3	Institute of Nutrition of Central America and Panama, Westinghouse; Institute for Resource Development. Guatemala Demographic and Health Survey 1987. Fairfax, United States of America: ICF International.	Selected cohorts excluded from administrative data bias modeling as a methodological decision to improve model performance
32127	Guatemala	Guatemala National Survey of Living Conditions 2006	2006	all	HepB3, Hib3	National Institute of Statistics (Guatemala), National Statistical System (Guatemala), World Bank (WB), United Nations Development Programme (UNDP), United Nations Economic Commission for Latin America and the Caribbean (CEPAL), Rafael Landívar University, United Nations Population Fund (UNFPA), Norwegian Agency for Development (NORAD), Swedish International Development Agency (SIDA), Secretary of Planning and Programming (SEGEPLAN) (Guatemala), Bank of Guatemala. Guatemala National Survey of Living Conditions 2006. Guatemala City, Guatemala: National Statistics Institute (Guatemala).	Survey precedes introduction of HepB3 and Hib3 to national immunization schedule. HepB3 and Hib3 removed from analysis.

GHDx ID	Country	Series	Year(s)	Cohort	Vaccine(s)	Citation	Rationale for exclusion
157031	Guatemala	Guatemala Demographic and Health Survey 2014-2015	2014-2015	select	MCV1, PCV3	ICF International, Institute of Nutrition of Central America and Panama, Ministry of Public Health and Social Assistance (Guatemala), National Statistics Institute (Guatemala), Secretary of Planning and Programming of the Presidency (Segeplan) (Guatemala). Guatemala Demographic and Health Survey 2014-2015. Fairfax, United States of America: ICF International, 2017.	MCV1 estimates are systematically high compared to administrative data and estimates from other established surveys series. Older age-cohorts precede introduction of PCV3 to national immunization schedule. MCV1 and select age-cohorts of PCV3 removed from analysis.
141983	Guinea	Guinea National Survey on Nutritional Status and Tracking Key Indicators of Child Survival 2007-2008	2007-2008	all	HepB3	National Institute of Statistics (Guinea). Guinea National Survey on Nutritional Status and Tracking Key Indicators of Child Survival 2007-2008.	Survey precedes introduction of HepB3 to national immunization schedule. HepB3 removed from analysis.
233671*	Guinea-Bissau	Guinea-Bissau EPI Review 1988	1988	select	DTP1, DTP3, Pol3	Guinea-Bissau EPI Review 1988.	Selected cohorts excluded from administrative data bias modeling as a methodological decision to improve model performance
233666*	Guinea-Bissau	Guinea-Bissau EPI Review 1994	1994	select	DTP3	Ministry of Public Health (Guinea-Bissau). Guinea-Bissau EPI Review 1994.	Selected cohorts excluded from administrative data bias modeling as a methodological decision to improve model performance
4808	Guinea-Bissau	Guinea-Bissau Multiple Indicator Cluster Survey 2000	2000	select	DTP1, DTP3, MCV1	Secretary State of Planning, National Institute of Statistics and Census (INEC), United Nations Children's Fund (UNICEF). Guinea-Bissau Multiple Indicator Cluster Survey 2000. New York, United States: United Nations Children's Fund (UNICEF).	MCV1 estimates do not follow a reasonable age structure. Older children have significantly higher coverage than reported in either administrative data or other survey series, suggesting survey is capturing results of catch-up immunization. Select DTP3 age-cohorts are also removed from analysis due to implausibly low coverage compared to administrative data and estimates from other established survey series (Guinea-Bissau Multiple Indicator Cluster Survey 2006).
4818*	Guinea-Bissau	Guinea-Bissau Multiple Indicator Cluster Survey 2006	2006	select	MCV1	United Nations Children's Fund (UNICEF), Government of Guinea-Bissau. Guinea-Bissau Multiple Indicator Cluster Survey 2006. New York, United States: United Nations Children's Fund (UNICEF).	Selected cohorts excluded from administrative data bias modeling as a methodological decision to improve model performance

GHDx ID	Country	Series	Year(s)	Cohort	Vaccine(s)	Citation	Rationale for exclusion
502015*	Guinea-Bissau	Guinea-Bissau Expanded Program on Immunization Vaccination Coverage Survey 2017	2017	select	BCG1, DTP1, DTP3, MCV1, Pol3	Ministry of Public Health (Guinea-Bissau), National Statistics Institute (Guinea-Bissau), World Health Organization (WHO). Guinea-Bissau Expanded Program on Immunization Vaccination Coverage Survey 2017.	Selected cohorts excluded from administrative data bias modeling as a methodological decision to improve model performance
457894*	Guinea-Bissau	Guinea-Bissau Multiple Indicator Cluster Survey 2018-2019	2018-2019	all	RCV1	National Statistics Institute (Guinea-Bissau), United Nations Children's Fund (UNICEF). Guinea-Bissau Multiple Indicator Cluster Survey 2018-2019. New York, United States of America: United Nations Children's Fund (UNICEF), 2020.	Survey precedes introduction of RCV1 to national immunization schedule. RCV1 removed from analysis. In addition, selected cohorts excluded from administrative data bias modeling as a methodological decision to improve model performance
457894*	Guinea-Bissau	Guinea-Bissau Multiple Indicator Cluster Survey 2018-2019	2018-2019	select	BCG1, DTP1, DTP3, MCV1, Pol3	National Statistics Institute (Guinea-Bissau), United Nations Children's Fund (UNICEF). Guinea-Bissau Multiple Indicator Cluster Survey 2018-2019. New York, United States of America: United Nations Children's Fund (UNICEF), 2020.	Selected cohorts excluded from administrative data bias modeling as a methodological decision to improve model performance
21348	Guyana	Guyana Demographic and Health Survey 2009	2009	all	MCV2	Bureau of Statistics (Guyana), ICF Macro, Ministry of Health (Guyana). Guyana Demographic and Health Survey 2009. Fairfax, United States of America: ICF International, 2011.	MCV2 estimates are systematically low compared to administrative data and estimates from other established surveys series (Guyana Multiple Indicator Cluster Survey 2006-2007). MCV2 removed from analysis.
474221	Guyana	Guyana Multiple Indicator Cluster Survey 2019-2020	2019-2020	all	Pol3	Bureau of Statistics (Guyana), Ministry of Health (Guyana), United Nations Children's Fund (UNICEF). Guyana Multiple Indicator Cluster Survey 2019-2020. New York, United States of America: United Nations Children's Fund (UNICEF), 2021.	Survey reports higher Polio2 coverage than Polio1 coverage. Pol3 removed from analysis due to reliability concerns.
19708*	Haiti	Haiti Demographic and Health Survey 2000	2000	select	MCV1	Haitian Institute of Childhood (IHE), Macro International, Inc. Haiti Demographic and Health Survey 2000. Fairfax, United States of America: ICF International.	Selected cohorts excluded from administrative data bias modeling as a methodological decision to improve model performance
26680*	Haiti	Haiti Global Fund Household Survey 2008	2008	select	BCG1	Global Fund to Fight Aids Tuberculosis and Malaria (GFATM). Haiti Global Fund Household Survey 2008.	Selected cohorts excluded from administrative data bias modeling as a methodological decision to improve model performance

GHDx ID	Country	Series	Year(s)	Cohort	Vaccine(s)	Citation	Rationale for exclusion
19695*	Haiti	Haiti Demographic and Health Survey 1994-1995	1994-1995	select	MCV1	Haitian Institute of Childhood (IHE), Macro International, Inc. Haiti Demographic and Health Survey 1994-1995. Calverton, United States of America: Macro International, Inc.	Selected cohorts excluded from administrative data bias modeling as a methodological decision to improve model performance
19720*	Haiti	Haiti Demographic and Health Survey 2005-2006	2005-2006	select	MCV1	Haitian Institute of Childhood (IHE), Haitian Institute of Statistics and Informatics, Macro International, Inc. Haiti Demographic and Health Survey 2005-2006. Fairfax, United States of America: ICF International.	Selected cohorts excluded from administrative data bias modeling as a methodological decision to improve model performance
27542	Honduras	Honduras Reproductive Health Survey 1996	1996	all	MCV2	Ministry of Health (Honduras), Honduras Family Planning Association (ASHONPLAFA) and Division of Reproductive Health-Centers for Disease Control and Prevention (CDC). (1997) Honduras Reproductive Health Survey 1996. Tegucigalpa, Honduras: ASHONPLAFA.	Survey precedes introduction of MCV2 to national immunization schedule. MCV2 removed from analysis.
5009	Honduras	Honduras Survey of Living Conditions 2004	2004	all	BCG, DTP1, DTP3, MCV1, MCV2, Pol3, RCV1	National Institute of Statistics (Honduras). Honduras Survey of Living Conditions 2004. Tegucigalpa, Honduras: National Institute of Statistics (Honduras).	Estimates considered implausible. Survey estimates are inconsistent with administrative data and estimates from other established survey series (Honduras Demographic and Health Survey 2005-2006). Survey removed from analysis.
490966	Honduras	Honduras Multiple Indicator Cluster Survey 2019	2019	all	MCV2	European Union (EU), Government of Canada, Government of Honduras, National Institute of Statistics (Honduras), Secretary of Health (Honduras), United Nations Children's Fund (UNICEF), United Nations Population Fund (UNFPA). Honduras Multiple Indicator Cluster Survey 2019. New York, United States of America: United Nations Children's Fund (UNICEF), 2021.	Survey precedes introduction of MCV2 to national immunization schedule. MCV2 removed from analysis.

GHDx ID	Country	Series	Year(s)	Cohort	Vaccine(s)	Citation	Rationale for exclusion
95440	Honduras	Honduras Demographic and Health Survey 2011-2012	2011-2012	select	PCV3, RotaC	ICF Macro, National Institute of Statistics (Honduras). Honduras Demographic and Health Survey 2011-2012. Fairfax, United States of America: ICF International.	Older age-cohorts precede introduction of PCV3 and RotaC to national immunization schedule. Select age-cohorts removed from analysis for PCV3 and RotaC.
65220	India	India Concurrent Evaluation of National Rural Health Mission 2009	2009	all	BCG, DTP1, DTP3, MCV1, Pol3	International Institute for Population Sciences (India), Ministry of Health and Family Welfare (India). India Concurrent Evaluation of National Rural Health Mission 2009.	Estimates considered implausible. Survey estimates are systematically high compared to administrative data and estimates from other established surveys series (India Demographic and Health Survey 2005-2006). Survey removed from analysis.
60398	India	India Family Welfare Statistics 2011	1980-2011	all	BCG, DTP3, MCV1, Pol3	Ministry of Health and Family Welfare (India). India Family Welfare Statistics 2011. New Delhi, India: Ministry of Health and Family Welfare (India), 2011.	Estimates considered implausible. Survey estimates are systematically high compared to administrative data and estimates from other established surveys series (India Demographic and Health Survey 2005-2006). Survey removed from analysis.
5243	India	India National Sample Survey Round 52 1995-1996	1995-1996	all	BCG, DTP3, MCV1, Pol3	Ministry of Statistics and Programme Implementation (India). India National Sample Survey Round 52 1995-1996. New Delhi, India: Ministry of Statistics and Programme Implementation (India).	Estimates considered implausible. Survey estimates are systematically high compared to administrative data and estimates from other established surveys series (India Demographic and Health Survey 1992-1993). Survey removed from analysis.
26919	India	India Human Development Survey 2004-2005	2004-2005	all	BCG, DTP1, DTP3, MCV1, Pol3	Desai, Sonalde, Reeve Vanneman, and National Council of Applied Economic Research, New Delhi. India Human Development Survey (IHDS), 2005. ICPSR22626-v8. Ann Arbor, MI: Inter-university Consortium for Political and Social Research [distributor], 2010-06-29. doi:10.3886/ICPSR22626.v8.	Estimates considered implausible. Survey estimates are systematically low compared to administrative data and estimates from other established surveys series (India Demographic and Health Survey 2005-2006). Survey removed from analysis.
23258	India	India District Level Household Survey 2007-2008	2007-2008	all	BCG, DTP1, DTP3, MCV1, Pol3	International Institute for Population Sciences (India). India District Level Household Survey 2007-2008. Mumbai, India: International Institute for Population Sciences (India), 2010.	Estimates considered implausible. Survey estimates are systematically high compared to administrative data and estimates from other established surveys series (India Demographic and Health Survey 2005-2006). Survey removed from analysis.
65181	India	India Coverage Evaluation Survey 2009-2010	2009-2010	all	BCG, DTP1, DTP3, HepB3, MCV1, Pol3, RCV1	Ministry of Health and Family Welfare (India), ORG Centre for Social Research (ORG CSR), United Nations Children's Fund (UNICEF). India Coverage Evaluation Survey 2009-2010.	Estimates considered implausible. Survey estimates are systematically high compared to administrative data and estimates from other established surveys series (India Demographic and Health Survey 2005-2006). Survey removed from analysis.

GHDx ID	Country	Series	Year(s)	Cohort	Vaccine(s)	Citation	Rationale for exclusion
165390	India	India District Level Household Survey 2012-2014	2012-2014	all	BCG, DTP1, DTP3, MCV1, Pol3	International Institute for Population Sciences (India). India District Level Household Survey 2012-2014. New Delhi, India: Ministry of Health and Family Welfare (India).	Estimates are systematically high compared to administrative data and estimates from other established surveys series. These vaccines removed from analysis.
6614	Indonesia	Indonesia National Socioeconomic Survey 1992	1992	all	DTP1, DTP3, Pol3	Central Bureau of Statistics (Indonesia). Indonesia National Socioeconomic Survey 1992.	Estimates considered implausible. Survey estimates are inconsistent with administrative data and estimates from other established survey series (Indonesia Demographic and Health Survey 1994). Survey removed from analysis.
6638	Indonesia	Indonesia National Socioeconomic Survey 1993	1993	all	DTP1, DTP3, Pol3	Central Bureau of Statistics (Indonesia). Indonesia National Socioeconomic Survey 1993.	Estimates considered implausible. Survey estimates are inconsistent with administrative data and estimates from other established survey series (Indonesia Demographic and Health Survey 1994). Survey removed from analysis.
6656	Indonesia	Indonesia National Socioeconomic Survey 1994	1994	all	DTP1, DTP3, Pol3	Central Bureau of Statistics (Indonesia). Indonesia National Socioeconomic Survey 1994.	Estimates considered implausible. Survey estimates are inconsistent with administrative data and estimates from other established survey series (Indonesia Demographic and Health Survey 1994). Survey removed from analysis.
6687	Indonesia	Indonesia National Socioeconomic Survey 1995	1995	all	DTP1, DTP3, Pol3	Central Bureau of Statistics (Indonesia), Ministry of Health (Indonesia), United Nations Children's Fund (UNICEF). Indonesia National Socioeconomic Survey 1995.	Estimates considered implausible. Survey estimates are inconsistent with administrative data and estimates from other established survey series (Indonesia Demographic and Health Survey 1994). Survey removed from analysis.
6706	Indonesia	Indonesia National Socioeconomic Survey 1996	1996	all	DTP1, DTP3, Pol3	Central Bureau of Statistics (Indonesia), Ministry of Health (Indonesia), United Nations Children's Fund (UNICEF). Indonesia National Socioeconomic Survey 1996.	Estimates considered implausible. Survey estimates are inconsistent with administrative data and estimates from other established survey series (Indonesia Demographic and Health Survey 1994, Indonesia Demographic and Health Survey 1997). Survey removed from analysis.
5827	Indonesia	Indonesia Family Life Survey 1997	1997	all	BCG, DTP1, DTP3, HepB3, MCV1, Pol3	Macro International, Inc, RAND Corporation, University of California, Los Angeles (UCLA), University of Indonesia. Indonesia Family Life Survey 1997.	Estimates considered implausible. Survey estimates are inconsistent with administrative data and estimates from other established survey series (Indonesia Demographic and Health Survey 1994, Indonesia Demographic and Health Survey 1997). Survey removed from analysis.
6724	Indonesia	Indonesia National Socioeconomic Survey 1997	1997	all	DTP1, DTP3, Pol3	Central Bureau of Statistics (Indonesia), Ministry of Health (Indonesia), United Nations Children's Fund (UNICEF). Indonesia National Socioeconomic Survey 1997.	Estimates considered implausible. Survey estimates are inconsistent with administrative data and estimates from other established survey series (Indonesia Demographic and Health Survey 1994, Indonesia Demographic and Health Survey 1997). Survey removed from analysis.

GHDx ID	Country	Series	Year(s)	Cohort	Vaccine(s)	Citation	Rationale for exclusion
6767	Indonesia	Indonesia National Socioeconomic Survey 1998	1998	all	DTP1, DTP3, MCV1, MCV2, Pol3	Central Bureau of Statistics (Indonesia), Ministry of Health (Indonesia), World Bank. Indonesia National Socioeconomic Survey 1998.	Estimates considered implausible. Survey estimates are inconsistent with administrative data and estimates from other established survey series (Indonesia Demographic and Health Survey 1997). Survey removed from analysis.
6790	Indonesia	Indonesia National Socioeconomic Survey 1999	1999	all	DTP1, DTP3, MCV1, MCV2, Pol3	Central Bureau of Statistics (Indonesia), Ministry of Health (Indonesia), World Bank. Indonesia National Socioeconomic Survey 1999.	Estimates considered implausible. Survey estimates are inconsistent with administrative data and estimates from other established survey series (Indonesia Demographic and Health Survey 1997, Indonesia Demographic and Health Survey 2002-2003). Survey removed from analysis.
6111	Indonesia	Indonesia Family Life Survey 2000	2000	all	BCG, DTP1, DTP3, HepB3, MCV1, Pol3	Center for Population and Policy Studies, Gadjah Mada University (Indonesia), RAND Corporation. Indonesia Family Life Survey 2000. Santa Monica, United States of America: RAND Corporation.	Survey estimates include implausibly high coverage rates of 100%. Survey removed from analysis.
6842	Indonesia	Indonesia National Socioeconomic Survey 2001	2001	all	MCV1	Central Bureau of Statistics (Indonesia), Ministry of Health (Indonesia), World Bank. Indonesia National Socioeconomic Survey 2001.	Estimates considered implausible. Survey estimates are inconsistent with administrative data and estimates from other established survey series (Indonesia Demographic and Health Survey 1997, Indonesia Demographic and Health Survey 2002-2003). Survey removed from analysis.
6904	Indonesia	Indonesia National Socioeconomic Survey 2004	2004	all	DTP1, DTP3, HepB3, MCV1, MCV2, Pol3	Statistics Indonesia. Indonesia National Socioeconomic Survey 2004.	Estimates considered implausible. Survey estimates are inconsistent with administrative data and estimates from other established survey series (Indonesia Demographic and Health Survey 2002-2003). Survey removed from analysis.
5376	Indonesia	Indonesia National Socioeconomic Survey 2005	2005	all	DTP1, DTP3, HepB3, MCV1, MCV2, Pol3	Statistics Indonesia. Indonesia National Socioeconomic Survey 2005.	Estimates considered implausible. Survey estimates are inconsistent with administrative data and estimates from other established survey series (Indonesia Demographic and Health Survey 2007). Survey removed from analysis.
44865	Indonesia	Indonesia National Socioeconomic Survey - Poverty Program Evaluation 2006	2006	all	DTP1, DTP3, HepB3, MCV1, MCV2, Pol3	Statistics Indonesia. Indonesia National Socioeconomic Survey - Poverty Program Evaluation 2006. Jakarta, Indonesia: Statistics Indonesia.	Estimates considered implausible. Survey estimates are inconsistent with administrative data and estimates from other established survey series (Indonesia Demographic and Health Survey 2007). Survey removed from analysis.
5401	Indonesia	Indonesia National Socioeconomic Survey 2006	2006	all	DTP1, DTP3, HepB3, MCV1, MCV2, Pol3	Statistics Indonesia. Indonesia National Socioeconomic Survey 2006.	Estimates considered implausible. Survey estimates are inconsistent with administrative data and estimates from other established survey series (Indonesia Demographic and Health Survey 2007). Survey removed from analysis.

GHDx ID	Country	Series	Year(s)	Cohort	Vaccine(s)	Citation	Rationale for exclusion
6970	Indonesia	Indonesia National Socioeconomic Survey 2007	2007	all	DTP1, DTP3, HepB3, MCV1, MCV2, Pol3	Statistics Indonesia. Indonesia National Socioeconomic Survey 2007.	Estimates considered implausible. Survey estimates are inconsistent with administrative data and estimates from other established survey series (Indonesia Demographic and Health Survey 2002-2003, Indonesia Demographic and Health Survey 2007). Survey removed from analysis.
43526	Indonesia	Indonesia National Socioeconomic Survey 2008	2008	all	DTP1, DTP3, HepB3, MCV1, MCV2, Pol3	Statistics Indonesia. Indonesia National Socioeconomic Survey 2008.	Estimates considered implausible. Survey estimates are inconsistent with administrative data and estimates from other established survey series (Indonesia Demographic and Health Survey 2007, Indonesia Demographic and Health Survey 2012). Survey removed from analysis.
43552	Indonesia	Indonesia National Socioeconomic Survey 2009	2009	all	DTP1, DTP3, HepB3, MCV1, MCV2, Pol3	Statistics Indonesia. Indonesia National Socioeconomic Survey 2009.	Estimates considered implausible. Survey estimates are inconsistent with administrative data and estimates from other established survey series (Indonesia Demographic and Health Survey 2007, Indonesia Demographic and Health Survey 2012). Survey removed from analysis.
30235	Indonesia	Indonesia National Socioeconomic Survey 2010	2010	all	DTP1, DTP3, HepB3, MCV1, MCV2, Pol3	Statistics Indonesia. Indonesia National Socioeconomic Survey 2010.	Estimates considered implausible. Survey estimates are inconsistent with administrative data and estimates from other established survey series (Indonesia Demographic and Health Survey 2007, Indonesia Demographic and Health Survey 2012). Survey removed from analysis.
85265	Indonesia	Indonesia National Socioeconomic Survey 2011	2011	all	DTP1, DTP3, HepB3, MCV1, Pol3	Statistics Indonesia. Indonesia National Socioeconomic Survey 2011.	Estimates considered implausible. Survey estimates are inconsistent with administrative data and estimates from other established survey series (Indonesia Demographic and Health Survey 2007, Indonesia Demographic and Health Survey 2012). Survey removed from analysis.
219201	Indonesia	Indonesia Family Life Survey East 2012	2012	all	BCG, DTP1, DTP3, HepB3, MCV1, Pol3	National Team for the Acceleration of Poverty Reduction (TNP2K) (Indonesia), SurveyMETER, University of Southern California, World Bank. Indonesia Family Life Survey East 2012.	Estimates considered implausible. Survey estimates do not follow a reasonable age structure. Older children have significantly higher coverage than reported in either administrative data or other survey series, suggesting survey is capturing results of catch-up immunization. Survey removed from analysis.
150884	Indonesia	Indonesia National Socioeconomic Survey 2012	2012	all	DTP1, DTP3, HepB3, MCV1, Pol3	Central Bureau of Statistics (Indonesia). Indonesia National Socioeconomic Survey 2012. Jakarta, Indonesia: Central Bureau of Statistics (Indonesia).	Estimates considered implausible. Survey estimates do not follow a reasonable age structure. Older children have significantly higher coverage than reported in either administrative data or other survey series, suggesting survey is capturing results of catch-up immunization. Survey removed from analysis.

GHDx ID	Country	Series	Year(s)	Cohort	Vaccine(s)	Citation	Rationale for exclusion
151184	Indonesia	Indonesia National Socioeconomic Survey 2013	2013	all	DTP1, DTP3, HepB3, MCV1, MCV2, Pol3	Statistics Indonesia. Indonesia National Socioeconomic Survey 2013. Jakarta, Indonesia: Statistics Indonesia.	Estimates considered implausible. Survey estimates are inconsistent with administrative data and estimates from other established survey series (Indonesia Demographic and Health Survey 2012). Survey removed from analysis.
395694	Indonesia	Indonesia National Socioeconomic Survey 2017	2017	all	RCV1	Central Bureau of Statistics (Indonesia). Indonesia National Socioeconomic Survey 2017. Jakarta, Indonesia: Central Bureau of Statistics (Indonesia), 2018.	Survey precedes introduction of RCV1 to national immunization schedule. RCV1 removed from analysis.
414283	Indonesia	Indonesia Special Demographic and Health Survey 2017	2017	all	BCG, DTP1, DTP3, HepB3, MCV1, Pol3	ICF International, Ministry of Health (Indonesia), National Population and Family Planning Board (Indonesia), Statistics Indonesia. Indonesia Special Demographic and Health Survey 2017. Fairfax, United States of America: ICF International, 2019.	Survey removed from analysis due to small sample size and reliability concerns.
404228	Indonesia	Indonesia Basic Health Research 2018	2018	all	BCG, DTP1, DTP3, HepB3, PCV3, MCV1, Pol3	Agency of Health Research and Development (Indonesia). Indonesia Basic Health Research 2018.	Estimates considered implausible. Survey estimates are inconsistent with administrative data and estimates from other established survey series (Indonesia Demographic and Health Survey 2017). Survey removed from analysis.
354843	Indonesia	Indonesia Health Profile 2016	1991-2016	all	BCG, DTP1, DTP3, HepB3, PCV3, MCV1, Pol3	Ministry of Health (Indonesia). Indonesia Health Profile 2016. Jakarta, Indonesia: Ministry of Health (Indonesia), 2017.	Estimates considered implausible. Survey estimates are systematically high compared to administrative data and estimates from other established surveys series (Indonesia Demographic and Health Survey 1994, Indonesia Demographic and Health Survey 1997, Indonesia Demographic and Health Survey 2002-2003, Indonesia Demographic and Health Survey 2007, Indonesia Demographic and Health Survey 2012). Survey removed from analysis.
81416	Iran	Iran Multiple Indicator Demographic and Health Survey 2010	2010	select	MCV2	Ministry of Health and Medical Education (Iran), Statistical Centre of Iran. Iran Multiple Indicator Demographic and Health Survey 2010.	Oldest age-cohort for MCV2 removed from analysis due to implausible drop in coverage.
338778	Iran	Iran Communicable Diseases and Vaccination Coverage Surveillance 2006-2010	2006-2010	all	BCG, DTP3, HepB3, MCV1, Pol3, RCV1	Ministry of Health and Medical Education (Iran). Iran Communicable Diseases and Vaccination Coverage Surveillance 2006-2010.	Survey estimates include implausibly high coverage rates of 100%. Survey removed from analysis.

GHDx ID	Country	Series	Year(s)	Cohort	Vaccine(s)	Citation	Rationale for exclusion
385708	Iraq	Iraq Multiple Indicator Cluster Survey 2018	2018	select	PCV3	Central Statistical Organization (Iraq), United Nations Children's Fund (UNICEF). Iraq Multiple Indicator Cluster Survey 2018. New York, United States of America: United Nations Children's Fund (UNICEF), 2019.	Older age-cohorts precede introduction of PCV3 to national immunization schedule. Select age-cohorts removed from analysis for PCV3.
7248	Jamaica	Jamaica Survey of Living Conditions 1991	1991	all	BCG, DTP1, DTP3, MCV1, Pol3	Planning Institute of Jamaica, Statistical Institute of Jamaica. Jamaica Survey of Living Conditions 1991.	Estimates considered implausible. Survey estimates are systematically high compared to administrative data and estimates from other established surveys series (Jamaica Multiple Indicator Cluster Survey 2005). Survey removed from analysis.
45779	Jamaica	Jamaica Survey of Living Conditions 1993	1993	all	BCG, DTP1, DTP3, MCV1, Pol3	Planning Institute of Jamaica, Statistical Institute of Jamaica. Jamaica Survey of Living Conditions 1993.	Estimates considered implausible. Survey estimates are systematically high compared to administrative data and estimates from other established surveys series (Jamaica Multiple Indicator Cluster Survey 2005). Survey removed from analysis.
45784	Jamaica	Jamaica Survey of Living Conditions 1995	1995	all	BCG, DTP1, DTP3, MCV1, Pol3	Planning Institute of Jamaica, Statistical Institute of Jamaica. Jamaica Survey of Living Conditions 1995.	Estimates considered implausible. Survey estimates are systematically high compared to administrative data and estimates from other established surveys series (Jamaica Multiple Indicator Cluster Survey 2005). Survey removed from analysis.
45786	Jamaica	Jamaica Survey of Living Conditions 1996	1996	all	BCG, DTP1, DTP3, MCV1, Pol3	Planning Institute of Jamaica, Statistical Institute of Jamaica. Jamaica Survey of Living Conditions 1996.	Estimates considered implausible. Survey estimates are systematically high compared to administrative data and estimates from other established surveys series (Jamaica Multiple Indicator Cluster Survey 2005). Survey removed from analysis.
45850	Jamaica	Jamaica Survey of Living Conditions 1997	1997	all	BCG, DTP1, DTP3, MCV1, Pol3	Planning Institute of Jamaica, Statistical Institute of Jamaica. Jamaica Survey of Living Conditions 1997.	Estimates considered implausible. Survey estimates are systematically high compared to administrative data and estimates from other established surveys series (Jamaica Multiple Indicator Cluster Survey 2005). Survey removed from analysis.
45856	Jamaica	Jamaica Survey of Living Conditions 2000	2000	all	BCG, DTP1, DTP3, MCV1, Pol3	Planning Institute of Jamaica, Statistical Institute of Jamaica. Jamaica Survey of Living Conditions 2000.	Estimates considered implausible. Survey estimates are systematically high compared to administrative data and estimates from other established surveys series (Jamaica Multiple Indicator Cluster Survey 2005). Survey removed from analysis.

GHDx ID	Country	Series	Year(s)	Cohort	Vaccine(s)	Citation	Rationale for exclusion
141336	Jamaica	Jamaica Multiple Indicator Cluster Survey 2011	2011	select	HepB3, PCV3, MCV1	Statistical Institute of Jamaica, United Nations Children's Fund (UNICEF). Jamaica Multiple Indicator Cluster Survey 2011. New York, United States of America: United Nations Children's Fund (UNICEF), 2015.	Estimates considered implausible. Survey estimates are systematically low compared to administrative data and estimates from other established surveys series. Survey removed from analysis.
550789	Jamaica	Jamaica Multiple Indicator Cluster Survey 2022	2022	all	MCV2	Inter-American Development Bank (IDB), Planning Institute of Jamaica, Statistical Institute of Jamaica, United Nations Children's Fund (UNICEF). Jamaica Multiple Indicator Cluster Survey 2022. New York, United States of America: United Nations Children's Fund (UNICEF), 2024.	MCV estimates are systematically low compared to administrative data and estimates from other established surveys series. MCV removed from analysis.
7224	Jamaica	Jamaica Survey of Living Conditions 1988	1988-1998	all	BCG, DTP1, DTP3, MCV1, Pol3	Planning Institute of Jamaica, Statistical Institute of Jamaica. Jamaica Survey of Living Conditions 1988. Kingston, Jamaica: Planning Institute of Jamaica.	Estimates considered implausible. Survey estimates are systematically high compared to administrative data and estimates from other established surveys series (Jamaica Multiple Indicator Cluster Survey 2005). Survey removed from analysis.
20060	Jordan	Jordan Demographic and Health Survey 1997	1997	select	MCV2	Department of Statistics (Jordan), Macro International, Inc. Jordan Demographic and Health Survey 1997. Fairfax, United States of America: ICF International.	Older age-cohorts precede introduction of MCV2 to national immunization schedule. Select age-cohorts removed from analysis for MCV2.
20073	Jordan	Jordan Demographic and Health Survey 2002	2002	select	Hib3, RCV1	Department of Statistics (Jordan), Macro International, Inc. Jordan Demographic and Health Survey 2002. Fairfax, United States of America: ICF International.	Older age-cohorts precede introduction of Hib3 and RCV to national immunization schedule. Select age-cohorts removed from analysis for Hib3 and RCV.
560560	Jordan	Jordan Demographic and Health Survey 2023	2023	all	PCV3	Department of Statistics (Jordan), ICF International. Jordan Demographic and Health Survey 2023. Fairfax, United States of America: ICF International, 2024.	Survey precedes introduction of PCV to national immunization schedule. PCV removed from analysis.
20092*	Kazakhstan	Kazakhstan Demographic and Health Survey 1995	1995	select	DTP1, DTP3	Macro International, Inc, National Institute of Nutrition (Kazakhstan). Kazakhstan Demographic and Health Survey 1995. Fairfax, United States of America: ICF International.	Selected cohorts excluded from administrative data bias modeling as a methodological decision to improve model performance

GHDx ID	Country	Series	Year(s)	Cohort	Vaccine(s)	Citation	Rationale for exclusion
20103	Kazakhstan	Kazakhstan Demographic and Health Survey 1999	1999	select	HepB3	Academy of Preventive Medicine (Kazakhstan), Macro International, Inc. Kazakhstan Demographic and Health Survey 1999. Fairfax, United States of America: ICF International.	Survey precedes introduction of HepB3 to national immunization schedule. HepB3 removed from analysis.
7340	Kazakhstan	Kazakhstan Multiple Indicator Cluster Survey 2006	2006	all	BCG	Agency of the Republic of Kazakhstan on Statistics and United Nations Children's Fund (UNICEF). Kazakhstan Multiple Indicator Cluster Survey 2006. New York, United States: United Nations Children's Fund (UNICEF).	BCG coverage rates capture results of catch-up immunization during stockout years, as confirmed by WUENIC historical estimate reports. Estimates not representative of coverage from national routine immunization schedule. BCG removed from analysis.
76702	Kazakhstan	Kazakhstan Multiple Indicator Cluster Survey 2010-2011	2010-2011	all	HepB3	Agency of the Republic of Kazakhstan on Statistics, United Nations Children's Fund (UNICEF). Kazakhstan Multiple Indicator Cluster Survey 2010-2011. New York, United States of America: United Nations Children's Fund (UNICEF), 2013.	HepB3 estimates considered implausibly low and display unlikely cohort trend inconsistent with administrative data. HepB3 removed from analysis.
232807*	Kenya	Kenya EPI National Immunization Coverage Survey 1992	1992	select	DTP3, MCV1	Kenya EPI National Immunization Coverage Survey 1992.	Selected cohorts excluded from administrative data bias modeling as a methodological decision to improve model performance
20120*	Kenya	Kenya Demographic and Health Survey 1993	1993	select	BCG1, DTP3, MCV1, Pol3	Central Bureau of Statistics (Kenya), Macro International, Inc, National Council for Population Development (NCPD). Kenya Demographic and Health Survey 1993. Fairfax, United States of America: ICF International.	Selected cohorts excluded from administrative data bias modeling as a methodological decision to improve model performance
189463	Kenya	Kenya Multiple Indicator Cluster Survey 1996	1996	all	BCG, DTP1, DTP3, MCV1, Pol3	Kenya National Bureau of Statistics, United Nations Children's Fund (UNICEF). Kenya Multiple Indicator Cluster Survey 1996.	Estimates considered implausible. Survey estimates are systematically high compared to administrative data and estimates from other established surveys series (Kenya Demographic and Health Survey 1998). Survey removed from analysis.
7440	Kenya	Kenya Welfare Monitoring Survey III 1997	1997	all	BCG, DTP1, DTP3, MCV1, Pol3	Central Bureau of Statistics (Kenya). Kenya Welfare Monitoring Survey III 1997. Nairobi, Kenya: Kenya National Bureau of Statistics.	Estimates considered implausible. Survey estimates are systematically high compared to administrative data and estimates from other established surveys series (Kenya Demographic and Health Survey 1998). Survey removed from analysis.

GHDx ID	Country	Series	Year(s)	Cohort	Vaccine(s)	Citation	Rationale for exclusion
20132*	Kenya	Kenya Demographic and Health Survey 1998	1998	select	BCG1, DTP1, DTP3, MCV1, Pol3	Central Bureau of Statistics (Kenya), Macro International, Inc, National Council for Population Development (NCPD). Kenya Demographic and Health Survey 1998. Fairfax, United States of America: ICF International.	Selected cohorts excluded from administrative data bias modeling as a methodological decision to improve model performance
7387*	Kenya	Kenya Multiple Indicator Cluster Survey 2000	2000	select	BCG1, DTP3, MCV1, Pol3	Central Bureau of Statistics (Kenya), United Nations Children's Fund (UNICEF). Kenya Multiple Indicator Cluster Survey 2000. New York, United States: United Nations Children's Fund (UNICEF).	Selected cohorts excluded from administrative data bias modeling as a methodological decision to improve model performance
157057	Kenya	Kenya Demographic and Health Survey 2014	2014	select	PCV3	ICF International, Kenya Medical Research Institute (KEMRI), Kenya National Bureau of Statistics, Ministry of Health (Kenya), National AIDS Control Council (Kenya), National Council for Population and Development (Kenya). Kenya Demographic and Health Survey 2014. Fairfax, United States of America: ICF International.	Survey precedes introduction of PCV to national immunization schedule. PCV removed from analysis.
49586	Kenya	Kenya Integrated Household Budget Survey Tabular Data 2005-2006	2005-2006	all	Pol3	Kenya National Bureau of Statistics. Kenya Integrated Household Budget Survey Tabular Data 2005-2006. Nairobi, Kenya: Kenya National Bureau of Statistics.	Pol3 estimates are systematically high compared to administrative data and estimates from other established surveys series (Kenya Demographic and Health Survey 2008-2009). Pol3 removed from analysis.
287198	Kenya	Kenya Integrated Household Budget Survey 2015-2016	2015-2016	select	MCV2	Kenya National Bureau of Statistics. 2015-2016 Kenya Integrated Household Budget Survey. Nairobi, Kenya: Kenya National Bureau of Statistics, 2015.	Older age-cohorts precede introduction of MCV2 to national immunization schedule. Select age-cohorts removed from analysis for MCV2.
462027	Kosovo	Kosovo Multiple Indicator Cluster Survey 2019-2020	2019-2020	all	BCG, DTP1, DTP3, HepB3, PCV3, MCV1, Pol3, RCV1	Kosovo Agency of Statistics, United Nations Children's Fund (UNICEF). Kosovo Multiple Indicator Cluster Survey 2019-2020. Fairfax, United States of America: ICF International, 2020.	Kosovo, which is treated as Serbia, is non-representative, and if effectively a subnational survey. Survey removed from analysis.

GHDx ID	Country	Series	Year(s)	Cohort	Vaccine(s)	Citation	Rationale for exclusion
7503*	Kuwait	Kuwait Child Health Survey 1987	1987	all	BCG	Health Ministers Council for GCC States, Ministry of Health (Kuwait). Kuwait Child Health Survey 1987.	Survey precedes introduction of BCG to national immunization schedule. BCG removed from analysis. In addition, selected cohorts excluded from administrative data bias modeling as a methodological decision to improve model performance
7503*	Kuwait	Kuwait Child Health Survey 1987	1987	select	BCG1	Health Ministers' Council for GCC States, Ministry of Health (Kuwait). Kuwait Child Health Survey 1987.	Selected cohorts excluded from administrative data bias modeling as a methodological decision to improve model performance
7504	Kuwait	Kuwait Family Health Survey 1996	1996	all	BCG	Health Ministers Council for GCC States, Ministry of Health (Kuwait), United Nations Statistics Division (UNSD). Kuwait Family Health Survey 1996.	Survey precedes introduction of BCG to national immunization schedule. BCG removed from analysis.
77518	Kyrgyzstan	Kyrgyzstan Demographic and Health Survey 2012	2012	select	Hib3	ICF International, Ministry of Health (Kyrgyzstan), National Statistical Committee of the Kyrgyz Republic. Kyrgyzstan Demographic and Health Survey 2012. Fairfax, United States of America: ICF International.	Older age-cohorts precede introduction of Hib3 to national immunization schedule. Select age-cohorts removed from analysis for Hib3.
162283	Kyrgyzstan	Kyrgyzstan Multiple Indicator Cluster Survey 2014	2014	all	BCG, MCV1	National Statistical Committee of the Kyrgyz Republic, United Nations Children's Fund (UNICEF). Kyrgyzstan Multiple Indicator Cluster Survey 2014. New York, United States of America: United Nations Children's Fund (UNICEF), 2015.	BCG and MCV1 estimates are systematically low compared to administrative data and estimates from other established surveys series (Kyrgyzstan Multiple Indicator Cluster Survey 2018). BCG and MCV1 removed from analysis.
7540	Kyrgyzstan	Kyrgyzstan Multiple Indicator Cluster Survey 2005-2006	2005-2006	all	DTP1, DTP3, Pol3	United Nations Children's Fund (UNICEF), National Statistical Committee of the Kyrgyz Republic. Kyrgyzstan Multiple Indicator Cluster Survey 2005-2006. New York, United States: United Nations Children's Fund (UNICEF).	Estimates considered implausible. Survey estimates are systematically low compared to administrative data and estimates from other established surveys series (Kyrgyzstan Demographic and Health Survey 2012). Survey removed from analysis.
142213*	Lesotho	Lesotho Multiple Indicator Cluster Survey 1996	1996	select	BCG1	Bureau of Statistics (Lesotho), Ministry of Health (Lesotho), United Nations Children's Fund (UNICEF). Lesotho Multiple Indicator Cluster Survey 1996.	Selected cohorts excluded from administrative data bias modeling as a methodological decision to improve model performance

GHDx ID	Country	Series	Year(s)	Cohort	Vaccine(s)	Citation	Rationale for exclusion
427778*	Lesotho	Lesotho Multiple Indicator Cluster Survey 2018	2018	select	RotaC	Bureau of Statistics (Lesotho), United Nations Children's Fund (UNICEF). Lesotho Multiple Indicator Cluster Survey 2018. New York, United States of America: United Nations Children's Fund (UNICEF), 2019.	Older age-cohorts precede introduction of rotavirus vaccine to national immunization schedule. Select age-cohorts removed from analysis for rotavirus vaccine. In addition, selected cohorts excluded from administrative data bias modeling as a methodological decision to improve model performance
427778*	Lesotho	Lesotho Multiple Indicator Cluster Survey 2018	2018	select	BCG1, DTP1, DTP3, Pol3	Bureau of Statistics (Lesotho), United Nations Children's Fund (UNICEF). Lesotho Multiple Indicator Cluster Survey 2018. New York, United States of America: United Nations Children's Fund (UNICEF), 2019.	Selected cohorts excluded from administrative data bias modeling as a methodological decision to improve model performance
20167	Lesotho	Lesotho Demographic and Health Survey 2004-2005	2004-2005	select	HepB3	Bureau of Statistics (Lesotho), Macro International, Inc, Ministry of Health and Social Welfare (Lesotho). Lesotho Demographic and Health Survey 2004-2005. Fairfax, United States of America: ICF International.	Older age-cohorts precede introduction of HepB3 to national immunization schedule. Select age-cohorts removed from analysis for HepB3.
232515*	Liberia	Liberia Integrated Management of Childhood Illness Household Baseline Survey 2000	2000	select	BCG1, DTP1, DTP3, MCV1, Pol3	Liberia Integrated Management of Childhood Illness Household Baseline Survey 2000.	Selected cohorts excluded from administrative data bias modeling as a methodological decision to improve model performance
286768	Liberia	Liberia Malaria Indicator Survey 2016	2016	select	PCV3	ICF International, Liberia Institute for Statistics and Geo-information Services (LISGIS), National Malaria Control Program (Liberia). Liberia Malaria Indicator Survey 2016. Fairfax, United States of America: ICF International, 2017.	Older age-cohorts precede introduction of PCV3 to national immunization schedule. Select age-cohorts removed from analysis for PCV3.
141586*	Liberia	Liberia National Nutrition Survey 1999-2000	1999-2000	select	DTP1, Pol3	Christian Health Association of Liberia, Ministry of Health and Social Welfare (Liberia), United Nations Children's Fund (UNICEF). Liberia National Nutrition Survey 1999-2000.	Selected cohorts excluded from administrative data bias modeling as a methodological decision to improve model performance
124079	Libya	Libya Multiple Indicator Cluster Survey 2003	2003	all	DTP1	Public Commission for Health Care Planning (Libya), United Nations Children's Fund (UNICEF). Libya Multiple Indicator Cluster Survey 2003.	Specific number of doses per vaccine not provided; DTP1 estimate removed from analysis

GHDx ID	Country	Series	Year(s)	Cohort	Vaccine(s)	Citation	Rationale for exclusion
27020	Madagascar	Madagascar Multiple Indicator Cluster Survey 2000	2000	all	DTP1, DTP3	National Institute of Statistics (Madagascar), United Nations Children's Fund (UNICEF). Madagascar Multiple Indicator Cluster Survey 2000. New York, United States of America: United Nations Children's Fund (UNICEF).	Estimates considered implausible. Survey estimates are systematically low compared to administrative data and estimates from other established surveys series (Madagascar Demographic and Health Survey 2003-2004). Survey removed from analysis.
493562	Madagascar	Madagascar Demographic and Health Survey 2021	2021	all	RCV1	ICF Macro, National Institute of Statistics (Madagascar). Madagascar Demographic and Health Survey 2021. Fairfax, United States of America: ICF International, 2022.	Survey precedes introduction of RCV1 to national immunization schedule. RCV1 removed from analysis.
20223*	Madagascar	Madagascar Demographic and Health Survey 2003-2004	2003-2004	select	MCV1	Macro International, Inc, National Institute of Statistics (Madagascar). Madagascar Demographic and Health Survey 2003-2004. Fairfax, United States of America: ICF International.	Selected cohorts excluded from administrative data bias modeling as a methodological decision to improve model performance
209533	Madagascar	Madagascar National Survey on Monitoring the Millennium Development Goals 2012-2013	2012-2013	all	BCG, DTP1, DTP3, HepB3, PCV3, MCV1, Pol3	African Development Bank (AfDB), National Institute of Statistics (Madagascar), UNICEF Madagascar, United Nations Population Fund (UNFPA), United Nations Women's Fund (UNIFEM), World Food Programme (WFP). Madagascar National Survey on Monitoring the Millennium Development Goals 2012-2013.	Estimates considered implausible. Survey estimates are systematically low compared to administrative data and estimates from other established surveys series (Madagascar Demographic and Health Survey 2008, Madagascar Multiple Indicator Cluster Survey 2012). Survey removed from analysis.
20263	Malawi	Malawi Demographic and Health Survey 2004-2005	2004-2005	select	HepB3, Hib3	Macro International, Inc, National Statistical Office of Malawi. Malawi Demographic and Health Survey 2004-2005. Fairfax, United States of America: ICF International.	Older age-cohorts precede introduction of HepB3 and Hib3 to national immunization schedule. Select age-cohorts removed from analysis for HepB3 and Hib3.
218581	Malawi	Malawi Demographic and Health Survey 2015-2016	2015-2016	all	MCV2	Emory University and Centers for Disease Control & Prevention Collaboration, ICF International, Ministry of Health (Malawi), National Statistical Office of Malawi. Malawi Demographic and Health Survey 2015-2016. Fairfax, United States of America: ICF International, 2017.	Older age-cohorts precede introduction of MCV2 to national immunization schedule. Select age-cohorts removed from analysis for MCV2.

GHDx ID	Country	Series	Year(s)	Cohort	Vaccine(s)	Citation	Rationale for exclusion
20283*	Mali	Mali Demographic and Health Survey 1987	1987	select	MCV1	Sahel Institute, Westinghouse; Institute for Resource Development. Mali Demographic and Health Survey 1987. Fairfax, United States of America: ICF International.	Selected cohorts excluded from administrative data bias modeling as a methodological decision to improve model performance
20274	Mali	Mali Demographic and Health Survey 2006	2006	all	HepB3	Macro International, Inc, Ministry of Health (Mali), National Directorate of Statistics and Informatics (DNSI) (Mali). Mali Demographic and Health Survey 2006. Fairfax, United States of America: ICF International.	HepB3 estimates are systematically low compared to administrative data and estimates from other established surveys series. HepB3 removed from analysis.
248224	Mali	Mali Multiple Indicator Cluster Survey 2015	2015	select	RCV1, RotaC	Ministry of Health (Mali), Ministry of Planning (Mali), National Institute of Statistics (INSTAT) (Mali), United Nations Children's Fund (UNICEF). Mali Multiple Indicator Cluster Survey 2015. New York, United States of America: United Nations Children's Fund (UNICEF), 2017.	Survey precedes introduction of RCV1 and some age-cohorts of rotavirus vaccine to national immunization schedule. RCV1 and select age-cohorts of rotavirus vaccines removed from analysis.
270627	Mali	Mali Multiple Indicator Cluster Survey 2009-2010	2009-2010	all	BCG, DTP1, DTP3, HepB3, MCV1, Pol3	Ministry of Health (Mali), National Institute of Statistics (INSTAT) (Mali), United Nations Children's Fund (UNICEF). Mali Multiple Indicator Cluster Survey 2009-2010. New York, United States of America: United Nations Children's Fund (UNICEF), 2017.	Estimates considered implausible. Survey estimates are systematically high compared to administrative data and estimates from other established surveys series. Survey removed from analysis.
77388	Mali	Mali Demographic and Health Survey 2012-2013	2012-2013	all	HepB3, PCV3, Pol3	ICF International, INFO-STAT (Mali), Ministry of Health (Mali), National Institute of Statistics (INSTAT) (Mali), Planning and Statistics Unit, Ministry of Health (Mali). Mali Demographic and Health Survey 2012-2013. Fairfax, United States of America: ICF International, 2014.	HepB3, Hib3, and Pol3 estimates are systematically low compared to administrative data and estimates from other established surveys series. HepB3, Hib3, and Pol3 removed from analysis.
24152*	Marshall Islands	Marshall Islands Community Survey 2006	2006	select	BCG1, DTP3, MCV1, Pol3	Economic Policy, Planning and Statistics Office (Marshall Islands). Marshall Islands Community Survey 2006.	Selected cohorts excluded from administrative data bias modeling as a methodological decision to improve model performance

GHDx ID	Country	Series	Year(s)	Cohort	Vaccine(s)	Citation	Rationale for exclusion
21440	Marshall Islands	Marshall Islands Demographic and Health Survey 2007	2007	all	BCG, DTP1, DTP3, MCV1, Pol3	Economic Policy, Planning and Statistics Office (Marshall Islands), Secretariat of the Pacific Community (SPC). Marshall Islands Demographic and Health Survey 2007.	Estimates considered implausible. Survey estimates are systematically low compared to administrative data and estimates from other established surveys series. Survey removed from analysis.
152783	Mauritania	Mauritania Multiple Indicator Cluster Survey 2011	2011	select	Hib3	National Office of Statistics (Mauritania), United Nations Children's Fund (UNICEF). Mauritania Multiple Indicator Cluster Survey 2011. New York, United States of America: United Nations Children's Fund (UNICEF), 2015.	Older age-cohorts precede introduction of Hib3 to national immunization schedule. Select age-cohorts removed from analysis for Hib3.
20322*	Mauritania	Mauritania Demographic and Health Survey 2000-2001	2000-2001	select	MCV1	Macro International, Inc, National Office of Statistics (Mauritania). Mauritania Demographic and Health Survey 2000-2001. Fairfax, United States of America: ICF International.	Selected cohorts excluded from administrative data bias modeling as a methodological decision to improve model performance
20326*	Mexico	Mexico Demographic and Health Survey 1987	1987	select	BCG1, DTP1, MCV1	Macro Systems, Inc.; Institute for Resource Development, Secretariat of Health (Mexico). Mexico Demographic and Health Survey 1987. Fairfax, United States of America: ICF International.	Selected cohorts excluded from administrative data bias modeling as a methodological decision to improve model performance
8442	Mexico	Mexico Family Life Survey 2002	2002	all	DTP1, DTP3, PCV3	Center for Research and Teaching in Economics (CIDE) (Mexico), Ibero-American University, National Institute of Perinatology (Mexico), National Institute of Statistics and Geography (INEGI) (Mexico). Mexico Family Life Survey 2002.	Estimates considered implausible. Survey estimates are systematically low compared to administrative data and estimates from other established surveys series. Survey removed from analysis.
23982	Mexico	Mexico National Survey of Demographic Dynamics 2006	2006	all	DTP1, DTP3, PCV3, Pol3	National Institute of Statistics, Geography, and Informatics (Mexico), National Institute for Public Health (Mexico), National Population Council (Mexico). Mexico National Survey of Demographic Dynamics 2006. Aguascalientes, Mexico: National Institute of Statistics, Geography, and Informatics (Mexico).	Estimates considered implausible. Survey estimates are systematically low compared to administrative data and estimates from other established surveys series. Survey removed from analysis.

GHDx ID	Country	Series	Year(s)	Cohort	Vaccine(s)	Citation	Rationale for exclusion
264590	Mexico	Mexico Multiple Indicator Cluster Survey 2015	2015	select	BCG, DTP1, DTP3, PCV3, MCV1, PCV3, Pol3, RotaC	National Institute of Public Health (Mexico), United Nations Children's Fund (UNICEF). Mexico Multiple Indicator Cluster Survey 2015. New York, United States of America: United Nations Children's Fund (UNICEF), 2017.	Estimates from survey microdata do not match coverage from survey report. Select age-cohorts removed from analysis.
505231*	Mexico	Mexico Continuous National Health and Nutrition Survey 2021	2021	select	BCG1	Government of Mexico, National Institute of Public Health (Mexico), Secretariat of Health (Mexico). Mexico Continuous National Health and Nutrition Survey 2021. Cuernavaca, Mexico: National Institute of Public Health (Mexico), 2022.	Selected cohorts excluded from administrative data bias modeling as a methodological decision to improve model performance
8555	Mexico	Mexico National Health Survey 1999-2000	1999-2000	all	DTP1, DTP3, PCV3, MCV1, Pol3	National Institute of Public Health (Mexico). Mexico National Health Survey 1999-2000.	Estimates considered implausible. Survey estimates are systematically high compared to administrative data and estimates from other established surveys series. Survey removed from analysis.
8684	Mexico	Mexico National Performance Evaluation Survey 2002-2003	2002-2003	all	DTP1, DTP3	National Institute of Public Health (Mexico), Secretariat of Health (Mexico), World Health Organization (WHO). Mexico National Performance Evaluation Survey 2002-2003.	Estimates considered implausible. Survey estimates are systematically low compared to administrative data and estimates from other established surveys series. Survey removed from analysis.
58419	Mexico	Mexico Family Life Survey 2005-2006	2005-2006	all	BCG, DTP1, DTP3, PCV3, MCV1, Pol3, RCV1	California Center for Population Research (CCPR), University of California Los Angeles (UCLA), Center for Research and Teaching in Economics (CIDE) (Mexico), Ibero-American University, National Institute of Public Health (Mexico). Mexico Family Life Survey 2005-2006.	Estimates considered implausible. Survey estimates are inconsistent with administrative data and estimates from other established survey series. Survey removed from analysis.
160781	Mexico	Mexico Family Life Survey 2008-2013	2008-2013	all	BCG, DTP1, DTP3, PCV3, MCV1, Pol3, RCV1	Center for Research and Teaching in Economics (CIDE) (Mexico), Duke University, Ibero-American University, National Institute of Public Health (Mexico), University of California, Los Angeles (UCLA). Mexico Family Life Survey 2008-2013.	Estimates considered implausible. Survey estimates are inconsistent with administrative data and estimates from other established survey series. Survey removed from analysis.

GHDx ID	Country	Series	Year(s)	Cohort	Vaccine(s)	Citation	Rationale for exclusion
81748	Mexico	Mexico National Survey of Health and Nutrition 2011-2012	2011-2012	select	PCV3	National Institute of Public Health (Mexico). Mexico National Survey of Health and Nutrition 2011-2012. Cuernavaca, Mexico: National Institute of Public Health (Mexico).	Older age-cohorts precede introduction of PCV3 to national immunization schedule. Select age-cohorts removed from analysis for PCV3.
126909	Morocco	Morocco National Survey on Population and Family Health 2010-2011	2010-2011	select	DTP1, DTP3	Ministry of Health (Morocco), Pan Arab Project for Family Health (PAPFAM), United Nations Children's Fund (UNICEF), United Nations Population Fund (UNFPA), World Health Organization (WHO). Morocco National Survey on Population and Family Health 2010-2011.	Survey reports DTP3 coverage greater than DTP1 coverage for some age-cohorts. Select age-cohorts removed for DTP3 due to reliability concerns.
55975	Mozambique	Mozambique Demographic and Health Survey 2011	2011	select	Hib3	ICF Macro, Manhica Health Research Center (CISM), Ministry of Health (Mozambique), National Institute of Statistics (INE) (Mozambique). Mozambique Demographic and Health Survey 2011. Fairfax, United States of America: ICF International.	Older age-cohorts precede introduction of Hib3 to national immunization schedule. Select age-cohorts removed from analysis for Hib3.
157060	Mozambique	Mozambique AIDS Indicator Survey 2015	2015	select	PCV3	Centers for Disease Control and Prevention (CDC), ICF International, Ministry of Health (Mozambique), National Institute of Health (Mozambique), National Institute of Statistics (INE) (Mozambique). Mozambique AIDS Indicator Survey 2015. Fairfax, United States of America: ICF International, 2018.	Older age-cohorts precede introduction of PCV3 to national immunization schedule. Select age-cohorts removed from analysis for PCV3.
20404	Namibia	Namibia Demographic and Health Survey 1992	1992	select	BCG, DTP1, DTP3, MCV1, Pol3	Central Statistics Office (Namibia), Macro International, Inc, Ministry of Health and Social Services (Namibia). Namibia Demographic and Health Survey 1992. Fairfax, United States of America: ICF International.	Older age-cohorts precede introduction of BCG, DTP1, DTP3, MCV1, and Pol3 to national immunization schedule. Select age-cohorts removed from analysis for BCG, DTP1, DTP3, MCV1, and Pol3.

GHDx ID	Country	Series	Year(s)	Cohort	Vaccine(s)	Citation	Rationale for exclusion
20462	Nepal	Nepal Demographic and Health Survey 2006	2006	select	HepB3	Macro International, Inc, Ministry of Health and Population (Nepal), New ERA. Nepal Demographic and Health Survey 2006. Fairfax, United States of America: ICF International.	Older age-cohorts precede introduction of HepB3 to national immunization schedule. Select age-cohorts removed from analysis for HepB3.
21240	Nepal	Nepal Demographic and Health Survey 2011	2011	select	Hib3	ICF Macro, Ministry of Health and Population (Nepal), New ERA. Nepal Demographic and Health Survey 2011. Fairfax, United States of America: ICF International.	Older age-cohorts precede introduction of Hib3 to national immunization schedule. Select age-cohorts removed from analysis for Hib3.
46480	Nepal	Nepal Living Standards Measurement Survey 2003-2004	2003-2004	all	BCG, DTP1, DTP3, MCV1, Pol3	Central Bureau of Statistics (Nepal), World Bank. Nepal Living Standards Measurement Survey 2003-2004. Kathmandu, Nepal: Central Bureau of Statistics (Nepal).	Estimates considered implausible. Survey estimates are systematically high compared to administrative data and estimates from other established surveys series (Nepal Demographic and Health Survey 2006). Survey removed from analysis.
286782	Nepal	Nepal Demographic and Health Survey 2016-2017	2016-2017	select	PCV3	ICF International, Ministry of Health (Nepal), New ERA. Nepal Demographic and Health Survey 2016-2017. Fairfax, United States of America: ICF International, 2017.	Survey precedes introduction of PCV3 to national immunization schedule. PCV3 removed from analysis.
9310	Nicaragua	Nicaragua Living Standards Measurement Survey 1993	1993	all	MCV2	National Institute of Statistics and Censuses (Nicaragua), World Bank. Nicaragua Living Standards Measurement Survey 1993.	Survey precedes introduction of second measles dose to national immunization schedule. MCV2 removed from analysis.
9422	Nicaragua	Nicaragua Living Standards Measurement Survey 2001	2001	select	DTP1, DTP3, RCV1	National Institute of Statistics and Censuses (Nicaragua), World Bank. Nicaragua Living Standards Measurement Survey 2001.	Survey precedes introduction of RCV1 to national immunization schedule and reports DTP3 coverage greater than DTP1 coverage for some age-cohorts. RCV1 and select age-cohorts of DTP3 removed from analysis.
9370	Nicaragua	Nicaragua Living Standards Measurement Survey 1998-1999	1998-1999	all	MCV2	National Institute of Statistics and Censuses (Nicaragua), World Bank. Nicaragua Living Standards Measurement Survey 1998-1999.	Survey precedes introduction of second measles dose to national immunization schedule. MCV2 removed from analysis.
20518*	Niger	Niger Demographic and Health Survey 1992	1992	select	DTP3, MCV1, Pol3	Department of Statistics and National Accounts (Niger), Macro International, Inc. Niger Demographic and Health Survey 1992. Fairfax, United States of America: ICF International.	Selected cohorts excluded from administrative data bias modeling as a methodological decision to improve model performance

GHDx ID	Country	Series	Year(s)	Cohort	Vaccine(s)	Citation	Rationale for exclusion
9465*	Niger	Niger Continuous Survey on Economic and Social Conditions 1995-1996	1995	select	MCV1	Department of Statistics and National Accounts (Niger), United Nations Department of Economic and Social Development. Niger Continuous Survey on Economic and Social Conditions 1995-1996. Niamey, Niger: Department of Statistics and National Accounts (Niger).	Selected cohorts excluded from administrative data bias modeling as a methodological decision to improve model performance
485235	Niger	Niger Integrated Measles Campaign Evaluation Survey 2013	2013	all	BCG, DTP1, DTP3, HepB3, PCV3, MCV1, Pol3	National Institute of Statistics (Niger), United Nations Children's Fund (UNICEF), World Health Organization (WHO). Niger Integrated Measles Campaign Evaluation Survey 2013. 2013.	Estimate derived from a post-campaign coverage survey (2012 post-campaign survey). Select age-cohorts removed from analysis.
524153	Niger	Niger National Survey on Fertility and Mortality of Children Under Five Years 2021	2021	all	BCG, DTP1, DTP3, HepB3, PCV3, MCV1, MCV2, PCV3, Pol3, RCV1, RotaC	Ministry of Public Health (Niger), National Institute of Statistics (Niger), Utica International. Niger National Survey on Fertility and Mortality of Children Under Five Years 2021. Niamey, Niger: National Institute of Statistics (Niger), 2022.	Estimates considered implausible. Survey estimates are systematically low compared to administrative data and estimates from other established surveys series. Survey removed from analysis.
20552*	Nigeria	Nigeria Demographic and Health Survey 1990	1990	select	MCV1	Federal Office of Statistics (Nigeria), Institute for Resource Development, Macro Systems. Nigeria Demographic and Health Survey 1990. Fairfax, United States of America: ICF International.	Selected cohorts excluded from administrative data bias modeling as a methodological decision to improve model performance
24890	Nigeria	Nigeria General Household Survey 2007	2007	all	BCG, DTP1, DTP3, MCV1, Pol3, RCV1	Central Bank of Nigeria, National Bureau of Statistics (Nigeria), Nigerian Communications Commission (NCC). Nigeria General Household Survey 2007. Abuja, Nigeria: National Bureau of Statistics (Nigeria).	Estimates considered implausible. Survey estimates are systematically low compared to administrative data and estimates from other established surveys series. Survey removed from analysis.
9516	Nigeria	Nigeria Multiple Indicator Cluster Survey 2007	2007	select	PCV3	United Nations Children's Fund (UNICEF), National Bureau of Statistics (Nigeria). Nigeria Multiple Indicator Cluster Survey 2007. New York, United States: United Nations Children's Fund (UNICEF).	Older age-cohorts precede introduction of PCV3 to national immunization schedule. Select age-cohorts removed from analysis for PCV3.

GHDx ID	Country	Series	Year(s)	Cohort	Vaccine(s)	Citation	Rationale for exclusion
77390	Nigeria	Nigeria Demographic and Health Survey 2013	2013	select	Hib3	ICF International, National Population Commission of Nigeria. Nigeria Demographic and Health Survey 2013. Fairfax, United States of America: ICF International.	Older age-cohorts precede introduction of Hib3 to national immunization schedule. Select age-cohorts removed from analysis for Hib3.
408484*	Nigeria	Nigeria Demographic and Health Survey 2018	2018	all	MCV2	Federal Ministry of Health (Nigeria), ICF International, National Population Commission (NPC). Nigeria Demographic and Health Survey 2018. Fairfax, United States of America: ICF International, 2020.	Survey precedes introduction of MCV2 to national immunization schedule. MCV2 removed from analysis. In addition, selected cohorts excluded from administrative data bias modeling as a methodological decision to improve model performance
408484*	Nigeria	Nigeria Demographic and Health Survey 2018	2018	select	BCG1, DTP1, DTP3, MCV1, Pol3	Federal Ministry of Health (Nigeria), ICF International, National Population Commission (NPC). Nigeria Demographic and Health Survey 2018. Fairfax, United States of America: ICF International, 2020.	Selected cohorts excluded from administrative data bias modeling as a methodological decision to improve model performance
494197*	Nigeria	Nigeria National Nutrition and Health Survey 2018	2018	select	DTP1, DTP3, MCV1	Federal Ministry of Health (Nigeria), National Bureau of Statistics (Nigeria), National Population Commission of Nigeria. Nigeria National Nutrition and Health Survey 2018.	Selected cohorts excluded from administrative data bias modeling as a methodological decision to improve model performance
20573*	Nigeria	Nigeria - Ondo Special Demographic and Health Survey 1986-1987	1986-1987	select	BCG1, DTP1, DTP3, MCV1, Pol3	Federal Ministry of Health (Nigeria), Macro Systems, Inc.; Institute for Resource Development, National Population Bureau (Nigeria). Nigeria - Ondo Special Demographic and Health Survey 1986-1987. Fairfax, United States of America: ICF International.	Selected cohorts excluded from administrative data bias modeling as a methodological decision to improve model performance
222875	Nigeria	Nigeria Annual Report and Statement of Accounts 2001	1991-2001	all	BCG, DTP3, MCV1, Pol3	Central Bank of Nigeria. Nigeria Annual Report and Statement of Accounts 2001. Abuja, Nigeria: Central Bank of Nigeria, 2002.	Estimates considered implausible. Survey estimates are systematically high compared to administrative data and estimates from other established surveys series (Nigeria Multiple Indicator Cluster Survey 1999). Survey removed from analysis.
222872	Nigeria	Nigeria Annual Report and Statement of Accounts 2002	1991-2002	all	BCG, DTP3, MCV1, Pol3	Central Bank of Nigeria. Nigeria Annual Report and Statement of Accounts 2002. Abuja, Nigeria: Central Bank of Nigeria, 2003.	Estimates considered implausible. Survey estimates are systematically high compared to administrative data and estimates from other established surveys series (Nigeria Multiple Indicator Cluster Survey 1999). Survey removed from analysis.

GHDx ID	Country	Series	Year(s)	Cohort	Vaccine(s)	Citation	Rationale for exclusion
151719	Nigeria	Nigeria Living Standards Survey 2008-2010	2008-2010	all	MCV1	National Bureau of Statistics (Nigeria). Nigeria Living Standards Survey 2008-2010. Abuja, Nigeria: National Bureau of Statistics (Nigeria).	DTP1 and MCV1 estimates are systematically high compared to administrative data and estimates from other established surveys series (Nigeria Multiple Indicator Cluster Survey 2011). DTP1 and MCV1 removed from analysis.
151802	Nigeria	Nigeria General Household Survey 2010-2011	2010-2011	all	BCG, DTP1, DTP3, MCV1, Pol3, RCV1	National Bureau of Statistics (Nigeria). Nigeria General Household Survey 2010-2011. Abuja, Nigeria: National Bureau of Statistics (Nigeria).	Survey estimates are systematically high compared to administrative data and estimates from other established survey series (Nigeria Demographic and Health Survey 2013). Survey removed from analysis.
151797	Nigeria	Nigeria General Household Survey, Panel 2012-2013, Wave 2	2012-2013	select	BCG, DTP1, DTP3, MCV1, Pol3, RCV1	National Bureau of Statistics (Nigeria). Nigeria General Household Survey, Panel 2012-2013, Wave 2. Washington DC, United States of America: World Bank.	DTP3 estimates are systematically high compared to administrative data and estimates from other established surveys series (Nigeria Demographic and Health Survey 2013). DTP3 removed from analysis.
7467	North Korea	North Korea Multiple Indicator Cluster Survey 2000	2000	all	DTP1, DTP3	Central Bureau of Statistics (North Korea), United Nations Children's Fund (UNICEF). North Korea Multiple Indicator Cluster Survey 2000.	Estimates considered implausible. Survey estimates are inconsistent with administrative data. DTP1, DTP3 removed from analysis.
91324	North Macedonia	Macedonia Multiple Indicator Cluster Survey 2011	2011	select	Hib3	Institute of Public Health (Republic of North Macedonia), Ipsos Strategic Puls, Ministry of Education and Science (Macedonia), Ministry of Labor and Social Policy (Macedonia), United Nations Children's Fund (UNICEF). Macedonia Multiple Indicator Cluster Survey 2011. New York, United States of America: United Nations Children's Fund (UNICEF), 2013.	Older age-cohorts precede introduction of Hib3 to national immunization schedule. Select age-cohorts removed from analysis for Hib3.
265082	Pakistan	Pakistan Social and Living Standards Measurement Survey 2014-2015	2014-2015	all	BCG, DTP1, DTP3, HepB3, MCV1, MCV2, Pol3	Pakistan Bureau of Statistics. Pakistan Social and Living Standards Measurement Survey 2014-2015. Islamabad, Pakistan: Pakistan Bureau of Statistics, 2016.	Estimates considered implausible. Survey estimates are inconsistent with administrative data and estimates from other established survey series (Pakistan Demographic and Health Survey 2017-2018). Survey removed from analysis.

GHDx ID	Country	Series	Year(s)	Cohort	Vaccine(s)	Citation	Rationale for exclusion
286783	Pakistan	Pakistan Demographic and Health Survey 2017-2018	2017-2018	all	RCV1	ICF International, Ministry of National Health Services, Regulations & Coordination (Pakistan), National Institute of Population Studies (Pakistan). Pakistan Demographic and Health Survey 2017-2018. Fairfax, United States of America: ICF International, 2018.	Survey precedes introduction of RCV1 to national immunization schedule. RCV1 removed from analysis.
142186	Palestine	Palestine - West Bank and Gaza Strip Multiple Indicator Cluster Survey 1996 - UNICEF	1996	all	BCG	Palestine - West Bank and Gaza Strip Health Survey 1996	Estimates considered implausible. Survey estimates are systematically low compared to administrative data and estimates from other established surveys series (West Bank and Gaza Strip Multiple Indicator Cluster Survey 2000). Survey removed from analysis.
9999	Palestine	Palestine Family Health Survey 2006-2007	2006-2007	select	DTP1, DTP3, RCV1	League of Arab States, Palestinian Central Bureau of Statistics, United Nations Children's Fund (UNICEF). Palestine Family Health Survey 2006-2007.	DTP3 estimates are systematically low compared to administrative data and estimates from other established surveys series (Palestine Multiple Indicator Cluster Survey 2010). Older age cohorts precede introduction of RCV1 to national immunization schedule. DTP3 and select age-cohorts of RCV1 removed from analysis.
46517	Panama	Panama Living Standard Measurement Survey 2008	2008	select	HepB3, RotaC	Census and Statistics Directorate (Panama), Ministry of Economy and Finance (Panama), World Bank. Panama Living Standard Measurement Survey 2008. Washington DC, United States of America: World Bank.	HepB3 estimates include implausibly low coverage rates of 0%. Older age-cohorts precede introduction of rotavirus vaccine to national immunization schedule. HepB3 and select age-cohorts of RotaC removed from analysis.
161587	Panama	Panama Multiple Indicator Cluster Survey 2013	2013	select	MCV2, PCV3	National Institute of Statistics and Census (Panama), United Nations Children's Fund (UNICEF). Panama Multiple Indicator Cluster Survey 2013. New York, United States of America: United Nations Children's Fund (UNICEF).	Older age-cohorts precede introduction of these vaccines to national immunization schedule. Select age cohorts removed from analysis for these vaccines.
20599*	Papua New Guinea	Papua New Guinea Demographic and Health Survey 1996-1997	1996-1997	select	MCV1	National Statistical Office (Papua New Guinea). Papua New Guinea Demographic and Health Survey 1996-1997.	Selected cohorts excluded from administrative data bias modeling as a methodological decision to improve model performance
426238	Papua New Guinea	Papua New Guinea Demographic and Health Survey 2016-2018	2016-2018	all	MCV2	ICF International, National Statistical Office (Papua New Guinea). Papua New Guinea Demographic and Health Survey 2016-2018. Fairfax, United States of America: ICF International, 2019.	Survey precedes introduction of MCV2 to national immunization schedule. MCV2 removed from analysis.

GHDx ID	Country	Series	Year(s)	Cohort	Vaccine(s)	Citation	Rationale for exclusion
10370	Paraguay	Paraguay Reproductive Health Survey 2004	2004	select	HepB3, Hib3	Division of Reproductive Health-Centers for Disease Control and Prevention (CDC). (2005): Paraguay Reproductive Health Survey 2004. Asunción, Paraguay, Paraguayan Center for Population Studies (CEPEP).	Older age-cohorts precede introduction of HepB3 and Hib3 to national immunization schedule. Select age-cohorts removed from analysis for HepB3 and Hib3.
20649	Peru	Peru Demographic and Health Survey 2000	2000	select	HepB3, Hib3	Macro International, Inc, National Institute of Statistics (Peru). Peru Demographic and Health Survey 2000. Fairfax, United States of America: ICF International.	HepB3 and older age-cohorts of Hib3 precede introduction to national immunization schedule. HepB3 and select age-cohorts of Hib3 removed from analysis.
270404	Peru	Peru Continuous Demographic and Health Survey 2009	2009	select	MCV2, PCV3, RCV1, RotaC	National Institute of Statistics and Informatics (Peru), ORC Macro. Peru Continuous Demographic and Health Survey 2009. Fairfax, United States of America: ICF International.	PCV3, RCV1, RotaC and older age-cohorts of MCV2 precede introduction to national immunization schedule. PCV3, RCV1, RotaC and select age-cohorts of MCV2 removed from analysis.
270469	Peru	Peru Continuous Demographic and Health Survey 2010	2010	select	MCV2, PCV3, RCV1, RotaC	National Institute of Statistics and Informatics (Peru). Peru Continuous Demographic and Health Survey 2010. Fairfax, United States of America: ICF International.	MCV2, RCV1 and older age-cohorts of RotaC and PCV3 precede introduction to national immunization schedule. MCV2, RCV1 and select age-cohorts of RotaC and PCV3 removed from analysis.
270470	Peru	Peru Continuous Demographic and Health Survey 2011	2011	select	MCV2, PCV3, RCV1	Macro International, Inc, National Institute of Statistics and Informatics (Peru). Peru Continuous Demographic and Health Survey 2011. Fairfax, United States of America: ICF International.	MCV2, RCV1 and older age-cohorts of PCV3 precede introduction to national immunization schedule. MCV2, RCV1 and select age-cohorts of PCV3 removed from analysis.
270471	Peru	Peru Continuous Demographic and Health Survey 2012	2012	select	MCV2, PCV3, RCV1	Macro International, Inc, National Institute of Statistics and Informatics (Peru). Peru Continuous Demographic and Health Survey 2012. Fairfax, United States of America: ICF International.	MCV2, RCV1 and older age-cohorts of PCV3 precede introduction to national immunization schedule. MCV2, RCV1 and select age-cohorts of PCV3 removed from analysis.
275090	Peru	Peru Continuous Demographic and Health Survey 2003-2008	2003-2008	select	HepB3, MCV1, MCV2, PCV3, RCV1	Ministry of Economy and Finance (Peru), National Institute of Statistics and Informatics (Peru), ORC Macro. Peru Continuous Demographic and Health Survey 2003-2008. Fairfax, United States of America: ICF International.	Required survey indicators for RCV1 and MCV1 missing from survey data. Survey precedes introduction of MCV2, PCV3, HepB3 to national immunization schedule. HepB3, MCV1, MCV2, PCV3, RCV1 removed from analysis.

GHDx ID	Country	Series	Year(s)	Cohort	Vaccine(s)	Citation	Rationale for exclusion
21421	Philippines	Philippines Demographic and Health Survey 2008	2008	all	Hib3	Macro International, Inc, National Statistics Office (Philippines). Philippines Demographic and Health Survey 2008. Fairfax, United States of America: ICF International, 2010.	Survey precedes introduction of Hib3 to national immunization schedule. Hib3 removed from analysis.
142943	Philippines	Philippines Demographic and Health Survey 2013	2013	select	HepB3, RCV1, RotaC	ICF International, Philippines Statistics Authority. Philippines Demographic and Health Survey 2013. Fairfax, United States of America: ICF International, 2014.	HepB3 estimates are implausibly low compared to administrative data and estimates from other established survey series (Philippines Multiple Indicator Survey 2016). Older age-cohorts precede introduction of rotavirus vaccine and RCV1 to national immunization schedule. HepB3 and select age-cohorts of RotaC and RCV1 removed from analysis.
264955	Philippines	Philippines Multiple Indicator Survey 2016	2016	all	MCV2	Demographic Research and Development Foundation Inc. (Philippines), National Statistics Office (Philippines), United Nations Children's Fund (UNICEF). Philippines Multiple Indicator Survey 2016.	MCV2 estimates include implausibly low coverage rates of 0%. MCV2 removed from analysis.
337877	Philippines	Philippines Demographic and Health Survey 2017	2017	all	RCV1	ICF International, Philippines Statistics Authority, United States Agency for International Development (USAID). Philippines Demographic and Health Survey 2017. Fairfax, United States of America: ICF International, 2018.	Survey precedes introduction of RCV1 to national immunization schedule. RCV1 removed from analysis.
69911	Qatar	Qatar Multiple Indicator Cluster Survey 2012	2012	all	BCG, DTP1, DTP3, HepB3, PCV3, MCV1, MCV2, Pol3, RCV1	Ministry of Development Planning and Statistics (Qatar), Qatar Foundation for Education, Science and Community Development, Qatar Statistics Authority, Supreme Council of Health (Qatar), United Nations Children's Fund (UNICEF). Qatar Multiple Indicator Cluster Survey 2012. New York, United States of America: United Nations Children's Fund (UNICEF), 2018.	Estimates considered implausible. Survey estimates are systematically low compared to administrative data and estimates from other established surveys series. Survey removed from analysis.

GHDx ID	Country	Series	Year(s)	Cohort	Vaccine(s)	Citation	Rationale for exclusion
56040	Rwanda	Rwanda Demographic and Health Survey 2010-2011	2010-2011	select	HepB3, Hib3, PCV3	ICF Macro, Ministry of Health (Rwanda), National Institute of Statistics of Rwanda. Rwanda Demographic and Health Survey 2010-2011. Fairfax, United States of America: ICF International.	HepB3, Hib3, and PCV3 estimates do not follow a reasonable age structure. Older children have significantly higher coverage than reported in either administrative data or other survey series, suggesting survey is capturing results of catch-up immunization. Select age-cohorts removed from analysis for HepB3, Hib3, and PCV3.
157063	Rwanda	Rwanda Demographic and Health Survey 2014-2015	2014-2015	select	RCV1, RotaC	ICF International, Ministry of Health (Rwanda), National Institute of Statistics of Rwanda. Rwanda Demographic and Health Survey 2014-2015. Fairfax, United States of America: ICF International, 2016.	RCV1 and older age-cohorts of rotavirus vaccine precede introduction to national immunization schedule. RCV1 and select age-cohorts of rotavirus vaccine removed from analysis.
456286*	Samoa	Samoa Multiple Indicator Cluster Survey 2019-2020	2019-2020	select	DTP1	Pacific Community Statistics for Development Division, Samoa Bureau of Statistics, United Nations Children's Fund (UNICEF). Samoa Multiple Indicator Cluster Survey 2019-2020. New York, United States of America: United Nations Children's Fund (UNICEF), 2022.	Selected cohorts excluded from administrative data bias modeling as a methodological decision to improve model performance
27055	São Tomé and Príncipe	Sao Tome and Principe Multiple Indicator Cluster Survey 2000	2000	select	BCG, DTP1, DTP3, MCV1, Pol3	National Institute of Statistics (Sao Tome and Principe), United Nations Children's Fund (UNICEF). Sao Tome and Principe Multiple Indicator Cluster Survey 2000. New York, United States of America: United Nations Children's Fund (UNICEF).	Estimates considered implausible. Survey estimates do not follow a reasonable age structure. Older children have significantly higher coverage than reported in either administrative data or other survey series, suggesting survey is capturing results of catch-up immunization. Select age-cohorts removed from analysis.
526191	Saudi Arabia	Saudi Arabia World Health Survey 2019	2019	all	DTP1, DTP3, HepB3, PCV3, MCV1, MCV2, RCV1	Ministry of Health (Saudi Arabia). Saudi Arabia World Health Survey 2019.	Data reporting error between denominator and coverage. Survey removed from analysis.
286772	Senegal	Senegal Continuous Demographic and Health Survey 2016	2016	select	MCV2	ICF International, Ministry of Health and Social Action (Senegal), National Agency of Statistics and Demography (Senegal). Senegal Continuous Demographic and Health Survey 2016. Fairfax, United States of America: ICF International, 2017.	Older age-cohorts precede introduction of MCV2 to national immunization schedule. Select age-cohorts removed from analysis for MCV2.

GHDx ID	Country	Series	Year(s)	Cohort	Vaccine(s)	Citation	Rationale for exclusion
460813	Senegal	Senegal Continuous Demographic and Health Survey 2019	2019	select	BCG, DTP1, DTP3, HepB3, PCV3, MCV1, PCV3, Pol3, RotaC	ICF International, Ministry of Health and Social Action (Senegal), National Agency of Statistics and Demography (Senegal), United States Agency for International Development (USAID). Senegal Continuous Demographic and Health Survey 2019. Fairfax, United States of America: ICF International, 2020.	Estimates considered implausible. Survey estimates do not follow a reasonable age structure. Older children have significantly higher coverage than reported in either administrative data or other survey series, suggesting survey is capturing results of catch-up immunization. Select age-cohorts removed from analysis.
459477	Serbia	Serbia Multiple Indicator Cluster Survey 2019	2019	select	PCV3	Statistical Office of the Republic of Serbia and UNICEF. 2019. Serbia Multiple Indicator Cluster Survey and Serbia Roma Settlements Multiple Indicator Cluster Survey, 2019, Survey Findings Report. Belgrade, Serbia: Statistical Office of the Republic of Serbia and UNICEF.	Older age-cohorts precede introduction of PCV3 to national immunization schedule. Select age-cohorts removed from analysis for PCV3.
231398	Sierra Leone	Sierra Leone National EPI Coverage Evaluation Survey 1991	1991	all	BCG, DTP3, MCV1, Pol3	Sierra Leone National EPI Coverage Evaluation Survey 1991.	Estimates considered implausible. Survey estimates are systematically high compared to administrative data and estimates from other established surveys series (Sierra Leone Multiple Indicator Survey 2000). Survey removed from analysis.
231396	Sierra Leone	Sierra Leone National EPI Coverage Evaluation Survey 1993	1993	all	BCG, DTP3, MCV1, Pol3	Sierra Leone National EPI Coverage Evaluation Survey 1993.	Estimates considered implausible. Survey estimates are systematically high compared to administrative data and estimates from other established surveys series (Sierra Leone Multiple Indicator Survey 2000). Survey removed from analysis.
11639*	Sierra Leone	Sierra Leone Multiple Indicator Cluster Survey 2000	2000	select	MCV1	Central Statistics Office (Sierra Leone), United Nations Children's Fund (UNICEF). Sierra Leone Multiple Indicator Cluster Survey 2000. New York, United States of America: United Nations Children's Fund (UNICEF).	Selected cohorts excluded from administrative data bias modeling as a methodological decision to improve model performance
11649	Sierra Leone	Sierra Leone Multiple Indicator Cluster Survey 2005	2005	all	RCV1	United Nations Children's Fund (UNICEF), Statistics Sierra Leone. Sierra Leone Multiple Indicator Cluster Survey 2005. New York, United States: United Nations Children's Fund (UNICEF).	Survey precedes introduction of RCV1 to national immunization schedule. RCV1 removed from analysis.

GHDx ID	Country	Series	Year(s)	Cohort	Vaccine(s)	Citation	Rationale for exclusion
21258	Sierra Leone	Sierra Leone Demographic and Health Survey 2008	2008	select	HepB3, Hib3	Macro International, Inc, Statistics Sierra Leone. Sierra Leone Demographic and Health Survey 2008. Fairfax, United States of America: ICF International.	Older age-cohorts precede introduction of HepB3 and Hib3 to national immunization schedule. Select age-cohorts removed from analysis for HepB3 and Hib3.
76700	Sierra Leone	Sierra Leone Multiple Indicator Cluster Survey 2010	2010	select	HepB3, MCV1	Statistics Sierra Leone, United Nations Children's Fund (UNICEF). Sierra Leone Multiple Indicator Cluster Survey 2010. New York, United States of America: United Nations Children's Fund (UNICEF).	Older age-cohorts precede introduction of HepB3 to national immunization schedule. MCV1 estimates from survey microdata do not match coverage from survey report. MCV1 and select age-cohorts of HepB3 removed from analysis.
131467	Sierra Leone	Sierra Leone Demographic and Health Survey 2013	2013	select	PCV3	ICF International, Ministry of Health and Sanitation (Sierra Leone), Statistics Sierra Leone. Sierra Leone Demographic and Health Survey 2013. Fairfax, United States of America: ICF International, 2014.	Older age-cohorts precede introduction of PCV3 to national immunization schedule. Select age-cohorts removed from analysis for PCV3.
425283	Sierra Leone	Sierra Leone Demographic and Health Survey 2019	2019	select	BCG, DTP1, DTP3, HepB3, PCV3, MCV1, PCV3, Pol3, RotaC	ICF International, Ministry of Health and Sanitation (Sierra Leone), Statistics Sierra Leone. Sierra Leone Demographic and Health Survey 2019. Fairfax, United States of America: ICF International, 2020.	Estimates considered implausible for some age-cohorts due to sudden drop in coverage. Coverage trends are inconsistent with administrative data and estimates from other established survey series. Select age-cohorts removed from analysis.
151893*	Somalia	Somalia Family Health Survey 1983	1983	all	DTP1, DTP3	Ministry of Health (Somalia), Ministry of National Planning (Somalia), Westinghouse Health Systems, Inc.. Somalia Family Health Survey 1983.	Survey reports higher DTP3 coverage than DTP1 coverage. DTP1 removed from analysis due to reliability concerns. In addition, selected cohorts excluded from administrative data bias modeling as a methodological decision to improve model performance
151893*	Somalia	Somalia Family Health Survey 1983	1983	select	BCG1, MCV1, Pol3	Ministry of Health (Somalia), Ministry of National Planning (Somalia), Westinghouse Health Systems, Inc.. Somalia Family Health Survey 1983.	Selected cohorts excluded from administrative data bias modeling as a methodological decision to improve model performance

GHDx ID	Country	Series	Year(s)	Cohort	Vaccine(s)	Citation	Rationale for exclusion
559069	Somalia	Somalia Post Campaign Coverage Survey and Routine Immunization Coverage Evaluation Survey 2023	2023	all	BCG, DTP1, DTP3, HepB3, PCV3, MCV1, Pol3	EQS Advisors, Ministry of Health (Somalia), Somali National University, United Nations Children's Fund (UNICEF), World Health Organization (WHO). Somalia Post Campaign Coverage Survey and Routine Immunization Coverage Evaluation Survey 2023. Geneva, Switzerland: World Health Organization (WHO).	Survey is not nationally representative. Survey removed from analysis.
20796	South Africa	South Africa Demographic and Health Survey 1998	1998	select	HepB3	Department of Health (South Africa), Macro International, Inc, South African Medical Research Council. South Africa Demographic and Health Survey 1998. Fairfax, United States of America: ICF International.	Older age-cohorts precede introduction of HepB3 to national immunization schedule. Select age-cohorts removed from analysis for HepB3.
21864	South Africa	South Africa World Health Survey 2002-2003	2002-2003	all	DTP1, DTP3, MCV1	World Health Organization (WHO). South Africa World Health Survey 2002-2003. Geneva, Switzerland: World Health Organization (WHO), 2005.	Estimates considered implausible. Survey estimates are inconsistent with administrative data and estimates from other established survey series (South Africa Demographic and Health Survey 2003-2004). Survey removed from analysis.
32189	South Sudan	South Sudan Multiple Indicator Cluster Survey 2010	2010	select	BCG, DTP1, DTP3, HepB3, PCV3, MCV1, Pol3	Central Bureau of Statistics (Sudan), Federal Ministry of Health (Sudan), Government of Sudan, Ministry of Health (South Sudan), Southern Sudan Centre for Census, Statistics and Evaluation. Sudan - South Multiple Indicator Cluster Survey 2010. New York, United States of America: United Nations Children's Fund (UNICEF), 2015.	Older age-cohorts precede introduction of these vaccines to national immunization schedule. Select age cohorts removed from analysis for these vaccines.
20806*	Sri Lanka	Sri Lanka Demographic and Health Survey 1987	1987	select	MCV1	Department of Census and Statistics (Sri Lanka), Westinghouse; Institute for Resource Development. Sri Lanka Demographic and Health Survey 1987. Fairfax, United States of America: ICF International.	Older age-cohorts precede introduction of MCV1 to national immunization schedule. Select age-cohorts removed from analysis for MCV1. In addition, selected cohorts excluded from administrative data bias modeling as a methodological decision to improve model performance

GHDx ID	Country	Series	Year(s)	Cohort	Vaccine(s)	Citation	Rationale for exclusion
20806*	Sri Lanka	Sri Lanka Demographic and Health Survey 1987	1987	select	MCV1	Department of Census and Statistics (Sri Lanka), Westinghouse; Institute for Resource Development. Sri Lanka Demographic and Health Survey 1987. Fairfax, United States of America: ICF International.	Selected cohorts excluded from administrative data bias modeling as a methodological decision to improve model performance
90705	Sri Lanka	Sri Lanka Demographic and Health Survey 1993	1993	all	DTP1, DTP3	Department of Census and Statistics (Sri Lanka). Sri Lanka Demographic and Health Survey 1993. 2009.	Survey reports higher DTP3 coverage than DTP1 coverage. DTP1 removed from analysis due to reliability concerns.
18815	Sri Lanka	Sri Lanka Demographic and Health Survey 2006-2007	2006-2007	select	HepB3	Department of Census and Statistics (Sri Lanka), Macro International, Inc. Sri Lanka Demographic and Health Survey 2006-2007. 2009.	HepB3 estimates do not follow a reasonable age structure. Older children have significantly higher coverage than reported in either administrative data or other survey series, suggesting survey is capturing results of catch-up immunization. Select age cohorts removed from analysis for HepB3.
24143	Sudan	Sudan Family Health Survey 2006	2006	all	BCG, DTP1, DTP3, MCV1, Pol3	Ministry of Health (Southern Sudan), Federal Ministry of Health (Sudan), Southern Sudan Centre for Census, Statistics and Evaluation (SSCCSE), Central Bureau of Statistics (Sudan). Sudan Family Health Survey 2006.	Estimates considered implausible. Survey estimates are systematically low compared to administrative data and estimates from other established surveys series (Sudan Multiple Indicator Cluster Survey 2010). Survey removed from analysis.
153643	Sudan	Sudan - North Multiple Indicator Cluster Survey 2010	2010	select	BCG, MCV1	Central Bureau of Statistics (Sudan), Ministry of Health (South Sudan). Sudan - North Multiple Indicator Cluster Survey 2010. New York, United States of America: United Nations Children's Fund (UNICEF), 2015.	Survey removed from analysis due to small sample size and reliability concerns.
200617	Sudan	Sudan Multiple Indicator Cluster Survey 2014	2014	all	MCV2	Central Bureau of Statistics (Sudan), Federal Ministry of Health (Sudan), United Nations Children's Fund (UNICEF). Sudan Multiple Indicator Cluster Survey 2014. New York, United States of America: United Nations Children's Fund (UNICEF), 2016.	Survey reports implausibly high MCV2 coverage in introduction year. Select age-cohorts removed from analysis for MCV2.

GHDx ID	Country	Series	Year(s)	Cohort	Vaccine(s)	Citation	Rationale for exclusion
20813	Sudan	Sudan Demographic and Health Survey 1989-1990	1989-1990	all	BCG, DTP1, DTP3, MCV1, Pol3	Ministry of Finance and Economic Planning, Department of Statistics, Macro Systems, Inc.; Institute for Resource Development. Sudan Demographic and Health Survey 1989-1990. Columbia, United States: Macro Systems, Inc.	Estimates considered implausible. Survey estimates are systematically high compared to administrative data and estimates from other established surveys series. Survey removed from analysis.
12259	Sudan	Sudan Maternal and Child Health Survey 1992-1993	1992-1993	all	BCG, DTP3, MCV1, Pol3	Federal Ministry of Health (Sudan), League of Arab States. Sudan Maternal and Child Health Survey 1992-1993.	Estimates considered implausible. Survey estimates are systematically high compared to administrative data and estimates from other established surveys series (Sudan Multiple Indicator Survey 2000). Survey removed from analysis.
81203	Suriname	Suriname Multiple Indicator Cluster Survey 2010	2010	all	DTP1, DTP3, HepB3	General Bureau of Statistics (Suriname), Ministry of Planning and Development Cooperation (Suriname), Ministry of Social Affairs and Housing (Suriname), United Nations Children's Fund (UNICEF). Suriname Multiple Indicator Cluster Survey 2010. New York, United States of America: United Nations Children's Fund (UNICEF), 2013.	Estimates considered implausible. DTP1, DTP3, HepB3 estimates are systematically low compared to administrative data and estimates from other established surveys series. DTP1, DTP3, HepB3 removed from analysis.
12389	Syria	Syria Multiple Indicator Cluster Survey 1995	1995	all	Hib3	United Nations Children's Fund (UNICEF). Syria Multiple Indicator Cluster Survey 1995.	Survey precedes introduction of Hib3 to national immunization schedule. Hib3 removed from analysis.
12379	Syria	Syria Family Health Survey 2001	2001	select	RCV1	Central Bureau of Statistics (Syria), League of Arab States. Syria Family Health Survey 2001.	Older age-cohorts precede introduction of RCV1 to national immunization schedule. Select age-cohorts removed from analysis for RCV1.
10023	Syria	Palestinians in Syria Multiple Indicator Cluster Survey 2006	2006	all	BCG, DTP1, DTP3, HepB3, MCV1, Pol3	General Administration for Palestine Arab Refugees (GAPAR), Palestinian Central Bureau of Statistics, Pan Arab Project for Family Health (PAPFAM), United Nations Children's Fund (UNICEF). Palestinians in Syria Multiple Indicator Cluster Survey 2006.	Survey is not nationally representative. Survey removed from analysis.
229595*	Syrian Arab Republic	Syria Immunization Campaign 1986	1986	select	DTP3, MCV1, Pol3	Syria Immunization Campaign 1986.	Selected cohorts excluded from administrative data bias modeling as a methodological decision to improve model performance

GHDx ID	Country	Series	Year(s)	Cohort	Vaccine(s)	Citation	Rationale for exclusion
20875	Tanzania	Tanzania Demographic and Health Survey 2004-2005	2004-2005	all	HepB3, PCV3	Macro International, Inc, National Bureau of Statistics (Tanzania). Tanzania Demographic and Health Survey 2004-2005. Fairfax, United States of America: ICF International.	Survey precedes introduction of HepB3 and PCV3 in national immunization schedule. HepB3 and PCV3 removed from analysis.
21331	Tanzania	Tanzania Demographic and Health Survey 2009-2010	2009-2010	all	HepB3, PCV3	ICF Macro, National Bureau of Statistics (Tanzania). Tanzania Demographic and Health Survey 2009-2010. Fairfax, United States of America: ICF International.	Estimates considered implausible. Survey estimates are systematically low compared to administrative data and estimates from other established surveys series. Survey removed from analysis.
218593	Tanzania	Tanzania Demographic and Health Survey 2015-2016	2015-2016	all	MCV2	ICF International, Ministry of Health (Zanzibar), Ministry of Health, Community Development, Gender, Elderly and Children (MoHCDEC) (Tanzania), National Bureau of Statistics (Tanzania), Office of the Chief Government Statistician (OCGS) (Zanzibar). Tanzania Demographic and Health Survey 2015-2016. Fairfax, United States of America: ICF International, 2016.	Older age-cohorts precede introduction of MCV2 to national immunization schedule. Select age-cohorts removed from analysis for MCV2.
20887*	Thailand	Thailand Demographic and Health Survey 1987	1987	select	MCV1	Chulalongkorn University, Institute of Population Studies (Thailand), Westinghouse; Institute for Resource Development. Thailand Demographic and Health Survey 1987. Fairfax, United States of America: ICF International.	Older age-cohorts precede introduction of MCV1 to national immunization schedule. Select age-cohorts removed from analysis for MCV1. In addition, selected cohorts excluded from administrative data bias modeling as a methodological decision to improve model performance
20887*	Thailand	Thailand Demographic and Health Survey 1987	1987	select	MCV1	Chulalongkorn University, Institute of Population Studies (Thailand), Westinghouse; Institute for Resource Development. Thailand Demographic and Health Survey 1987. Fairfax, United States of America: ICF International.	Selected cohorts excluded from administrative data bias modeling as a methodological decision to improve model performance

GHDx ID	Country	Series	Year(s)	Cohort	Vaccine(s)	Citation	Rationale for exclusion
148649	Thailand	Thailand Multiple Indicator Cluster Survey 2012	2012	select	HepB3	College of Population Studies, Chulalongkorn University (Thailand), Institute for Population and Social Research, Mahidol University (Thailand), International Health Policy Program (Thailand), Ministry of Education (Thailand), Ministry of Public Health (Thailand), Ministry of Social Development and Human Security (MSDHS) (Thailand), National Health Security Office (Thailand), National Statistical Office (Thailand), Thai Health Promotion Foundation, United Nations Children's Fund (UNICEF). Thailand Multiple Indicator Cluster Survey 2012. New York, United States of America: United Nations Children's Fund (UNICEF), 2016.	Older age-cohorts for HepB3 are implausibly low compared to administrative data and estimates from other established surveys series (Thailand Multiple Indicator Cluster Survey 2012). Select age-cohorts removed from analysis for HepB3.
91506	The Gambia	Gambia Multiple Indicator Cluster Survey 2010	2010	select	HepB3, PCV3	Gambia Bureau of Statistics (GBOS), United Nations Children's Fund (UNICEF). Gambia Multiple Indicator Cluster Survey 2010. New York, United States of America: United Nations Children's Fund (UNICEF), 2018.	HepB3, PCV3 estimates are systematically low compared to administrative data and estimates from other established surveys series (Gambia Demographic and Health Survey 2013). HepB3, PCV3 removed from analysis.
77384	The Gambia	Gambia Demographic and Health Survey 2013	2013	all	MCV2	Gambia Bureau of Statistics (GBOS), ICF International, Ministry of Health and Social Welfare (The Gambia). Gambia Demographic and Health Survey 2013. Fairfax, United States of America: ICF International, 2015.	Survey precedes introduction of MCV2 to national immunization schedule. MCV2 removed from analysis.
20888	Timor-Leste	Timor-Leste Demographic and Health Survey 2003	2003	all	HepB3	ACIL Australia Pty Ltd., Australian National University, Ministry of Health (Timor-Leste), National Statistics Directorate (Timor-Leste), University of Newcastle (Australia). Timor-Leste Demographic and Health Survey 2003. Newcastle, Australia: University of Newcastle (Australia).	Survey precedes introduction of HepB3 in national immunization schedule. HepB3 removed from analysis.

GHDx ID	Country	Series	Year(s)	Cohort	Vaccine(s)	Citation	Rationale for exclusion
21274	Timor-Leste	Timor-Leste Demographic and Health Survey 2009-2010	2009-2010	select	HepB3	ICF Macro, Ministry of Finance (Timor-Leste), National Statistics Directorate (Timor-Leste). Timor-Leste Demographic and Health Survey 2009-2010. Fairfax, United States of America: ICF International.	Older age-cohorts precede introduction of HepB3 to national immunization schedule. Select age-cohorts removed from analysis for HepB3.
20909*	Togo	Togo Demographic and Health Survey 1998	1998	select	BCG1, DTP1, DTP3, MCV1, Pol3	Department of Statistics (Togo), Macro International, Inc. Togo Demographic and Health Survey 1998. Fairfax, United States of America: ICF International.	Selected cohorts excluded from administrative data bias modeling as a methodological decision to improve model performance
12886*	Togo	Togo Multiple Indicator Cluster Survey 2000	2000	select	BCG1, DTP1, DTP3, MCV1, Pol3	United Nations Children's Fund (UNICEF). Togo Multiple Indicator Cluster Survey 2000. New York, United States of America: United Nations Children's Fund (UNICEF).	Selected cohorts excluded from administrative data bias modeling as a methodological decision to improve model performance
40021	Togo	Togo Multiple Indicator Cluster Survey 2010	2010	select	HepB3, Hib3	Directorate General of Statistics and National Accounting (Togo), United Nations Children's Fund (UNICEF). Togo Multiple Indicator Cluster Survey 2010. New York, United States: United Nations Children's Fund (UNICEF).	Older age-cohorts precede introduction of HepB3 and Hib3 to national immunization schedule. Select age-cohorts removed from analysis for HepB3 and Hib3.
229471	Tonga	Tonga Demographic and Health Survey 2012	2012	all	BCG, DTP1, DTP3, MCV1, Pol3	Macro International, Inc, Ministry of Health (Tonga), Secretariat of the Pacific Community (SPC), Tonga Department of Statistics. Tonga Demographic and Health Survey 2012.	Estimates considered implausible. Survey estimates are systematically low compared to administrative data and estimates from other established surveys series. Survey removed from analysis.
453346	Tonga	Tonga Multiple Indicator Cluster Survey 2019	2019	all	BCG, DTP1, DTP3, HepB3, PCV3, MCV1, MCV2, Pol3, RCV1	Government of Tonga, Pacific Community Statistics for Development Division, Tonga Department of Statistics, United Nations Children's Fund (UNICEF), United Nations Population Fund (UNFPA). Tonga Multiple Indicator Cluster Survey 2019. New York, United States of America: United Nations Children's Fund (UNICEF), 2020.	Survey removed from analysis due to small sample size and reliability concerns.

GHDx ID	Country	Series	Year(s)	Cohort	Vaccine(s)	Citation	Rationale for exclusion
20917	Trinidad and Tobago	Trinidad and Tobago Demographic and Health Survey 1987	1987	all	DTP1, DTP3, MCV1, Pol3	Family Planning Association (Trinidad and Tobago), Westinghouse; Institute for Resource Development. Trinidad and Tobago Demographic and Health Survey 1987. Fairfax, United States of America: ICF International.	Estimates considered implausible. Survey estimates are systematically high compared to administrative data and estimates from other established surveys series. Survey removed from analysis.
12940	Trinidad and Tobago	Trinidad and Tobago Multiple Indicator Cluster Survey 2000	2000	all	DTP1, DTP3, MCV1, Pol3	Central Statistical Office (Trinidad and Tobago), United Nations Children's Fund (UNICEF). Trinidad and Tobago Multiple Indicator Cluster Survey 2000. New York, United States of America: United Nations Children's Fund (UNICEF).	Estimates considered implausible. Survey estimates are systematically low compared to administrative data and estimates from other established surveys series (Trinidad and Tobago Multiple Indicator Cluster Survey 2006). Survey removed from analysis.
12950	Trinidad and Tobago	Trinidad and Tobago Multiple Indicator Cluster Survey 2006	2006	select	HepB3, Hib3	Central Statistical Office (Trinidad and Tobago) and United Nations Children's Fund (UNICEF). Trinidad and Tobago Multiple Indicator Cluster Survey 2006. New York, United States: United Nations Children's Fund (UNICEF).	Older age-cohorts of HepB3 and all age-cohorts of Hib3 precede introduction to national immunization schedule. Hib3 and select age-cohorts of HepB3 removed from analysis.
332558	Trinidad and Tobago	Trinidad and Tobago Multiple Indicator Cluster Survey 2011	2011	all	PCV3, Pol3	Central Statistical Office (Trinidad and Tobago), Ministry of Social Development and Family Services (Trinidad and Tobago), United Nations Children's Fund (UNICEF). Trinidad and Tobago Multiple Indicator Cluster Survey 2011. New York, United States of America: United Nations Children's Fund (UNICEF), 2018.	Survey precedes introduction of PCV to national immunization schedule. Pol3 estimates are implausibly high compared to administrative data. PCV and Pol3 removed from analysis.
20926	Tunisia	Tunisia Demographic and Health Survey 1988	1988	all	BCG, DTP1, DTP3, MCV1, Pol3	Macro Systems, Inc.; Institute for Resource Development, National Office for Family and Population, Ministry of Public Health (Tunisia). Tunisia Demographic and Health Survey 1988. Fairfax, United States of America: ICF International.	Survey estimates are implausibly high compared to administrative data and estimates from other established survey series. Survey removed from analysis.
12983	Tunisia	Tunisia Multiple Indicator Cluster Survey 2000	2000	all	PCV3, MCV1	Ministry of Public Health (Tunisia), United Nations Children's Fund (UNICEF). Tunisia Multiple Indicator Cluster Survey 2000.	MCV1 estimates are systematically low compared to administrative data and estimates from other established surveys series. MCV1 removed from analysis.

GHDx ID	Country	Series	Year(s)	Cohort	Vaccine(s)	Citation	Rationale for exclusion
409558	Tunisia	Tunisia Multiple Indicator Cluster Survey 2018	2018	all	BCG, DTP1, DTP3, HepB3, PCV3, MCV1, MCV2, PCV3, Pol3, RCV1	National Institute of Statistics (Tunisia), United Nations Children's Fund (UNICEF). Tunisia Multiple Indicator Cluster Survey 2018. New York, United States of America: United Nations Children's Fund (UNICEF), 2019.	Estimates considered implausible. Survey estimates are systematically low compared to administrative data and estimates from other established surveys series. Survey removed from analysis.
546694	Tunisia	Tunisia Multiple Indicator Cluster Survey 2023	2023	all	MCV2, Pol3	National Institute of Statistics (Tunisia), United Nations Children's Fund (UNICEF). Tunisia Multiple Indicator Cluster Survey 2023. New York, United States of America: United Nations Children's Fund (UNICEF), 2023.	MCV2 and Pol3 estimates considered implausible. Survey estimates are inconsistent with administrative data and estimates from other established survey series. MCV2 and Pol3 removed from analysis.
228736*	Türkiye	Turkey National EPI Program Review 1988	1988	select	BCG1	Turkey National EPI Program Review 1988.	Selected cohorts excluded from administrative data bias modeling as a methodological decision to improve model performance
228654*	Türkiye	Turkey Immunisation Coverage Survey 1989	1989	select	BCG1	Turkey Immunisation Coverage Survey 1989.	Selected cohorts excluded from administrative data bias modeling as a methodological decision to improve model performance
20947	Türkiye	Turkey Demographic and Health Survey 1998	1998	all	HepB3	Institute of Population Studies, Hacettepe University, Macro International, Inc. Turkey Demographic and Health Survey 1998. Fairfax, United States of America: ICF International.	Survey precedes introduction of HepB to national immunization schedule. HepB removed from analysis.
223669	Türkiye	Turkey Demographic and Health Survey 2013-2014	2013-2014	all	HepB3, PCV3, PCV3	Institute of Population Studies, Hacettepe University, Ministry of Development (Turkey), Ministry of Health (Turkey). Turkey Demographic and Health Survey 2013-2014. Fairfax, United States of America: ICF International, 2019.	Estimates considered implausible. Survey estimates are systematically low compared to administrative data and estimates from other established surveys series (Turkey Demographic and Health Survey 2008). Survey removed from analysis.
440151	Türkiye	Turkey Demographic and Health Survey 2018-2019	2018-2019	all	DTP1, DTP3, HepB3, PCV3, PCV3	Institute of Population Studies, Hacettepe University, Ministry of Development (Turkey), Ministry of Health (Turkey). Turkey Demographic and Health Survey 2018-2019.	Estimates considered implausible. Survey estimates are systematically low compared to administrative data and estimates from other established surveys series. Survey removed from analysis.
18818	Tuvalu	Tuvalu Demographic and Health Survey 2007	2007	all	BCG, DTP1, DTP3, MCV1, Pol3	Macro International, Inc, Secretariat of the Pacific Community (SPC), Tuvalu Central Statistics Division. Tuvalu Demographic and Health Survey 2007.	Estimates considered implausible. Survey estimates are systematically low compared to administrative data and estimates from other established surveys series. Survey removed from analysis.

GHDx ID	Country	Series	Year(s)	Cohort	Vaccine(s)	Citation	Rationale for exclusion
23289	Uganda	Uganda Child Verbal Autopsy Study 2007	2007	select	BCG, DTP1, DTP3, HepB3, PCV3, MCV1, Pol3	MEASURE Evaluation Project, Carolina Population Center, University of North Carolina, Macro International, Inc, Ministry of Health (Uganda), Uganda Bureau of Statistics. Uganda Child Verbal Autopsy Study 2007. Calverton, United States of America: Macro International, Inc.	Survey removed from analysis due to small sample size and reliability concerns.
20964*	Uganda	Uganda Demographic and Health Survey 1988-1989	1988-1989	select	DTP1, DTP3, MCV1, Pol3	Macro Systems, Inc.; Institute for Resource Development, Makerere University, Ministry of Health (Uganda). Uganda Demographic and Health Survey 1988-1989. Fairfax, United States of America: ICF International.	Selected cohorts excluded from administrative data bias modeling as a methodological decision to improve model performance
13084	Uganda	Uganda AIDS Indicator Survey 2004-2005	2004-2005	select	HepB3	Division of Reproductive Health, Centers for Disease Control and Prevention (CDC), Ministry of Health (Uganda). Uganda AIDS Indicator Survey 2004-2005.	Older age-cohorts precede introduction of HepB3 to national immunization schedule. Select age-cohorts removed from analysis for HepB3.
81004	Uganda	Uganda Living Standards Measurement Survey - Integrated Survey on Agriculture 2009-2010	2009-2010	select	DTP1, MCV1	Uganda Bureau of Statistics. Uganda Living Standards Measurement Survey - Integrated Survey on Agriculture 2009-2010. Washington DC, United States of America: World Bank.	Survey removed from analysis due to small sample size and reliability concerns.
264959	Uganda	Uganda National Panel Survey 2013-2014	2013-2014	select	DTP1, MCV1	Government of the Netherlands, Uganda Bureau of Statistics, World Bank. Uganda National Panel Survey 2013-2014. Washington DC, United States of America: World Bank, 2022.	Survey removed from analysis due to small sample size and reliability concerns.
132739*	Ukraine	Ukraine Multiple Indicator Cluster Survey 2012	2012	select	BCG1, DTP1, DTP3, MCV1, Pol3	StatInform Consulting, State Statistics Service (Ukraine), Ukrainian Center for Social Reforms (UCSR), United Nations Children's Fund (UNICEF). Ukraine Multiple Indicator Cluster Survey 2012. New York, United States of America: United Nations Children's Fund (UNICEF), 2014.	Selected cohorts excluded from administrative data bias modeling as a methodological decision to improve model performance

GHDx ID	Country	Series	Year(s)	Cohort	Vaccine(s)	Citation	Rationale for exclusion
13224	United Arab Emirates	United Arab Emirates Child Health Survey 1987	1987	select	BCG, DTP1, DTP3, MCV1, Pol3	Ministry of Health and Prevention (United Arab Emirates). United Arab Emirates Child Health Survey 1987.	Estimates considered implausible. Survey estimates do not follow a reasonable age structure. Older children have significantly higher coverage than reported in either administrative data or other survey series, suggesting survey is capturing results of catch-up immunization. Select age cohorts removed from analysis.
467505	United Arab Emirates	United Arab Emirates World Health Survey 2017-2018	2017-2018	select	MCV2, Pol3, RotaC	Abu Dhabi Department of Health (United Arab Emirates), Dubai Health Authority, Dubai Statistics Center (United Arab Emirates), Federal Competitiveness and Statistics Authority (United Arab Emirates), IQVIA, Ministry of Health and Prevention (United Arab Emirates), Statistics Centre Abu Dhabi. United Arab Emirates World Health Survey 2017-2018.	Older age-cohorts precede introduction of these vaccines to national immunization schedule. Select age cohorts removed from analysis for these vaccines.
40330	USA	United States National Health Interview Survey 1991	1991	all	DTP1, DTP3, PCV3, MCV1, Pol3	National Center for Health Statistics (NCHS), Centers for Disease Control and Prevention (CDC), US Census Bureau. United States National Health Interview Survey 1991. Hyattsville, United States: National Center for Health Statistics (NCHS), Centers for Disease Control and Prevention (CDC).	Survey estimates are systematically low and suggest an implausible drop in coverage in the 1980's and 1990's. Survey removed from analysis.
40749	USA	United States National Health Interview Survey 1992	1992	all	DTP1, DTP3, HepB3, PCV3, MCV1, Pol3	National Center for Health Statistics (NCHS), Centers for Disease Control and Prevention (CDC), US Census Bureau. United States National Health Interview Survey 1992. Hyattsville, United States: National Center for Health Statistics (NCHS), Centers for Disease Control and Prevention (CDC).	Survey estimates are systematically low and suggest an implausible drop in coverage in the 1980's and 1990's. Survey removed from analysis.

GHDx ID	Country	Series	Year(s)	Cohort	Vaccine(s)	Citation	Rationale for exclusion
40766	USA	United States National Health Interview Survey 1993	1993	all	DTP1, DTP3, PCV3, MCV1, Pol3	National Center for Health Statistics (NCHS), Centers for Disease Control and Prevention (CDC), US Census Bureau. United States National Health Interview Survey 1993. Hyattville, United States: National Center for Health Statistics (NCHS), Centers for Disease Control and Prevention (CDC).	Survey estimates are systematically low and suggest an implausible drop in coverage in the 1980's and 1990's. Survey removed from analysis.
40821	USA	United States National Health Interview Survey 1994	1994	all	DTP1, DTP3, HepB3, PCV3, MCV1, Pol3	National Center for Health Statistics (NCHS), Centers for Disease Control and Prevention (CDC), US Census Bureau. United States National Health Interview Survey 1994. Hyattsville, United States: National Center for Health Statistics (NCHS), Centers for Disease Control and Prevention (CDC).	Survey estimates are systematically low and suggest an implausible drop in coverage in the 1980's and 1990's. Survey removed from analysis.
84752	USA	United States National Immunization Survey-Child 2001-2002	2001-2002	select	PCV3	Abt Associates Inc., National Center for Health Statistics, Centers for Disease Control and Prevention, National Center for Immunization and Respiratory Diseases, CDC (NCIRD). United States National Immunization Survey-Child 2001-2002. Hyattsville, United States of America: National Center for Health Statistics, Centers for Disease Control and Prevention.	Older age-cohorts of PCV3 and all age-cohorts of RotaC precede introduction to national immunization schedule. PCV3 and select age-cohorts of RotaC removed from analysis.
84707	USA	United States National Immunization Survey-Child 2006-2007	2006-2007	all	RotaC	National Center for Health Statistics, Centers for Disease Control and Prevention, National Center for Immunization and Respiratory Diseases, CDC (NCIRD), National Opinion Research Center (NORC) (United States). United States National Immunization Survey-Child 2006-2007. Hyattsville, United States of America: National Center for Health Statistics, Centers for Disease Control and Prevention.	Survey precedes introduction of rotavirus vaccine to national immunization schedule. Rotavirus vaccine removed from analysis.

GHDx ID	Country	Series	Year(s)	Cohort	Vaccine(s)	Citation	Rationale for exclusion
84694	USA	United States National Immunization Survey-Child 2007-2008	2007-2008	select	RotaC	National Center for Health Statistics, Centers for Disease Control and Prevention, National Center for Immunization and Respiratory Diseases, CDC (NCIRD), National Opinion Research Center (NORC) (United States). United States National Immunization Survey-Child 2007-2008. Hyattsville, United States of America: National Center for Health Statistics, Centers for Disease Control and Prevention.	Older age-cohorts precede introduction of rotavirus vaccine to national immunization schedule. Select age-cohorts removed from analysis for rotavirus vaccine.
561483	Vanuatu	Vanuatu Multiple Indicator Cluster Survey 2023	2023	all	MCV2	Bureau of Statistics (Vanuatu), New Zealand Aid Programme, Secretariat of the Pacific Community (SPC), United Nations Children's Fund (UNICEF), United Nations Population Fund (UNFPA). Vanuatu Multiple Indicator Cluster Survey 2023. New York, United States of America: United Nations Children's Fund (UNICEF), 2024.	Survey precedes introduction of MCV2 to national immunization schedule. MCV2 removed from analysis.
13465*	Vanuatu	Vanuatu Multiple Indicator Cluster Survey 2007-2008	2007-2008	all	RCV1	Ministry of Health (Vanuatu), United Nations Children's Fund (UNICEF). Vanuatu Multiple Indicator Cluster Survey 2007-2008. New York, United States of America: United Nations Children's Fund (UNICEF).	Survey precedes introduction of RCV1 to national immunization schedule. RCV1 removed from analysis. In addition, selected cohorts excluded from administrative data bias modeling as a methodological decision to improve model performance
13465*	Vanuatu	Vanuatu Multiple Indicator Cluster Survey 2007-2008	2007-2008	select	BCG1, DTP1, DTP3, MCV1, Pol3	Ministry of Health (Vanuatu), United Nations Children's Fund (UNICEF). Vanuatu Multiple Indicator Cluster Survey 2007-2008. New York, United States of America: United Nations Children's Fund (UNICEF).	Selected cohorts excluded from administrative data bias modeling as a methodological decision to improve model performance
13516*	Venezuela (Bolivarian Republic of)	Venezuela Multiple Indicator Cluster Survey 2000	2000	select	DTP1	National Institute of Statistics (Venezuela), United Nations Children's Fund (UNICEF). Venezuela Multiple Indicator Cluster Survey 2000. New York, United States: United Nations Children's Fund (UNICEF).	Selected cohorts excluded from administrative data bias modeling as a methodological decision to improve model performance

GHDx ID	Country	Series	Year(s)	Cohort	Vaccine(s)	Citation	Rationale for exclusion
228331	Viet Nam	Vietnam Children Indicators 2001	2001	all	BCG, DTP3, MCV1, Pol3	Vietnam Children Indicators 2001.	Estimates considered implausible. Survey estimates are systematically high compared to administrative data and estimates from other established surveys series (Vietnam Demographic and Health Survey 2002). Survey removed from analysis.
228239	Yemen	Yemen Immunization, Diarrhoeal Disease, Maternal and Child Mortality Survey 1990	1990	all	BCG, DTP1, DTP3, MCV1, Pol3	Yemen Immunization, Diarrhoeal Disease, Maternal and Child Mortality Survey 1990.	Estimates considered implausible. Survey estimates are systematically high compared to administrative data and estimates from other established surveys series. Survey removed from analysis.
13795	Yemen	Yemen Family Health Survey 2003	2003	all	Pol3	Central Statistical Organization (Yemen), League of Arab States, Ministry of Public Health and Population (Yemen), Pan Arab Project for Family Health (PAPFAM). Yemen Family Health Survey 2003.	Pol3 estimates are systematically low compared to administrative data and estimates from other established surveys series (Yemen Household Budget Survey 2005-2006). Pol3 removed from analysis.
13816	Yemen	Yemen Multiple Indicator Cluster Survey 2006	2006	select	Hib3	Ministry of Health (Yemen) and United Nations Children's Fund (UNICEF). Yemen Multiple Indicator Cluster Survey 2006. New York, United States: United Nations Children's Fund (UNICEF).	Older age-cohorts precede introduction of Hib3 to national immunization schedule. Select age-cohorts removed from analysis for Hib3.
112500	Yemen	Yemen Demographic and Health Survey 2013	2013	select	PCV3	Central Statistical Organization (Yemen), ICF International, Ministry of Public Health and Population (Yemen). Yemen Demographic and Health Survey 2013. Fairfax, United States of America: ICF International.	Older age-cohorts precede introduction of PCV3 to national immunization schedule. Select age-cohorts removed from analysis for PCV3.
21068	Yemen	Yemen Demographic and Health Survey 1991-1992	1991-1992	all	BCG, DTP1, DTP3, MCV1, Pol3	Central Statistical Organization (Yemen), League of Arab States, Macro International, Inc. Yemen Demographic and Health Survey 1991-1992. Fairfax, United States of America: ICF International.	Estimates considered implausible. Survey estimates are systematically high compared to administrative data and estimates from other established surveys series. Survey removed from analysis.
249499	Yemen	Yemen National Social Protection Monitoring Survey 2012-2013	2012-2013	all	BCG, DTP1, DTP3, HepB3, PCV3, MCV1, MCV2, PCV3, Pol3	Ministry of Planning and International Cooperation (Yemen), International Policy Center for Inclusive Growth, Interaction in Development (Yemen), UNICEF Yemen. Yemen National Social Protection Monitoring Survey 2012-2013.	Survey estimates include implausibly high coverage rates of 100%. Survey removed from analysis.

GHDx ID	Country	Series	Year(s)	Cohort	Vaccine(s)	Citation	Rationale for exclusion
13973	Zambia	Zambia Living Conditions Monitoring Survey 1996	1996	all	MCV2	Central Statistical Office (Zambia). Zambia Living Conditions Monitoring Survey 1996. Lusaka, Zambia: Central Statistical Office (Zambia).	Survey precedes introduction of MCV2 to national immunization schedule. MCV2 removed from analysis.
14015	Zambia	Zambia Living Conditions Monitoring Survey 1998	1998	all	MCV2	Central Statistical Office (Zambia), London School of Hygiene and Tropical Medicine. Zambia Living Conditions Monitoring Survey 1998. Lusaka, Zambia: Central Statistical Office (Zambia).	Survey precedes introduction of MCV2 to national immunization schedule. MCV2 removed from analysis.
14122	Zambia	Zambia Multiple Indicator Cluster Survey 1999	1999	all	BCG	Central Statistical Office (Zambia), Food Security, Health and Nutrition Information System (Zambia), United Nations Children's Fund (UNICEF). Zambia Multiple Indicator Cluster Survey 1999. New York, United States of America: United Nations Children's Fund (UNICEF).	BCG estimates are implausibly low compared to administrative data and estimates from other established surveys series for some age-cohorts (Zambia Living Conditions Monitoring Survey 1998). Select age-cohorts removed from analysis for BCG.
21117	Zambia	Zambia Demographic and Health Survey 2007	2007	select	HepB3, Hib3	Central Statistical Office (Zambia), Macro International, Inc. Zambia Demographic and Health Survey 2007. Fairfax, United States of America: ICF International.	Older age-cohorts precede introduction of HepB3 and Hib3 to national immunization schedule. Select age-cohorts removed from analysis for HepB3 and Hib3.
26702	Zambia	Zambia Global Fund Household Health Coverage Survey 2008	2008	select	DTP1, DTP3, HepB3	Central Statistical Office (Zambia). Zambia Global Fund Household Health Coverage Survey 2008. Lusaka, Zambia: Central Statistical Office (Zambia).	DTP3 estimates are implausibly low compared to administrative data and estimates from other established surveys series for some age-cohorts (Zambia Demographic and Health Survey 2007). Older age-cohorts precede introduction of HepB3 to national immunization schedule. Select age-cohorts removed from analysis for HepB3 and DTP3.
58660	Zambia	Zambia Living Conditions Monitoring Survey 2010	2010	select	BCG, DTP1, DTP3, MCV1, MCV2, Pol3	Central Statistical Office (Zambia). Zambia Living Conditions Monitoring Survey 2010.	Older age-cohorts precede introduction of these vaccines to national immunization schedule. Select age cohorts removed from analysis for these vaccines.
284177	Zambia	Zambia Living Conditions Monitoring Survey 2015	2015	all	MCV2	Central Statistical Office (Zambia), World Bank. Zambia Living Conditions Monitoring Survey 2015.	Survey precedes introduction of MCV2 to national immunization schedule. MCV2 removed from analysis.

GHDx ID	Country	Series	Year(s)	Cohort	Vaccine(s)	Citation	Rationale for exclusion
14027	Zambia	Zambia Living Conditions Monitoring Survey 2002-2003	2002-2003	all	MCV2	Central Statistical Office (Zambia). Zambia Living Conditions Monitoring Survey 2002-2003. Lusaka, Zambia: Central Statistical Office (Zambia).	Survey precedes introduction of MCV2 to national immunization schedule. MCV2 removed from analysis.
14063	Zambia	Zambia Living Conditions Monitoring Survey 2004-2005	2004-2005	all	MCV2	Central Statistical Office (Zambia). Zambia Living Conditions Monitoring Survey 2004-2005. Lusaka, Zambia: Central Statistical Office (Zambia).	Survey precedes introduction of MCV2 to national immunization schedule. MCV2 removed from analysis.
152720	Zimbabwe	Zimbabwe Multiple Indicator Cluster Survey 2014	2014	select	PCV3	United Nations Children's Fund (UNICEF), Zimbabwe National Statistics Agency. Zimbabwe Multiple Indicator Cluster Survey 2014. New York, United States of America: United Nations Children's Fund (UNICEF), 2015.	Older age-cohorts precede introduction of PCV3 to national immunization schedule. Select age-cohorts removed from analysis for PCV3.
157066	Zimbabwe	Zimbabwe Demographic and Health Survey 2015	2015	select	RotaC	ICF International, National Microbiology Reference Laboratory, Harare Central Hospital (NMRL) (Zimbabwe), Zimbabwe National Statistics Agency. Zimbabwe Demographic and Health Survey 2015. Fairfax, United States of America: ICF International, 2016.	Older age-cohorts precede introduction of RotaC to national immunization schedule. Select age-cohorts removed from analysis for RotaC.
21163	Zimbabwe	Zimbabwe Demographic and Health Survey 2005-2006	2005-2006	all	MCV2	Central Statistical Office (Zimbabwe), Macro International, Inc. Zimbabwe Demographic and Health Survey 2005-2006. Fairfax, United States of America: ICF International.	Survey precedes introduction of MCV2 to national immunization schedule. MCV2 removed from analysis.
55992	Zimbabwe	Zimbabwe Demographic and Health Survey 2010-2011	2010-2011	select	Hib3	ICF Macro, Zimbabwe National Statistics Agency. Zimbabwe Demographic and Health Survey 2010-2011. Calverton, United States of America: ICF Macro, 2012.	Older age-cohorts precede introduction of Hib3 to national immunization schedule. Select age-cohorts removed from analysis for Hib3.

Table S4 Supplemental stockout and disruption events

Supplemental disruption events in national and subnational units, disruption meaning vaccine-specific acute disruption to vaccine delivery.

Country	Year	Reason for disruption	Source
Afghanistan	1994	Discontinuity in administrative data assumed to be stockout based upon review of historical WHO/UNICEF Review of National Immunization Coverage 1980-2008. Donor support beginning in 1994 led to an increase in coverage through 1997.	United Nations Children's Fund (UNICEF), World Health Organization (WHO). WHO Immunization Data Portal - WHO and UNICEF Estimates of National Immunization Coverage 1980-2020 as of July 15 2021. Geneva, Switzerland: World Health Organization (WHO), 2021.
Angola	1999	The resurgence of the war in December 1998 weakened the Ministry of Health's capacity to carry out immunization activities. In 1999 only 18 out of 164 districts reported.	United Nations Children's Fund (UNICEF), World Health Organization (WHO). WHO Immunization Data Portal - WHO and UNICEF Estimates of National Immunization Coverage 1980-2020 as of July 15 2021. Geneva, Switzerland: World Health Organization (WHO), 2021.
Bahrain	2010	Rotavirus vaccine was suspended from March to June, leading to a disruption in vaccine coverage	United Nations Children's Fund (UNICEF), World Health Organization (WHO). WHO Immunization Data Portal - WHO and UNICEF Estimates of National Immunization Coverage 1980-2020 as of July 15 2021. Geneva, Switzerland: World Health Organization (WHO), 2021.
Barbados	2001	Vaccine disruption caused by introduction year of HepB and Hib	World Health Organization (WHO). Introduction of Hepatitis B vaccine - WHO Immunization Data Portal. Geneva, Switzerland: World Health Organization (WHO), 2021.
Belarus	2003	Vaccine shortage	United Nations Children's Fund (UNICEF), World Health Organization (WHO). WHO Immunization Data Portal - WHO and UNICEF Estimates of National Immunization Coverage 1980-2020 as of July 15 2021. Geneva, Switzerland: World Health Organization (WHO), 2021.
Bosnia and Herzegovina	1992	Conflict / War / Civil unrest	Centers for Disease Control and Prevention. (1995). Update: Vaccine side effects, adverse reactions, contraindications, and precautions: Recommendations of the Advisory Committee on Immunization Practices (ACIP). MMWR. Retrieved January 24, 2025, from https://www.cdc.gov/mmwr/preview/mmwrhtml/00031513.htm
Bosnia and Herzegovina	1993	Conflict / War / Civil unrest	Centers for Disease Control and Prevention. (1995). Update: Vaccine side effects, adverse reactions, contraindications, and precautions: Recommendations of the Advisory Committee on Immunization Practices (ACIP). MMWR. Retrieved January 24, 2025, from https://www.cdc.gov/mmwr/preview/mmwrhtml/00031513.htm
Bosnia and Herzegovina	1994	Conflict / War / Civil unrest	Centers for Disease Control and Prevention. (1995). Update: Vaccine side effects, adverse reactions, contraindications, and precautions: Recommendations of the Advisory Committee on Immunization Practices (ACIP). MMWR. Retrieved January 24, 2025, from https://www.cdc.gov/mmwr/preview/mmwrhtml/00031513.htm
Bosnia and Herzegovina	1995	Conflict / War / Civil unrest	Centers for Disease Control and Prevention. (1995). Update: Vaccine side effects, adverse reactions, contraindications, and precautions: Recommendations of the Advisory Committee on Immunization Practices (ACIP). MMWR. Retrieved January 24, 2025, from https://www.cdc.gov/mmwr/preview/mmwrhtml/00031513.htm
Brazil	2017	One-month vaccine stock-out caused a temporary disruption in vaccine availability and coverage	United Nations Children's Fund (UNICEF), World Health Organization (WHO). WHO Immunization Data Portal - WHO and UNICEF Estimates of National Immunization Coverage 1980-2020 as of July 15 2021. Geneva, Switzerland: World Health Organization (WHO), 2021.

Country	Year	Reason for disruption	Source
Central African Republic	1987	Conflict / War / Civil unrest	United Nations Children's Fund (UNICEF), World Health Organization (WHO). WHO Immunization Data Portal - WHO and UNICEF Estimates of National Immunization Coverage 1980-2020 as of July 15 2021. Geneva, Switzerland: World Health Organization (WHO), 2021.
Central African Republic	1992	Conflict / War / Civil unrest	United Nations Children's Fund (UNICEF), World Health Organization (WHO). WHO Immunization Data Portal - WHO and UNICEF Estimates of National Immunization Coverage 1980-2020 as of July 15 2021. Geneva, Switzerland: World Health Organization (WHO), 2021.
Central African Republic	2013	Conflict / War / Civil unrest	United Nations Children's Fund (UNICEF), World Health Organization (WHO). WHO Immunization Data Portal - WHO and UNICEF Estimates of National Immunization Coverage 1980-2020 as of July 15 2021. Geneva, Switzerland: World Health Organization (WHO), 2021.
Chad	1998	Discontinuity in administrative data assumed to be stockout based upon review of historical WHO/UNICEF Review of National Immunization Coverage 1980-2008.	United Nations Children's Fund (UNICEF), World Health Organization (WHO). WHO Immunization Data Portal - WHO and UNICEF Estimates of National Immunization Coverage 1980-2020 as of July 15 2021. Geneva, Switzerland: World Health Organization (WHO), 2021.
Chad	2004	Irregular vaccine access	United Nations Children's Fund (UNICEF), World Health Organization (WHO). WHO Immunization Data Portal - WHO and UNICEF Estimates of National Immunization Coverage 1980-2020 as of July 15 2021. Geneva, Switzerland: World Health Organization (WHO), 2021.
Chad	2008	Irregular vaccine access	United Nations Children's Fund (UNICEF), World Health Organization (WHO). WHO Immunization Data Portal - WHO and UNICEF Estimates of National Immunization Coverage 1980-2020 as of July 15 2021. Geneva, Switzerland: World Health Organization (WHO), 2021.
Cook Islands	2009	Small birth cohort	United Nations Children's Fund (UNICEF), World Health Organization (WHO). WHO Immunization Data Portal - WHO and UNICEF Estimates of National Immunization Coverage 1980-2020 as of July 15 2021. Geneva, Switzerland: World Health Organization (WHO), 2021.
Costa Rica	2009	Three to six-month vaccine stock-out caused a temporary disruption in vaccine availability and coverage	United Nations Children's Fund (UNICEF), World Health Organization (WHO). WHO Immunization Data Portal - WHO and UNICEF Estimates of National Immunization Coverage 1980-2020 as of July 15 2021. Geneva, Switzerland: World Health Organization (WHO), 2021.
Costa Rica	2011	One-month vaccine stock-out caused a temporary disruption in vaccine availability and coverage	United Nations Children's Fund (UNICEF), World Health Organization (WHO). WHO Immunization Data Portal - WHO and UNICEF Estimates of National Immunization Coverage 1980-2020 as of July 15 2021. Geneva, Switzerland: World Health Organization (WHO), 2021.
Cote d'Ivoire	2008	Vaccine shortages in 70 districts	United Nations Children's Fund (UNICEF), World Health Organization (WHO). WHO Immunization Data Portal - WHO and UNICEF Estimates of National Immunization Coverage 1980-2020 as of July 15 2021. Geneva, Switzerland: World Health Organization (WHO), 2021.
Cote d'Ivoire	2011	Vaccine shortages in 70 districts	United Nations Children's Fund (UNICEF), World Health Organization (WHO). WHO Immunization Data Portal - WHO and UNICEF Estimates of National Immunization Coverage 1980-2020 as of July 15 2021. Geneva, Switzerland: World Health Organization (WHO), 2021.
Democratic People's Republic of Korea	1997	1994-1996 DPR Korea experienced a period of both economic crisis and natural disasters which compromised the government's ability to produce vaccine.	United Nations Children's Fund (UNICEF), World Health Organization (WHO). WHO Immunization Data Portal - WHO and UNICEF Estimates of National Immunization Coverage 1980-2020 as of July 15 2021. Geneva, Switzerland: World Health Organization (WHO), 2021.

Country	Year	Reason for disruption	Source
Democratic Republic of the Congo	1991	Conflict / War / Civil unrest	Wikipedia contributors. (n.d.). 1991 Zaire unrest - Aftermath. Wikipedia. Retrieved January 24, 2025, from https://en.wikipedia.org/wiki/1991_Zaire_unrest#Aftermath
Democratic Republic of the Congo	2015	National-level vaccine stock-out for two months	United Nations Children's Fund (UNICEF), World Health Organization (WHO). WHO Immunization Data Portal - WHO and UNICEF Estimates of National Immunization Coverage 1980-2020 as of July 15 2021. Geneva, Switzerland: World Health Organization (WHO), 2021.
Denmark	1996	Discontinuity in administrative data assumed to be stockout based upon review of historical WHO/UNICEF Review of National Immunization Coverage 1980-2008.	United Nations Children's Fund (UNICEF), World Health Organization (WHO). WHO Immunization Data Portal - WHO and UNICEF Estimates of National Immunization Coverage 1980-2020 as of July 15 2021. Geneva, Switzerland: World Health Organization (WHO), 2021.
Djibouti	1982	Conflict / War / Civil unrest and cold chain issue	United Nations Children's Fund (UNICEF), World Health Organization (WHO). WHO Immunization Data Portal - WHO and UNICEF Estimates of National Immunization Coverage 1980-2020 as of July 15 2021. Geneva, Switzerland: World Health Organization (WHO), 2021.
Djibouti	1986	Conflict / War / Civil unrest and cold chain issue	United Nations Children's Fund (UNICEF), World Health Organization (WHO). WHO Immunization Data Portal - WHO and UNICEF Estimates of National Immunization Coverage 1980-2020 as of July 15 2021. Geneva, Switzerland: World Health Organization (WHO), 2021.
Djibouti	1987	Conflict / War / Civil unrest and cold chain issue	United Nations Children's Fund (UNICEF), World Health Organization (WHO). WHO Immunization Data Portal - WHO and UNICEF Estimates of National Immunization Coverage 1980-2020 as of July 15 2021. Geneva, Switzerland: World Health Organization (WHO), 2021.
Djibouti	1993	Conflict / War / Civil unrest and cold chain issue	United Nations Children's Fund (UNICEF), World Health Organization (WHO). WHO Immunization Data Portal - WHO and UNICEF Estimates of National Immunization Coverage 1980-2020 as of July 15 2021. Geneva, Switzerland: World Health Organization (WHO), 2021.
Djibouti	1998	Cold chain issue	United Nations Children's Fund (UNICEF), World Health Organization (WHO). WHO Immunization Data Portal - WHO and UNICEF Estimates of National Immunization Coverage 1980-2020 as of July 15 2021. Geneva, Switzerland: World Health Organization (WHO), 2021.
Djibouti	1999	Cold chain issue	United Nations Children's Fund (UNICEF), World Health Organization (WHO). WHO Immunization Data Portal - WHO and UNICEF Estimates of National Immunization Coverage 1980-2020 as of July 15 2021. Geneva, Switzerland: World Health Organization (WHO), 2021.
Djibouti	2000	Conflict / War / Civil unrest and cold chain issue	United Nations Children's Fund (UNICEF), World Health Organization (WHO). WHO Immunization Data Portal - WHO and UNICEF Estimates of National Immunization Coverage 1980-2020 as of July 15 2021. Geneva, Switzerland: World Health Organization (WHO), 2021.
Djibouti	2001	Conflict / War / Civil unrest and cold chain issue	United Nations Children's Fund (UNICEF), World Health Organization (WHO). WHO Immunization Data Portal - WHO and UNICEF Estimates of National Immunization Coverage 1980-2020 as of July 15 2021. Geneva, Switzerland: World Health Organization (WHO), 2021.
Dominican Republic	1996	Discontinuity in administrative data assumed to be stockout based upon review of historical WHO/UNICEF Review of National Immunization Coverage 1980-2008	United Nations Children's Fund (UNICEF), World Health Organization (WHO). WHO Immunization Data Portal - WHO and UNICEF Estimates of National Immunization Coverage 1980-2020 as of July 15 2021. Geneva, Switzerland: World Health Organization (WHO), 2021.

Country	Year	Reason for disruption	Source
Ecuador	1980	Conflict / War / Civil unrest	United Nations Children's Fund (UNICEF), World Health Organization (WHO). WHO Immunization Data Portal - WHO and UNICEF Estimates of National Immunization Coverage 1980-2020 as of July 15 2021. Geneva, Switzerland: World Health Organization (WHO), 2021.
Ecuador	1983	Conflict / War / Civil unrest	United Nations Children's Fund (UNICEF), World Health Organization (WHO). WHO Immunization Data Portal - WHO and UNICEF Estimates of National Immunization Coverage 1980-2020 as of July 15 2021. Geneva, Switzerland: World Health Organization (WHO), 2021.
Ecuador	1985	Conflict / War / Civil unrest	United Nations Children's Fund (UNICEF), World Health Organization (WHO). WHO Immunization Data Portal - WHO and UNICEF Estimates of National Immunization Coverage 1980-2020 as of July 15 2021. Geneva, Switzerland: World Health Organization (WHO), 2021.
Ecuador	1986	Conflict / War / Civil unrest	United Nations Children's Fund (UNICEF), World Health Organization (WHO). WHO Immunization Data Portal - WHO and UNICEF Estimates of National Immunization Coverage 1980-2020 as of July 15 2021. Geneva, Switzerland: World Health Organization (WHO), 2021.
Ecuador	1987	Conflict / War / Civil unrest	United Nations Children's Fund (UNICEF), World Health Organization (WHO). WHO Immunization Data Portal - WHO and UNICEF Estimates of National Immunization Coverage 1980-2020 as of July 15 2021. Geneva, Switzerland: World Health Organization (WHO), 2021.
Ecuador	1989	Conflict / War / Civil unrest	United Nations Children's Fund (UNICEF), World Health Organization (WHO). WHO Immunization Data Portal - WHO and UNICEF Estimates of National Immunization Coverage 1980-2020 as of July 15 2021. Geneva, Switzerland: World Health Organization (WHO), 2021.
Ecuador	1991	Conflict / War / Civil unrest	United Nations Children's Fund (UNICEF), World Health Organization (WHO). WHO Immunization Data Portal - WHO and UNICEF Estimates of National Immunization Coverage 1980-2020 as of July 15 2021. Geneva, Switzerland: World Health Organization (WHO), 2021.
El Salvador	2009	Vaccine shortage	United Nations Children's Fund (UNICEF), World Health Organization (WHO). WHO Immunization Data Portal - WHO and UNICEF Estimates of National Immunization Coverage 1980-2020 as of July 15 2021. Geneva, Switzerland: World Health Organization (WHO), 2021.
Equatorial Guinea	2013	Six-month vaccine stock-out at the national level	United Nations Children's Fund (UNICEF), World Health Organization (WHO). WHO Immunization Data Portal - WHO and UNICEF Estimates of National Immunization Coverage 1980-2020 as of July 15 2021. Geneva, Switzerland: World Health Organization (WHO), 2021.
Eswatini	1991	Vaccine shortage	
Georgia	2009	Three-month vaccine shortage in 56 rayons	United Nations Children's Fund (UNICEF), World Health Organization (WHO). WHO Immunization Data Portal - WHO and UNICEF Estimates of National Immunization Coverage 1980-2020 as of July 15 2021. Geneva, Switzerland: World Health Organization (WHO), 2021.
Germany	1993	Change in DTP vaccine component	United Nations Children's Fund (UNICEF), World Health Organization (WHO). WHO Immunization Data Portal - WHO and UNICEF Estimates of National Immunization Coverage 1980-2020 as of July 15 2021. Geneva, Switzerland: World Health Organization (WHO), 2021.
Guatemala	1980	Syringe shortage	United Nations Children's Fund (UNICEF), World Health Organization (WHO). WHO Immunization Data Portal - WHO and UNICEF Estimates of National Immunization Coverage 1980-2020 as of July 15 2021. Geneva, Switzerland: World Health Organization (WHO), 2021.

Country	Year	Reason for disruption	Source
Guatemala	1985	Syringe shortage	United Nations Children's Fund (UNICEF), World Health Organization (WHO). WHO Immunization Data Portal - WHO and UNICEF Estimates of National Immunization Coverage 1980-2020 as of July 15 2021. Geneva, Switzerland: World Health Organization (WHO), 2021.
Guatemala	1986	Health employee strikes	United Nations Children's Fund (UNICEF), World Health Organization (WHO). WHO Immunization Data Portal - WHO and UNICEF Estimates of National Immunization Coverage 1980-2020 as of July 15 2021. Geneva, Switzerland: World Health Organization (WHO), 2021.
Guatemala	1987	Health employee strikes	United Nations Children's Fund (UNICEF), World Health Organization (WHO). WHO Immunization Data Portal - WHO and UNICEF Estimates of National Immunization Coverage 1980-2020 as of July 15 2021. Geneva, Switzerland: World Health Organization (WHO), 2021.
Guatemala	1989	Re-allocation of public health resources	United Nations Children's Fund (UNICEF), World Health Organization (WHO). WHO Immunization Data Portal - WHO and UNICEF Estimates of National Immunization Coverage 1980-2020 as of July 15 2021. Geneva, Switzerland: World Health Organization (WHO), 2021.
Guatemala	1991	Re-allocation of public health resources	United Nations Children's Fund (UNICEF), World Health Organization (WHO). WHO Immunization Data Portal - WHO and UNICEF Estimates of National Immunization Coverage 1980-2020 as of July 15 2021. Geneva, Switzerland: World Health Organization (WHO), 2021.
Guatemala	2014	Stock-out and inadequate infrastructure caused disruptions	United Nations Children's Fund (UNICEF), World Health Organization (WHO). WHO Immunization Data Portal - WHO and UNICEF Estimates of National Immunization Coverage 1980-2020 as of July 15 2021. Geneva, Switzerland: World Health Organization (WHO), 2021.
Guyana	1984	Vaccine shortage	United Nations Children's Fund (UNICEF), World Health Organization (WHO). WHO Immunization Data Portal - WHO and UNICEF Estimates of National Immunization Coverage 1980-2020 as of July 15 2021. Geneva, Switzerland: World Health Organization (WHO), 2021.
Indonesia	2014	Discontinuity in administrative data assumed to be stockout based upon review of historical WHO/UNICEF Review of National Immunization Coverage 1980-2008	United Nations Children's Fund (UNICEF), World Health Organization (WHO). WHO Immunization Data Portal - WHO and UNICEF Estimates of National Immunization Coverage 1980-2020 as of July 15 2021. Geneva, Switzerland: World Health Organization (WHO), 2021.
Iran (Islamic Republic of)	1986	Conflict / War / Civil unrest	United Nations Children's Fund (UNICEF), World Health Organization (WHO). WHO Immunization Data Portal - WHO and UNICEF Estimates of National Immunization Coverage 1980-2020 as of July 15 2021. Geneva, Switzerland: World Health Organization (WHO), 2021.
Iran (Islamic Republic of)	1987	Conflict / War / Civil unrest	United Nations Children's Fund (UNICEF), World Health Organization (WHO). WHO Immunization Data Portal - WHO and UNICEF Estimates of National Immunization Coverage 1980-2020 as of July 15 2021. Geneva, Switzerland: World Health Organization (WHO), 2021.
Iraq	1998	Irregular vaccine access	United Nations Children's Fund (UNICEF), World Health Organization (WHO). WHO Immunization Data Portal - WHO and UNICEF Estimates of National Immunization Coverage 1980-2020 as of July 15 2021. Geneva, Switzerland: World Health Organization (WHO), 2021.
Ireland	2004	Discontinuity in administrative data assumed to be stockout based upon review of historical WHO/UNICEF Review of National Immunization Coverage 1980-2008	United Nations Children's Fund (UNICEF), World Health Organization (WHO). WHO Immunization Data Portal - WHO and UNICEF Estimates of National Immunization Coverage 1980-2020 as of July 15 2021. Geneva, Switzerland: World Health Organization (WHO), 2021.

Country	Year	Reason for disruption	Source
Ireland	2005	Discontinuity in administrative data assumed to be stockout based upon review of historical WHO/UNICEF Review of National Immunization Coverage 1980-2008	United Nations Children's Fund (UNICEF), World Health Organization (WHO). WHO Immunization Data Portal - WHO and UNICEF Estimates of National Immunization Coverage 1980-2020 as of July 15 2021. Geneva, Switzerland: World Health Organization (WHO), 2021.
Ireland	2016	EU vaccine supplier shortage. BCG given only to select populations.	Houses of the Oireachtas. (2019, April 16). Written answers: Vaccine coverage. Retrieved January 24, 2025, from https://www.oireachtas.ie/en/debates/question/2019-04-16/301/
Jamaica	1992	Irregular vaccine access	United Nations Children's Fund (UNICEF), World Health Organization (WHO). WHO Immunization Data Portal - WHO and UNICEF Estimates of National Immunization Coverage 1980-2020 as of July 15 2021. Geneva, Switzerland: World Health Organization (WHO), 2021.
Jamaica	2007	Hurricane disrupted vaccine coverage	United Nations Children's Fund (UNICEF), World Health Organization (WHO). WHO Immunization Data Portal - WHO and UNICEF Estimates of National Immunization Coverage 1980-2020 as of July 15 2021. Geneva, Switzerland: World Health Organization (WHO), 2021.
Jordan	1999	Discontinuity in administrative data assumed to be stockout based upon review of historical WHO/UNICEF Review of National Immunization Coverage 1980-2008	United Nations Children's Fund (UNICEF), World Health Organization (WHO). WHO Immunization Data Portal - WHO and UNICEF Estimates of National Immunization Coverage 1980-2020 as of July 15 2021. Geneva, Switzerland: World Health Organization (WHO), 2021.
Kazakhstan	2004	Five-month BCG vaccine stock-out	United Nations Children's Fund (UNICEF), World Health Organization (WHO). WHO Immunization Data Portal - WHO and UNICEF Estimates of National Immunization Coverage 1980-2020 as of July 15 2021. Geneva, Switzerland: World Health Organization (WHO), 2021.
Kazakhstan	2005	Five-month BCG vaccine stock-out	United Nations Children's Fund (UNICEF), World Health Organization (WHO). WHO Immunization Data Portal - WHO and UNICEF Estimates of National Immunization Coverage 1980-2020 as of July 15 2021. Geneva, Switzerland: World Health Organization (WHO), 2021.
Kiribati	2012	Small birth cohort	United Nations Children's Fund (UNICEF), World Health Organization (WHO). WHO Immunization Data Portal - WHO and UNICEF Estimates of National Immunization Coverage 1980-2020 as of July 15 2021. Geneva, Switzerland: World Health Organization (WHO), 2021.
Kiribati	2016	Small birth cohort	Asian Development Bank. (n.d.). Supporting documents: Project data sheet. Retrieved January 24, 2025, from https://www.adb.org/sites/default/files/linked-documents/50282-001-sd-02.pdf
Kuwait	1980	Discontinuity in administrative data assumed to be stockout based upon review of historical WHO/UNICEF Review of National Immunization Coverage 1980-2008	United Nations Children's Fund (UNICEF), World Health Organization (WHO). WHO Immunization Data Portal - WHO and UNICEF Estimates of National Immunization Coverage 1980-2020 as of July 15 2021. Geneva, Switzerland: World Health Organization (WHO), 2021.
Lao People's Democratic Republic	1992	Discontinuity in administrative data assumed to be stockout based upon review of historical WHO/UNICEF Review of National Immunization Coverage 1980-2008	United Nations Children's Fund (UNICEF), World Health Organization (WHO). WHO Immunization Data Portal - WHO and UNICEF Estimates of National Immunization Coverage 1980-2020 as of July 15 2021. Geneva, Switzerland: World Health Organization (WHO), 2021.
Latvia	2012	Vaccine schedule changes	United Nations Children's Fund (UNICEF), World Health Organization (WHO). WHO Immunization Data Portal - WHO and UNICEF Estimates of National Immunization Coverage 1980-2020 as of July 15 2021. Geneva, Switzerland: World Health Organization (WHO), 2021.
Lebanon	2006	Conflict / War / Civil unrest	United Nations Children's Fund (UNICEF), World Health Organization (WHO). WHO Immunization Data Portal - WHO and UNICEF Estimates of National Immunization Coverage 1980-2020 as of July 15 2021. Geneva, Switzerland: World Health Organization (WHO), 2021.

Country	Year	Reason for disruption	Source
Liberia	2014	Ebola outbreak disrupted vaccine coverage	United Nations Children's Fund (UNICEF), World Health Organization (WHO). WHO Immunization Data Portal - WHO and UNICEF Estimates of National Immunization Coverage 1980-2020 as of July 15 2021. Geneva, Switzerland: World Health Organization (WHO), 2021.
Marshall Islands	2000	Discontinuity in administrative data assumed to be stockout based upon review of historical WHO/UNICEF Review of National Immunization Coverage 1980-2008	United Nations Children's Fund (UNICEF), World Health Organization (WHO). WHO Immunization Data Portal - WHO and UNICEF Estimates of National Immunization Coverage 1980-2020 as of July 15 2021. Geneva, Switzerland: World Health Organization (WHO), 2021.
Marshall Islands	2009	Small birth cohort	United Nations Children's Fund (UNICEF), World Health Organization (WHO). WHO Immunization Data Portal - WHO and UNICEF Estimates of National Immunization Coverage 1980-2020 as of July 15 2021. Geneva, Switzerland: World Health Organization (WHO), 2021.
Marshall Islands	2016	Small birth cohort	United Nations Children's Fund (UNICEF), World Health Organization (WHO). WHO Immunization Data Portal - WHO and UNICEF Estimates of National Immunization Coverage 1980-2020 as of July 15 2021. Geneva, Switzerland: World Health Organization (WHO), 2021.
Mexico	2017	Irregular vaccine access	United Nations Children's Fund (UNICEF), World Health Organization (WHO). WHO Immunization Data Portal - WHO and UNICEF Estimates of National Immunization Coverage 1980-2020 as of July 15 2021. Geneva, Switzerland: World Health Organization (WHO), 2021.
Myanmar	2011	Vaccine stock-out	United Nations Children's Fund (UNICEF), World Health Organization (WHO). WHO Immunization Data Portal - WHO and UNICEF Estimates of National Immunization Coverage 1980-2020 as of July 15 2021. Geneva, Switzerland: World Health Organization (WHO), 2021.
Nauru	2006	Vaccine stock-out	United Nations Children's Fund (UNICEF), World Health Organization (WHO). WHO Immunization Data Portal - WHO and UNICEF Estimates of National Immunization Coverage 1980-2020 as of July 15 2021. Geneva, Switzerland: World Health Organization (WHO), 2021.
Niger	2011	Data recalibrated to 2010-2012 levels with three-month vaccine stock-out reflected	United Nations Children's Fund (UNICEF), World Health Organization (WHO). WHO Immunization Data Portal - WHO and UNICEF Estimates of National Immunization Coverage 1980-2020 as of July 15 2021. Geneva, Switzerland: World Health Organization (WHO), 2021.
Niger	2013	Five-month vaccine stock-out at the national level	United Nations Children's Fund (UNICEF), World Health Organization (WHO). WHO Immunization Data Portal - WHO and UNICEF Estimates of National Immunization Coverage 1980-2020 as of July 15 2021. Geneva, Switzerland: World Health Organization (WHO), 2021.
Nigeria	1997	Discontinuity in administrative data assumed to be stockout based upon review of historical WHO/UNICEF Review of National Immunization Coverage 1980-2008	United Nations Children's Fund (UNICEF), World Health Organization (WHO). WHO Immunization Data Portal - WHO and UNICEF Estimates of National Immunization Coverage 1980-2020 as of July 15 2021. Geneva, Switzerland: World Health Organization (WHO), 2021.
Nigeria	1998	Discontinuity in administrative data assumed to be stockout based upon review of historical WHO/UNICEF Review of National Immunization Coverage 1980-2008	United Nations Children's Fund (UNICEF), World Health Organization (WHO). WHO Immunization Data Portal - WHO and UNICEF Estimates of National Immunization Coverage 1980-2020 as of July 15 2021. Geneva, Switzerland: World Health Organization (WHO), 2021.
Niue	2016	Reported vaccine stock-out	Asian Development Bank. (n.d.). Supporting documents: Project data sheet. Retrieved January 24, 2025, from https://www.adb.org/sites/default/files/linked-documents/50282-001-sd-02.pdf

Country	Year	Reason for disruption	Source
Pakistan	1994	Irregular vaccine access	United Nations Children's Fund (UNICEF), World Health Organization (WHO). WHO Immunization Data Portal - WHO and UNICEF Estimates of National Immunization Coverage 1980-2020 as of July 15 2021. Geneva, Switzerland: World Health Organization (WHO), 2021.
Papua New Guinea	2016	Reported vaccine stock-out	Asian Development Bank. (n.d.). Supporting documents: Project data sheet. Retrieved January 24, 2025, from https://www.adb.org/sites/default/files/linked-documents/50282-001-sd-02.pdf
Peru	2013	Polio vaccine stock-out and sequential schedule introduction contributed to the decline in coverage	United Nations Children's Fund (UNICEF), World Health Organization (WHO). WHO Immunization Data Portal - WHO and UNICEF Estimates of National Immunization Coverage 1980-2020 as of July 15 2021. Geneva, Switzerland: World Health Organization (WHO), 2021.
Philippines	2014	Four-month vaccine stock-out and private sector serving 10 percent of eligible population	United Nations Children's Fund (UNICEF), World Health Organization (WHO). WHO Immunization Data Portal - WHO and UNICEF Estimates of National Immunization Coverage 1980-2020 as of July 15 2021. Geneva, Switzerland: World Health Organization (WHO), 2021.
Philippines	2018	Public distrust and distribution challenges	United Nations Children's Fund (UNICEF), World Health Organization (WHO). WHO Immunization Data Portal - WHO and UNICEF Estimates of National Immunization Coverage 1980-2020 as of July 15 2021. Geneva, Switzerland: World Health Organization (WHO), 2021.
Qatar	2000	Vaccine shortage	United Nations Children's Fund (UNICEF), World Health Organization (WHO). WHO Immunization Data Portal - WHO and UNICEF Estimates of National Immunization Coverage 1980-2020 as of July 15 2021. Geneva, Switzerland: World Health Organization (WHO), 2021.
Saint Lucia	2000	Discontinuity in administrative data assumed to be stockout based upon review of historical WHO/UNICEF Review of National Immunization Coverage 1980-2008	United Nations Children's Fund (UNICEF), World Health Organization (WHO). WHO Immunization Data Portal - WHO and UNICEF Estimates of National Immunization Coverage 1980-2020 as of July 15 2021. Geneva, Switzerland: World Health Organization (WHO), 2021.
Saint Vincent and the Grenadines	2009	Inadequate infrastructure impacted coverage	United Nations Children's Fund (UNICEF), World Health Organization (WHO). WHO Immunization Data Portal - WHO and UNICEF Estimates of National Immunization Coverage 1980-2020 as of July 15 2021. Geneva, Switzerland: World Health Organization (WHO), 2021.
Samoa	2003	Vaccine shortage	United Nations Children's Fund (UNICEF), World Health Organization (WHO). WHO Immunization Data Portal - WHO and UNICEF Estimates of National Immunization Coverage 1980-2020 as of July 15 2021. Geneva, Switzerland: World Health Organization (WHO), 2021.
Samoa	2004	Vaccine shortage	United Nations Children's Fund (UNICEF), World Health Organization (WHO). WHO Immunization Data Portal - WHO and UNICEF Estimates of National Immunization Coverage 1980-2020 as of July 15 2021. Geneva, Switzerland: World Health Organization (WHO), 2021.
Samoa	2008	Small birth cohort	United Nations Children's Fund (UNICEF), World Health Organization (WHO). WHO Immunization Data Portal - WHO and UNICEF Estimates of National Immunization Coverage 1980-2020 as of July 15 2021. Geneva, Switzerland: World Health Organization (WHO), 2021.
Samoa	2009	Small birth cohort	United Nations Children's Fund (UNICEF), World Health Organization (WHO). WHO Immunization Data Portal - WHO and UNICEF Estimates of National Immunization Coverage 1980-2020 as of July 15 2021. Geneva, Switzerland: World Health Organization (WHO), 2021.

Country	Year	Reason for disruption	Source
Samoa	2010	Small birth cohort	United Nations Children's Fund (UNICEF), World Health Organization (WHO). WHO Immunization Data Portal - WHO and UNICEF Estimates of National Immunization Coverage 1980-2020 as of July 15 2021. Geneva, Switzerland: World Health Organization (WHO), 2021.
Samoa	2015	Small birth cohort	United Nations Children's Fund (UNICEF), World Health Organization (WHO). WHO Immunization Data Portal - WHO and UNICEF Estimates of National Immunization Coverage 1980-2020 as of July 15 2021. Geneva, Switzerland: World Health Organization (WHO), 2021.
Samoa	2018	Measles outbreak, vaccine hesitancy, and low coverage contributed to vaccine disruption	United Nations Children's Fund (UNICEF), World Health Organization (WHO). WHO Immunization Data Portal - WHO and UNICEF Estimates of National Immunization Coverage 1980-2020 as of July 15 2021. Geneva, Switzerland: World Health Organization (WHO), 2021.
Sao Tome and Principe	2006	Vaccine shortage	United Nations Children's Fund (UNICEF), World Health Organization (WHO). WHO Immunization Data Portal - WHO and UNICEF Estimates of National Immunization Coverage 1980-2020 as of July 15 2021. Geneva, Switzerland: World Health Organization (WHO), 2021.
Saudi Arabia	2019	Stock-out due to changes in BCG vaccine schedule	United Nations Children's Fund (UNICEF), World Health Organization (WHO). WHO Immunization Data Portal - WHO and UNICEF Estimates of National Immunization Coverage 1980-2020 as of July 15 2021. Geneva, Switzerland: World Health Organization (WHO), 2021.
Senegal	2004	National shortage of measles vaccine for three months	United Nations Children's Fund (UNICEF), World Health Organization (WHO). WHO Immunization Data Portal - WHO and UNICEF Estimates of National Immunization Coverage 1980-2020 as of July 15 2021. Geneva, Switzerland: World Health Organization (WHO), 2021.
Sierra Leone	2000	Discontinuity in administrative data assumed to be stockout based upon review of historical WHO/UNICEF Review of National Immunization Coverage 1980-2008	United Nations Children's Fund (UNICEF), World Health Organization (WHO). WHO Immunization Data Portal - WHO and UNICEF Estimates of National Immunization Coverage 1980-2020 as of July 15 2021. Geneva, Switzerland: World Health Organization (WHO), 2021.
Sierra Leone	2001	Discontinuity in administrative data assumed to be stockout based upon review of historical WHO/UNICEF Review of National Immunization Coverage 1980-2008	United Nations Children's Fund (UNICEF), World Health Organization (WHO). WHO Immunization Data Portal - WHO and UNICEF Estimates of National Immunization Coverage 1980-2020 as of July 15 2021. Geneva, Switzerland: World Health Organization (WHO), 2021.
Syrian Arab Republic	1987	Discontinuity in administrative data assumed to be stockout based upon review of historical WHO/UNICEF Review of National Immunization Coverage 1980-2008	United Nations Children's Fund (UNICEF), World Health Organization (WHO). WHO Immunization Data Portal - WHO and UNICEF Estimates of National Immunization Coverage 1980-2020 as of July 15 2021. Geneva, Switzerland: World Health Organization (WHO), 2021.
Syrian Arab Republic	1988	Discontinuity in administrative data assumed to be stockout based upon review of historical WHO/UNICEF Review of National Immunization Coverage 1980-2008	United Nations Children's Fund (UNICEF), World Health Organization (WHO). WHO Immunization Data Portal - WHO and UNICEF Estimates of National Immunization Coverage 1980-2020 as of July 15 2021. Geneva, Switzerland: World Health Organization (WHO), 2021.
Syrian Arab Republic	2012	Conflict / War / Civil unrest	United Nations Children's Fund (UNICEF), World Health Organization (WHO). WHO Immunization Data Portal - WHO and UNICEF Estimates of National Immunization Coverage 1980-2020 as of July 15 2021. Geneva, Switzerland: World Health Organization (WHO), 2021.
Syrian Arab Republic	2014	Conflict / War / Civil unrest	United Nations Children's Fund (UNICEF), World Health Organization (WHO). WHO Immunization Data Portal - WHO and UNICEF Estimates of National Immunization Coverage 1980-2020 as of July 15 2021. Geneva, Switzerland: World Health Organization (WHO), 2021.

Country	Year	Reason for disruption	Source
Syrian Arab Republic	2015	Conflict / War / Civil unrest	United Nations Children's Fund (UNICEF), World Health Organization (WHO). WHO Immunization Data Portal - WHO and UNICEF Estimates of National Immunization Coverage 1980-2020 as of July 15 2021. Geneva, Switzerland: World Health Organization (WHO), 2021.
Syrian Arab Republic	2018	Conflict / War / Civil unrest	United Nations Children's Fund (UNICEF), World Health Organization (WHO). WHO Immunization Data Portal - WHO and UNICEF Estimates of National Immunization Coverage 1980-2020 as of July 15 2021. Geneva, Switzerland: World Health Organization (WHO), 2021.
Timor-Leste	2005	Discontinuity in administrative data assumed to be stockout based upon review of historical WHO/UNICEF Review of National Immunization Coverage 1980-2008	United Nations Children's Fund (UNICEF), World Health Organization (WHO). WHO Immunization Data Portal - WHO and UNICEF Estimates of National Immunization Coverage 1980-2020 as of July 15 2021. Geneva, Switzerland: World Health Organization (WHO), 2021.
Togo	2014	Syringe shortage	United Nations Children's Fund (UNICEF), World Health Organization (WHO). WHO Immunization Data Portal - WHO and UNICEF Estimates of National Immunization Coverage 1980-2020 as of July 15 2021. Geneva, Switzerland: World Health Organization (WHO), 2021.
Turkmenistan	2003	Six-month vaccine shortage	United Nations Children's Fund (UNICEF), World Health Organization (WHO). WHO Immunization Data Portal - WHO and UNICEF Estimates of National Immunization Coverage 1980-2020 as of July 15 2021. Geneva, Switzerland: World Health Organization (WHO), 2021.
Ukraine	2014	Stock-out and public distrust disrupted coverage	United Nations Children's Fund (UNICEF), World Health Organization (WHO). WHO Immunization Data Portal - WHO and UNICEF Estimates of National Immunization Coverage 1980-2020 as of July 15 2021. Geneva, Switzerland: World Health Organization (WHO), 2021.
Ukraine	2015	Five-month vaccine stock-out at the national level	United Nations Children's Fund (UNICEF), World Health Organization (WHO). WHO Immunization Data Portal - WHO and UNICEF Estimates of National Immunization Coverage 1980-2020 as of July 15 2021. Geneva, Switzerland: World Health Organization (WHO), 2021.
Ukraine	2016	Six-month vaccine stock-out with 78 percent decline in coverage	United Nations Children's Fund (UNICEF), World Health Organization (WHO). WHO Immunization Data Portal - WHO and UNICEF Estimates of National Immunization Coverage 1980-2020 as of July 15 2021. Geneva, Switzerland: World Health Organization (WHO), 2021.
Uzbekistan	1992	Conflict / War / Civil unrest	Kotloff, K. L., Wasserman, S. S., Losonsky, G. A., & Levine, M. M. (2000). Immunogenicity of a Highly Immunogenic Shigella Conjugate Vaccine in Healthy Young Adults in North America. The Journal of Infectious Diseases, 181(Supplement_1), S104–S107. https://doi.org/10.1086/315913
Uzbekistan	1993	Conflict / War / Civil unrest	Kotloff, K. L., Wasserman, S. S., Losonsky, G. A., & Levine, M. M. (2000). Immunogenicity of a Highly Immunogenic Shigella Conjugate Vaccine in Healthy Young Adults in North America. The Journal of Infectious Diseases, 181(Supplement_1), S104–S107. https://doi.org/10.1086/315913
Venezuela (Bolivarian Republic of)	2017	Vaccine shortage	United Nations Children's Fund (UNICEF), World Health Organization (WHO). WHO Immunization Data Portal - WHO and UNICEF Estimates of National Immunization Coverage 1980-2020 as of July 15 2021. Geneva, Switzerland: World Health Organization (WHO), 2021.
Venezuela (Bolivarian Republic of)	2018	Vaccine shortage	United Nations Children's Fund (UNICEF), World Health Organization (WHO). WHO Immunization Data Portal - WHO and UNICEF Estimates of National Immunization Coverage 1980-2020 as of July 15 2021. Geneva, Switzerland: World Health Organization (WHO), 2021.

Country	Year	Reason for disruption	Source
Viet Nam	2002	Vaccine shortage	United Nations Children's Fund (UNICEF), World Health Organization (WHO). WHO Immunization Data Portal - WHO and UNICEF Estimates of National Immunization Coverage 1980-2020 as of July 15 2021. Geneva, Switzerland: World Health Organization (WHO), 2021.
Viet Nam	2007	Vaccine shortage	United Nations Children's Fund (UNICEF), World Health Organization (WHO). WHO Immunization Data Portal - WHO and UNICEF Estimates of National Immunization Coverage 1980-2020 as of July 15 2021. Geneva, Switzerland: World Health Organization (WHO), 2021.
Viet Nam	2013	Vaccine quality caused a disruption in the vaccination process	United Nations Children's Fund (UNICEF), World Health Organization (WHO). WHO Immunization Data Portal - WHO and UNICEF Estimates of National Immunization Coverage 1980-2020 as of July 15 2021. Geneva, Switzerland: World Health Organization (WHO), 2021.

Table S5 Super-regions and corresponding countries and territories

Groups of countries and territories identified for analysis and comparison on the bases of epidemiological homogeneity, and geographical contiguity in line with earlier GBD studies.

Super Region	Countries and territories
Central Europe, Eastern Europe, and Central Asia	Albania, Armenia, Azerbaijan, Belarus, Bosnia and Herzegovina, Bulgaria, Croatia, Czechia, Estonia, Georgia, Hungary, Kazakhstan, Kyrgyzstan, Latvia, Lithuania, Mongolia, Montenegro, North Macedonia, Poland, Republic of Moldova, Romania, Russian Federation, Serbia, Slovakia, Slovenia, Tajikistan, Turkmenistan, Ukraine, Uzbekistan
High-income	Andorra, Argentina, Australia, Austria, Belgium, Brunei Darussalam, Canada, Chile, Cyprus, Denmark, Finland, France, Germany, Greece, Greenland, Iceland, Ireland, Israel, Italy, Japan, Luxembourg, Malta, Monaco, Netherlands, New Zealand, Norway, Portugal, Republic of Korea, San Marino, Singapore, Spain, Sweden, Switzerland, United Kingdom, United States of America, Uruguay
Latin America and Caribbean	Antigua and Barbuda, Bahamas, Barbados, Belize, Bolivia (Plurinational State of), Brazil, Colombia, Costa Rica, Cuba, Dominica, Dominican Republic, Ecuador, El Salvador, Grenada, Guatemala, Guyana, Haiti, Honduras, Jamaica, Mexico, Nicaragua, Panama, Paraguay, Peru, Saint Kitts and Nevis, Saint Lucia, Saint Vincent and the Grenadines, Suriname, Trinidad and Tobago, Venezuela (Bolivarian Republic of)
North Africa and Middle East	Afghanistan, Algeria, Bahrain, Egypt, Iran (Islamic Republic of), Iraq, Jordan, Kuwait, Lebanon, Libya, Morocco, Oman, Palestine, Qatar, Saudi Arabia, Sudan, Syrian Arab Republic, Tunisia, Türkiye, United Arab Emirates, Yemen
South Asia	Bangladesh, Bhutan, India, Nepal, Pakistan
Southeast Asia, East Asia, and Oceania	Cambodia, China, Democratic People's Republic of Korea, Fiji, Indonesia, Kiribati, Lao People's Democratic Republic, Malaysia, Maldives, Marshall Islands, Mauritius, Micronesia (Federated States of), Myanmar, Nauru, Palau, Papua New Guinea, Philippines, Samoa, Seychelles, Solomon Islands, Sri Lanka, Taiwan, Thailand, Timor-Leste, Tonga, Tuvalu, Vanuatu, Viet Nam
Sub-Saharan Africa	Angola, Benin, Botswana, Burkina Faso, Burundi, Cabo Verde, Cameroon, Central African Republic, Chad, Comoros, Congo, Côte d'Ivoire, Democratic Republic of the Congo, Djibouti, Equatorial Guinea, Eritrea, Eswatini, Ethiopia, Gabon, Gambia, Ghana, Guinea, Guinea-Bissau, Kenya, Lesotho, Liberia, Madagascar, Malawi, Mali, Mauritania, Mozambique, Namibia, Niger, Nigeria, Rwanda, Sao Tome and Principe, Senegal, Sierra Leone, Somalia, South Africa, South Sudan, Togo, Uganda, United Republic of Tanzania, Zambia, Zimbabwe

Table S6 COVID-inclusive vs. COVID-free counterfactual coverage of EPI vaccines by super-region in 2023

COVID-inclusive vs. COVID-free counterfactual coverage of EPI vaccines by super-region in 2023, as well as differences in coverage for the target population between these scenarios calculated by draw. COVID-free and COVID-inclusive pairs where the difference is within one percentage point of each other are in bold. BCG=Bacillus Calmette-Guérin. DTP1=diphtheria-tetanus-pertussis, first dose. DTP3=diphtheria-tetanus-pertussis, third dose. MCV1=measles-containing vaccine, first dose. Pol3=Pol vaccine, third dose.

Super Region	Vaccine	COVID-Free (mean, lower and upper 95% uncertainty interval) (%)	COVID-Inclusive (mean, lower and upper 95% uncertainty interval) (%)	COVID Scenario Difference
**Central Europe, Eastern Europe, and Central Asia**	**BCG**	**91·2 (89·5–92·4)**	**90·9 (89·2–92·0)**	**0·3 pp (–0·4 to 0·6)**
**Central Europe, Eastern Europe, and Central Asia**	**DTP1**	**96·1 (94·6–97·0)**	**96·8 (95·7–97·4)**	**a decrease of 0·7 pp (0·4–1·5)**
**Central Europe, Eastern Europe, and Central Asia**	**DTP3**	**92·2 (89·9–93·5)**	**93·0 (92·0–93·8)**	**a decrease of 0·9 pp (0·2–2·4)**
Central Europe, Eastern Europe, and Central Asia	HepB3	89·6 (87·5–90·9)	90·8 (89·8–91·6)	a decrease of 1·2 pp (0·5–2·7)
Central Europe, Eastern Europe, and Central Asia	Hib3	90·9 (88·7–92·2)	92·0 (90·9–92·8)	a decrease of 1·1 pp (0·4–2·6)
**Central Europe, Eastern Europe, and Central Asia**	**MCV1**	**92·9 (91·3–94·1)**	**93·8 (92·9–94·6)**	**a decrease of 0·9 pp (0·3–2·0)**
Central Europe, Eastern Europe, and Central Asia	MCV2	90·2 (85·7–92·0)	92·5 (91·6–93·3)	a decrease of 2·3 pp (1·0–6·6)
Central Europe, Eastern Europe, and Central Asia	PCV3	76·1 (74·1–77·3)	78·4 (77·4–79·2)	a decrease of 2·4 pp (1·7–3·8)
Central Europe, Eastern Europe, and Central Asia	Pol3	91·5 (90·0–92·8)	92·6 (91·2–93·7)	a decrease of 1·1 pp (0·8–1·8)
Central Europe, Eastern Europe, and Central Asia	RCV1	92·8 (91·2–94·0)	93·7 (92·8–94·5)	a decrease of 0·9 pp (0·3–2·0)
Central Europe, Eastern Europe, and Central Asia	RotaC	35·9 (34·2–36·7)	35·4 (35·0–35·8)	0·5 pp (–1·1 to 1·2)
High-income	BCG	15·7 (15·1–16·2)	16·8 (16·5–17·1)	a decrease of 1·1 pp (0·9–1·4)
High-income	DTP1	95·1 (91·2–96·8)	97·2 (96·2–97·9)	a decrease of 2·0 pp (0·9–5·8)
High-income	DTP3	91·6 (87·4–93·7)	94·5 (93·3–95·5)	a decrease of 3·0 pp (1·4–7·1)
High-income	HepB3	88·9 (84·7–91·1)	92·2 (91·0–93·1)	a decrease of 3·2 pp (1·6–7·2)
High-income	Hib3	90·7 (86·4–92·8)	93·7 (92·5–94·6)	a decrease of 3·0 pp (1·5–7·1)
High-income	MCV1	92·9 (88·3–94·8)	95·4 (93·8–96·3)	a decrease of 2·5 pp (1·1–7·1)
High-income	MCV2	87·7 (81·3–90·6)	93·7 (92·0–94·7)	a decrease of 6·0 pp (3·5–12·4)
High-income	PCV3	89·0 (84·5–91·5)	92·7 (91·5–93·7)	a decrease of 3·7 pp (1·8–8·2)
High-income	Pol3	92·0 (87·1–94·4)	94·1 (92·4–95·6)	a decrease of 2·1 pp (0·8–7·0)
High-income	RCV1	92·8 (88·2–94·6)	95·3 (93·7–96·2)	a decrease of 2·5 pp (1·1–7·0)
High-income	RotaC	70·4 (65·4–73·0)	74·2 (73·0–75·1)	a decrease of 3·8 pp (1·6–8·6)
Latin America and Caribbean	BCG	83·6 (80·3–86·0)	88·3 (85·6–90·3)	a decrease of 4·7 pp (4·2–5·5)
Latin America and Caribbean	DTP1	85·1 (82·6–87·4)	91·8 (90·3–93·0)	a decrease of 6·7 pp (4·8–8·8)
Latin America and Caribbean	DTP3	75·6 (71·8–78·7)	81·8 (78·4–84·4)	a decrease of 6·3 pp (4·3–8·7)

Super Region	Vaccine	COVID-Free (mean, lower and upper 95% uncertainty interval) (%)	COVID-Inclusive (mean, lower and upper 95% uncertainty interval) (%)	COVID Scenario Difference
Latin America and Caribbean	HepB3	75·4 (71·7–78·5)	77·3 (74·2–79·6)	a decrease of 1·8 pp (–4·3 to 0·2)
Latin America and Caribbean	Hib3	75·4 (71·6–78·5)	81·3 (77·9–83·8)	a decrease of 5·9 pp (4·0–8·3)
Latin America and Caribbean	MCV1	85·9 (83·1–87·7)	89·9 (88·1–90·8)	a decrease of 4·0 pp (2·9–5·2)
Latin America and Caribbean	MCV2	69·0 (65·0–72·6)	73·6 (69·9–76·9)	a decrease of 4·6 pp (3·4–5·8)
Latin America and Caribbean	PCV3	65·2 (61·8–68·0)	76·8 (73·3–79·5)	a decrease of 11·6 pp (9·6–13·9)
Latin America and Caribbean	Pol3	75·7 (73·0–78·1)	78·8 (76·2–81·0)	a decrease of 3·1 pp (2·8–3·6)
Latin America and Caribbean	RCV1	85·8 (83·0–87·6)	89·8 (88·0–90·7)	a decrease of 4·0 pp (2·9–5·2)
Latin America and Caribbean	RotaC	68·8 (65·1–71·8)	76·9 (73·3–79·5)	a decrease of 8·1 pp (6·2–10·4)
North Africa and Middle East	BCG	85·9 (84·8–86·6)	89·2 (88·2–89·9)	a decrease of 3·3 pp (2·8–4·3)
North Africa and Middle East	DTP1	86·3 (84·5–88·1)	89·9 (88·3–91·3)	a decrease of 3·6 pp (3·0–4·5)
North Africa and Middle East	DTP3	80·6 (78·7–82·4)	84·3 (82·8–85·7)	a decrease of 3·7 pp (3·1–4·8)
North Africa and Middle East	HepB3	80·5 (78·6–82·2)	84·2 (82·7–85·6)	a decrease of 3·7 pp (3·1–4·8)
North Africa and Middle East	Hib3	80·5 (78·6–82·3)	84·2 (82·8–85·6)	a decrease of 3·7 pp (3·1–4·8)
North Africa and Middle East	MCV1	83·0 (81·1–84·5)	87·0 (85·8–88·2)	a decrease of 4·0 pp (3·2–5·6)
North Africa and Middle East	MCV2	77·4 (75·3–78·9)	83·1 (81·9–84·4)	a decrease of 5·8 pp (4·7–7·7)
North Africa and Middle East	PCV3	42·4 (40·6–44·0)	48·5 (47·2–49·7)	a decrease of 6·1 pp (5·5–7·3)
North Africa and Middle East	Pol3	82·1 (80·5–83·3)	85·8 (84·6–86·7)	a decrease of 3·7 pp (3·3–4·8)
North Africa and Middle East	RCV1	73·2 (71·6–74·4)	74·1 (73·0–75·1)	a decrease of 0·9 pp (0·5–2·4)
North Africa and Middle East	RotaC	28·8 (27·4–30·0)	33·2 (32·2–34·2)	a decrease of 4·4 pp (3·9–5·4)
South Asia	BCG	93·2 (90·3–94·9)	94·4 (92·1–95·6)	a decrease of 1·2 pp (0·7–1·8)
**South Asia**	**DTP1**	**93·6 (91·8–94·6)**	**94·2 (92·7–95·0)**	**a decrease of 0·6 pp (0·4–0·8)**
South Asia	DTP3	86·7 (83·1–89·4)	88·2 (85·0–90·6)	a decrease of 1·5 pp (1·1–1·9)
South Asia	HepB3	86·6 (83·0–89·3)	88·1 (84·9–90·5)	a decrease of 1·5 pp (1·1–1·9)
South Asia	Hib3	86·6 (82·9–89·3)	88·1 (84·9–90·5)	a decrease of 1·5 pp (1·1–1·9)
South Asia	MCV1	84·2 (79·7–87·0)	86·5 (82·7–88·6)	a decrease of 2·4 pp (1·5–3·1)
South Asia	MCV2	80·8 (76·2–83·8)	83·1 (79·1–85·5)	a decrease of 2·3 pp (1·5–3·0)
South Asia	PCV3	79·9 (76·2–82·6)	81·3 (78·0–83·7)	a decrease of 1·4 pp (1·0–1·8)
**South Asia**	**Pol3**	**82·7 (78·0–87·1)**	**83·5 (79·0–87·8)**	**a decrease of 0·8 pp (0·6–1·0)**
South Asia	RCV1	84·0 (79·5–86·8)	86·4 (82·6–88·5)	a decrease of 2·4 pp (1·5–3·1)
South Asia	RotaC	76·4 (73·0–78·9)	77·8 (74·8–80·0)	a decrease of 1·4 pp (1·0–1·9)
Southeast Asia, East Asia, and Oceania	BCG	93·0 (91·6–93·9)	95·2 (94·2–95·7)	a decrease of 2·2 pp (1·8–2·7)
Southeast Asia, East Asia, and Oceania	DTP1	90·8 (89·5–91·9)	94·2 (93·6–94·5)	a decrease of 3·4 pp (2·5–4·2)
Southeast Asia, East Asia, and Oceania	DTP3	82·6 (80·3–84·6)	86·9 (85·2–88·4)	a decrease of 4·3 pp (3·5–5·2)
Southeast Asia, East Asia, and Oceania	HepB3	82·5 (80·2–84·5)	86·8 (85·1–88·3)	a decrease of 4·3 pp (3·5–5·2)
Southeast Asia, East Asia, and Oceania	Hib3	51·3 (48·5–53·8)	55·0 (52·6–57·1)	a decrease of 3·6 pp (2·9–4·4)

Super Region	Vaccine	COVID-Free (mean, lower and upper 95% uncertainty interval) (%)	COVID-Inclusive (mean, lower and upper 95% uncertainty interval) (%)	COVID Scenario Difference
Southeast Asia, East Asia, and Oceania	MCV1	86·1 (83·7–88·0)	90·8 (89·0–92·0)	a decrease of 4·7 pp (3·9–5·6)
Southeast Asia, East Asia, and Oceania	MCV2	81·8 (78·9–84·0)	87·1 (85·1–88·7)	a decrease of 5·4 pp (4·5–6·5)
Southeast Asia, East Asia, and Oceania	PCV3	33·0 (31·1–34·7)	34·3 (32·5–36·2)	a decrease of 1·3 pp (0·9–2·2)
Southeast Asia, East Asia, and Oceania	Pol3	81·3 (79·1–83·4)	86·9 (85·1–88·4)	a decrease of 5·6 pp (5·0–6·1)
Southeast Asia, East Asia, and Oceania	RCV1	86·0 (83·5–87·9)	90·7 (88·9–91·8)	a decrease of 4·7 pp (3·9–5·7)
Southeast Asia, East Asia, and Oceania	RotaC	10·1 (8·9–11·8)	11·2 (10·1–12·9)	a decrease of 1·1 pp (0·9–1·9)
**Sub-Saharan Africa**	**BCG**	**81·8 (78·7–84·5)**	**82·0 (79·0–84·7)**	**a decrease of 0·2 pp (–0·5 to 0·1)**
Sub-Saharan Africa	DTP1	78·1 (75·8–80·1)	80·0 (77·9–81·9)	a decrease of 1·9 pp (1·7–2·1)
Sub-Saharan Africa	DTP3	65·6 (63·6–67·5)	68·7 (66·6–70·5)	a decrease of 3·0 pp (2·8–3·2)
Sub-Saharan Africa	HepB3	65·6 (63·5–67·4)	68·6 (66·5–70·4)	a decrease of 3·0 pp (2·8–3·2)
Sub-Saharan Africa	Hib3	65·6 (63·5–67·4)	68·6 (66·6–70·4)	a decrease of 3·0 pp (2·8–3·2)
Sub-Saharan Africa	MCV1	67·6 (65·1–69·8)	70·2 (67·7–72·4)	a decrease of 2·6 pp (2·5–2·9)
Sub-Saharan Africa	MCV2	47·1 (43·3–50·9)	51·0 (47·1–54·9)	a decrease of 3·9 pp (3·6–4·3)
Sub-Saharan Africa	PCV3	61·2 (59·3–63·0)	65·1 (63·2–66·9)	a decrease of 3·9 pp (3·7–4·1)
Sub-Saharan Africa	Pol3	62·2 (60·5–63·8)	65·5 (64·0–67·0)	a decrease of 3·3 pp (3·1–3·6)
Sub-Saharan Africa	RCV1	34·0 (33·0–34·9)	35·3 (34·5–36·1)	a decrease of 1·3 pp (1·2–1·6)
Sub-Saharan Africa	RotaC	58·4 (56·4–60·2)	64·1 (62·2–65·8)	a decrease of 5·7 pp (5·4–6·0)

Table S7 Zero-dose counts and Immunization Coverage for 2019 and 2023, with 2030 Projections

Zero-dose counts for 2019 and 2023, along with immunization coverage estimates for both, and projections for the 2030 population and coverage goal for each country and territory. Zero-dose children are defined as those to have never received a routine childhood immunization of any kind, approximated as $(1 - DTP1\ coverage) * population\ under$ 1 for analyses (DTP1=diphtheria-tetanus-pertussis, first dose).

Country	Coverage 2019 (mean, lower and upper 95% uncertainty interval) (%)	Coverage 2023 (mean, lower and upper 95% uncertainty interval) (%)	Zero-dose counts in 2019	Zero-dose counts in 2023	Projected 2030 population	2030 coverage goal (%)
Afghanistan	70·8 (68·0 - 73·3)	72·6 (68·3 - 77·0)	323 672	335 187	1 438 464	88.7
Albania	99·8 (99·5 - 100·0)	98·9 (96·6 - 99·8)	45	302	23 426	99.9
Algeria	95·8 (94·5 - 96·9)	94·4 (91·8 - 96·6)	40 197	47 922	709 892	97.2
Am Samoa	99·8 (99·1 - 100·0)	97·7 (87·3 - 100·0)	2	16	813	99.9
Andorra	99·3 (98·7 - 99·7)	98·1 (95·7 - 99·2)	3	8	366	99.6
Angola	65·8 (62·0 - 69·4)	65·6 (59·0 - 72·1)	386 806	414 006	1 324 695	85.4
Antigua and Barbuda	98·4 (97·2 - 99·1)	98·9 (97·7 - 99·5)	17	11	954	99.1
Argentina	87·6 (83·8 - 90·7)	77·5 (69·7 - 84·5)	74 580	118 776	514 988	92.8
Armenia	98·4 (96·7 - 99·4)	98·2 (95·8 - 99·4)	590	576	26 618	98.9
Australia	94·1 (91·8 - 96·0)	92·9 (88·6 - 95·9)	17 569	20 938	292 092	97.0
Austria	93·8 (91·2 - 95·8)	91·0 (81·7 - 95·8)	5 275	7 542	76 257	96.5
Azerbaijan	93·2 (90·9 - 95·2)	87·4 (81·8 - 92·0)	9 749	15 843	113 173	95.7
Bahamas	91·0 (85·9 - 94·8)	98·5 (97·1 - 99·3)	361	59	4 043	95.5
Bahrain	96·5 (95·2 - 97·4)	97·1 (95·4 - 98·2)	663	491	16 794	98.0
Bangladesh	99·6 (99·1 - 99·8)	99·8 (99·4 - 99·9)	12 755	5 938	2 326 902	99.7
Barbados	93·0 (90·1 - 95·0)	90·1 (84·9 - 93·9)	190	255	2 463	96.2
Belarus	96·4 (94·8 - 97·6)	96·5 (94·0 - 98·0)	3 326	2 746	66 202	97.5
Belgium	98·3 (97·1 - 99·1)	97·7 (95·6 - 99·0)	2 003	2 646	113 386	99.1
Belize	95·6 (94·1 - 96·9)	85·1 (79·0 - 89·9)	328	1 149	8 140	98.0
Benin	77·4 (74·9 - 79·9)	79·9 (75·9 - 83·7)	108 625	105 229	589 787	90.8
Bermuda	96·4 (94·0 - 98·0)	96·9 (94·3 - 98·5)	19	14	397	97.6
Bhutan	99·7 (99·0 - 100·0)	99·8 (99·2 - 100·0)	34	26	10 804	99.8
Bolivia	94·4 (91·5 - 96·6)	76·5 (65·6 - 85·0)	13 563	55 953	232 265	97.1
Bosnia and Herzegovina	94·1 (90·9 - 96·4)	89·3 (81·9 - 94·2)	1 726	2 931	24 449	96.5
Botswana	90·7 (88·3 - 92·7)	90·2 (86·5 - 93·1)	4 444	4 601	45 274	95.1
Brazil	79·7 (74·0 - 84·6)	85·9 (80·2 - 90·6)	687 328	452 030	2 901 133	88.2
Brunei Darussalam	99·8 (99·4 - 99·9)	99·8 (99·4 - 100·0)	14	11	5 265	99.9
Bulgaria	95·6 (93·6 - 97·1)	95·3 (92·0 - 97·4)	2 711	2 622	47 825	97.2
Burkina Faso	94·7 (92·7 - 96·2)	98·1 (96·5 - 99·0)	45 994	18 263	1 082 801	97.9
Burundi	93·5 (91·6 - 95·2)	81·5 (76·1 - 86·4)	28 351	86 944	529 040	97.3
Cabo Verde	96·5 (94·5 - 98·0)	96·0 (86·8 - 99·0)	310	326	7 365	97.9
Cambodia	93·8 (92·2 - 95·2)	91·5 (87·8 - 94·3)	21 878	29 279	319 728	96.6
Cameroon	76·4 (72·5 - 79·9)	77·0 (71·1 - 82·0)	233 629	236 100	1 058 632	89.0
Canada	91·2 (87·2 - 94·3)	92·1 (87·5 - 95·6)	32 766	28 954	373 793	95.6

Country	Coverage 2019 (mean, lower and upper 95% uncertainty interval) (%)	Coverage 2023 (mean, lower and upper 95% uncertainty interval) (%)	Zero-dose counts in 2019	Zero-dose counts in 2023	Projected 2030 population	2030 coverage goal (%)
CAR	59·8 (47·9 - 69·6)	63·6 (44·1 - 80·3)	71 556	66 182	184 453	80.6
Chad	64·5 (61·0 - 68·0)	83·2 (79·6 - 86·4)	267 485	146 220	1 115 809	88.0
Chile	98·5 (97·3 - 99·3)	98·9 (97·6 - 99·6)	3 140	2 263	192 693	99.2
China	98·7 (97·7 - 99·3)	97·6 (95·3 - 98·9)	192 653	242 022	7 937 634	98.8
Colombia	92·2 (89·6 - 94·2)	89·1 (84·1 - 93·0)	53 788	71 456	599 414	95.5
Comoros	86·5 (81·9 - 90·6)	86·9 (80·3 - 92·1)	2 253	2 175	15 795	92.9
Congo	79·8 (75·1 - 83·7)	76·2 (68·2 - 82·9)	26 177	29 595	126 392	89.6
Cook Islands	99·8 (99·6 - 99·9)	94·9 (90·9 - 97·6)	0	11	221	99.9
Costa Rica	95·0 (93·3 - 96·4)	94·3 (91·3 - 96·5)	3 121	2 991	48 644	96.8
Cote d'Ivoire	79·2 (76·1 - 82·0)	78·5 (73·1 - 83·4)	186 996	200 712	963 196	90.3
Croatia	95·2 (93·0 - 96·8)	93·9 (89·5 - 96·5)	1 769	2 058	28 181	96.9
Cuba	97·7 (97·0 - 98·2)	96·5 (95·0 - 97·7)	2 563	3 367	83 069	98.5
Cyprus	99·5 (98·9 - 99·9)	99·7 (99·3 - 99·9)	70	41	12 162	99.7
Czechia	99·5 (99·1 - 99·7)	95·9 (92·4 - 98·1)	557	4 147	87 328	99.7
Denmark	97·0 (95·3 - 98·3)	97·3 (95·0 - 98·7)	1 813	1 761	66 649	98.6
Djibouti	82·8 (79·2 - 86·3)	87·1 (82·3 - 91·1)	5 094	3 615	23 900	89.3
Dominica	99·6 (99·2 - 99·9)	99·6 (99·1 - 99·9)	3	2	633	99.8
Dominican Rep.	91·8 (90·2 - 93·3)	92·4 (89·8 - 94·7)	17 087	15 617	192 487	95.6
DRC	73·6 (69·8 - 77·3)	68·9 (62·6 - 74·8)	733 362	882 061	3 078 015	88.1
Ecuador	83·2 (79·3 - 86·5)	86·5 (75·8 - 92·0)	55 603	42 344	309 901	91.0
Egypt	96·6 (94·3 - 98·2)	98·2 (96·4 - 99·4)	92 936	44 267	2 534 428	98.2
El Salvador	89·2 (87·1 - 91·1)	91·6 (88·5 - 94·2)	13 045	9 354	96 173	93.2
Eq. Guinea	64·9 (55·9 - 73·6)	81·7 (71·4 - 89·5)	13 042	6 959	41 424	84.3
Eritrea	99·2 (98·5 - 99·7)	95·1 (90·6 - 97·9)	1 402	9 512	203 695	99.7
Estonia	92·4 (89·1 - 95·0)	91·2 (80·1 - 96·2)	1 063	1 069	9 758	94.6
Eswatini	96·6 (94·6 - 98·1)	96·9 (94·1 - 98·6)	985	894	28 125	98.2
Ethiopia	75·4 (72·3 - 78·2)	77·9 (72·8 - 82·5)	803 547	781 604	3 972 233	89.9
Fiji	90·5 (87·8 - 92·7)	93·1 (89·8 - 95·5)	1 758	1 220	17 034	94.8
Finland	98·1 (96·8 - 99·0)	96·4 (93·2 - 98·3)	887	1 731	47 738	99.1
France	98·8 (97·5 - 99·5)	98·5 (96·6 - 99·5)	8 713	10 487	669 624	99.3
Gabon	82·2 (77·8 - 86·0)	79·4 (71·6 - 85·8)	7 685	8 721	41 646	90.8
Gambia	97·6 (96·7 - 98·3)	88·4 (81·6 - 93·0)	1 768	8 953	80 687	98.9
Georgia	93·0 (91·2 - 94·6)	90·1 (85·9 - 93·4)	3 390	4 177	36 459	95.4
Germany	98·0 (95·2 - 99·3)	96·0 (85·8 - 99·4)	15 708	31 053	751 022	99.0
Ghana	97·4 (96·0 - 98·3)	98·5 (97·2 - 99·2)	24 591	14 646	902 344	98.6
Greece	99·6 (98·9 - 99·9)	99·6 (98·9 - 99·9)	340	327	73 127	99.8
Greenland	97·8 (95·9 - 98·9)	97·9 (95·5 - 99·2)	18	16	700	98.7
Grenada	94·1 (91·9 - 95·9)	88·9 (83·3 - 93·2)	83	146	1 211	96.6
Guam	98·0 (95·6 - 99·3)	96·7 (85·9 - 99·6)	55	87	2 710	99.0

Country	Coverage 2019 (mean, lower and upper 95% uncertainty interval) (%)	Coverage 2023 (mean, lower and upper 95% uncertainty interval) (%)	Zero-dose counts in 2019	Zero-dose counts in 2023	Projected 2030 population	2030 coverage goal (%)
Guatemala	93·1 (89·7 - 95·5)	92·3 (87·4 - 95·7)	21 129	22 343	279 393	96.2
Guinea	69·3 (63·9 - 74·3)	62·9 (53·3 - 71·9)	141 531	184 040	563 104	87.4
Guinea-Bissau	91·2 (88·4 - 93·7)	83·4 (76·2 - 89·0)	6 066	11 779	73 777	95.9
Guyana	90·7 (89·2 - 92·0)	93·1 (91·2 - 94·6)	1 441	992	12 995	94.5
Haiti	84·4 (74·2 - 91·0)	85·0 (70·3 - 94·3)	50 530	48 846	290 405	91.3
Honduras	97·2 (96·3 - 97·9)	79·4 (71·7 - 86·0)	6 153	45 628	219 588	98.6
Hungary	99·8 (99·6 - 99·9)	99·8 (99·4 - 99·9)	160	212	76 442	99.9
Iceland	95·6 (93·5 - 97·1)	95·3 (92·0 - 97·5)	194	219	4 476	97.8
India	93·2 (91·6 - 94·5)	93·2 (90·0 - 95·7)	1521 486	1439 882	19 449 017	96.1
Indonesia	91·9 (89·3 - 94·0)	87·5 (81·0 - 92·4)	357 089	538 483	4 115 694	95.7
Iran	98·7 (97·8 - 99·3)	98·8 (97·7 - 99·5)	16 074	11 482	802 594	99.0
Iraq	88·0 (84·9 - 90·8)	88·6 (83·5 - 92·5)	103 582	93 409	809 950	93.6
Ireland	95·8 (93·6 - 97·3)	91·0 (85·6 - 94·9)	2 460	4 942	52 348	97.7
Israel	98·3 (96·8 - 99·2)	96·4 (84·9 - 99·5)	3 008	6 652	196 702	99.2
Italy	99·8 (99·4 - 100·0)	99·8 (99·4 - 100·0)	760	674	365 447	99.9
Jamaica	98·5 (97·1 - 99·2)	99·5 (99·0 - 99·8)	521	147	27 771	99.1
Japan	98·6 (97·6 - 99·3)	97·9 (95·9 - 99·1)	12 357	17 845	765 651	99.2
Jordan	92·4 (90·7 - 93·8)	98·6 (97·8 - 99·1)	16 306	3 081	235 494	96.5
Kazakhstan	97·9 (96·5 - 98·8)	97·1 (94·0 - 98·8)	8 273	11 509	360 586	98.9
Kenya	93·6 (90·5 - 96·0)	95·7 (92·4 - 97·9)	77 365	49 983	1 243 344	96.9
Kiribati	63·6 (60·0 - 66·9)	65·6 (59·6 - 70·7)	1 046	981	2 747	81.0
Kuwait	99·1 (98·0 - 99·7)	99·6 (99·0 - 99·9)	474	181	35 021	99.3
Kyrgyzstan	94·6 (93·3 - 95·7)	91·0 (87·3 - 93·8)	8 652	13 439	143 588	97.0
Laos	75·0 (72·5 - 77·2)	78·8 (75·0 - 82·2)	42 824	35 915	151 138	85.8
Latvia	97·9 (96·3 - 98·9)	98·4 (96·6 - 99·3)	394	245	11 499	98.3
Lebanon	90·9 (88·5 - 92·9)	81·1 (74·4 - 86·6)	7 979	14 035	64 674	93.8
Lesotho	98·4 (97·4 - 99·0)	90·9 (83·4 - 95·6)	684	3 720	39 760	99.1
Liberia	91·7 (87·4 - 94·9)	93·5 (88·1 - 96·9)	13 095	10 420	162 709	96.0
Libya	99·4 (98·5 - 99·8)	96·5 (91·3 - 99·1)	514	2 671	68 201	99.6
Lithuania	95·8 (93·6 - 97·4)	93·2 (88·7 - 96·2)	1 127	1 564	18 596	97.0
Luxembourg	99·7 (99·4 - 99·9)	97·6 (87·8 - 99·9)	17	159	6 631	99.9
Madagascar	75·9 (74·0 - 77·6)	72·9 (68·0 - 77·6)	201 289	225 371	882 781	88.6
Malawi	94·6 (92·9 - 96·0)	95·3 (92·4 - 97·3)	29 917	26 906	622 106	97.6
Malaysia	97·3 (94·2 - 99·0)	99·5 (98·7 - 99·9)	13 205	2 545	457 626	98.6
Maldives	91·1 (89·7 - 92·3)	91·6 (89·7 - 93·3)	562	486	4 948	94.3
Mali	75·4 (71·4 - 78·9)	70·9 (63·2 - 77·9)	232 912	309 036	1 258 717	90.7
Malta	97·9 (95·9 - 99·0)	98·4 (96·4 - 99·4)	92	68	3 758	98.8
Marshall Islands	96·8 (94·7 - 98·2)	97·7 (95·2 - 99·0)	37	26	1 210	98.5
Mauritania	90·9 (88·5 - 92·9)	94·4 (91·5 - 96·6)	12 138	7 689	146 919	95.9

Country	Coverage 2019 (mean, lower and upper 95% uncertainty interval) (%)	Coverage 2023 (mean, lower and upper 95% uncertainty interval) (%)	Zero-dose counts in 2019	Zero-dose counts in 2023	Projected 2030 population	2030 coverage goal (%)
Mauritius	97·4 (95·3 - 98·7)	97·4 (94·9 - 98·9)	332	321	11 040	98.5
Mexico	80·7 (77·2 - 83·9)	80·4 (74·7 - 85·4)	387 523	358 647	1 742 480	88.9
Micronesia	97·3 (95·3 - 98·5)	97·9 (95·9 - 99·1)	52	39	1 879	98.6
Moldova	92·6 (90·3 - 94·5)	83·9 (77·8 - 89·2)	2 305	4 277	22 017	94.8
Monaco	99·7 (99·2 - 99·9)	97·5 (87·8 - 99·9)	1	8	286	99.8
Mongolia	93·4 (91·2 - 95·2)	95·5 (92·7 - 97·6)	5 190	3 241	64 525	96.0
Montenegro	94·8 (92·6 - 96·5)	92·5 (88·2 - 95·7)	378	515	6 329	97.0
Morocco	97·4 (95·8 - 98·5)	95·8 (85·2 - 99·1)	17 891	25 591	536 514	98.3
Mozambique	91·3 (89·1 - 93·1)	90·2 (87·1 - 92·9)	90 627	108 089	1 222 840	96.3
Myanmar	88·9 (86·4 - 91·0)	77·0 (70·2 - 82·9)	116 661	240 249	976 330	94.0
N Korea	99·7 (99·6 - 99·8)	31·9 (24·6 - 39·9)	817	191 393	238 690	99.8
N Mariana	82·9 (78·0 - 87·1)	95·3 (92·3 - 97·3)	109	30	665	91.8
Namibia	99·2 (98·3 - 99·7)	96·9 (92·4 - 99·1)	451	1 757	58 715	99.6
Nauru	99·9 (99·7 - 100·0)	97·6 (86·6 - 100·0)	0	7	300	99.9
Nepal	93·4 (91·3 - 95·1)	98·2 (96·9 - 99·1)	41 707	11 145	533 392	96.1
Netherlands	95·5 (93·5 - 97·1)	93·5 (89·4 - 96·4)	7 655	11 566	173 948	97.8
New Zealand	93·9 (91·0 - 95·9)	92·5 (87·6 - 95·8)	3 659	4 627	63 978	97.1
Nicaragua	99·9 (99·5 - 100·0)	99·9 (99·5 - 100·0)	191	176	117 999	99.9
Niger	90·7 (86·9 - 93·6)	92·9 (87·7 - 96·3)	96 452	86 582	1 594 125	97.0
Nigeria	68·7 (63·9 - 73·7)	69·7 (61·3 - 77·5)	2414 338	2477 568	8 852 219	86.4
Niue	99·8 (99·5 - 100·0)	99·8 (99·5 - 100·0)	0	0	23	99.9
North Macedonia	95·9 (94·5 - 97·0)	90·3 (85·8 - 93·9)	841	1 735	14 948	97.2
Norway	99·8 (99·6 - 99·9)	98·8 (97·4 - 99·6)	93	694	58 587	99.9
Oman	99·8 (99·5 - 100·0)	99·8 (99·5 - 100·0)	148	125	72 233	99.9
Pakistan	89·8 (86·5 - 92·4)	93·2 (88·6 - 96·3)	608 781	418 690	6 218 140	95.1
Palau	99·5 (98·7 - 99·9)	99·6 (98·9 - 99·9)	1	1	181	99.7
Palestine	97·2 (96·5 - 97·7)	87·4 (82·8 - 91·1)	3 461	15 336	133 722	98.7
Panama	93·2 (92·1 - 94·1)	82·9 (77·6 - 87·2)	4 940	11 988	72 665	96.6
Papua New Guinea	47·7 (42·8 - 52·4)	42·6 (34·7 - 51·2)	163 590	192 583	343 455	76.2
Paraguay	96·2 (94·1 - 97·6)	80·1 (70·8 - 87·1)	4 895	25 112	119 985	98.0
Peru	98·1 (97·4 - 98·7)	91·5 (85·6 - 95·4)	12 297	55 915	615 720	99.0
Philippines	92·8 (89·8 - 95·2)	96·2 (93·4 - 98·0)	163 889	85 907	2 266 643	96.4
Poland	99·3 (98·3 - 99·7)	96·9 (84·7 - 99·7)	2 762	10 087	255 666	99.5
Portugal	99·3 (98·5 - 99·7)	99·3 (98·4 - 99·8)	595	516	72 446	99.6
Puerto Rico	96·8 (92·5 - 98·9)	95·2 (85·2 - 99·3)	687	848	15 921	97.8
Qatar	97·7 (96·5 - 98·6)	98·1 (96·4 - 99·1)	856	689	27 400	98.4
Romania	96·3 (94·5 - 97·6)	94·0 (83·4 - 97·8)	6 964	10 311	154 459	97.7
Russia	97·1 (95·5 - 98·4)	95·5 (91·9 - 97·9)	43 488	57 097	1 125 638	98.1
Rwanda	99·6 (99·3 - 99·8)	95·0 (91·2 - 97·6)	1 336	18 483	381 003	99.8

Country	Coverage 2019 (mean, lower and upper 95% uncertainty interval) (%)	Coverage 2023 (mean, lower and upper 95% uncertainty interval) (%)	Zero-dose counts in 2019	Zero-dose counts in 2023	Projected 2030 population	2030 coverage goal (%)
S Korea	97·8 (95·9 - 98·9)	97·4 (94·6 - 98·9)	6 542	6 722	243 014	98.7
S. Africa	94·3 (91·5 - 96·3)	89·5 (81·8 - 94·4)	57 450	99 130	890 223	96.8
Samoa	85·6 (83·8 - 87·1)	82·8 (79·5 - 85·8)	856	1 070	6 614	93.5
San Marino	90·6 (87·5 - 93·2)	92·0 (87·7 - 95·0)	23	18	219	94.9
Sao Tome and Principe	96·7 (94·9 - 97·9)	90·0 (84·5 - 94·0)	168	492	5 053	98.3
Saudi Arabia	96·1 (94·1 - 97·4)	96·0 (93·1 - 97·8)	19 075	17 340	353 071	97.3
Senegal	97·2 (96·3 - 98·0)	90·2 (85·9 - 93·5)	13 036	47 227	509 255	98.7
Serbia	96·0 (94·9 - 97·0)	95·4 (93·1 - 97·1)	2 777	3 027	58 683	97.6
Seychelles	99·6 (98·8 - 99·9)	99·4 (98·1 - 99·9)	6	9	1 448	99.8
Sierra Leone	94·6 (92·1 - 96·6)	92·6 (82·2 - 97·0)	15 025	21 876	308 213	97.6
Singapore	99·7 (99·0 - 99·9)	99·8 (99·4 - 100·0)	171	103	46 359	99.8
Slovakia	96·4 (94·5 - 97·8)	96·0 (92·8 - 98·0)	2 061	2 152	44 319	97.7
Slovenia	97·0 (95·6 - 98·0)	91·6 (87·1 - 95·1)	585	1 483	15 486	98.1
Solomon Is.	97·2 (95·5 - 98·5)	93·8 (87·9 - 97·4)	538	1 254	20 074	98.7
Somalia	23·7 (20·0 - 27·9)	25·7 (20·0 - 31·8)	645 562	709 567	1 136 147	71.6
South Sudan	53·6 (39·6 - 66·6)	56·3 (35·4 - 75·2)	151 234	168 077	471 326	84.0
Spain	98·0 (96·6 - 98·9)	96·7 (94·1 - 98·4)	7 390	10 734	288 408	98.7
Sri Lanka	98·6 (97·8 - 99·2)	97·7 (95·9 - 98·9)	4 321	6 553	262 255	99.2
St. Kitts and Nevis	97·3 (95·3 - 98·6)	97·9 (95·5 - 99·2)	16	12	520	98.4
St. Lucia	98·0 (97·0 - 98·8)	94·4 (90·5 - 97·0)	35	91	1 518	98.9
St. Vincent and the Grenadines	99·3 (98·9 - 99·7)	92·1 (86·0 - 96·1)	9	99	1 162	99.6
Sudan	86·5 (84·7 - 87·9)	44·2 (36·4 - 52·4)	157 079	626 917	1 088 460	92.8
Suriname	83·4 (79·8 - 86·9)	86·4 (81·1 - 91·0)	1 497	1 173	8 549	91.2
Sweden	98·1 (96·9 - 98·9)	95·8 (92·4 - 98·0)	2 145	4 705	108 345	99.0
Switzerland	97·4 (96·0 - 98·4)	96·8 (94·2 - 98·4)	2 247	2 820	82 803	98.6
Syria	73·8 (69·4 - 78·0)	69·0 (61·1 - 76·0)	53 190	61 639	219 994	87.9
Taiwan	97·7 (96·1 - 98·8)	99·4 (98·6 - 99·8)	4 000	931	131 485	98.5
Tajikistan	93·5 (91·8 - 94·9)	94·2 (91·5 - 96·2)	17 873	15 822	261 490	96.6
Tanzania	93·4 (91·6 - 94·9)	98·8 (98·0 - 99·3)	119 724	22 051	1 992 457	97.0
Thailand	97·9 (96·9 - 98·5)	96·5 (94·2 - 98·1)	12 111	17 888	445 835	98.6
Timor-Leste	85·4 (82·3 - 88·0)	89·6 (85·9 - 92·6)	5 458	4 308	43 162	93.7
Togo	87·8 (84·6 - 90·5)	89·4 (84·4 - 93·2)	29 329	25 118	236 675	93.8
Tokelau	95·8 (92·9 - 97·9)	99·1 (98·0 - 99·6)	1	0	18	97.9
Tonga	99·8 (99·5 - 99·9)	99·8 (99·6 - 100·0)	6	5	3 126	99.9
Trinidad and Tobago	74·4 (70·8 - 77·7)	86·2 (82·6 - 89·4)	4 079	1 963	12 963	84.3
Tunisia	99·7 (99·3 - 99·9)	99·3 (98·1 - 99·8)	500	1 027	130 153	99.8
Turkiye	93·8 (91·3 - 95·9)	93·1 (82·4 - 97·1)	68 887	67 149	899 861	96.2
Turkmenistan	99·8 (99·4 - 100·0)	99·8 (99·4 - 100·0)	202	179	100 581	99.9

Country	Coverage 2019 (mean, lower and upper 95% uncertainty interval) (%)	Coverage 2023 (mean, lower and upper 95% uncertainty interval) (%)	Zero-dose counts in 2019	Zero-dose counts in 2023	Projected 2030 population	2030 coverage goal (%)
Tuvalu	99·0 (97·3 - 99·7)	99·0 (97·2 - 99·7)	3	3	267	99.5
UAE	96·8 (95·1 - 97·9)	93·4 (89·3 - 96·1)	2 765	4 730	67 023	97.9
Uganda	95·1 (93·2 - 96·5)	94·8 (91·6 - 97·0)	74 352	82 346	1 773 404	97.9
Ukraine	82·9 (78·4 - 86·5)	90·1 (85·6 - 93·7)	53 565	25 341	217 349	87.7
United Kingdom	95·6 (93·5 - 97·1)	94·2 (90·5 - 96·8)	31 575	39 894	654 213	97.6
United States	97·4 (97·0 - 97·8)	95·4 (85·2 - 98·3)	94 931	166 306	3 506 416	98.6
Uruguay	99·6 (99·1 - 99·8)	98·6 (96·9 - 99·5)	167	481	33 946	99.8
Uzbekistan	99·5 (99·1 - 99·8)	99·8 (99·5 - 99·9)	3 678	1 771	730 964	99.7
Vanuatu	93·8 (91·8 - 95·4)	83·8 (78·5 - 88·3)	523	1 414	9 261	97.2
Venezuela	81·8 (78·1 - 85·2)	60·1 (51·4 - 68·3)	90 731	174 530	411 977	89.0
Viet Nam	95·5 (93·9 - 96·8)	78·9 (71·0 - 85·4)	72 765	314 588	1 264 046	97.1
Virgin Isl US	77·4 (57·8 - 91·2)	79·6 (57·3 - 94·2)	188	144	722	87.0
Yemen	62·9 (57·0 - 68·9)	67·8 (58·7 - 76·2)	357 142	309 077	993 480	82.0
Zambia	93·1 (90·9 - 94·7)	76·7 (69·3 - 82·8)	41 587	139 755	643 677	96.8
Zimbabwe	93·7 (90·6 - 96·1)	93·8 (89·1 - 97·1)	28 405	28 658	500 230	97.2

Table S8 Global model fit parameters used as priors in country-level post-introduction scale-up modelling

Scale-up model global fit parameters ± standard error. Parameters fit to global models of coverage scale-up for newer vaccine ratios, used as priors in country-level model fits. DTP3=diphtheria-tetanus-pertussis, third dose. HepB3=hepatitis B vaccine, third dose. Hib3=Haemophilus influenzae type b vaccine, third dose. MCV1=measles-containing vaccine, first dose. MCV2=measles-containing vaccine, second dose. PCV3=pneumococcal conjugate vaccine, third dose. RCV1=rubella-containing vaccine, first dose. RotaC=completed rotavirus series. YSI=years since introduction.

Vaccine ratio	β_0 (intercept)	β_1 (stockout)	β_2 (YSI spline knot 1)	β_3 (YSI spline knot 2)	β_4 (YSI spline knot 3)	β_5 (YSI spline knot 4)	β_6 (YSI spline knot 5)
HepB3/DTP3	-0.989 ± 1.671	-1.236 ± 0.448	6.435 ± 1.329	6.971 ± 1.039	7.694 ± 1.262	8.353 ± 1.316	8.353 ± 1.716
Hib3/DTP3	6.278 ± 1.083	-1.1± 0.614	0.052 ± 0.622	0.207 ± 0.667	0.502 ± 0.765	0.755 ± 0.959	0.895 ± 1.427
MCV2/MCV1	0.632 ± 2.279	-0.975 ± 0.351	1.021 ± 1.723	2.395 ± 1.493	3.819 ± 1.674	3.815 ± 1.639	3.815 ± 1.725
PCV3/DTP3	-0.65 ± 2.421	-1.049 ± 0.364	4.393 ± 1.595	5.486 ± 1.157	5.977 ± 1.333	6.35 ± 1.661	6.582 ± 2.247
RotaC/DTP3	-0.537 ± 3.018	-1.136 ± 0.352	3.899 ± 2.077	4.844 ± 1.397	5.526 ± 1.496	6.032 ± 1.866	6.113 ± 1.877
RCV1/MCV1	6.871 ± 0.741	-1.014 ± 0.716	0.001 ± 0.705	0.005 ± 0.745	0.012 ± 0.765	0.018 ± 0.789	0.022 ± 0.805

Table S9 Validation metrics for ST-GPR models

In-sample and out-of-sample (using 5-fold cross validation modelling) metrics assessing goodness of fit for vaccine coverage and coverage ratio models fit using ST-GPR. MedAE=median absolute error. MedErr=median error. RMSE=root mean square error. BCG=Bacillus Calmette-Guérin. DTP1=diphtheria-tetanus-pertussis, first dose. DTP3=diphtheria-tetanus-pertussis, third dose. HepB3=hepatitis B vaccine, third dose. Hib3=Haemophilus influenzae type b vaccine, third dose. MCV1=measles-containing vaccine, first dose. MCV2=measles-containing vaccine, second dose. PCV3=pneumococcal conjugate vaccine, third dose. RCV1=rubella-containing vaccine, first dose. RotaC=completed rotavirus series. ST-GPR= Spatiotemporal Gaussian Process Regression.

Model Name	In Sample MedAE	Out of Sample MedAE	In Sample MedErr	Out of Sample MedErr	In Sample RMSE	Out of Sample RMSE
BCG coverage	0.0277	0.0532	0.0074	0.0012	0.0748	0.1262
DTP1 coverage	0.0187	0.0352	0.0069	0.0057	0.0608	0.1126
MCV1 coverage	0.0352	0.0662	0.0089	0.0125	0.0793	0.1372
Pol3 coverage	0.0354	0.0621	0.0105	0.0078	0.0747	0.1409
DTP3-DTP1 ratio	0.0238	0.0402	0.0032	-0.0011	0.0762	0.1183
HepB3-DTP3 ratio	0.0164	0.0279	0.001	0.0009	0.1274	0.1964
Hib3-DTP3 ratio	0.0085	0.0122	0.0008	0.0002	0.2265	0.2408
PCV3-DTP3 ratio	0.0183	0.0286	0.0041	0.0061	0.1191	0.1343
RotaC-DTP3 ratio	0.0248	0.0396	0.001	0.0011	0.0558	0.1078
MCV2-MCV1 ratio	0.0963	0.1274	-0.045	-0.0793	0.2789	0.302
RCV1-MCV1 ratio	0.0009	0.0022	<0.0001	<0.0001	0.0979	0.1032

Table S10 Validation metrics for MR-BRT first stage models

In-sample and out-of-sample (using 5-fold cross validation modelling) metrics assessing goodness of fit for vaccine coverage ratio models fit as first stage models for ST-GPR where scale-up for newer vaccine ratios was fit using MR-BRT. MedAE=median absolute error. MedErr=median error. RMSE=root mean square error. DTP3=diphtheria-tetanus-pertussis, third dose. HepB3=hepatitis B vaccine, third dose. Hib3=Haemophilus influenzae type b vaccine, third dose. MCV1=measles-containing vaccine, first dose. MCV2=measles-containing vaccine, second dose. PCV3=pneumococcal conjugate vaccine, third dose. RCV1=rubella-containing vaccine, first dose. RotaC=completed rotavirus series. MR-BRT= Meta Regression; Bayesian; Regularized; Trimmed.

Model Name	In Sample MedAE	Out of Sample MedAE	In Sample MedErr	Out of Sample MedErr	In Sample RMSE	Out of Sample RMSE
HepB3-DTP3 ratio	0.0359	0.037	-0.0334	-0.0333	0.1556	0.1783
Hib3-DTP3 ratio	0.0133	0.0122	-0.0133	-0.0122	0.0785	0.0836
PCV3-DTP3 ratio	0.0403	0.0415	-0.0318	-0.0283	0.1403	0.1499
RotaC-DTP3 ratio	0.2964	0.3055	-0.2954	-0.3043	0.3165	0.3309
MCV2-MCV1 ratio	0.1223	0.1175	-0.1222	-0.1175	0.3271	0.3278
RCV1-MCV1 ratio	0.0005	0.0004	-0.0003	-0.0001	0.0592	0.0524

Section 6 Supplemental figures

Figure S1 Flow chart of modelling processes

Conceptual flow of GBD vaccine coverage modelling pipeline. White ovals refer to screened and processed vaccination coverage data, covariates are shown as blue cylinders, modelling and processing steps are shown in light peach rectangles, intermediate outputs are shown in green parallelograms, and final coverage outputs are represented with the blue parallelogram. Estimation begins with data preparation steps (upper light blue box) and ends with coverage modelling and post-processing (lower light blue box). JRF=Joint Reporting Form. SDI=Socio-demographic Index. ST-GPR=spatiotemporal Gaussian process regression. HAQ Index=Healthcare Access and Quality Index. MR-BRT=meta-regression—Bayesian, regularised, trimmed. GAM=generalize additive model. UI=uncertainty interval. BCG=Bacillus Calmette-Guérin. DTP1=diphtheria-tetanus-pertussis, first dose. DTP3=diphtheria-tetanus-pertussis, third dose. HepB3=hepatitis B vaccine, third dose. Hib3=Haemophilus influenzae type b vaccine, third dose. MCV1=measles-containing vaccine, first dose. MCV2=measles-containing vaccine, second dose. PCV3=pneumococcal conjugate vaccine, third dose. Pol3=polio vaccine, third dose. RCV1=rubella-containing vaccine, first dose. RotaC=completed rotavirus series.

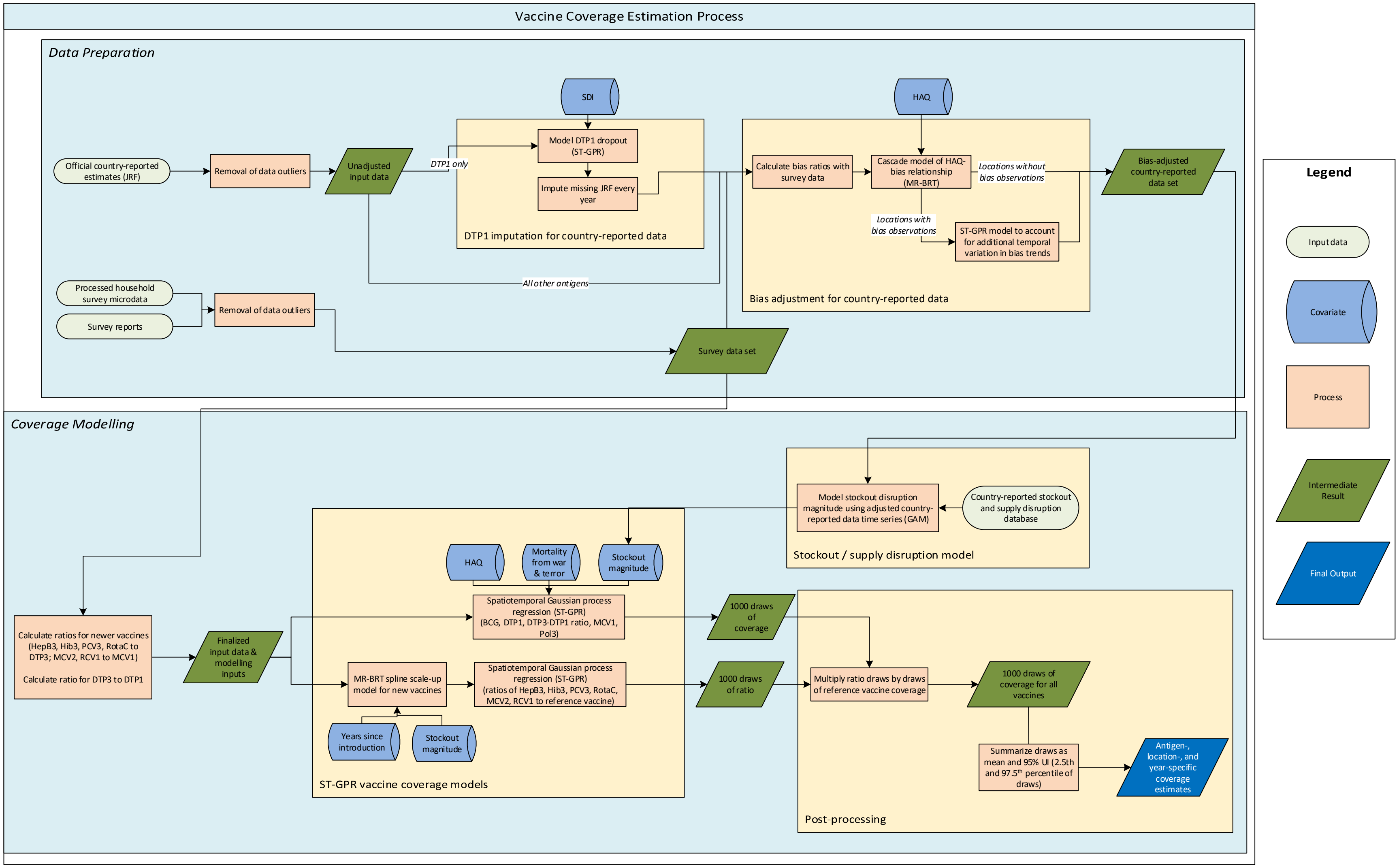

Vaccine Coverage Estimation Process
Data Preparation
SDI
HAQ
Official country-reported estimates (JRF)
Removal of data outliers
Unadjusted input data
DTP1 only
Model DTP1 dropout (ST-GPR)
Impute missing JRF every year
DTP1 imputation for country-reported data
Calculate bias ratios with survey data
Cascade model of HAQ-bias relationship (MR-BRT)
Locations without bias observations
Locations with bias observations
ST-GPR model to account for additional temporal variation in bias trends
Bias adjustment for country-reported data
Bias-adjusted country-reported data set
All other antigens
Processed household survey microdata
Survey reports
Removal of data outliers
Survey data set
Coverage Modelling
Model stockout disruption magnitude using adjusted country-reported data time series (GAM)
Country-reported stockout and supply disruption database
Stockout / supply disruption model
HAQ
Mortality from war & terror
Stockout magnitude
Spatiotemporal Gaussian process regression (ST-GPR) (BCG, DTP1, DTP3-DTP1 ratio, MCV1, Pol3)
Calculate ratios for newer vaccines (HepB3, Hib3, PCV3, RotaC to DTP3; MCV2, RCV1 to MCV1)
Calculate ratio for DTP3 to DTP1
Finalized input data & modelling inputs
MR-BRT spline scale-up model for new vaccines
Spatiotemporal Gaussian process regression (ST-GPR) (ratios of HepB3, Hib3, PCV3, RotaC, MCV2, RCV1 to reference vaccine)
Years since introduction
Stockout magnitude
ST-GPR vaccine coverage models
1000 draws of coverage
1000 draws of ratio
Multiply ratio draws by draws of reference vaccine coverage
1000 draws of coverage for all vaccines
Summarize draws as mean and 95% UI (2.5th and 97.5th percentile of draws)
Antigen-, location-, and year-specific coverage estimates
Post-processing
Legend
Input data
Covariate
Process
Intermediate Result
Final Output

Figure S2 Flow chart of data screening processes

Flow chart describing the processes for intake of raw survey and country reported data (blue squares) and screening processes (green ovals) to generate intermediate data (turquoise squares), to exclude data given listed criteria (red squares), and to ultimately derive final model input data (purple square). GHDx=Global Health Data Exchange. WUENIC=WHO-UNICEF Estimates of National Immunization Coverage. N= sample size.

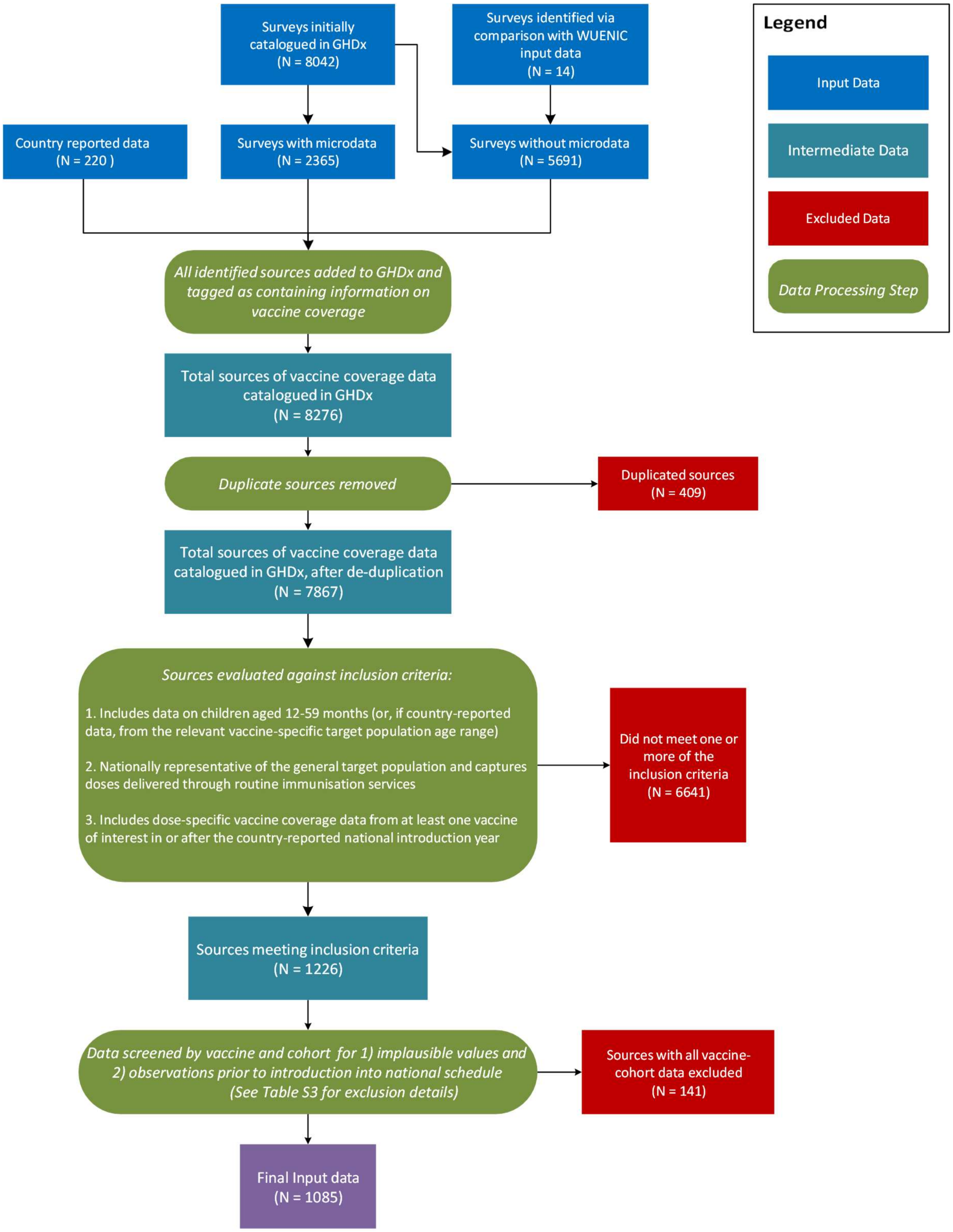

Figure S3 Predicted fits of global scale-up models by newer vaccine ratio

These predictions of scale up of coverage ratios with 95% uncertainty intervals across years-since-introduction (YSI) were fit using meta-regression—Bayesian, regularised, trimmed (MR-BRT) across a global dataset. The parameters fit in these global models served as priors for country-specific models where data was unavailable. DTP3=diphtheria-tetanus-pertussis, third dose. HepB3=hepatitis B vaccine, third dose. Hib3=Haemophilus influenzae type b vaccine, third dose. MCV1=measles-containing vaccine, first dose. MCV2=measles-containing vaccine, second dose. PCV3=pneumococcal conjugate vaccine, third dose. RCV1=rubella-containing vaccine, first dose. RotaC=completed rotavirus series.

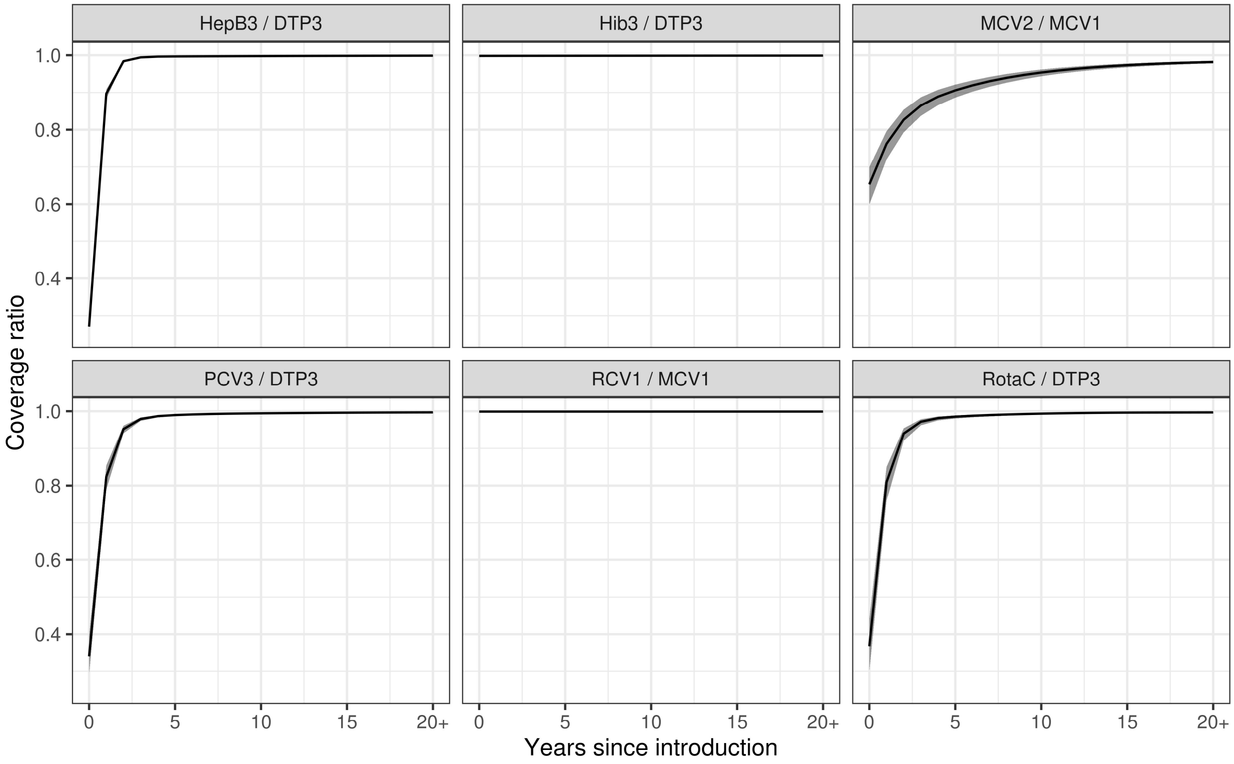

Figure S4 Global disruption magnitudes by year

The global population-weighted mean aggregate percent difference, by vaccine or vaccine ratio, between estimates including and excluding disruptions by year and vaccine (ie, disruption magnitude). Blue bars indicate disruption magnitudes in years preceding COVID, compared to red bars indicating disruption magnitudes during the COVID-19 pandemic. Vaccine-country-draw means across years preceding the COVID-19 pandemic (ie, 2015–2019) were used to subsequently adjust disruption levels during the pandemic to account for disruptions that may have occurred absent the COVID-19 pandemic. BCG=Bacillus Calmette-Guérin. DTP1=diphtheria-tetanus-pertussis, first dose. DTP3=diphtheria-tetanus-pertussis, third dose. HepB3=hepatitis B vaccine, third dose. Hib3=Haemophilus influenzae type b vaccine, third dose. MCV1=measles-containing vaccine, first dose. MCV2=measles-containing vaccine, second dose. PCV3=pneumococcal conjugate vaccine, third dose. Pol3=polio vaccine, third dose. RCV1=rubella-containing vaccine, first dose. RotaC=completed rotavirus series.

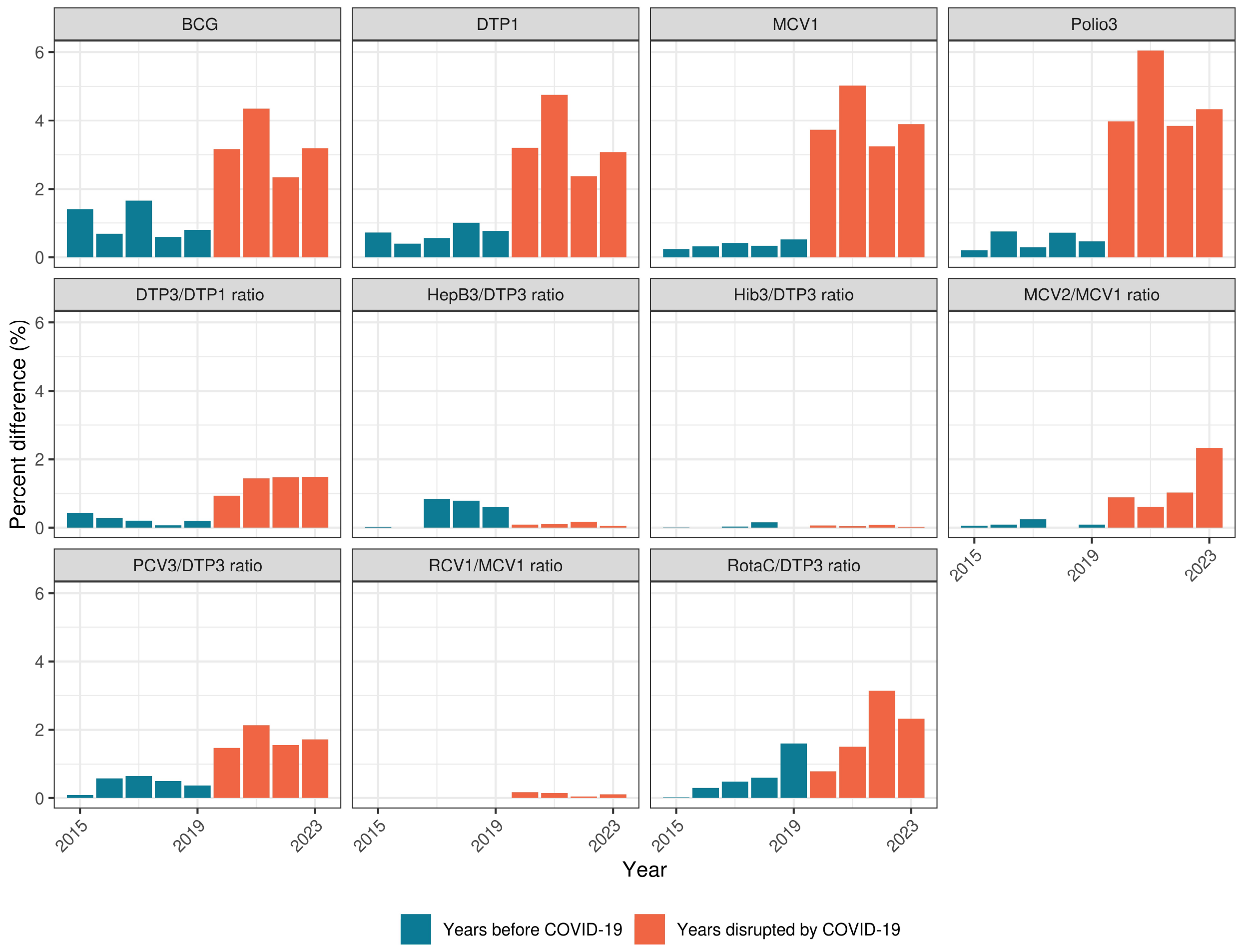

Figure S5 Vaccine coverage time series plots

Vaccine coverage time series. Country- and vaccine-specific estimates of coverage proportions for the target population, 1980-2023. Data used in the model are coloured in black. Data excluded from the model are coloured in light blue. Point shapes correspond to different input data types. The solid green line illustrates estimated annual coverage. 95% uncertainty intervals are represented by the shaded, light green band around each mean estimate. WUENIC estimates released July 2024 are shown in light blue. WUENIC=WHO-UNICEF Estimates of National Immunization Coverage. Original Country reported= raw, unadjusted official country-reported data from the Joint Reporting Form (JRF). GBD = Global Burden of Disease. Country-reported=official country-reported data from the JRF, after bias adjustment. DHS=Demographics and Health Survey. MICS=Multiple Indicator Cluster Survey. Other microdata= Non-DHS, non-MICS survey microdata. Other tabulation=survey report tabulation. Imputed=statistically imputed as described in appendix 1 section 1.2. BCG=Bacillus Calmette-Guérin. DTP1=diphtheria-tetanus-pertussis, first dose. DTP3=diphtheria-tetanus-pertussis, third dose. HepB3=hepatitis B vaccine, third dose. Hib3=Haemophilus influenzae type b vaccine, third dose. MCV1=measles-containing vaccine, first dose. MCV2=measles-containing vaccine, second dose. PCV3=pneumococcal conjugate vaccine, third dose. Pol3=polio vaccine, third dose. RCV1=rubella-containing vaccine, first dose. RotaC=completed rotavirus series.

Afghanistan (AFG)

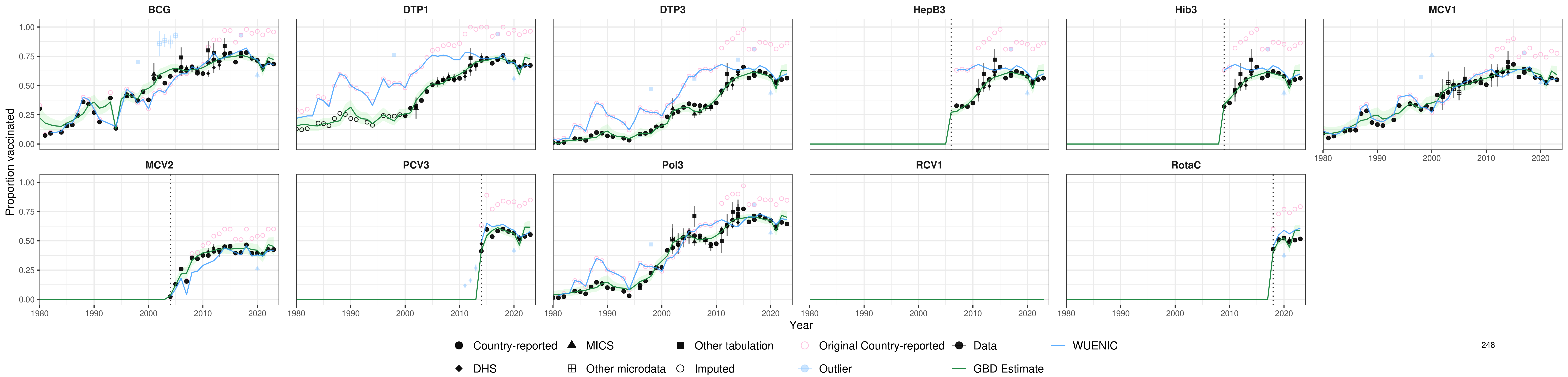

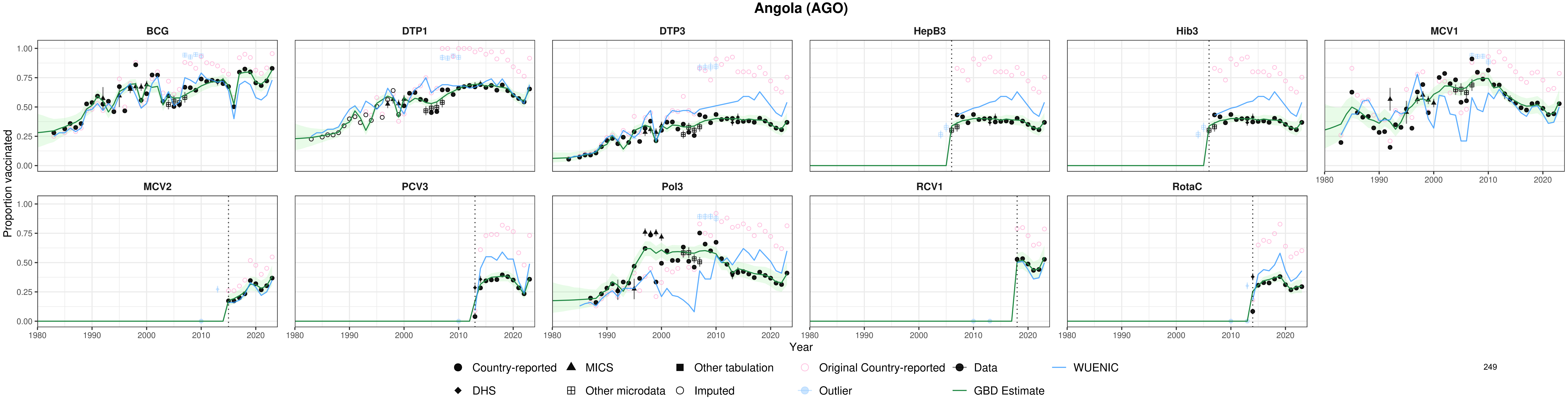
Angola (AGO)
BCG
DTP1
DTP3
HepB3
Hib3
MCV1
MCV2
PCV3
Pol3
RCV1
RotaC
Proportion vaccinated
0.00
0.25
0.50
0.75
1.00
1980
1990
2000
2010
2020
Year
Country-reported
MICS
Other tabulation
Original Country-reported
Data
WUENIC
DHS
Other microdata
Imputed
Outlier
GBD Estimate

Albania (ALB)

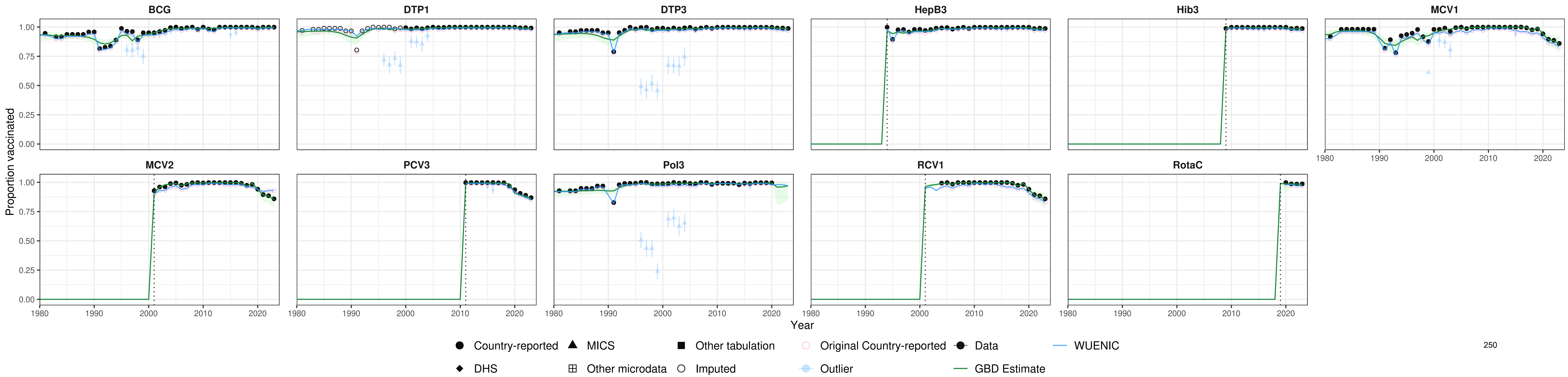
BCG
DTP1
DTP3
HepB3
Hib3
MCV1
MCV2
PCV3
Pol3
RCV1
RotaC
Proportion vaccinated
Year
1.00
0.75
0.50
0.25
0.00
1980
1990
2000
2010
2020
Country-reported
MICS
Other tabulation
Original Country-reported
Data
WUENIC
DHS
Other microdata
Imputed
Outlier
GBD Estimate

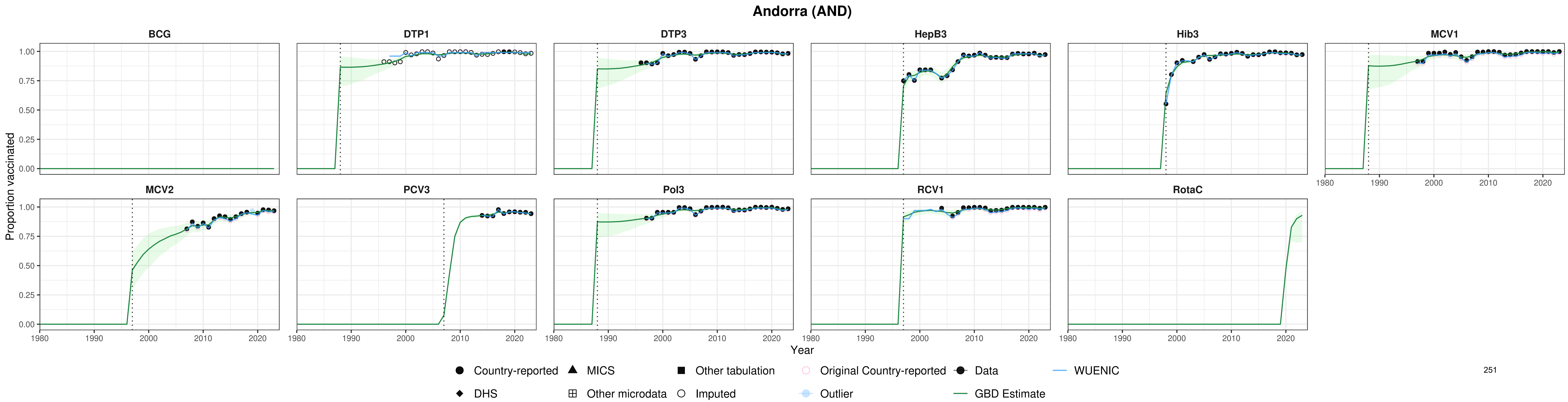
Andorra (AND)
BCG
DTP1
DTP3
HepB3
Hib3
MCV1
MCV2
PCV3
Pol3
RCV1
RotaC
Proportion vaccinated
0.00
0.25
0.50
0.75
1.00
1980
1990
2000
2010
2020
Year
Country-reported
MICS
Other tabulation
Original Country-reported
Data
WUENIC
DHS
Other microdata
Imputed
Outlier
GBD Estimate

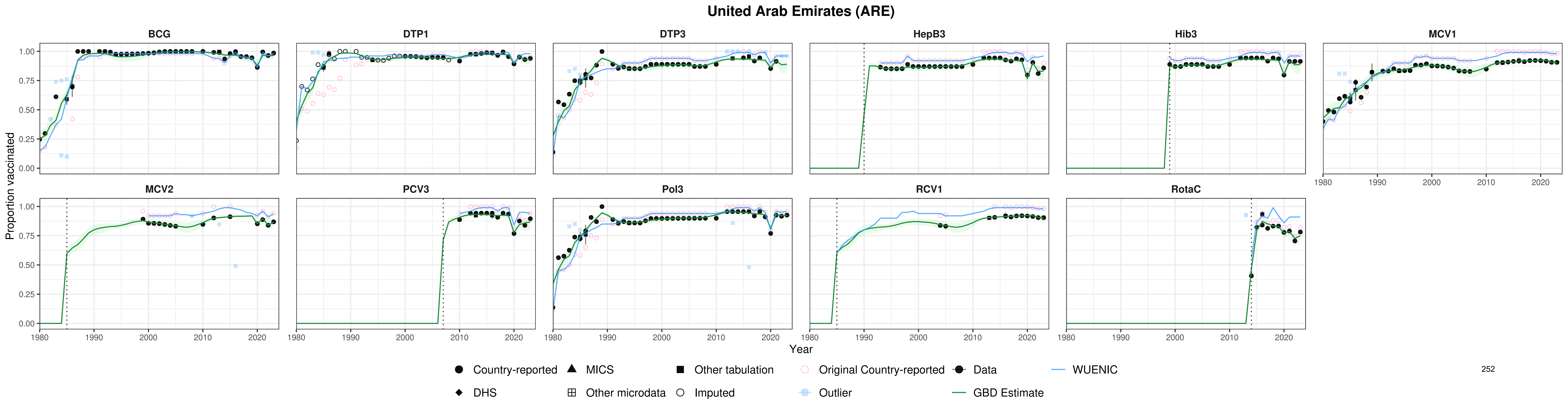
United Arab Emirates (ARE)
BCG
DTP1
DTP3
HepB3
Hib3
MCV1
MCV2
PCV3
Pol3
RCV1
RotaC
Proportion vaccinated
Year
Country-reported
MICS
Other tabulation
Original Country-reported
Data
WUENIC
DHS
Other microdata
Imputed
Outlier
GBD Estimate

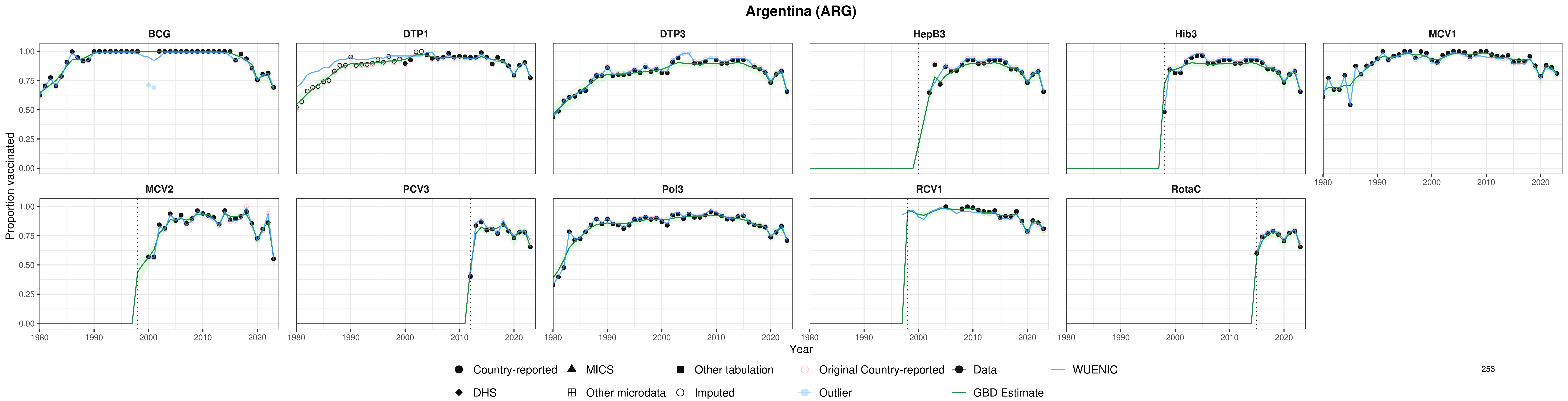

Argentina (ARG)
BCG
DTP1
DTP3
HepB3
Hib3
MCV1
MCV2
PCV3
Pol3
RCV1
RotaC
Proportion vaccinated
Year
Country-reported
MICS
Other tabulation
Original Country-reported
Data
WUENIC
DHS
Other microdata
Imputed
Outlier
GBD Estimate

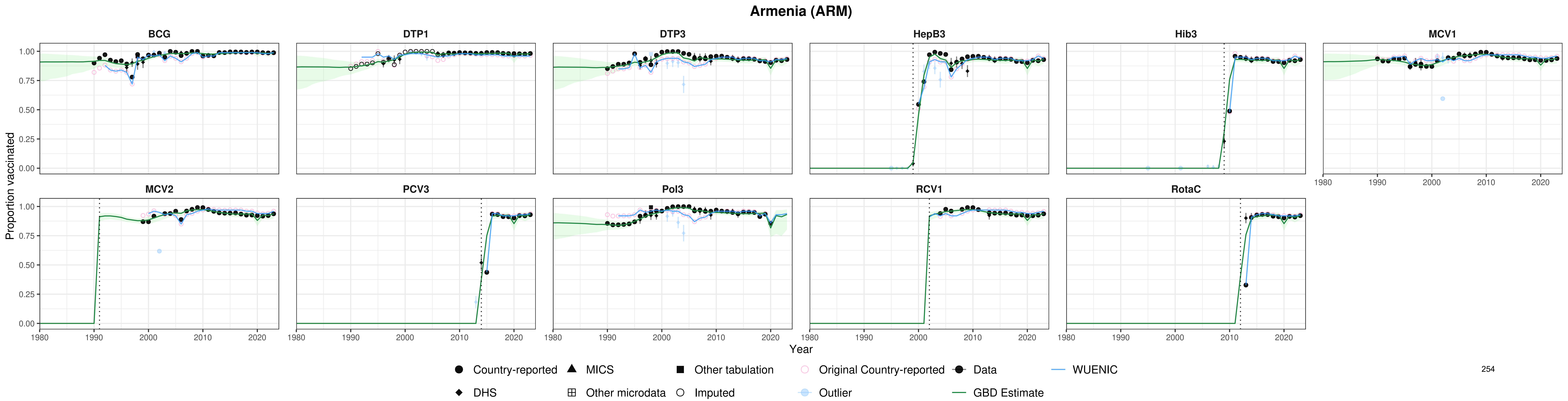
Armenia (ARM)
BCG
DTP1
DTP3
HepB3
Hib3
MCV1
MCV2
PCV3
Pol3
RCV1
RotaC
Proportion vaccinated
Year
Country-reported
MICS
Other tabulation
Original Country-reported
Data
WUENIC
DHS
Other microdata
Imputed
Outlier
GBD Estimate

American Samoa (ASM)

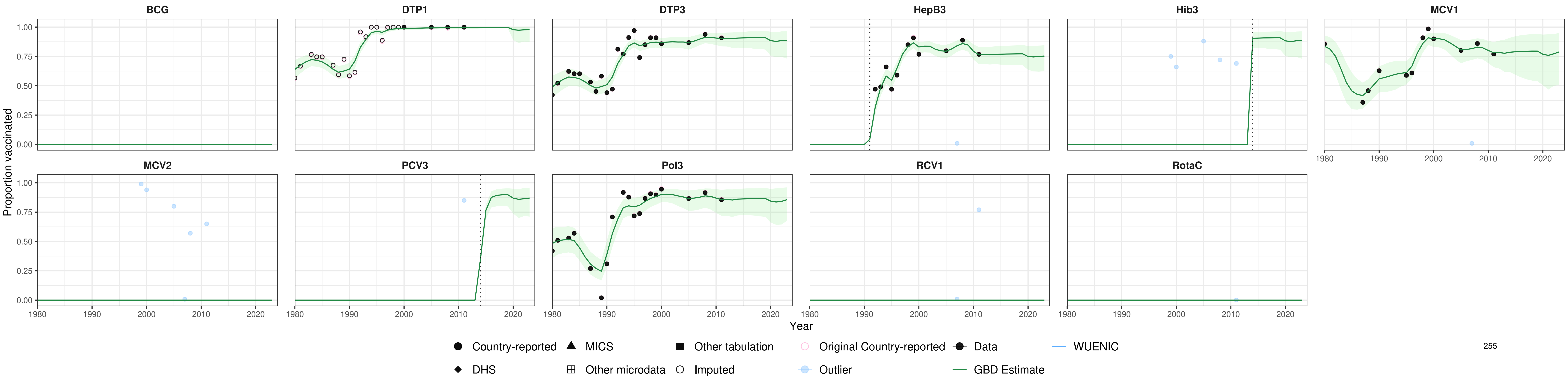

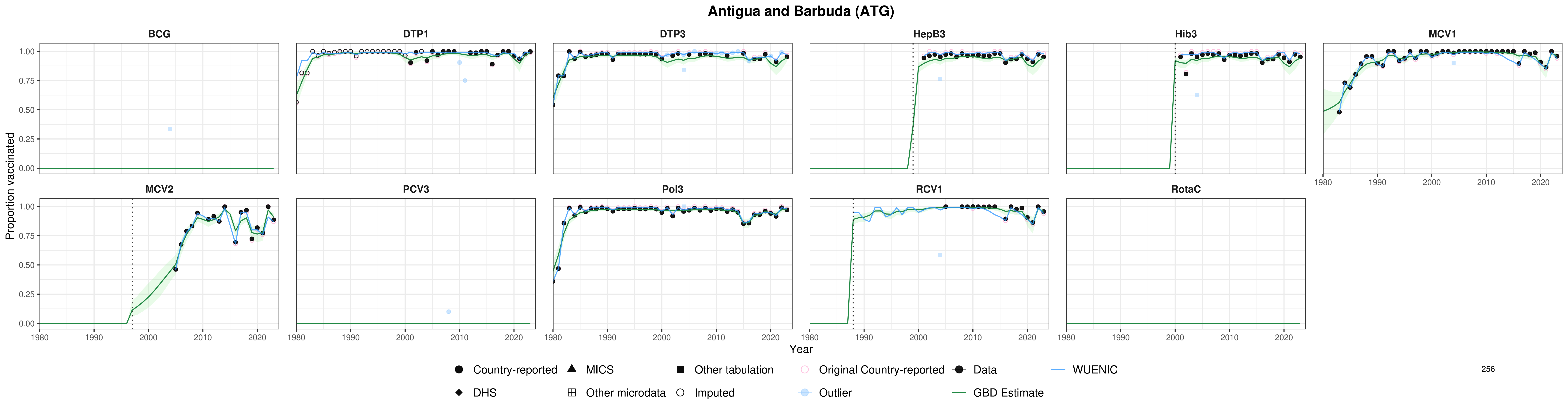

Antigua and Barbuda (ATG)
BCG
DTP1
DTP3
HepB3
Hib3
MCV1
MCV2
PCV3
Pol3
RCV1
RotaC
Proportion vaccinated
Year
Country-reported
MICS
Other tabulation
Original Country-reported
Data
WUENIC
DHS
Other microdata
Imputed
Outlier
GBD Estimate

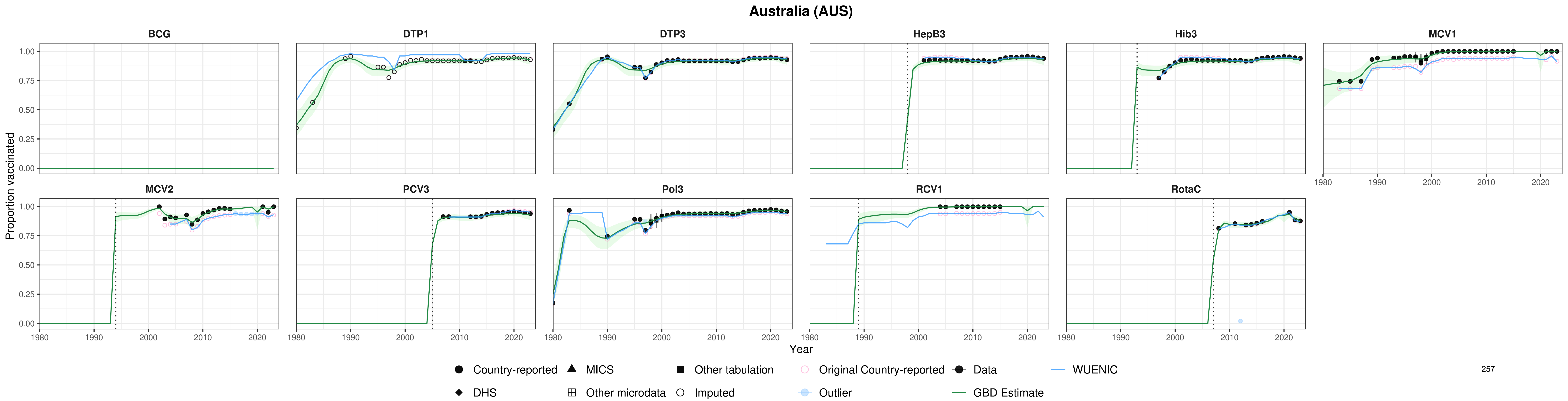
Australia (AUS)
BCG
DTP1
DTP3
HepB3
Hib3
MCV1
MCV2
PCV3
Pol3
RCV1
RotaC
Proportion vaccinated
Year
Country-reported
MICS
Other tabulation
Original Country-reported
Data
WUENIC
DHS
Other microdata
Imputed
Outlier
GBD Estimate

Austria (AUT)

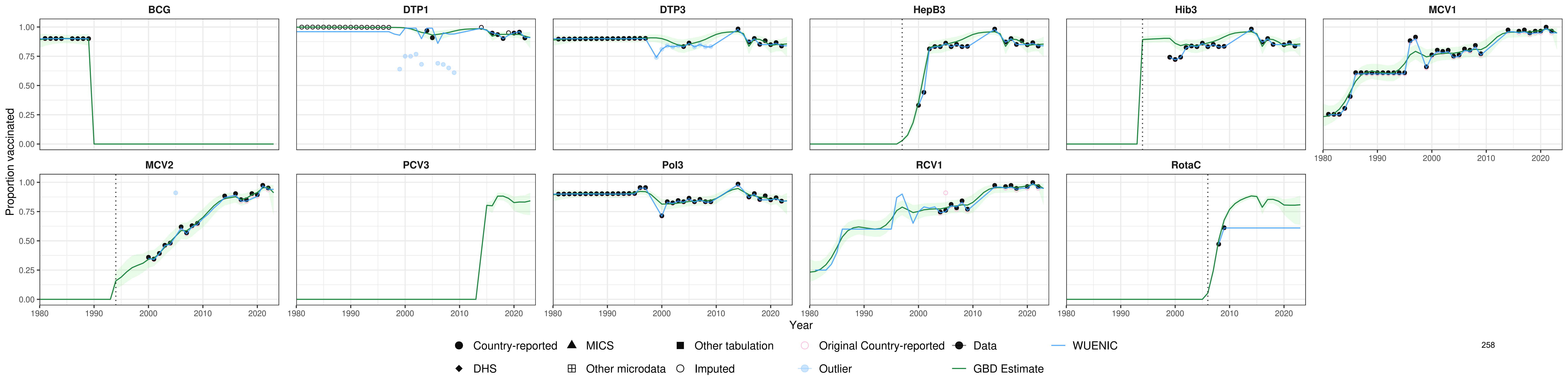

BCG
DTP1
DTP3
HepB3
Hib3
MCV1
MCV2
PCV3
Pol3
RCV1
RotaC
Proportion vaccinated
Year
1.00
0.75
0.50
0.25
0.00
1980
1990
2000
2010
2020
Country-reported
MICS
Other tabulation
Original Country-reported
Data
WUENIC
DHS
Other microdata
Imputed
Outlier
GBD Estimate

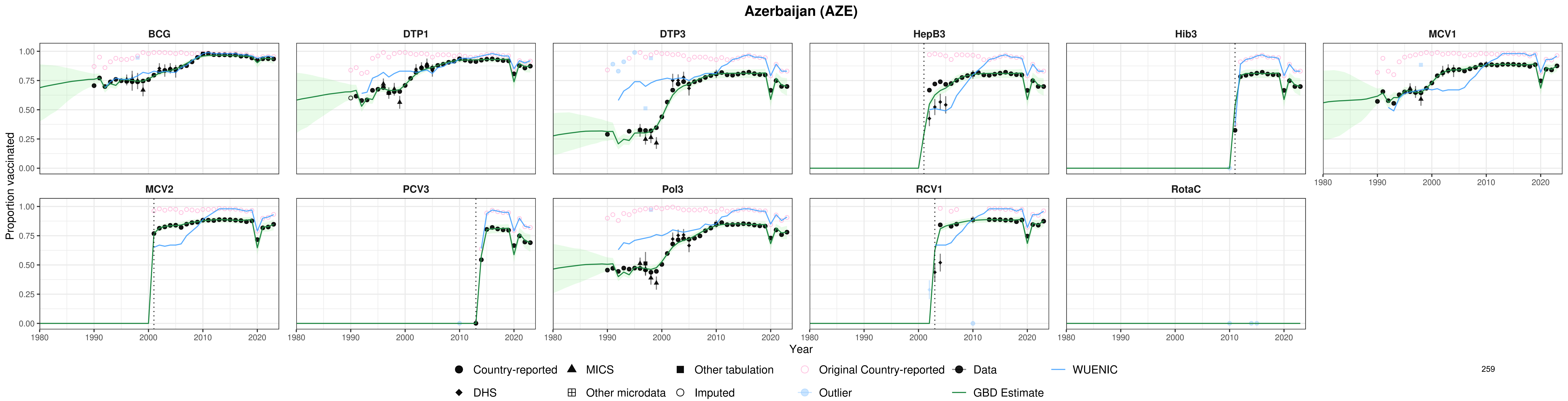
Azerbaijan (AZE)
BCG
DTP1
DTP3
HepB3
Hib3
MCV1
MCV2
PCV3
Pol3
RCV1
RotaC
Proportion vaccinated
Year
Country-reported
MICS
Other tabulation
Original Country-reported
Data
WUENIC
DHS
Other microdata
Imputed
Outlier
GBD Estimate

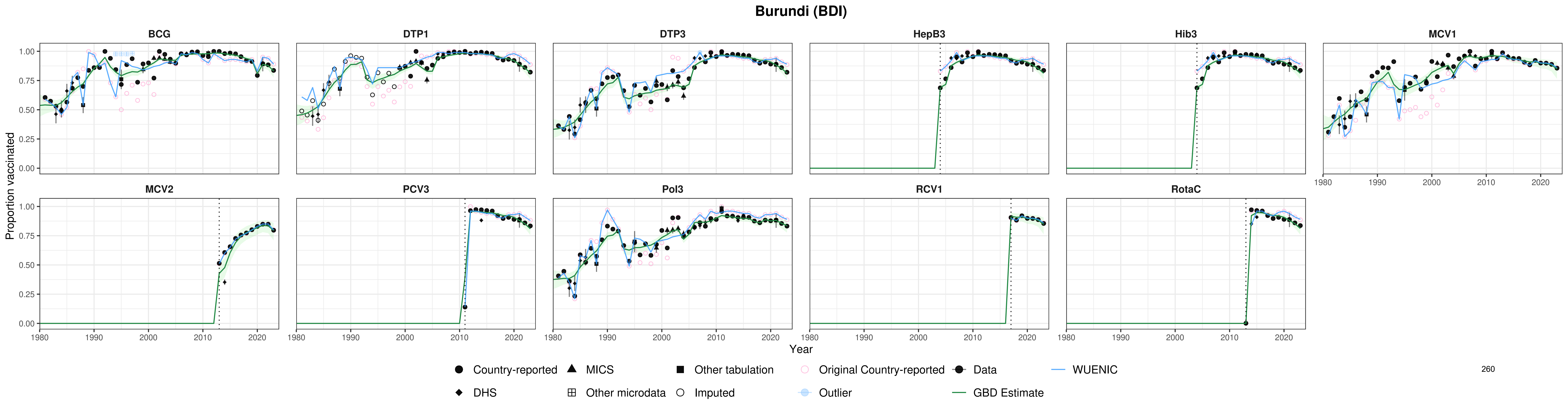

Burundi (BDI)
BCG
DTP1
DTP3
HepB3
Hib3
MCV1
MCV2
PCV3
Pol3
RCV1
RotaC
Proportion vaccinated
Year
Country-reported
DHS
MICS
Other microdata
Other tabulation
Imputed
Original Country-reported
Outlier
Data
GBD Estimate
WUENIC

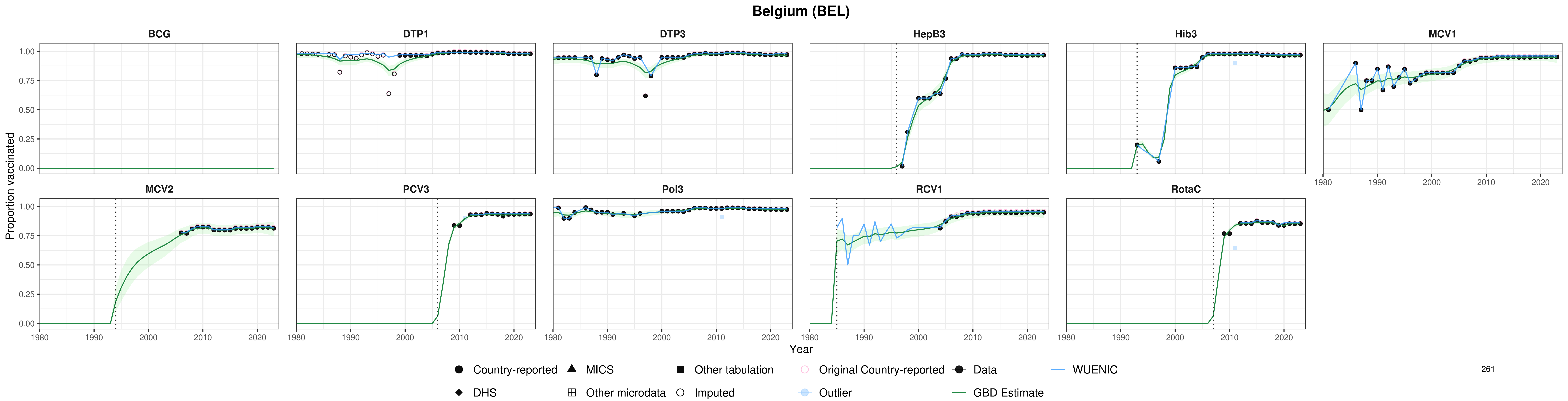

Belgium (BEL)
BCG
DTP1
DTP3
HepB3
Hib3
MCV1
MCV2
PCV3
Pol3
RCV1
RotaC
Proportion vaccinated
Year
Country-reported
MICS
Other tabulation
Original Country-reported
Data
WUENIC
DHS
Other microdata
Imputed
Outlier
GBD Estimate

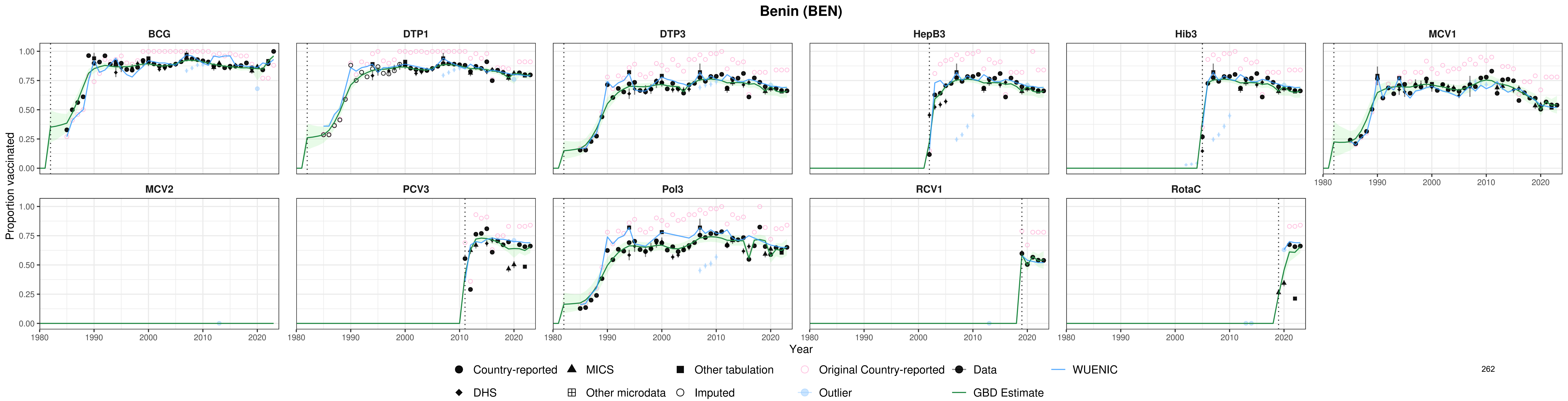
Benin (BEN)
BCG
DTP1
DTP3
HepB3
Hib3
MCV1
MCV2
PCV3
Pol3
RCV1
RotaC
Proportion vaccinated
Year
Country-reported
MICS
Other tabulation
Original Country-reported
Data
WUENIC
DHS
Other microdata
Imputed
Outlier
GBD Estimate

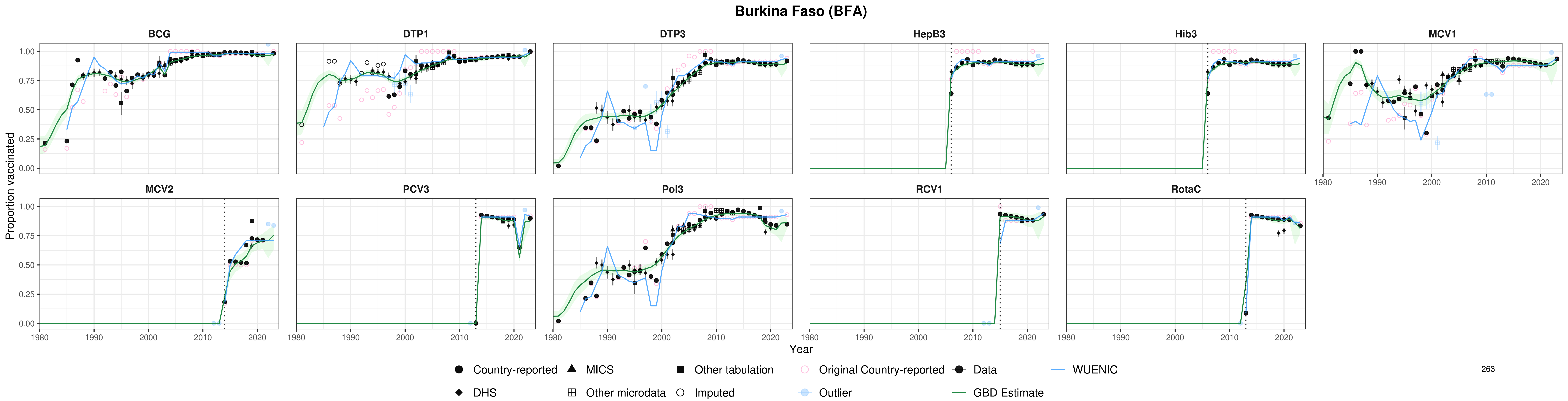

Burkina Faso (BFA)
BCG
DTP1
DTP3
HepB3
Hib3
MCV1
MCV2
PCV3
Pol3
RCV1
RotaC
Proportion vaccinated
Year
Country-reported
MICS
Other tabulation
Original Country-reported
Data
WUENIC
DHS
Other microdata
Imputed
Outlier
GBD Estimate

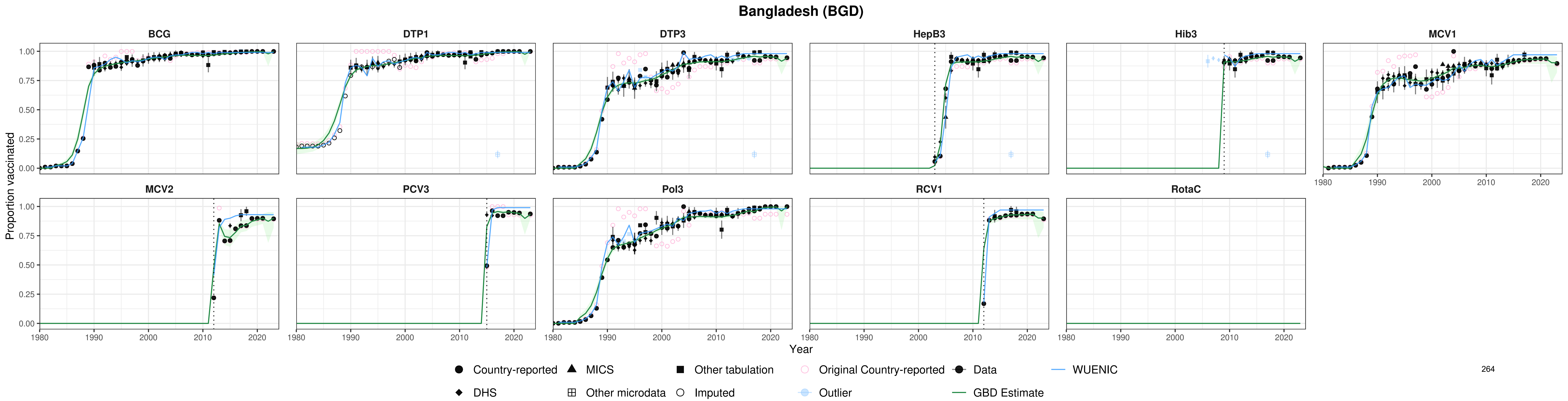
Bangladesh (BGD)
BCG
DTP1
DTP3
HepB3
Hib3
MCV1
MCV2
PCV3
Pol3
RCV1
RotaC
Proportion vaccinated
0.00
0.25
0.50
0.75
1.00
1980
1990
2000
2010
2020
Year
Country-reported
DHS
MICS
Other microdata
Other tabulation
Imputed
Original Country-reported
Outlier
Data
GBD Estimate
WUENIC

Bulgaria (BGR)

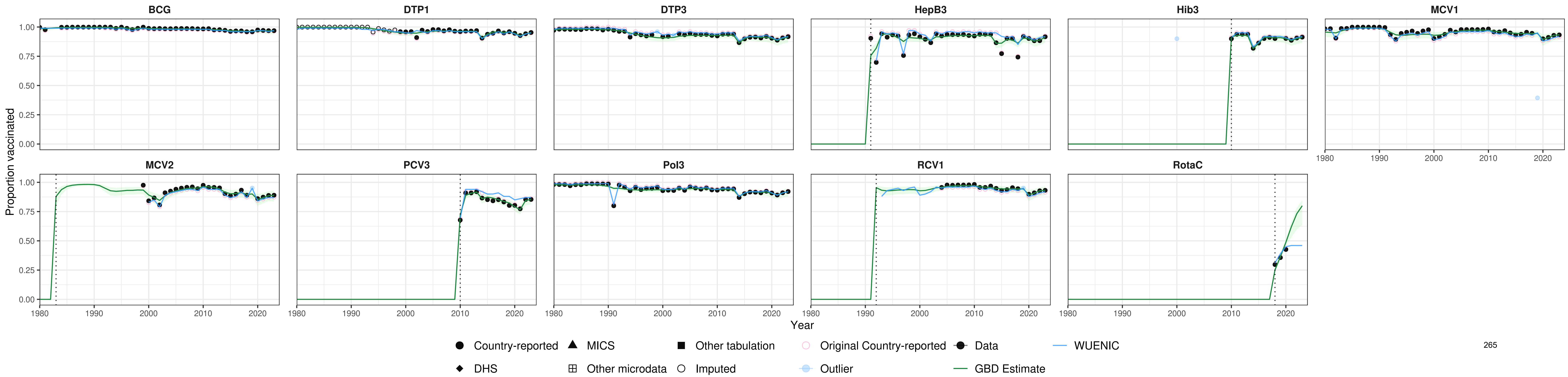

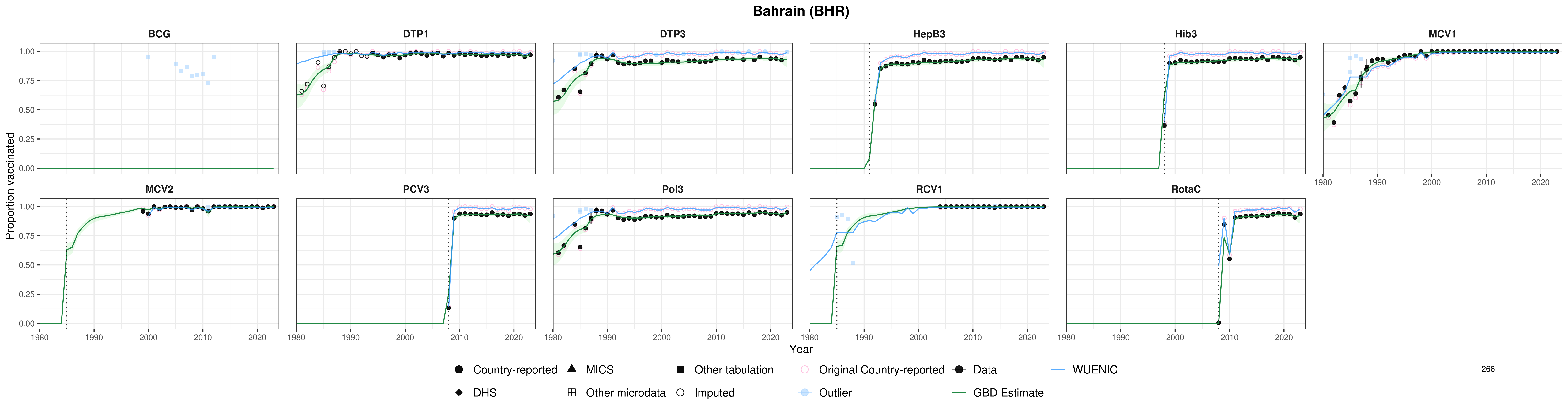
Bahrain (BHR)
BCG
DTP1
DTP3
HepB3
Hib3
MCV1
MCV2
PCV3
Pol3
RCV1
RotaC
Proportion vaccinated
Year
Country-reported
MICS
Other tabulation
Original Country-reported
Data
WUENIC
DHS
Other microdata
Imputed
Outlier
GBD Estimate

The Bahamas (BHS)

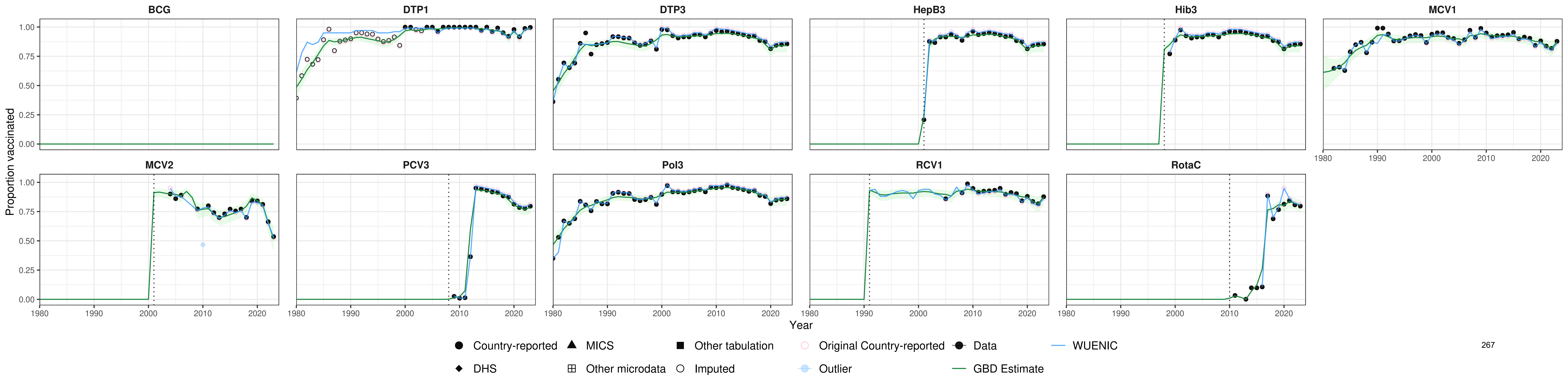

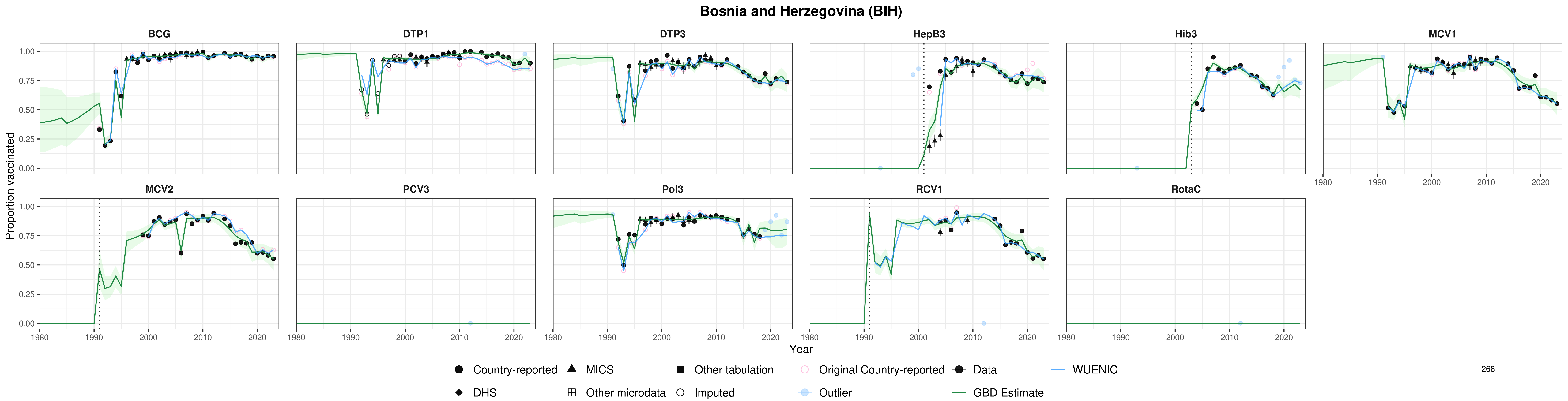
Bosnia and Herzegovina (BIH)
BCG
DTP1
DTP3
HepB3
Hib3
MCV1
MCV2
PCV3
Pol3
RCV1
RotaC
Proportion vaccinated
Year
1.00
0.75
0.50
0.25
0.00
1980
1990
2000
2010
2020
Country-reported
MICS
Other tabulation
Original Country-reported
Data
WUENIC
DHS
Other microdata
Imputed
Outlier
GBD Estimate

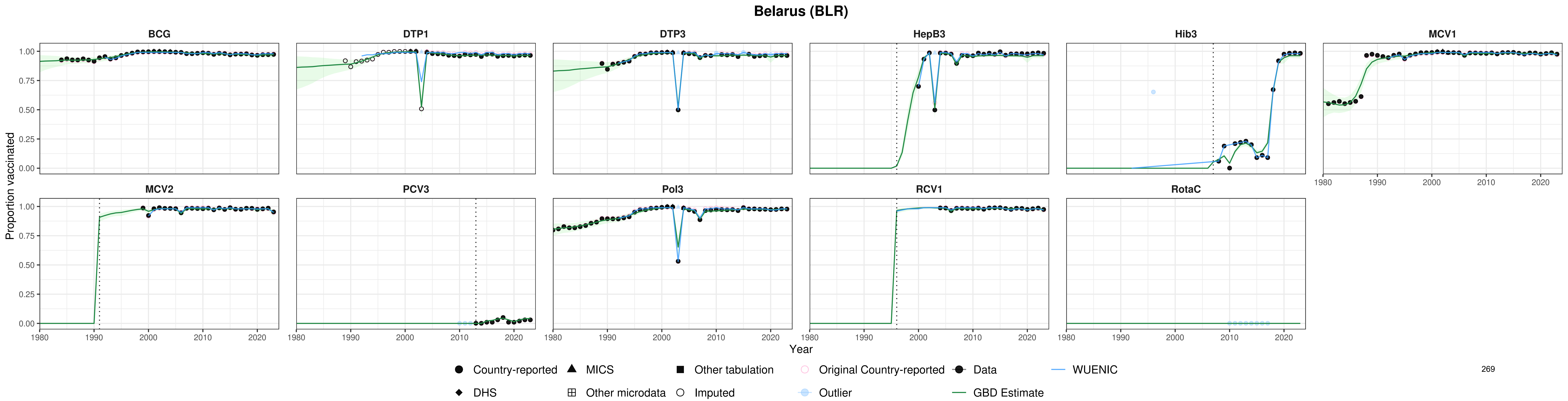

Belarus (BLR)
BCG
DTP1
DTP3
HepB3
Hib3
MCV1
MCV2
PCV3
Pol3
RCV1
RotaC
Proportion vaccinated
Year
Country-reported
MICS
Other tabulation
Original Country-reported
Data
WUENIC
DHS
Other microdata
Imputed
Outlier
GBD Estimate

Belize (BLZ)

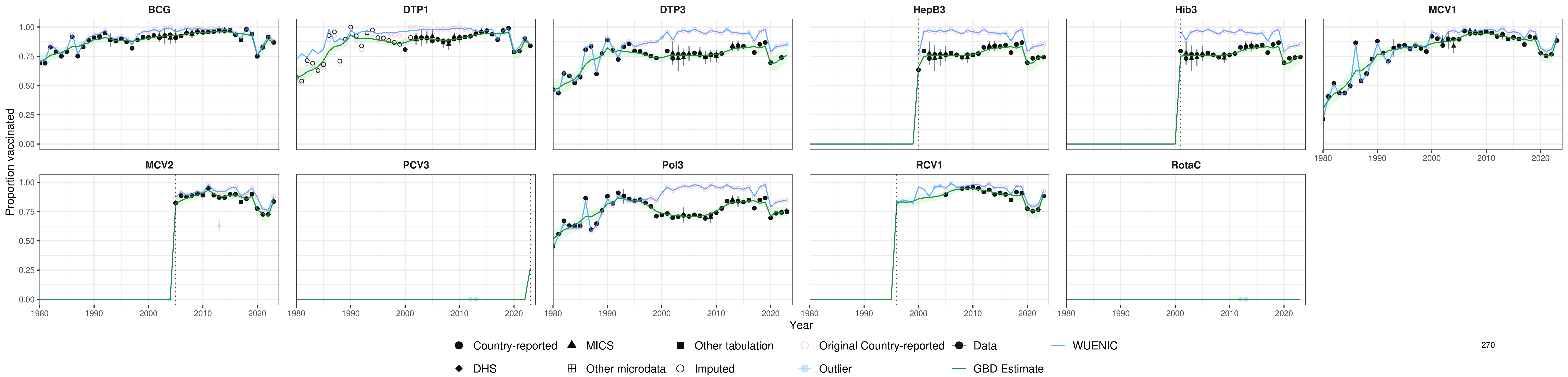
BCG
DTP1
DTP3
HepB3
Hib3
MCV1
MCV2
PCV3
Pol3
RCV1
RotaC
Proportion vaccinated
Year
Country-reported
MICS
Other tabulation
Original Country-reported
Data
WUENIC
DHS
Other microdata
Imputed
Outlier
GBD Estimate

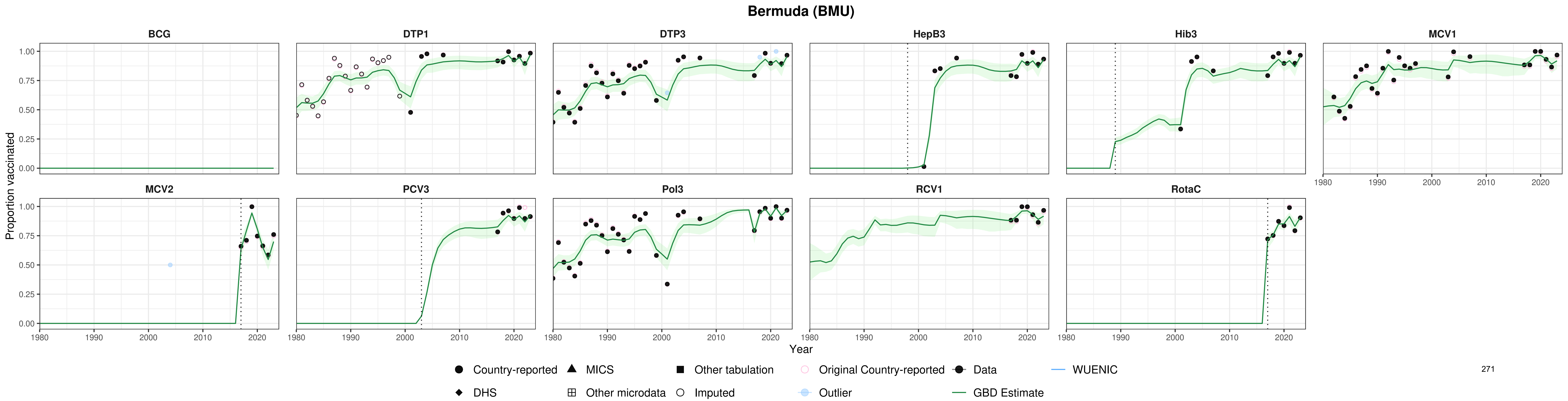
Bermuda (BMU)
BCG
DTP1
DTP3
HepB3
Hib3
MCV1
MCV2
PCV3
Pol3
RCV1
RotaC
Proportion vaccinated
0.00
0.25
0.50
0.75
1.00
1980
1990
2000
2010
2020
Year
Country-reported
MICS
Other tabulation
Original Country-reported
Data
WUENIC
DHS
Other microdata
Imputed
Outlier
GBD Estimate

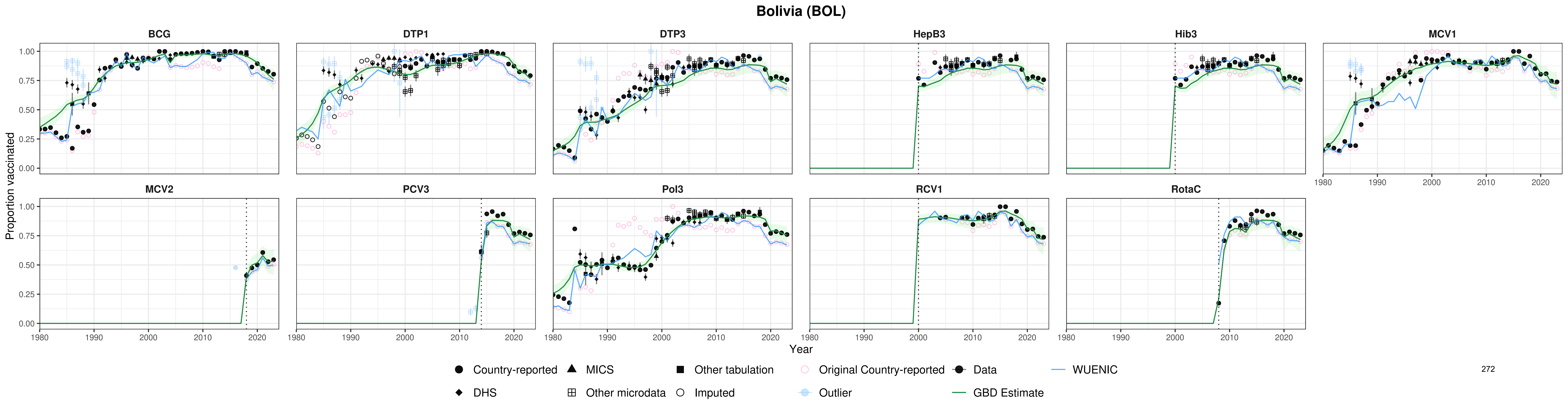
Bolivia (BOL)
BCG
DTP1
DTP3
HepB3
Hib3
MCV1
MCV2
PCV3
Pol3
RCV1
RotaC
Proportion vaccinated
Year
Country-reported
MICS
Other tabulation
Original Country-reported
Data
WUENIC
DHS
Other microdata
Imputed
Outlier
GBD Estimate

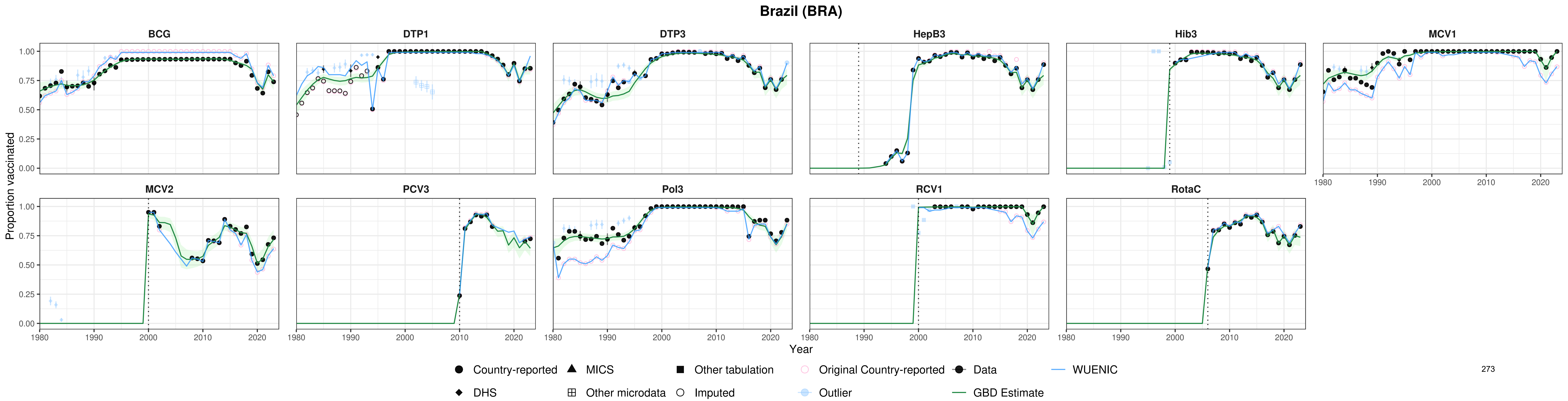

Brazil (BRA)
BCG
DTP1
DTP3
HepB3
Hib3
MCV1
MCV2
PCV3
Pol3
RCV1
RotaC
Proportion vaccinated
Year
Country-reported
MICS
Other tabulation
Original Country-reported
Data
WUENIC
DHS
Other microdata
Imputed
Outlier
GBD Estimate

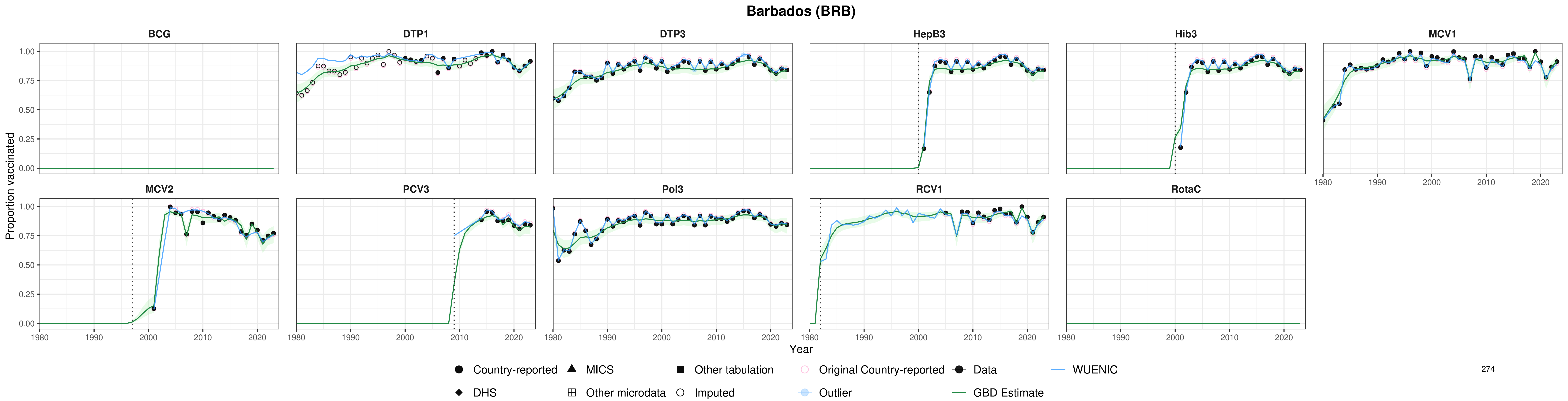

Barbados (BRB)
BCG
DTP1
DTP3
HepB3
Hib3
MCV1
MCV2
PCV3
Pol3
RCV1
RotaC
Proportion vaccinated
Year
Country-reported
MICS
Other tabulation
Original Country-reported
Data
WUENIC
DHS
Other microdata
Imputed
Outlier
GBD Estimate

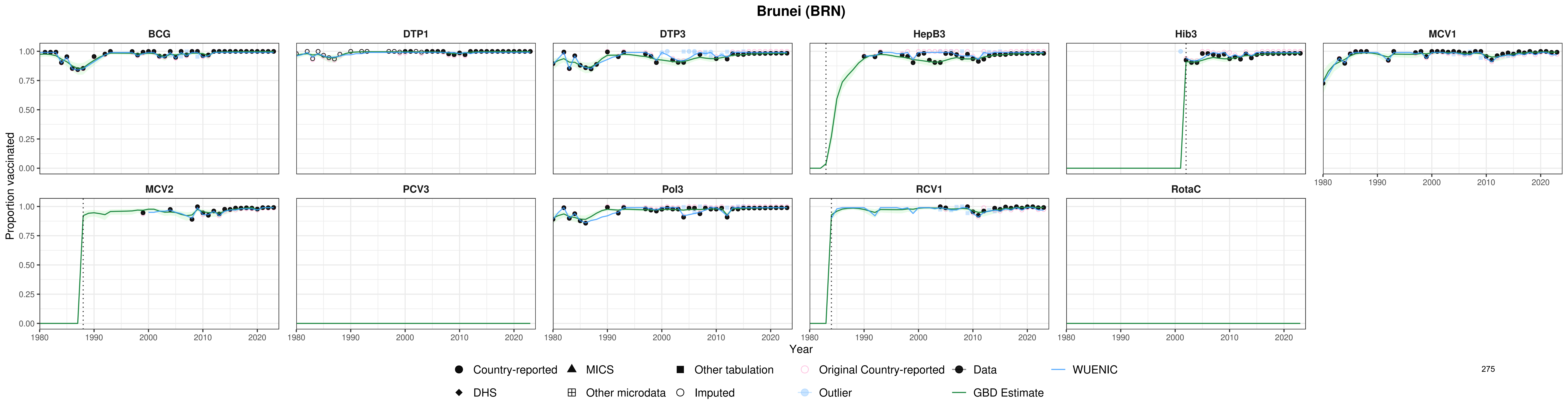

Brunei (BRN)
BCG
DTP1
DTP3
HepB3
Hib3
MCV1
MCV2
PCV3
Pol3
RCV1
RotaC
Proportion vaccinated
Year
1.00
0.75
0.50
0.25
0.00
1980
1990
2000
2010
2020
Country-reported
MICS
Other tabulation
Original Country-reported
Data
WUENIC
DHS
Other microdata
Imputed
Outlier
GBD Estimate

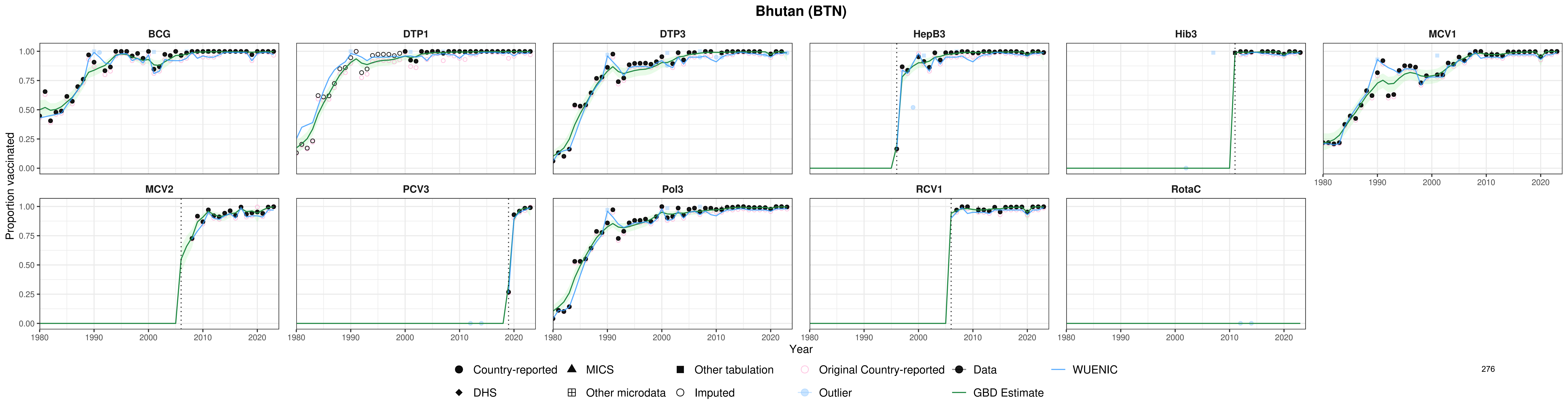
Bhutan (BTN)
BCG
DTP1
DTP3
HepB3
Hib3
MCV1
MCV2
PCV3
Pol3
RCV1
RotaC
Proportion vaccinated
Year
Country-reported
MICS
Other tabulation
Original Country-reported
Data
WUENIC
DHS
Other microdata
Imputed
Outlier
GBD Estimate

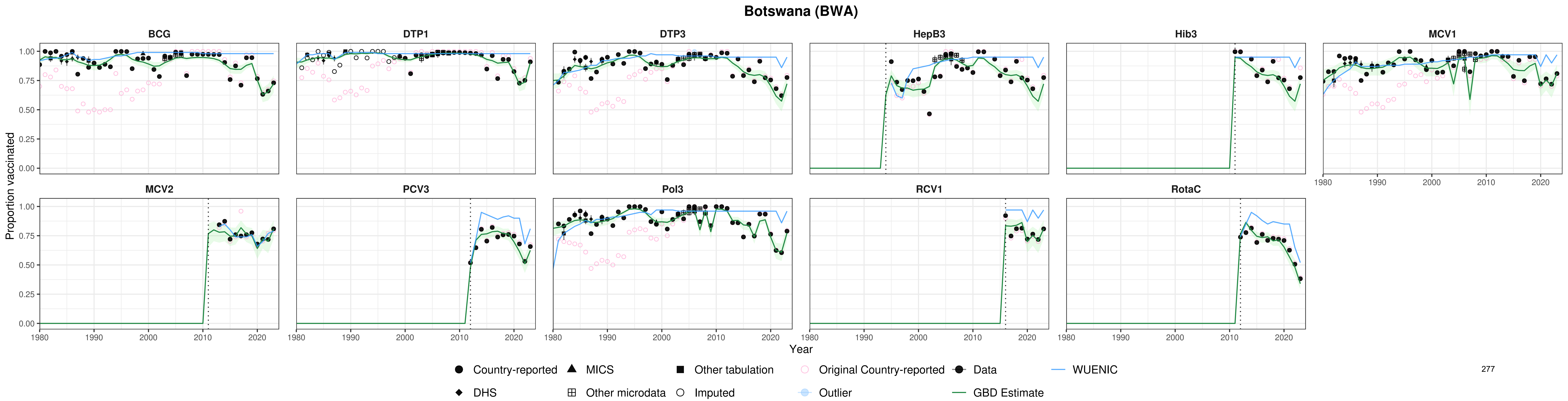

Botswana (BWA)
BCG
DTP1
DTP3
HepB3
Hib3
MCV1
MCV2
PCV3
Pol3
RCV1
RotaC
Proportion vaccinated
Year
0.00
0.25
0.50
0.75
1.00
1980
1990
2000
2010
2020
Country-reported
MICS
Other tabulation
Original Country-reported
Data
WUENIC
DHS
Other microdata
Imputed
Outlier
GBD Estimate

Central African Republic (CAF)

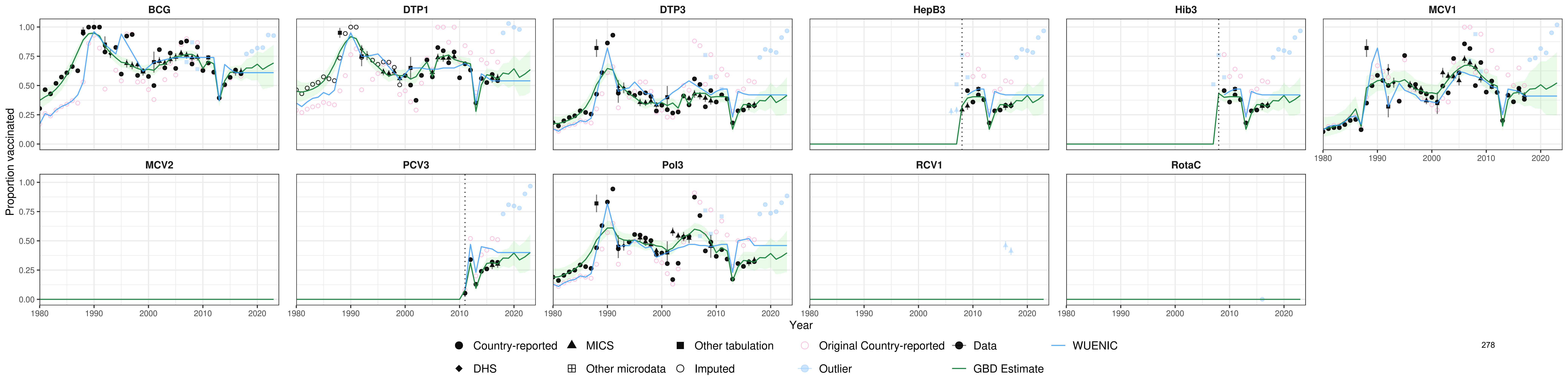

Canada (CAN)

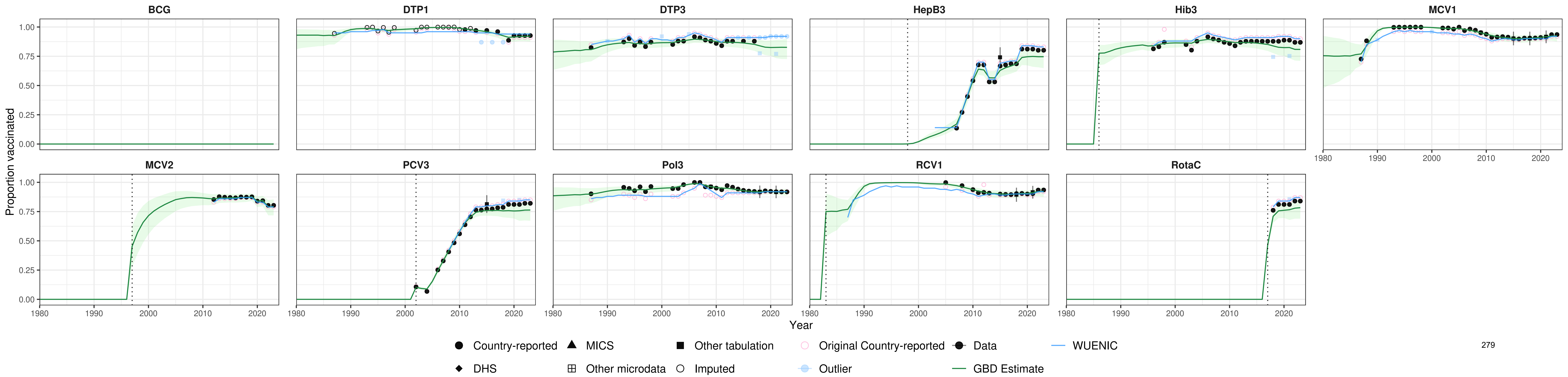
BCG
DTP1
DTP3
HepB3
Hib3
MCV1
MCV2
PCV3
Pol3
RCV1
RotaC
Proportion vaccinated
Year
0.00
0.25
0.50
0.75
1.00
1980
1990
2000
2010
2020
Country-reported
MICS
Other tabulation
Original Country-reported
Data
WUENIC
DHS
Other microdata
Imputed
Outlier
GBD Estimate

Switzerland (CHE)

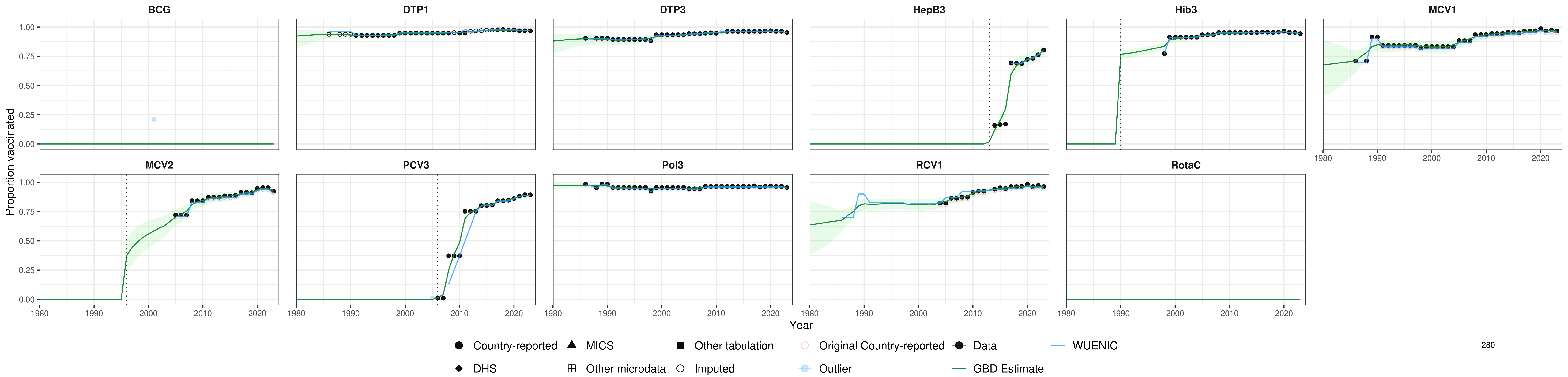

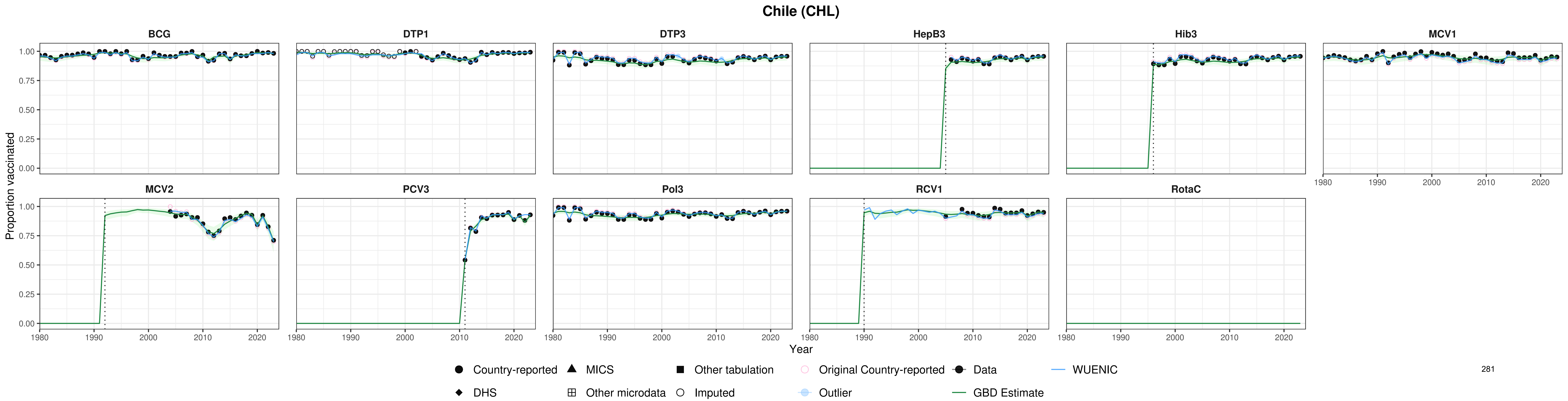

Chile (CHL)
BCG
DTP1
DTP3
HepB3
Hib3
MCV1
MCV2
PCV3
Pol3
RCV1
RotaC
Proportion vaccinated
Year
Country-reported
MICS
Other tabulation
Original Country-reported
Data
WUENIC
DHS
Other microdata
Imputed
Outlier
GBD Estimate

China (CHN)

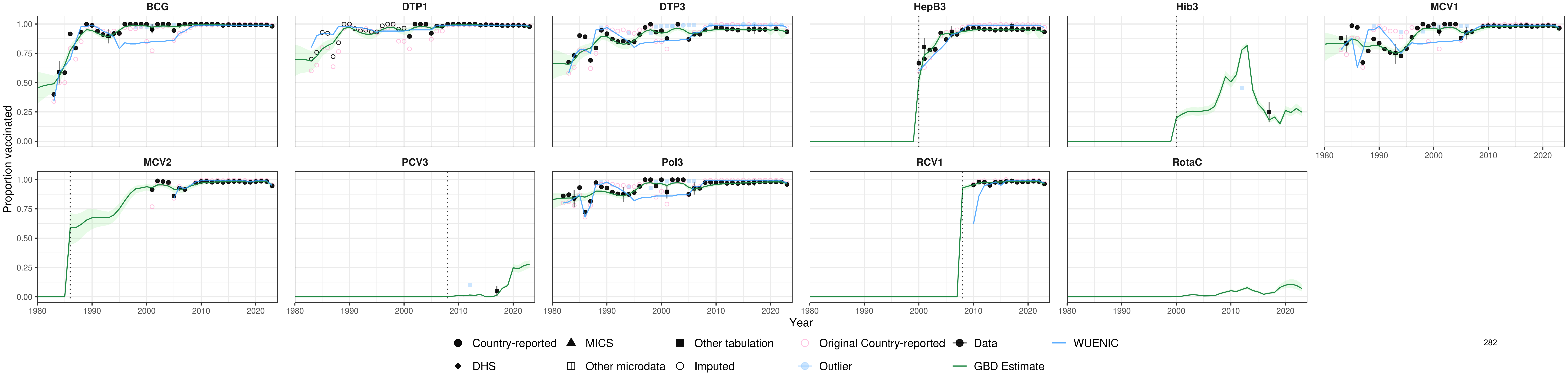

BCG
DTP1
DTP3
HepB3
Hib3
MCV1
MCV2
PCV3
Pol3
RCV1
RotaC
Proportion vaccinated
Year
1.00
0.75
0.50
0.25
0.00
1980
1990
2000
2010
2020
Country-reported
MICS
Other tabulation
Original Country-reported
Data
WUENIC
DHS
Other microdata
Imputed
Outlier
GBD Estimate

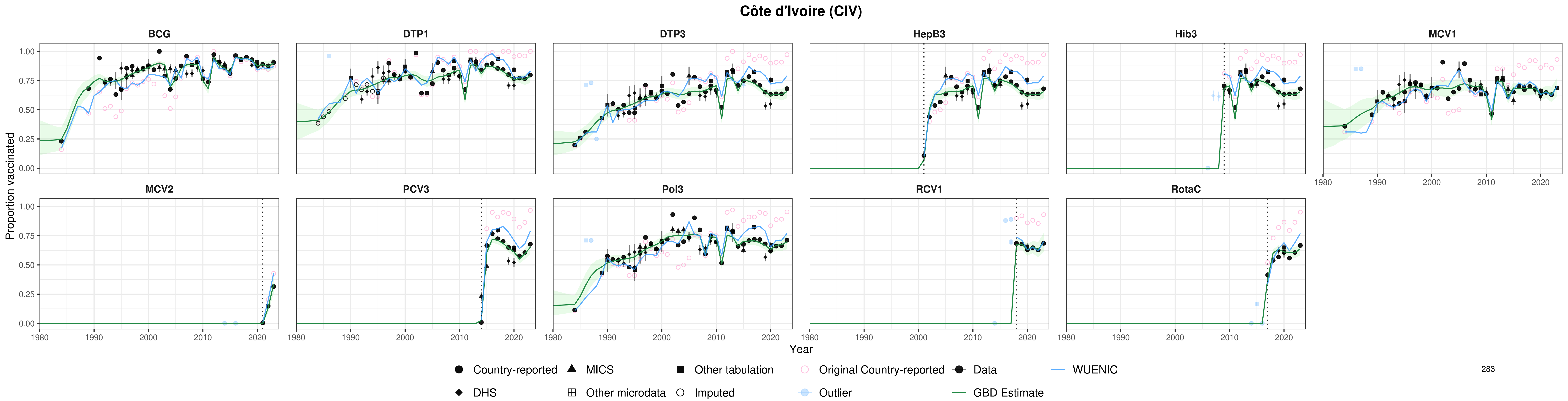
Côte d'Ivoire (CIV)
BCG
DTP1
DTP3
HepB3
Hib3
MCV1
MCV2
PCV3
Pol3
RCV1
RotaC
Proportion vaccinated
Year
Country-reported
MICS
Other tabulation
Original Country-reported
Data
WUENIC
DHS
Other microdata
Imputed
Outlier
GBD Estimate

Cameroon (CMR)

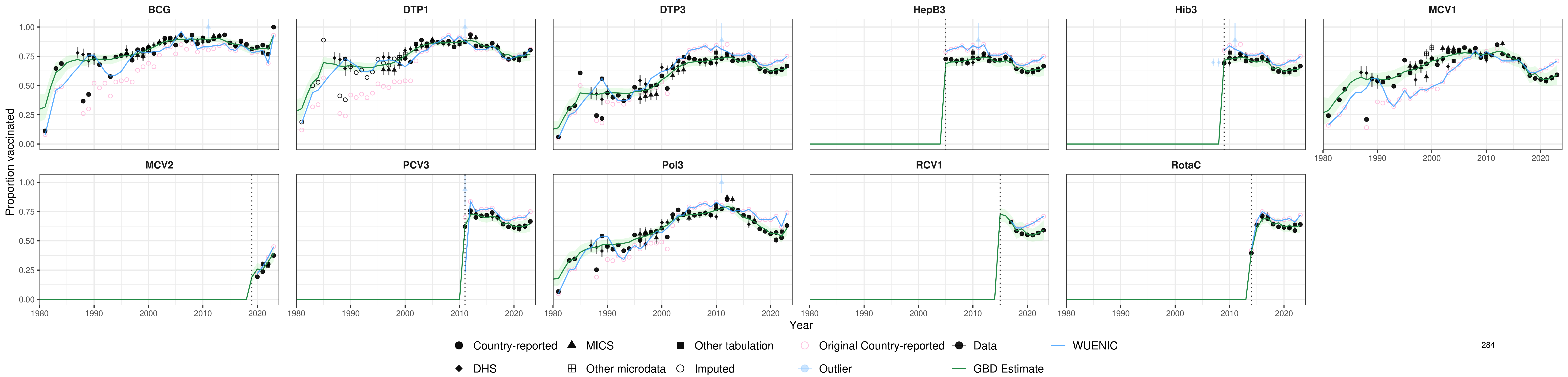

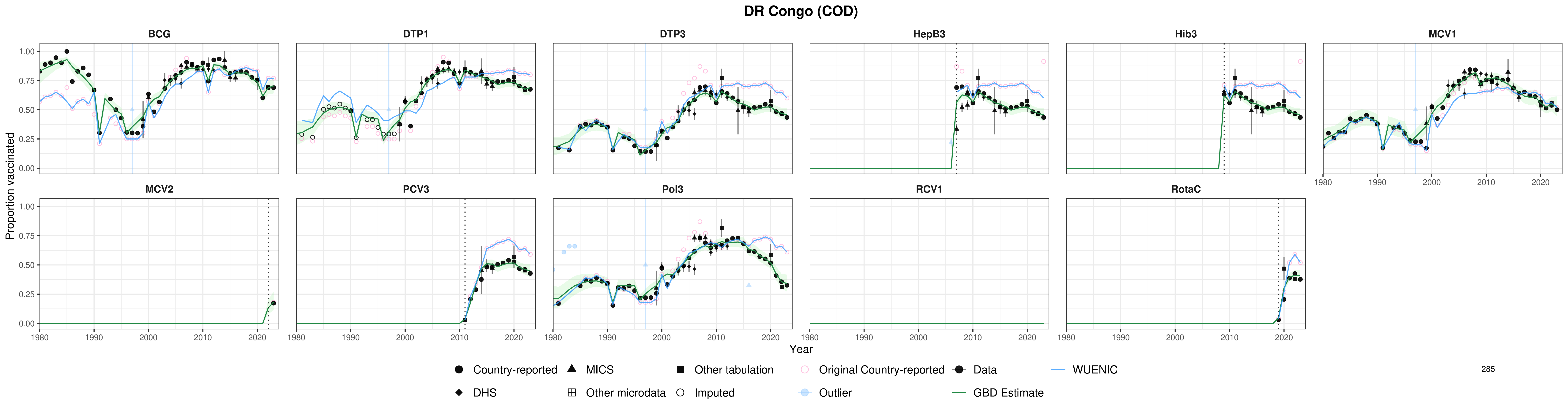

DR Congo (COD)
BCG
DTP1
DTP3
HepB3
Hib3
MCV1
MCV2
PCV3
Pol3
RCV1
RotaC
Proportion vaccinated
Year
1.00
0.75
0.50
0.25
0.00
1980
1990
2000
2010
2020
Country-reported
MICS
Other tabulation
Original Country-reported
Data
WUENIC
DHS
Other microdata
Imputed
Outlier
GBD Estimate

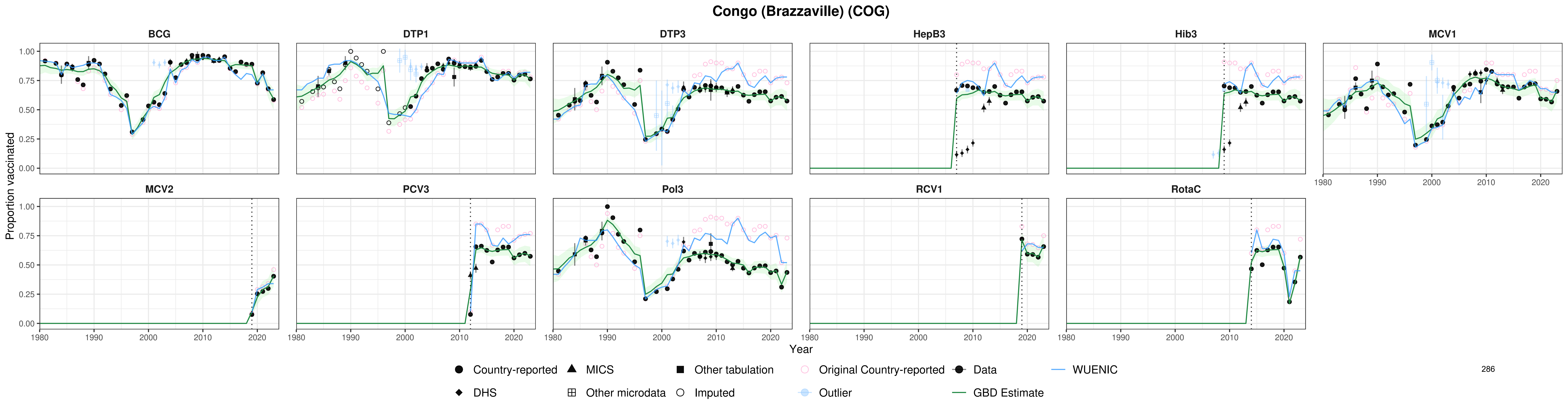
Congo (Brazzaville) (COG)
BCG
DTP1
DTP3
HepB3
Hib3
MCV1
MCV2
PCV3
Pol3
RCV1
RotaC
Proportion vaccinated
Year
Country-reported
MICS
Other tabulation
Original Country-reported
Data
WUENIC
DHS
Other microdata
Imputed
Outlier
GBD Estimate

Cook Islands (COK)

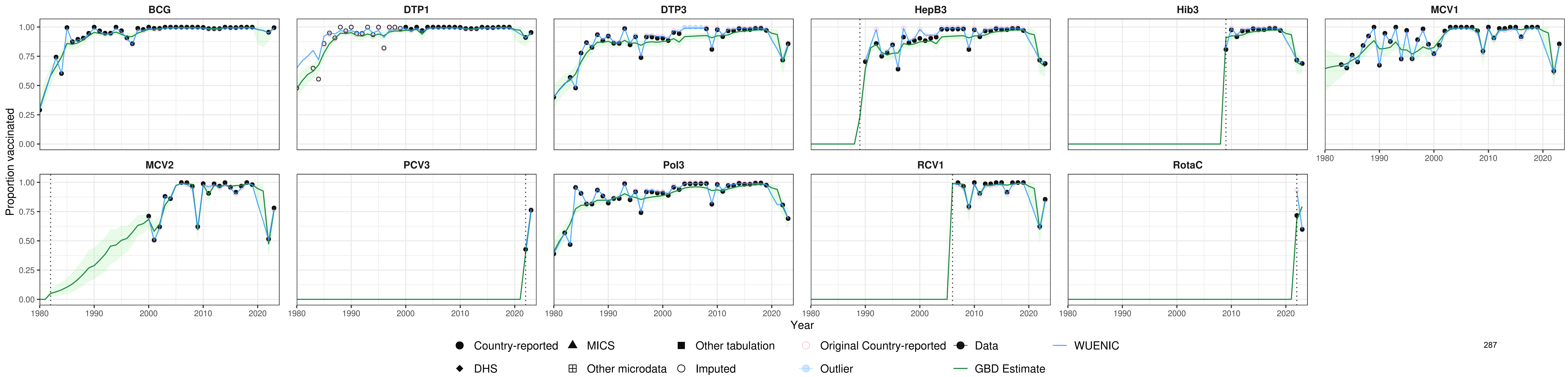

Colombia (COL)

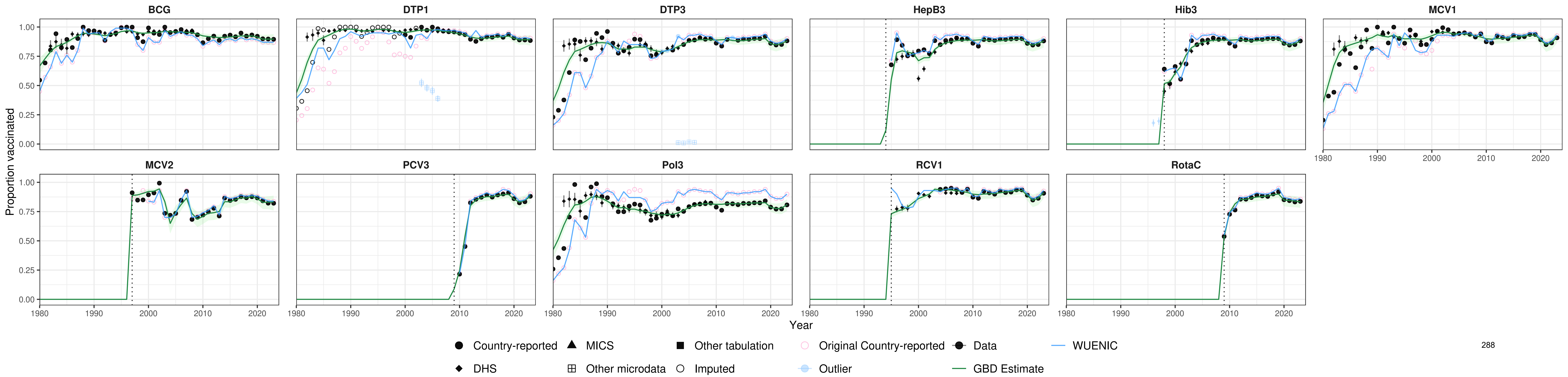

Comoros (COM)

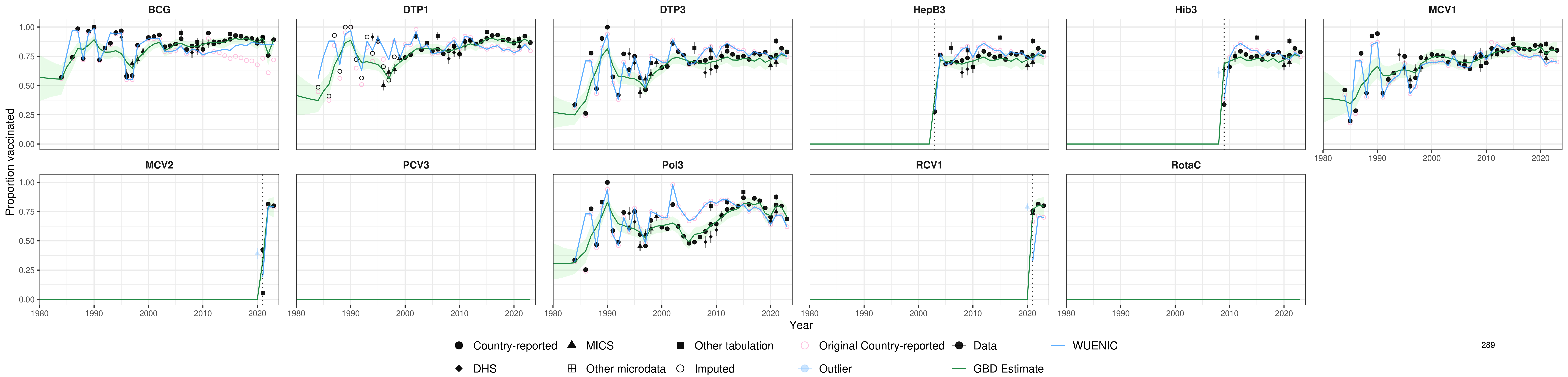
BCG
DTP1
DTP3
HepB3
Hib3
MCV1
MCV2
PCV3
Pol3
RCV1
RotaC
Proportion vaccinated
Year
Country-reported
MICS
Other tabulation
Original Country-reported
Data
WUENIC
DHS
Other microdata
Imputed
Outlier
GBD Estimate

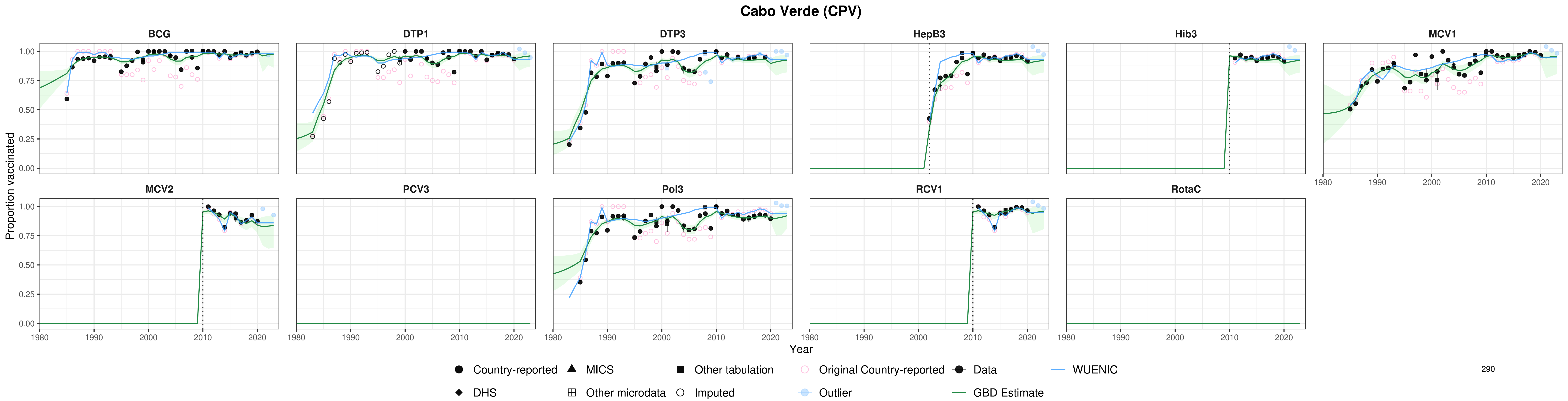

Cabo Verde (CPV)
BCG
DTP1
DTP3
HepB3
Hib3
MCV1
MCV2
PCV3
Pol3
RCV1
RotaC
Proportion vaccinated
0.00
0.25
0.50
0.75
1.00
1980
1990
2000
2010
2020
Year
Country-reported
MICS
Other tabulation
Original Country-reported
Data
WUENIC
DHS
Other microdata
Imputed
Outlier
GBD Estimate

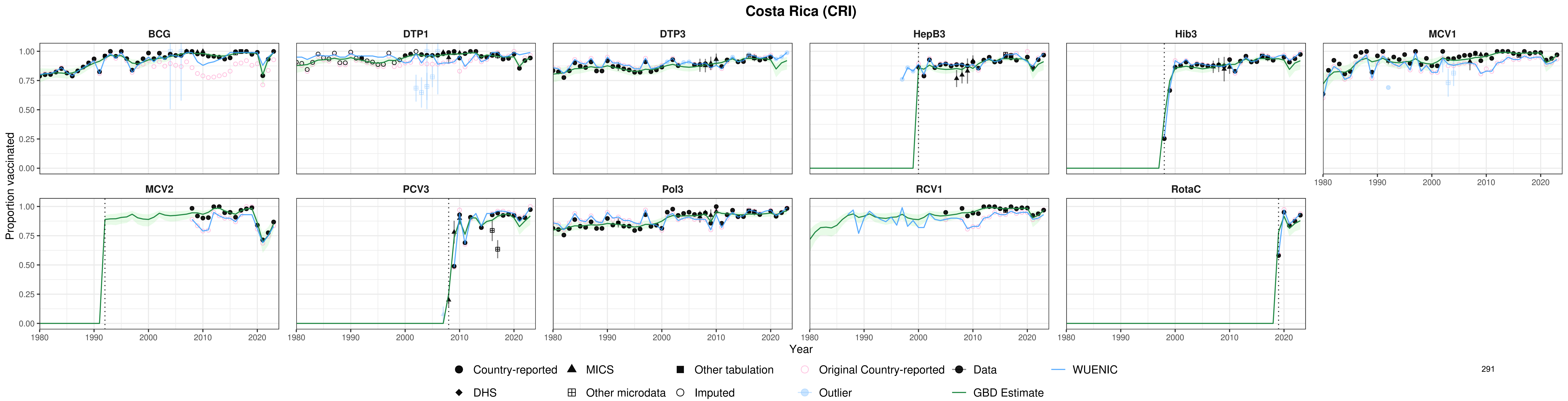

Costa Rica (CRI)
BCG
DTP1
DTP3
HepB3
Hib3
MCV1
MCV2
PCV3
Pol3
RCV1
RotaC
Proportion vaccinated
Year
Country-reported
MICS
Other tabulation
Original Country-reported
Data
WUENIC
DHS
Other microdata
Imputed
Outlier
GBD Estimate

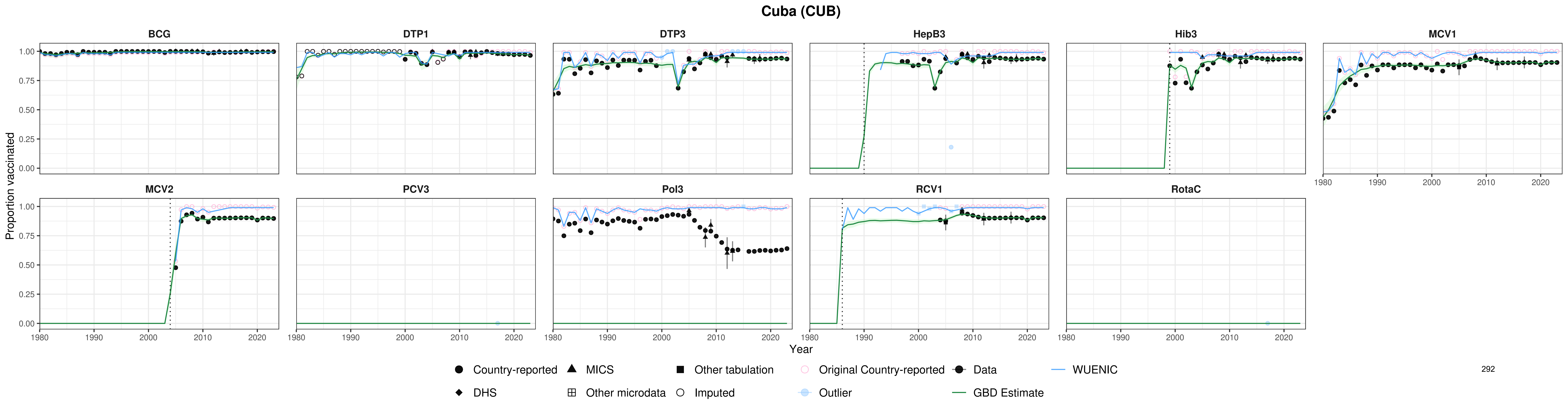

Cuba (CUB)
BCG
DTP1
DTP3
HepB3
Hib3
MCV1
MCV2
PCV3
Pol3
RCV1
RotaC
Proportion vaccinated
Year
0.00
0.25
0.50
0.75
1.00
1980
1990
2000
2010
2020
Country-reported
MICS
Other tabulation
Original Country-reported
Data
WUENIC
DHS
Other microdata
Imputed
Outlier
GBD Estimate

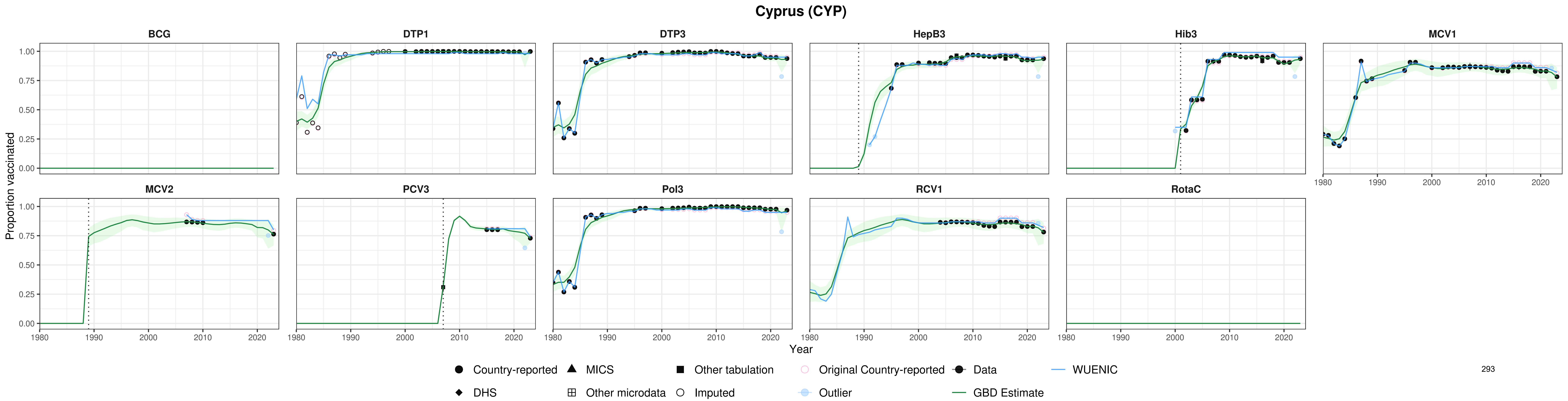
Cyprus (CYP)
BCG
DTP1
DTP3
HepB3
Hib3
MCV1
MCV2
PCV3
Pol3
RCV1
RotaC
Proportion vaccinated
Year
Country-reported
MICS
Other tabulation
Original Country-reported
Data
WUENIC
DHS
Other microdata
Imputed
Outlier
GBD Estimate

Czechia (CZE)

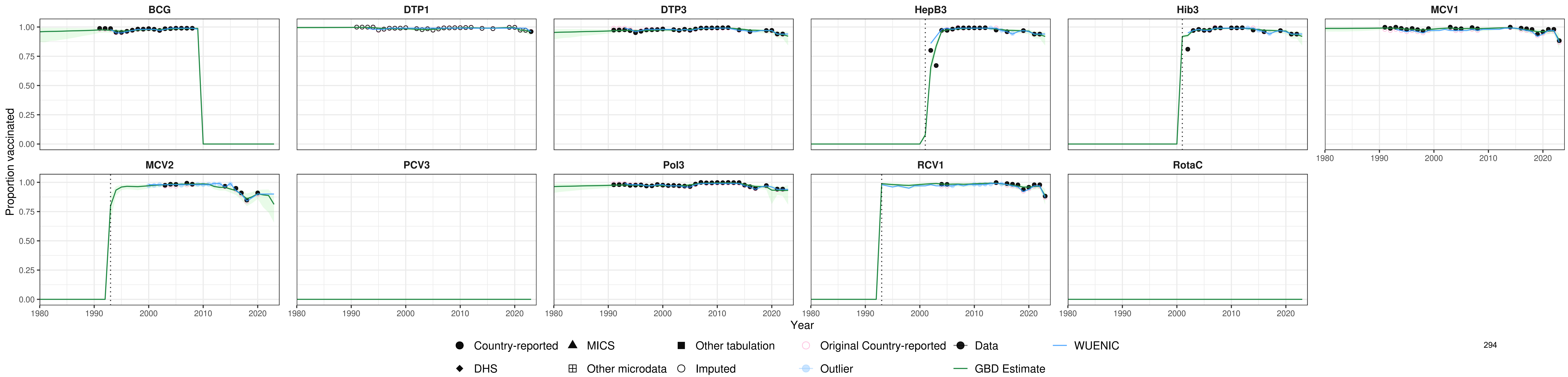

BCG
DTP1
DTP3
HepB3
Hib3
MCV1
MCV2
PCV3
Pol3
RCV1
RotaC
Proportion vaccinated
1.00
0.75
0.50
0.25
0.00
1980
1990
2000
2010
2020
Year
Country-reported
MICS
Other tabulation
Original Country-reported
Data
WUENIC
DHS
Other microdata
Imputed
Outlier
GBD Estimate

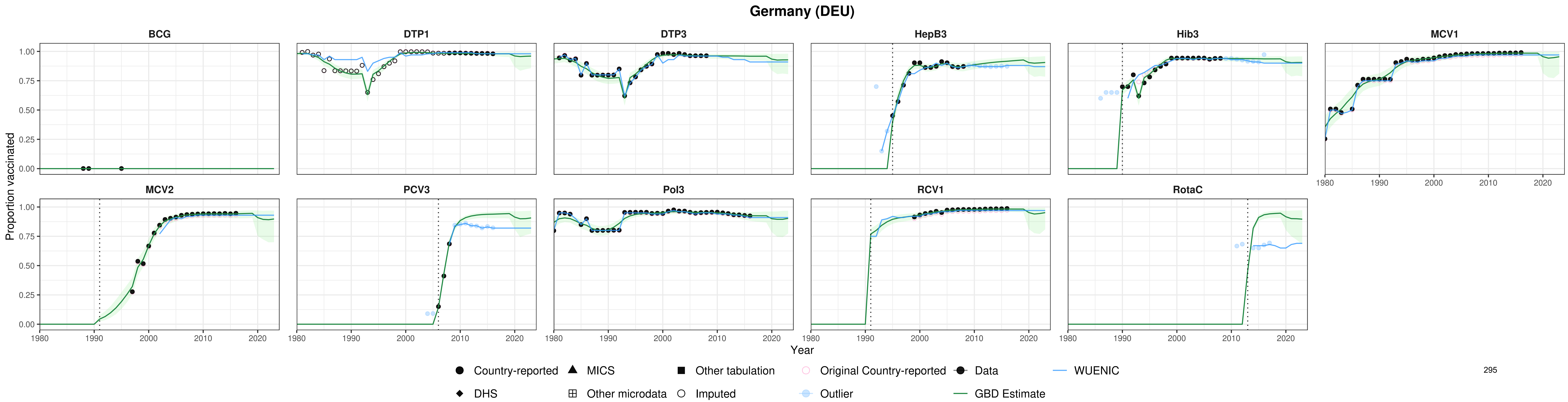
Germany (DEU)
BCG
DTP1
DTP3
HepB3
Hib3
MCV1
MCV2
PCV3
Pol3
RCV1
RotaC
Proportion vaccinated
Year
Country-reported
MICS
Other tabulation
Original Country-reported
Data
WUENIC
DHS
Other microdata
Imputed
Outlier
GBD Estimate

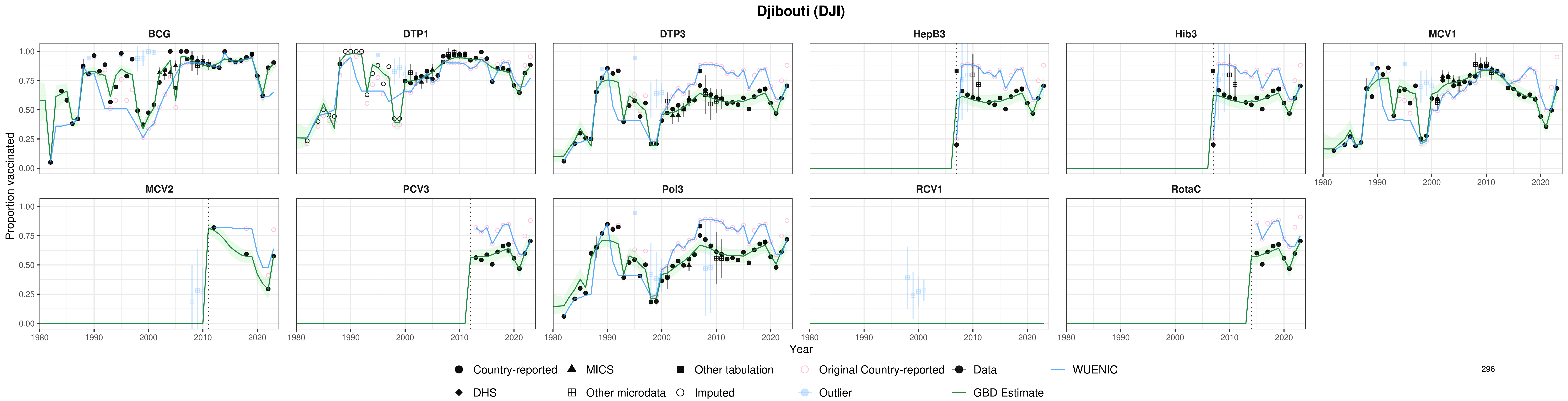

Djibouti (DJI)
BCG
DTP1
DTP3
HepB3
Hib3
MCV1
MCV2
PCV3
Pol3
RCV1
RotaC
Proportion vaccinated
0.00
0.25
0.50
0.75
1.00
1980
1990
2000
2010
2020
Year
Country-reported
DHS
MICS
Other microdata
Other tabulation
Imputed
Original Country-reported
Outlier
Data
GBD Estimate
WUENIC

Dominica (DMA)

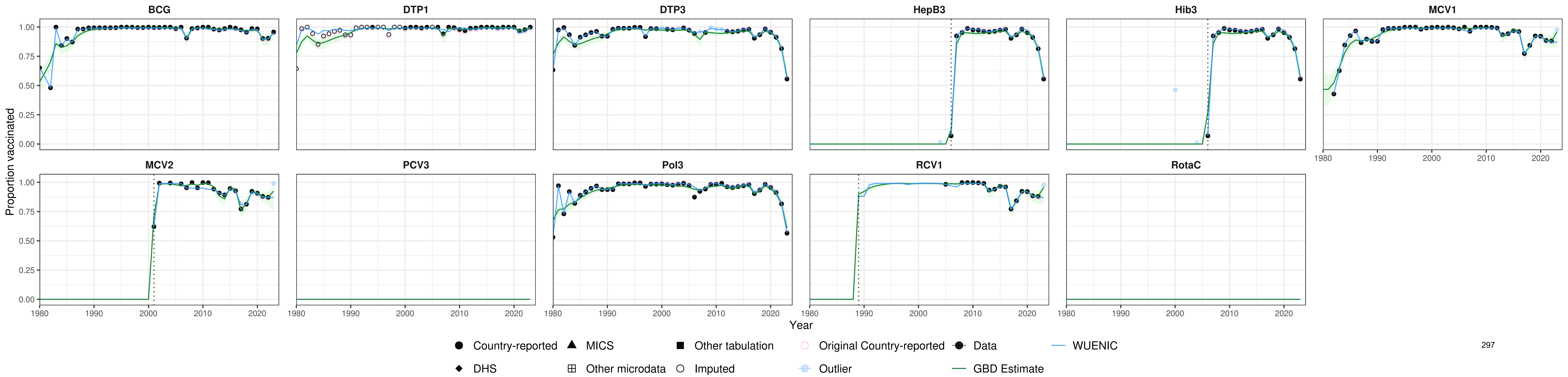

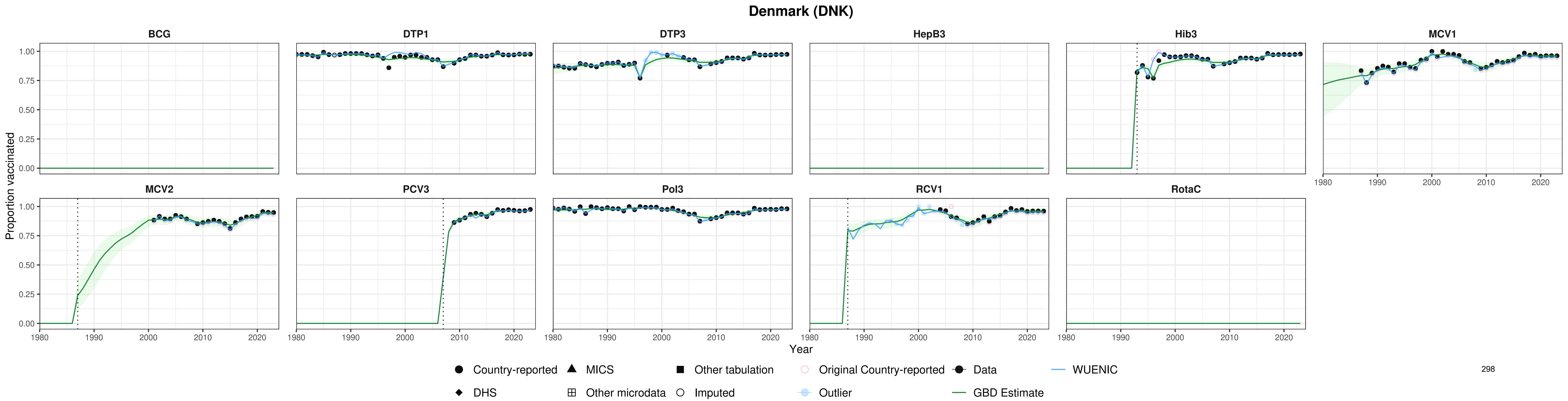
Denmark (DNK)
BCG
DTP1
DTP3
HepB3
Hib3
MCV1
MCV2
PCV3
Pol3
RCV1
RotaC
Proportion vaccinated
Year
Country-reported
MICS
Other tabulation
Original Country-reported
Data
WUENIC
DHS
Other microdata
Imputed
Outlier
GBD Estimate

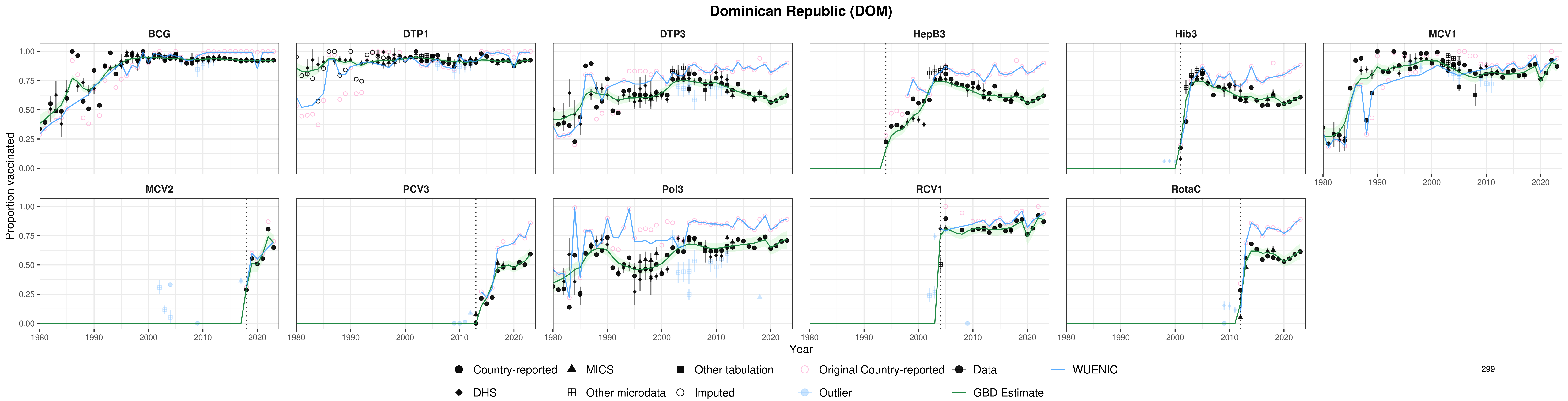
Dominican Republic (DOM)
BCG
DTP1
DTP3
HepB3
Hib3
MCV1
MCV2
PCV3
Pol3
RCV1
RotaC
Proportion vaccinated
Year
Country-reported
MICS
Other tabulation
Original Country-reported
Data
WUENIC
DHS
Other microdata
Imputed
Outlier
GBD Estimate

Algeria (DZA)

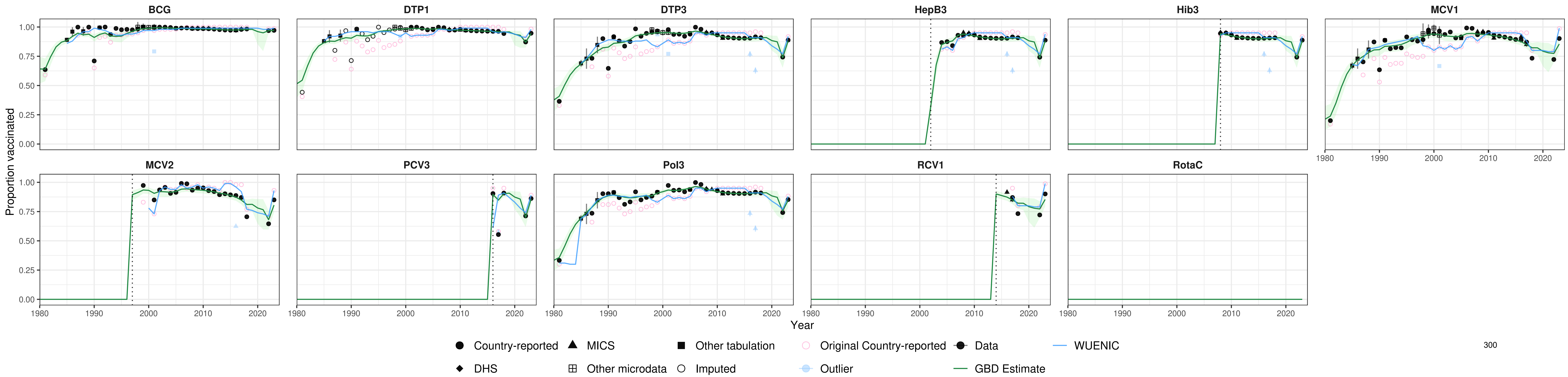

BCG
DTP1
DTP3
HepB3
Hib3
MCV1
MCV2
PCV3
Pol3
RCV1
RotaC
Proportion vaccinated
Year
Country-reported
MICS
Other tabulation
Original Country-reported
Data
WUENIC
DHS
Other microdata
Imputed
Outlier
GBD Estimate

Ecuador (ECU)

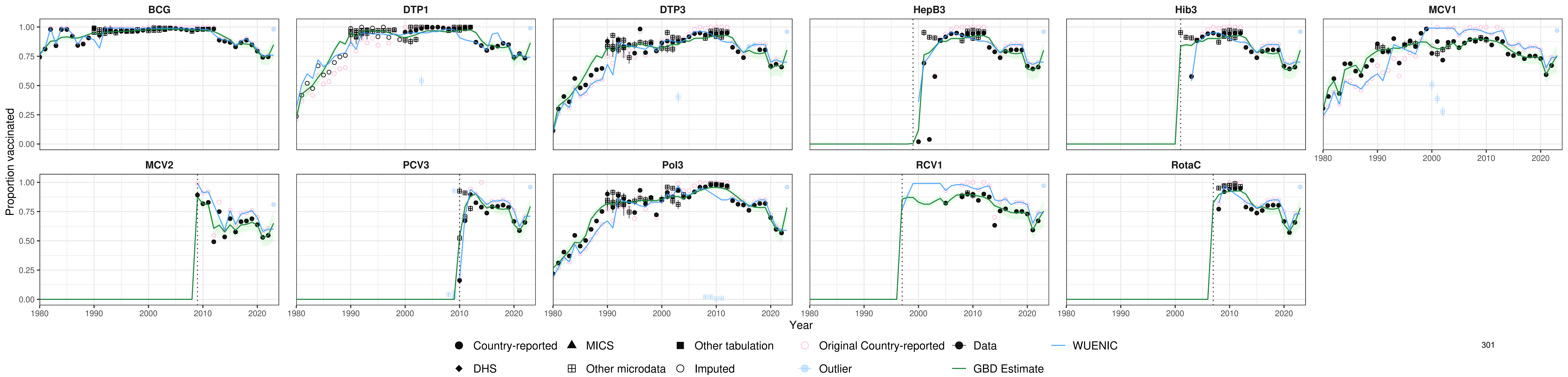

BCG
DTP1
DTP3
HepB3
Hib3
MCV1
MCV2
PCV3
Pol3
RCV1
RotaC
Proportion vaccinated
Year
Country-reported
MICS
Other tabulation
Original Country-reported
Data
WUENIC
DHS
Other microdata
Imputed
Outlier
GBD Estimate

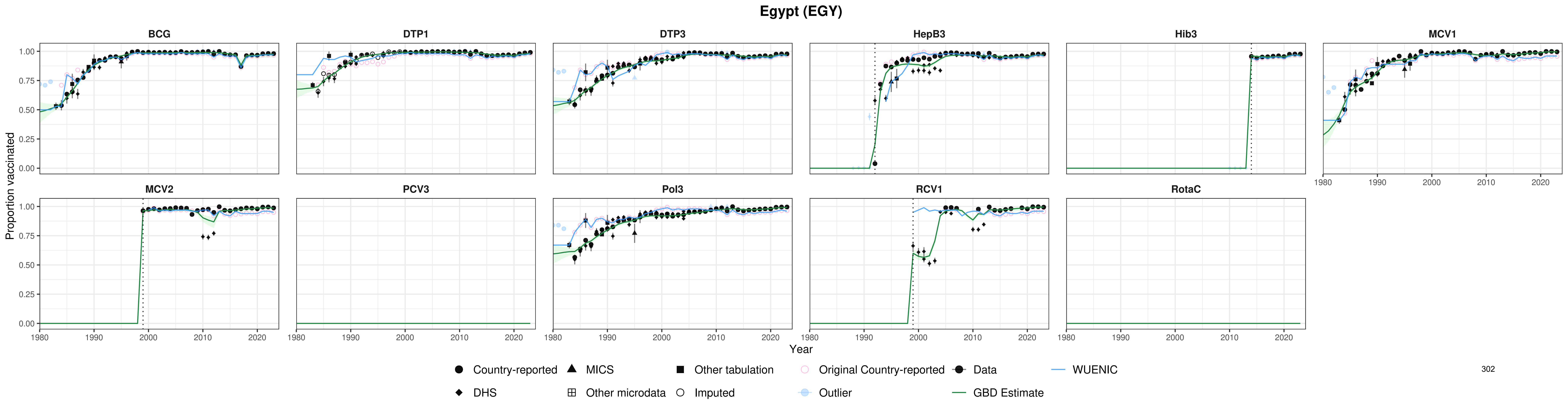
Egypt (EGY)
BCG
DTP1
DTP3
HepB3
Hib3
MCV1
MCV2
PCV3
Pol3
RCV1
RotaC
Proportion vaccinated
Year
Country-reported
MICS
Other tabulation
Original Country-reported
Data
WUENIC
DHS
Other microdata
Imputed
Outlier
GBD Estimate

Eritrea (ERI)

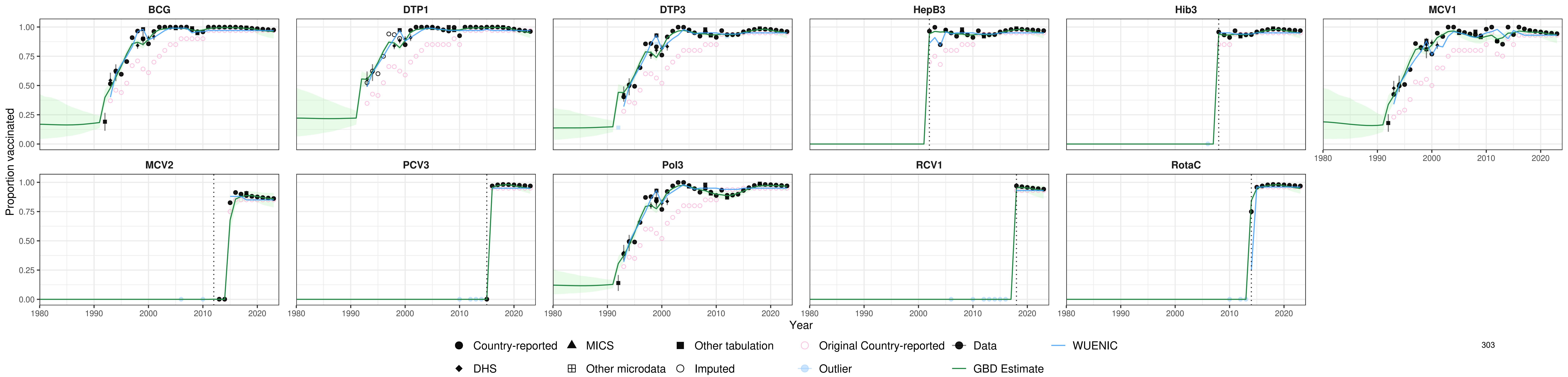

BCG
DTP1
DTP3
HepB3
Hib3
MCV1
MCV2
PCV3
Pol3
RCV1
RotaC
Proportion vaccinated
Year
Country-reported
MICS
Other tabulation
Original Country-reported
Data
WUENIC
DHS
Other microdata
Imputed
Outlier
GBD Estimate

Spain (ESP)

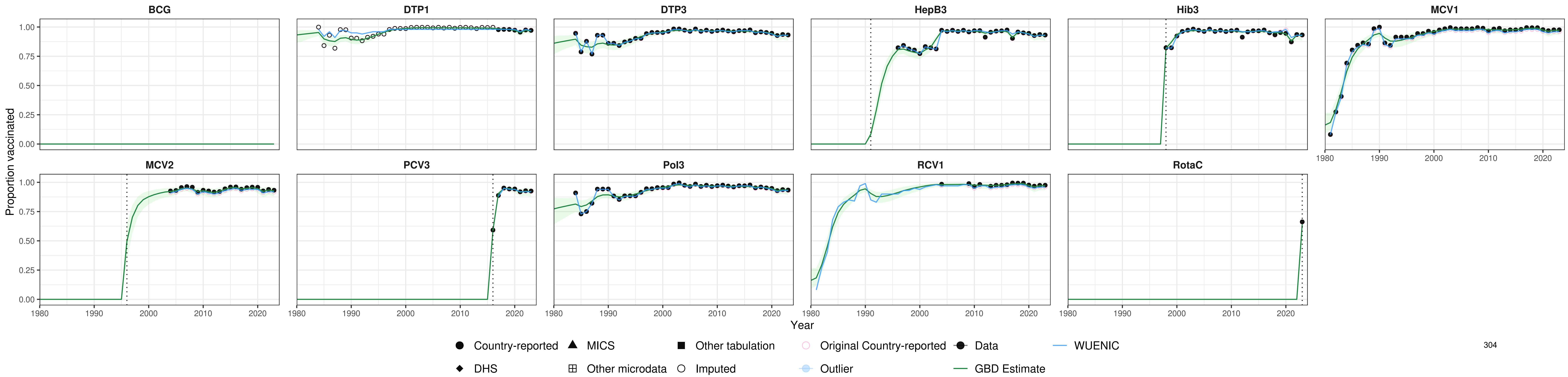

BCG
DTP1
DTP3
HepB3
Hib3
MCV1
MCV2
PCV3
Pol3
RCV1
RotaC
Proportion vaccinated
0.00
0.25
0.50
0.75
1.00
1980
1990
2000
2010
2020
Year
Country-reported
MICS
Other tabulation
Original Country-reported
Data
WUENIC
DHS
Other microdata
Imputed
Outlier
GBD Estimate

Estonia (EST)

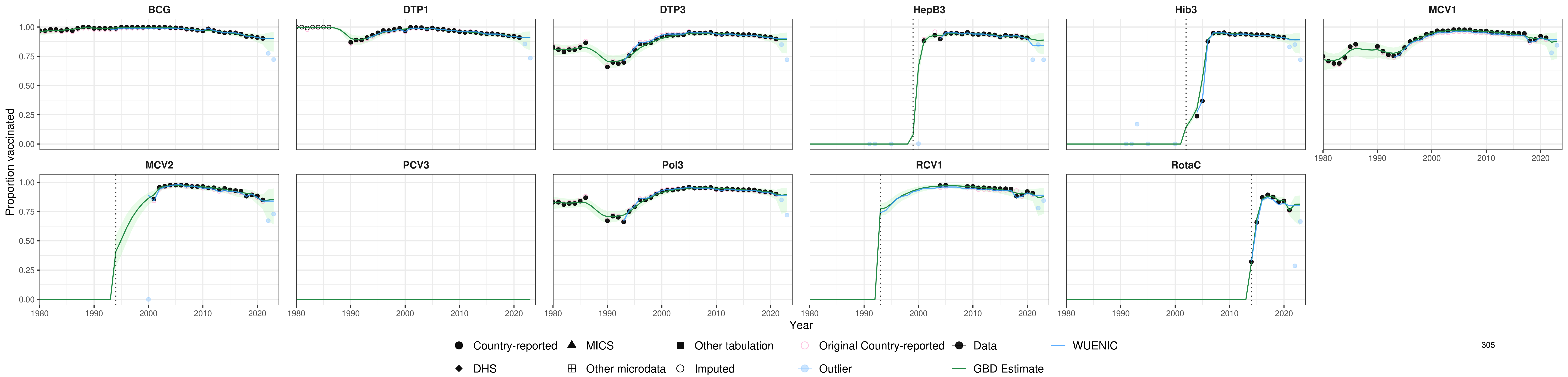

BCG
DTP1
DTP3
HepB3
Hib3
MCV1
MCV2
PCV3
Pol3
RCV1
RotaC
Proportion vaccinated
1.00
0.75
0.50
0.25
0.00
1980
1990
2000
2010
2020
Year
Country-reported
MICS
Other tabulation
Original Country-reported
Data
WUENIC
DHS
Other microdata
Imputed
Outlier
GBD Estimate

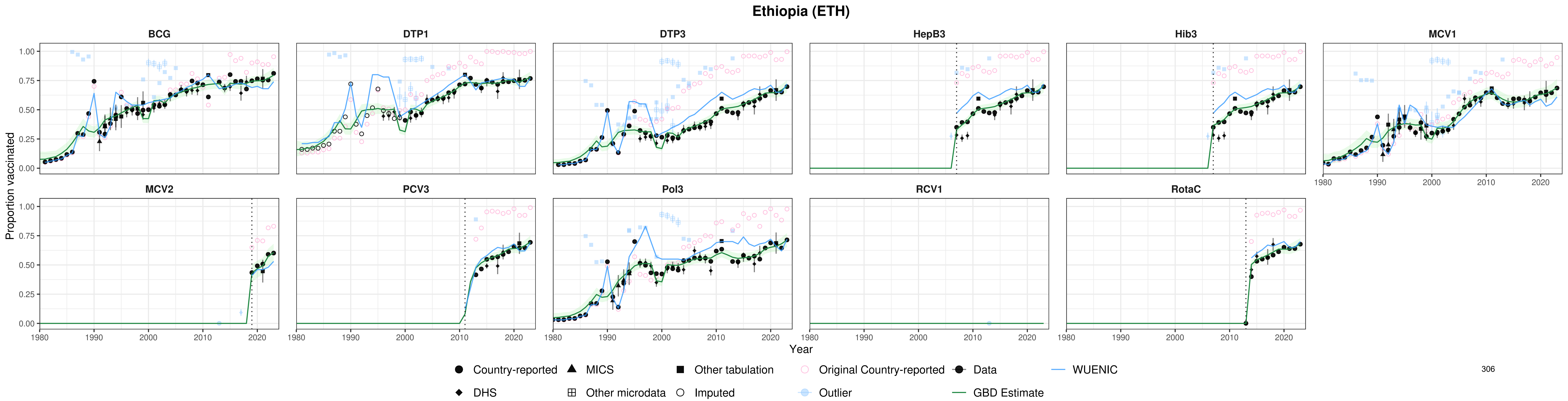

Ethiopia (ETH)
BCG
DTP1
DTP3
HepB3
Hib3
MCV1
MCV2
PCV3
Pol3
RCV1
RotaC
Proportion vaccinated
Year
0.00
0.25
0.50
0.75
1.00
1980
1990
2000
2010
2020
Country-reported
MICS
Other tabulation
Original Country-reported
Data
WUENIC
DHS
Other microdata
Imputed
Outlier
GBD Estimate

Finland (FIN)

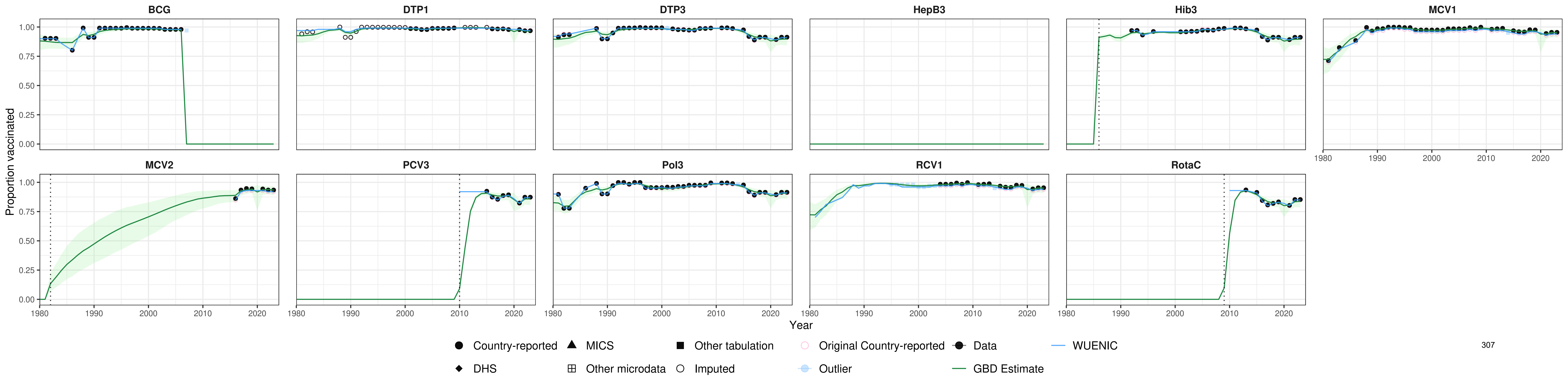

BCG
DTP1
DTP3
HepB3
Hib3
MCV1
MCV2
PCV3
Pol3
RCV1
RotaC
Proportion vaccinated
Year
Country-reported
MICS
Other tabulation
Original Country-reported
Data
WUENIC
DHS
Other microdata
Imputed
Outlier
GBD Estimate

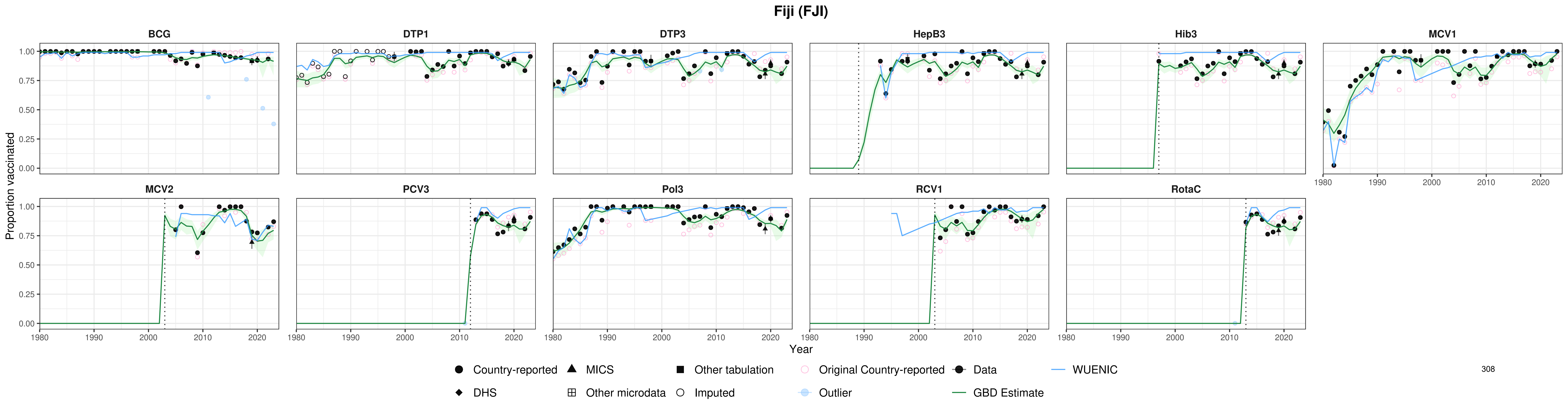

Fiji (FJI)
BCG
DTP1
DTP3
HepB3
Hib3
MCV1
MCV2
PCV3
Pol3
RCV1
RotaC
Proportion vaccinated
Year
Country-reported
MICS
Other tabulation
Original Country-reported
Data
WUENIC
DHS
Other microdata
Imputed
Outlier
GBD Estimate

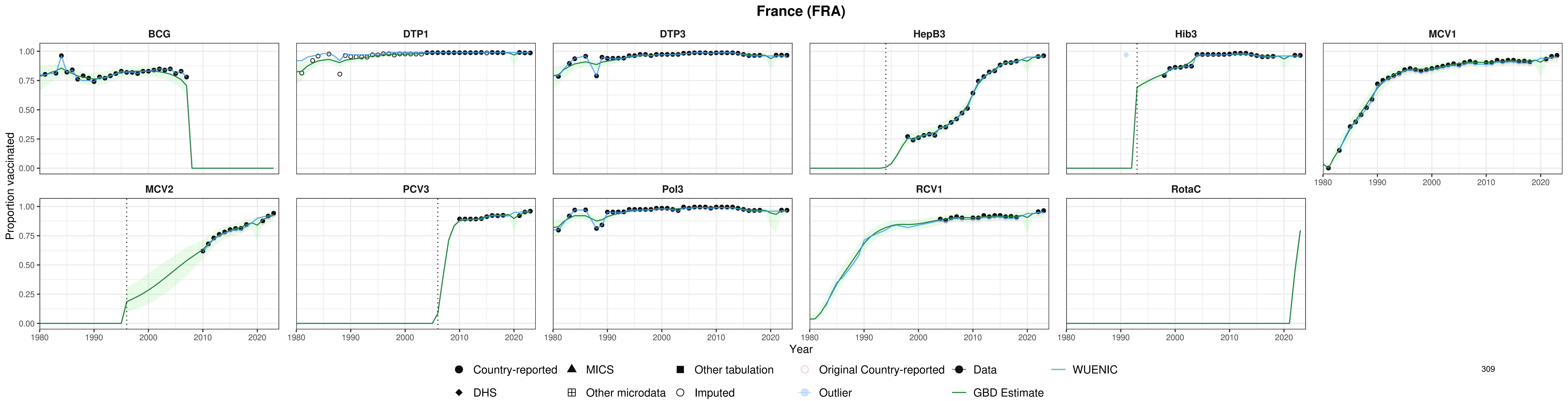

France (FRA)
BCG
DTP1
DTP3
HepB3
Hib3
MCV1
MCV2
PCV3
Pol3
RCV1
RotaC
Proportion vaccinated
Year
Country-reported
MICS
Other tabulation
Original Country-reported
Data
WUENIC
DHS
Other microdata
Imputed
Outlier
GBD Estimate

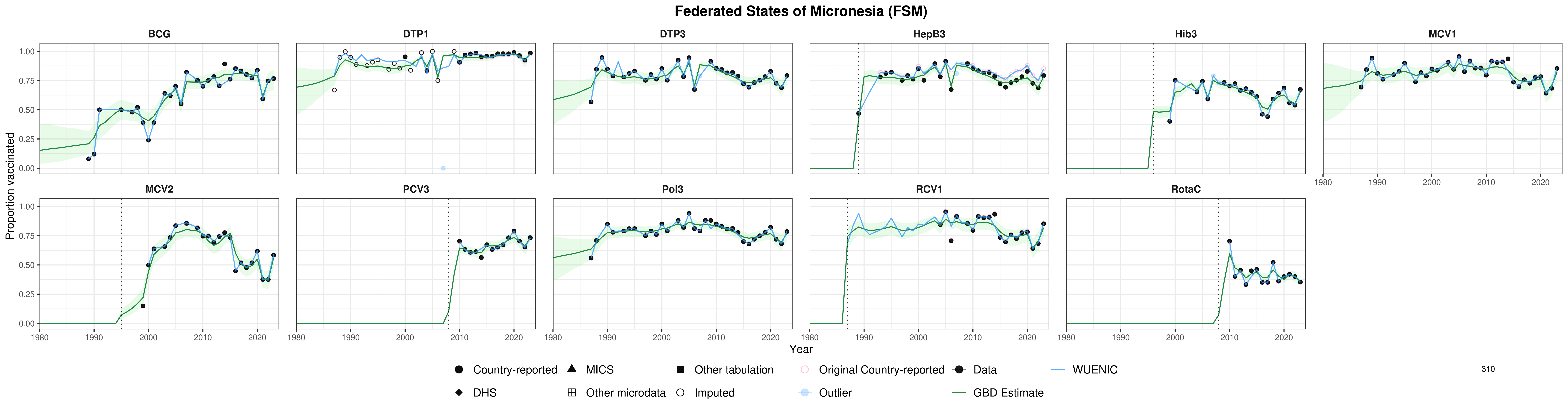

Federated States of Micronesia (FSM)
BCG
DTP1
DTP3
HepB3
Hib3
MCV1
MCV2
PCV3
Pol3
RCV1
RotaC
Proportion vaccinated
Year
Country-reported
MICS
Other tabulation
Original Country-reported
Data
WUENIC
DHS
Other microdata
Imputed
Outlier
GBD Estimate

Gabon (GAB)

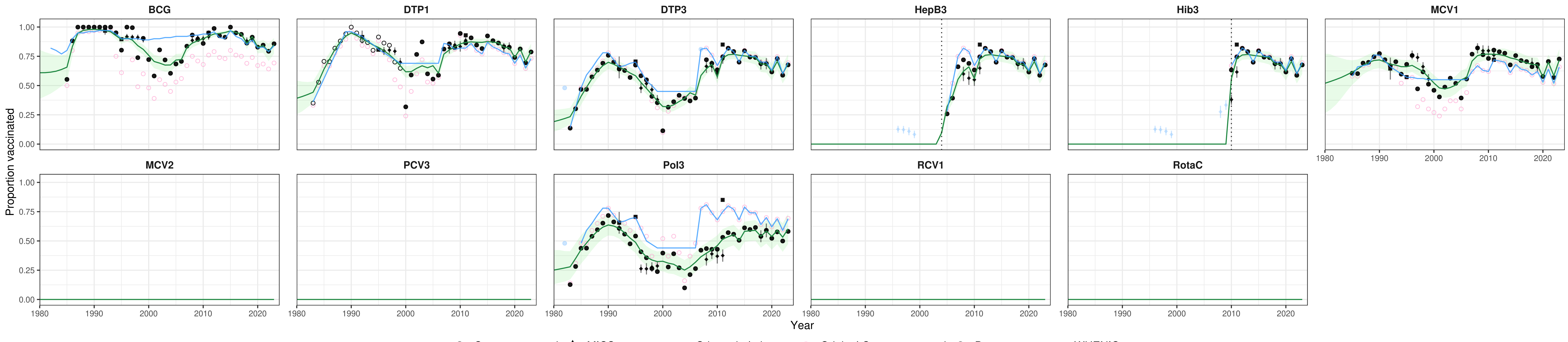

BCG
DTP1
DTP3
HepB3
Hib3
MCV1
MCV2
PCV3
Pol3
RCV1
RotaC
Proportion vaccinated
Year
0.00
0.25
0.50
0.75
1.00
1980
1990
2000
2010
2020
Country-reported
MICS
Other tabulation
Original Country-reported
Data
WUENIC
DHS
Other microdata
Imputed
Outlier
GBD Estimate

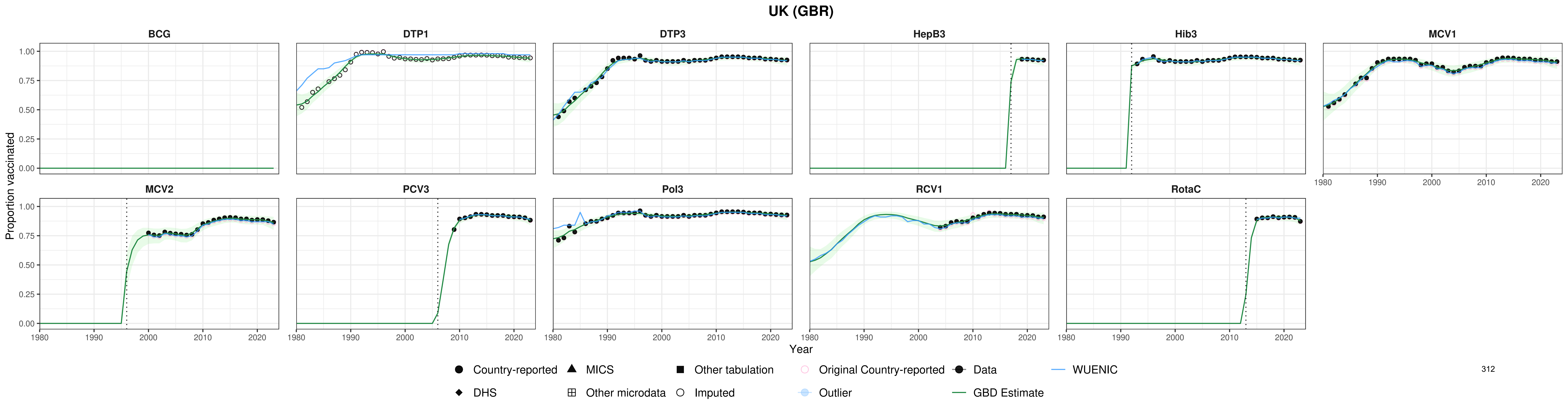

UK (GBR)
BCG
DTP1
DTP3
HepB3
Hib3
MCV1
MCV2
PCV3
Pol3
RCV1
RotaC
Proportion vaccinated
Year
Country-reported
MICS
Other tabulation
Original Country-reported
Data
WUENIC
DHS
Other microdata
Imputed
Outlier
GBD Estimate

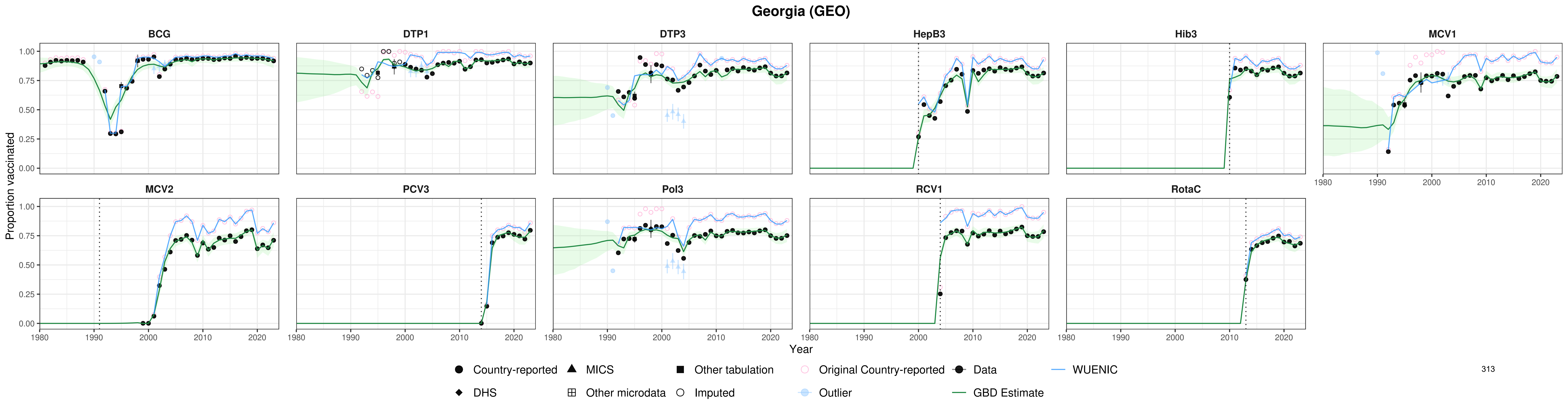
Georgia (GEO)
BCG
DTP1
DTP3
HepB3
Hib3
MCV1
MCV2
PCV3
Pol3
RCV1
RotaC
Proportion vaccinated
Year
Country-reported
MICS
Other tabulation
Original Country-reported
Data
WUENIC
DHS
Other microdata
Imputed
Outlier
GBD Estimate

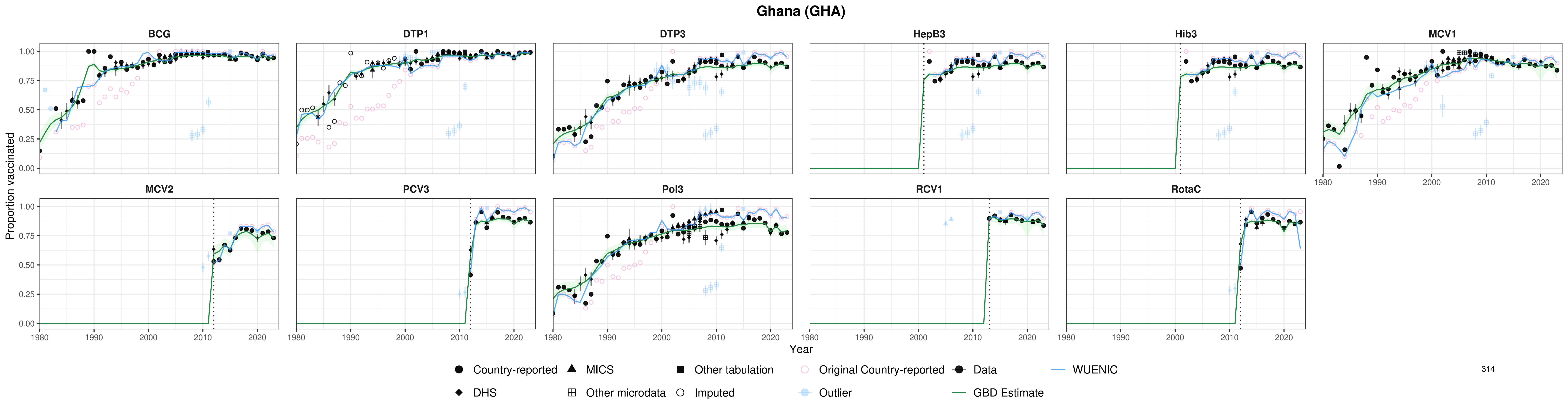

Ghana (GHA)
BCG
DTP1
DTP3
HepB3
Hib3
MCV1
MCV2
PCV3
Pol3
RCV1
RotaC
Proportion vaccinated
Year
Country-reported
DHS
MICS
Other microdata
Other tabulation
Imputed
Original Country-reported
Outlier
Data
GBD Estimate
WUENIC

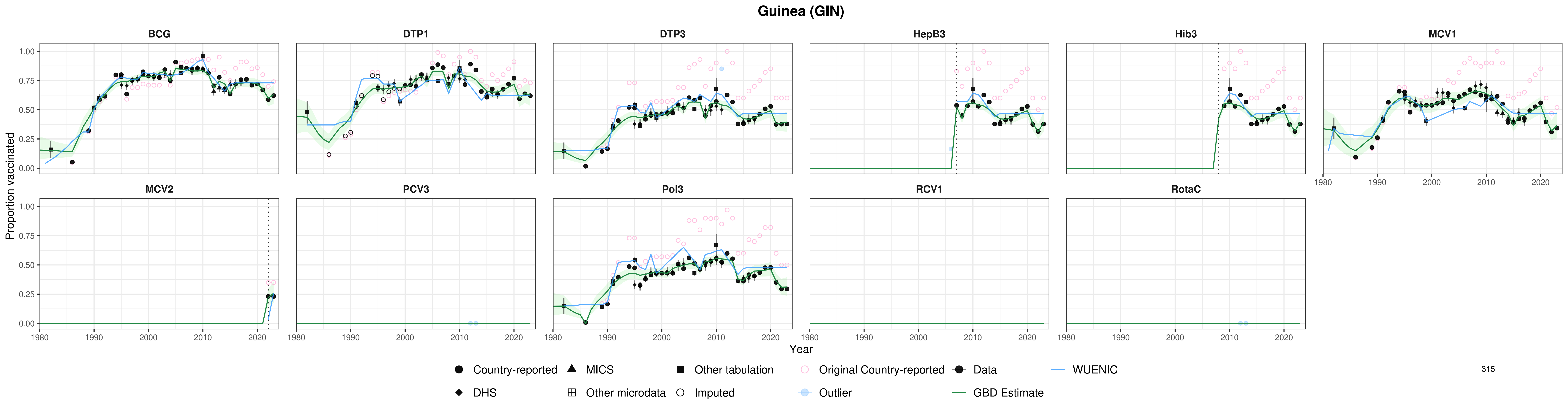
Guinea (GIN)
BCG
DTP1
DTP3
HepB3
Hib3
MCV1
MCV2
PCV3
Pol3
RCV1
RotaC
Proportion vaccinated
Year
Country-reported
MICS
Other tabulation
Original Country-reported
Data
WUENIC
DHS
Other microdata
Imputed
Outlier
GBD Estimate

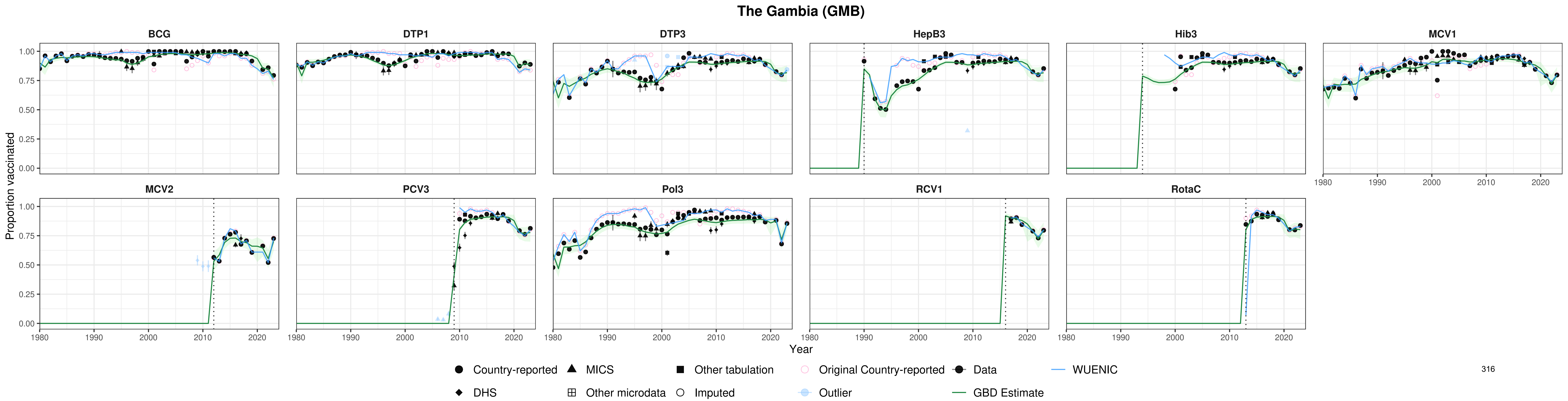
The Gambia (GMB)
BCG
DTP1
DTP3
HepB3
Hib3
MCV1
MCV2
PCV3
Pol3
RCV1
RotaC
Proportion vaccinated
Year
1.00
0.75
0.50
0.25
0.00
1980
1990
2000
2010
2020
Country-reported
MICS
Other tabulation
Original Country-reported
Data
WUENIC
DHS
Other microdata
Imputed
Outlier
GBD Estimate

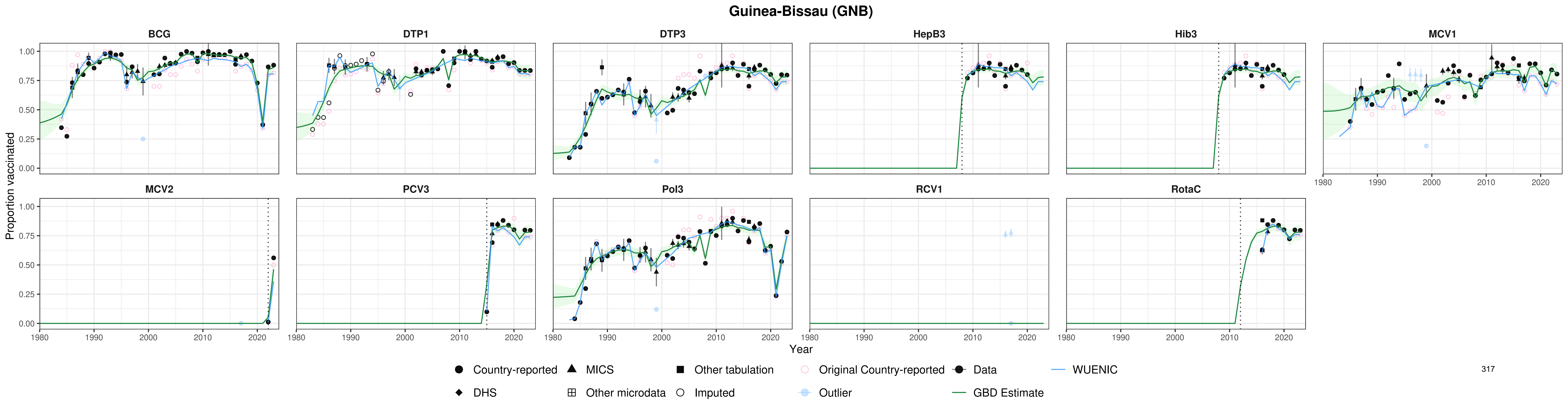

Guinea-Bissau (GNB)
BCG
DTP1
DTP3
HepB3
Hib3
MCV1
MCV2
PCV3
Pol3
RCV1
RotaC
Proportion vaccinated
Year
0.00
0.25
0.50
0.75
1.00
1980
1990
2000
2010
2020
Country-reported
MICS
Other tabulation
Original Country-reported
Data
WUENIC
DHS
Other microdata
Imputed
Outlier
GBD Estimate

Equatorial Guinea (GNQ)

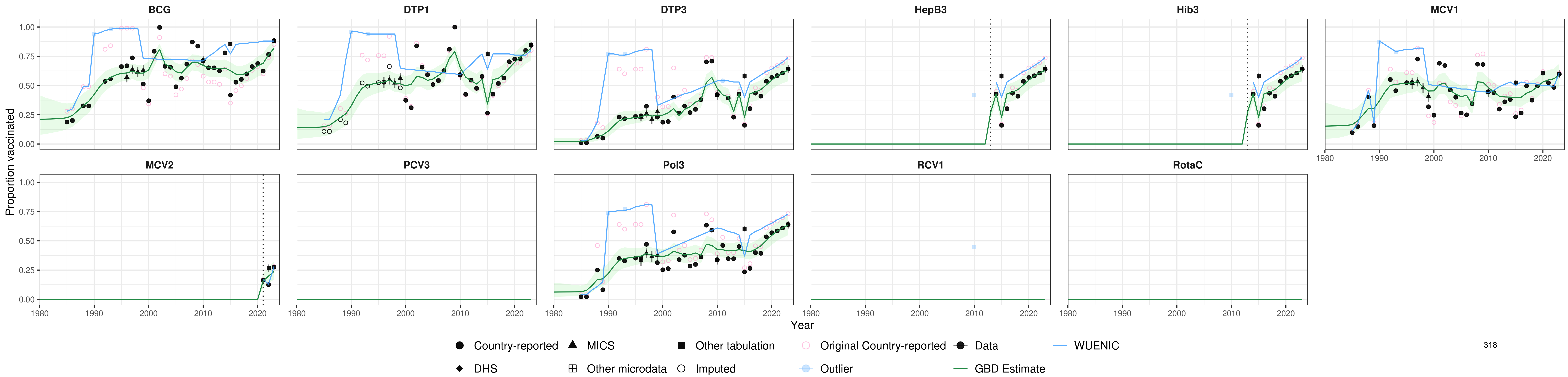

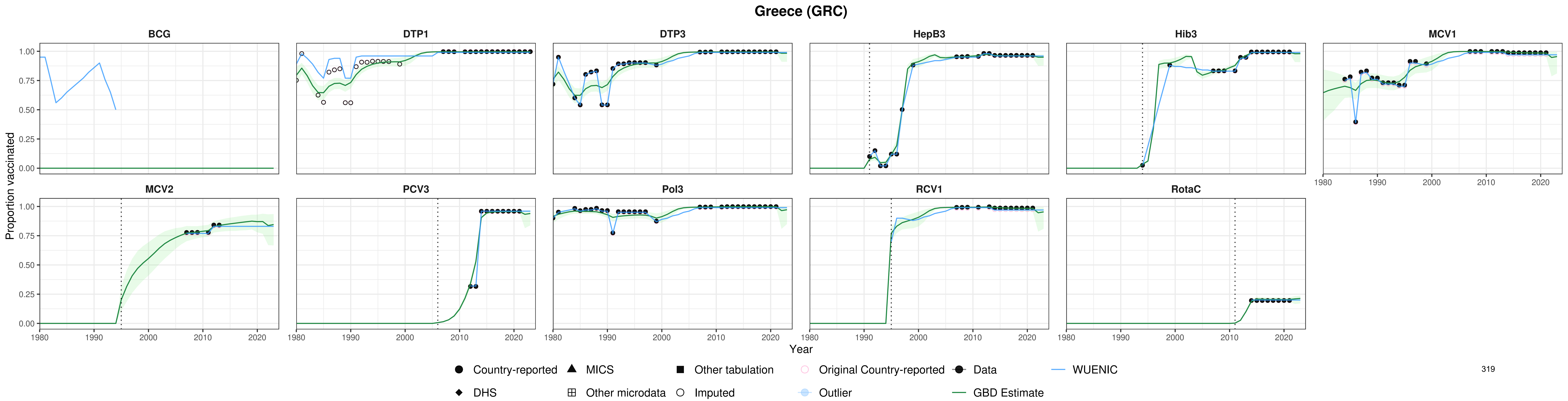
Greece (GRC)
BCG
DTP1
DTP3
HepB3
Hib3
MCV1
MCV2
PCV3
Pol3
RCV1
RotaC
Proportion vaccinated
Year
Country-reported
MICS
Other tabulation
Original Country-reported
Data
WUENIC
DHS
Other microdata
Imputed
Outlier
GBD Estimate

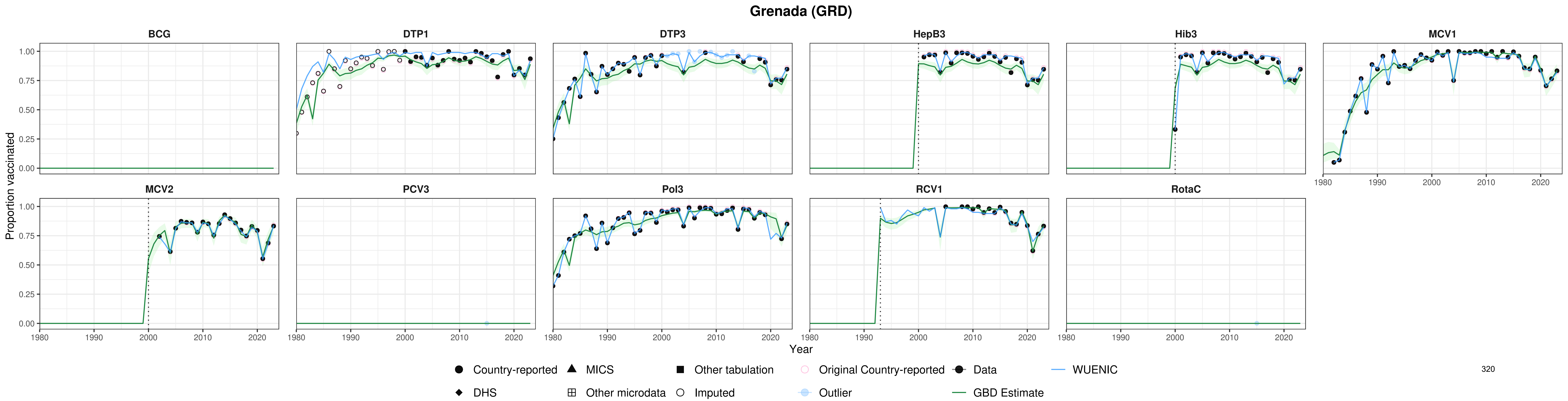
Grenada (GRD)
BCG
DTP1
DTP3
HepB3
Hib3
MCV1
MCV2
PCV3
Pol3
RCV1
RotaC
Proportion vaccinated
Year
Country-reported
MICS
Other tabulation
Original Country-reported
Data
WUENIC
DHS
Other microdata
Imputed
Outlier
GBD Estimate

Greenland (GRL)

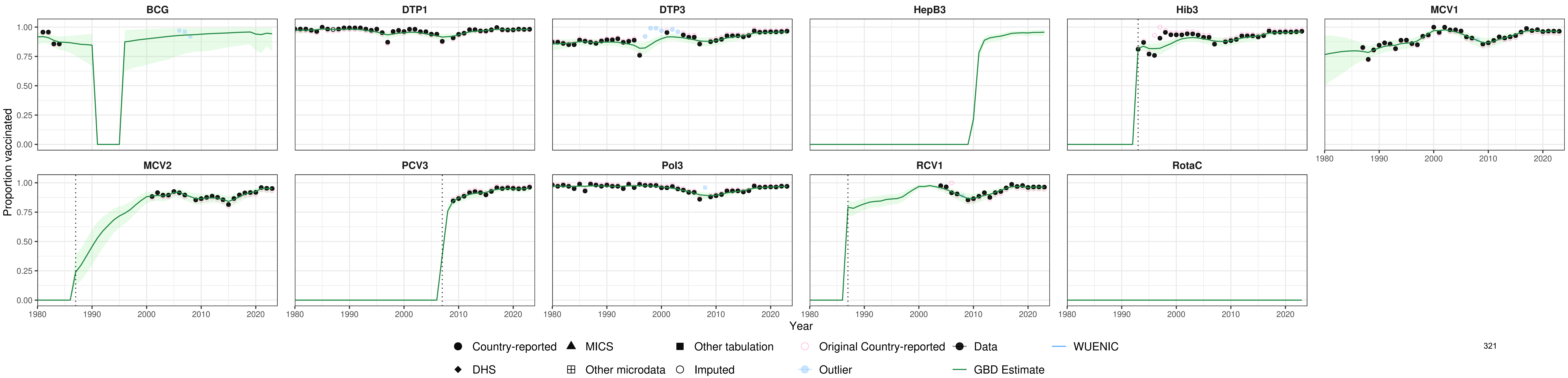

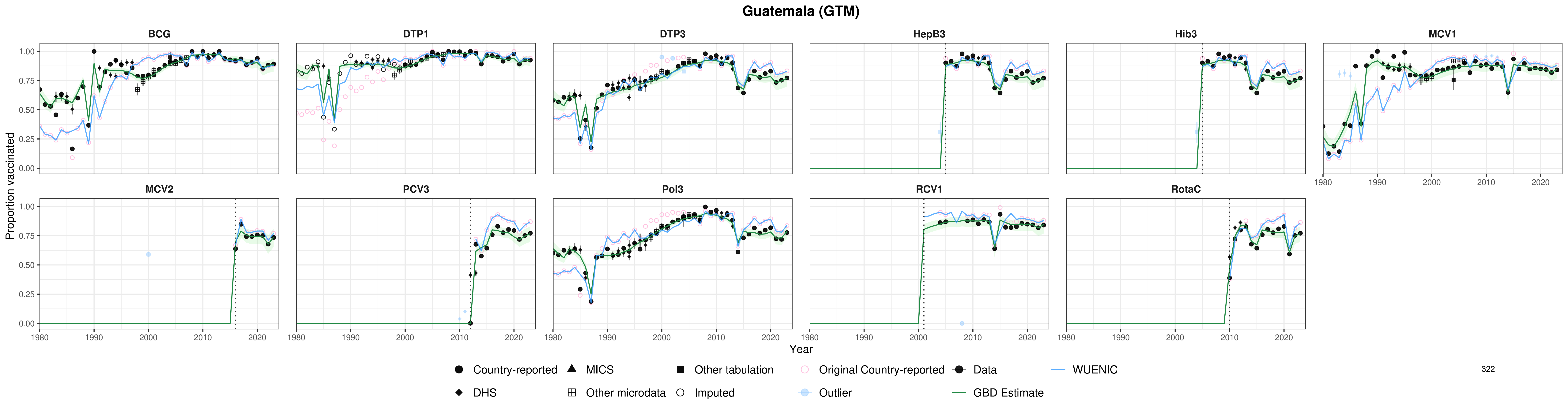

Guatemala (GTM)
BCG
DTP1
DTP3
HepB3
Hib3
MCV1
MCV2
PCV3
Pol3
RCV1
RotaC
Proportion vaccinated
Year
Country-reported
MICS
Other tabulation
Original Country-reported
Data
WUENIC
DHS
Other microdata
Imputed
Outlier
GBD Estimate

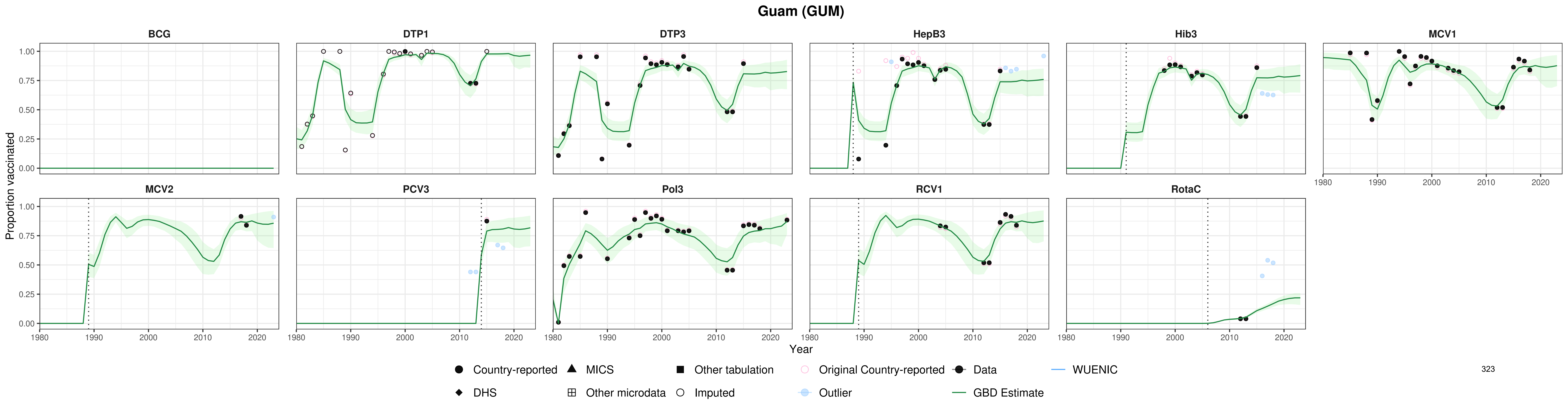
Guam (GUM)
BCG
DTP1
DTP3
HepB3
Hib3
MCV1
MCV2
PCV3
Pol3
RCV1
RotaC
Proportion vaccinated
Year
Country-reported
MICS
Other tabulation
Original Country-reported
Data
WUENIC
DHS
Other microdata
Imputed
Outlier
GBD Estimate

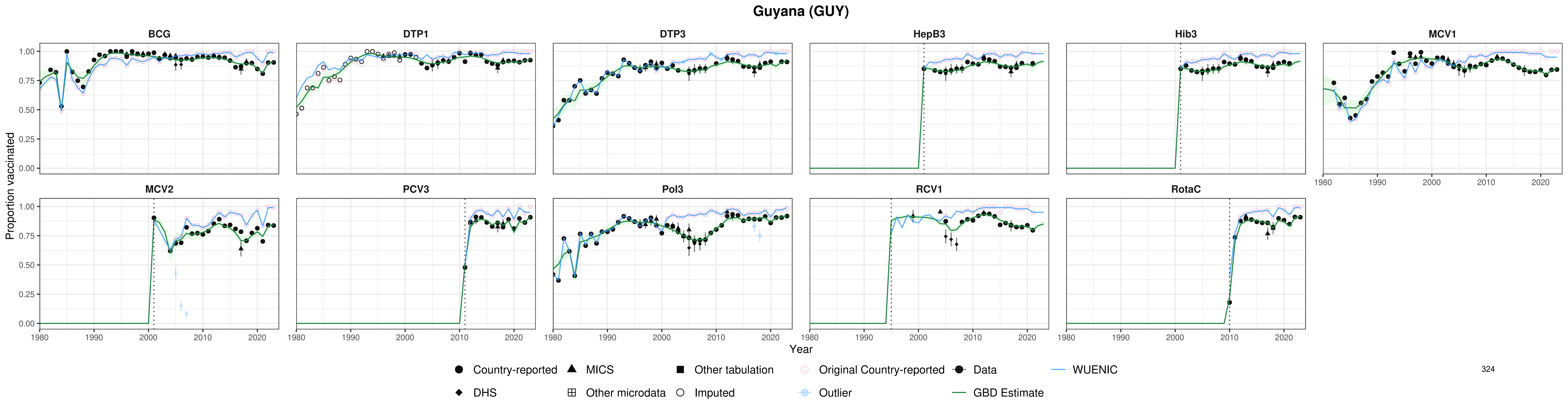

Guyana (GUY)
BCG
DTP1
DTP3
HepB3
Hib3
MCV1
MCV2
PCV3
Pol3
RCV1
RotaC
Proportion vaccinated
0.00
0.25
0.50
0.75
1.00
1980
1990
2000
2010
2020
Year
Country-reported
MICS
Other tabulation
Original Country-reported
Data
WUENIC
DHS
Other microdata
Imputed
Outlier
GBD Estimate

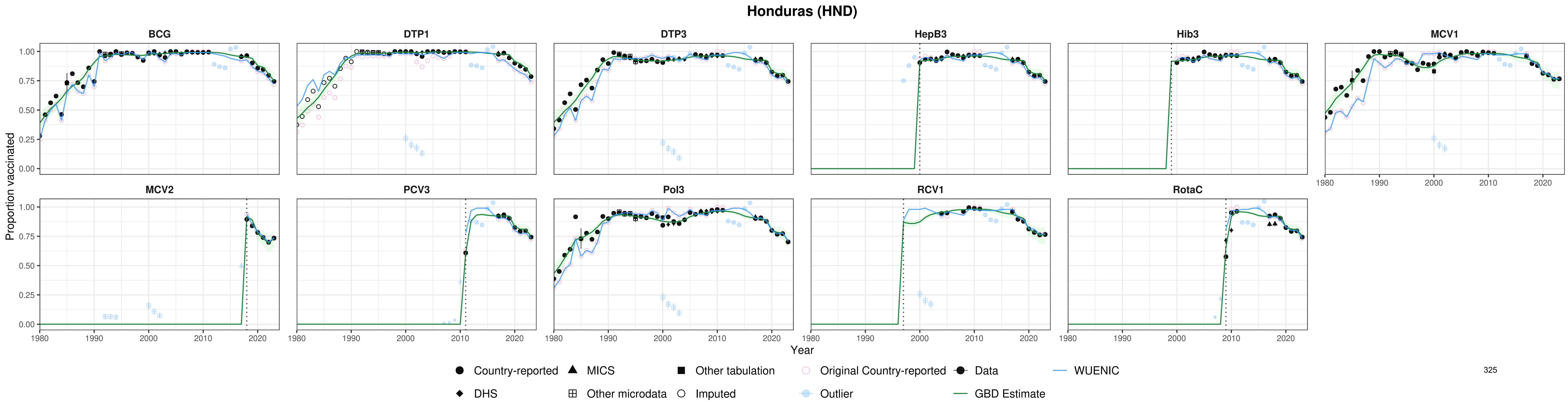
Honduras (HND)
BCG
DTP1
DTP3
HepB3
Hib3
MCV1
MCV2
PCV3
Pol3
RCV1
RotaC
Proportion vaccinated
Year
Country-reported
MICS
Other tabulation
Original Country-reported
Data
WUENIC
DHS
Other microdata
Imputed
Outlier
GBD Estimate

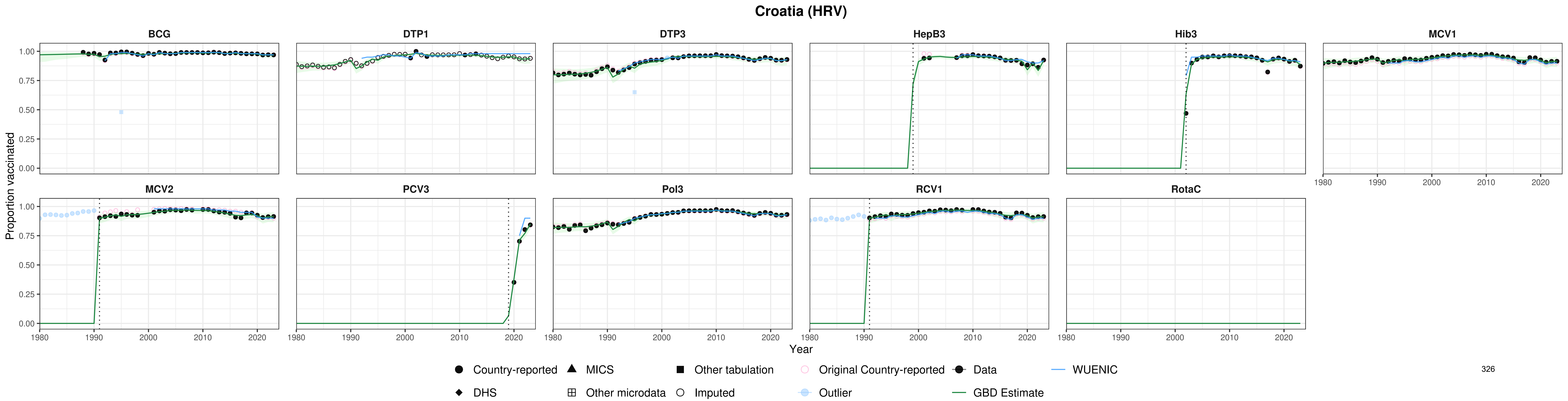

Croatia (HRV)
BCG
DTP1
DTP3
HepB3
Hib3
MCV1
MCV2
PCV3
Pol3
RCV1
RotaC
Proportion vaccinated
Year
Country-reported
MICS
Other tabulation
Original Country-reported
Data
WUENIC
DHS
Other microdata
Imputed
Outlier
GBD Estimate

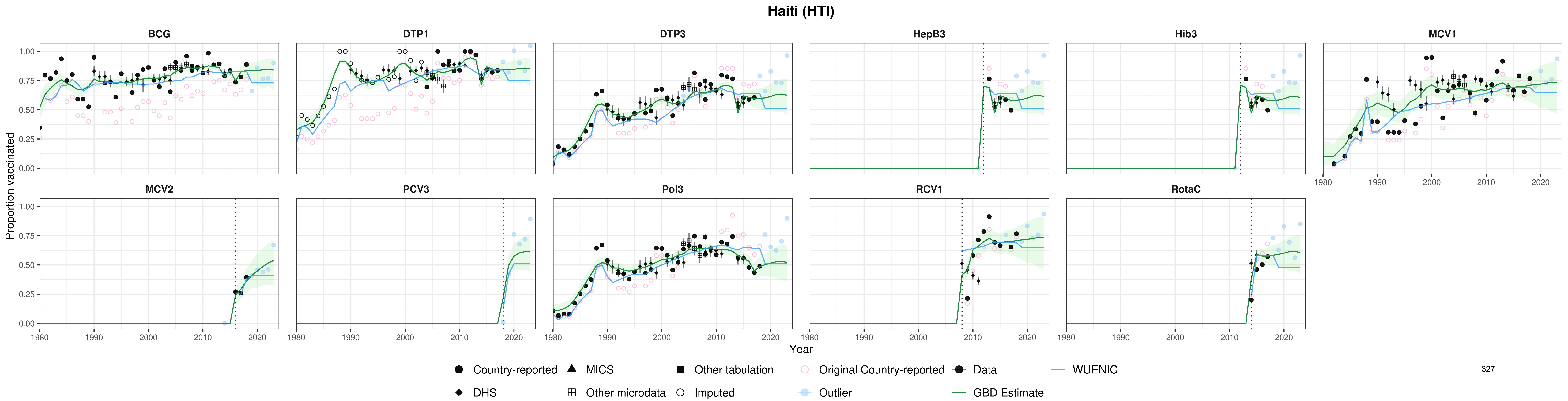

Haiti (HTI)
BCG
DTP1
DTP3
HepB3
Hib3
MCV1
MCV2
PCV3
Pol3
RCV1
RotaC
Proportion vaccinated
Year
0.00
0.25
0.50
0.75
1.00
1980
1990
2000
2010
2020
Country-reported
MICS
Other tabulation
Original Country-reported
Data
WUENIC
DHS
Other microdata
Imputed
Outlier
GBD Estimate

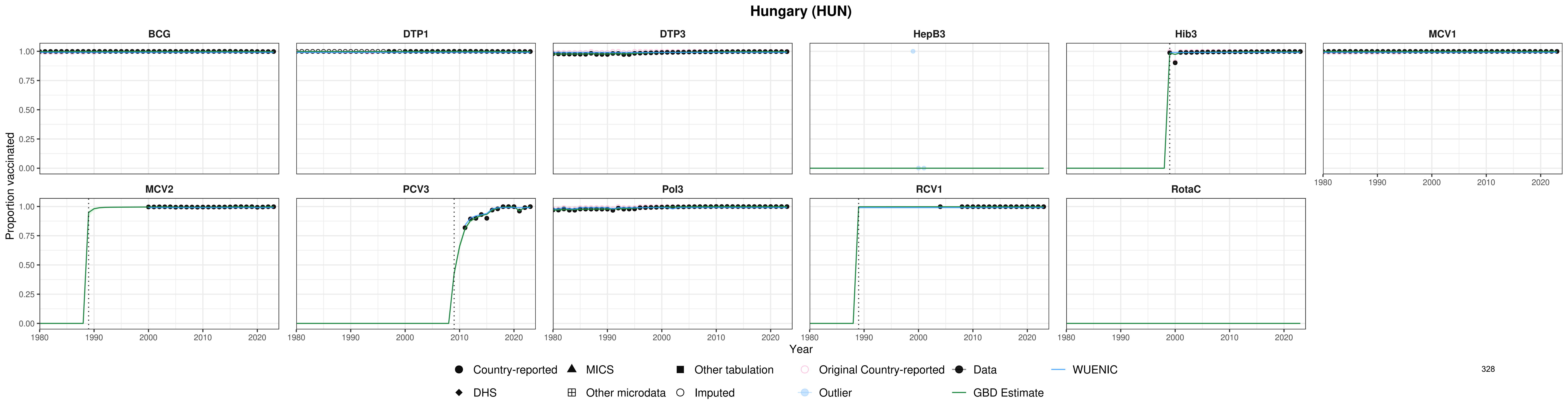

Hungary (HUN)
BCG
DTP1
DTP3
HepB3
Hib3
MCV1
MCV2
PCV3
Pol3
RCV1
RotaC
Proportion vaccinated
0.00
0.25
0.50
0.75
1.00
1980
1990
2000
2010
2020
Year
Country-reported
MICS
Other tabulation
Original Country-reported
Data
WUENIC
DHS
Other microdata
Imputed
Outlier
GBD Estimate

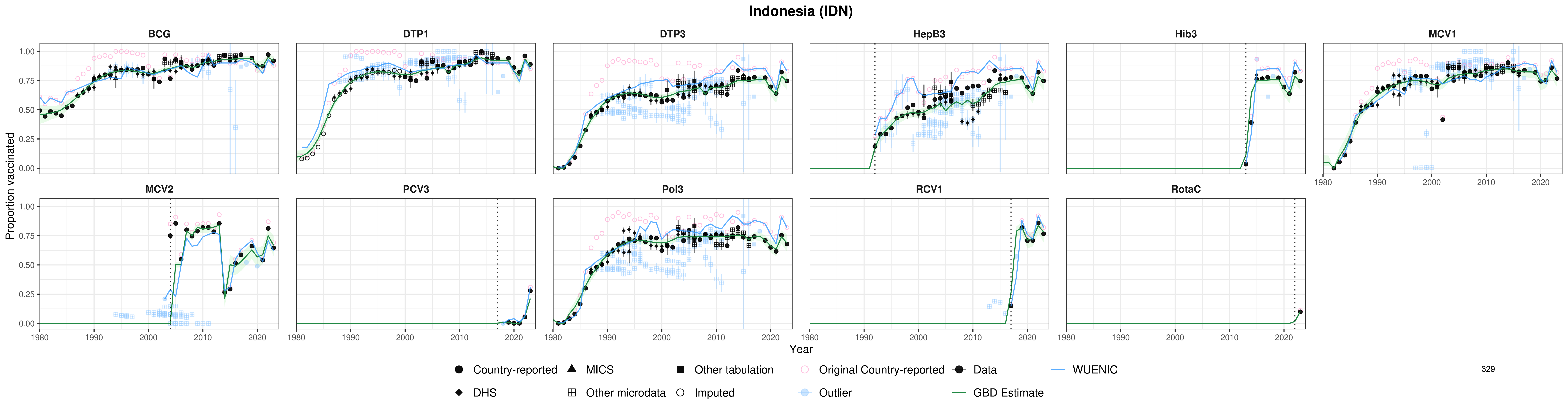

Indonesia (IDN)
BCG
DTP1
DTP3
HepB3
Hib3
MCV1
MCV2
PCV3
Pol3
RCV1
RotaC
Proportion vaccinated
Year
Country-reported
DHS
MICS
Other microdata
Other tabulation
Imputed
Original Country-reported
Outlier
Data
GBD Estimate
WUENIC

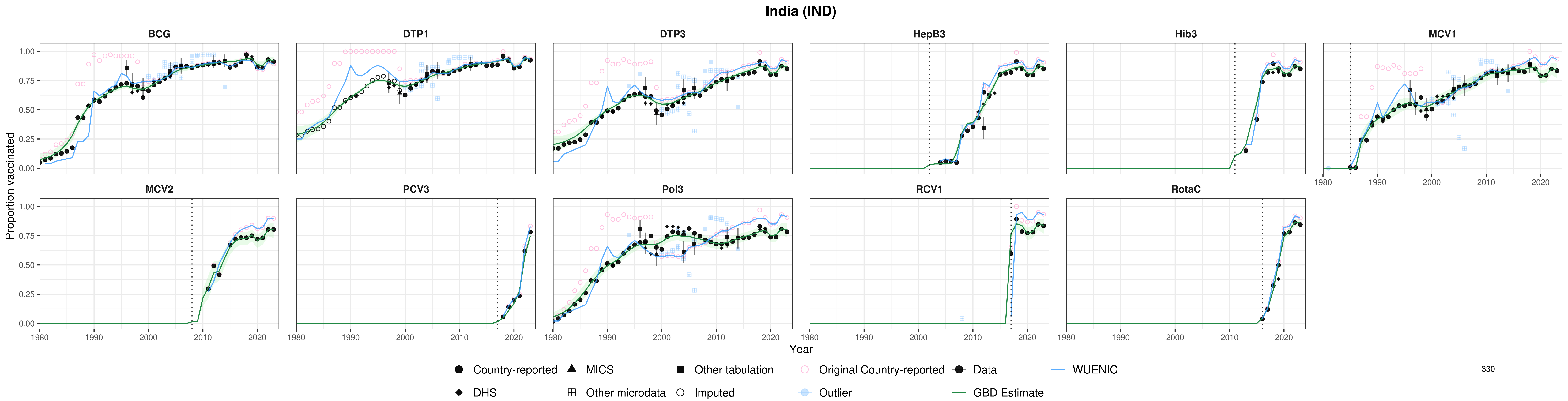
India (IND)
BCG
DTP1
DTP3
HepB3
Hib3
MCV1
MCV2
PCV3
Pol3
RCV1
RotaC
Proportion vaccinated
Year
Country-reported
MICS
Other tabulation
Original Country-reported
Data
WUENIC
DHS
Other microdata
Imputed
Outlier
GBD Estimate

Ireland (IRL)

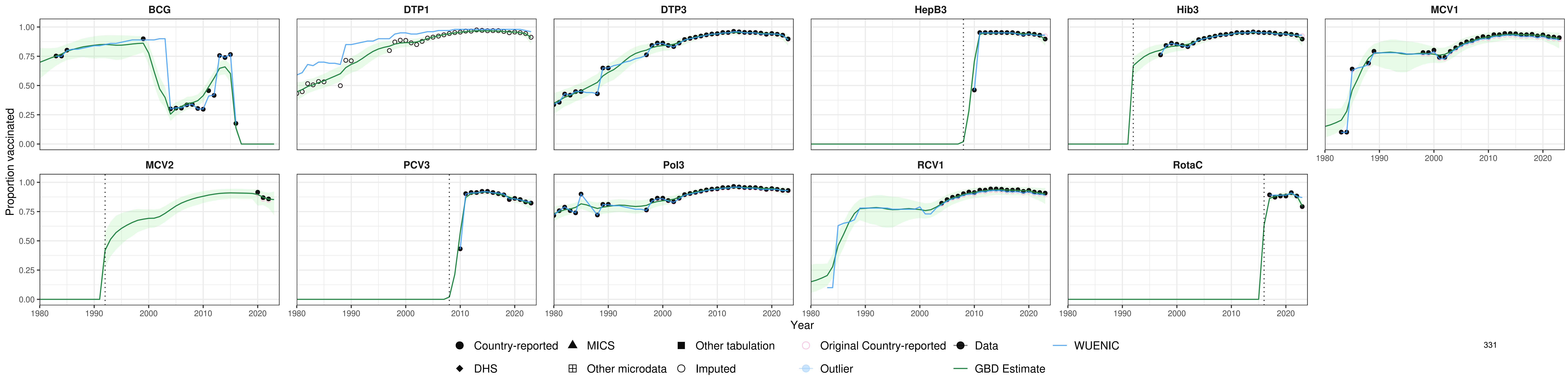

BCG
DTP1
DTP3
HepB3
Hib3
MCV1
MCV2
PCV3
Pol3
RCV1
RotaC
Proportion vaccinated
Year
Country-reported
MICS
Other tabulation
Original Country-reported
Data
WUENIC
DHS
Other microdata
Imputed
Outlier
GBD Estimate

Iran (IRN)

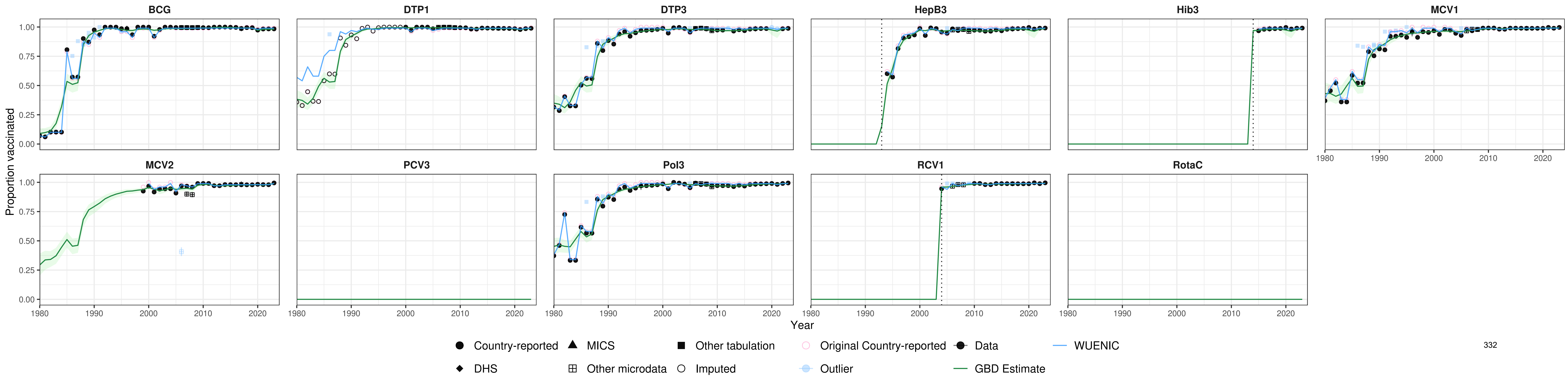

BCG
DTP1
DTP3
HepB3
Hib3
MCV1
MCV2
PCV3
Pol3
RCV1
RotaC
Proportion vaccinated
1.00
0.75
0.50
0.25
0.00
1980
1990
2000
2010
2020
Year
Country-reported
MICS
Other tabulation
Original Country-reported
Data
WUENIC
DHS
Other microdata
Imputed
Outlier
GBD Estimate

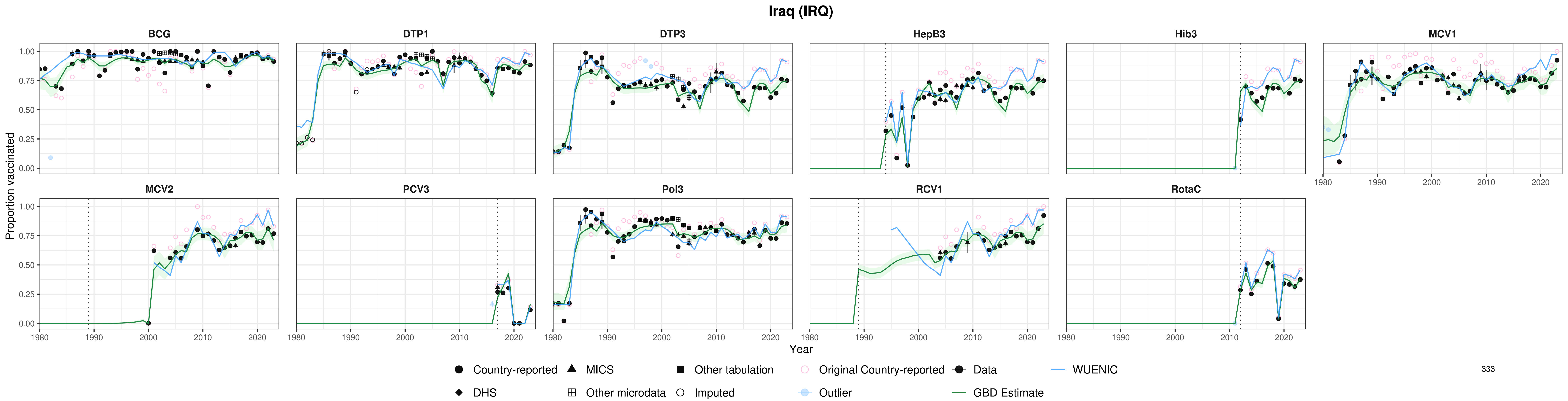

Iraq (IRQ)
BCG
DTP1
DTP3
HepB3
Hib3
MCV1
MCV2
PCV3
Pol3
RCV1
RotaC
Proportion vaccinated
Year
Country-reported
MICS
Other tabulation
Original Country-reported
Data
WUENIC
DHS
Other microdata
Imputed
Outlier
GBD Estimate

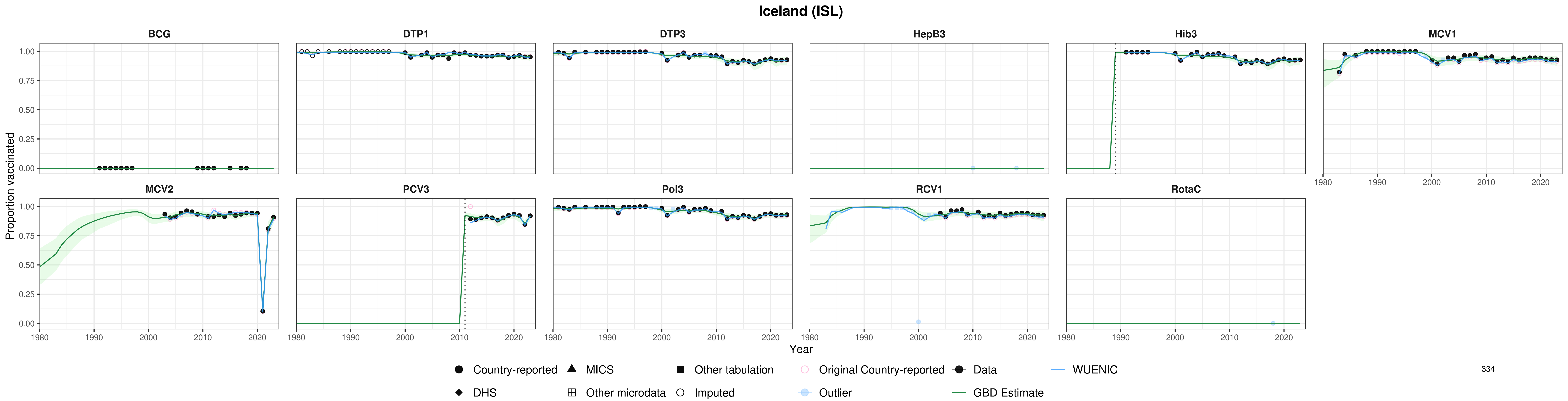

Iceland (ISL)
BCG
DTP1
DTP3
HepB3
Hib3
MCV1
MCV2
PCV3
Pol3
RCV1
RotaC
Proportion vaccinated
0.00
0.25
0.50
0.75
1.00
1980
1990
2000
2010
2020
Year
Country-reported
MICS
Other tabulation
Original Country-reported
Data
WUENIC
DHS
Other microdata
Imputed
Outlier
GBD Estimate

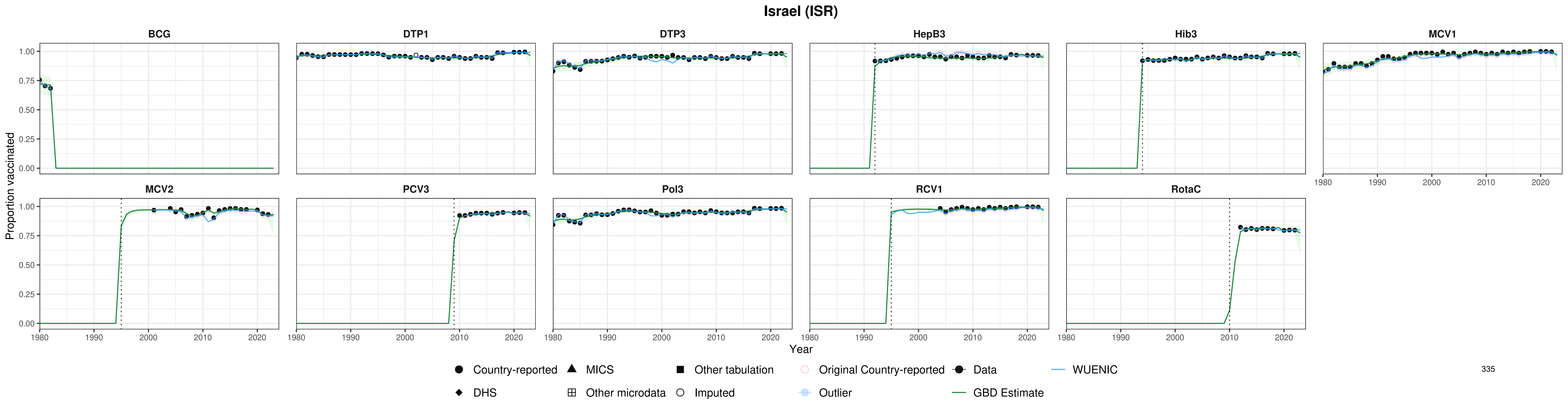
Israel (ISR)
BCG
DTP1
DTP3
HepB3
Hib3
MCV1
MCV2
PCV3
Pol3
RCV1
RotaC
Proportion vaccinated
Year
1.00
0.75
0.50
0.25
0.00
1980
1990
2000
2010
2020
Country-reported
MICS
Other tabulation
Original Country-reported
Data
WUENIC
DHS
Other microdata
Imputed
Outlier
GBD Estimate

Italy (ITA)

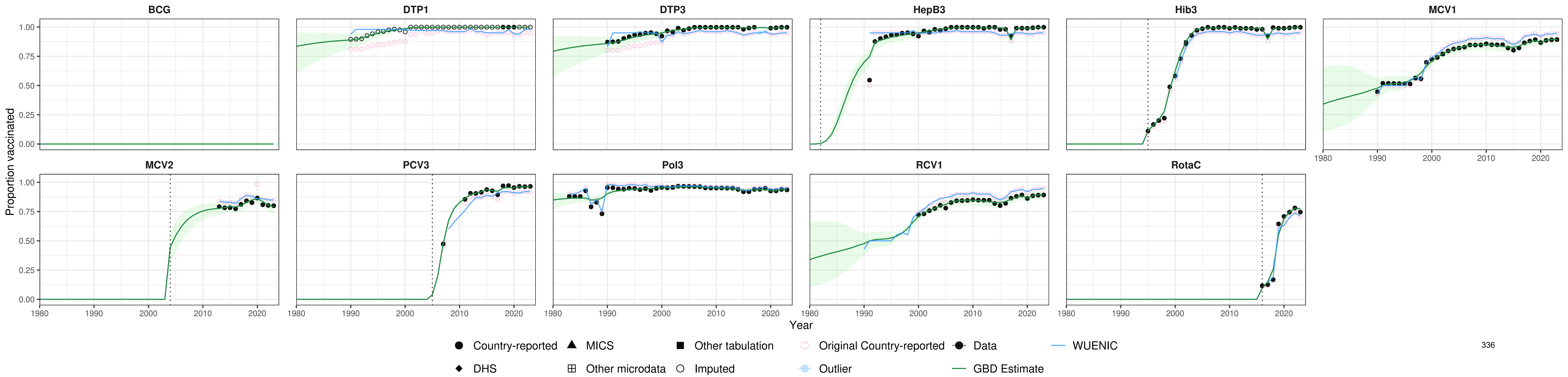

BCG
DTP1
DTP3
HepB3
Hib3
MCV1
MCV2
PCV3
Pol3
RCV1
RotaC
Proportion vaccinated
1.00
0.75
0.50
0.25
0.00
1980
1990
2000
2010
2020
Year
Country-reported
MICS
Other tabulation
Original Country-reported
Data
WUENIC
DHS
Other microdata
Imputed
Outlier
GBD Estimate

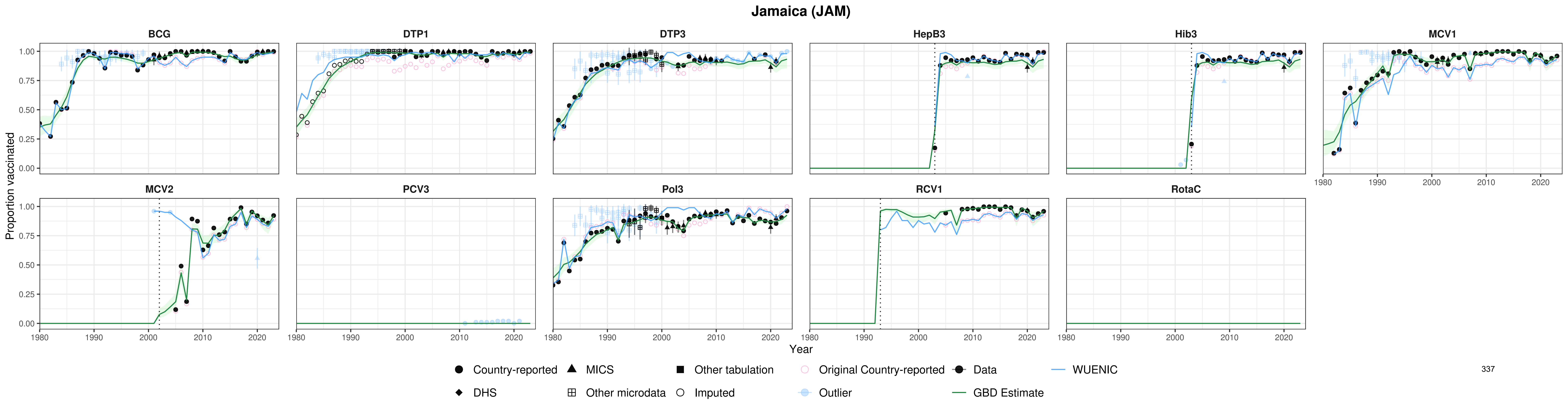

Jamaica (JAM)
BCG
DTP1
DTP3
HepB3
Hib3
MCV1
MCV2
PCV3
Pol3
RCV1
RotaC
Proportion vaccinated
Year
Country-reported
MICS
Other tabulation
Original Country-reported
Data
WUENIC
DHS
Other microdata
Imputed
Outlier
GBD Estimate

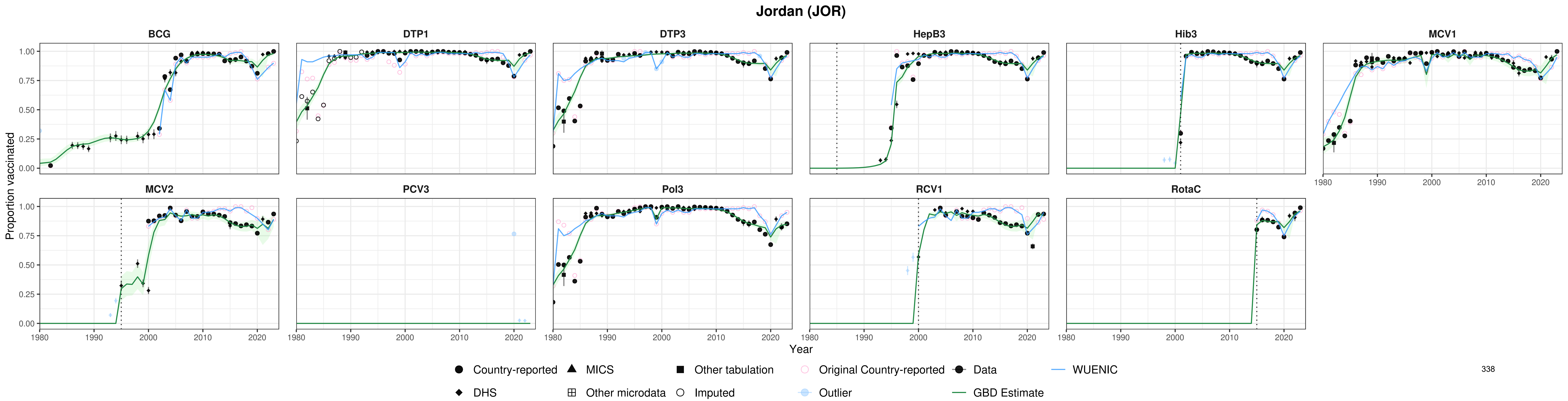

Jordan (JOR)
BCG
DTP1
DTP3
HepB3
Hib3
MCV1
MCV2
PCV3
Pol3
RCV1
RotaC
Proportion vaccinated
Year
Country-reported
MICS
Other tabulation
Original Country-reported
Data
WUENIC
DHS
Other microdata
Imputed
Outlier
GBD Estimate

Japan (JPN)

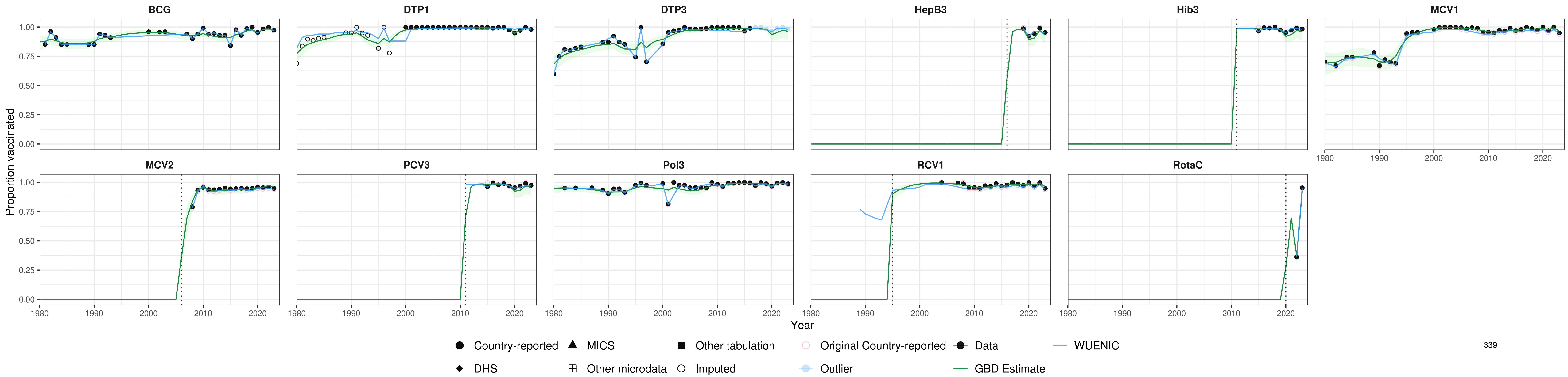

BCG
DTP1
DTP3
HepB3
Hib3
MCV1
MCV2
PCV3
Pol3
RCV1
RotaC
Proportion vaccinated
Year
0.00
0.25
0.50
0.75
1.00
1980
1990
2000
2010
2020
Country-reported
MICS
Other tabulation
Original Country-reported
Data
WUENIC
DHS
Other microdata
Imputed
Outlier
GBD Estimate

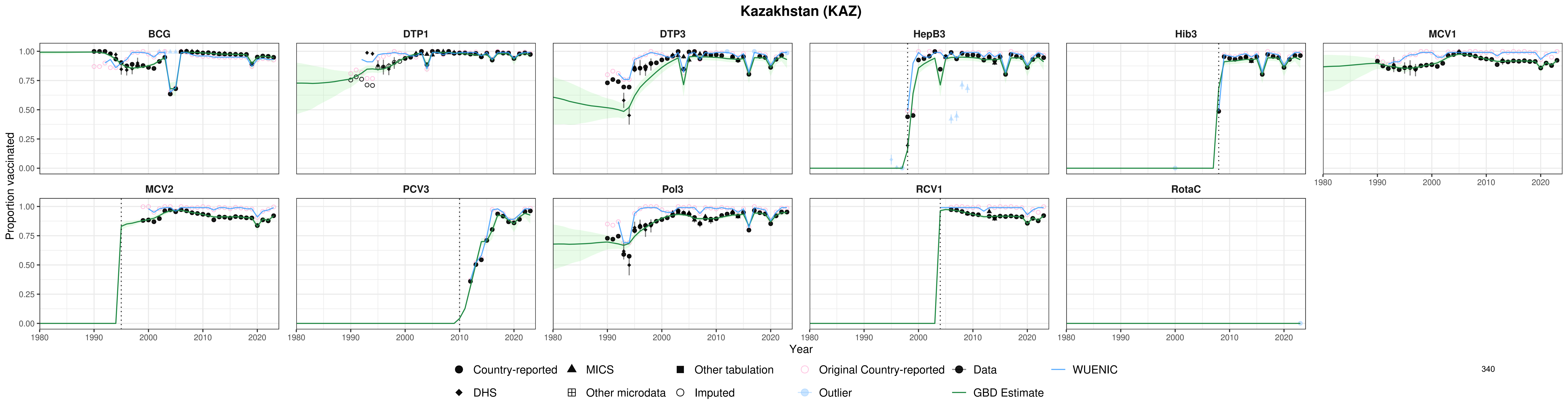
Kazakhstan (KAZ)
BCG
DTP1
DTP3
HepB3
Hib3
MCV1
MCV2
PCV3
Pol3
RCV1
RotaC
Proportion vaccinated
Year
1.00
0.75
0.50
0.25
0.00
1980
1990
2000
2010
2020
Country-reported
MICS
Other tabulation
Original Country-reported
Data
WUENIC
DHS
Other microdata
Imputed
Outlier
GBD Estimate

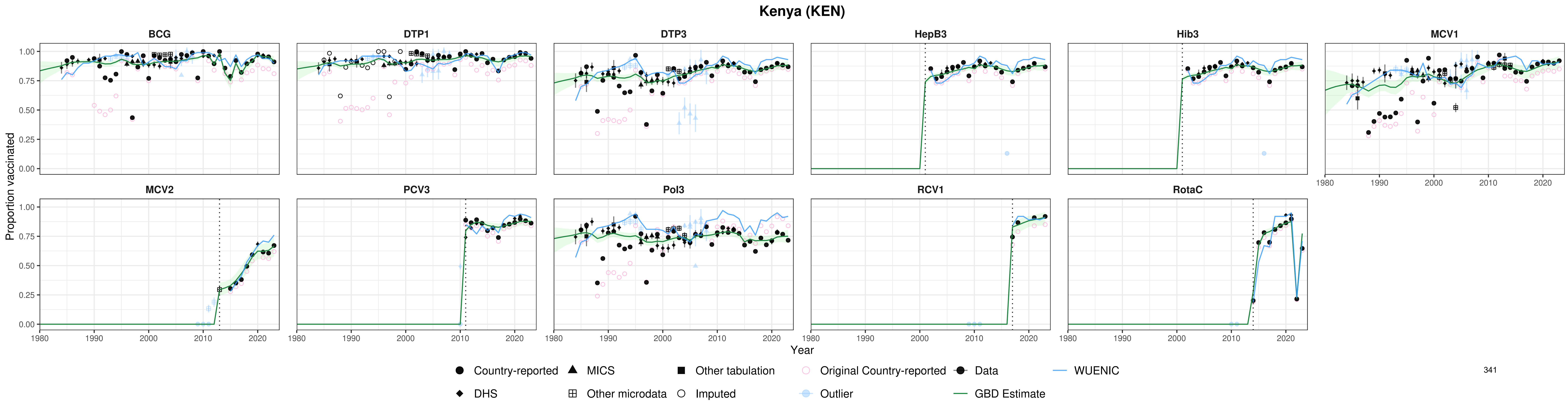

Kenya (KEN)
BCG
DTP1
DTP3
HepB3
Hib3
MCV1
MCV2
PCV3
Pol3
RCV1
RotaC
Proportion vaccinated
Year
1.00
0.75
0.50
0.25
0.00
1980
1990
2000
2010
2020
Country-reported
DHS
MICS
Other microdata
Other tabulation
Imputed
Original Country-reported
Outlier
Data
GBD Estimate
WUENIC

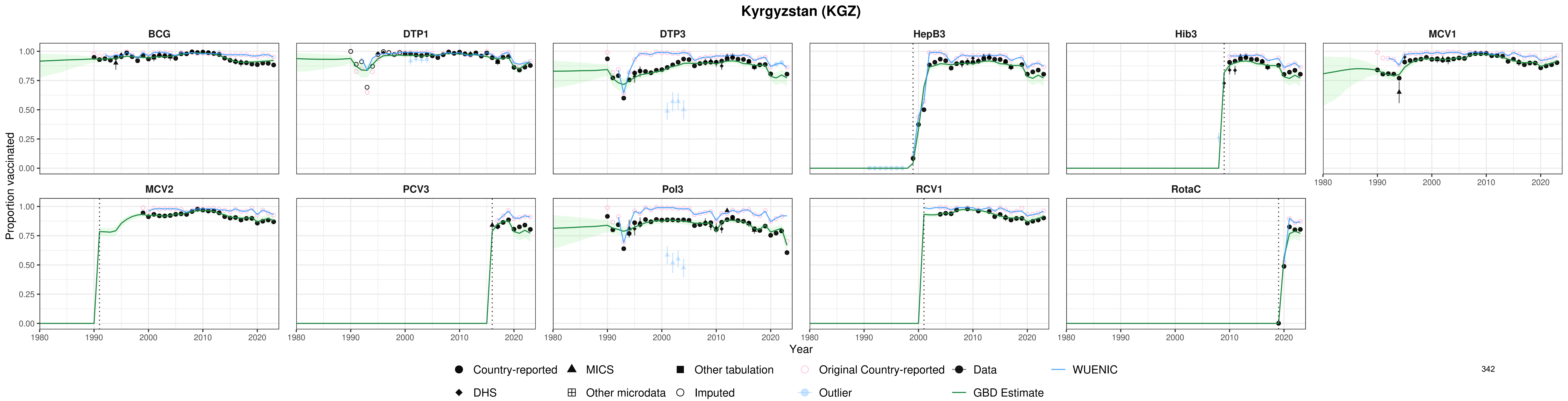

Kyrgyzstan (KGZ)
BCG
DTP1
DTP3
HepB3
Hib3
MCV1
MCV2
PCV3
Pol3
RCV1
RotaC
Proportion vaccinated
Year
Country-reported
MICS
Other tabulation
Original Country-reported
Data
WUENIC
DHS
Other microdata
Imputed
Outlier
GBD Estimate

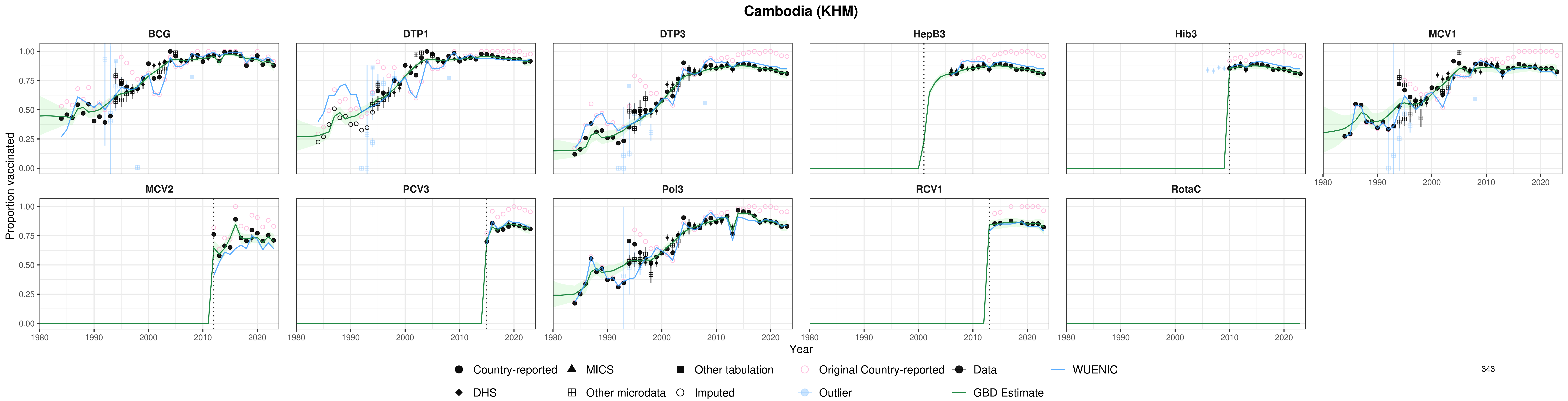

Cambodia (KHM)
BCG
DTP1
DTP3
HepB3
Hib3
MCV1
MCV2
PCV3
Pol3
RCV1
RotaC
Proportion vaccinated
Year
Country-reported
MICS
Other tabulation
Original Country-reported
Data
WUENIC
DHS
Other microdata
Imputed
Outlier
GBD Estimate

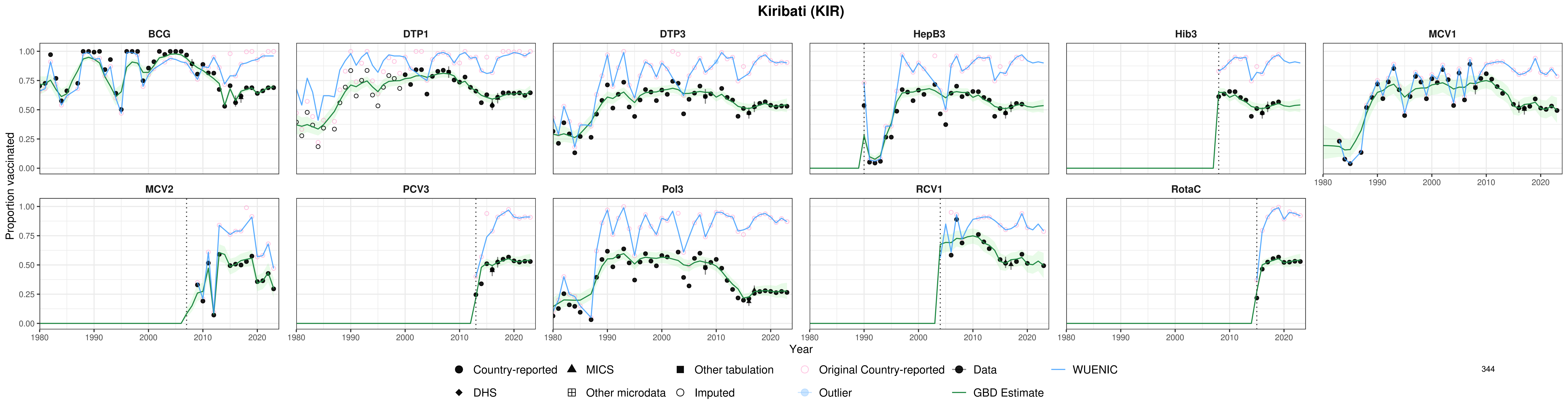
Kiribati (KIR)
BCG
DTP1
DTP3
HepB3
Hib3
MCV1
MCV2
PCV3
Pol3
RCV1
RotaC
Proportion vaccinated
Year
Country-reported
MICS
Other tabulation
Original Country-reported
Data
WUENIC
DHS
Other microdata
Imputed
Outlier
GBD Estimate

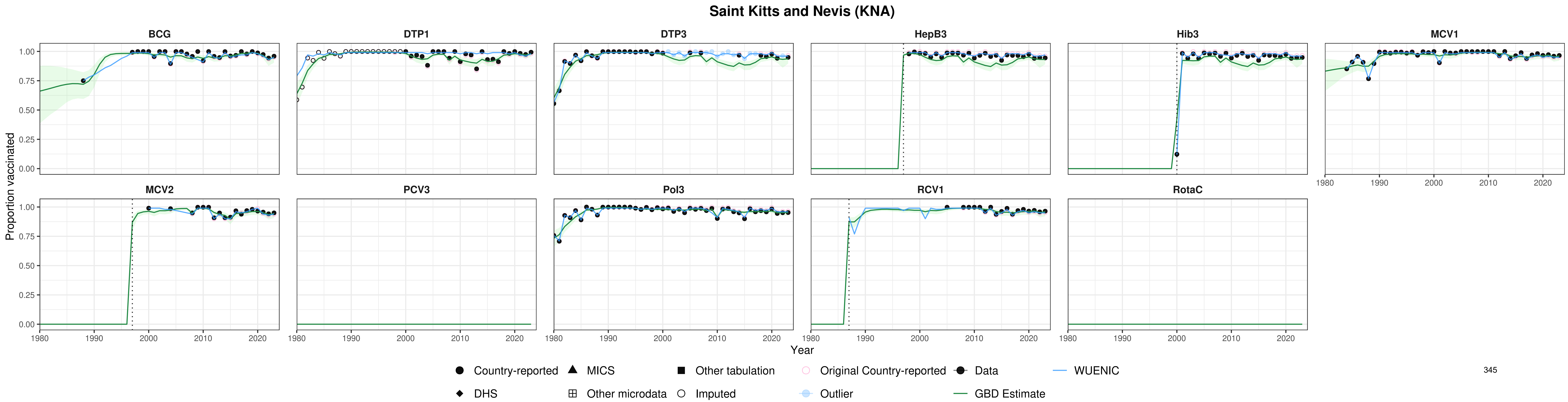
Saint Kitts and Nevis (KNA)
BCG
DTP1
DTP3
HepB3
Hib3
MCV1
MCV2
PCV3
Pol3
RCV1
RotaC
Proportion vaccinated
Year
Country-reported
MICS
Other tabulation
Original Country-reported
Data
WUENIC
DHS
Other microdata
Imputed
Outlier
GBD Estimate

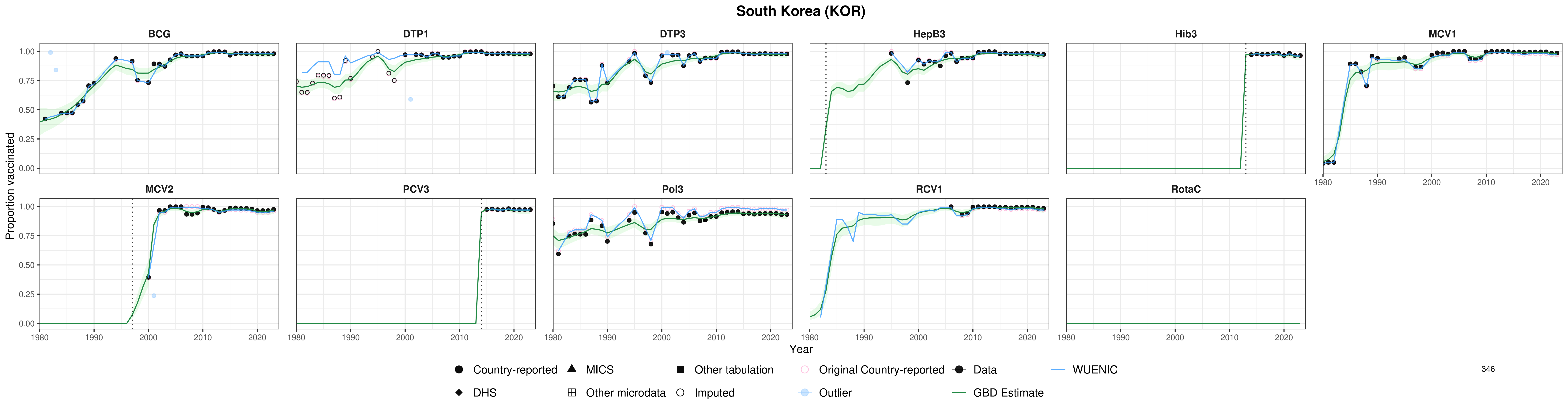
South Korea (KOR)
BCG
DTP1
DTP3
HepB3
Hib3
MCV1
MCV2
PCV3
Pol3
RCV1
RotaC
Proportion vaccinated
Year
Country-reported
MICS
Other tabulation
Original Country-reported
Data
WUENIC
DHS
Other microdata
Imputed
Outlier
GBD Estimate

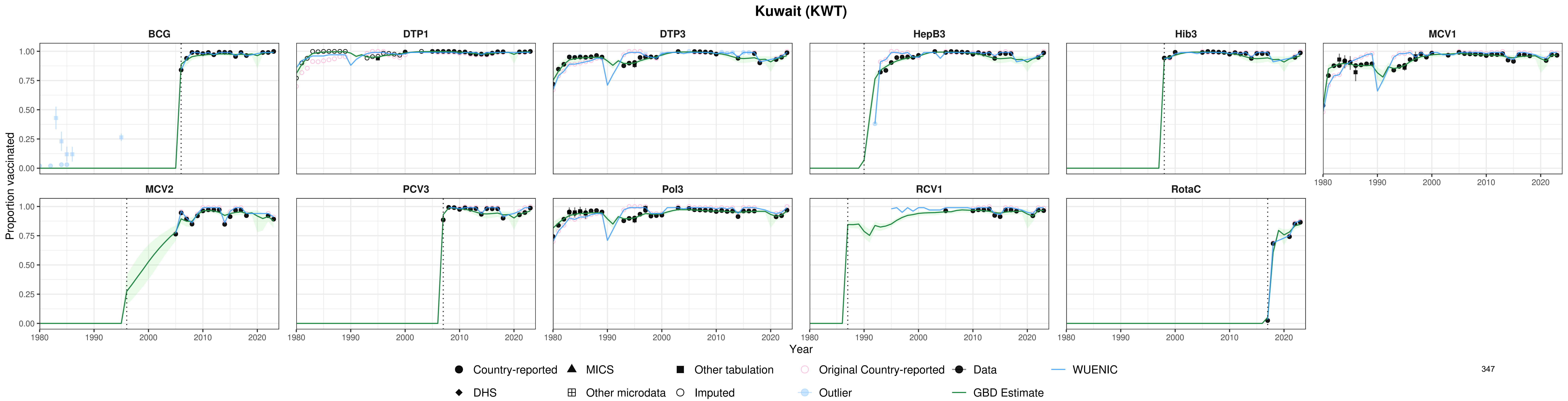
Kuwait (KWT)
BCG
DTP1
DTP3
HepB3
Hib3
MCV1
MCV2
PCV3
Pol3
RCV1
RotaC
Proportion vaccinated
Year
Country-reported
MICS
Other tabulation
Original Country-reported
Data
WUENIC
DHS
Other microdata
Imputed
Outlier
GBD Estimate

Laos (LAO)

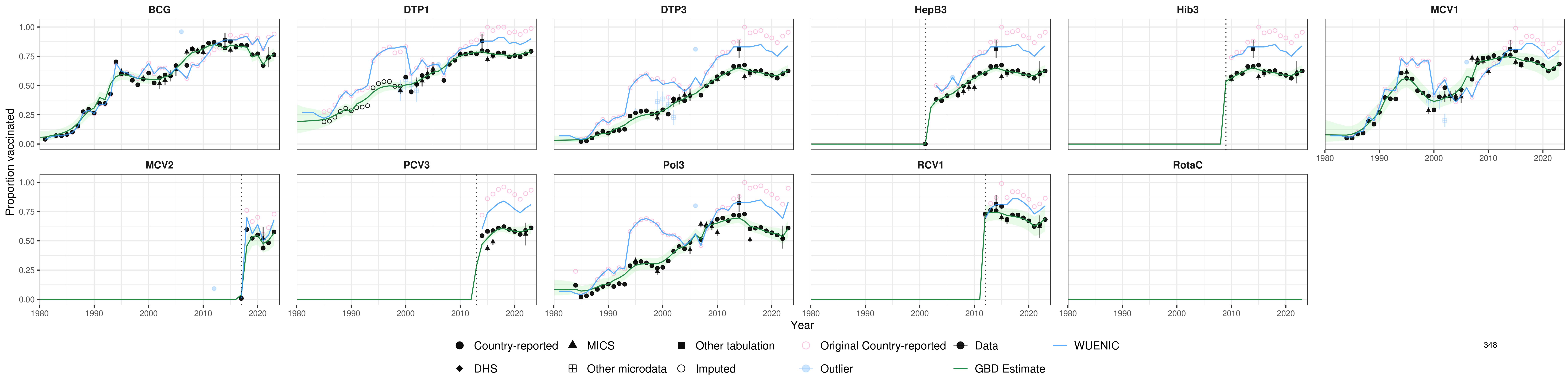
BCG
DTP1
DTP3
HepB3
Hib3
MCV1
MCV2
PCV3
Pol3
RCV1
RotaC
Proportion vaccinated
Year
Country-reported
MICS
Other tabulation
Original Country-reported
Data
WUENIC
DHS
Other microdata
Imputed
Outlier
GBD Estimate

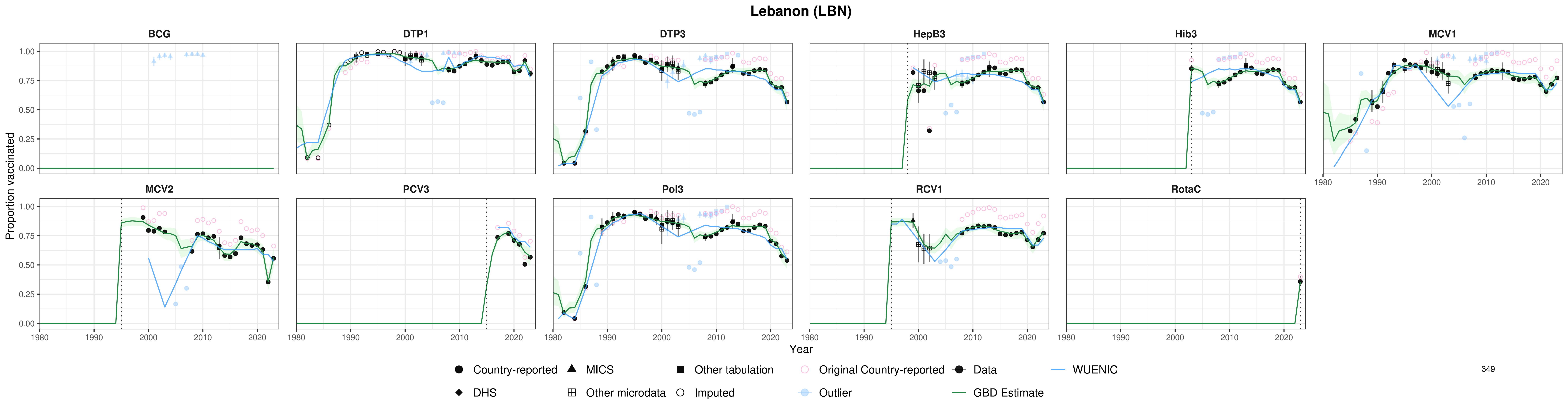

Lebanon (LBN)
BCG
DTP1
DTP3
HepB3
Hib3
MCV1
MCV2
PCV3
Pol3
RCV1
RotaC
Proportion vaccinated
Year
Country-reported
MICS
Other tabulation
Original Country-reported
Data
WUENIC
DHS
Other microdata
Imputed
Outlier
GBD Estimate

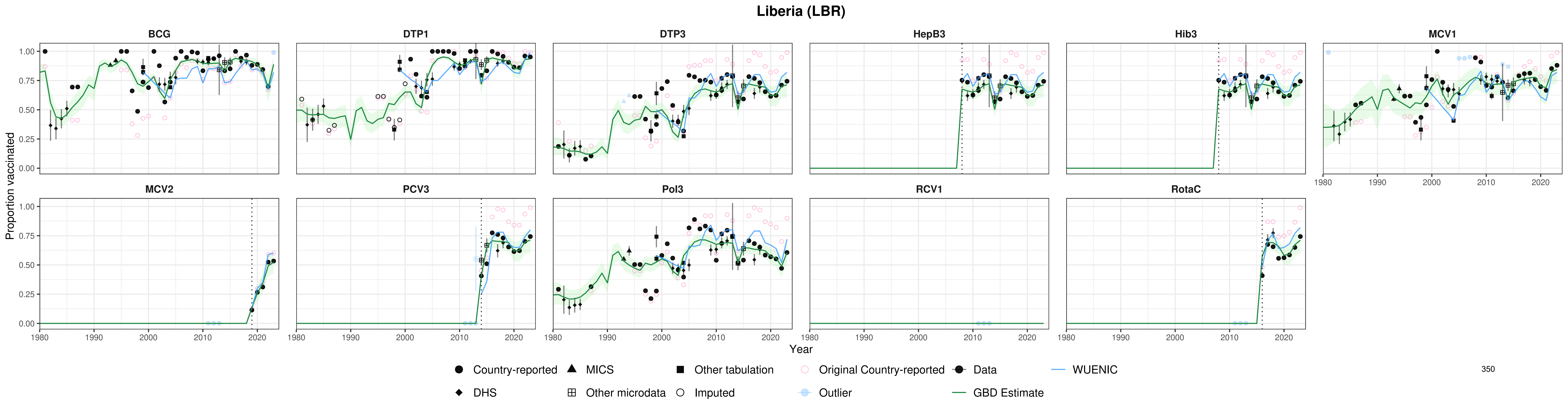

Liberia (LBR)
BCG
DTP1
DTP3
HepB3
Hib3
MCV1
MCV2
PCV3
Pol3
RCV1
RotaC
Proportion vaccinated
Year
Country-reported
MICS
Other tabulation
Original Country-reported
Data
WUENIC
DHS
Other microdata
Imputed
Outlier
GBD Estimate

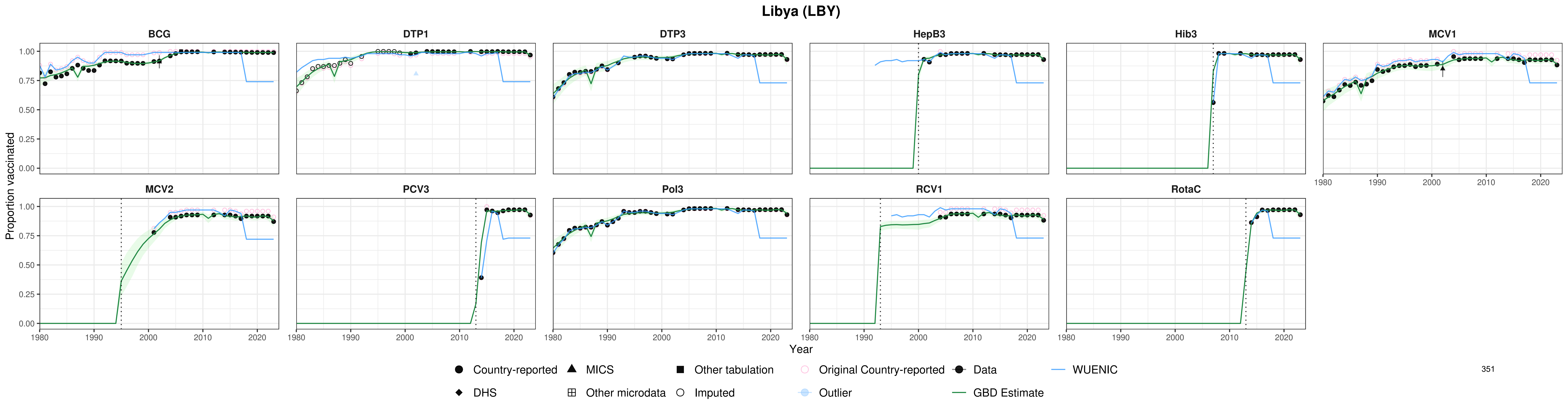
Libya (LBY)
BCG
DTP1
DTP3
HepB3
Hib3
MCV1
MCV2
PCV3
Pol3
RCV1
RotaC
Proportion vaccinated
Year
Country-reported
MICS
Other tabulation
Original Country-reported
Data
WUENIC
DHS
Other microdata
Imputed
Outlier
GBD Estimate

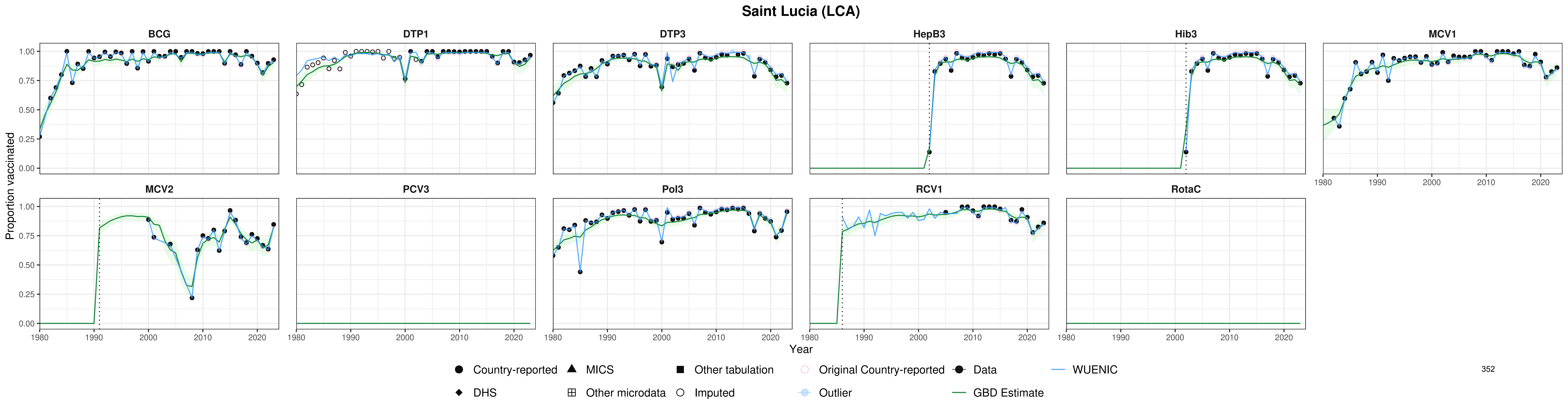

Saint Lucia (LCA)
BCG
DTP1
DTP3
HepB3
Hib3
MCV1
MCV2
PCV3
Pol3
RCV1
RotaC
Proportion vaccinated
0.00
0.25
0.50
0.75
1.00
1980
1990
2000
2010
2020
Year
Country-reported
MICS
Other tabulation
Original Country-reported
Data
WUENIC
DHS
Other microdata
Imputed
Outlier
GBD Estimate

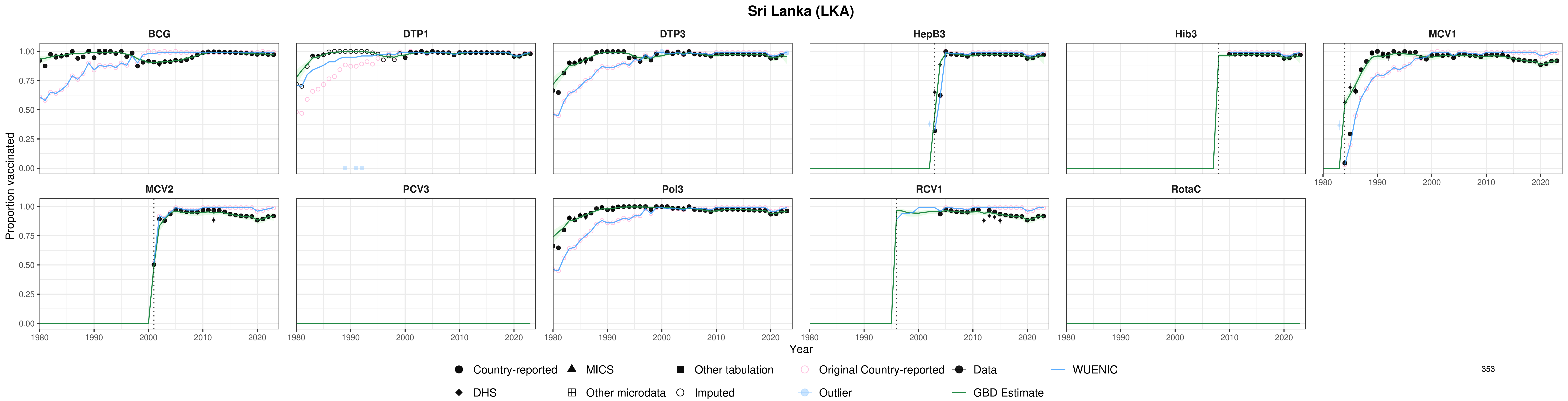

Sri Lanka (LKA)
BCG
DTP1
DTP3
HepB3
Hib3
MCV1
MCV2
PCV3
Pol3
RCV1
RotaC
Proportion vaccinated
Year
Country-reported
MICS
Other tabulation
Original Country-reported
Data
WUENIC
DHS
Other microdata
Imputed
Outlier
GBD Estimate

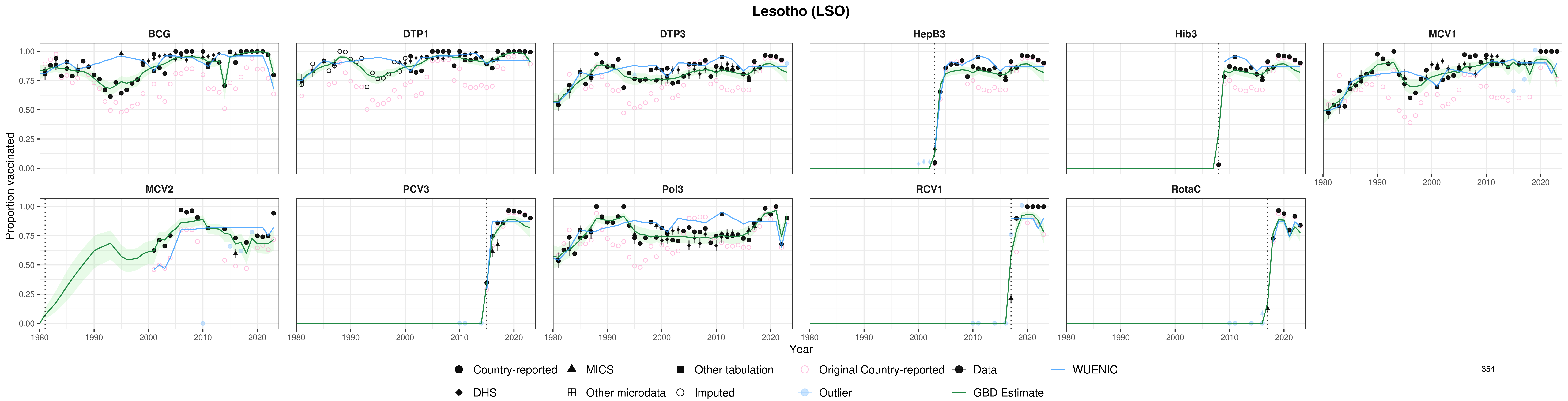

Lesotho (LSO)
BCG
DTP1
DTP3
HepB3
Hib3
MCV1
MCV2
PCV3
Pol3
RCV1
RotaC
Proportion vaccinated
Year
Country-reported
MICS
Other tabulation
Original Country-reported
Data
WUENIC
DHS
Other microdata
Imputed
Outlier
GBD Estimate

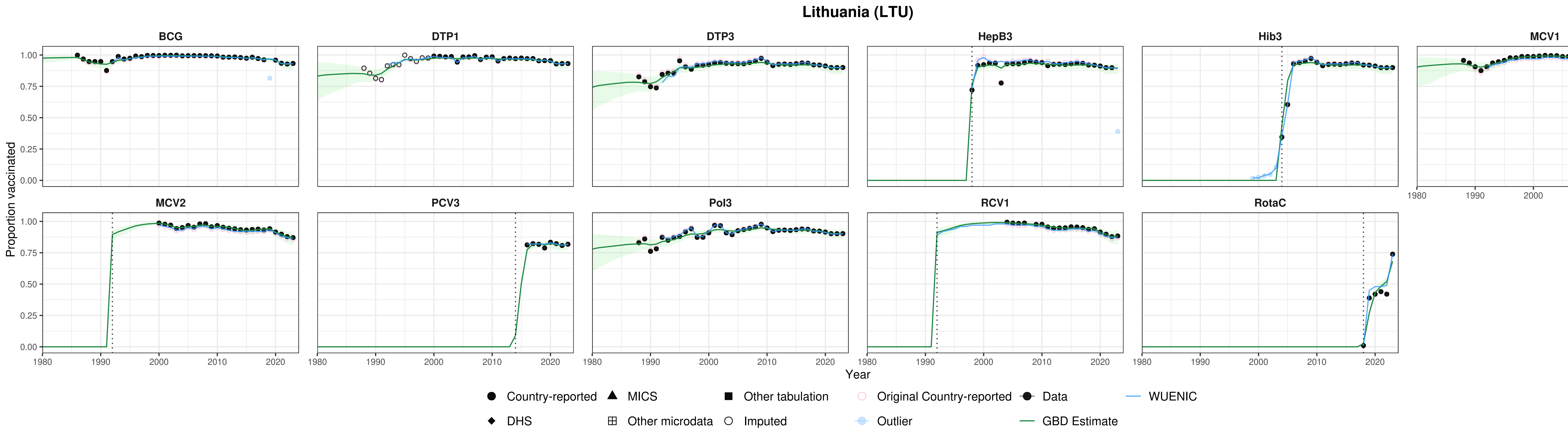
Lithuania (LTU)
BCG
DTP1
DTP3
HepB3
Hib3
MCV1
MCV2
PCV3
Pol3
RCV1
RotaC
Proportion vaccinated
Year
Country-reported
MICS
Other tabulation
Original Country-reported
Data
WUENIC
DHS
Other microdata
Imputed
Outlier
GBD Estimate

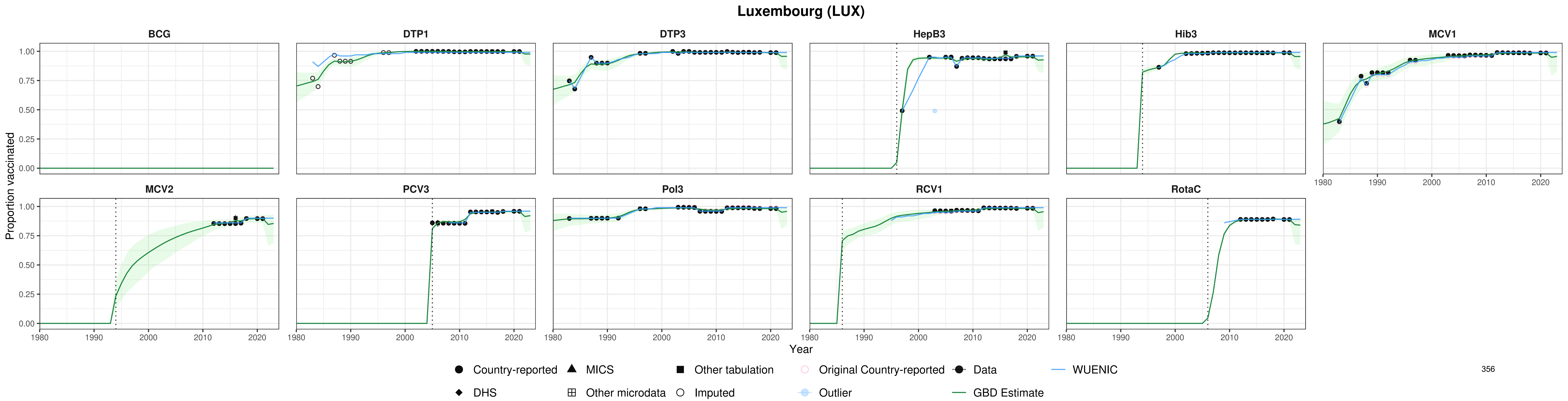
Luxembourg (LUX)
BCG
DTP1
DTP3
HepB3
Hib3
MCV1
MCV2
PCV3
Pol3
RCV1
RotaC
Proportion vaccinated
Year
1.00
0.75
0.50
0.25
0.00
1980
1990
2000
2010
2020
Country-reported
MICS
Other tabulation
Original Country-reported
Data
WUENIC
DHS
Other microdata
Imputed
Outlier
GBD Estimate

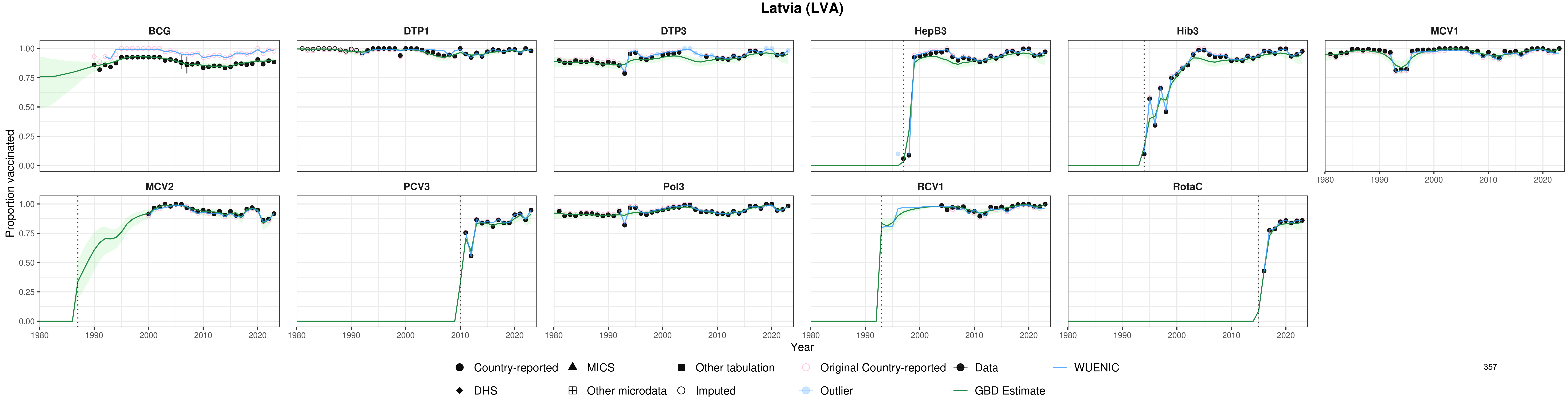

Latvia (LVA)
BCG
DTP1
DTP3
HepB3
Hib3
MCV1
MCV2
PCV3
Pol3
RCV1
RotaC
Proportion vaccinated
Year
Country-reported
MICS
Other tabulation
Original Country-reported
Data
WUENIC
DHS
Other microdata
Imputed
Outlier
GBD Estimate

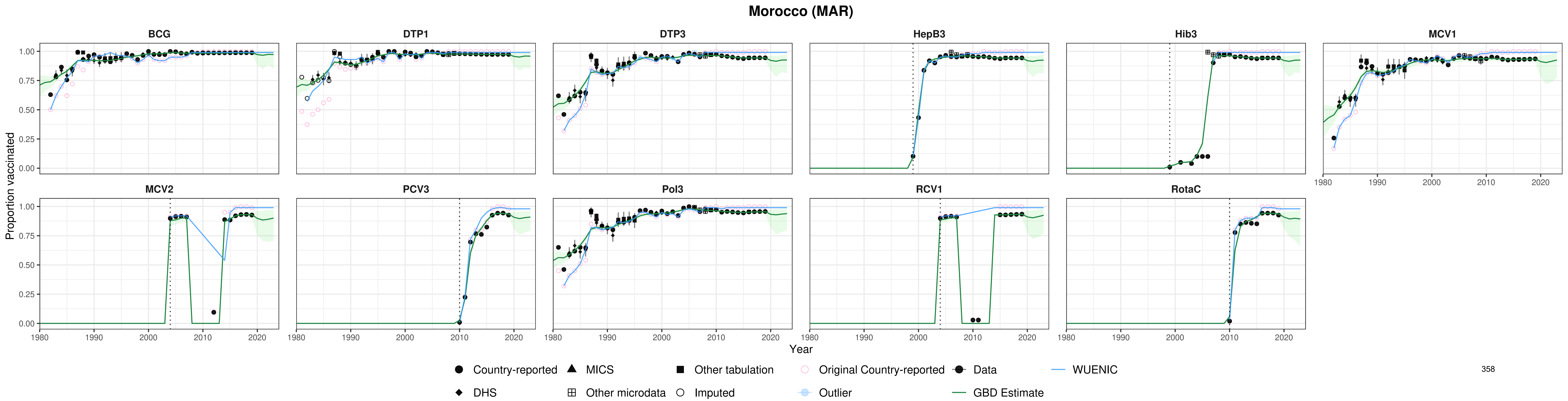

Morocco (MAR)
BCG
DTP1
DTP3
HepB3
Hib3
MCV1
MCV2
PCV3
Pol3
RCV1
RotaC
Proportion vaccinated
Year
Country-reported
MICS
Other tabulation
Original Country-reported
Data
WUENIC
DHS
Other microdata
Imputed
Outlier
GBD Estimate

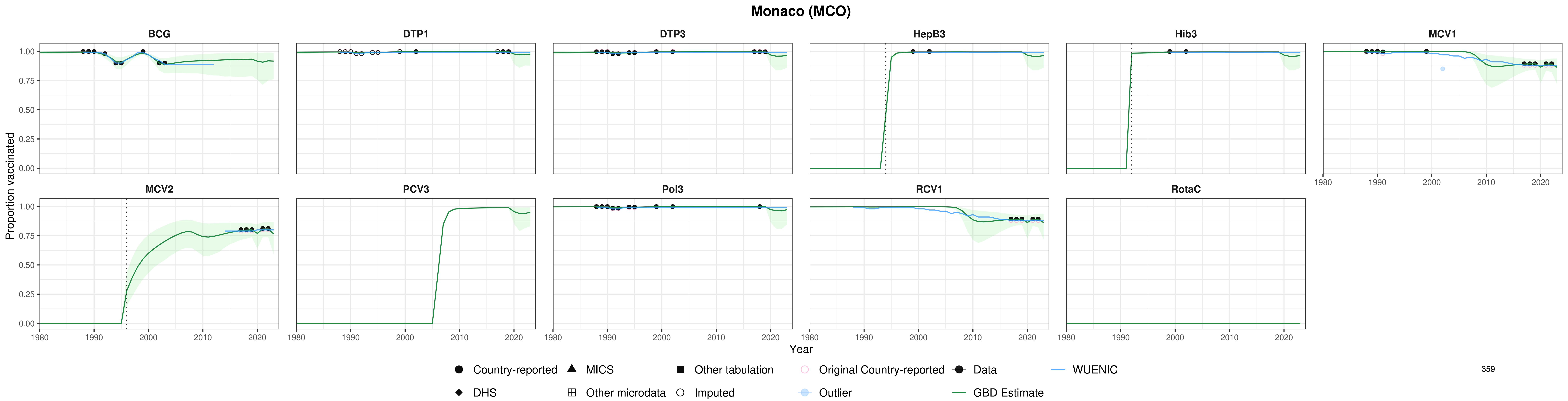
Monaco (MCO)
BCG
DTP1
DTP3
HepB3
Hib3
MCV1
MCV2
PCV3
Pol3
RCV1
RotaC
Proportion vaccinated
Year
Country-reported
MICS
Other tabulation
Original Country-reported
Data
WUENIC
DHS
Other microdata
Imputed
Outlier
GBD Estimate

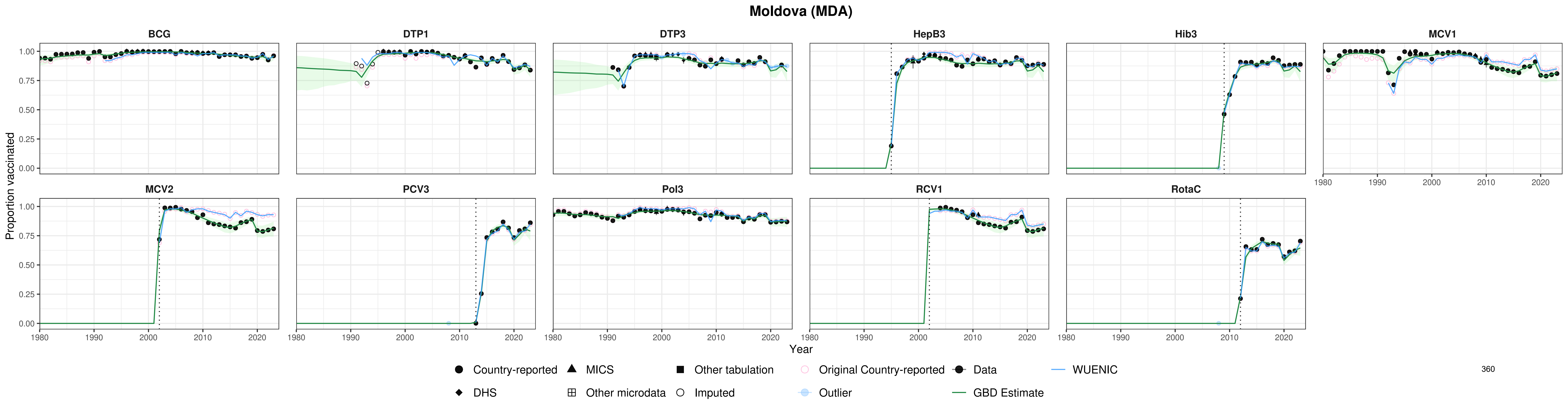
Moldova (MDA)
BCG
DTP1
DTP3
HepB3
Hib3
MCV1
MCV2
PCV3
Pol3
RCV1
RotaC
Proportion vaccinated
Year
Country-reported
MICS
Other tabulation
Original Country-reported
Data
WUENIC
DHS
Other microdata
Imputed
Outlier
GBD Estimate

Madagascar (MDG)

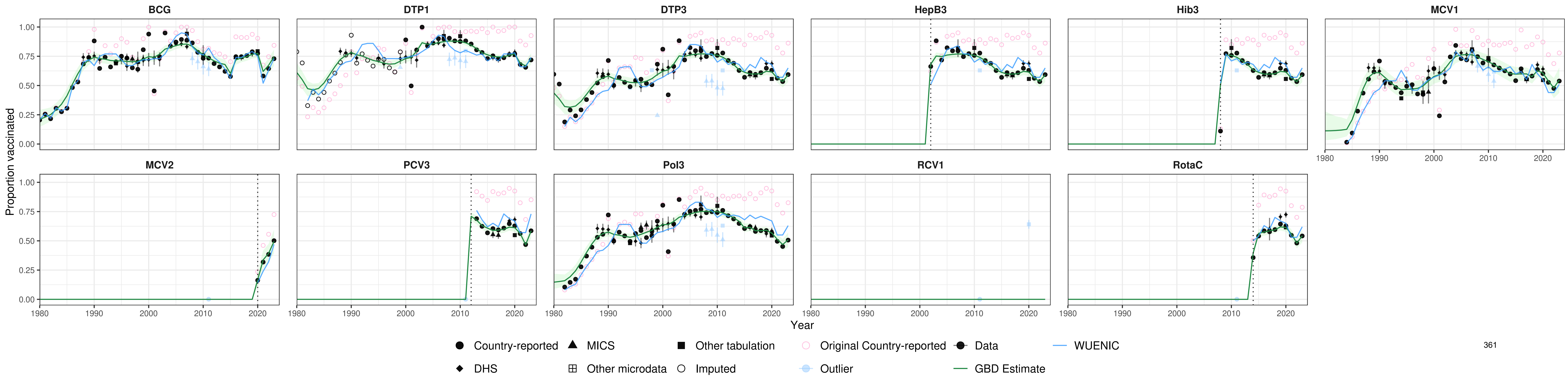

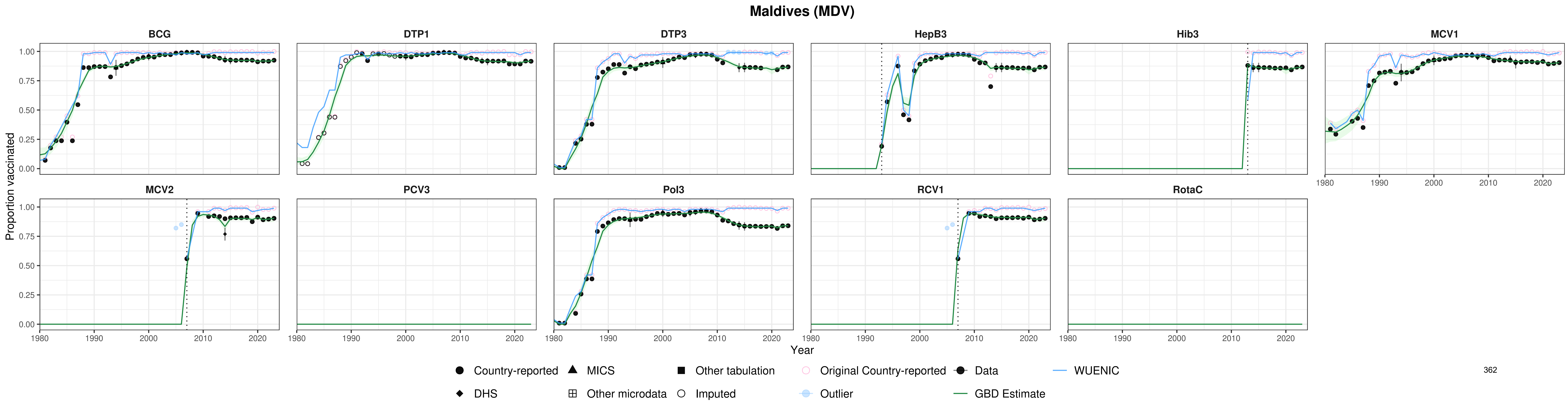

Maldives (MDV)
BCG
DTP1
DTP3
HepB3
Hib3
MCV1
MCV2
PCV3
Pol3
RCV1
RotaC
Proportion vaccinated
Year
Country-reported
MICS
Other tabulation
Original Country-reported
Data
WUENIC
DHS
Other microdata
Imputed
Outlier
GBD Estimate

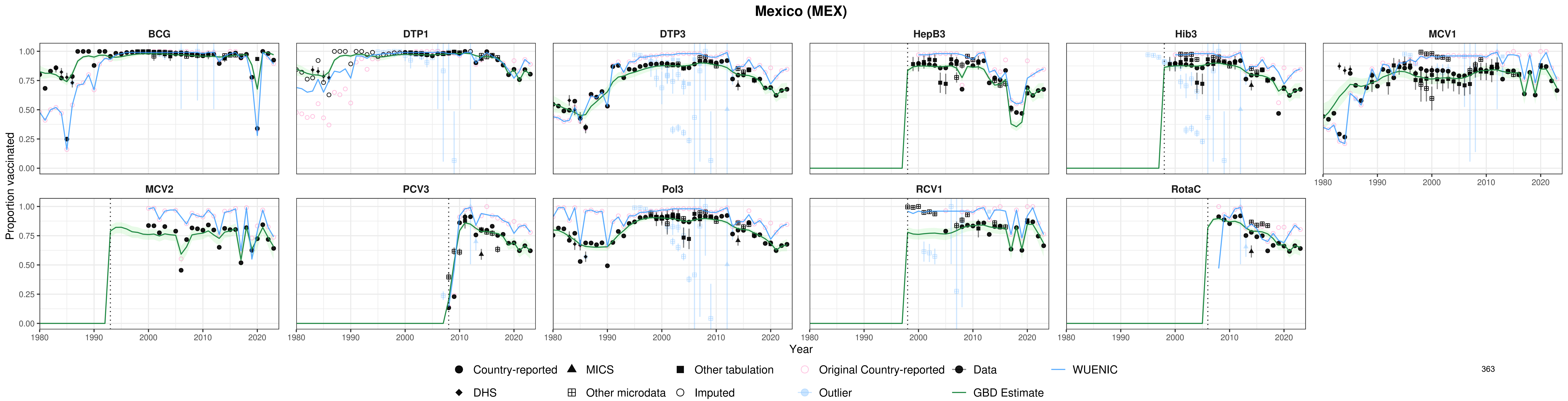

Mexico (MEX)
BCG
DTP1
DTP3
HepB3
Hib3
MCV1
MCV2
PCV3
Pol3
RCV1
RotaC
Proportion vaccinated
Year
0.00
0.25
0.50
0.75
1.00
1980
1990
2000
2010
2020
Country-reported
MICS
Other tabulation
Original Country-reported
Data
WUENIC
DHS
Other microdata
Imputed
Outlier
GBD Estimate

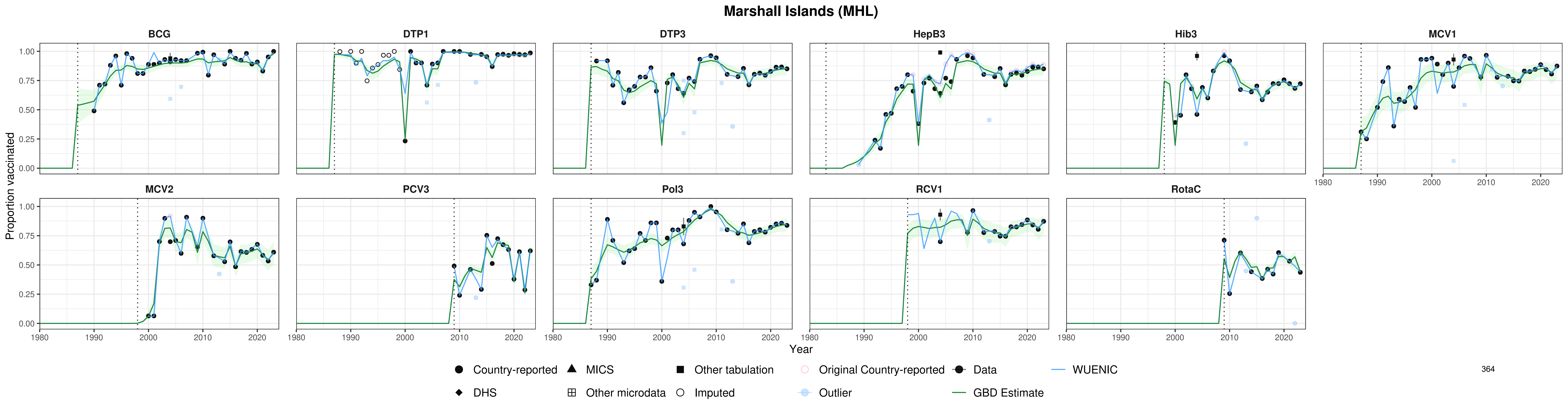

Marshall Islands (MHL)
BCG
DTP1
DTP3
HepB3
Hib3
MCV1
MCV2
PCV3
Pol3
RCV1
RotaC
Proportion vaccinated
Year
Country-reported
MICS
Other tabulation
Original Country-reported
Data
WUENIC
DHS
Other microdata
Imputed
Outlier
GBD Estimate

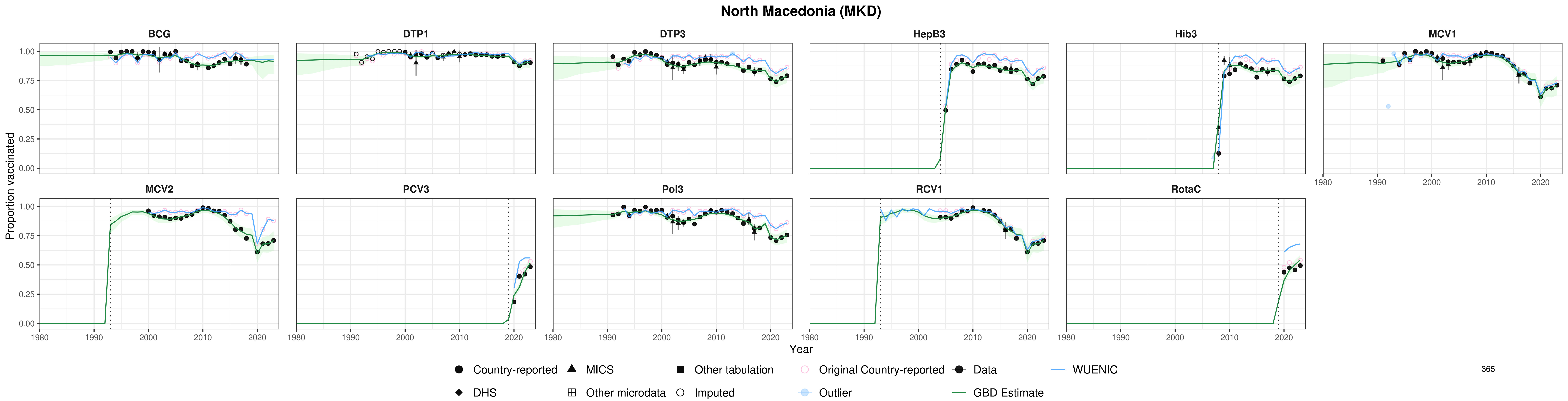

North Macedonia (MKD)
BCG
DTP1
DTP3
HepB3
Hib3
MCV1
MCV2
PCV3
Pol3
RCV1
RotaC
Proportion vaccinated
Year
Country-reported
MICS
Other tabulation
Original Country-reported
Data
WUENIC
DHS
Other microdata
Imputed
Outlier
GBD Estimate

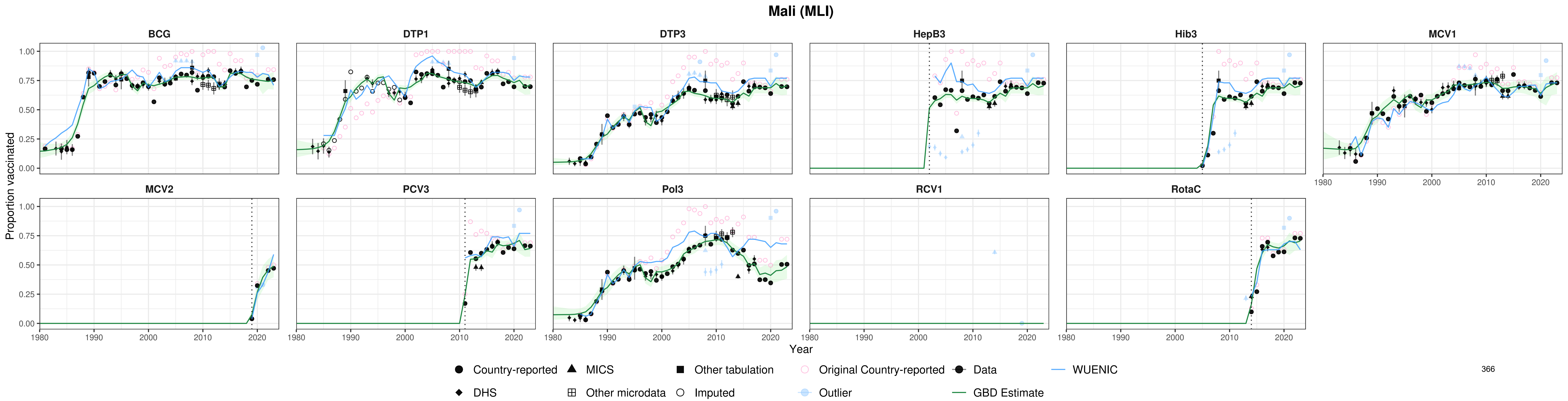

Mali (MLI)
BCG
DTP1
DTP3
HepB3
Hib3
MCV1
MCV2
PCV3
Pol3
RCV1
RotaC
Proportion vaccinated
Year
0.00
0.25
0.50
0.75
1.00
1980
1990
2000
2010
2020
Country-reported
DHS
MICS
Other microdata
Other tabulation
Imputed
Original Country-reported
Outlier
Data
GBD Estimate
WUENIC

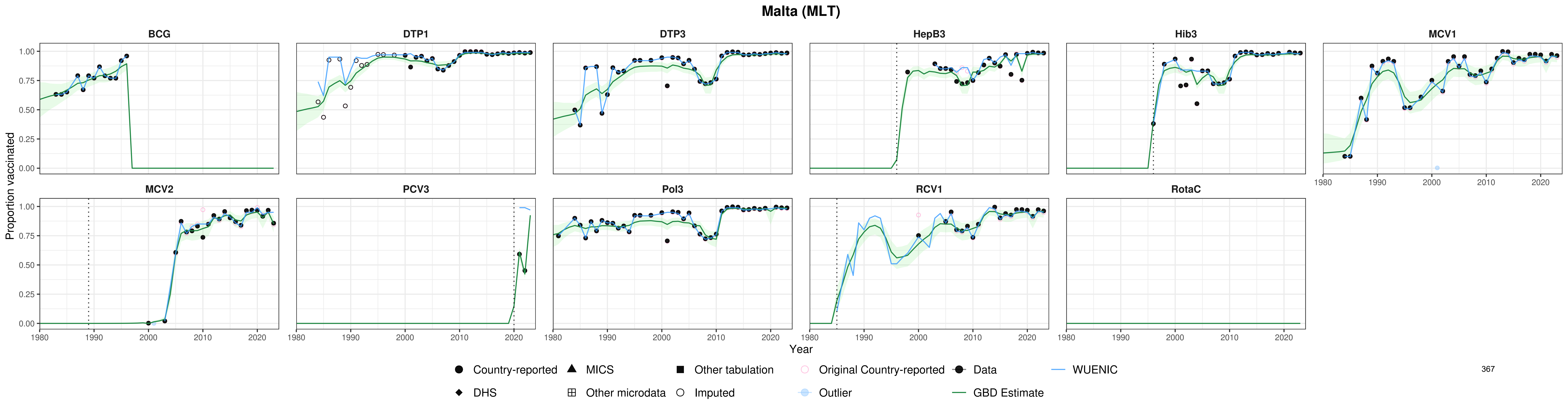
Malta (MLT)
BCG
DTP1
DTP3
HepB3
Hib3
MCV1
MCV2
PCV3
Pol3
RCV1
RotaC
Proportion vaccinated
Year
Country-reported
MICS
Other tabulation
Original Country-reported
Data
WUENIC
DHS
Other microdata
Imputed
Outlier
GBD Estimate

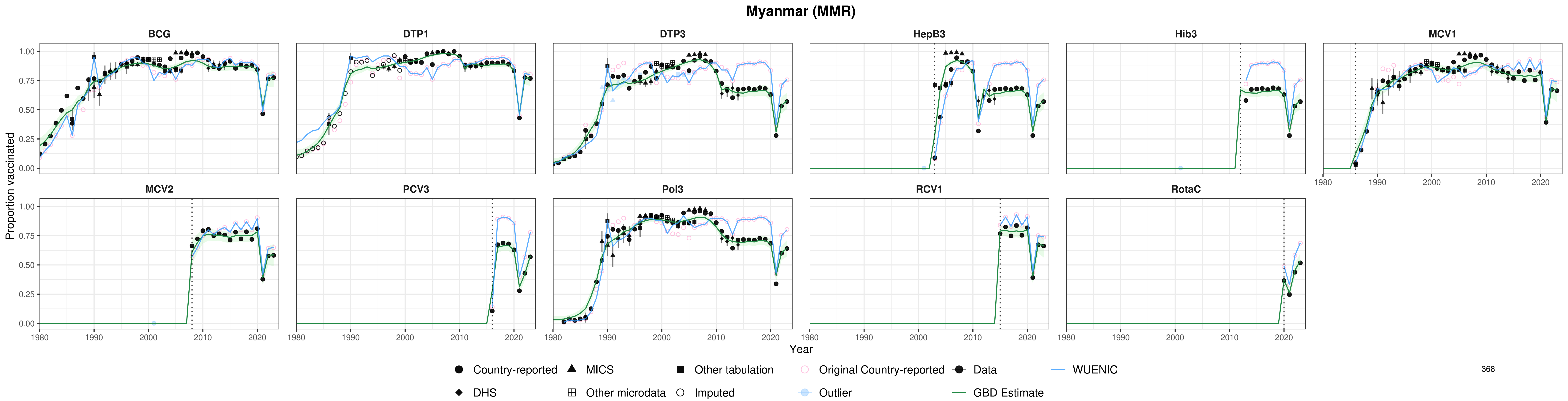

Myanmar (MMR)
BCG
DTP1
DTP3
HepB3
Hib3
MCV1
MCV2
PCV3
Pol3
RCV1
RotaC
Proportion vaccinated
Year
0.00
0.25
0.50
0.75
1.00
1980
1990
2000
2010
2020
Country-reported
MICS
Other tabulation
Original Country-reported
Data
WUENIC
DHS
Other microdata
Imputed
Outlier
GBD Estimate

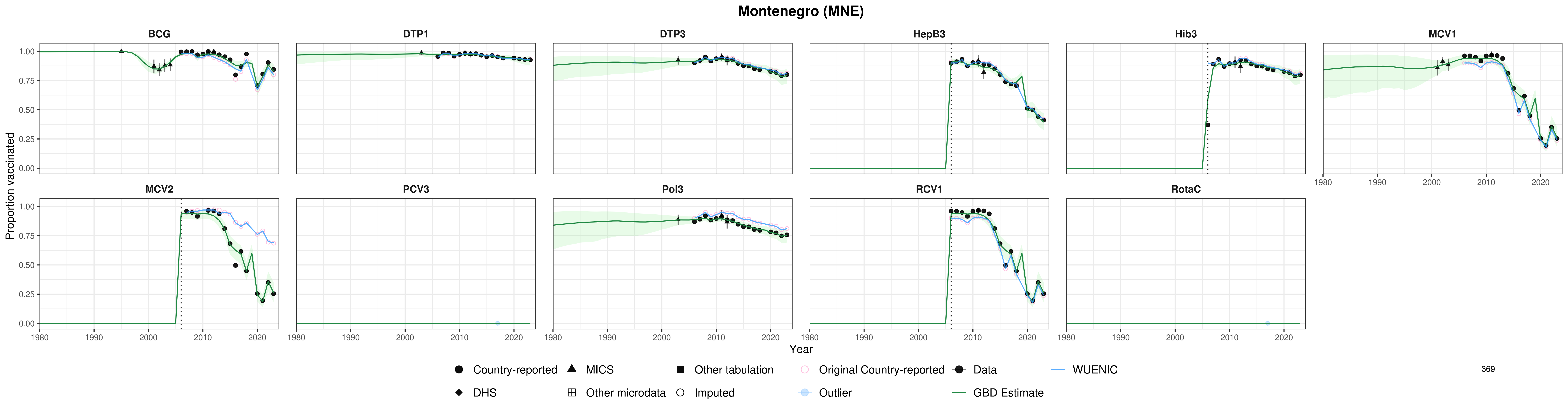

Montenegro (MNE)
BCG
DTP1
DTP3
HepB3
Hib3
MCV1
MCV2
PCV3
Pol3
RCV1
RotaC
Proportion vaccinated
Year
1.00
0.75
0.50
0.25
0.00
1980
1990
2000
2010
2020
Country-reported
MICS
Other tabulation
Original Country-reported
Data
WUENIC
DHS
Other microdata
Imputed
Outlier
GBD Estimate

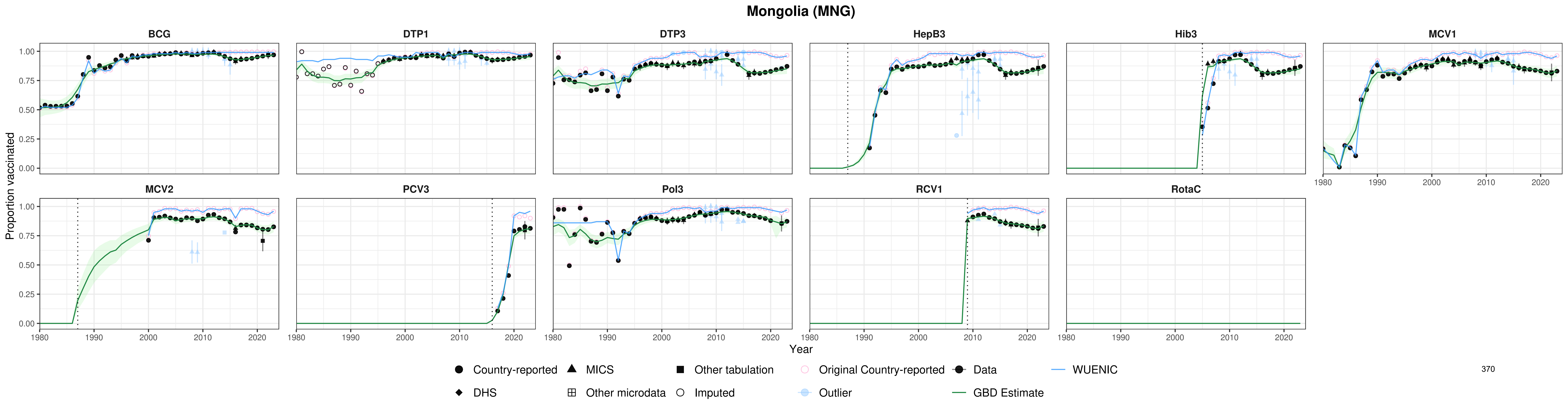

Mongolia (MNG)
BCG
DTP1
DTP3
HepB3
Hib3
MCV1
MCV2
PCV3
Pol3
RCV1
RotaC
Proportion vaccinated
Year
Country-reported
MICS
Other tabulation
Original Country-reported
Data
WUENIC
DHS
Other microdata
Imputed
Outlier
GBD Estimate

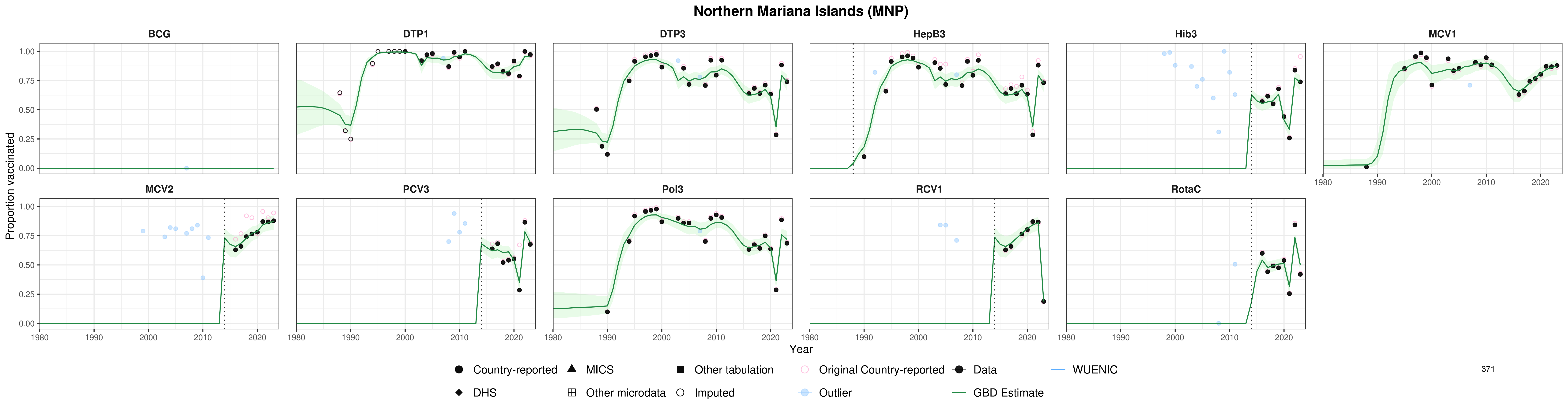
Northern Mariana Islands (MNP)
BCG
DTP1
DTP3
HepB3
Hib3
MCV1
MCV2
PCV3
Pol3
RCV1
RotaC
Proportion vaccinated
Year
Country-reported
MICS
Other tabulation
Original Country-reported
Data
WUENIC
DHS
Other microdata
Imputed
Outlier
GBD Estimate

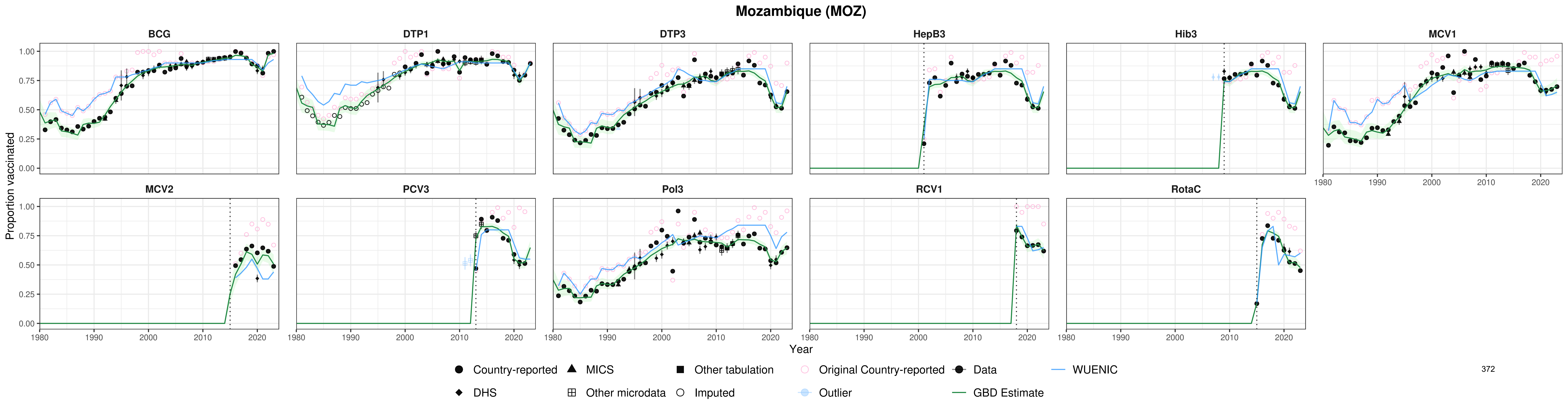

Mozambique (MOZ)
BCG
DTP1
DTP3
HepB3
Hib3
MCV1
MCV2
PCV3
Pol3
RCV1
RotaC
Proportion vaccinated
0.00
0.25
0.50
0.75
1.00
1980
1990
2000
2010
2020
Year
Country-reported
MICS
Other tabulation
Original Country-reported
Data
WUENIC
DHS
Other microdata
Imputed
Outlier
GBD Estimate

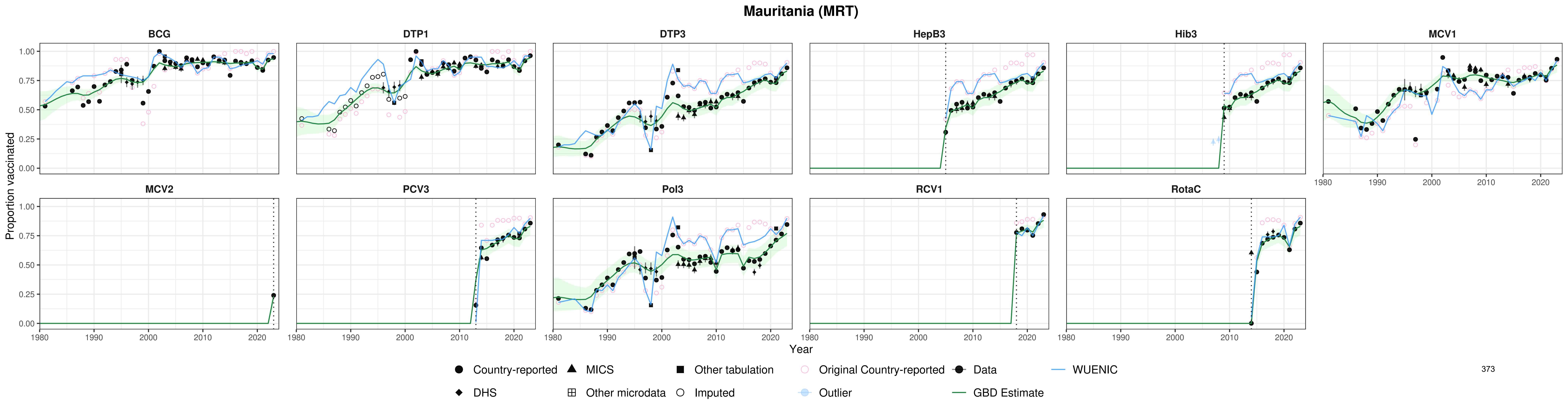
Mauritania (MRT)
BCG
DTP1
DTP3
HepB3
Hib3
MCV1
MCV2
PCV3
Pol3
RCV1
RotaC
Proportion vaccinated
Year
Country-reported
MICS
Other tabulation
Original Country-reported
Data
WUENIC
DHS
Other microdata
Imputed
Outlier
GBD Estimate

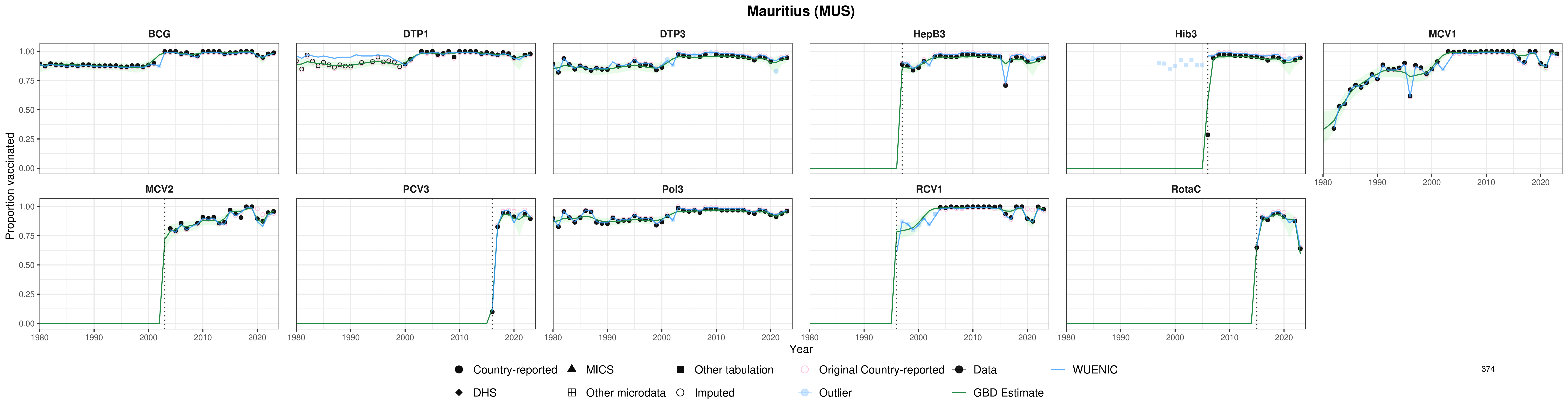

Mauritius (MUS)
BCG
DTP1
DTP3
HepB3
Hib3
MCV1
MCV2
PCV3
Pol3
RCV1
RotaC
Proportion vaccinated
Year
Country-reported
MICS
Other tabulation
Original Country-reported
Data
WUENIC
DHS
Other microdata
Imputed
Outlier
GBD Estimate

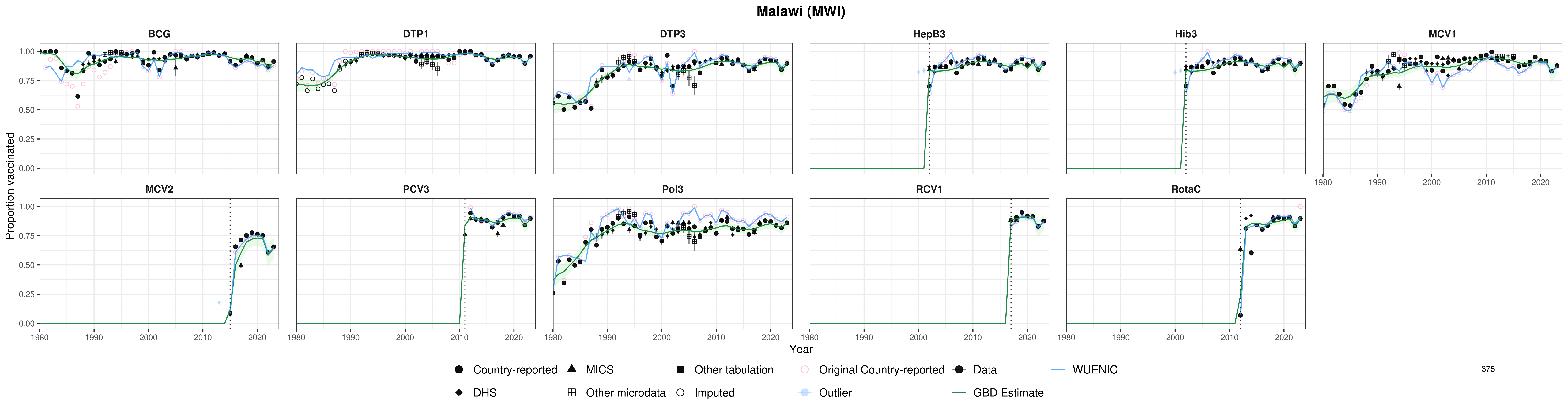
Malawi (MWI)
BCG
DTP1
DTP3
HepB3
Hib3
MCV1
MCV2
PCV3
Pol3
RCV1
RotaC
Proportion vaccinated
Year
1.00
0.75
0.50
0.25
0.00
1980
1990
2000
2010
2020
Country-reported
MICS
Other tabulation
Original Country-reported
Data
WUENIC
DHS
Other microdata
Imputed
Outlier
GBD Estimate

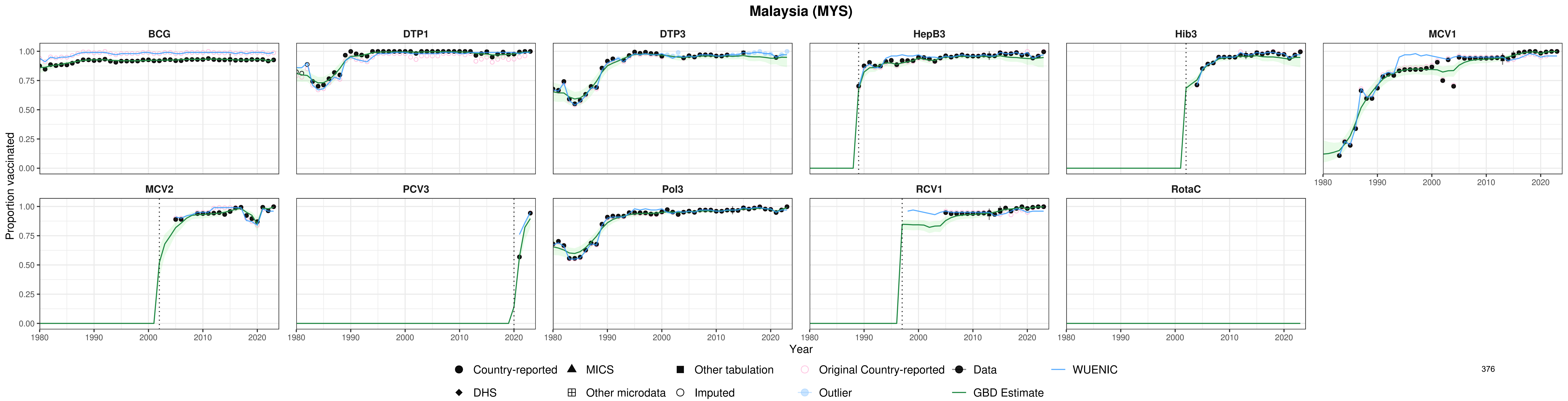

Malaysia (MYS)
BCG
DTP1
DTP3
HepB3
Hib3
MCV1
MCV2
PCV3
Pol3
RCV1
RotaC
Proportion vaccinated
Year
0.00
0.25
0.50
0.75
1.00
1980
1990
2000
2010
2020
Country-reported
MICS
Other tabulation
Original Country-reported
Data
WUENIC
DHS
Other microdata
Imputed
Outlier
GBD Estimate

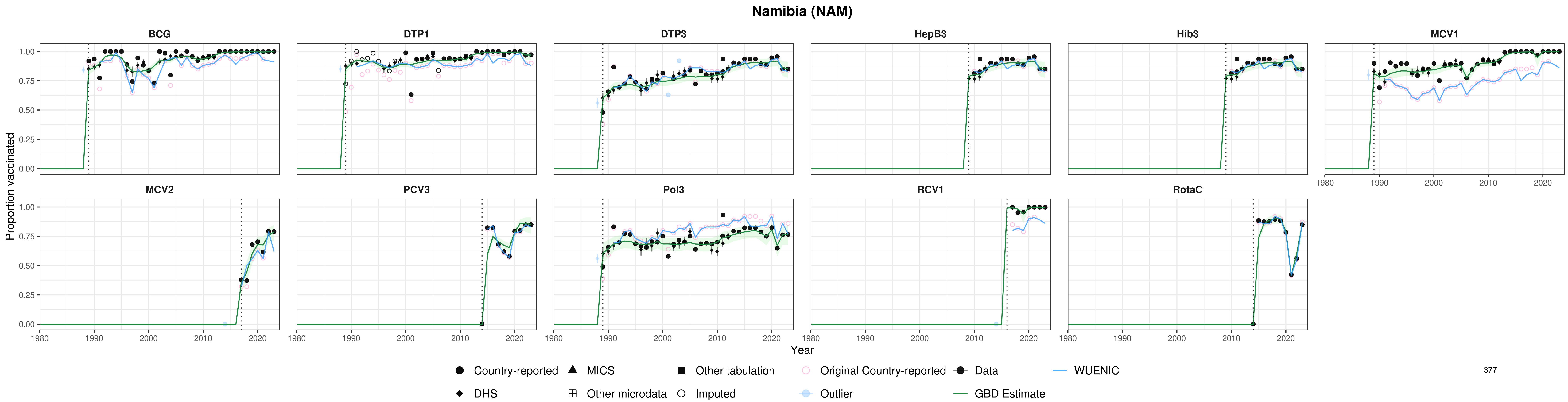

Namibia (NAM)
BCG
DTP1
DTP3
HepB3
Hib3
MCV1
MCV2
PCV3
Pol3
RCV1
RotaC
Proportion vaccinated
Year
Country-reported
MICS
Other tabulation
Original Country-reported
Data
WUENIC
DHS
Other microdata
Imputed
Outlier
GBD Estimate

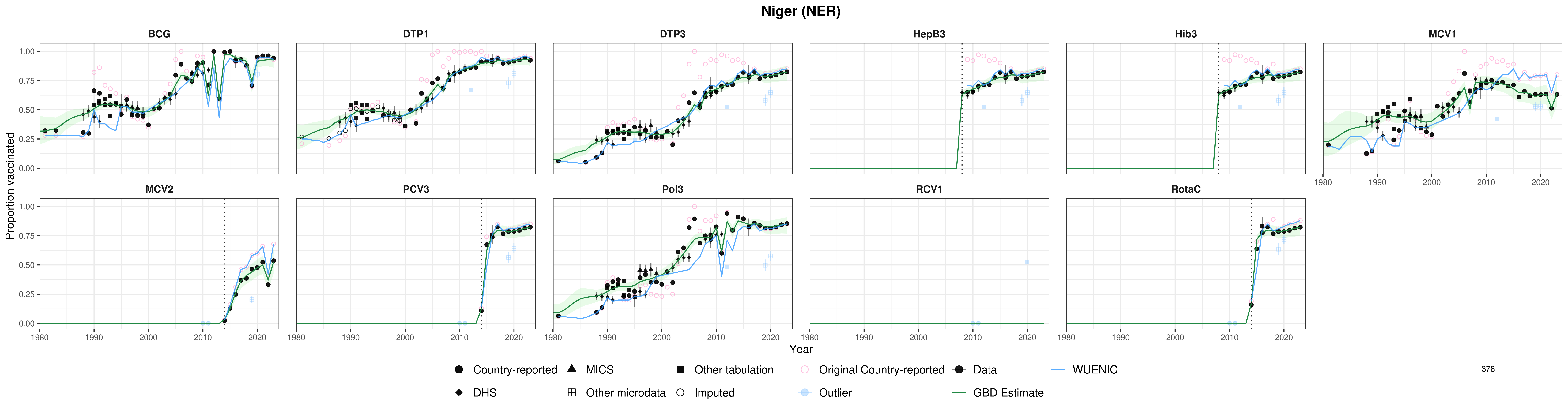
Niger (NER)
BCG
DTP1
DTP3
HepB3
Hib3
MCV1
MCV2
PCV3
Pol3
RCV1
RotaC
Proportion vaccinated
Year
0.00
0.25
0.50
0.75
1.00
1980
1990
2000
2010
2020
Country-reported
MICS
Other tabulation
Original Country-reported
Data
WUENIC
DHS
Other microdata
Imputed
Outlier
GBD Estimate

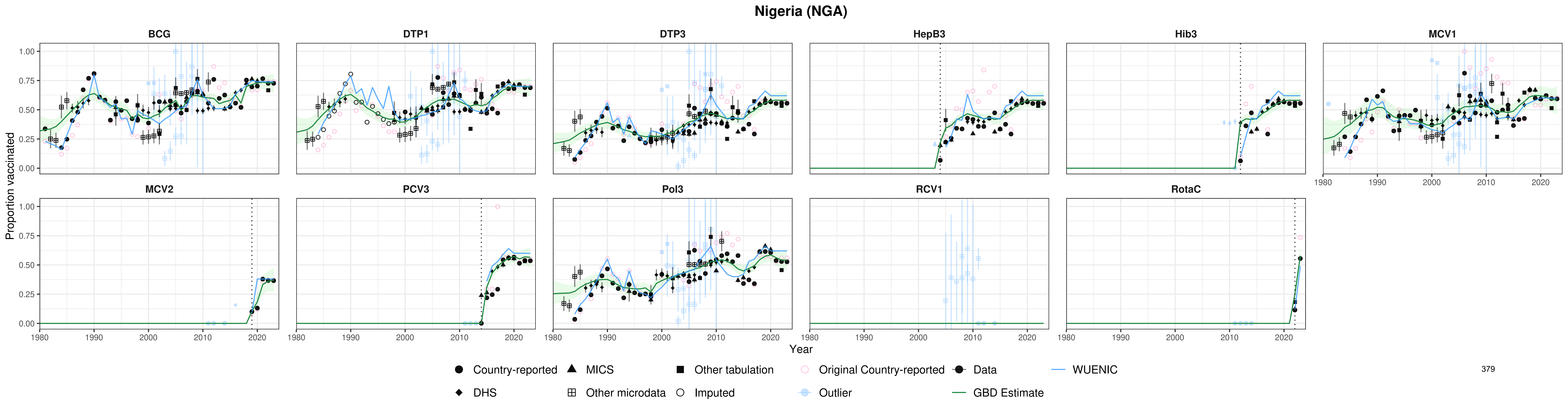
Nigeria (NGA)
BCG
DTP1
DTP3
HepB3
Hib3
MCV1
MCV2
PCV3
Pol3
RCV1
RotaC
Proportion vaccinated
Year
0.00
0.25
0.50
0.75
1.00
1980
1990
2000
2010
2020
Country-reported
MICS
Other tabulation
Original Country-reported
Data
WUENIC
DHS
Other microdata
Imputed
Outlier
GBD Estimate

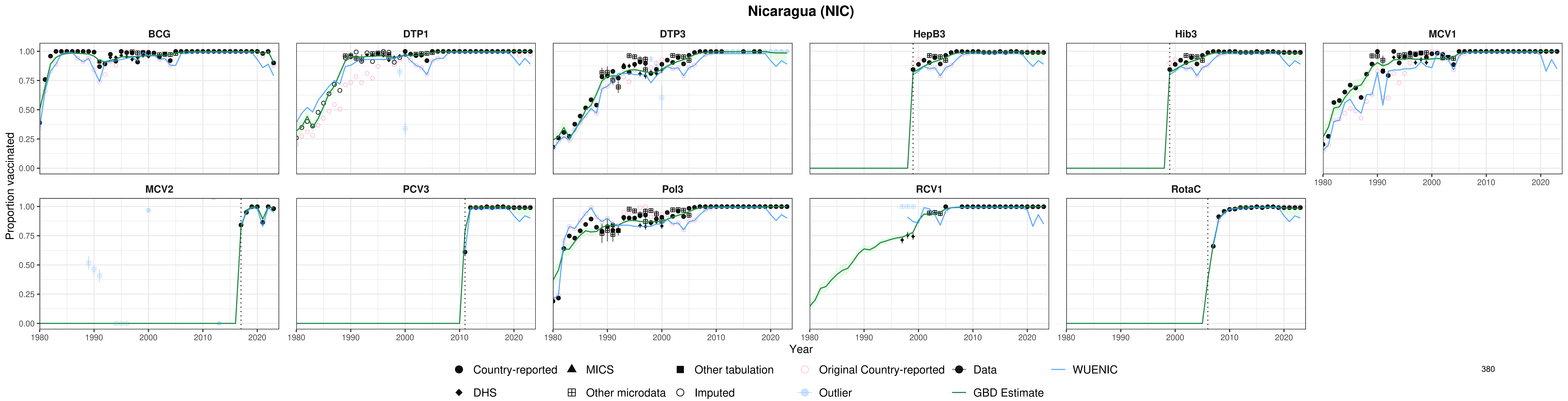
Nicaragua (NIC)
BCG
DTP1
DTP3
HepB3
Hib3
MCV1
MCV2
PCV3
Pol3
RCV1
RotaC
Proportion vaccinated
Year
Country-reported
MICS
Other tabulation
Original Country-reported
Data
WUENIC
DHS
Other microdata
Imputed
Outlier
GBD Estimate

Niue (NIU)

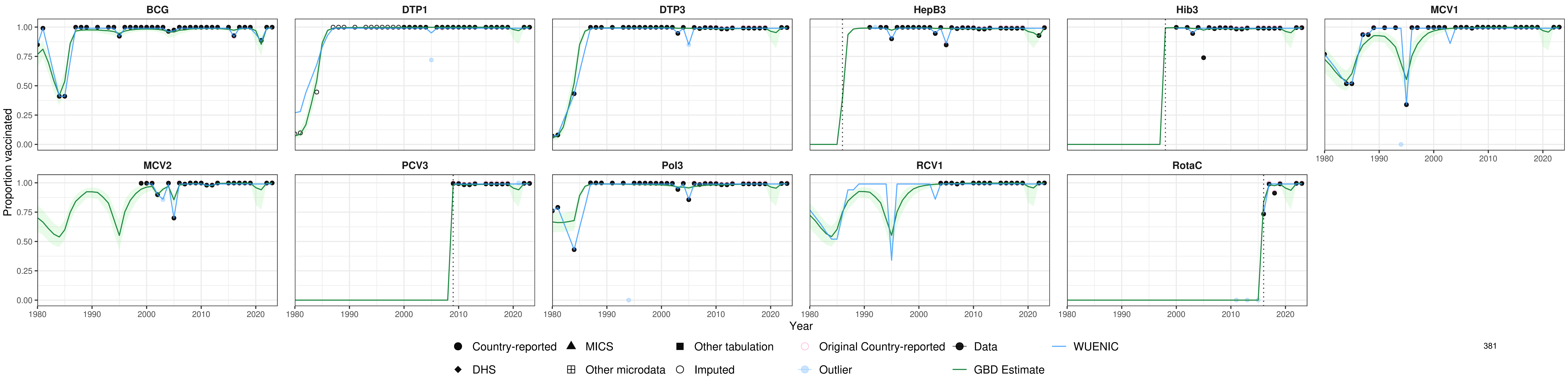

BCG
DTP1
DTP3
HepB3
Hib3
MCV1
MCV2
PCV3
Pol3
RCV1
RotaC
Proportion vaccinated
Year
1.00
0.75
0.50
0.25
0.00
1980
1990
2000
2010
2020
Country-reported
MICS
Other tabulation
Original Country-reported
Data
WUENIC
DHS
Other microdata
Imputed
Outlier
GBD Estimate

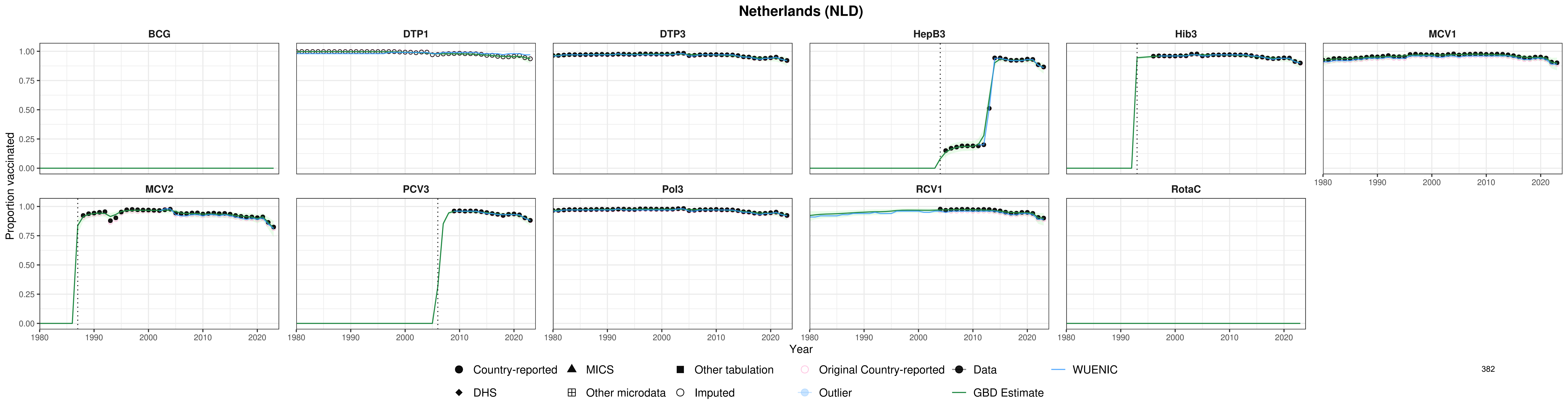

Netherlands (NLD)
BCG
DTP1
DTP3
HepB3
Hib3
MCV1
MCV2
PCV3
Pol3
RCV1
RotaC
Proportion vaccinated
0.00
0.25
0.50
0.75
1.00
1980
1990
2000
2010
2020
Year
Country-reported
MICS
Other tabulation
Original Country-reported
Data
WUENIC
DHS
Other microdata
Imputed
Outlier
GBD Estimate

Norway (NOR)

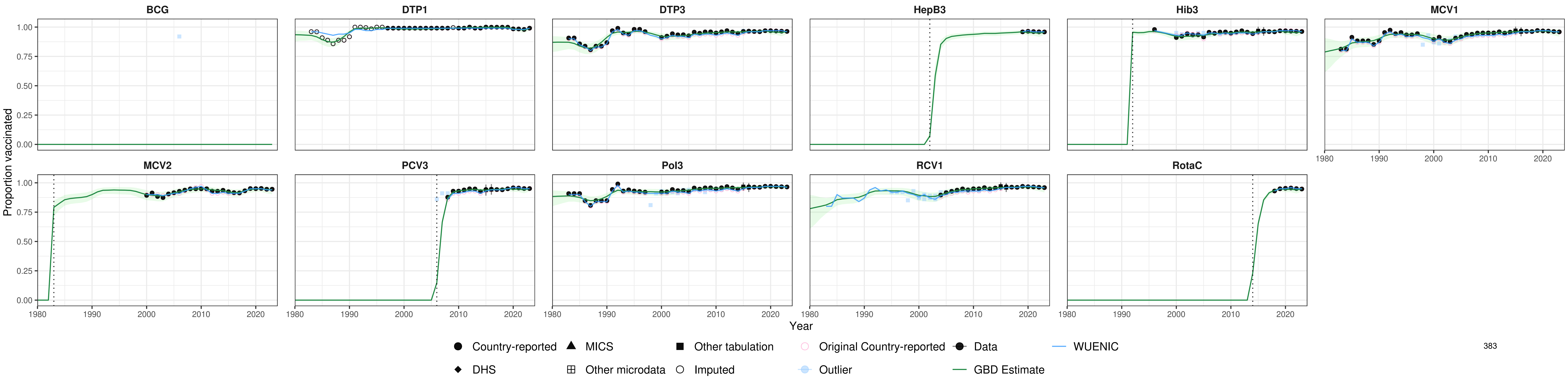

BCG
DTP1
DTP3
HepB3
Hib3
MCV1
MCV2
PCV3
Pol3
RCV1
RotaC
Proportion vaccinated
Year
Country-reported
MICS
Other tabulation
Original Country-reported
Data
WUENIC
DHS
Other microdata
Imputed
Outlier
GBD Estimate

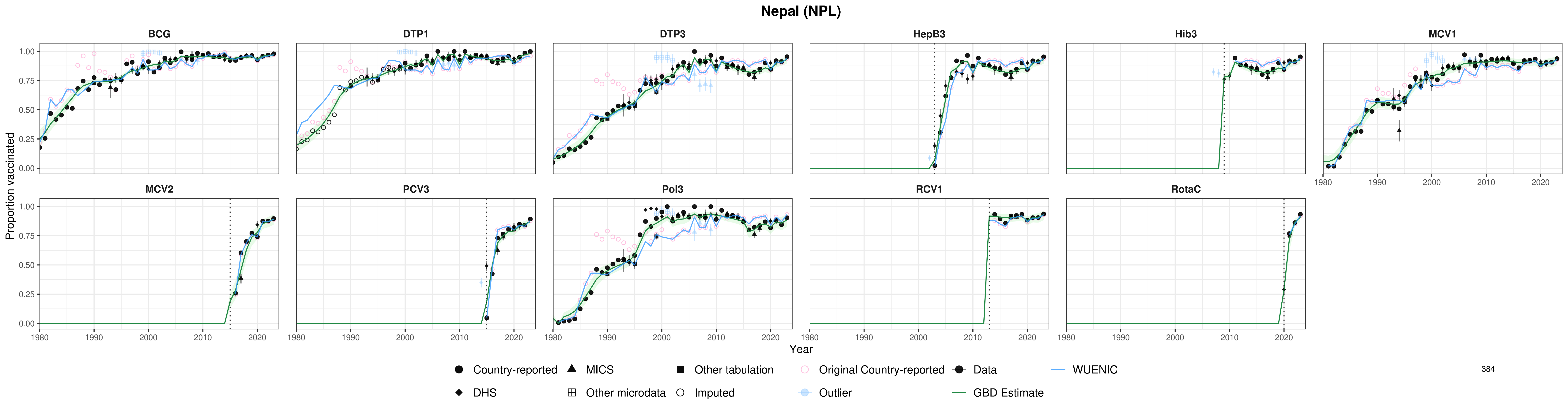
Nepal (NPL)
BCG
DTP1
DTP3
HepB3
Hib3
MCV1
MCV2
PCV3
Pol3
RCV1
RotaC
Proportion vaccinated
Year
Country-reported
MICS
Other tabulation
Original Country-reported
Data
WUENIC
DHS
Other microdata
Imputed
Outlier
GBD Estimate

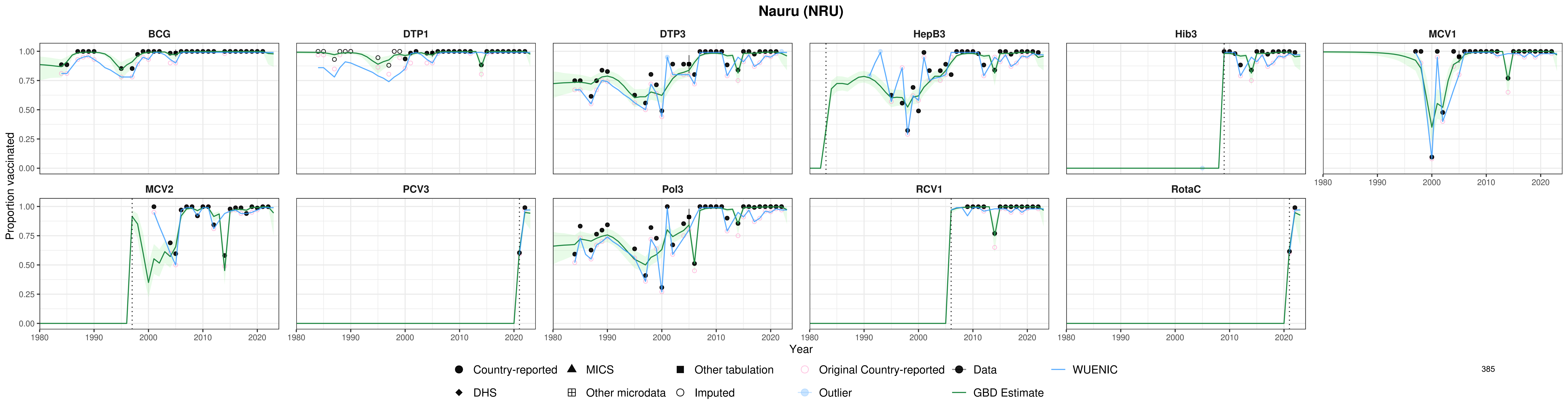

Nauru (NRU)
BCG
DTP1
DTP3
HepB3
Hib3
MCV1
MCV2
PCV3
Pol3
RCV1
RotaC
Proportion vaccinated
Year
0.00
0.25
0.50
0.75
1.00
1980
1990
2000
2010
2020
Country-reported
MICS
Other tabulation
Original Country-reported
Data
WUENIC
DHS
Other microdata
Imputed
Outlier
GBD Estimate

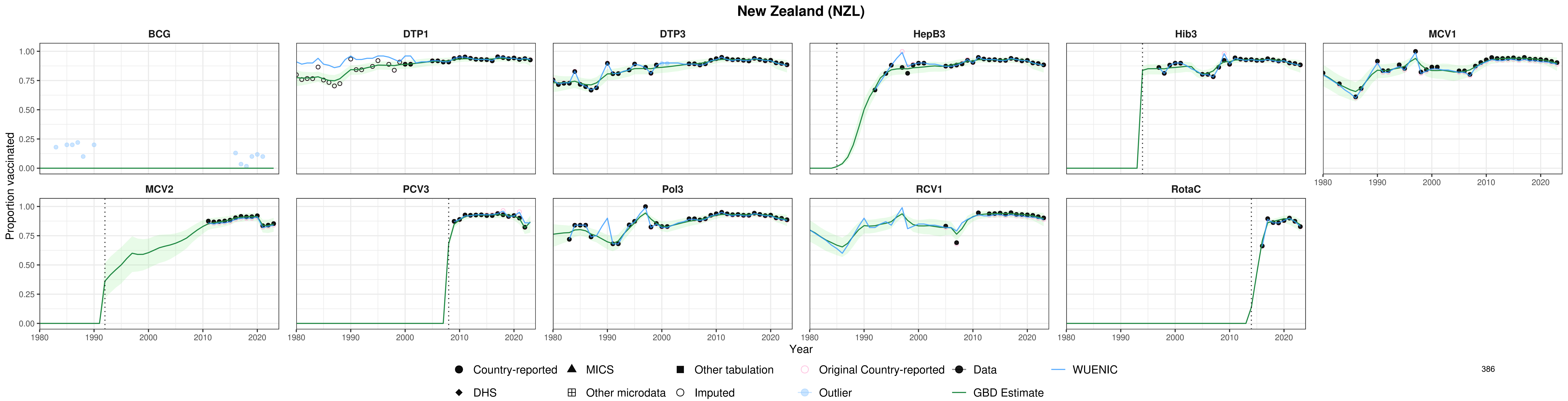

New Zealand (NZL)
BCG
DTP1
DTP3
HepB3
Hib3
MCV1
MCV2
PCV3
Pol3
RCV1
RotaC
Proportion vaccinated
Year
Country-reported
MICS
Other tabulation
Original Country-reported
Data
WUENIC
DHS
Other microdata
Imputed
Outlier
GBD Estimate

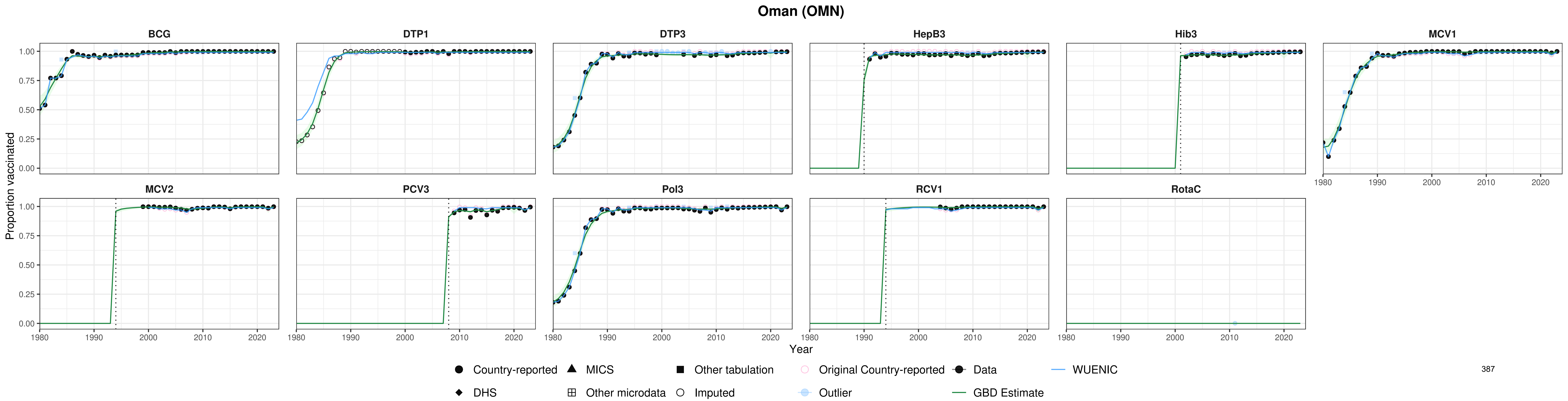

Oman (OMN)
BCG
DTP1
DTP3
HepB3
Hib3
MCV1
MCV2
PCV3
Pol3
RCV1
RotaC
Proportion vaccinated
Year
1.00
0.75
0.50
0.25
0.00
1980
1990
2000
2010
2020
Country-reported
MICS
Other tabulation
Original Country-reported
Data
WUENIC
DHS
Other microdata
Imputed
Outlier
GBD Estimate

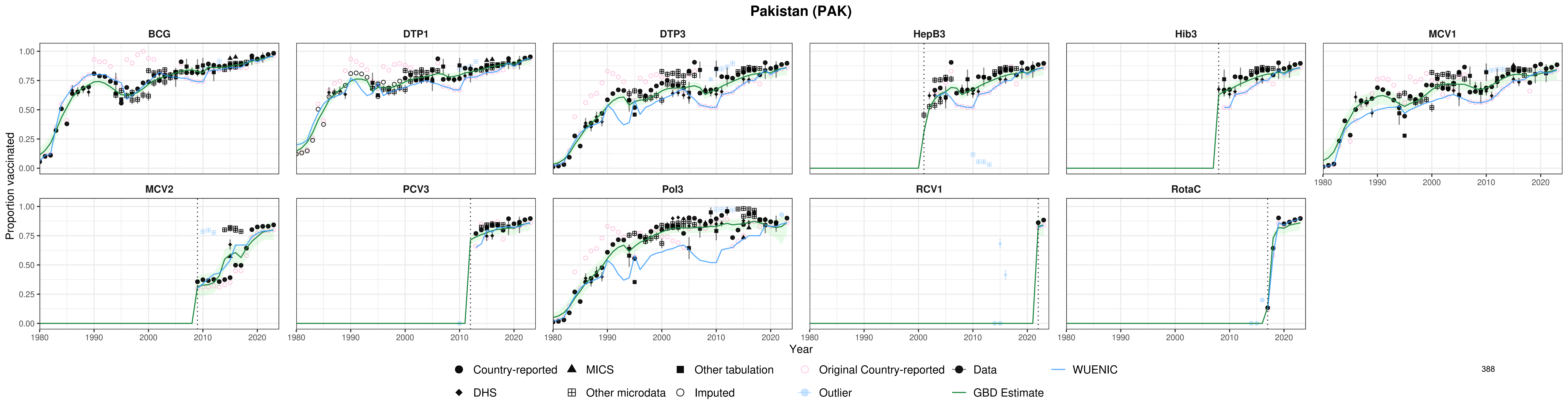

Pakistan (PAK)
BCG
DTP1
DTP3
HepB3
Hib3
MCV1
MCV2
PCV3
Pol3
RCV1
RotaC
Proportion vaccinated
Year
Country-reported
DHS
MICS
Other microdata
Other tabulation
Imputed
Original Country-reported
Outlier
Data
GBD Estimate
WUENIC

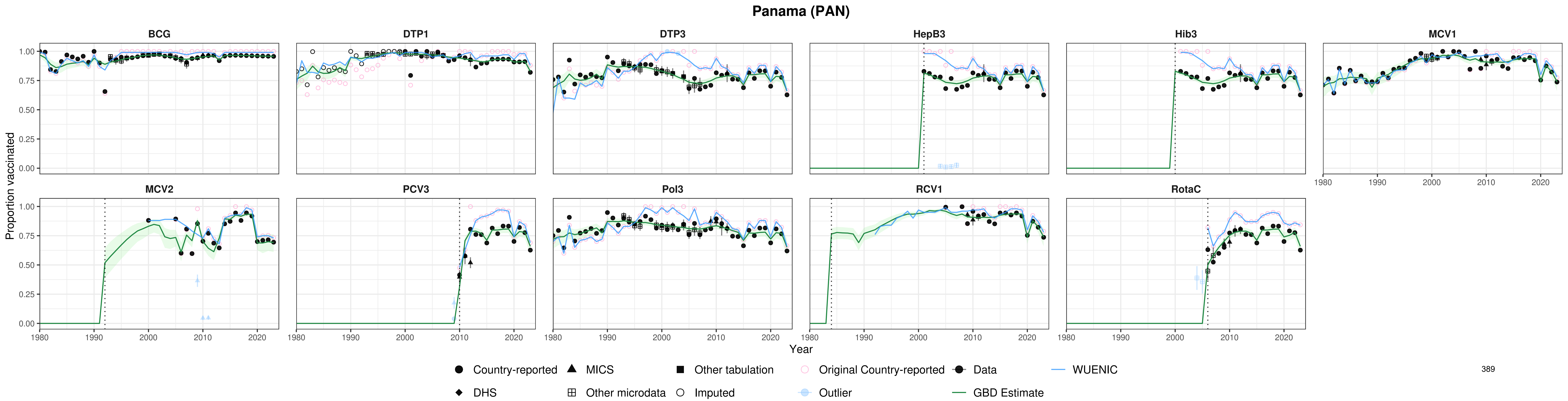

Panama (PAN)
BCG
DTP1
DTP3
HepB3
Hib3
MCV1
MCV2
PCV3
Pol3
RCV1
RotaC
Proportion vaccinated
Year
1.00
0.75
0.50
0.25
0.00
1980
1990
2000
2010
2020
Country-reported
MICS
Other tabulation
Original Country-reported
Data
WUENIC
DHS
Other microdata
Imputed
Outlier
GBD Estimate

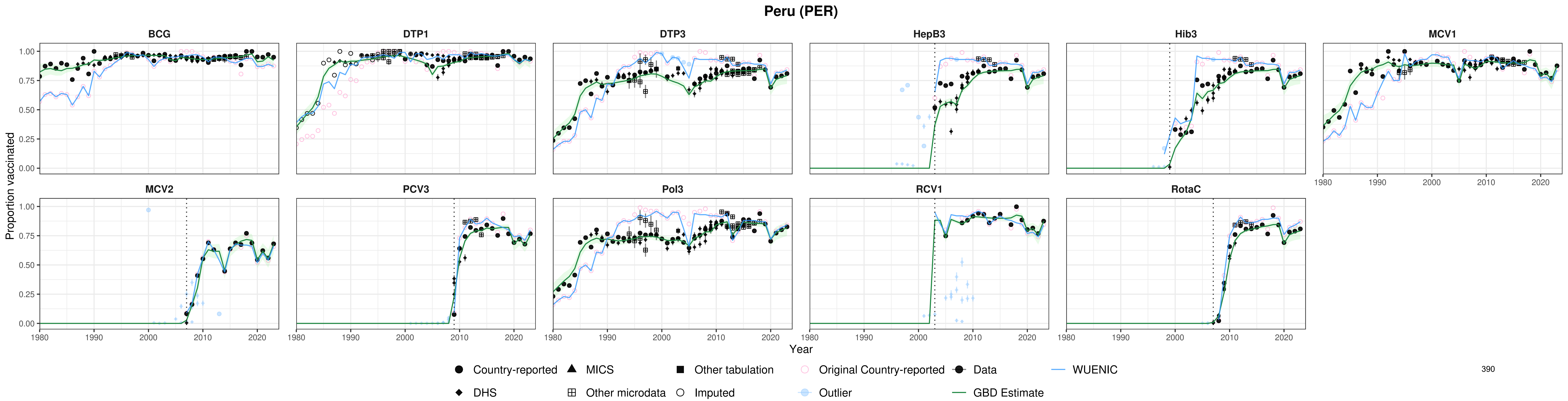

Peru (PER)
BCG
DTP1
DTP3
HepB3
Hib3
MCV1
MCV2
PCV3
Pol3
RCV1
RotaC
Proportion vaccinated
Year
Country-reported
DHS
MICS
Other microdata
Other tabulation
Imputed
Original Country-reported
Outlier
Data
GBD Estimate
WUENIC

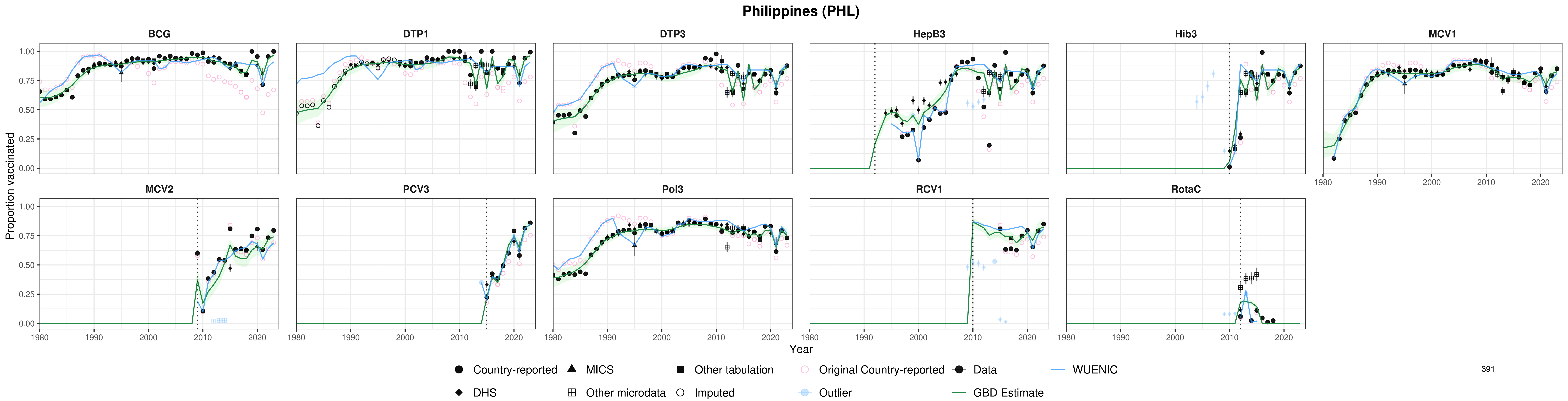

Philippines (PHL)
BCG
DTP1
DTP3
HepB3
Hib3
MCV1
MCV2
PCV3
Pol3
RCV1
RotaC
Proportion vaccinated
Year
0.00
0.25
0.50
0.75
1.00
1980
1990
2000
2010
2020
Country-reported
DHS
MICS
Other microdata
Other tabulation
Imputed
Original Country-reported
Outlier
Data
GBD Estimate
WUENIC

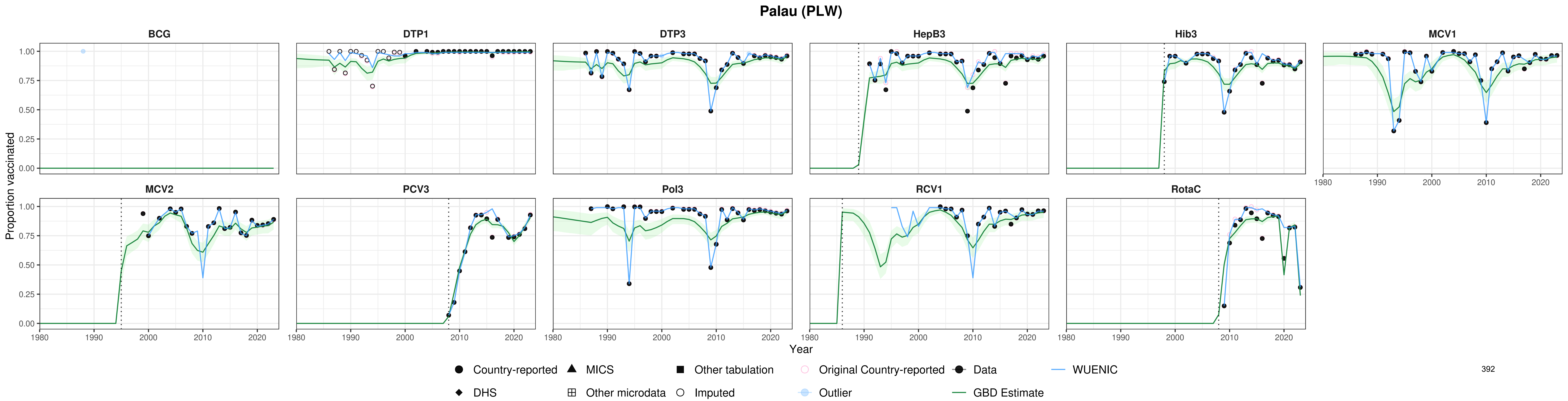
Palau (PLW)
BCG
DTP1
DTP3
HepB3
Hib3
MCV1
MCV2
PCV3
Pol3
RCV1
RotaC
Proportion vaccinated
Year
Country-reported
MICS
Other tabulation
Original Country-reported
Data
WUENIC
DHS
Other microdata
Imputed
Outlier
GBD Estimate

Papua New Guinea (PNG)

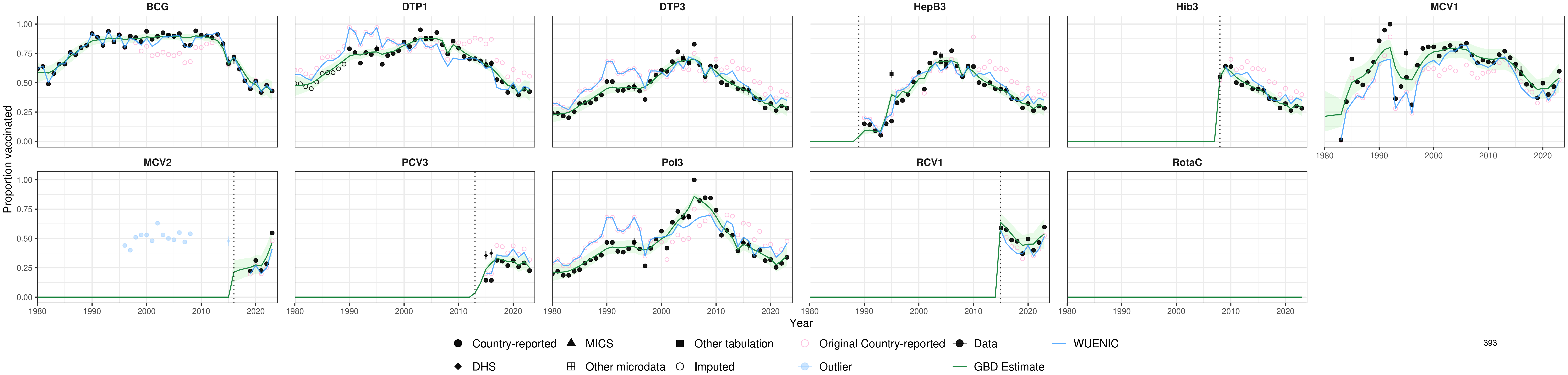

Poland (POL)

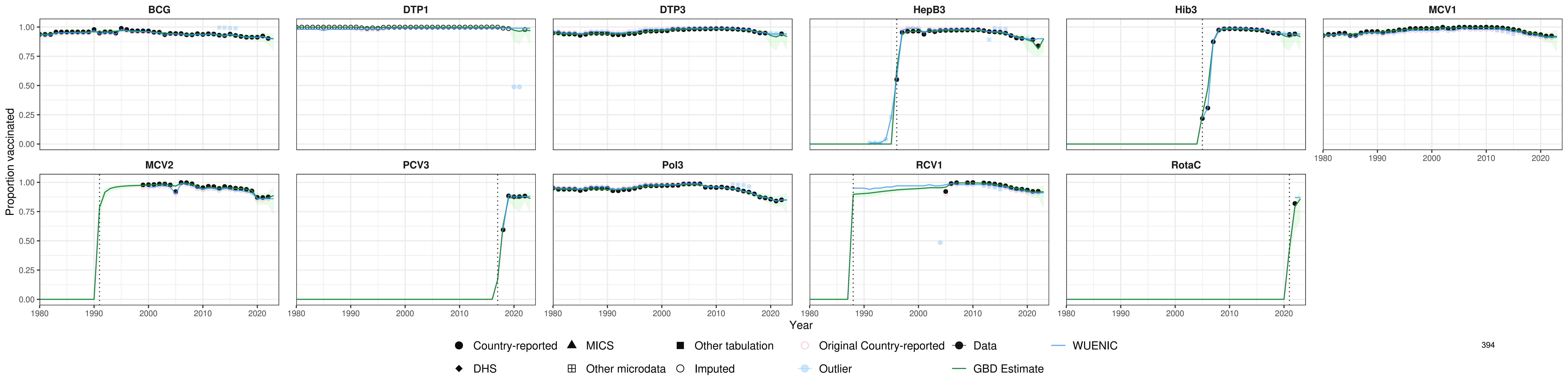

394

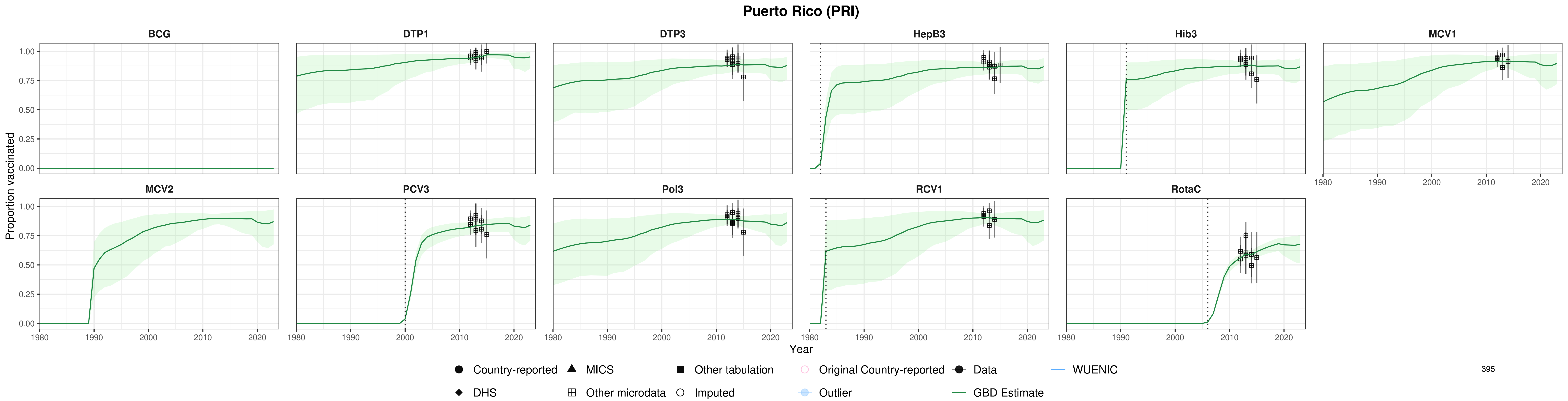

Puerto Rico (PRI)
BCG
DTP1
DTP3
HepB3
Hib3
MCV1
MCV2
PCV3
Pol3
RCV1
RotaC
Proportion vaccinated
0.00
0.25
0.50
0.75
1.00
1980
1990
2000
2010
2020
Year
Country-reported
MICS
Other tabulation
Original Country-reported
Data
WUENIC
DHS
Other microdata
Imputed
Outlier
GBD Estimate

North Korea (PRK)

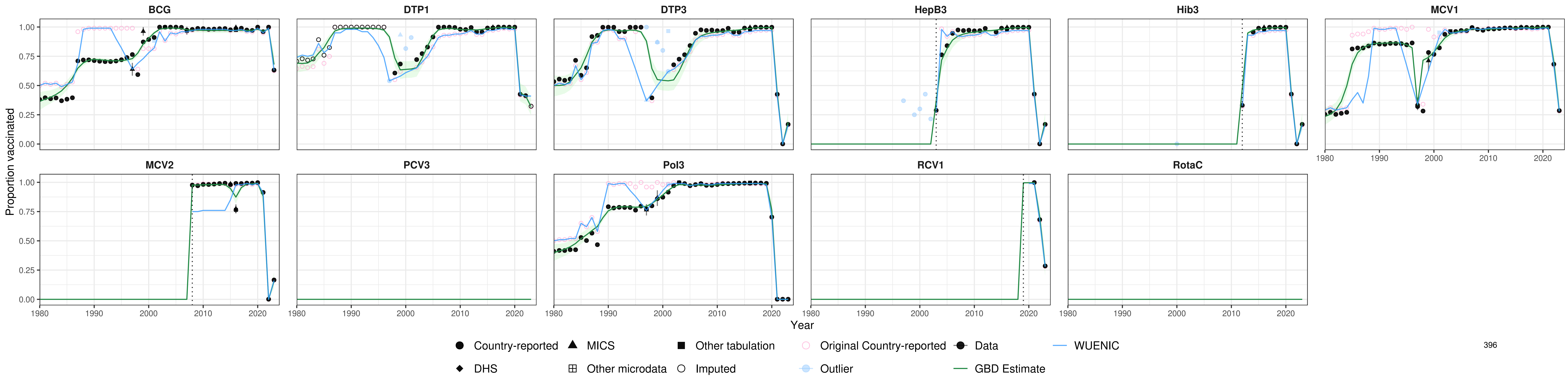

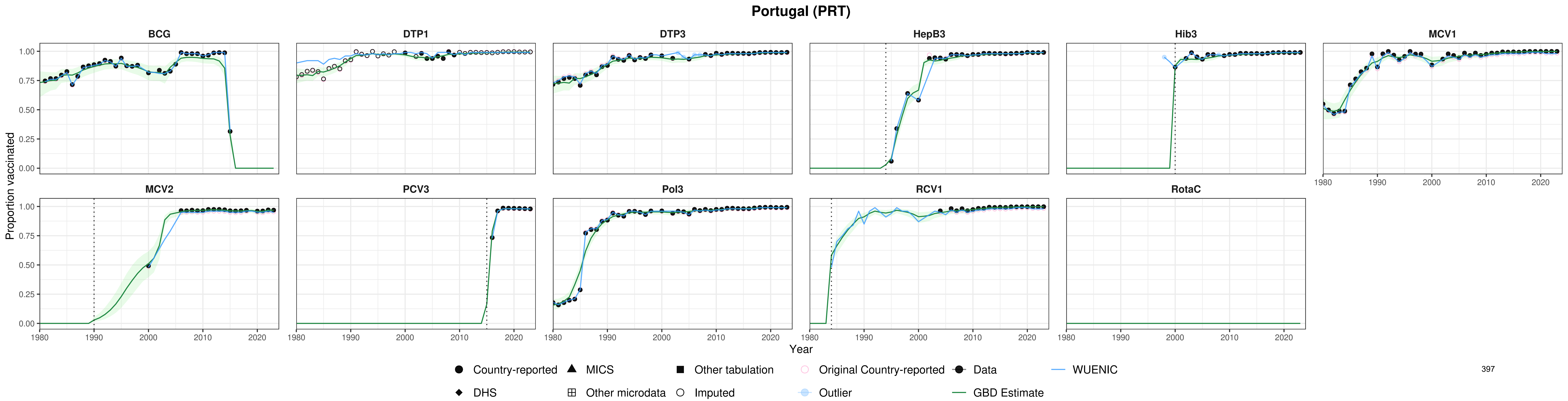
Portugal (PRT)
BCG
DTP1
DTP3
HepB3
Hib3
MCV1
MCV2
PCV3
Pol3
RCV1
RotaC
Proportion vaccinated
Year
Country-reported
MICS
Other tabulation
Original Country-reported
Data
WUENIC
DHS
Other microdata
Imputed
Outlier
GBD Estimate

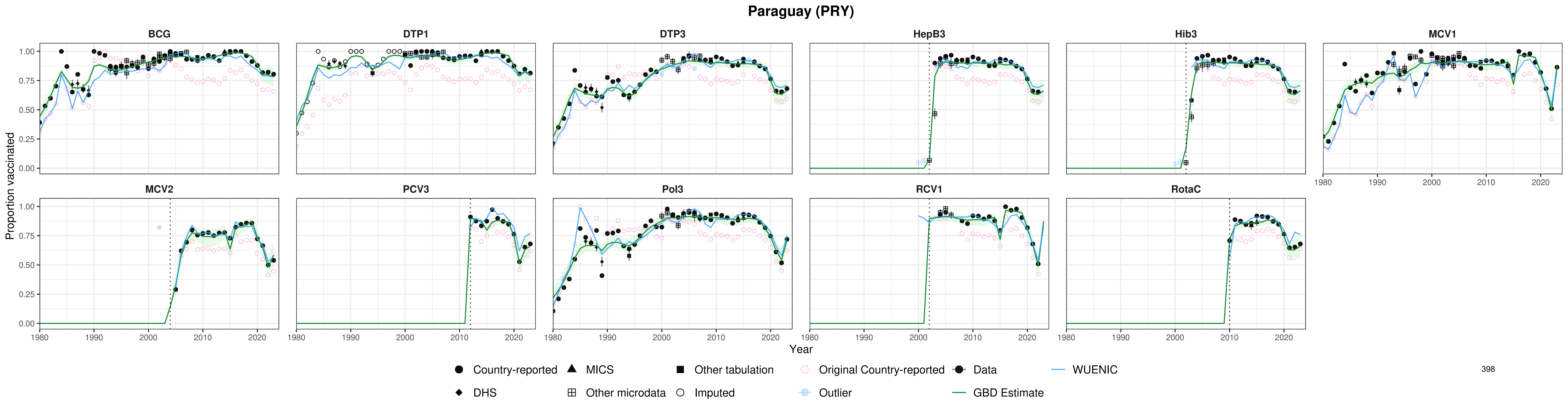
Paraguay (PRY)
BCG
DTP1
DTP3
HepB3
Hib3
MCV1
MCV2
PCV3
Pol3
RCV1
RotaC
Proportion vaccinated
Year
0.00
0.25
0.50
0.75
1.00
1980
1990
2000
2010
2020
Country-reported
MICS
Other tabulation
Original Country-reported
Data
WUENIC
DHS
Other microdata
Imputed
Outlier
GBD Estimate

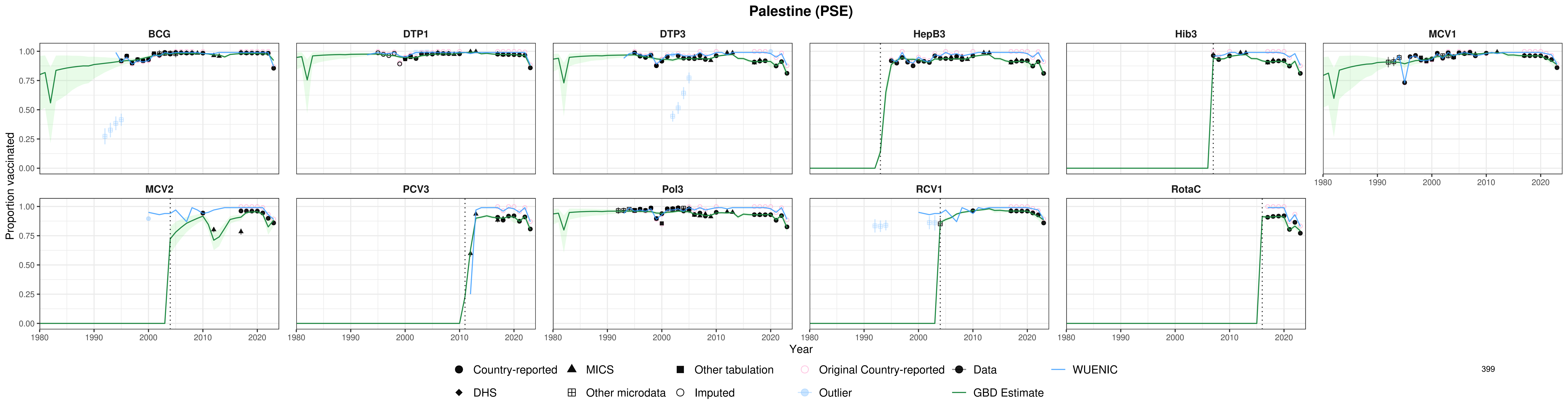

Palestine (PSE)
BCG
DTP1
DTP3
HepB3
Hib3
MCV1
MCV2
PCV3
Pol3
RCV1
RotaC
Proportion vaccinated
Year
0.00
0.25
0.50
0.75
1.00
1980
1990
2000
2010
2020
Country-reported
MICS
Other tabulation
Original Country-reported
Data
WUENIC
DHS
Other microdata
Imputed
Outlier
GBD Estimate

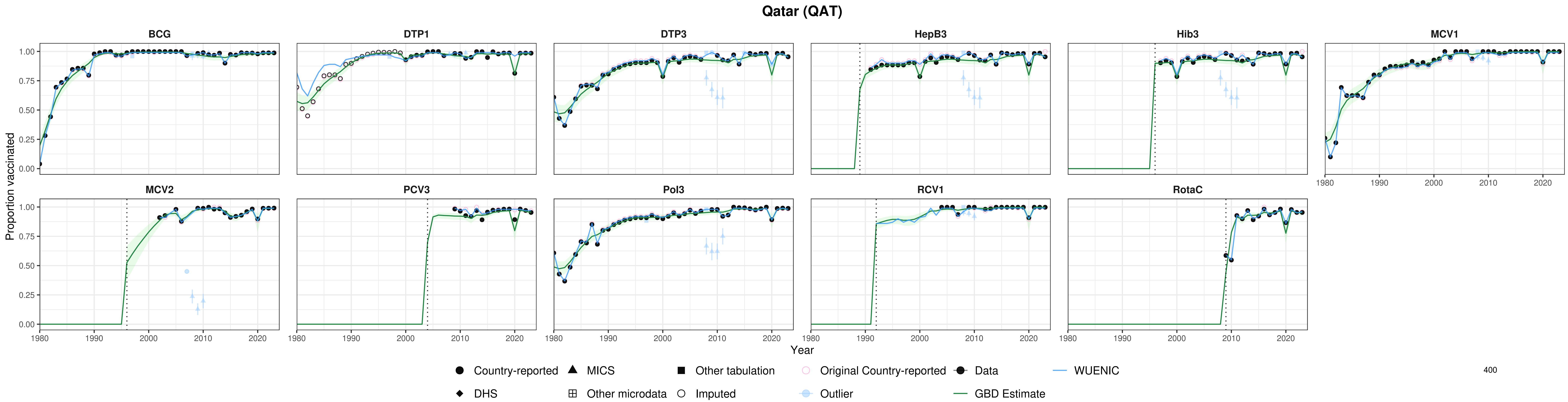

Qatar (QAT)
BCG
DTP1
DTP3
HepB3
Hib3
MCV1
MCV2
PCV3
Pol3
RCV1
RotaC
Proportion vaccinated
Year
Country-reported
MICS
Other tabulation
Original Country-reported
Data
WUENIC
DHS
Other microdata
Imputed
Outlier
GBD Estimate

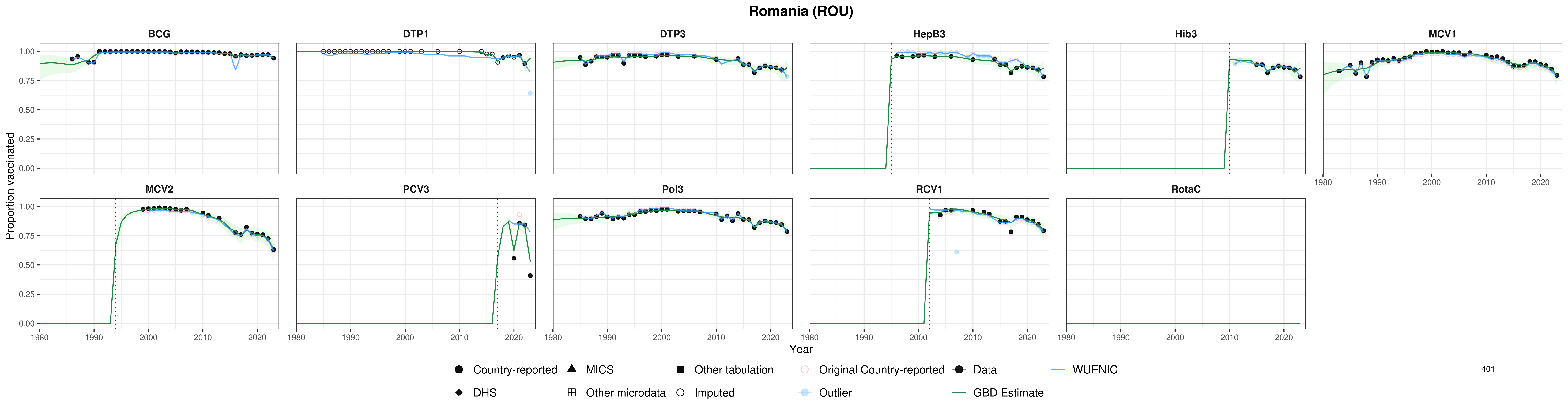

Romania (ROU)
BCG
DTP1
DTP3
HepB3
Hib3
MCV1
MCV2
PCV3
Pol3
RCV1
RotaC
Proportion vaccinated
Year
Country-reported
MICS
Other tabulation
Original Country-reported
Data
WUENIC
DHS
Other microdata
Imputed
Outlier
GBD Estimate

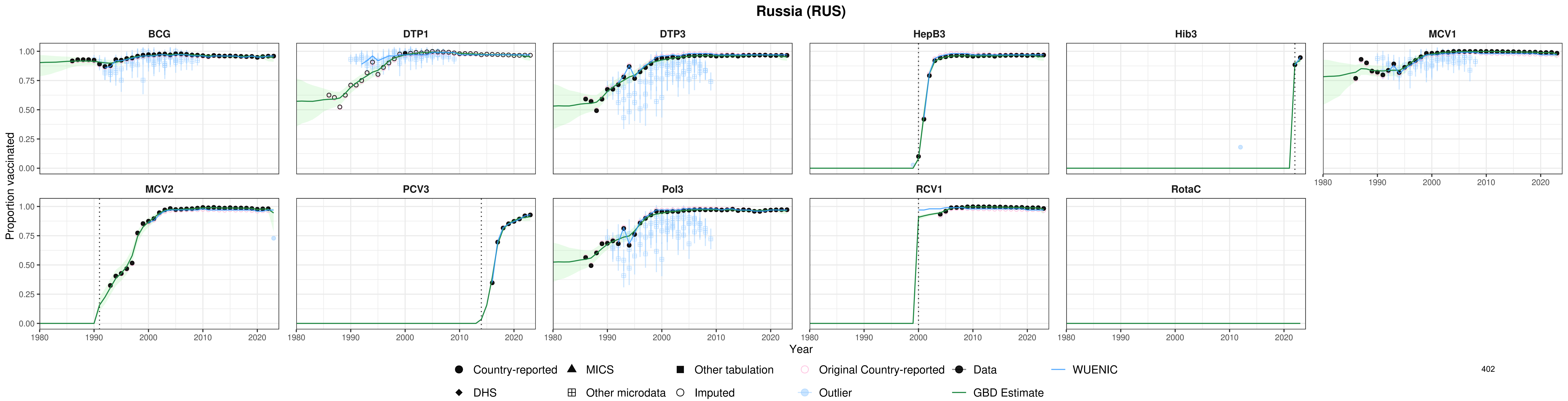

Russia (RUS)
BCG
DTP1
DTP3
HepB3
Hib3
MCV1
MCV2
PCV3
Pol3
RCV1
RotaC
Proportion vaccinated
Year
1.00
0.75
0.50
0.25
0.00
1980
1990
2000
2010
2020
Country-reported
MICS
Other tabulation
Original Country-reported
Data
WUENIC
DHS
Other microdata
Imputed
Outlier
GBD Estimate

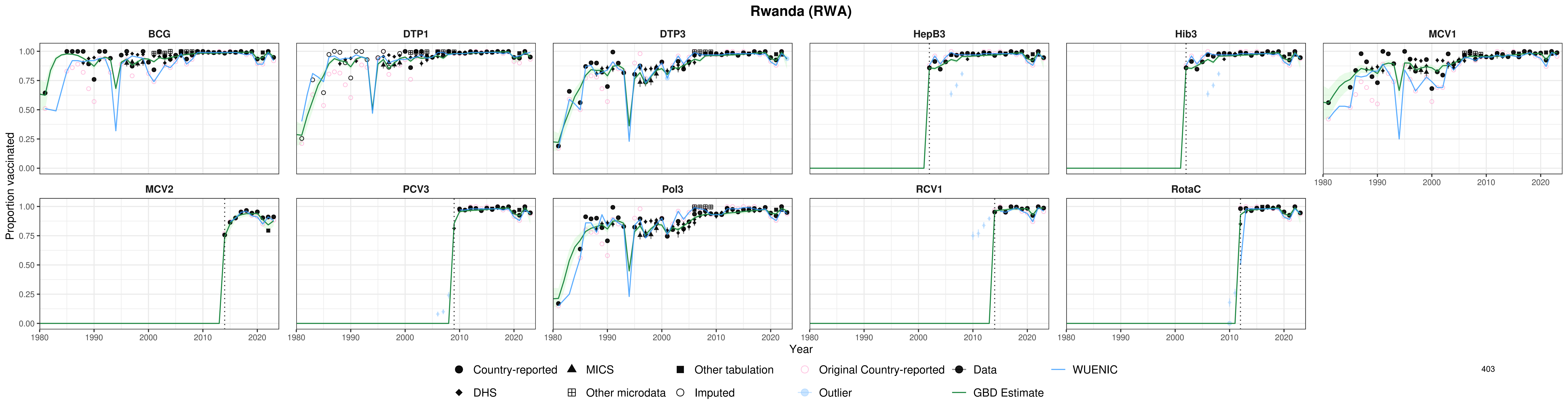
Rwanda (RWA)
BCG
DTP1
DTP3
HepB3
Hib3
MCV1
MCV2
PCV3
Pol3
RCV1
RotaC
Proportion vaccinated
Year
1.00
0.75
0.50
0.25
0.00
1980
1990
2000
2010
2020
Country-reported
DHS
MICS
Other microdata
Other tabulation
Imputed
Original Country-reported
Outlier
Data
GBD Estimate
WUENIC

Saudi Arabia (SAU)

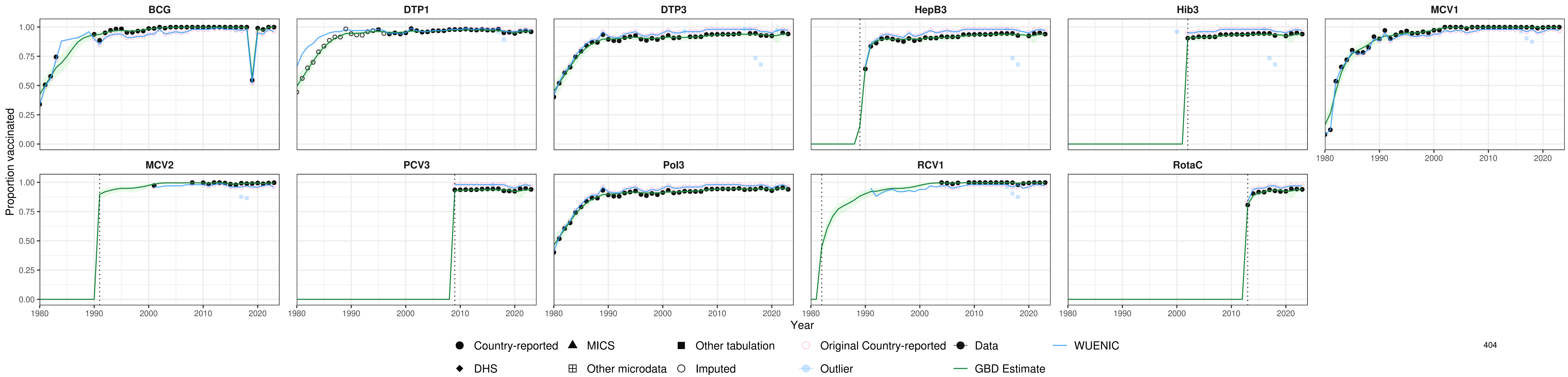

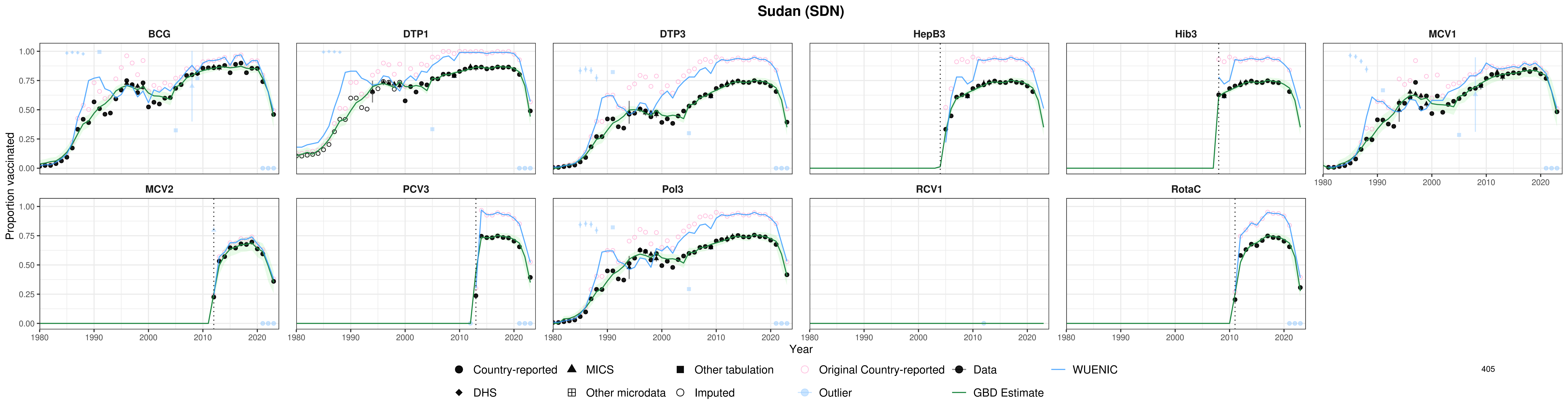
Sudan (SDN)
BCG
DTP1
DTP3
HepB3
Hib3
MCV1
MCV2
PCV3
Pol3
RCV1
RotaC
Proportion vaccinated
0.00
0.25
0.50
0.75
1.00
1980
1990
2000
2010
2020
Year
Country-reported
MICS
Other tabulation
Original Country-reported
Data
WUENIC
DHS
Other microdata
Imputed
Outlier
GBD Estimate

Senegal (SEN)

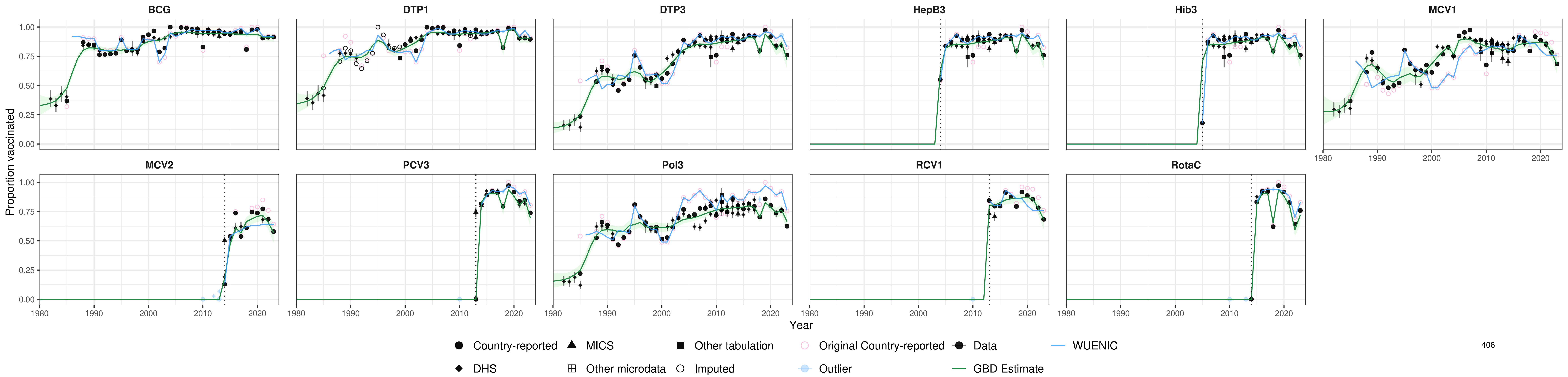

BCG
DTP1
DTP3
HepB3
Hib3
MCV1
MCV2
PCV3
Pol3
RCV1
RotaC
Proportion vaccinated
Year
Country-reported
MICS
Other tabulation
Original Country-reported
Data
WUENIC
DHS
Other microdata
Imputed
Outlier
GBD Estimate

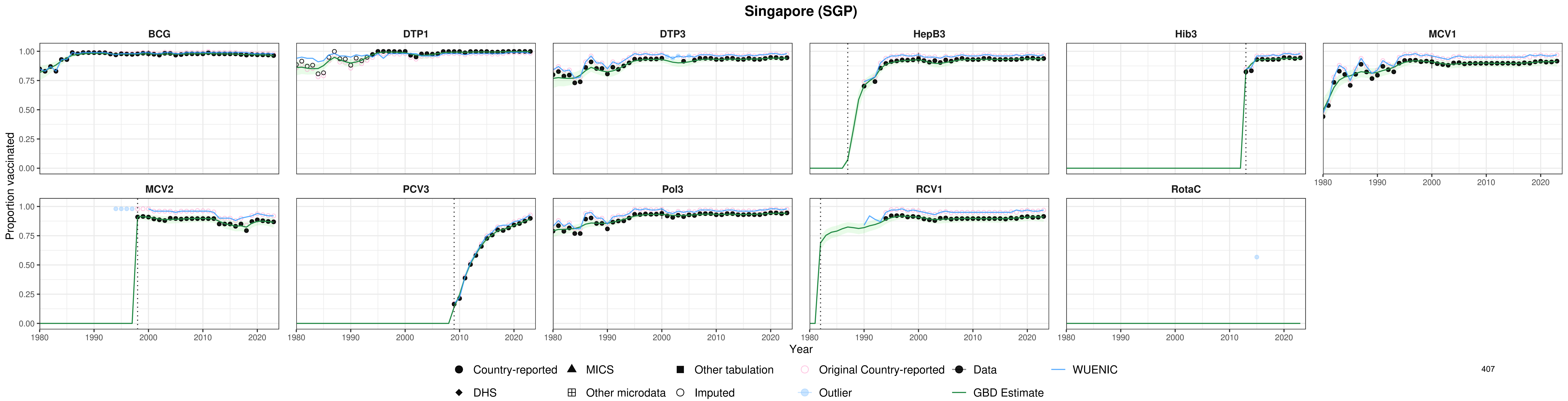
Singapore (SGP)
BCG
DTP1
DTP3
HepB3
Hib3
MCV1
MCV2
PCV3
Pol3
RCV1
RotaC
Proportion vaccinated
Year
1.00
0.75
0.50
0.25
0.00
1980
1990
2000
2010
2020
Country-reported
MICS
Other tabulation
Original Country-reported
Data
WUENIC
DHS
Other microdata
Imputed
Outlier
GBD Estimate

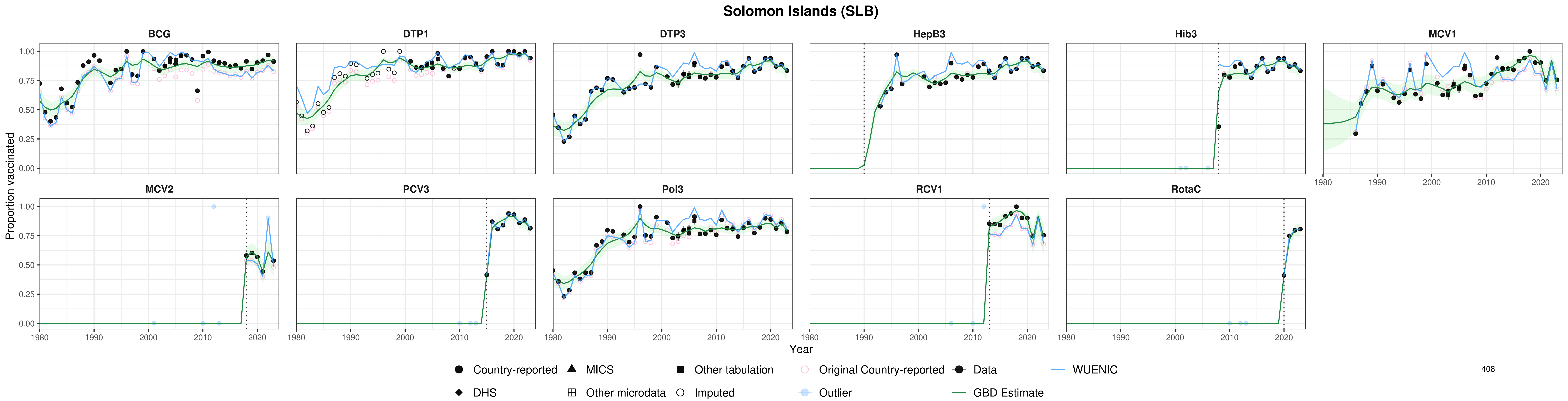
Solomon Islands (SLB)
BCG
DTP1
DTP3
HepB3
Hib3
MCV1
MCV2
PCV3
Pol3
RCV1
RotaC
Proportion vaccinated
Year
Country-reported
MICS
Other tabulation
Original Country-reported
Data
WUENIC
DHS
Other microdata
Imputed
Outlier
GBD Estimate

Sierra Leone (SLE)

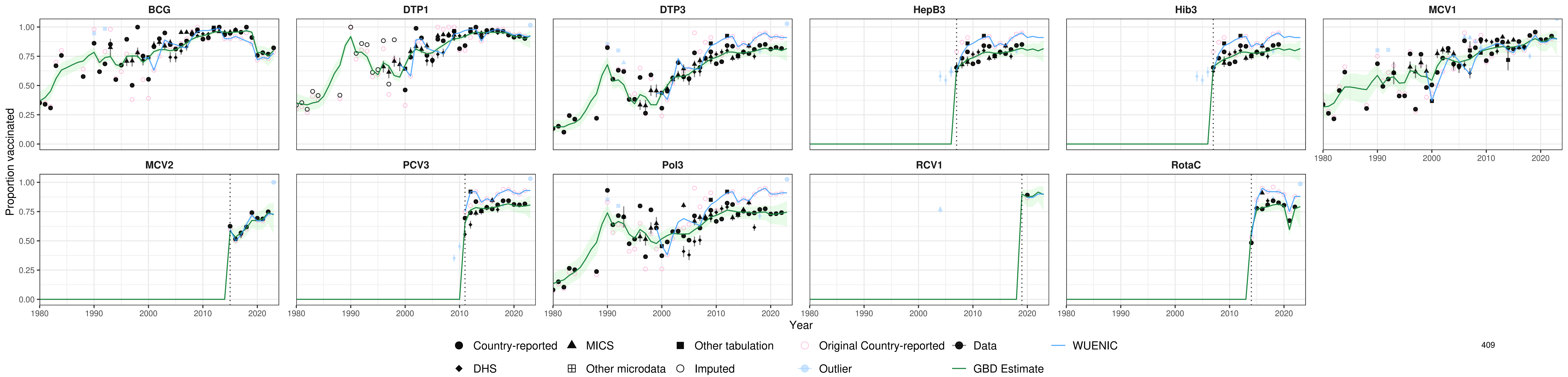

El Salvador (SLV)

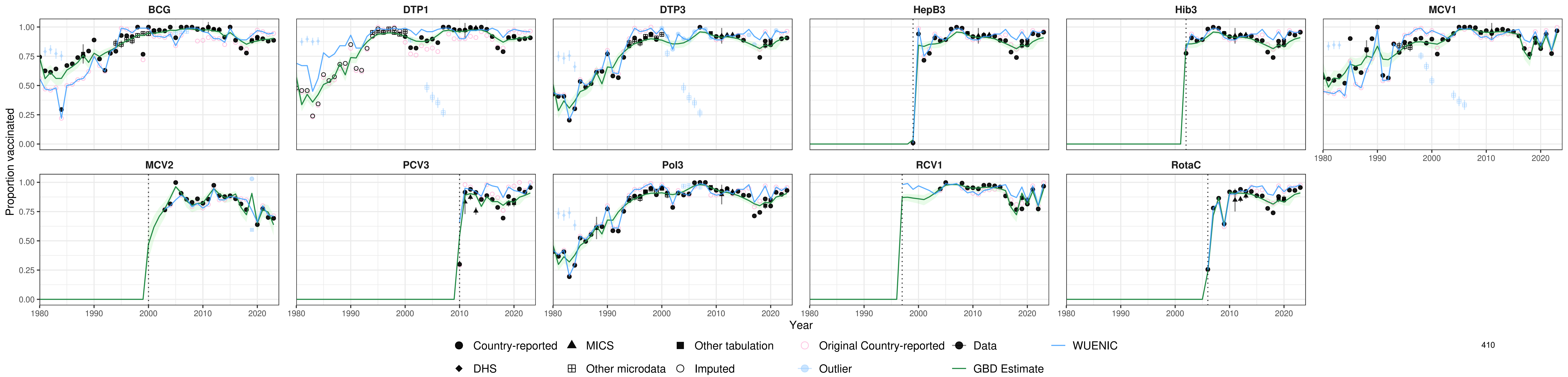

San Marino (SMR)

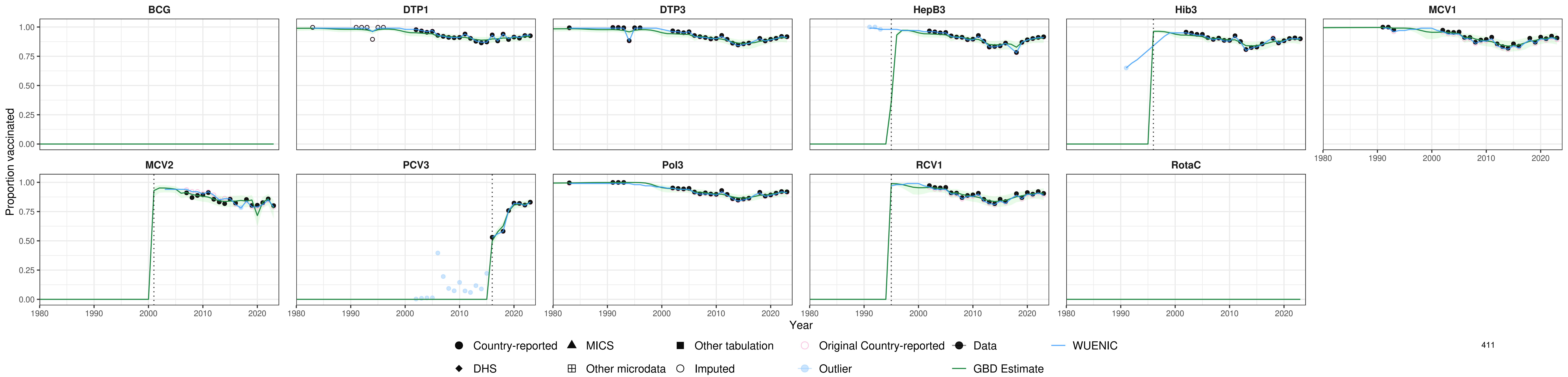

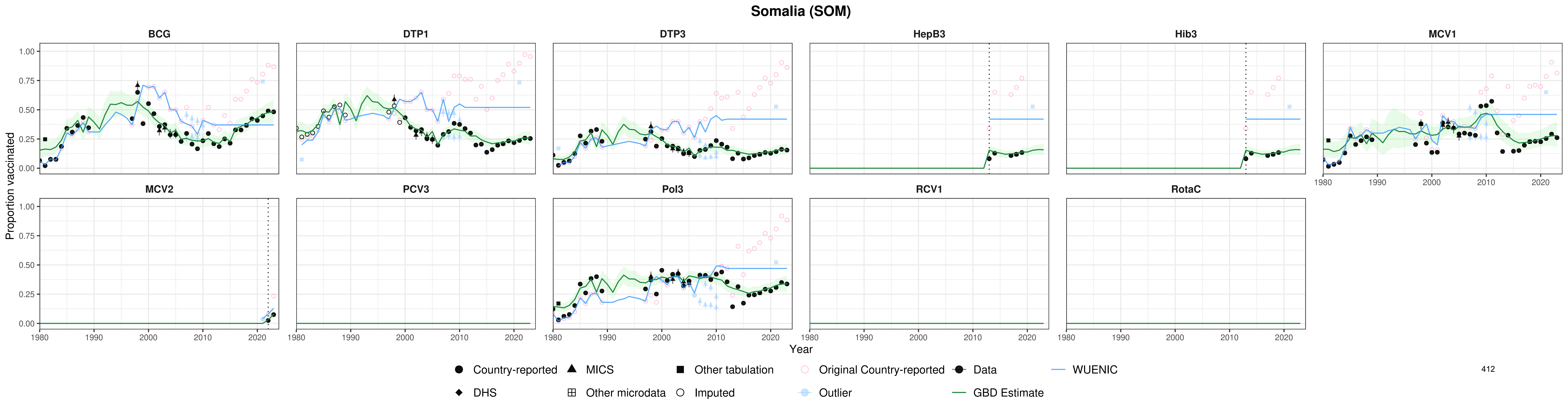

Somalia (SOM)
BCG
DTP1
DTP3
HepB3
Hib3
MCV1
MCV2
PCV3
Pol3
RCV1
RotaC
Proportion vaccinated
1.00
0.75
0.50
0.25
0.00
1980
1990
2000
2010
2020
Year
Country-reported
MICS
Other tabulation
Original Country-reported
Data
WUENIC
DHS
Other microdata
Imputed
Outlier
GBD Estimate

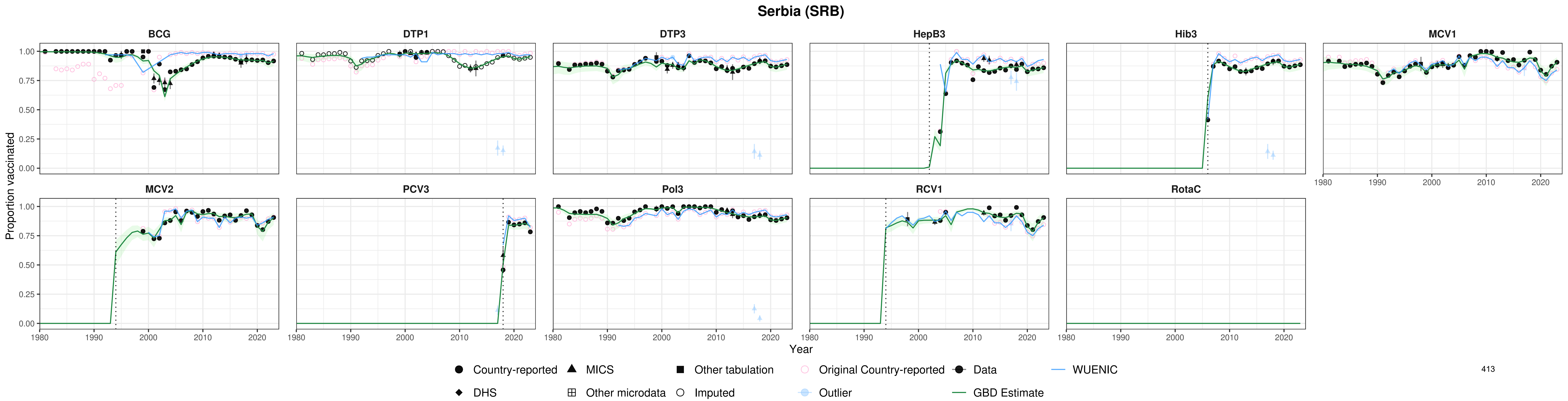
Serbia (SRB)
BCG
DTP1
DTP3
HepB3
Hib3
MCV1
MCV2
PCV3
Pol3
RCV1
RotaC
Proportion vaccinated
Year
Country-reported
MICS
Other tabulation
Original Country-reported
Data
WUENIC
DHS
Other microdata
Imputed
Outlier
GBD Estimate

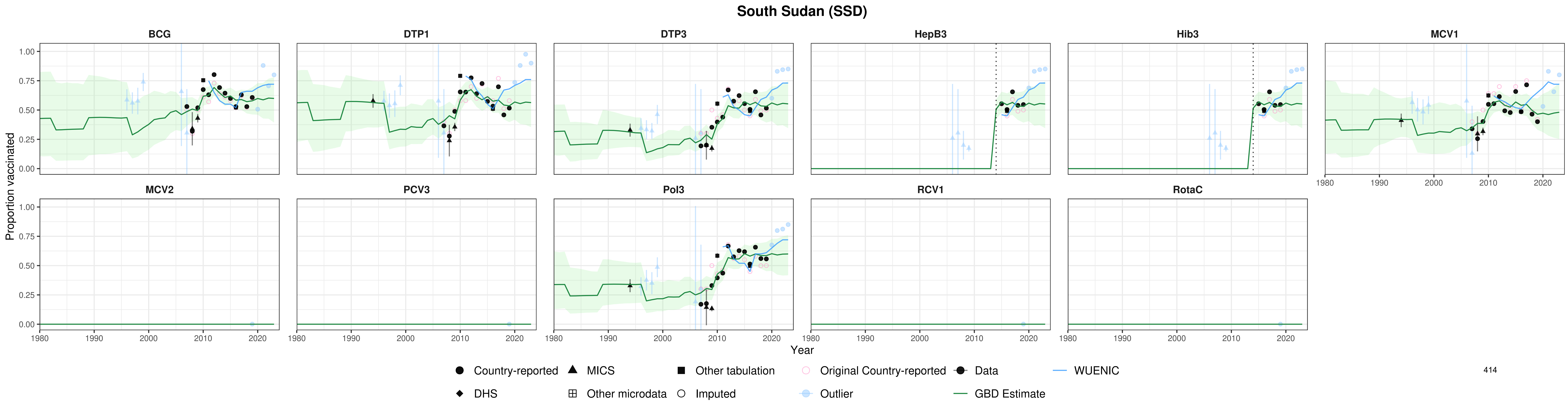

South Sudan (SSD)
BCG
DTP1
DTP3
HepB3
Hib3
MCV1
MCV2
PCV3
Pol3
RCV1
RotaC
Proportion vaccinated
0.00
0.25
0.50
0.75
1.00
1980
1990
2000
2010
2020
Year
Country-reported
MICS
Other tabulation
Original Country-reported
Data
WUENIC
DHS
Other microdata
Imputed
Outlier
GBD Estimate

São Tomé and Príncipe (STP)

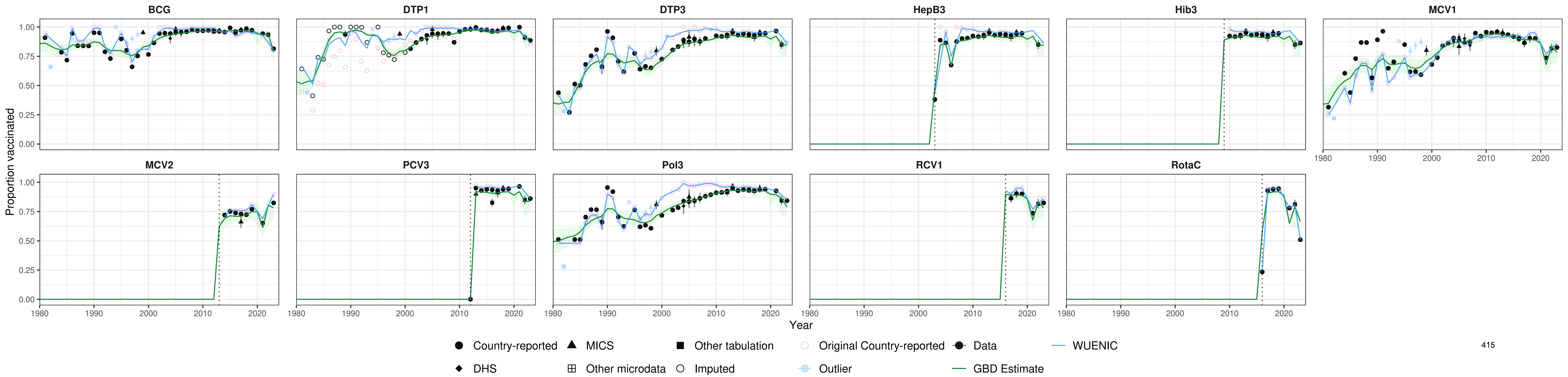

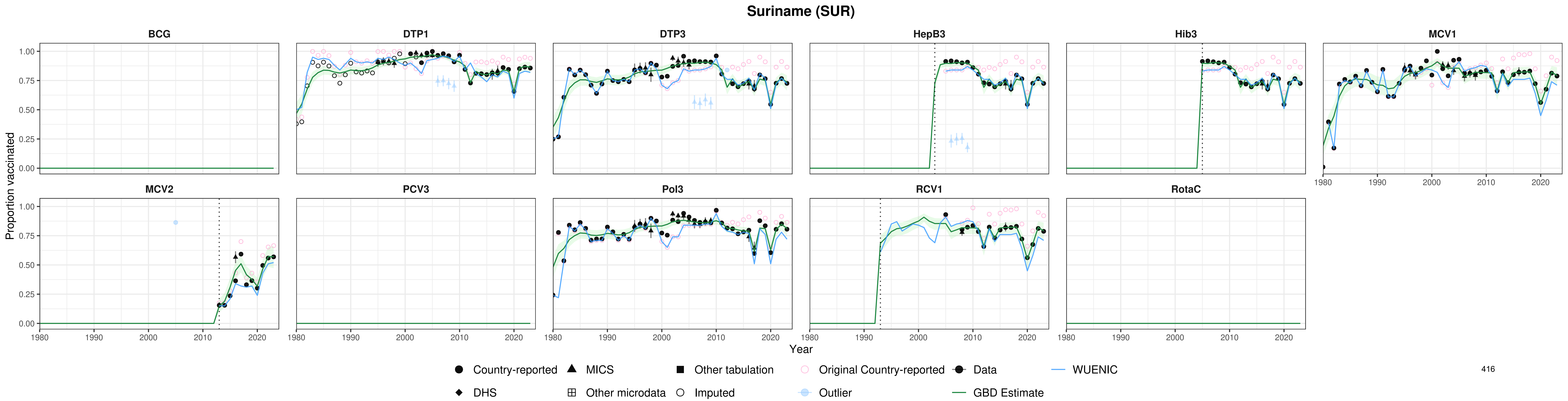

Suriname (SUR)
BCG
DTP1
DTP3
HepB3
Hib3
MCV1
MCV2
PCV3
Pol3
RCV1
RotaC
Proportion vaccinated
Year
Country-reported
MICS
Other tabulation
Original Country-reported
Data
WUENIC
DHS
Other microdata
Imputed
Outlier
GBD Estimate

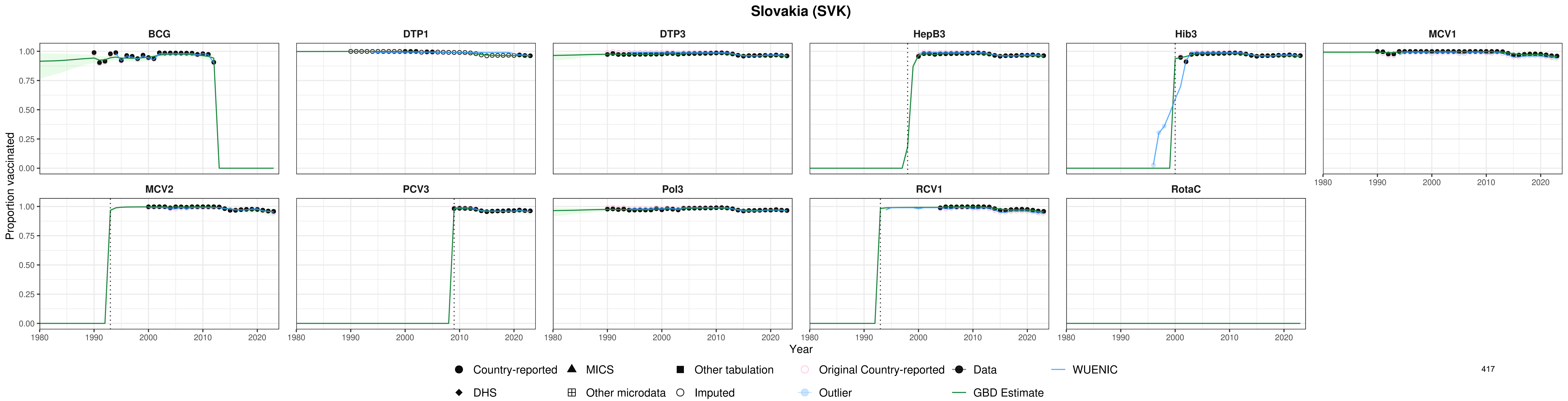
Slovakia (SVK)
BCG
DTP1
DTP3
HepB3
Hib3
MCV1
MCV2
PCV3
Pol3
RCV1
RotaC
Proportion vaccinated
1.00
0.75
0.50
0.25
0.00
1980
1990
2000
2010
2020
Year
Country-reported
MICS
Other tabulation
Original Country-reported
Data
WUENIC
DHS
Other microdata
Imputed
Outlier
GBD Estimate

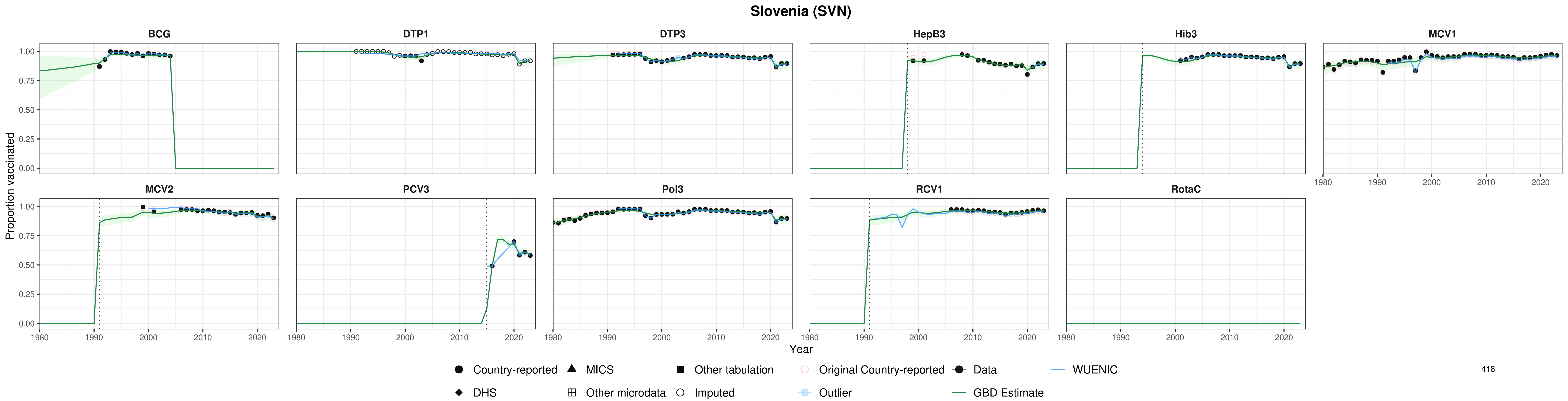

Slovenia (SVN)
BCG
DTP1
DTP3
HepB3
Hib3
MCV1
MCV2
PCV3
Pol3
RCV1
RotaC
Proportion vaccinated
1.00
0.75
0.50
0.25
0.00
1980
1990
2000
2010
2020
Year
Country-reported
MICS
Other tabulation
Original Country-reported
Data
WUENIC
DHS
Other microdata
Imputed
Outlier
GBD Estimate

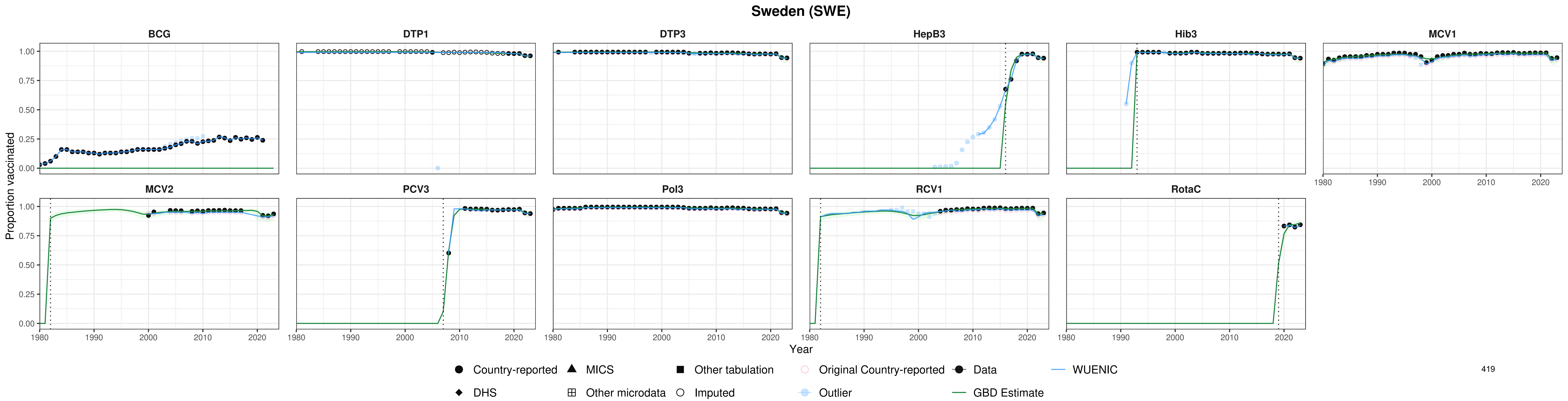
Sweden (SWE)
BCG
DTP1
DTP3
HepB3
Hib3
MCV1
MCV2
PCV3
Pol3
RCV1
RotaC
Proportion vaccinated
Year
Country-reported
MICS
Other tabulation
Original Country-reported
Data
WUENIC
DHS
Other microdata
Imputed
Outlier
GBD Estimate

Eswatini (SWZ)

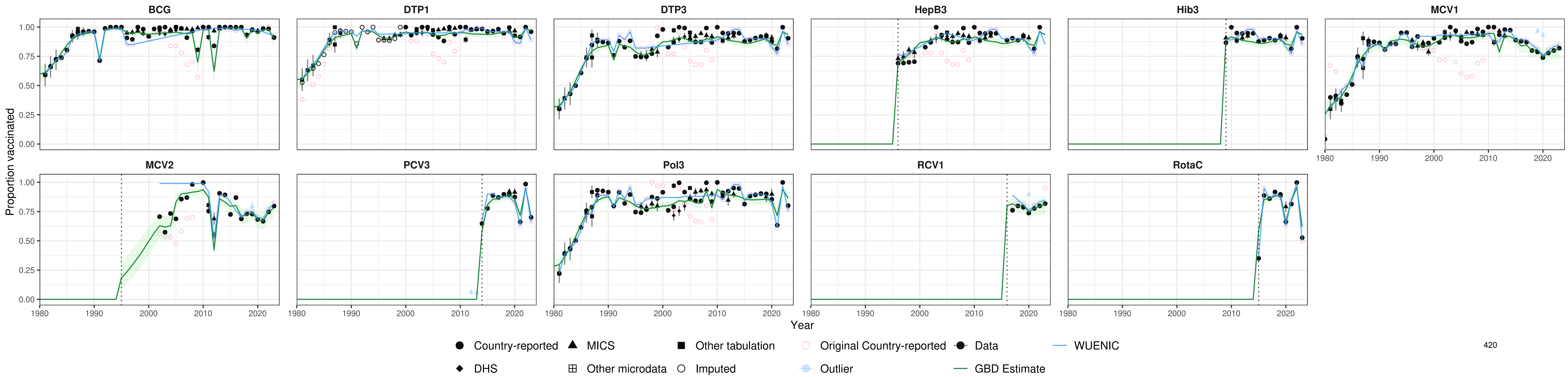

Seychelles (SYC)

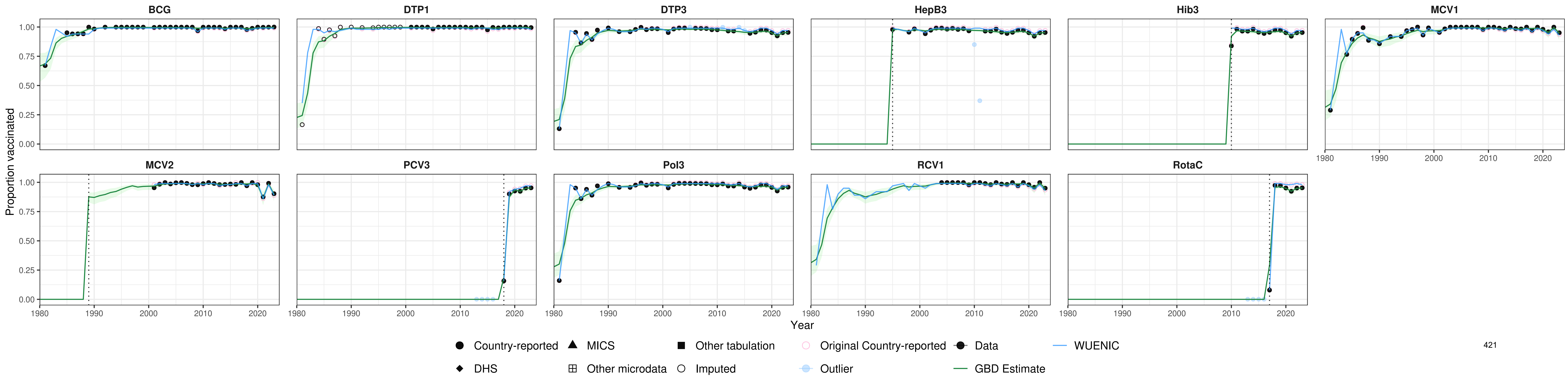

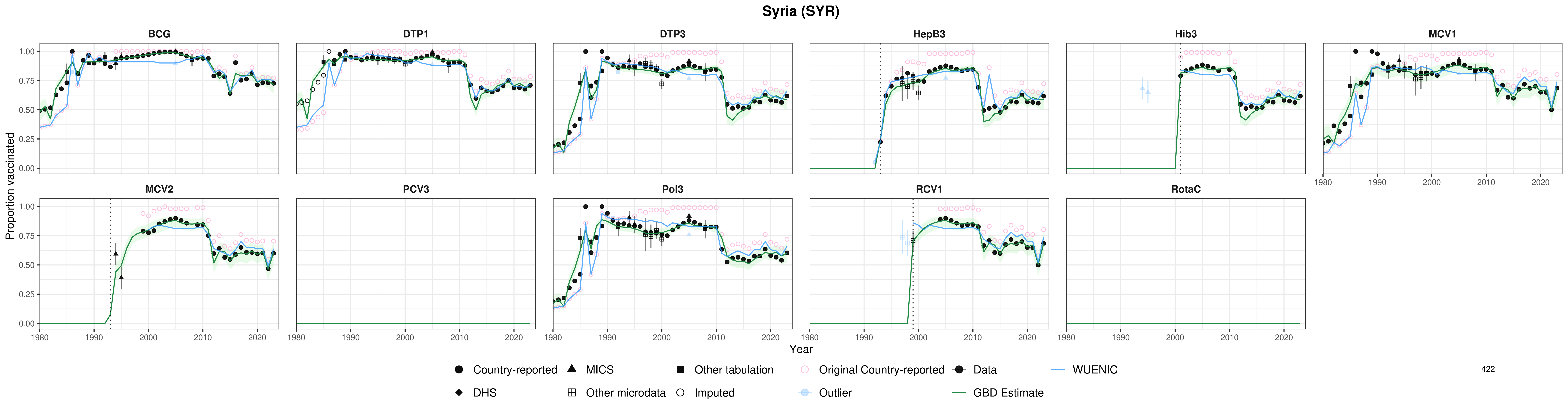

Syria (SYR)
BCG
DTP1
DTP3
HepB3
Hib3
MCV1
MCV2
PCV3
Pol3
RCV1
RotaC
Proportion vaccinated
Year
Country-reported
MICS
Other tabulation
Original Country-reported
Data
WUENIC
DHS
Other microdata
Imputed
Outlier
GBD Estimate

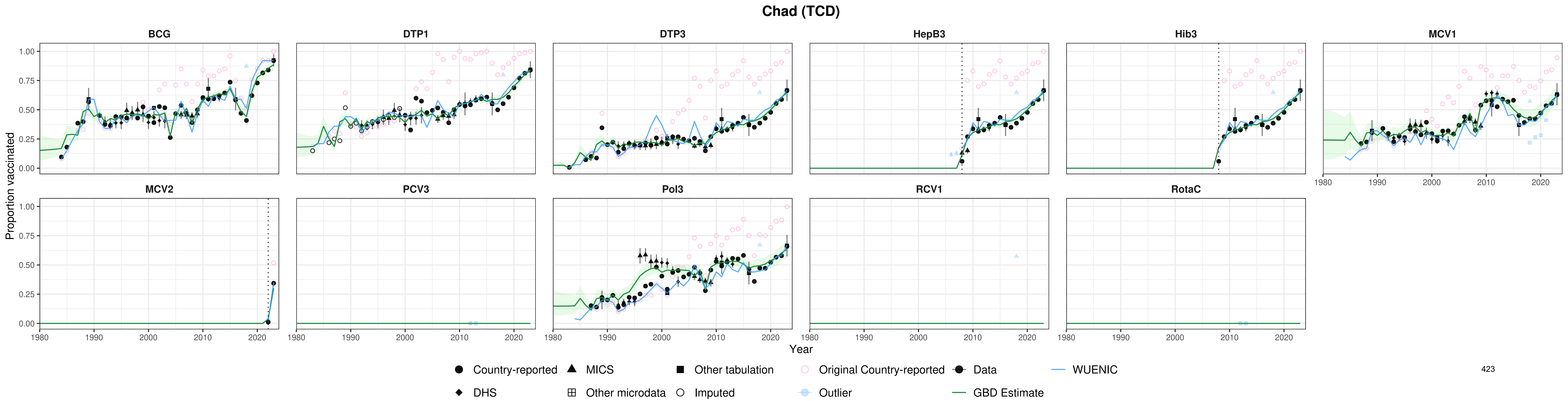
Chad (TCD)
BCG
DTP1
DTP3
HepB3
Hib3
MCV1
MCV2
PCV3
Pol3
RCV1
RotaC
Proportion vaccinated
Year
Country-reported
MICS
Other tabulation
Original Country-reported
Data
WUENIC
DHS
Other microdata
Imputed
Outlier
GBD Estimate

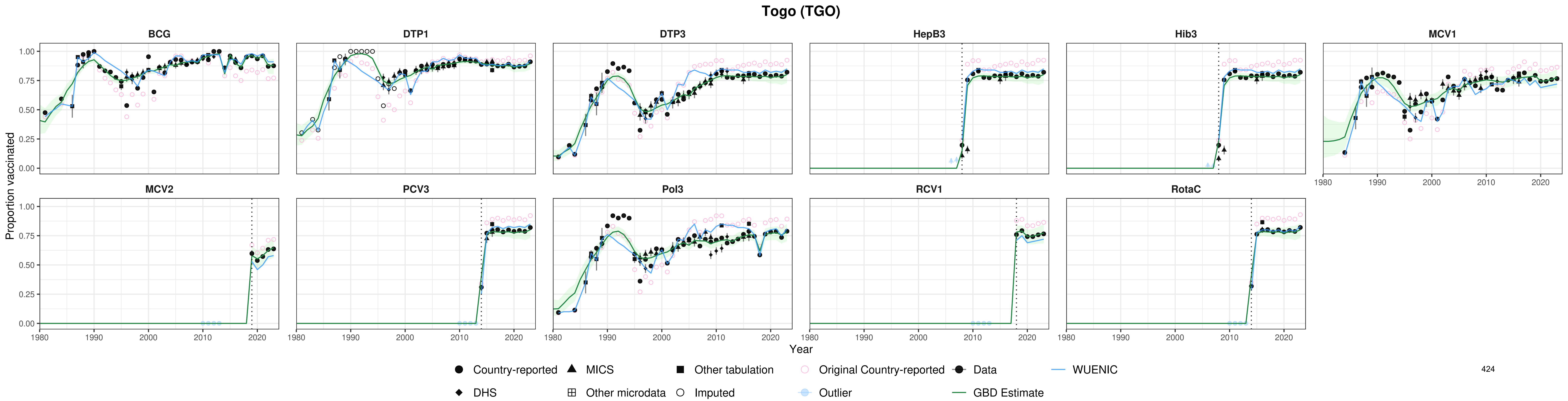
Togo (TGO)
BCG
DTP1
DTP3
HepB3
Hib3
MCV1
MCV2
PCV3
Pol3
RCV1
RotaC
Proportion vaccinated
Year
Country-reported
MICS
Other tabulation
Original Country-reported
Data
WUENIC
DHS
Other microdata
Imputed
Outlier
GBD Estimate

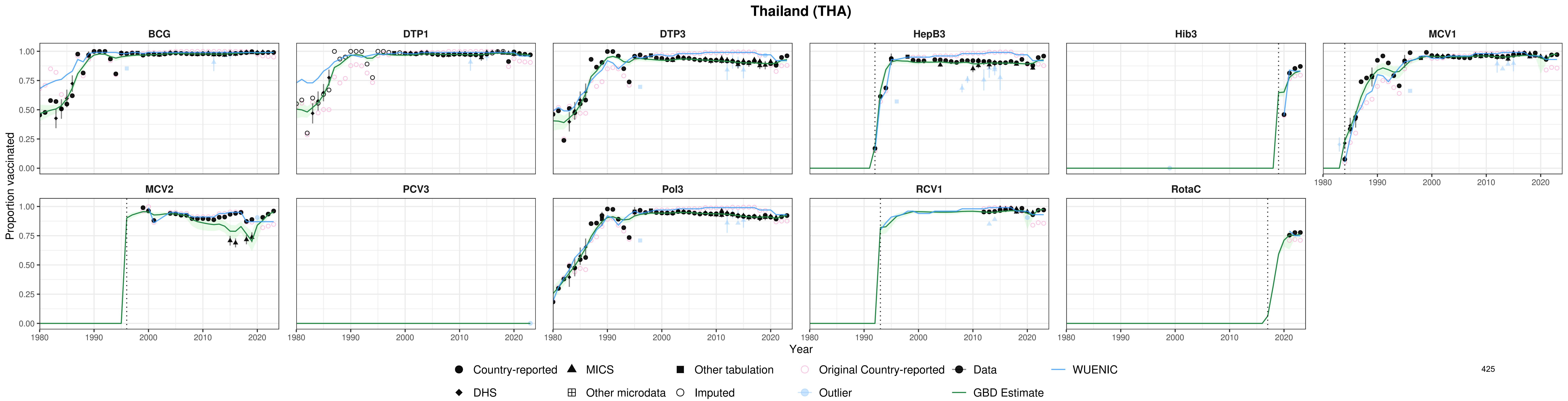

Thailand (THA)
BCG
DTP1
DTP3
HepB3
Hib3
MCV1
MCV2
PCV3
Pol3
RCV1
RotaC
Proportion vaccinated
Year
Country-reported
MICS
Other tabulation
Original Country-reported
Data
WUENIC
DHS
Other microdata
Imputed
Outlier
GBD Estimate

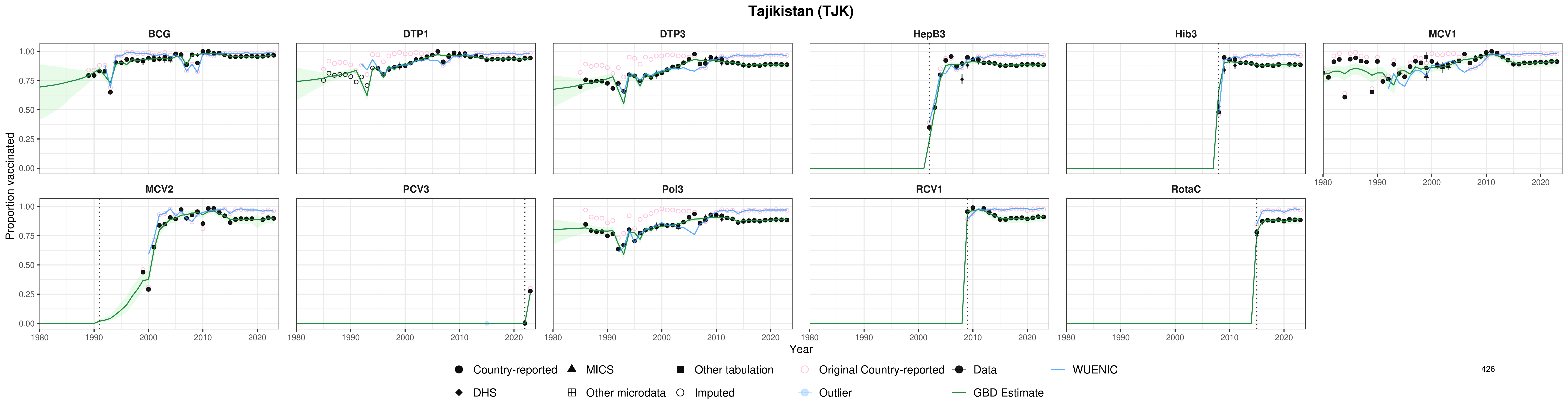

Tajikistan (TJK)
BCG
DTP1
DTP3
HepB3
Hib3
MCV1
MCV2
PCV3
Pol3
RCV1
RotaC
Proportion vaccinated
Year
Country-reported
MICS
Other tabulation
Original Country-reported
Data
WUENIC
DHS
Other microdata
Imputed
Outlier
GBD Estimate

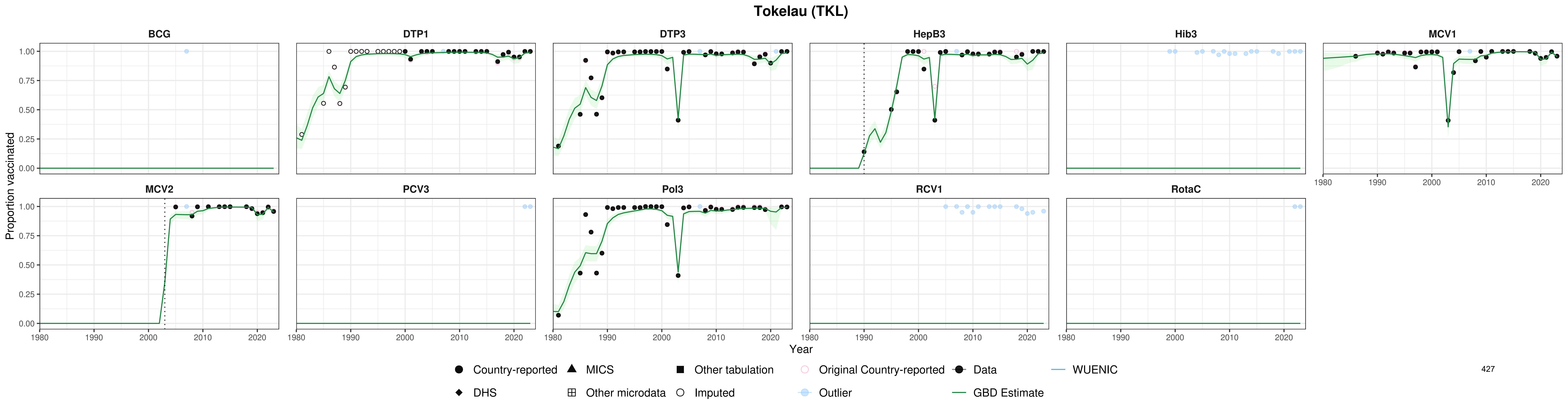

Tokelau (TKL)
BCG
DTP1
DTP3
HepB3
Hib3
MCV1
MCV2
PCV3
Pol3
RCV1
RotaC
Proportion vaccinated
0.00
0.25
0.50
0.75
1.00
1980
1990
2000
2010
2020
Year
Country-reported
MICS
Other tabulation
Original Country-reported
Data
WUENIC
DHS
Other microdata
Imputed
Outlier
GBD Estimate

Turkmenistan (TKM)

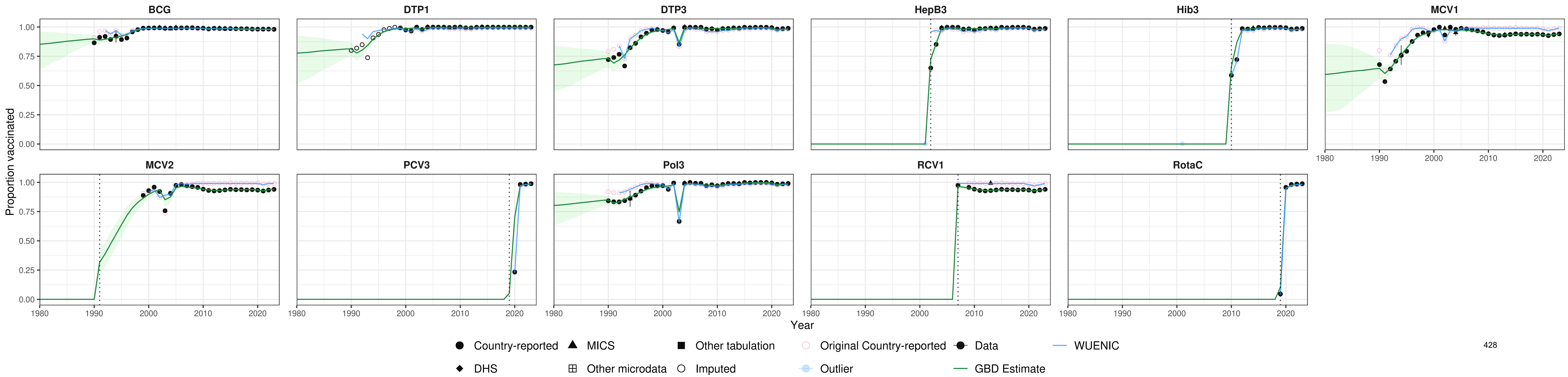

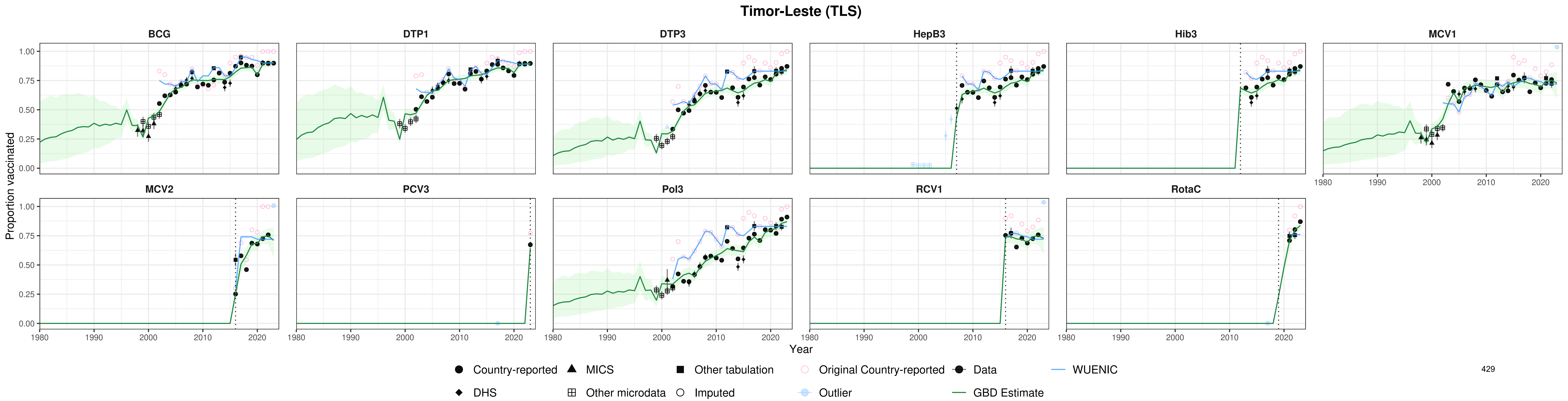

Timor-Leste (TLS)
BCG
DTP1
DTP3
HepB3
Hib3
MCV1
MCV2
PCV3
Pol3
RCV1
RotaC
Proportion vaccinated
Year
0.00
0.25
0.50
0.75
1.00
1980
1990
2000
2010
2020
Country-reported
MICS
Other tabulation
Original Country-reported
Data
WUENIC
DHS
Other microdata
Imputed
Outlier
GBD Estimate

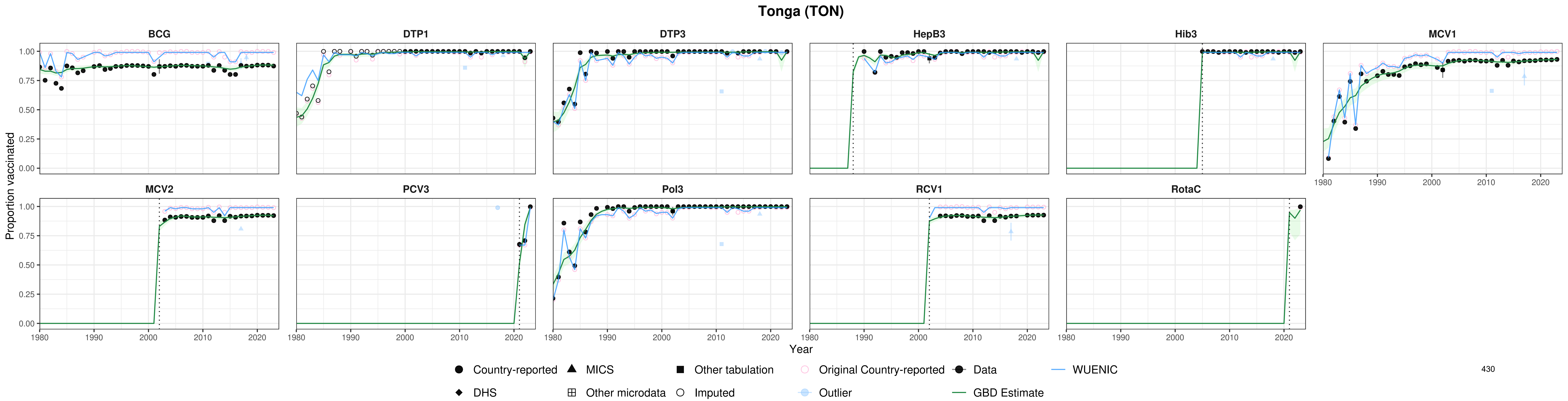
Tonga (TON)
BCG
DTP1
DTP3
HepB3
Hib3
MCV1
MCV2
PCV3
Pol3
RCV1
RotaC
Proportion vaccinated
Year
Country-reported
MICS
Other tabulation
Original Country-reported
Data
WUENIC
DHS
Other microdata
Imputed
Outlier
GBD Estimate

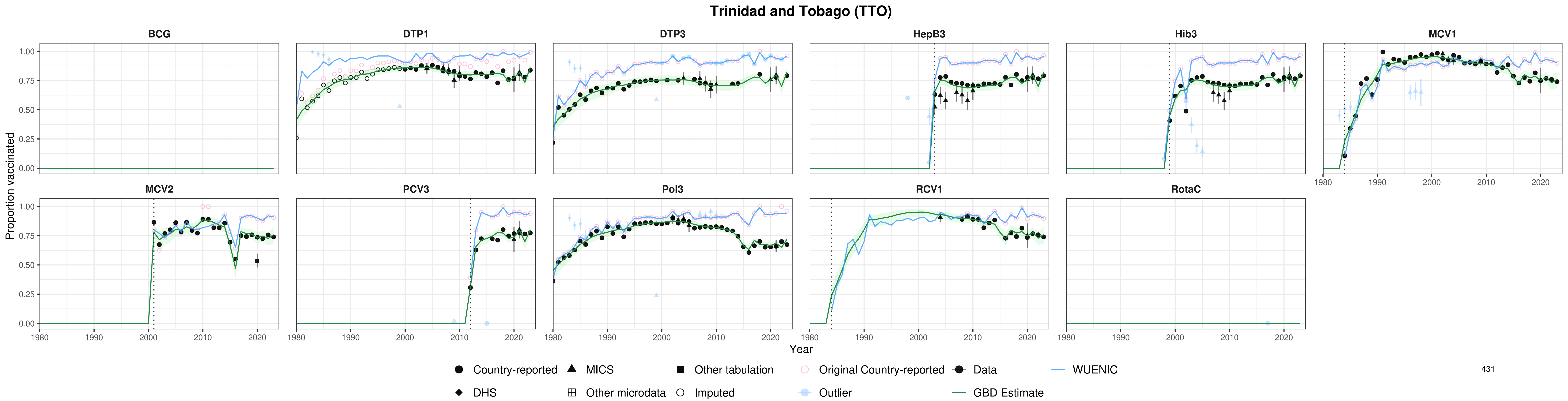

Trinidad and Tobago (TTO)
BCG
DTP1
DTP3
HepB3
Hib3
MCV1
MCV2
PCV3
Pol3
RCV1
RotaC
Proportion vaccinated
Year
1980
1990
2000
2010
2020
0.00
0.25
0.50
0.75
1.00
Country-reported
MICS
Other tabulation
Original Country-reported
Data
WUENIC
DHS
Other microdata
Imputed
Outlier
GBD Estimate

Tunisia (TUN)

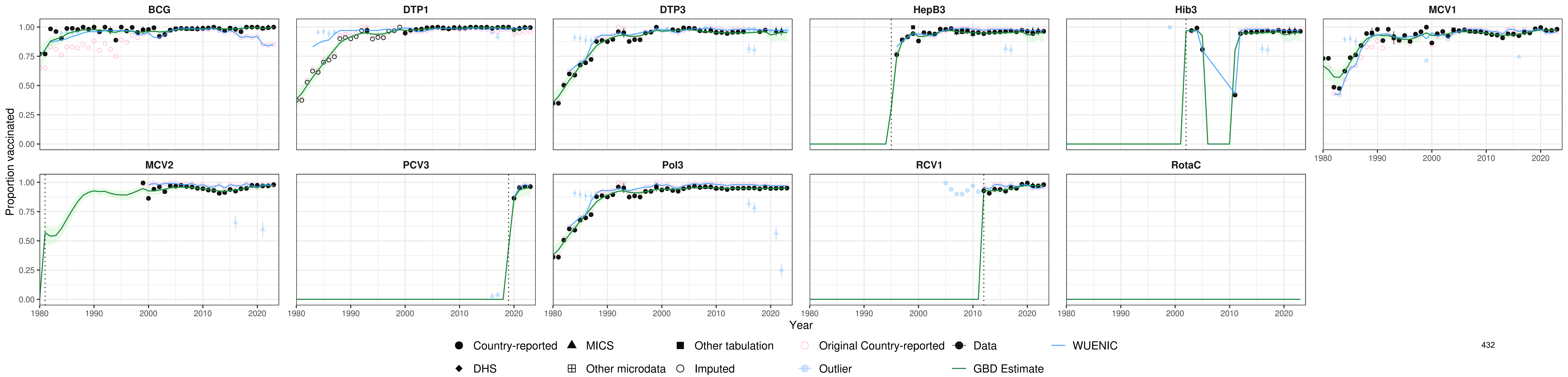

BCG
DTP1
DTP3
HepB3
Hib3
MCV1
MCV2
PCV3
Pol3
RCV1
RotaC
Proportion vaccinated
1.00
0.75
0.50
0.25
0.00
1980
1990
2000
2010
2020
Year
Country-reported
MICS
Other tabulation
Original Country-reported
Data
WUENIC
DHS
Other microdata
Imputed
Outlier
GBD Estimate

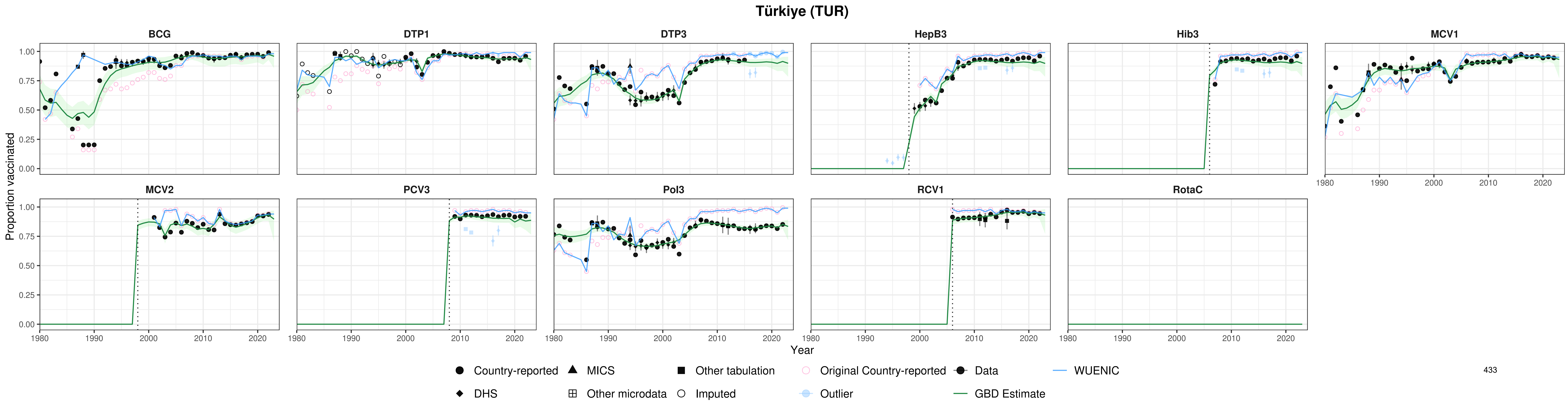

Türkiye (TUR)
BCG
DTP1
DTP3
HepB3
Hib3
MCV1
MCV2
PCV3
Pol3
RCV1
RotaC
Proportion vaccinated
Year
Country-reported
MICS
Other tabulation
Original Country-reported
Data
WUENIC
DHS
Other microdata
Imputed
Outlier
GBD Estimate

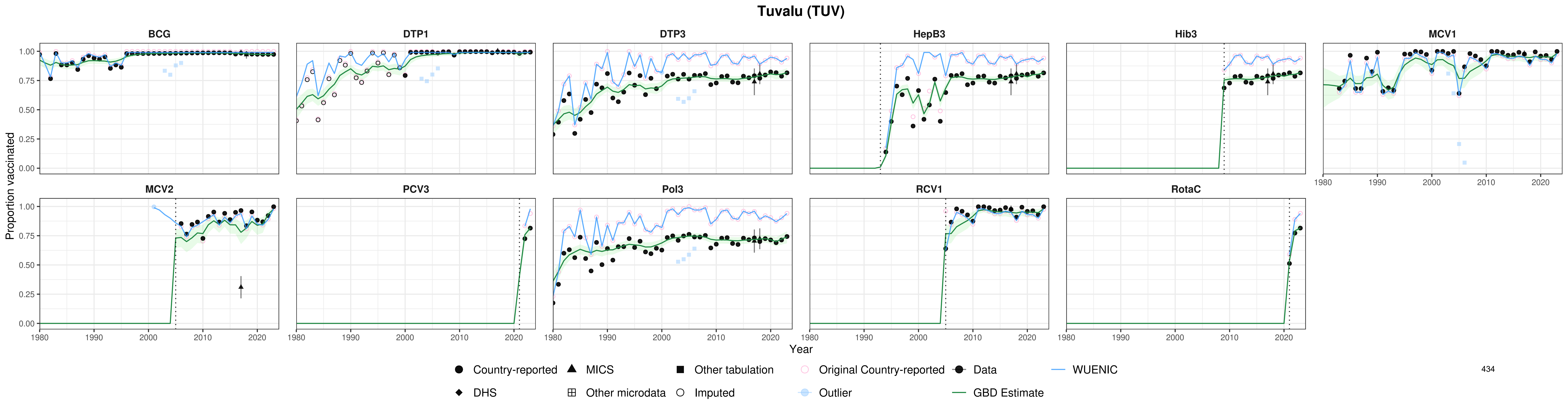
Tuvalu (TUV)
BCG
DTP1
DTP3
HepB3
Hib3
MCV1
MCV2
PCV3
Pol3
RCV1
RotaC
Proportion vaccinated
Year
1.00
0.75
0.50
0.25
0.00
1980
1990
2000
2010
2020
Country-reported
MICS
Other tabulation
Original Country-reported
Data
WUENIC
DHS
Other microdata
Imputed
Outlier
GBD Estimate

Taiwan (TWN)

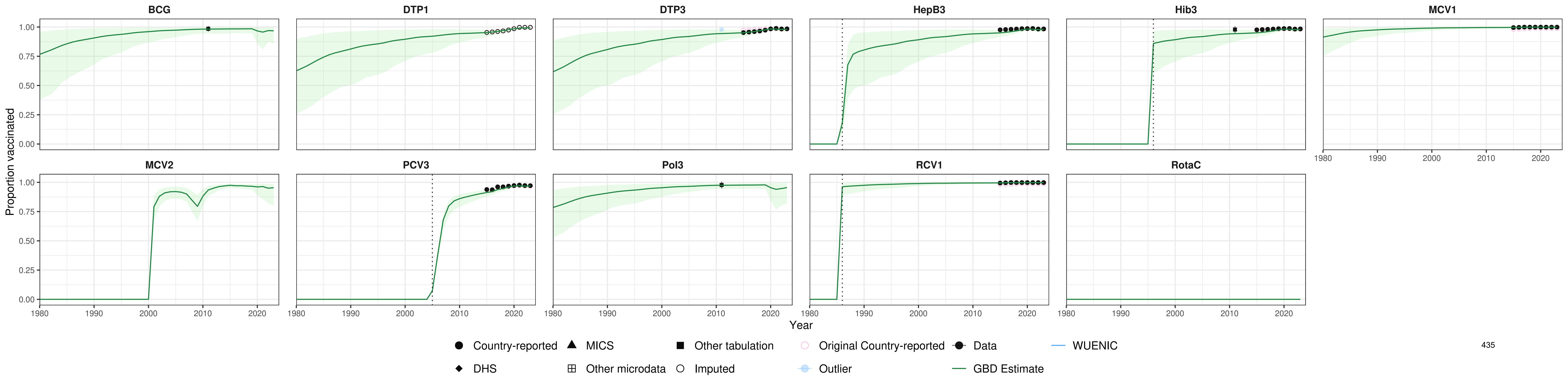

BCG
DTP1
DTP3
HepB3
Hib3
MCV1
MCV2
PCV3
Pol3
RCV1
RotaC
Proportion vaccinated
Year
Country-reported
MICS
Other tabulation
Original Country-reported
Data
WUENIC
DHS
Other microdata
Imputed
Outlier
GBD Estimate

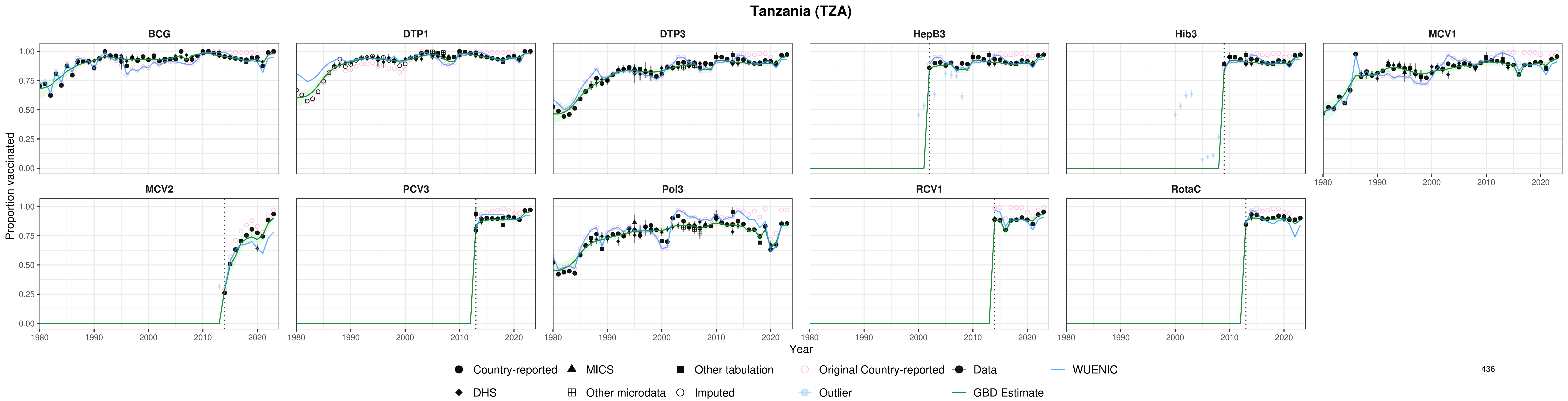

Tanzania (TZA)
BCG
DTP1
DTP3
HepB3
Hib3
MCV1
MCV2
PCV3
Pol3
RCV1
RotaC
Proportion vaccinated
Year
1.00
0.75
0.50
0.25
0.00
1980
1990
2000
2010
2020
Country-reported
MICS
Other tabulation
Original Country-reported
Data
WUENIC
DHS
Other microdata
Imputed
Outlier
GBD Estimate

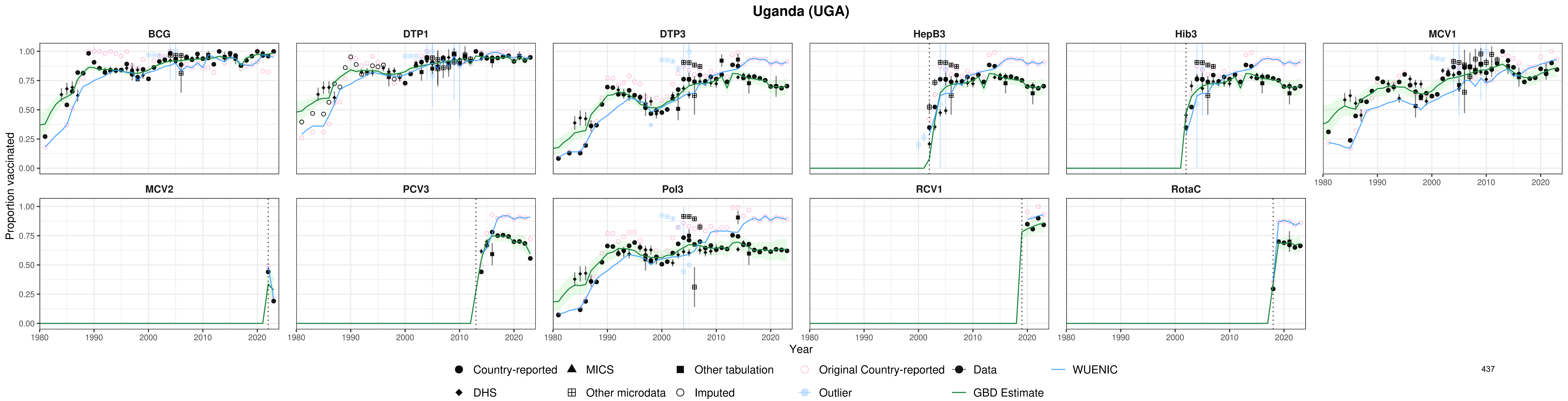

Uganda (UGA)
BCG
DTP1
DTP3
HepB3
Hib3
MCV1
MCV2
PCV3
Pol3
RCV1
RotaC
Proportion vaccinated
Year
Country-reported
DHS
MICS
Other microdata
Other tabulation
Imputed
Original Country-reported
Outlier
Data
GBD Estimate
WUENIC

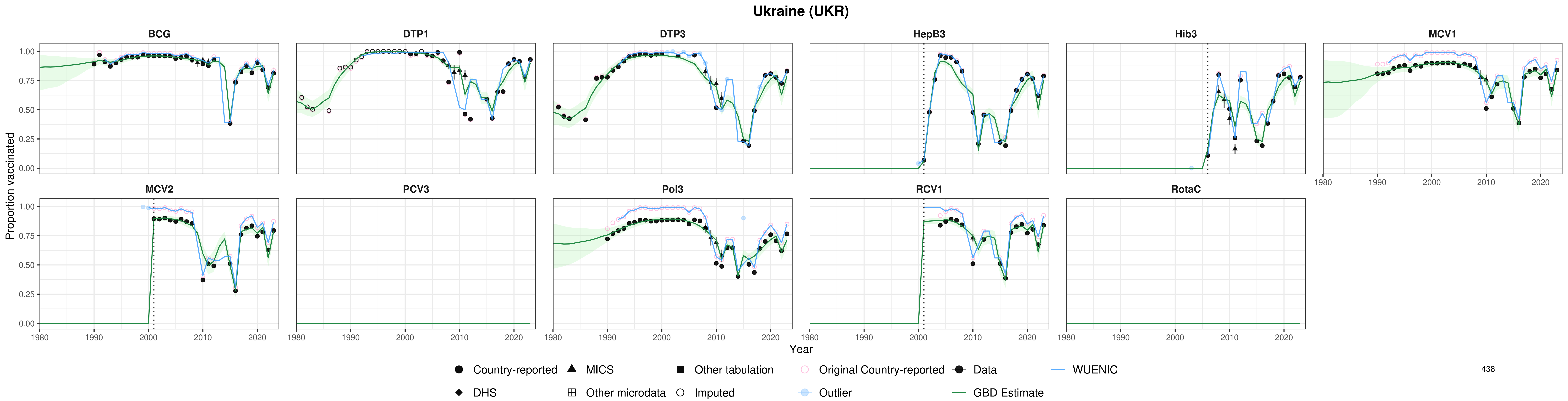

Ukraine (UKR)
BCG
DTP1
DTP3
HepB3
Hib3
MCV1
MCV2
PCV3
Pol3
RCV1
RotaC
Proportion vaccinated
Year
Country-reported
MICS
Other tabulation
Original Country-reported
Data
WUENIC
DHS
Other microdata
Imputed
Outlier
GBD Estimate

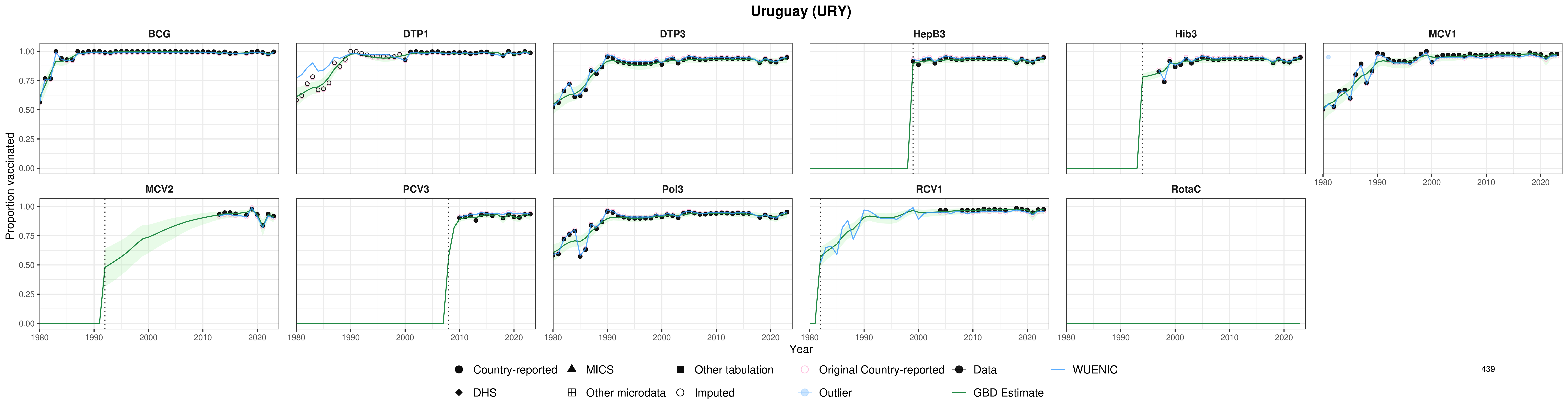

Uruguay (URY)
BCG
DTP1
DTP3
HepB3
Hib3
MCV1
MCV2
PCV3
Pol3
RCV1
RotaC
Proportion vaccinated
Year
Country-reported
MICS
Other tabulation
Original Country-reported
Data
WUENIC
DHS
Other microdata
Imputed
Outlier
GBD Estimate

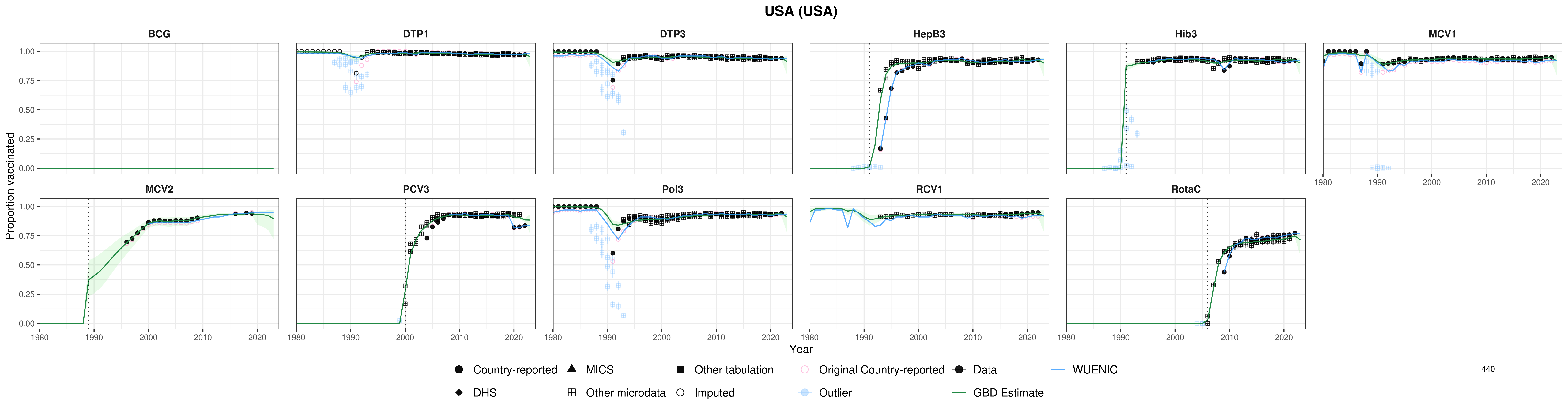
USA (USA)
BCG
DTP1
DTP3
HepB3
Hib3
MCV1
MCV2
PCV3
Pol3
RCV1
RotaC
Proportion vaccinated
Year
Country-reported
MICS
Other tabulation
Original Country-reported
Data
WUENIC
DHS
Other microdata
Imputed
Outlier
GBD Estimate

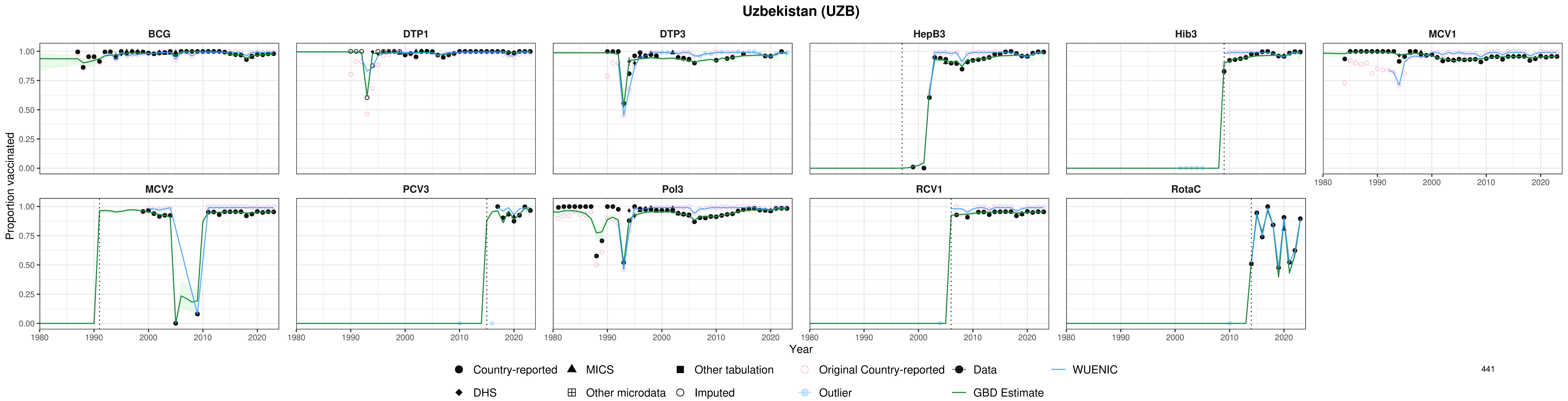

Uzbekistan (UZB)
BCG
DTP1
DTP3
HepB3
Hib3
MCV1
MCV2
PCV3
Pol3
RCV1
RotaC
Proportion vaccinated
Year
Country-reported
MICS
Other tabulation
Original Country-reported
Data
WUENIC
DHS
Other microdata
Imputed
Outlier
GBD Estimate

Saint Vincent and the Grenadines (VCT)

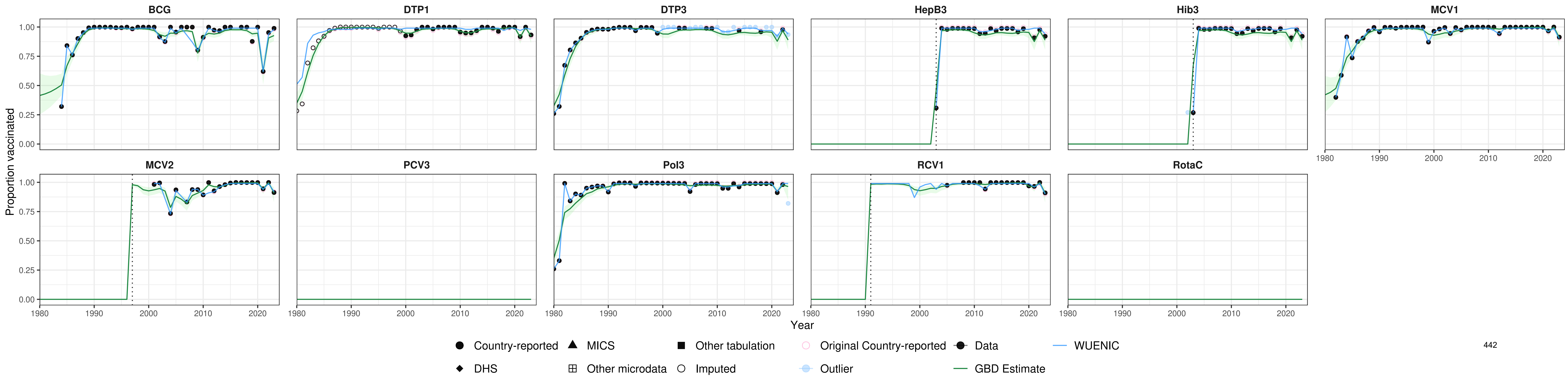

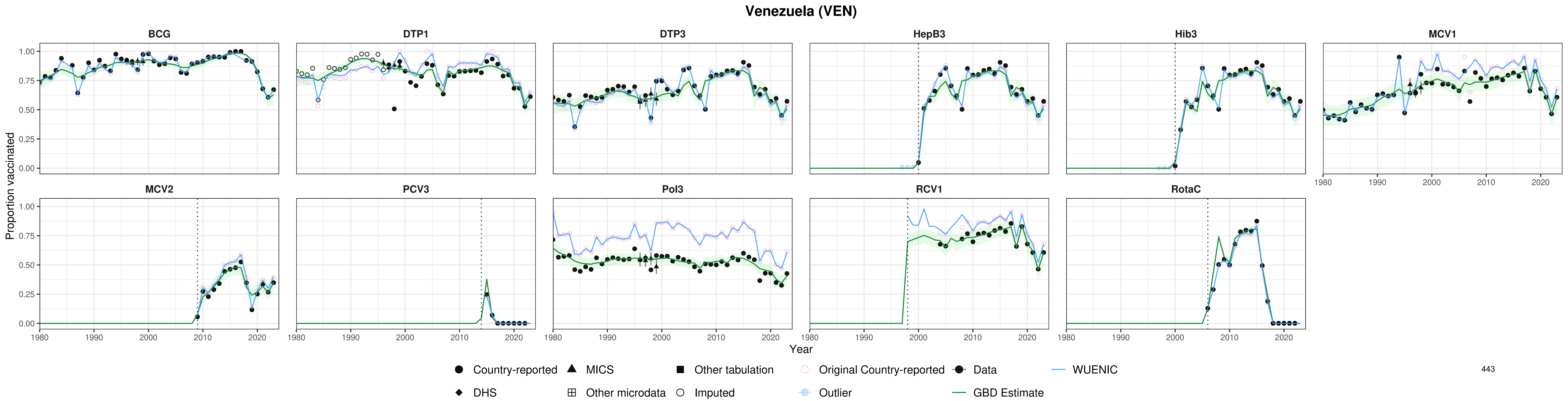
Venezuela (VEN)
BCG
DTP1
DTP3
HepB3
Hib3
MCV1
MCV2
PCV3
Pol3
RCV1
RotaC
Proportion vaccinated
Year
Country-reported
MICS
Other tabulation
Original Country-reported
Data
WUENIC
DHS
Other microdata
Imputed
Outlier
GBD Estimate

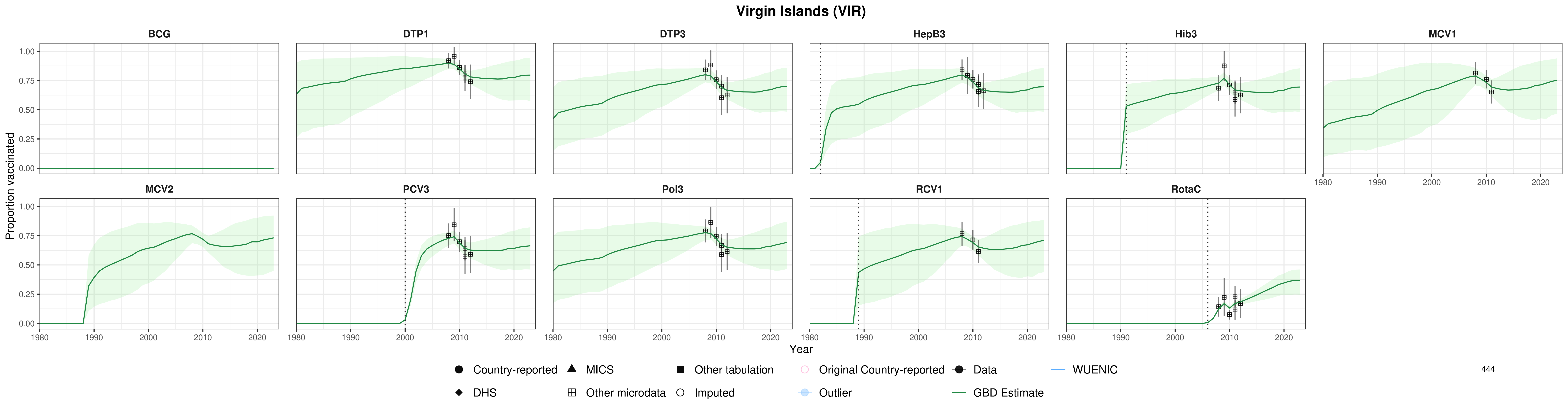
Virgin Islands (VIR)
BCG
DTP1
DTP3
HepB3
Hib3
MCV1
MCV2
PCV3
Pol3
RCV1
RotaC
Proportion vaccinated
Year
Country-reported
MICS
Other tabulation
Original Country-reported
Data
WUENIC
DHS
Other microdata
Imputed
Outlier
GBD Estimate

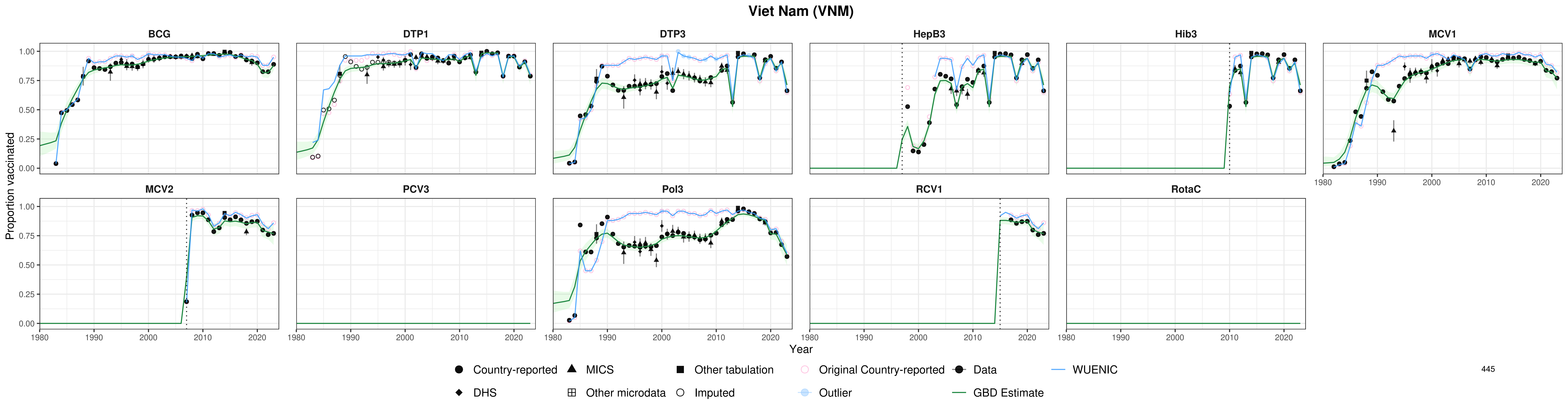
Viet Nam (VNM)
BCG
DTP1
DTP3
HepB3
Hib3
MCV1
MCV2
PCV3
Pol3
RCV1
RotaC
Proportion vaccinated
0.00
0.25
0.50
0.75
1.00
1980
1990
2000
2010
2020
Year
Country-reported
MICS
Other tabulation
Original Country-reported
Data
WUENIC
DHS
Other microdata
Imputed
Outlier
GBD Estimate

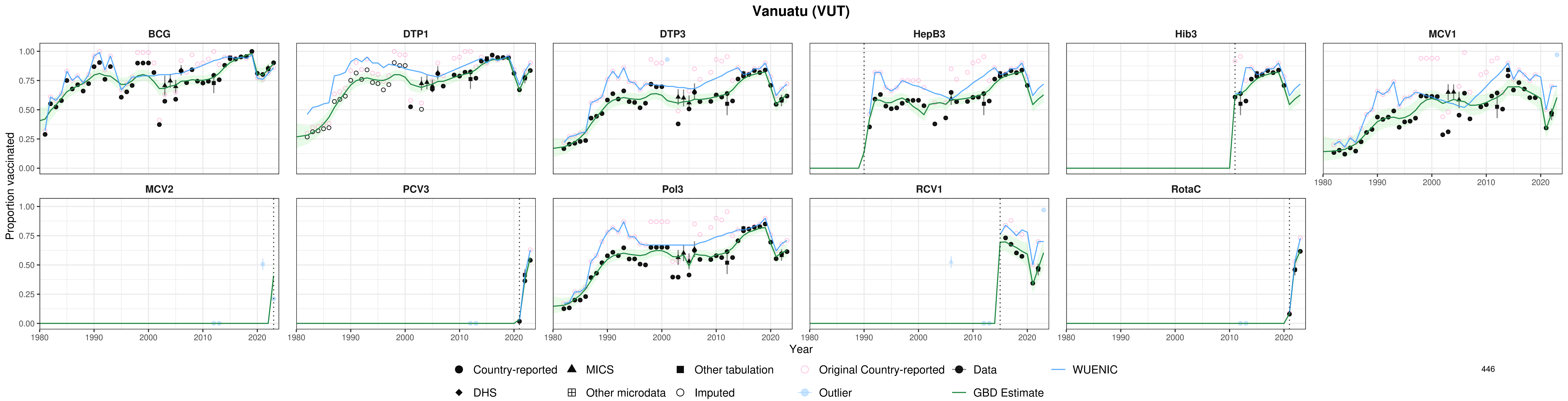

Vanuatu (VUT)
BCG
DTP1
DTP3
HepB3
Hib3
MCV1
MCV2
PCV3
Pol3
RCV1
RotaC
Proportion vaccinated
Year
0.00
0.25
0.50
0.75
1.00
1980
1990
2000
2010
2020
Country-reported
MICS
Other tabulation
Original Country-reported
Data
WUENIC
DHS
Other microdata
Imputed
Outlier
GBD Estimate

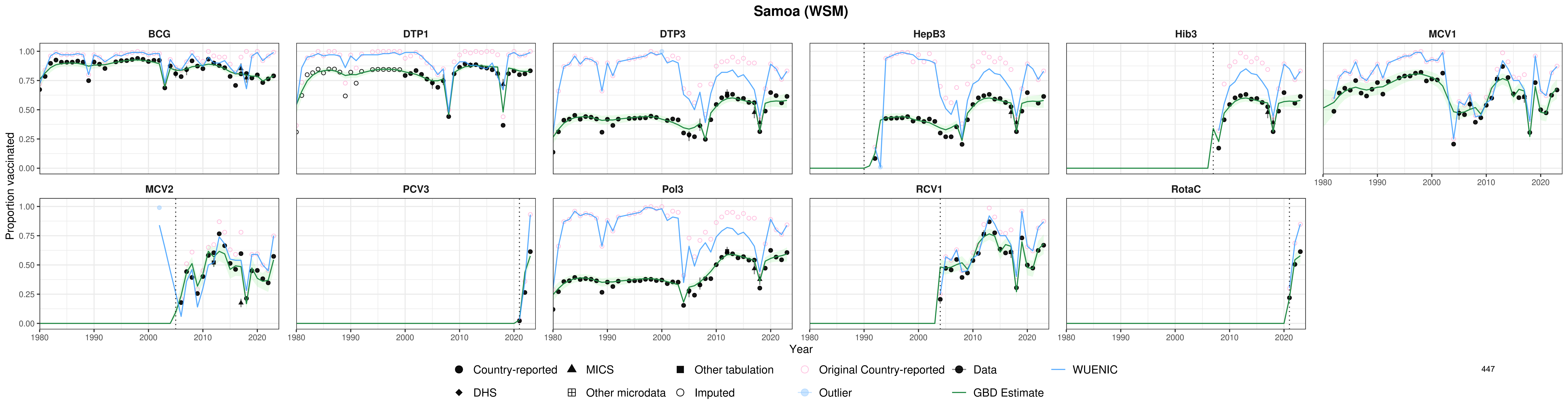

Samoa (WSM)
BCG
DTP1
DTP3
HepB3
Hib3
MCV1
MCV2
PCV3
Pol3
RCV1
RotaC
Proportion vaccinated
Year
0.00
0.25
0.50
0.75
1.00
1980
1990
2000
2010
2020
Country-reported
MICS
Other tabulation
Original Country-reported
Data
WUENIC
DHS
Other microdata
Imputed
Outlier
GBD Estimate

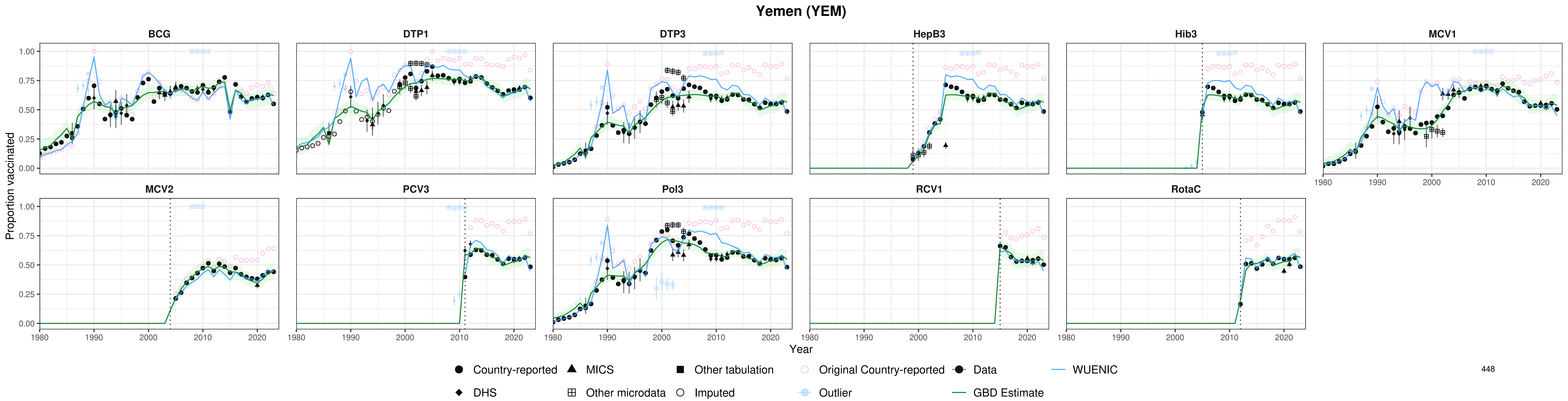

Yemen (YEM)
BCG
DTP1
DTP3
HepB3
Hib3
MCV1
MCV2
PCV3
Pol3
RCV1
RotaC
Proportion vaccinated
Year
0.00
0.25
0.50
0.75
1.00
1980
1990
2000
2010
2020
Country-reported
MICS
Other tabulation
Original Country-reported
Data
WUENIC
DHS
Other microdata
Imputed
Outlier
GBD Estimate

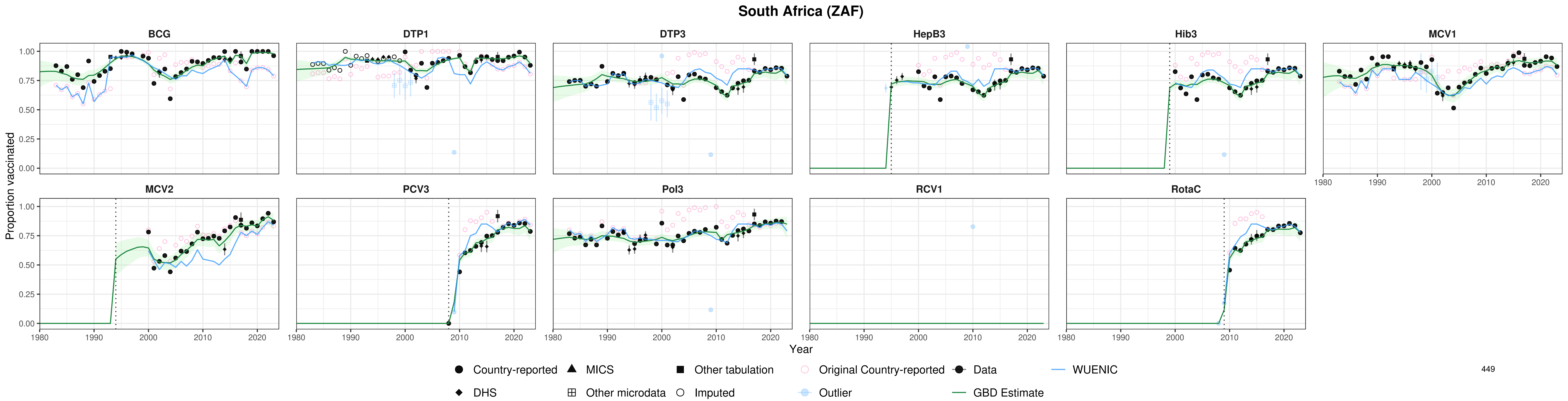
South Africa (ZAF)
BCG
DTP1
DTP3
HepB3
Hib3
MCV1
MCV2
PCV3
Pol3
RCV1
RotaC
Proportion vaccinated
Year
Country-reported
MICS
Other tabulation
Original Country-reported
Data
WUENIC
DHS
Other microdata
Imputed
Outlier
GBD Estimate

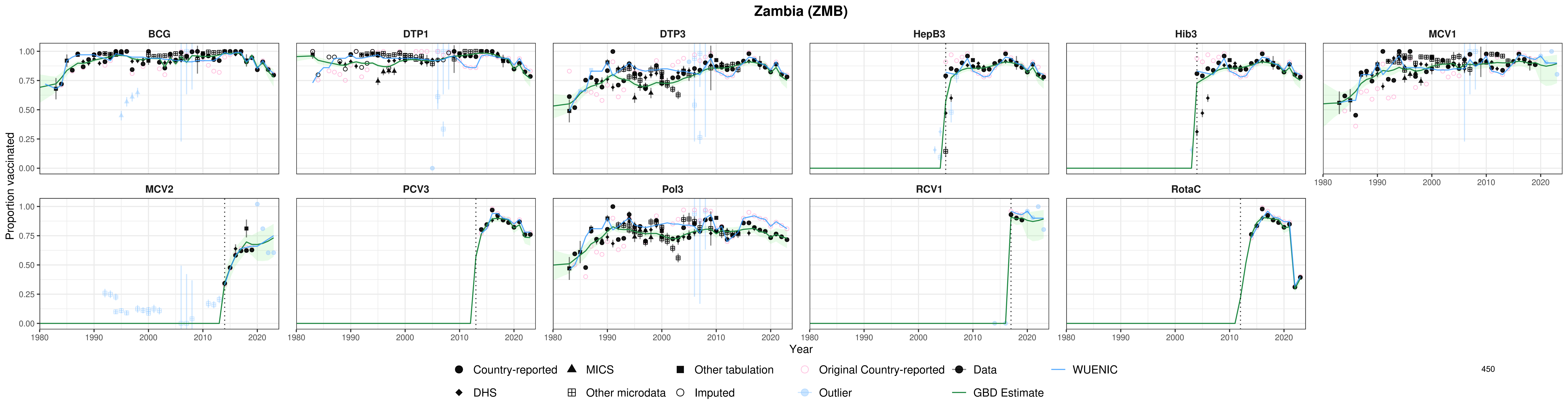
Zambia (ZMB)
BCG
DTP1
DTP3
HepB3
Hib3
MCV1
MCV2
PCV3
Pol3
RCV1
RotaC
Proportion vaccinated
Year
0.00
0.25
0.50
0.75
1.00
1980
1990
2000
2010
2020
Country-reported
MICS
Other tabulation
Original Country-reported
Data
WUENIC
DHS
Other microdata
Imputed
Outlier
GBD Estimate

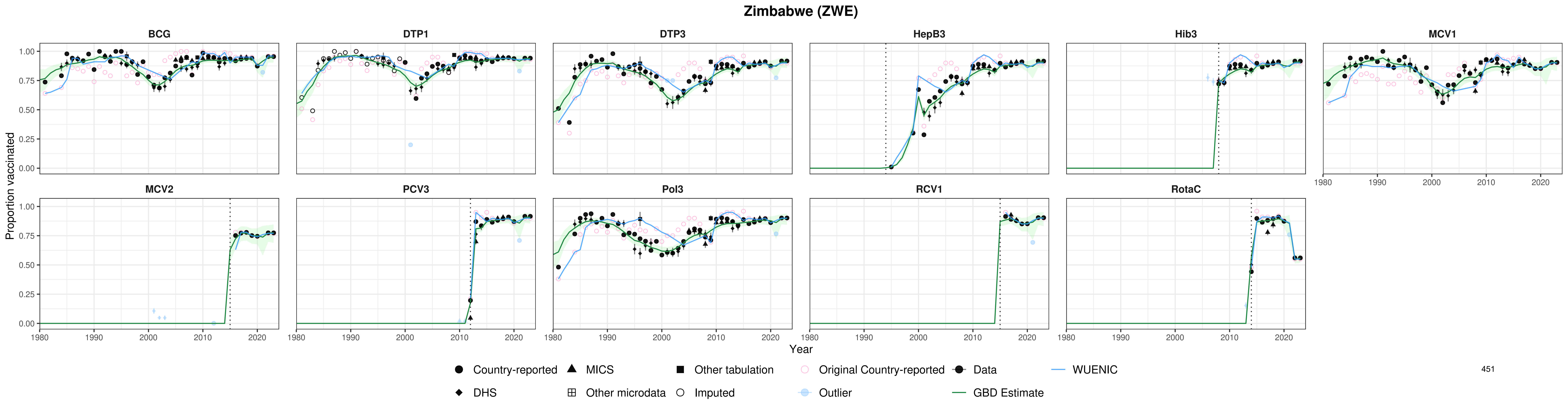
Zimbabwe (ZWE)
BCG
DTP1
DTP3
HepB3
Hib3
MCV1
MCV2
PCV3
Pol3
RCV1
RotaC
Proportion vaccinated
Year
Country-reported
MICS
Other tabulation
Original Country-reported
Data
WUENIC
DHS
Other microdata
Imputed
Outlier
GBD Estimate

Figure S6 Vaccine coverage maps

For all vaccines (BCG, DTP1, DTP3, HepB3, Hib3, MCV1, MCV2, PCV3, Pol3, RCV1, and RotaC), maps showing vaccine coverage for the target population for select years (1980, 1990, 2000, 2010, 2019, 2020, 2021, 2022, and 2023). Dark blue indicates high coverage, dark red indicates low-to-no coverage, light grey indicates the vaccine pre-introduction year, and dark grey indicates no data Insets magnify coverage in smaller countries and territories. BCG=Bacillus Calmette-Guérin. DTP1=diphtheria-tetanus-pertussis, first dose. DTP3=diphtheria-tetanus-pertussis, third dose. HepB3=hepatitis B vaccine, third dose. Hib3=Haemophilus influenzae type b vaccine, third dose. MCV1=measles-containing vaccine, first dose. MCV2=measles-containing vaccine, second dose. PCV3=pneumococcal conjugate vaccine, third dose. Pol3=polio vaccine, third dose. RCV1=rubella-containing vaccine, first dose. RotaC=completed rotavirus series.

BCG : 1980

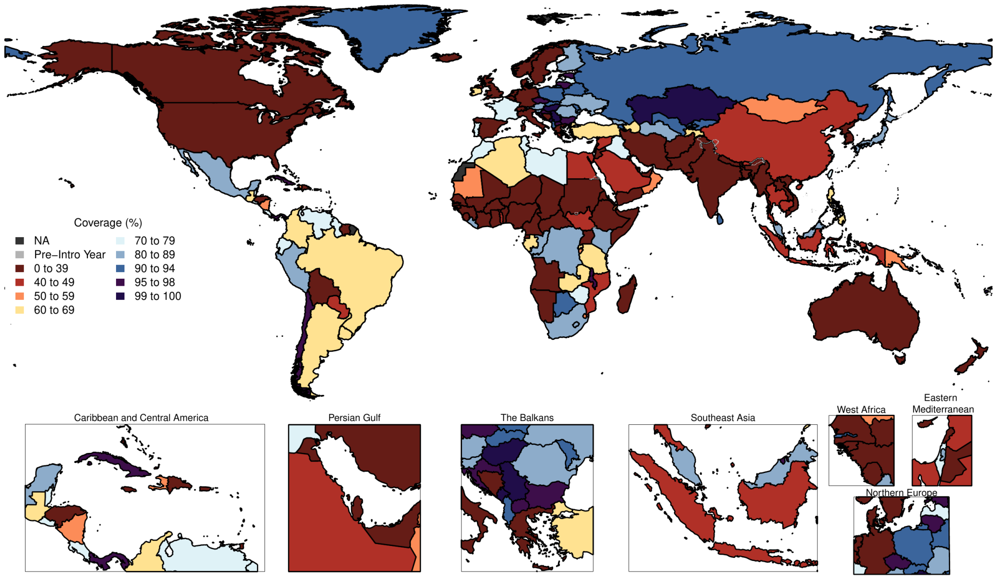

BCG : 1990

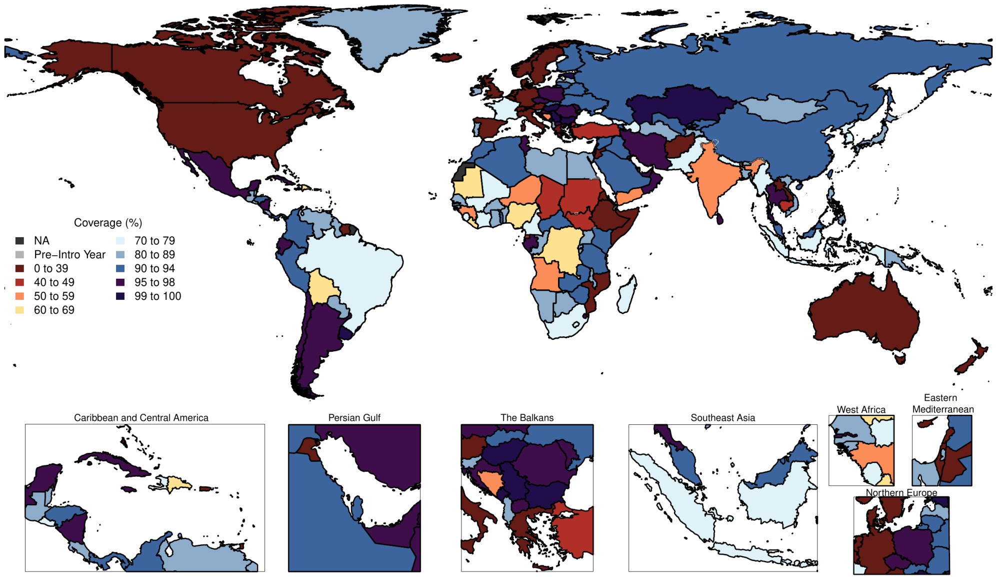

BCG : 2000

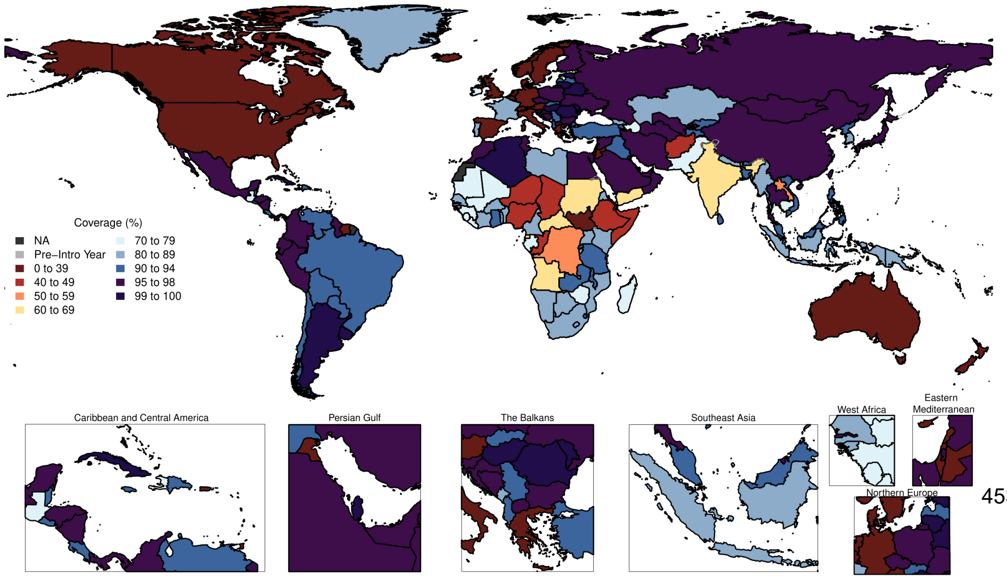

BCG : 2010

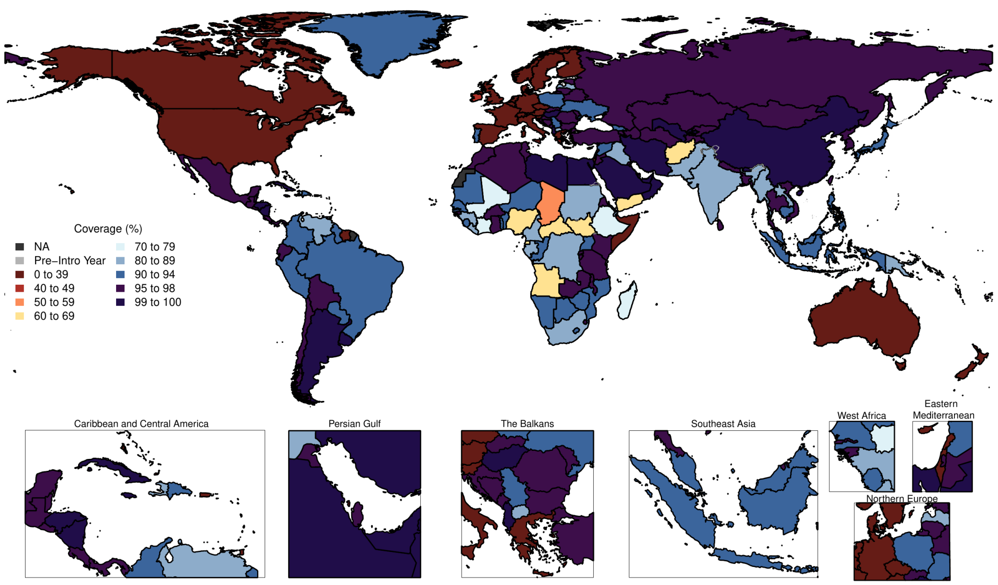

BCG : 2019

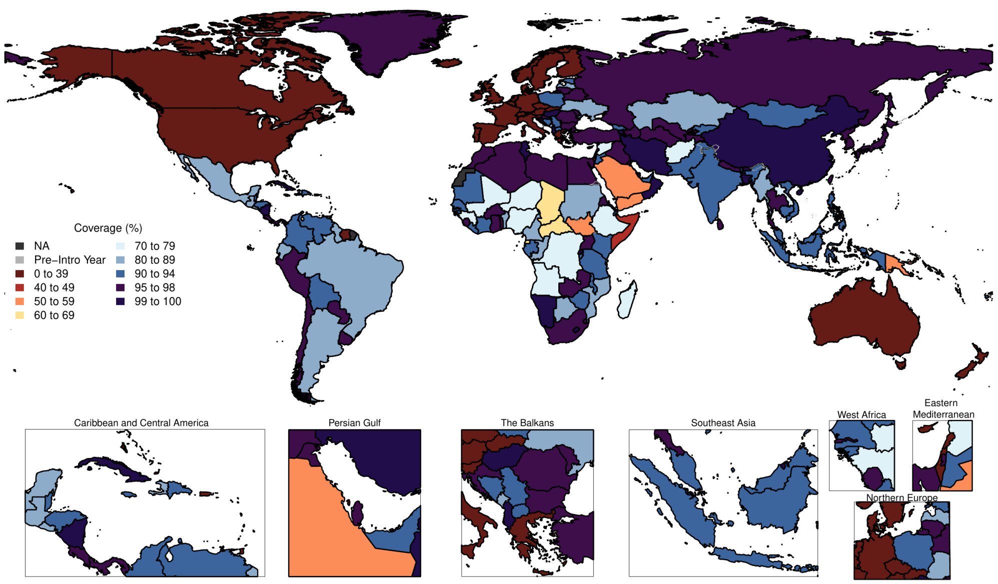

BCG : 2020

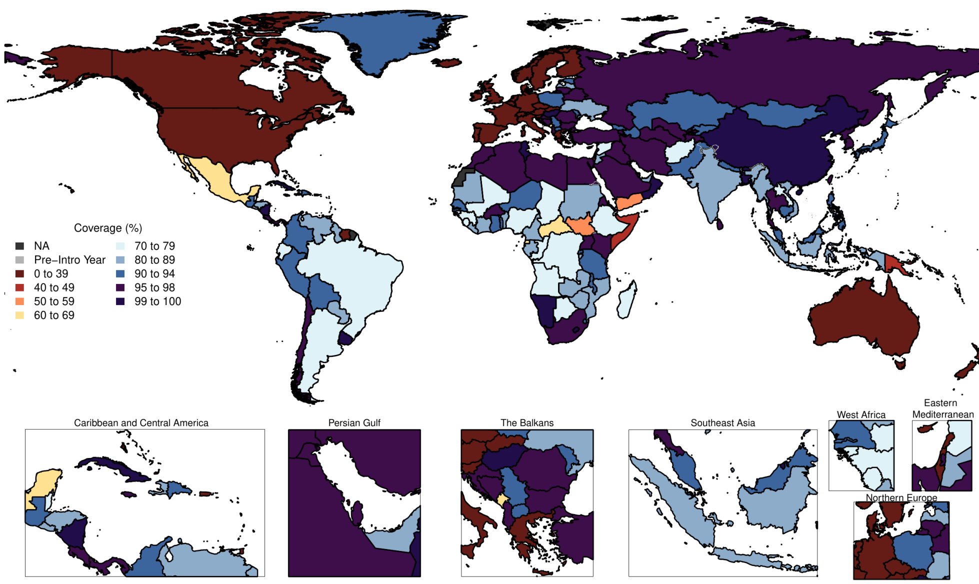

BCG : 2021

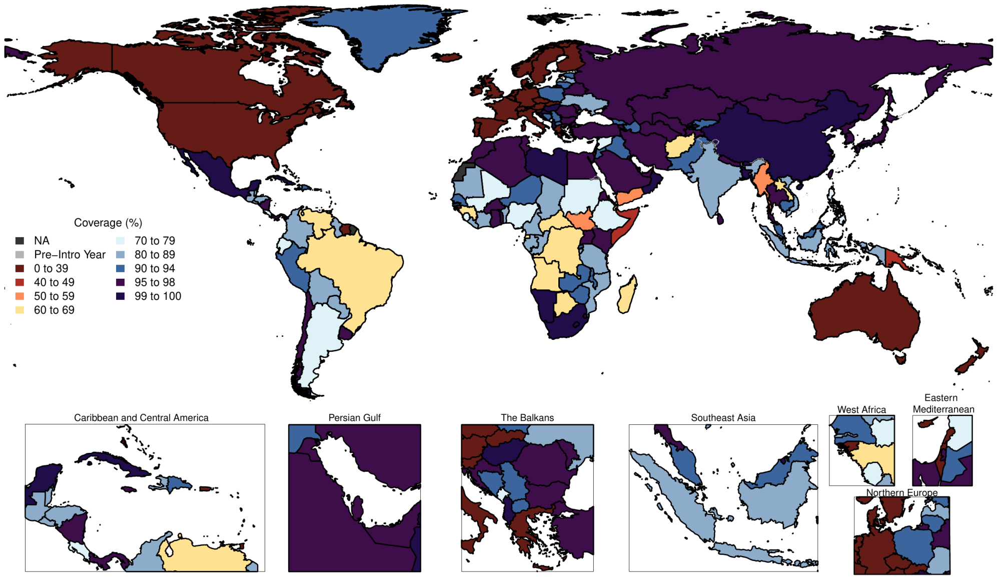

BCG : 2022

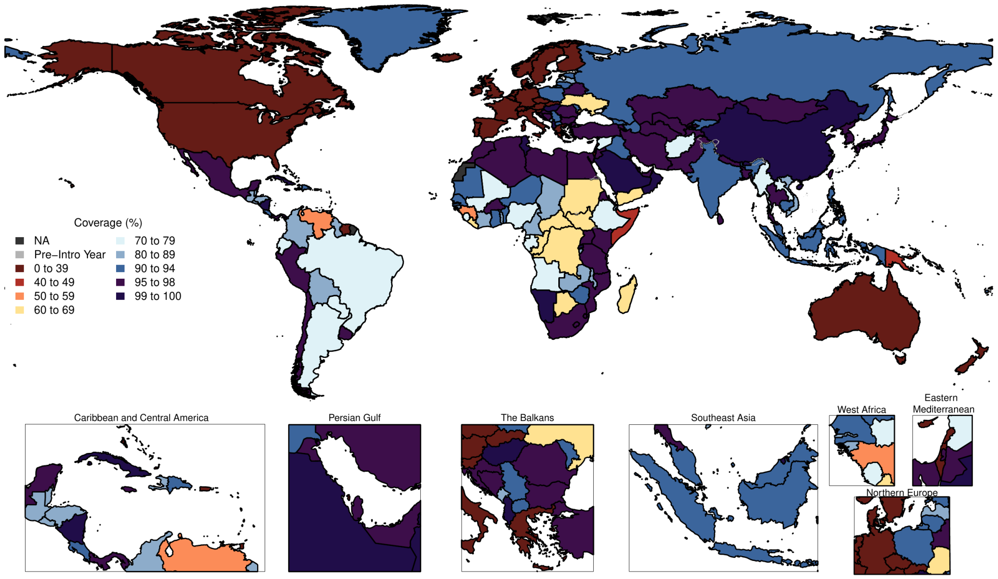

BCG : 2023

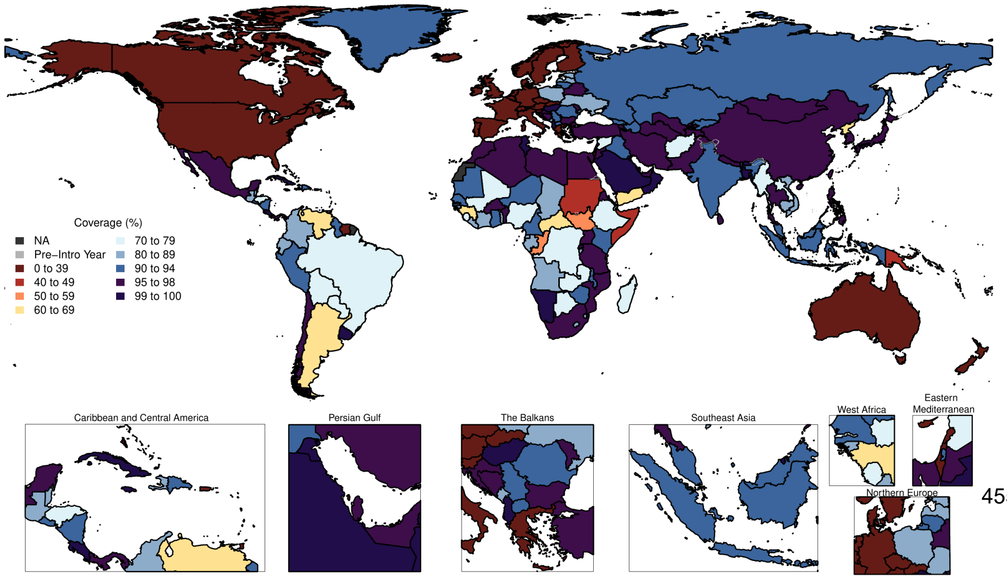

DTP1 : 1980

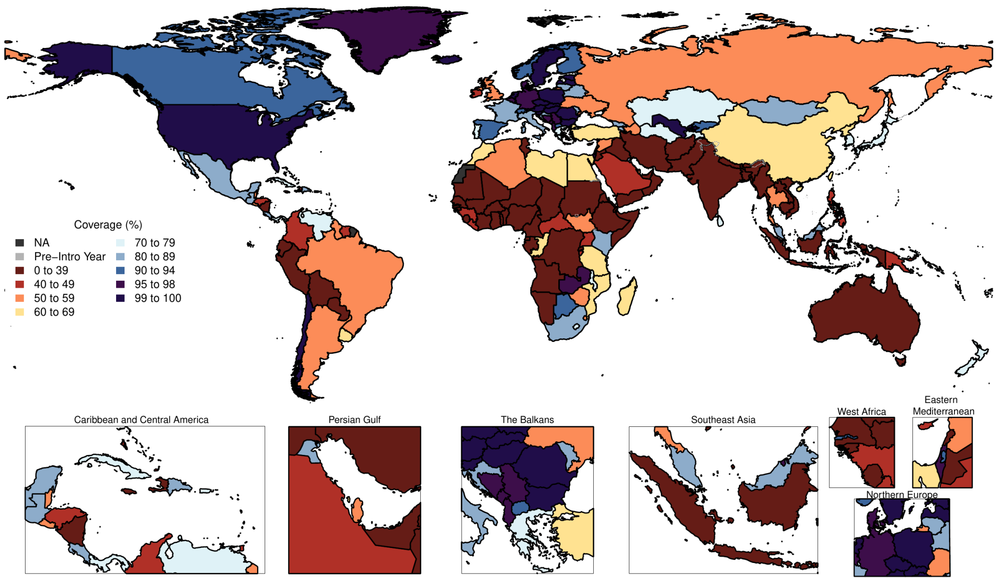

DTP1 : 1990

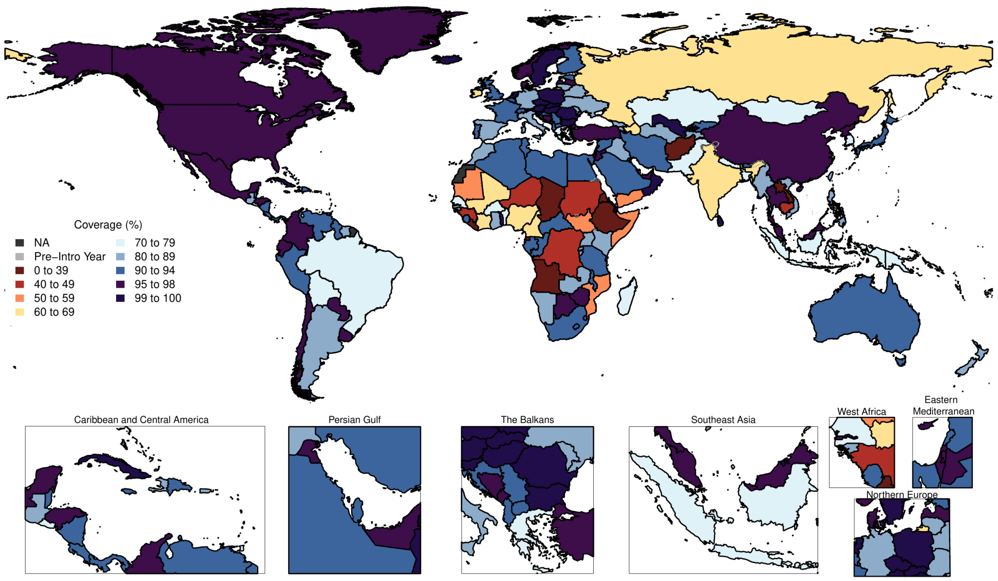

DTP1 : 2000

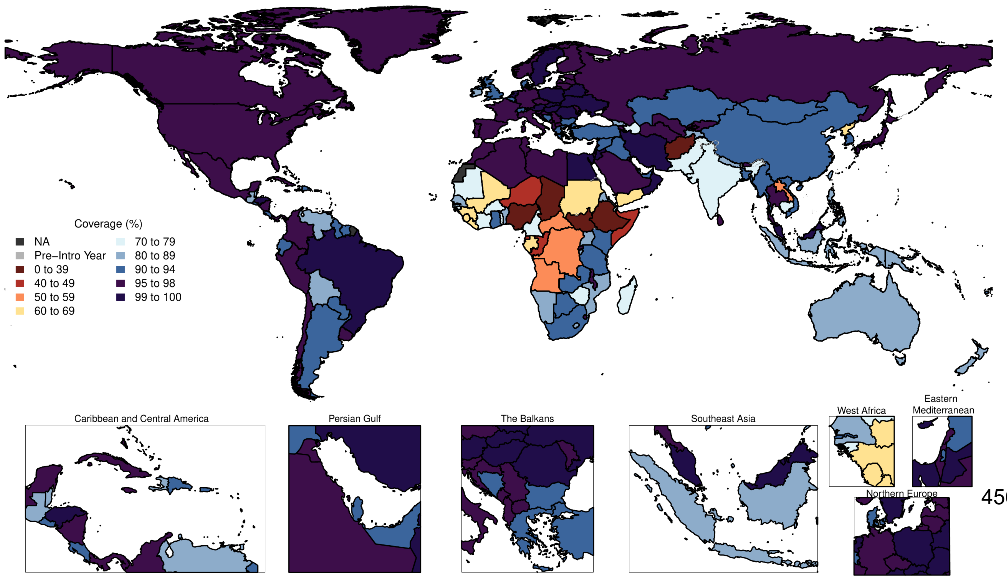

DTP1 : 2010

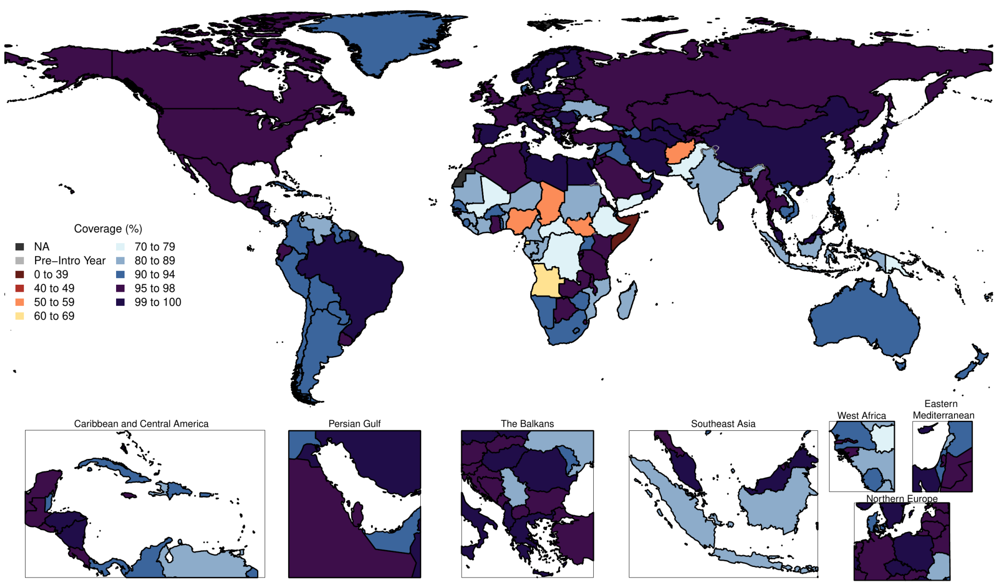

DTP1 : 2019

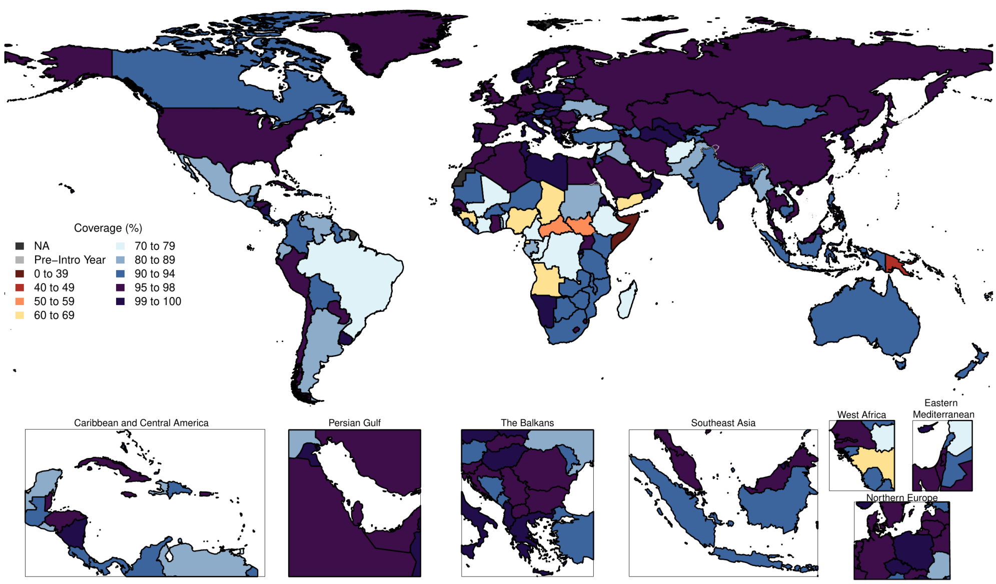

DTP1 : 2020

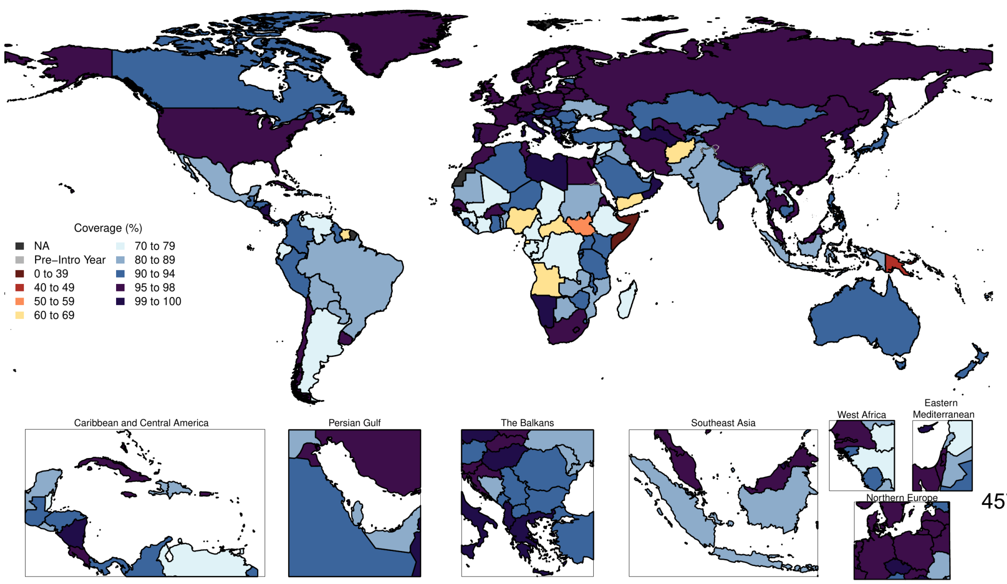

DTP1 : 2021

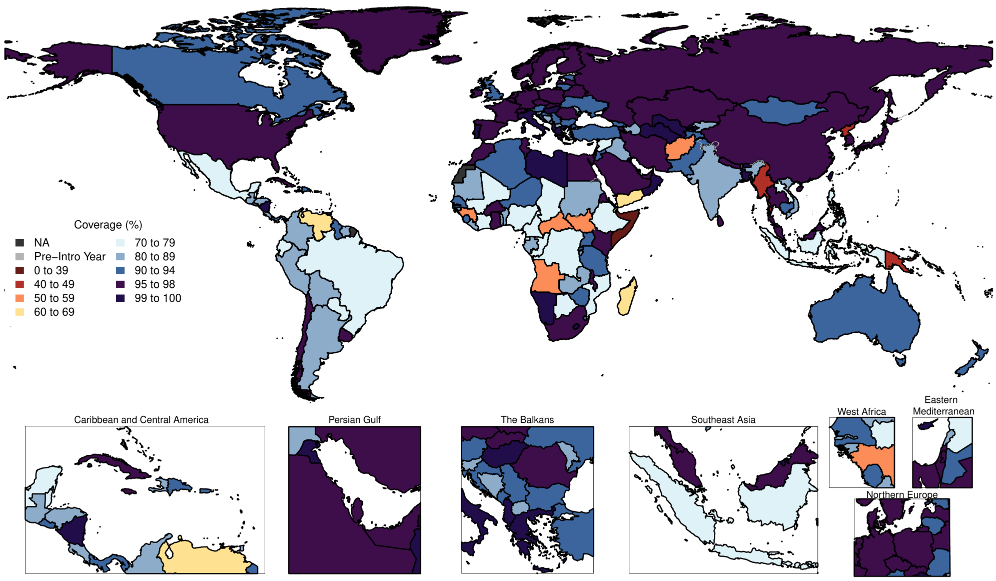

DTP1 : 2022

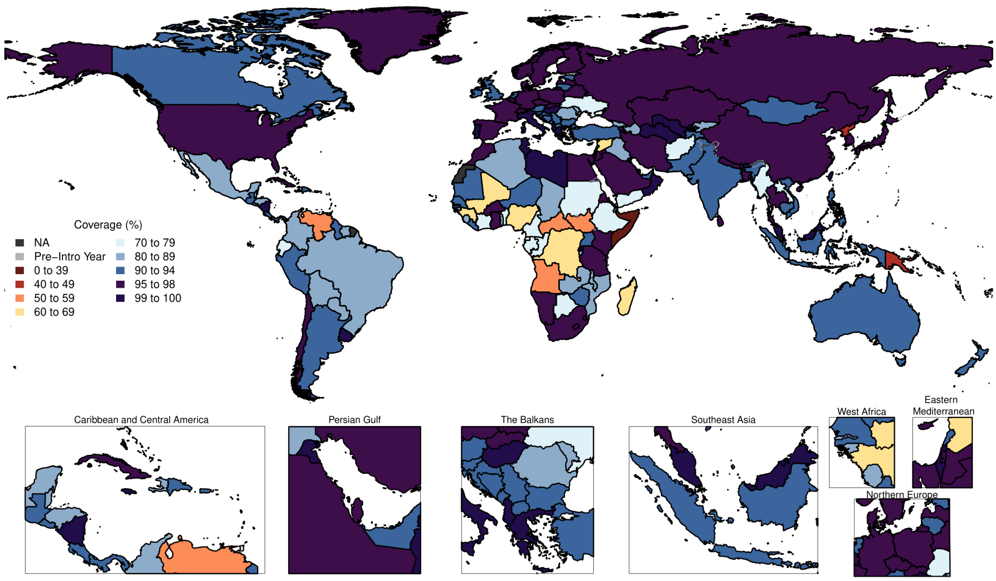

DTP1 : 2023

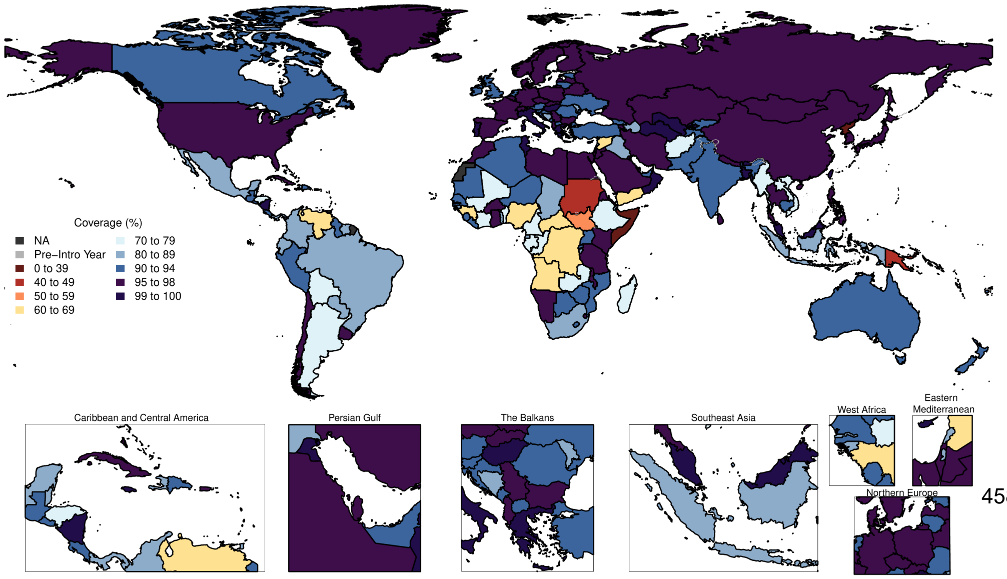

DTP3 : 1980

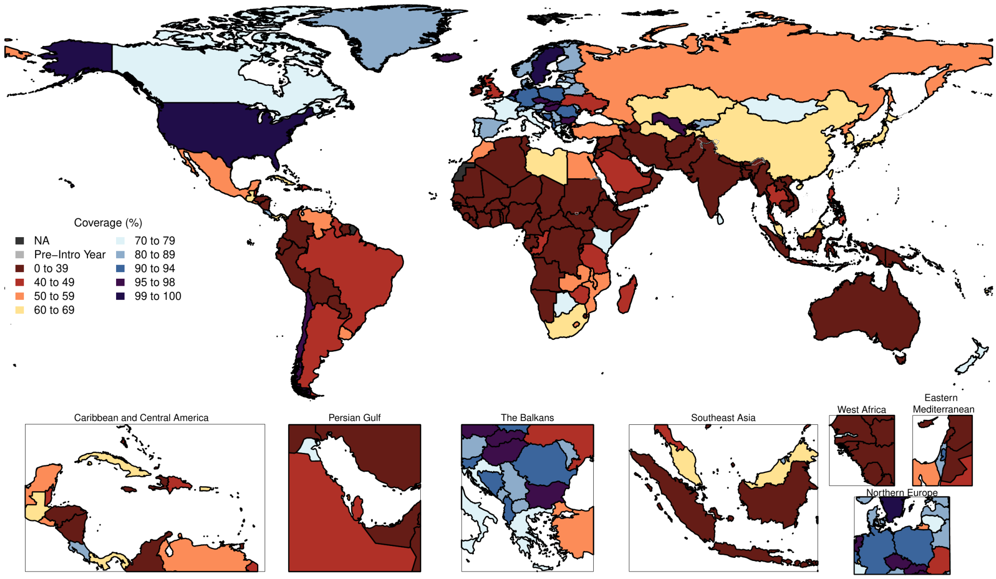

DTP3 : 1990

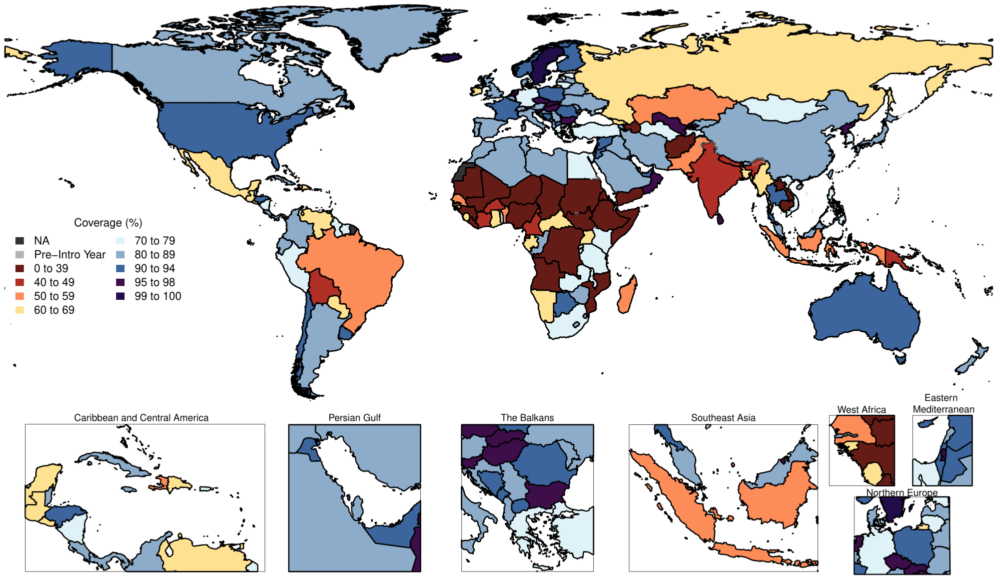

DTP3 : 2000

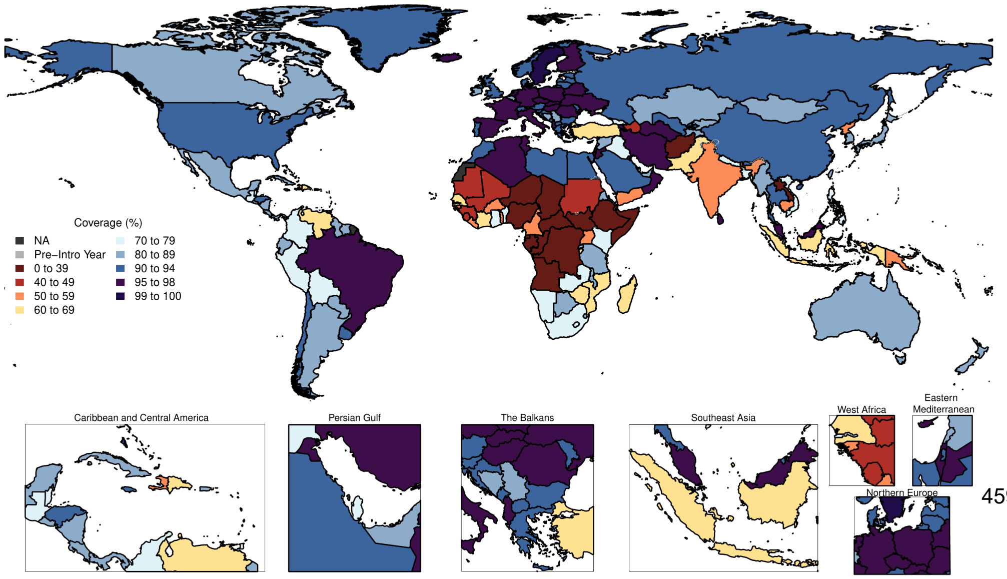

DTP3 : 2010

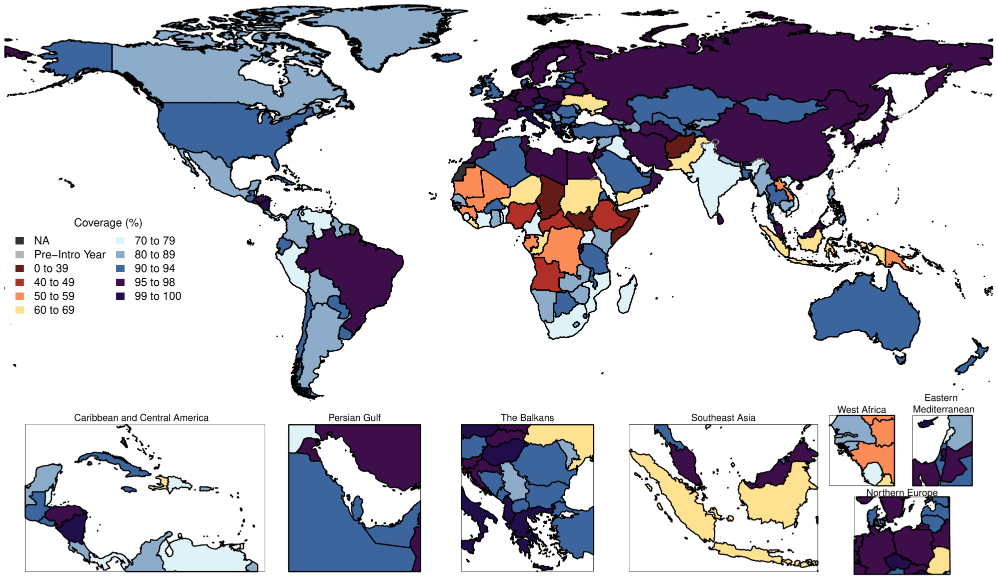

DTP3 : 2019

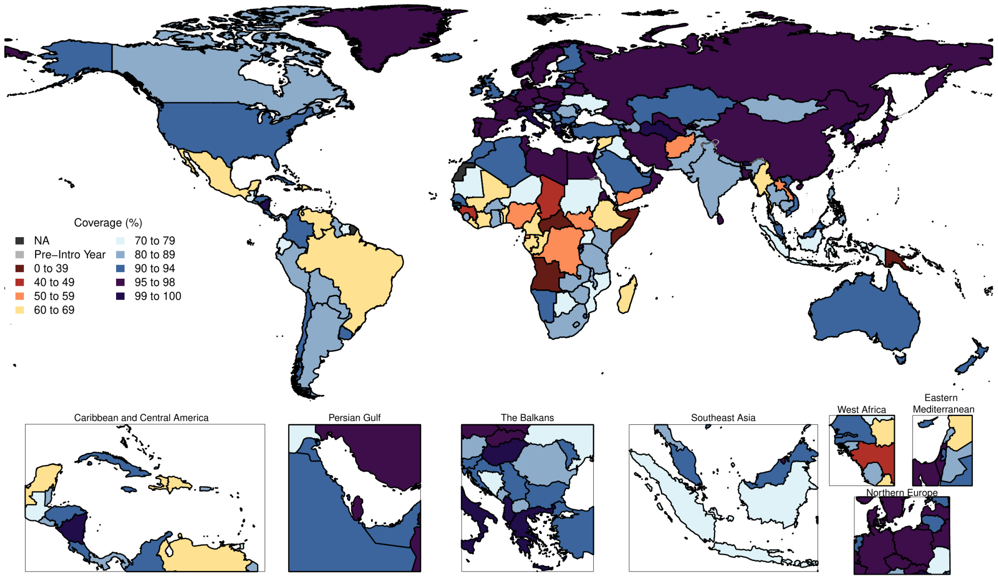

DTP3 : 2020

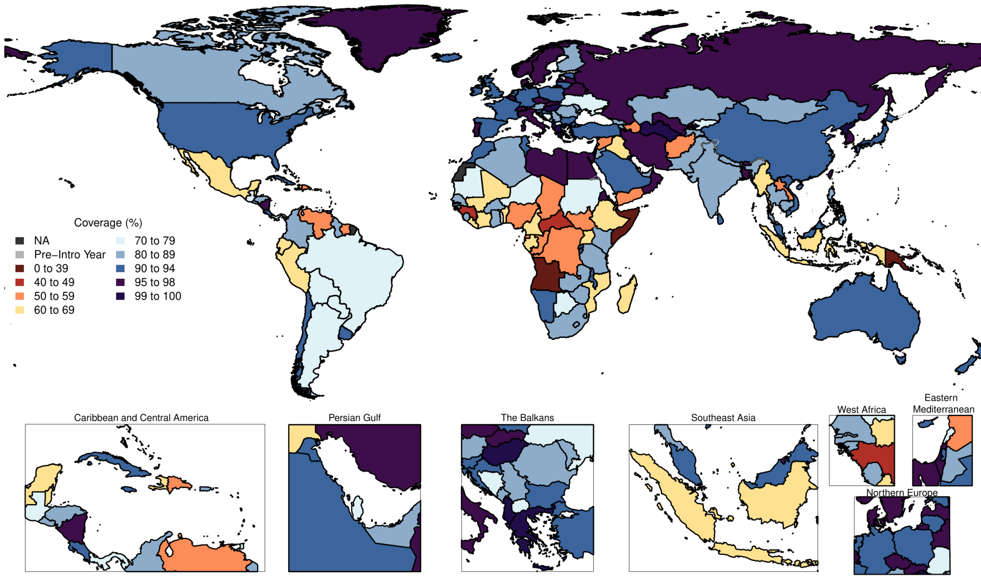

DTP3 : 2021

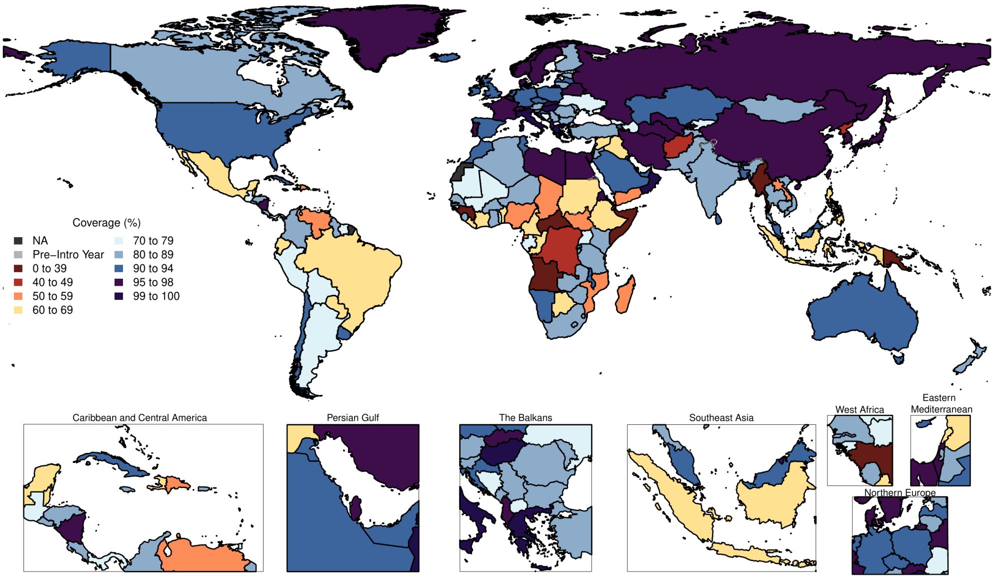

DTP3 : 2022

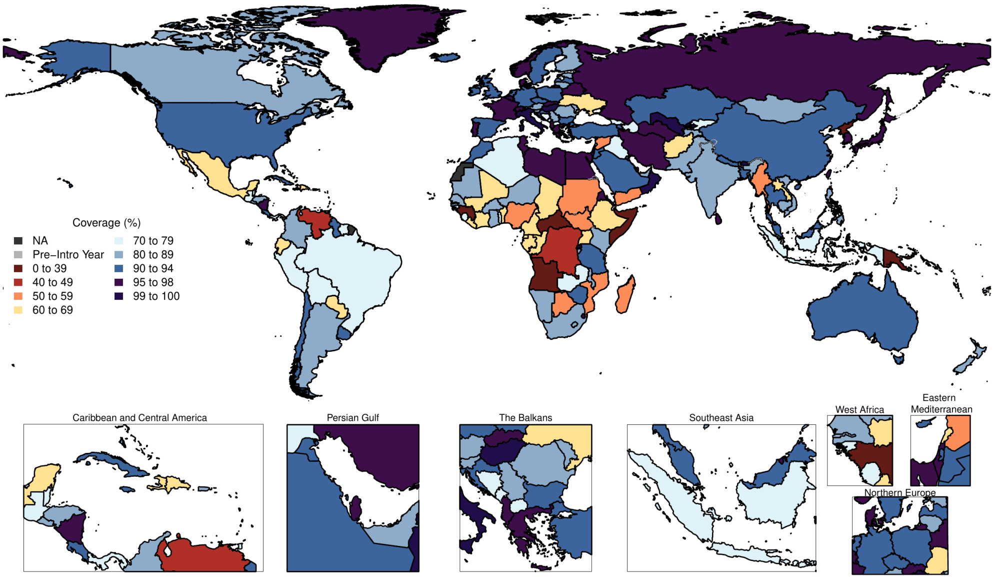

DTP3 : 2023

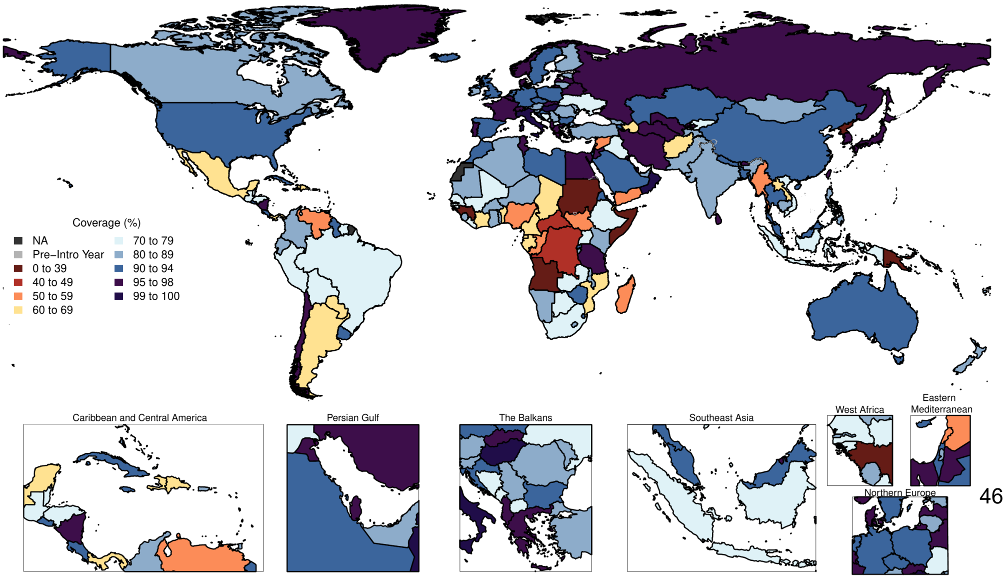

HepB3 : 1980

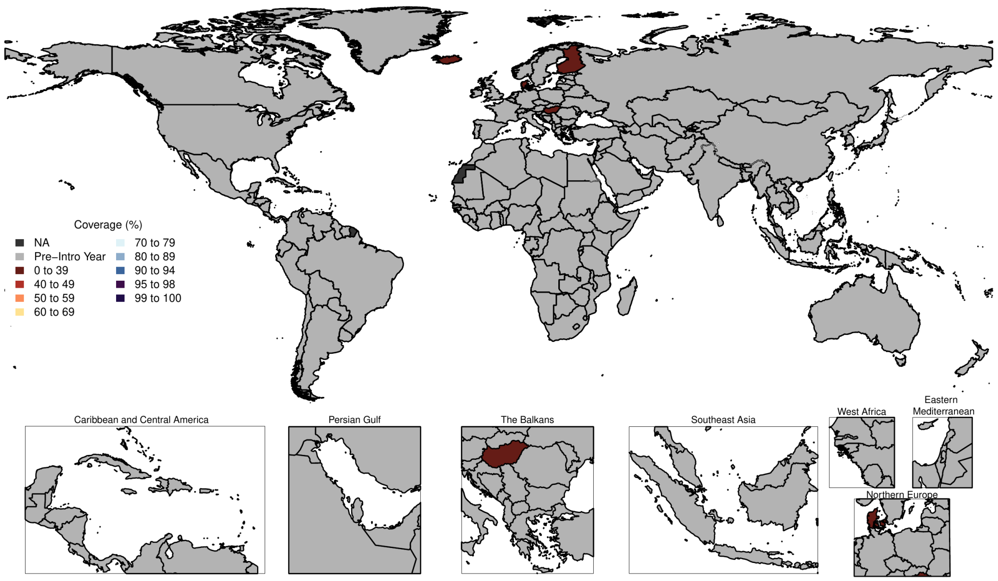

HepB3 : 1990

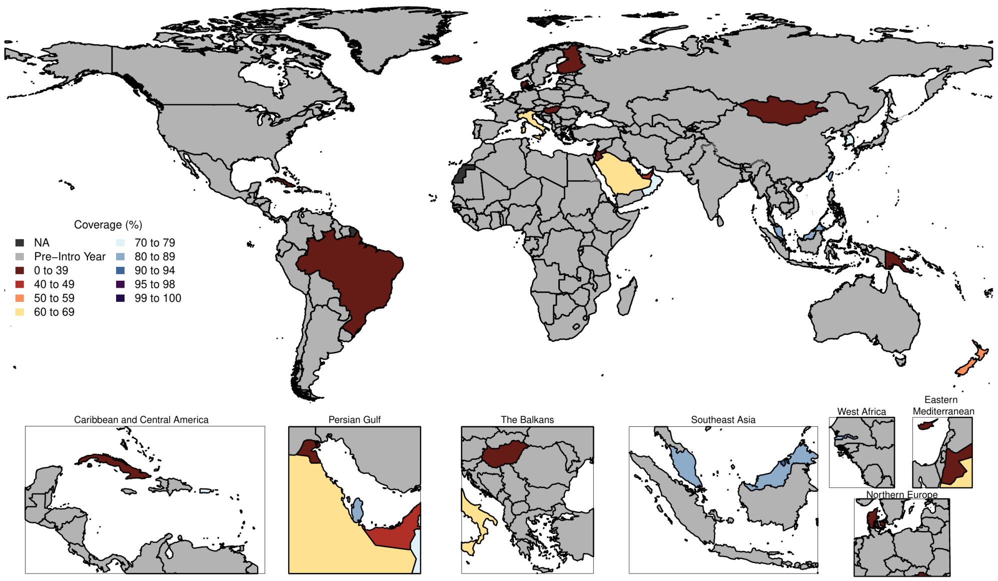

HepB3 : 2000

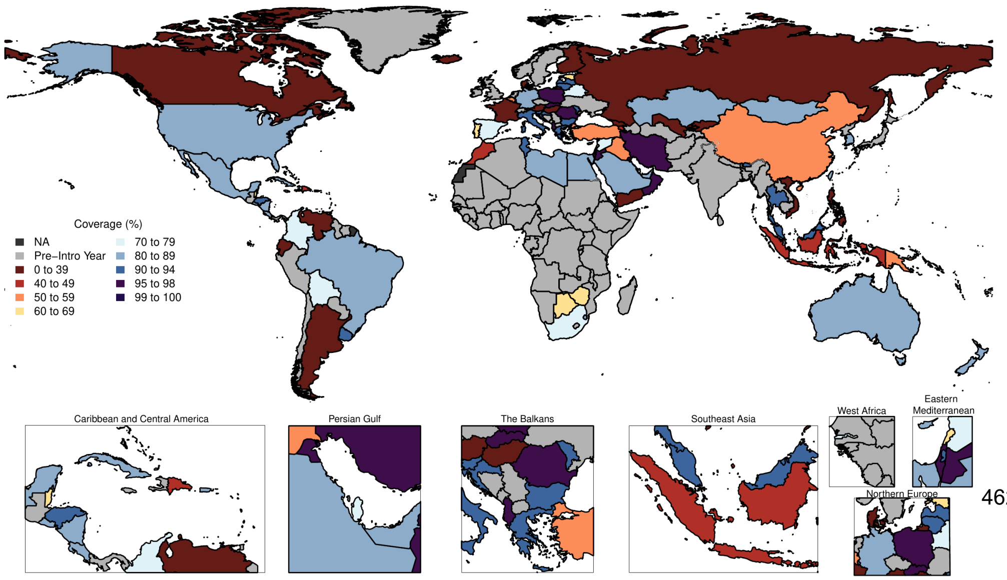

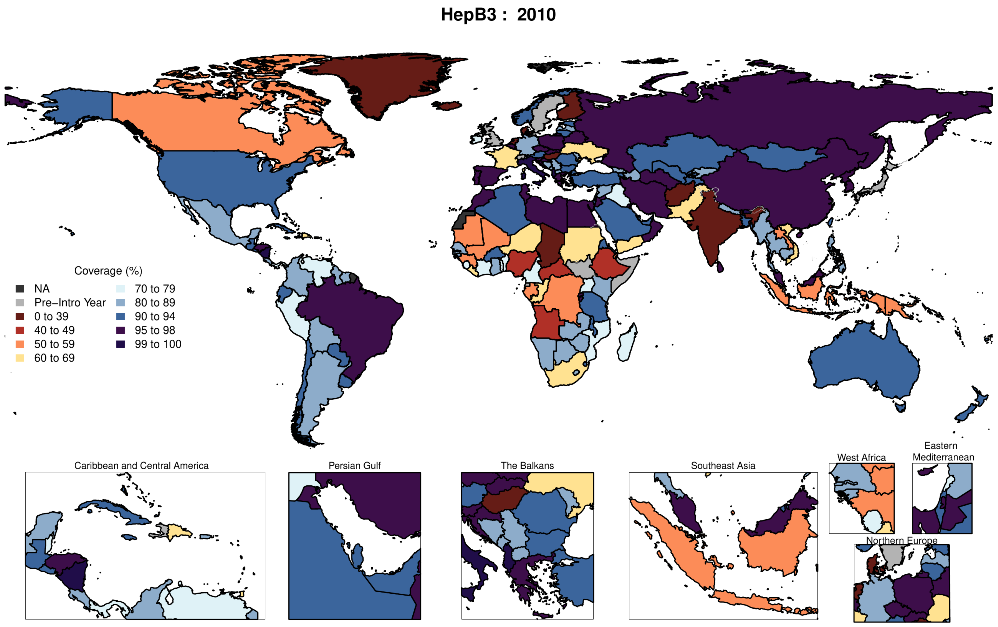
HepB3 : 2010
Coverage (%)
NA
Pre−Intro Year
0 to 39
40 to 49
50 to 59
60 to 69
70 to 79
80 to 89
90 to 94
95 to 98
99 to 100
Caribbean and Central America
Persian Gulf
The Balkans
Southeast Asia
West Africa
Eastern Mediterranean
Northern Europe

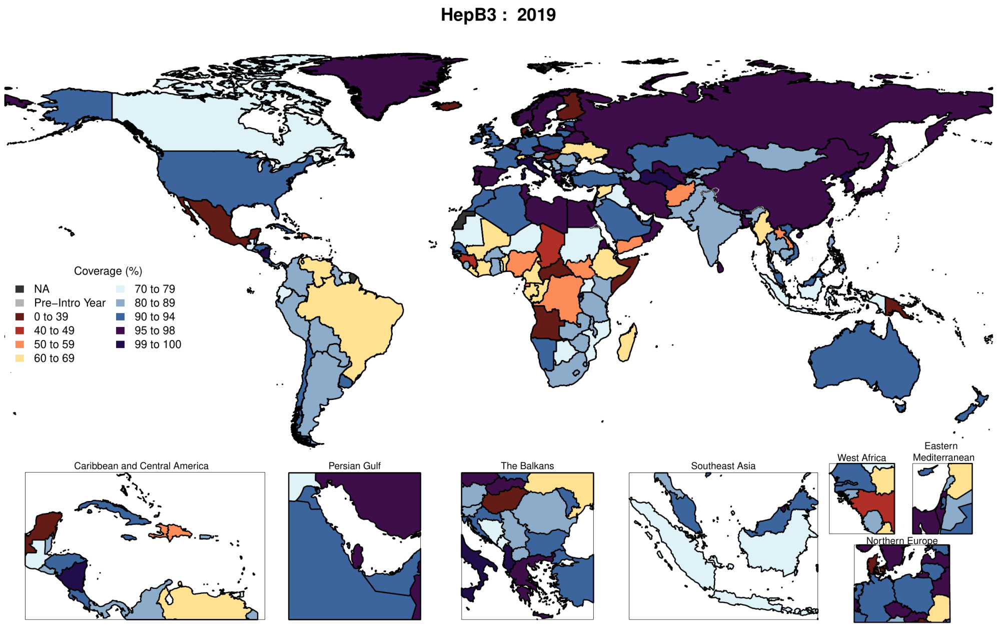
HepB3 : 2019
Coverage (%)
NA
Pre−Intro Year
0 to 39
40 to 49
50 to 59
60 to 69
70 to 79
80 to 89
90 to 94
95 to 98
99 to 100
Caribbean and Central America
Persian Gulf
The Balkans
Southeast Asia
West Africa
Eastern Mediterranean
Northern Europe

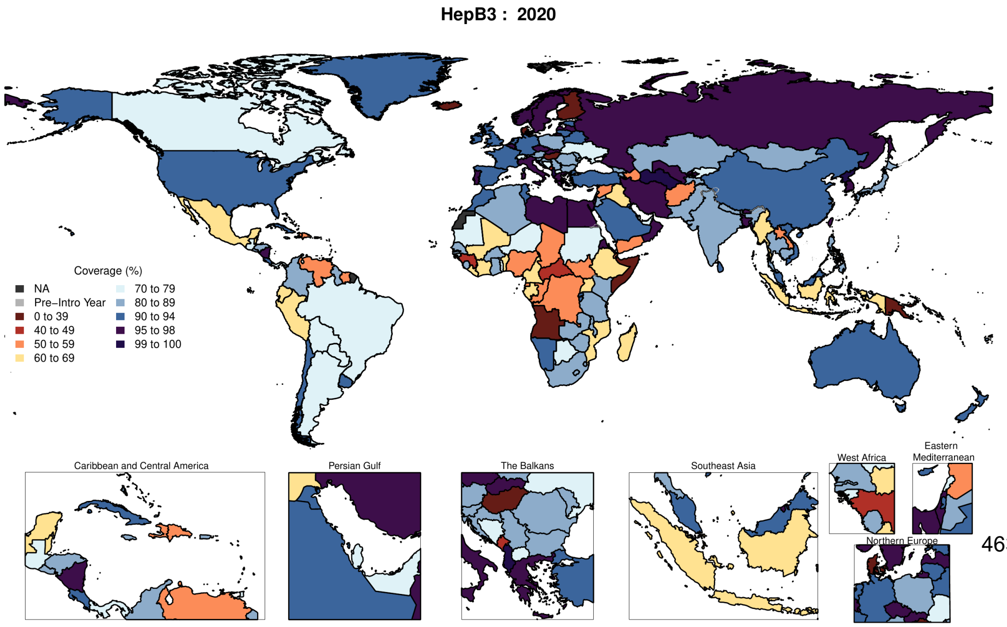
HepB3 : 2020
Coverage (%)
NA
Pre−Intro Year
0 to 39
40 to 49
50 to 59
60 to 69
70 to 79
80 to 89
90 to 94
95 to 98
99 to 100
Caribbean and Central America
Persian Gulf
The Balkans
Southeast Asia
West Africa
Eastern Mediterranean
Northern Europe

HepB3 : 2021

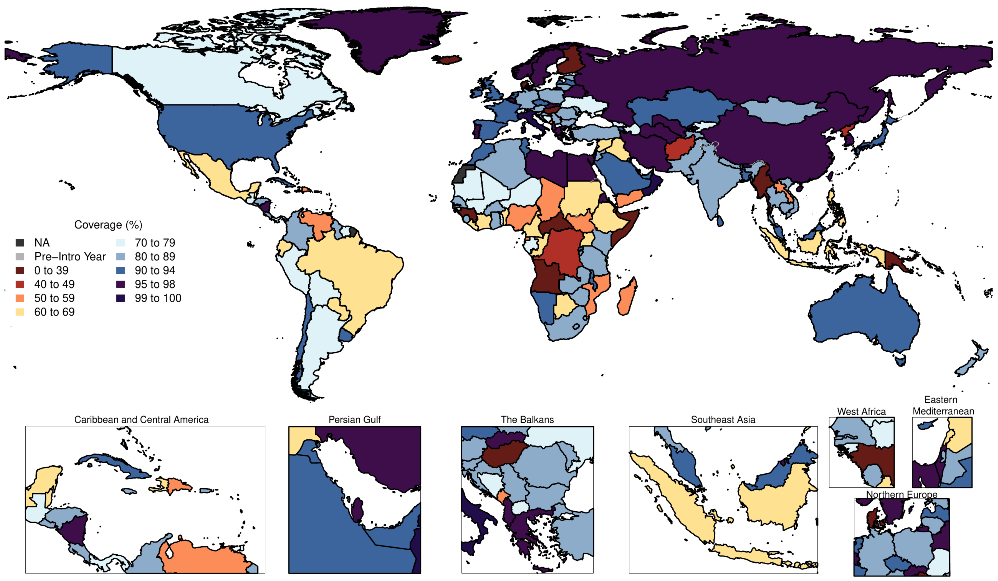

HepB3 : 2022

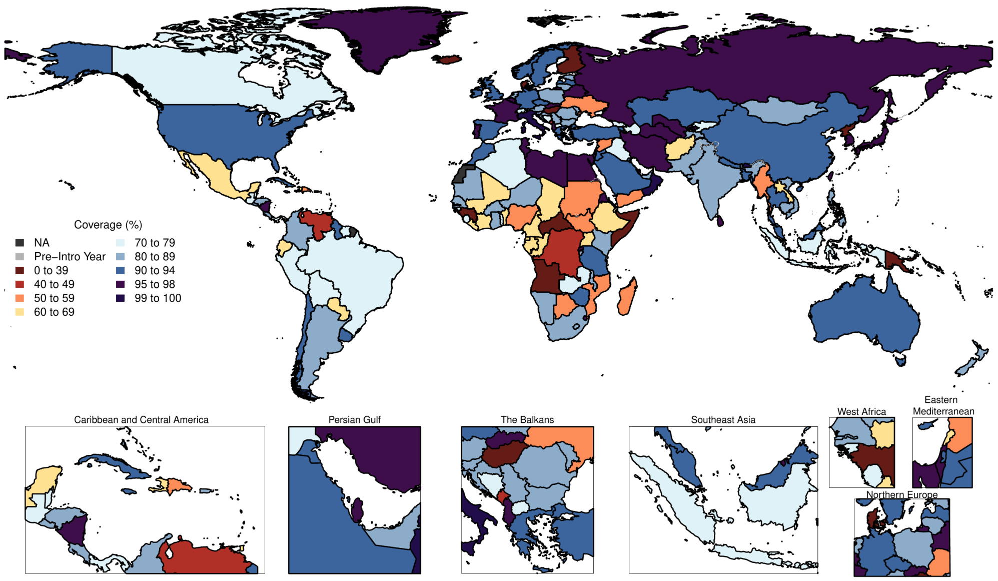

HepB3 : 2023

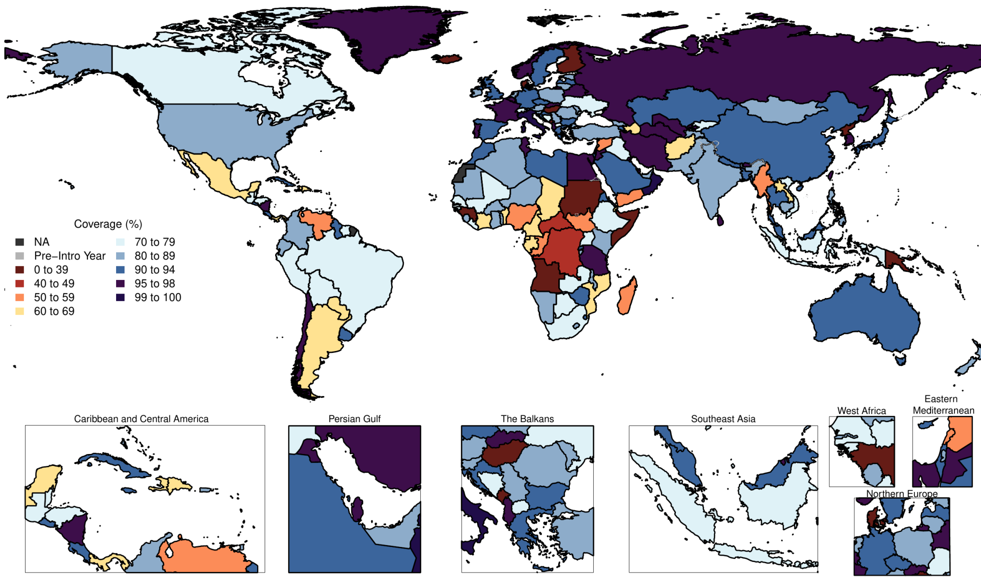

Hib3 : 1980

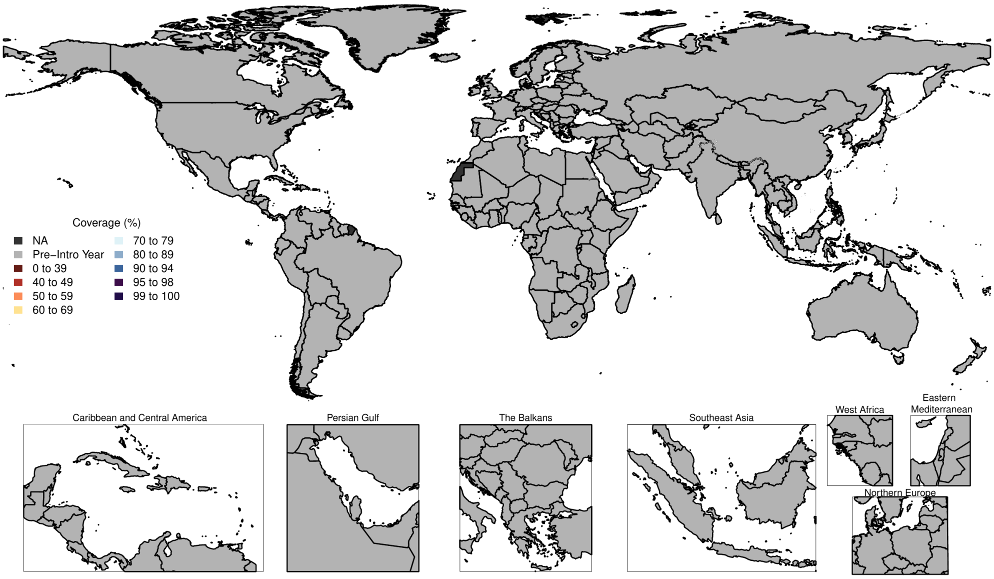

Hib3 : 1990

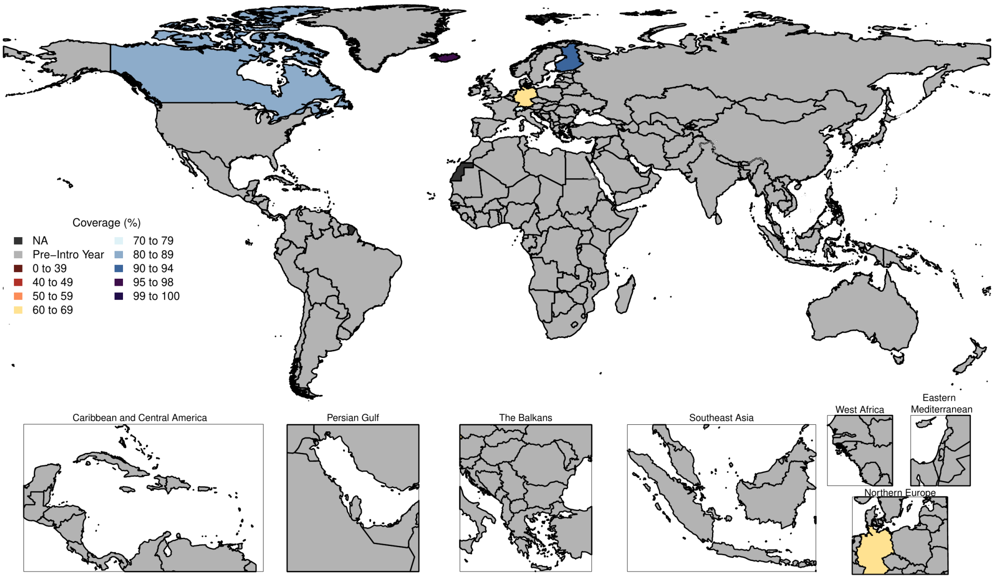

Hib3 : 2000

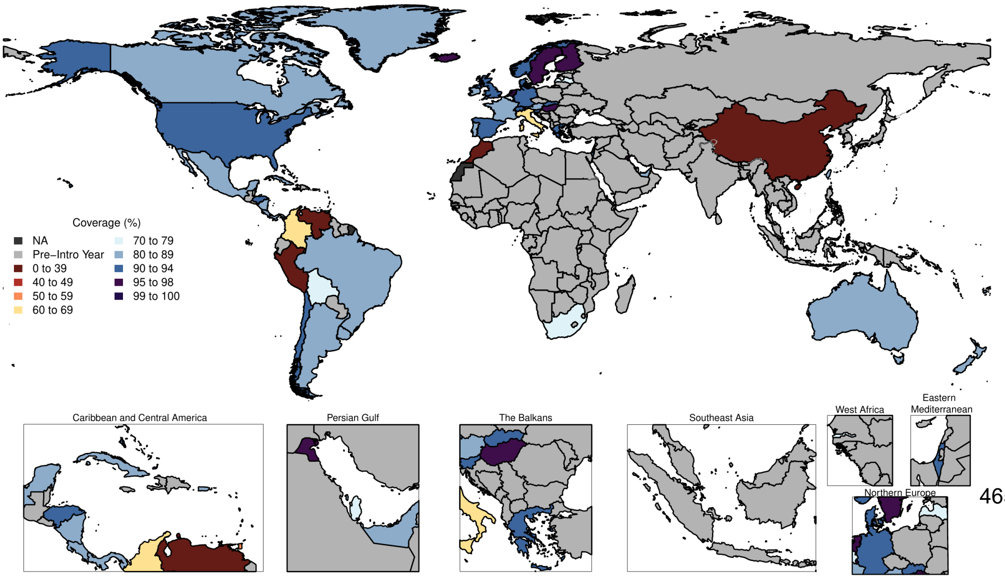

Hib3 : 2010

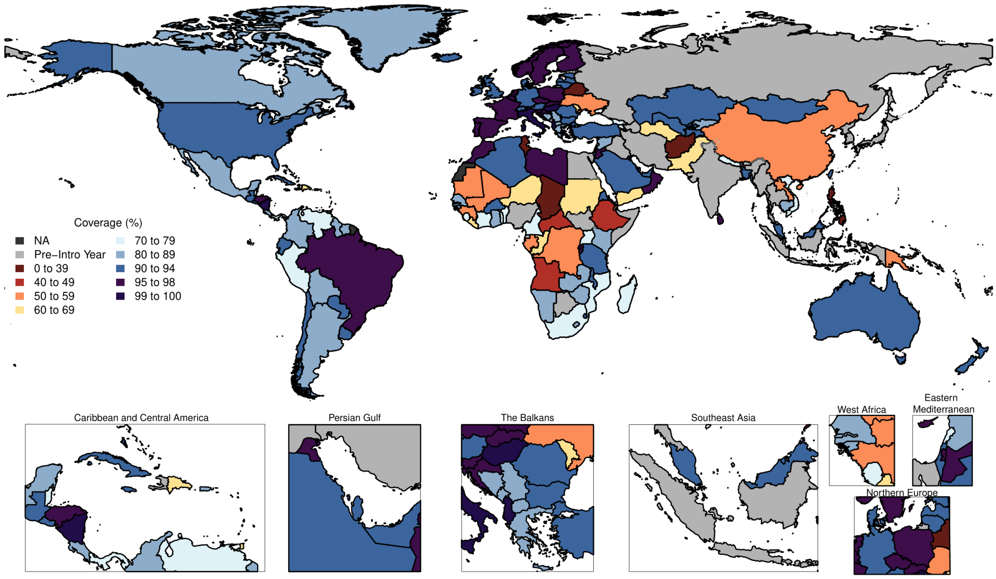

Hib3 : 2019

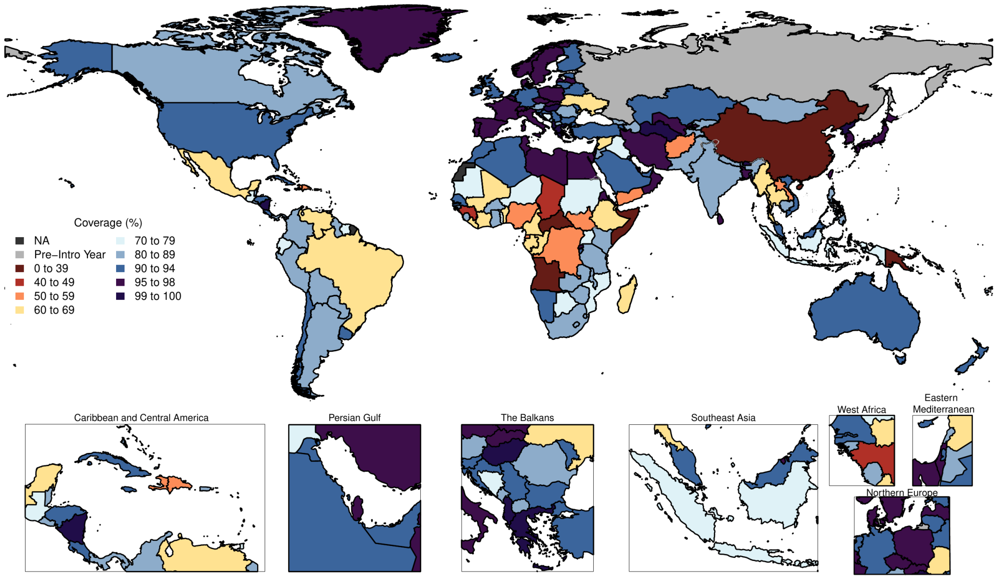

Hib3 : 2020

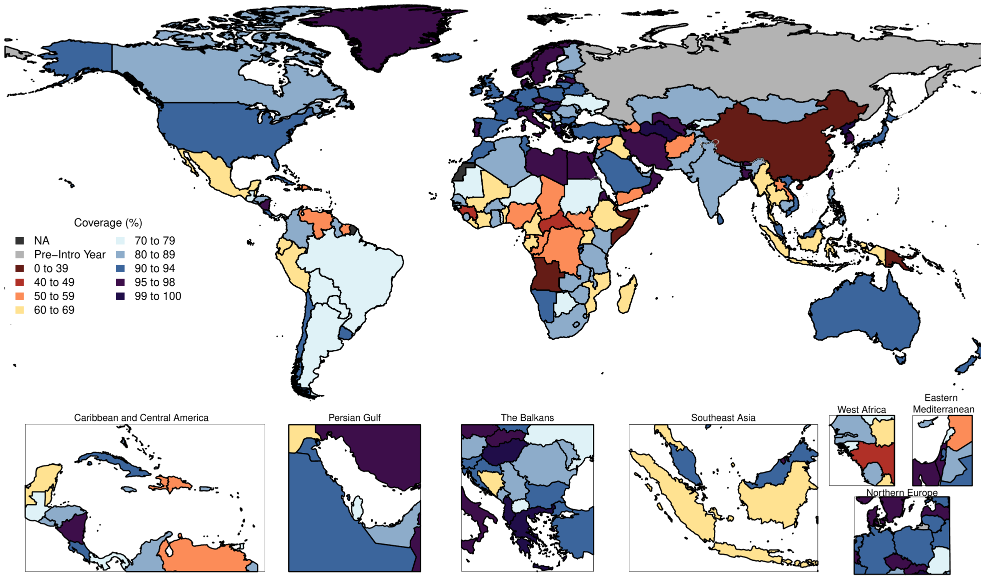

Hib3 : 2021

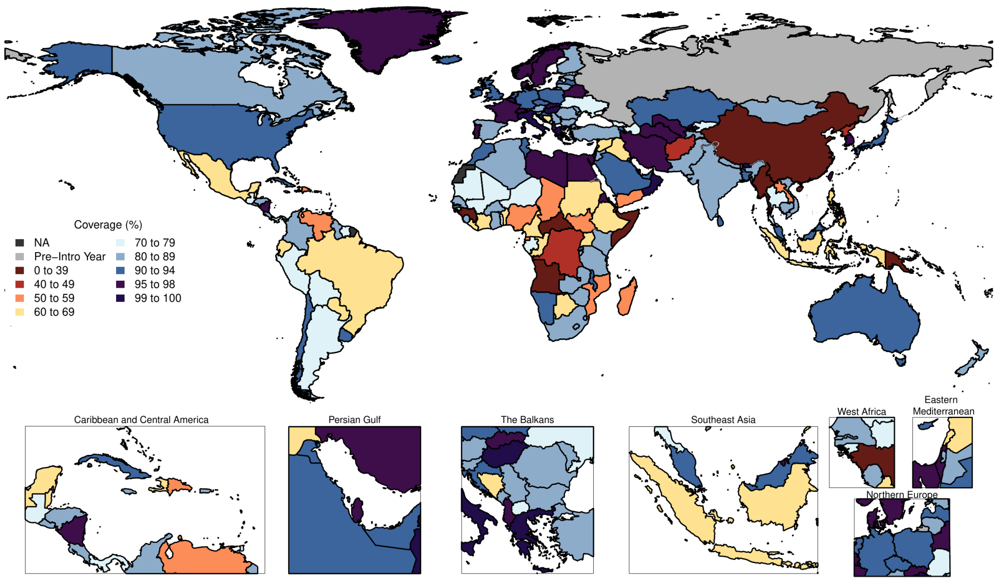

Hib3 : 2022

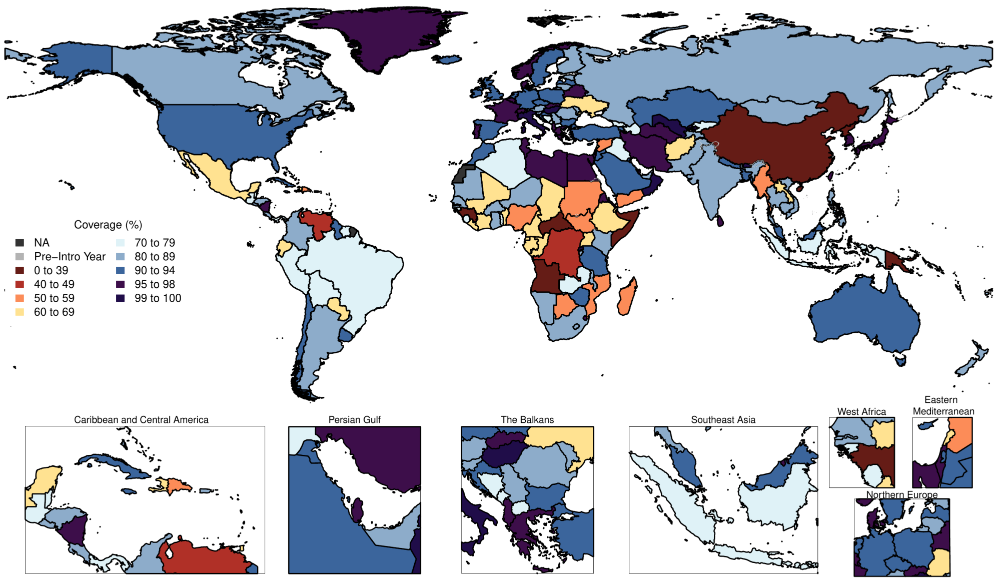

Hib3 : 2023

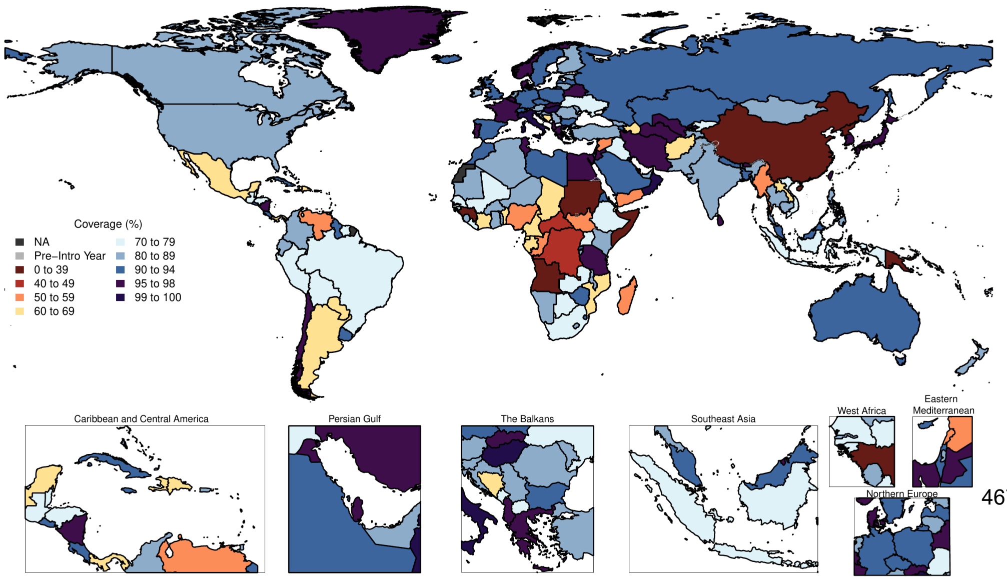

MCV1 : 1980

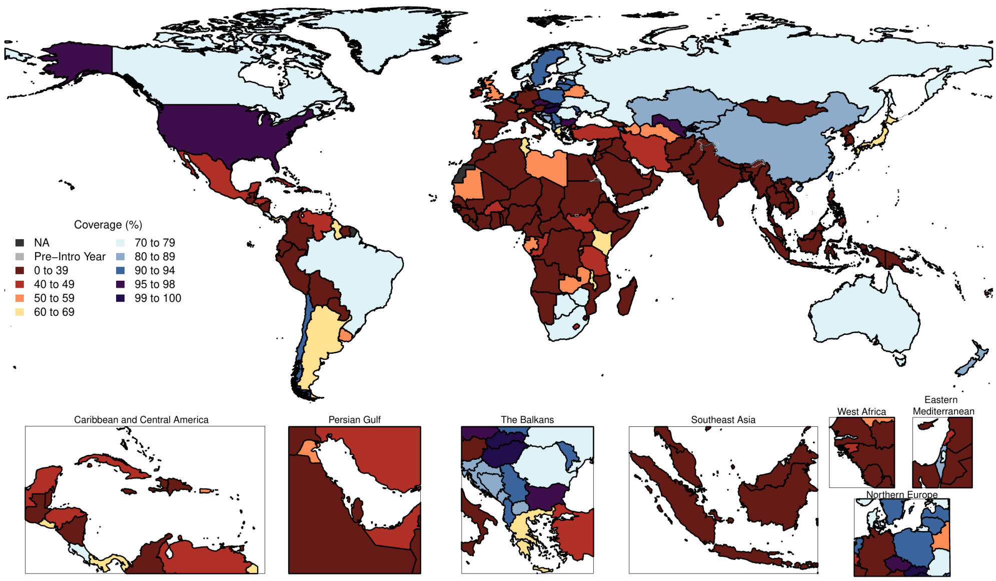

MCV1 : 1990

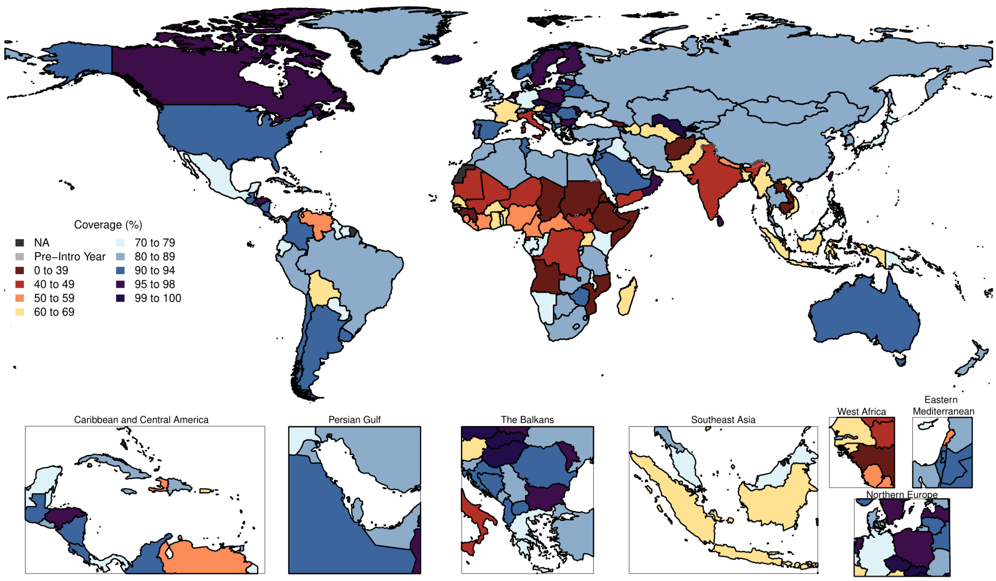

MCV1 : 2000

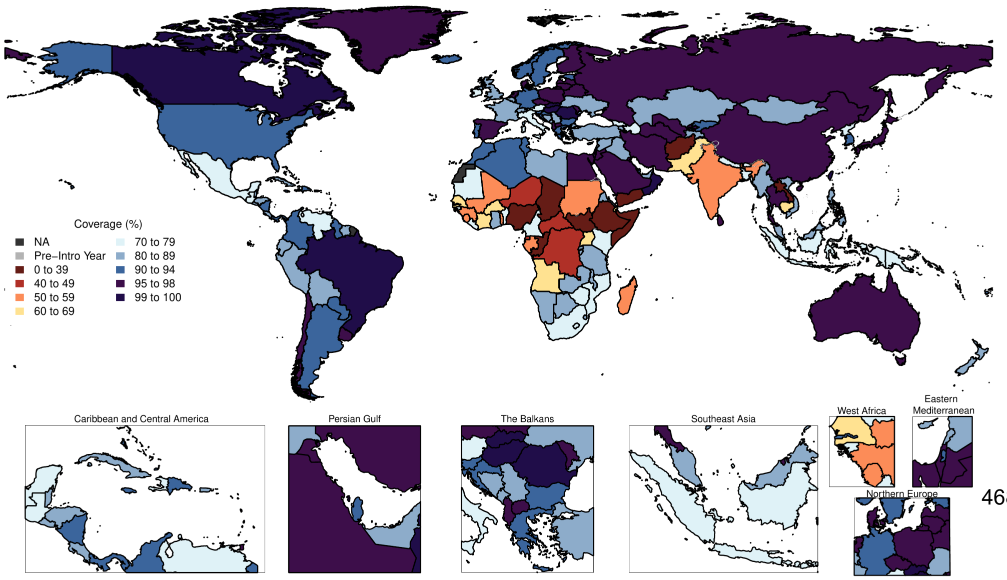

MCV1 : 2010

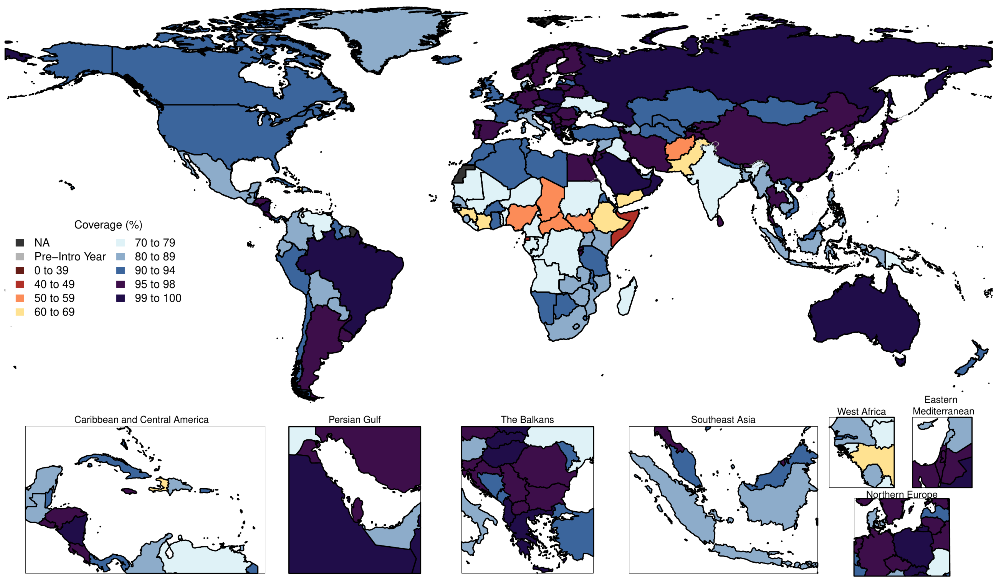

MCV1 : 2019

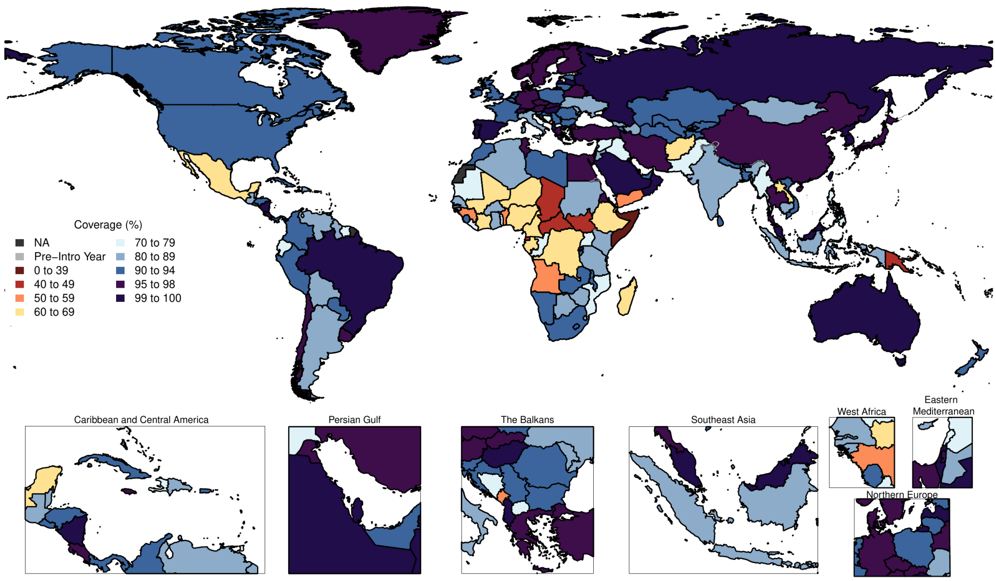

MCV1 : 2020

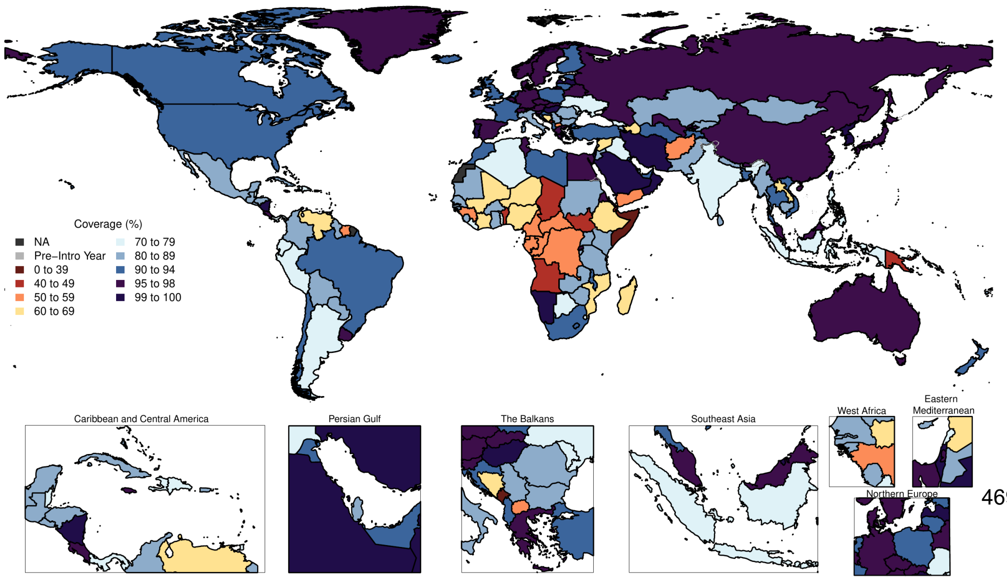

MCV1 : 2021

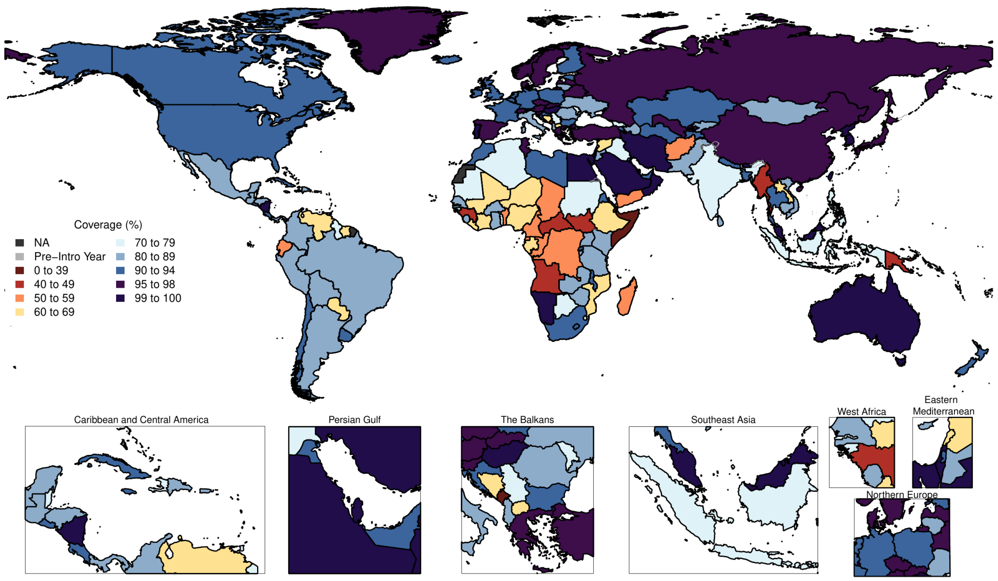

MCV1 : 2022

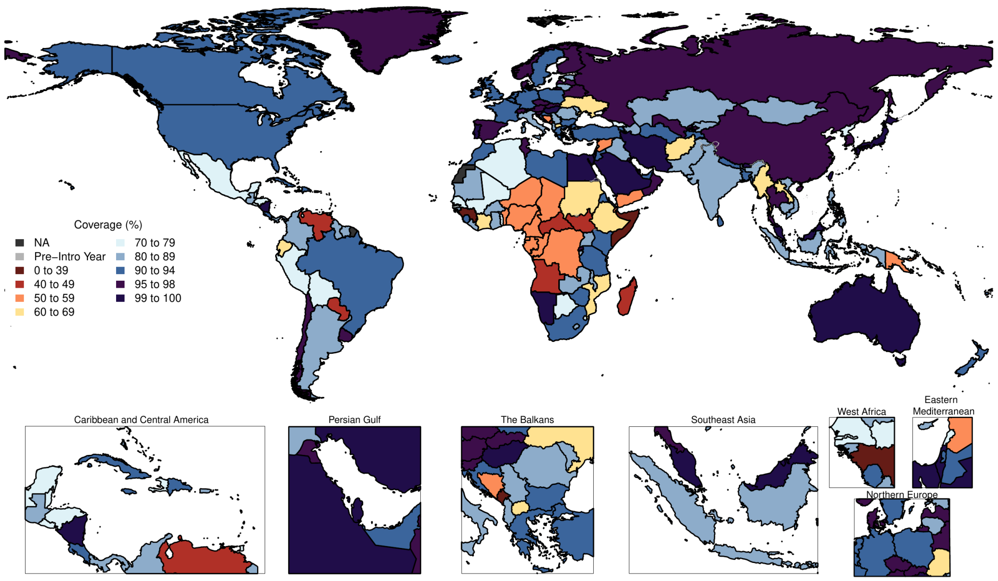

MCV1 : 2023

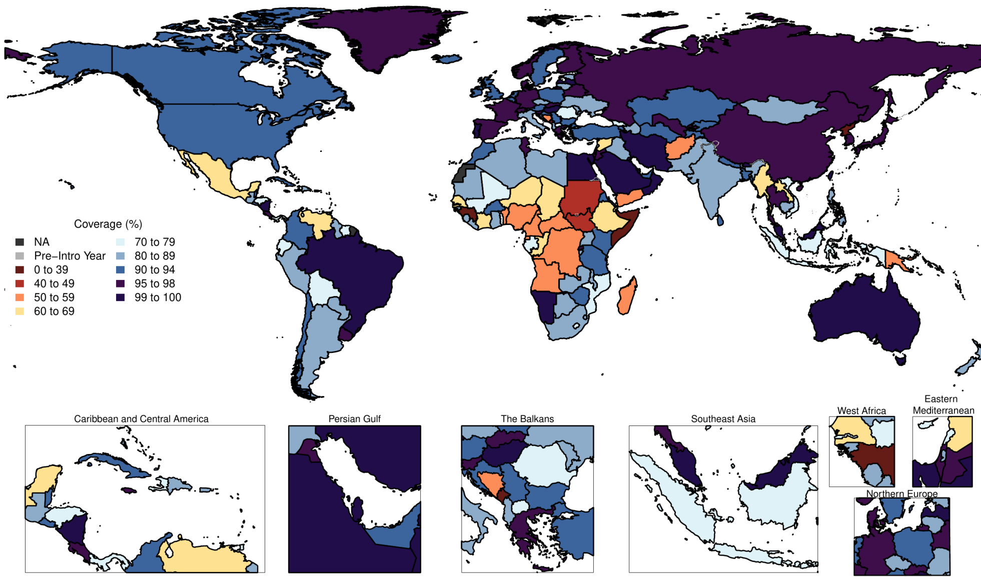

MCV2 : 1980

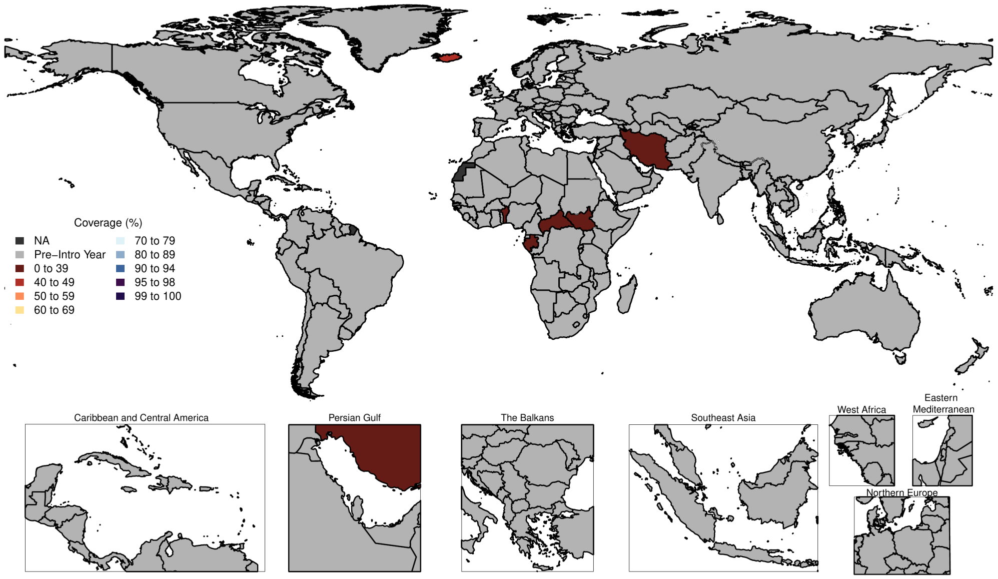

MCV2 : 1990

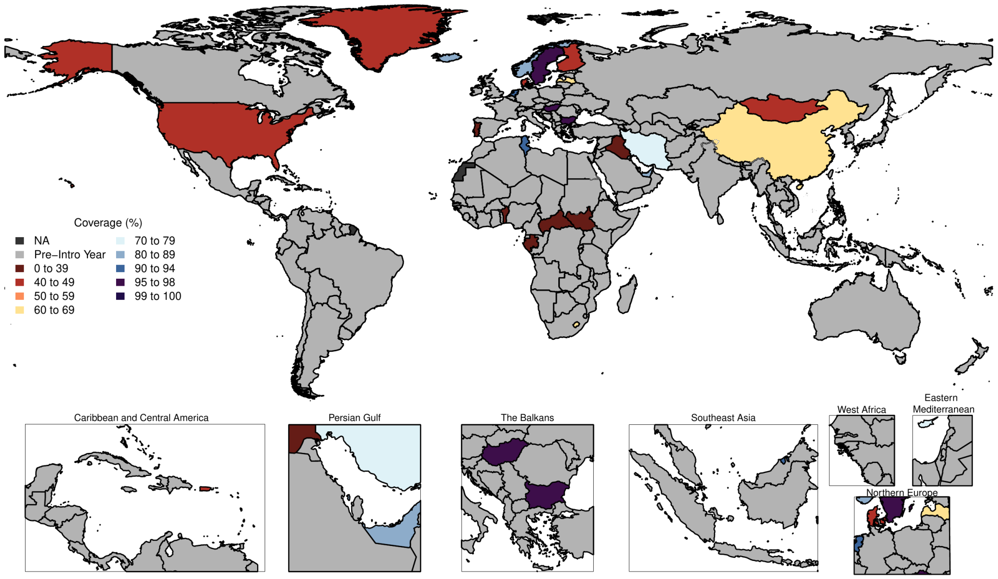

MCV2 : 2000

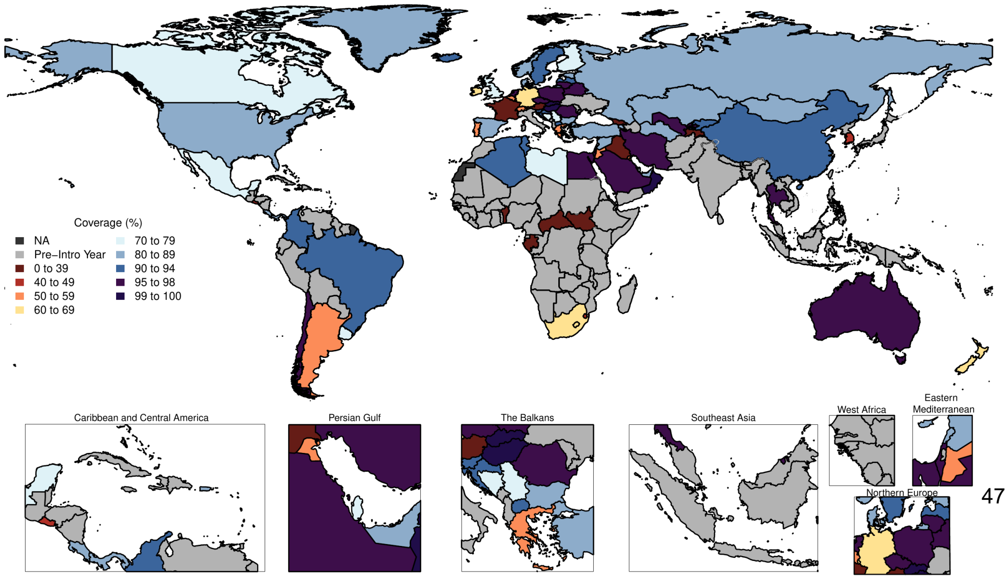

MCV2 : 2010

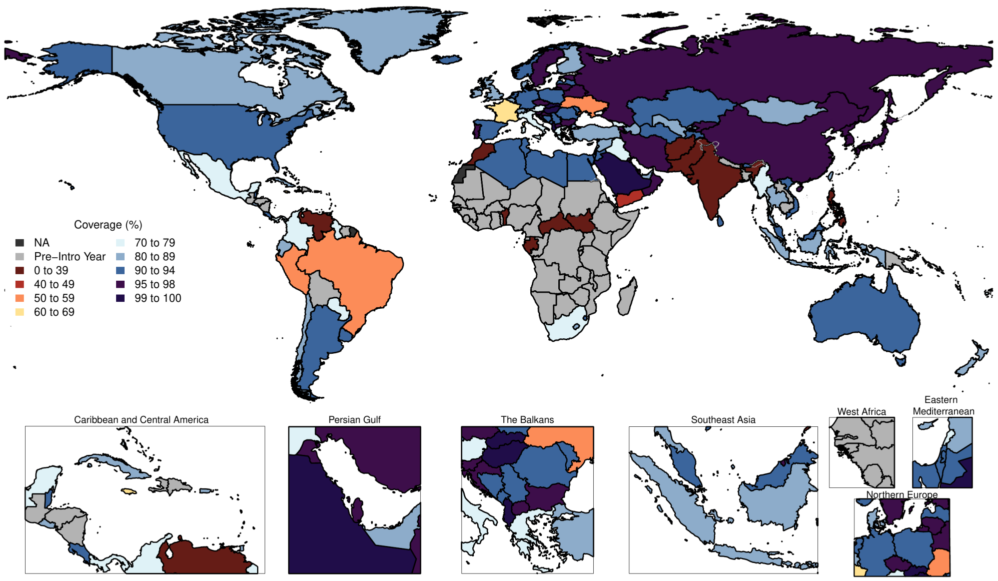

MCV2 : 2019

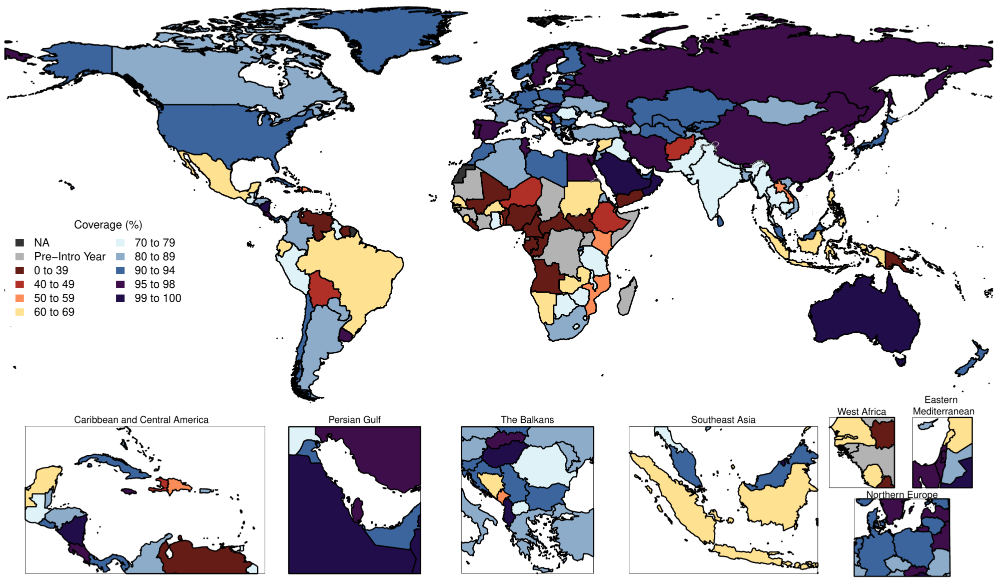

MCV2 : 2020

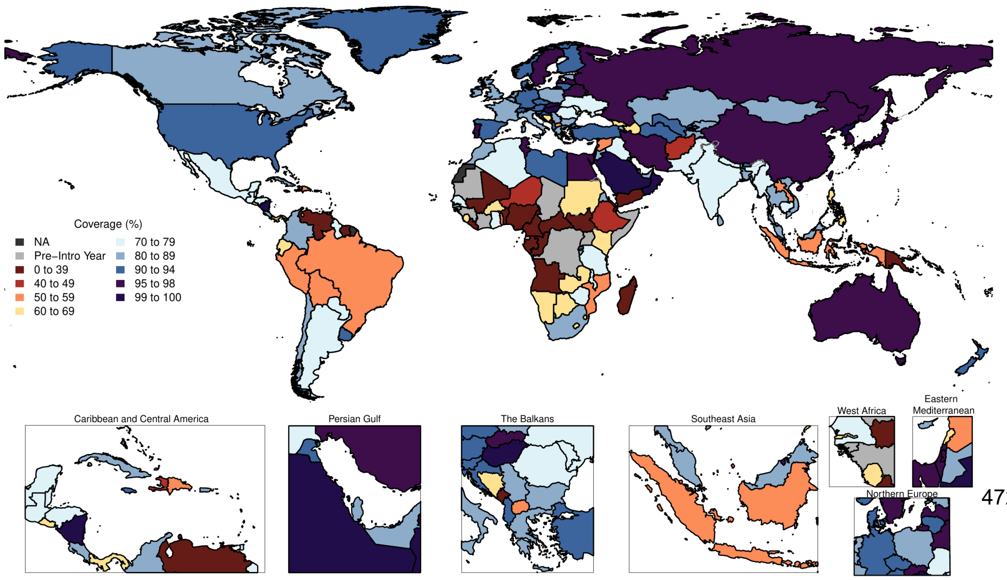

MCV2 : 2021

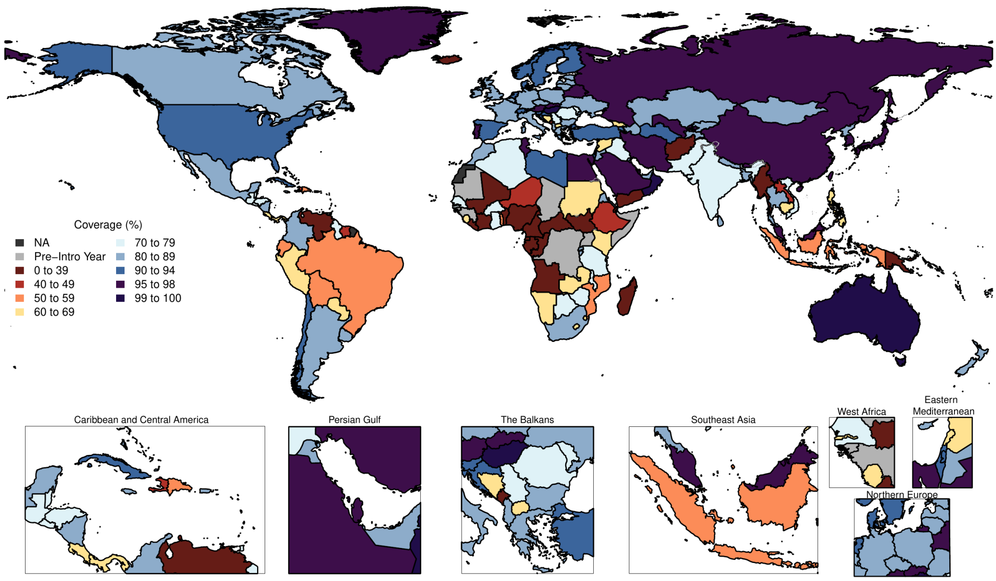

MCV2 : 2022

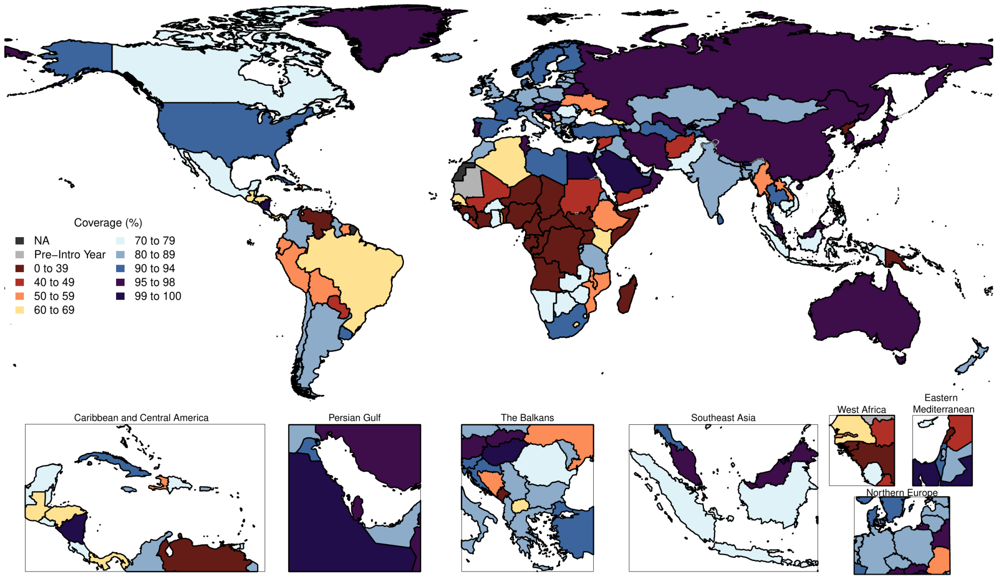

MCV2 : 2023

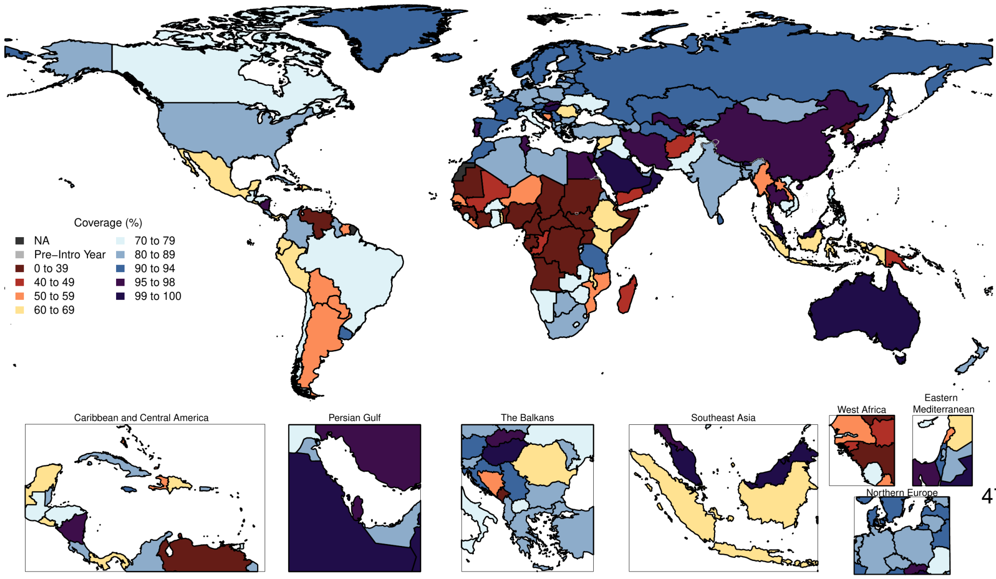

PCV3 : 1980

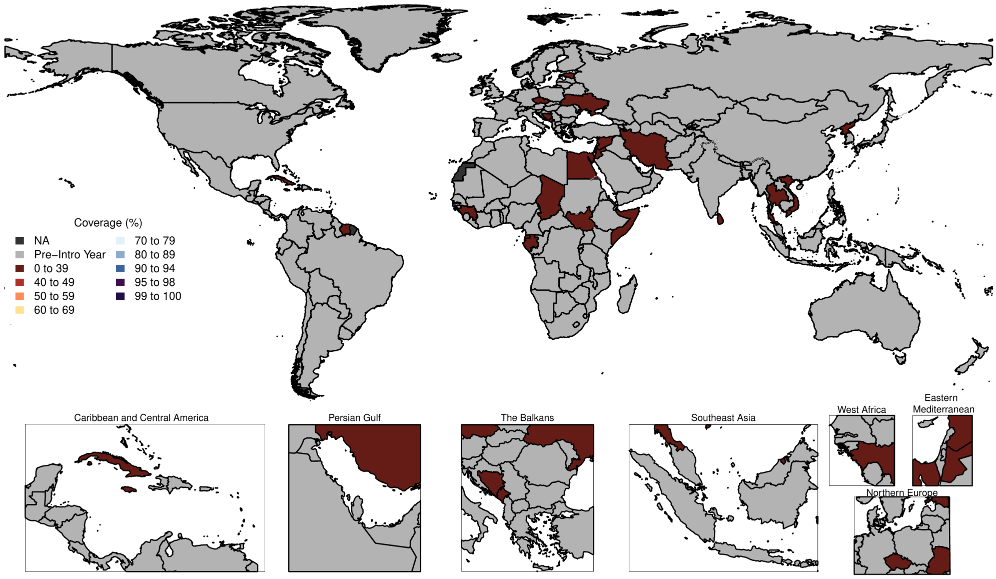

PCV3 : 1990

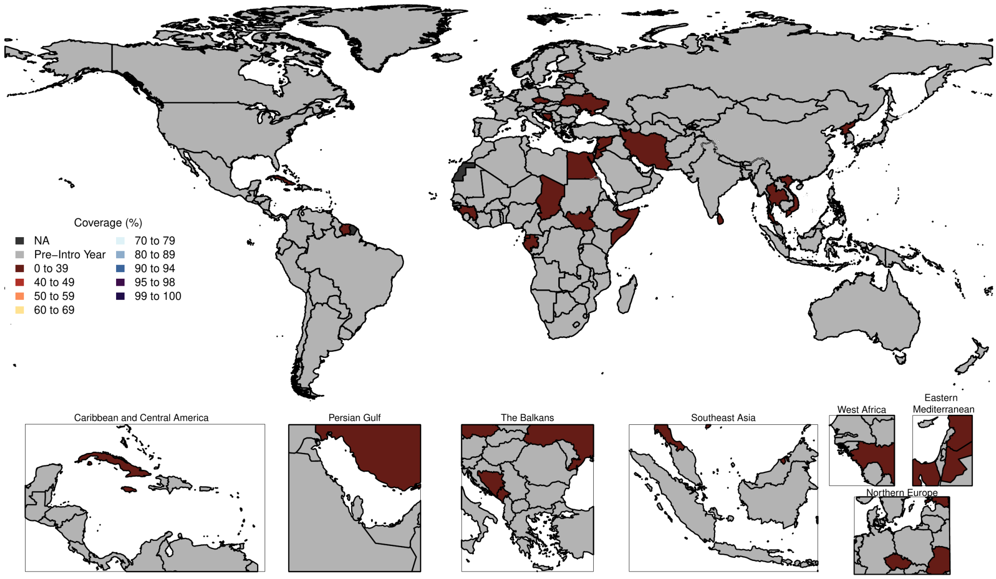

PCV3 : 2000

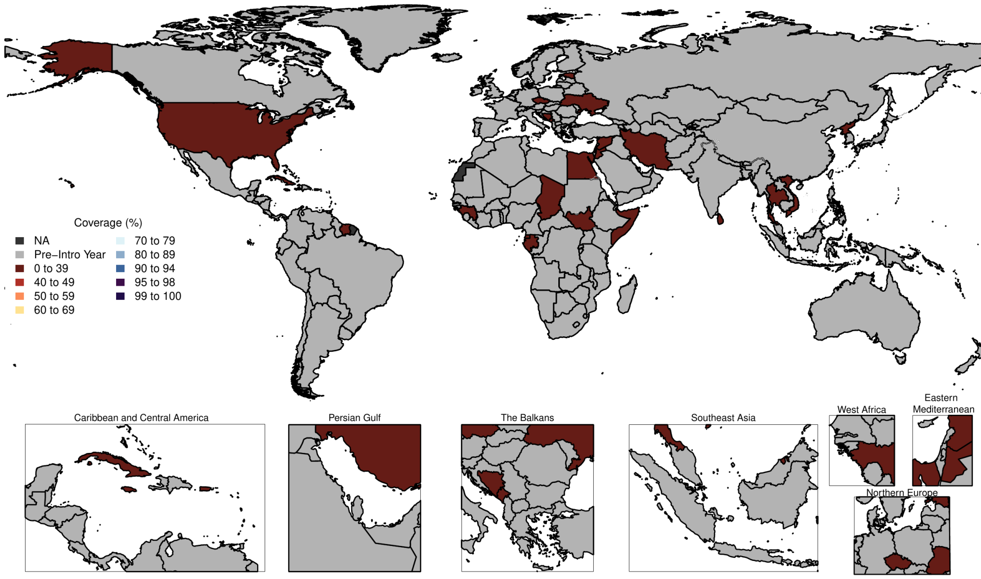

PCV3 : 2010

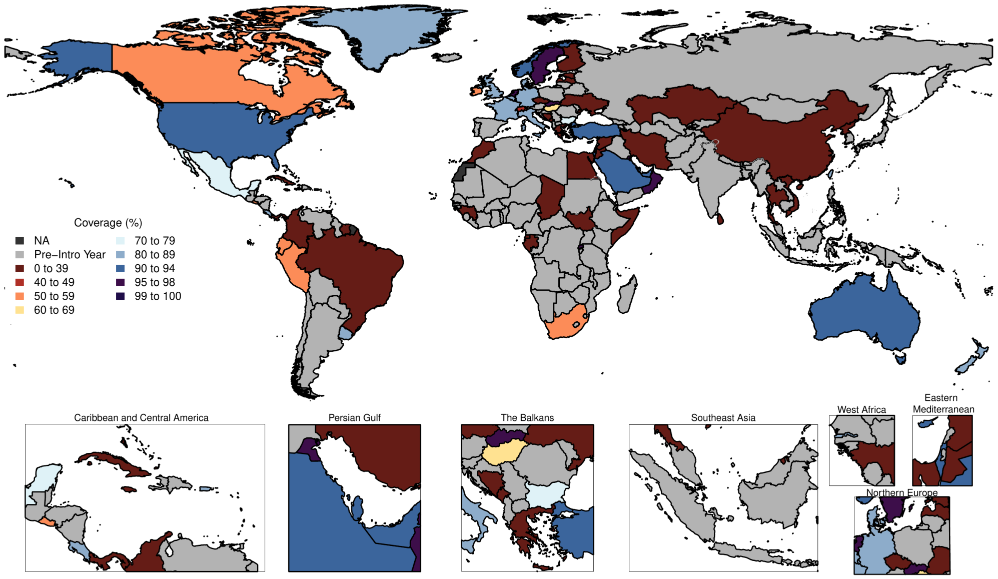

PCV3 : 2019

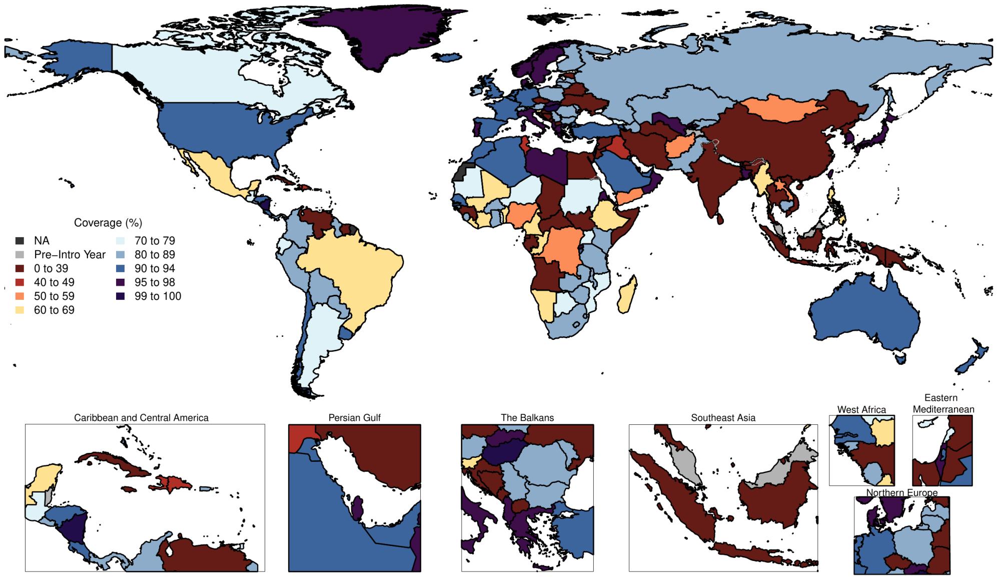

PCV3 : 2020

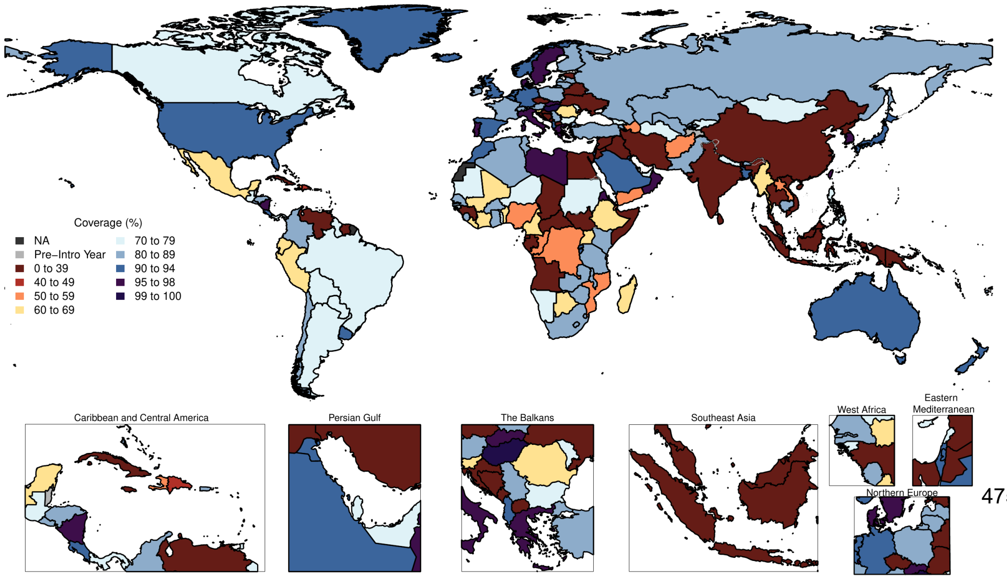

PCV3 : 2021

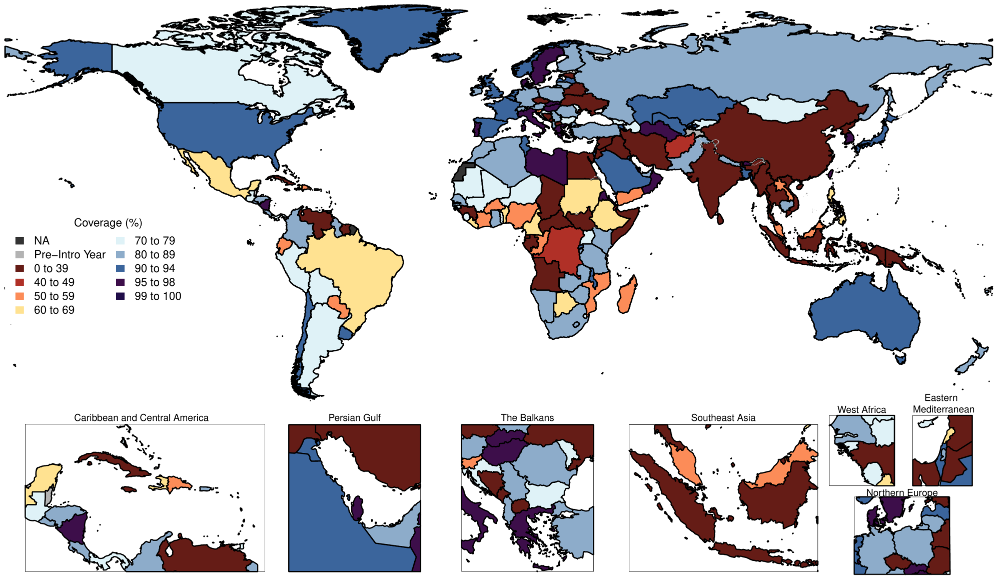

PCV3 : 2022

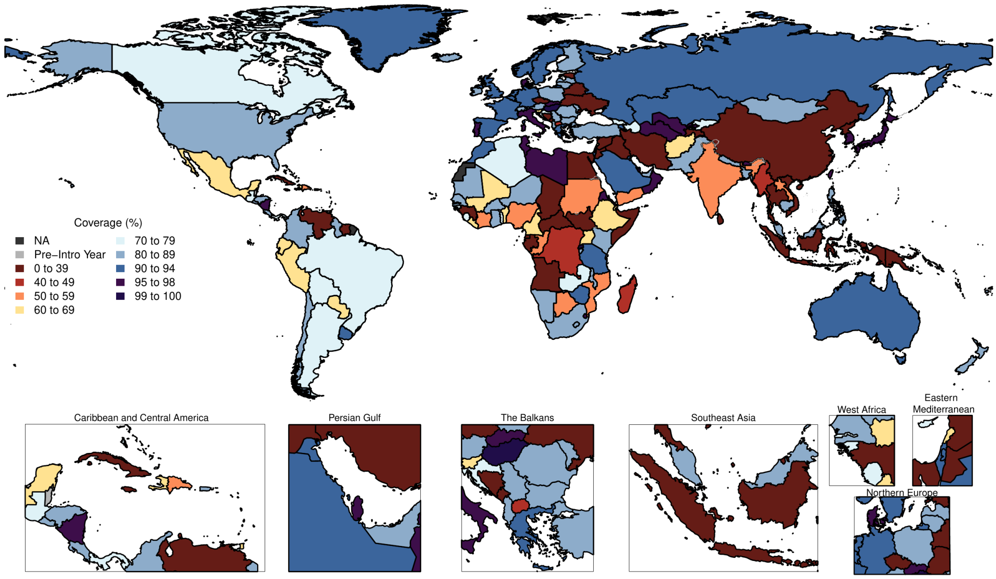

PCV3 : 2023

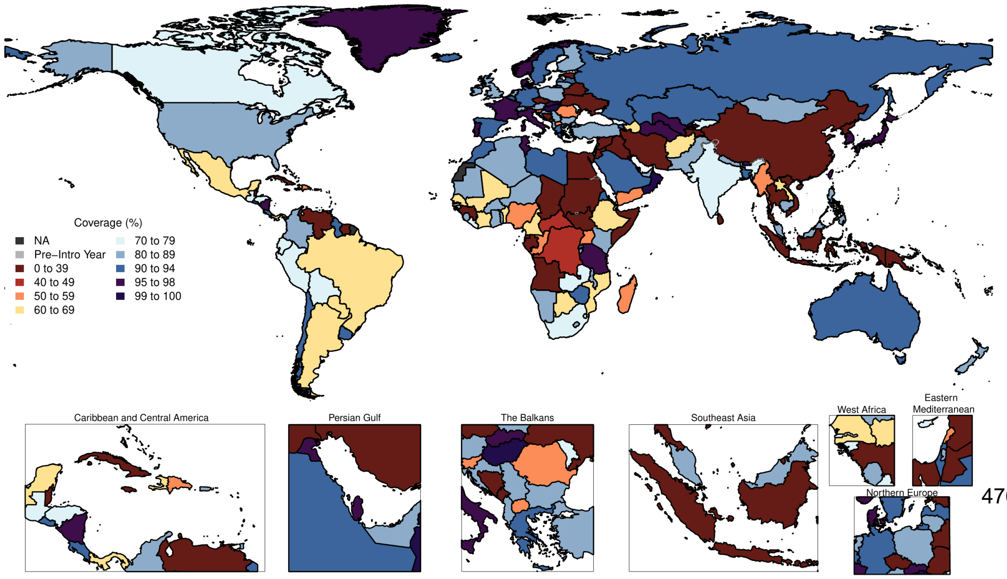

Pol3 : 1980

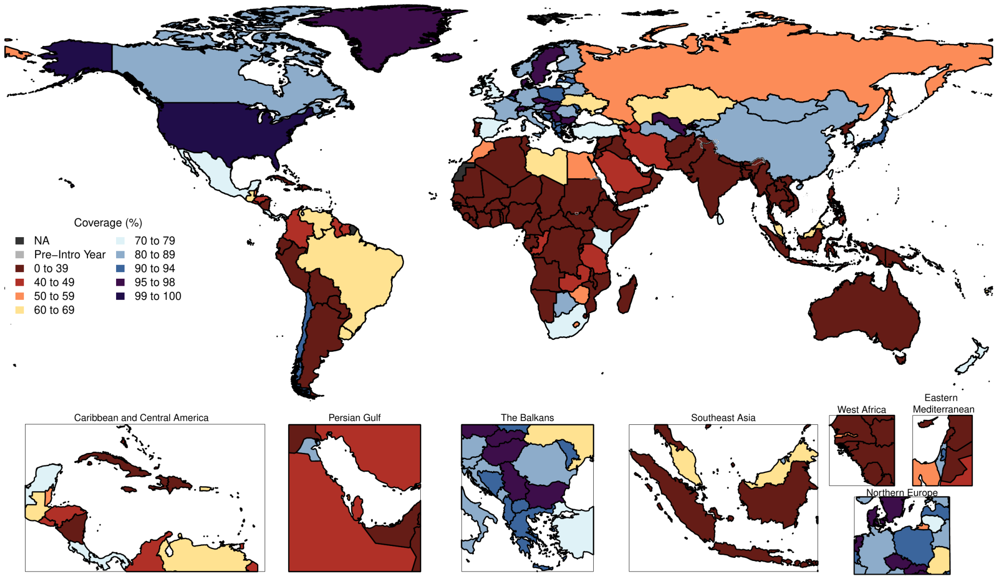

Pol3 : 1990

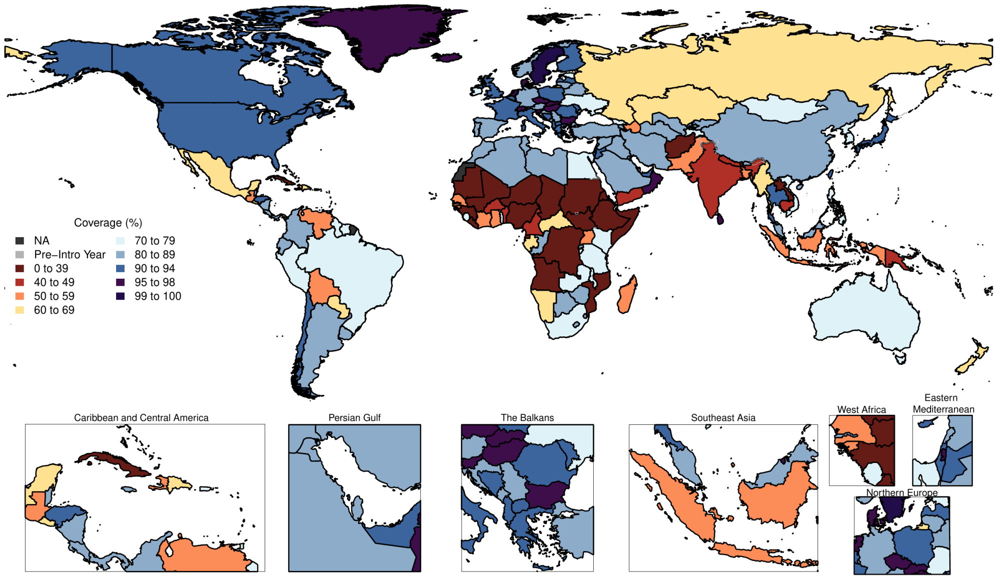

Pol3 : 2000

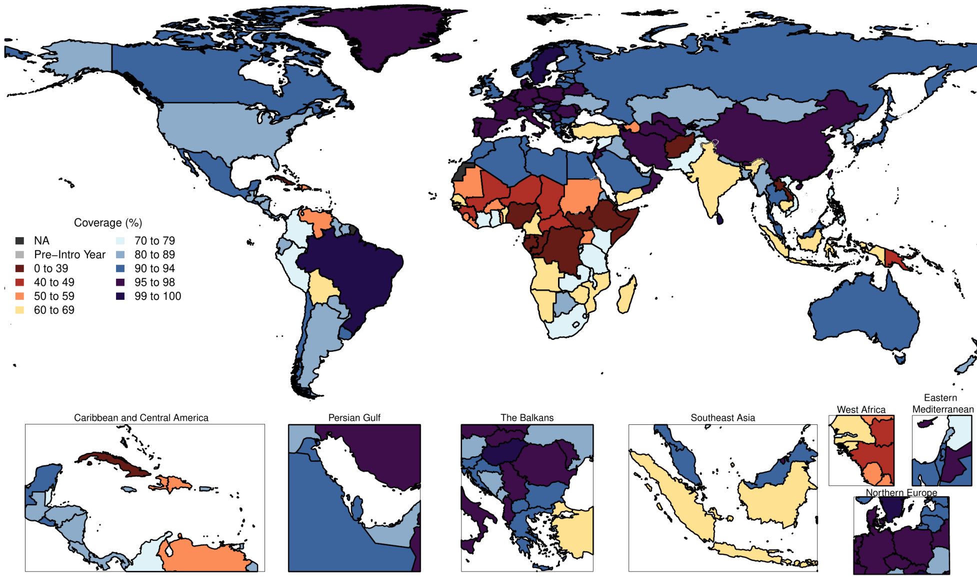

Pol3 : 2010

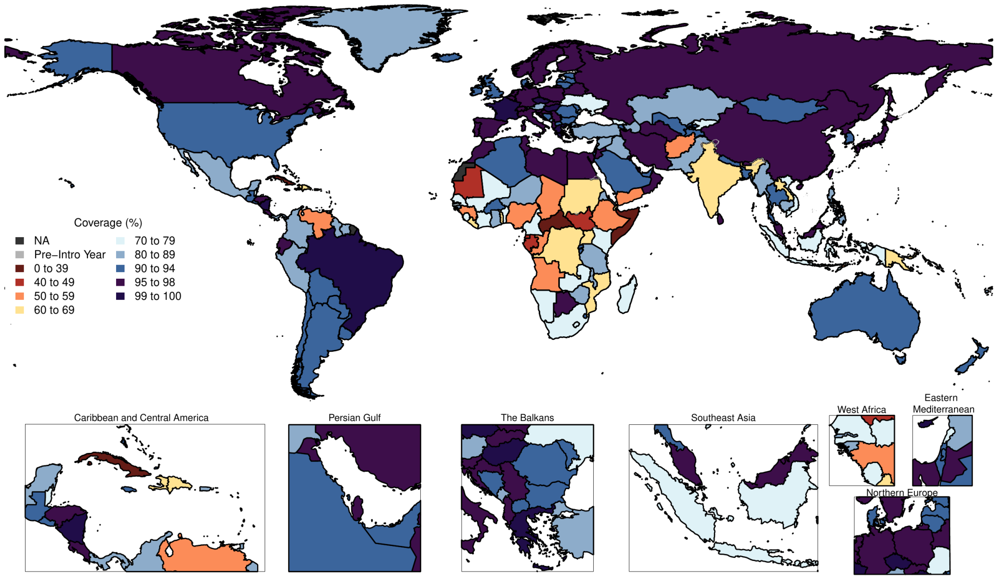

Pol3 : 2019

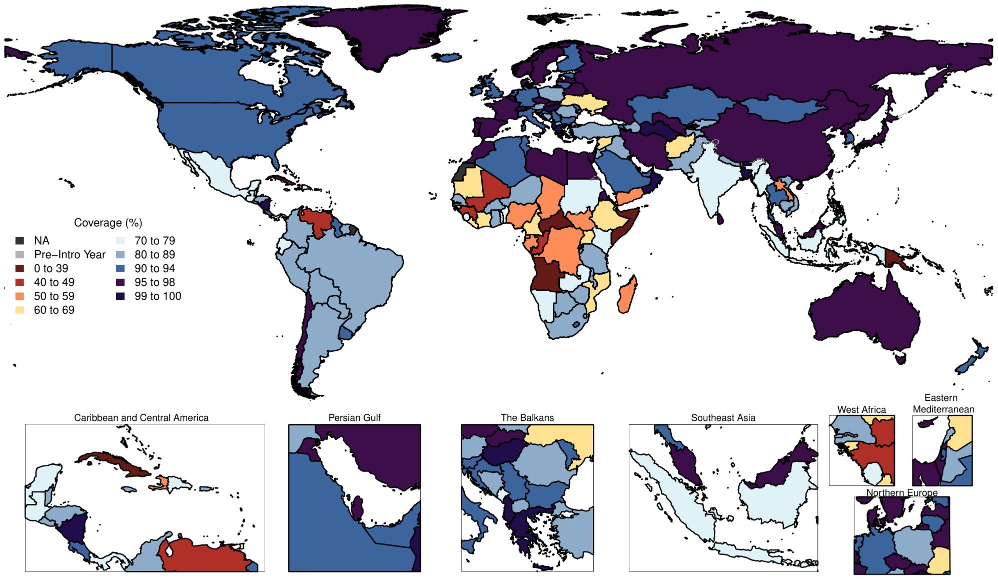

Pol3 : 2020

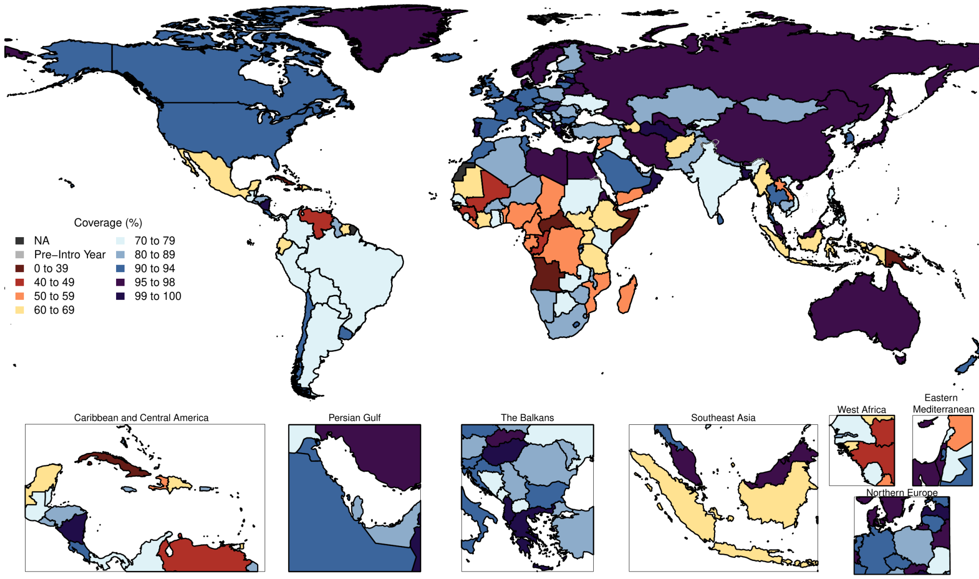

Pol3 : 2021

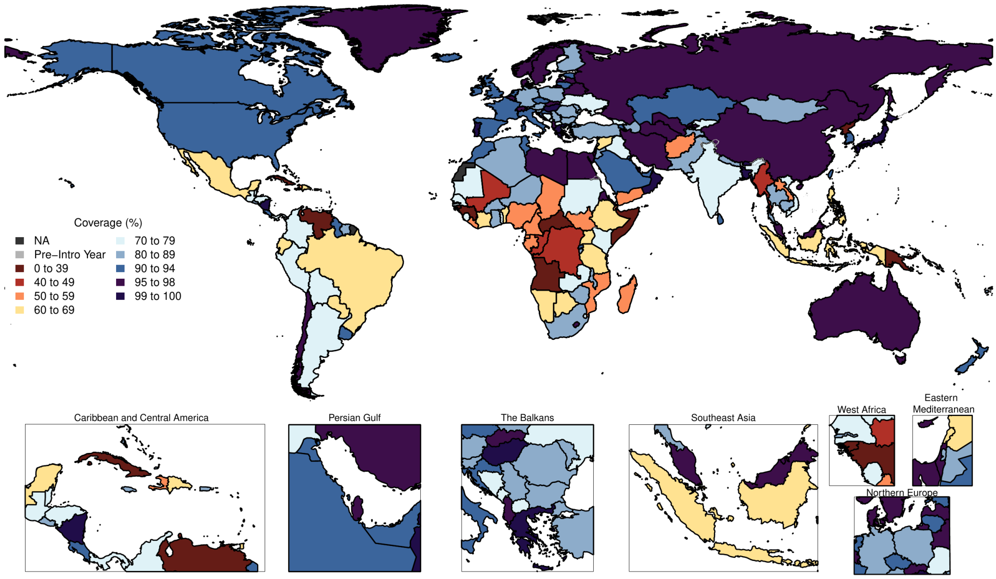

Pol3 : 2022

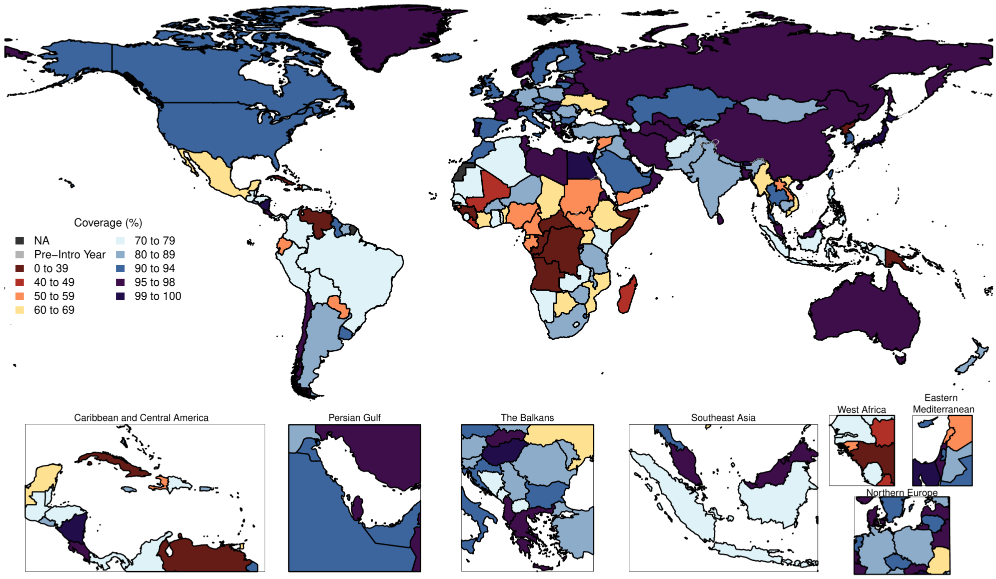

Pol3 : 2023

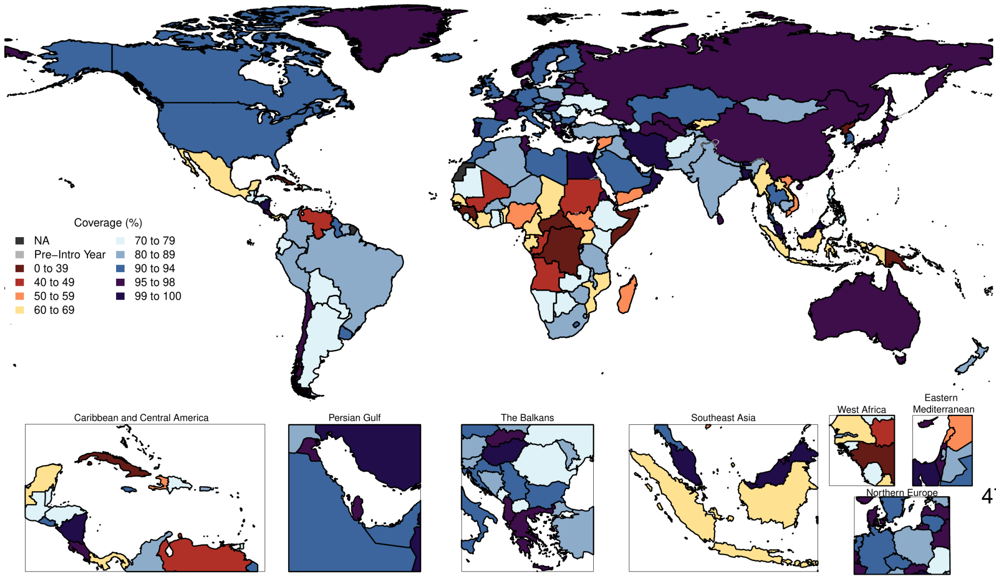

RCV1 : 1980

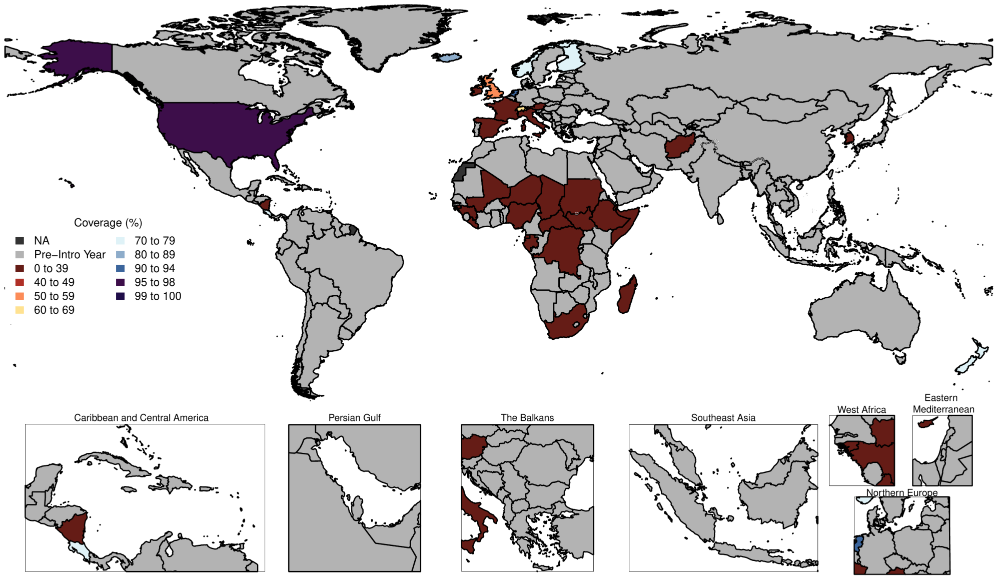

RCV1 : 1990

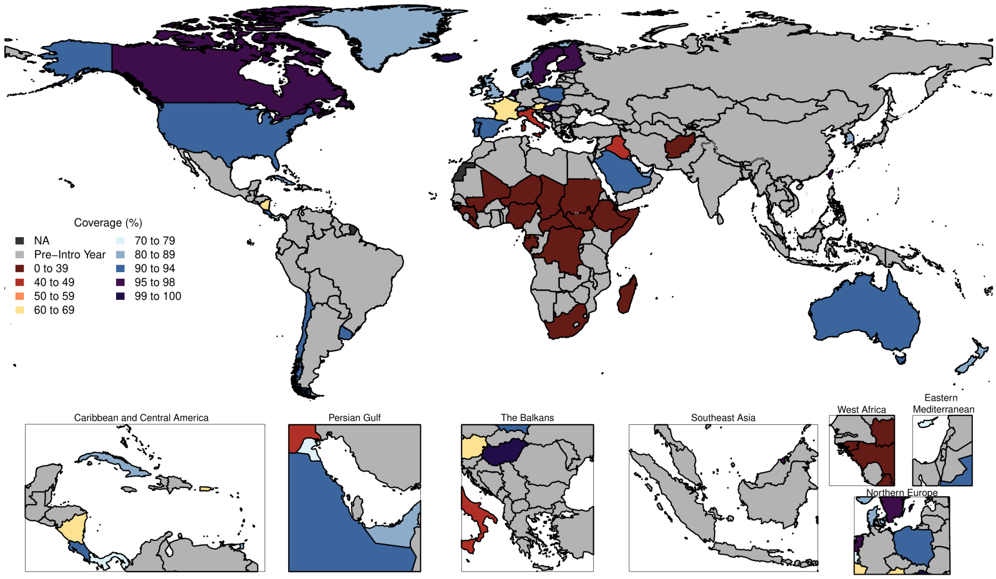

RCV1 : 2000

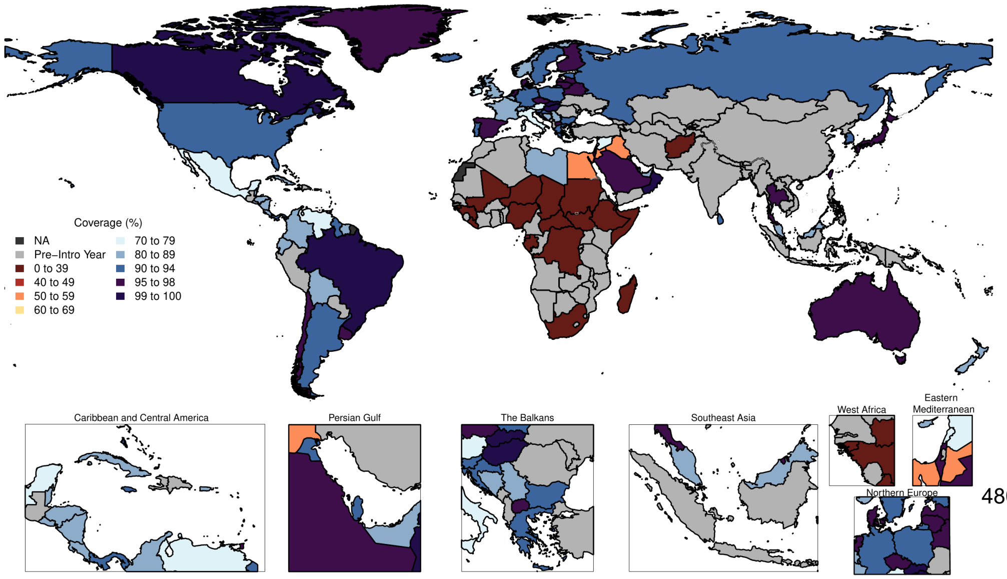

RCV1 : 2010

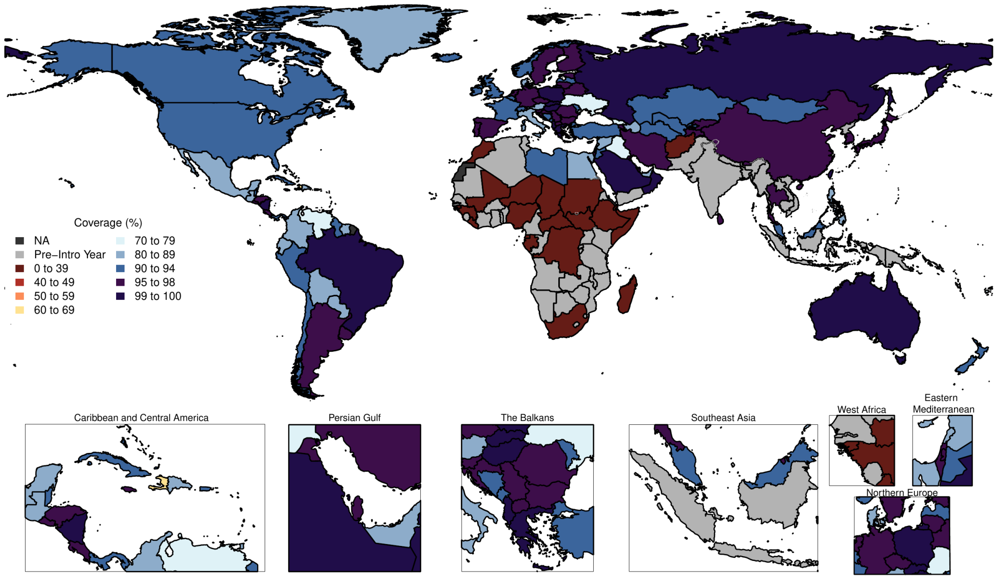

RCV1 : 2019

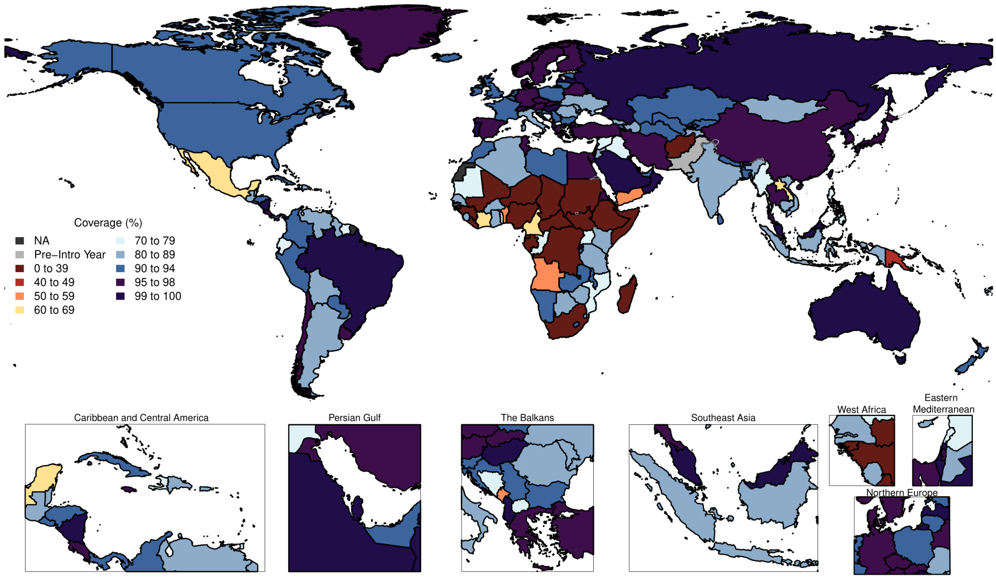

RCV1 : 2020

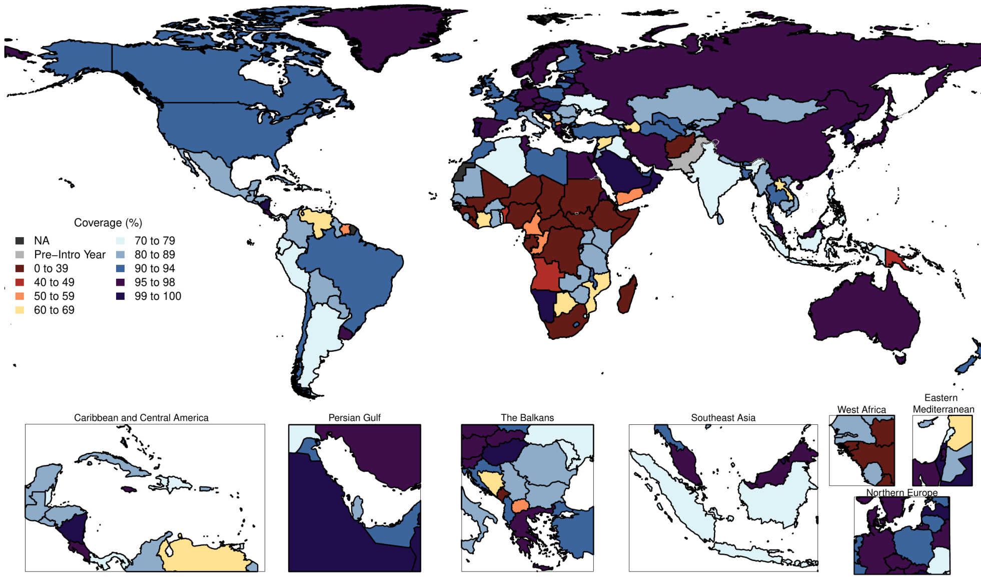

RCV1 : 2021

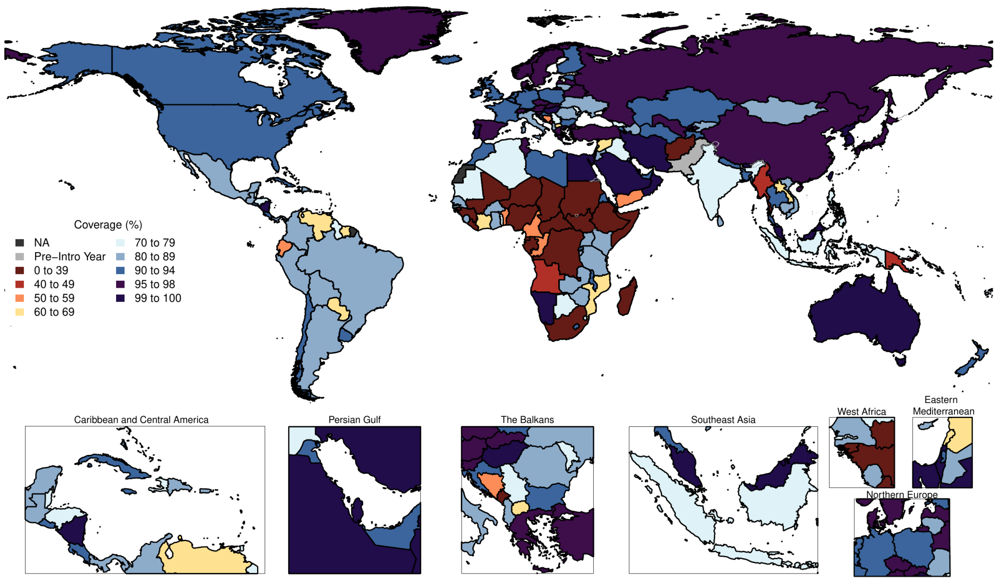

RCV1 : 2022

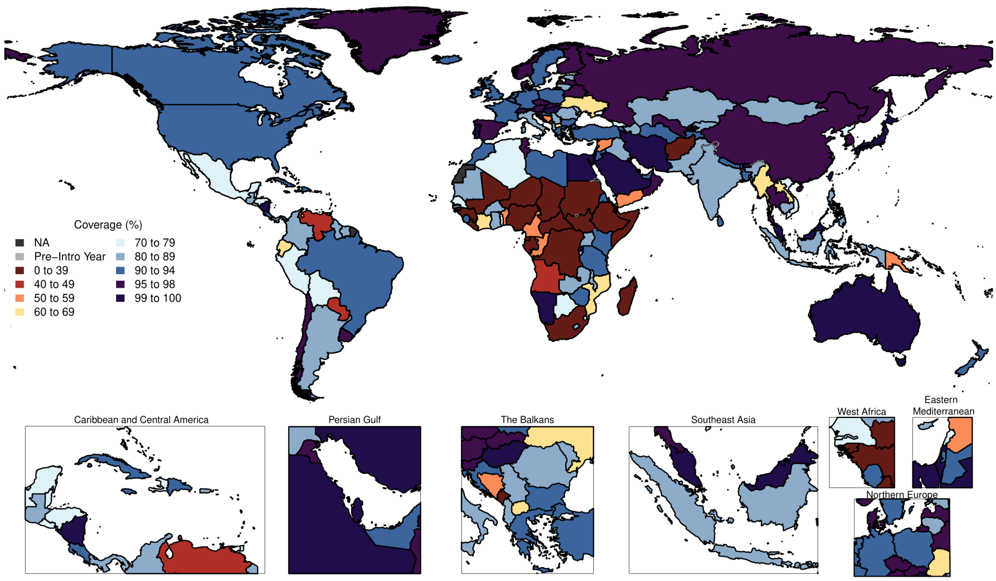

RCV1 : 2023

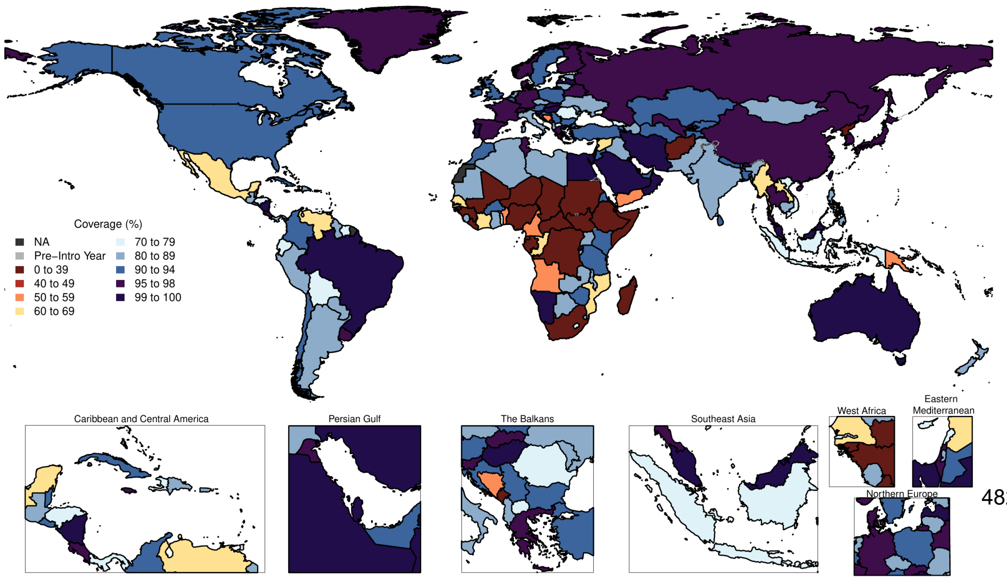

RotaC : 1980

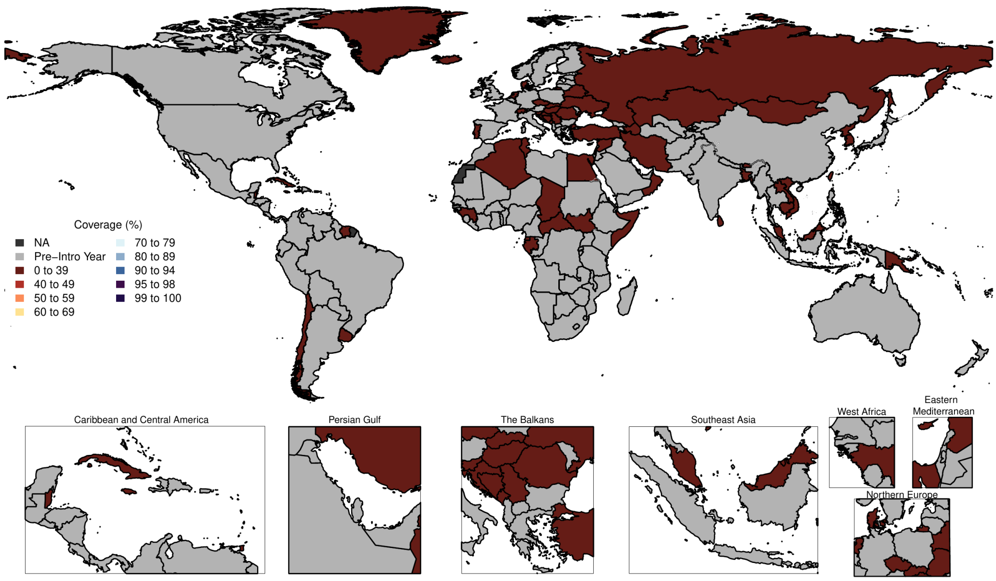

RotaC : 1990

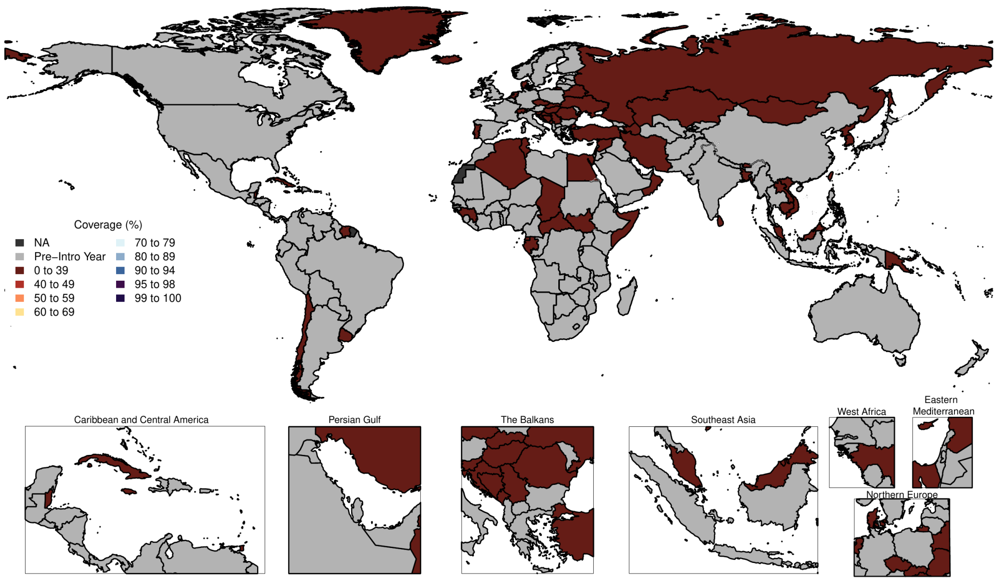

RotaC : 2000

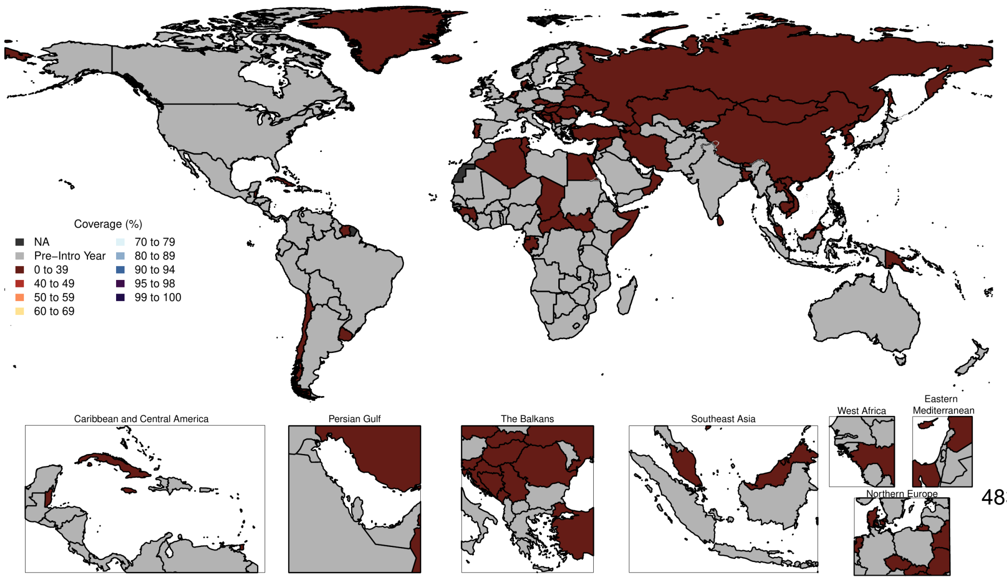

RotaC : 2010

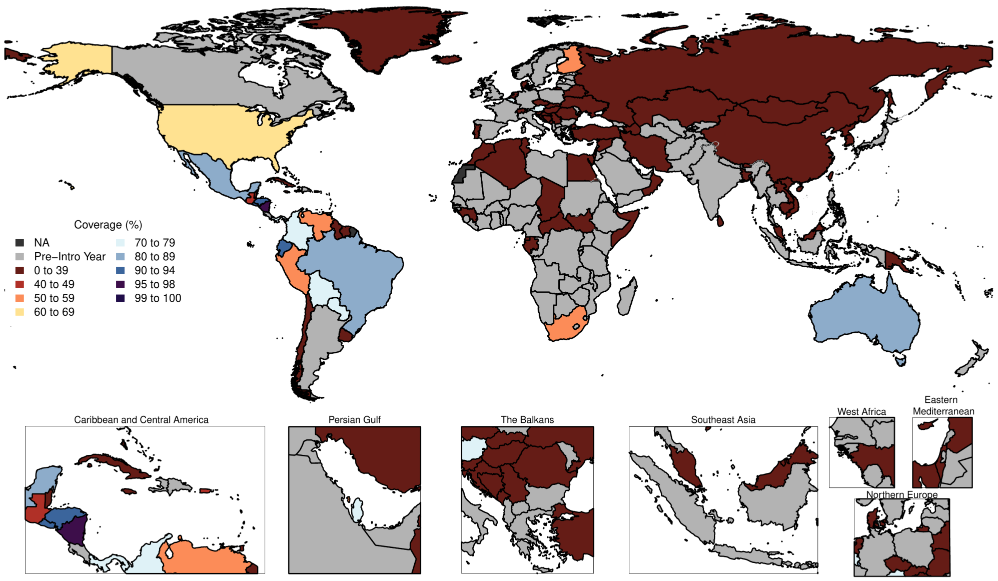

RotaC : 2019

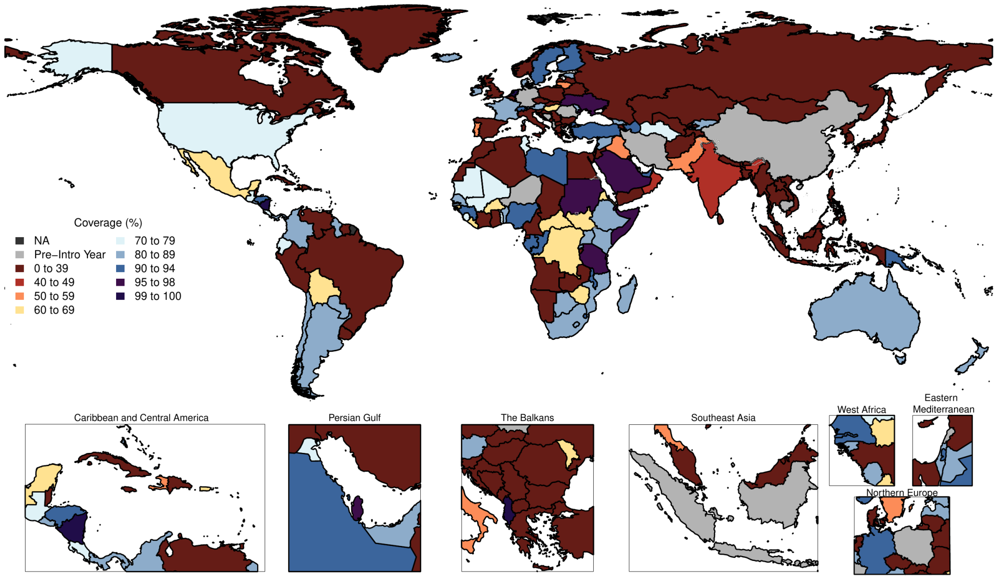

RotaC : 2020

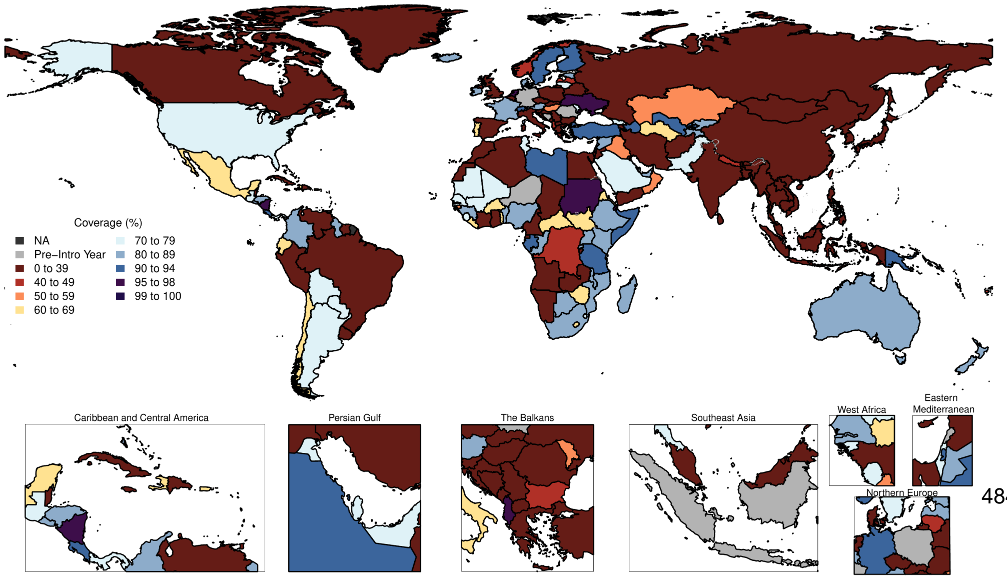

RotaC : 2021

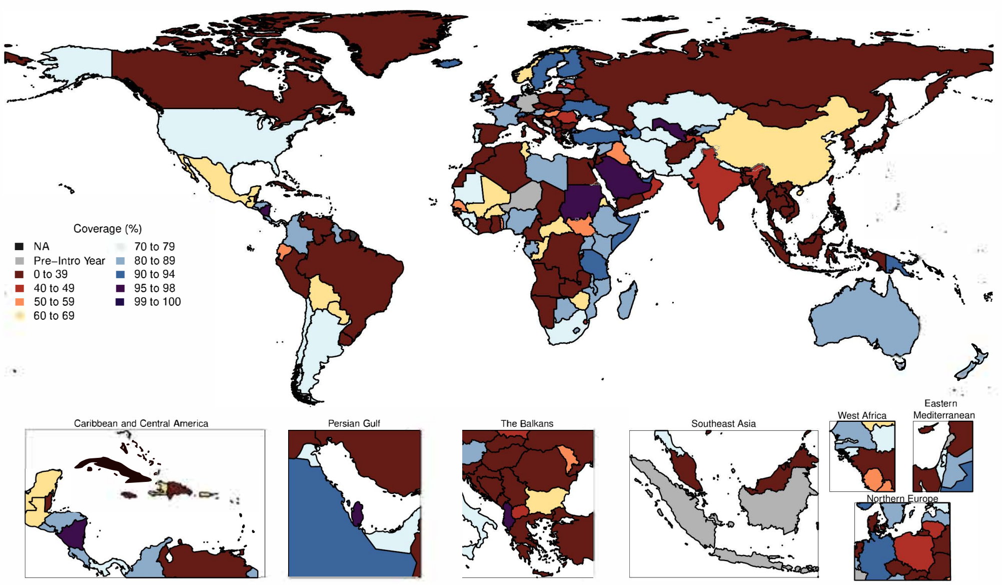

RotaC : 2022

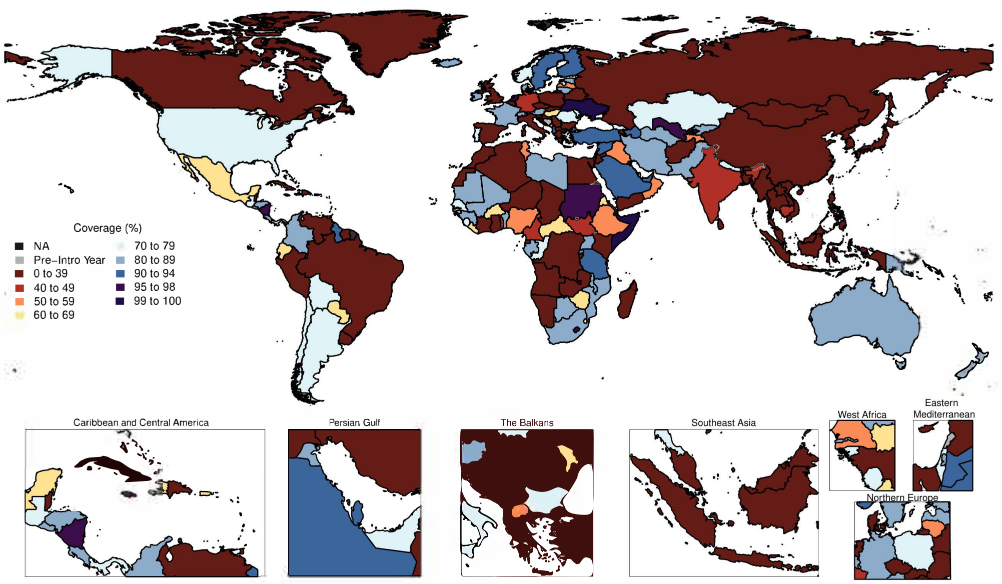

RotaC : 2023

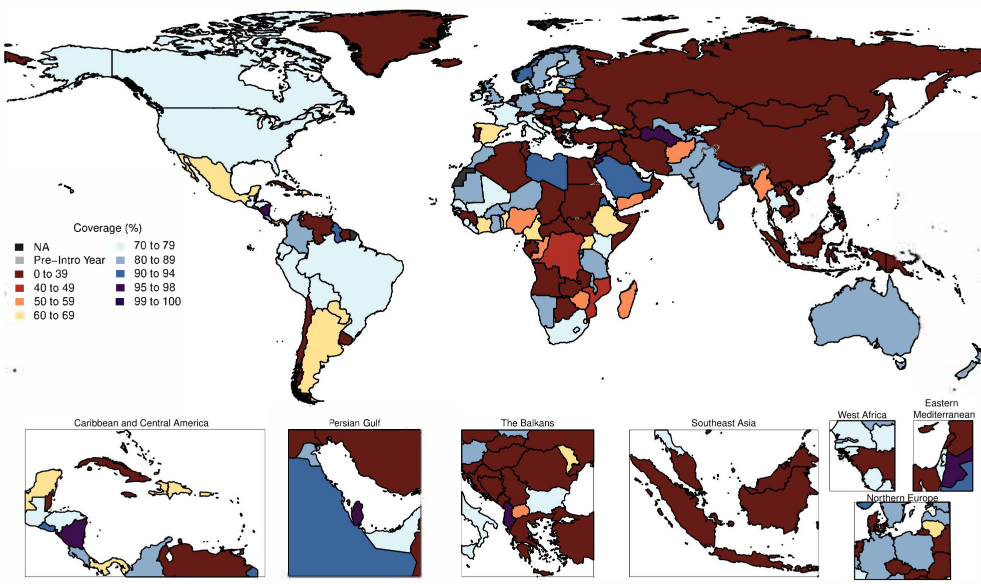

Figure S7 Impact of COVID-19 pandemic on vaccine coverage by super-region.
Vaccine coverage aggregated by super-region for years 2015–2023, depicting mean coverage inclusive of COVID-19 pandemic-related disruptions ("COVID inclusive") during years 2020-2023 (blue lines), as well as coverage in a "COVID free counterfactual" scenario absent disruptions related to COVID-19 (orange lines), as well as 95% uncertainty intervals for both. DTP1=diphtheria-tetanus-pertussis, first dose. DTP3=diphtheria-tetanus-pertussis, third dose. HepB3=hepatitis B vaccine, third dose. Hib3=Haemophilus influenzae type b vaccine, third dose. MCV1=measles-containing vaccine, first dose. MCV2=measles-containing vaccine, second dose. PCV3=pneumococcal conjugate vaccine, third dose. Pol3=polio vaccine, third dose. RCV1=rubella-containing vaccine, first dose. RotaC=completed rotavirus series.

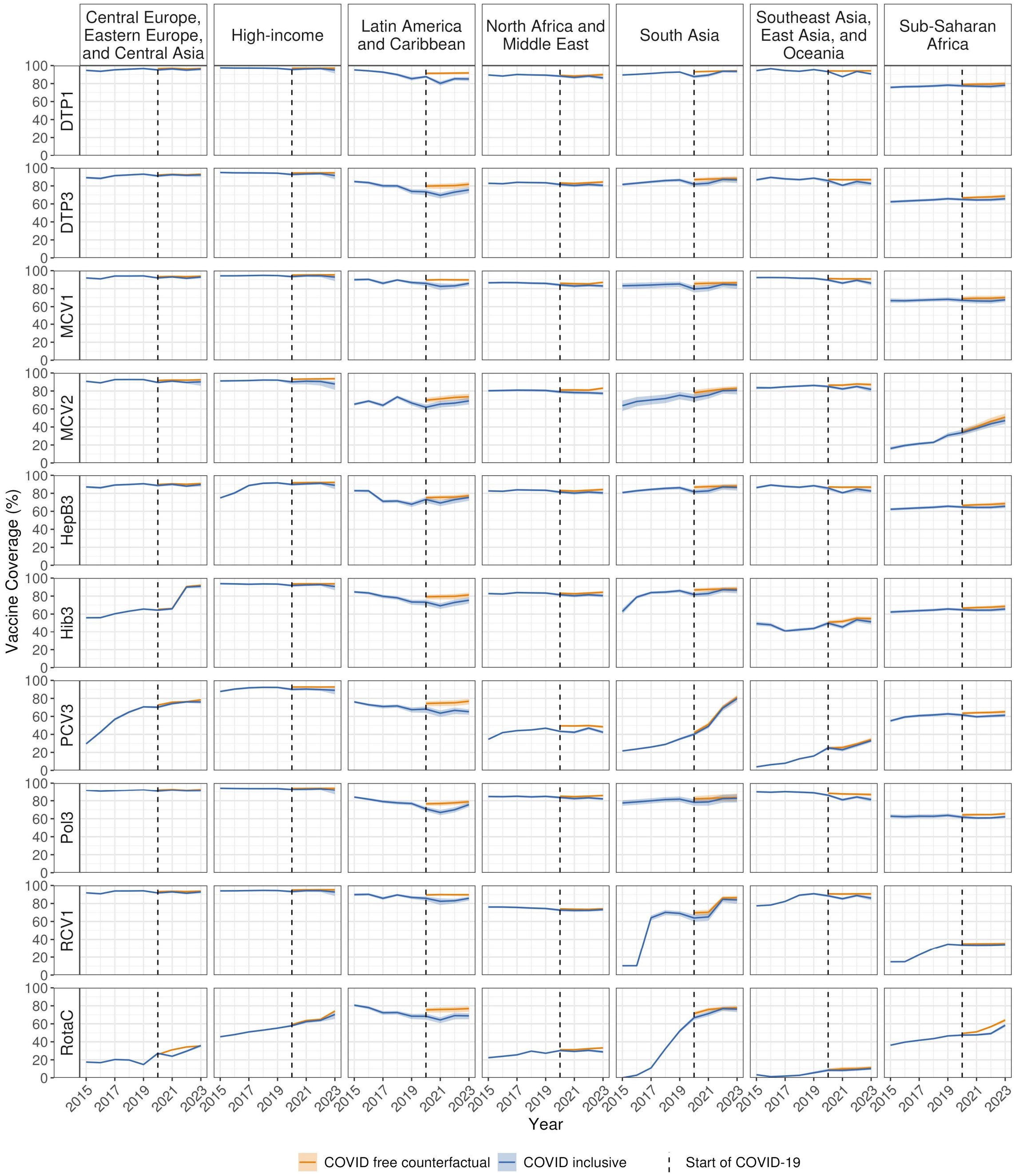

Figure S8 Forecast vaccine coverage for years 2024-2030 by super-region

Historic vaccine coverage means for DTP3, MCV2 and PCV3 (“life course vaccines”) for the target population by super-region for years 1980–2023, as well as mean forecasted vaccine coverage for years 2024–2030 under “reference” (black), “better” (green) and “worse” (pink) scenarios, as well as 95% uncertainty intervals for all scenarios. DTP3=diphtheria-tetanus-pertussis, third dose. MCV2=measles-containing vaccine, second dose. PCV3=pneumococcal conjugate vaccine, third dose.

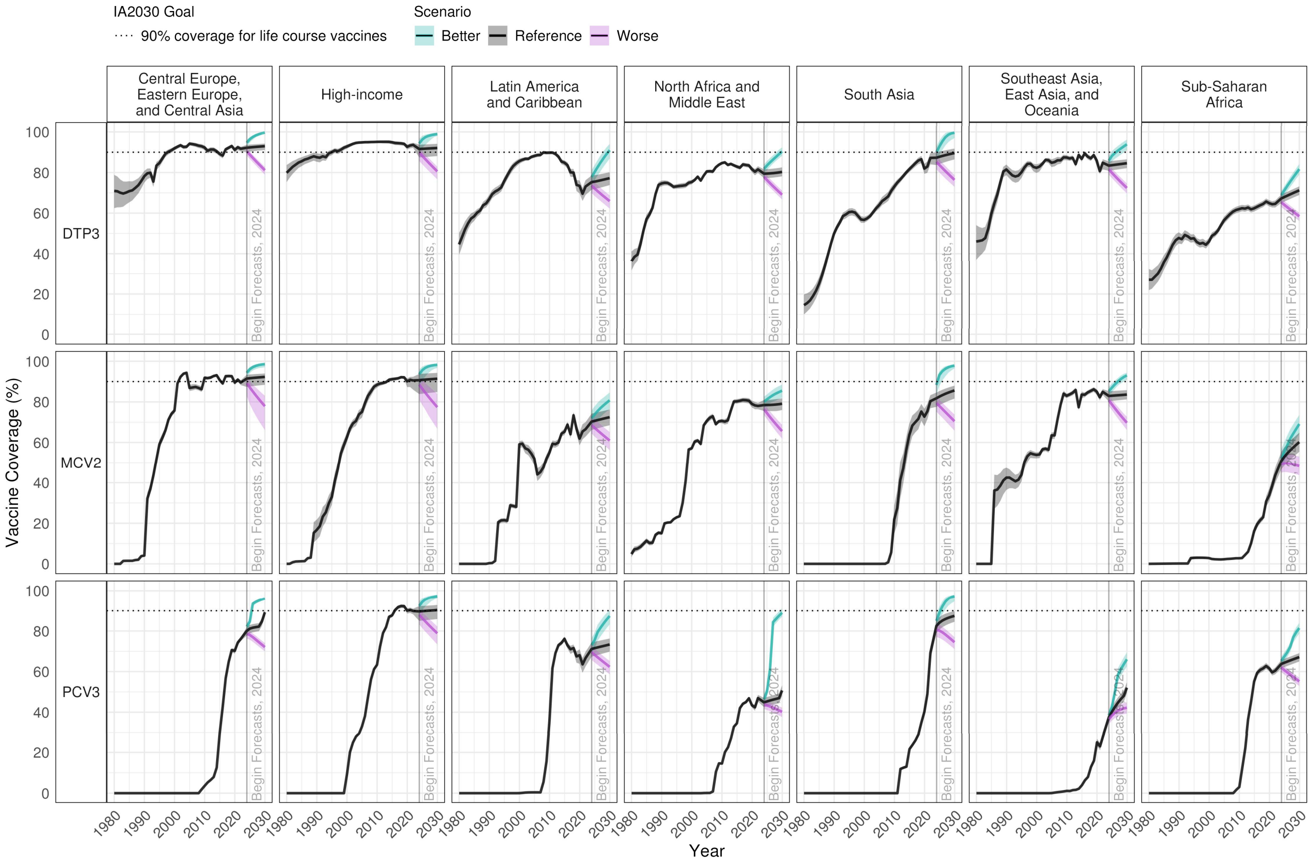

Figure S9 Vaccine coverage in 2023 vs. forecast vaccine coverage in 2030 by super-region

Comparison of historic vaccine coverage in 2023 for DTP3, MCV2 and PCV3 for the target population compared to forecasted vaccine coverage in 2030 under the “worse”, “reference” and “better” scenarios. Points represent individual countries and territories, coloured by super-region and sized according to population under one year of age, forecasted for the year 2030. DTP3=diphtheria-tetanus-pertussis, third dose. MCV2=measles-containing vaccine, second dose. PCV3=pneumococcal conjugate vaccine, third dose.

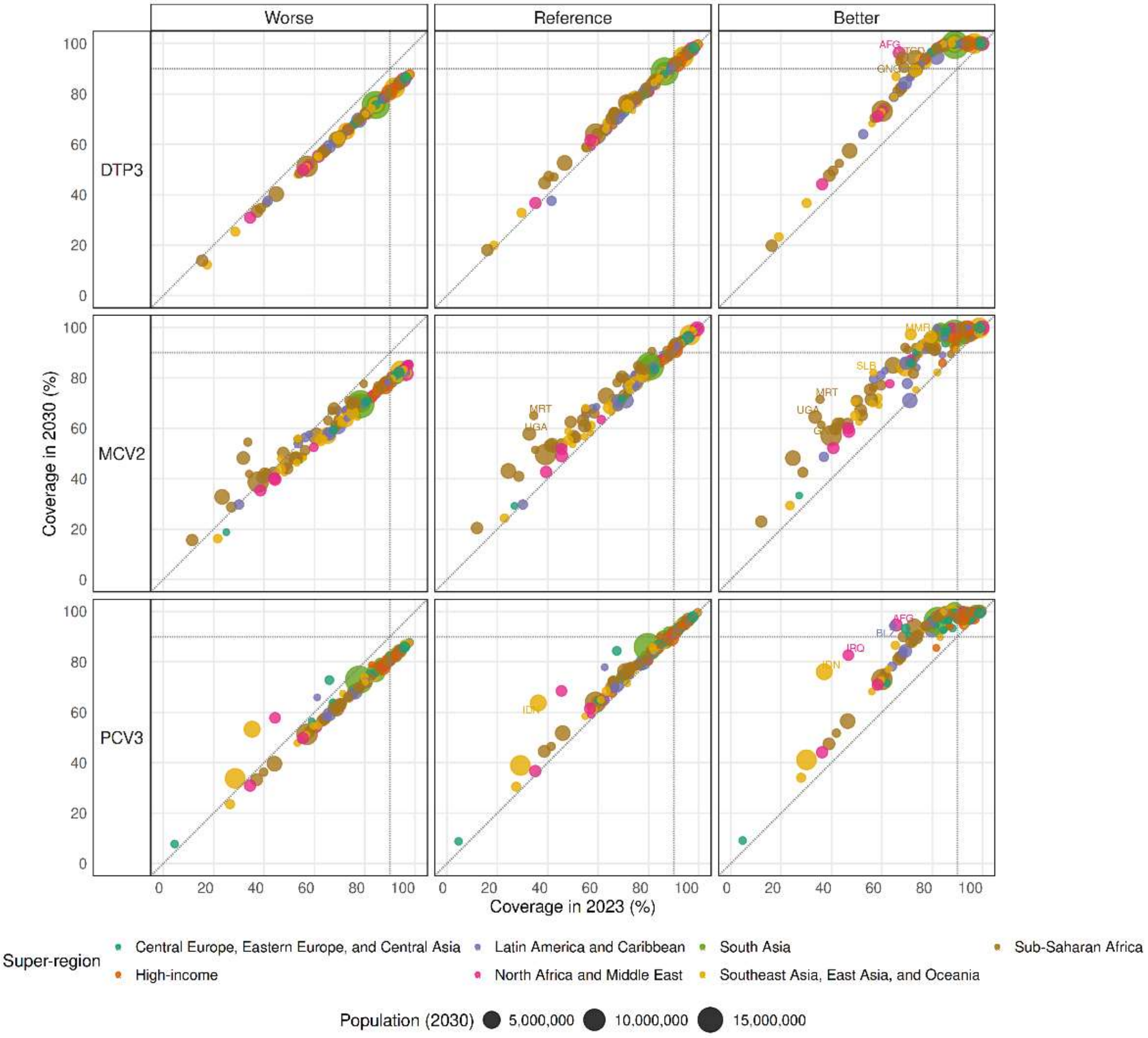

Figure S10 Distributions sampled to impute COVID-related disruption magnitudes by year. Product of simulated binomial and exponential distributions of the global percent difference between COVID inclusive and COVID-free counterfactual scenario vaccine coverage estimates by draw, for each vaccine or vaccine ratio modelled, by year, for years 2020–2023. These binomial and exponential distributions were simulated based on differences between coverage scenarios for vaccine-country-years where country-reported data was available for estimating disruption magnitudes, and these distributions were sampled from to apply imputed disruption estimates in country-vaccine-years with such country-reported data missing. BCG=Bacillus Calmette-Guérin. DTP1=diphtheria-tetanus-pertussis, first dose. DTP3=diphtheria-tetanus-pertussis, third dose. HepB3=hepatitis B vaccine, third dose. Hib3=Haemophilus influenzae type b vaccine, third dose. MCV1=measles-containing vaccine, first dose. MCV2=measles-containing vaccine, second dose. PCV3=pneumococcal conjugate vaccine, third dose. Pol3=polio vaccine, third dose. RCV1=rubella-containing vaccine, first dose. RotaC=completed rotavirus series.

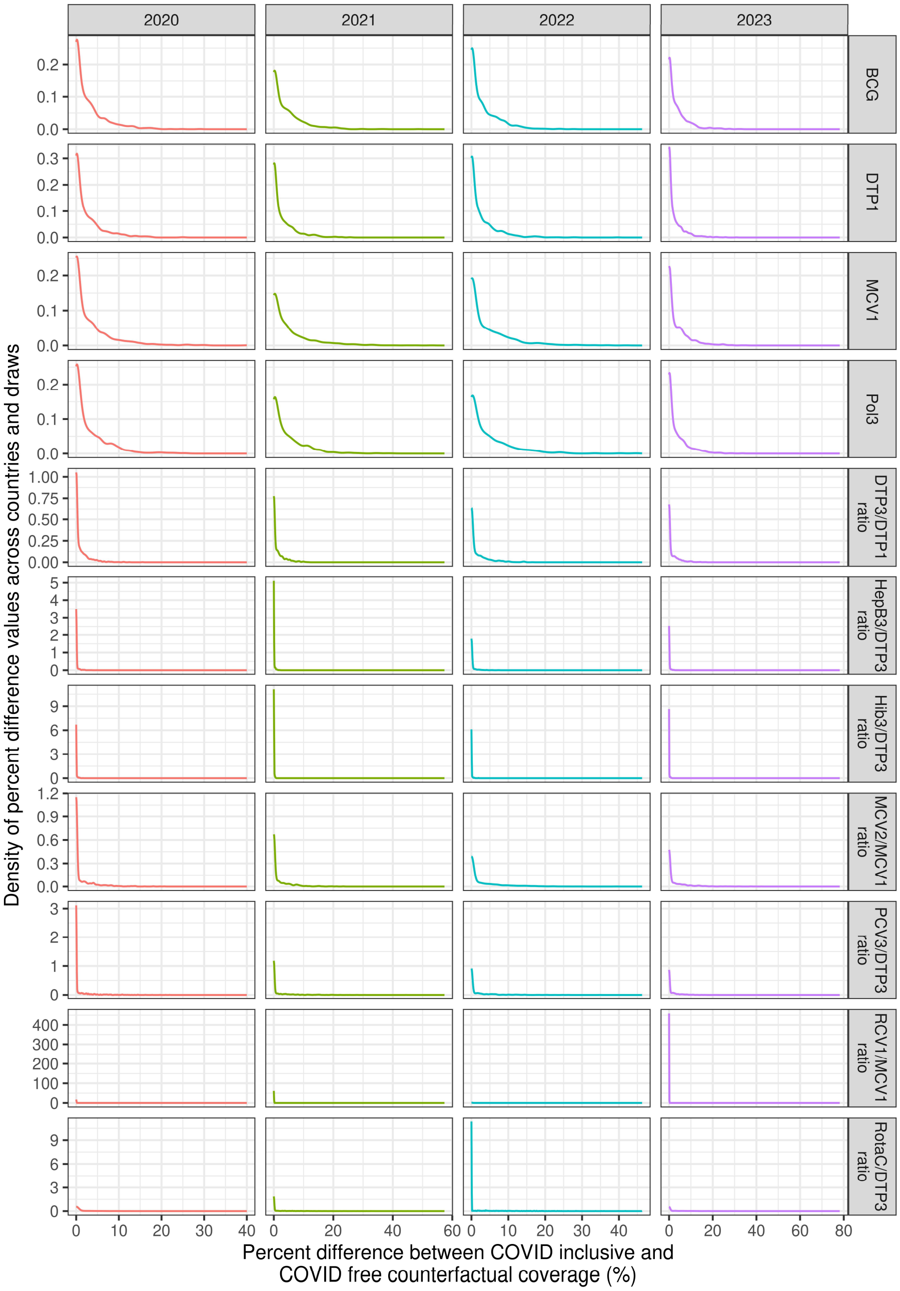

Figure S11 Global vaccine coverage time series plots, GBD 2023 vs GBD 2020 vs. WUENIC

Global vaccine-specific estimates of coverage proportions, 1980–2023. The solid red line illustrates GBD2023 estimated annual coverage for the target population, and previously published GBD2020 estimates are shown in solid purple. 95% uncertainty intervals surround each GBD mean estimate. WUENIC estimates released July 2024 are shown in light blue. WUENIC=WHO-UNICEF Estimates of National Immunization Coverage. GBD = Global Burden of Disease. DTP1=diphtheria-tetanus-pertussis, first dose. DTP3=diphtheria-tetanus-pertussis, third dose. HepB3=hepatitis B vaccine, third dose. Hib3=Haemophilus influenzae type b vaccine, third dose. MCV1=measles-containing vaccine, first dose. MCV2=measles-containing vaccine, second dose. PCV3=pneumococcal conjugate vaccine, third dose. Pol3=polio vaccine, third dose. RCV1=rubella-containing vaccine, first dose. RotaC=completed rotavirus series.

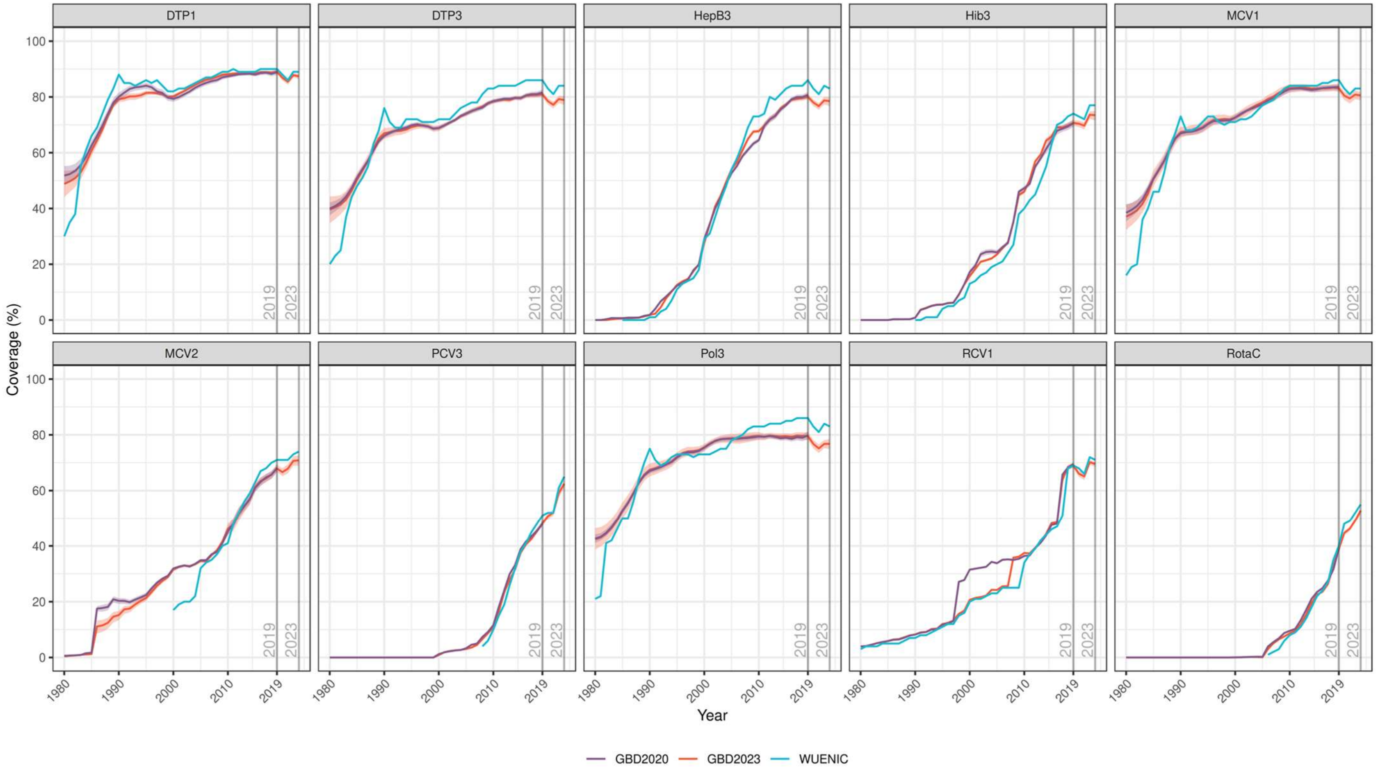

Figure S12 Vaccine coverage in GBD2020 vs. vaccine coverage in GBD2023

Comparison of vaccine coverage estimates in 2019, comparing the GBD 2023 vs. GBD 2020 manuscripts. Each point represents one country-year of estimated coverage for the target population, by GBD super-region, sized by the population under one year of age. BCG=Bacillus Calmette-Guérin. DTP1=diphtheria-tetanus-pertussis, first dose. DTP3=diphtheria-tetanus-pertussis, third dose. HepB3=hepatitis B vaccine, third dose. Hib3=Haemophilus influenzae type b vaccine, third dose. MCV1=measles-containing vaccine, first dose. MCV2=measles-containing vaccine, second dose. PCV3=pneumococcal conjugate vaccine, third dose. Pol3=polio vaccine, third dose. RCV1=rubella-containing vaccine, first dose. RotaC=completed rotavirus series.

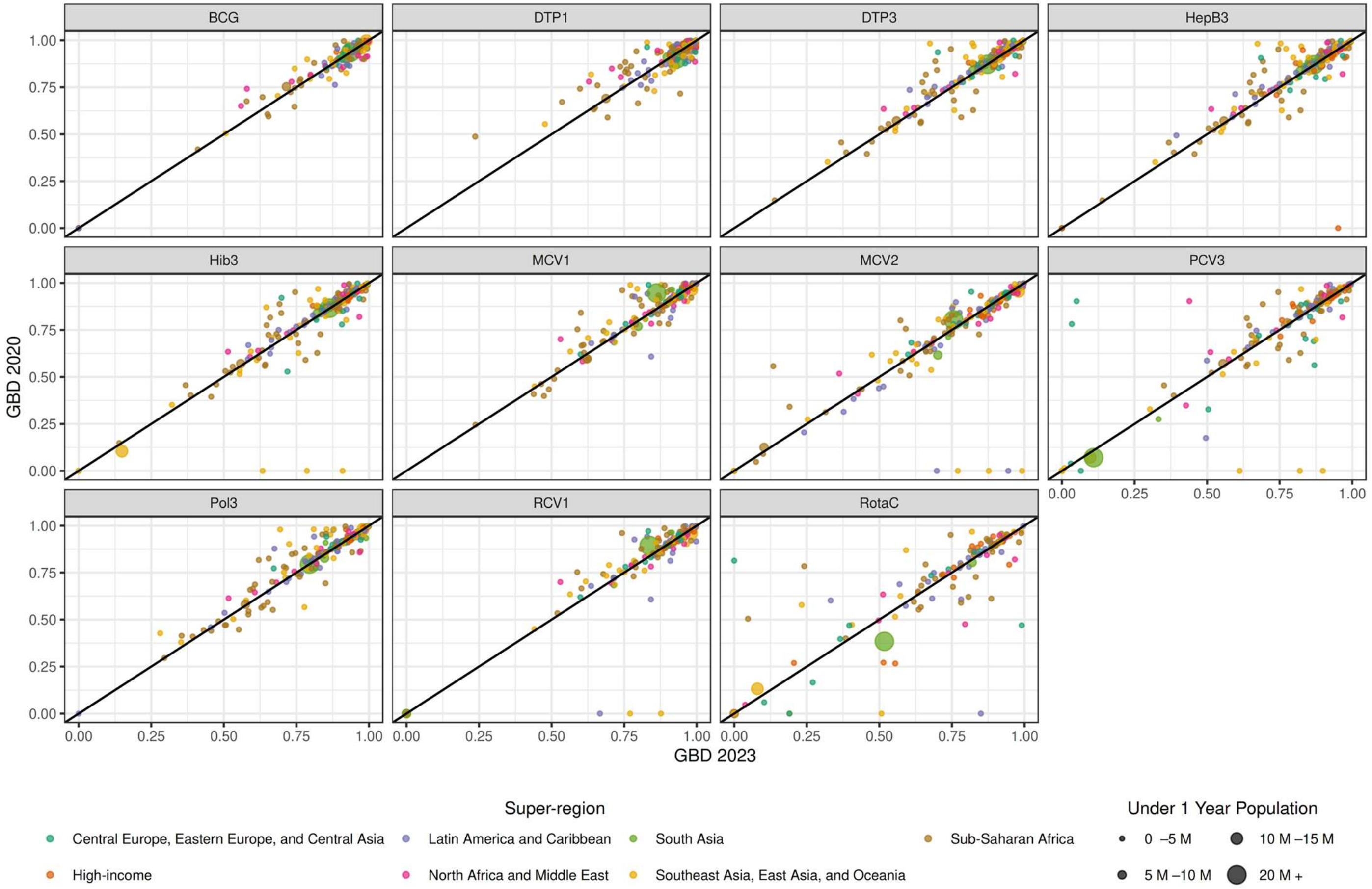

Figure S13 WUENIC vaccine coverage vs. GBD vaccine coverage in 2023

Comparison of historic vaccine coverage in 2023, comparing WUENIC estimates to GBD2023. Each point represents one country-year of mean estimated coverage for the target population, by GBD super-region, sized by the population under one year of age (comparison limited to 195 locations common to both GBD and WUENIC estimates). BCG=Bacillus Calmette-Guérin. DTP1=diphtheria-tetanus-pertussis, first dose. DTP3=diphtheria-tetanus-pertussis, third dose. HepB3=hepatitis B vaccine, third dose. Hib3=Haemophilus influenzae type b vaccine, third dose. MCV1=measles-containing vaccine, first dose. MCV2=measles-containing vaccine, second dose. PCV3=pneumococcal conjugate vaccine, third dose. Pol3=polio vaccine, third dose. RCV1=rubella-containing vaccine, first dose. RotaC=completed rotavirus series.

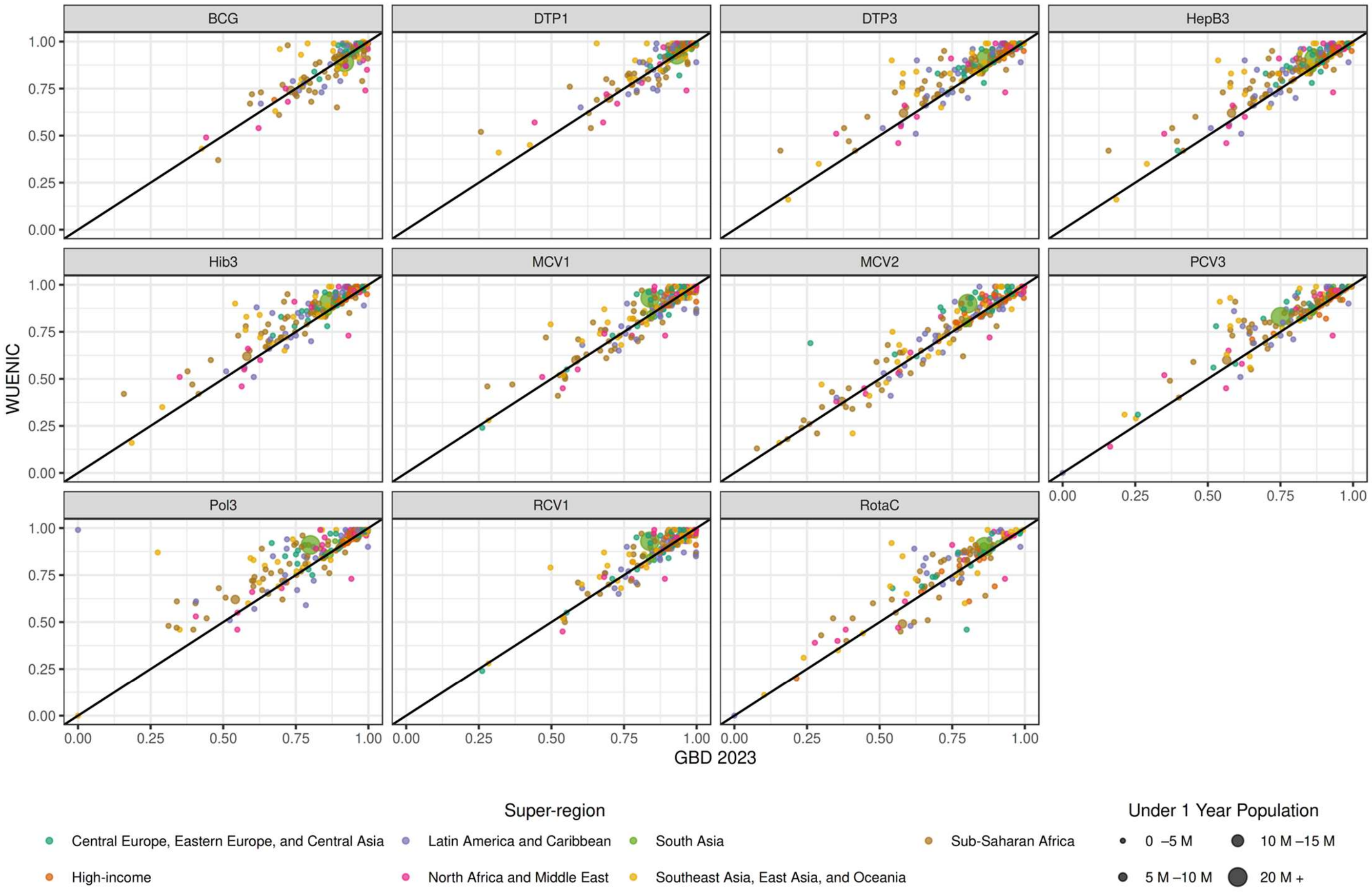

Supplement: Appendix 1 [file NIHMS2095684-supplement-Appendix_1.pdf]
